# GHT-SELEX demonstrates unexpectedly high intrinsic sequence specificity and complex DNA binding of many human transcription factors

**DOI:** 10.1038/s41592-026-03177-9

**Published:** 2026-08-05

**Authors:** Arttu Jolma, Aldo Hernandez-Corchado, Ally W. H. Yang, Ali Fathi, Kaitlin U. Laverty, Alexander Brechalov, Rozita Razavi, Mihai Albu, Hong Zheng, The Codebook Consortium, Arttu Jolma, Aldo Hernandez-Corchado, Ally W. H. Yang, Ali Fathi, Kaitlin U. Laverty, Alexander Brechalov, Rozita Razavi, Mihai Albu, Hong Zheng, Philipp Bucher, Bart Deplancke, Oriol Fornes, Jan Grau, Ivo Grosse, Fedor A. Kolpakov, Vsevolod J. Makeev, Marjan Barazandeh, Zhenfeng Deng, Chun Hu, Samuel A. Lambert, Zain M. Patel, Sara E. Pour, Mikhail Salnikov, Isaac Yellan, Georgy Meshcheryakov, Giovanna Ambrosini, Antoni J. Gralak, Sachi Inukai, Judith F. Kribelbauer-Swietek, Marie-Luise Plescher, Semyon Kolmykov, Ivan Yevshin, Nikita Gryzunov, Ivan Kozin, Mikhail Nikonov, Vladimir Nozdrin, Arsenii Zinkevich, Katerina Faltejskova, Pavel Kravchenko, Sergey Abramov, Alexandr Boytsov, Vasilii Kamenets, Dmitry Penzar, Anton Vlasov, Ilya E. Vorontsov, Quaid Morris, Xiaoting Chen, Matthew T. Weirauch, Ivan V. Kulakovskiy, Hamed S. Najafabadi, Timothy R. Hughes, Ivan V. Kulakovskiy, Hamed S. Najafabadi, Timothy R. Hughes

**Affiliations:** 1 Donnelly Centre, University of Toronto, Toronto, Ontario Canada; 2 Department of Human Genetics, McGill University, Montreal, Quebec Canada; 3 Victor P. Dahdaleh Institute of Genomic Medicine, Montreal, Quebec Canada; 4 Department of Molecular Genetics, University of Toronto, Toronto, Ontario Canada; 5 Computational and Systems Biology Program, Sloan Kettering Institute, Memorial Sloan Kettering Cancer Center, New York, NY USA; 6 Institute of Protein Research, Russian Academy of Sciences, Pushchino, Russia; 7 Institute of Biochemistry and Genetics, Ufa Federal Research Centre of Russian Academy of Sciences, Ufa, Russia; 8 Vavilov Institute of General Genetics, Russian Academy of Sciences, Moscow, Russia; 9 Swiss Institute of Bioinformatics, Lausanne, Switzerland; 10 Laboratory of Systems Biology and Genetics, Institute of Bioengineering, School of Life Sciences, École Polytechnique Fédérale de Lausanne, Lausanne, Switzerland; 11 Department of Medical Genetics, Centre for Molecular Medicine and Therapeutics, BC Children’s Hospital Research Institute, University of British Columbia, Vancouver, British Columbia Canada; 12 Institute of Computer Science, Martin Luther University Halle-Wittenberg, Halle, Germany; 13 Department of Computational Biology, Sirius University of Science and Technology, Sirius, Russia; 14 Bioinformatics Laboratory, Federal Research Center for Information and Computational Technologies, Novosibirsk, Russia; 15 Bioinformatics Competence Center, Ecole Polytechnique Fédérale de Lausanne, Lausanne, Switzerland; 16 Bioinformatics Competence Center, Université de Lausanne, Lausanne, Switzerland; 17 Chugai Pharmaceutical Co. Ltd, Tokyo, Japan; 18 Biosoft.Ru LLC, Novosibirsk, Russia; 19 Faculty of Bioengineering and Bioinformatics, Lomonosov Moscow State University, Moscow, Russia; 20 Institute of Organic Chemistry and Biochemistry of the Czech Academy of Sciences, Prague, Czech Republic; 21 Computer Science Institute, Faculty of Mathematics and Physics, Charles University, Prague, Czech Republic; 22 Max Planck Institute of Biochemistry, Planegg, Germany; 23 Altius Institute for Biomedical Sciences, Seattle, WA USA; 24 Center for Molecular and Cellular Bioengineering (CMCB), TUD Dresden University of Technology, Biotechnologisches Zentrum (BIOTEC), Dresden, Germany; 25 Cincinnati Children’s Hospital, Cincinnati, OH USA; 26 Present Address: Cancer Research UK National Biomarker Centre, University of Manchester, Manchester, UK

**Keywords:** Gene regulation, Transcription, Computational models, Genome-wide analysis of gene expression, Genomics

## Abstract

There is ongoing debate regarding the degree to which transcription factors (TFs) independently specify genomic binding: TF binding motifs are typically short and degenerate, yielding many more binding site predictions than observed in cells. Here we present genomic high-throughput SELEX (GHT-SELEX)—a scalable method that surveys intrinsic binding of purified TFs to the fragmented, naked and unmodified genome. GHT-SELEX peaks for 179 diverse human TFs display surprisingly high overlap with chromatin immunoprecipitation sequencing peaks for the same TF. Comparable overlap can be obtained from motifs using appropriate analytical approaches. For C2H2 zinc finger (zf) proteins—the largest class of human TFs—GHT-SELEX shows that modular, alternative engagement of C2H2-zf domains is the norm, enabling several types of distinct target sites, and frequently involving internal duplication and divergence within the C2H2-zf array. Altogether, it is common for TFs to delineate a large fraction of in vivo genomic binding sites independently of other cellular factors.

## Abstract (web summary)

GHT-SELEX—high-throughput SELEX with fragmented genomic DNA—shows that transcription factors can be rather particular about which genomic loci they bind, and that C2H2 zinc finger proteins often engage different fingers at different sites.

## Main

The DNA-binding sequence preference of a TF is referred to typically as a motif, and is modeled most commonly as a position weight matrix (PWM), which describes the relative preference of the TF for each base in the binding site^1^. Human TF binding motifs are generally short and flexible; PWMs are typically 8–14 bases long^2–4^ and several bases can be tolerated at many positions^5,6^. Thus, a typical TF PWM scan with default parameters yields more than a million potential binding sites in the 3-billion-base human genome, often with several high-scoring matches per gene. Very few of the potential target sites are utilized in cells^7^, however, and the actual number of bound sites, as measured by chromatin immunoprecipitation sequencing followed by sequencing (ChIP–seq)^8–10^ or other assays^11^, is typically much lower than the number of motif matches.

This deficit in specificity has been rationalized conceptually by cooperative binding and synergy among TFs^5,6,12,13^, and dominance of the chromatin landscape on binding site selection^14^, in which only a special class of ‘pioneer’ TFs can access target sequences to control the local chromatin. For example, CTCF binds most of its strongest motif matches in the genome^15^, repositioning the surrounding nucleosomes^16^, and PRDM9, which controls recombination hotspots, has been reported to independently specify roughly half of its binding sites in the genome^17^. Another possible explanation for the generally low apparent specificity of TF motifs, however, is that PWMs are inaccurate, or that the PWM model is fundamentally flawed^18^. PWMs are often derived from a noncomprehensive set of bound versus unbound sequences, and there is ongoing controversy regarding the best methods for derivation, underlying representation and scanning of TF motifs^1,19^, as well as the impact of DNA shape^20^, dependencies among base positions^18,21^, lower-affinity binding sites^22^ and cooperative multimeric binding^23,24^. For example, proteins that contain only one CXXC-zf domain, which recognizes one CG dinucleotide^25^, bind preferentially to clusters of unmethylated CG dinucleotides within CpG islands^26^.

Many human TFs, including hundreds of C2H2-zf proteins^27^, still lack binding motifs (that is, PWMs). C2H2-zf proteins recognize DNA sequences that approximate a concatenation of the three or four base specificities of their sequential constituent C2H2-zf domains^28,29^. Different C2H2-zf proteins can bind very different motifs due to both the malleability of the individual C2H2-zf domains and rearrangement of the individual C2H2-zf domains^30^. C2H2-zf can, in theory, also recognize very long sequences: the median number of C2H2-zf domains in human TFs is 11, which could contact up to 33 DNA bases—more than what is required to specify most individual genomic positions. Some C2H2-zf proteins use only a subset of their C2H2-zfs to contact DNA, and whether and how frequently human C2H2-zf proteins utilize different segments of the C2H2-zf domain array to bind different sequences has been a long-standing question. In a well-studied example, CTCF binding sites seem to reflect a constitutive ‘core,’ bound by fingers 4–7 of the 11 C2H2-zf domain array, flanked by sequences that are bound by alternative usage of upstream and/or downstream C2H2-zf domains^31,32^. In another example, mouse Znf335 was found to bind to two distinct motifs utilizing distinct C2H2-zf arrays^33^. Identification of the precise DNA-binding sites of C2H2-zf proteins to the genome can be difficult because they often bind repeat elements such as endogenous retroelements^34^, confounding conventional motif discovery. This can be ameliorated by incorporating information about the bases that are probably preferred at each position of the binding site, as predicted by a C2H2-zf ‘recognition code’ that relates the C2H2-zf amino acid sequences to their binding preferences, thus isolating the most plausible protein–DNA interactions^35^.

Methods for the study of TF sequence specificity in vitro are important because they remove confounding impact of cellular factors beyond the TF of interest. Systematic evolution of ligands by exponential enrichment (SELEX)^36^ uses several cycles of affinity capture interleaved with PCR amplification of the selected DNA ligands. In high-throughput SELEX (HT-SELEX), the protein-bound DNA is immobilized in microwell plates and multiplexed Illumina sequencing is performed on all selection cycles. HT-SELEX is rapid and unbiased, and has been highly successful^37,38^, but has the disadvantage that increasingly long subsequences are present in a decreasing proportion of pool sequences. This issue is of particular concern for C2H2-zfs, due to the potential for very large binding sites, and long C2H2-zf proteins have a lower success rate in HT-SELEX than those with few C2H2-zf domains^37,39^. An alternative to randomized DNA is to use fragmented genomic DNA. SELEX has been performed previously with *Escherichia coli* genomic DNA with either a sequencing^40^ or microarray^41^ readout. More recent innovations, including CAP-seq^42^, Affinity-seq^17^, DAP-seq^43^ and PB-exo^44^ have used only a single round of selection, allowing direct comparison (and similar peak calling) to ChIP–seq. These approaches have revealed remarkably high specificity for some TFs. They are not cost-effective when applied to the human genome, however, due to its large size. To our knowledge, only two human proteins (PRDM9 (ref. ^17^) and Cfp1 (ref. ^42^)) have been studied by these methods.

Here we describe genomic DNA HT-SELEX (GHT-SELEX)—an adaptation of HT-SELEX^38^ that utilizes genomic DNA instead of random sequences—and we introduce an associated statistical analysis method: model-based analysis of genomic intervals with exponential enrichment (MAGIX). GHT-SELEX combines the advantages of HT-SELEX (the use of barcoding, magnetic affinity beads and laboratory automation makes it possible to run GHT-SELEX in parallel with hundreds of samples over numerous cycles) with the strengths of genomic DNA selection protocols^17,43^ (representation of long genomic sites such as repeat sequences, and determination of individual genomic binding sites). We developed GHT-SELEX in the context of the Codebook Consortium Project^45^, aimed primarily at analysis of uncharacterized putative TFs, providing comparison data from several other platforms for the same set of TFs (HT-SELEX, ChIP–seq, protein binding microarrays^46^ and SMiLE-seq^47^). We applied GHT-SELEX successfully to 139 uncharacterized TFs and 40 control TFs. For dozens of TFs, including some that are considered well characterized, GHT-SELEX peaks correspond closely to in vivo binding (assessed by ChIP–seq). We also derived motif models that predict genomic binding with similar accuracy in most of these cases. GHT-SELEX is particularly effective for C2H2-zf proteins and shows that they often use alternative subsets of their C2H2-zf domains to engage with different genomic target sites. We explore both explanations and ramifications of these observations.

## Results

### Development and testing of GHT-SELEX

**Fig. 1:**
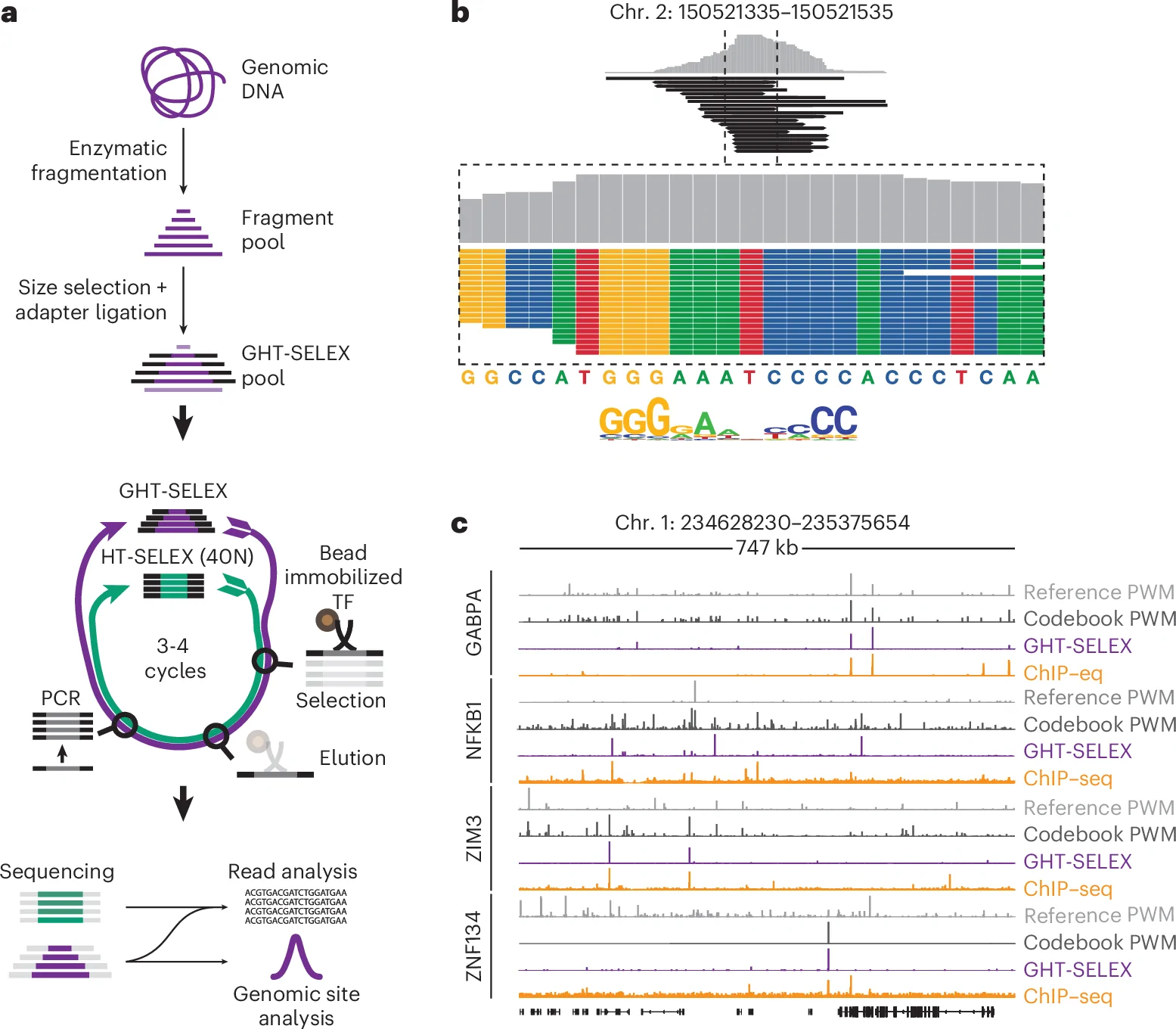
Overview of GHT-SELEX. **a**, Schematic of GHT-SELEX, showing parallels with HT-SELEX. **b**, Example of read accumulation over a TF motif match for NFKB1. **c**, Genomic binding for four positive control TFs on a genomic region showing (top to bottom) PWM scanning scores (moving average of affinity scores, from MOODS^62^ scan in linear domain, using a window of size 200 bp) for reference PWMs (CIS-BP identifiers:M02996 (GABPA), M03448 (NFKB1), M08312 (ZIM3) and M08392 (ZNF134)) and Codebook PWMs, followed by read coverage signal observed in GHT-SELEX and ChIP–seq. Source data

We developed GHT-SELEX to run in parallel with HT-SELEX (Fig. 1a; see Supplementary Table 1 for oligomer designs and Supplementary Table 2 for experimental parameters) in the context of the Codebook consortium^45^—a systematic effort to obtain DNA-binding motifs for 332 uncharacterized putative TFs (defined as proteins containing DNA-binding domains (DBDs) or having previous literature evidence but lacking known motifs^27^), as well as 61 control TFs with established motifs (Supplementary Table 3). The GHT-SELEX DNA pool used in this study was produced by nonspecific enzymatic fragmentation (Fragmentase) of HEK293 DNA to fragments with a median length of ~64 bp. The pool was PCR amplified before selection and is thus unmethylated. We used HEK293 DNA for compatibility with ChIP–seq data generated simultaneously (see accompanying manuscript^48^), and the length of the DNA was chosen to mimic standard HT-SELEX procedures and provide relatively high resolution.

**Fig. 2:**
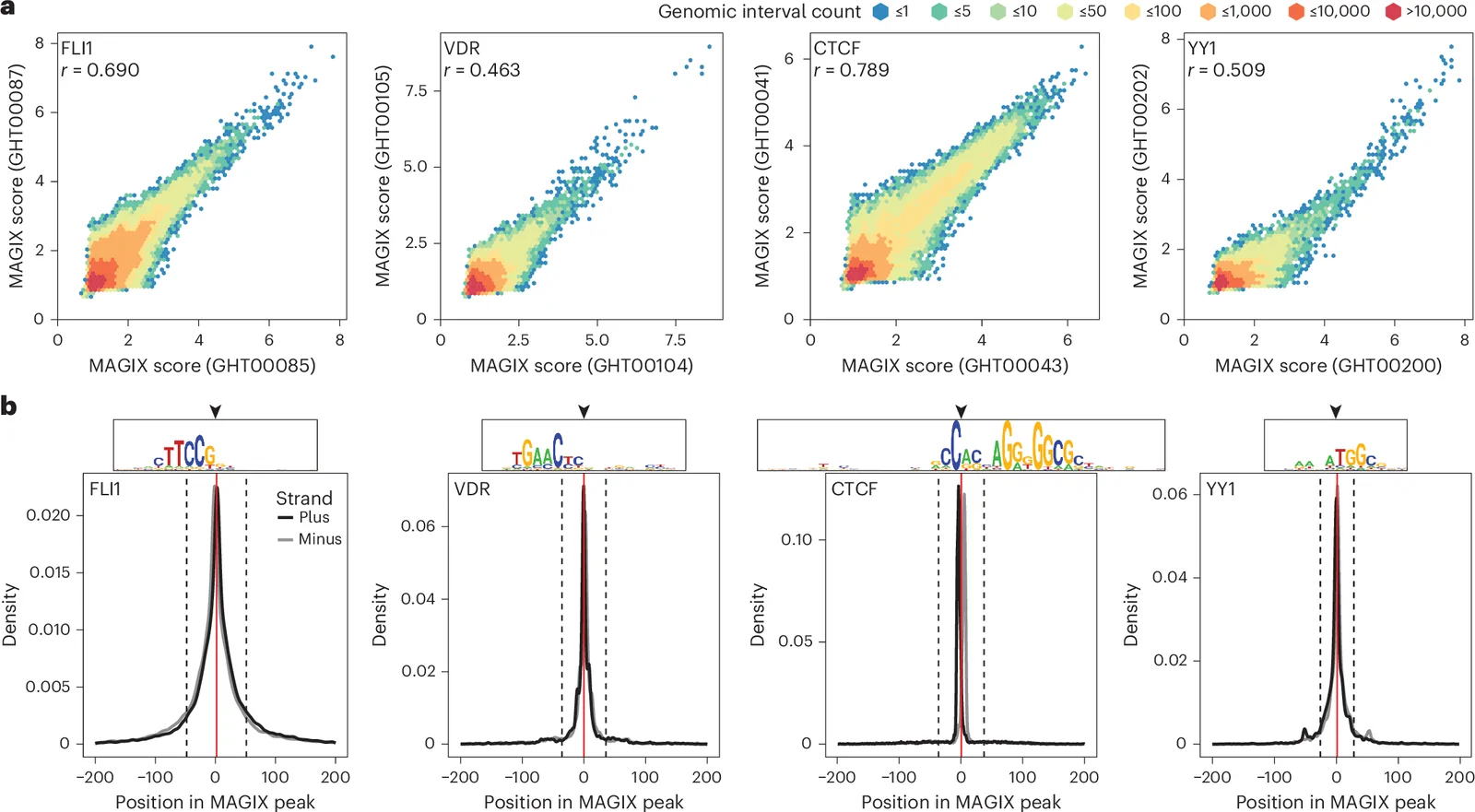
MAGIX method for interpretation of GHT-SELEX data. **a**, GHT-SELEX reproducibility. Scatterplots for four control TFs show MAGIX score values for all 200 bp genomic bins between replicate GHT-SELEX experiments. **b**, Base-level distribution of PWM hits for the top-ranked TF PWM (highest AUROC on GHT-peaks as determined in the accompanying study^50^) within the MAGIX peaks (up to the 10,000 highest scoring peaks). PWM hits were identified with MOODS^62^ (match *P* value threshold of *P* < 0.0001) and the hit location is defined as the center of the PWM, depicted above each plot with an arrow. Solid vertical red lines: mean PWM hit position within MAGIX peaks; dashed lines: 1 s.d. about the mean distance from peak center. Hits on the plus and minus strands are differentiated as indicated. *P* values calculated within MOODS using log-odds scoring of position weight matrices against a background nucleotide distribution. Source data

We initially tested GHT-SELEX on the Codebook control proteins. Thirty of the control TFs represented a sampling of well-studied TFs with different classes of DBDs, most of which were analyzed previously using the independent in vitro SMiLE-seq platform^47^. An additional 31 controls were C2H2-zf proteins for which published ChIP–seq data yielded motifs^49^. Individual mapped reads typically accumulated at sites in which all reads overlap with what seems to be a motif match (see example in Fig. 1b). Moreover, the GHT-SELEX data typically had a strong resemblance to ChIP–seq data, forming strong peaks found sparsely across the genome, consistent with results from CAP-seq^42^, Affinity-seq^17^ and DAP-seq^43^. Figure 1c shows raw read density for four control TFs, comparing GHT-SELEX to ChIP–seq and to target site predictions based on existing and Codebook PWM models for the TFs (see Supplementary Data 1 for representative PWMs). GHT-SELEX experiments showed generally good reproducibility between replicates (see examples in Fig. 2a).

**Extended Data Fig. 1:**
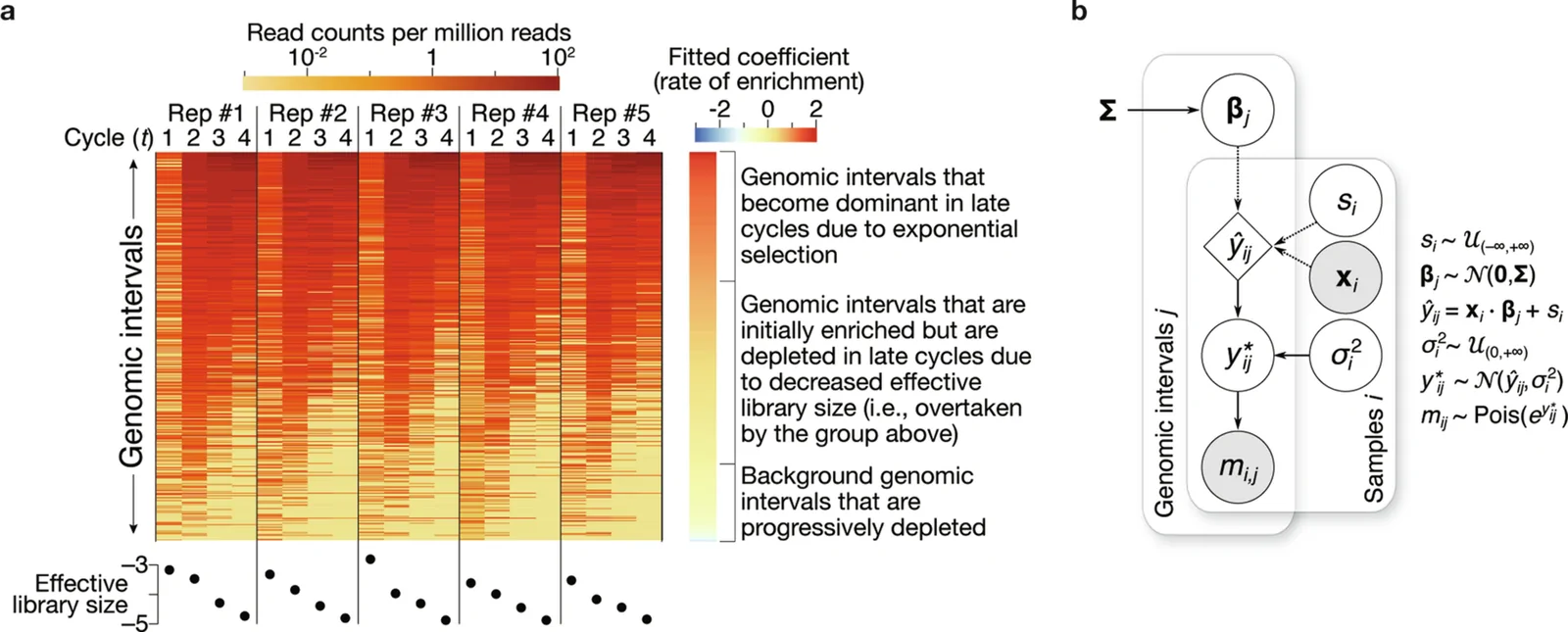
Characteristics of GHT-SELEX data and the MAGIX framework. **a**. Example of actual read count data for CTCF over five replicates of four cycles, illustrating enrichment patterns, fitted coefficients *(right)*, and estimated library sizes *(bottom)*. **b**. A brief overview of the statistical framework of the generative model of MAGIX. Open circles, closed circles, and the diamonds represent latent variables, observed variables, and deterministic computations, respectively. *s*_*i*_: library size for sample *i*; **x**_*i*_: vector of sample-level variables for sample *i*, including an intercept term and a term for the SELEX cycle, in addition to other terms for batch and background effects; **β**_*j*_: vector of model coefficients for interval *j*; *m*_*ij*_: number of observed reads mapping to interval *j* in sample *i*. See **Methods** for description of other variables.

Peak calling from the GHT-SELEX data with conventional algorithms is confounded by inherent properties of SELEX assays. In initial cycles the DNA pool has relatively few high-affinity target sites, leading to low sampling rates, whereas later selection cycles can be dominated by strongest binding sites (Extended Data Fig. 1a). Consequently, enrichment information is distributed across the fragments from several selection cycles, with weaker peaks first appearing and disappearing as they are outcompeted by the strongest peaks in later cycles (Extended Data Fig. 1b). To adapt to these phenomena, we developed the analytical framework MAGIX, which capitalizes on the added information gained from several SELEX cycles (Extended Data Fig. 1b and Methods). The approach relies on a statistical method that models explicitly the exponential growth of TF-bound genomic regions over the SELEX cycles, which leads to a progressively higher proportion of TF-bound fragments relative to genomic background. The fragment abundances, in turn, are modeled as latent variables that determine the number of observed reads through a Poisson process. This hierarchical Bayesian model enables the integration of information across different selection cycles, experiments and batches to calculate an estimated enrichment coefficient (MAGIX score) and associated false discovery rate (FDR) (Extended Data Fig. 1b).

Among the 61 control proteins, we deemed 40 as successful in at least one GHT-SELEX experiment, based primarily on enrichment of the expected motif at the peak center (Fig. 2b) (see Methodsand accompanying papers^45,50^ for details of how success was determined, and how PWMs were derived). The control proteins that were not successful in GHT-SELEX were mainly (15 out of 21) from among the C2H2-zf proteins for which published ChIP–seq data yielded motifs^49^. These experiments were performed early in the study, before technical innovations that dramatically improved success rates with this class of proteins. The success rate for proteins for which previously reported SMiLE-seq data yielded motifs may be a more realistic metric to gauge the success rate of GHT-SELEX in its current form (24 out of 30 (80%)).

Among the 40 control TFs that were successful, 23 were represented by more than one successful experiment (Supplementary Table 4), allowing us to assess the reproducibility of the combined GHT-SELEX/MAGIX pipeline. Figure 2a shows the enrichment values over genomic bins for replicates of FLI1, VDR, YY1 and CTCF. Similar plots for all successful replicates are given in Supplementary Data 2, and Pearson correlations for replicate pairs are in Supplementary Table 5. We conclude that enrichment values across the genome are reproducible. MAGIX readily accommodates several experiments into a single analysis, and all subsequent analyses in this study utilize merged experimental data for each distinct protein.

Analysis of data for the 40 successful controls by MAGIX resulted in between 926 and 82,045 peaks (median 9,927) with enrichment coefficient FDR less than 5% (Methods). The number of strong PWM hits declines, on average, rapidly at ~50 bp from peak centers, consistent with the utilized DNA fragment size (Fig. 2b); similar plots for all TFs analyzed are shown in Supplementary Data 3. In addition, higher PWM scores (which would, in theory, predict higher relative affinity) are associated clearly with a higher GHT-SELEX enrichment coefficient.

### Application of GHT-SELEX to the full Codebook TF set

We next performed GHT-SELEX and, in parallel, HT-SELEX using random 40N ligands (Supplementary Table 1) to assess DNA sequence specificity of 331 poorly characterized putative human TFs, as part of the Codebook project. Because the binding motifs were not known in advance, we gauged the success of each protein in each experiment, including the GHT-SELEX experiments, based on whether similar DNA-binding motifs (that is, PWMs) were obtained from different types of experiment, with all data types considered in aggregate by a team of expert curators^50^. We modulated several experimental variables over the course of the study in an effort to improve success rates and evaluate experimental procedures (Supplementary Table 2 and Methods), but analyzed the data in aggregate. Selection of a single PWM for each TF for subsequent analyses is also described in the accompanying study^45^; PWMs logos, and information about their derivation are available in the accompanying study^45^ and online at https://codebook.ccbr.utoronto.ca, https://mex.autosome.org and https://cisbp.ccbr.utoronto.ca (ref. ^51^).

**Fig. 3:**
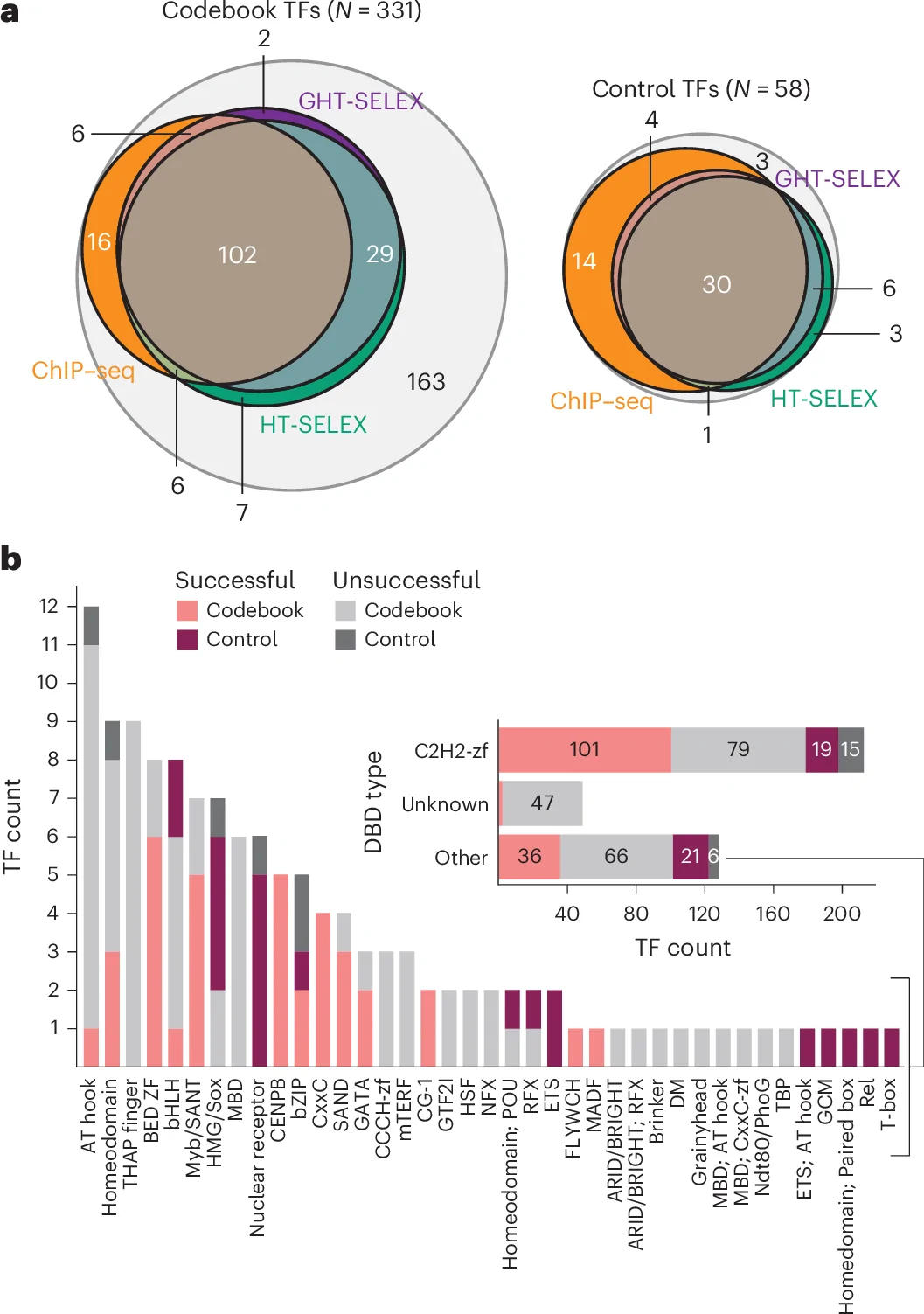
Analysis of 331 Codebook proteins and 61 control TFs using GHT-SELEX. **a**, Venn diagram displaying the number of TFs with successful experiments in GHT-SELEX, HT-SELEX and ChIP–seq for all Codebook (left) and control (right) TFs assayed with GHT- and HT-SELEX. **b**, Barchart showing the number of TFs with at least one successful GHT-SELEX experiment, categorized based on DBD type. C2H2-zf proteins and those with an unknown DBD (at the beginning of the project) are inset due to large numbers. Source data

**Extended Data Fig. 2:**
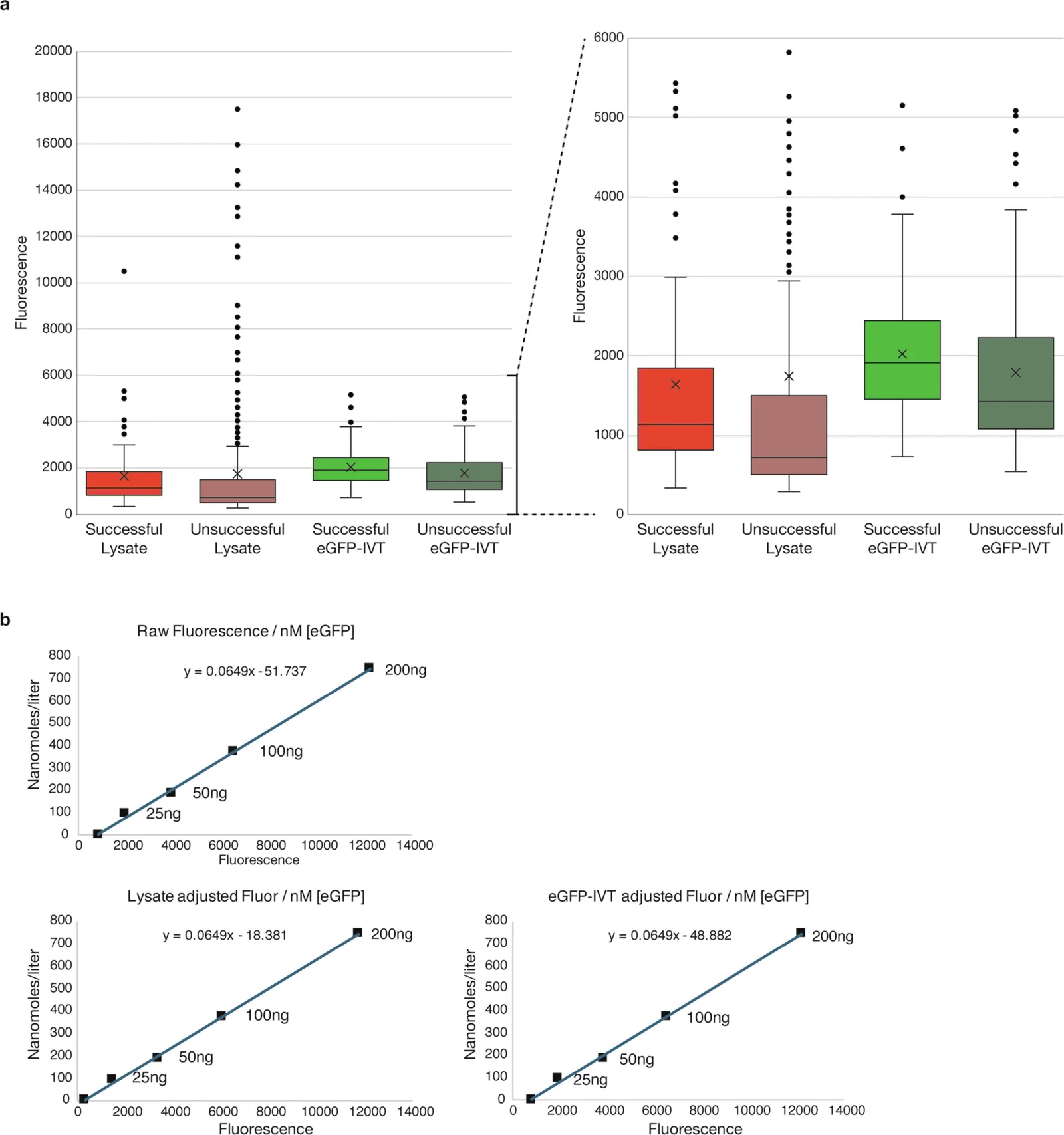
Expression level measurements for TFs in SELEX experiments. **a**. TF expression levels are plotted for four categories based on experiment success and protein production. Data is shown for the full range of fluorescence (*top)* and for the region with most of the signal (*bottom)*. Note that even though the successful samples have generally higher expression levels in both protein production systems it is not an effective predictor of experiment success. Fluorescence was measured for 137 successful lysate, 399 unsuccessful lysate, 138 successful eGFP-IVT and 149 unsuccessful eGFP-IVT protein preparations. Each data point represents one protein preparation that directly corresponds to a GHT-SELEX experiment. Preparations were generated across 13 batches. Box plots show the median (centre line), with boxes representing the interquartile range (25th–75th percentiles). Whiskers extend to the most extreme data points within 1.5× the interquartile range from the box, and points beyond this range are shown as outliers. Individual data points are overlaid. The mean is indicated by an “X”. **b**. Estimation of molarity based on fluorescence measured in our context. Y-axis displays the fluorescence of reference recombinant eGFP protein (ref, 95% purity) and X-axis shows the concentration (pmol/µl), values on the measurements are for the amount of protein used in the 10 µl volume. Source data

In total, 139 of the 331 Codebook putative TFs had at least one successful GHT-SELEX experiment, of which 131 were also successful in HT-SELEX, 108 in ChIP–seq and 102 in all three (Fig. 3a and Supplementary Table 3). The 139 were comprised mainly of C2H2-zf proteins, which are prevalent in the Codebook set (Fig. 3b). A total of 24 types of DBD were present among the successful experiments (Fig. 3b), illustrating that the method can capture motif-containing genomic target site locations of diverse TF families. An additional 163 of the putative TFs did not yield motifs in any of these three assays despite the use of several expression systems and several constructs (Supplementary Table 6). Successful and unsuccessful proteins produced as enhanced green fluorescent protein (eGFP) fusions yielded largely overlapping distributions of fluorescence signals and estimated protein concentrations (Extended Data Fig. 2 and Supplementary Table 3) and, on western blots, ~90% of sampled proteins exhibited a single prominent band at the correct molecular weight (Supplementary Table 7 and Supplementary Figs. 1 and 2) for both successful and unsuccessful samples, suggesting that most are expressed and properly folded. It is possible that some of the putative TFs require post-translational modifications or cofactors, that our data analysis, motif derivation and testing methods did not capture their specificity or that they are false-negatives for some other reason. However, many of the tested putative TFs may not bind DNA with sequence specificity, as only an additional nine were successful in other assays used in the Codebook project (SMiLE-seq and Protein Binding Microarrays)^45^ (Supplementary Table 6). If we assume the remainder do not bind DNA, then the success rate of GHT-SELEX on the Codebook proteins is 78% (139 of 177). Regardless, a central conclusion of this study is the identification of binding motifs for more than 100 poorly characterized human TFs. For most of them, we produced three types of data (HT-SELEX, GHT-SELEX and ChIP–seq) that provide differing perspectives on their sequence specificity and DNA binding.

### Unexpectedly high overlap between TF binding to the genome in vitro and in vivo

**Fig. 4:**
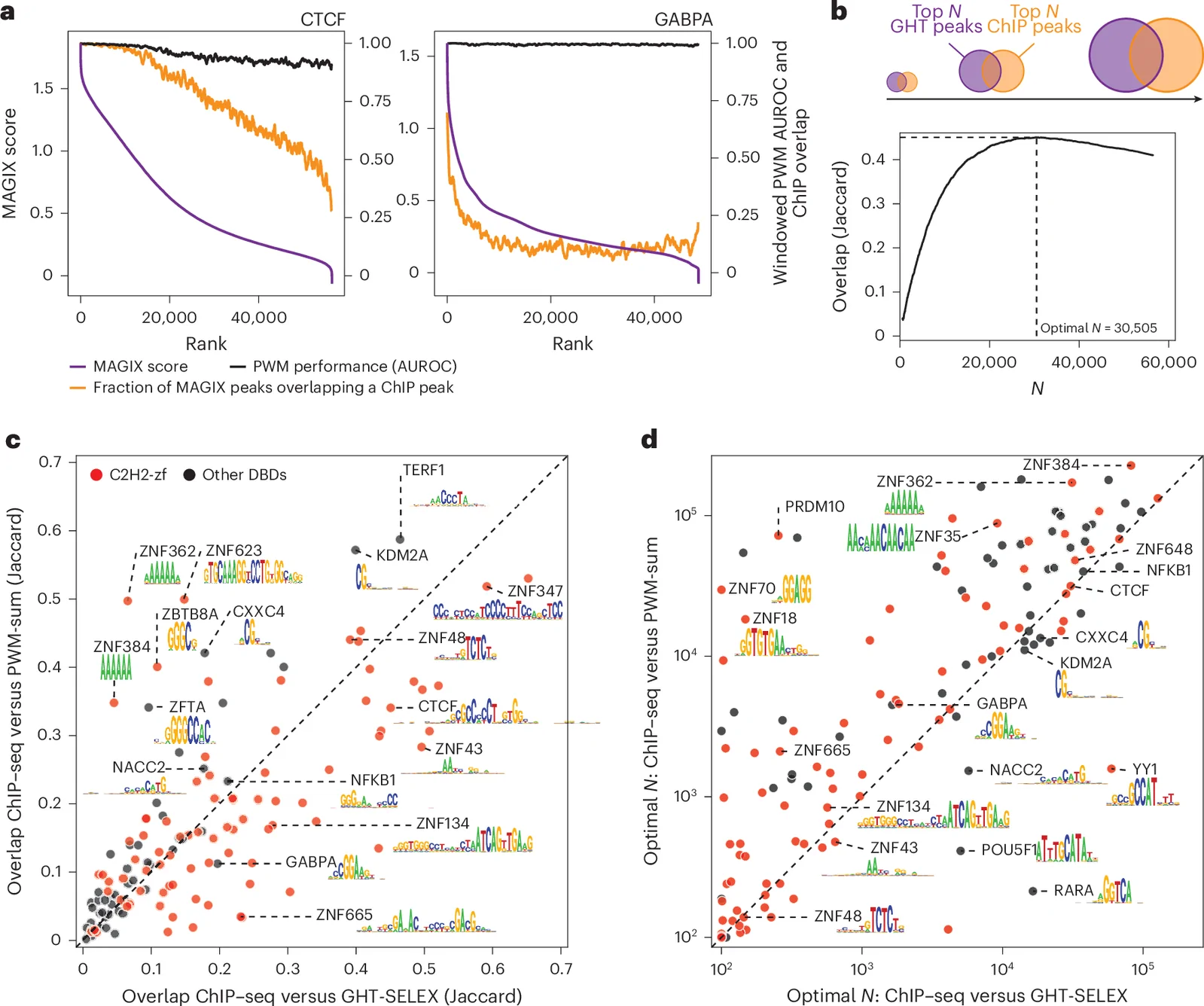
Correspondence between GHT-SELEX, ChIP–seq peaks and PWM predictions. **a**, Enrichment of ChIP–seq peaks and PWM hits within MAGIX peaks, for two example control TFs. MAGIX peaks are sorted by their MAGIX score (purple, left *y* axis). Orange line: proportion of peaks (in a sliding window of 500 peaks over the ranked peaks, with a step size of 50) that overlap with a ChIP–seq peak defined using MACS (*P* < 0.001; background signal modeled using a Poisson distribution). Black line: AUROC for PWM affinity scores (calculated by AffiMx^52^) of MAGIX peaks in the same window versus 500 random genomic sites. **b**, Illustration of peak number optimization (for CTCF as an example). **c**, Scatter plot of optimal Jaccard value between GHT-SELEX peaks and ChIP–seq peaks (*x* axis) versus optimal Jaccard value between PWM-predicted sites and ChIP–seq peaks (*y* axis), for all 137 TFs (dots). **d**, Scatter plot of optimal *N* (peak number) for the peak set comparisons shown in **c**. Source data

We next sought to quantify the overlap between GHT-SELEX/MAGIX peaks and ChIP–seq peaks. For 137 TFs with GHT-SELEX data, encompassing 101 Codebook and 36 control TFs, ChIP–seq data in HEK293 cells were also available from a parallel Codebook study^48^, which used heterologous expression in HEK293 cells. Scoring peak overlaps requires first defining peak sets, which is typically accomplished using a threshold on enrichment values or statistical tests rather than biological relevance. GHT-SELEX, like ChIP–seq, produces peaks with a continuum of enrichment coefficient values and other associated statistics, rather than a bimodal distribution that would discriminate more readily bound from unbound loci. The overlap with ChIP–seq peaks and the enrichment of PWM scores within peaks were also generally continuous (examples in Fig. 4a; distributions for all TFs in Supplementary Data 3). Moreover, the MAGIX score and associated FDR values at which the overlap of GHT-SELEX peaks with either ChIP–seq peaks or PWM matches dropped to random values varied dramatically between TFs, such that no unifying thresholds emerged that could be generally applied convincingly to all TFs. We therefore implemented a system to draw thresholds on the GHT-SELEX and ChIP–seq peak sets simultaneously, and individually for each TF (Fig. 4b). This system identifies thresholds on a TF-by-TF basis by maximizing the overlap statistic (the Jaccard coefficient) between the in vitro and in vivo peak sets (Fig. 4b and Methods). We reasoned that this approach would account for unobserved TF-specific parameters in both GHT-SELEX and ChIP–seq assays, including different binding kinetics for both sequence-specific and nonspecific DNA binding, the effective concentration of the TF and the ability of the TF to compete or cooperate with nucleosomes and other cofactors in vivo.

**Extended Data Fig. 3:**
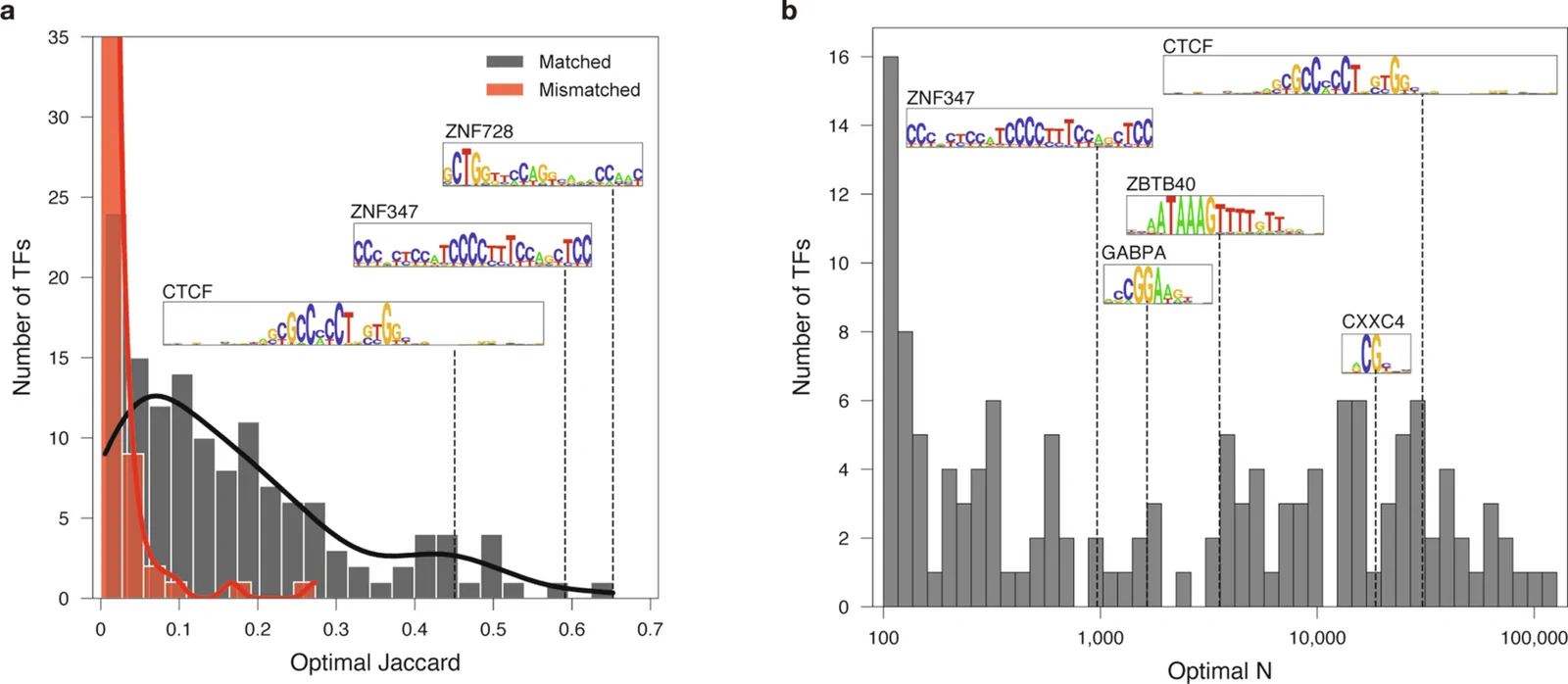
Optimal Jaccard and number of peaks between ChIP-seq and GHT-SELEX. **a**. Histogram of optimal Jaccard values, compared to the maximum Jaccard for mismatched TFs (that is between GHT-SELEX for one TF and ChIP-seq for a randomly selected TF). **b**. Histogram of the optimal values of N (peak count) for the 137 TFs that have both GHT-SELEX and ChIP-seq peaks. Source data

This approach yielded a very striking result, which is that, for many TFs, a peak number can be identified with a surprisingly high Jaccard value (median 0.127 over all 137 TFs) (Extended Data Fig. 3a,b and Supplementary Table 8), meaning that almost 30% of GHT-SELEX peaks at the chosen threshold overlap with a ChIP–seq peak and vice versa. In permuted experiments (that is, mismatched TFs), the median Jaccard value is 0.00254 (Wilcoxon *P* = 1.3 × 10^−39^) (Extended Data Fig. 3a). Using the same optimization process, the median Jaccard value was 0.56 for ChIP–seq replicates^48^; this number presumably represents an upper bound on experimental reproducibility. Overall, this outcome indicates that many TFs intrinsically (that is, independently) specify many of their in vivo binding sites. We do not expect the overlap to be perfect due to the impact of chromatin and cofactors, but this result nonetheless contrasts with the traditional expectation that an individual TF would not be able to specify independently the vast majority of their DNA targets in a genome^7^. The peak numbers yielding these high Jaccard values are often relatively low (Extended Data Fig. 3b), indicating that the number of in vivo binding sites that are specified intrinsically by TFs is relatively small.

One potential explanation for this high overlap between GHT-SELEX and ChIP–seq peaks is that the Codebook dataset is dominated by C2H2-zfs, which often have long sites with high information content. Indeed, among TFs with Jaccard >0.1, 80% are C2H2-zf proteins (63 of 79), versus 34% (20 of 58) for those with Jaccard <0.1 and, overall, the median Jaccard value for C2H2-zf proteins is 0.1836, versus 0.0559 for non-C2H2-zf proteins (Wilcoxon *P* = 6.08 × 10^−9^). CTCF—a control TF known to possess a large number of genomic target sites, unusually high intrinsic sequence specificity, and ability to control nucleosome positions^15,16^—is among those with high Jaccard values (0.45), although it is not the highest scoring in this dataset, and overall is clearly not unique in its capacity to independently specify where it binds in living cells. Counterintuitively, however, high Jaccard maxima were also obtained for a subset of TFs with relatively short motifs, including TERF1, ZNF48, ZBTB8A, NACC2 and several CXXC proteins, such as CXXC4 and KDM2A, which bind essentially CG dinucleotides^52^ (Fig. 4c).

### Several explanations for the high sequence specificity observed in GHT-SELEX

The traditional assumption that individual TFs have relatively low ability to specify their genomic targets is based largely on the results of motif scans^7^, which are dependent on the accuracy of the PWM. To revisit this assumption, we performed a maximization of the overlap between the Codebook PWM matches across the genome and the ChIP–seq peaks, using the same Jaccard-based method described above (Methods). We found that the overlap between PWM predictions and ChIP–seq peaks is, in fact, similar to the overlap between GHT-SELEX/MAGIX and ChIP–seq peaks (Fig. 4c and Supplementary Table 8). The number of peaks at which the maximum Jaccard was obtained is also typically similar (Fig. 4d). Thus, PWMs are often more accurate than generally believed and comparable to the GHT-SELEX data. It is important to note that we selected the Codebook PWMs for their accuracy across datasets, that is, other PWMs for the same TF would display lower overlap. Therefore, the PWM derivation method, including the experimental data used, is critical for representing TF binding in vivo.

**Extended Data Fig. 4:**
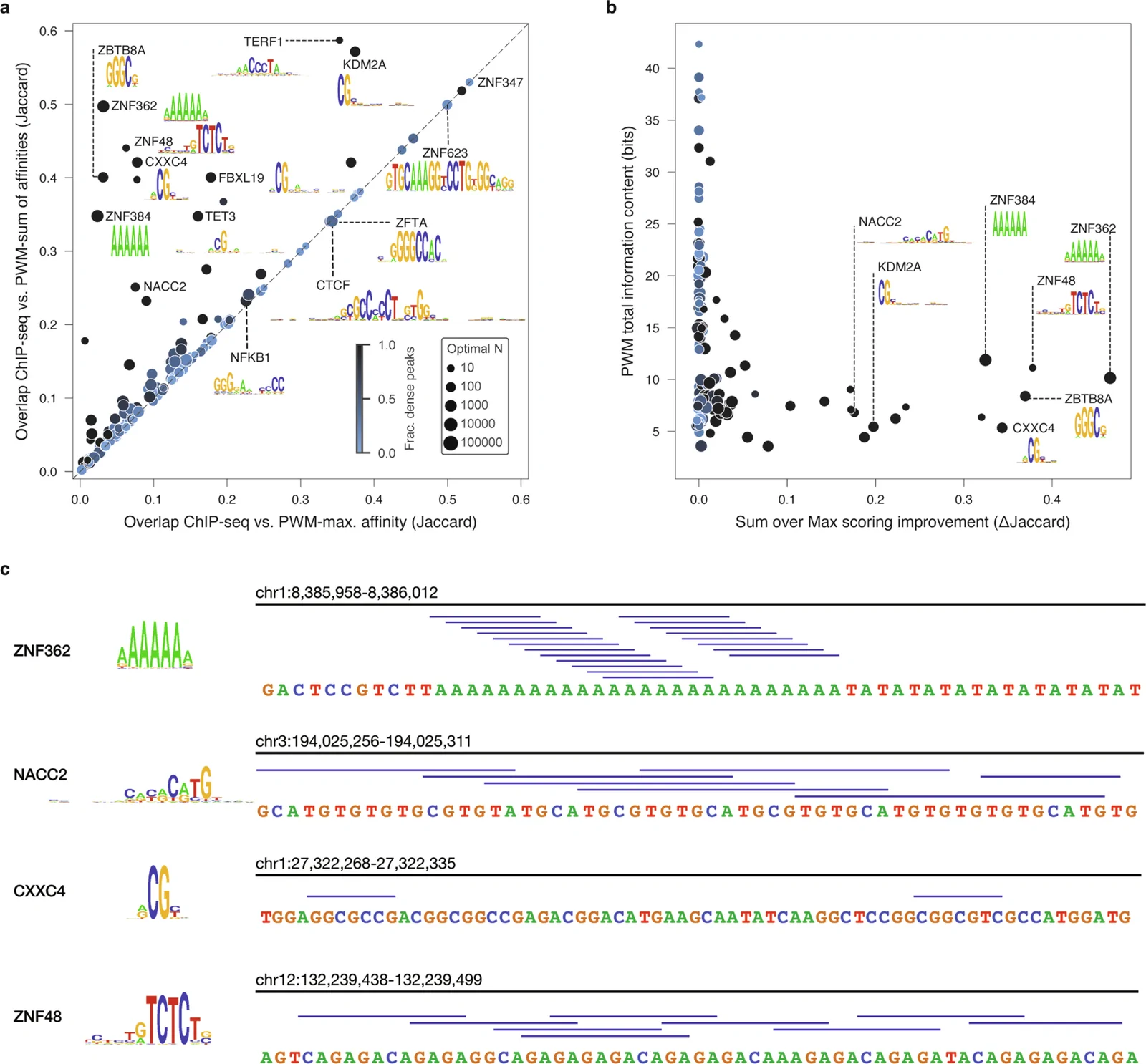
Comparison of PWM binding site prediction methods and the importance of multimeric binding. **a**. Scatter plot showing optimal Jaccard value between PWM-predicted sites and ChIP-seq peaks, for maximum-affinity PWM scoring and sum-of-affinities PWM scoring. Points (TFs) are scaled based on the optimal number of peaks (in the sum scoring), and the color reflects the fraction of binding sites comprised of multiple PWM hits. **b**. Scatter plot of the improvement in the optimal Jaccard value associated with sum-of-affinities PWM scoring vs. information content of the PWM. Points’ size and color are the same as panel (**a**). **c**. Examples of four TFs with multiple motif matches within a single ChIP-seq peak. Source data

We also found that the motif scanning method is important to achieve the highest overlap between PWM matches and ChIP–seq peaks. Consistent with a recent report^53^, for some TFs, the sum of predicted affinity scores over a sequence window (the sum of the PWM probability scores at individual positions), results in a considerably higher maximum Jaccard value than taking the maximum or sum of log-odds PWM scores (which are output by most PWM scanning tools and generally thought to represent binding energy^54,55^) (Extended Data Fig. 4a). This sum-of-affinity scoring (also known as sum-occupancy^56^) presumably reflects the cooperation of several adjacent binding sites, traditionally referred to as ‘avidity’^57^. The effect is most striking for a subset of TFs that bind short or repetitive sequences, including CG dinucleotides and poly-A stretches (Extended Data Fig. 4b), but it also seems to underpin the specificity of NACC2 and ZNF48, which have unique, nonrepetitive motifs (Extended Data Fig. 4c). Points above the diagonal in Fig. 4c, in which PWM prediction shows higher overlap with ChIP–seq than the GHT-SELEX, may therefore represent the impact of TF binding sites over a larger window, which would be detected in ChIP–seq but not GHT-SELEX (ChIP–seq fragments are 100–300 bp, and the PWM scans are performed with a 200-bp window, whereas the GHT-SELEX fragments are only ~65 bp). For example, scanning 200-base windows with the short CG motif for CXXC4 may be better suited for the detection of CpG islands (which dominate the CXXC4 binding sites^45^), in which the CG dinucleotides will be distributed over a large region (by definition ≥200 bp).

In contrast—and despite evaluation of hundreds to thousands of PWMs for each TF—there were still many TFs for which we could not derive a PWM that rivals GHT-SELEX data in correspondence to ChIP–seq peaks (those below the diagonal in Fig. 4c). These TFs are almost entirely proteins with a long array of C2H2-zf domains, which we examine more closely in the next section.

### Alternate usage of C2H2-zf domains within large arrays

**Fig. 5:**
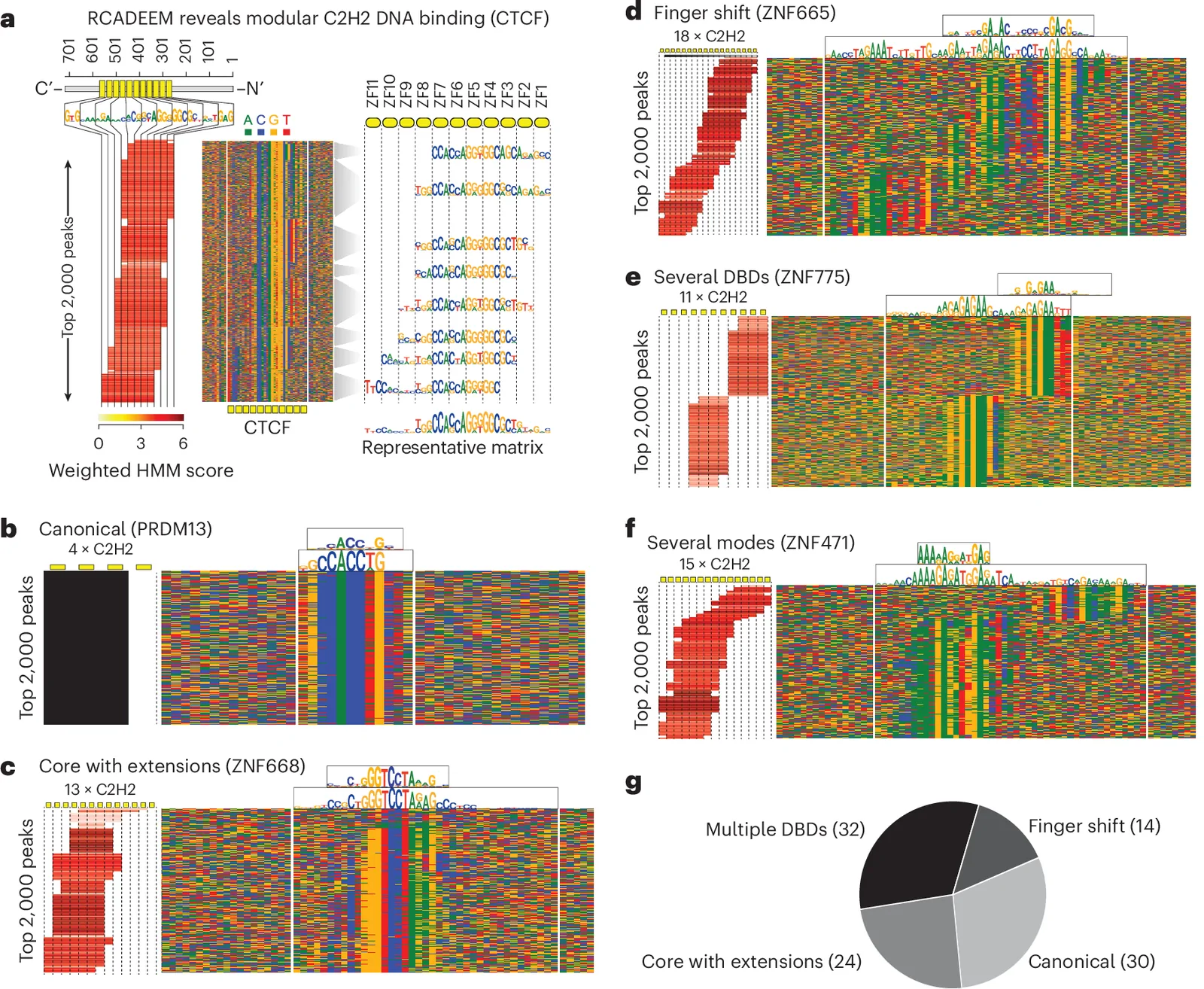
Alternative engagement of individual C2H2-zf domains at genomic binding sites inferred from the recognition code. **a**, RCADEEM applied to CTCF. Middle: top 2,000 nonrepetitive GHT-SELEX peaks. White vertical bars: region expected to contact the DNA based on the assumption that each of the C2H2-zf domains defines three contiguous bases. Sequences are aligned against the closest match to the recognition code (their assumed binding register relative to the C2H2-zf domains). Left: C2H2-zf domains inferred to engage each DNA sequence, which is used to determine the row order in the figure. Right: motifs for the main subsites, derived from base frequencies in the sequence alignment. **b**–**f**, Top 2,000 nonrepeat peak sequences, as in **a**, for representative TFs with different binding modes (canonical (**b**), core with extensions (**c**), finger shift (**d**), several DBDs (**e**) and several modes (**f**)), as described in the main text. Above each is shown the sequence logo for the single representative Codebook PWM (top) and a motif generated by RCADEEM that represents all of the observed sequences (bottom). **g**, Number of occurrences of each category among all 86 C2H2-zf proteins for which RCADEEM converged; note that a TF might appear in several or no categories. Source data

The expansive collection of GHT-SELEX, HT-SELEX and ChIP–seq data for C2H2-zf proteins provided an opportunity to examine the long-standing issue of the usage of individual C2H2-zf domains within large arrays. Proving differential engagement of the specific C2H2-zf domains is challenging due to low statistical power (there are many possible C2H2-zf sub-arrays, and a limited number of highly enriched peaks) and the fact that the genome is highly nonrandom and repeat-rich. To minimize the impact of these issues, we developed a new method that utilizes the C2H2-zf recognition code to assess which sets of C2H2-zf domains are likely to be engaged at any individual binding sites; that is, given the C2H2-zf protein sequence and a set of binding sites, it outputs an alignment of the binding sites to C2H2-zf array, using recognition code predictions. It also provides an estimate of whether each C2H2-zf domain is utilized in selecting each site. We call this method recognition code-assisted discovery of regulatory elements by expectation-maximization (EM) (RCADEEM) (Methods). Figure 5a shows a schematic and the results of applying RCADEEM to CTCF, illustrating that, as in previous findings^31^, it produces a ‘core’ motif recognized by fingers 4–7 at all sites, and alternative usage of flanking C2H2-zf domains in a subset of sites.

We applied RCADEEM to all 120 C2H2-zf proteins for which we had successful data from GHT-SELEX (Supplementary Table 9). We applied RCADEEM on GHT-SELEX data and separately, if available, on HT-SELEX and ChIP–seq; for GHT-SELEX and ChIP–seq, we applied RCADEEM both with and without repeat sequences (that is, removing any peaks that overlap with the UCSC RepeatMasker track). In total, we obtained RCADEEM predictions for 86 of these TFs (Supplementary Table 9), which are available in the accompanying web resources (https://codebook.ccbr.utoronto.ca/). (For the remaining 34, the algorithm did not converge, suggesting that the sequence preferences of the protein do not closely follow the recognition code.) Most of the 86 displayed apparent alternative usage of segments of the C2H2-zf domain array on different DNA molecules (for example, different genomic loci) within the same experiment (Supplementary Data 4). These patterns could not be accounted for by truncations or other potential artifacts (Supplementary Figs. 1 and 2).

We classified the C2H2-zf domain usage manually into the following categories (Fig. 5b–g): (1) canonical (30 instances); the same set of C2H2-zf domains is used at all sites. (2) Core with extensions (24 instances); a sequence motif found at all sites, corresponding to a subset of the C2H2-zf domains, is supplemented by recognition of flanking sequences by adjacent C2H2-zf domains at some sites. (3) Finger shift (14 instances); a range of tiled target sites corresponding to variable subsets of adjacent C2H2-zf domains. (4) Several DBDs (32 instances); subsets of the C2H2-zf domain array function as independent DBDs. The last three binding modes are not mutually exclusive. For example, ZNF471 displays both several DBDs and core with extensions with one of the DBDs (Fig. 5f), whereas the long finger shift in ZNF665 (Fig. 5d) leads effectively to several DBDs, as the target sites of most N-terminal and C-terminal ends do not overlap with each other. Supplementary Table 9 lists the annotations for all 86 proteins.

### Evolution of C2H2-zf protein DNA-binding specificities through internal duplication

**Extended Data Fig. 5:**
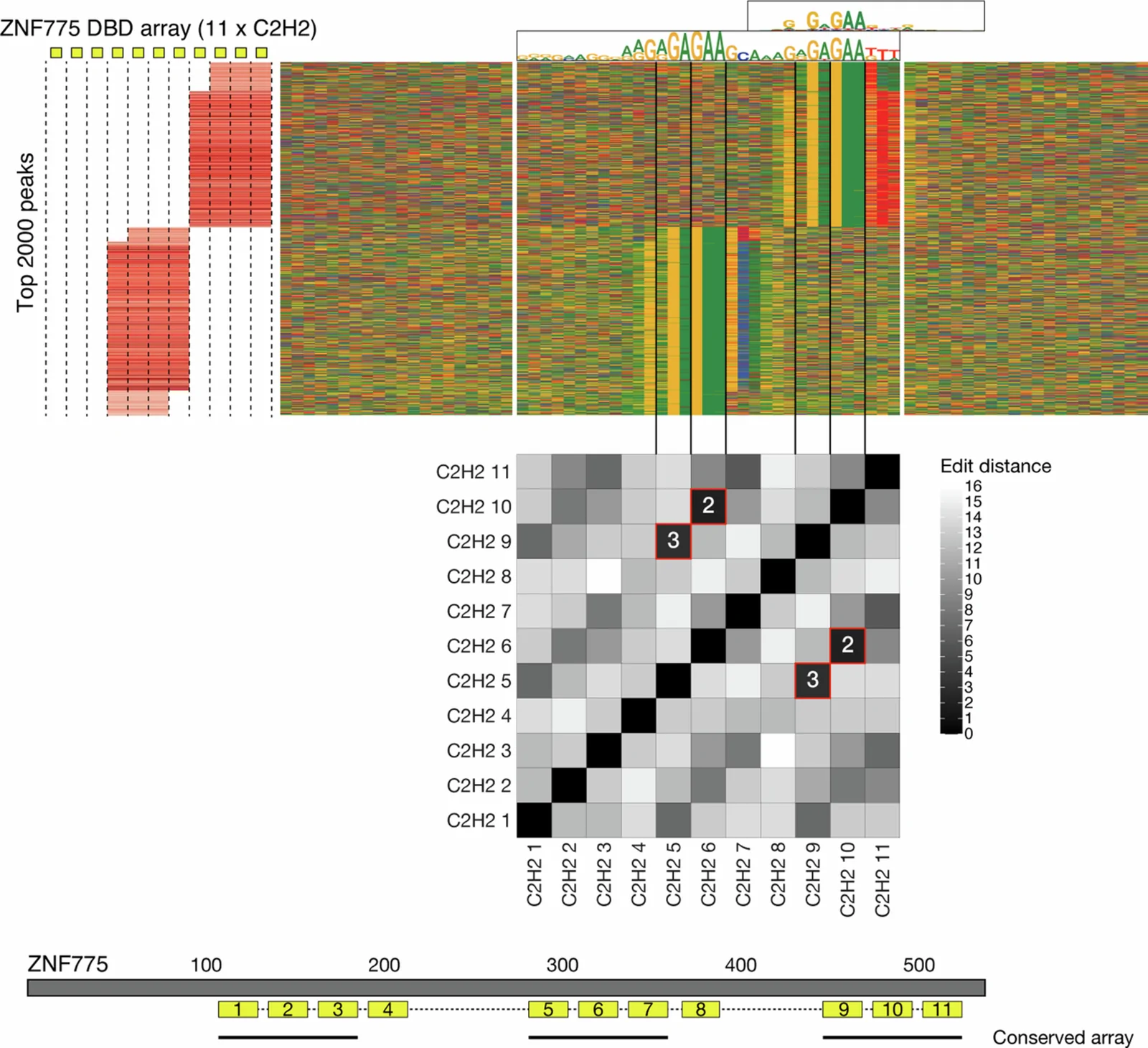
DBD array duplications in ZNF775. (*top*) RCADEEM diagram, as in Fig. 5, with additional markup to indicate the regions predicted to be bound by the most conserved pair of adjacent domains. *(middle)* Similarity of ZNF775 C2H2-zf domain amino acid sequences based on the number of mismatches in the global alignment. Off-diagonal comparisons with ≤ 3 mismatches are indicated with a red border and labeled with the number of mismatches. (*bottom*) Positions of the C2H2-zfs within the full-length protein, showing the physical separation of the internally duplicated conserved arrays.

In the RCADEEM outputs, different segments of a C2H2-zf domain array (that is, different DNA binding regions of the protein) are often predicted to bind similar yet distinct sets of sequences. For example, ZNF775 (Fig. 5e) binds two types of site that contain a shared GNWGAA consensus, followed by either TTT or GCA trinucleotides. RCADEEM predicts that these two sites are recognized by C2H2-zf domain arrays 1–4 and 5–8, respectively. Indeed, arrays 1–3 and 5–7, as well as 9–11, are homologous, on the basis of sequence identity (Extended Data Fig. 5) suggesting that they arose from duplications. All three arrays are present in Tasmanian devil, indicating that the duplications predate divergence from marsupials and have since been conserved. The cellular and physiological functions of this protein are unknown, to our knowledge, but the high conservation suggests an important role across mammals.

**Fig. 6:**
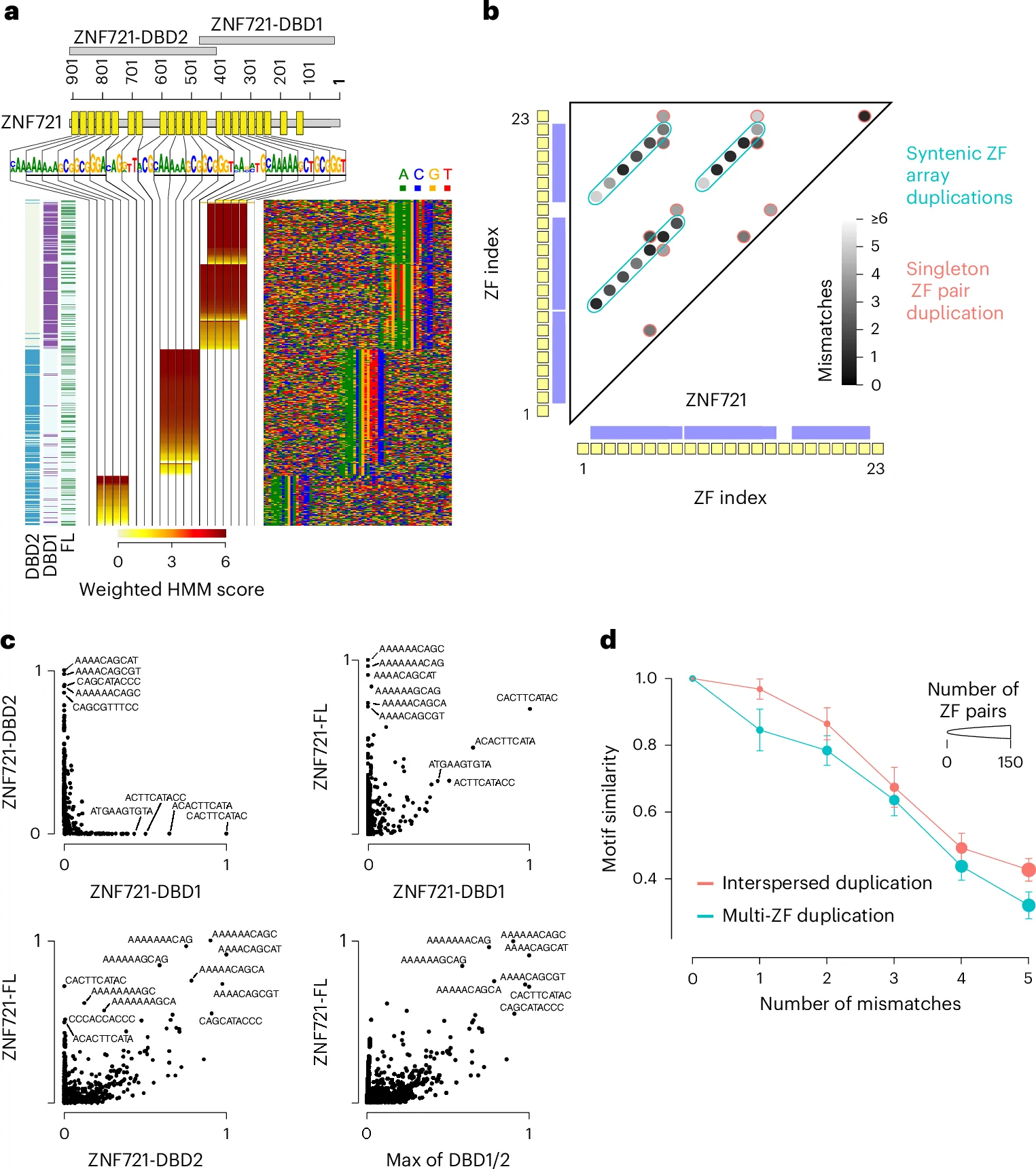
Evolution of C2H2-zf protein DNA-binding specificities through internal duplication of DBDs and DBD arrays. **a**, RCADEEM results for the pool of top 500 peaks from full-length ZNF721 and two DBD constructs, after removing peaks that overlap repeats. The construct in which the peak was observed is indicated on the left. **b**, Similarity of C2H2-zf domains of ZNF721 (based on the number of mismatches in the global alignment). Circled in blue: apparent duplicated arrays (syntenic duplications); circled in red: single pairs that may also be duplicates. **c**, Scatterplots of the HT-SELEX *k*-mer scores^63^ (relative counts) across the three ZNF721 constructs **d**, Comparison of average per-base similarity (correlation of nucleotide frequency) in PWMs predicted by the recognition code, for those present in duplicated arrays versus those duplicated as individual C2H2-zf domains, with duplicated C2H2-zf domains taken as pairs that are separated from each other by five or fewer edits. DBD pairs have been filtered to contain only combinations where both DBDs are likely to have retained their ability to bind DNA (have a DNA binding functionality score^64^ > 0.5). Error bars: s.e.m.; dot size reflects the number of observations. Total number of pairwise comparisons for different numbers of mismatches: (0) 6, 15; (1): 14, 24; (2) 20, 47; (3) 50, 61; (4): 87, 104;(5): 156, 119, for interspersed and array located pairs, respectively. Source data

Another example is ZNF721: RCADEEM indicates that it has three DNA-binding modes, with related but distinct motifs (Fig. 6a), corresponding to homologous C2H2-zf domain arrays containing fingers 6–13, 12–16 and 18–22 (Fig. 6b). The distinct sequence preferences of the duplicated ZNF721 arrays are supported by experimental data for partial ‘DBD1’ and ‘DBD2’ constructs, corresponding roughly to the first and second half of the full array, which recognize largely distinct subsets of the genomic sites bound by full-length TF in GHT-SELEX (Fig. 6a) and prefer almost entirely distinct ten-mers in HT-SELEX (Fig. 6c). The function of ZNF721 has not been determined, but sequences recognized by the first (6–13) and third (18–22) duplicated C2H2-zf domain arrays of ZNF721 are found in the highly numerous Alpha repeats, which are fast-evolving elements found at primate centromeres^58^. ZNF721 itself is present only in primates. ZNF721 also binds thousands of unique loci outside known repeat elements, and associates physically with TRIM28/KAP1 (ref. ^59^), suggesting a role in gene silencing or heterochromatin formation.

To survey the prevalence of internal duplication of C2H2-zf domains, we compared all pairs of individual human C2H2-zf domains occurring in the same protein and found that 185 human C2H2-zf proteins (~25%) contain at least one pair of C2H2-zf domains that differ by three or fewer edits (substitutions, deletions or insertions; Supplementary Table 9), indicating that they are derived from recent duplications. Furthermore, as in ZNF775 and ZNF721, there are 140 proteins with apparent internal C2H2-zf domain array duplications, defined as two (or more) adjacent C2H2-zf domains (that is, an array) related to a second such array of C2H2-zf domains, with five or fewer edits per C2H2-zf domain. Based on recognition code predictions, C2H2-zf domains within internal array duplications have more diverged sequence specificities from each other than individually duplicated C2H2-zf domains (Fig. 6d and Supplementary Table 10). The prevalence and diversification of internal C2H2-zf domain array duplications suggest that they are a common modality for evolution of new functional roles for this large class of proteins.

## Discussion

GHT-SELEX analyzes direct binding of individual TFs to the unmethylated and unchromatinized genome in vitro, revealing surprisingly specific intrinsic sequence preferences for many human TFs. The unexpectedly high overlap between ChIP–seq, GHT-SELEX and PWM scans could be explained partly by technical shortcomings in standard PWM-based genome scans. HT-SELEX and other in vitro approaches utilizing random sequences are unbiased in terms of sequence composition^60^, but are inherently limited in sequence length and context that can be surveyed, limiting motif discovery. ChIP–seq does not inherently discern among direct, indirect and nonspecific binding, which can also bias motif discovery. GHT-SELEX provides a powerful intermediate.

GHT-SELEX is particularly effective with C2H2-zf proteins and, together with RCADEEM, has an unprecedented ability to both obtain and dissect in vitro the several binding modes that are characteristic of this family, and inherently more difficult to represent as a single PWM. The existence of several binding modes also provides a potential explanation for the large number of C2H2-zf domains in each protein. These large arrays are often derived from internal duplications of segments of the C2H2-zf arrays, possibly facilitating generation of evolutionary novelty through duplication and divergence.

The high GHT-SELEX versus ChIP–seq peak overlaps observed here are not inconsistent with previous observations. The Codebook TFs are, by definition, depleted for the most well-studied TFs, often containing homeodomain, bHLH, bZIP, nuclear receptor and Sox domains, which are typically conserved, and often dictate specific biological processes (for example, morphogenesis, body plan, lineage specification, and so on)^27^. TFs in these classes were mainly controls (for example, LEUTX, BATF2, RARA and SRY), and they displayed only limited overlap between GHT-SELEX and ChIP–seq peaks, indicating that many of them cannot specify in vivo binding locations, and hence target genes, independently. It has long been known that TFs controlling chromatin in yeast are largely distinct from those that regulate specific pathways; we speculate that a similar division may exist in humans and other animals.

GHT-SELEX data, together with the larger Codebook dataset, provide an extensive new dataset of TF motifs (that is, PWMs), encompassing most putative TFs currently lacking them. The accompanying papers provide a thorough analysis of the results of this project, underscoring many challenges and benefits of accurate motif representations. Representation of TF sequence specificity remains an open challenge, more than four decades after the introduction of the standard PWM model^61^. The data here clearly support other recent studies^24,53^ showing that clusters of simple sequence motifs, which are common in genomic sequences, strongly promote binding both in vitro and in vivo.

More accurate representations of multimeric binding and large and complex binding sites, in particular for C2H2-zf proteins, will be useful for a variety of purposes. We propose that obtaining data from GHT-SELEX for additional TFs with ‘known’ motifs and genomic binding sites from ChIP–seq will produce a more detailed view of their intrinsic DNA binding abilities and how this intrinsic ability dictates TF-genome interactions in living cells.

## Methods

### TFs and constructs

Selection of TFs, design of constructs for gene synthesis and expression vectors are described in detail in the accompanying study^45^. Briefly, for each TF, the inserts contained the full sequence of a representative isoform, and either all or a subset of its predicted DBDs. Inserts were cloned into up to three types of expression vector, to enable production by three different expression systems (N-terminal eGFP fusions in both wheat germ extracts and HEK293 cells, and N-terminal GST fusions in *E. coli* extracts). In total, we generated 1,315 constructs encompassing the 61 control TFs and 331 of the 332 putative TFs in the Codebook set of poorly characterized proteins. Sequences and other information are available as described below in ‘Data Availability.’

### Protein production and quality control

We used three protein expression systems, which we refer to in Supplementary Table 2 and below as Lysate, in vitro transcription–translation (IVT) and eGFP-IVT, respectively. The Lysate system used recombinant HEK293 cells, created in the accompanying study^48^, and in a previous study^49^, which express eGFP-tagged full-length proteins from a Tet-inducible promoter (plasmid backbones pTH13195 (ref. ^49^) and pTH12027). We induced expression by doxycycline treatment for 24 h before harvest and confirmed by fluorescent microscopy. Whole cell lysates were then harvested from a 10-cm plate (~10 million cells) for each line using 1 ml of lysis buffer (50 mM Tris-Cl at pH 7.4 containing 150 mM NaCl and 1% Triton X-100), supplemented with protease-inhibitor cocktail (Roche cOmplete mini, cat. no. 04693159001). Each of the SELEX cycles used 50 µl of lysate. IVT used an in vitro transcription–translation reaction (PURExpress In Vitro Protein Synthesis Kit, NEB, cat. no. E6800L) to express T7-driven, GST-tagged proteins (either full-length or DBDs) (plasmid backbone pTH6838 (ref. ^51^)). eGFP-IVT uses the TNT SP6 high-yield wheat germ protein expression system (Promega, cat. no. L3260) to express SP6-driven, eGFP-tagged proteins (either full-length or DBDs) (plasmid backbone pTH16505, an SP6-promoter driven, N-terminal eGFP-tagged vector, modified from pF3A–eGFP^47^ to contain *Asc*I and *Sbf*I restriction sites after the eGFP). For IVT and eGFP-IVT production systems, we performed reactions according to kit instructions, but using a smaller volume: 7.5 μl of IVT or 5 μl of eGFP-IVT reaction sample was used in each binding reaction of each SELEX cycle. Fluorescence levels and estimated protein concentrations shown in Supplementary Table 3 and Extended Data Fig. 2 were measured using a BIOTEK Synergy Neo microplate reader from 10 μl volume of lysate or wheat germ extracts on a conical transparent 96-well plate using bottom optics and with a gain setting of 85. Protein concentration was estimated by making a dilution series of a reference eGFP protein (ProsPec (PRO-1606), >95% purity based on high-performance liquid chromatography and SDS–polyacrylamide gel electrophoresis) into the wheat germ IVT expression mixture, measuring the fluorescence in same way as the samples and performing linear interpolation. Reference eGFP measurements were performed more than 3 years after measurements of the samples and, due to some unknown difference, they displayed higher background fluorescence in the blank wells. We adjusted for this change by using the median of the lowest 5% of the experiments as the blank value and subtracting the difference between reference blank and lowest 5% blank measurements from the reference eGFP values. This was performed separately for eGFP-IVT and lysate samples (Supplementary Table 11 and Extended Data Fig. 2b). A subset of these proteins was also analyzed on western blots (Supplementary Table 6 and Supplementary Figs. 1 and 2), with the same anti-GFP antibody (cat. no. ab290, Abcam) as used in SELEX, and a secondary horseradish peroxidase fusion antibody (Anti-Rabbit IgG HRP Conjugate, cat. no. W401B, Promega) and Immobilon Western Chemiluminescent HRP Substrate (cat. no. WBKLS0050, Millipore Sigma). Blots were imaged with Odyssey Fc Imager (LICORbio).

### GHT-SELEX and HT-SELEX library preparation

We fragmented HEK293 genomic DNA (Genscript; cat. no. M00094) for 45 min using NEBNext dsDNA Fragmentase enzyme mix (NEB, cat. no. M0348S), and then performed a size selection step to reduce the amounts of fragments larger than 200 bp. In the size selection we added 0.9× volume of bead suspension (magnetic SPRI beads, supplied with the kit, NEB, cat. no. E7103S) to the fragmented DNA, mixed the reaction for a minute and then removed the large DNA fragment bound beads with a magnet, after which we diluted the supernatant 5× with water, followed by purification with a PCR purification kit (NEB, cat. no. T1030S), to recover fragments as small as 25 bp. Next, the fragments were converted to an Illumina sequencing compatible library using NEBNext Ultra II DNA Library Prep kit (NEB, cat. no. E7103S) and NEB E7350 adapters. After adapter ligation, we purified the library with a PCR purification kit (NEB, cat. no. T1030S) and then amplified it for five PCR cycles to convert the partially single-stranded adapter flanks to fully double-stranded DNA, to increase the amount of the product and reduce the amount of methylated cytosine residues in the initial library. The 96 HT-SELEX ligands were prepared as described through annealing two oligonucleotides on 3’ regions and extending, followed by PCR amplification^65^, with the exception that the reverse primer was replaced with a primer (5’-CTGGAGTTCAGACGTGTGCTCTTCCGATCT-3’) that does not contain a T7 promoter sequence. HT-SELEX ligands differ from each other by containing a well-specific variable region that flanks the randomized 40 bases indicated in the name of the experiments (for example, AA40NCCAGTG contains 40 bases flanked by AA and CCAGTG sequences and partial Illumina adapter sequences). All primers and library preparation schemes are given in Supplementary Table 1.

### HT-SELEX and GHT-SELEX analysis overview

**Extended Data Fig. 6:**
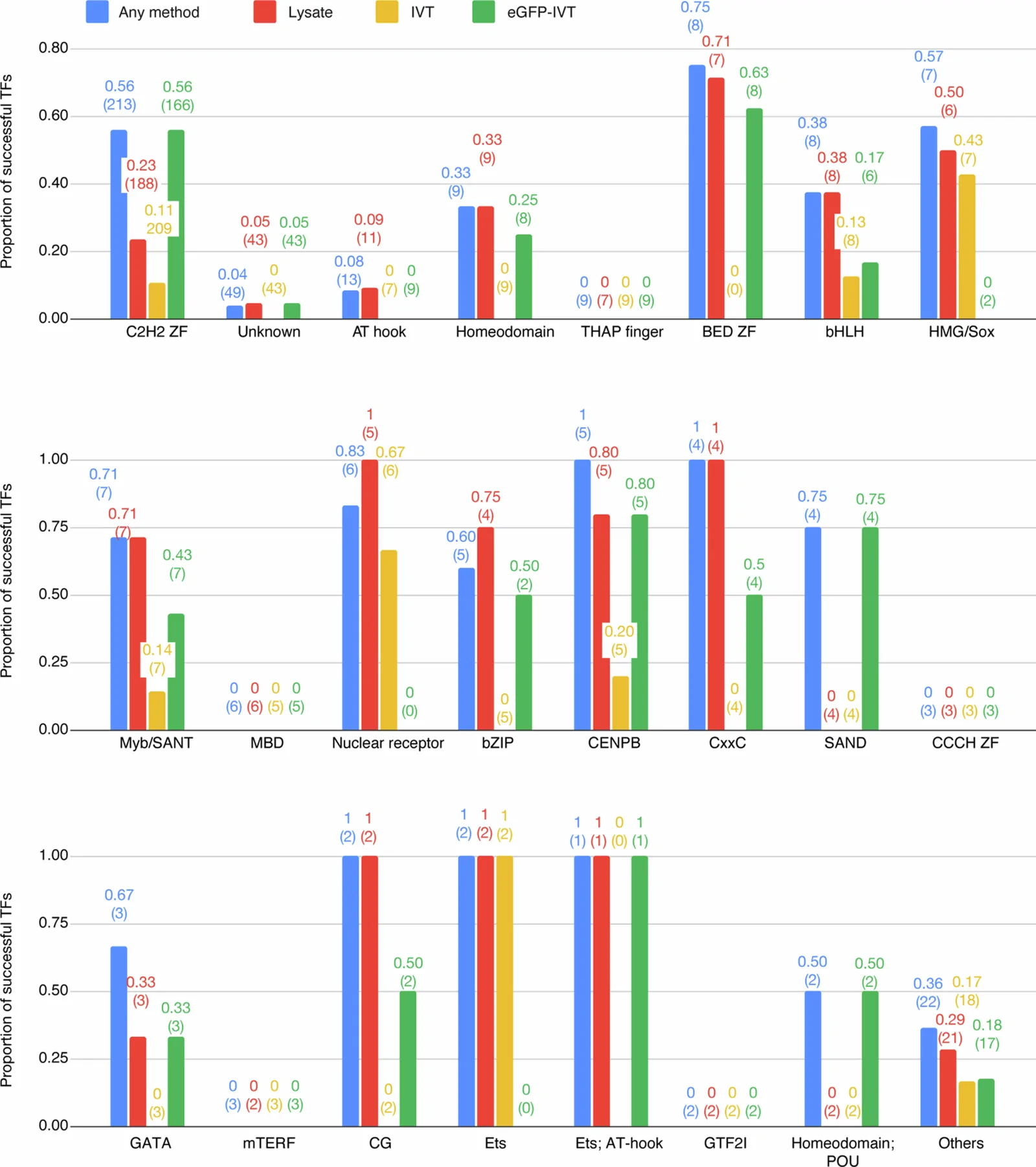
TF success rate by protein production method and structural class. Each set of four bar graphs shows success rates for one type of DNA-binding domain, as indicated. The bars show the proportion for all protein production methods combined (blue), and the three individual methods (other colours, as indicated). The proportion and absolute numbers are also given numerically, superimposed over the bars. Source data

We performed a total of 1,534 GHT-SELEX and 1,578 HT-SELEX experiments (Supplementary Table 3). We performed 21 different batches of SELEX, which varied to accommodate the three protein production systems and in which we modulated several experimental variables over the course of the study (the number of washes, and digestion of single-stranded DNA with mung bean nuclease between cycles) in an effort to improve success rates. The protein production method seems to be most critical, particularly for C2H2-zf domain arrays (56%, 17% and 10% success rates using wheat germ extract, mammalian cell and *E. coli* extract-based expression systems, respectively; see Supplementary Table 2 for description of conditions used in each experimental batch and Extended Data Fig. 6).

The number of experiments per putative TF varied due to the heterogeneity of the TFs and consequently heterogeneity in the clone collection; for example, small proteins were represented with only a single insert, whereas larger, multi-DBD proteins were represented with both full-length and partial constructs. The vast majority (371 of 392 (95%)) of the proteins were tested two or more times in GHT-SELEX experiments, with a median of four experiments for each. A subset (27%; 420 of 1,534) of the GHT-SELEX experiments were strict replicates (that is, same construct, same expression system), covering a total of 198 potential TFs in 204 experiments (Supplementary Table 3 and Supplementary Table 4), whereas the remainder were not strict replicates, allowing us to compare results from different expression systems. Different protein production methods could potentially yield different DNA-binding activities for the same TF, for example, through post-translational modifications, or if the intended protein associates with interaction partners that also bind DNA. It is also possible that the isolated DBD could bind different DNA sequences from the full-length protein. To ask whether we observe such differences, we evaluated 217 pairs of experiments where the same TF was expressed by two different expression systems (for example, wheat germ versus mammalian cells), and a further 72 pairs that used the same expression system, by plotting enrichment of 3, 5, 7 and 9 bp k-mers in the HT-SELEX data (a total of 289 instances encompassing 62 TFs; Supplementary Data 5 and Supplementary Table 12). We identified minor technical differences, including randomly occurring low-level enrichment of homopolymers and concentration-dependent multimeric binding (see first page of Supplementary Data 5 for full description), but we observed no evidence for cofactor contamination or unexpected differences in sequence specificity.

### HT-SELEX and GHT-SELEX

We modified protocols from a previously described HT-SELEX procedure^38^. HT-SELEX and the GHT-SELEX ligands contain the same flanking constant regions, and thus there were no differences in the selections or sequencing library preparations. We conducted the magnetic bead washing operations below using a Biotek 405TS plate washer fitted with a magnetic carrier. Protein immobilization was carried out in buffers based either on Lysis buffer (150 mM NaCl and 1% Triton X-100 in Tris-Cl, pH 8) or low stringency binding buffer (140 mM KCl, 5 mM NaCl, 1 mM K2HPO4, 2 mM MgSO4, 100 µM EGTA, 1 mM ZnSO4, and 0.1% Tween20 in 20 mM HEPES-HCl, pH 7). All DNA-protein reactions used low stringency binding buffer. For GST-tagged proteins, we used glutathione magnetic beads (Sigma-Aldrich, cat. no. G0924-1ML), and for GFP-protein immobilization, we used GFP-Trap Magnetic Agarose (Chromotek, gtma-100) for initial batches, and anti-GFP antibody (cat. no. ab290, Abcam) immobilized to Protein G Mag Sepharose Xtra (Cytiva, cat. no. 28-9670-70) for later batches, as the latter showed a higher success rate. All selections used 1 μl of the magnetic bead slurry, a volume that in most cases, according to manufacturers’ information, contains excess protein binding capacity but is still visible in microwell plates, allowing quality control of the washing steps.

#### SELEX process

All protocols (Supplementary Table 2) followed these general steps: (1) affinity beads and 96-well plates were blocked with bovine serum albumin for 15 min. (2) Beads and plates were washed to remove unbound bovine serum albumin. (3) Protein was immobilized into beads for 1 h at room temperature on a shaker. (4) Beads were washed to remove nonspecific proteins and carryover DNA. (5) Protein-coated beads were incubated with 10 μl of DNA ligand for 1 h at room temperature to allow the proteins to bind their target sites. (6) Unbound and weakly bound DNA ligands were removed with extensive washing. (7) DNA ligands were eluted by suspending the beads into 25 μl of heat elution buffer (0.4 μM forward and reverse primers, 1 mM EDTA and 1% Tween 20 in 10 mM Tris-Cl, pH 8) transferring the suspension into a conical PCR plate and heat treating it in a PCR machine using a program that cycled between temperatures of 98 °C and 60 °C to denature the proteins and DNA, use convection to drive the DNA into the solution and hybridize DNA to the amplification primers. (8) Bead suspension obtained from heat elution was used as template in PCR and qPCR reactions. (9) An additional DNA amplification cycle was performed with 2× more primers and dNTPs to ensure that most of the ligands are in fully double-stranded state. (10) In batch YWO and an associated batch YWN, we tested thesame set of proteins with (YWO) or without (YWN) mung bean nuclease treatment to digest partially single-stranded ligands from the experiments, to reduce enrichment of DNA with artifactual sites, such as potential aptamers. This strategy proved effective in HT-SELEX experiments and was used in the following batches (P, Q, R and S). For these last four batches, the TFs were mainly in alphabetical order. In each mung bean nuclease reaction, the pH of the solution (PCR reaction) was first lowered by the addition of 1:10 volume of 100 mM acetic acid, followed by addition of 1 μl (0.75 U) of the enzyme and incubation for 1 h at 30 °C.

### Sequencing

Samples were prepared for sequencing by performing a PCR reaction that indexes each sample and its selection cycle with a unique combination of i7 and i5 barcodes, followed by a double-stranding reaction with primers that target regions of DNA outside indices (Supplementary Table 1). Following this step, DNA libraries were pooled, purified with a PCR purification kit (NEB, cat. no. T1030S), and then subjected to Illumina sequencing with 60 bp reads at ~3 million reads per sample (Donnelly Center sequencing core facility).

### HT-SELEX and GHT-SELEX read processing and mapping

HT-SELEX reads were filtered by Phred quality score (Q ≥ 30 in at least 90% of bases). GHT-SELEX reads were parsed with Trimmomatic^66^ to remove the constant regions from genomic fragments that were shorter than the sequencing read length (options: ILLUMINACLIP:CustomAdapters.fa:2:5:5, LEADING:3, TRAILING:3 MINLEN:25). The custom adapters in the fasta file were AGATCGGAAGAGCACACGTCTGAACTCCAG and AGATCGGAAGAGCGTCGTGTAGGGAAAGAGTGTTA. For GHT-SELEX, we mapped trimmed reads to the human genome build hg38 with bowtie2 (options:–very-sensitive,–no-unal). The mapped reads were further filtered using Samtools (options: -F 1548, version 1.20)^67^. Data were processed further for MAGIX-based analyses: each cycle of individual experiments was filtered through, sorting mapped fragments by read name, deduplicating them by position (samtools command: markdup, option: -r), and keeping only properly paired reads (option: –f 2).

### HT-SELEX k-mer enrichment-based comparison of protein production methods

To identify binding specificity differences conferred by either protein production method or using a different clone type that contains all predicted DBDs (DBD or FL), we identified 62 TFs for which we had at least one pair of successful experiments performed with a lysate HT-SELEX experiment and another with either of the IVT systems. For each successful experiment performed for these 62 TFs, we counted occurrences of 3-, 5-, 7- and 9-mers in the unique reads contained in the final two cycles, as well as in the corresponding DNA library (cycle 0) using jellyfish (v2.3.1)^63^. For all possible experiment comparisons, log_2_ fold change was compared for each k-mer size.

### MAGIX statistical framework

At the core of MAGIX is a generative model that connects the enrichment of TF-bound genomic intervals explicitly to the fragment counts observed across GHT-SELEX cycles. MAGIX models how TF-bound intervals occupy a progressively higher proportion of the selected fragments pool in each cycle relative to the genomic background. These fragment proportions, in turn, are treated as latent variables in the model that, together with a sample-specific library size factor, determine the number of observed reads through a Poisson process. Consider the genomic interval *j*∈{1,...,*G*}, where *G* is the total number of unique genomic intervals that we are modeling. Assume that fragments originating from interval *j* have a starting abundance of *a*_*j*_ in the library. We also assume an exponential enrichment for the fragments, that in each cycle of SELEX, the abundance of these fragments changes by a factor of ${e}^{{b}_{\!j}}$, where *b*_*j*_ is the log fold change in abundance per cycle (referred to as ‘enrichment coefficient’), associated conceptually with biophysical parameters such as binding energies. Therefore, at cycle *t*, the abundance of the fragment originating from interval *j* is given by:

$${f}_{j}({t})={a}_{j}{\left({e}^{{b}_{\!j}}\right)}^{t}={a}_{\!j}{e}^{{{tb}}_{j}}$$

For convenience, we work with the logarithm of abundance, *y*_*j*_ = log *f*_*j*_, transforming the exponential equation above to a linear equation as follows:

$$E({\;y}_{{ij}})=\mathrm{log}({\;f}_{\!j}({t_i}))=\mathrm{log}\;{a}_{\!j}+{t}_{i}{b}_{\!j}$$

which can be seen as the dot product of a feature vector ${{\bf{x}}}_{i}=\left(1,\ {t}_{i}\right)$ for all the samples *i* (and across different cycles) corresponding to the TF of interest, and the interval-specific parameters ${{\boldsymbol{\beta }}}_{j}=(\log {a}_{\!j},\ {b}_{\!j})$.

We note that to model accurately the enrichment of each fragment per cycle, other factors, such as background or batch effects, also need to be taken into consideration and, therefore, the linear equation above needs to be fitted not only to the samples that correspond to the TF of interest, but also to samples from other experiments. We embed these dependencies in a design matrix *X*∈ℝ^*N*×*K*^, where *N* is the total number of samples and *K* is the number of variables to consider, including an intercept term (whose coefficient will correspond to log *a*_*j*_ above), a term for the SELEX cycle *t* (variable for the samples corresponding to a same TF), and other terms for batch and background effects. In addition to the variables included in *X*, the abundance of each fragment in each sample depends on a sample-specific scaling factor that is often referred to as the library size. Assume that this library effect, for each sample *i*∈{1,*...,N*}, is the scaling factor *s*_*i*_ (in logarithmic scale). Therefore:

$$E\left({{{y}}}_{{ij}}\right)={\hat{{{y}}}}_{{ij}}={{\mathbf{x}}}_{i}\cdot {\boldsymbol{\beta}}_{j}+{s}_{i}$$

Here, *ŷ*_*ij*_ corresponds to the expected logarithm of the abundance of interval *j* in sample *i*, **x**_*i*_∈ℝ^*K*^ is a vector representing the *i*’th row of the design matrix *X* (that is, the sample-level variables for sample *i*) and ***β***_*j*_∈ℝ^*K*^ is an interval-specific vector of coefficients for the *K* variables included in the model.

We note that the equation above does not have a unique solution. For example, any Δ***β*** can be added to ***β***_*j*_, followed by subtraction of *X*Δ***β*** from s, without any change in *ŷ*_*j*_:

$${\bf{x}}_{i}\cdot {\boldsymbol{\beta} }_{j}+{s}_{i}={\bf{x}}_{i}\cdot \left({\Delta }\boldsymbol{\beta} +{\boldsymbol{\beta} }_{j}\right)+\left({s}_{i}-{\bf{x}}_{i}\cdot {\Delta }\boldsymbol{\beta} \right)$$

Therefore, to make the model identifiable, we limit ***β***_*j*_ so that Σ_*j*∈{1,...,*G*}_***β***_*j*_ = **0**, where **0** is the zero vector of length *K*. This constraint is also useful as it means that, across all *G* intervals, the mean of each coefficient in ***β***, including the coefficient for the SELEX cycle, is zero; in other words, the enrichment per cycle for each interval is calculated relative to the mean of all *G* intervals.

To incorporate the experimental noise in the logarithm of the abundance of interval *j* in sample *i* (that is, building the real distribution of ${{{y}}}_{{ij}}$), we modeled it as a Gaussian random variable whose mean is given by *ŷ*_*ij*_ (the linear model above) with a sample-specific variance *σ*_*i*_^2^:

$${y}_{{ij}}{\mathscr{ \sim }}{\mathscr{N}}\left({\hat{y}}_{{ij}},{\sigma }_{i}^{2}\right)$$

To complete the Bayesian framework, we also assume a multivariate Gaussian prior for ***β***_*j*_:

$${{{\boldsymbol{\beta} }}}_{j}{\mathscr{ \sim }}{\mathscr{N}}\left({\bf{0}},{{\varSigma }}\right)$$

Here, *Σ*, the covariance matrix of the prior distribution, is shared across all intervals.

Altogether, the equations above form the following Bayesian model:

$${{{\boldsymbol{\beta} }}}_{j}{\mathscr{ \sim }}{\mathscr{N}}\left({\bf{0}},{{\varSigma }}\right)$$

$${s}_{i} \sim {\rm{U}}\left(-\infty ,+\infty \right)$$

$${\sigma }_{i}^{2} \sim {\rm{U}}\left(0,+\infty \right)$$

$${\hat{{{y}}}}_{{ij}}={{\mathbf{x}}}_{i}\cdot {\boldsymbol{\beta}}_{j}+{s}_{i}$$

$${y}_{{ij}}{\mathscr{ \sim }}{\mathscr{N}}\left({\hat{y}}_{{ij}},{\sigma }_{i}^{2}\right)$$

Assuming that the values for *y*_*ij*_ are observed directly, we can obtain the maximum a posteriori estimates of the parameters ***β***_*j*_ (for *j*∈{1,...,*G*}), s and *σ*_*i*_^2^ (for *i*∈(1,...,*N*)) through a block coordinate descent algorithm^48^.

The previous covariance matrix *Σ* is a hyperparameter that is obtained using an empirical Bayes approach. More specifically, we first obtain the maximum likelihood estimate of the parameters ***β***_*j*_, *s*_*i*_ and *σ*_*i*_^2^ without assuming any prior on ***β***_*j*_, and then estimate the values of *σ*_*βk*_^2^—the variance of each element *k* in ***β***_*j*_—using the maximum likelihood estimate solutions of all ***β***_*j*_ coefficients. The covariance matrix *Σ* is then constructed as *Σ* = diag(*σ*_*β*1_^2^,…, *σ*_*βK*_^2^).

We note, however, that the log-abundance *y*_*ij*_ values are not directly observable in GHT-SELEX data. Instead, we observe *m*_*ij*_, the count of reads mapping to interval *j* in sample *i* (see ‘HT- and GHT-SELEX read processing and mapping’). This parameter adds another step to the framework, leading to the following hierarchical Bayesian model:

$${{{\boldsymbol{\beta} }}}_{j}{\mathscr{ \sim }}{\mathscr{N}}\left({\bf{0}},{{\varSigma }}\right)$$

$${s}_{i} \sim {\rm{U}}\left(-\infty ,+\infty \right)$$

$${\sigma }_{i}^{2} \sim {\rm{U}}\left(0,+\infty \right)$$

$${\hat{{{y}}}}_{{ij}}={{\mathbf{x}}}_{i}\cdot {\boldsymbol{\beta}}_{j}+{s}_{i}$$

$${y}_{{ij}}^{* }{\mathscr{ \sim }}{\mathscr{N}}\left({\hat{y}}_{{ij}},{\sigma }_{i}^{2}\right)$$

$${m}_{{ij}} \sim {\rm{Pois}}\left({e}^{{y}_{{ij}}^{* }}\right)$$

Here, y^*^_ij_ is the logarithm of the true abundance of fragment *j* in sample *i*, which is latent. We obtain the maximum a posteriori estimates of the parameters ***β***_*j*_ (for *j*∈{1,*...,G*}), *s*_*i*_ and *σ*_*i*_^2^ (for *i*∈{1,...,*N*}) using an EM algorithm, in which at each E-step we obtain the expected value of each y^*^_ij_ given the observed read count *m*_*ij*_ and the current model parameters, followed by re-estimation of the model parameters in the M-step, similar to a previous method established for EM optimization of Poisson lognormal models^48^.

### Identifying GHT-SELEX peaks with MAGIX

The statistical framework described above calculates the rate of enrichment across GHT-SELEX cycles for a set of given genomic intervals, for example, a set of candidate peak regions. Below, we describe how we identify such candidate peaks.

For each TF, an aggregate BAM file is created across replicates and cycles. For each genomic region with continuous nonzero coverage in this aggregate BAM file, the maximum coverage is calculated. Regions with a maximum coverage smaller than a threshold are discarded, with the threshold being set to the total fragment count in the aggregate BAM file divided by 2 × 10^6^. Then, per region, the coordinate with the highest coverage is defined as the summit. The candidate peaks are defined as the 200-bp regions centered on the summits.

Once the candidate peaks are identified, their count profiles are calculated from the original (unaggregated) BAM files (per cycle and replicate) and used as input to MAGIX to calculate MAGIX scores (enrichment coefficients), using prefixed library sizes that are estimated by fitting a similar MAGIX model to count profiles of 200-bp nonoverlapping genomic intervals (a total of ~13 million bins). For each candidate peak, we also calculate a *P* value, representing the statistical significance of the enrichment coefficient (the null hypothesis is that the enrichment coefficient is zero). To do so, we obtain a maximum likelihood estimate of the model coefficients and perform a likelihood ratio test against a reduced model in which the enrichment coefficient is restricted to zero. The source code for MAGIX is available at https://github.com/csglab/MAGIX.

MAGIX peak sets analyzed throughout these papers and provided at https://codebook.ccbr.utoronto.ca/ were derived by merging all successful experiments performed with inserts that contain all predicted DBDs of the TF (marked with ‘DBD’ or ‘FL’). Experiments performed with specific subsets of DBDs (marked with ‘DBD1,’ ‘DBD2’ or ‘DBD3’) are analyzed and indicated separately.

### Selection of thresholds for peak sets

We sorted the GHT-SELEX peaks by their MAGIX score, or as named in the peaks BED files, coefficient.br, which estimates cycle enrichment. Similarly, we sorted the merged ChIP–seq peaks by *P* value. Then, for different values of *N* (between 100 and the total number of peaks), we took the top *N* peaks for both peak sets and calculated the Jaccard index (= O/(2*N* − O), in which O is the intersection of peaks). When a peak in a set overlaps several peaks in another set, we used the average of the overlaps for the intersection (that is, O = (O1 + O2)/2, in which O1 is the number of peaks in set1 overlapping with any peaks in set2 and vice versa). The value of *N* that yielded the maximum Jaccard value was identified, and the threshold for each peak set was taken as that which yielded this maximum *N*. The same process was applied to compare PWM-predicted binding sites and ChIP–seq peaks.

### GHT-SELEX experiment overlap comparison

To investigate the reproducibility of GHT-SELEX profiles, we focused on experiment pairs with the same TF construct and protein production approach. Per replicate, we fitted genome-wide MAGIX models to 200-bp nonoverlapping genomic intervals (~13 million genomic intervals), resulting in a model coefficient per genomic bin. The exponential of this coefficient represents the fold-enrichment per cycle, which we visualize as scatterplots and use to calculate the Pearson correlation between replicates.

### Motif derivation and assessment of experiment success

We evaluated the success of GHT- and HT-SELEX experiments systematically alongside other Codebook datasets generated with ChIP–seq, protein binding microarray and SMiLE-seq experiments (see Supplementary Table 6 for a summary of all experiments), by performing motif discovery with nine different programs and then testing their performance in all experiments for the same TF. Experiments were classified by expert curation as successful if they produced motifs that scored well in data generated by other methods (preferably, and in most cases) and, in rare cases, in replicate data from the same experimental method, when other methods failed. All motifs can be browsed at https://mex.autosome.org. See accompanying article for detailed description of the process^50^.

### Representative motif selection

For each TF, a list of up to 60 motifs was assembled automatically based on performance in AUROC, AUPRC and motif centrality metrics across all successful experiments^50^. These motifs were then assessed manually by an assembly of authors to select a representative motif. Besides scoring across all datasets, we considered whether the motif seems likely to describe the inherent specificity of the TF, rather than, for example, a larger sequence that is derived partially from a genomic repeat element (a scenario common with KRAB-domain containing zinc finger TFs). In cases where several motifs displayed highly similar performance, we prioritized motifs with higher information content. See Vorontsov and colleagues^50^ for further details and Supplementary Data 1 for the representative motifs.

### Comparing PWM scoring methods

To create in silico predicted binding sites for a TF, we first scanned the genome using the representative PWM, using MOODS^68^ with a *P* value threshold of 0.0001. We then merged the clusters of PWM hits with a distance less than 200 bp between neighboring hits, as this is the median length of ChIP–seq fragments, and the task is predicting in vivo binding sites. Singleton PWM hits and boundary hits (that is, the leftmost or rightmost hits within a cluster) were also expanded to have a width of at least 200 bp. The clusters of PWM hits were then rescored using sum-of-affinity (that is, with PWM log-odds scores at each base converted to linear/probability space, before calculation of the sum) and maximum-affinity methods, by either applying a sum or maximum, respectively, over the PWM scores of the cluster members. The resulting sites were sorted by their new score and processed through the same optimization procedure described above for peaks, to maximize their overlap with ChIP–seq peaks.

### Modeling alternative C2H2-zf binding modes with RCADEEM

RCADEEM uses a hidden Markov model (HMM) to represent several, alternative DNA-binding motifs, each corresponding to the binding preference of a C2H2-zf array. Briefly, the DNA sequences (for example, GHT-SELEX peaks) are modeled as sequences generated from a discrete Markov process with hidden states that include a background state (*S*_0_) and *M* motif states *S*_*m*_ (*m*∈[1,*M*]). The background state, with marginal probability *π*_0_, emits each nucleotide *n* with probability *b*_0_(*n*) (Σ_*n*_*b*_0_(*n*)=1). The background state can transition to itself (that is, consecutive DNA nucleotides can be generated from the background state) with probability *a*_0,0_, or to each motif state *S*_*m*_ (*m*∈[1,*M*]) with probability *a*_0,*m*_ (*a*_0,0 _+ *Σ*_m_*a*_0,m_ = 1). Each motif *m*, with marginal probability *π*_*m*_, generates a sequence of length *l*_*m*_, with each nucleotide *n* at position *i* emitted with probability *b*_*m*,*i*_(*n*) (*Σ*_*n*_*b*_*m*,*i*_(*n*)=1 ∀*m*∈[1,*M*], *i*∈[1,*l*_*m*_]). Note that, for each *m*∈[1,*M*], the values *b*_*m*,*i*_(*n*) form a position-specific frequency matrix (PFM, that is the exponential of the classical log-odds PWM) with width *l*_*m*_, which is fixed to be three times the number of zinc finger domains in the array represented by motif *m*, as each zinc finger domain binds to three nucleotides. Finally, each motif state *S*_*m*_ transitions to the background state with probability *a*_m,0_ = 1.

We start the model by including the motifs representing all possible consecutive zinc finger domain arrays^50^. We initialize the emission probabilities *b*_*m*,*i*_(*n*) for each motif *m* using the PFM predicted for the associated zinc finger array by a previously created C2H2-zf recognition code^51^—this recognition code is a machine learning model that, given the sequence of a zinc finger array, predicts the expected binding preference. The HMM parameters, including all marginal state probabilities, state transition probabilities and emission probabilities, are then optimized by EM using the Baum–Welch algorithm. Each of the optimized PFMs is then tested for (1) enrichment of the motif in actual sequences compared to dinucleotide-shuffled sequences, and (2) similarity to the original recognition code-predicted PFM. To achieve (1), for each position *x* in each DNA sequence *k*, we calculate γ_*k,x*_(*S*_*m*_), the probability that it was generated from motif state *S*_*m*_, using the forward–backward algorithm. The motif score for DNA sequence *k* is then calculated as *Σ*_*x*_γ_*k,x*_(*S*_*m*_)/*l*_*m*_, representing the expected number of times the state *S*_*m*_ is seen in sequence *k*. For each motif *m*, these scores are calculated both for actual GHT-SELEX peak sequences and their dinucleotide-shuffled version. The top 100 sequences with the largest scores for each motif are then tested to see whether they are enriched in the motif compared to shuffled sequences (Fisher’s exact test, FDR ≤ 0.01). Motifs that do not pass this cutoff are removed from the model. To achieve (2), each HMM-optimized PFM is first converted to log-scale (representing a PWM), followed by the calculation of the Pearson correlation of the PWM entries with those predicted by the recognition code. Pearson correlations are then converted using Fisher transformation to calculate a *P* value, followed by removal of motifs that do not pass the FDR cutoff ≤0.01. The remaining motifs are then used to reconstruct a smaller HMM, similar to the procedure described above, followed by another round of EM optimization. This procedure is repeated until all motifs pass the cut-offs for enrichment in GHT-SELEX sequences while maintaining significant similarity to the original recognition code-predicted sequences.

To visualize the binding modes predicted by RCADEEM, the resulting PWMs are used to identify their best match in each of the input sequences using AffiMx^52^. Then, for each sequence, the PWM with the highest weighted HMM score on the best match is kept as the predicted binding mode. To align the sequences, offsets are calculated based on the corresponding C2H2-zf domains (Fig. 5a–f). C2H2-zf proteins were categorized based on their alternative usage of C2H2-zf domains (that is, several DBDs, finger shift, canonical and core with extensions; Fig. 5) through an expert-curated evaluation (Supplementary Table 9). To make a motif model for each binding mode, we selected manually representative peaks corresponding to each binding mode over the 2000 GHT-SELEX peaks with the highest enrichment coefficient. The sequence (already aligned by RCADEEM) and C2H2-zf domain array coordinates of these peaks were used to create PFMs. The resulting PFMs for those C2H2-zf TFs are available in Supplementary Data 4 and online at https://cisbp.ccbr.utoronto.ca (ref. ^51^). The logos, coordinates, selected sequences, annotated sequence heatmaps and associated metadata are available online at https://codebook.ccbr.utoronto.ca. The source code for RCADEEM is available at https://github.com/csglab/RCADEEM.

### Comparison of C2H2 DBDs

C2H2 DBD similarities were compared by pairwise alignment with Needleman–Wunsch algorithm, as implemented in the R package Biostrings and counting substitutions, insertions and unmatched flanking bases as edits. DNA-binding functionality scores were determined using a random forest classifier^64^ and motif similarity calculated using MosBAT^69^.

### Reporting summary

Further information on research design is available in the Nature Portfolio Reporting Summary linked to this article.

## Online content

Any methods, additional references, Nature Portfolio reporting summaries, source data, extended data, supplementary information, acknowledgements, peer review information; details of author contributions and competing interests; and statements of data and code availability are available at https://doi.org/10.1038/s41592-026-03177-9.

## Supplementary information

Supplementary Information One supplementary note and Figs. 1 and 2.

Reporting Summary

Supplementary Table 1 HT- and GHT-SELEX ligand sequences and descriptions. Table lists the oligonucleotide sequences used in the assay and describes how they anneal with each other during the synthesis and amplification steps.

Supplementary Table 2 Experimental batch-specific protocol details. Table lists the reagents and experimental conditions that varied between different experimental batches.

Supplementary Table 3 GHT-SELEX-experiment metadata. Table lists all GHT- and HT-SELEX experiments performed in this study indicating: unique experiment identifier; human readable identifier; plasmid identifiers; HNGC symbol; experimental batch; construct type; protein production approach; position in the 96-well; sequencing strategy; number of selection cycles; measured fluorescence; estimated protein concentration and whether the experiment was successful or not. Note that GFP control experiments (that is, empty plasmids) are also included in the table (five for GHT-SELEX and seven for HT-SELEX).

Supplementary Table 4 Summary data of GHT-SELEX replicates. Table shows counts of successful and unsuccessful experiments for all TFs analyzed, listing both direct experiments, which were performed with the same construct and expression system and indirect replicates based on TF identity, regardless of protein production method or insert.

Supplementary Table 5 Reproducibility of GHT-SELEX experiments. Table lists 102 experiment pairs with Pearson correlation of MAGIX score (coefficient.br) values observed in genomic bins.

Supplementary Table 6 Summary of Codebook experiments and motifs. Table lists all GHT-SELEX, HT-SELEX, protein-binding microarray, SMiLE-seq and ChIP–seq experiments performed in the Codebook consortium and indicates experiments that were used to derive the representative PFMs.

Supplementary Table 7 List and annotations of western blot analyses for lysates and wheat germ extracts. Each row describes one of the 97 western blot wells in the associated Supplementary Fig. 2, and provides information on the following: associated experiment; HNGC identifier; construct type of the tested TF productions; structural class of the DBD; GHT-SELEX result; gel and well; all wells with this TF; protein expression method; gel examination based annotation of the protein production success; whether the western blot shows partial bands; whether the experiment is paired comparison of different construct for the same TF (FL versus DBD); whether a successful experiment for C2H2 TF displayed modular binding activities based on RCADEEM analysis; is a IVT versus lysate production comparison; eGFP fluorescence measured for the protein, protein construct amino acid count and estimated molecular weight.

Supplementary Table 8 Genomic region overlap of GHT-SELEX and ChIP–seq peaks and PWM-predicted target regions. Table shows the overlap of optimal ChIP–seq peaks with GHT-SELEX/MAGIX and PWM-based predictions for each of the TFs where both datasets were available. Columns show the highest Jaccard coefficient between each pair of datasets and the number of peaks that yielded it.

Supplementary Table 9 C2H2-zf protein DNA-binding mode annotation. Table lists the 86 C2H2 TFs for which RCADEEM result was obtained (out of 120 total C2H2-zf TFs with GHT-SELEX data available) with information on total number of C2H2 zinc finger domains; amino acid gaps between these DBDs; number of distinct motifs bound by the TF; modular binding activity annotated for it; whether the protein is likely to contain zinc fingers obtained from internal duplications and whether data was obtained from experiments that expressed different subsets of the TFs C2H2-zf domains.

Supplementary Table 10 Intra-protein C2H2-zf domain duplication dataset. Table displays all pairs of human C2H2-zf domains that are separated from each other by five or fewer edits.

Supplementary Table 11 Reference measurements for estimation of eGFP-fusion protein concentrations. Table shows fluorescence measurements for a dilution series of reference eGFP protein of >95% purity and resulting fluorescence (Extended Data Fig. 2b). The table also shows fluorescence values adjusted to correspond to the background level observed in the lysate and eGFP-IVT experiments.

Supplementary Table 12 Annotations of experiment pairs shown in Supplementary Data 5 (production method-derived differences in target specificity data). Table shows Pearson correlations of k-mer enrichment between pairs of replicate experiments.

Supplementary Data 1 Representative motifs for TFs analyzed by the Codebook Consortium. PFMs for all of the representative motifs chosen in the Codebook project.

Supplementary Data 2 GHT-SELEX reproducibility. Document shows scatterplots of coefficient-BR scores for all possible 15 million nonoverlapping 200 bp genomic bins for pairs of successful experiments that have been expressed with the same protein production system. See associated Supplementary Table 5 for details and summary data.

Supplementary Data 3 Motif centrality and enrichment in GHT-SELEX/MAGIX peaks and its correspondence with ChIP–seq peaks. Same plots as in Fig. 2c and Fig. 4c for all the TFs and DBD constructs in this study with successful GHT-SELEX experiments. Top, top-ranked TF PWM (highest AUROC on GHT-peaks as determined by ref. ^51^). Middle, distribution of PWM hits within the 5,000 highest scoring MAGIX peaks. Solid red lines: mean PWM hit position within MAGIX peaks; dashed lines: 1 s.d. about the mean. Bottom, enrichment of ChIP–seq peaks and PWM hits within MAGIX peaks. Orange line: proportion of peaks (in a sliding window of 500 peaks over the ranked peaks, with a step size of 50) that overlap with a ChIP–seq peak (at MACS threshold *P* < 0.001). Black line: AUROC for PWM affinity scores of MAGIX peaks in the same window versus 500 random genomic sites.

Supplementary Data 4 PFMs of C2H2-zf proteins with alternative DNA binding modes. PFMs representing the different binding modes of C2H2-zf proteins.

Supplementary Data 5 Analysis of k-mer enrichment in HT-SELEX experiment pairs. First page: description of likely mechanisms that cause systematic differences between replicate pairs between. Pages 2–290: enrichment of 3, 5, 7 and 9 bp k-mers are shown for 289 combinations of HT-SELEX experiments, see Supplementary Table 12 for details.

## Source data

Source Data Fig. 1c Data tracks of PWM hits as bedgraphs pasted into excel sheets. All data are available in bigwig format in GEO-database, under identifier GSE278858.

Source Data Fig. 2b Motif match centrality data for the figure panel.

Source Data Fig. 3 Information of each TF, the structural class of their DBD and success status in the analyses.

Source Data Fig. 4a Information of PWM performance, MAGIX scores and ChIP peak overlap for peak ranges, for GABPA and CTCF.

Source Data Fig. 4b Jaccard distance of overlaps CTCF GHT-SELEX and ChIP–seq datasets for different peak counts.

Source Data Figs. 4c and 4d Excel sheet of data used for plots.

Source Data Fig. 5g Classification of all C2H2-zf TFs for which RCADEEM analysis converged.

Source Data Fig. 6a Genomic location data, sequences and annotations.

Source Data Fig. 6c Normalized incidences of ten-mers in HT-SELEX-experiments performed for partial or full-length constructs of ZNF721.

Source Data Extended Data Fig. 2a Fluorescence values observed in the protein preparations, annotation of their success and the protein production system used.

Source Data Extended Data Fig. 2b Raw- and background adjusted fluorescence measurements for reference eGFP protein of known concentration.

Source Data Extended Data Fig. 3 Optimal Jaccard distances and the corresponding number of peaks between GHT-SELEX and ChIP–seq data.

Source Data Extended Data Fig. 4 Optimal Jaccard distances and the corresponding number of peaks between optimal PWM based predictions and ChIP–seq peaks, using either sum or maximum value-based scoring approaches.

Source Data Extended Data Fig. 6 Experimental success status for the tested putative TFs and the structural class of their DNA binding domain based on combination of all protein production types, and also for the individual protein production strategies.

## Data Availability

The sequencing raw data for the HT-SELEX and GHT-SELEX experiments have been deposited into the SRA database under identifiers PRJEB61115 (HT-SELEX) and PRJEB76622 (GHT-SELEX). Genomic interval information generated for the GHT-SELEX has been deposited into GEO under accession GSE278858. The entire Codebook data structure, with many accessory files and browsable results is available at https://codebook.ccbr.utoronto.ca. A larger collection of motifs generated for these experiments in an accompanying study^50^ can be browsed at https://mex.autosome.org. Source data are provided with this paper.

## Code availability

MAGIX and RCADEEM are available under the GNU General Public License (GPL) version 3. Source code is available via GitHub at https://github.com/csglab/MAGIX and https://github.com/csglab/RCADEEM, as well as via Zenodo at https://doi.org/10.5281/zenodo.20846978 (release v1.0.1)^70^ and https://doi.org/10.5281/zenodo.20847443 (release v1.0.0)^71^.
