# Supplementary material for: GHT-SELEX demonstrates unexpectedly high intrinsic sequence specificity and complex DNA binding of many human transcription factors
Source: Nat Methods. 2026 Aug 5;23(9):1775–85. doi: 10.1038/s41592-026-03177-9 (PMC13541613; doi:10.1038/s41592-026-03177-9)
Supplement: Supplementary file 1 [file 41592_2026_3177_MOESM16_ESM.pdf]

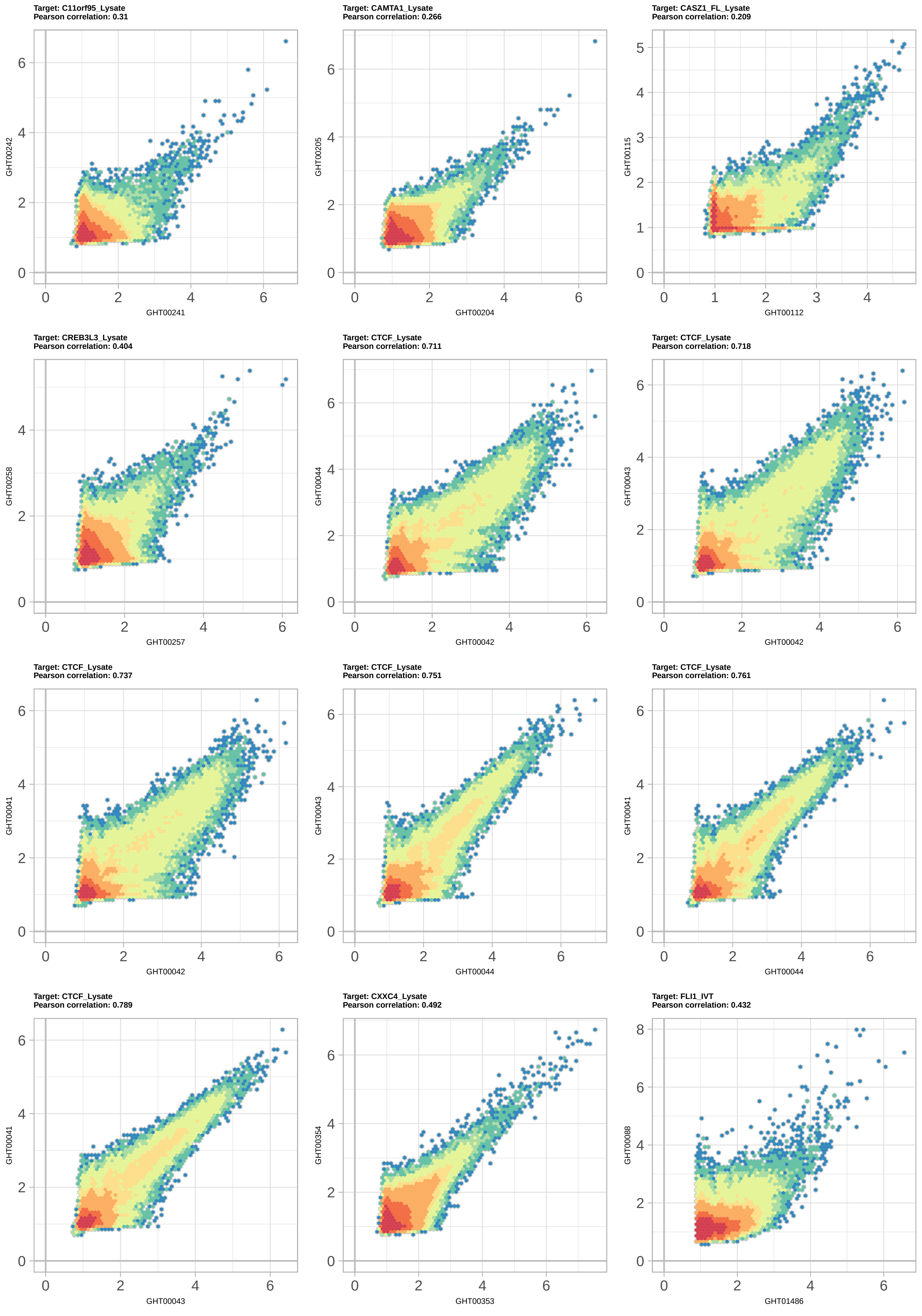
Target: C11orf95_Lysate
Pearson correlation: 0.31
GHT00242
GHT00241
Target: CAMTA1_Lysate
Pearson correlation: 0.266
GHT00205
GHT00204
Target: CASZ1_FL_Lysate
Pearson correlation: 0.209
GHT00115
GHT00112
Target: CREB3L3_Lysate
Pearson correlation: 0.404
GHT00258
GHT00257
Target: CTCF_Lysate
Pearson correlation: 0.711
GHT00044
GHT00042
Target: CTCF_Lysate
Pearson correlation: 0.718
GHT00043
GHT00042
Target: CTCF_Lysate
Pearson correlation: 0.737
GHT00041
GHT00042
Target: CTCF_Lysate
Pearson correlation: 0.751
GHT00043
GHT00044
Target: CTCF_Lysate
Pearson correlation: 0.761
GHT00041
GHT00044
Target: CTCF_Lysate
Pearson correlation: 0.789
GHT00041
GHT00043
Target: CXXC4_Lysate
Pearson correlation: 0.492
GHT00354
GHT00353
Target: FLI1_IVT
Pearson correlation: 0.432
GHT00088
GHT01486

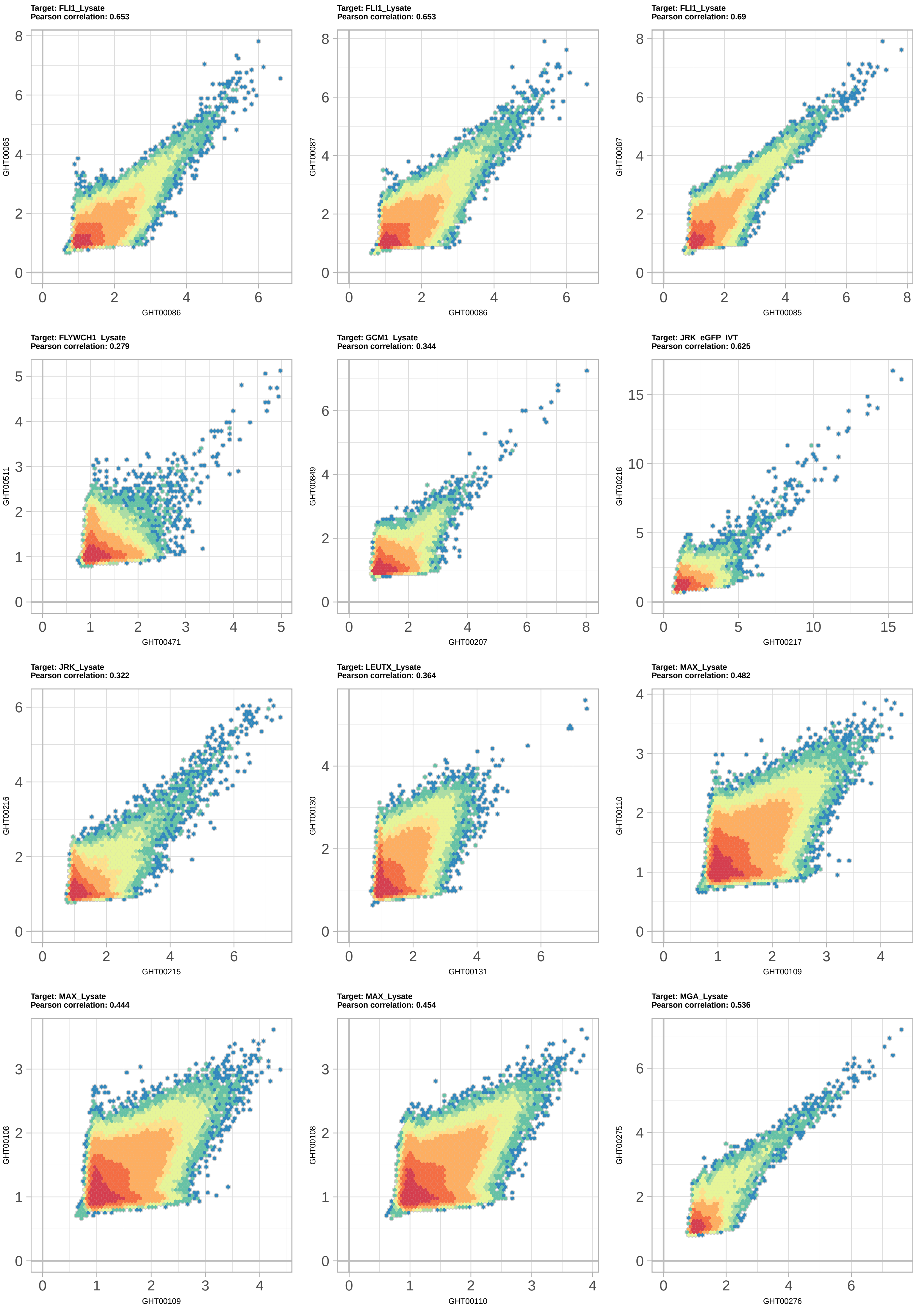

Target: FLI1_Lysate
Pearson correlation: 0.653
GHT00085
GHT00086
Target: FLI1_Lysate
Pearson correlation: 0.653
GHT00087
GHT00086
Target: FLI1_Lysate
Pearson correlation: 0.69
GHT00087
GHT00085
Target: FLYWCH1_Lysate
Pearson correlation: 0.279
GHT00511
GHT00471
Target: GCM1_Lysate
Pearson correlation: 0.344
GHT00849
GHT00207
Target: JRK_eGFP_IVT
Pearson correlation: 0.625
GHT00218
GHT00217
Target: JRK_Lysate
Pearson correlation: 0.322
GHT00216
GHT00215
Target: LEUTX_Lysate
Pearson correlation: 0.364
GHT00130
GHT00131
Target: MAX_Lysate
Pearson correlation: 0.482
GHT00110
GHT00109
Target: MAX_Lysate
Pearson correlation: 0.444
GHT00108
GHT00109
Target: MAX_Lysate
Pearson correlation: 0.454
GHT00108
GHT00110
Target: MGA_Lysate
Pearson correlation: 0.536
GHT00275
GHT00276

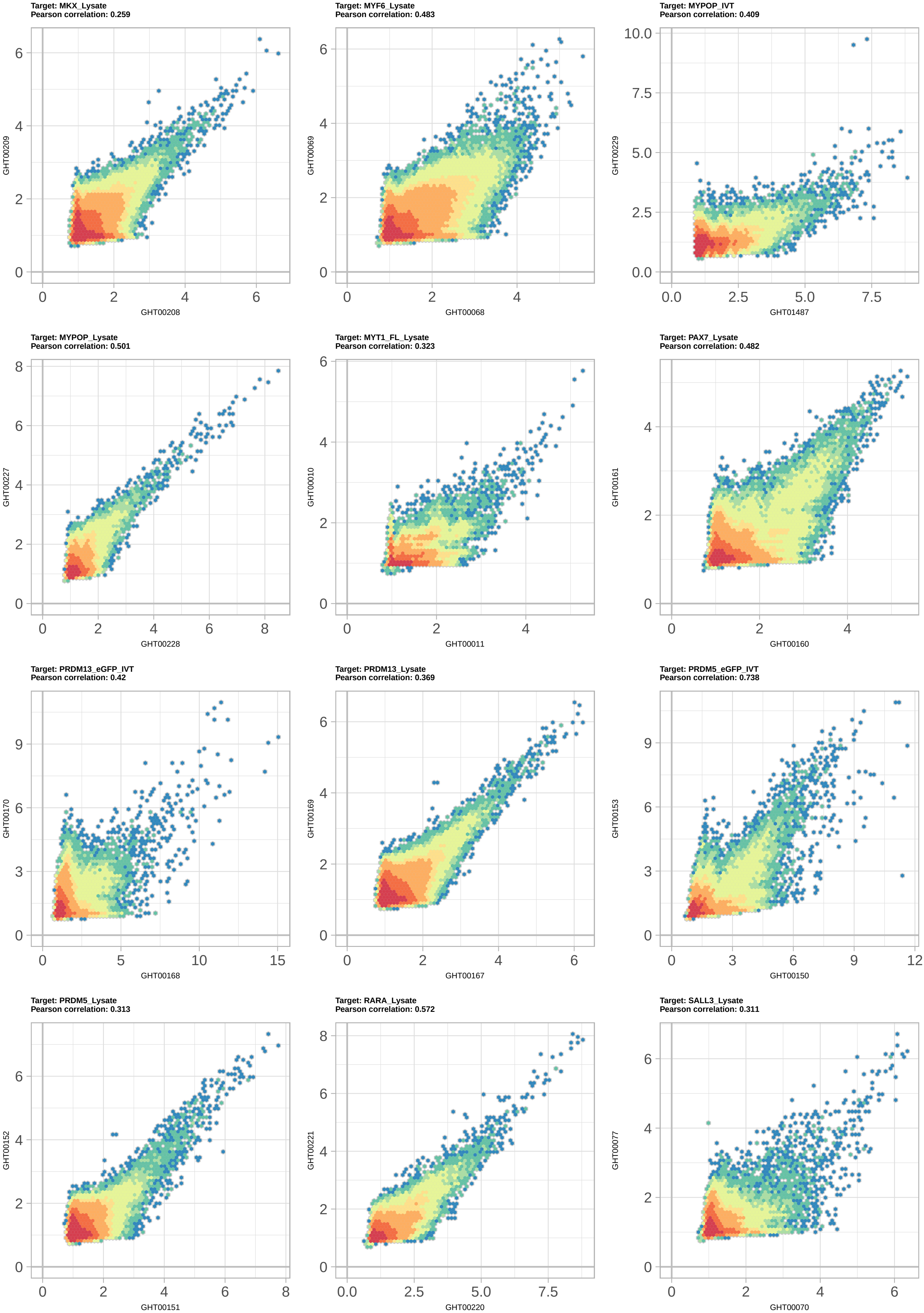
Target: MKX_Lysate
Pearson correlation: 0.259
GHT00208
GHT00209
Target: MYF6_Lysate
Pearson correlation: 0.483
GHT00068
GHT00069
Target: MYPOP_IVT
Pearson correlation: 0.409
GHT01487
GHT00229
Target: MYPOP_Lysate
Pearson correlation: 0.501
GHT00228
GHT00227
Target: MYT1_FL_Lysate
Pearson correlation: 0.323
GHT00011
GHT00010
Target: PAX7_Lysate
Pearson correlation: 0.482
GHT00160
GHT00161
Target: PRDM13_eGFP_IVT
Pearson correlation: 0.42
GHT00168
GHT00170
Target: PRDM13_Lysate
Pearson correlation: 0.369
GHT00167
GHT00169
Target: PRDM5_eGFP_IVT
Pearson correlation: 0.738
GHT00150
GHT00153
Target: PRDM5_Lysate
Pearson correlation: 0.313
GHT00151
GHT00152
Target: RARA_Lysate
Pearson correlation: 0.572
GHT00220
GHT00221
Target: SALL3_Lysate
Pearson correlation: 0.311
GHT00070
GHT00077

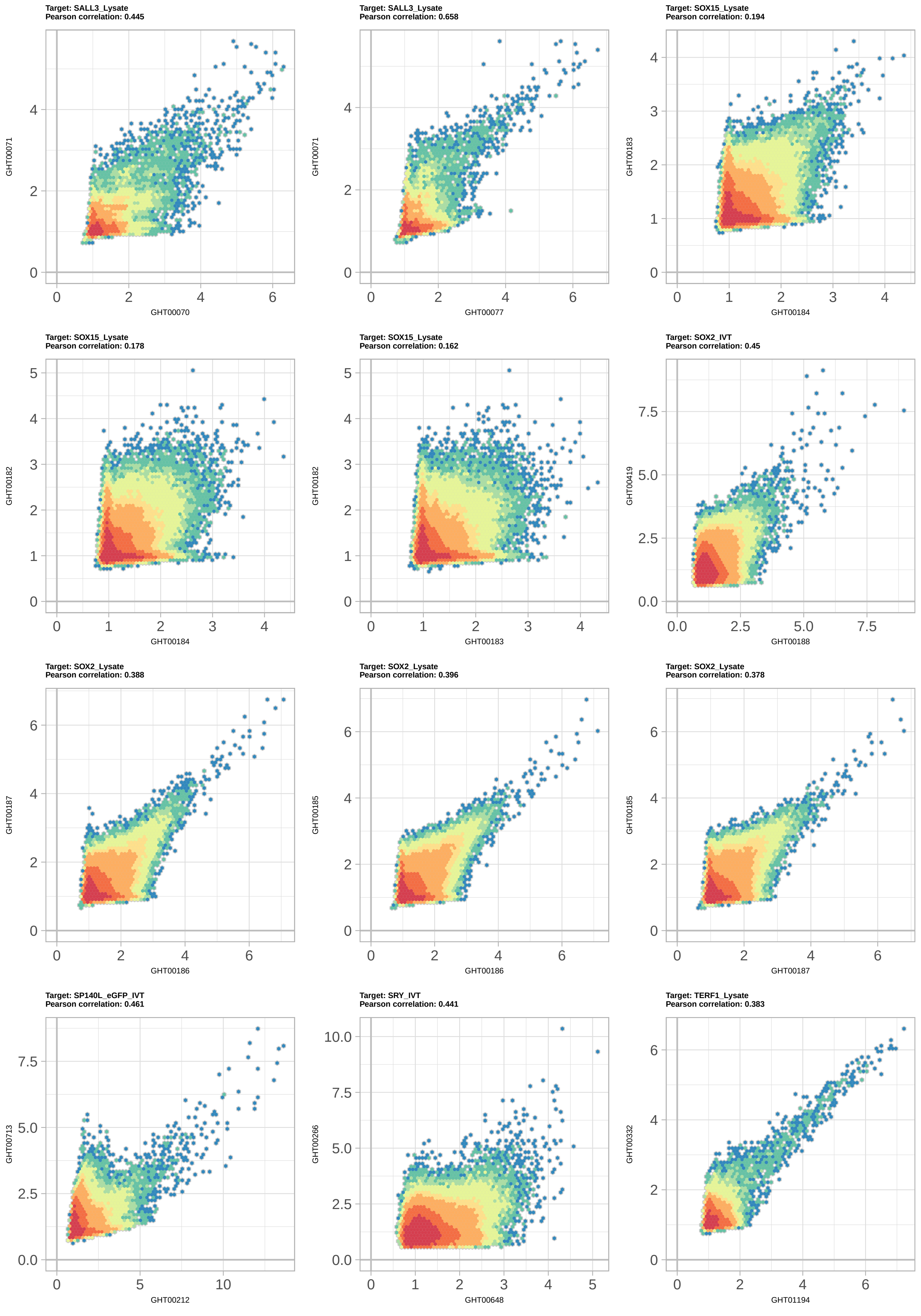
Target: SALL3_Lysate
Pearson correlation: 0.445
GHT00070
GHT00071
Target: SALL3_Lysate
Pearson correlation: 0.658
GHT00077
GHT00071
Target: SOX15_Lysate
Pearson correlation: 0.194
GHT00184
GHT00183
Target: SOX15_Lysate
Pearson correlation: 0.178
GHT00184
GHT00182
Target: SOX15_Lysate
Pearson correlation: 0.162
GHT00183
GHT00182
Target: SOX2_IVT
Pearson correlation: 0.45
GHT00188
GHT00419
Target: SOX2_Lysate
Pearson correlation: 0.388
GHT00186
GHT00187
Target: SOX2_Lysate
Pearson correlation: 0.396
GHT00186
GHT00185
Target: SOX2_Lysate
Pearson correlation: 0.378
GHT00187
GHT00185
Target: SP140L_eGFP_IVT
Pearson correlation: 0.461
GHT00212
GHT00713
Target: SRY_IVT
Pearson correlation: 0.441
GHT00648
GHT00266
Target: TERF1_Lysate
Pearson correlation: 0.383
GHT01194
GHT00332

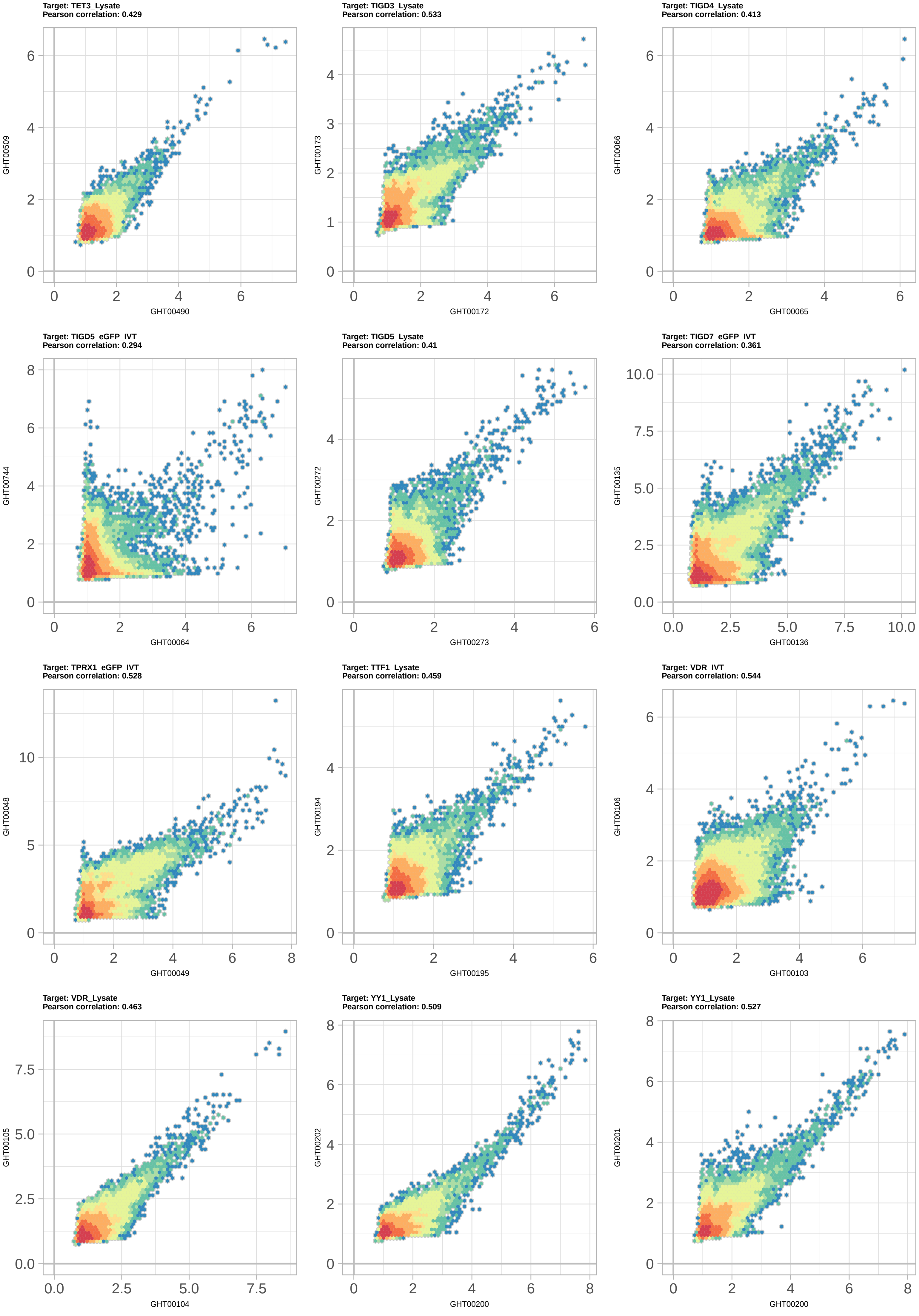
Target: TET3_Lysate
Pearson correlation: 0.429
GHT00509
GHT00490
Target: TIGD3_Lysate
Pearson correlation: 0.533
GHT00173
GHT00172
Target: TIGD4_Lysate
Pearson correlation: 0.413
GHT00066
GHT00065
Target: TIGD5_eGFP_IVT
Pearson correlation: 0.294
GHT00744
GHT00064
Target: TIGD5_Lysate
Pearson correlation: 0.41
GHT00272
GHT00273
Target: TIGD7_eGFP_IVT
Pearson correlation: 0.361
GHT00135
GHT00136
Target: TPRX1_eGFP_IVT
Pearson correlation: 0.528
GHT00048
GHT00049
Target: TTF1_Lysate
Pearson correlation: 0.459
GHT00194
GHT00195
Target: VDR_IVT
Pearson correlation: 0.544
GHT00106
GHT00103
Target: VDR_Lysate
Pearson correlation: 0.463
GHT00105
GHT00104
Target: YY1_Lysate
Pearson correlation: 0.509
GHT00202
GHT00200
Target: YY1_Lysate
Pearson correlation: 0.527
GHT00201
GHT00200

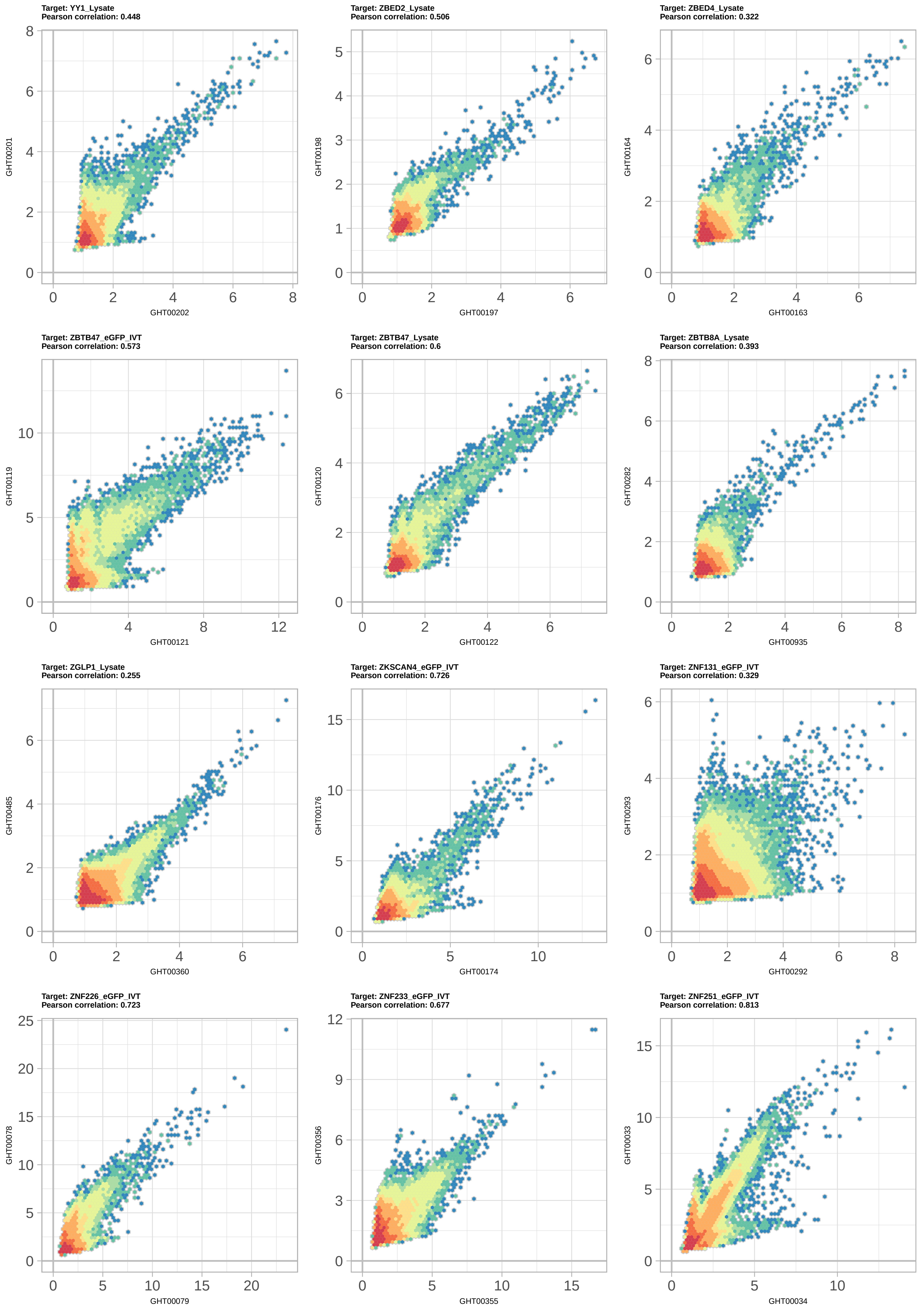
Target: YY1_Lysate
Pearson correlation: 0.448
GHT00201
GHT00202
Target: ZBED2_Lysate
Pearson correlation: 0.506
GHT00198
GHT00197
Target: ZBED4_Lysate
Pearson correlation: 0.322
GHT00164
GHT00163
Target: ZBTB47_eGFP_IVT
Pearson correlation: 0.573
GHT00119
GHT00121
Target: ZBTB47_Lysate
Pearson correlation: 0.6
GHT00120
GHT00122
Target: ZBTB8A_Lysate
Pearson correlation: 0.393
GHT00282
GHT00935
Target: ZGLP1_Lysate
Pearson correlation: 0.255
GHT00485
GHT00360
Target: ZKSCAN4_eGFP_IVT
Pearson correlation: 0.726
GHT00176
GHT00174
Target: ZNF131_eGFP_IVT
Pearson correlation: 0.329
GHT00293
GHT00292
Target: ZNF226_eGFP_IVT
Pearson correlation: 0.723
GHT00078
GHT00079
Target: ZNF233_eGFP_IVT
Pearson correlation: 0.677
GHT00356
GHT00355
Target: ZNF251_eGFP_IVT
Pearson correlation: 0.813
GHT00033
GHT00034

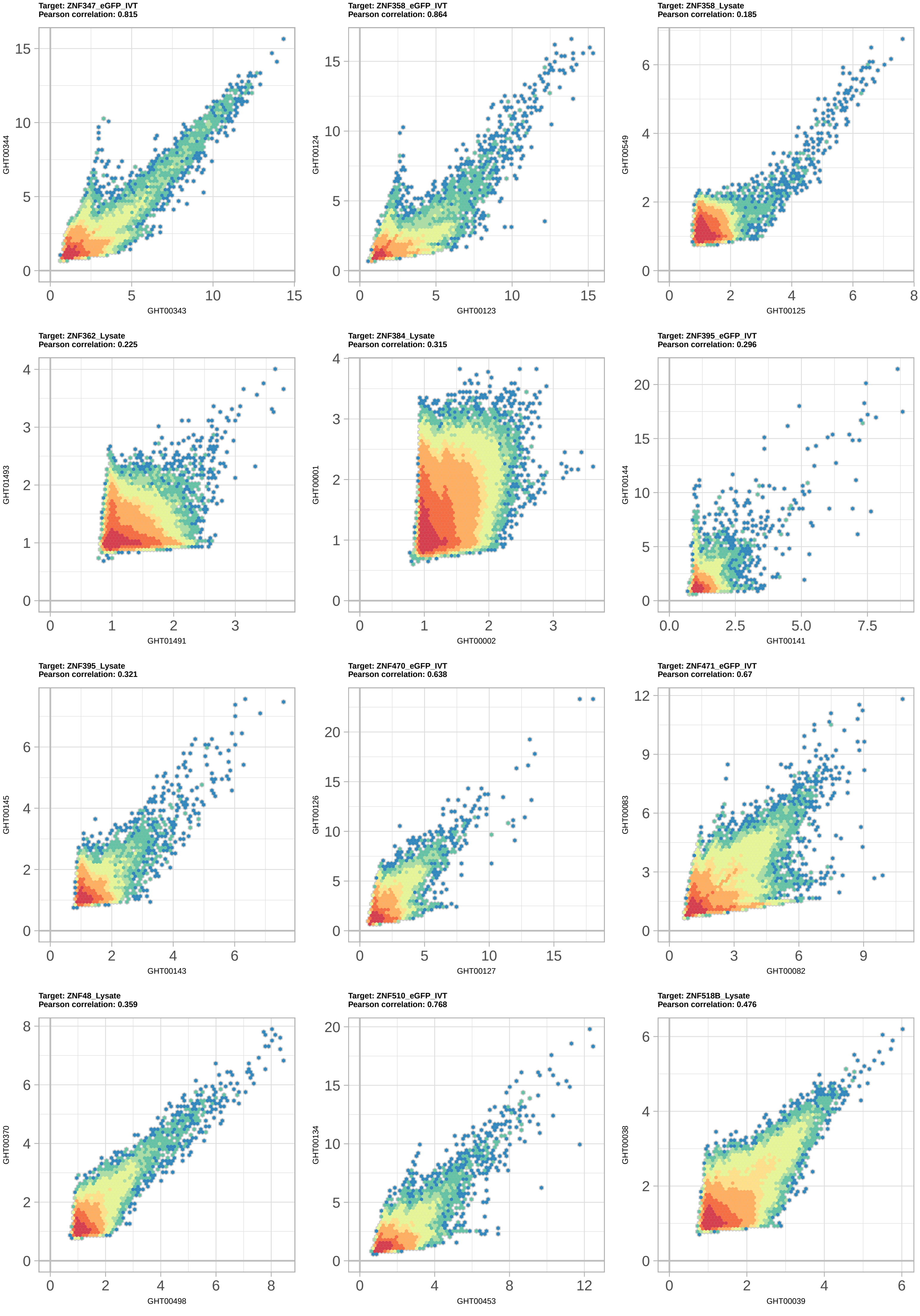
Target: ZNF347_eGFP_IVT
Pearson correlation: 0.815
GHT00344
GHT00343
Target: ZNF358_eGFP_IVT
Pearson correlation: 0.864
GHT00124
GHT00123
Target: ZNF358_Lysate
Pearson correlation: 0.185
GHT00549
GHT00125
Target: ZNF362_Lysate
Pearson correlation: 0.225
GHT01493
GHT01491
Target: ZNF384_Lysate
Pearson correlation: 0.315
GHT00001
GHT00002
Target: ZNF395_eGFP_IVT
Pearson correlation: 0.296
GHT00144
GHT00141
Target: ZNF395_Lysate
Pearson correlation: 0.321
GHT00145
GHT00143
Target: ZNF470_eGFP_IVT
Pearson correlation: 0.638
GHT00126
GHT00127
Target: ZNF471_eGFP_IVT
Pearson correlation: 0.67
GHT00083
GHT00082
Target: ZNF48_Lysate
Pearson correlation: 0.359
GHT00370
GHT00498
Target: ZNF510_eGFP_IVT
Pearson correlation: 0.768
GHT00134
GHT00453
Target: ZNF518B_Lysate
Pearson correlation: 0.476
GHT00038
GHT00039

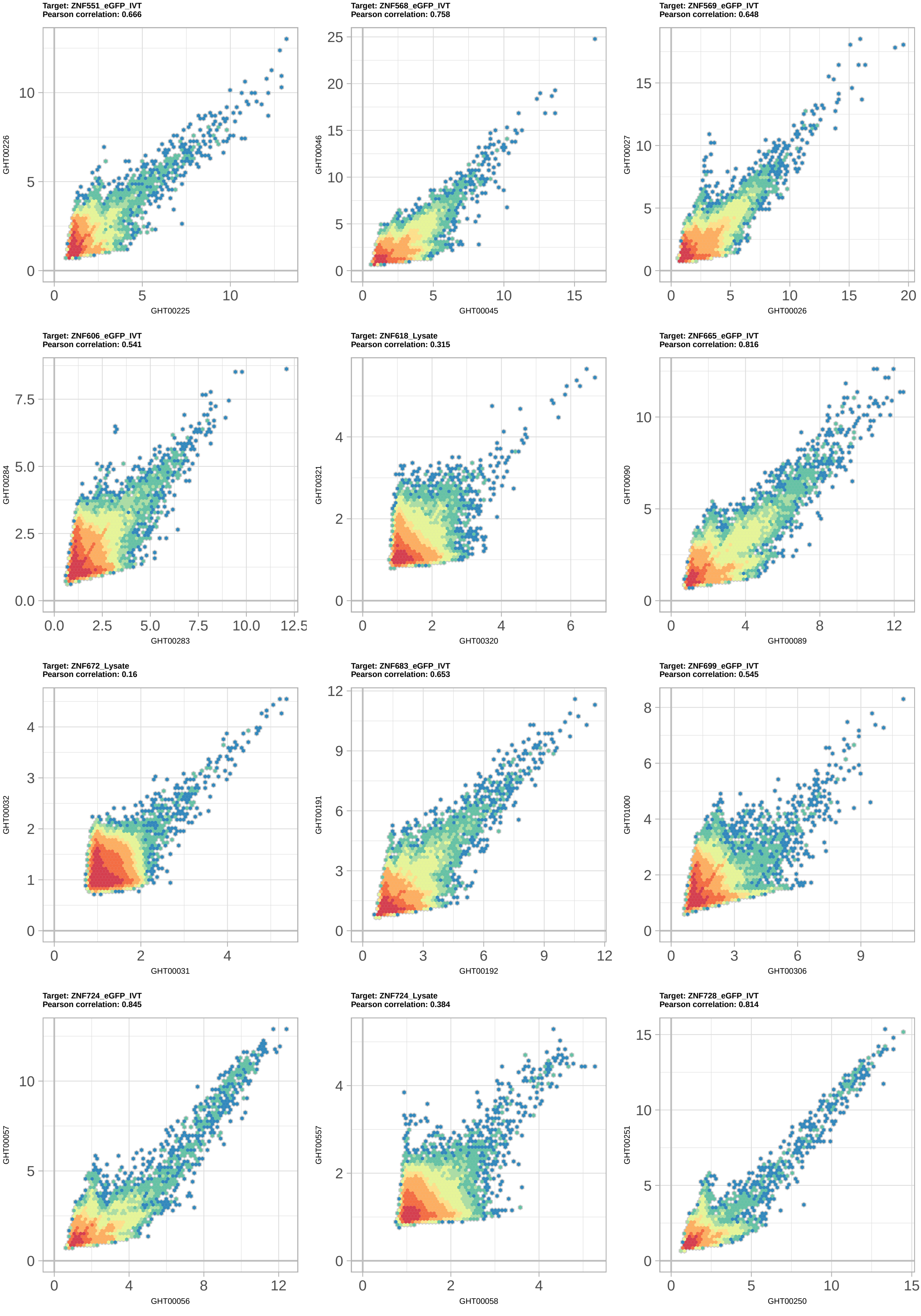
Target: ZNF551_eGFP_IVT
Pearson correlation: 0.666
GHT00226
GHT00225
Target: ZNF568_eGFP_IVT
Pearson correlation: 0.758
GHT00046
GHT00045
Target: ZNF569_eGFP_IVT
Pearson correlation: 0.648
GHT00027
GHT00026
Target: ZNF606_eGFP_IVT
Pearson correlation: 0.541
GHT00284
GHT00283
Target: ZNF618_Lysate
Pearson correlation: 0.315
GHT00321
GHT00320
Target: ZNF665_eGFP_IVT
Pearson correlation: 0.816
GHT00090
GHT00089
Target: ZNF672_Lysate
Pearson correlation: 0.16
GHT00032
GHT00031
Target: ZNF683_eGFP_IVT
Pearson correlation: 0.653
GHT00191
GHT00192
Target: ZNF699_eGFP_IVT
Pearson correlation: 0.545
GHT01000
GHT00306
Target: ZNF724_eGFP_IVT
Pearson correlation: 0.845
GHT00057
GHT00056
Target: ZNF724_Lysate
Pearson correlation: 0.384
GHT00557
GHT00058
Target: ZNF728_eGFP_IVT
Pearson correlation: 0.814
GHT00251
GHT00250

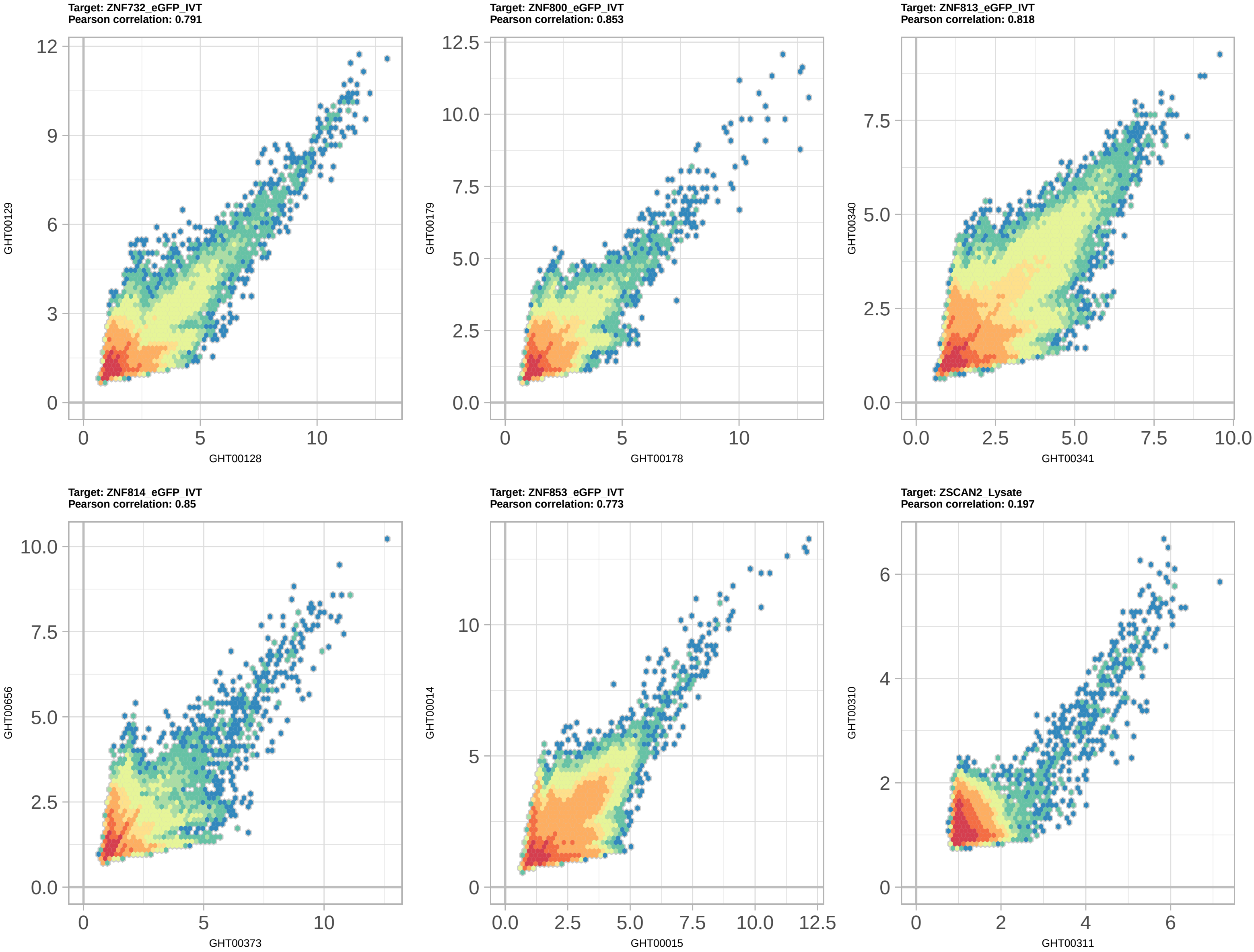
Target: ZNF732_eGFP_IVT
Pearson correlation: 0.791
GHT00129
GHT00128
Target: ZNF800_eGFP_IVT
Pearson correlation: 0.853
GHT00179
GHT00178
Target: ZNF813_eGFP_IVT
Pearson correlation: 0.818
GHT00340
GHT00341
Target: ZNF814_eGFP_IVT
Pearson correlation: 0.85
GHT00656
GHT00373
Target: ZNF853_eGFP_IVT
Pearson correlation: 0.773
GHT00014
GHT00015
Target: ZSCAN2_Lysate
Pearson correlation: 0.197
GHT00310
GHT00311
