# Supplementary material for: GHT-SELEX demonstrates unexpectedly high intrinsic sequence specificity and complex DNA binding of many human transcription factors
Source: Nat Methods. 2026 Aug 5;23(9):1775–85. doi: 10.1038/s41592-026-03177-9 (PMC13541613; doi:10.1038/s41592-026-03177-9)

BATF2

Top GRECO-BIT/MEX PWM: PWM000070

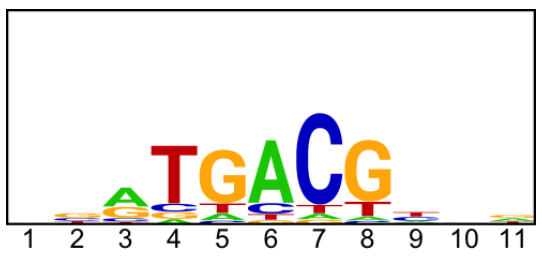

Location of top GRECO-BIT/MEX PWM in MAGIX peaks (up to the top 10,000)

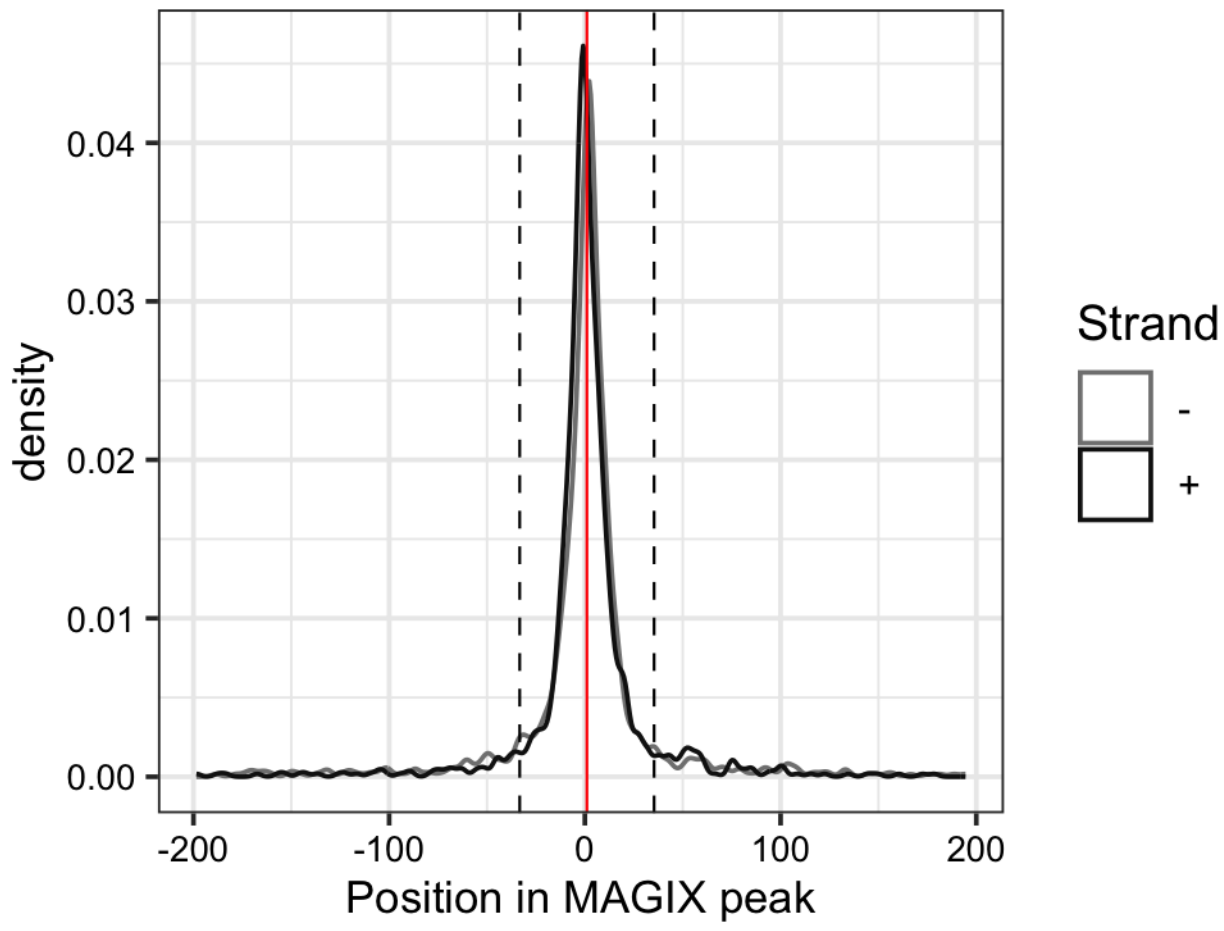

AUROC of top GRECO-BIT/MEX PWM in sliding windows (500 peaks, 50 peak step) along the MAGIX peaks (up to the top 75,000) ranked by the MAGIX enrichment coefficient and fraction of overlapping ChIP peaks in the same window

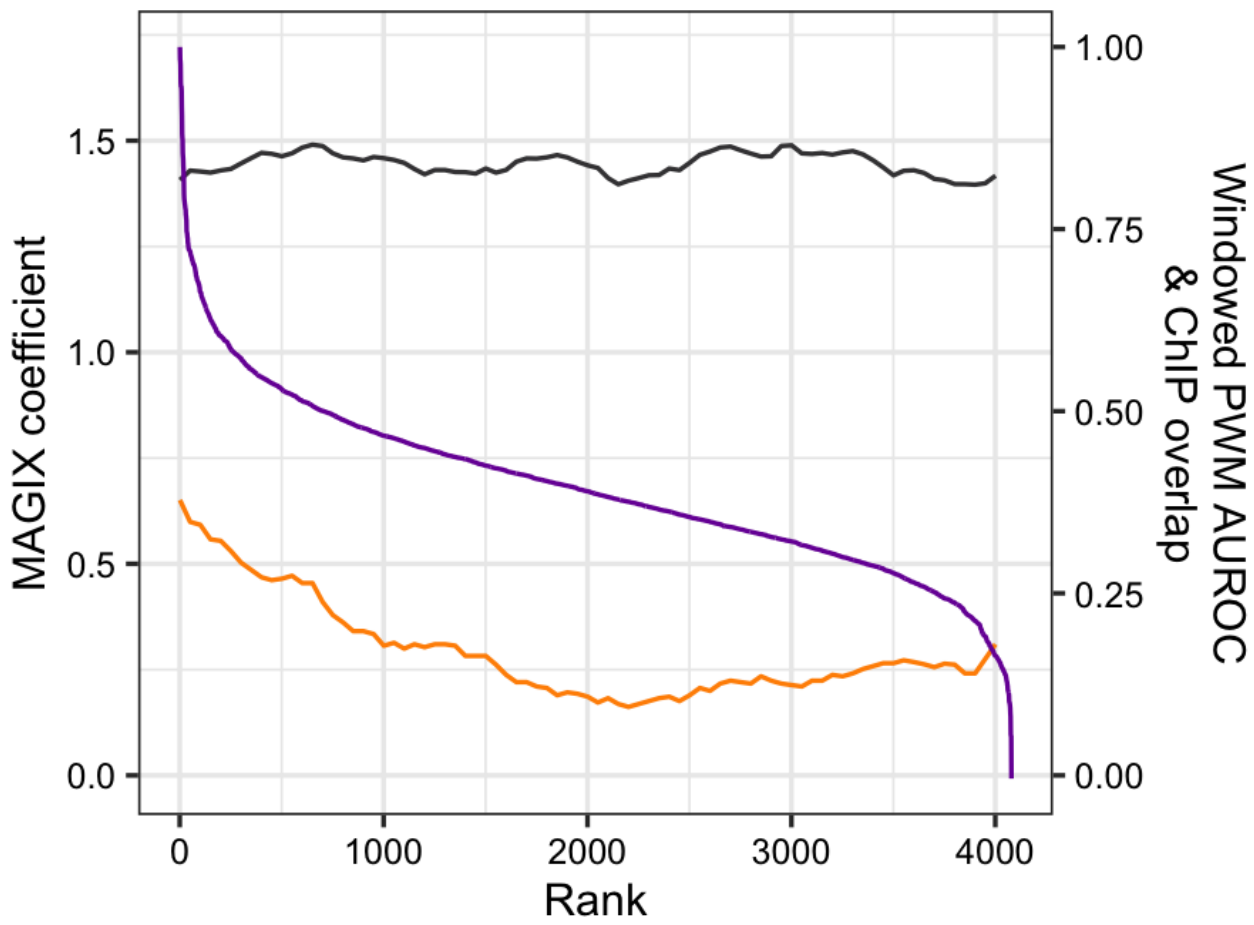

CAMTA1

Top GRECO-BIT/MEX PWM: PWM000117

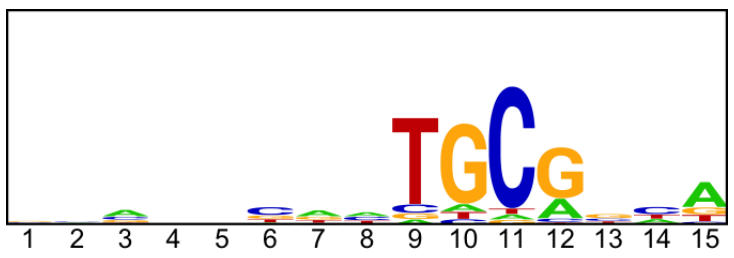

Location of top GRECO-BIT/MEX PWM in MAGIX peaks (up to the top 10,000)

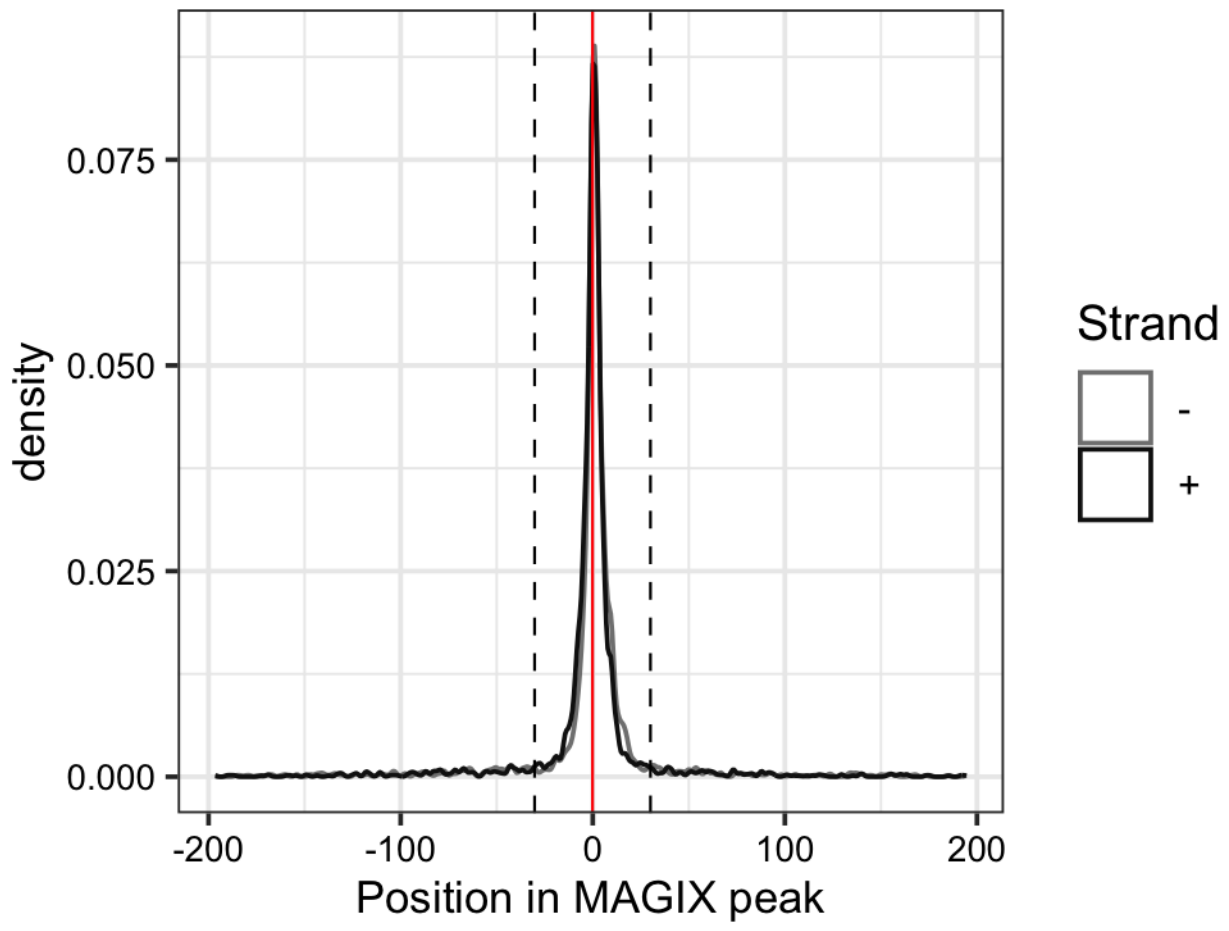

AUROC of top GRECO-BIT/MEX PWM in sliding windows (500 peaks, 50 peak step) along the MAGIX peaks (up to the top 75,000) ranked by the MAGIX enrichment coefficient and fraction of overlapping ChIP peaks in the same window

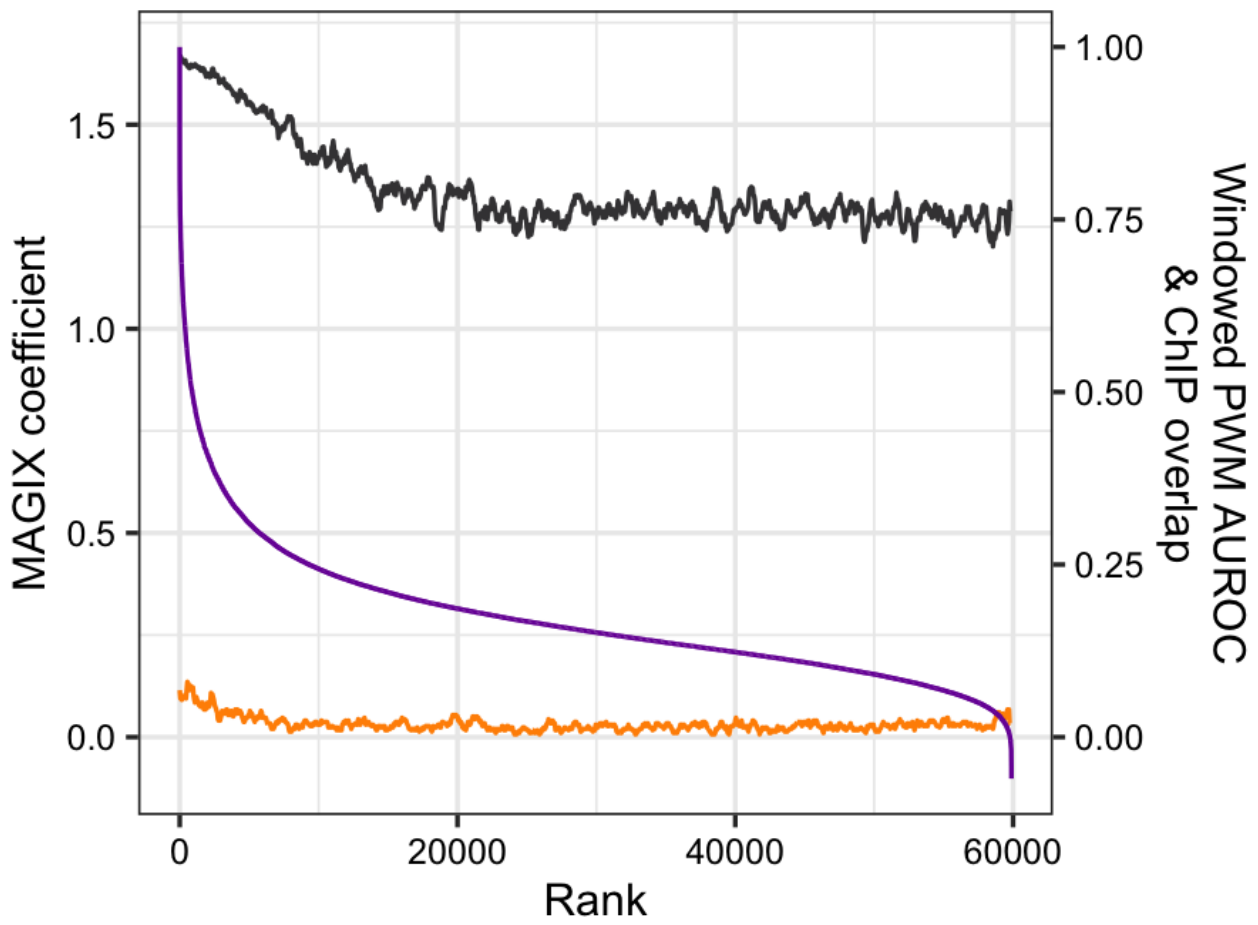

CAMTA2

Top GRECO-BIT/MEX PWM: PWM000141

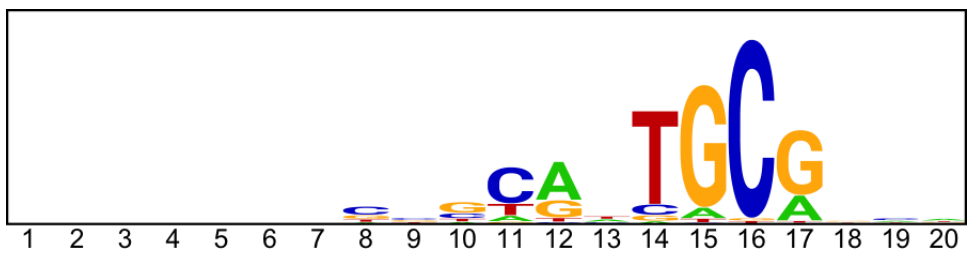

Location of top GRECO-BIT/MEX PWM in MAGIX peaks (up to the top 10,000)

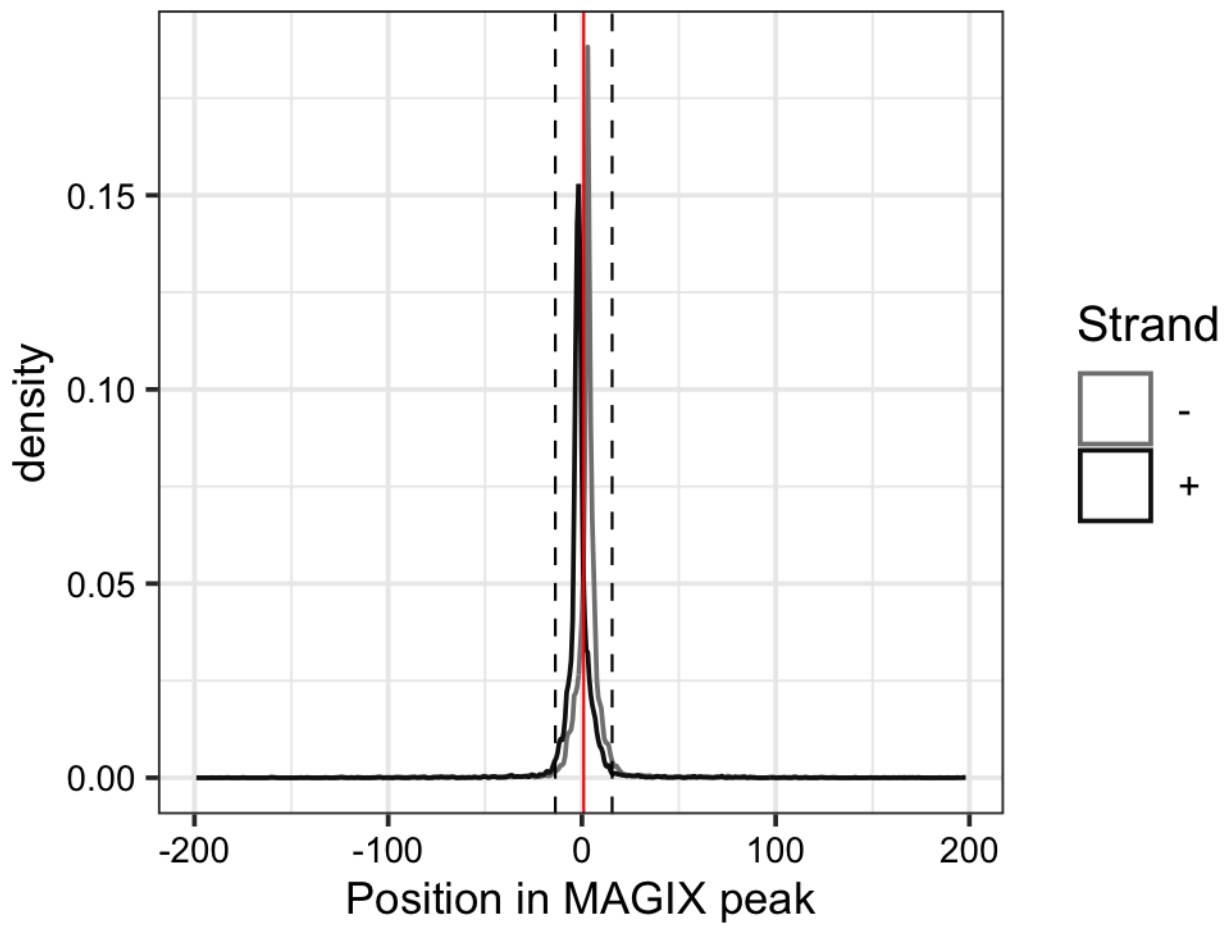

AUROC of top GRECO-BIT/MEX PWM in sliding windows (500 peaks, 50 peak step) along the MAGIX peaks (up to the top 75,000) ranked by the MAGIX enrichment coefficient and fraction of overlapping ChIP peaks in the same window

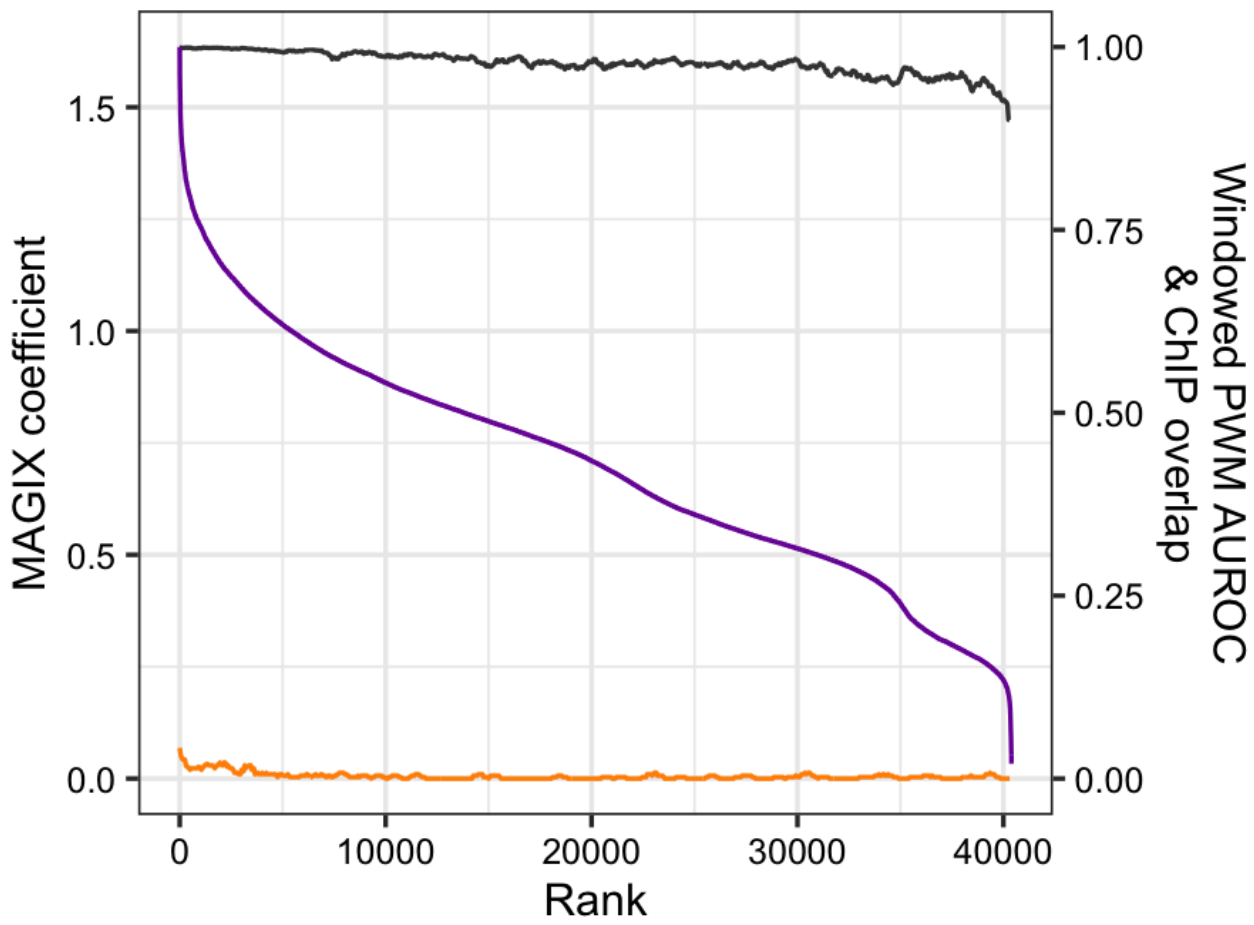

CASZ1.DBD1

Top GRECO-BIT/MEX PWM: PWM000184

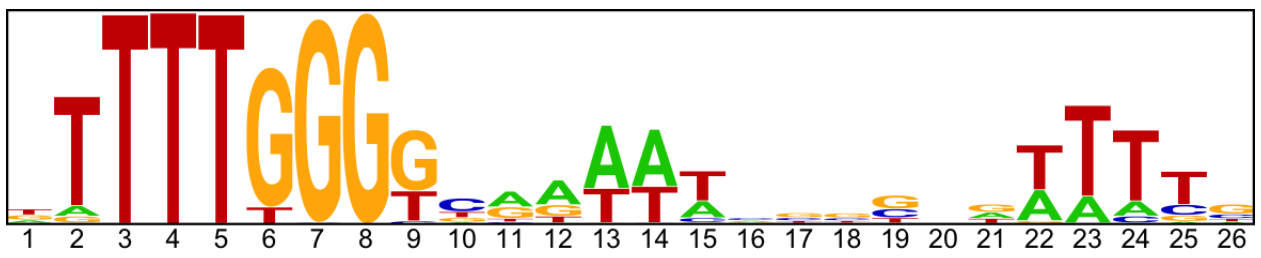

Location of top GRECO-BIT/MEX PWM in MAGIX peaks (up to the top 10,000)

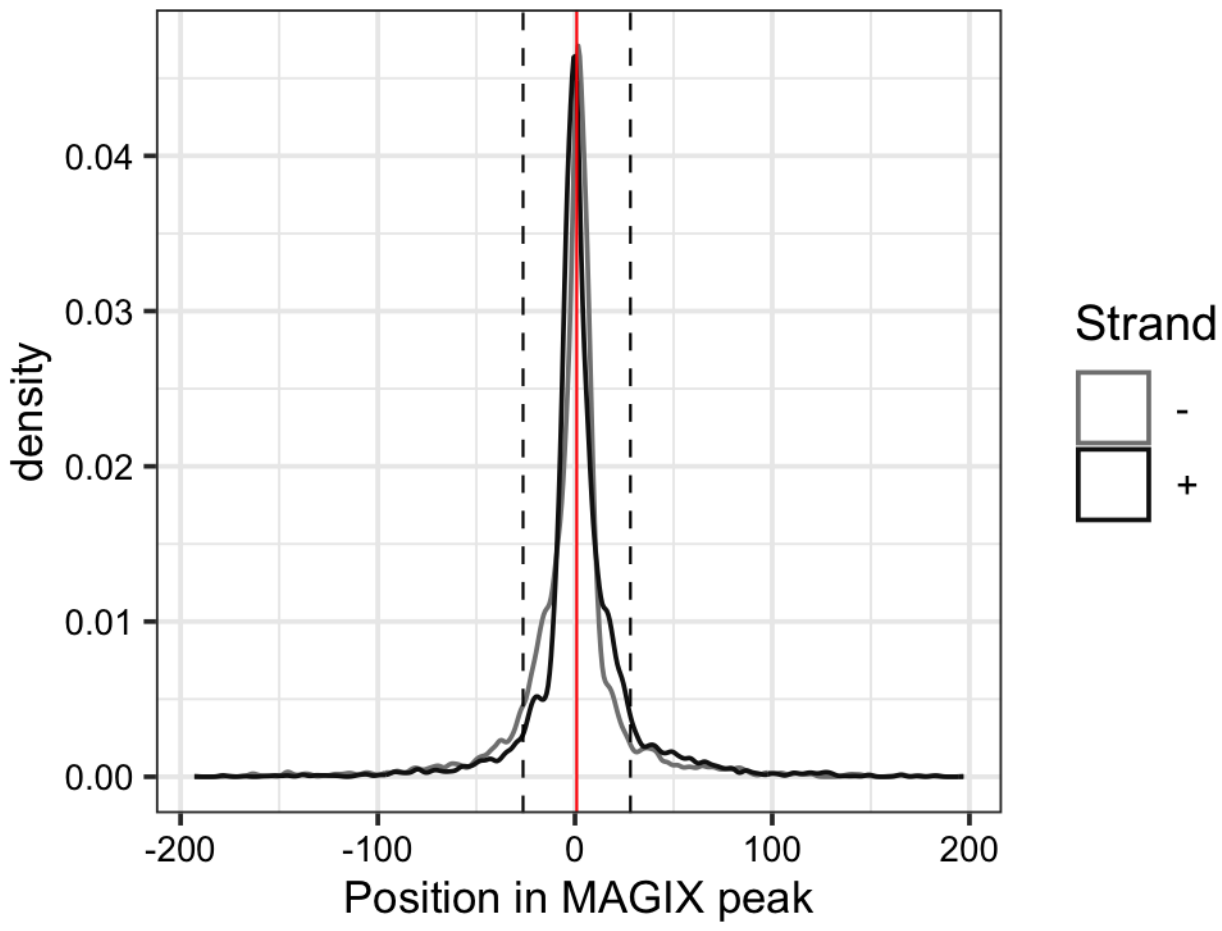

CASZ1.FL

Top GRECO-BIT/MEX PWM: PWM000181

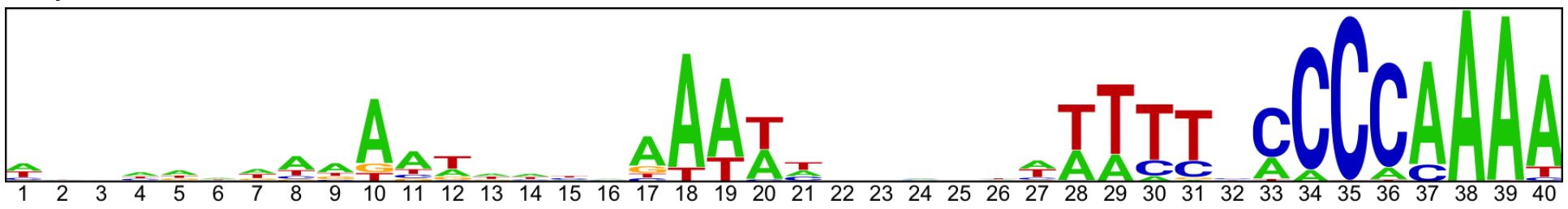

Location of top GRECO-BIT/MEX PWM in MAGIX peaks (up to the top 10,000)

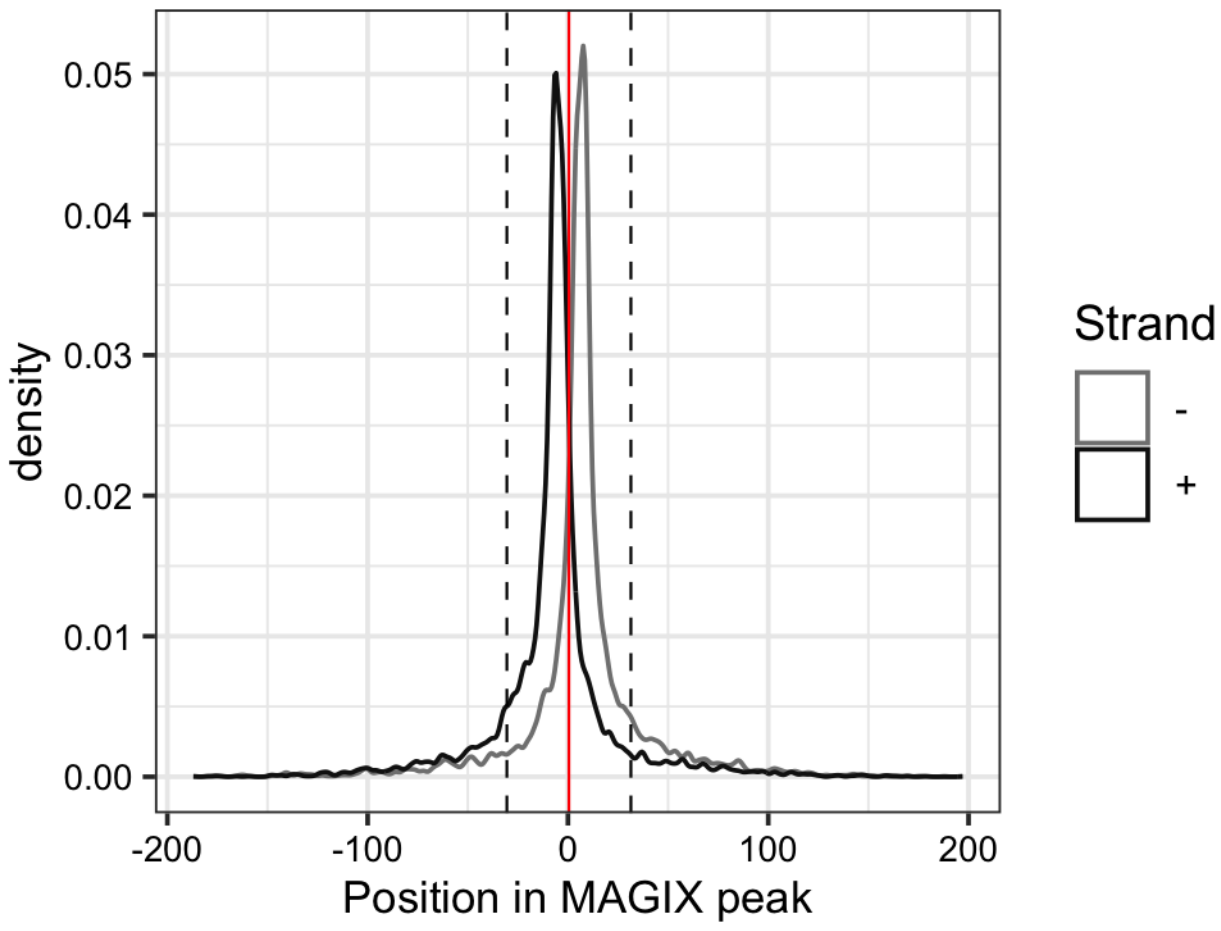

CGGBP1

Top GRECO-BIT/MEX PWM: PWM000216

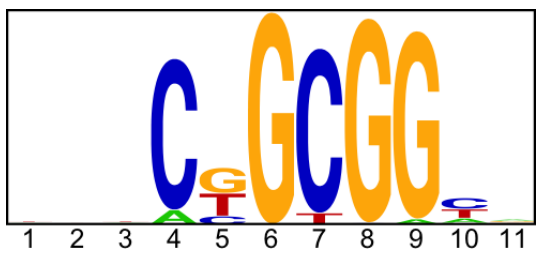

Location of top GRECO-BIT/MEX PWM in MAGIX peaks (up to the top 10,000)

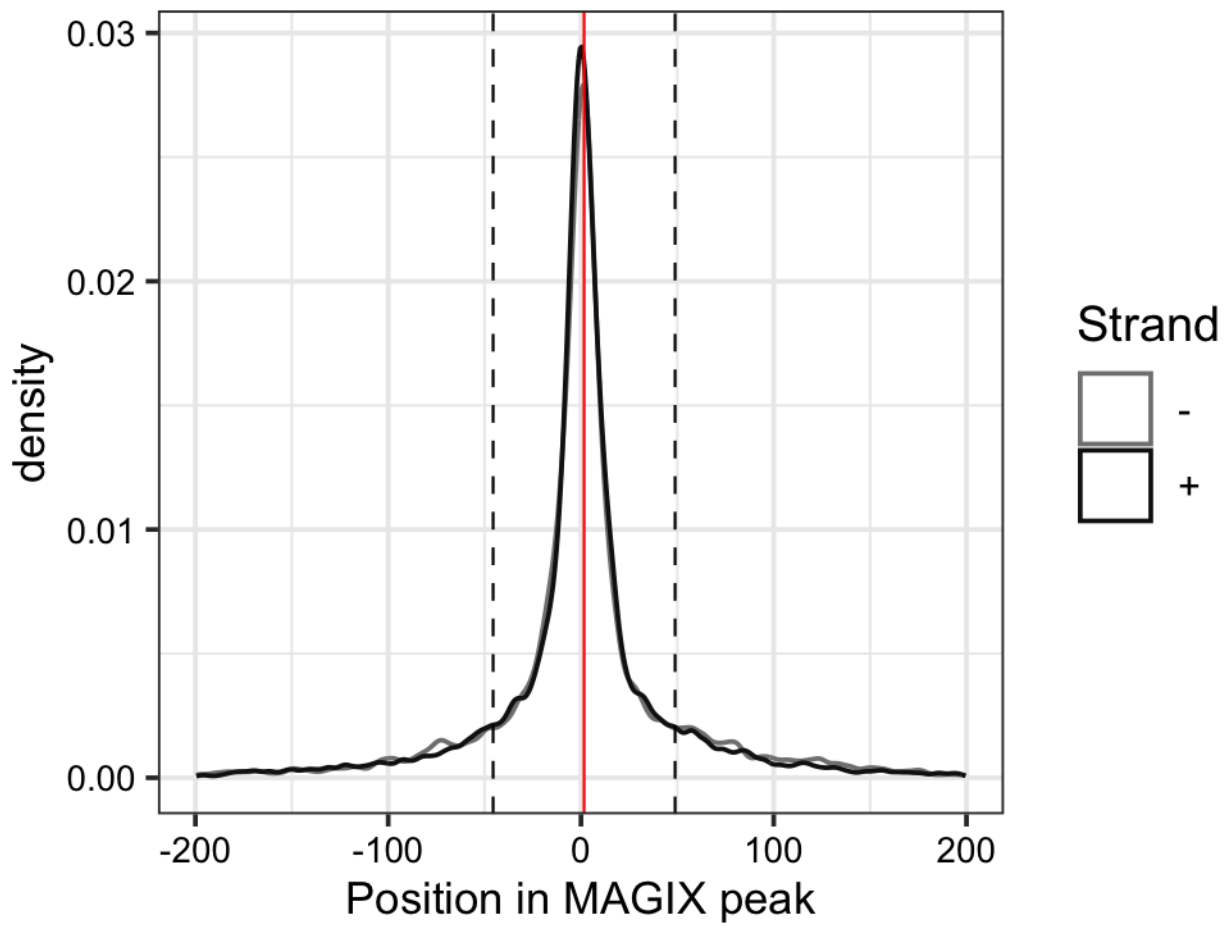

AUROC of top GRECO-BIT/MEX PWM in sliding windows (500 peaks, 50 peak step) along the MAGIX peaks (up to the top 75,000) ranked by the MAGIX enrichment coefficient and fraction of overlapping ChIP peaks in the same window

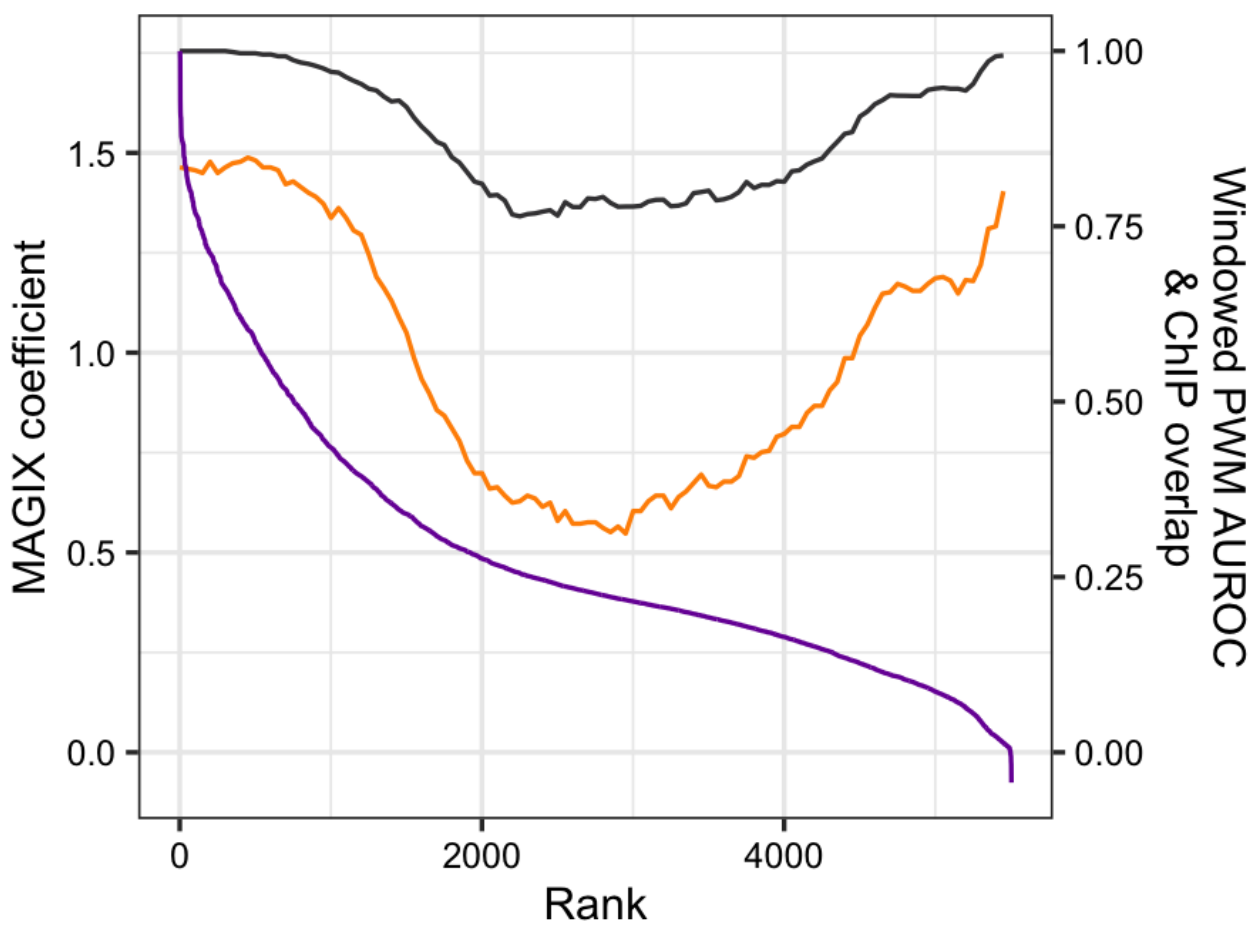

CREB3L3

Top GRECO-BIT/MEX PWM: PWM000262

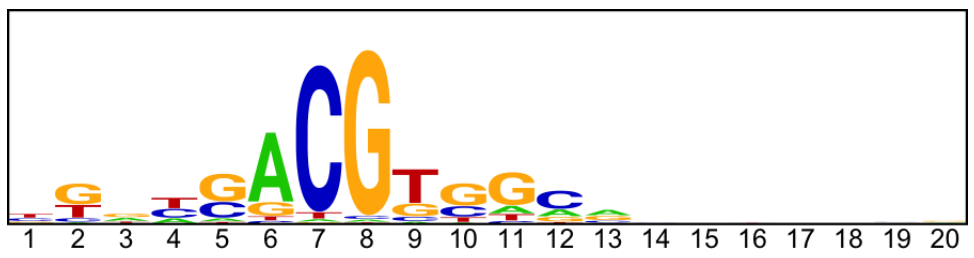

Location of top GRECO-BIT/MEX PWM in MAGIX peaks (up to the top 10,000)

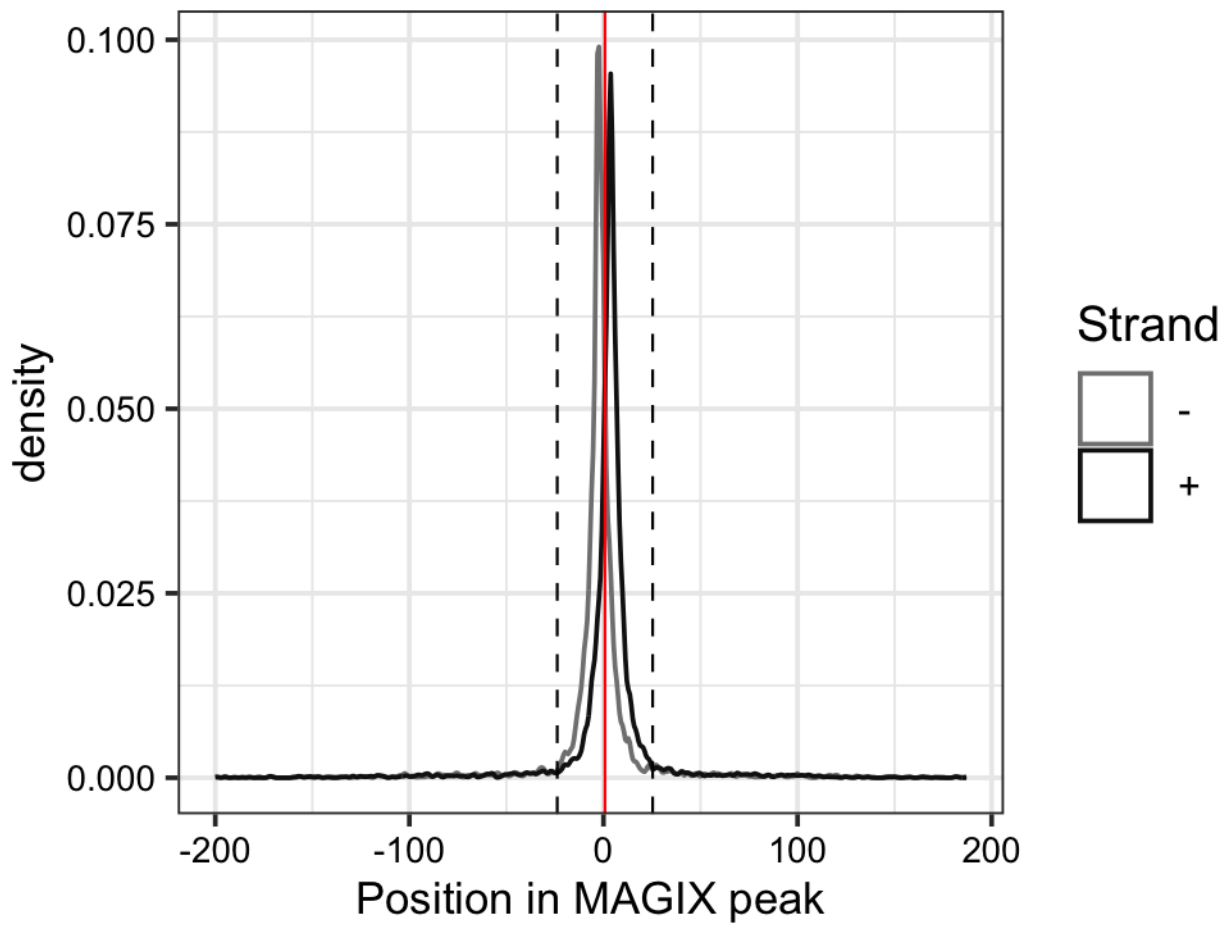

AUROC of top GRECO-BIT/MEX PWM in sliding windows (500 peaks, 50 peak step) along the MAGIX peaks (up to the top 75,000) ranked by the MAGIX enrichment coefficient and fraction of overlapping ChIP peaks in the same window

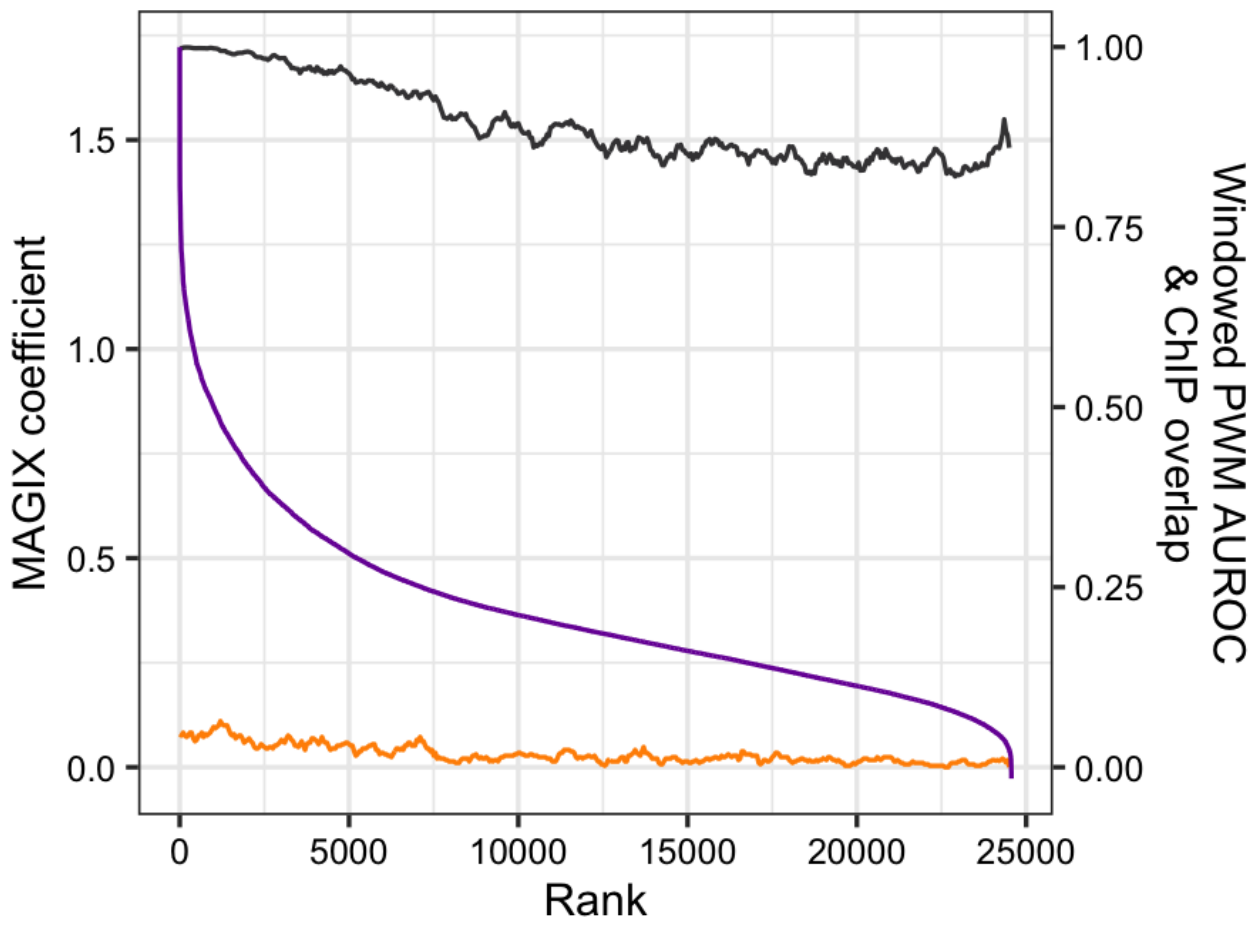

CTCF

Top GRECO-BIT/MEX PWM: PWM000307

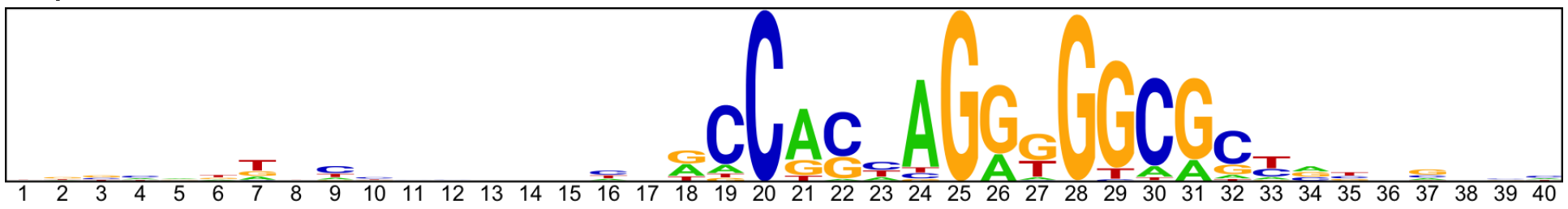

Location of top GRECO-BIT/MEX PWM in MAGIX peaks (up to the top 10,000)

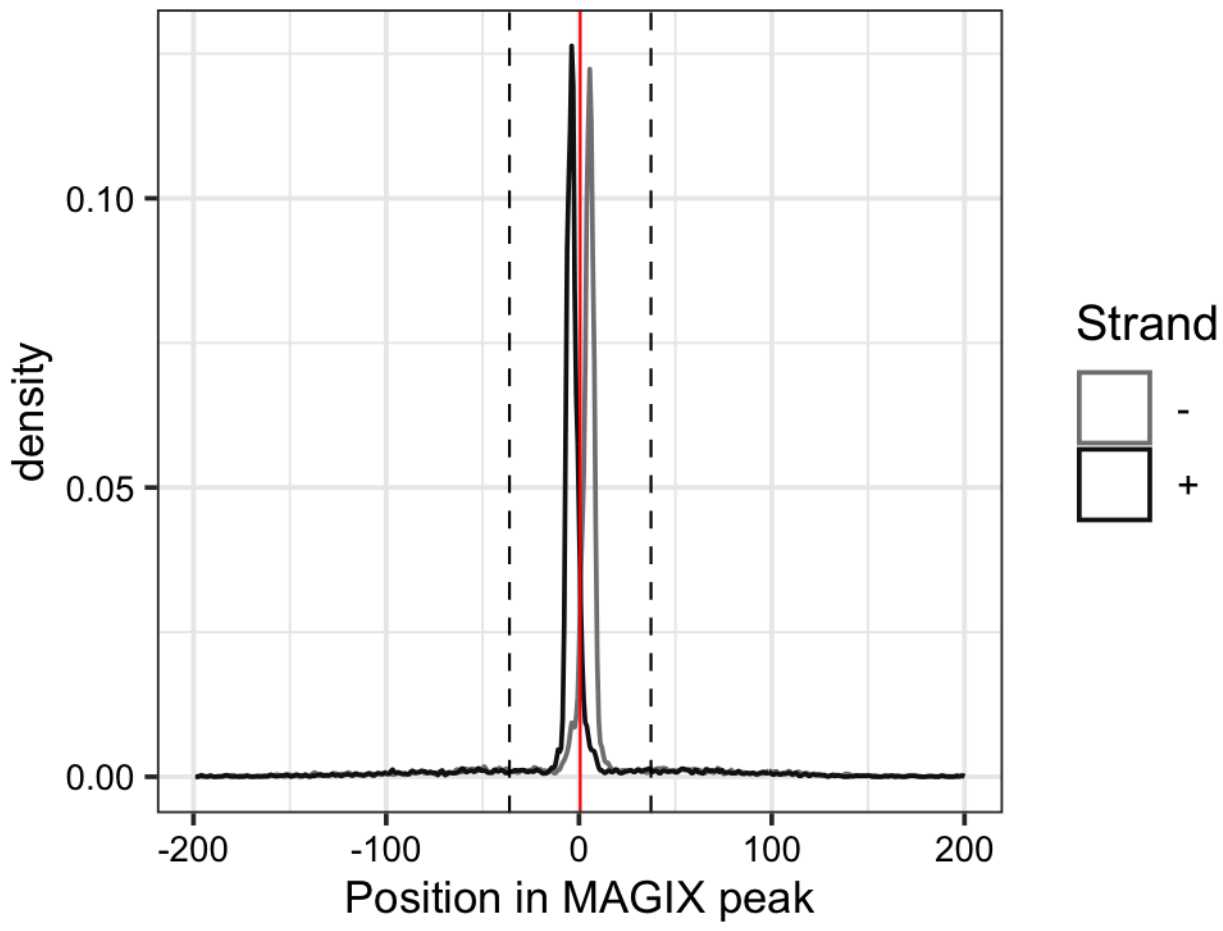

AUROC of top GRECO-BIT/MEX PWM in sliding windows (500 peaks, 50 peak step) along the MAGIX peaks (up to the top 75,000) ranked by the MAGIX enrichment coefficient and fraction of overlapping ChIP peaks in the same window

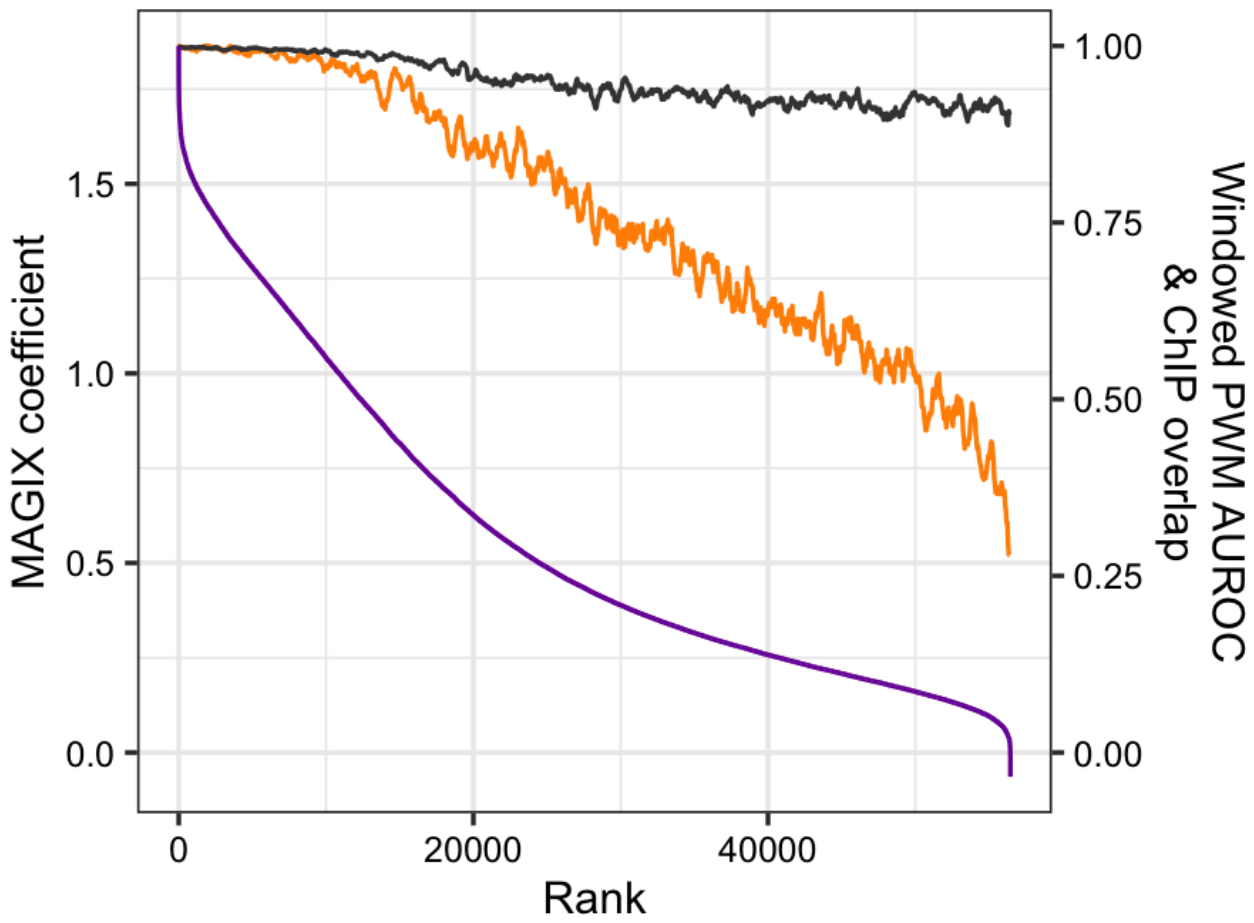

CXXC4

Top GRECO-BIT/MEX PWM: PWM000326

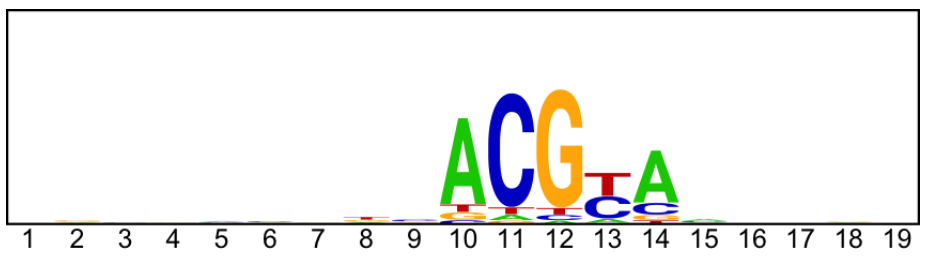

Location of top GRECO-BIT/MEX PWM in MAGIX peaks (up to the top 10,000)

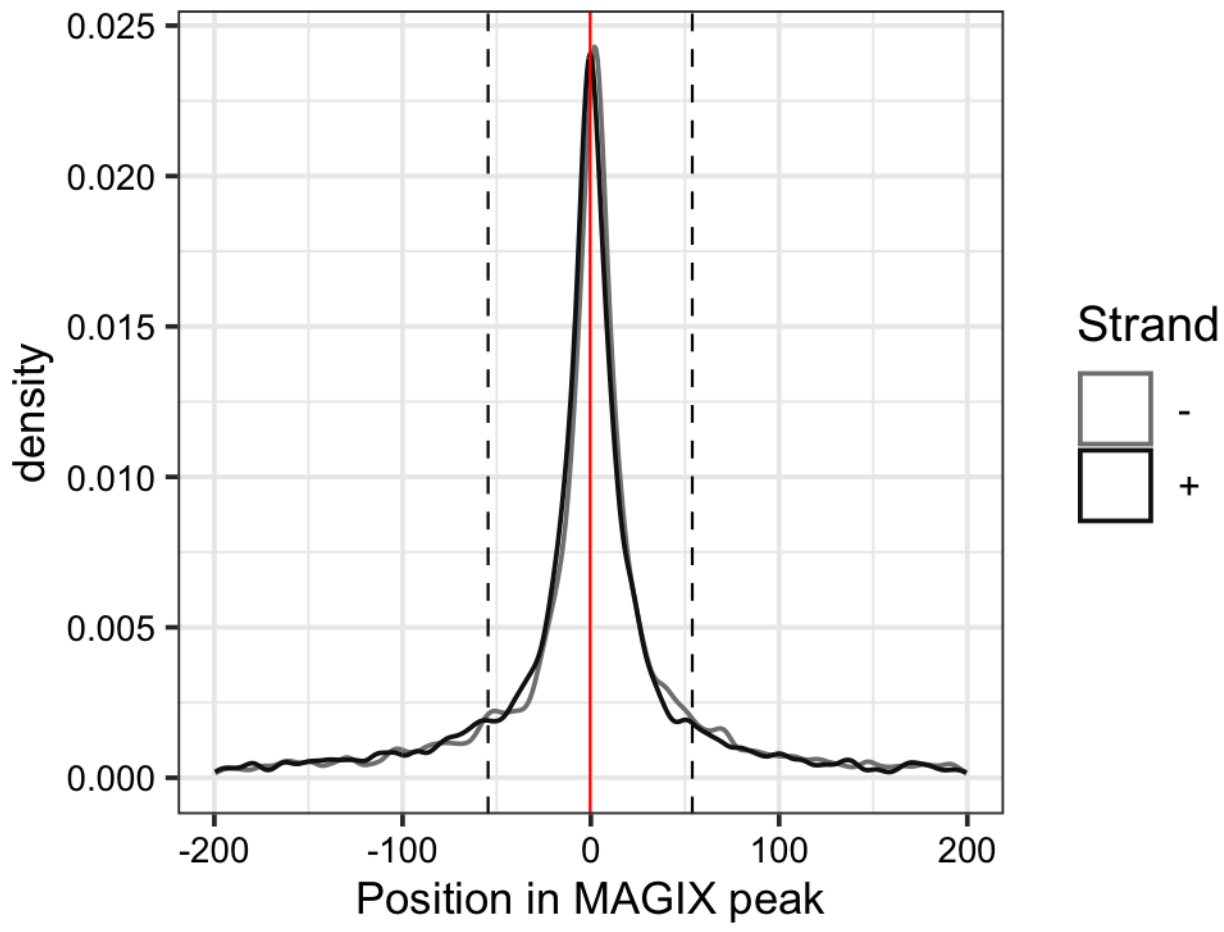

AUROC of top GRECO-BIT/MEX PWM in sliding windows (500 peaks, 50 peak step) along the MAGIX peaks (up to the top 75,000) ranked by the MAGIX enrichment coefficient and fraction of overlapping ChIP peaks in the same window

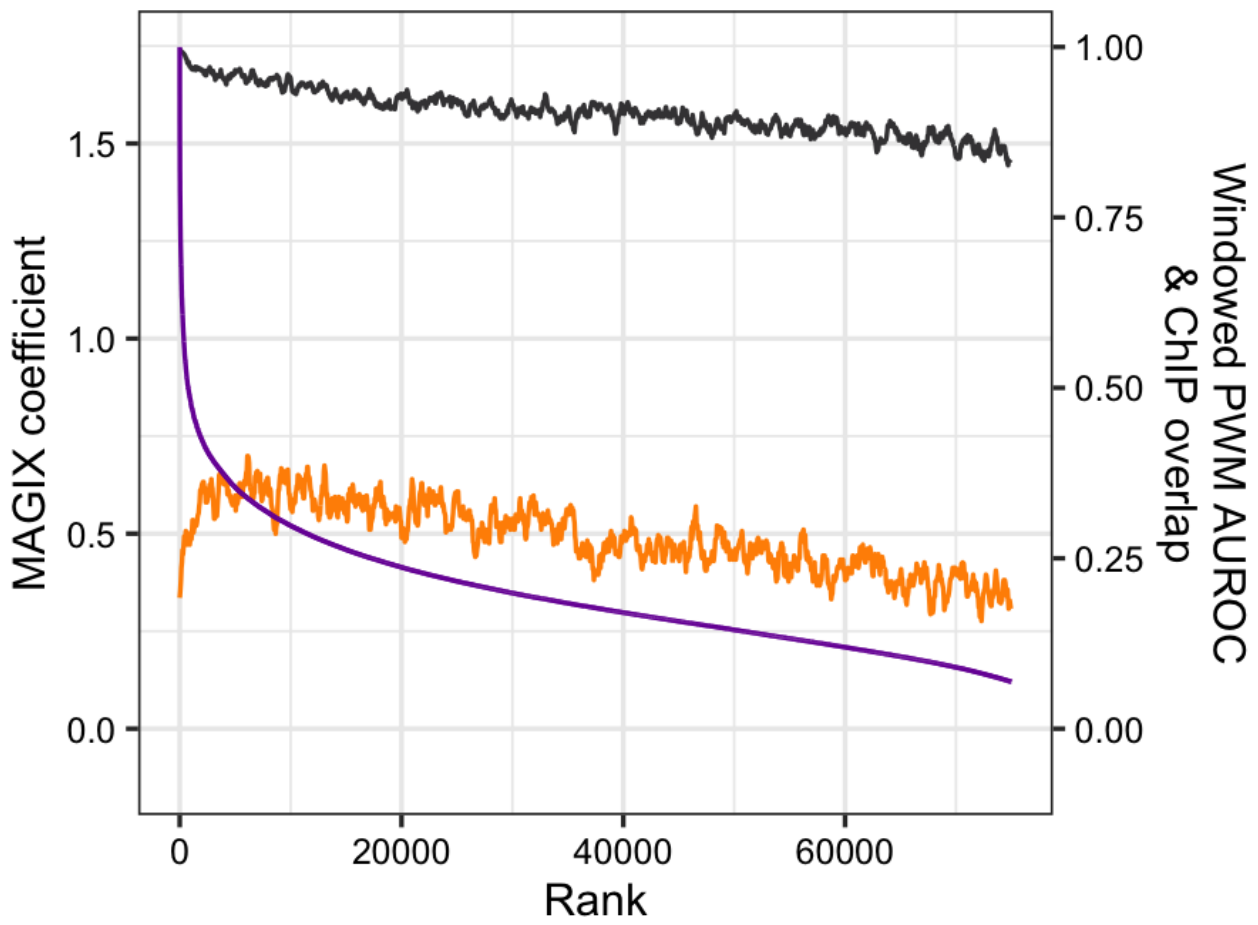

DMTF1

Top GRECO-BIT/MEX PWM: PWM000383

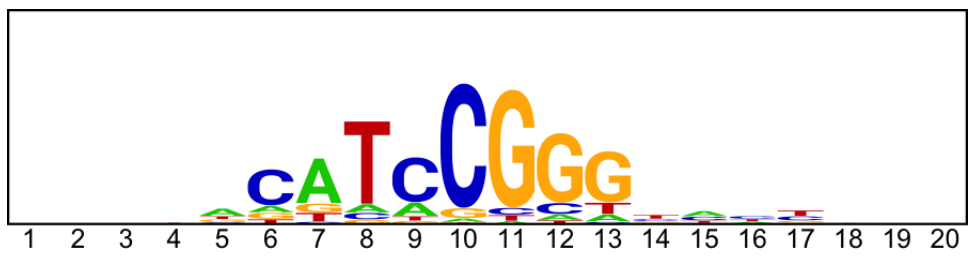

Location of top GRECO-BIT/MEX PWM in MAGIX peaks (up to the top 10,000)

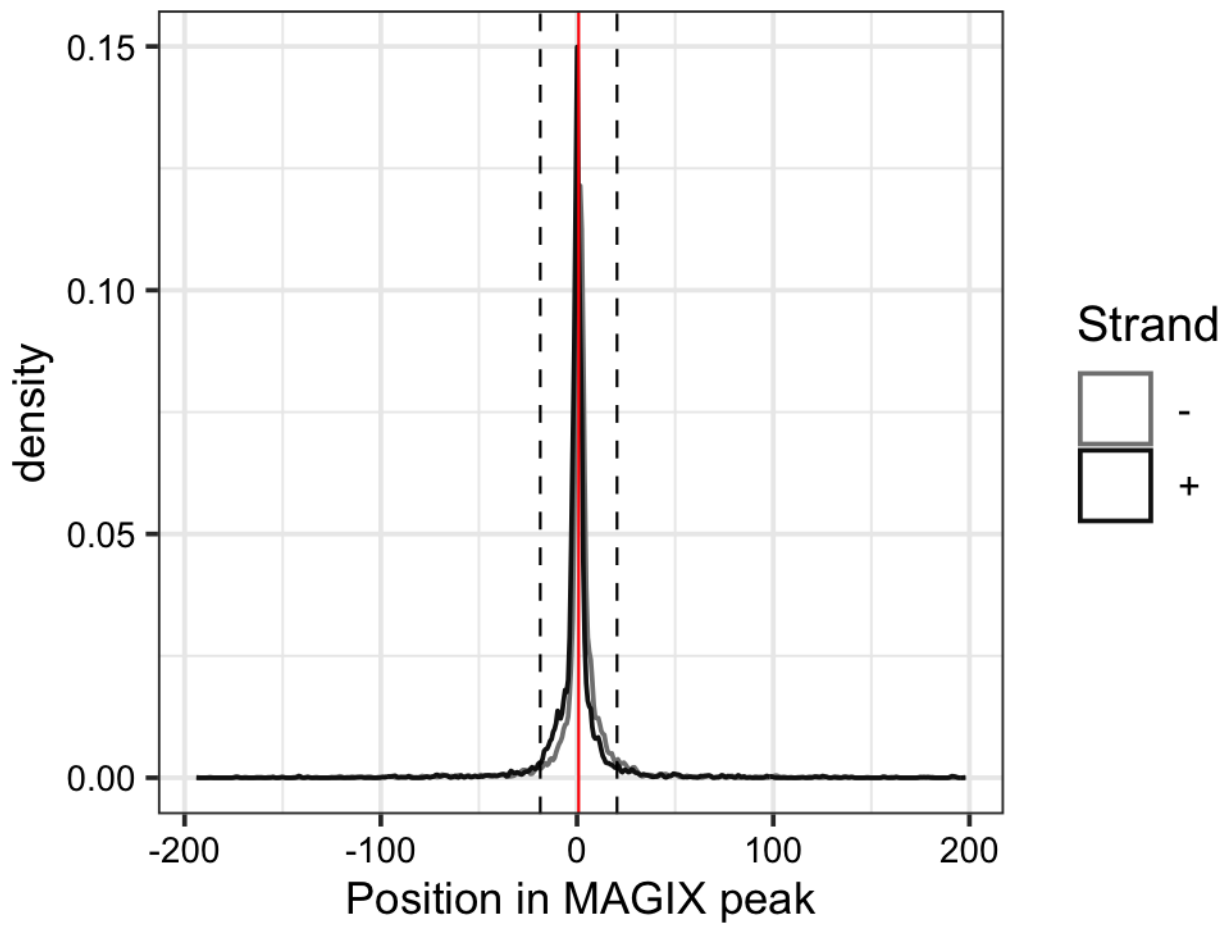

AUROC of top GRECO-BIT/MEX PWM in sliding windows (500 peaks, 50 peak step) along the MAGIX peaks (up to the top 75,000) ranked by the MAGIX enrichment coefficient and fraction of overlapping ChIP peaks in the same window

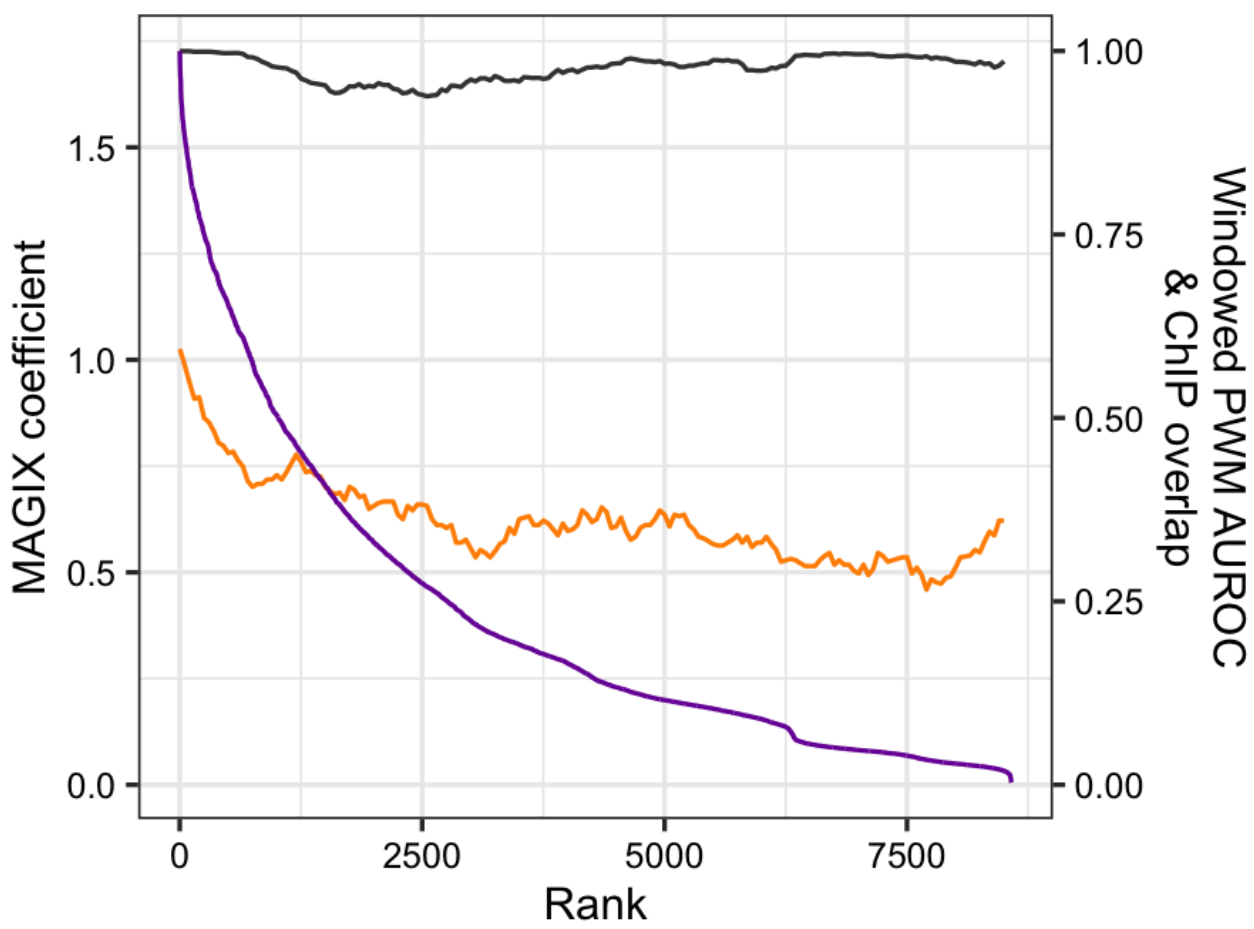

DNTTIP1

Top GRECO-BIT/MEX PWM: PWM000422

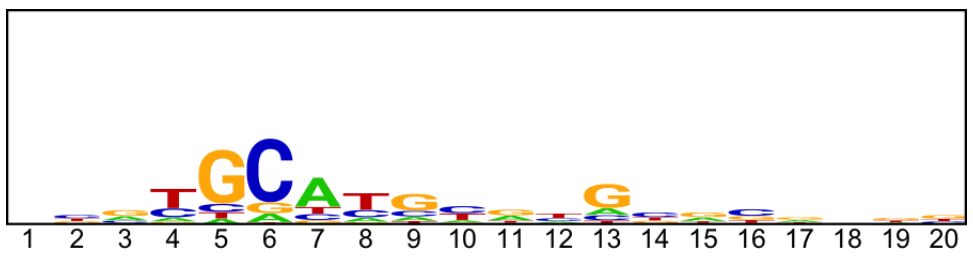

Location of top GRECO-BIT/MEX PWM in MAGIX peaks (up to the top 10,000)

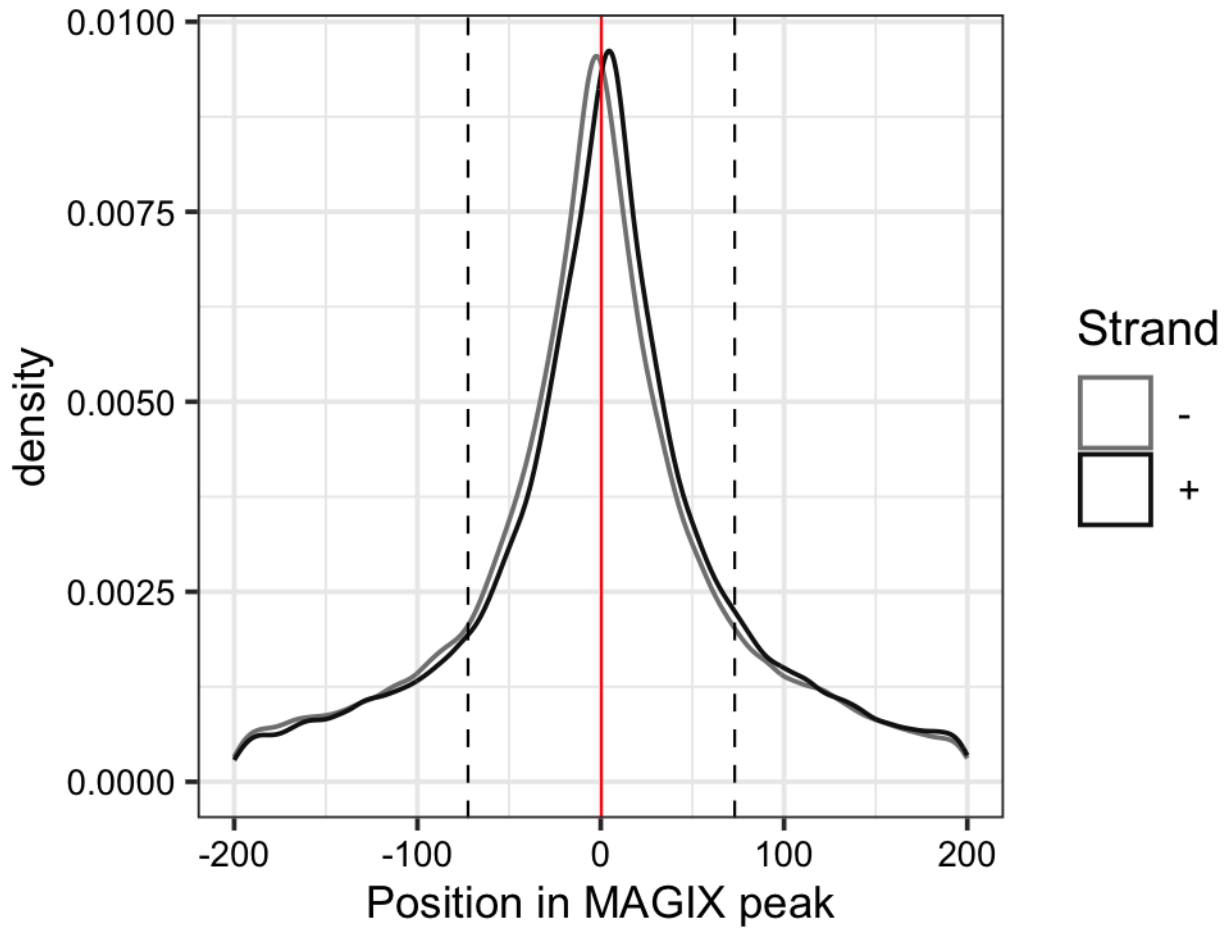

AUROC of top GRECO-BIT/MEX PWM in sliding windows (500 peaks, 50 peak step) along the MAGIX peaks (up to the top 75,000) ranked by the MAGIX enrichment coefficient and fraction of overlapping ChIP peaks in the same window

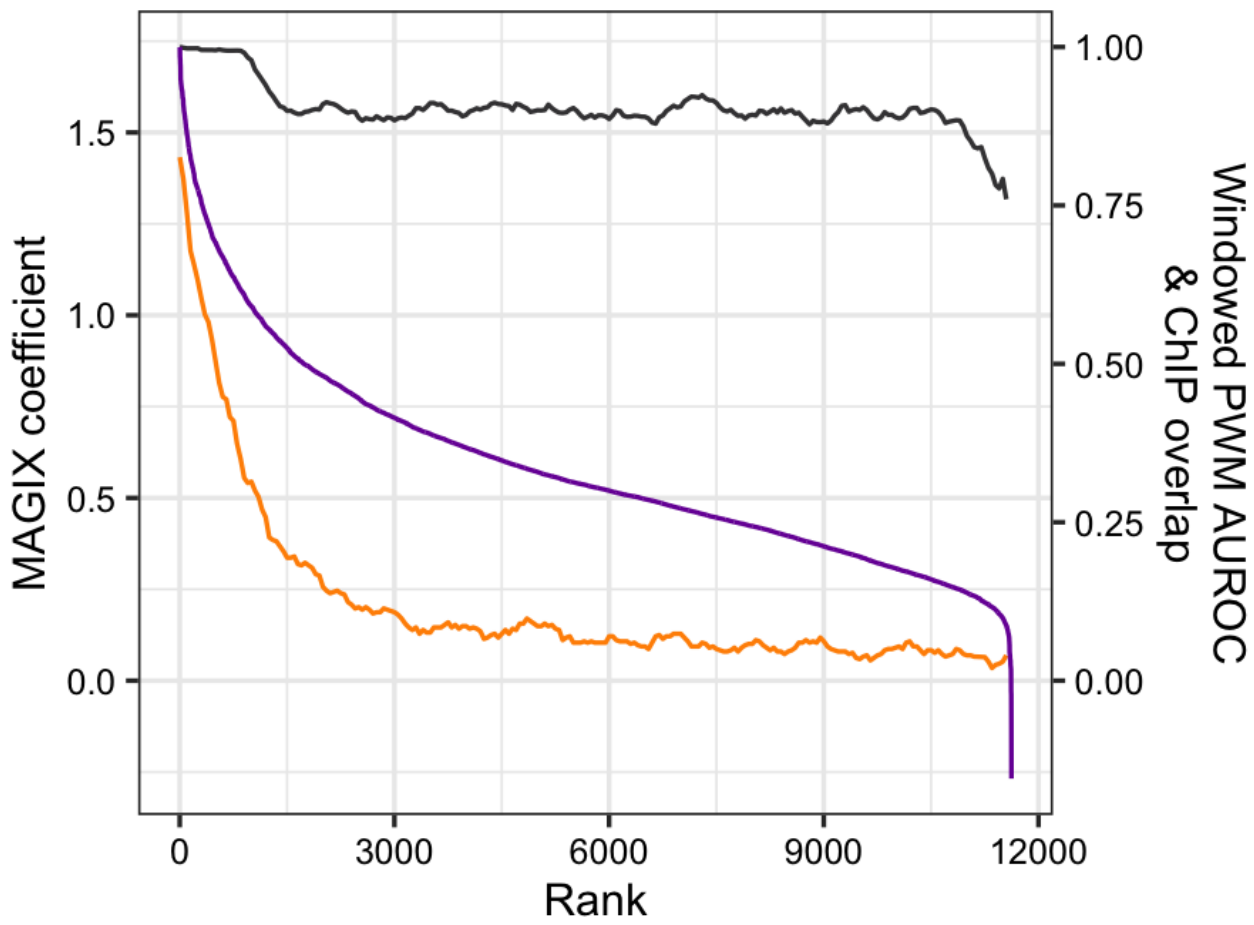

ELF3

Top GRECO-BIT/MEX PWM: PWM000447

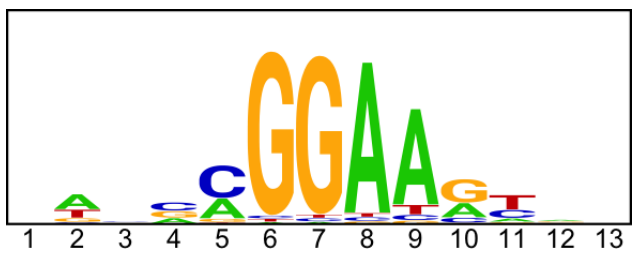

Location of top GRECO-BIT/MEX PWM in MAGIX peaks (up to the top 10,000)

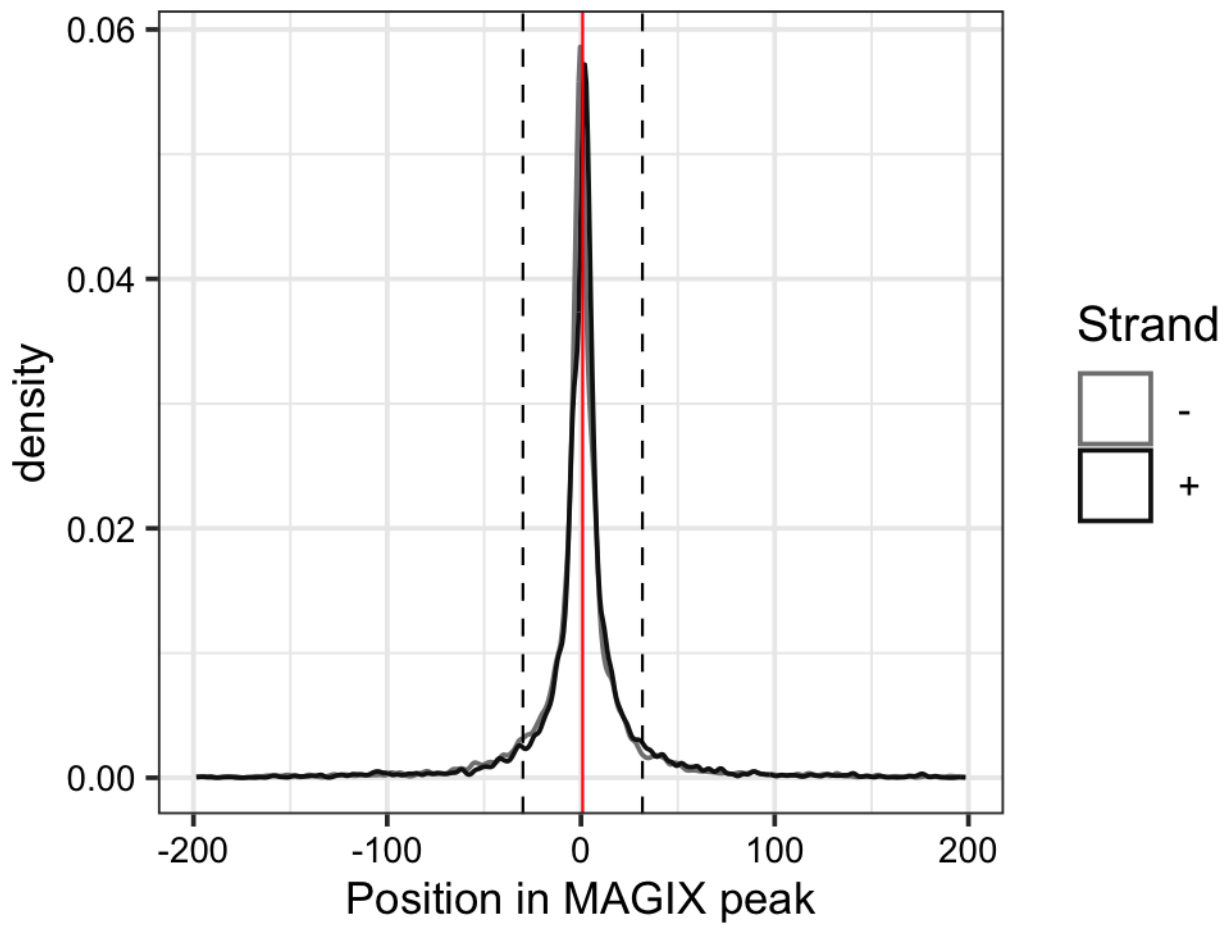

AUROC of top GRECO-BIT/MEX PWM in sliding windows (500 peaks, 50 peak step) along the MAGIX peaks (up to the top 75,000) ranked by the MAGIX enrichment coefficient and fraction of overlapping ChIP peaks in the same window

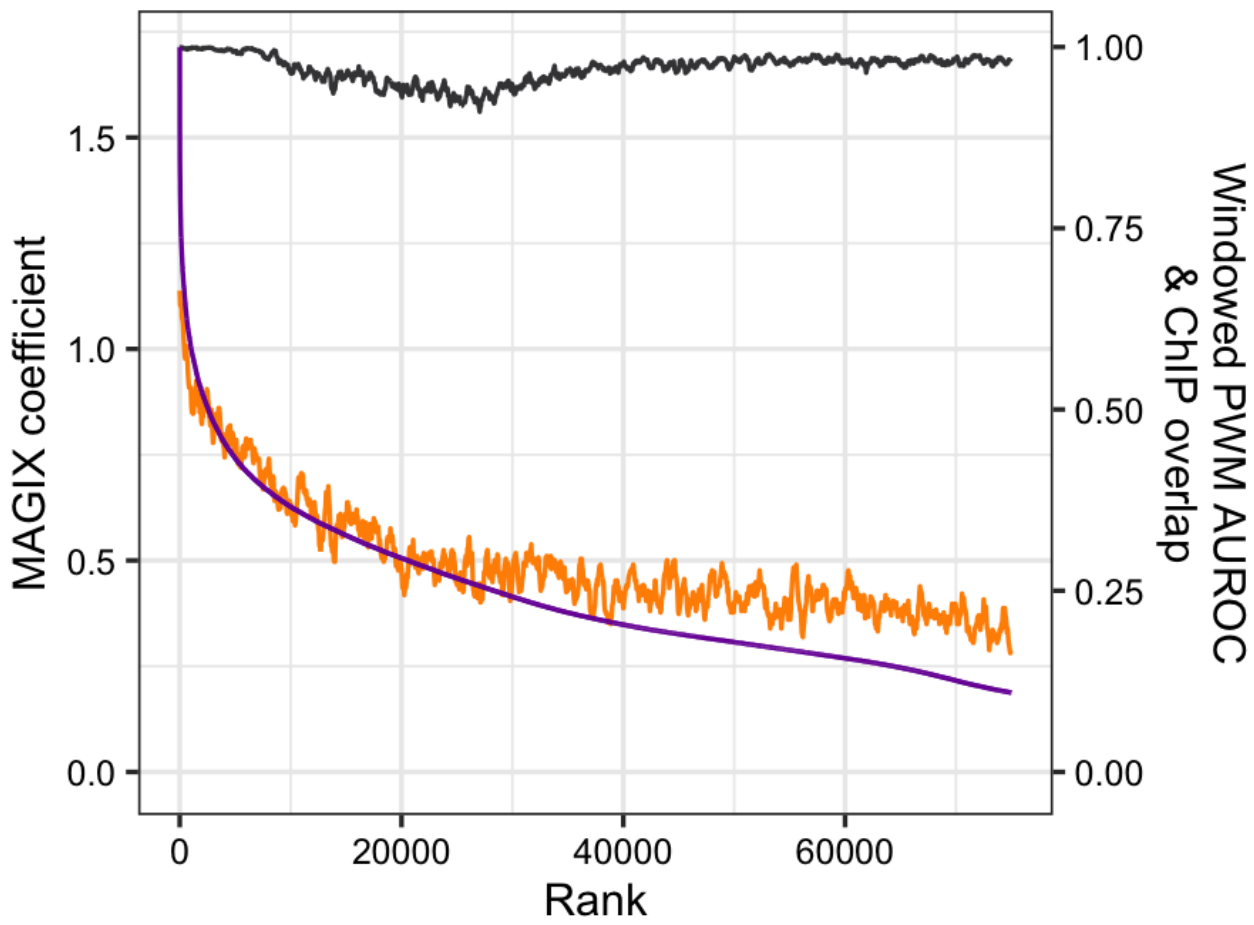

FAM200B

Top GRECO-BIT/MEX PWM: PWM000481

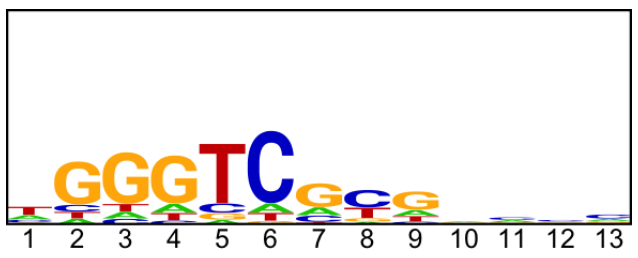

Location of top GRECO-BIT/MEX PWM in MAGIX peaks (up to the top 10,000)

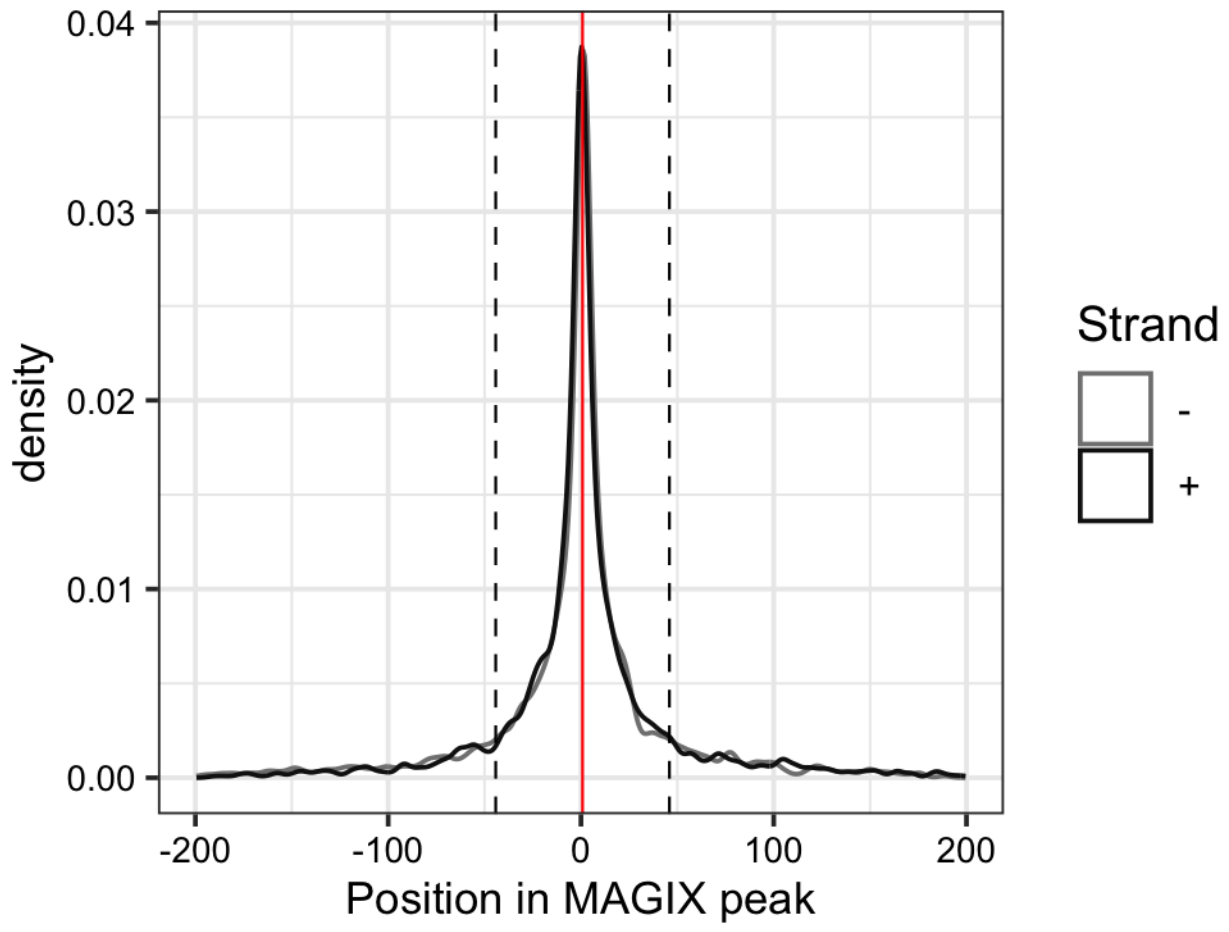

AUROC of top GRECO-BIT/MEX PWM in sliding windows (500 peaks, 50 peak step) along the MAGIX peaks (up to the top 75,000) ranked by the MAGIX enrichment coefficient and fraction of overlapping ChIP peaks in the same window

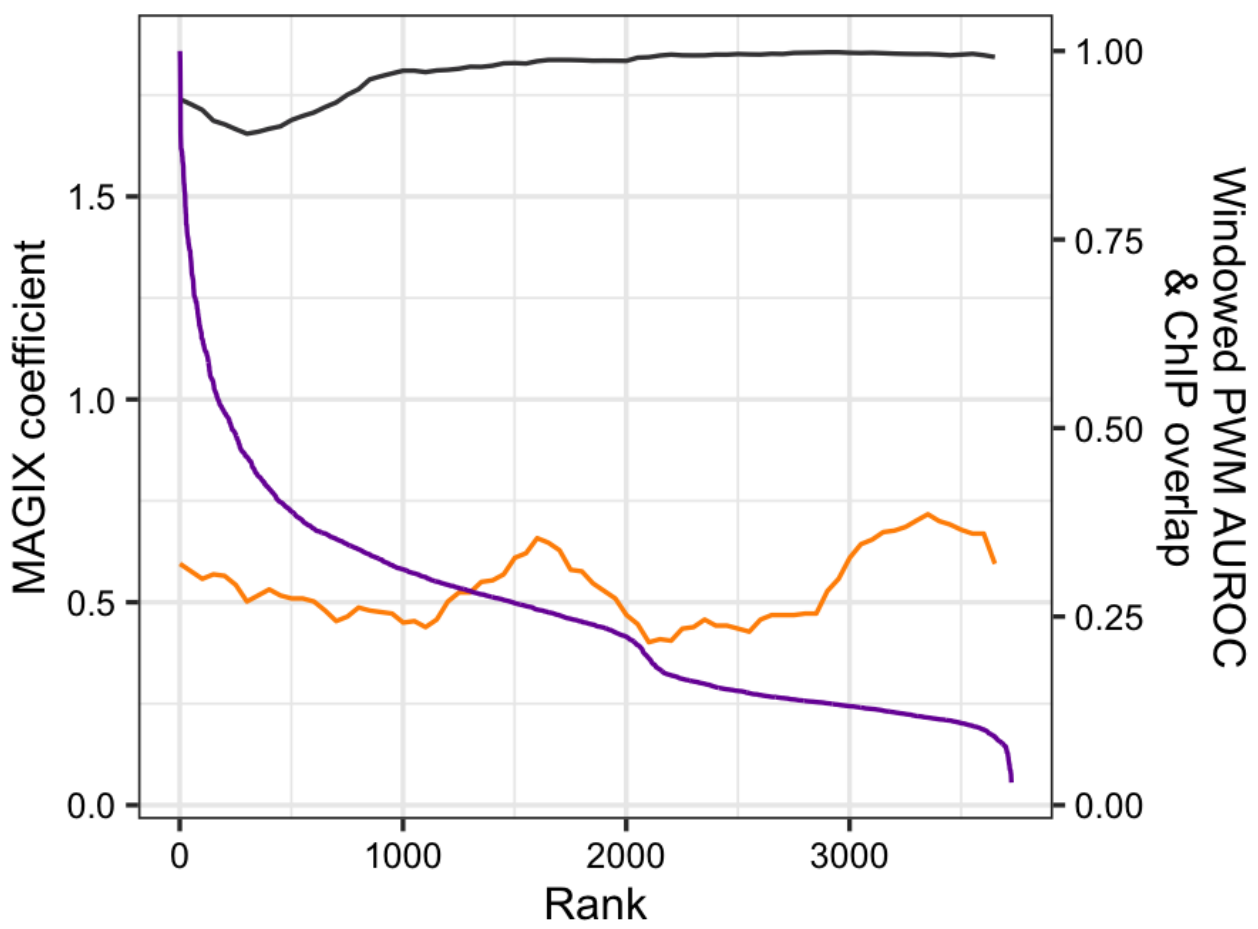

FBXL19

Top GRECO-BIT/MEX PWM: PWM000507

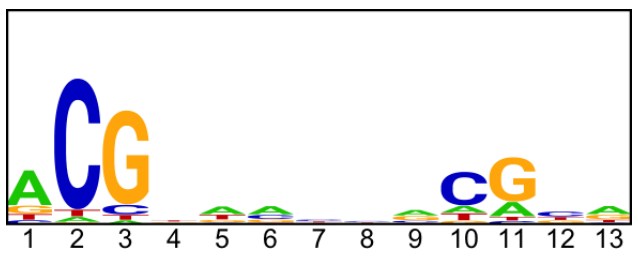

Location of top GRECO-BIT/MEX PWM in MAGIX peaks (up to the top 10,000)

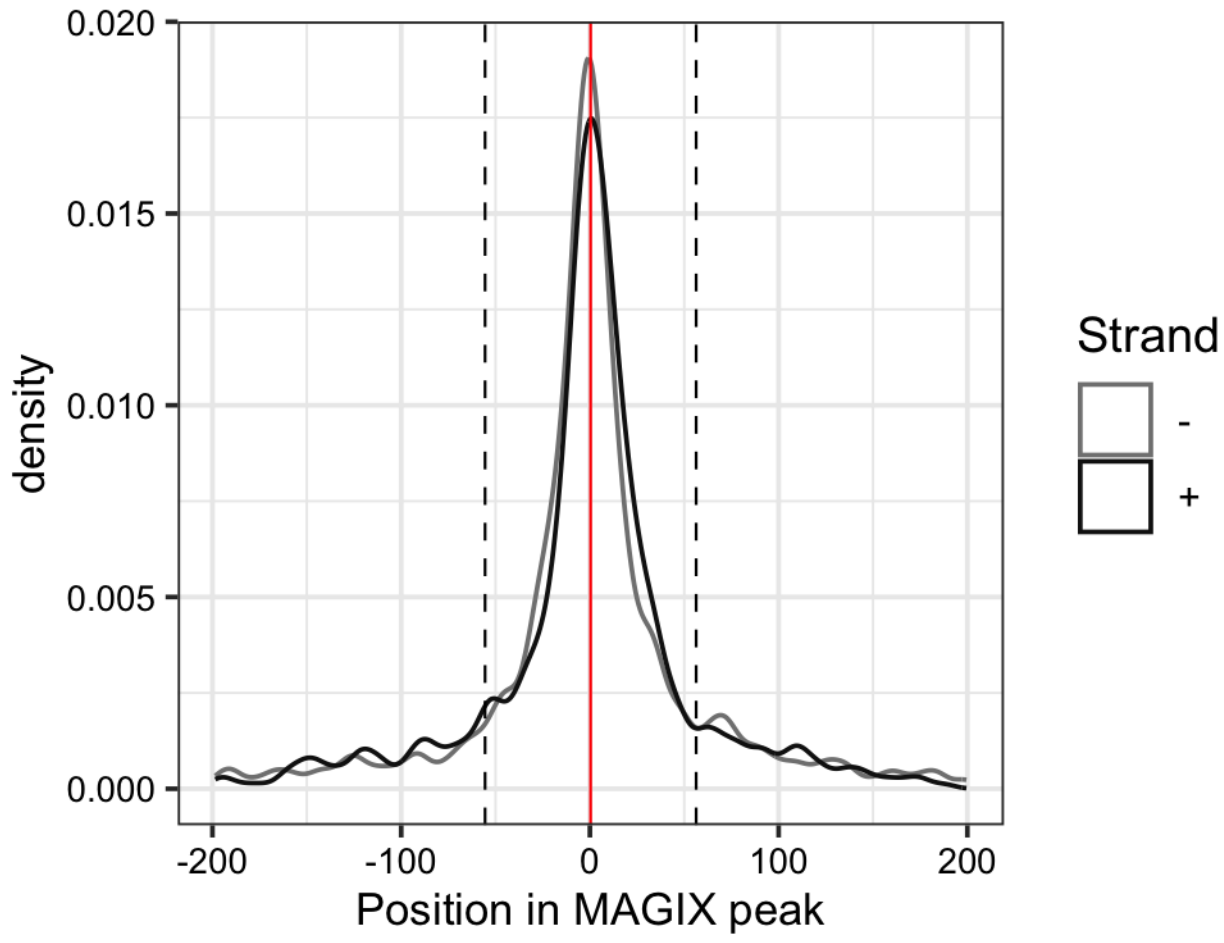

AUROC of top GRECO-BIT/MEX PWM in sliding windows (500 peaks, 50 peak step) along the MAGIX peaks (up to the top 75,000) ranked by the MAGIX enrichment coefficient and fraction of overlapping ChIP peaks in the same window

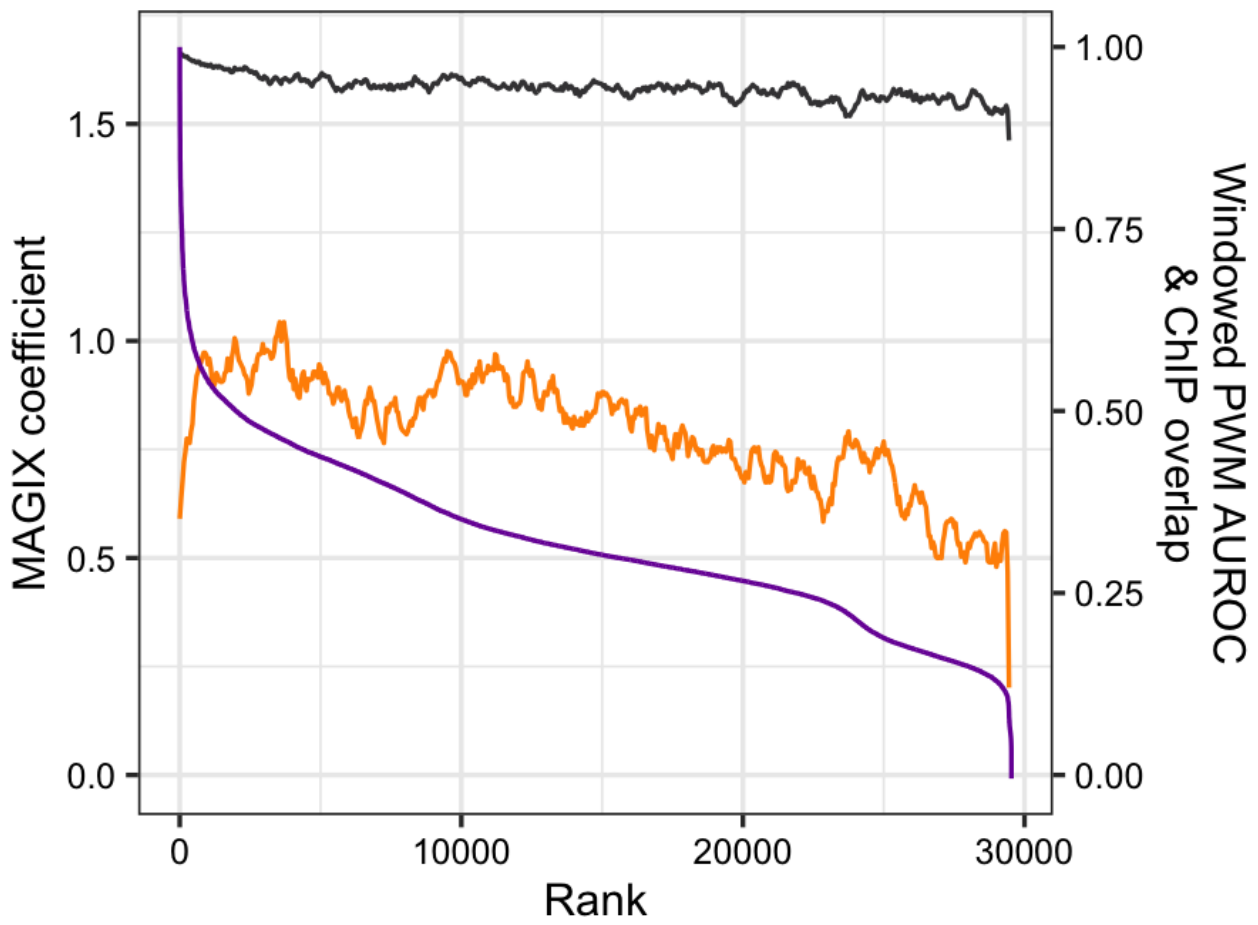

FIZ1

Top GRECO-BIT/MEX PWM: PWM000541

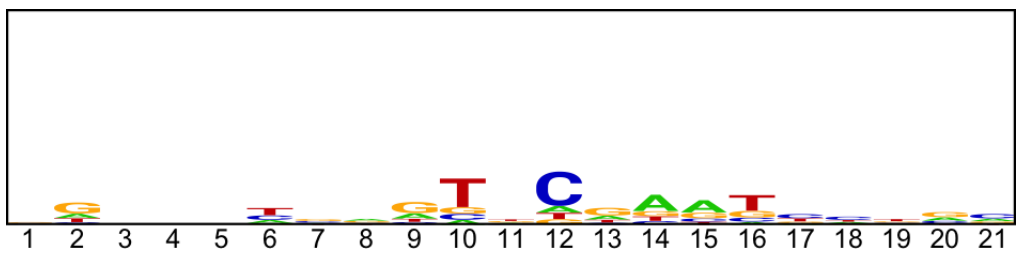

Location of top GRECO-BIT/MEX PWM in MAGIX peaks (up to the top 10,000)

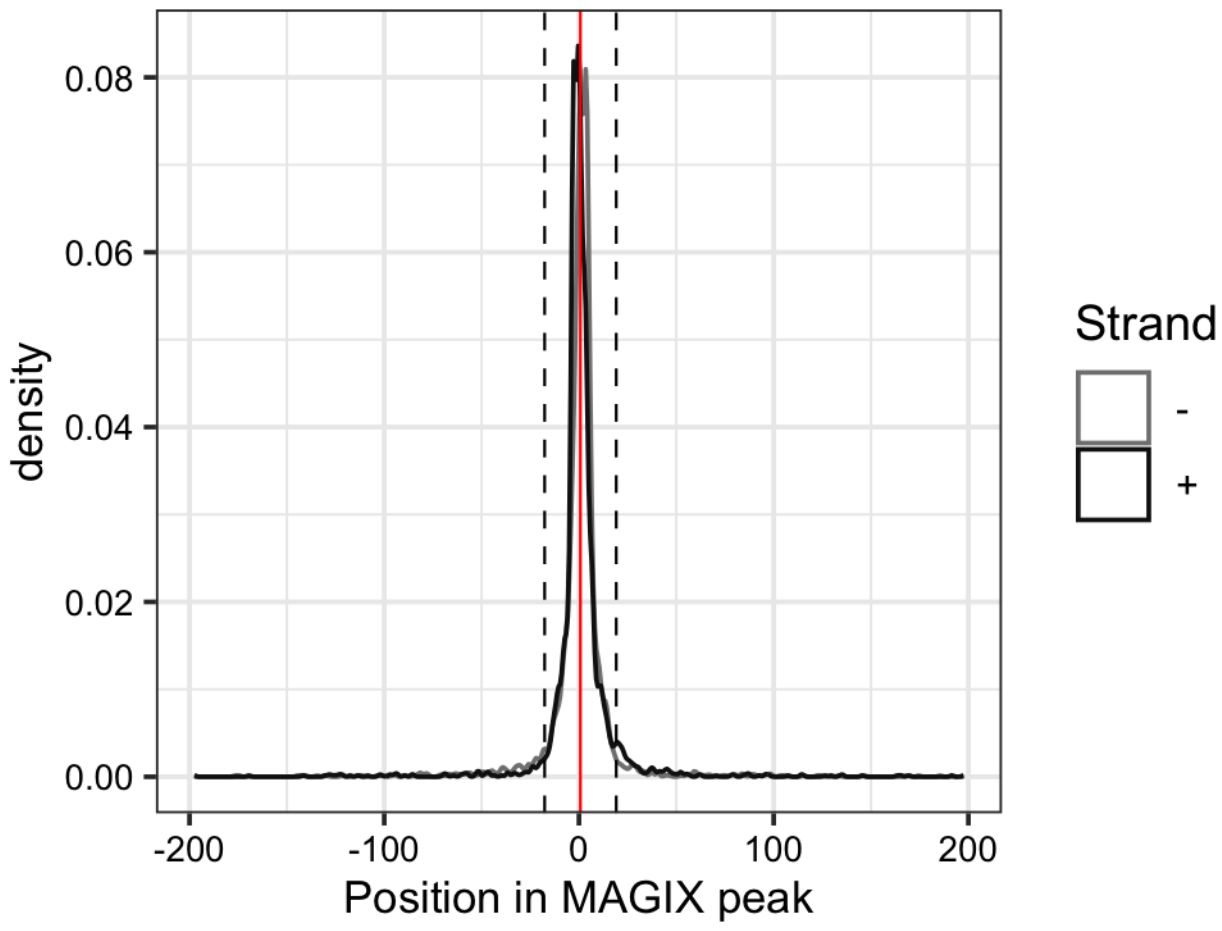

AUROC of top GRECO-BIT/MEX PWM in sliding windows (500 peaks, 50 peak step) along the MAGIX peaks (up to the top 75,000) ranked by the MAGIX enrichment coefficient and fraction of overlapping ChIP peaks in the same window

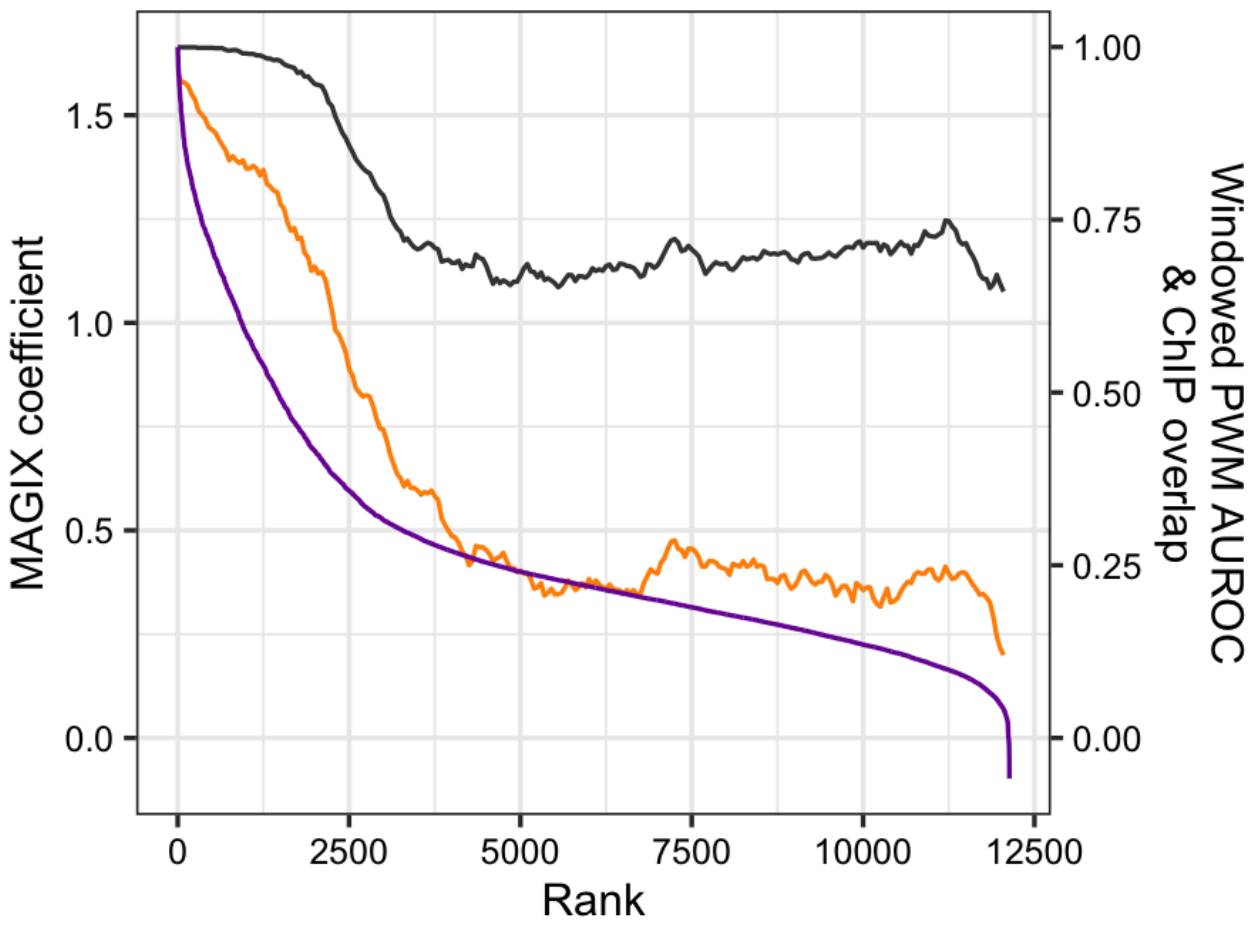

FLI1

Top GRECO-BIT/MEX PWM: PWM000587

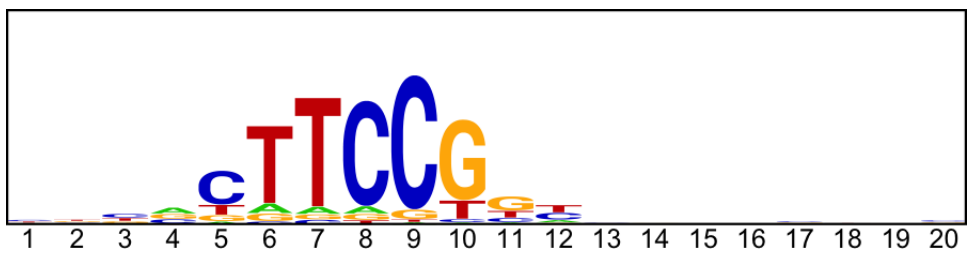

Location of top GRECO-BIT/MEX PWM in MAGIX peaks (up to the top 10,000)

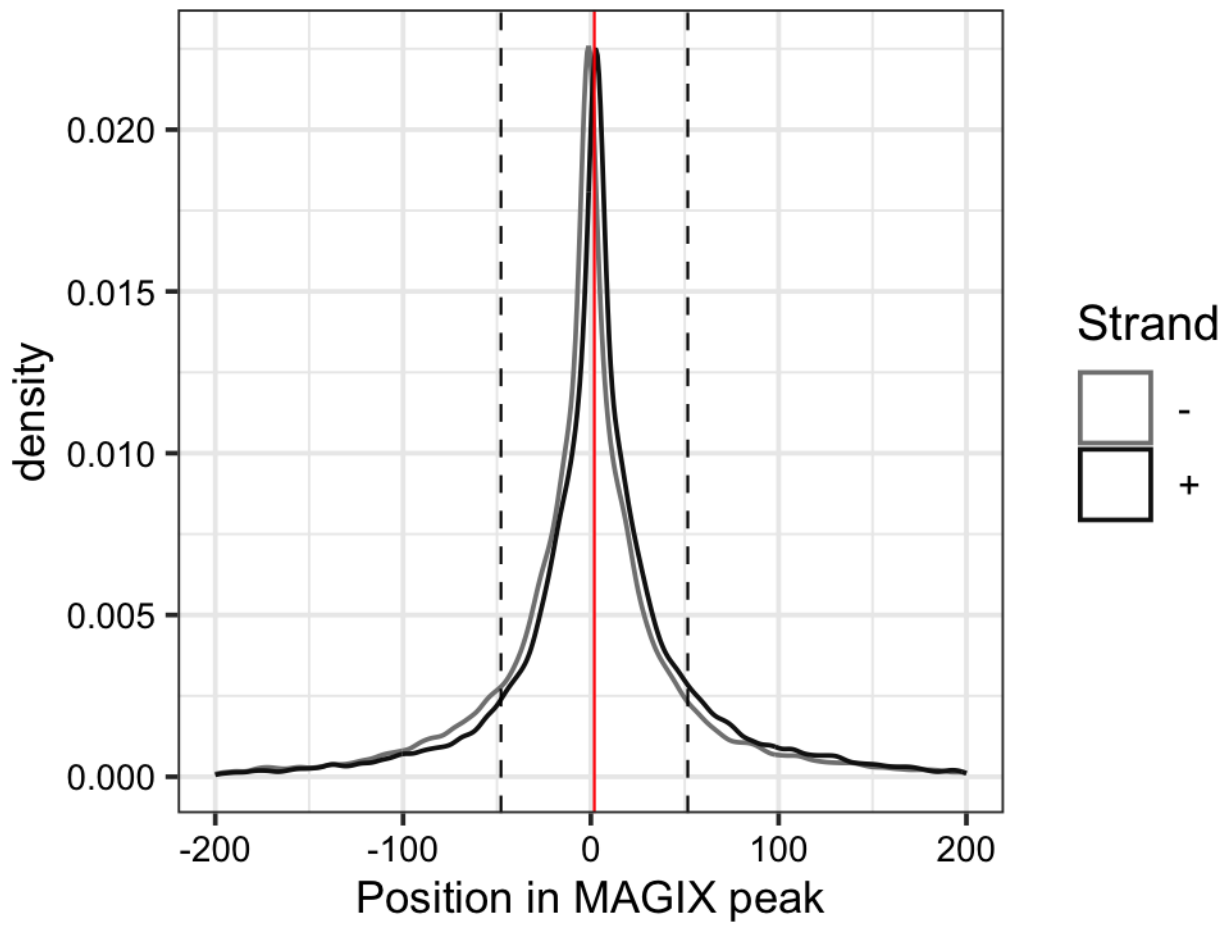

AUROC of top GRECO-BIT/MEX PWM in sliding windows (500 peaks, 50 peak step) along the MAGIX peaks (up to the top 75,000) ranked by the MAGIX enrichment coefficient and fraction of overlapping ChIP peaks in the same window

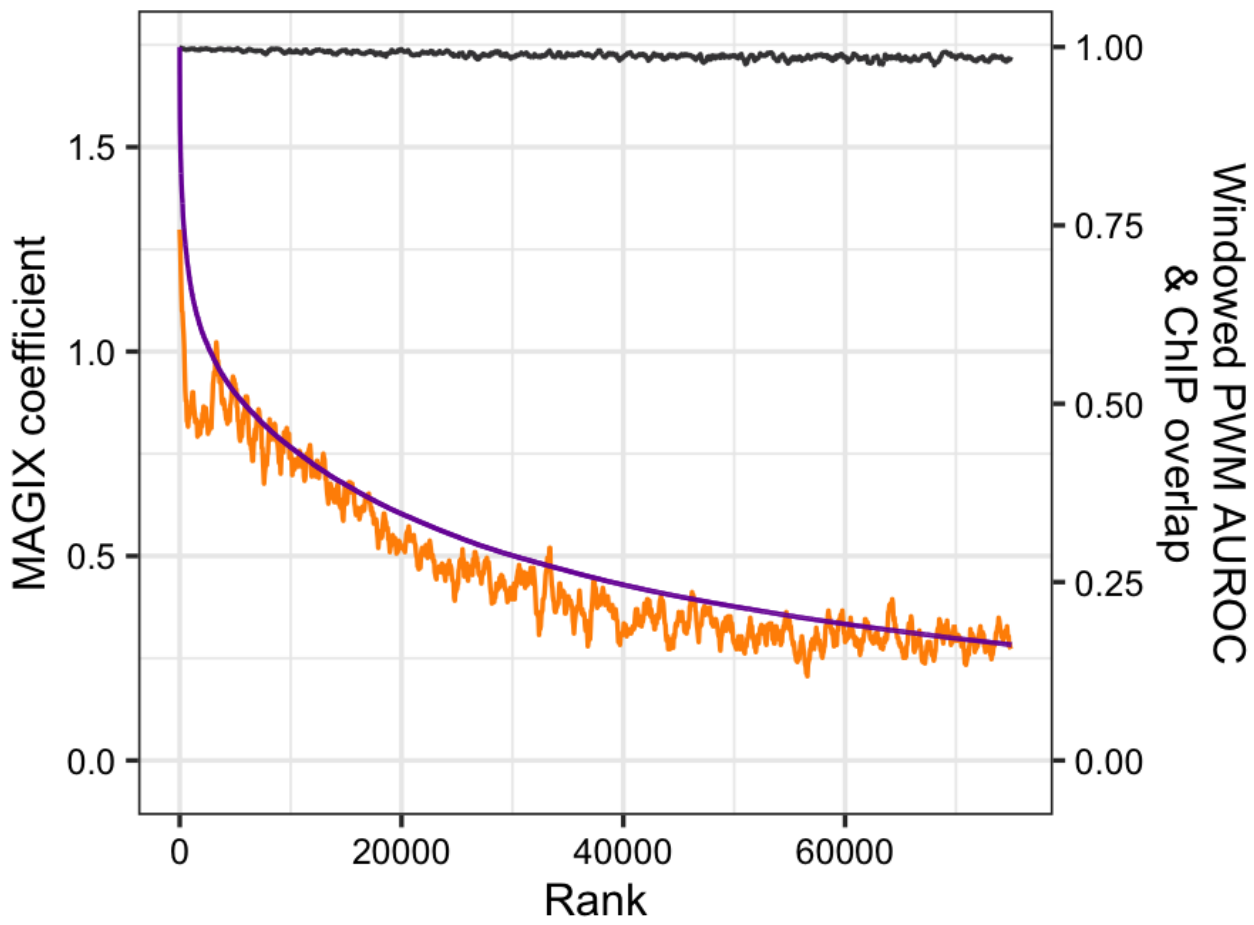

FLYWCH1

Top GRECO-BIT/MEX PWM: PWM000607

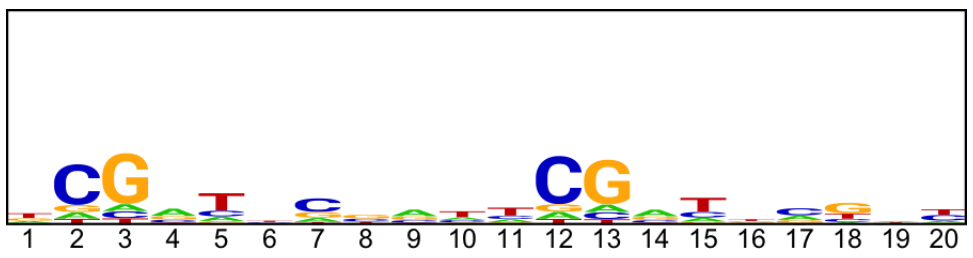

Location of top GRECO-BIT/MEX PWM in MAGIX peaks (up to the top 10,000)

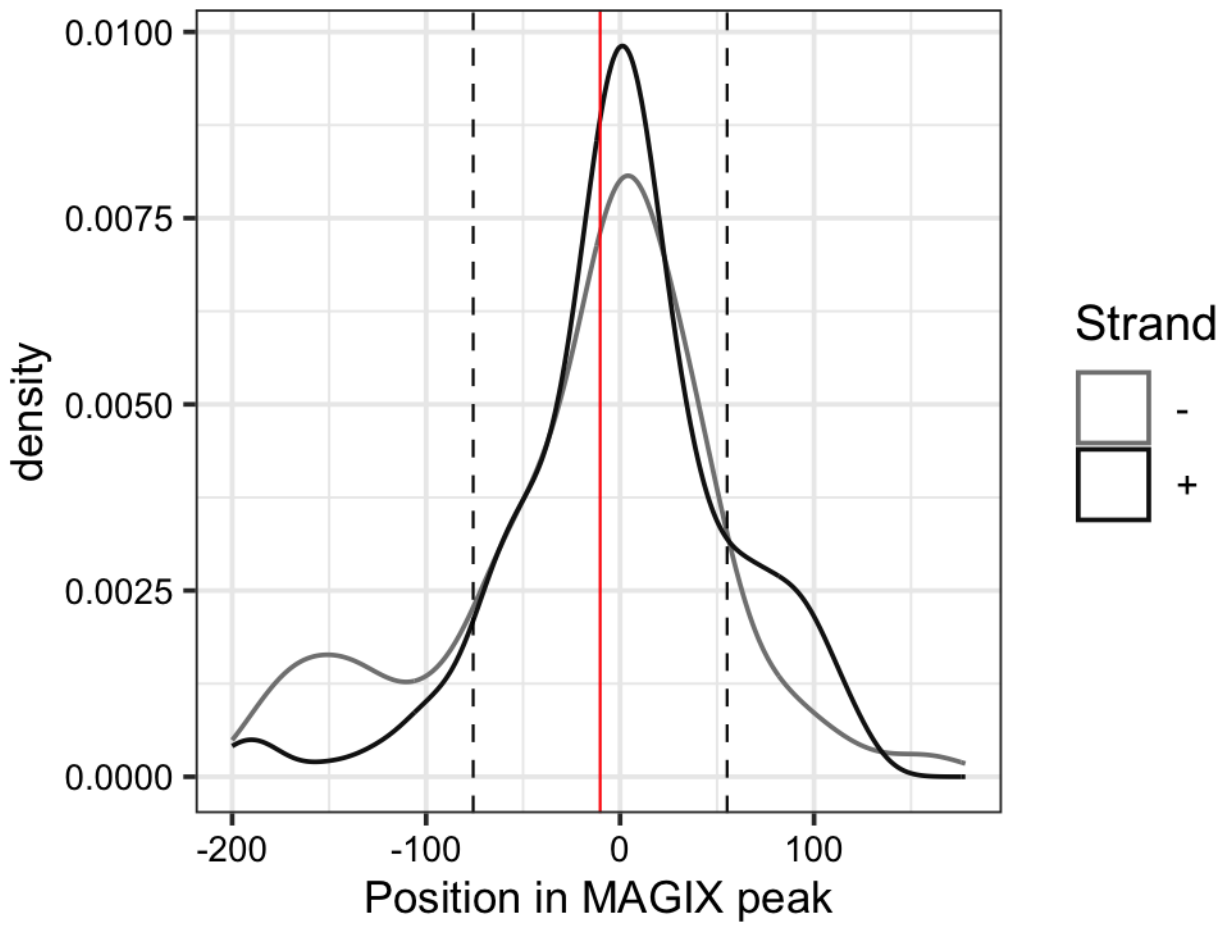

AUROC of top GRECO-BIT/MEX PWM in sliding windows (500 peaks, 50 peak step) along the MAGIX peaks (up to the top 75,000) ranked by the MAGIX enrichment coefficient and fraction of overlapping ChIP peaks in the same window

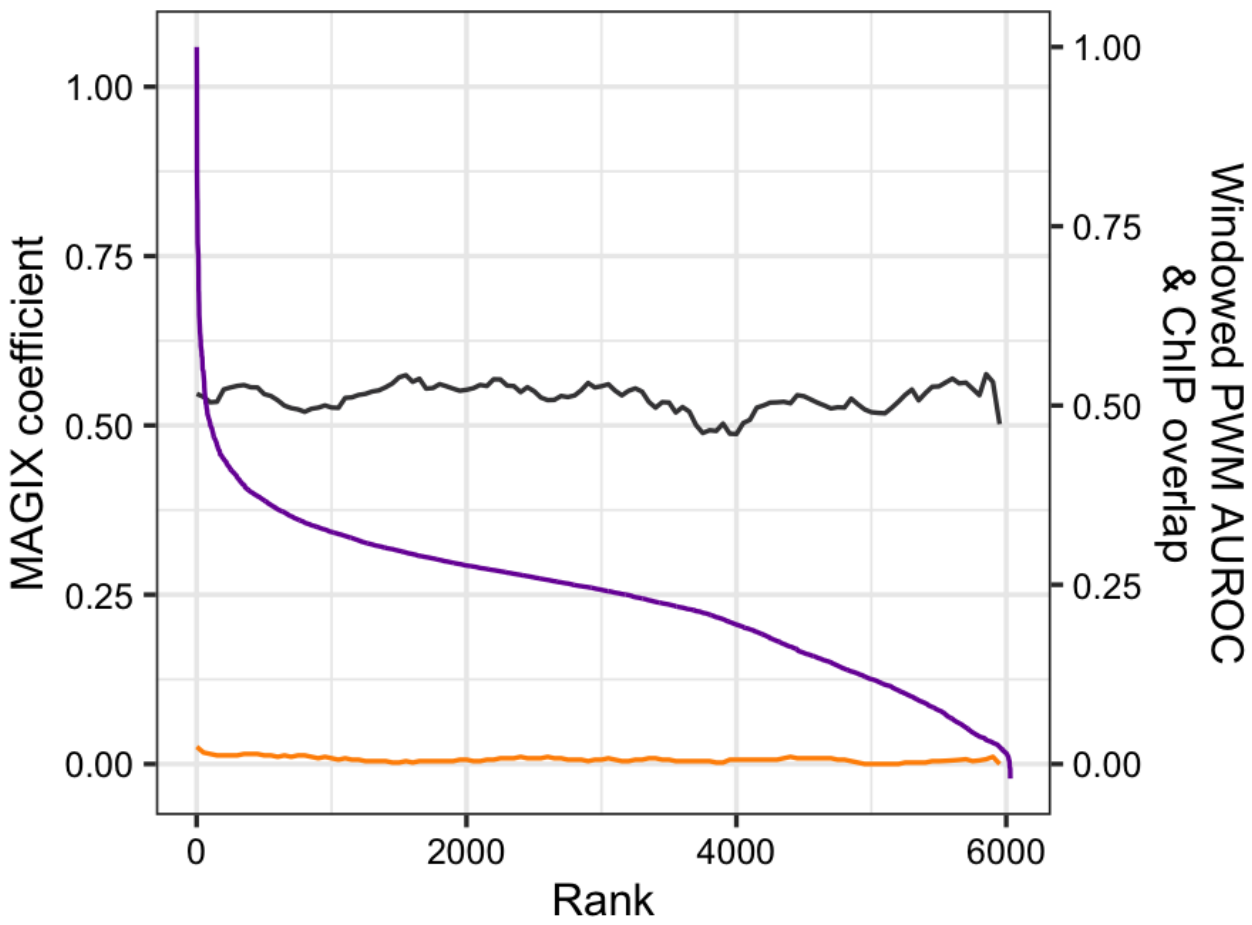

FOSL2

Top GRECO-BIT/MEX PWM: PWM000649

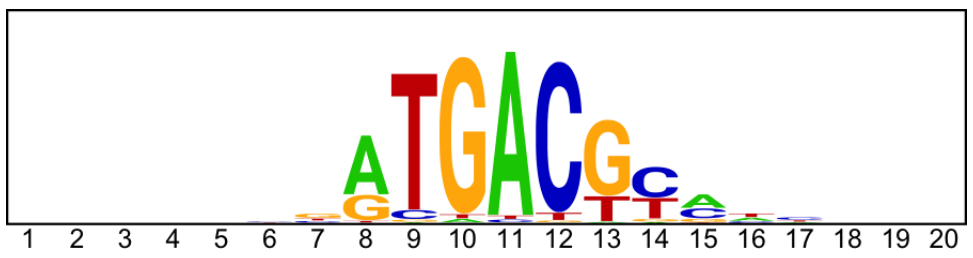

Location of top GRECO-BIT/MEX PWM in MAGIX peaks (up to the top 10,000)

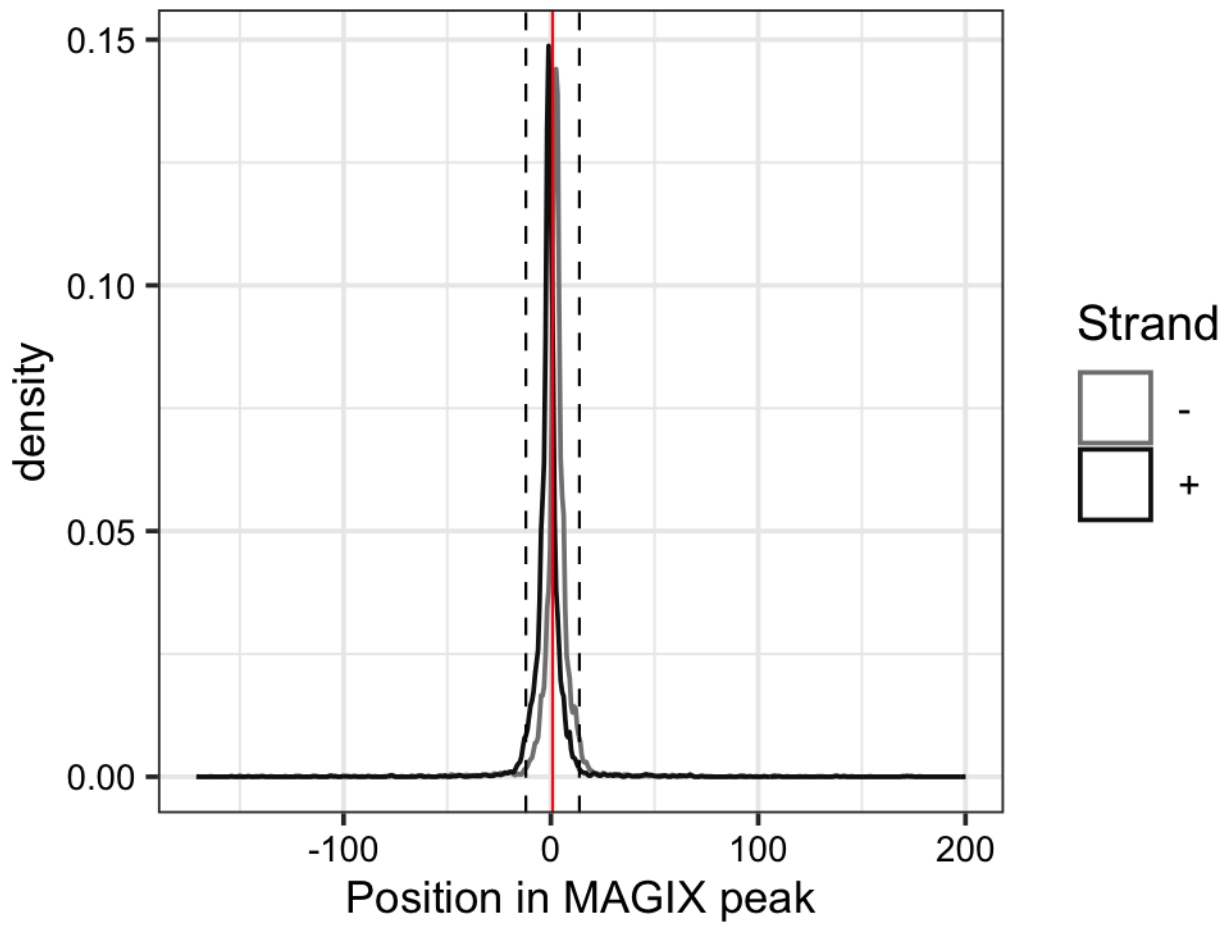

AUROC of top GRECO-BIT/MEX PWM in sliding windows (500 peaks, 50 peak step) along the MAGIX peaks (up to the top 75,000) ranked by the MAGIX enrichment coefficient and fraction of overlapping ChIP peaks in the same window

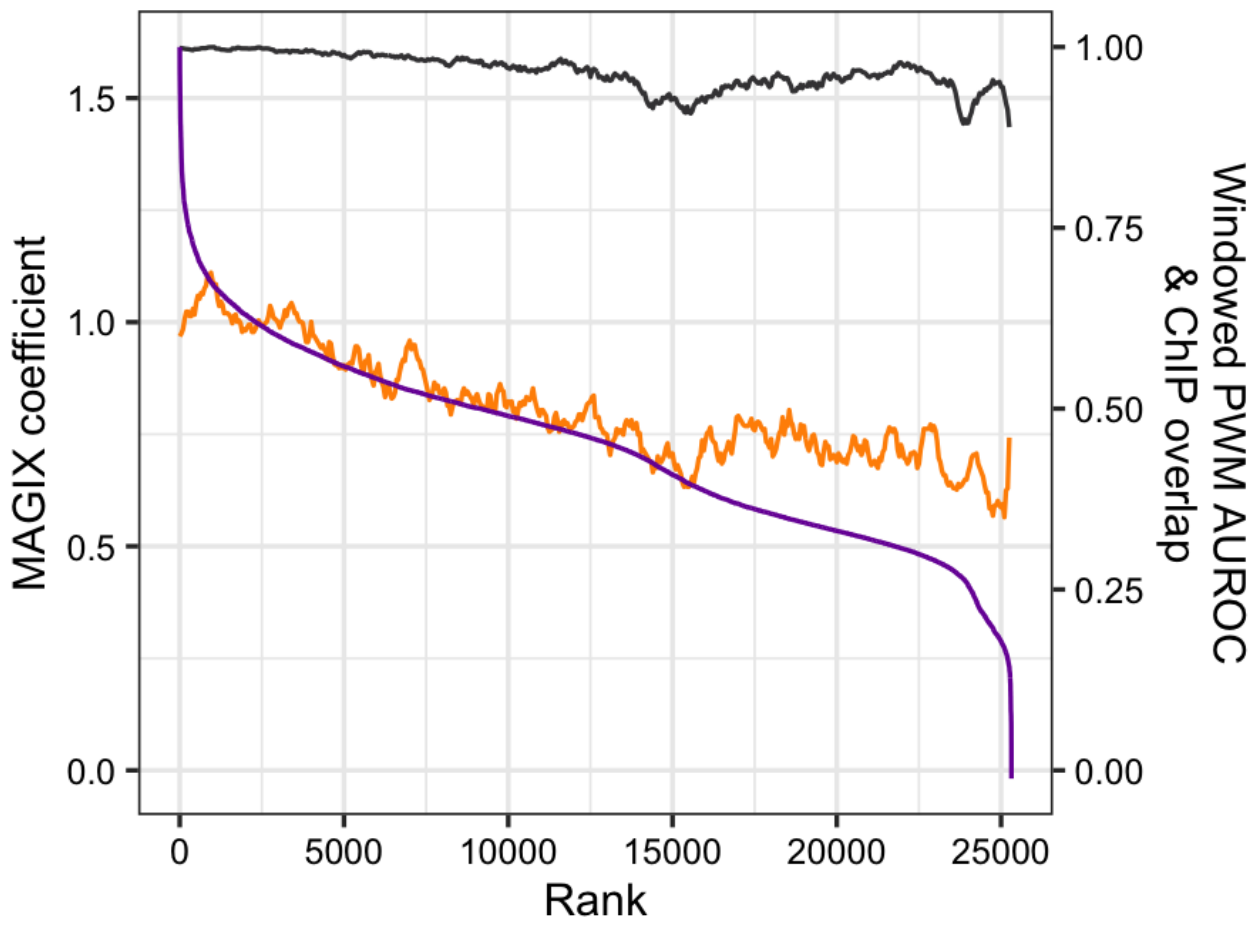

GABPA

Top GRECO-BIT/MEX PWM: PWM000687

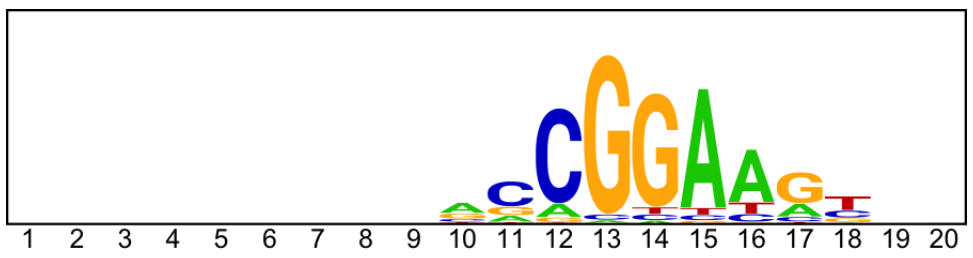

Location of top GRECO-BIT/MEX PWM in MAGIX peaks (up to the top 10,000)

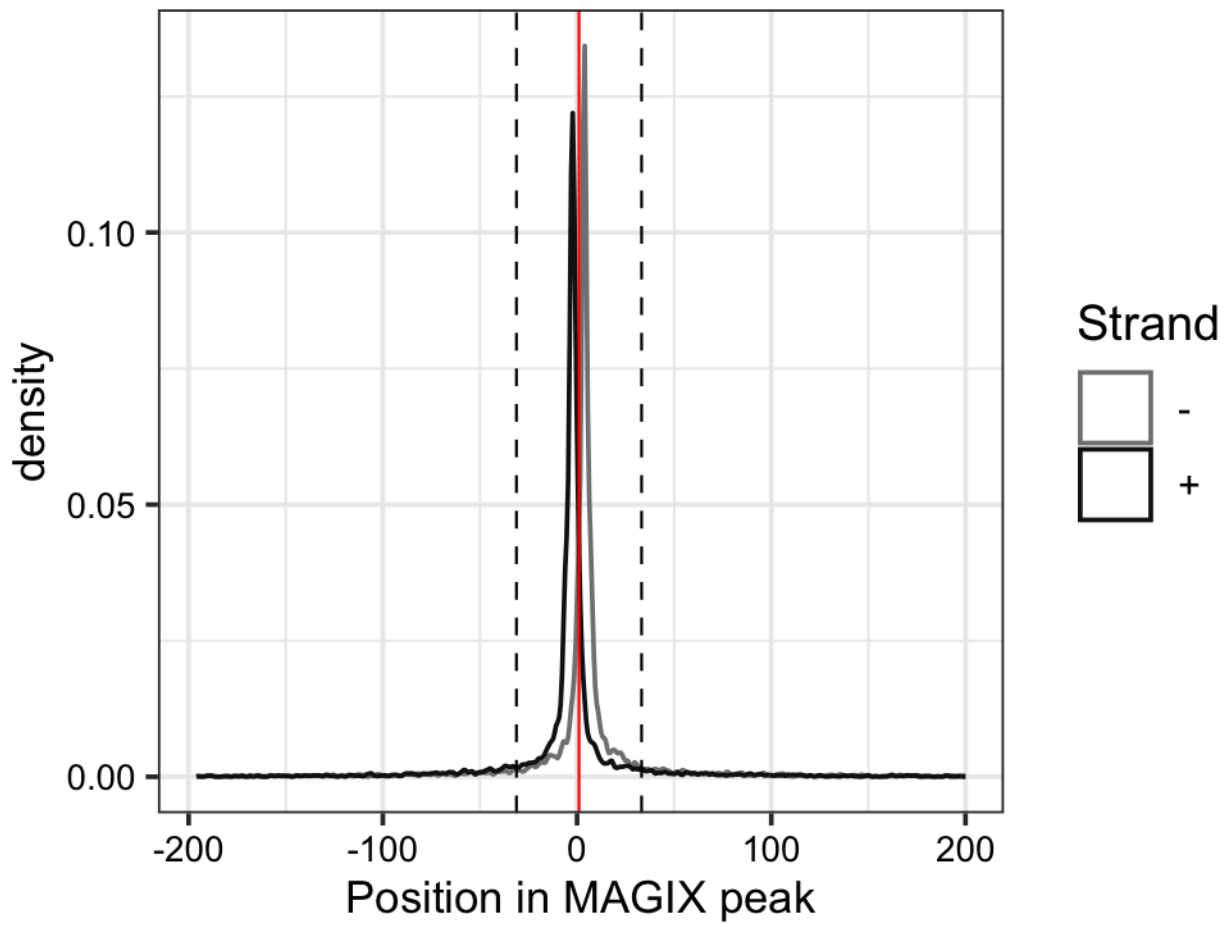

AUROC of top GRECO-BIT/MEX PWM in sliding windows (500 peaks, 50 peak step) along the MAGIX peaks (up to the top 75,000) ranked by the MAGIX enrichment coefficient and fraction of overlapping ChIP peaks in the same window

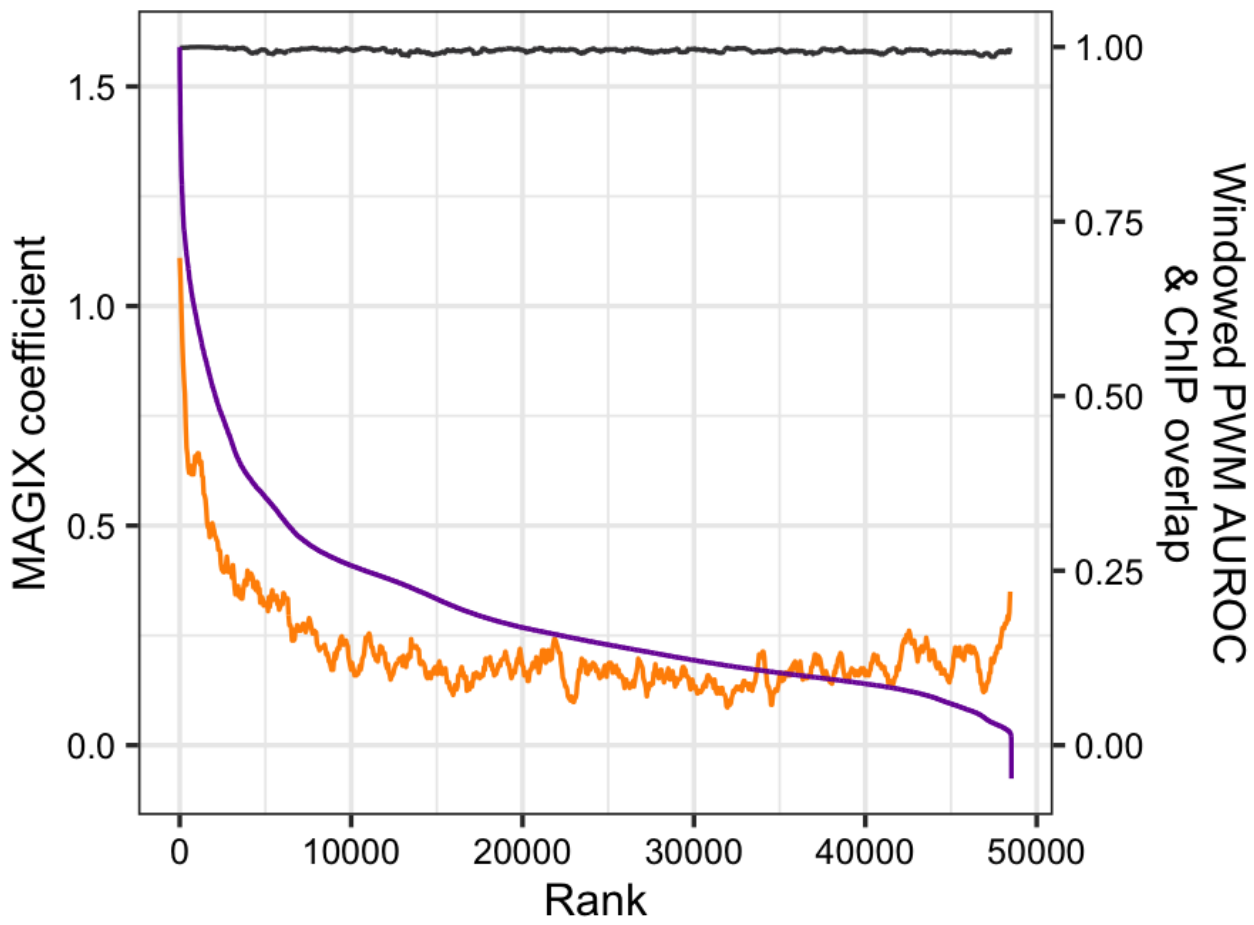

GATAD2A

Top GRECO-BIT/MEX PWM: PWM000707

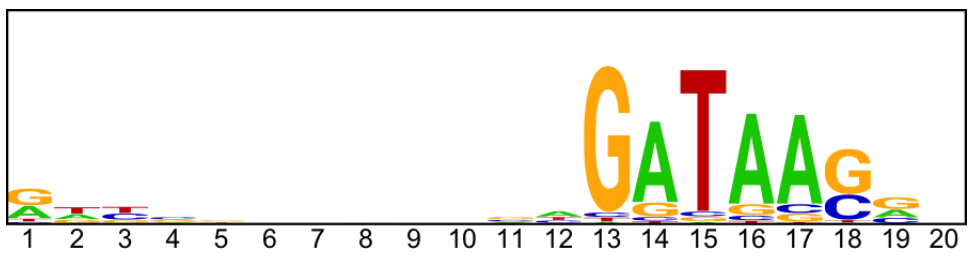

Location of top GRECO-BIT/MEX PWM in MAGIX peaks (up to the top 10,000)

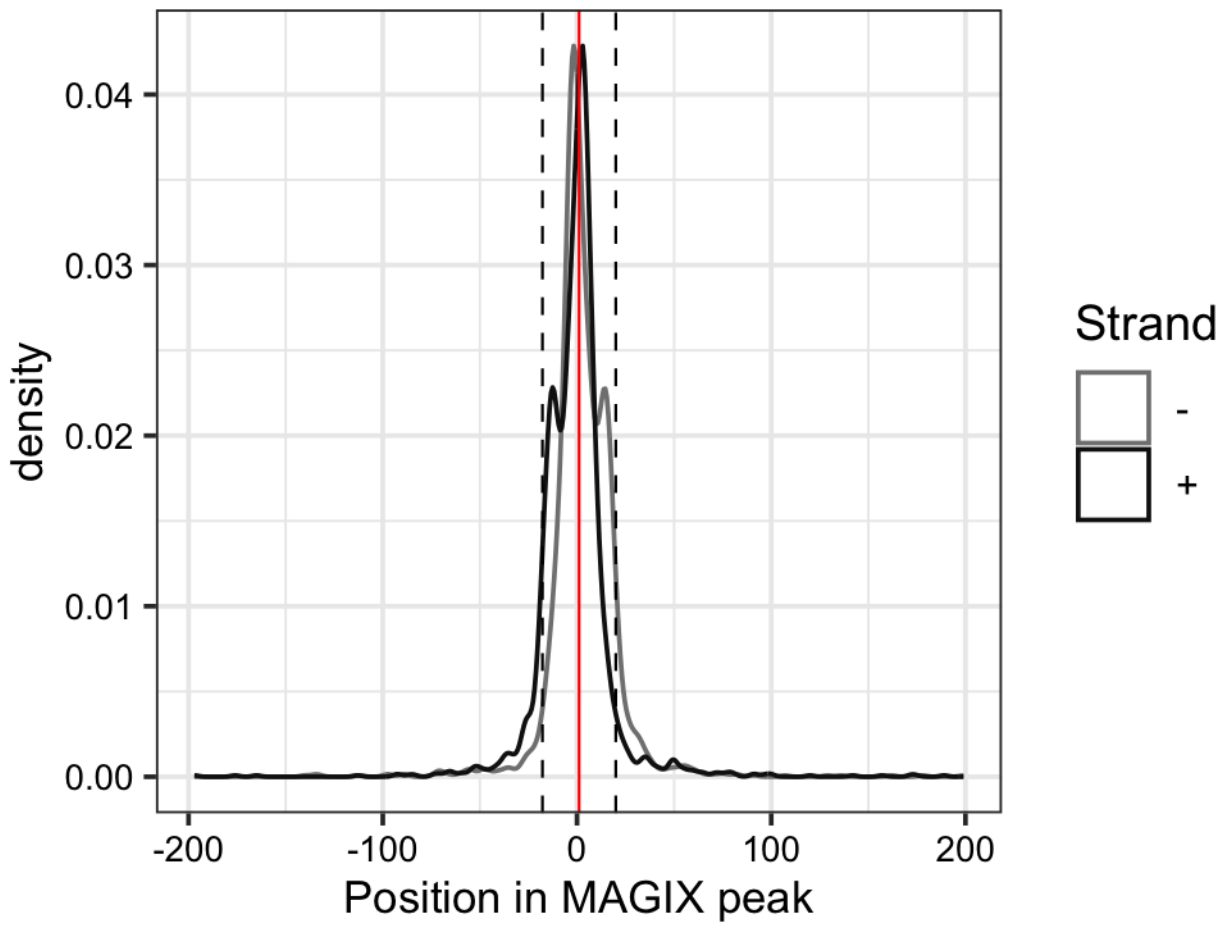

GCM1

Top GRECO-BIT/MEX PWM: PWM000755

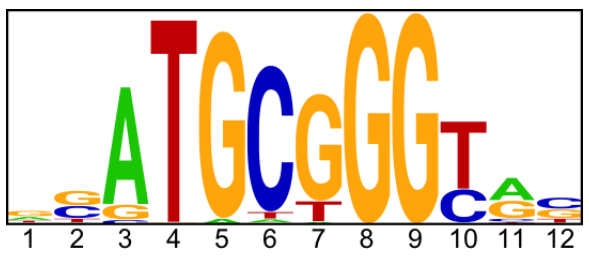

Location of top GRECO-BIT/MEX PWM in MAGIX peaks (up to the top 10,000)

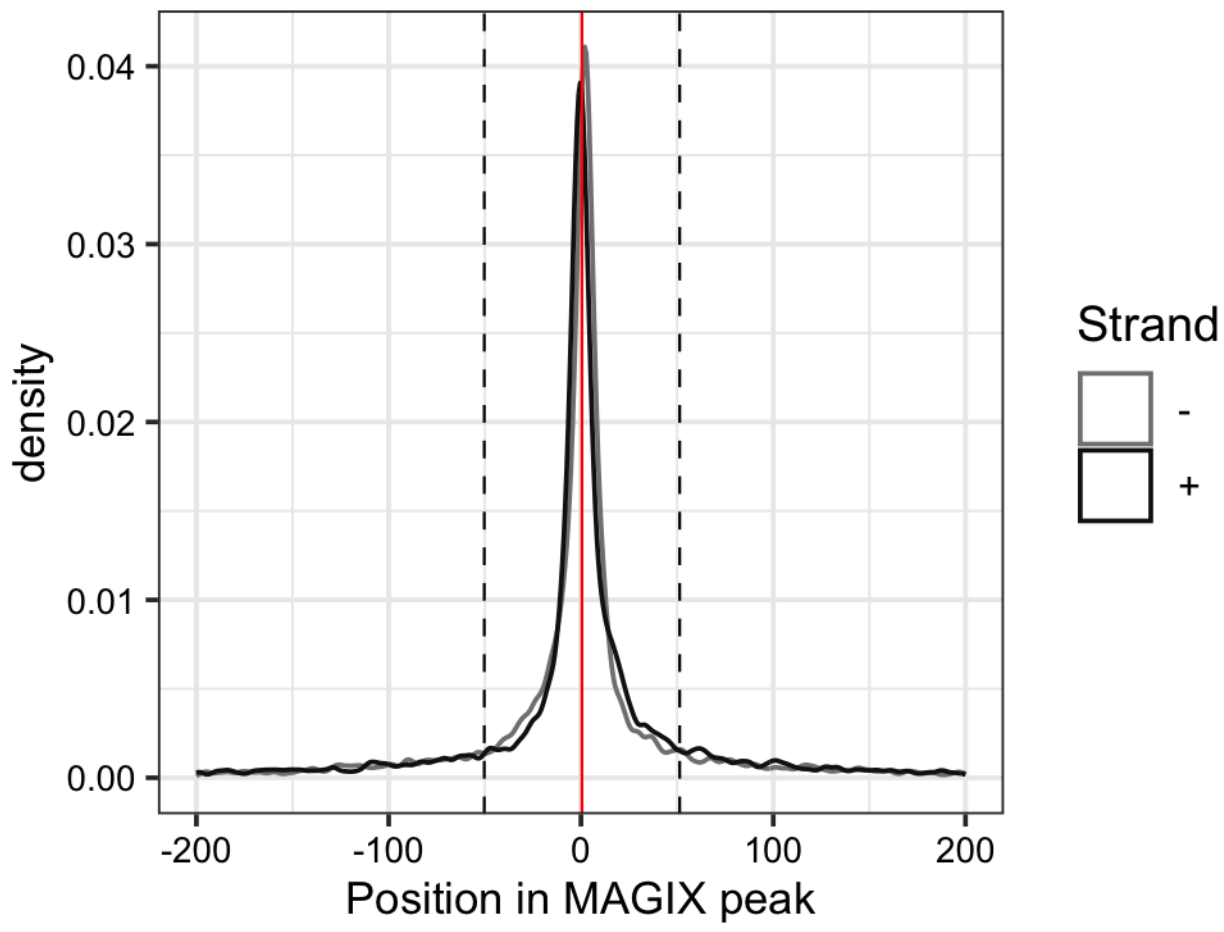

AUROC of top GRECO-BIT/MEX PWM in sliding windows (500 peaks, 50 peak step) along the MAGIX peaks (up to the top 75,000) ranked by the MAGIX enrichment coefficient and fraction of overlapping ChIP peaks in the same window

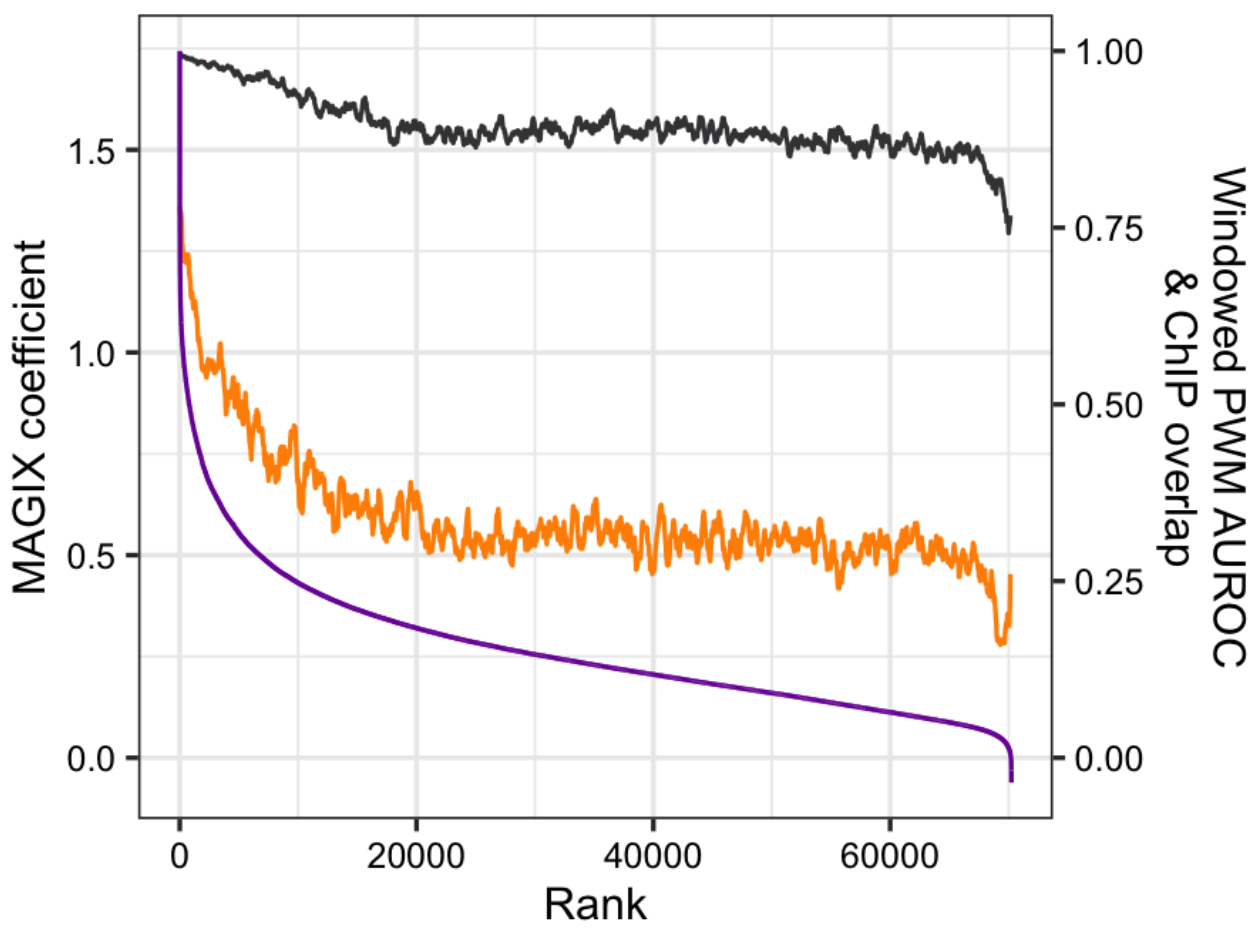

GLI4

Top GRECO-BIT/MEX PWM: PWM000773

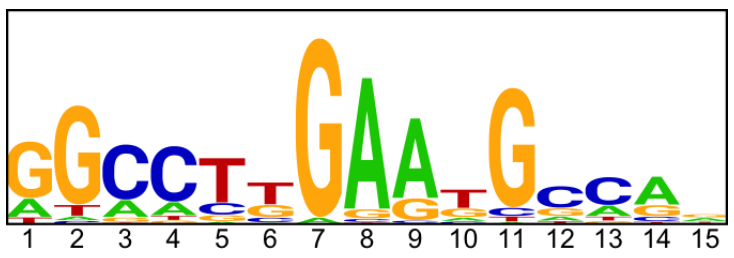

Location of top GRECO-BIT/MEX PWM in MAGIX peaks (up to the top 10,000)

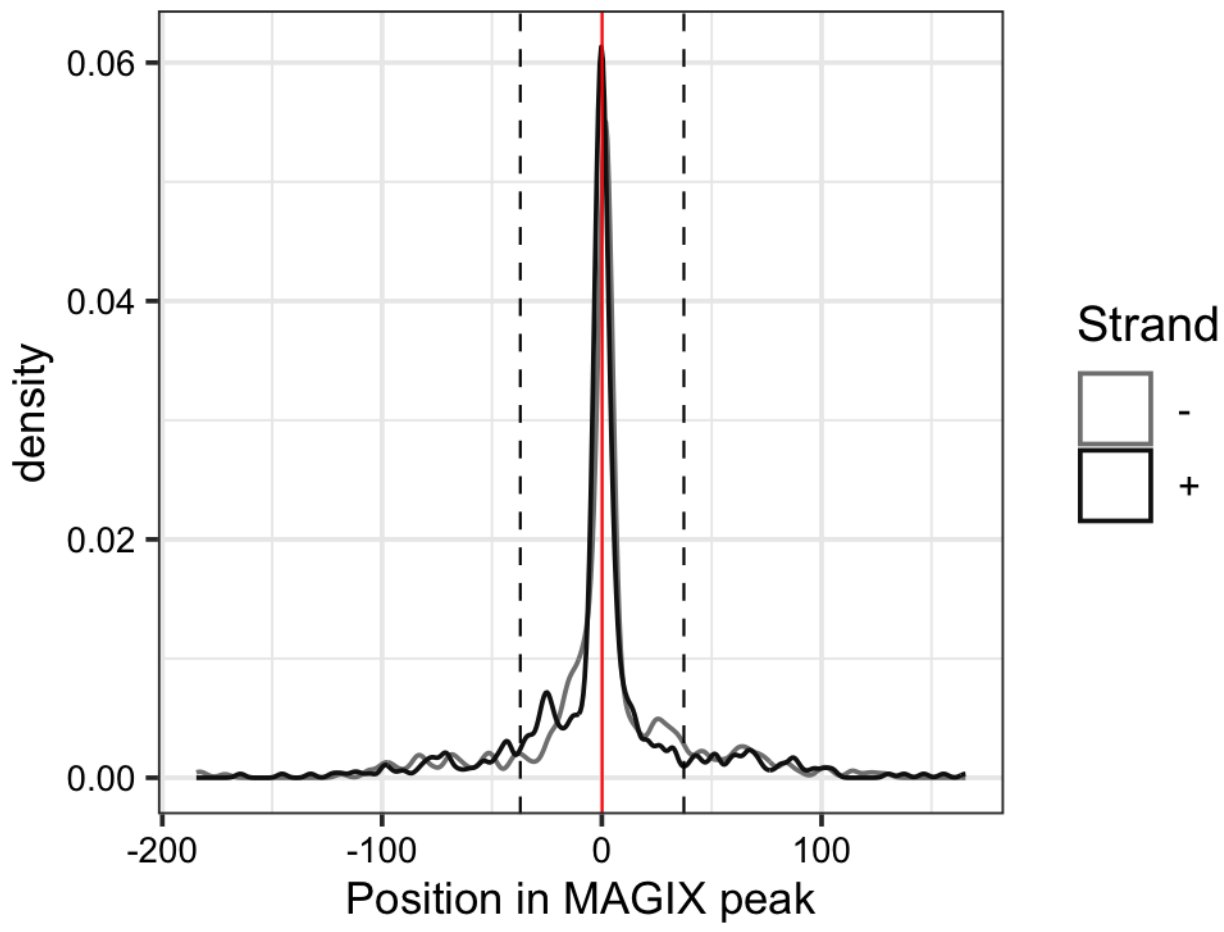

AUROC of top GRECO-BIT/MEX PWM in sliding windows (500 peaks, 50 peak step) along the MAGIX peaks (up to the top 75,000) ranked by the MAGIX enrichment coefficient and fraction of overlapping ChIP peaks in the same window

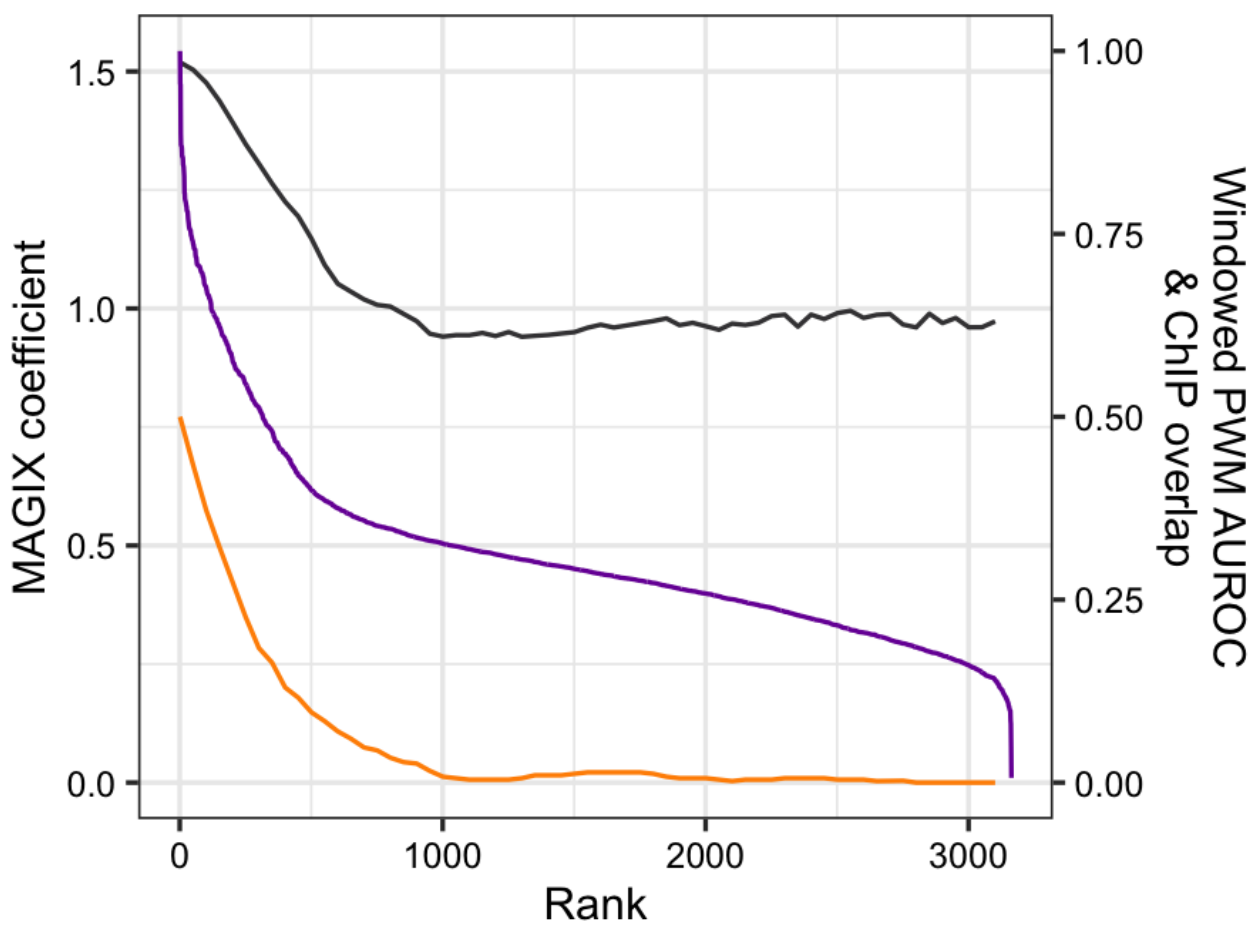

JRK

Top GRECO-BIT/MEX PWM: PWM000822

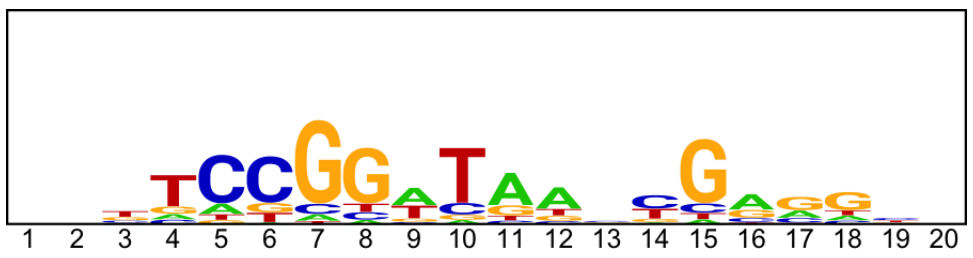

Location of top GRECO-BIT/MEX PWM in MAGIX peaks (up to the top 10,000)

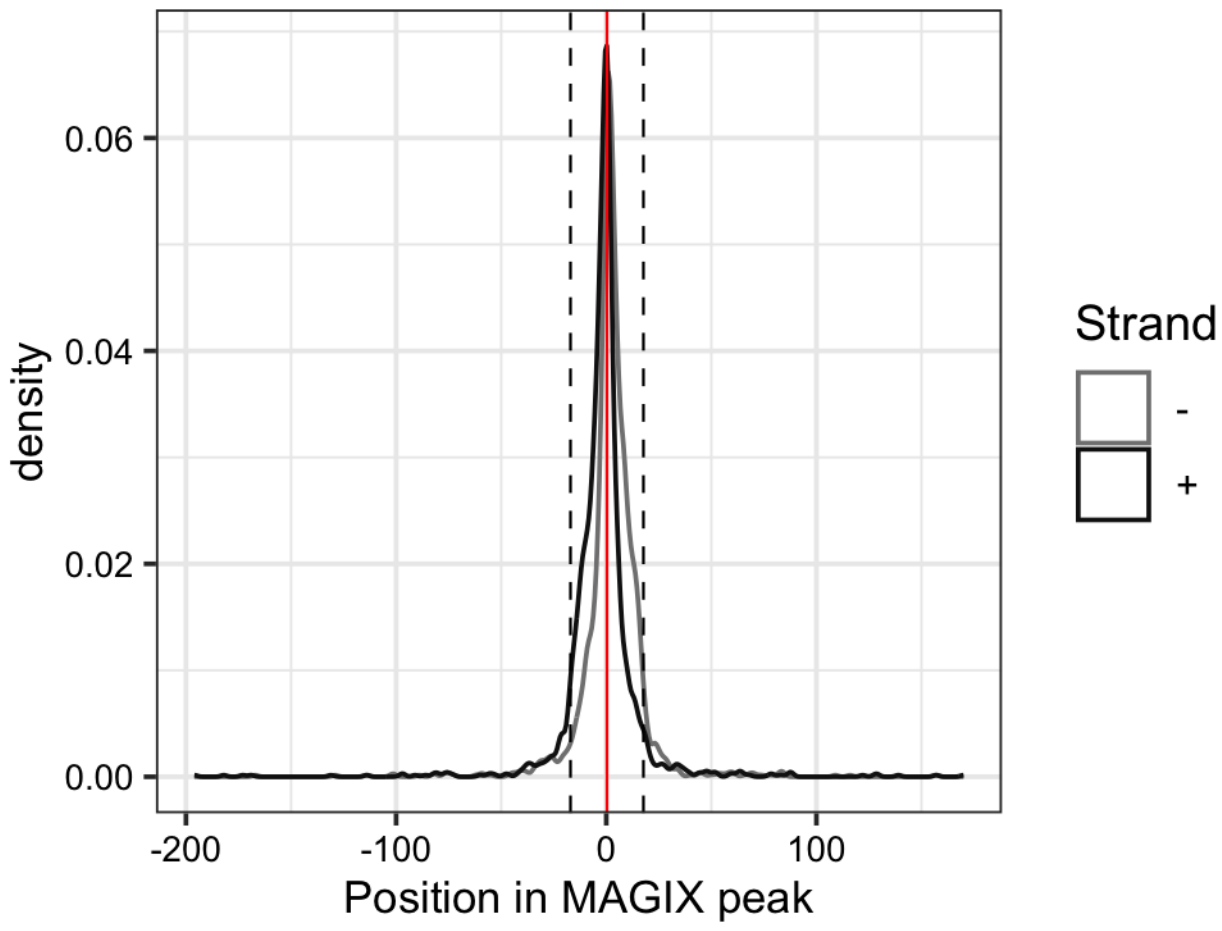

AUROC of top GRECO-BIT/MEX PWM in sliding windows (500 peaks, 50 peak step) along the MAGIX peaks (up to the top 75,000) ranked by the MAGIX enrichment coefficient and fraction of overlapping ChIP peaks in the same window

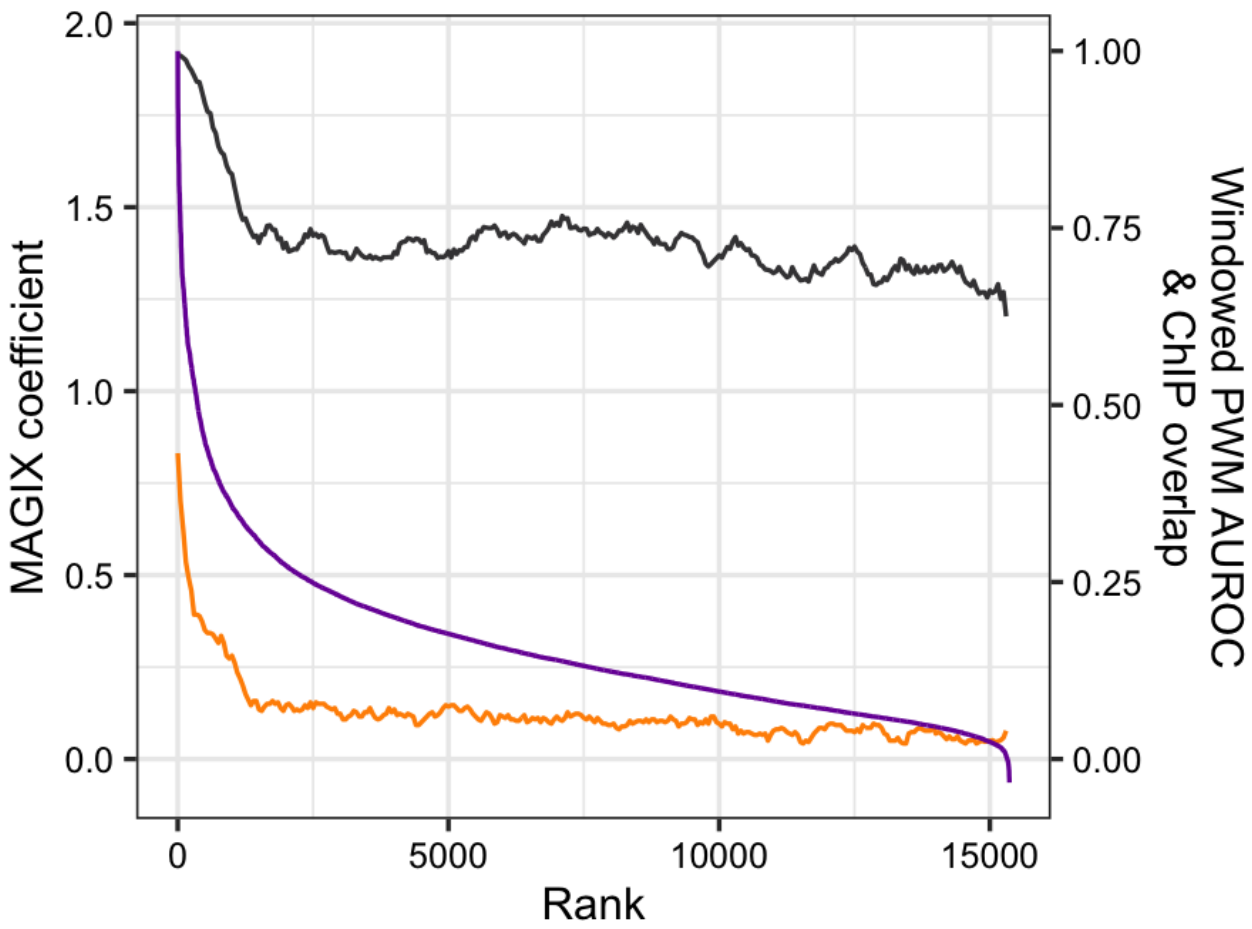

KDM2A

Top GRECO-BIT/MEX PWM: PWM000893

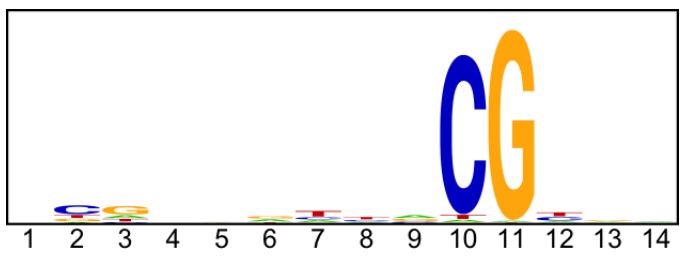

Location of top GRECO-BIT/MEX PWM in MAGIX peaks (up to the top 10,000)

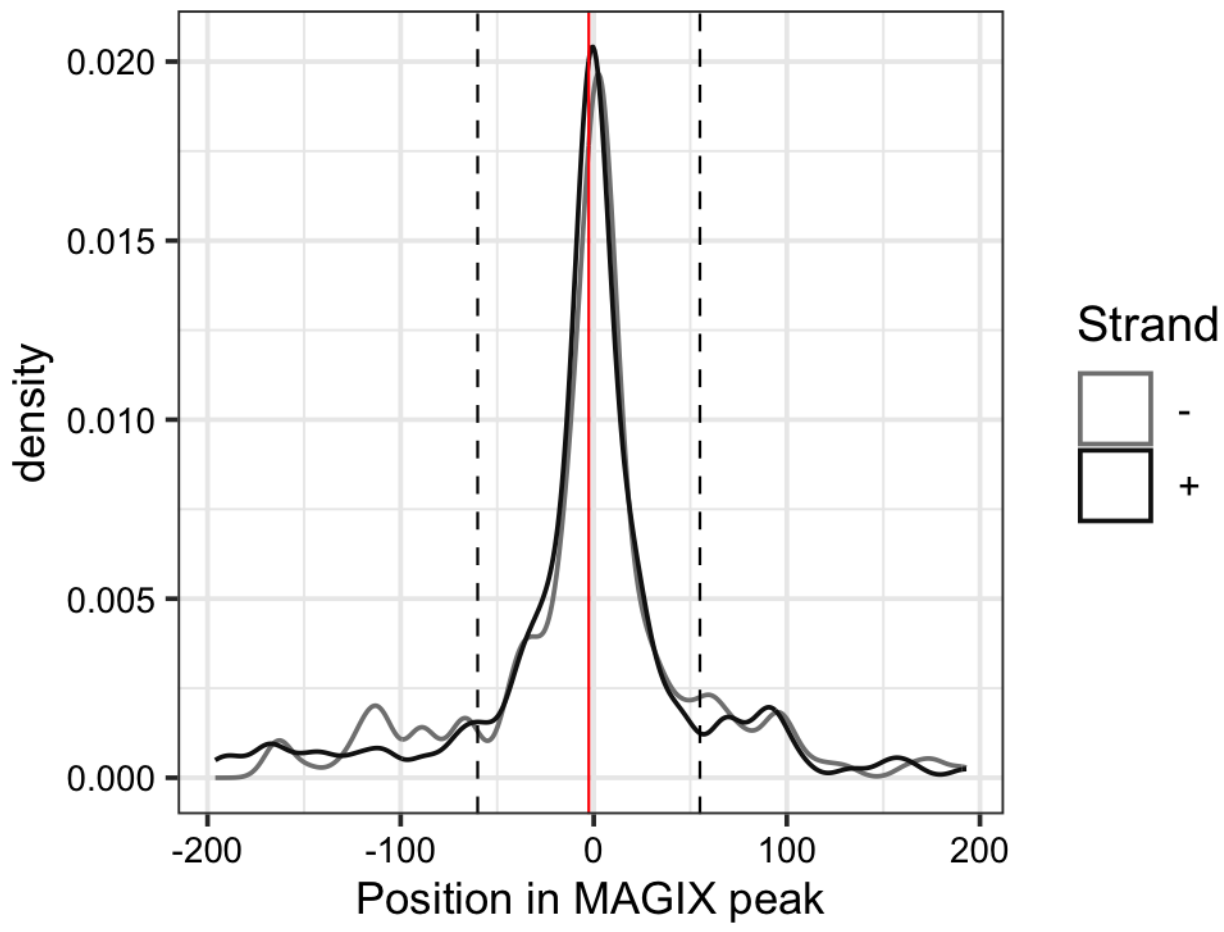

AUROC of top GRECO-BIT/MEX PWM in sliding windows (500 peaks, 50 peak step) along the MAGIX peaks (up to the top 75,000) ranked by the MAGIX enrichment coefficient and fraction of overlapping ChIP peaks in the same window

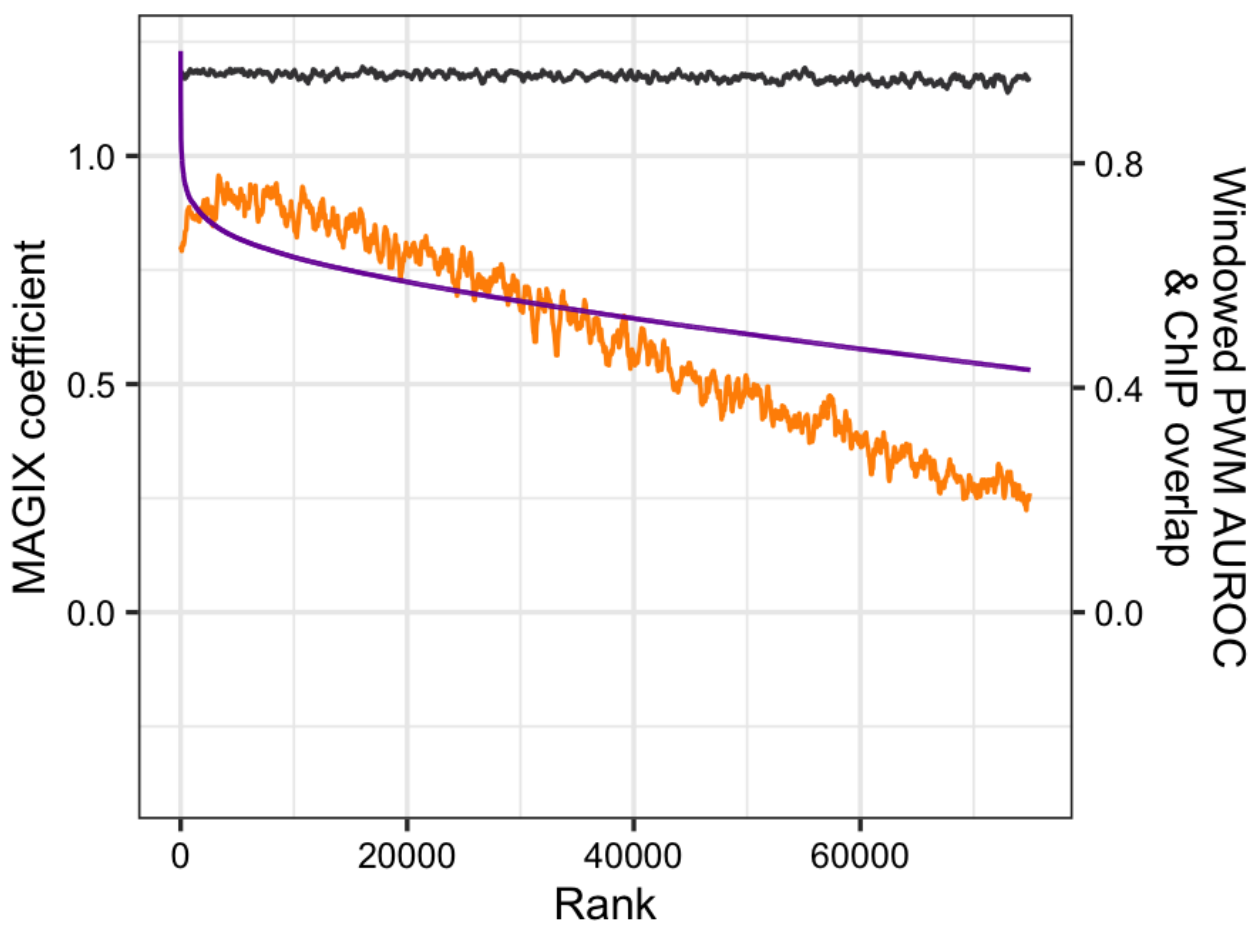

LEF1

Top GRECO-BIT/MEX PWM: PWM000926

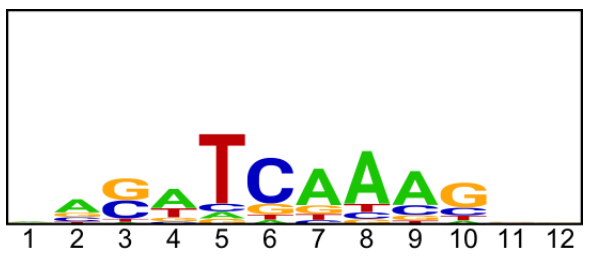

Location of top GRECO-BIT/MEX PWM in MAGIX peaks (up to the top 10,000)

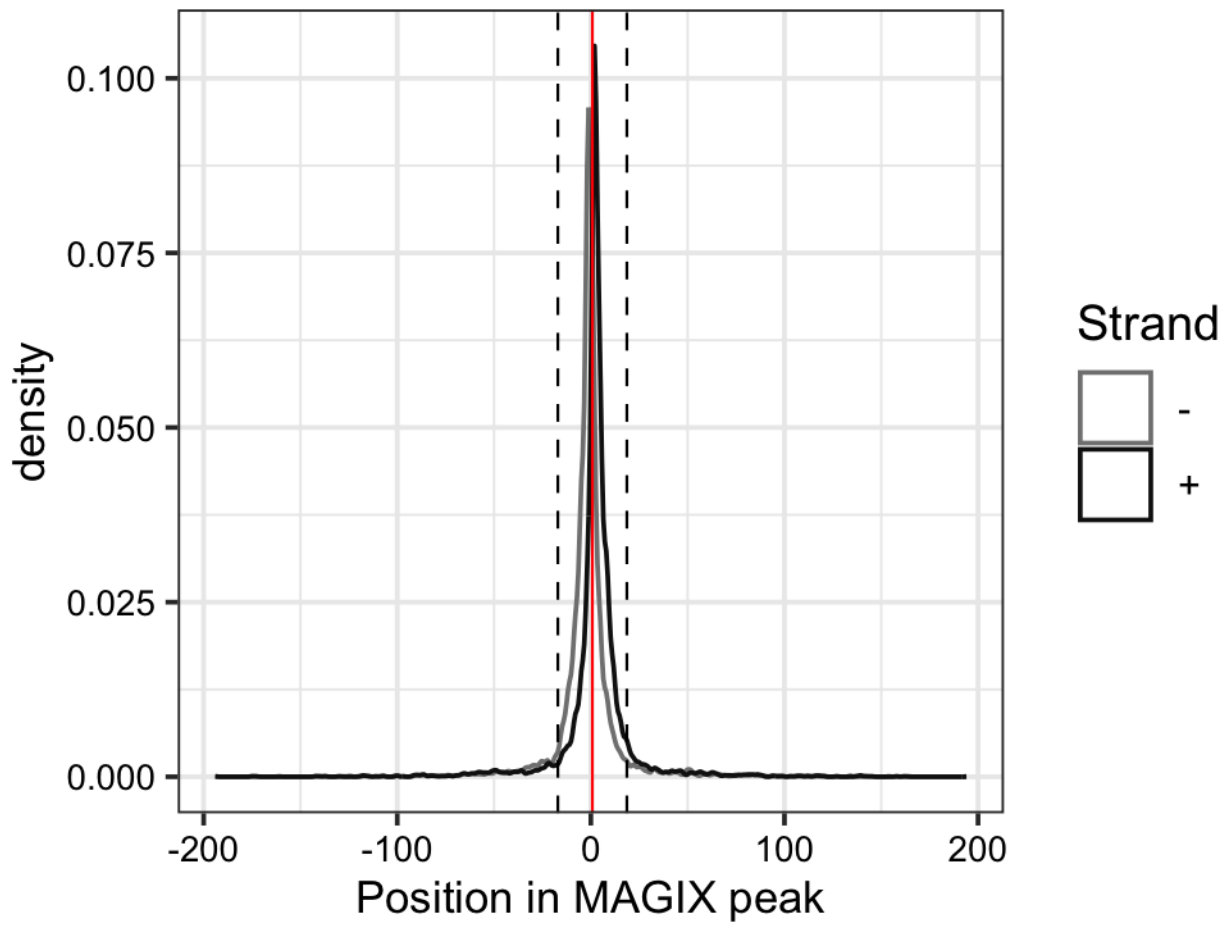

AUROC of top GRECO-BIT/MEX PWM in sliding windows (500 peaks, 50 peak step) along the MAGIX peaks (up to the top 75,000) ranked by the MAGIX enrichment coefficient and fraction of overlapping ChIP peaks in the same window

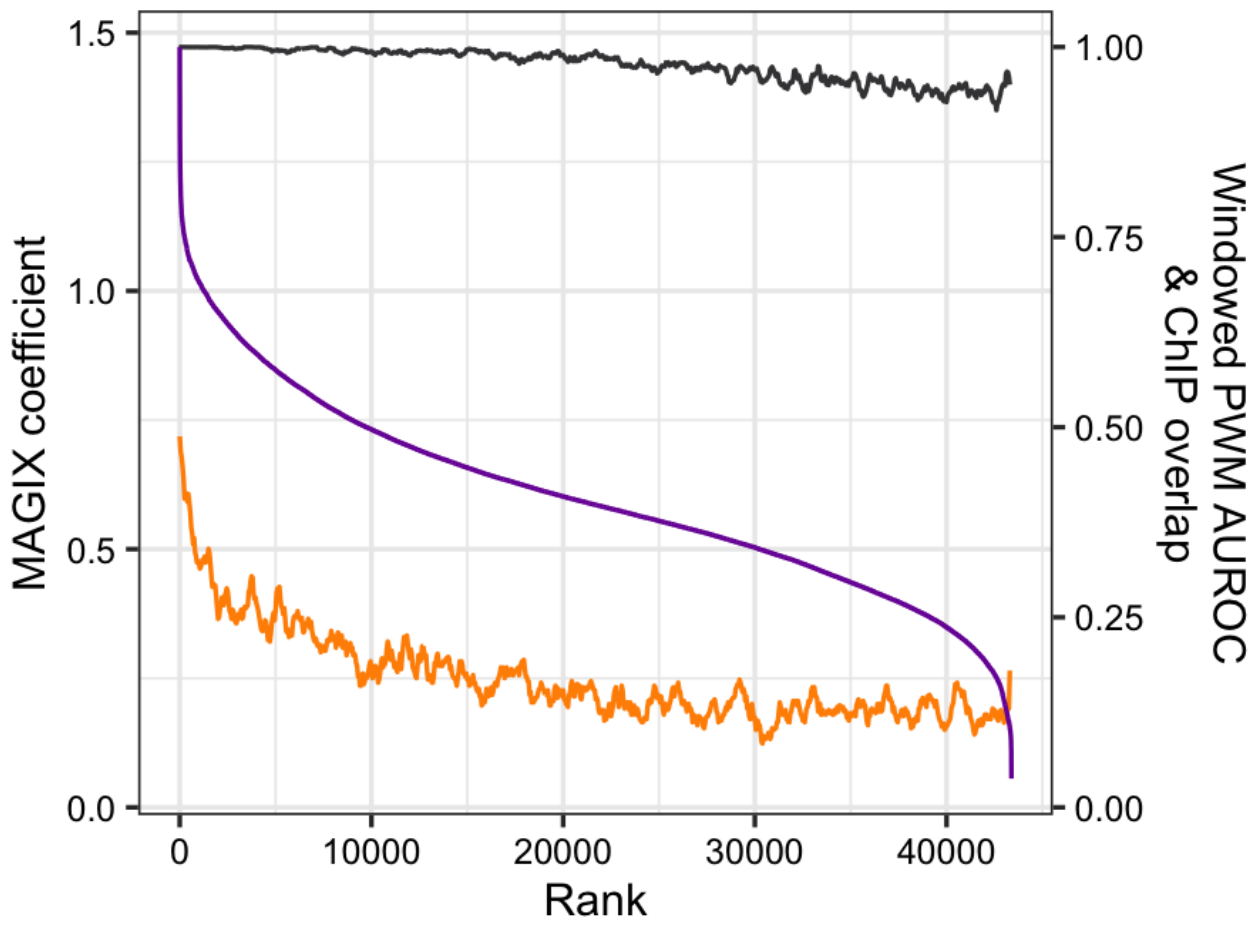

LEUTX

Top GRECO-BIT/MEX PWM: PWM000952

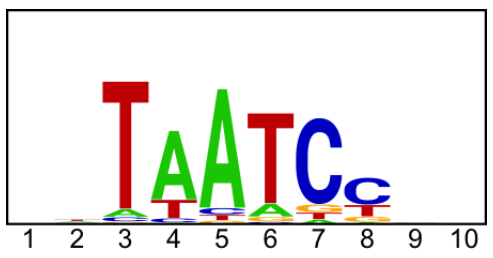

Location of top GRECO-BIT/MEX PWM in MAGIX peaks (up to the top 10,000)

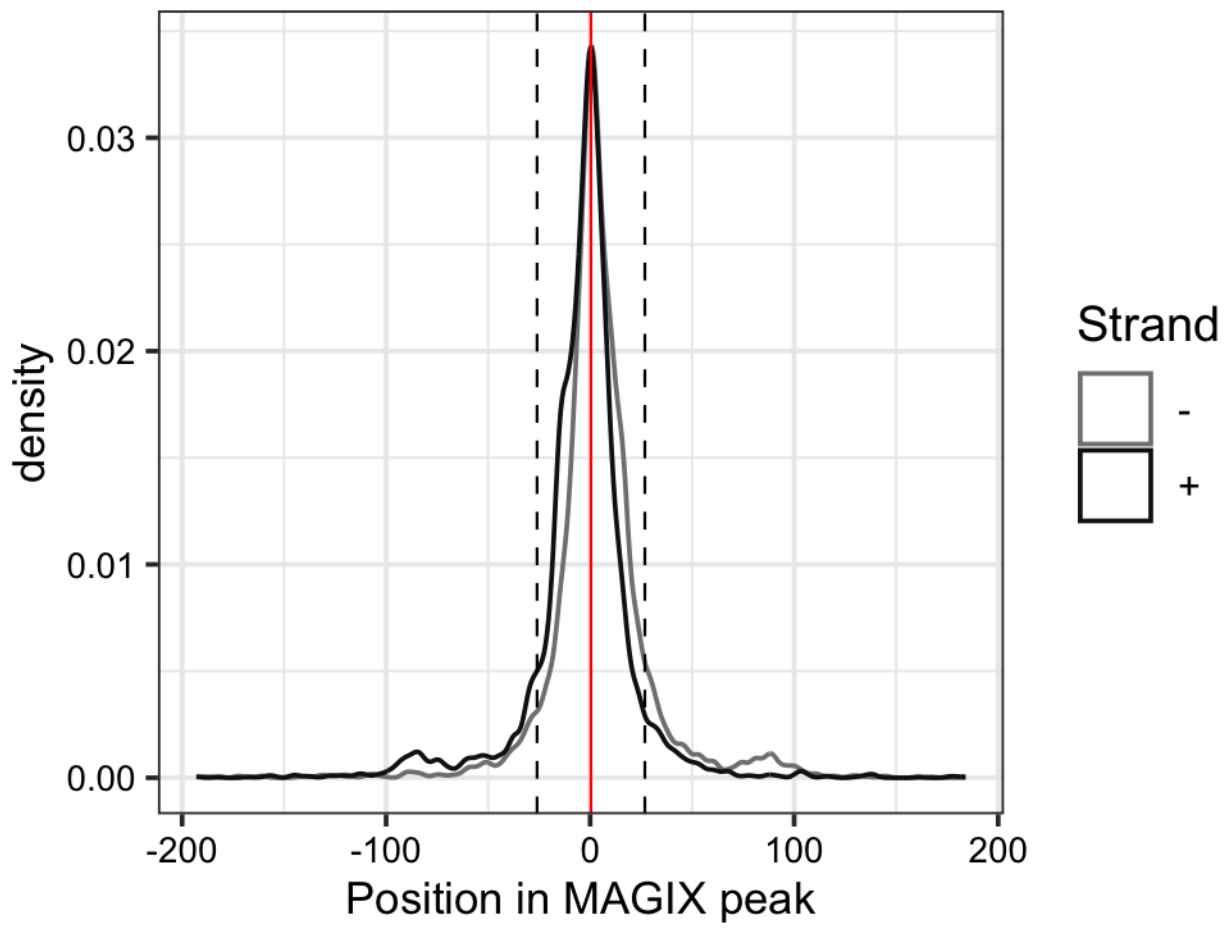

AUROC of top GRECO-BIT/MEX PWM in sliding windows (500 peaks, 50 peak step) along the MAGIX peaks (up to the top 75,000) ranked by the MAGIX enrichment coefficient and fraction of overlapping ChIP peaks in the same window

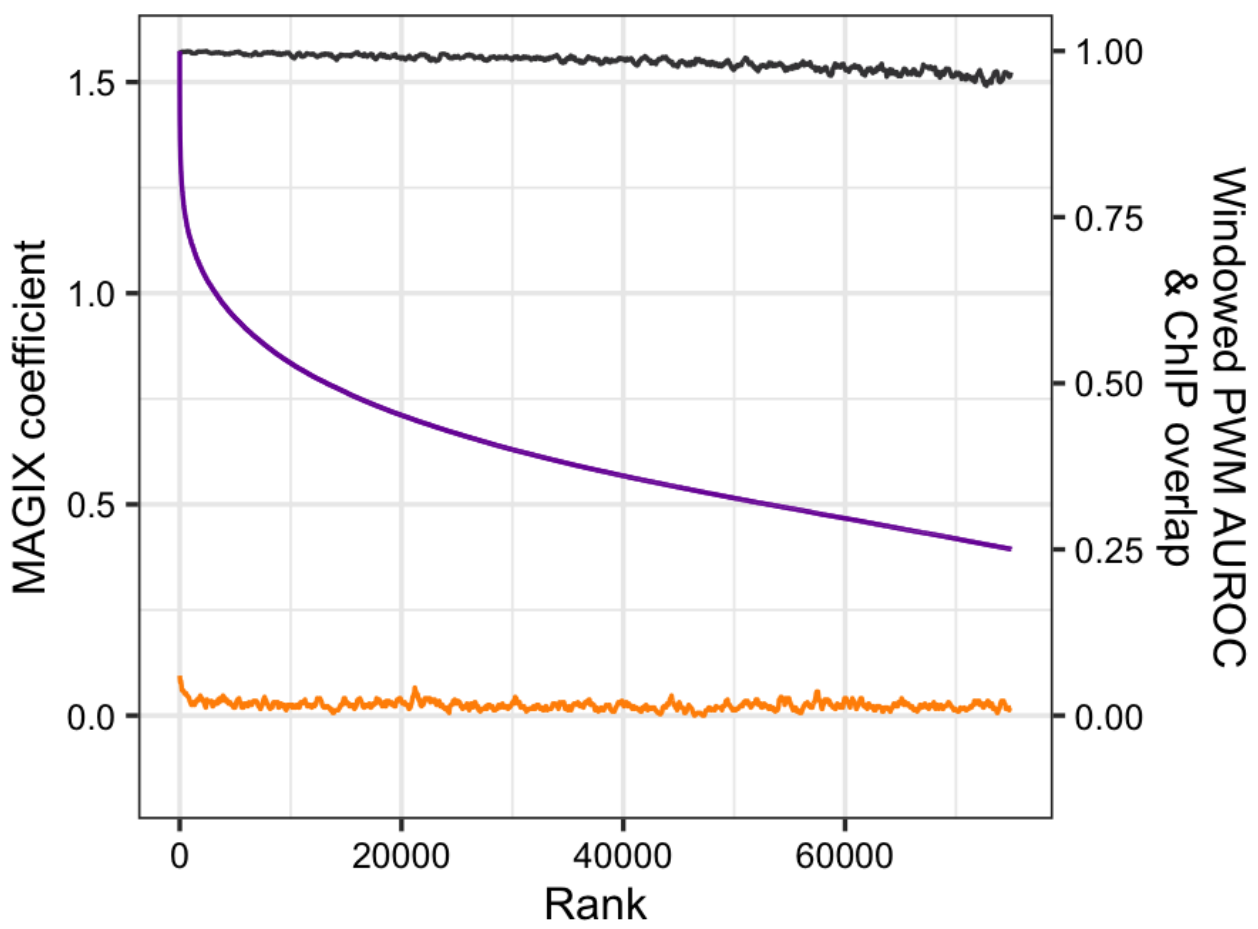

MAX

Top GRECO-BIT/MEX PWM: PWM001030

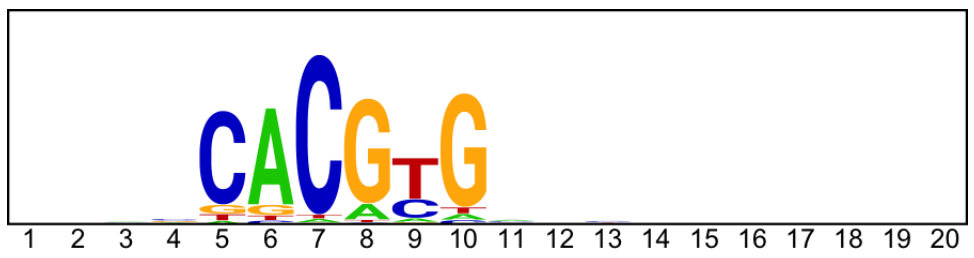

Location of top GRECO-BIT/MEX PWM in MAGIX peaks (up to the top 10,000)

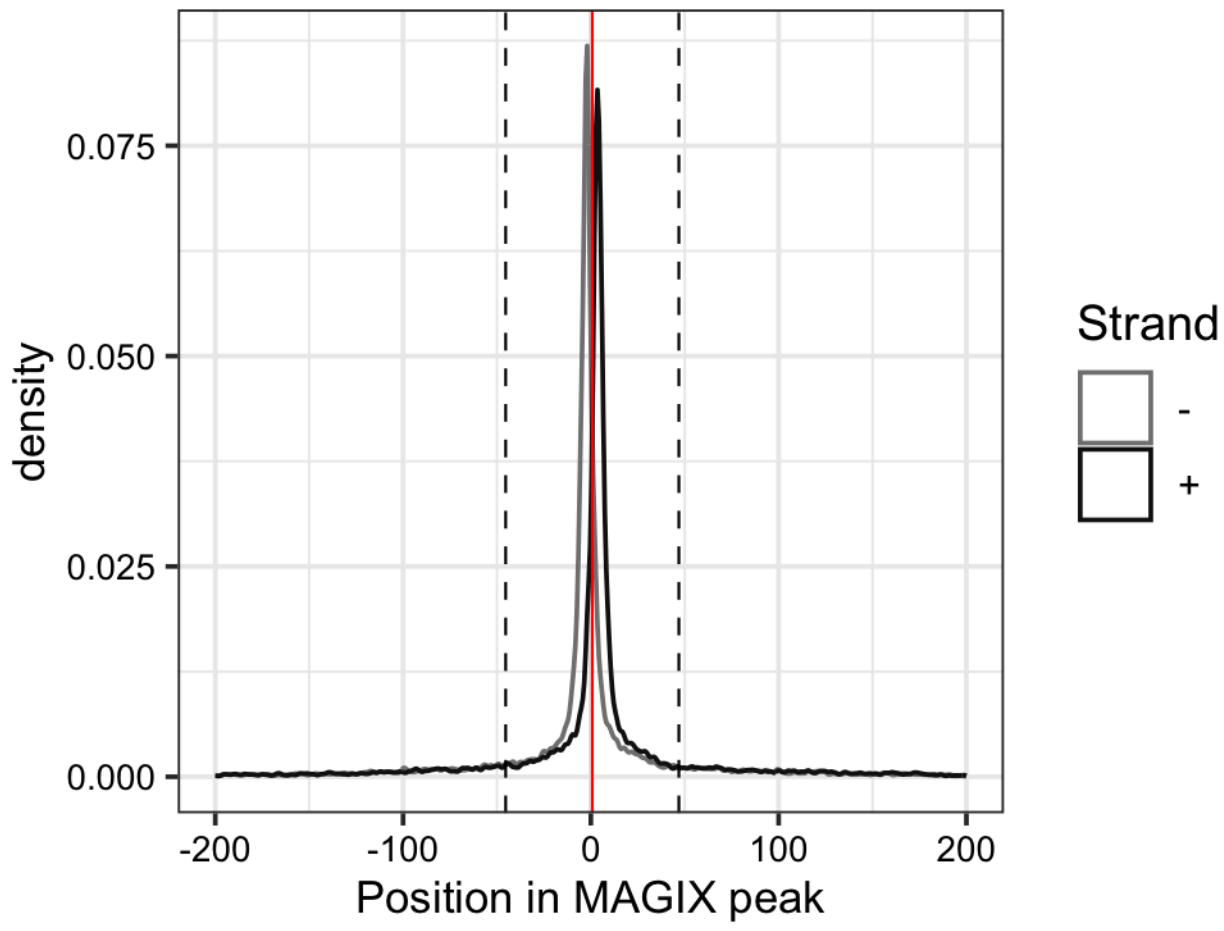

AUROC of top GRECO-BIT/MEX PWM in sliding windows (500 peaks, 50 peak step) along the MAGIX peaks (up to the top 75,000) ranked by the MAGIX enrichment coefficient and fraction of overlapping ChIP peaks in the same window

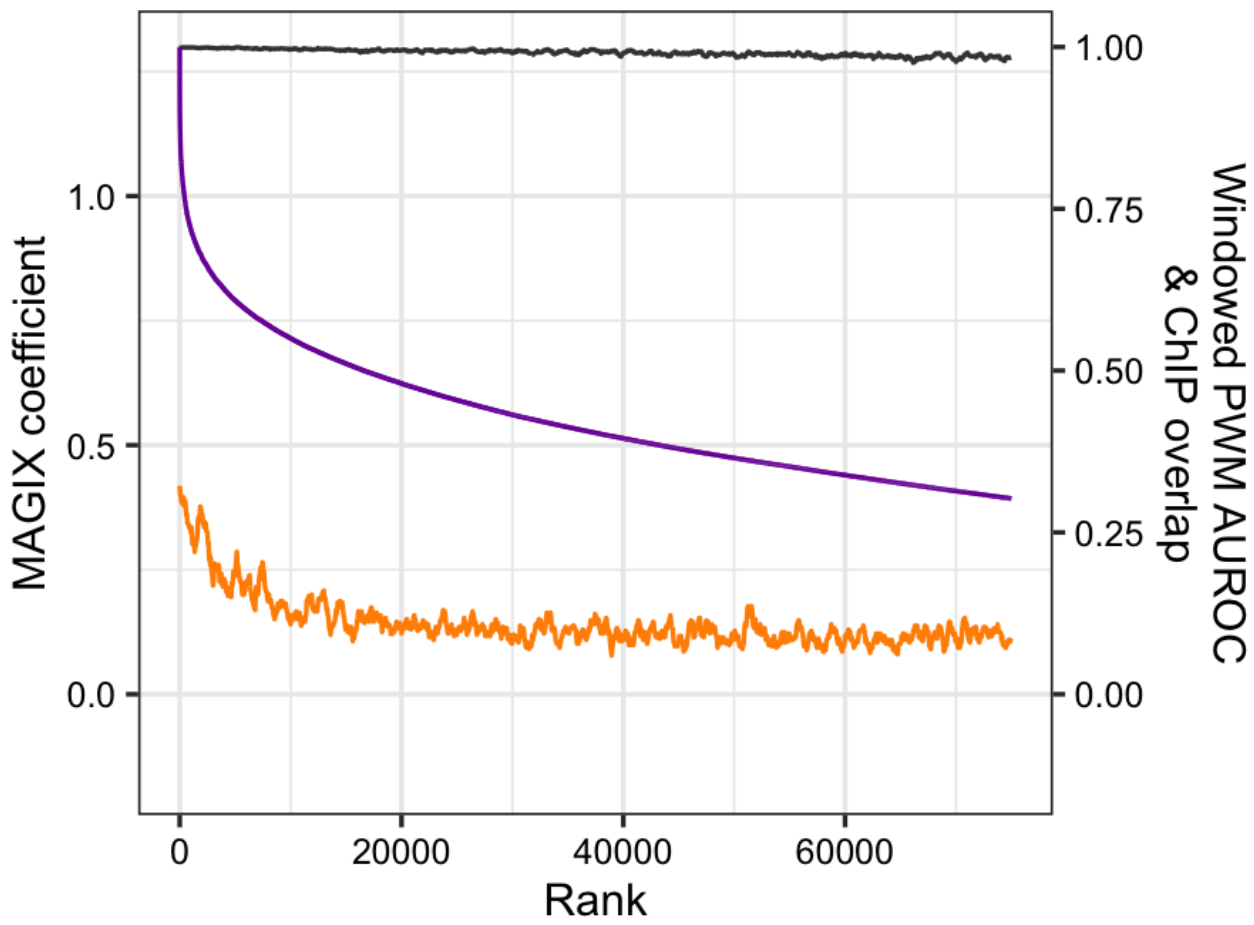

MGA

Top GRECO-BIT/MEX PWM: PWM001108

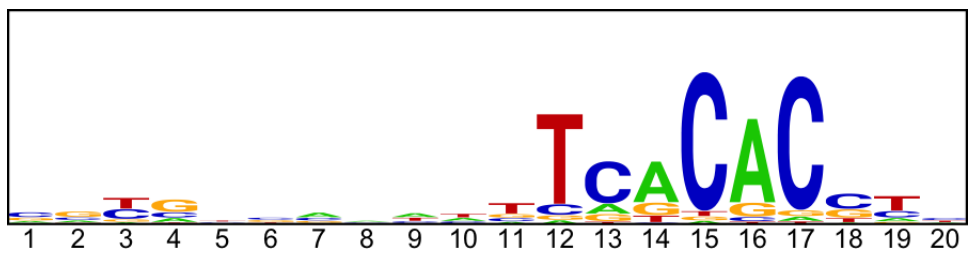

Location of top GRECO-BIT/MEX PWM in MAGIX peaks (up to the top 10,000)

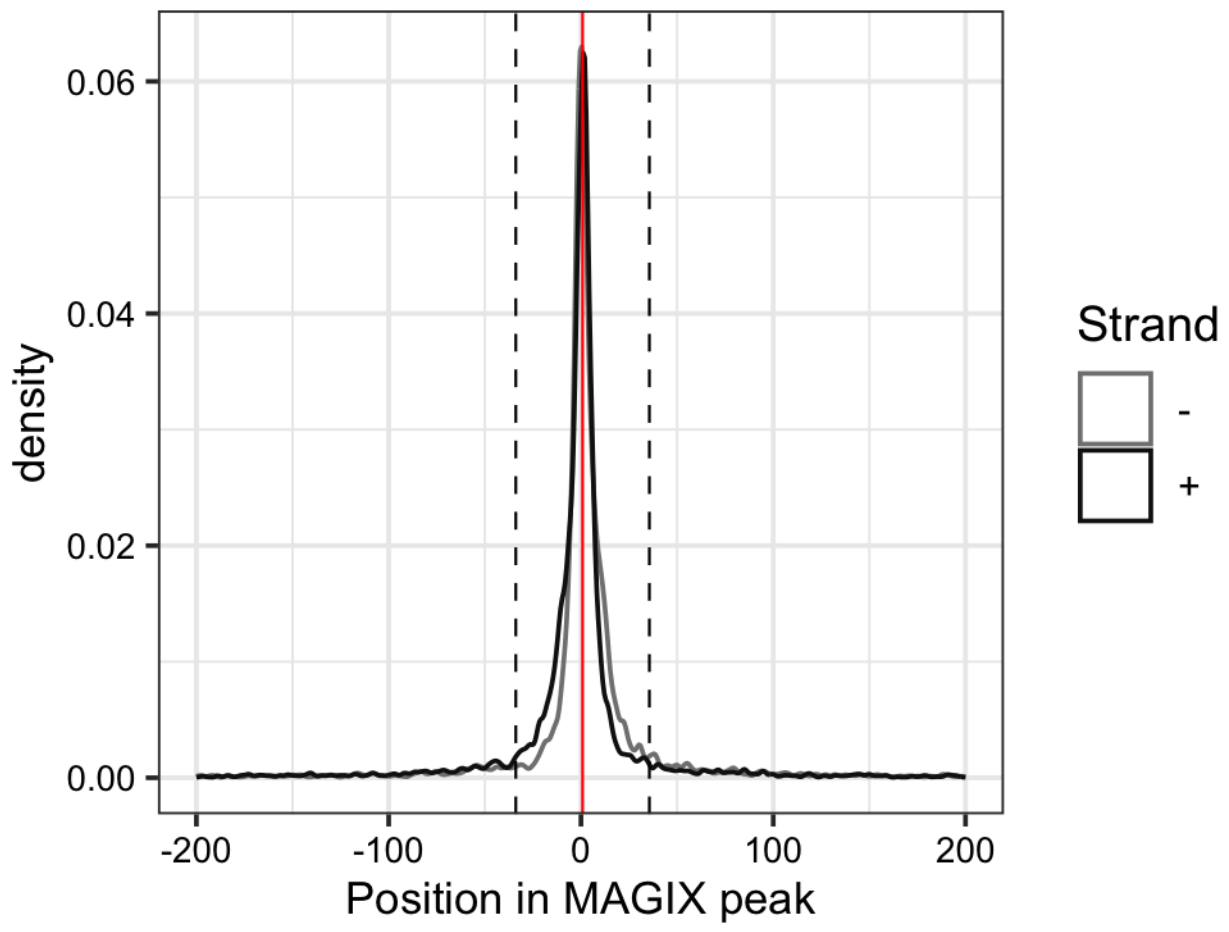

AUROC of top GRECO-BIT/MEX PWM in sliding windows (500 peaks, 50 peak step) along the MAGIX peaks (up to the top 75,000) ranked by the MAGIX enrichment coefficient and fraction of overlapping ChIP peaks in the same window

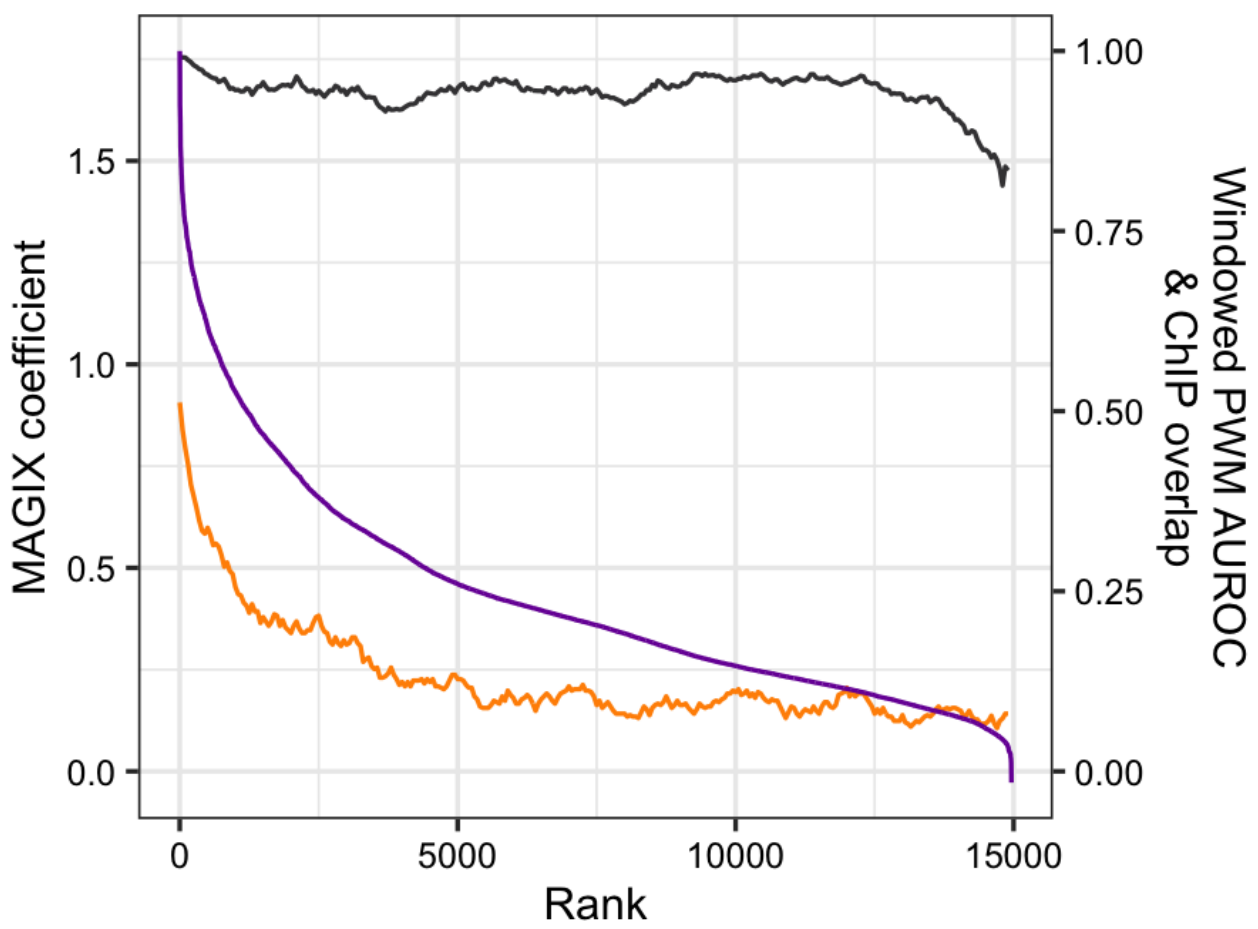

MKX

Top GRECO-BIT/MEX PWM: PWM001148

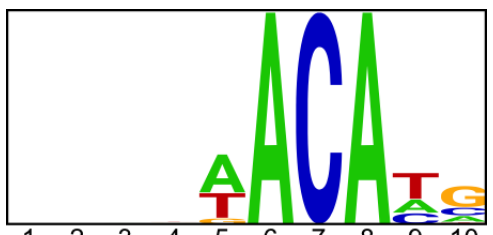

Location of top GRECO-BIT/MEX PWM in MAGIX peaks (up to the top 10,000)

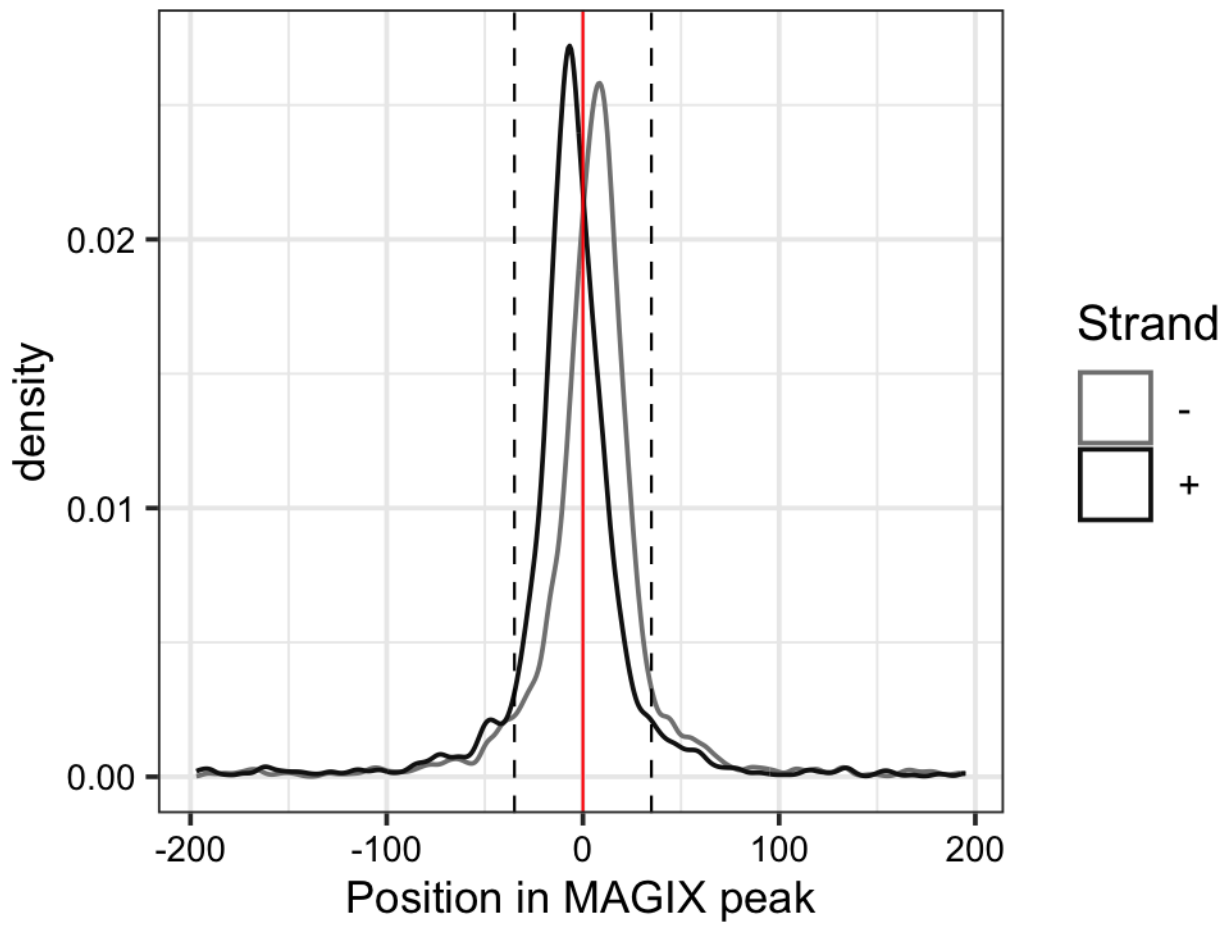

AUROC of top GRECO-BIT/MEX PWM in sliding windows (500 peaks, 50 peak step) along the MAGIX peaks (up to the top 75,000) ranked by the MAGIX enrichment coefficient and fraction of overlapping ChIP peaks in the same window

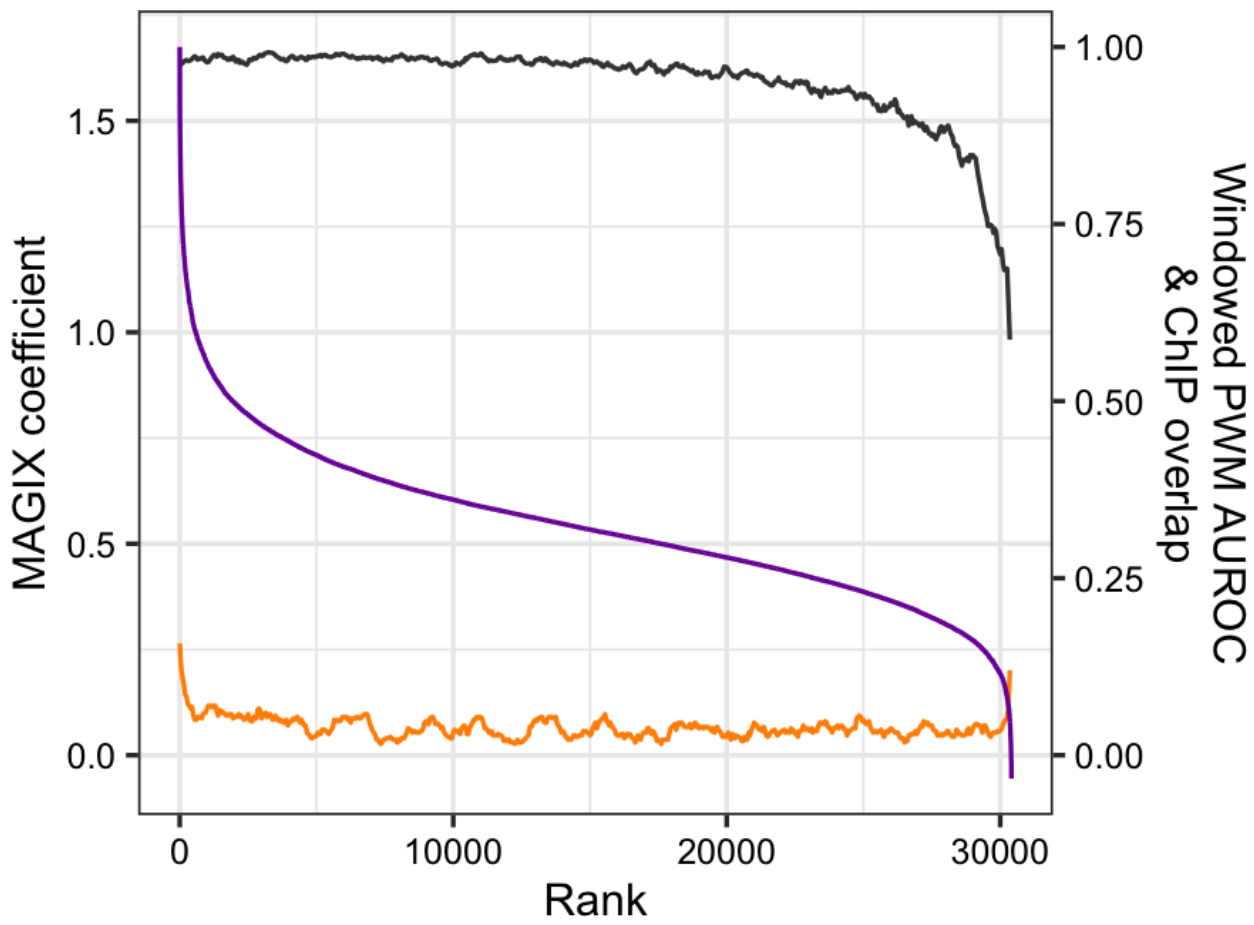

MSANTD1

Top GRECO-BIT/MEX PWM: PWM001171

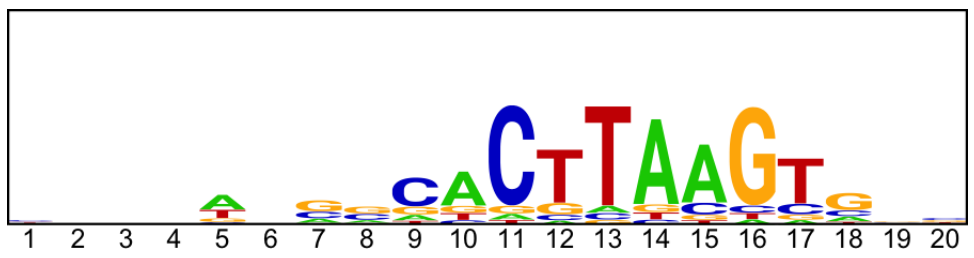

Location of top GRECO-BIT/MEX PWM in MAGIX peaks (up to the top 10,000)

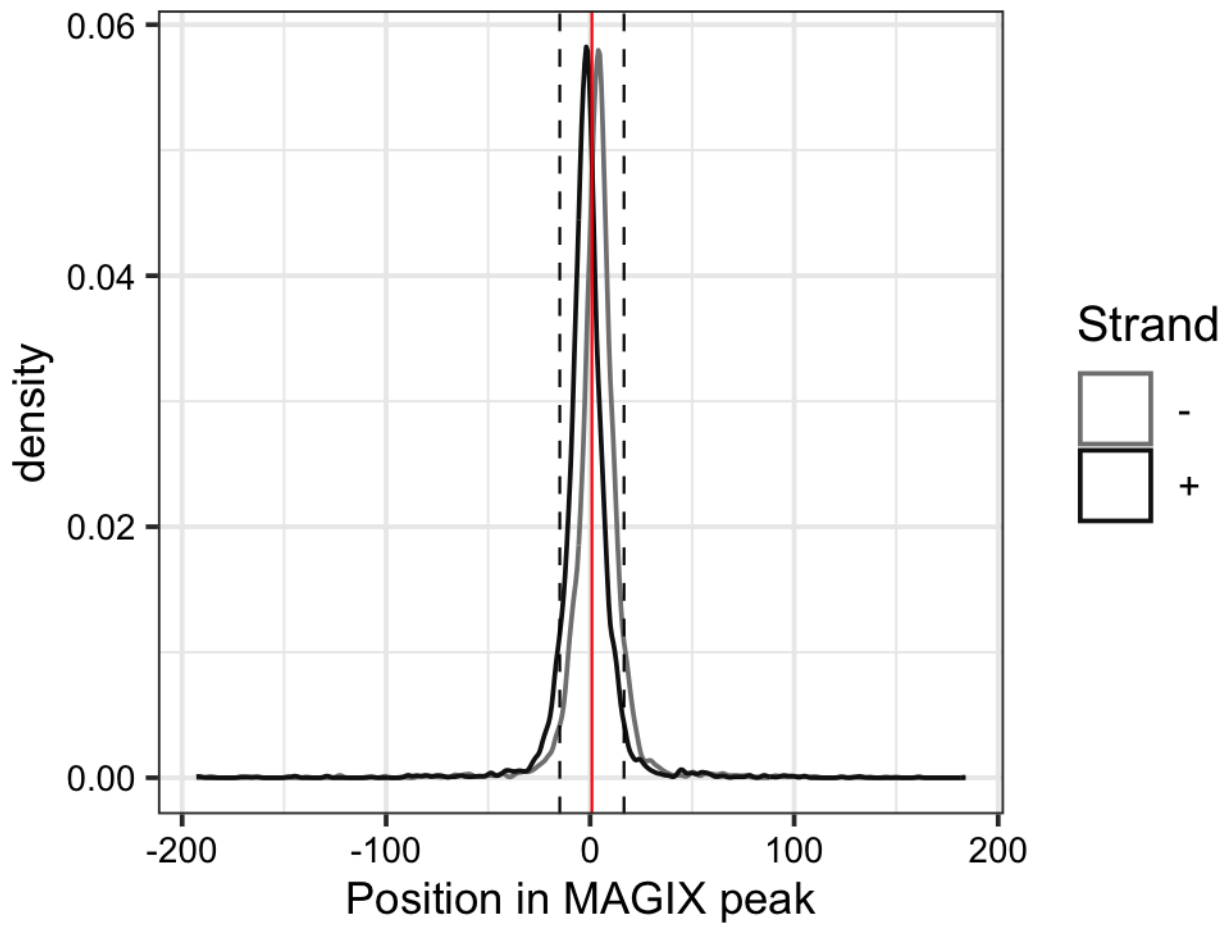

AUROC of top GRECO-BIT/MEX PWM in sliding windows (500 peaks, 50 peak step) along the MAGIX peaks (up to the top 75,000) ranked by the MAGIX enrichment coefficient and fraction of overlapping ChIP peaks in the same window

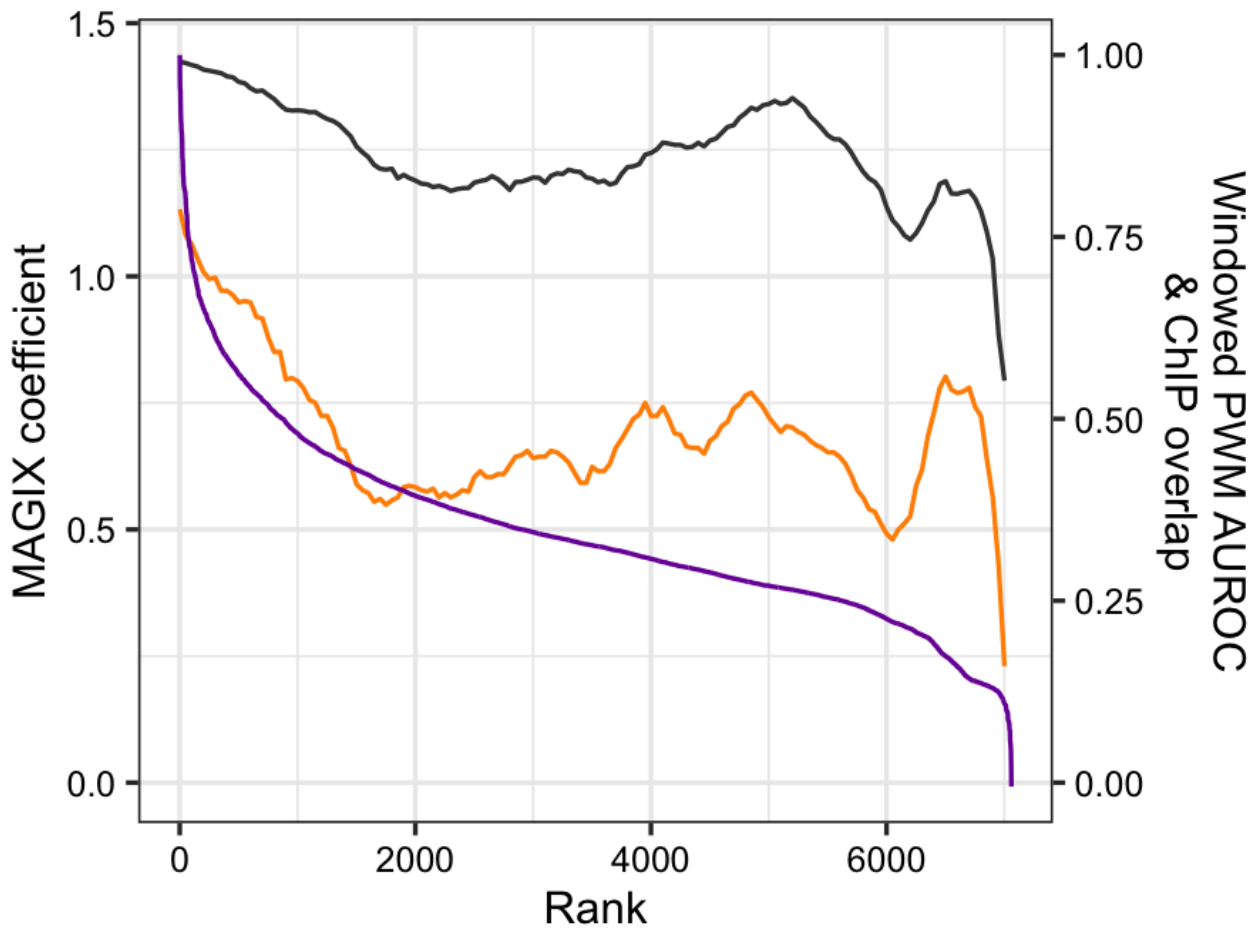

MSANTD4

Top GRECO-BIT/MEX PWM: PWM001209

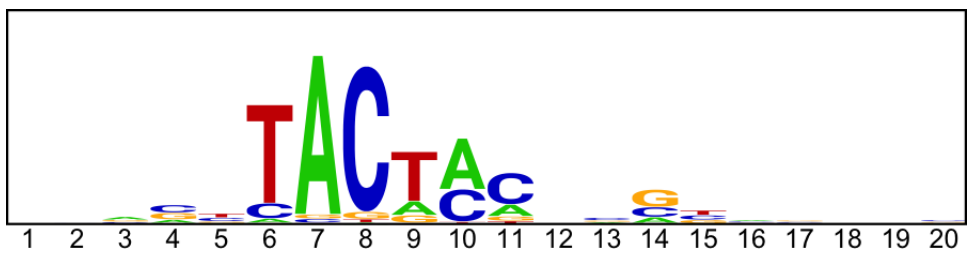

Location of top GRECO-BIT/MEX PWM in MAGIX peaks (up to the top 10,000)

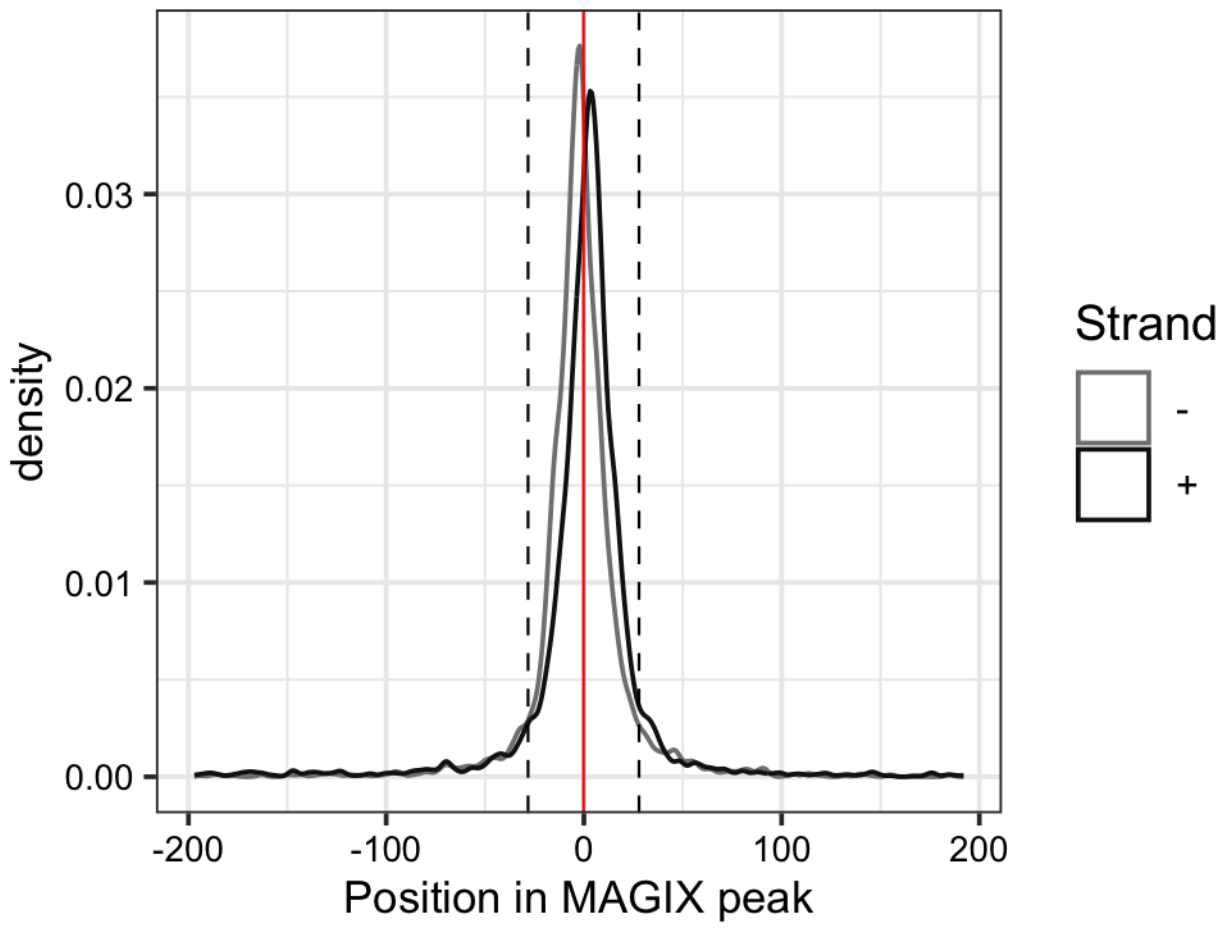

MYF6

Top GRECO-BIT/MEX PWM: PWM001265

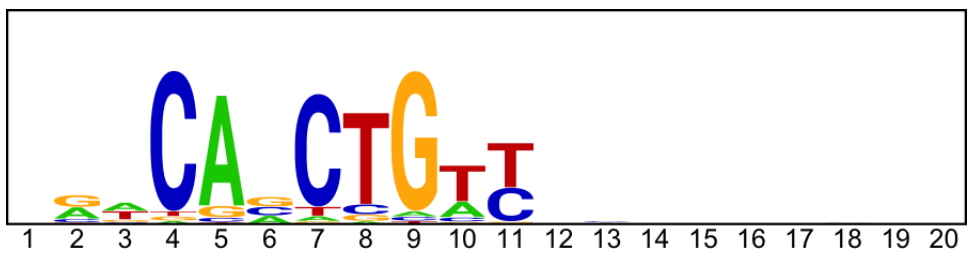

Location of top GRECO-BIT/MEX PWM in MAGIX peaks (up to the top 10,000)

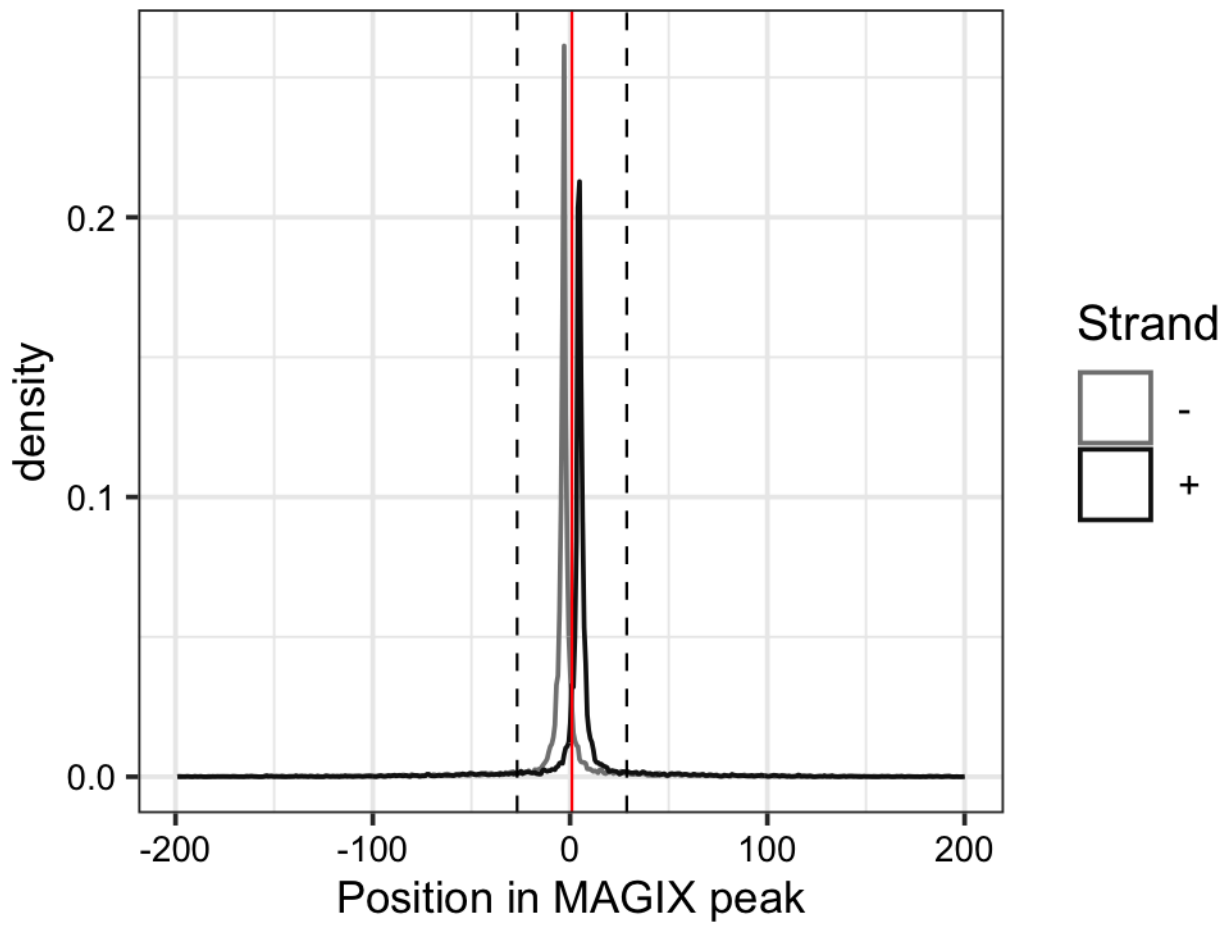

AUROC of top GRECO-BIT/MEX PWM in sliding windows (500 peaks, 50 peak step) along the MAGIX peaks (up to the top 75,000) ranked by the MAGIX enrichment coefficient and fraction of overlapping ChIP peaks in the same window

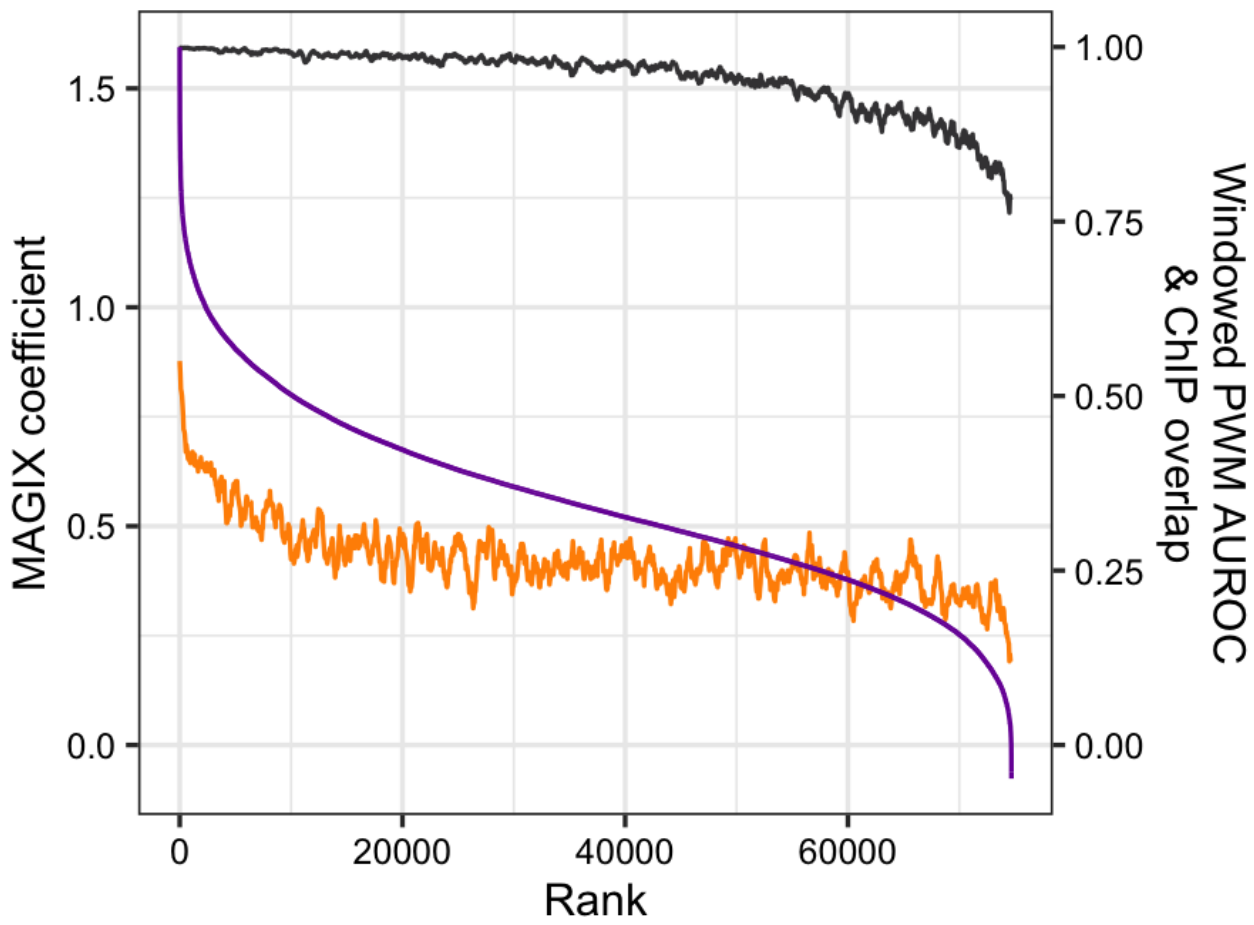

MYPOP

Top GRECO-BIT/MEX PWM: PWM001292

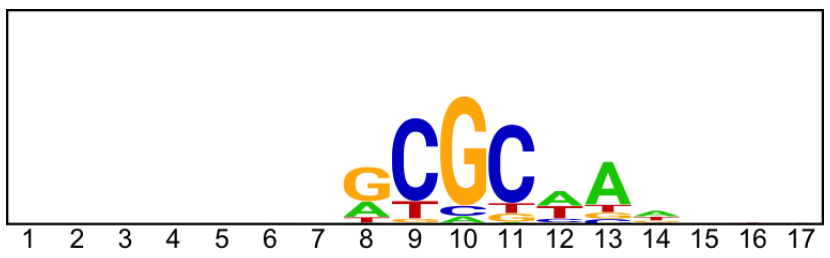

Location of top GRECO-BIT/MEX PWM in MAGIX peaks (up to the top 10,000)

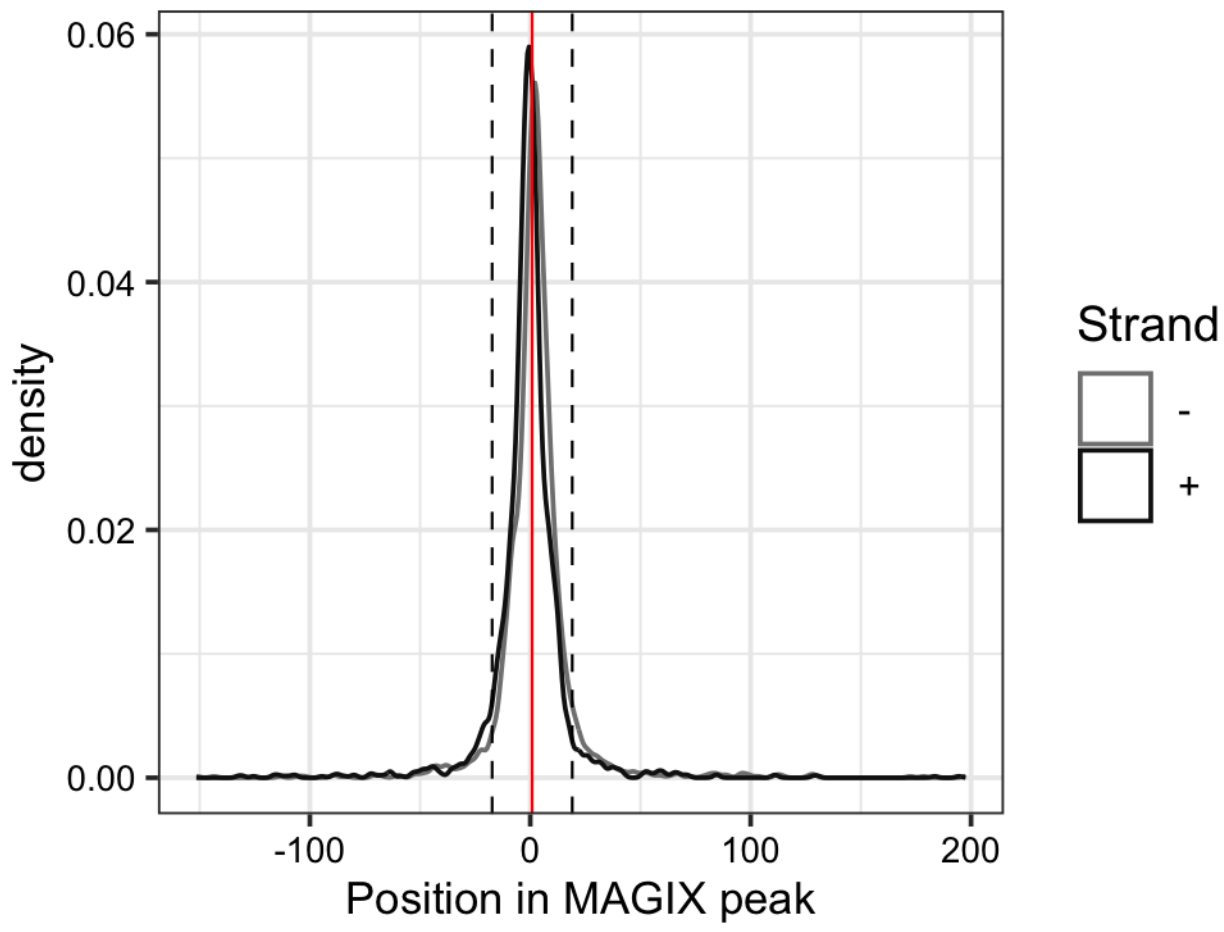

AUROC of top GRECO-BIT/MEX PWM in sliding windows (500 peaks, 50 peak step) along the MAGIX peaks (up to the top 75,000) ranked by the MAGIX enrichment coefficient and fraction of overlapping ChIP peaks in the same window

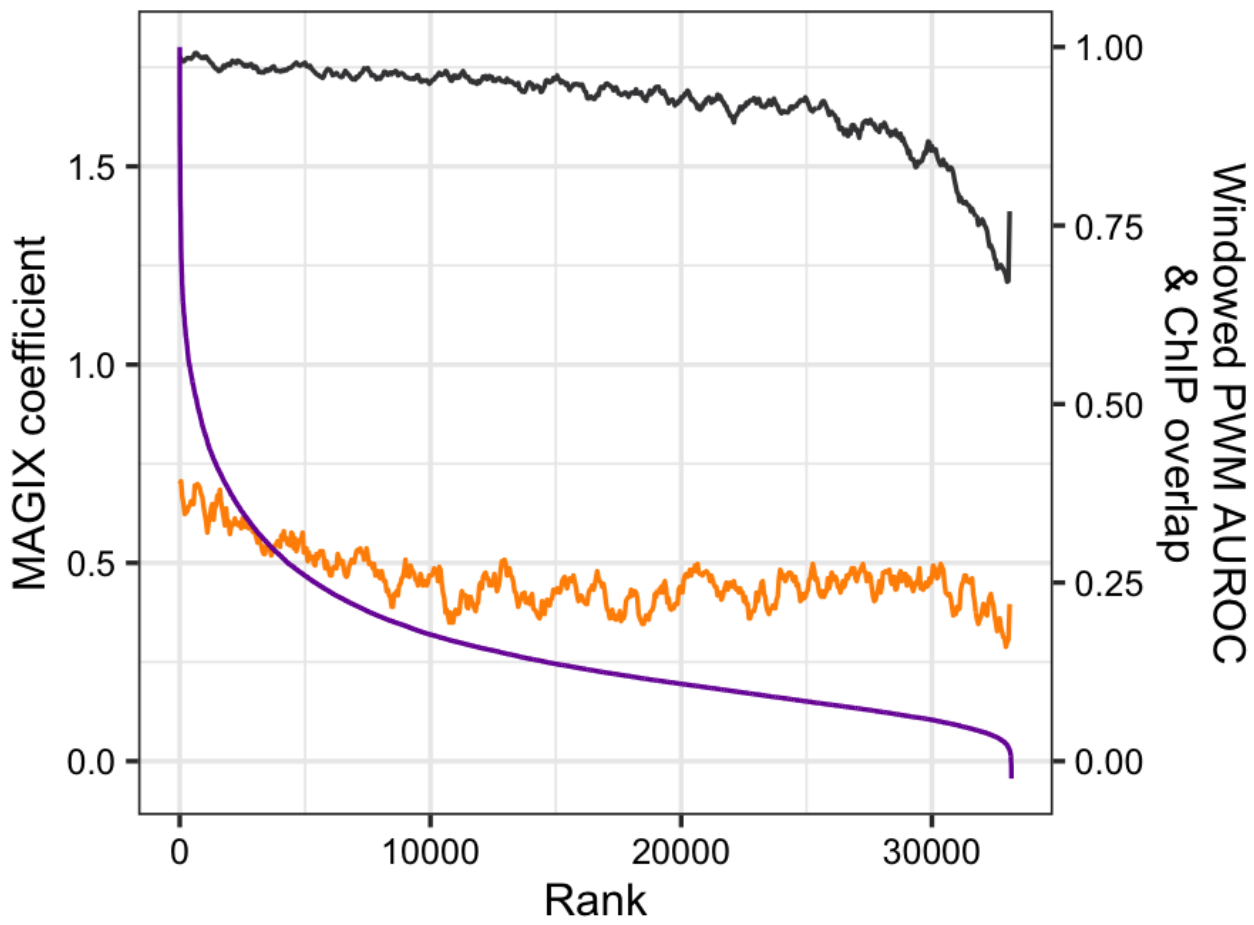

MYRFL

Top GRECO-BIT/MEX PWM: PWM002431

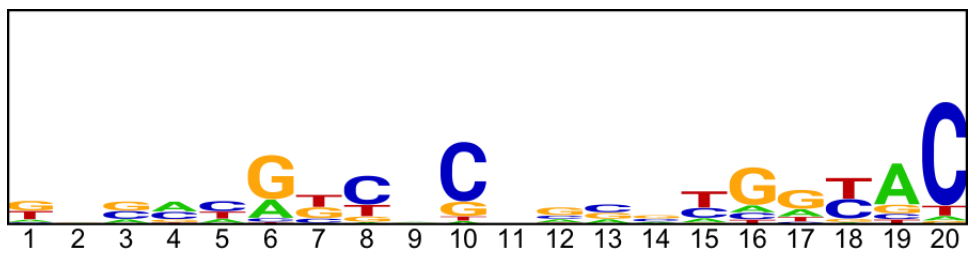

Location of top GRECO-BIT/MEX PWM in MAGIX peaks (up to the top 10,000)

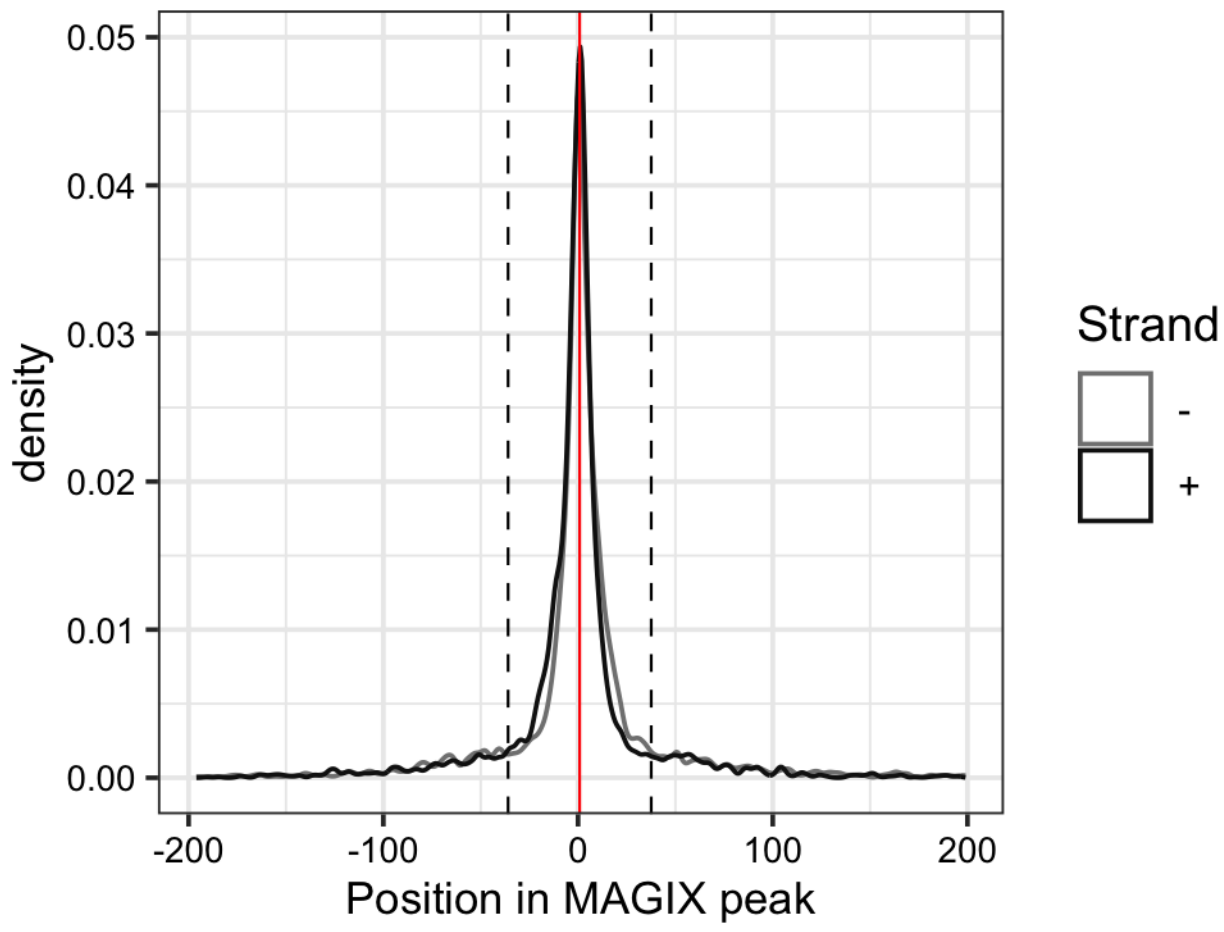

AUROC of top GRECO-BIT/MEX PWM in sliding windows (500 peaks, 50 peak step) along the MAGIX peaks (up to the top 75,000) ranked by the MAGIX enrichment coefficient and fraction of overlapping ChIP peaks in the same window

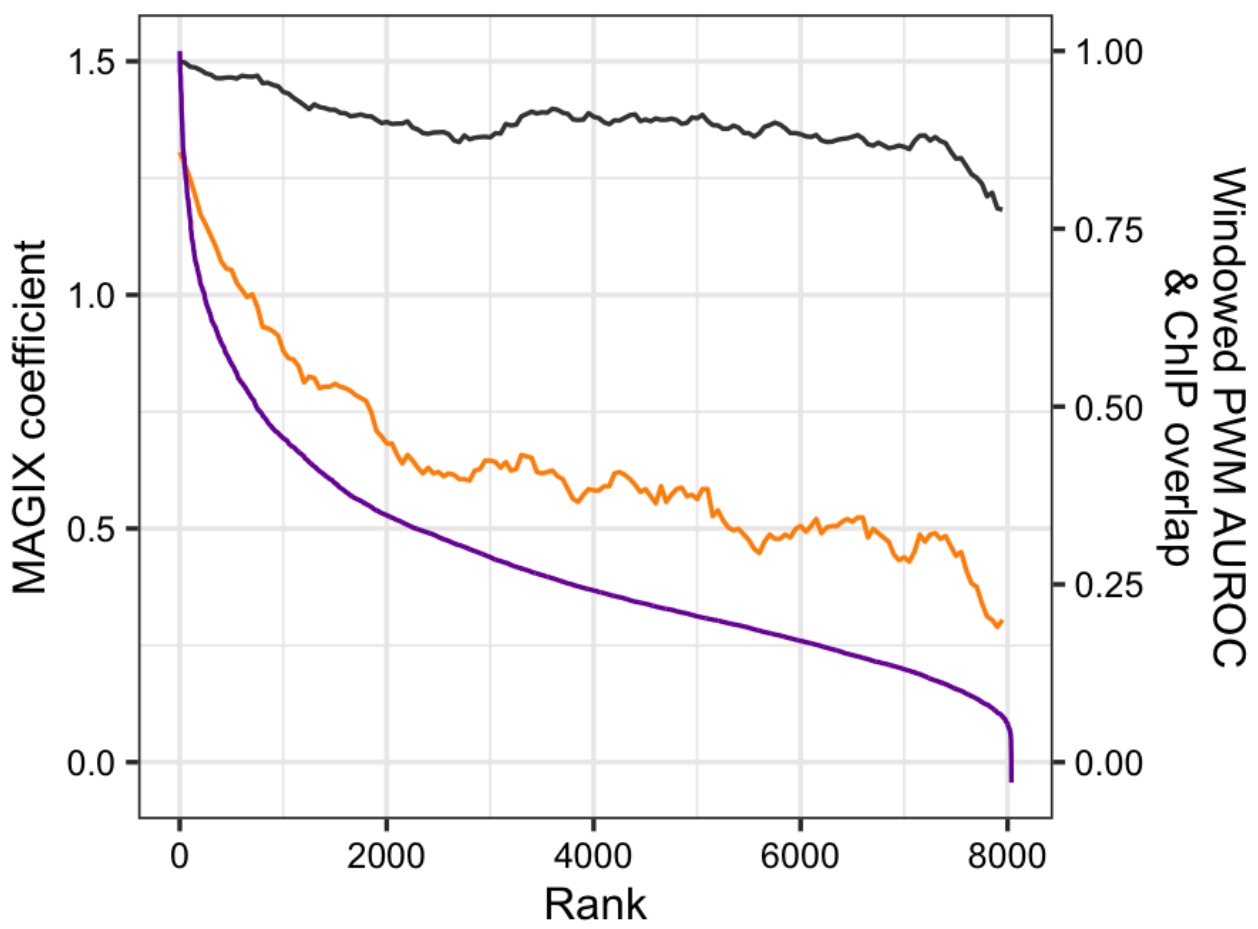

MYT1.DBD1

Top GRECO-BIT/MEX PWM: PWM053556

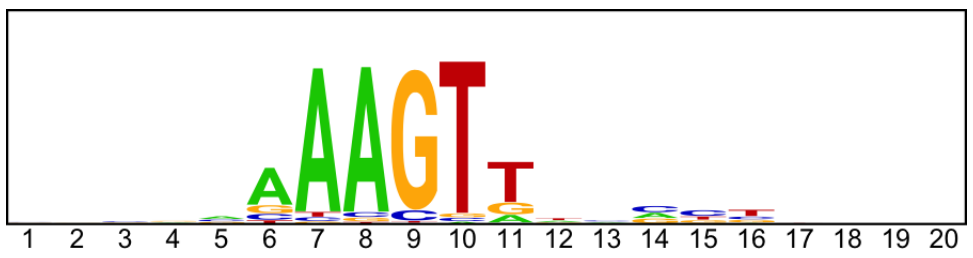

Location of top GRECO-BIT/MEX PWM in MAGIX peaks (up to the top 10,000)

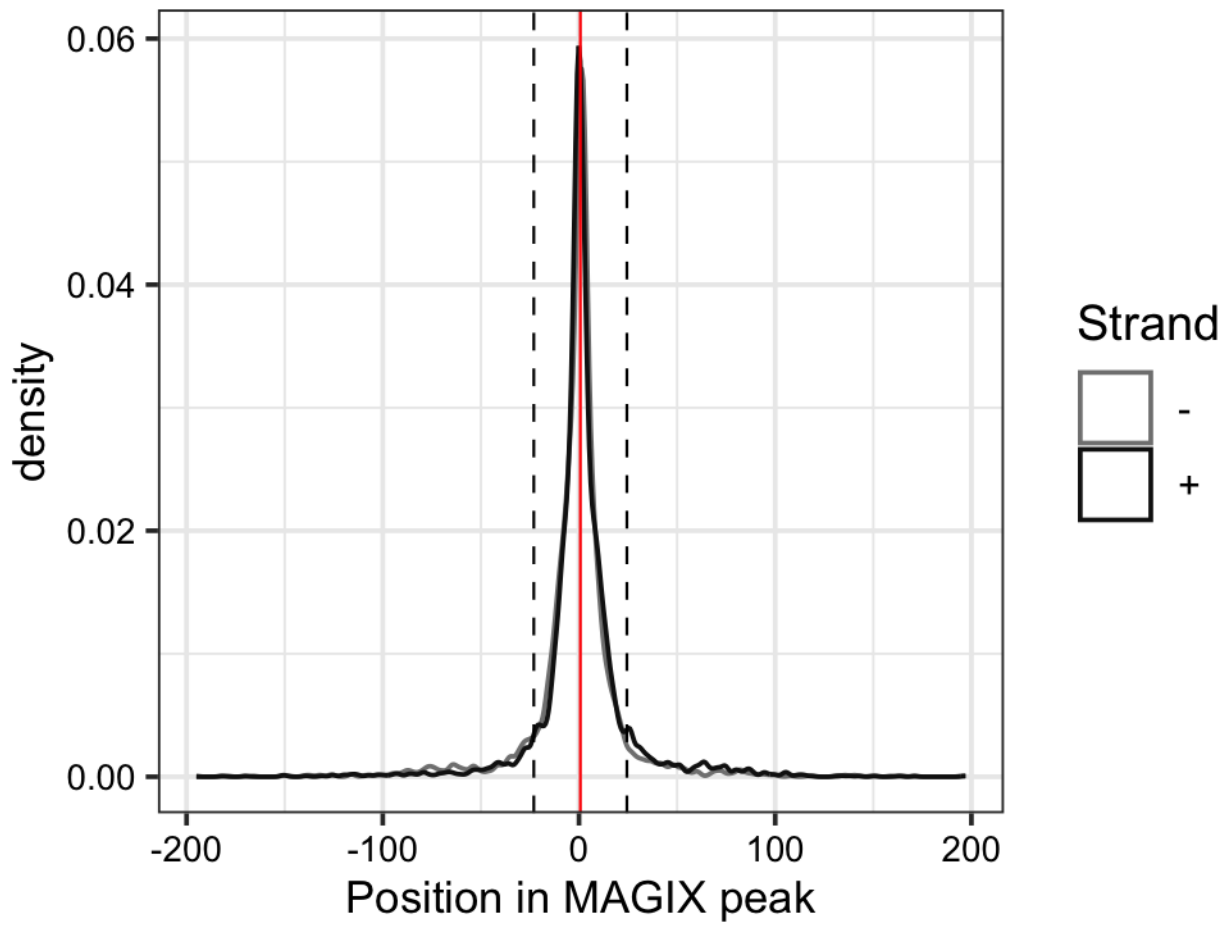

MYT1.DBD2

Top GRECO-BIT/MEX PWM: PWM001364

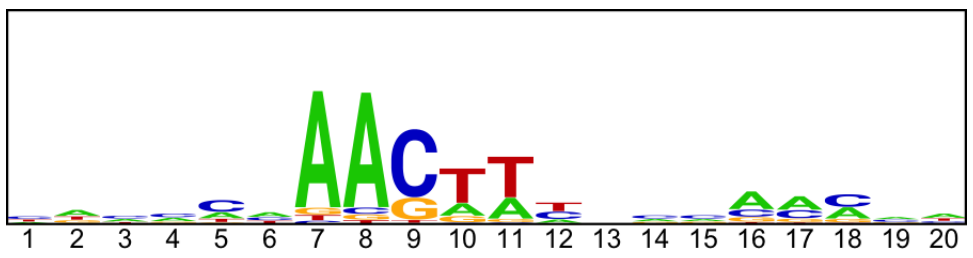

Location of top GRECO-BIT/MEX PWM in MAGIX peaks (up to the top 10,000)

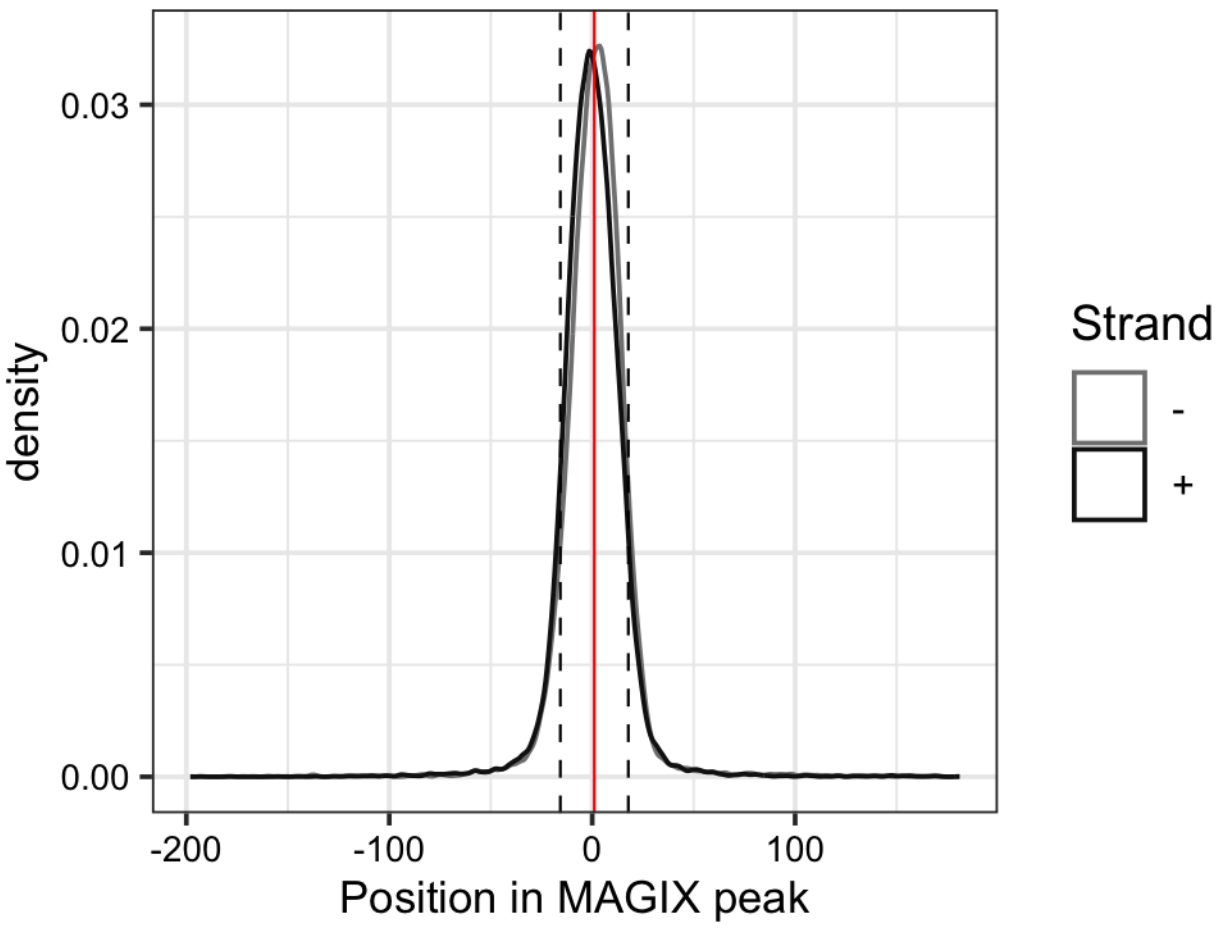

MYT1.FL

Top GRECO-BIT/MEX PWM: PWM001366

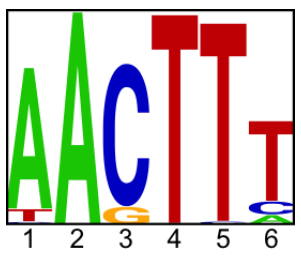

Location of top GRECO-BIT/MEX PWM in MAGIX peaks (up to the top 10,000)

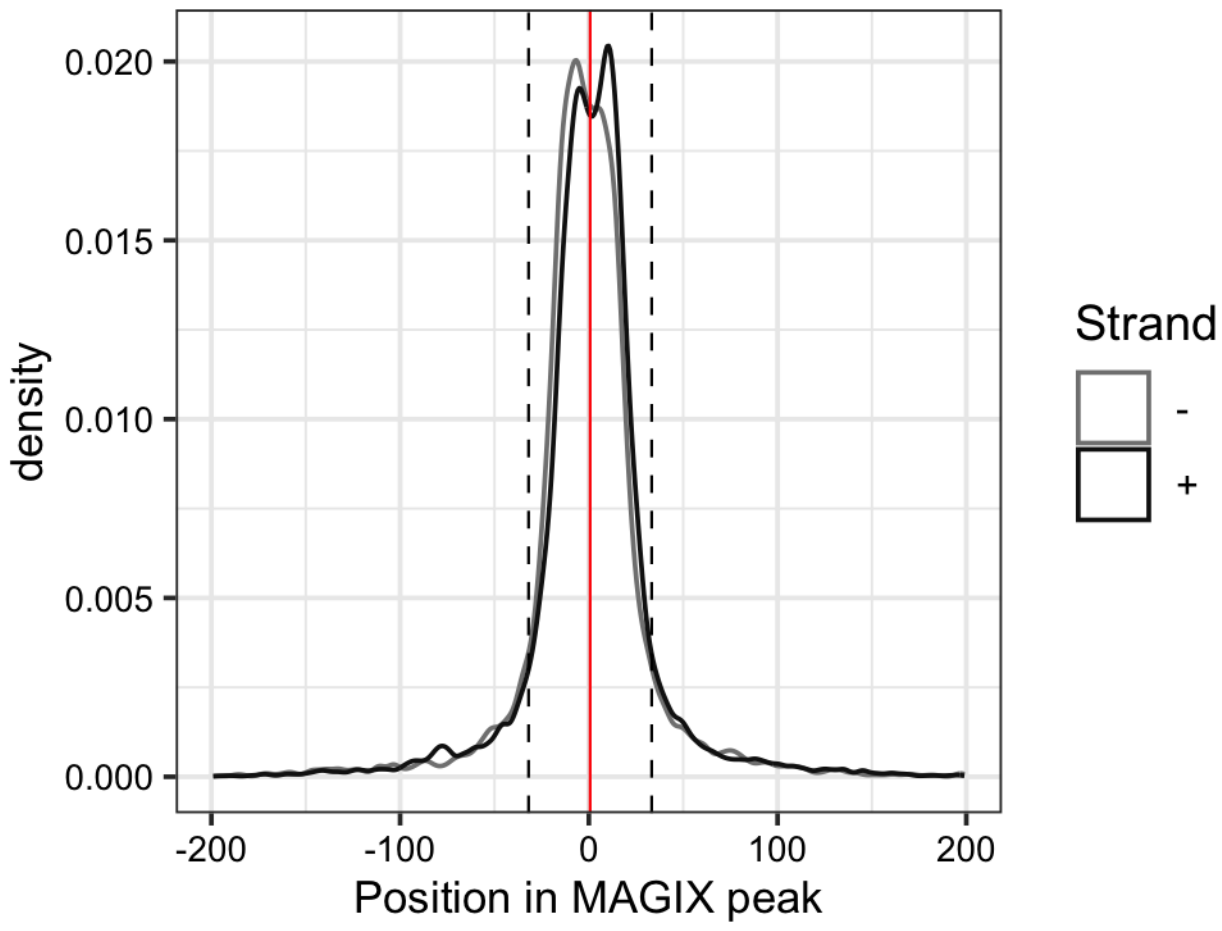

NACC2

Top GRECO-BIT/MEX PWM: PWM001390

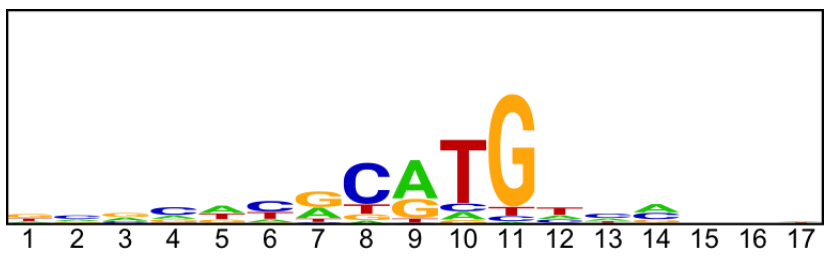

Location of top GRECO-BIT/MEX PWM in MAGIX peaks (up to the top 10,000)

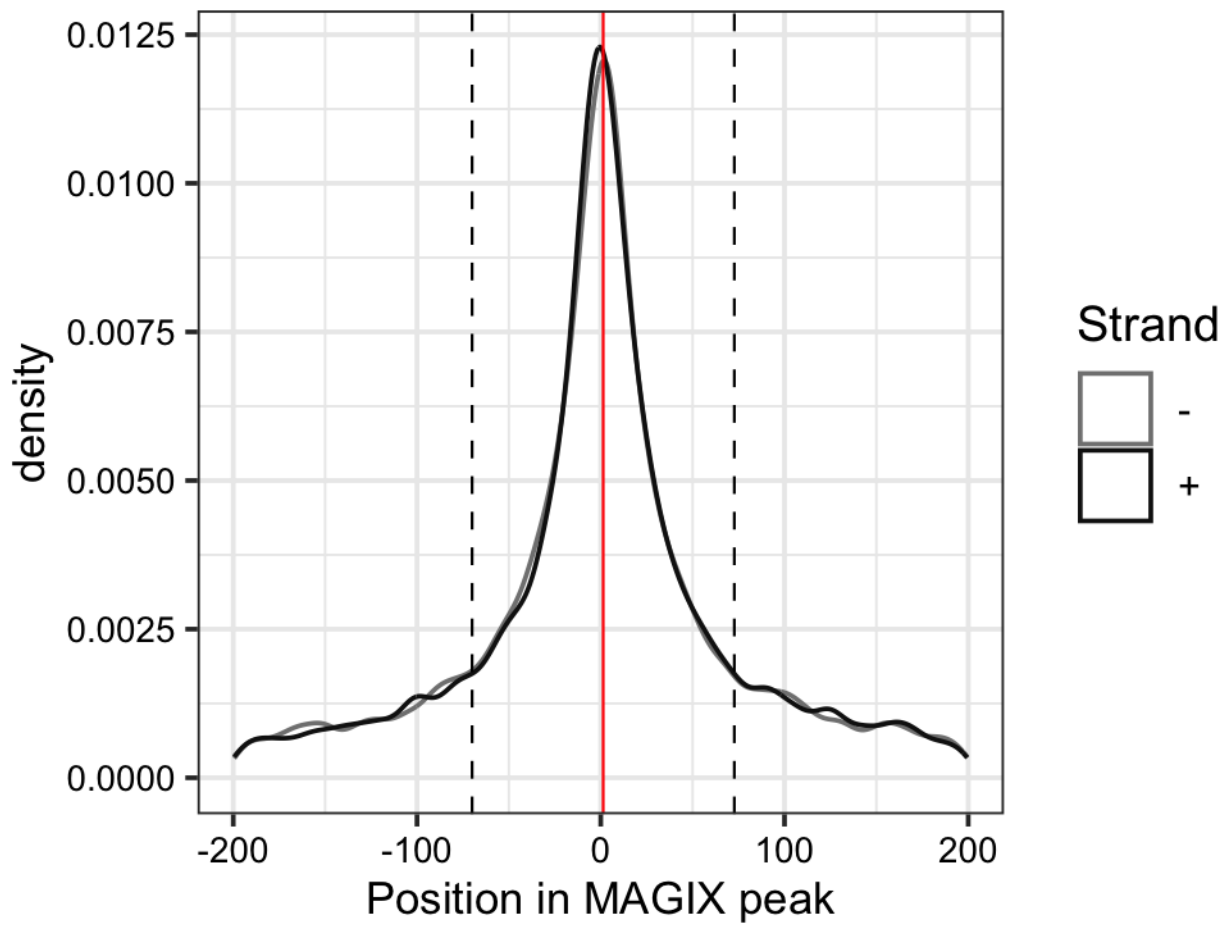

AUROC of top GRECO-BIT/MEX PWM in sliding windows (500 peaks, 50 peak step) along the MAGIX peaks (up to the top 75,000) ranked by the MAGIX enrichment coefficient and fraction of overlapping ChIP peaks in the same window

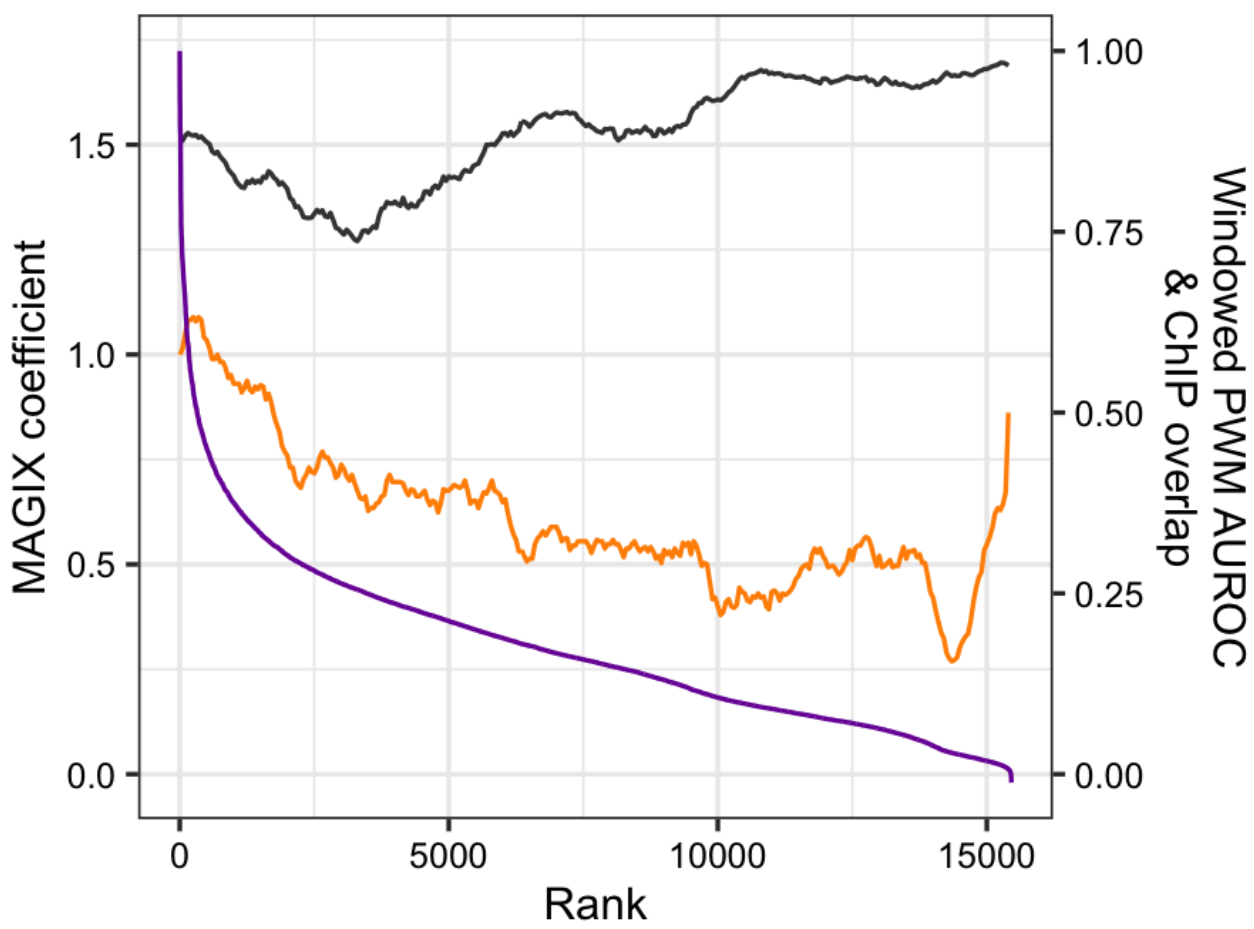

NFKB1

Top GRECO-BIT/MEX PWM: PWM001436

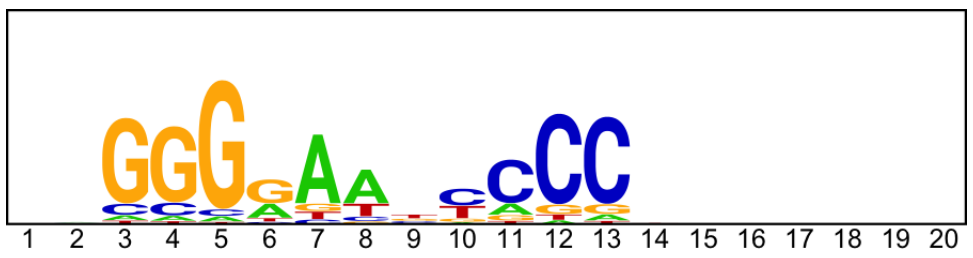

Location of top GRECO-BIT/MEX PWM in MAGIX peaks (up to the top 10,000)

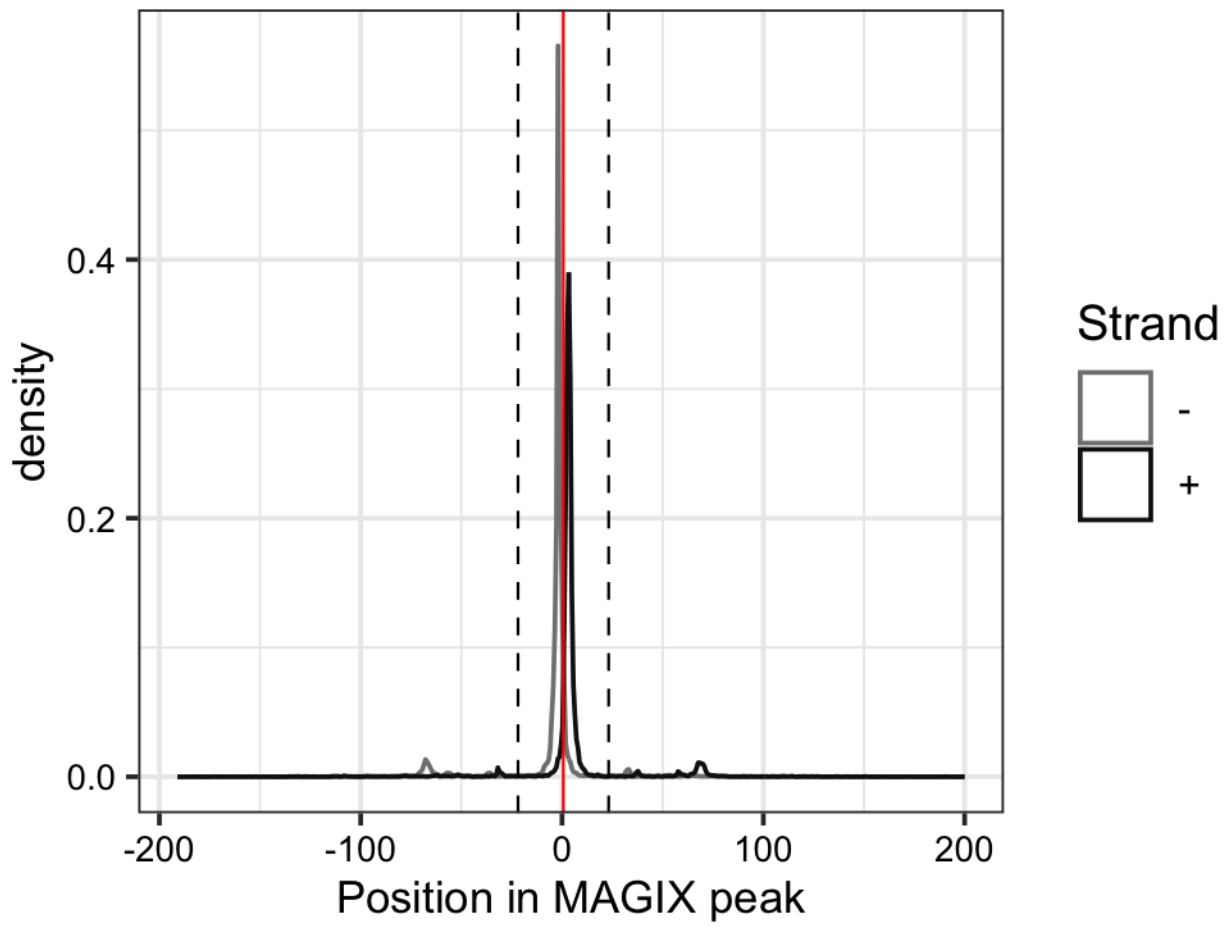

AUROC of top GRECO-BIT/MEX PWM in sliding windows (500 peaks, 50 peak step) along the MAGIX peaks (up to the top 75,000) ranked by the MAGIX enrichment coefficient and fraction of overlapping ChIP peaks in the same window

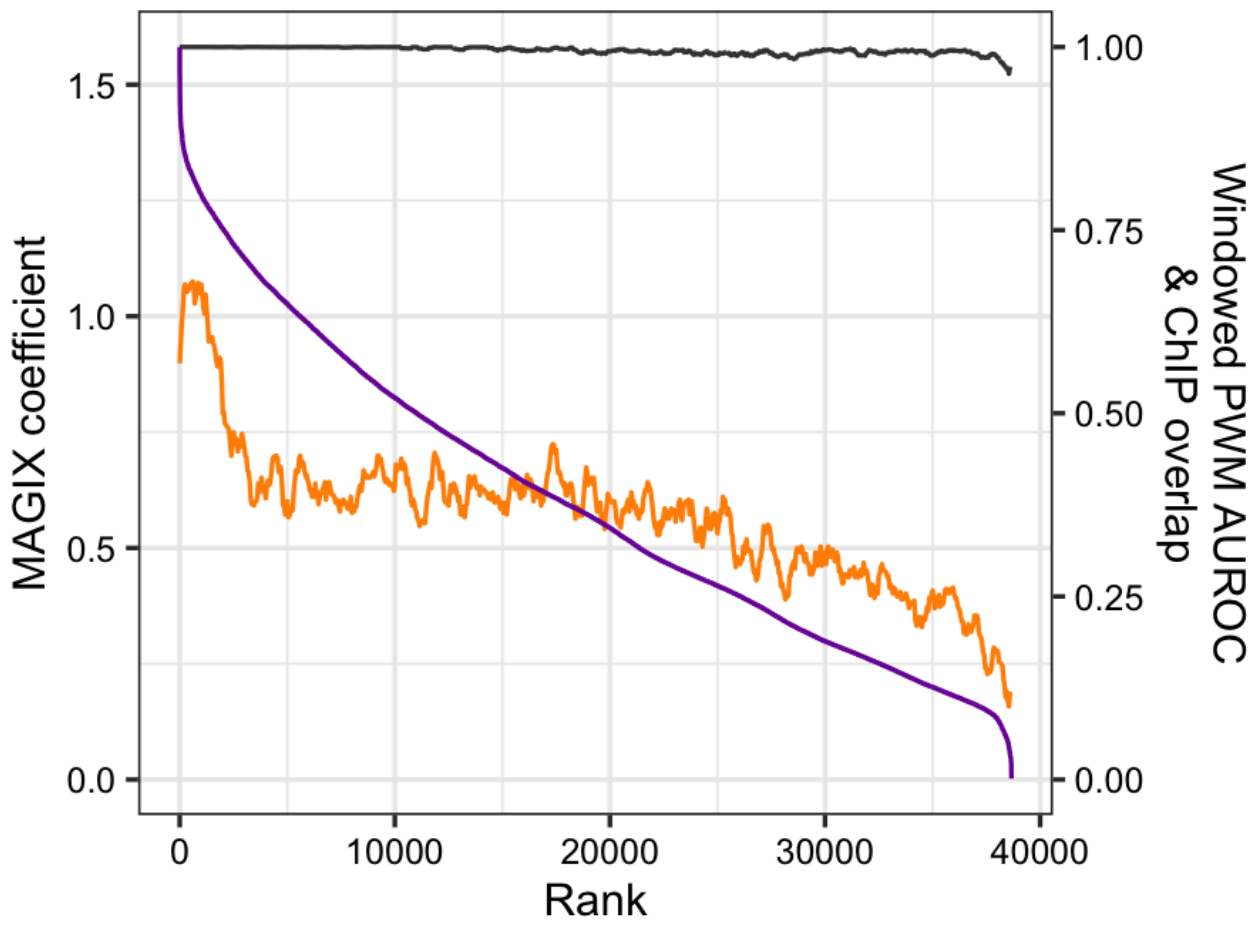

NR1H4

Top GRECO-BIT/MEX PWM: PWM001473

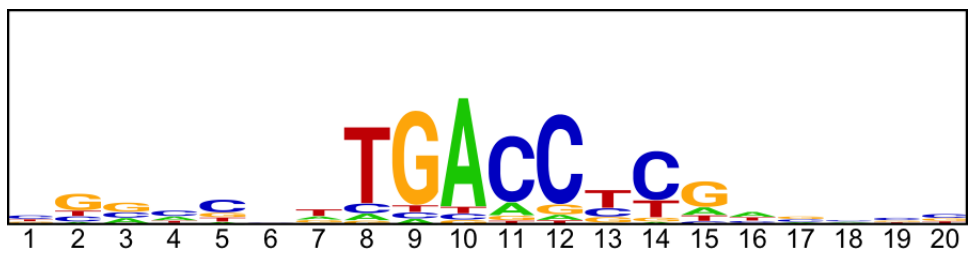

Location of top GRECO-BIT/MEX PWM in MAGIX peaks (up to the top 10,000)

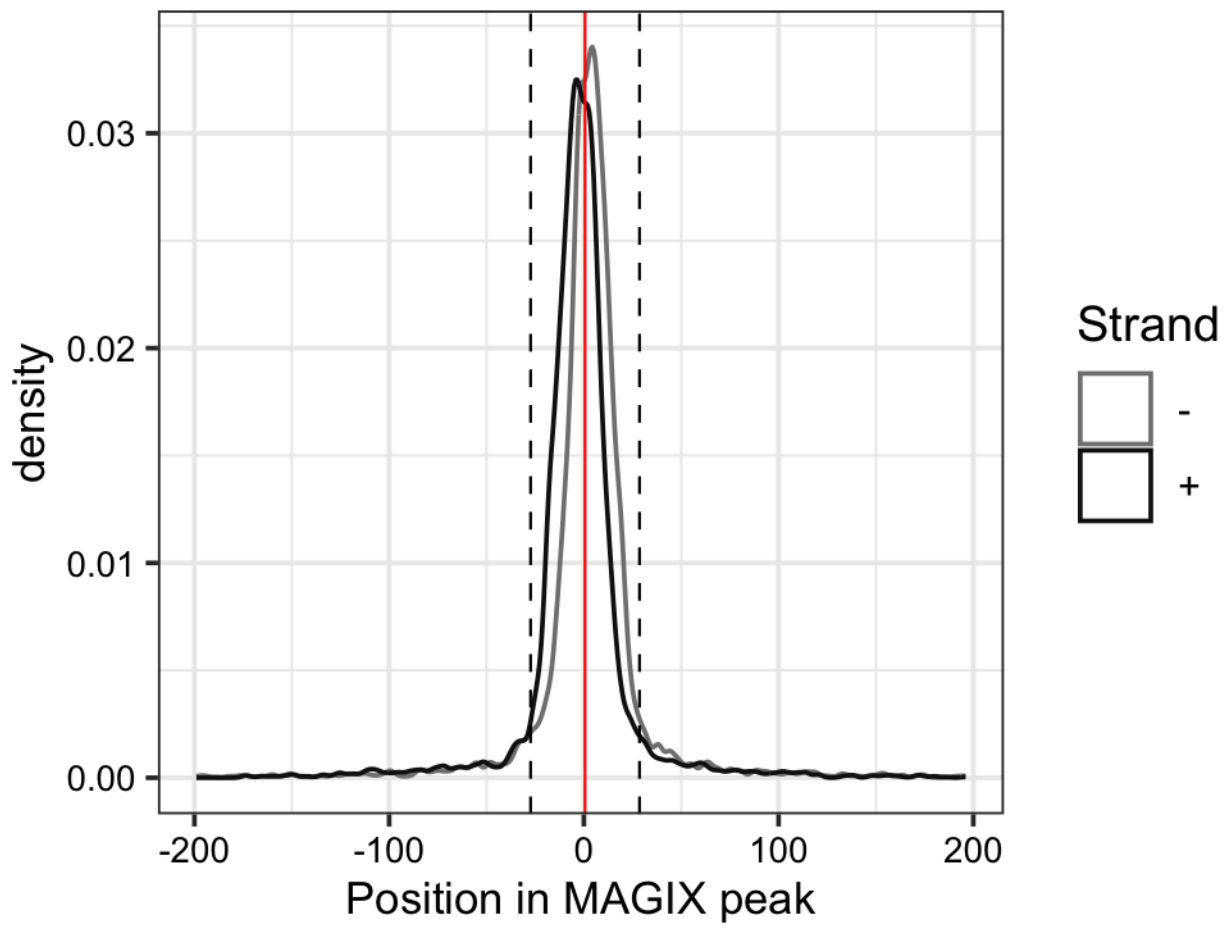

AUROC of top GRECO-BIT/MEX PWM in sliding windows (500 peaks, 50 peak step) along the MAGIX peaks (up to the top 75,000) ranked by the MAGIX enrichment coefficient and fraction of overlapping ChIP peaks in the same window

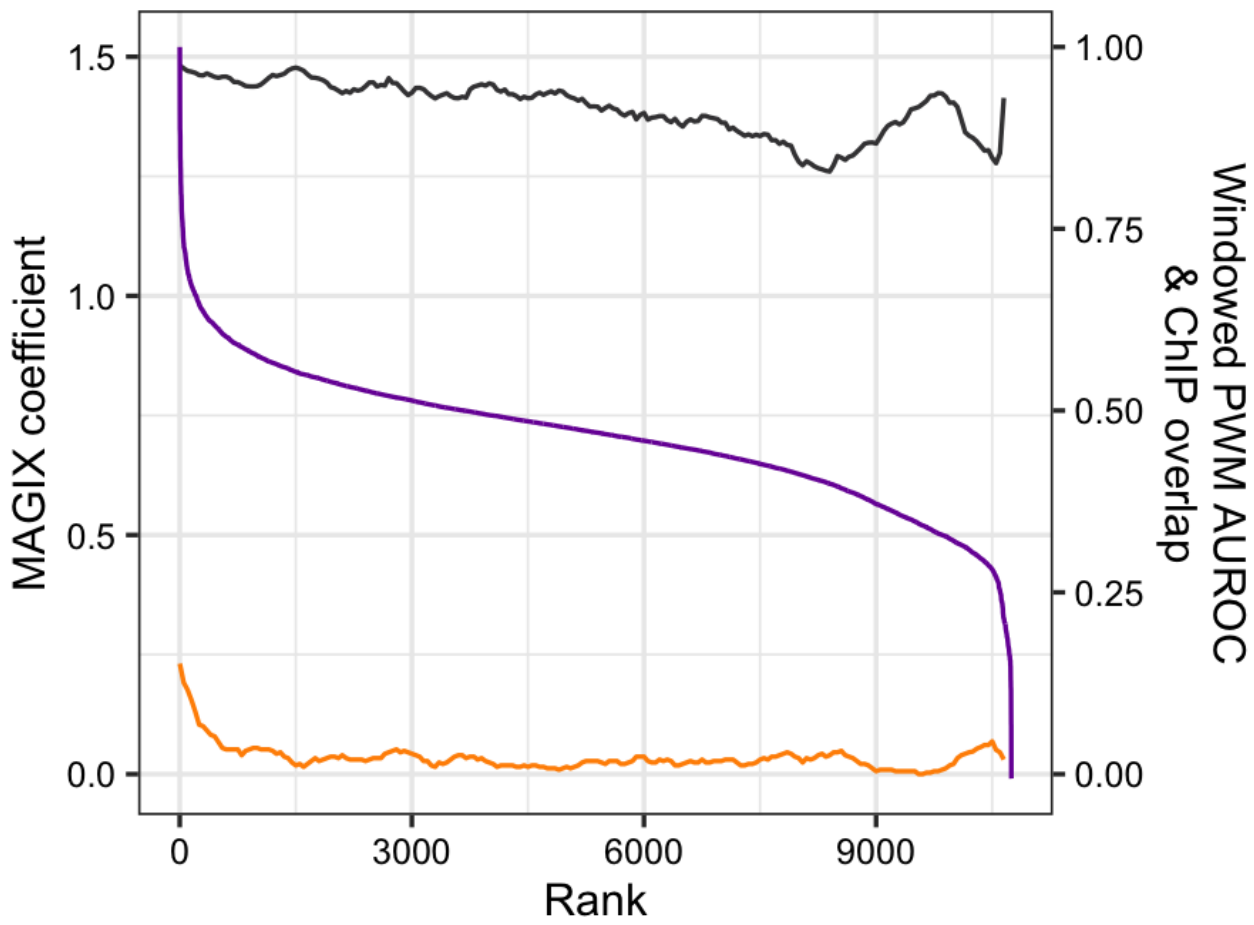

PAX7

Top GRECO-BIT/MEX PWM: PWM001530

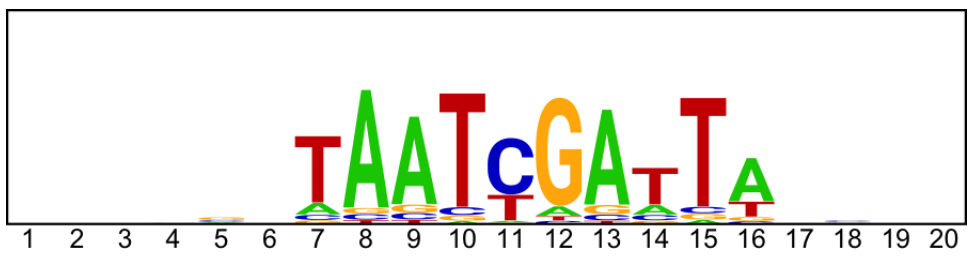

Location of top GRECO-BIT/MEX PWM in MAGIX peaks (up to the top 10,000)

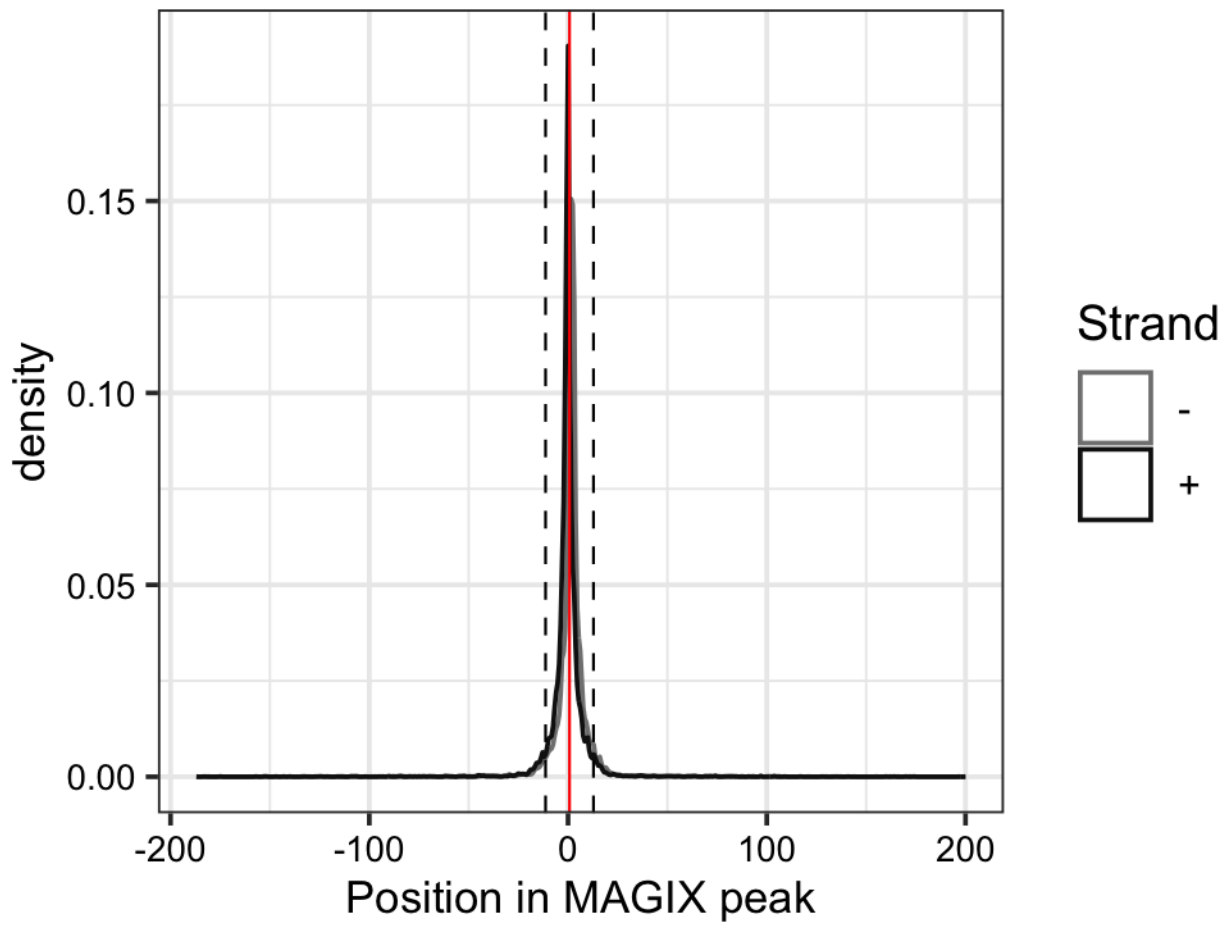

AUROC of top GRECO-BIT/MEX PWM in sliding windows (500 peaks, 50 peak step) along the MAGIX peaks (up to the top 75,000) ranked by the MAGIX enrichment coefficient and fraction of overlapping ChIP peaks in the same window

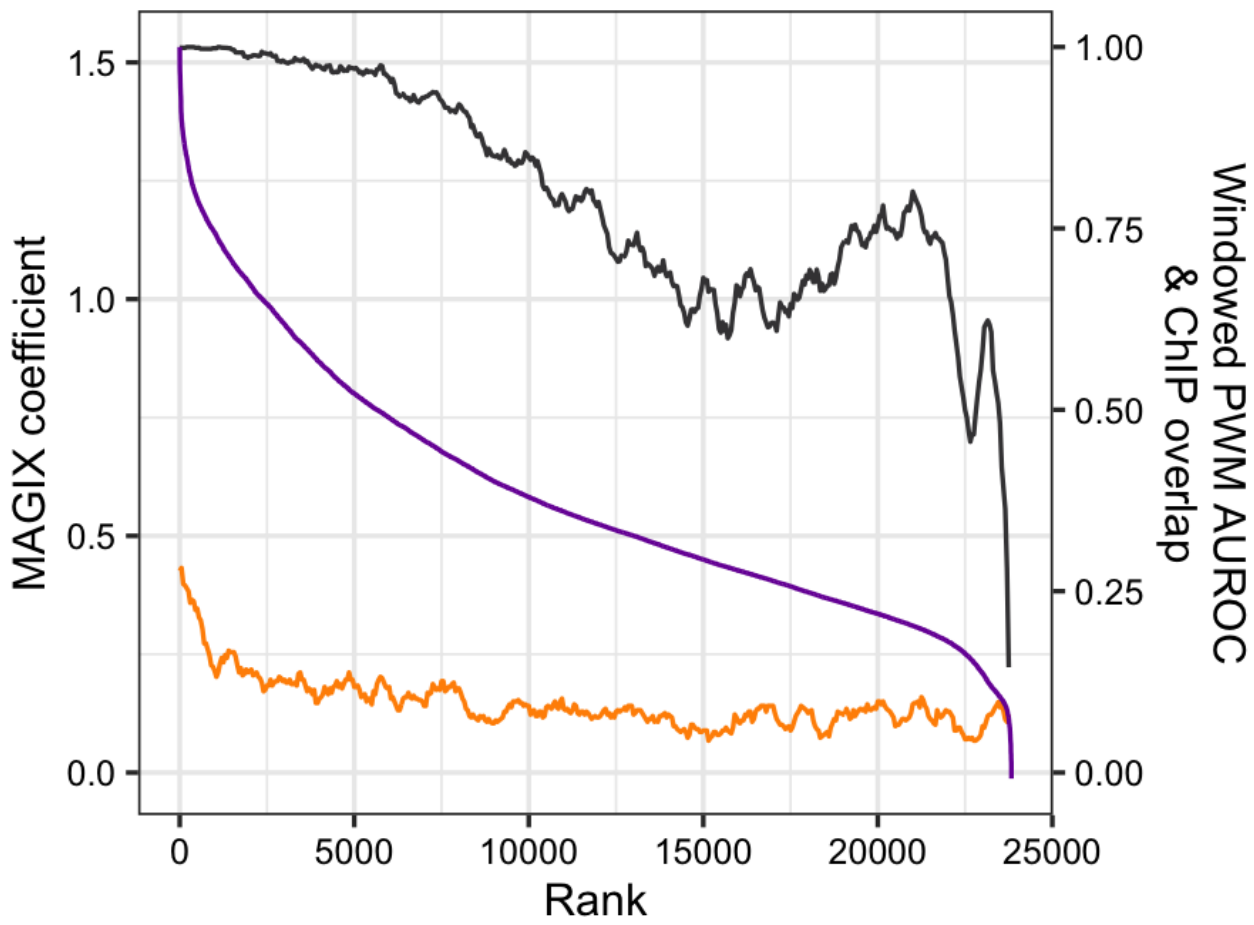

POU5F1

Top GRECO-BIT/MEX PWM: PWM001566

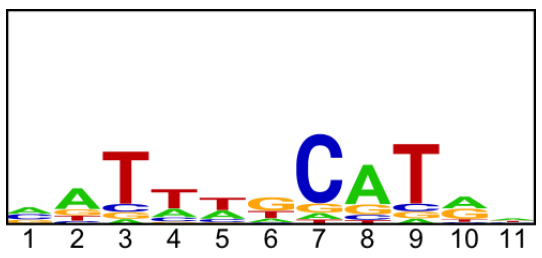

Location of top GRECO-BIT/MEX PWM in MAGIX peaks (up to the top 10,000)

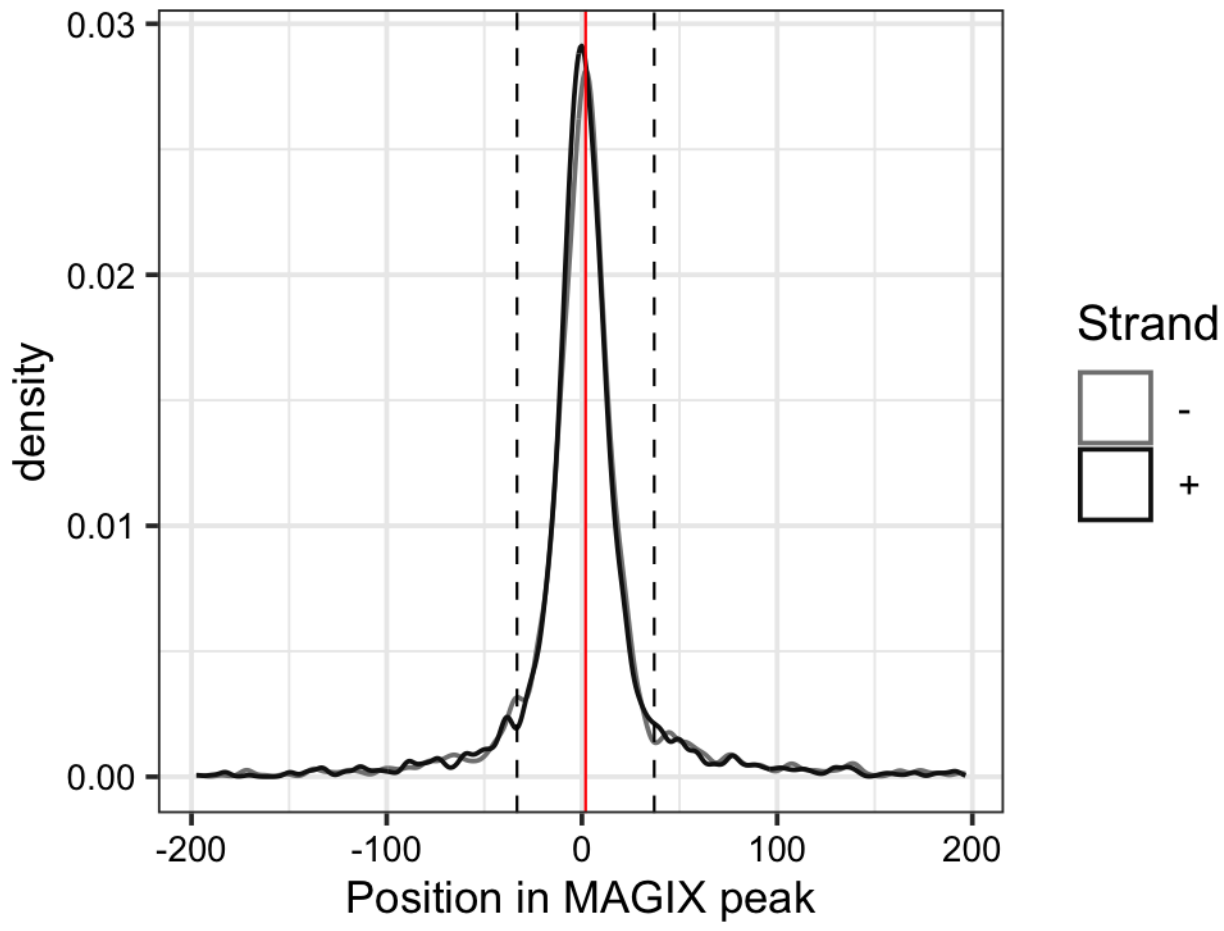

PRDM10

Top GRECO-BIT/MEX PWM: PWM001598

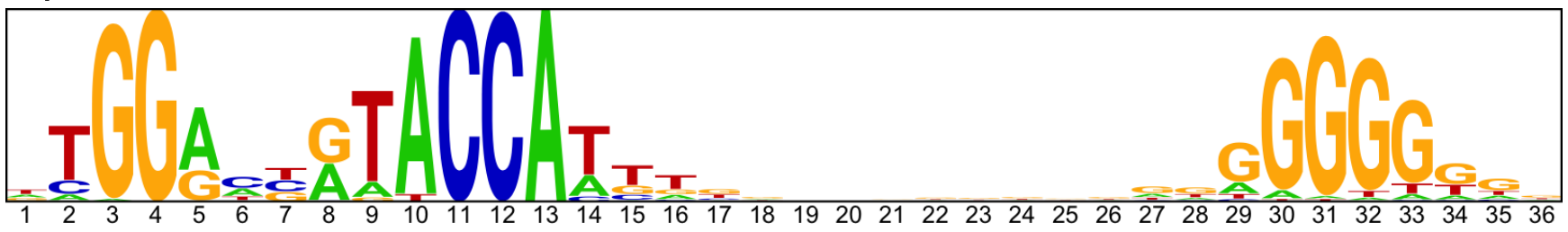

Location of top GRECO-BIT/MEX PWM in MAGIX peaks (up to the top 10,000)

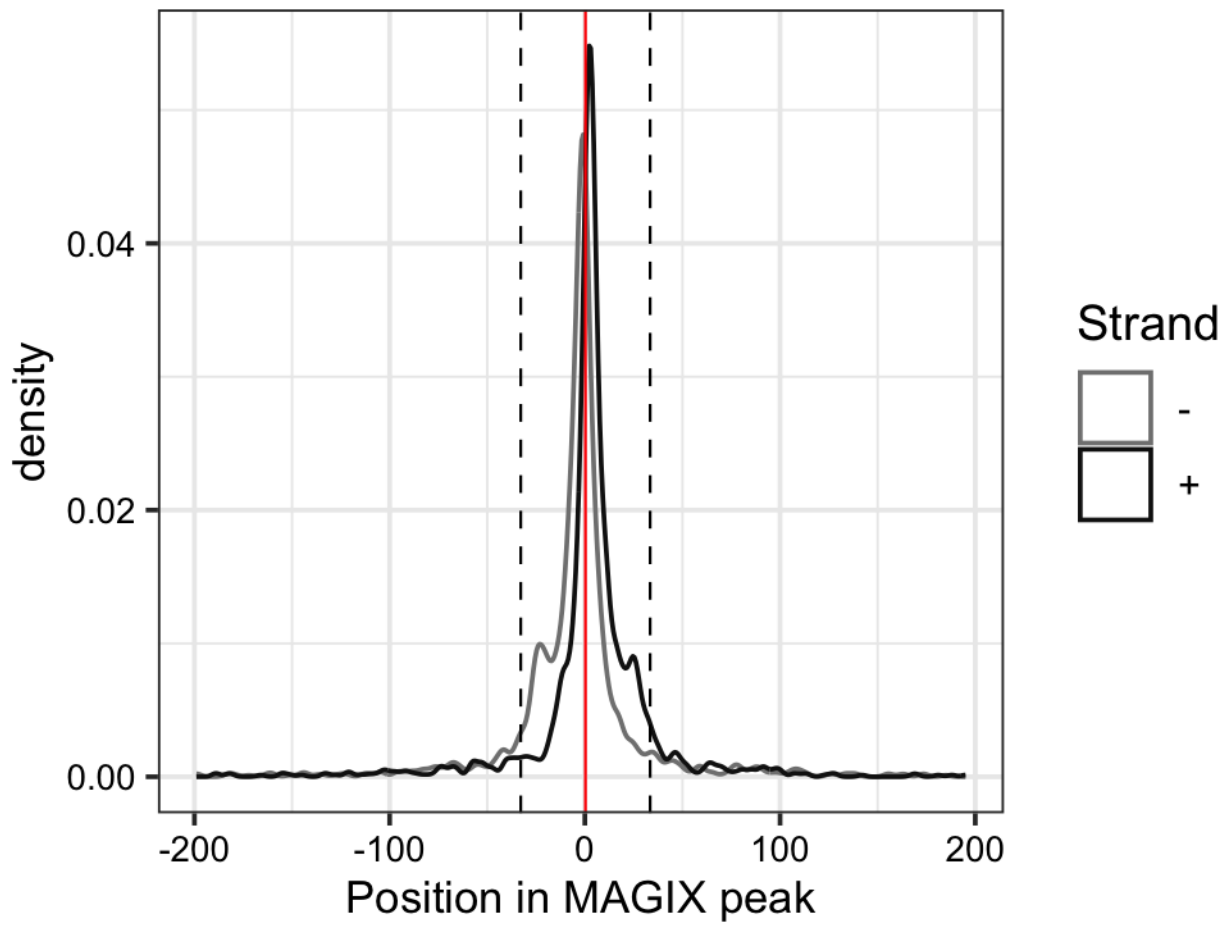

AUROC of top GRECO-BIT/MEX PWM in sliding windows (500 peaks, 50 peak step) along the MAGIX peaks (up to the top 75,000) ranked by the MAGIX enrichment coefficient and fraction of overlapping ChIP peaks in the same window

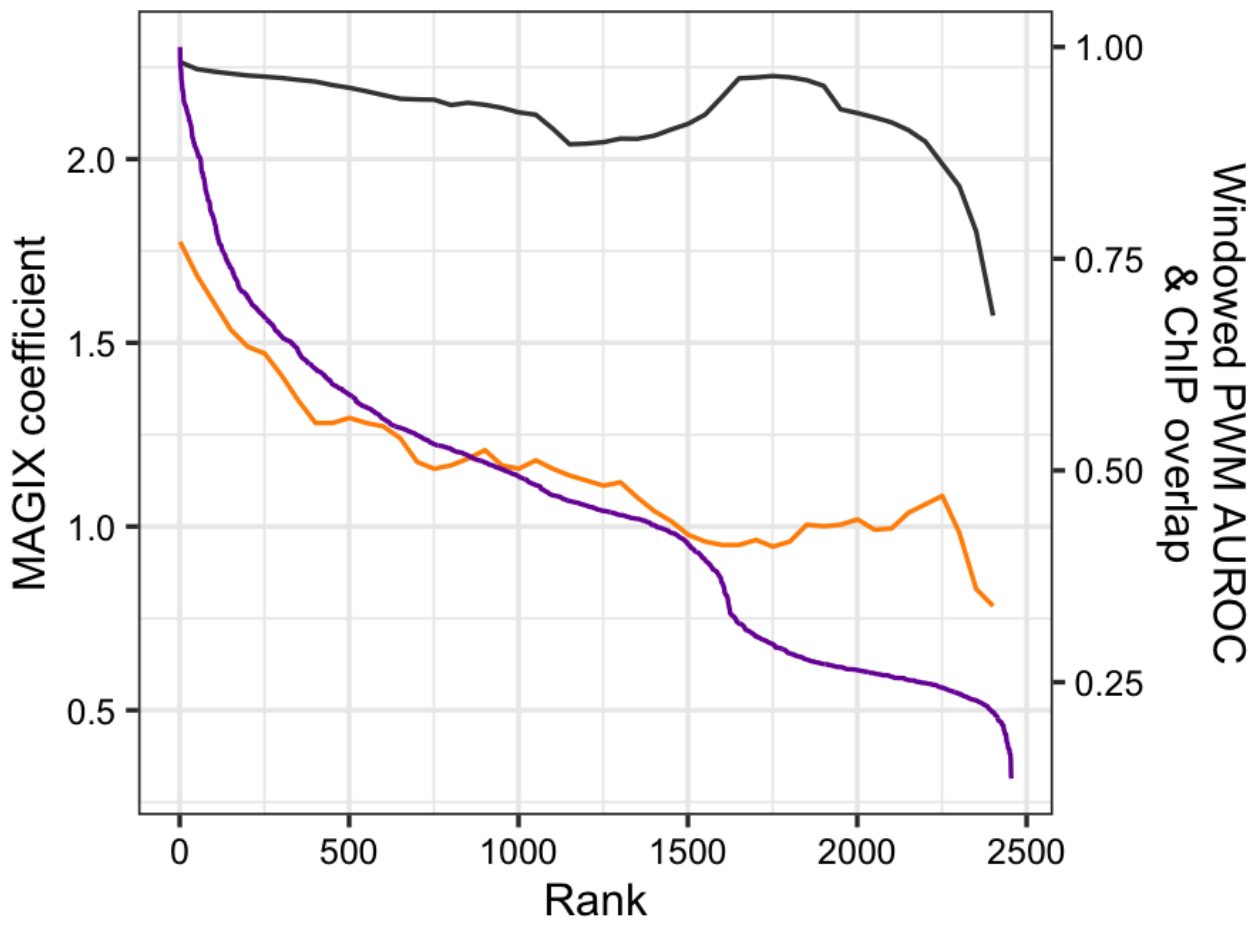

PRDM13

Top GRECO-BIT/MEX PWM: PWM001641

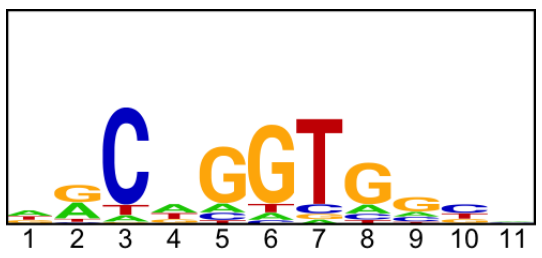

Location of top GRECO-BIT/MEX PWM in MAGIX peaks (up to the top 10,000)

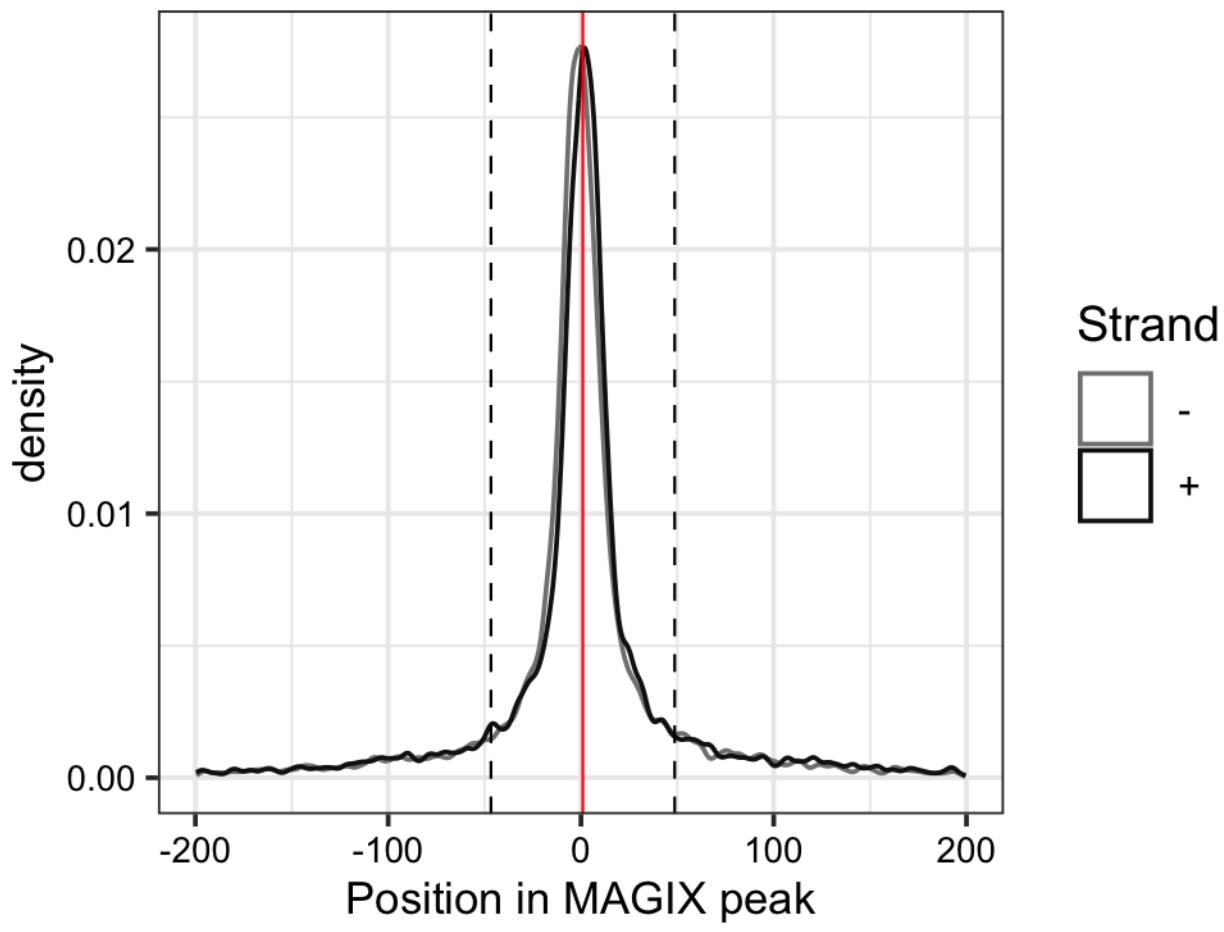

AUROC of top GRECO-BIT/MEX PWM in sliding windows (500 peaks, 50 peak step) along the MAGIX peaks (up to the top 75,000) ranked by the MAGIX enrichment coefficient and fraction of overlapping ChIP peaks in the same window

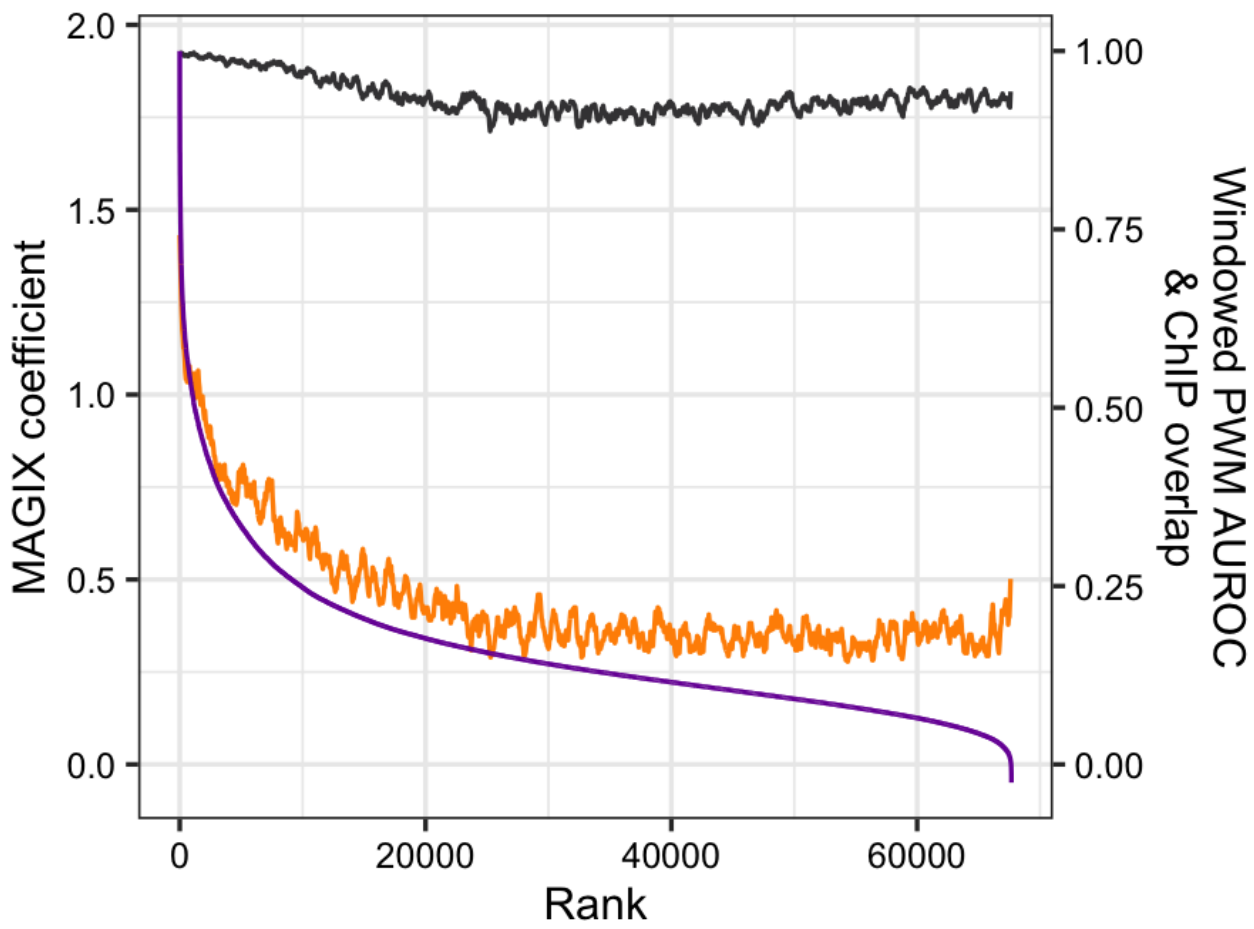

PRDM5

Top GRECO-BIT/MEX PWM: PWM001672

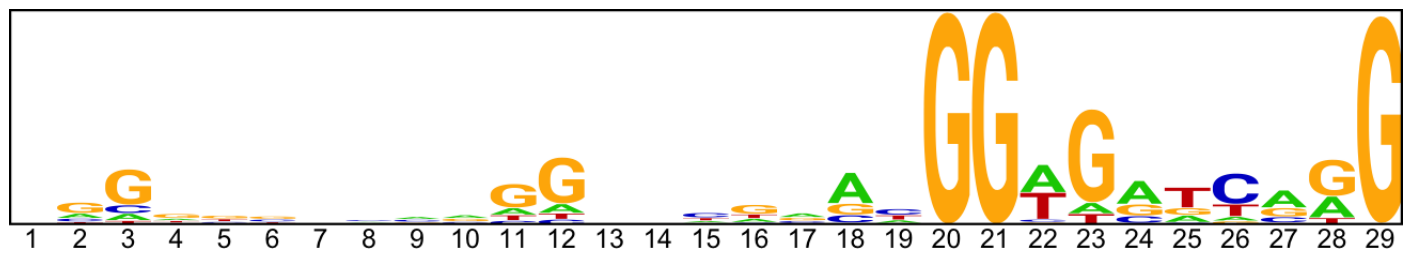

Location of top GRECO-BIT/MEX PWM in MAGIX peaks (up to the top 10,000)

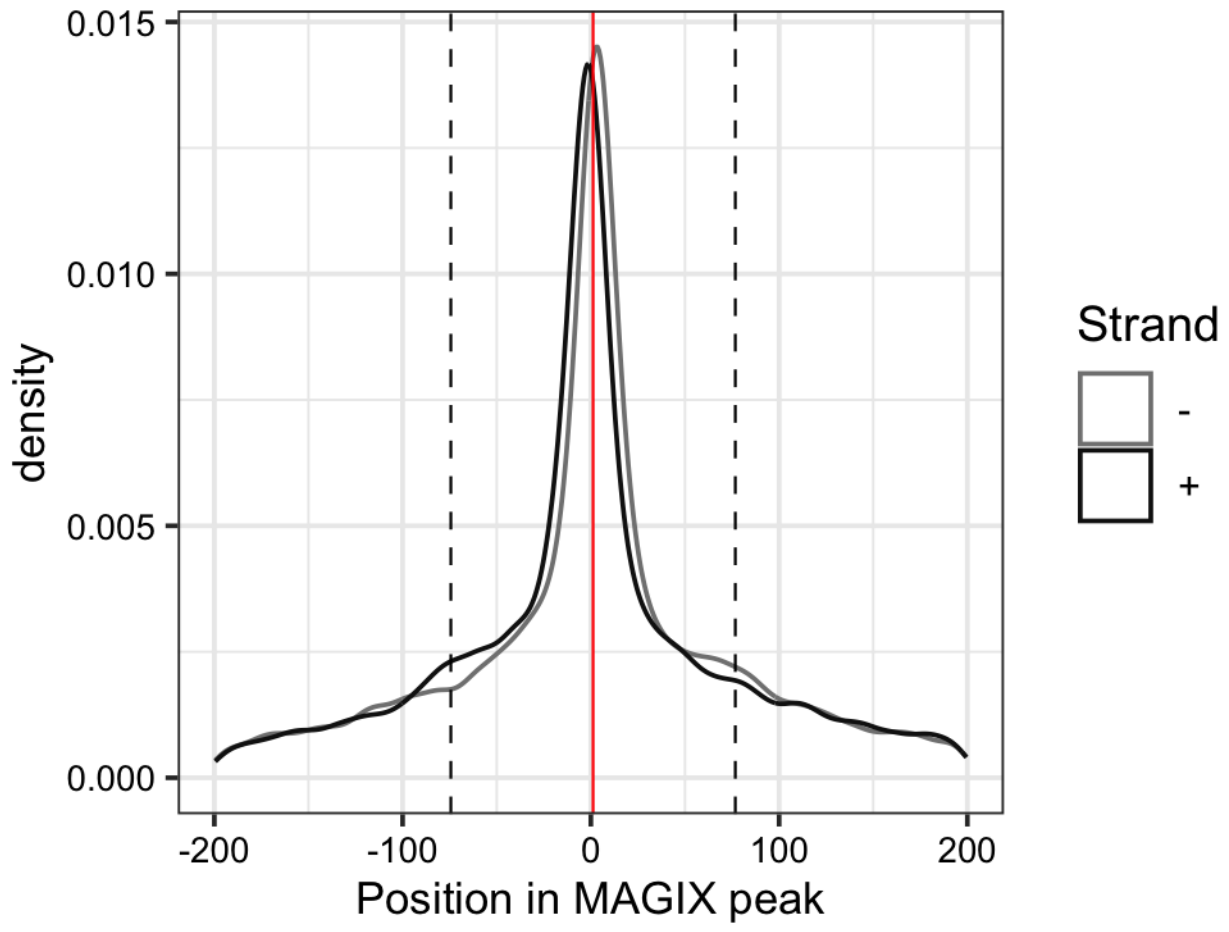

AUROC of top GRECO-BIT/MEX PWM in sliding windows (500 peaks, 50 peak step) along the MAGIX peaks (up to the top 75,000) ranked by the MAGIX enrichment coefficient and fraction of overlapping ChIP peaks in the same window

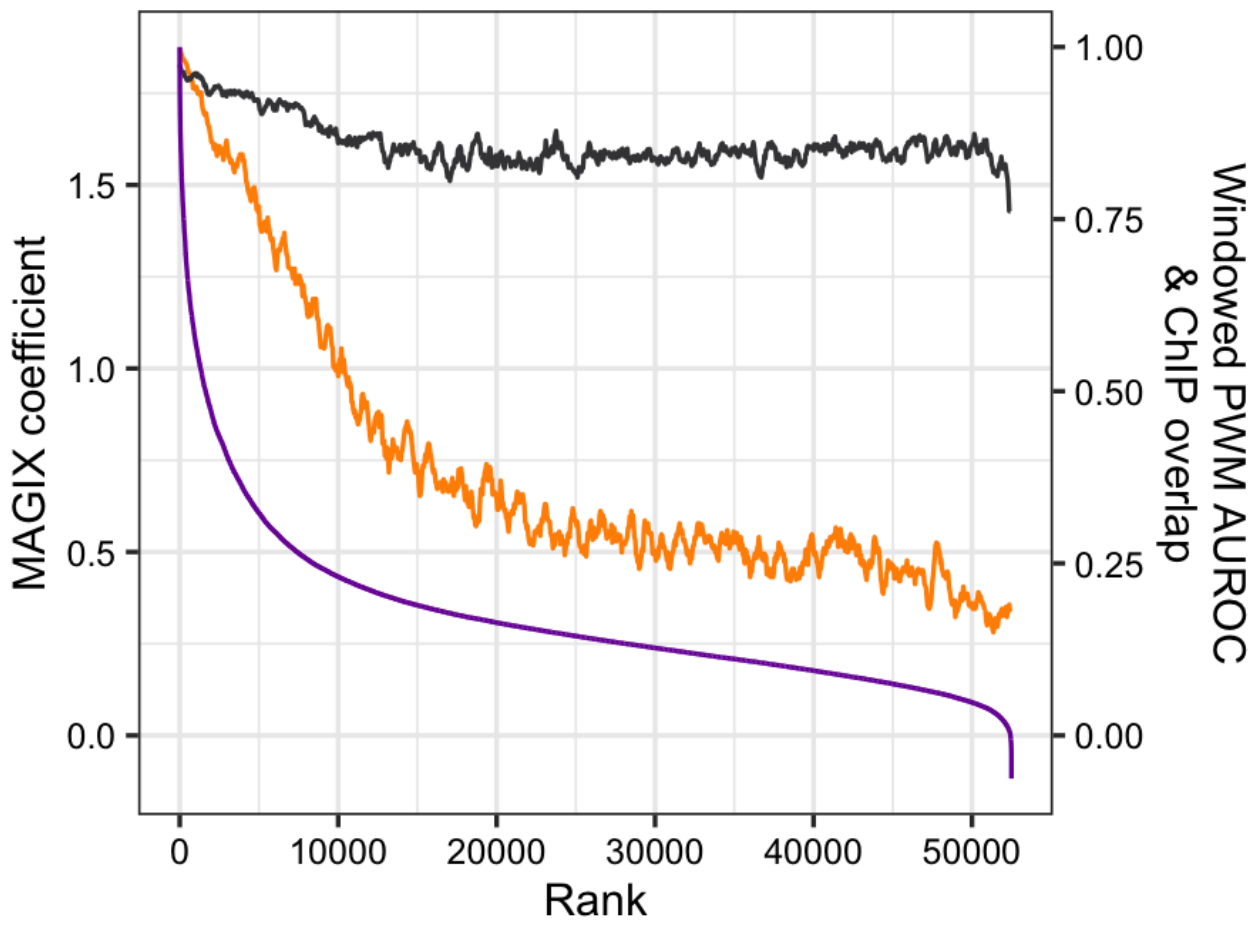

RARA

Top GRECO-BIT/MEX PWM: PWM001684

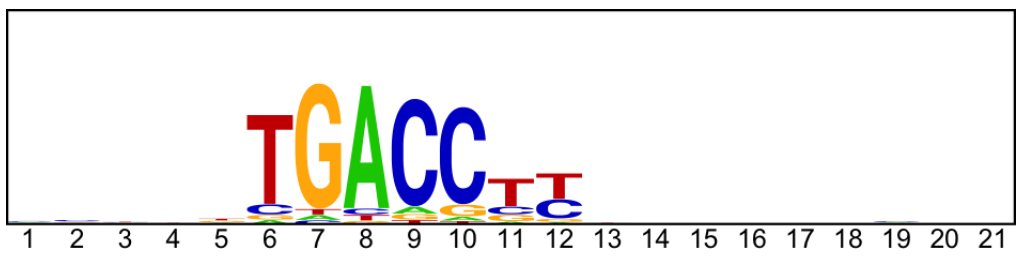

Location of top GRECO-BIT/MEX PWM in MAGIX peaks (up to the top 10,000)

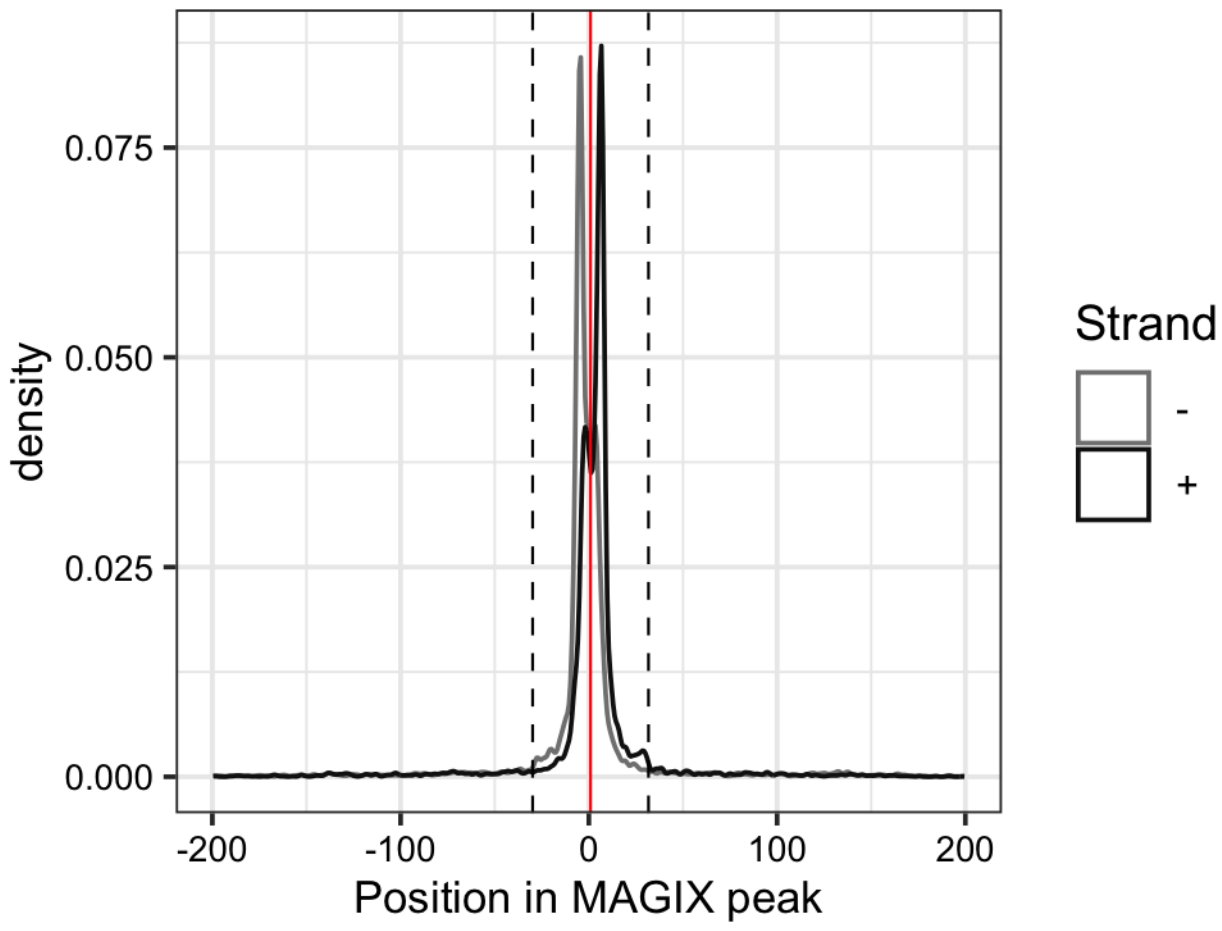

RFX5

Top GRECO-BIT/MEX PWM: PWM001724

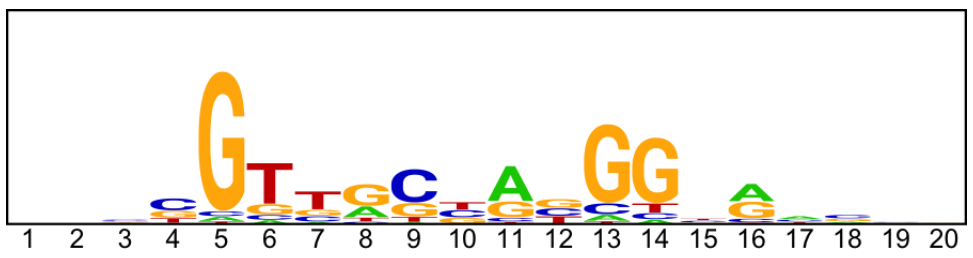

Location of top GRECO-BIT/MEX PWM in MAGIX peaks (up to the top 10,000)

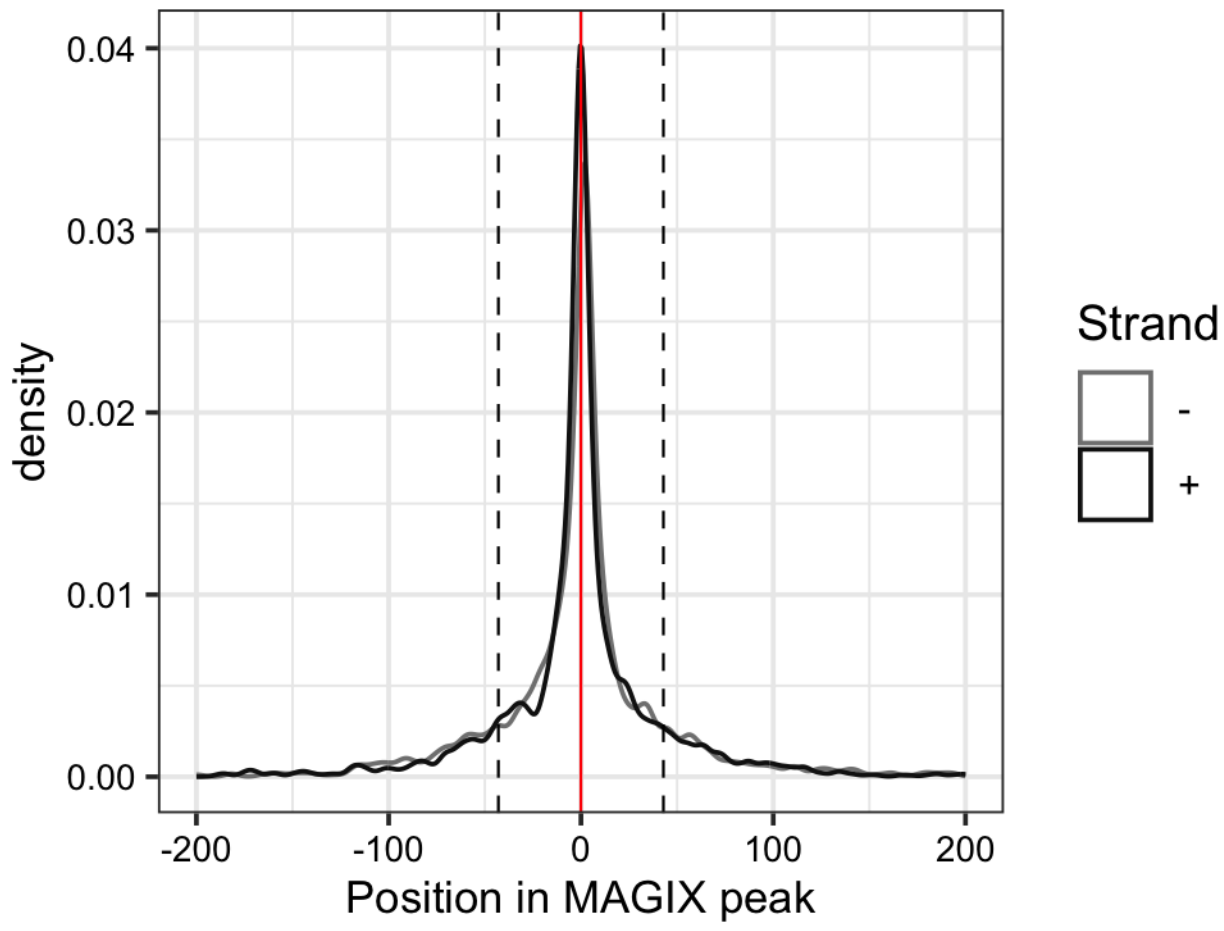

AUROC of top GRECO-BIT/MEX PWM in sliding windows (500 peaks, 50 peak step) along the MAGIX peaks (up to the top 75,000) ranked by the MAGIX enrichment coefficient and fraction of overlapping ChIP peaks in the same window

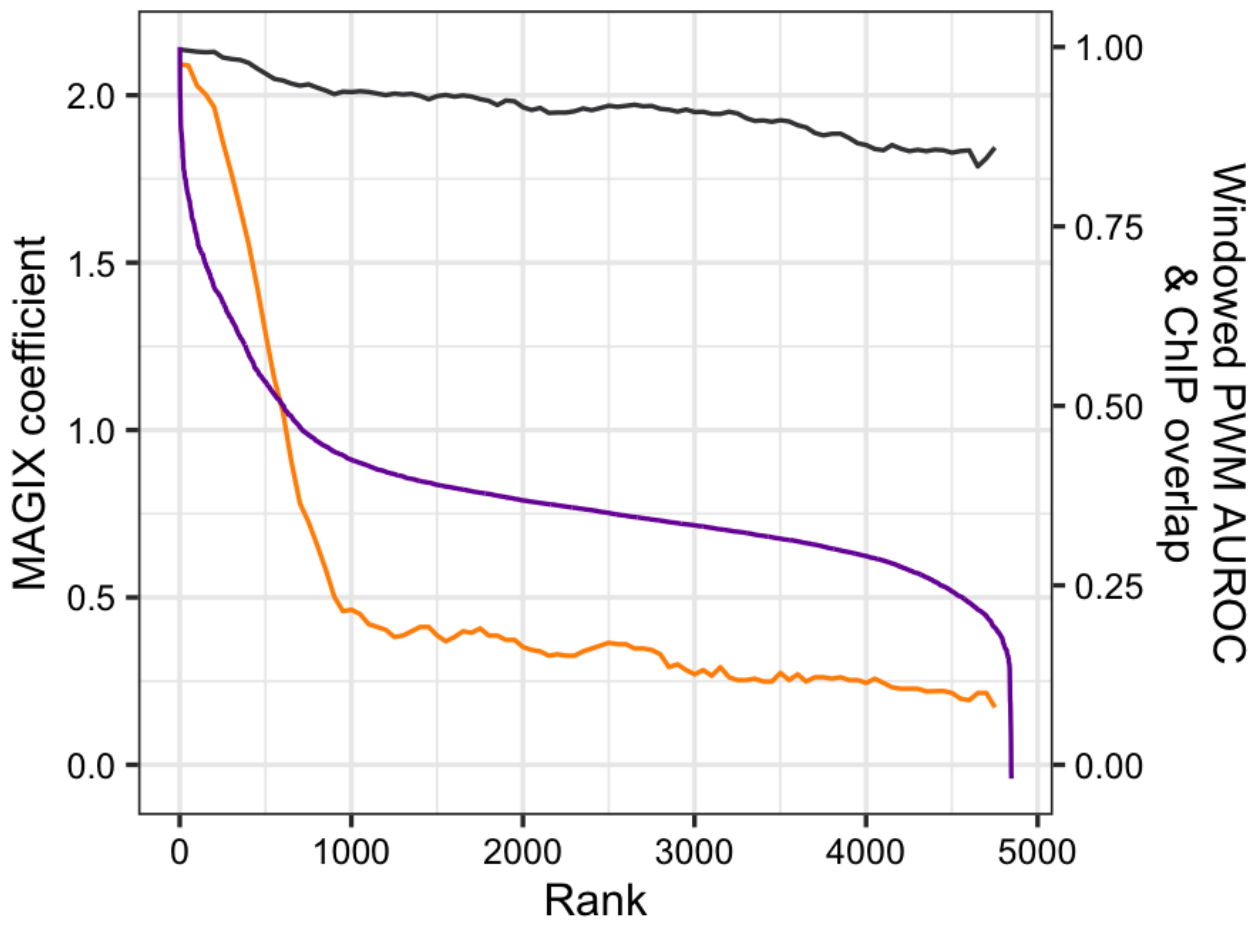

RLF.DBD2

Top GRECO-BIT/MEX PWM: PWM001771

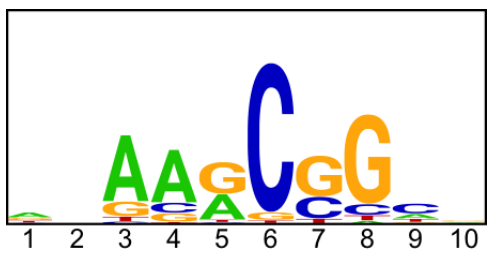

Location of top GRECO-BIT/MEX PWM in MAGIX peaks (up to the top 10,000)

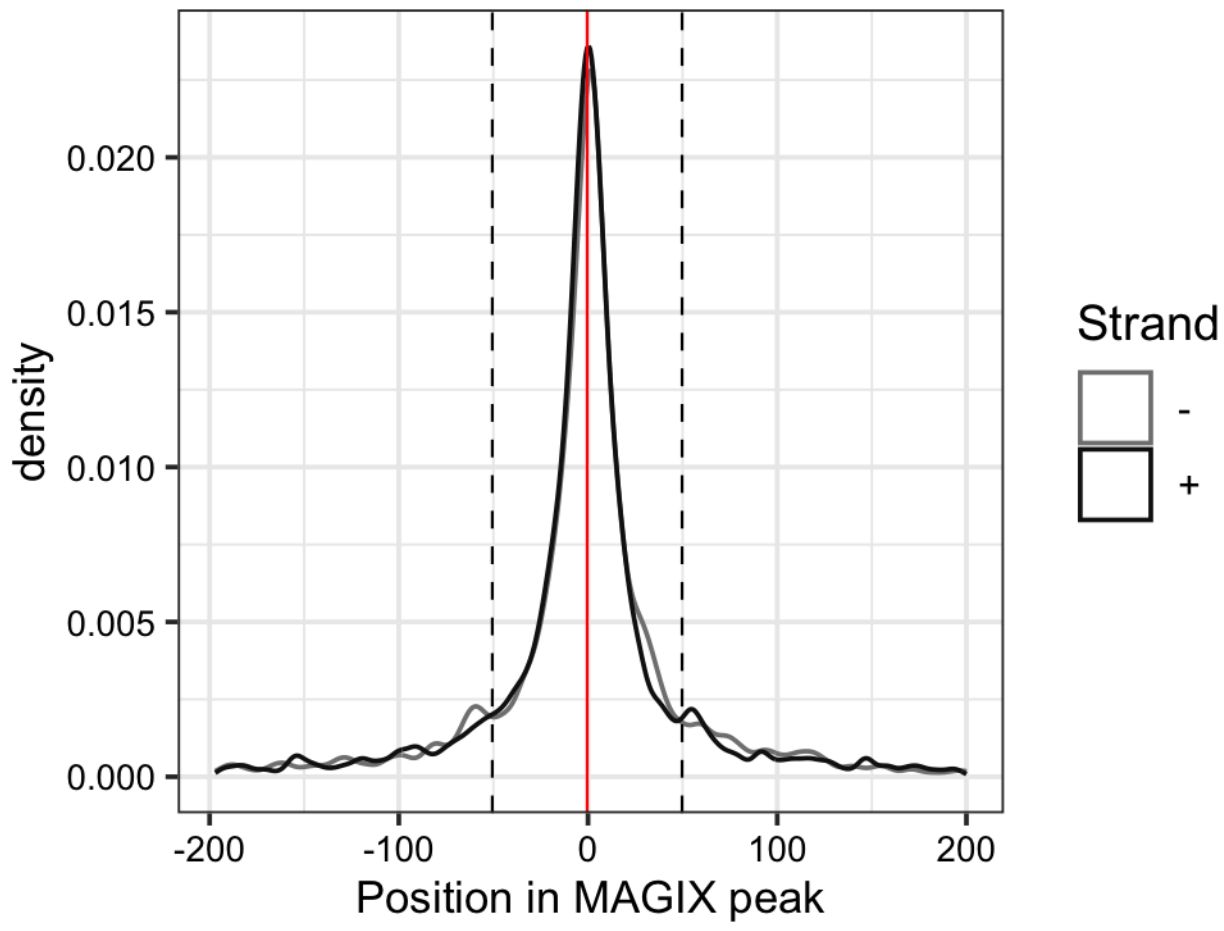

RORB

Top GRECO-BIT/MEX PWM: PWM001817

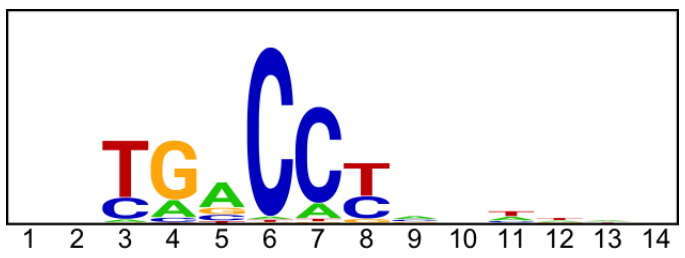

Location of top GRECO-BIT/MEX PWM in MAGIX peaks (up to the top 10,000)

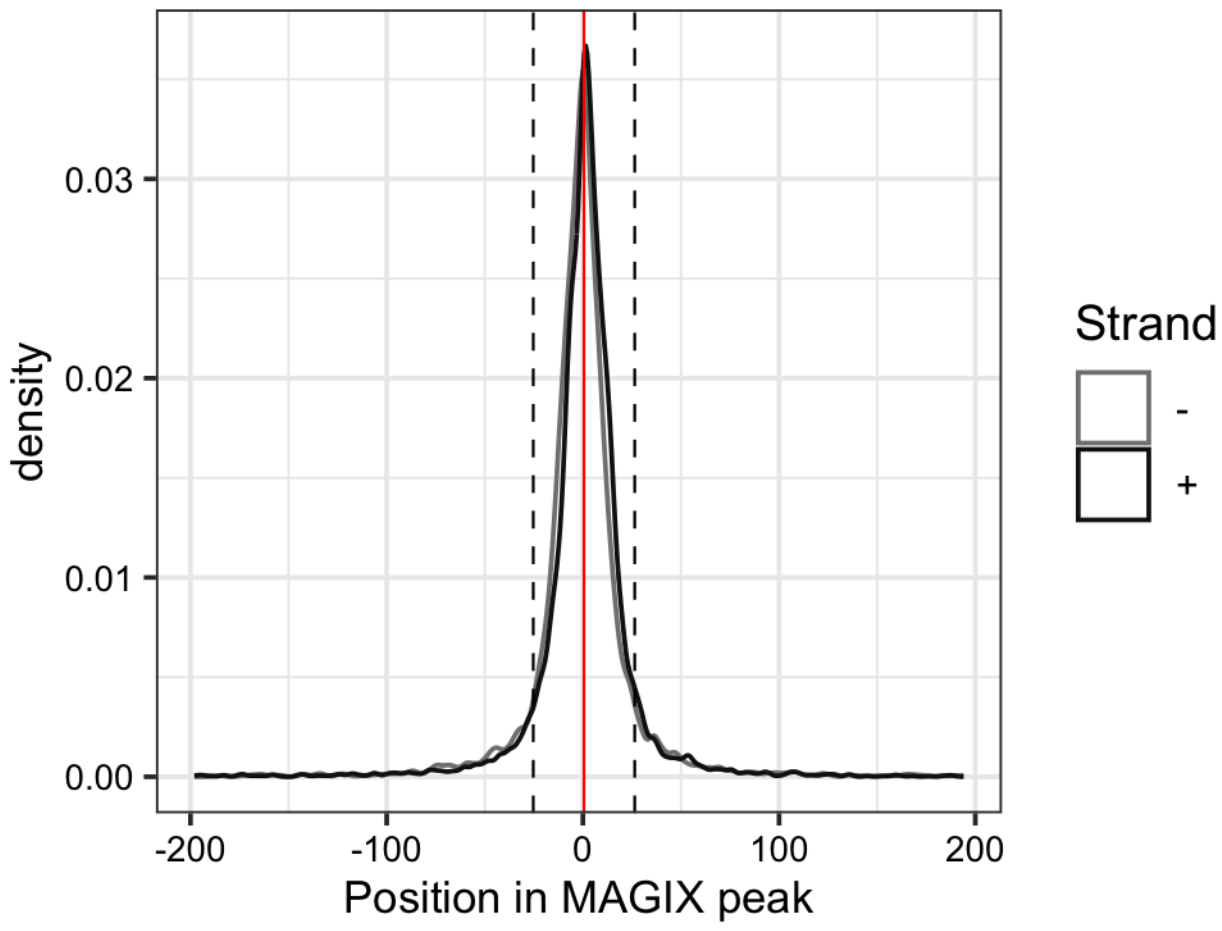

AUROC of top GRECO-BIT/MEX PWM in sliding windows (500 peaks, 50 peak step) along the MAGIX peaks (up to the top 75,000) ranked by the MAGIX enrichment coefficient and fraction of overlapping ChIP peaks in the same window

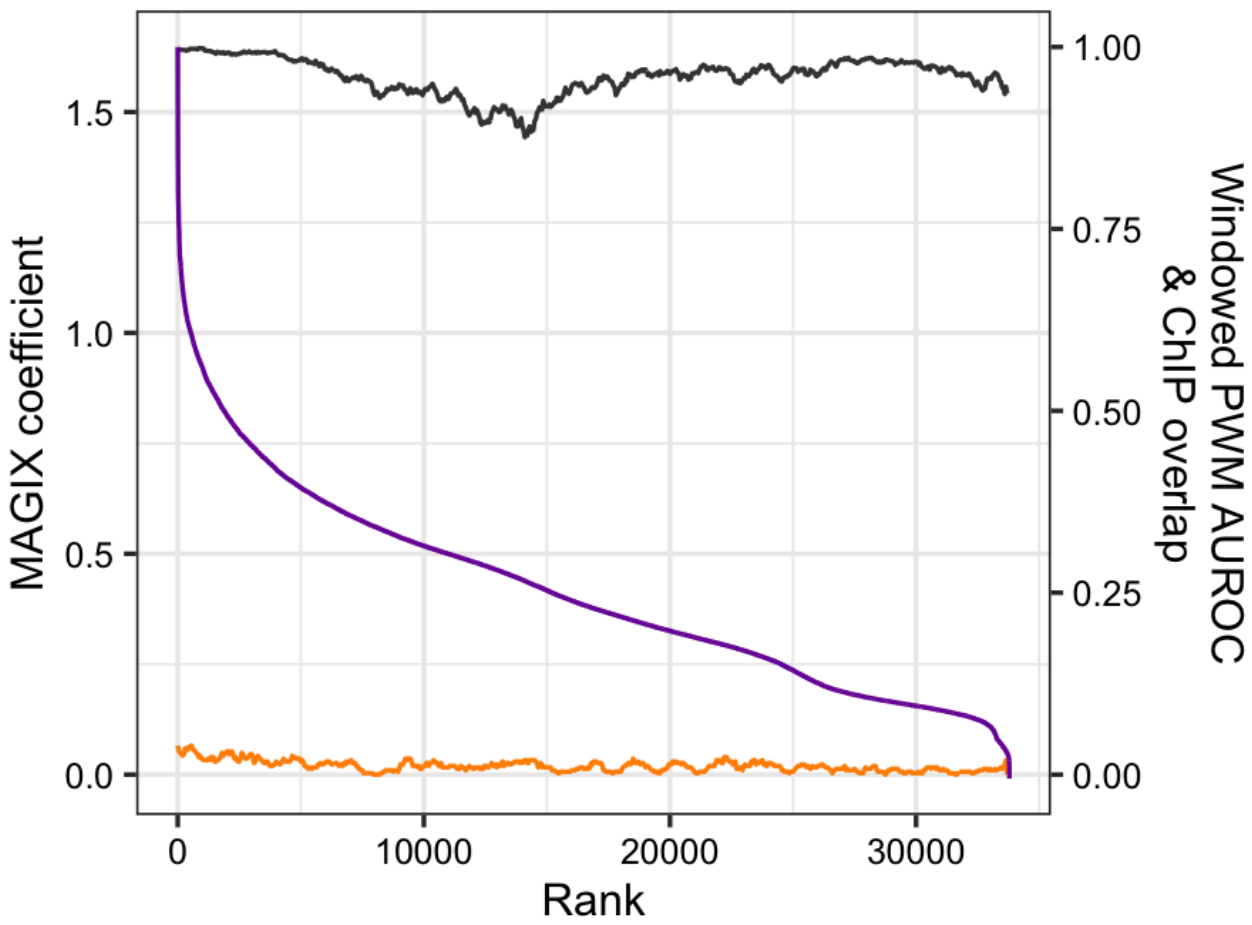

RXRA

Top GRECO-BIT/MEX PWM: PWM001833

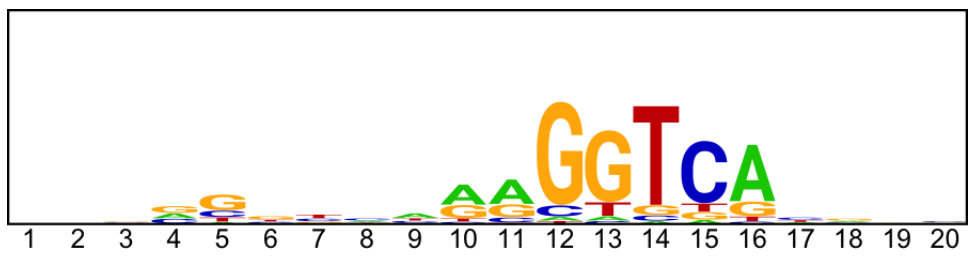

Location of top GRECO-BIT/MEX PWM in MAGIX peaks (up to the top 10,000)

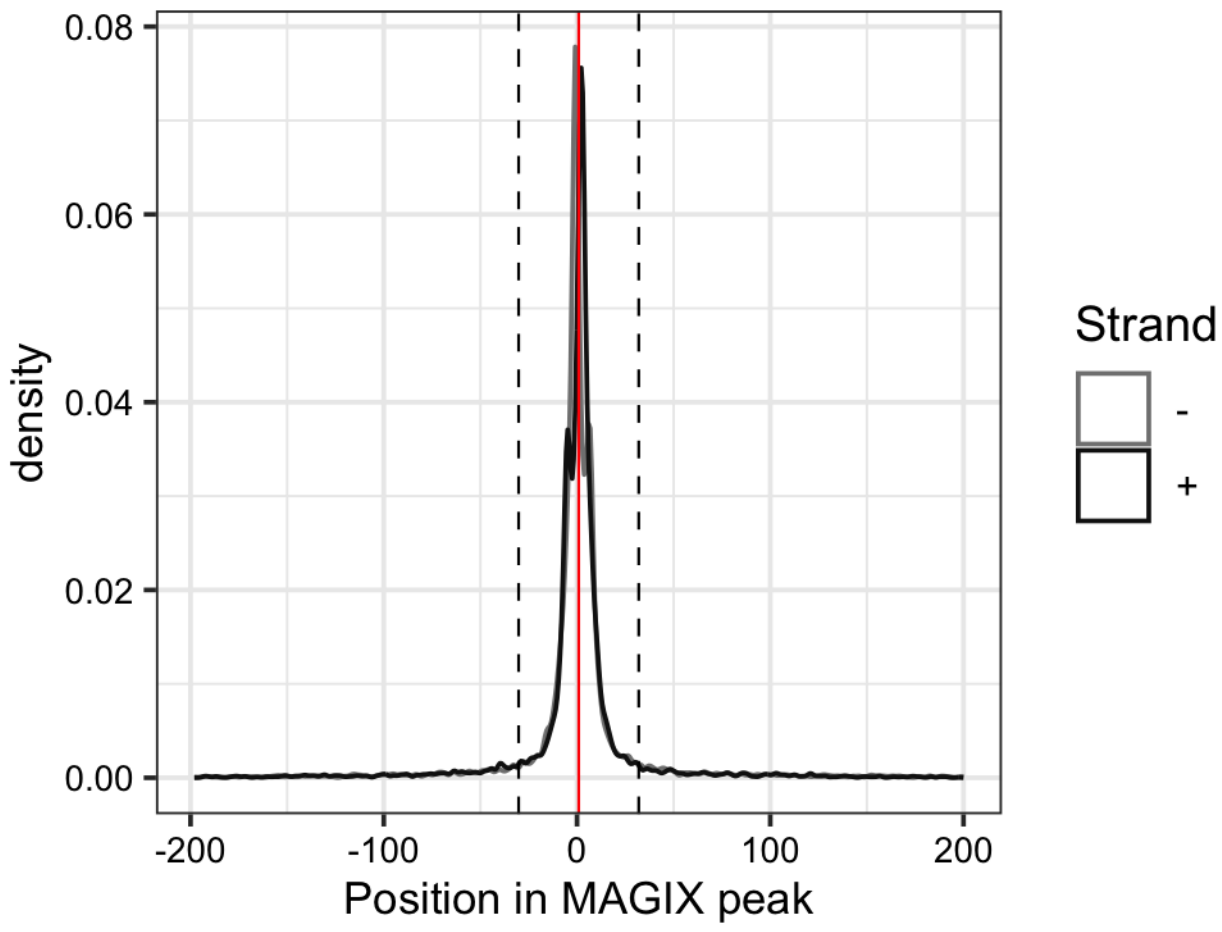

SALL3.DBD1

Top GRECO-BIT/MEX PWM: PWM070151

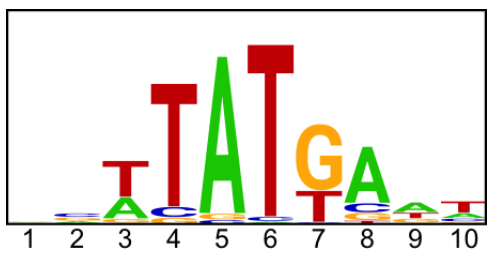

Location of top GRECO-BIT/MEX PWM in MAGIX peaks (up to the top 10,000)

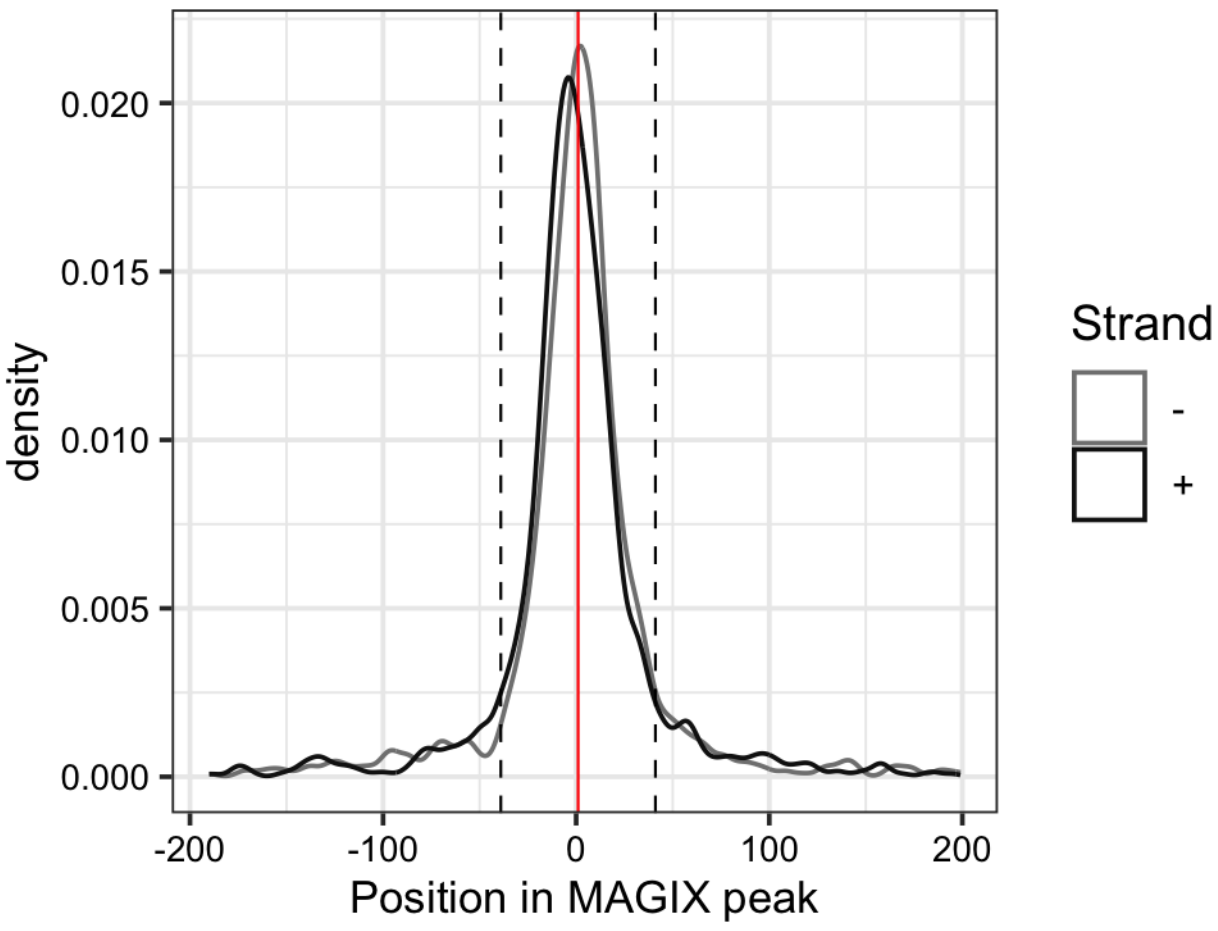

SALL3.DBD2

Top GRECO-BIT/MEX PWM: PWM001862

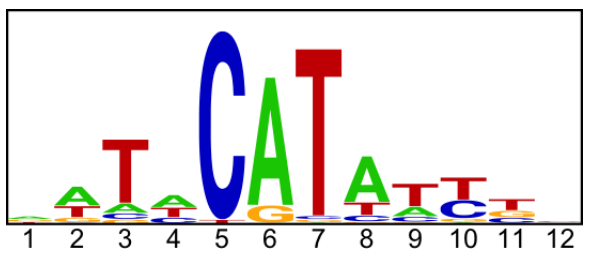

Location of top GRECO-BIT/MEX PWM in MAGIX peaks (up to the top 10,000)

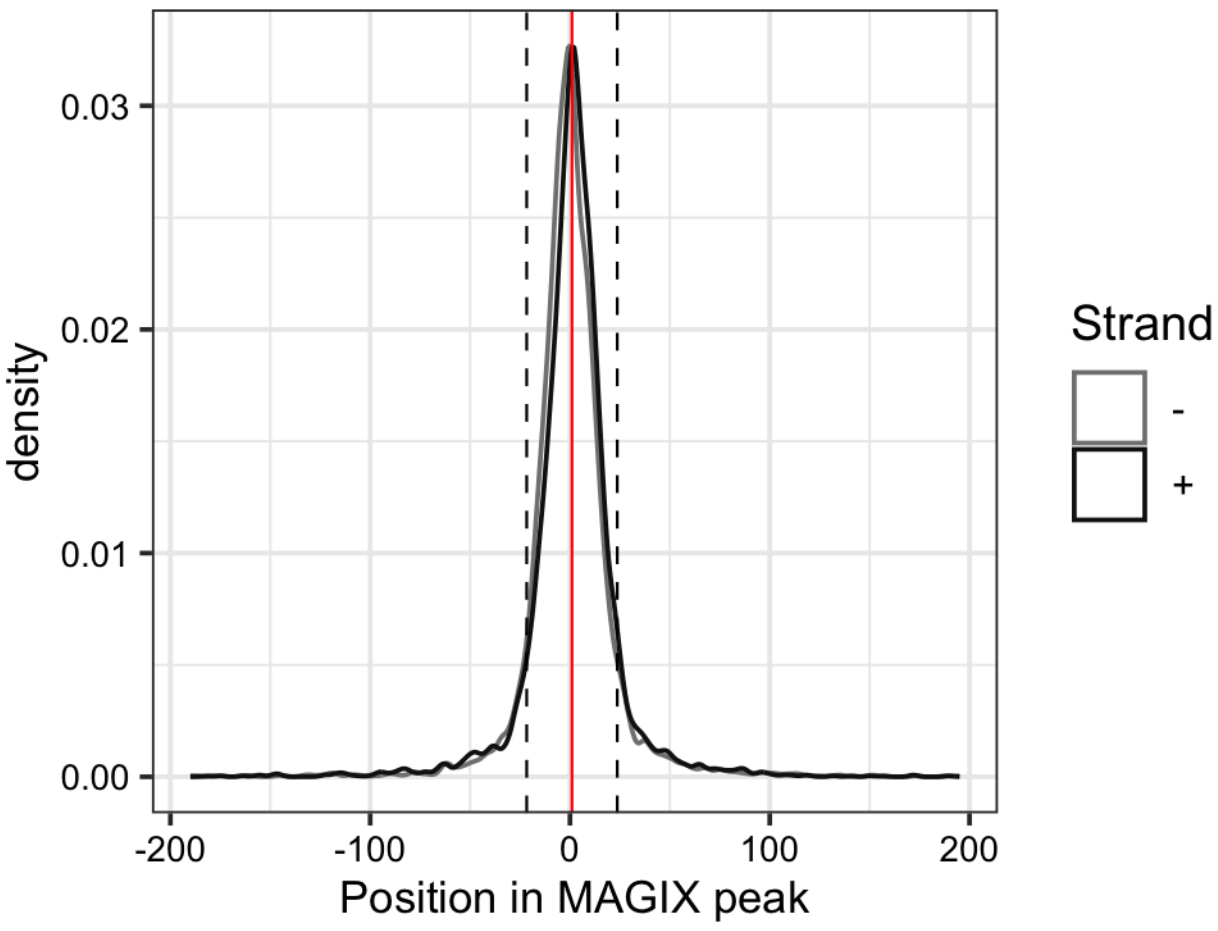

SALL3.FL

Top GRECO-BIT/MEX PWM: PWM001879

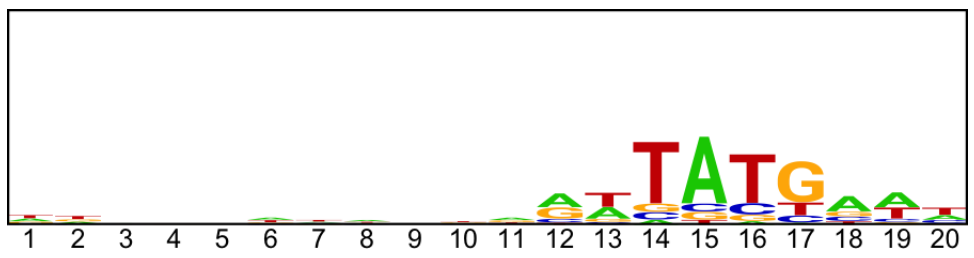

Location of top GRECO-BIT/MEX PWM in MAGIX peaks (up to the top 10,000)

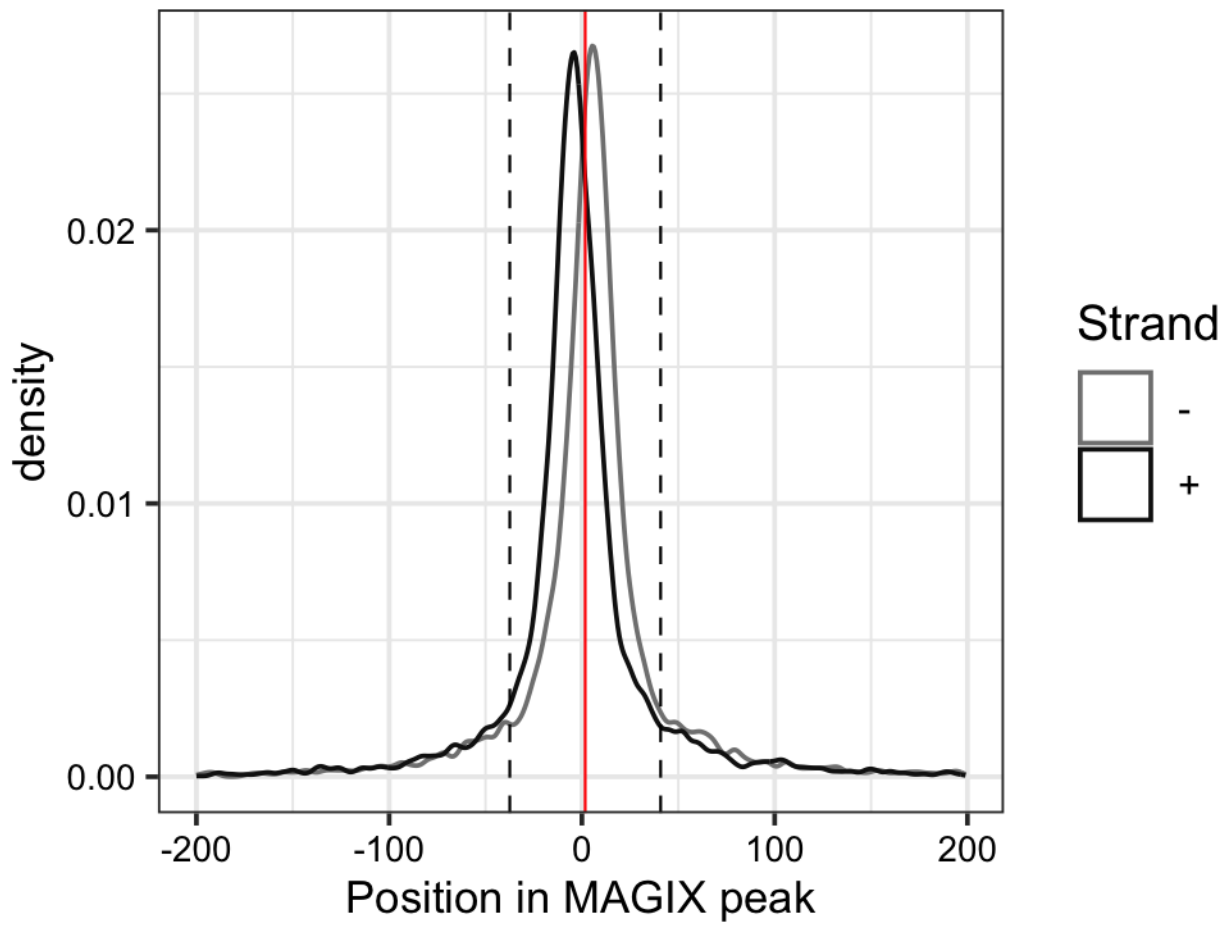

AUROC of top GRECO-BIT/MEX PWM in sliding windows (500 peaks, 50 peak step) along the MAGIX peaks (up to the top 75,000) ranked by the MAGIX enrichment coefficient and fraction of overlapping ChIP peaks in the same window

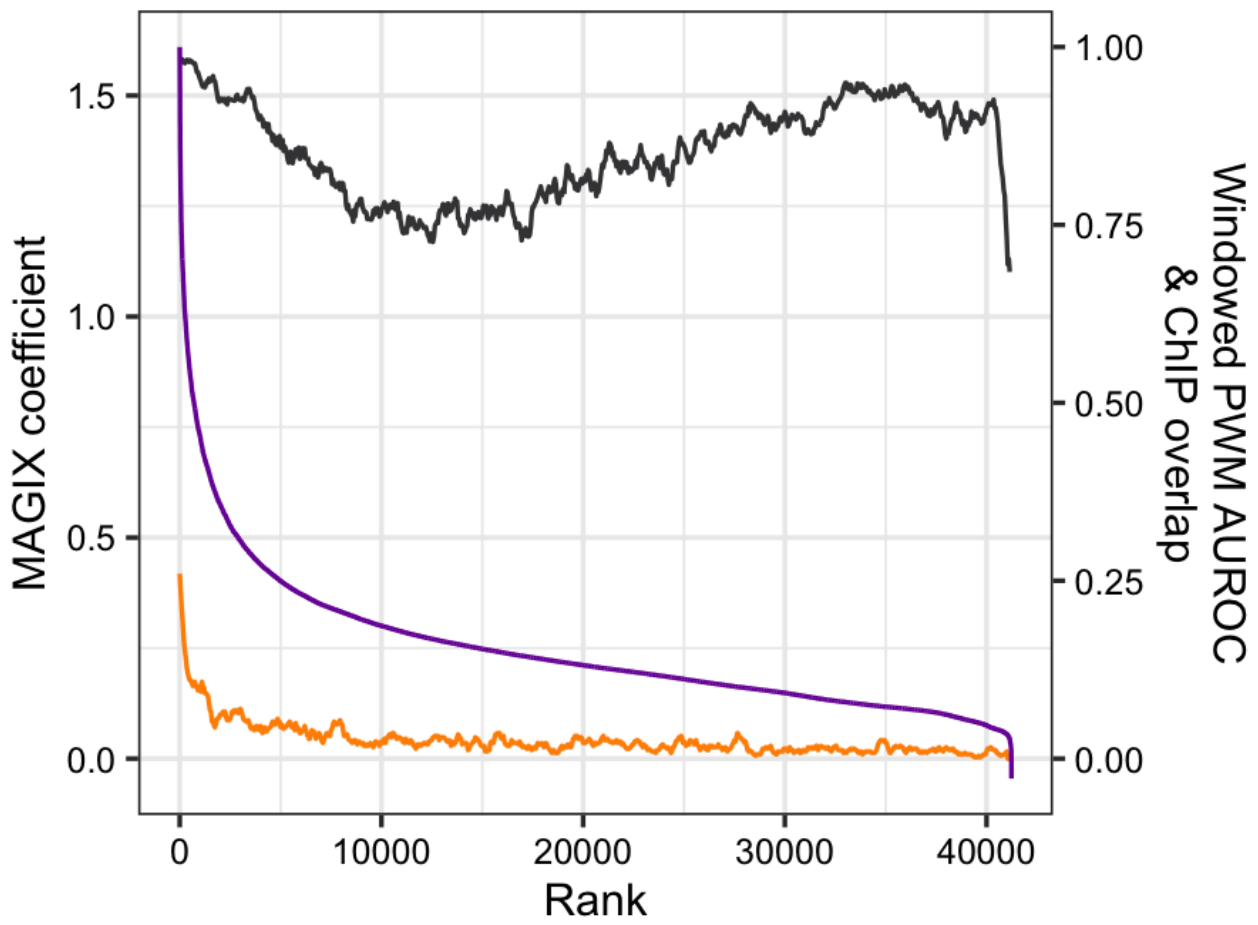

SLC2A4RG

Top GRECO-BIT/MEX PWM: PWM001919

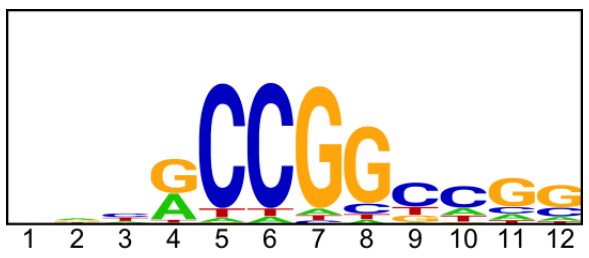

Location of top GRECO-BIT/MEX PWM in MAGIX peaks (up to the top 10,000)

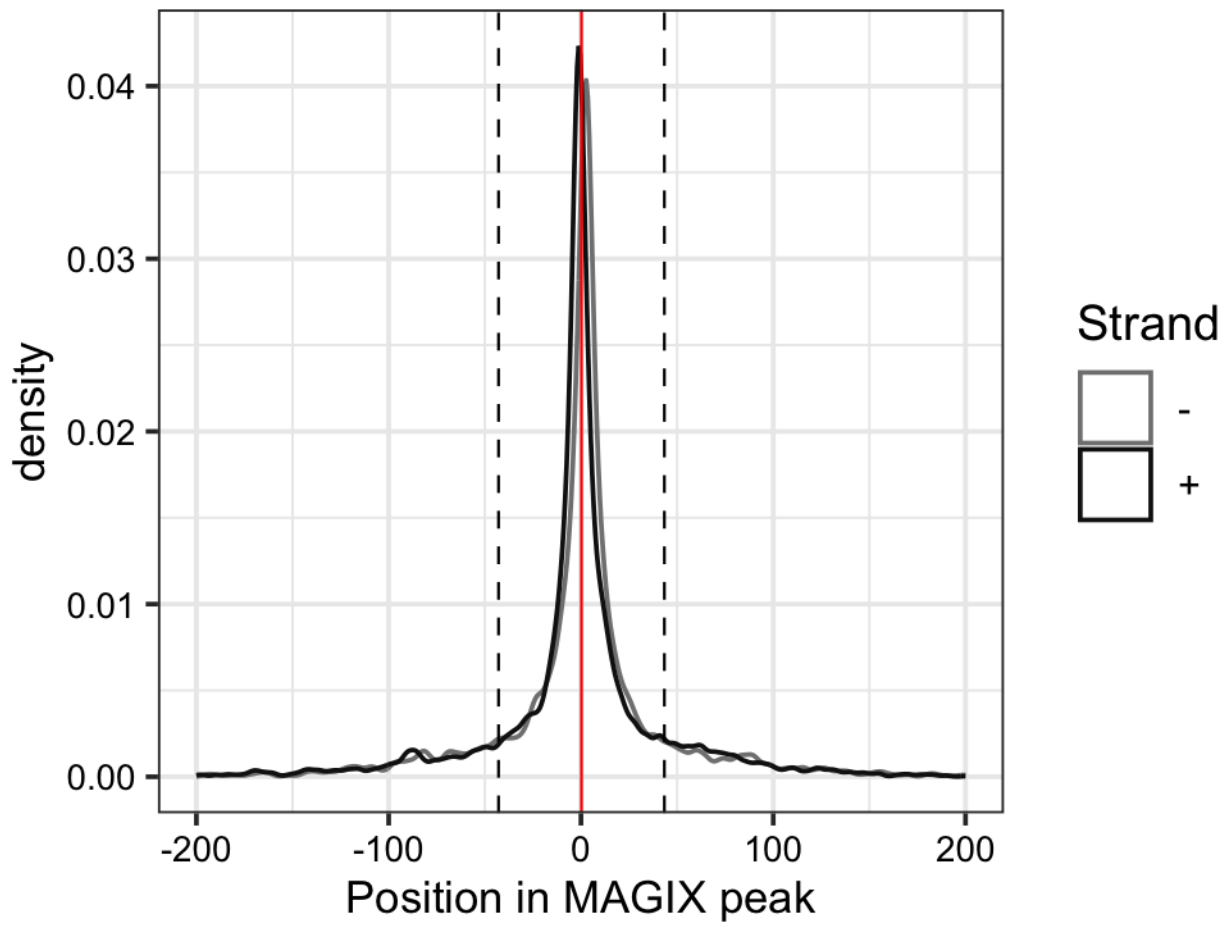

AUROC of top GRECO-BIT/MEX PWM in sliding windows (500 peaks, 50 peak step) along the MAGIX peaks (up to the top 75,000) ranked by the MAGIX enrichment coefficient and fraction of overlapping ChIP peaks in the same window

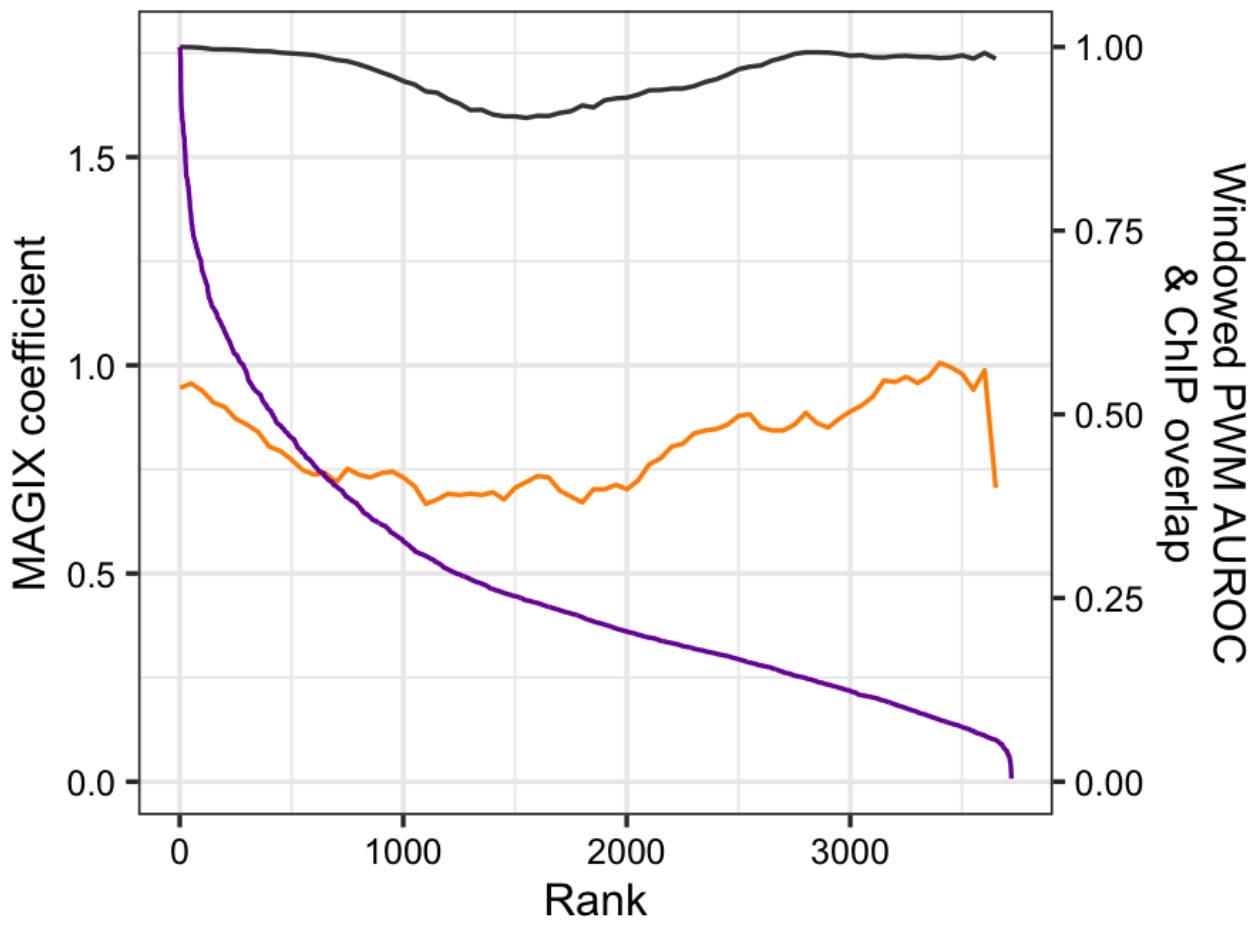

SNAI1

Top GRECO-BIT/MEX PWM: PWM001937

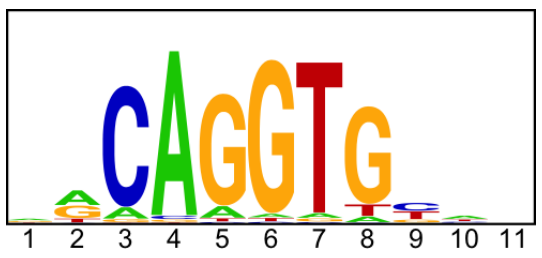

Location of top GRECO-BIT/MEX PWM in MAGIX peaks (up to the top 10,000)

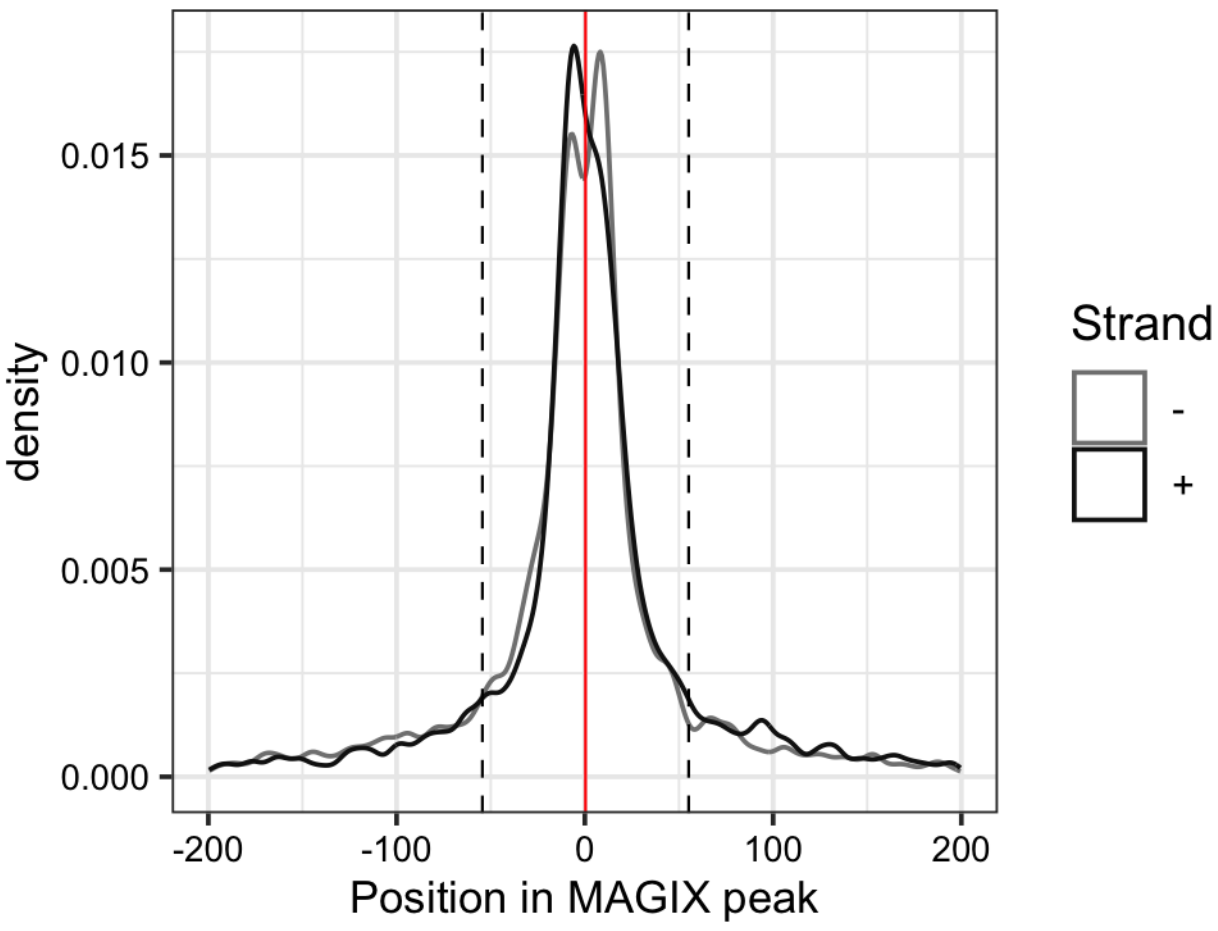

SOX15

Top GRECO-BIT/MEX PWM: PWM001963

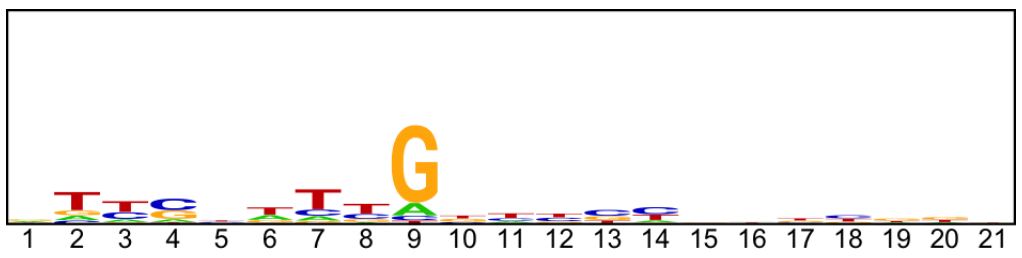

Location of top GRECO-BIT/MEX PWM in MAGIX peaks (up to the top 10,000)

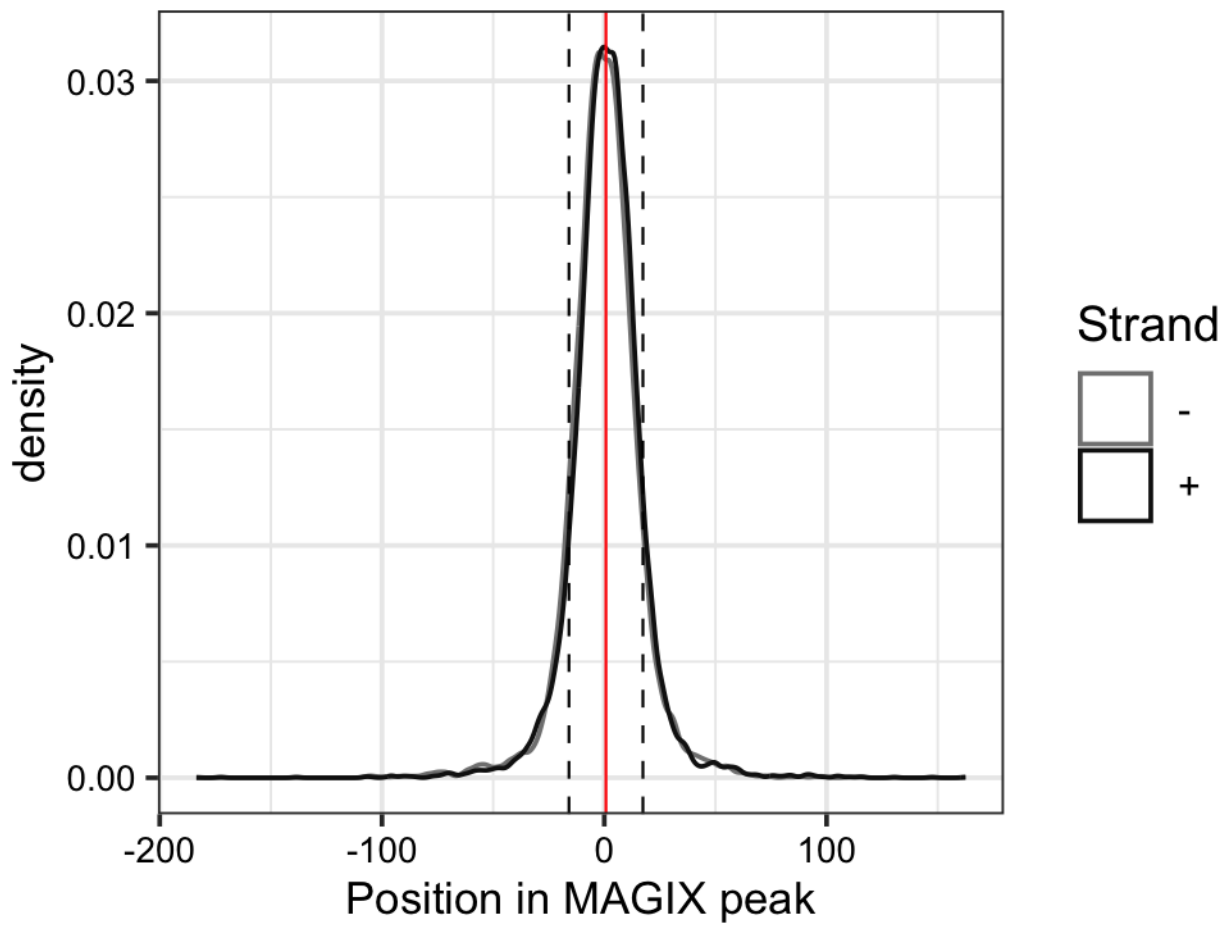

AUROC of top GRECO-BIT/MEX PWM in sliding windows (500 peaks, 50 peak step) along the MAGIX peaks (up to the top 75,000) ranked by the MAGIX enrichment coefficient and fraction of overlapping ChIP peaks in the same window

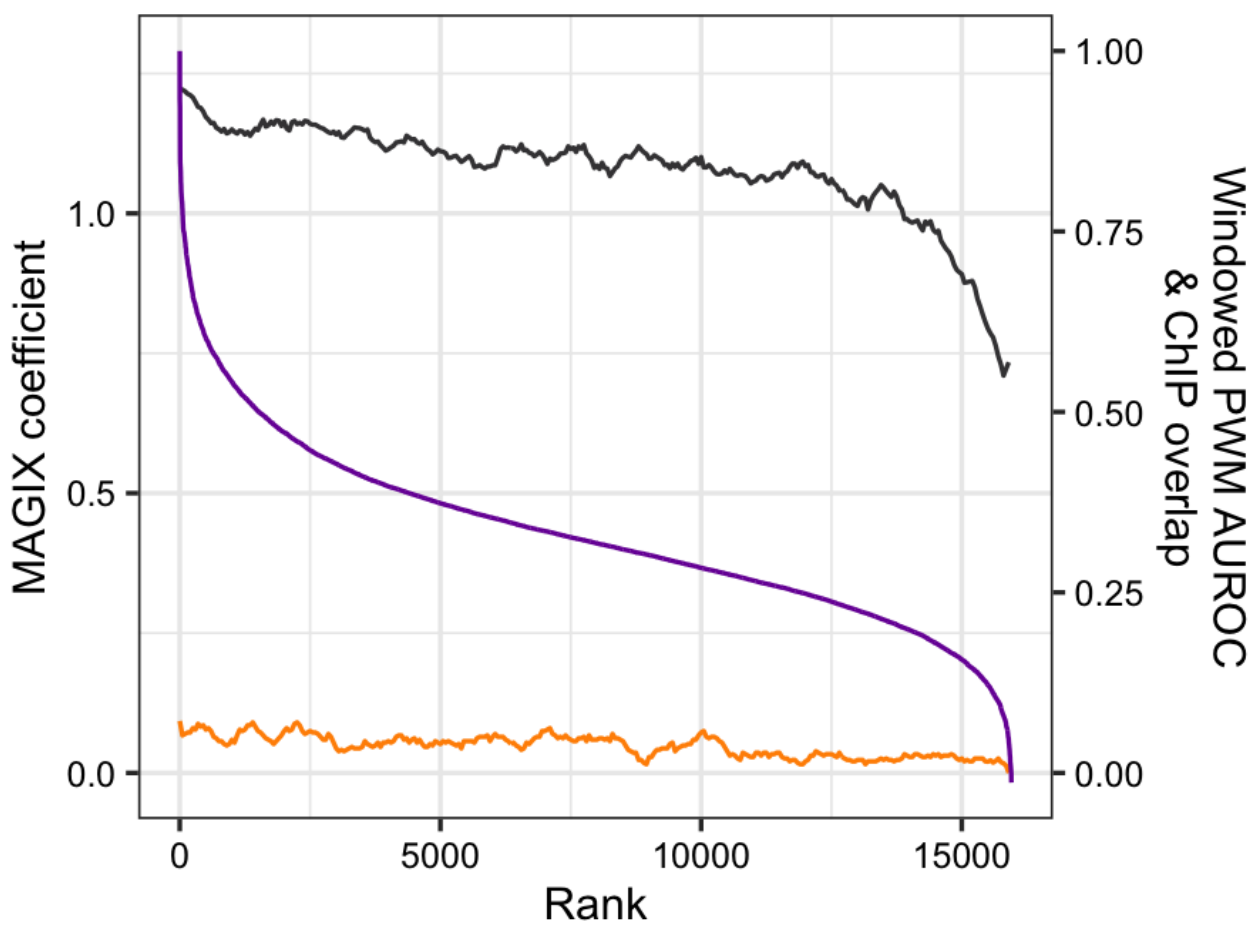

SOX2

Top GRECO-BIT/MEX PWM: PWM002014

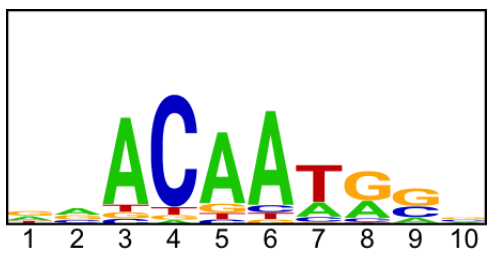

Location of top GRECO-BIT/MEX PWM in MAGIX peaks (up to the top 10,000)

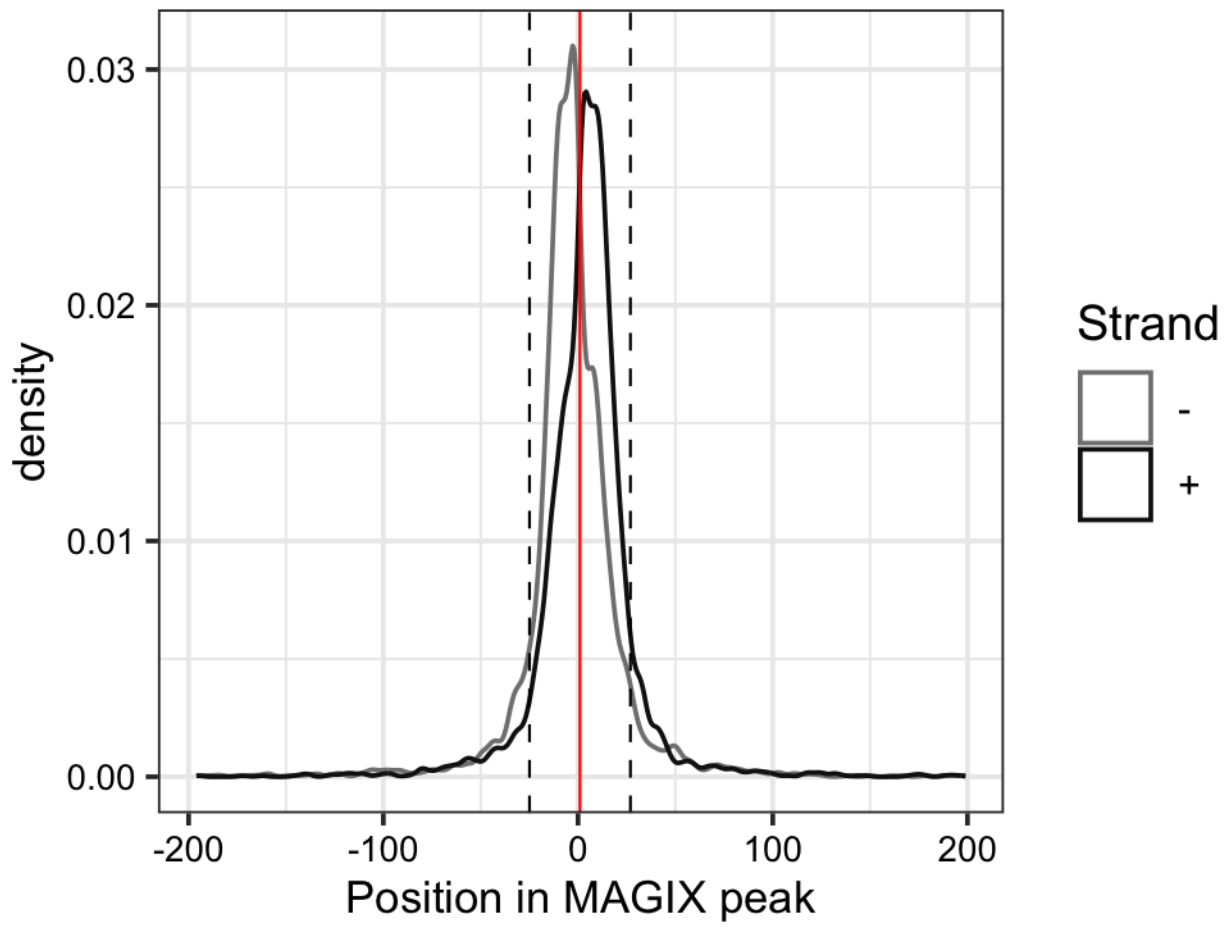

AUROC of top GRECO-BIT/MEX PWM in sliding windows (500 peaks, 50 peak step) along the MAGIX peaks (up to the top 75,000) ranked by the MAGIX enrichment coefficient and fraction of overlapping ChIP peaks in the same window

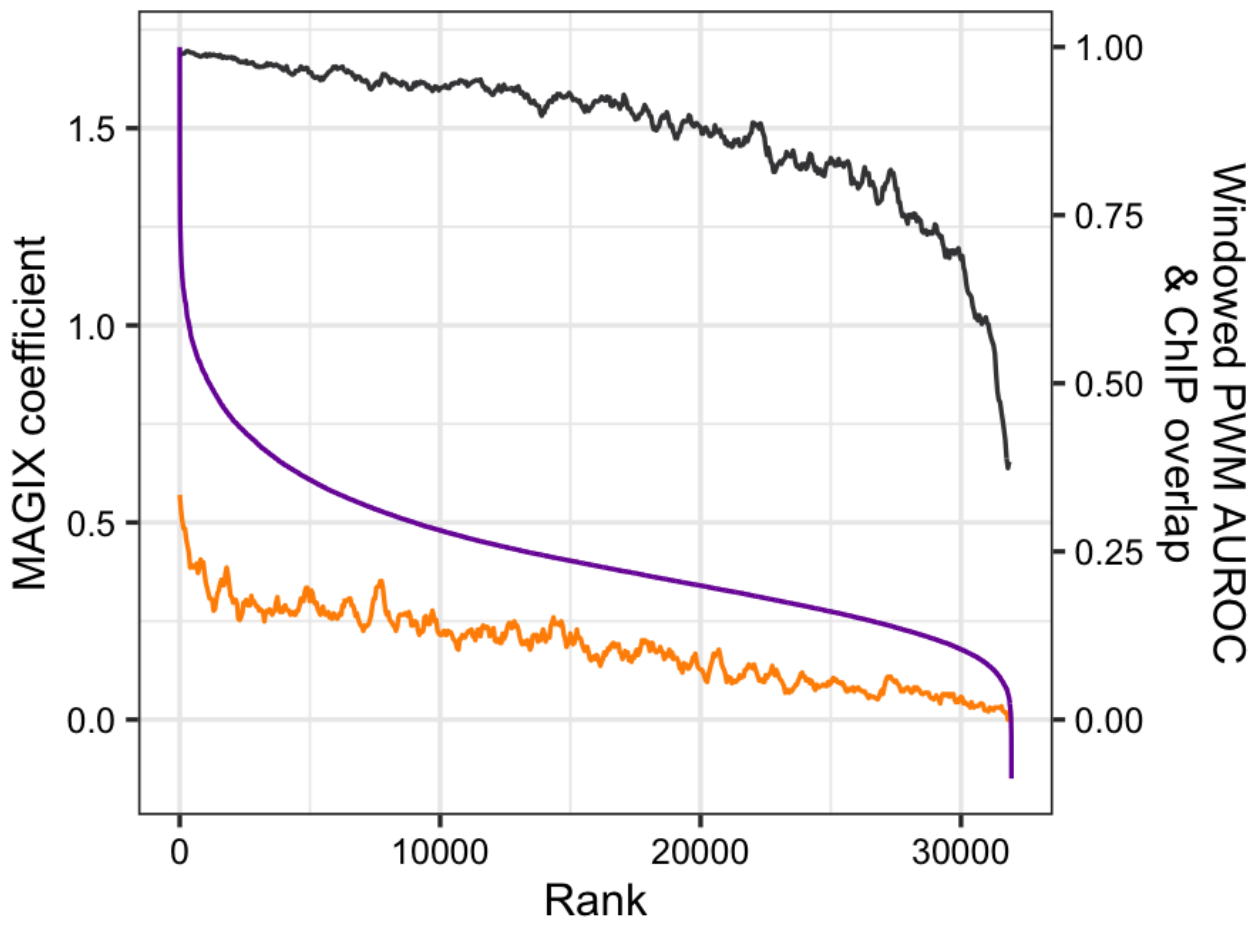

SP100

Top GRECO-BIT/MEX PWM: PWM002035

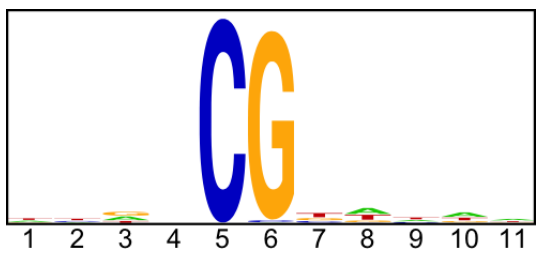

Location of top GRECO-BIT/MEX PWM in MAGIX peaks (up to the top 10,000)

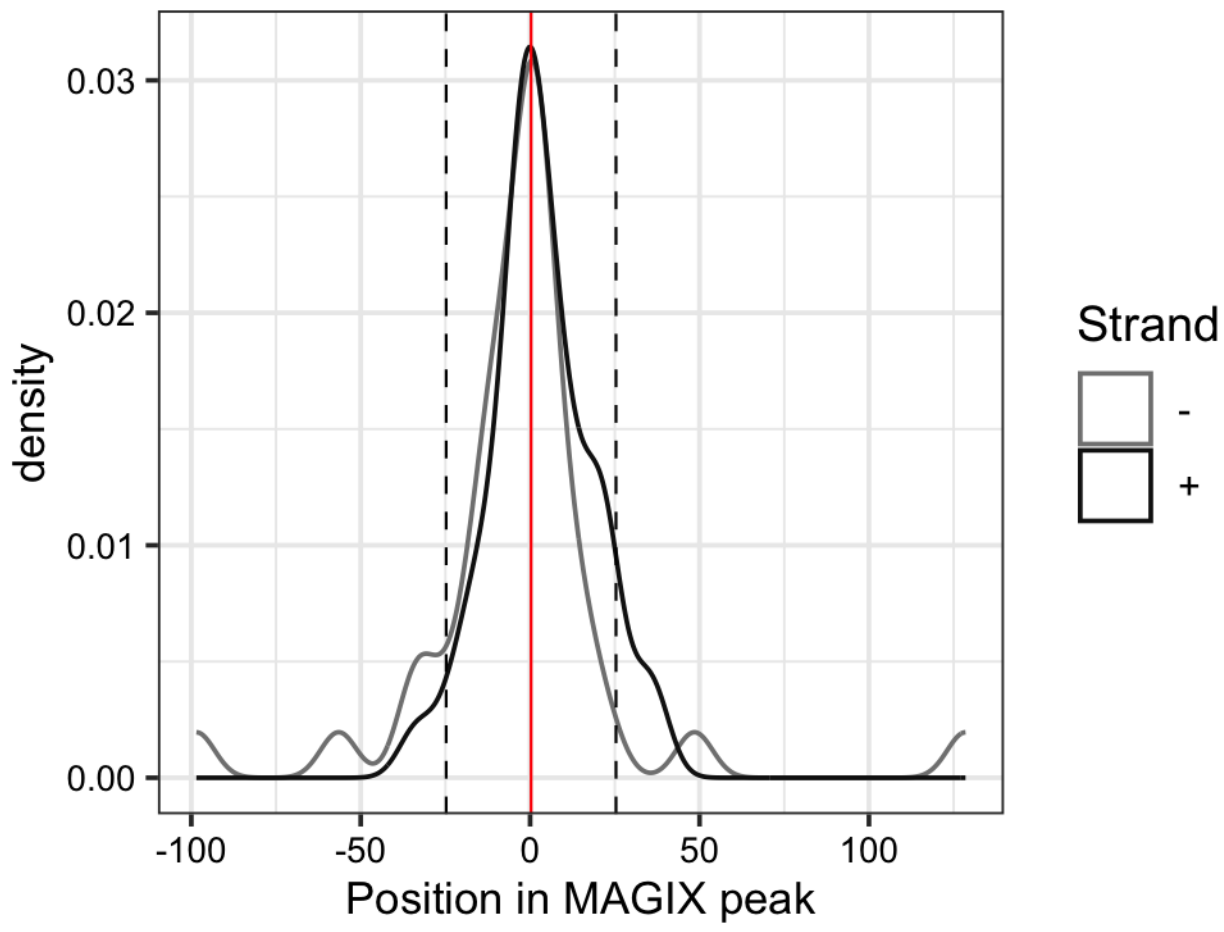

AUROC of top GRECO-BIT/MEX PWM in sliding windows (500 peaks, 50 peak step) along the MAGIX peaks (up to the top 75,000) ranked by the MAGIX enrichment coefficient and fraction of overlapping ChIP peaks in the same window

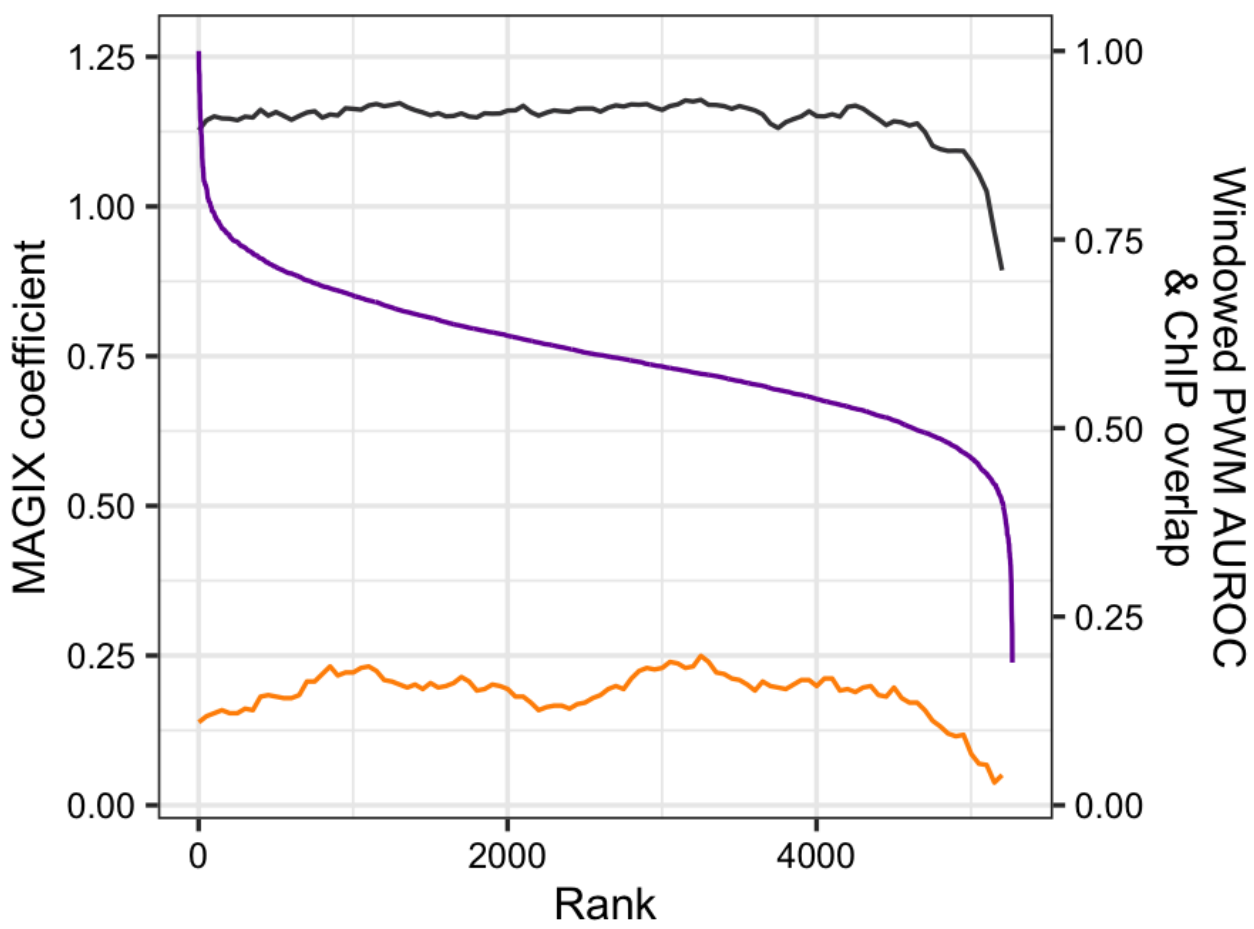

SP140

Top GRECO-BIT/MEX PWM: PWM002066

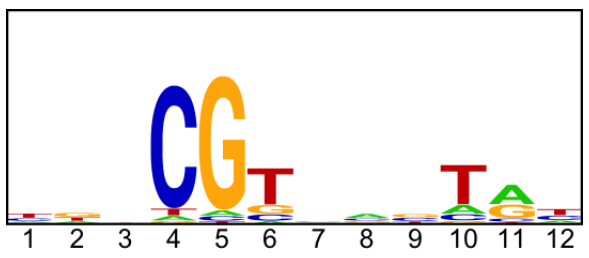

Location of top GRECO-BIT/MEX PWM in MAGIX peaks (up to the top 10,000)

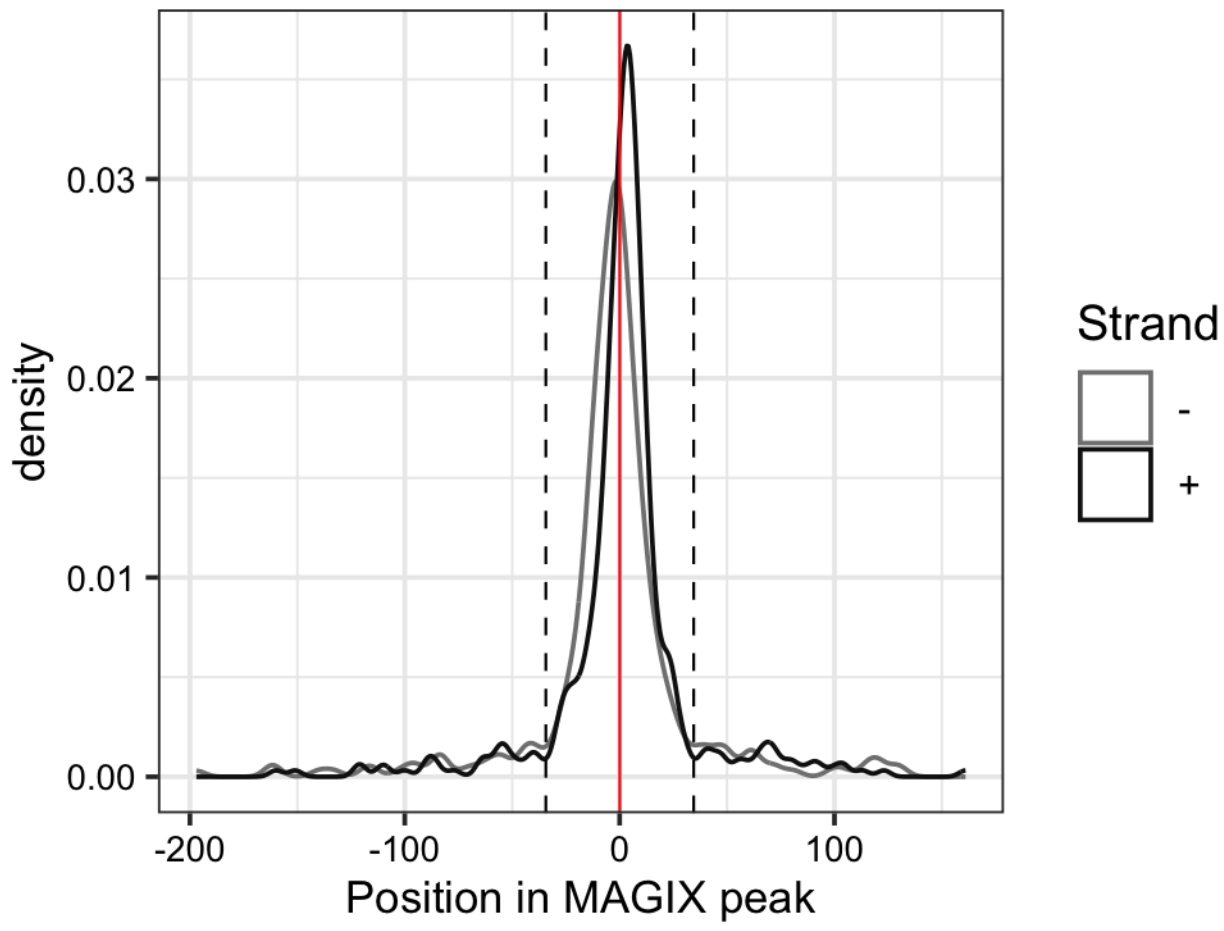

AUROC of top GRECO-BIT/MEX PWM in sliding windows (500 peaks, 50 peak step) along the MAGIX peaks (up to the top 75,000) ranked by the MAGIX enrichment coefficient and fraction of overlapping ChIP peaks in the same window

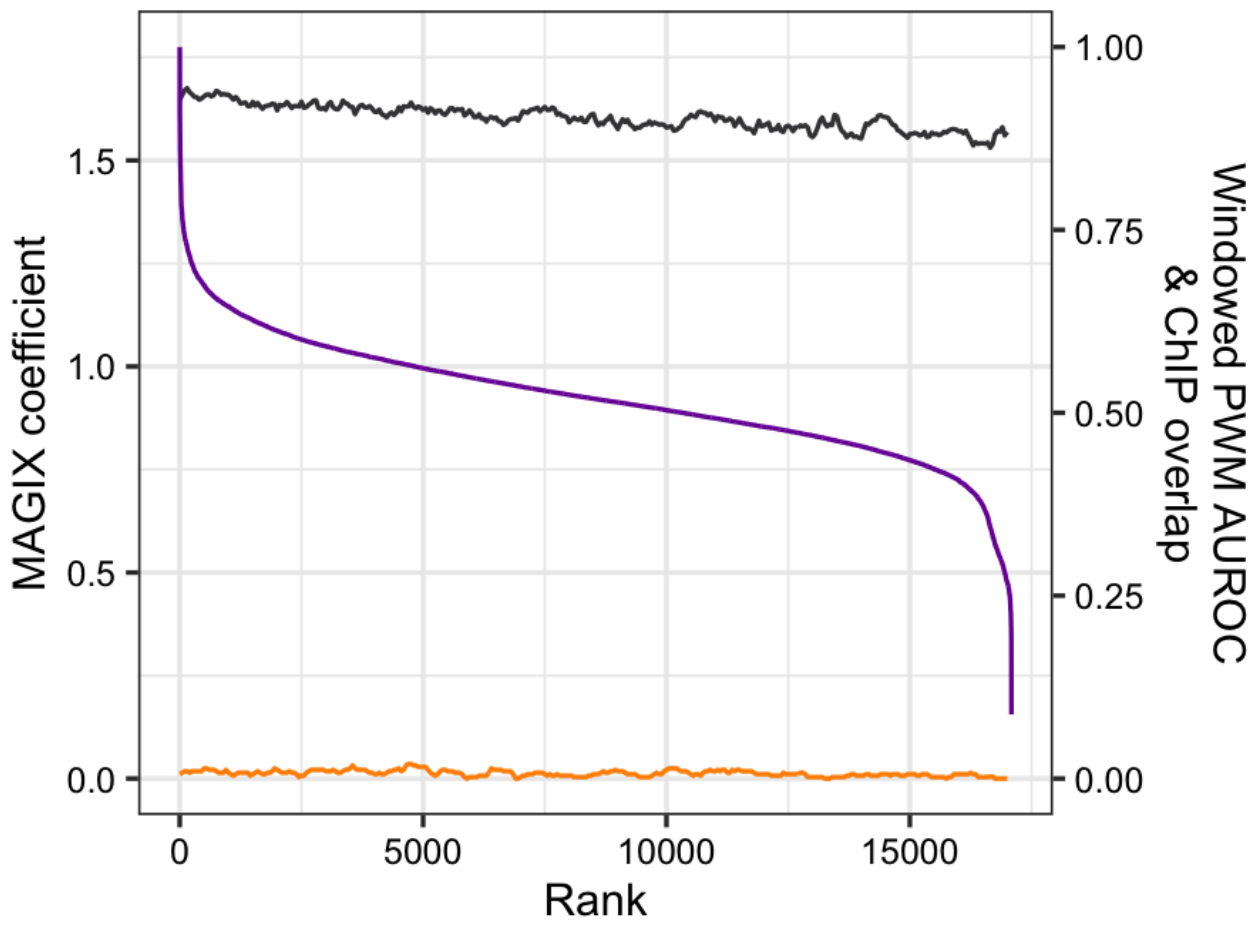

SP140L

Top GRECO-BIT/MEX PWM: PWM002102

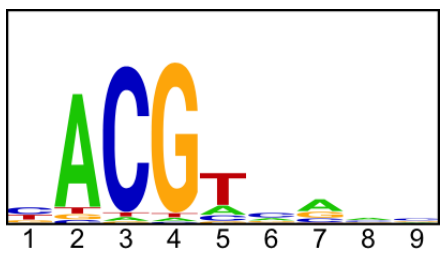

Location of top GRECO-BIT/MEX PWM in MAGIX peaks (up to the top 10,000)

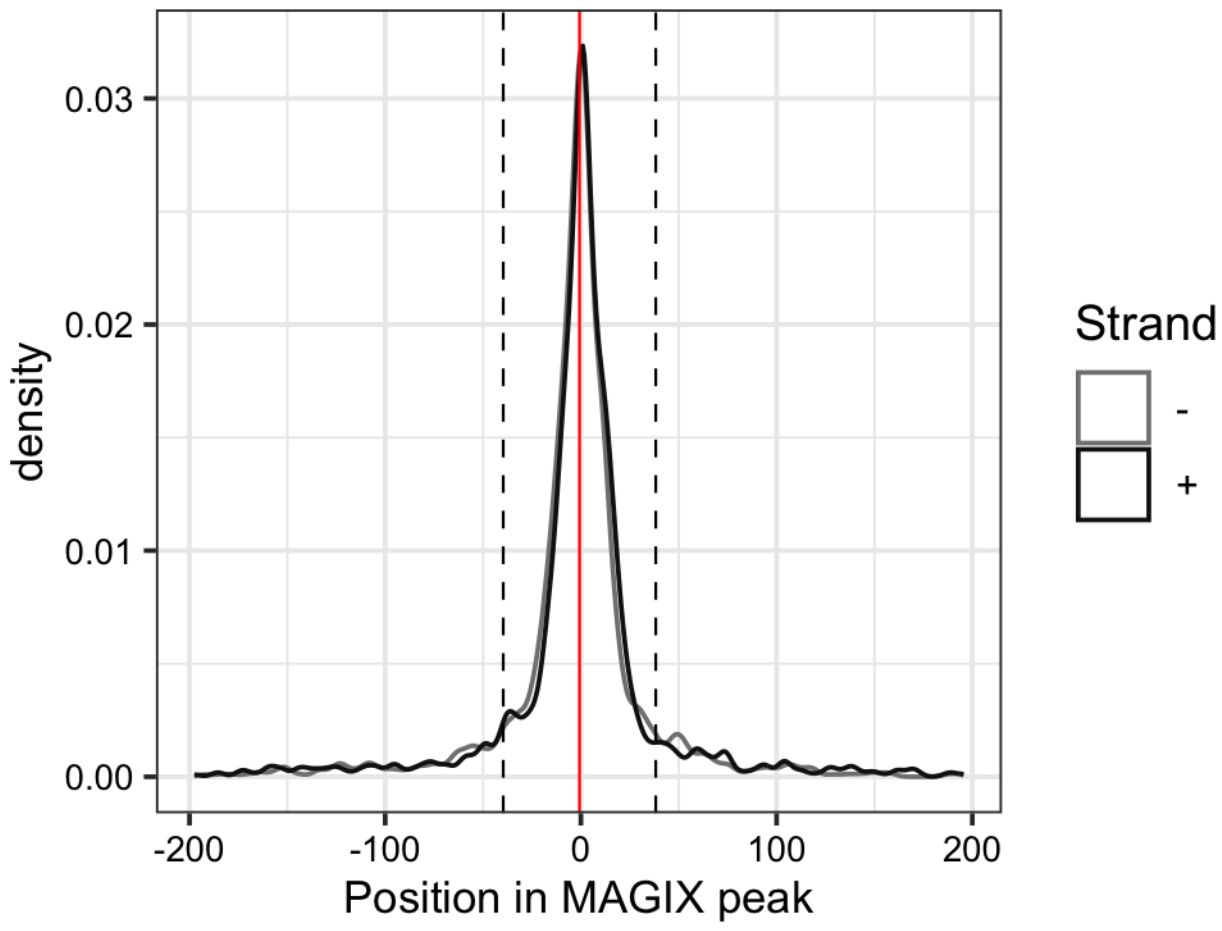

AUROC of top GRECO-BIT/MEX PWM in sliding windows (500 peaks, 50 peak step) along the MAGIX peaks (up to the top 75,000) ranked by the MAGIX enrichment coefficient and fraction of overlapping ChIP peaks in the same window

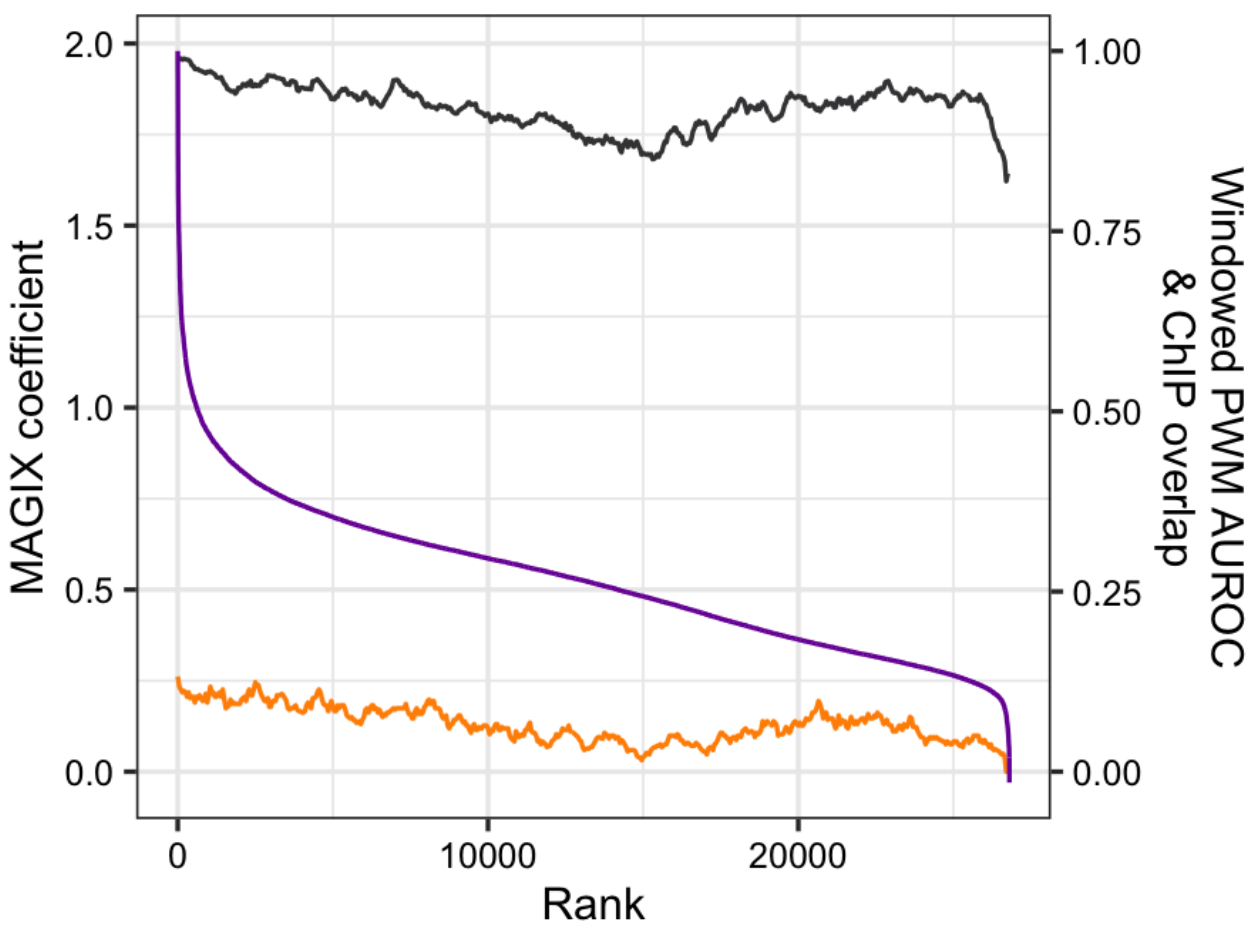

SRY

Top GRECO-BIT/MEX PWM: PWM002143

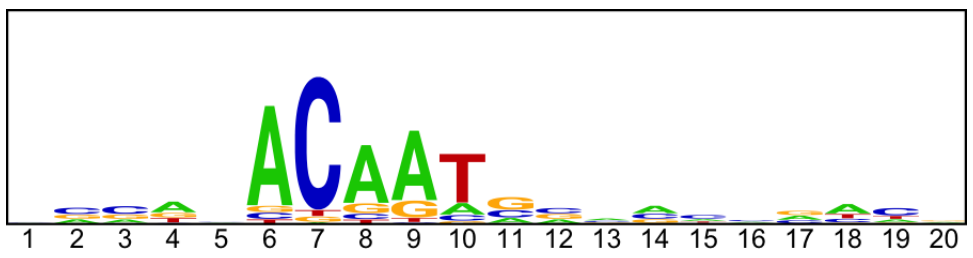

Location of top GRECO-BIT/MEX PWM in MAGIX peaks (up to the top 10,000)

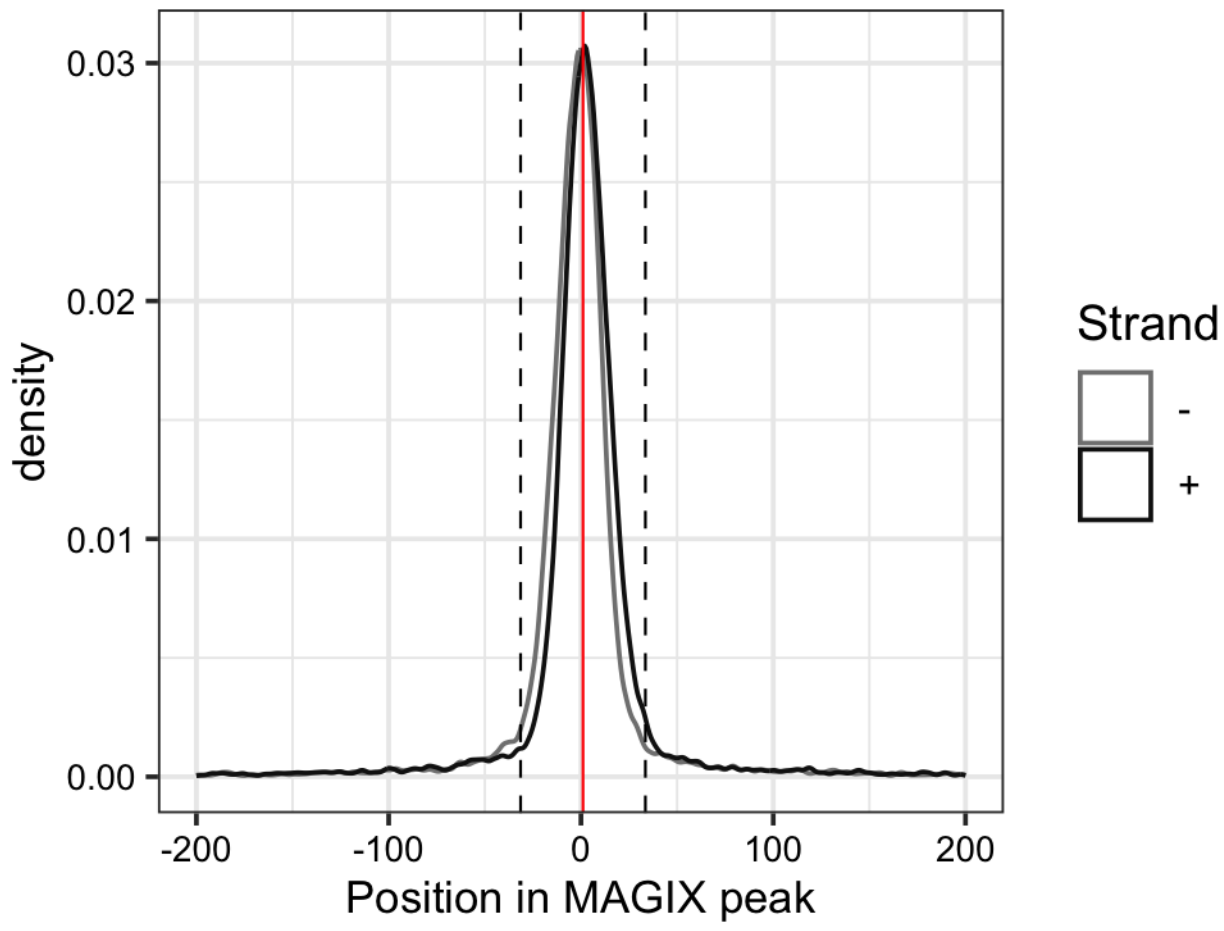

AUROC of top GRECO-BIT/MEX PWM in sliding windows (500 peaks, 50 peak step) along the MAGIX peaks (up to the top 75,000) ranked by the MAGIX enrichment coefficient and fraction of overlapping ChIP peaks in the same window

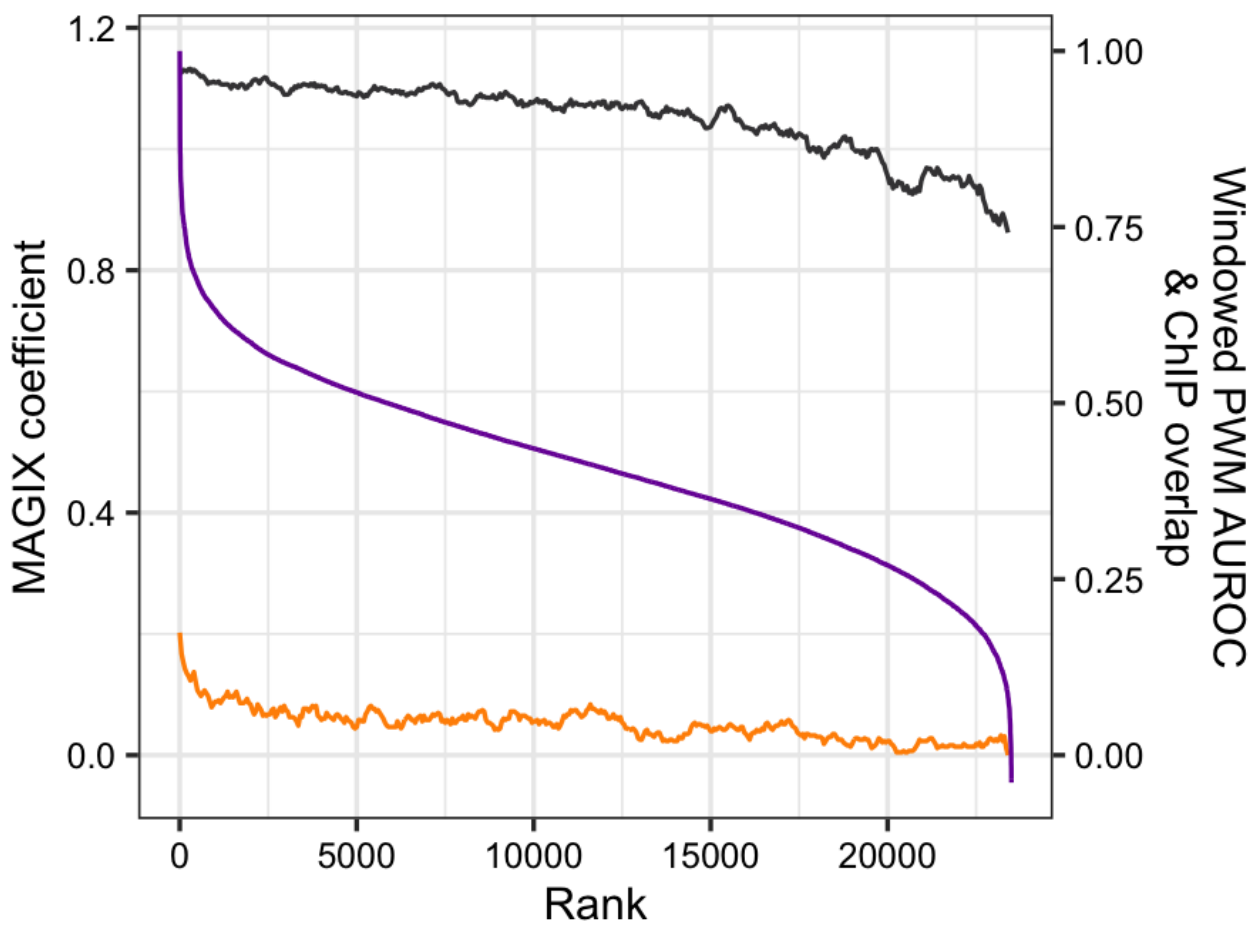

TERF1

Top GRECO-BIT/MEX PWM: PWM002154

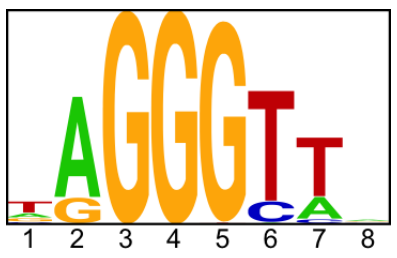

Location of top GRECO-BIT/MEX PWM in MAGIX peaks (up to the top 10,000)

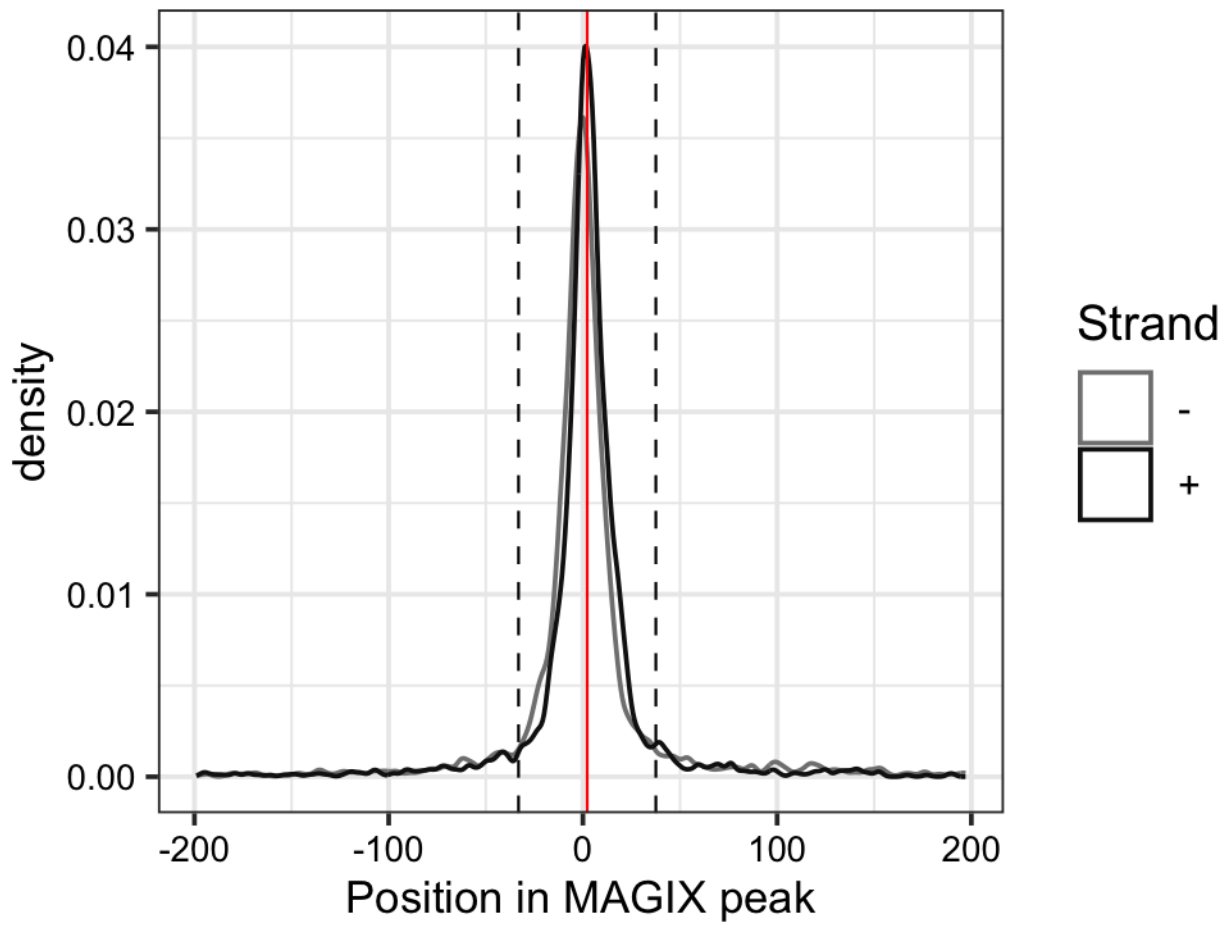

AUROC of top GRECO-BIT/MEX PWM in sliding windows (500 peaks, 50 peak step) along the MAGIX peaks (up to the top 75,000) ranked by the MAGIX enrichment coefficient and fraction of overlapping ChIP peaks in the same window

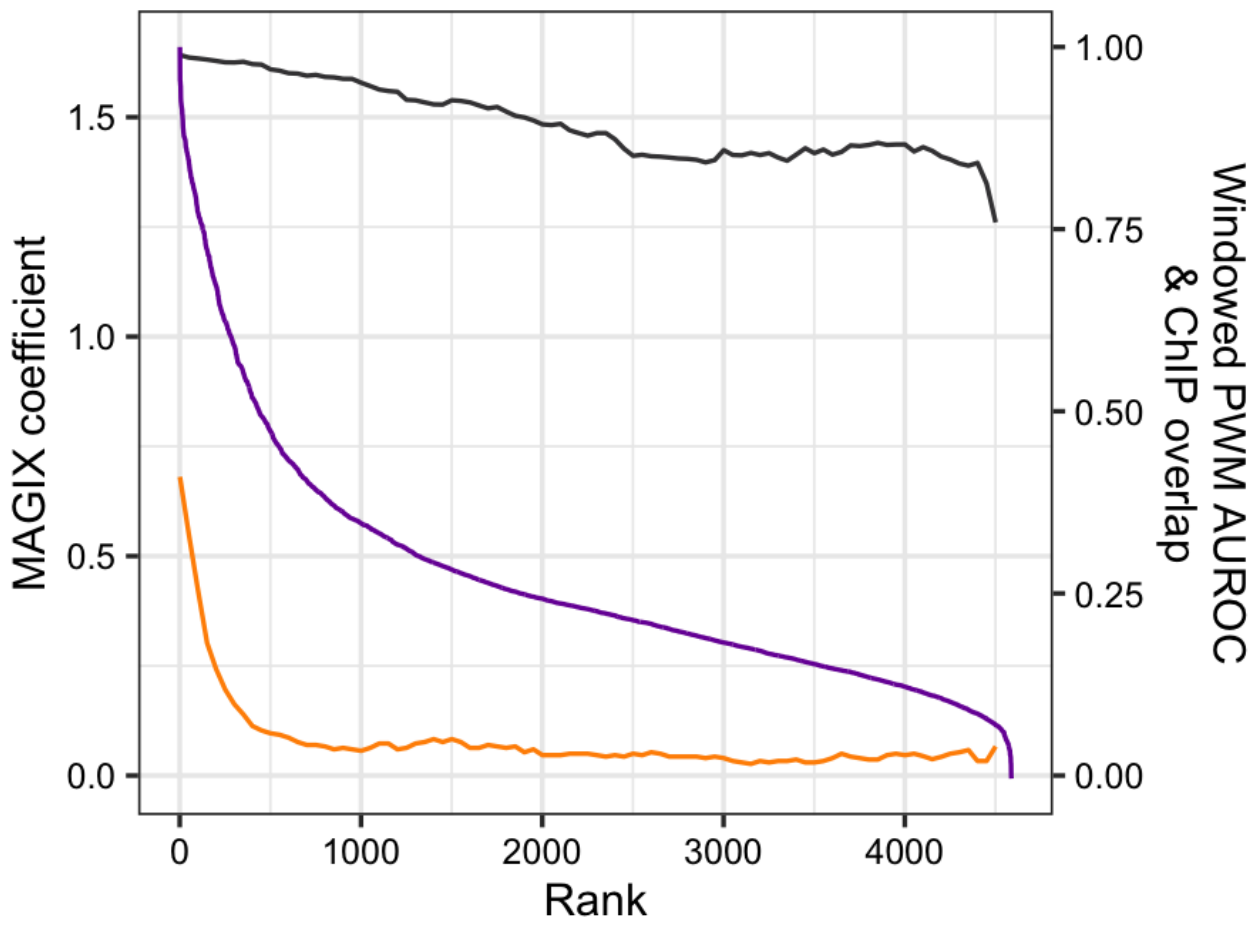

TET3

Top GRECO-BIT/MEX PWM: PWM002217

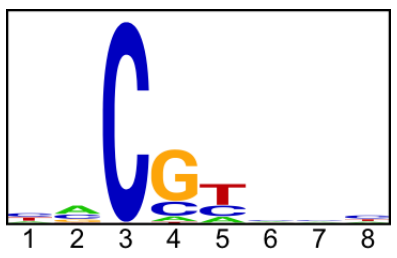

Location of top GRECO-BIT/MEX PWM in MAGIX peaks (up to the top 10,000)

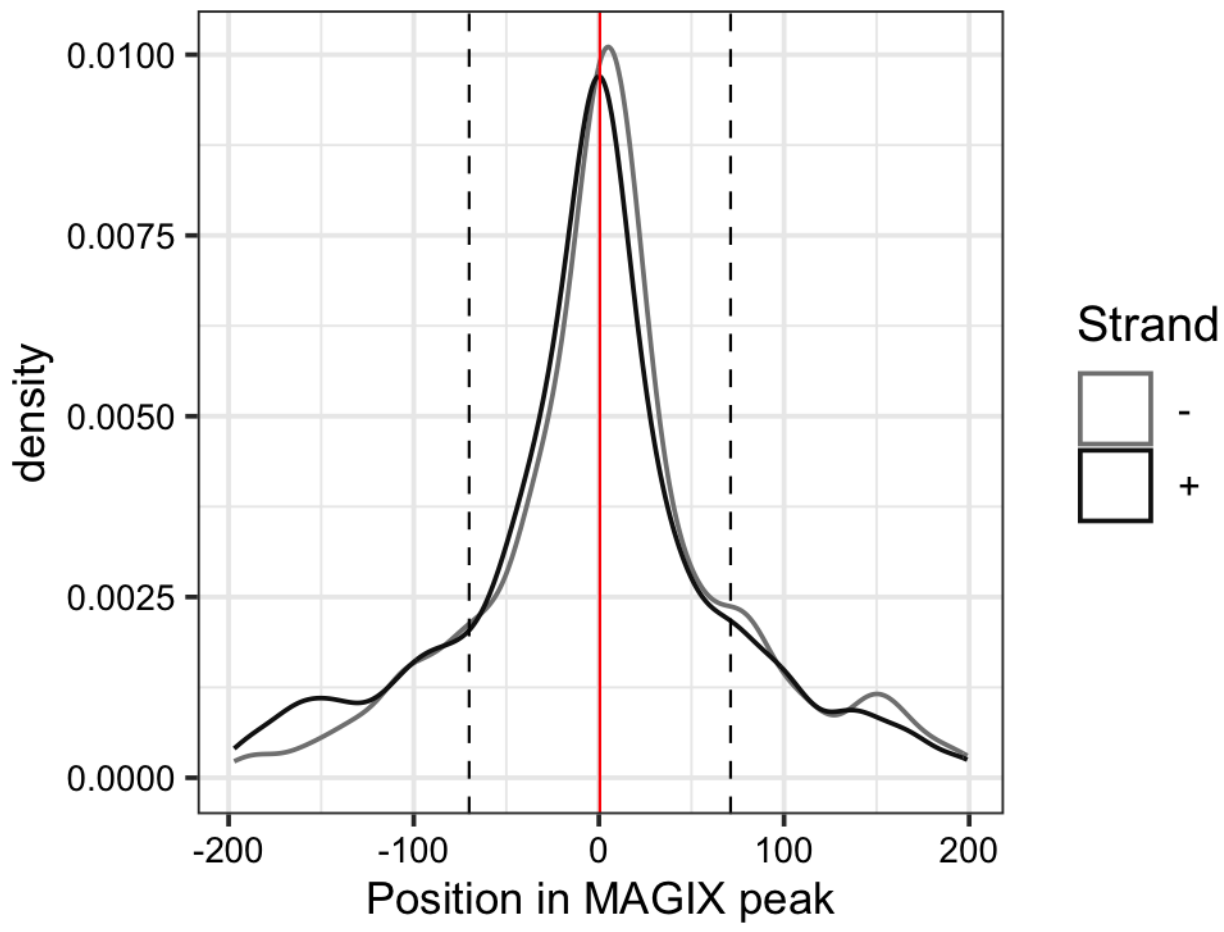

AUROC of top GRECO-BIT/MEX PWM in sliding windows (500 peaks, 50 peak step) along the MAGIX peaks (up to the top 75,000) ranked by the MAGIX enrichment coefficient and fraction of overlapping ChIP peaks in the same window

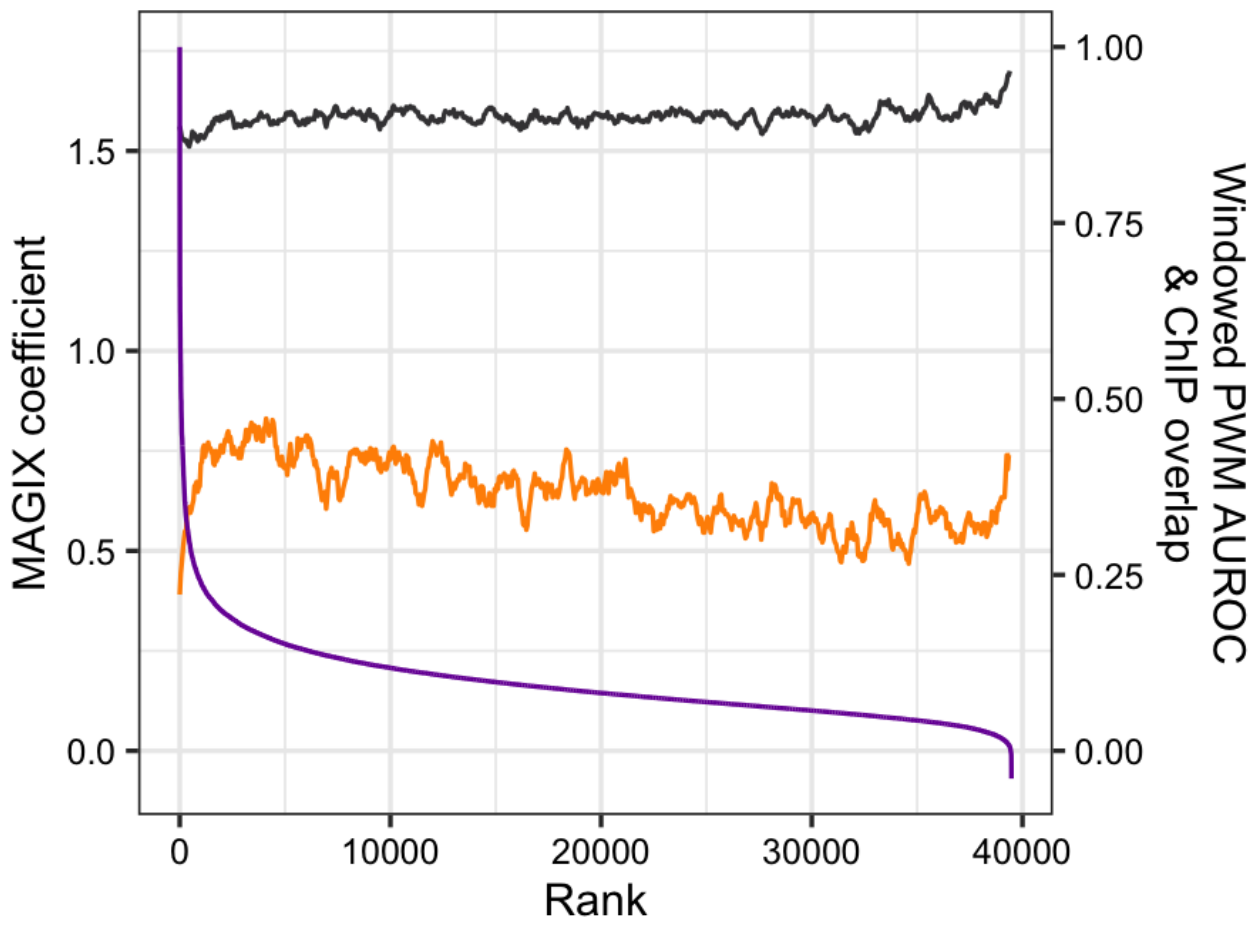

TIGD3

Top GRECO-BIT/MEX PWM: PWM002247

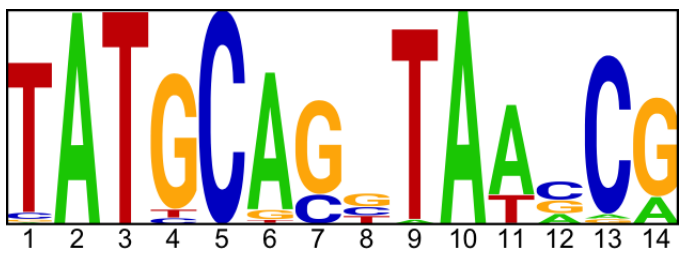

Location of top GRECO-BIT/MEX PWM in MAGIX peaks (up to the top 10,000)

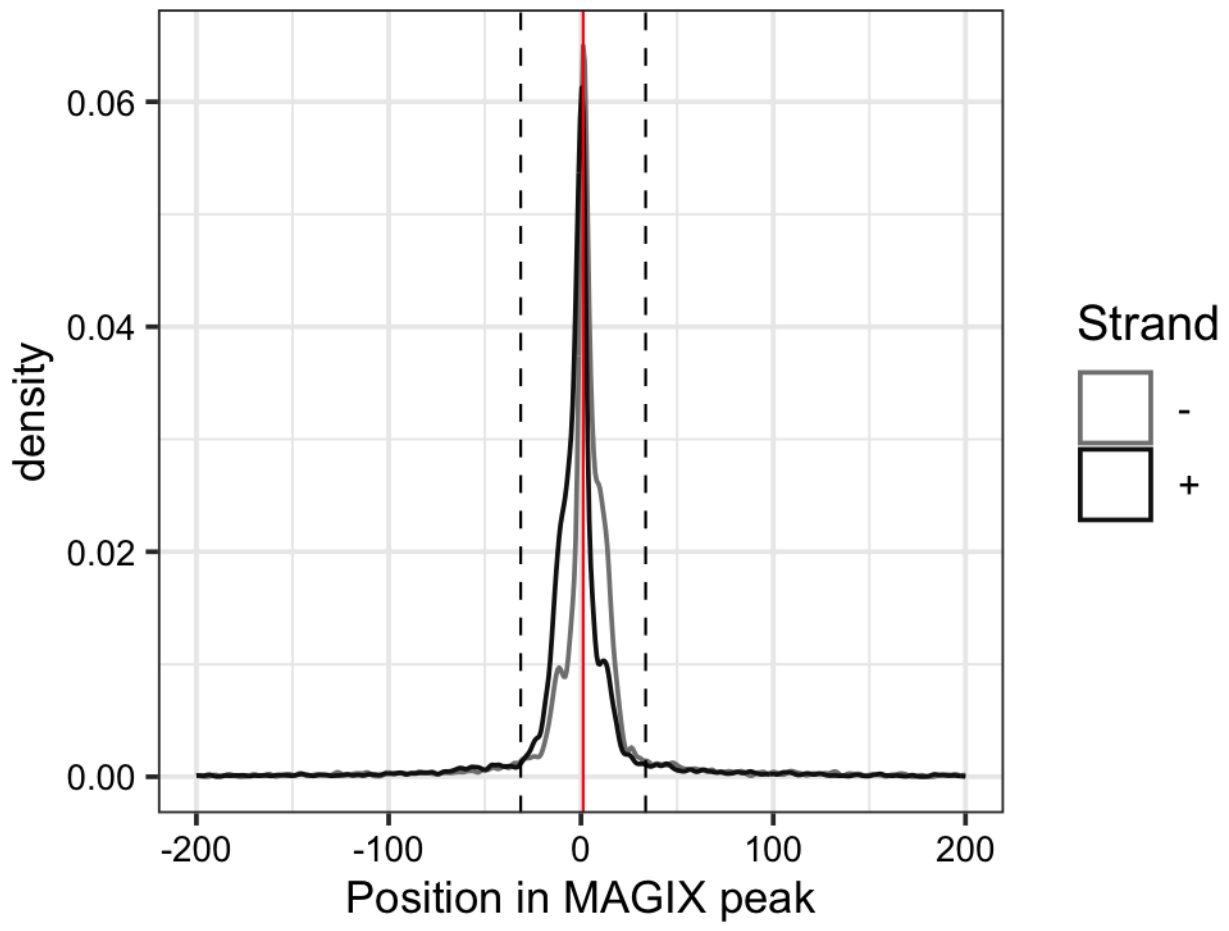

AUROC of top GRECO-BIT/MEX PWM in sliding windows (500 peaks, 50 peak step) along the MAGIX peaks (up to the top 75,000) ranked by the MAGIX enrichment coefficient and fraction of overlapping ChIP peaks in the same window

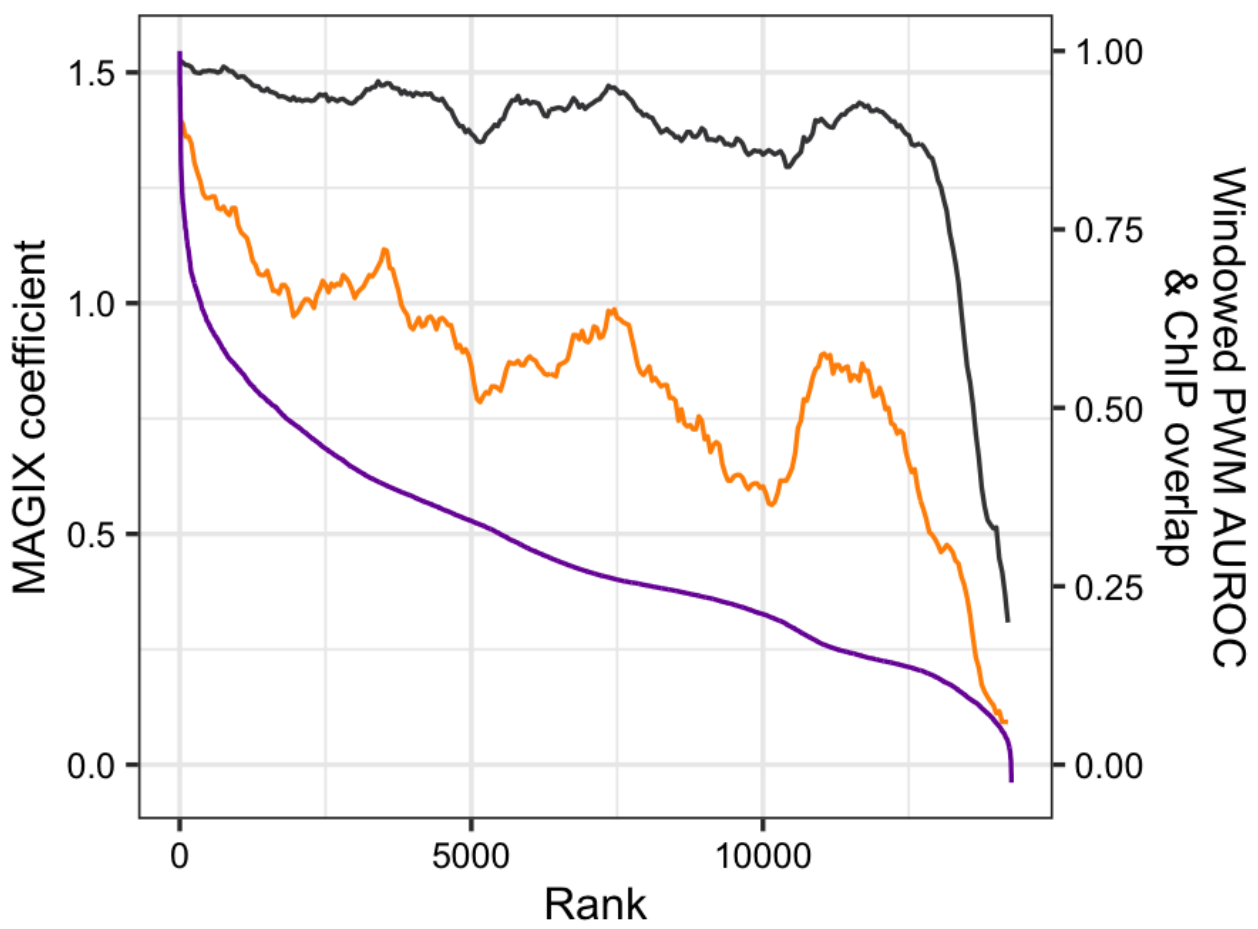

TIGD4

Top GRECO-BIT/MEX PWM: PWM089770

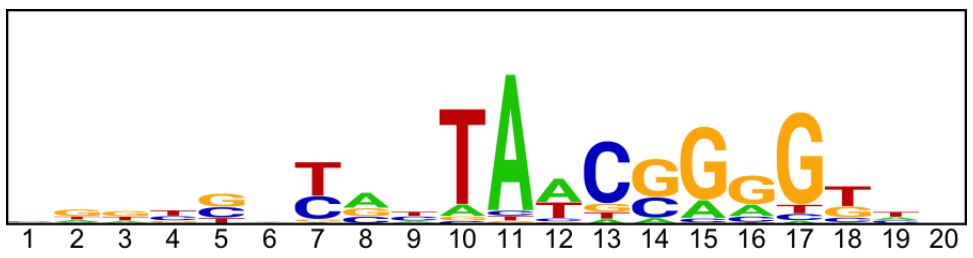

Location of top GRECO-BIT/MEX PWM in MAGIX peaks (up to the top 10,000)

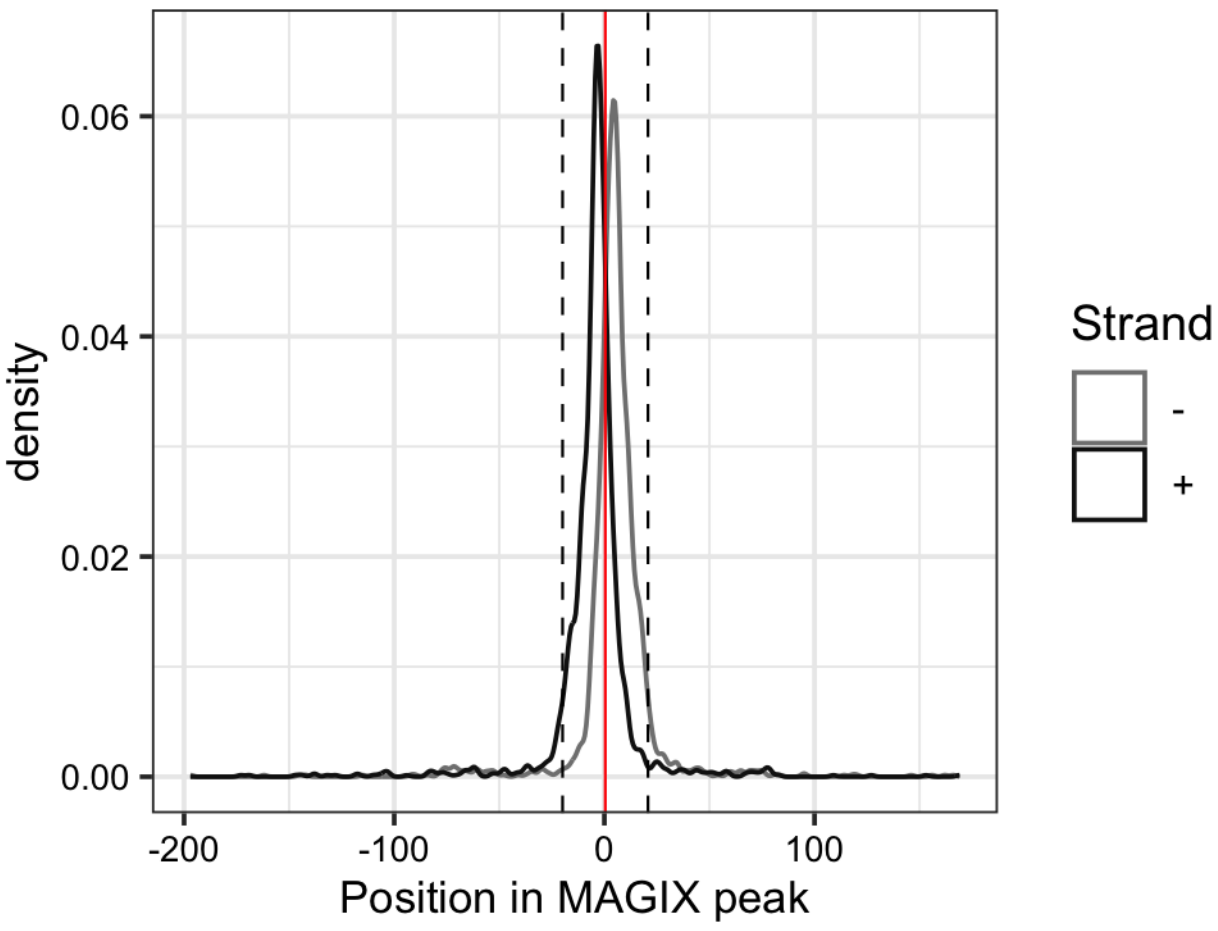

TIGD5

Top GRECO-BIT/MEX PWM: PWM002303

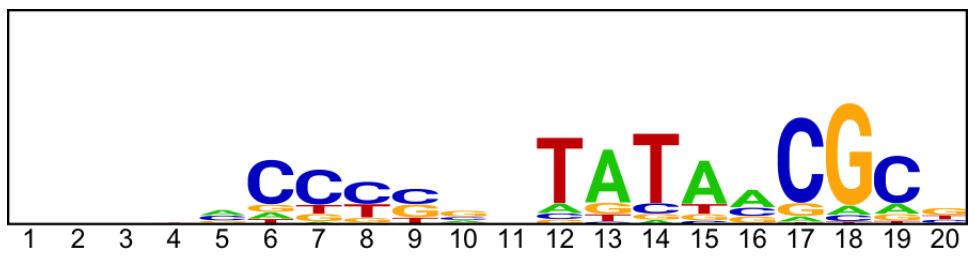

Location of top GRECO-BIT/MEX PWM in MAGIX peaks (up to the top 10,000)

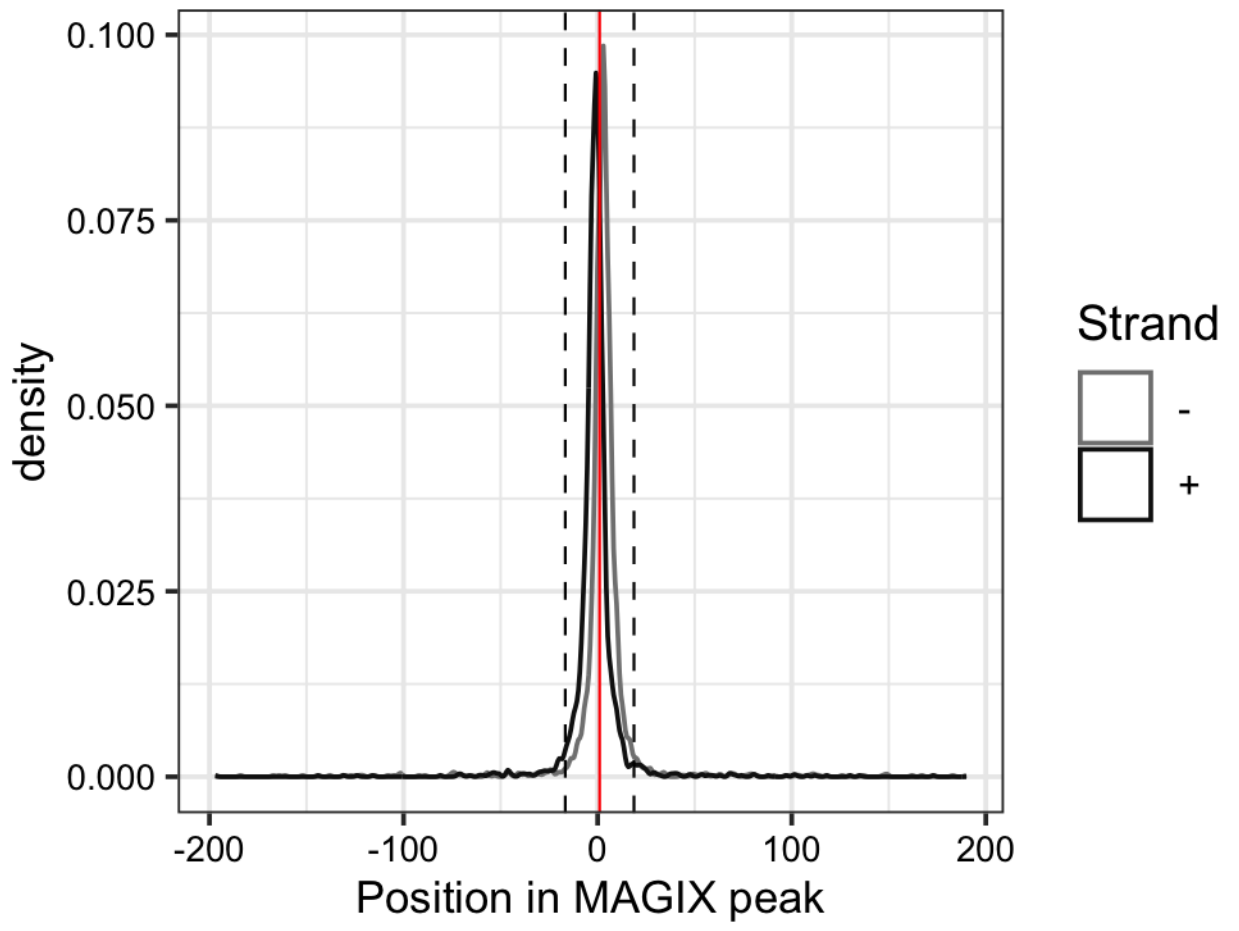

AUROC of top GRECO-BIT/MEX PWM in sliding windows (500 peaks, 50 peak step) along the MAGIX peaks (up to the top 75,000) ranked by the MAGIX enrichment coefficient and fraction of overlapping ChIP peaks in the same window

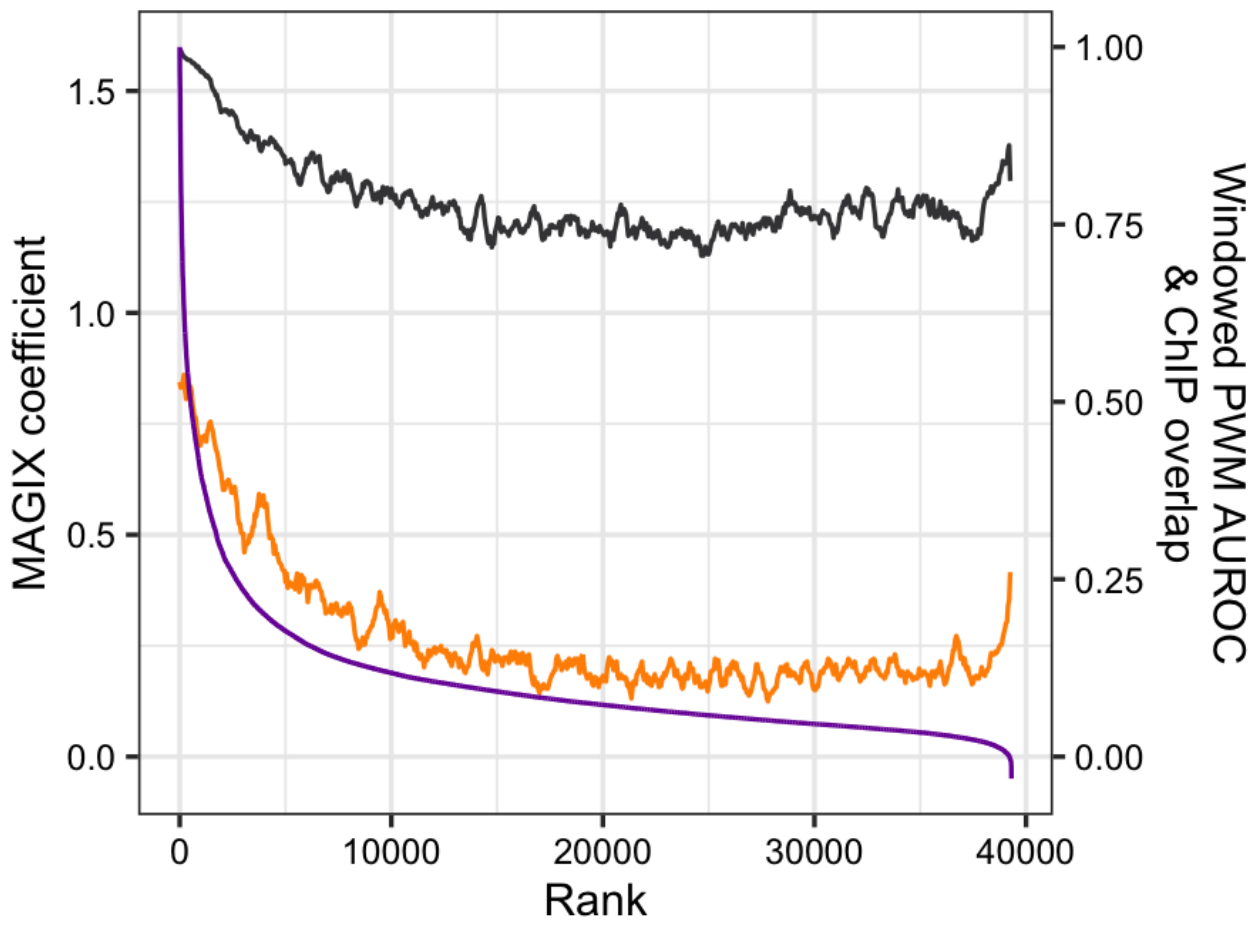

TIGD7

Top GRECO-BIT/MEX PWM: PWM002334

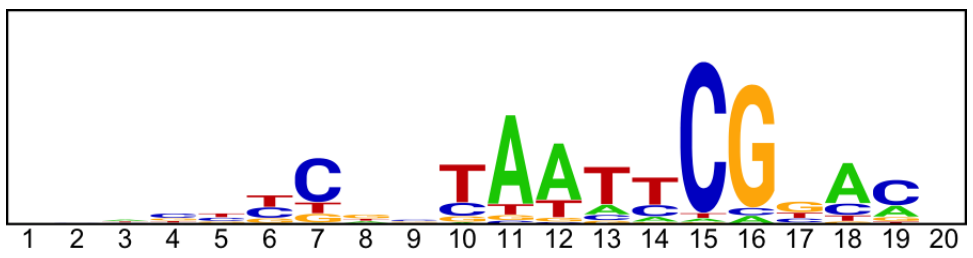

Location of top GRECO-BIT/MEX PWM in MAGIX peaks (up to the top 10,000)

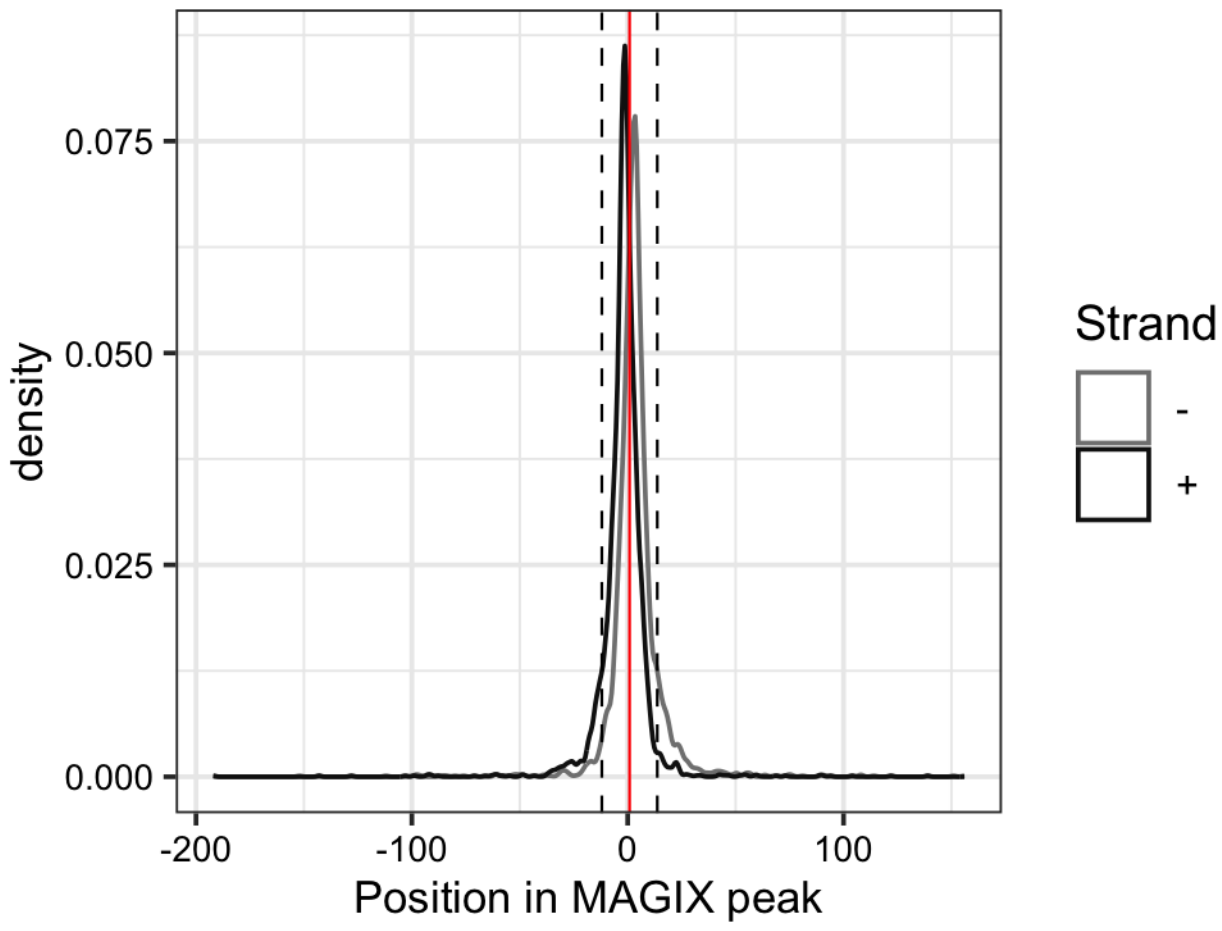

TPRX1

Top GRECO-BIT/MEX PWM: PWM002363

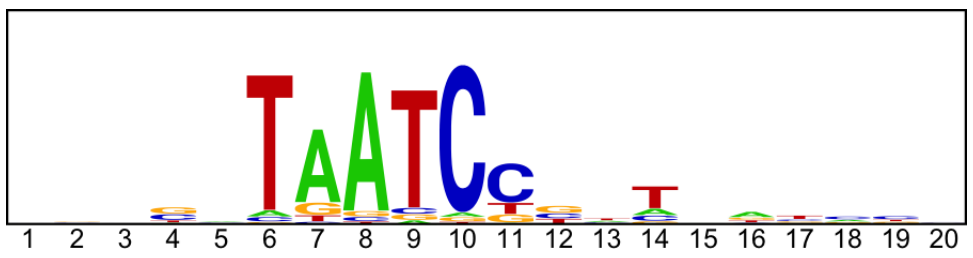

Location of top GRECO-BIT/MEX PWM in MAGIX peaks (up to the top 10,000)

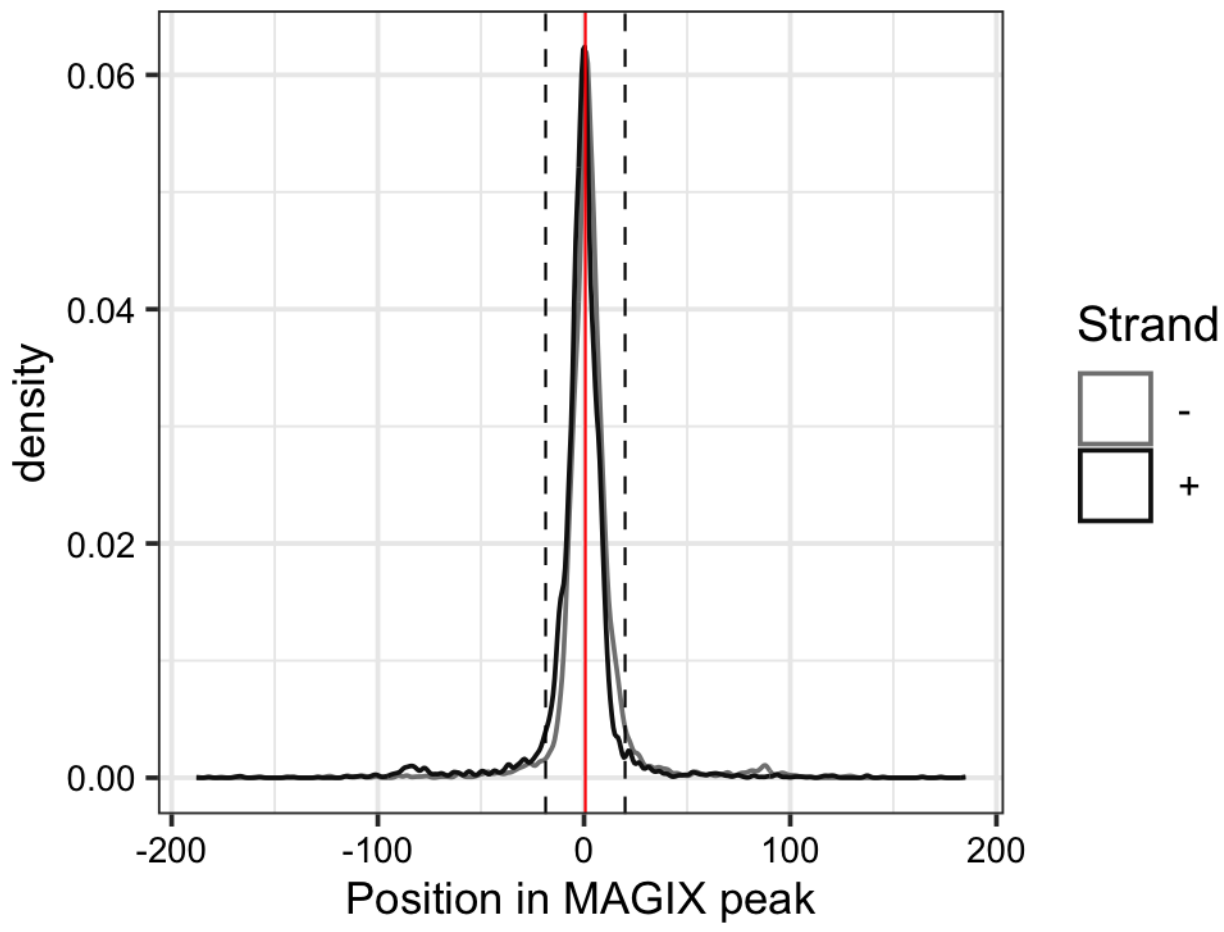

AUROC of top GRECO-BIT/MEX PWM in sliding windows (500 peaks, 50 peak step) along the MAGIX peaks (up to the top 75,000) ranked by the MAGIX enrichment coefficient and fraction of overlapping ChIP peaks in the same window

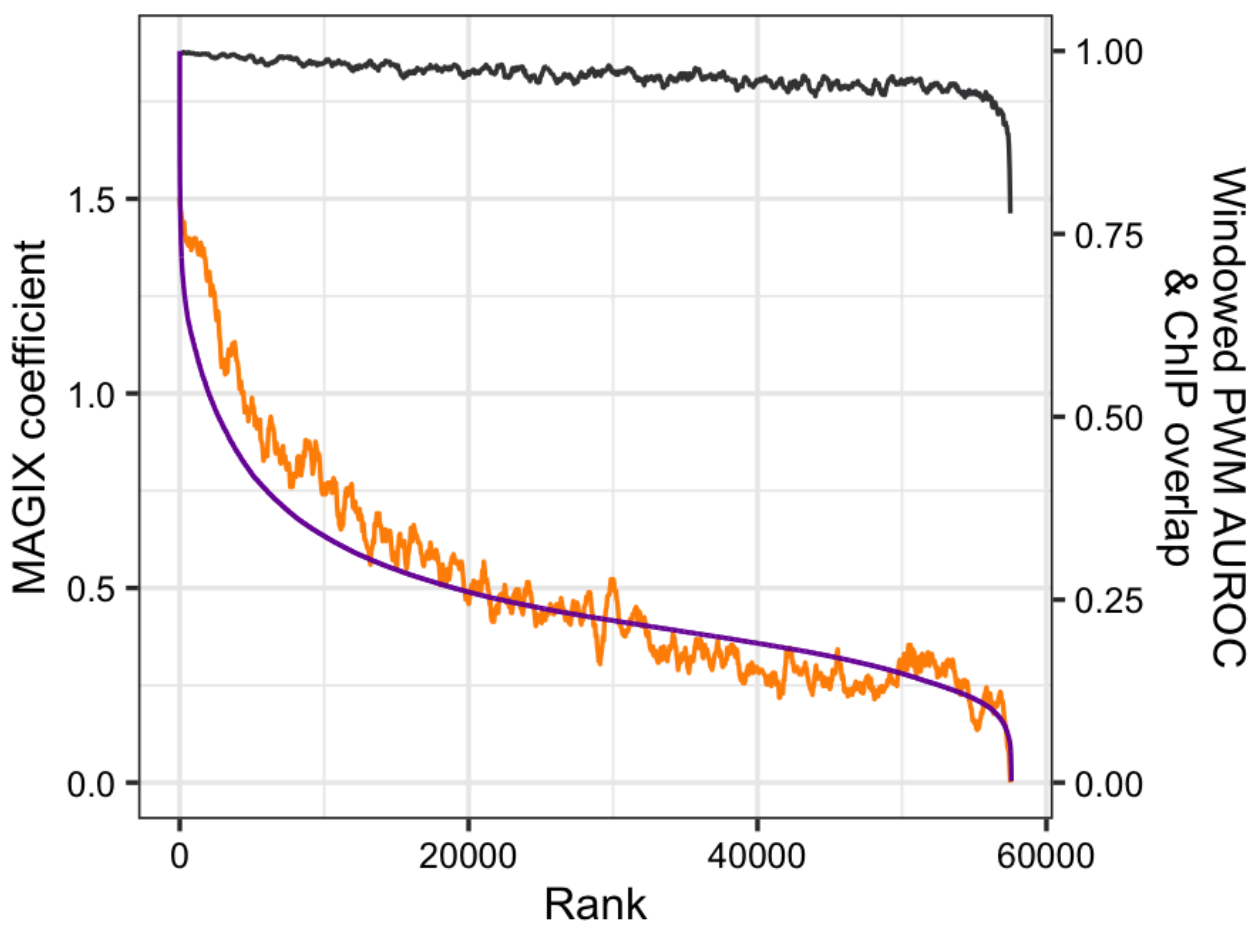

TSHZ2.DBD2

Top GRECO-BIT/MEX PWM: PWM002395

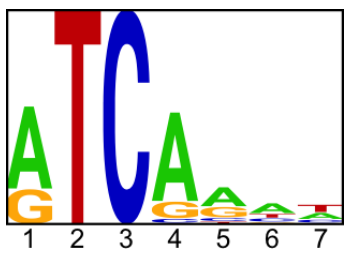

Location of top GRECO-BIT/MEX PWM in MAGIX peaks (up to the top 10,000)

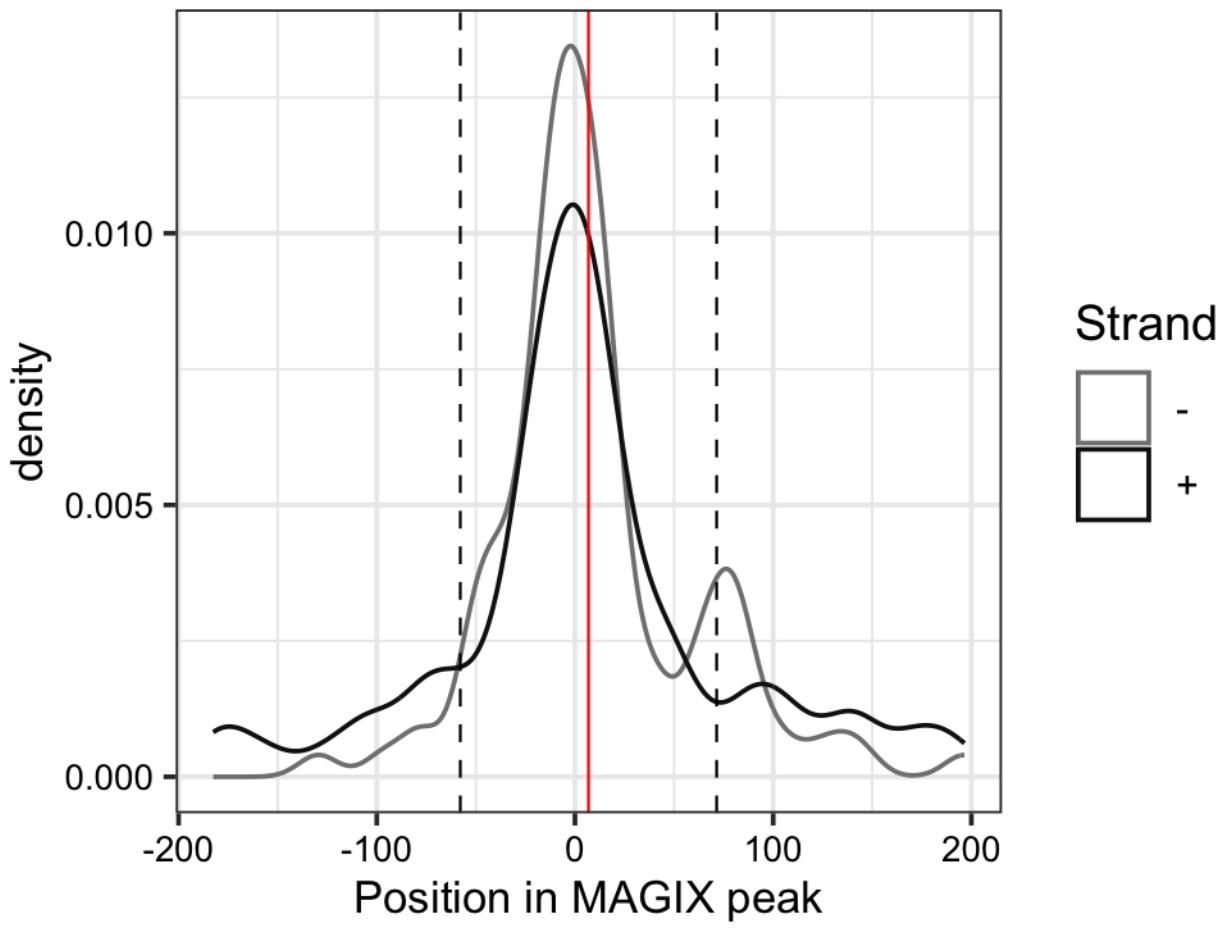

USF3

Top GRECO-BIT/MEX PWM: PWM002447

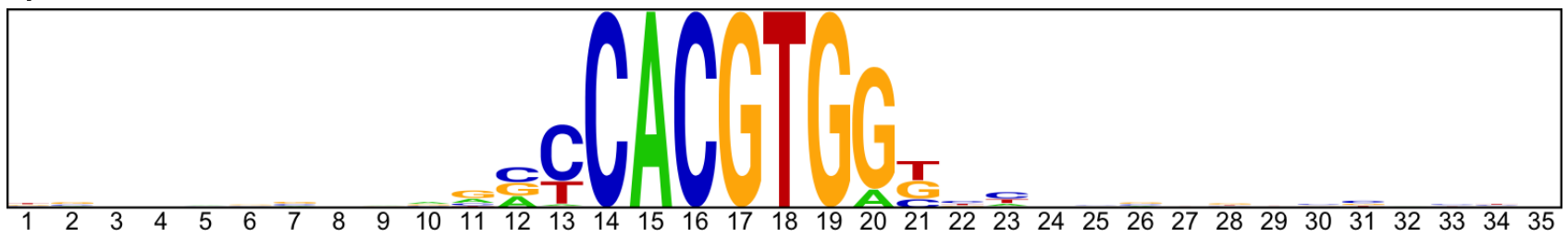

Location of top GRECO-BIT/MEX PWM in MAGIX peaks (up to the top 10,000)

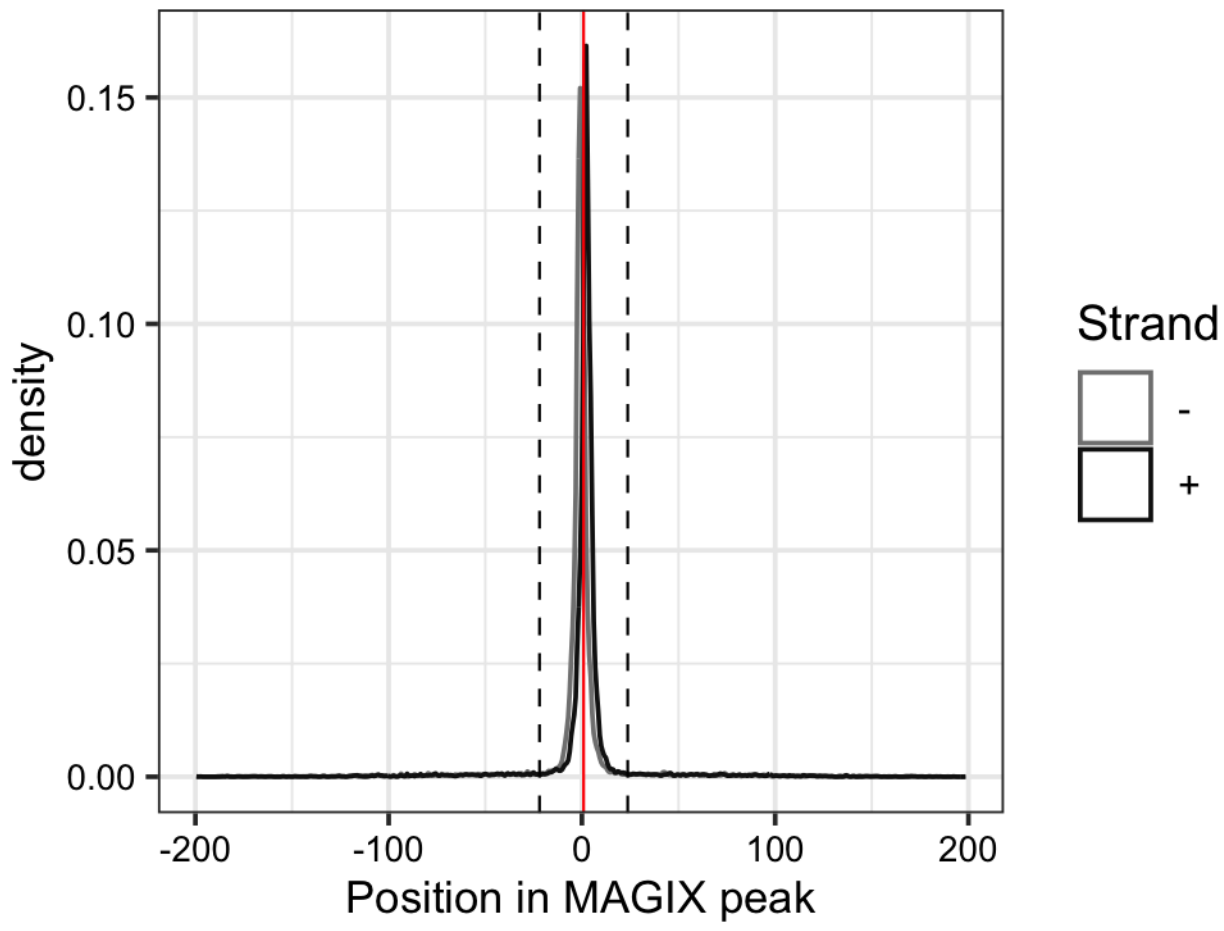

AUROC of top GRECO-BIT/MEX PWM in sliding windows (500 peaks, 50 peak step) along the MAGIX peaks (up to the top 75,000) ranked by the MAGIX enrichment coefficient and fraction of overlapping ChIP peaks in the same window

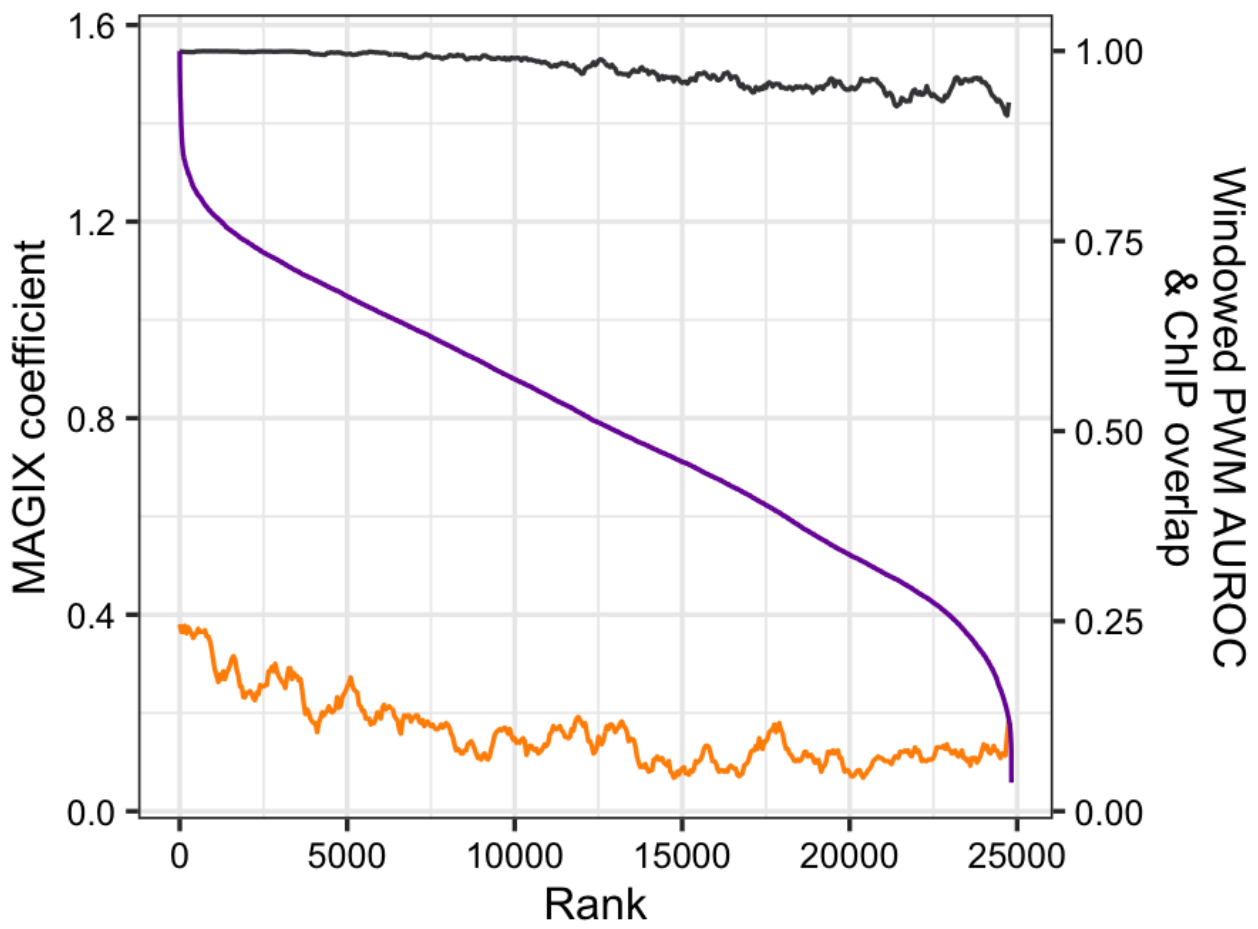

VDR

Top GRECO-BIT/MEX PWM: PWM002503

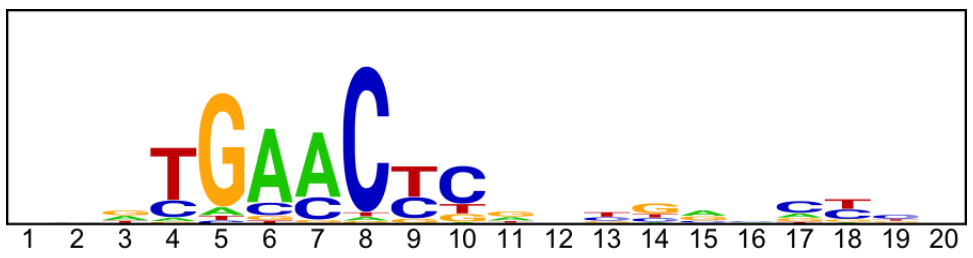

Location of top GRECO-BIT/MEX PWM in MAGIX peaks (up to the top 10,000)

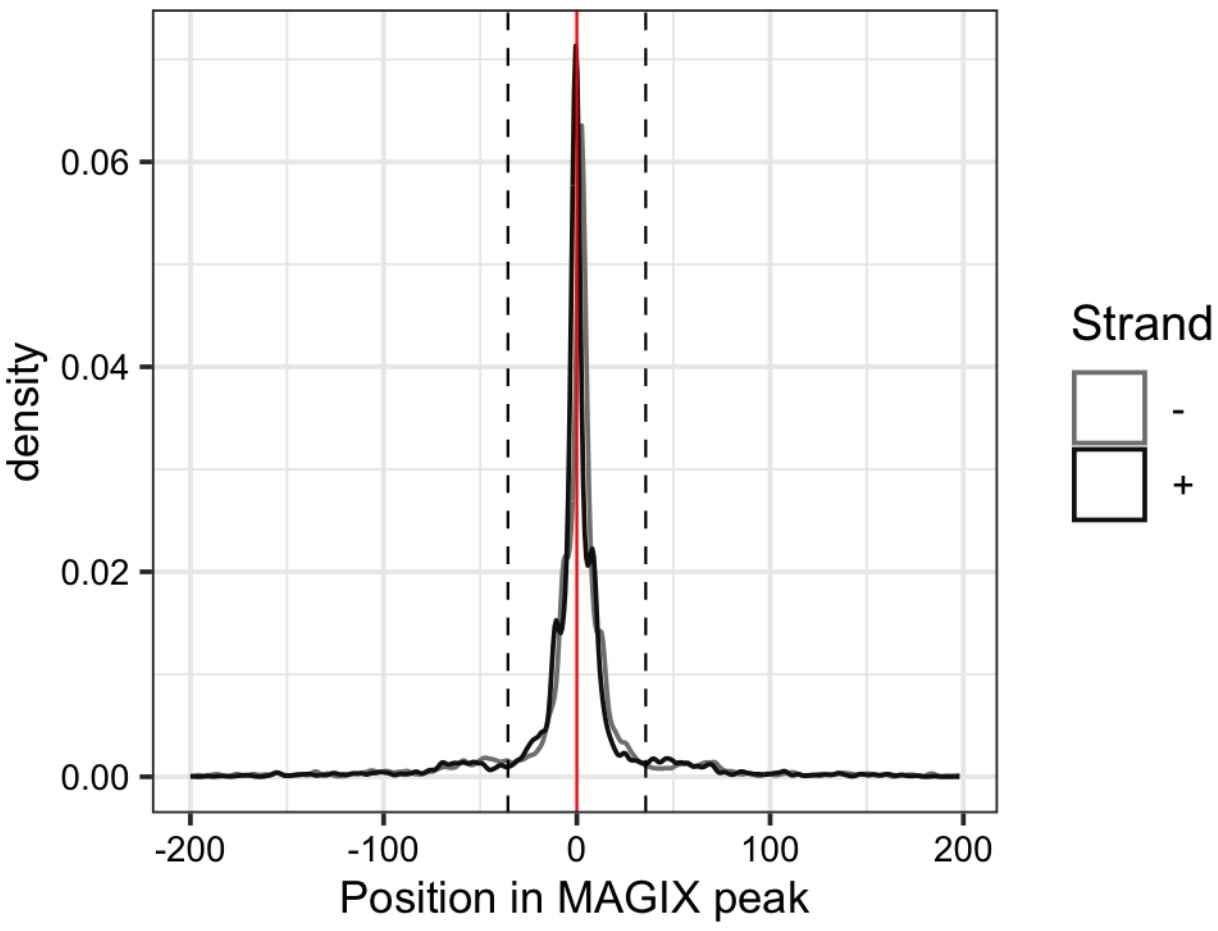

AUROC of top GRECO-BIT/MEX PWM in sliding windows (500 peaks, 50 peak step) along the MAGIX peaks (up to the top 75,000) ranked by the MAGIX enrichment coefficient and fraction of overlapping ChIP peaks in the same window

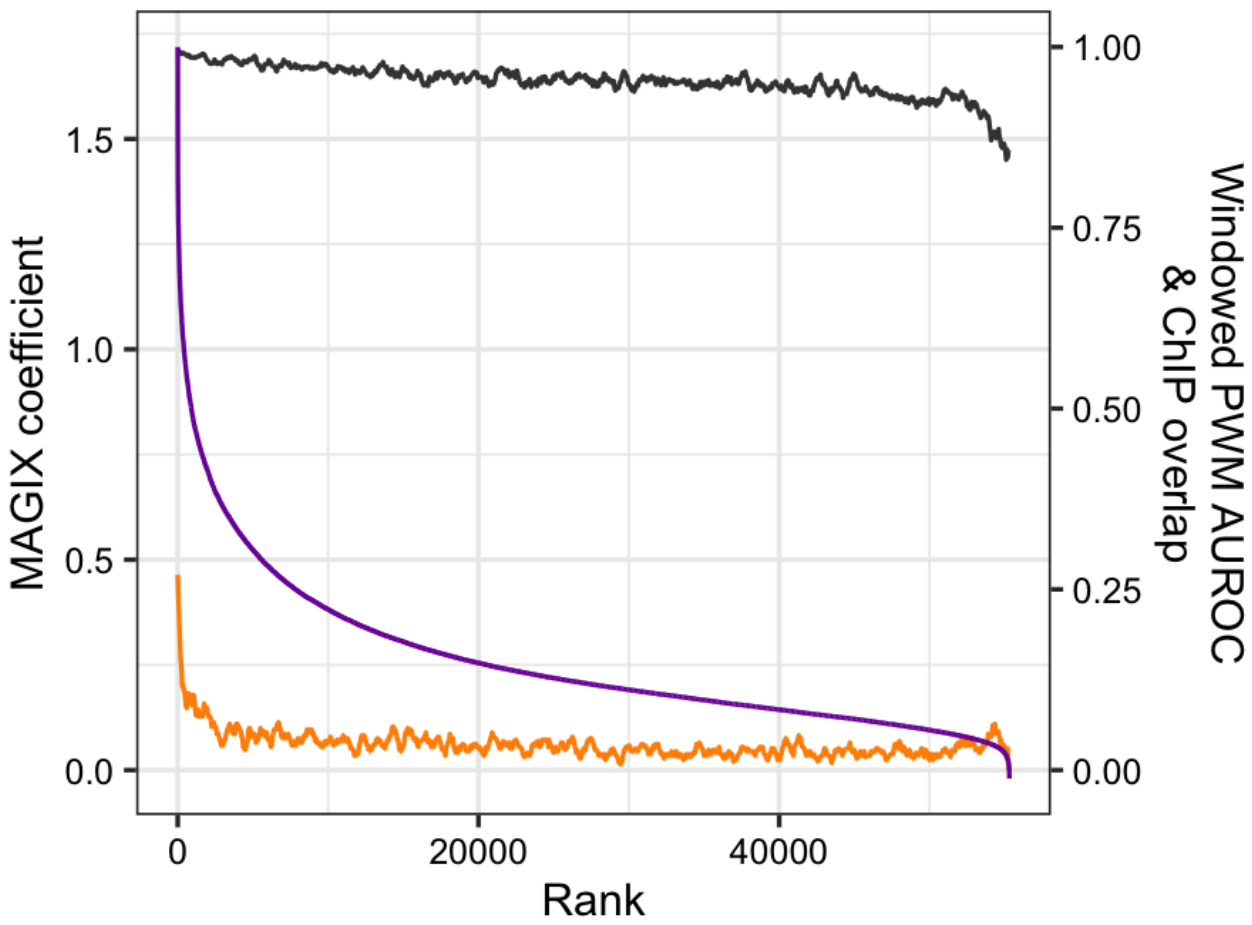

YY1

Top GRECO-BIT/MEX PWM: PWM002528

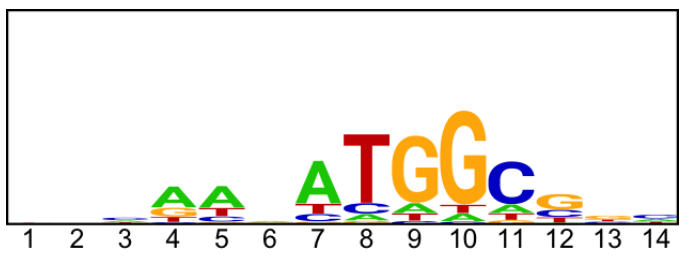

Location of top GRECO-BIT/MEX PWM in MAGIX peaks (up to the top 10,000)

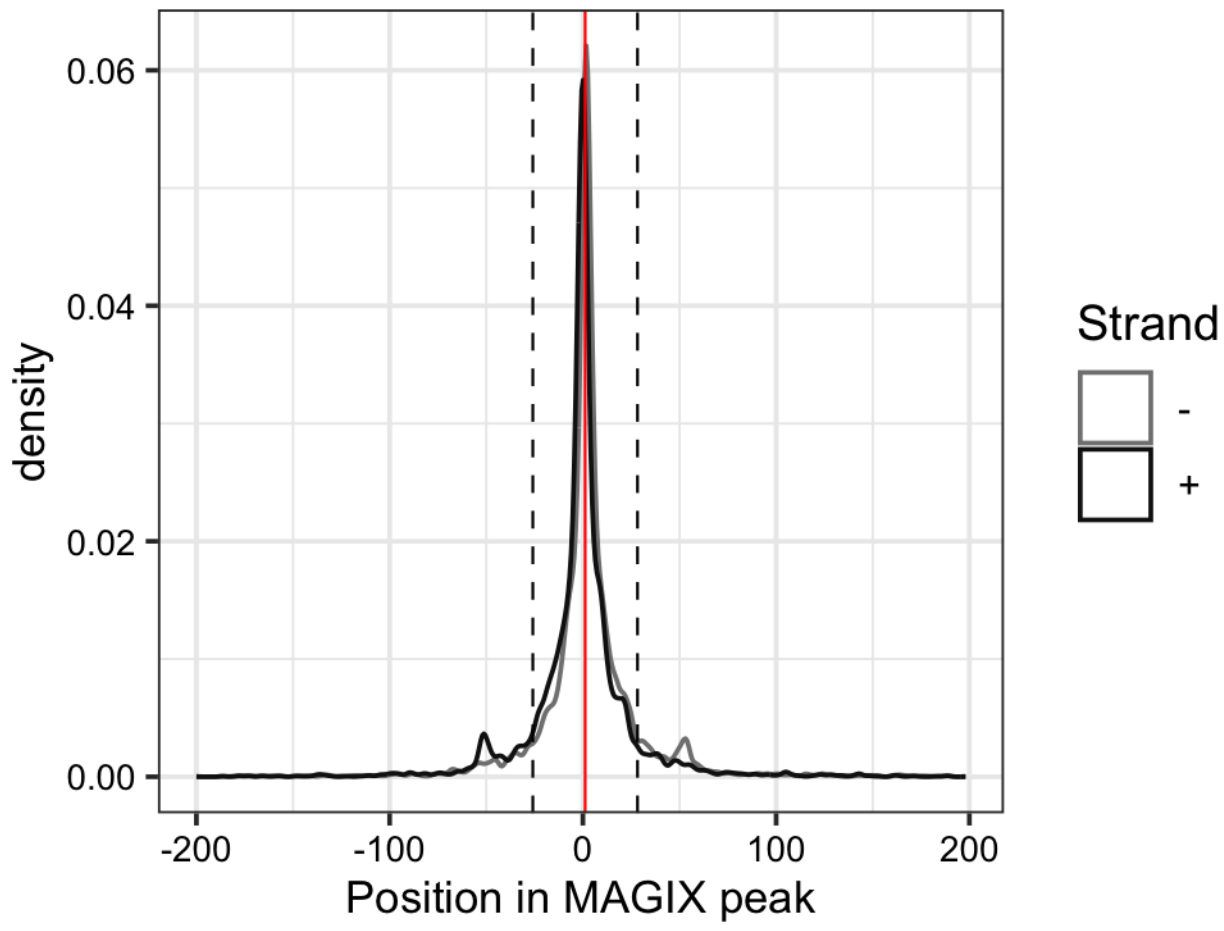

AUROC of top GRECO-BIT/MEX PWM in sliding windows (500 peaks, 50 peak step) along the MAGIX peaks (up to the top 75,000) ranked by the MAGIX enrichment coefficient and fraction of overlapping ChIP peaks in the same window

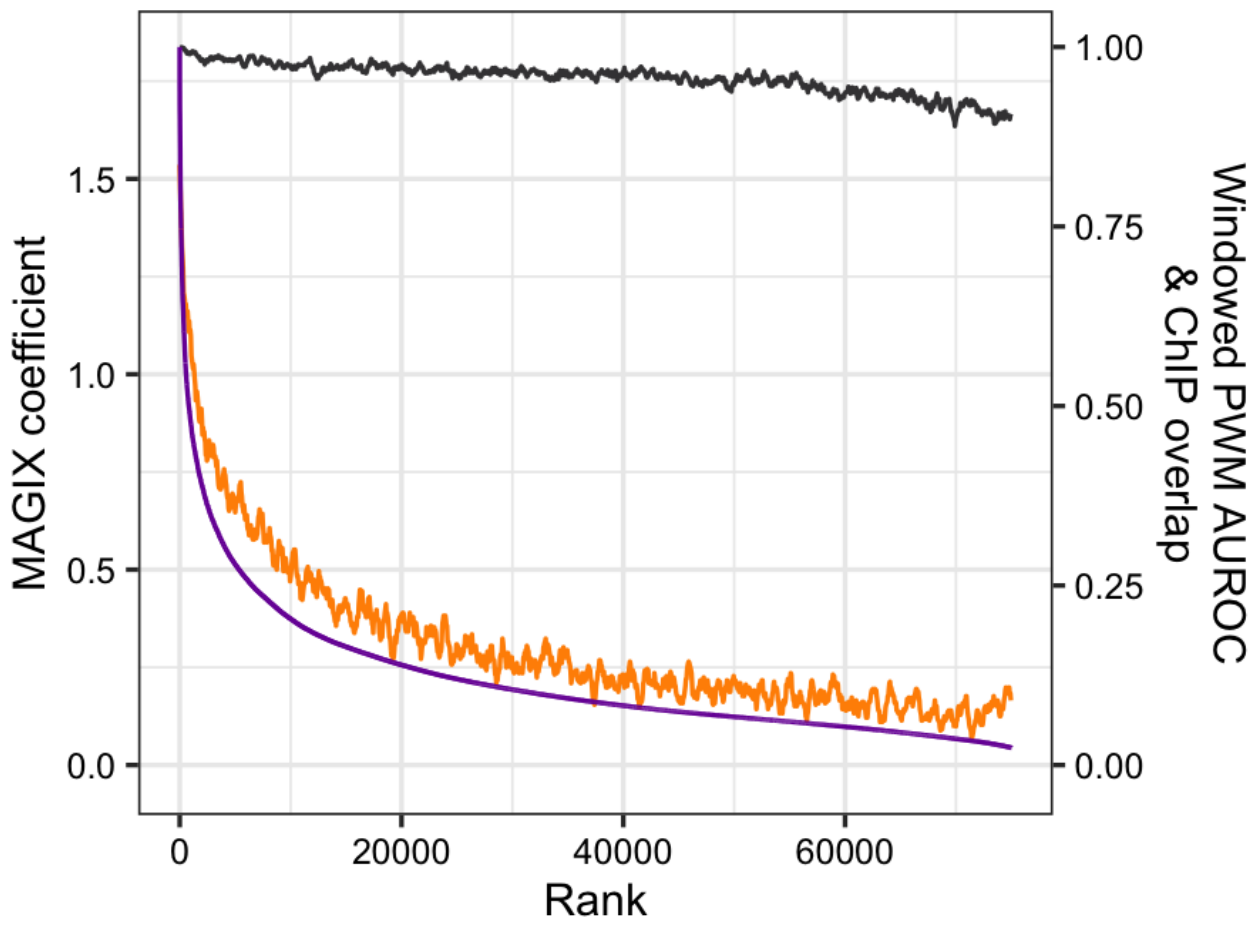

ZBED2

Top GRECO-BIT/MEX PWM: PWM002573

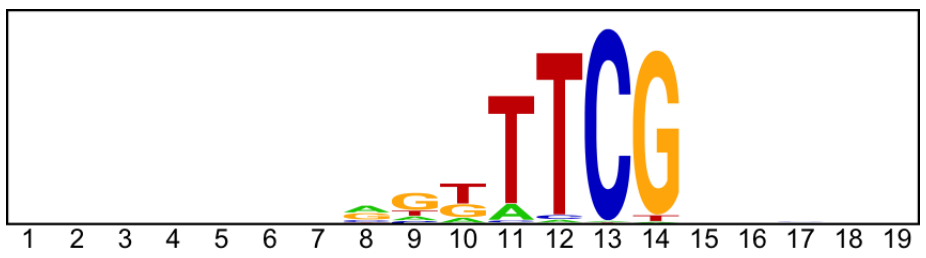

Location of top GRECO-BIT/MEX PWM in MAGIX peaks (up to the top 10,000)

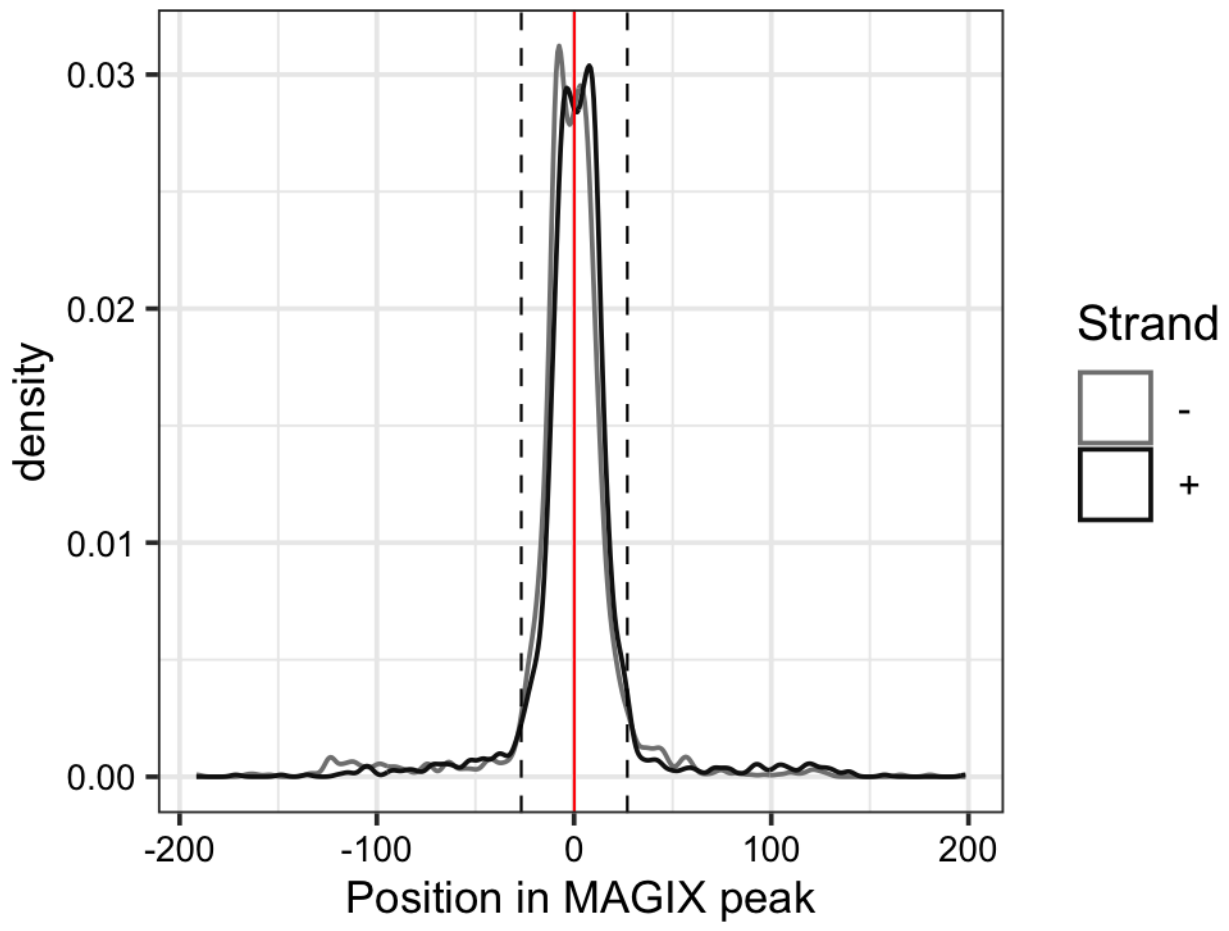

AUROC of top GRECO-BIT/MEX PWM in sliding windows (500 peaks, 50 peak step) along the MAGIX peaks (up to the top 75,000) ranked by the MAGIX enrichment coefficient and fraction of overlapping ChIP peaks in the same window

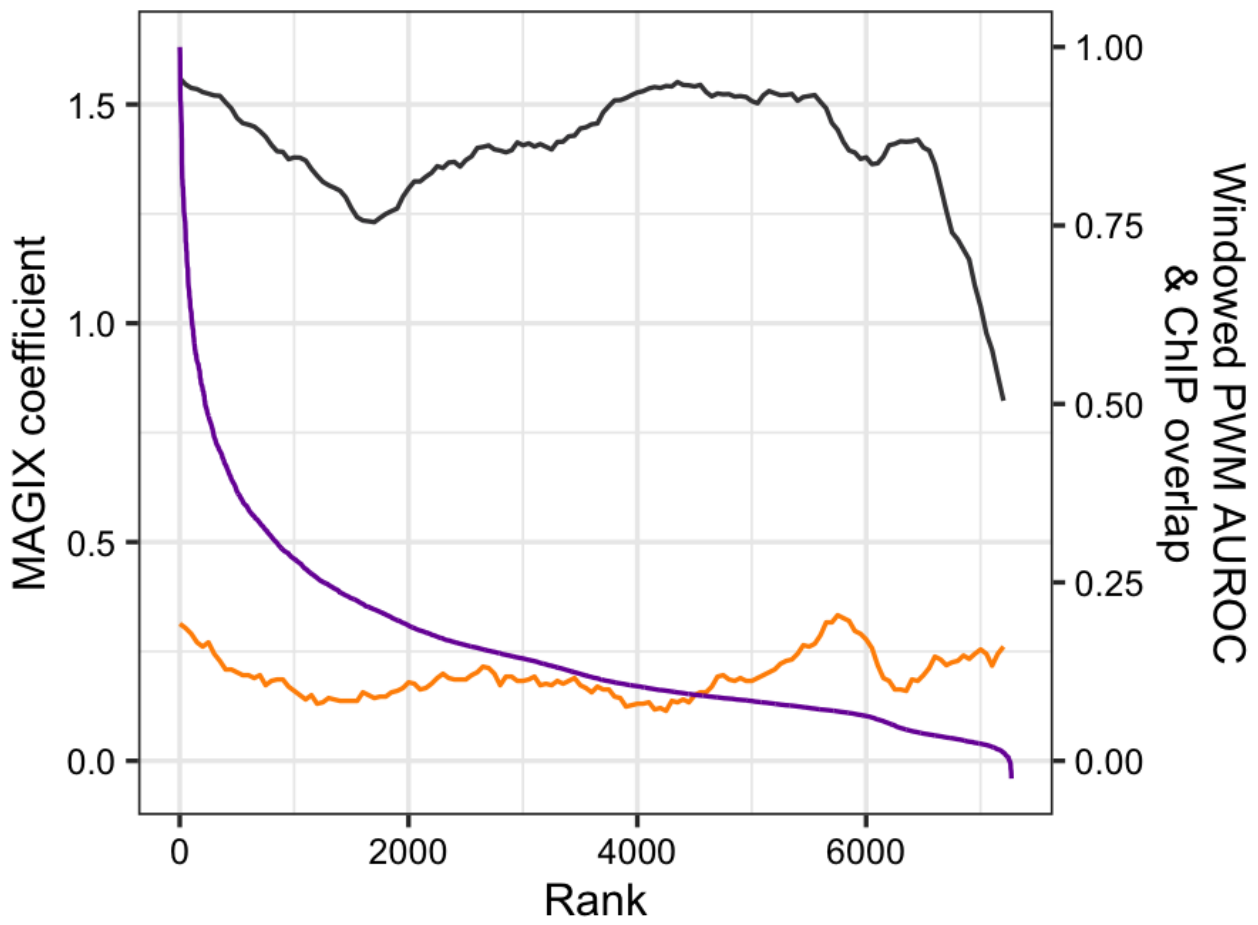

ZBED4

Top GRECO-BIT/MEX PWM: PWM002593

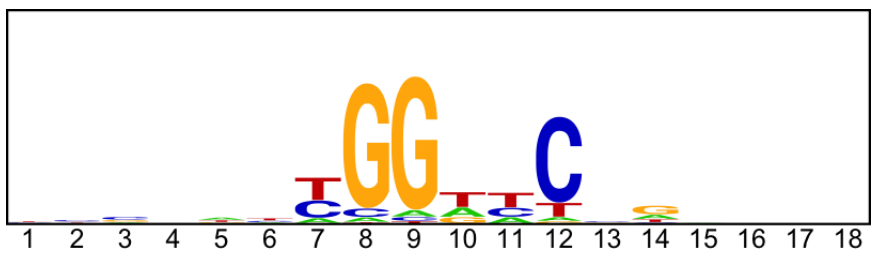

Location of top GRECO-BIT/MEX PWM in MAGIX peaks (up to the top 10,000)

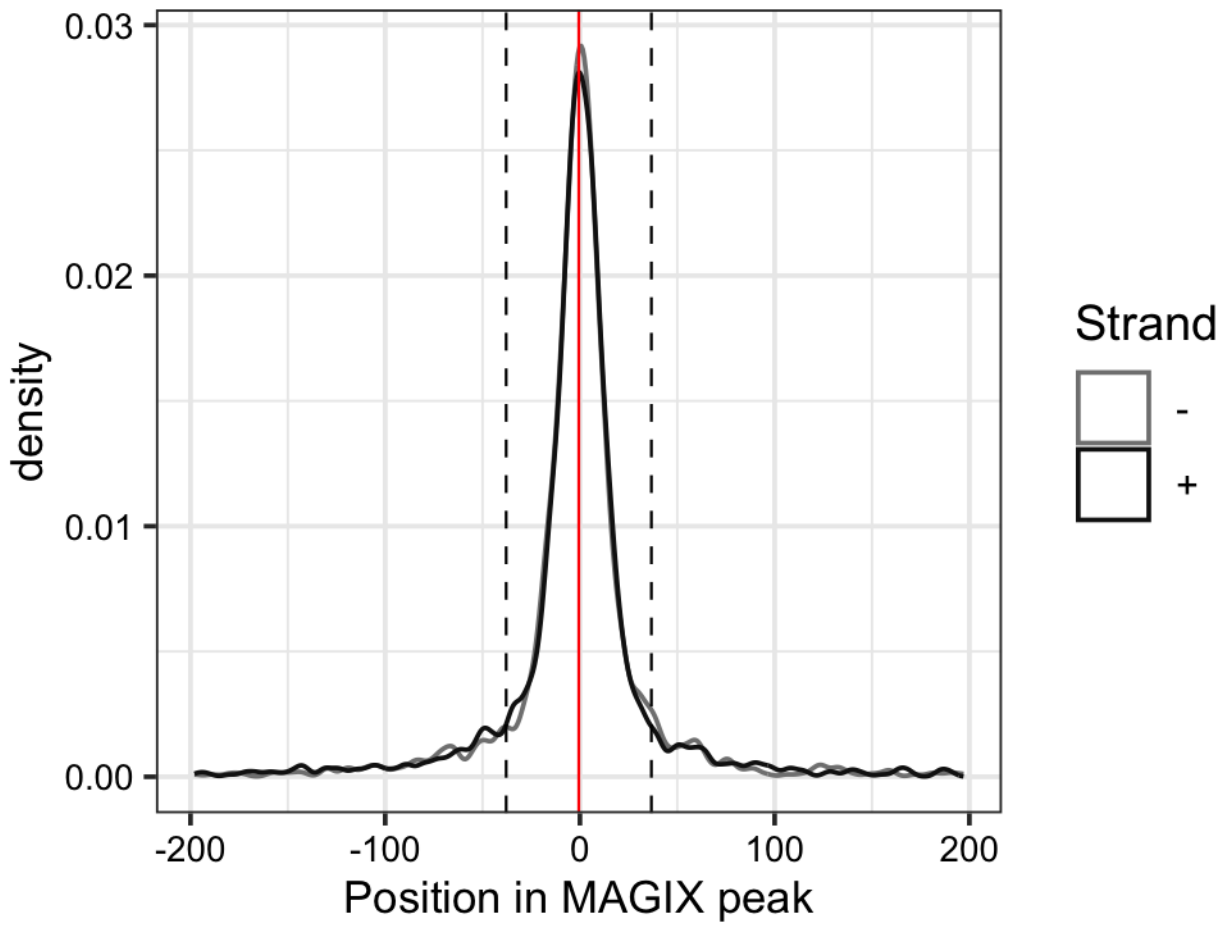

ZBED5

Top GRECO-BIT/MEX PWM: PWM002641

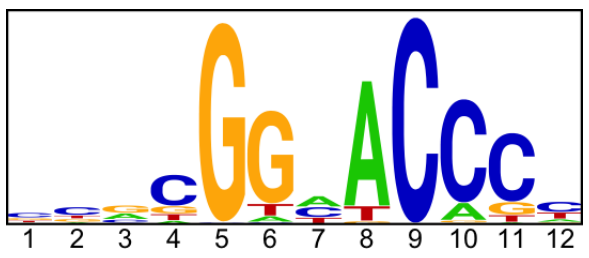

Location of top GRECO-BIT/MEX PWM in MAGIX peaks (up to the top 10,000)

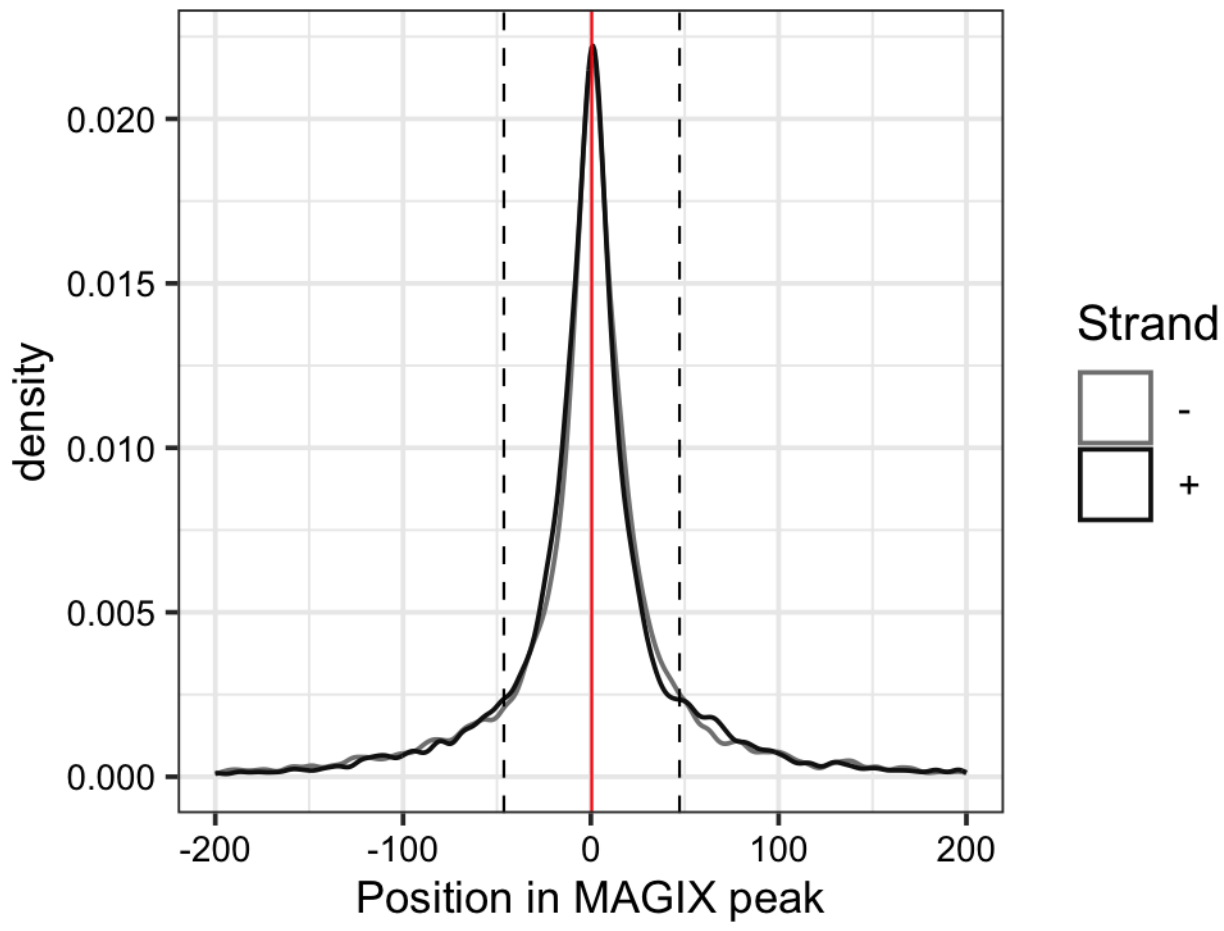

AUROC of top GRECO-BIT/MEX PWM in sliding windows (500 peaks, 50 peak step) along the MAGIX peaks (up to the top 75,000) ranked by the MAGIX enrichment coefficient and fraction of overlapping ChIP peaks in the same window

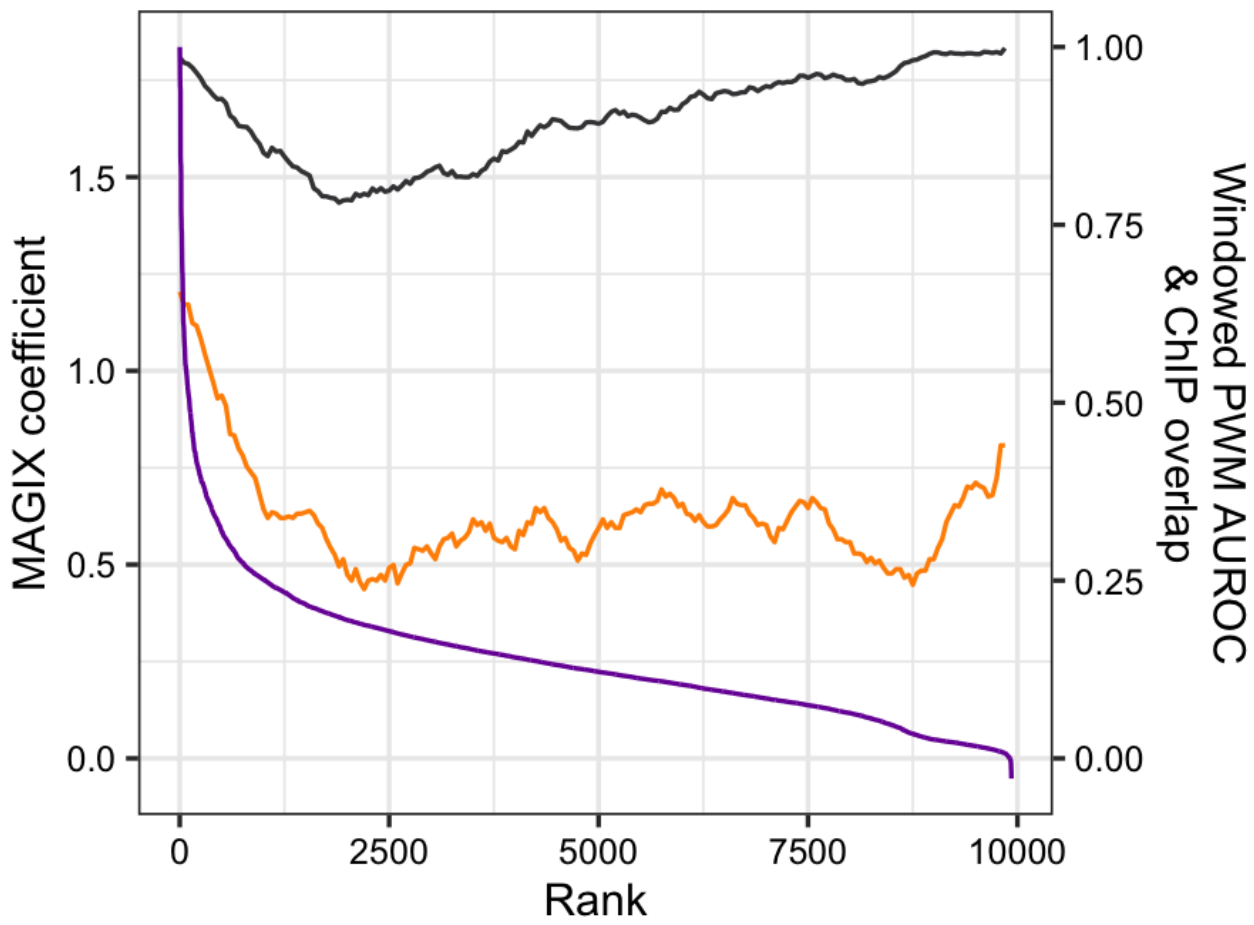

ZBED9

Top GRECO-BIT/MEX PWM: PWM002687

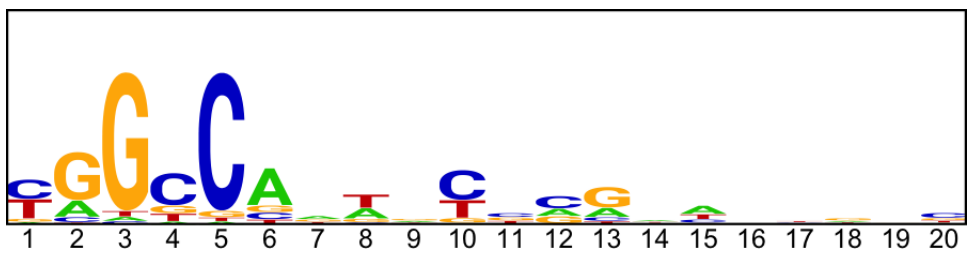

Location of top GRECO-BIT/MEX PWM in MAGIX peaks (up to the top 10,000)

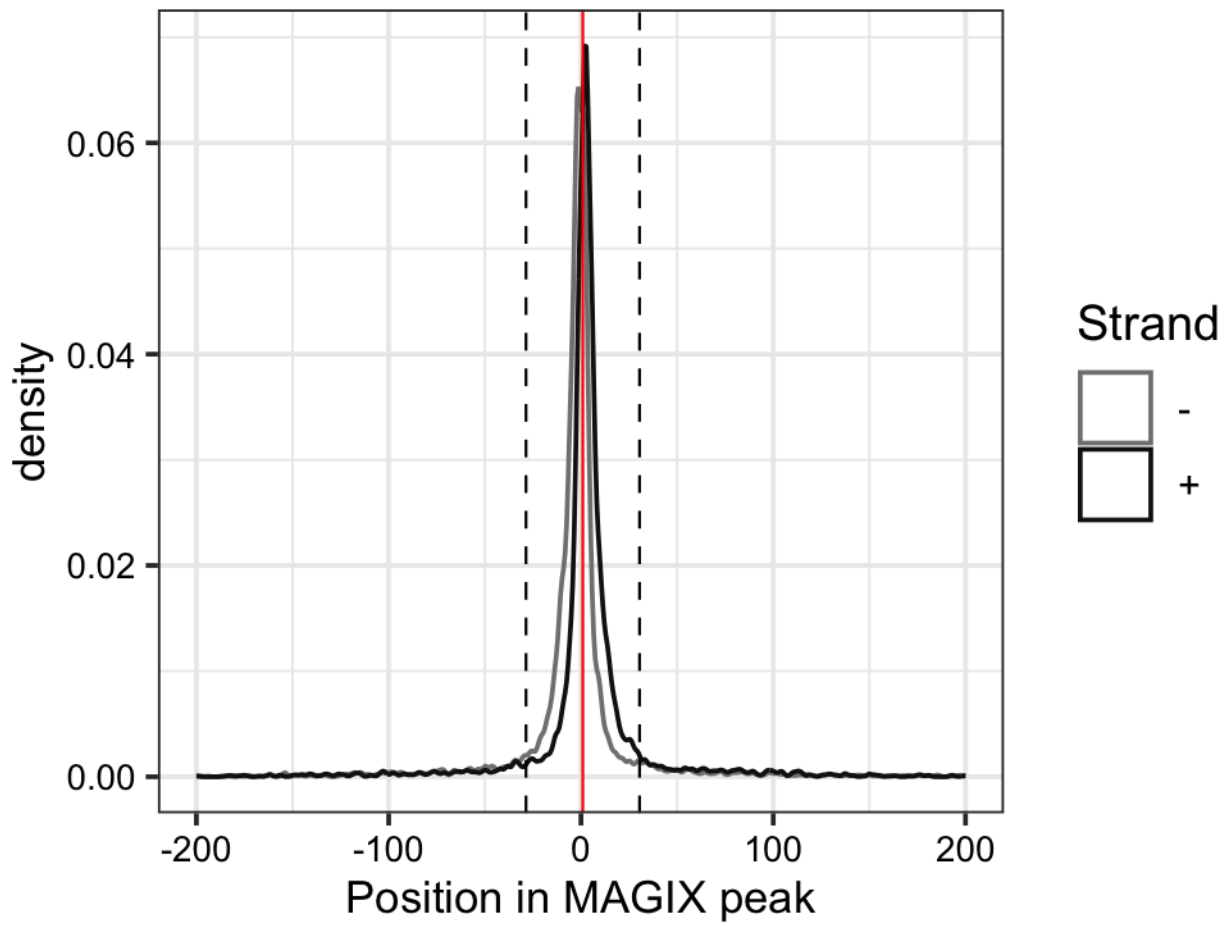

AUROC of top GRECO-BIT/MEX PWM in sliding windows (500 peaks, 50 peak step) along the MAGIX peaks (up to the top 75,000) ranked by the MAGIX enrichment coefficient and fraction of overlapping ChIP peaks in the same window

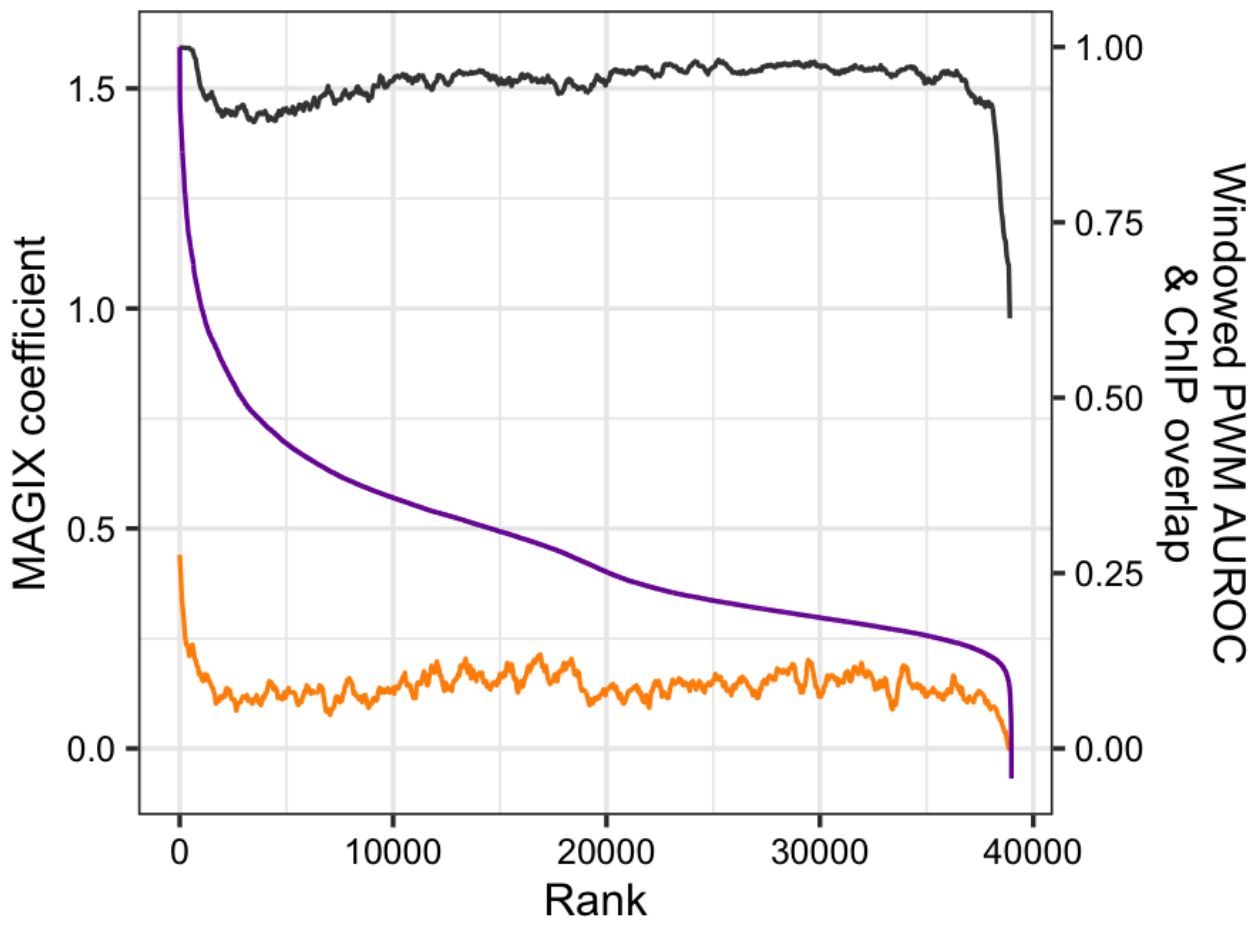

ZBTB24

Top GRECO-BIT/MEX PWM: PWM002706

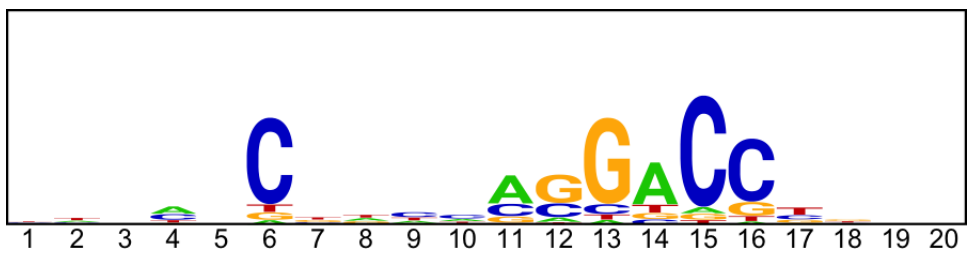

Location of top GRECO-BIT/MEX PWM in MAGIX peaks (up to the top 10,000)

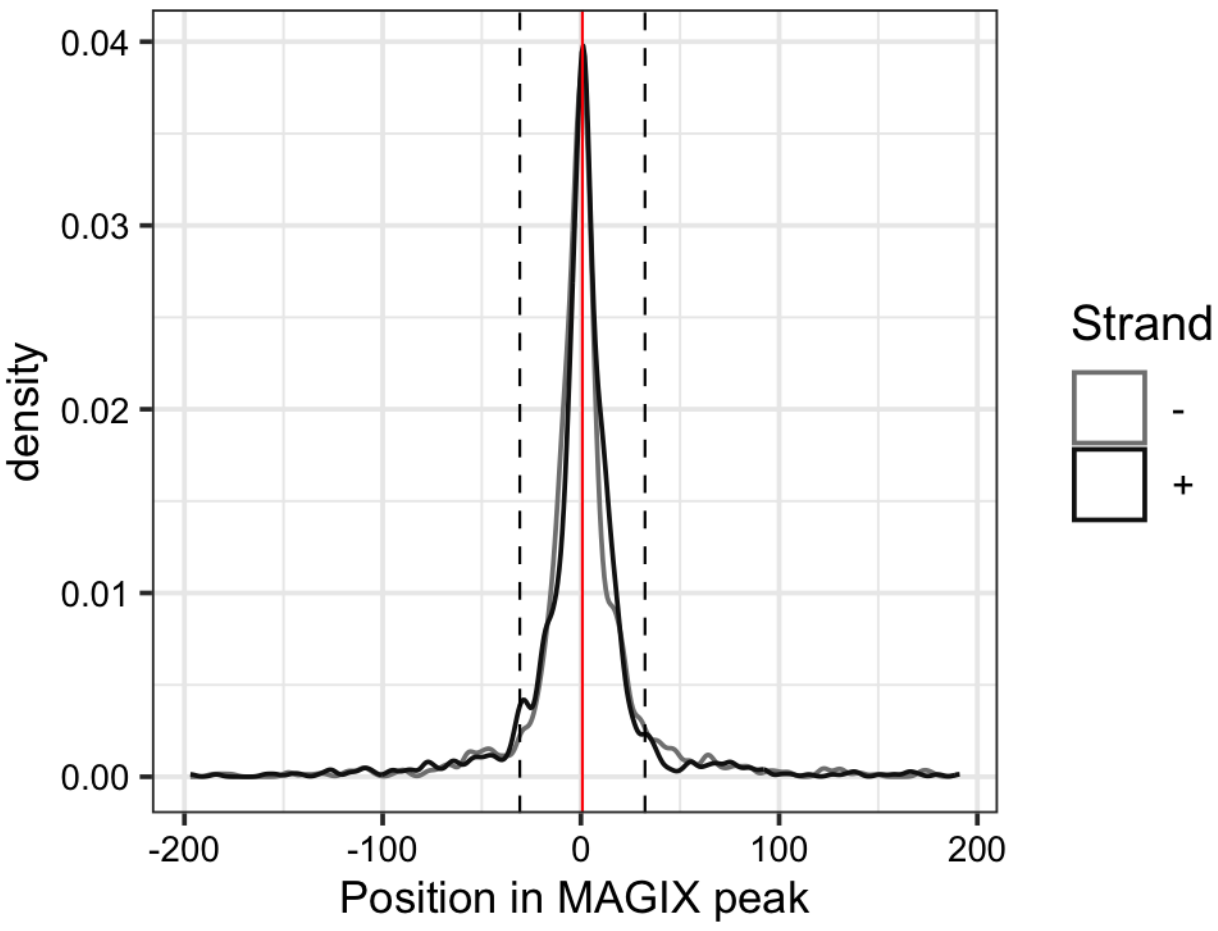

ZBTB40

Top GRECO-BIT/MEX PWM: PWM002730

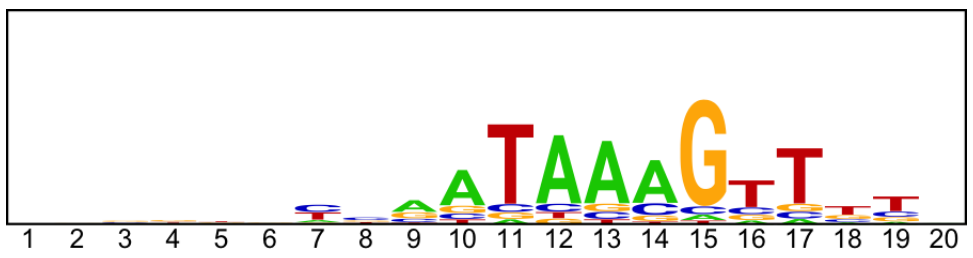

Location of top GRECO-BIT/MEX PWM in MAGIX peaks (up to the top 10,000)

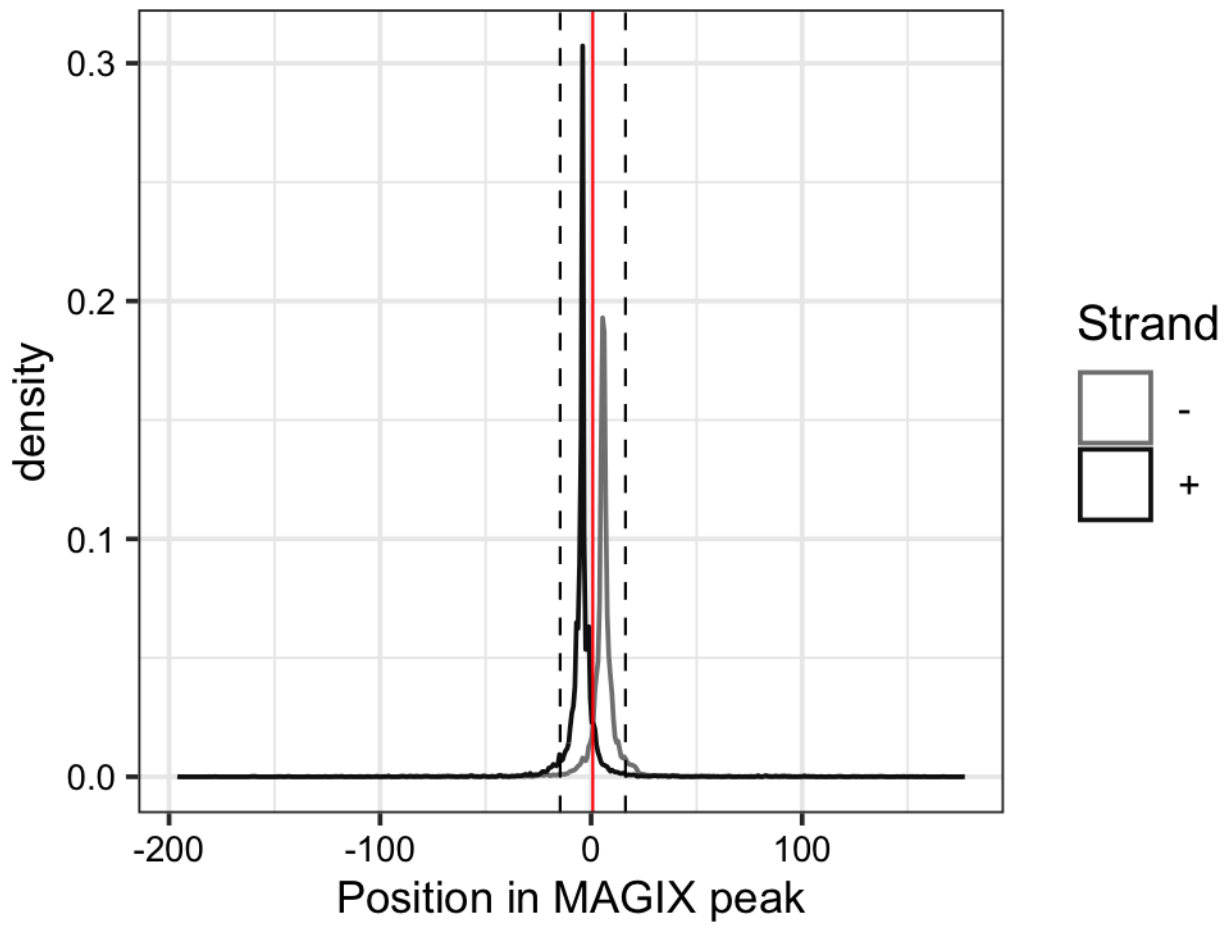

AUROC of top GRECO-BIT/MEX PWM in sliding windows (500 peaks, 50 peak step) along the MAGIX peaks (up to the top 75,000) ranked by the MAGIX enrichment coefficient and fraction of overlapping ChIP peaks in the same window

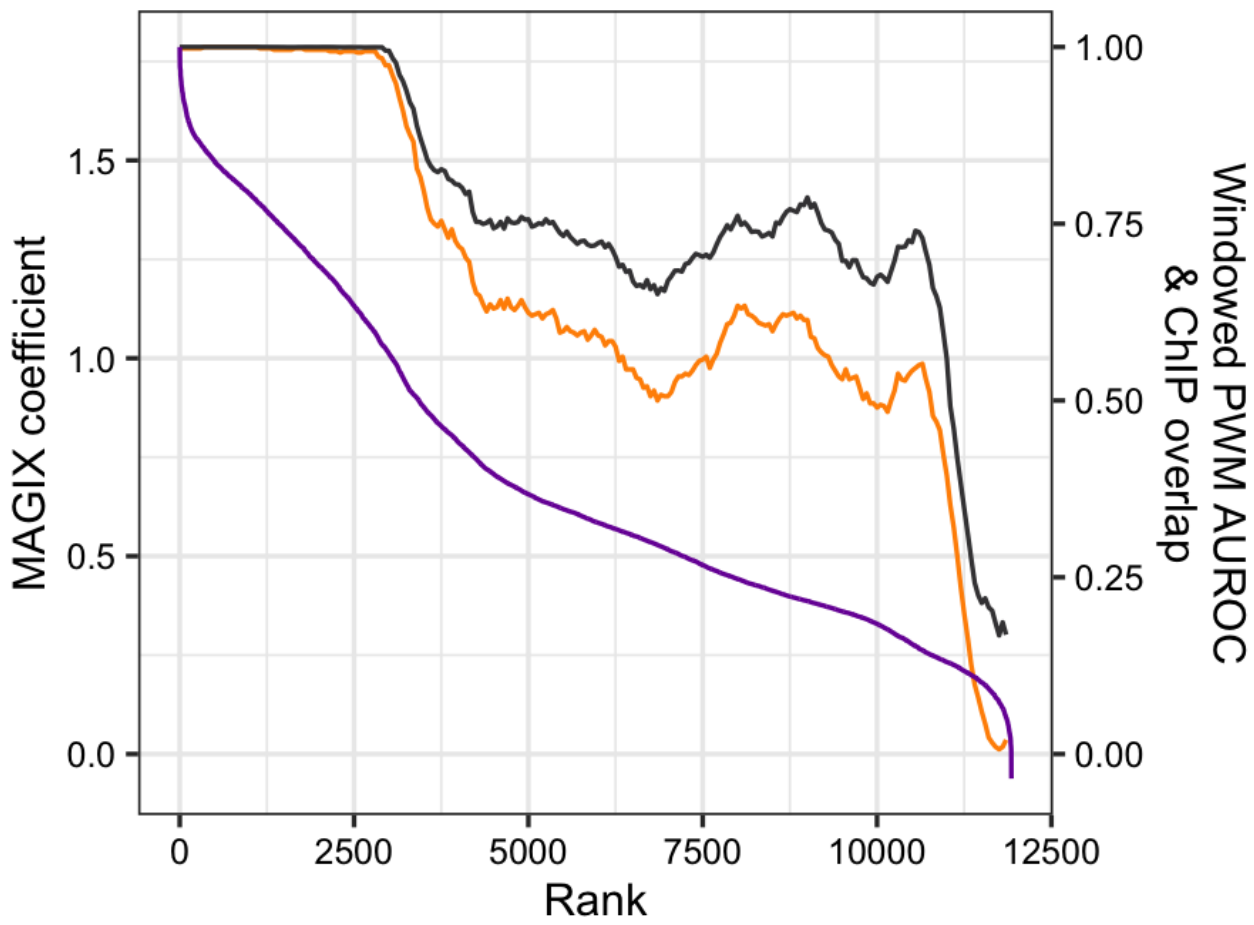

ZBTB41

Top GRECO-BIT/MEX PWM: PWM002773

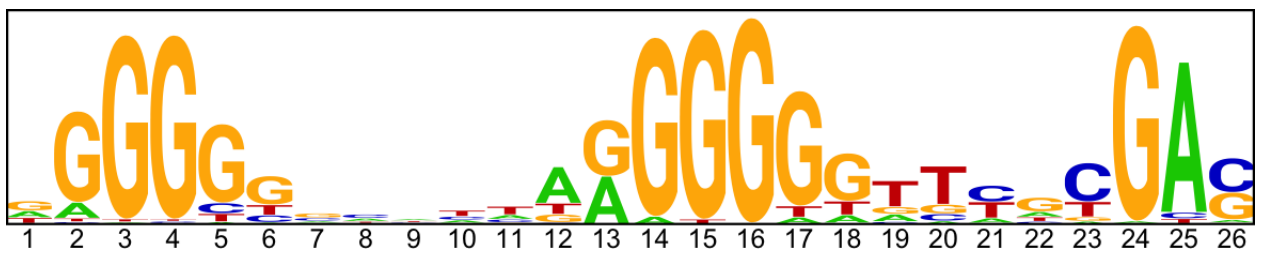

Location of top GRECO-BIT/MEX PWM in MAGIX peaks (up to the top 10,000)

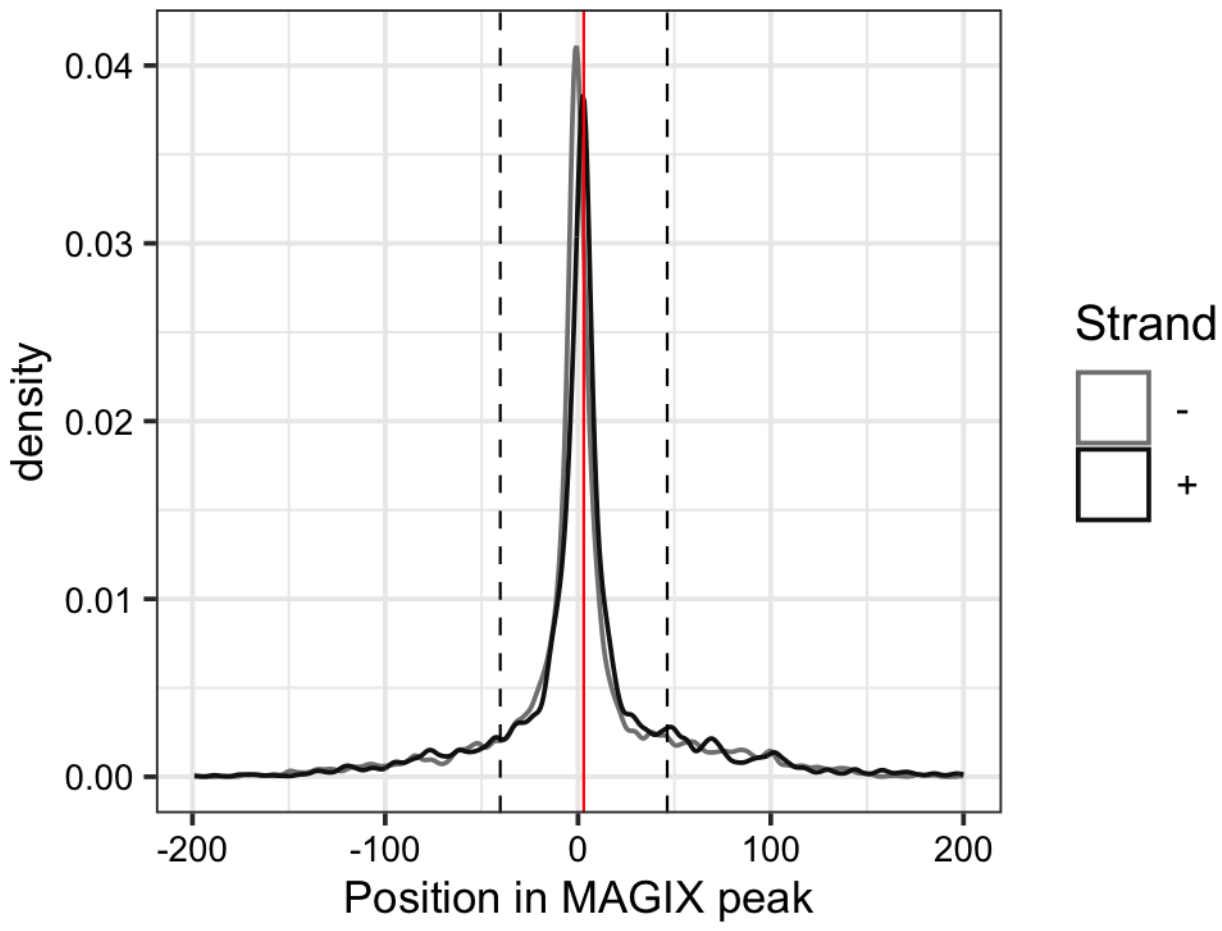

AUROC of top GRECO-BIT/MEX PWM in sliding windows (500 peaks, 50 peak step) along the MAGIX peaks (up to the top 75,000) ranked by the MAGIX enrichment coefficient and fraction of overlapping ChIP peaks in the same window

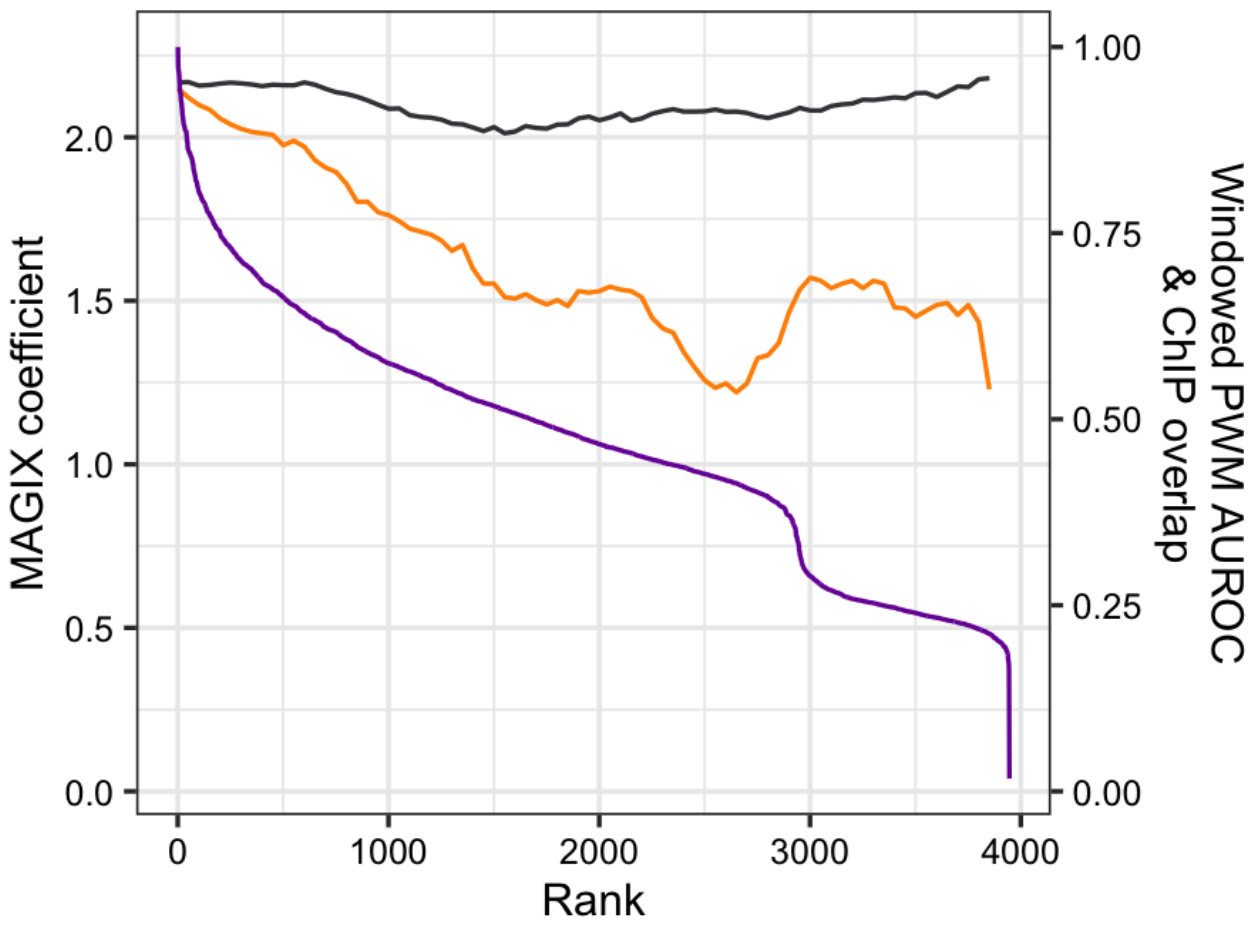

ZBTB47

Top GRECO-BIT/MEX PWM: PWM002799

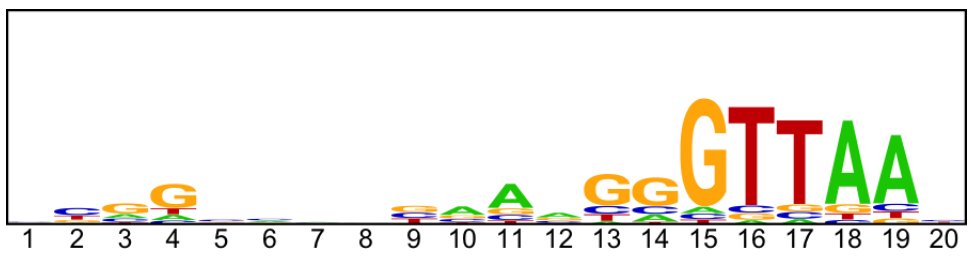

Location of top GRECO-BIT/MEX PWM in MAGIX peaks (up to the top 10,000)

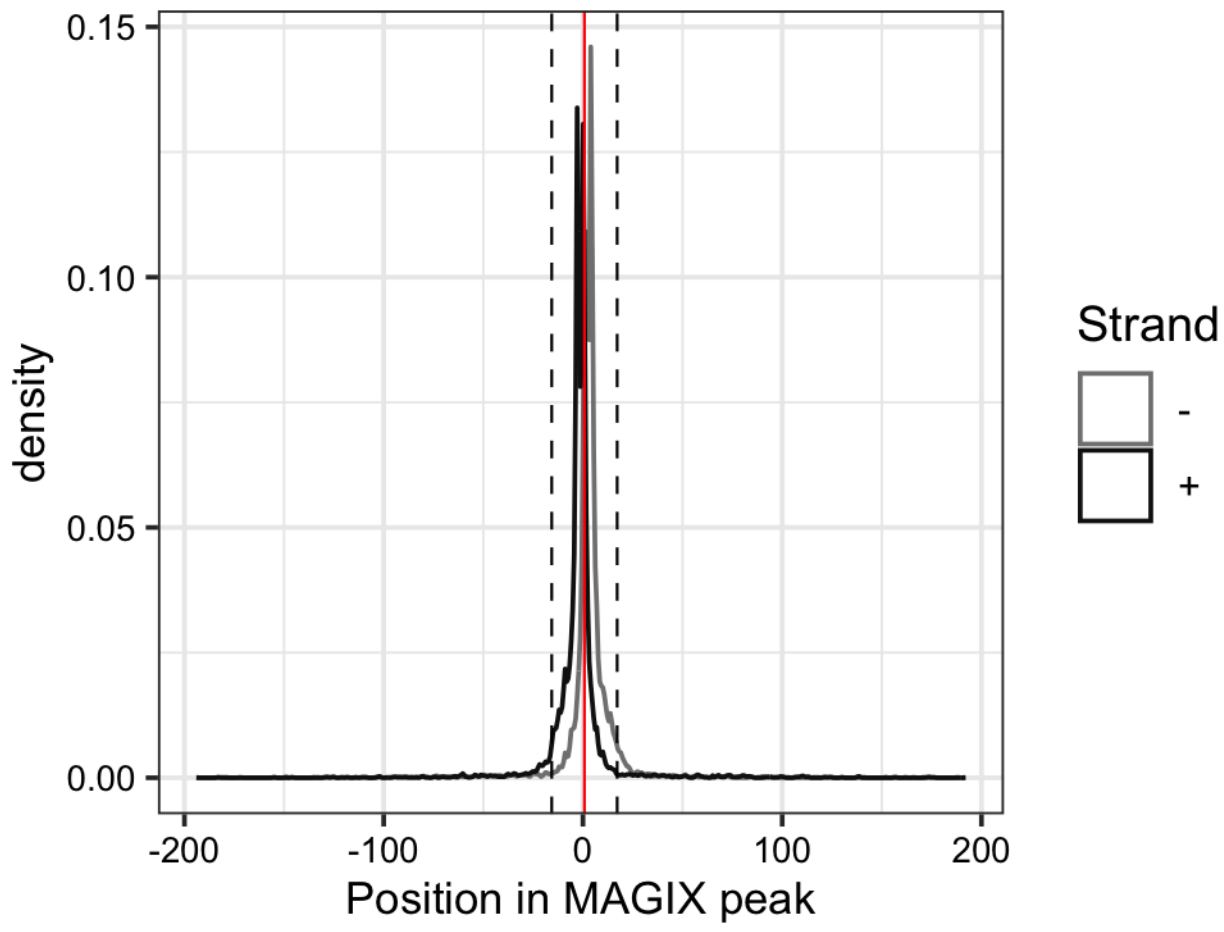

AUROC of top GRECO-BIT/MEX PWM in sliding windows (500 peaks, 50 peak step) along the MAGIX peaks (up to the top 75,000) ranked by the MAGIX enrichment coefficient and fraction of overlapping ChIP peaks in the same window

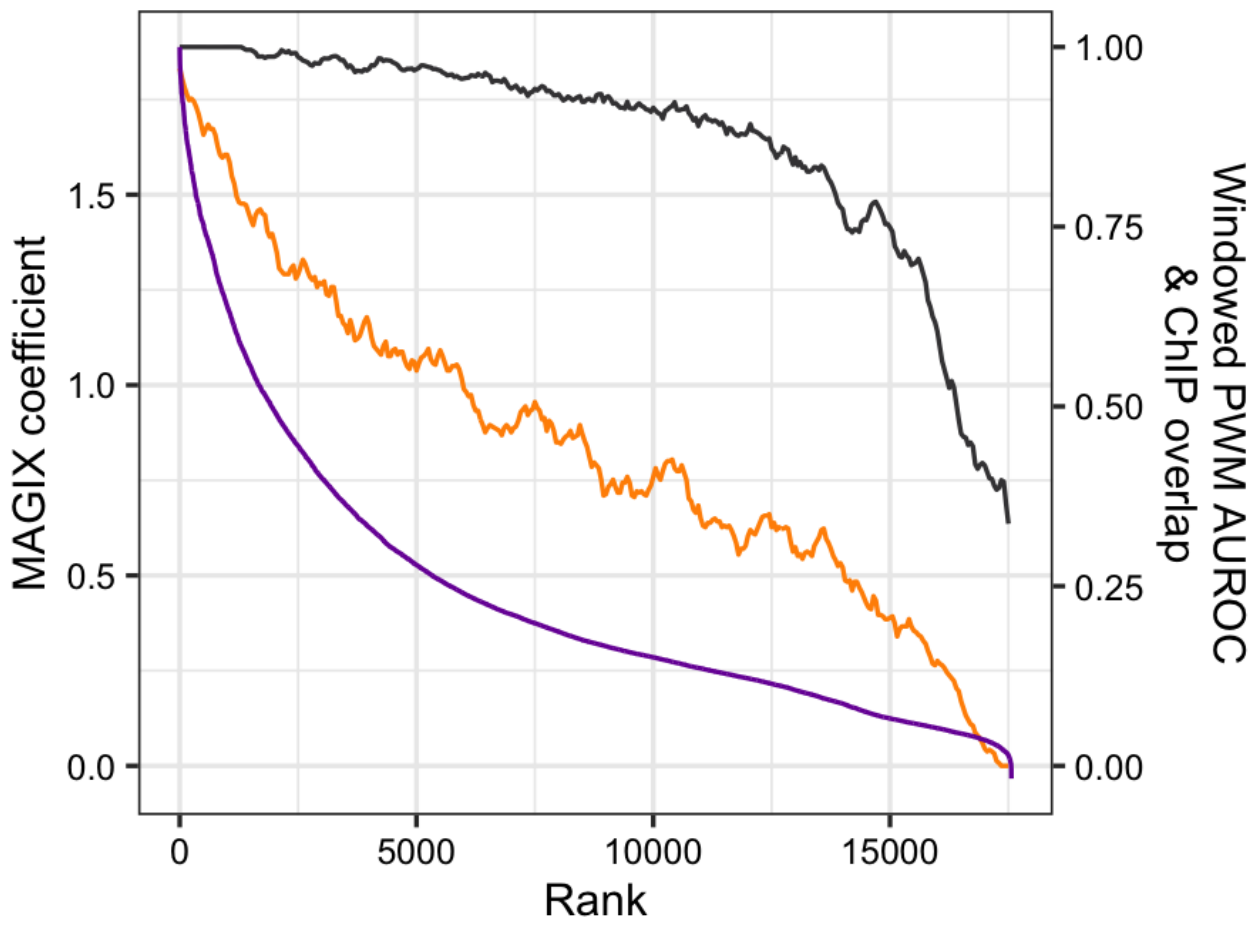

ZBTB5

Top GRECO-BIT/MEX PWM: PWM002811

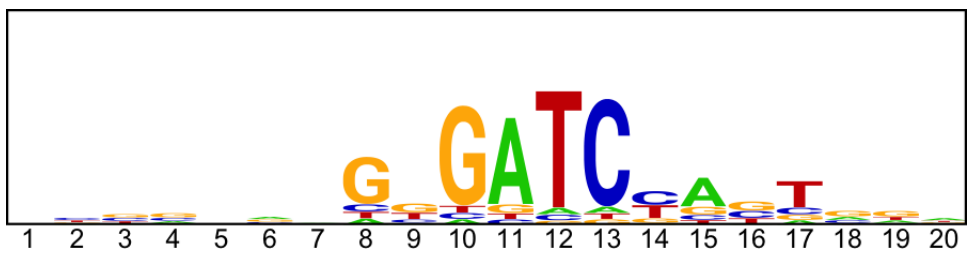

Location of top GRECO-BIT/MEX PWM in MAGIX peaks (up to the top 10,000)

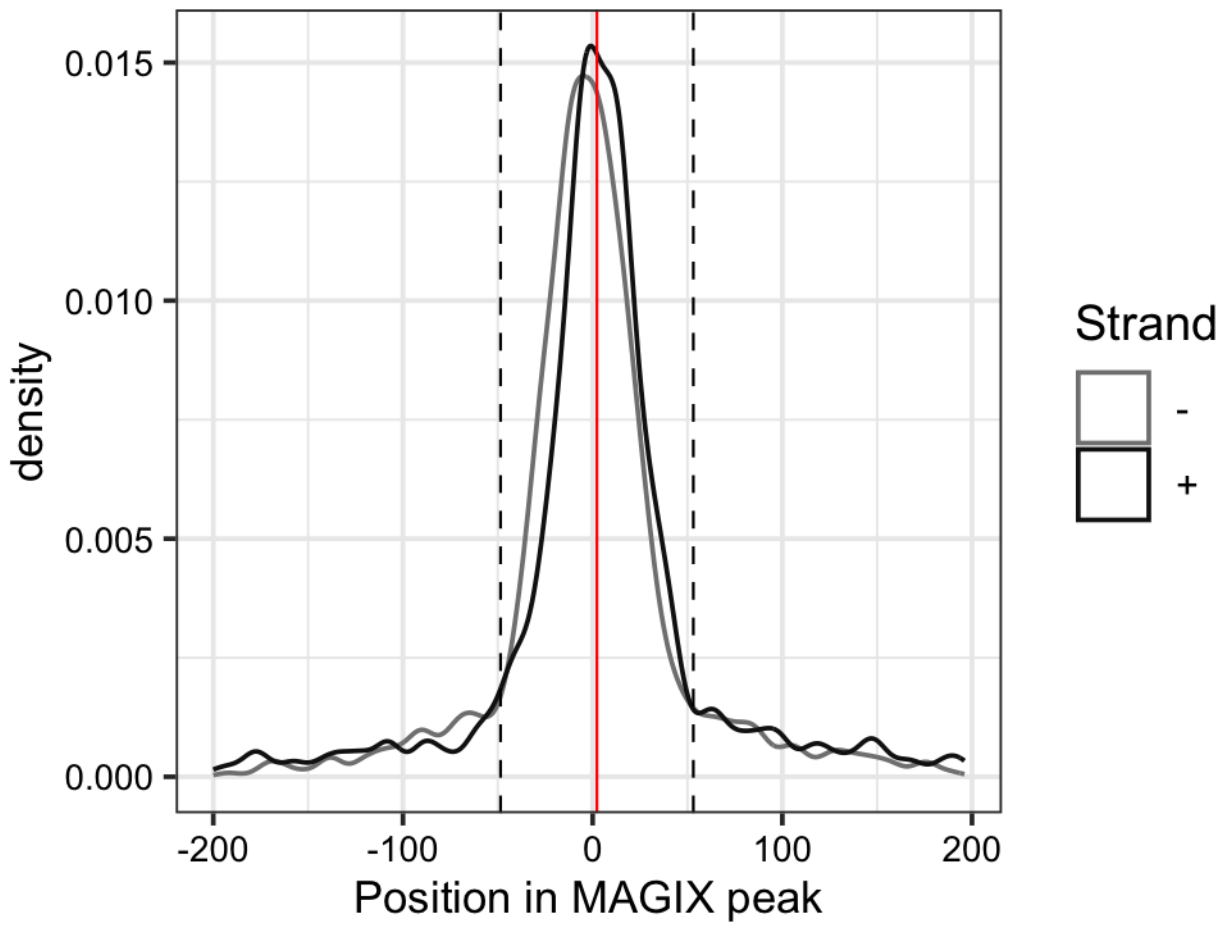

ZBTB8A

Top GRECO-BIT/MEX PWM: PWM002842

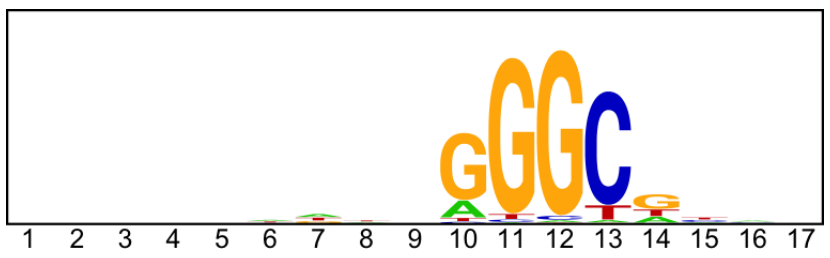

Location of top GRECO-BIT/MEX PWM in MAGIX peaks (up to the top 10,000)

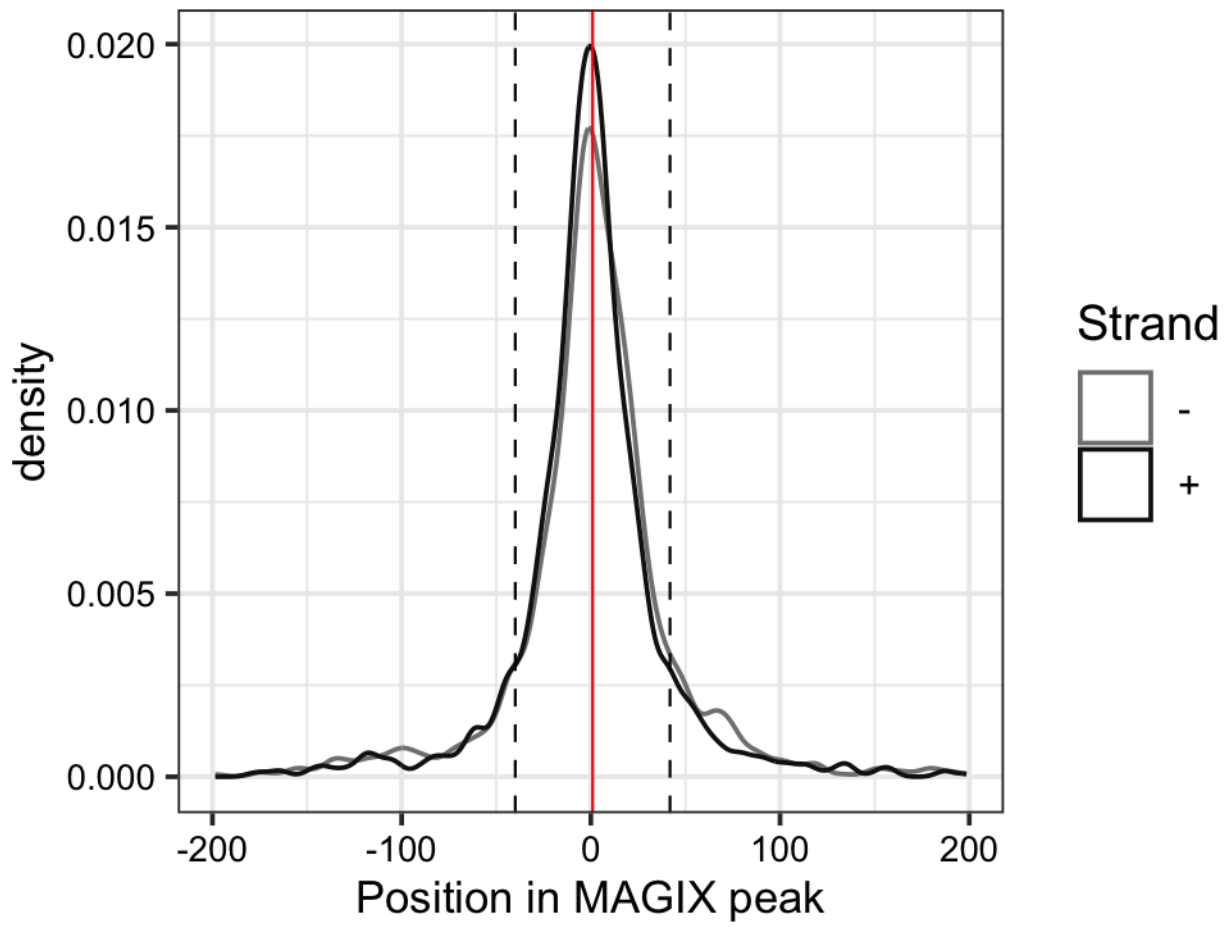

AUROC of top GRECO-BIT/MEX PWM in sliding windows (500 peaks, 50 peak step) along the MAGIX peaks (up to the top 75,000) ranked by the MAGIX enrichment coefficient and fraction of overlapping ChIP peaks in the same window

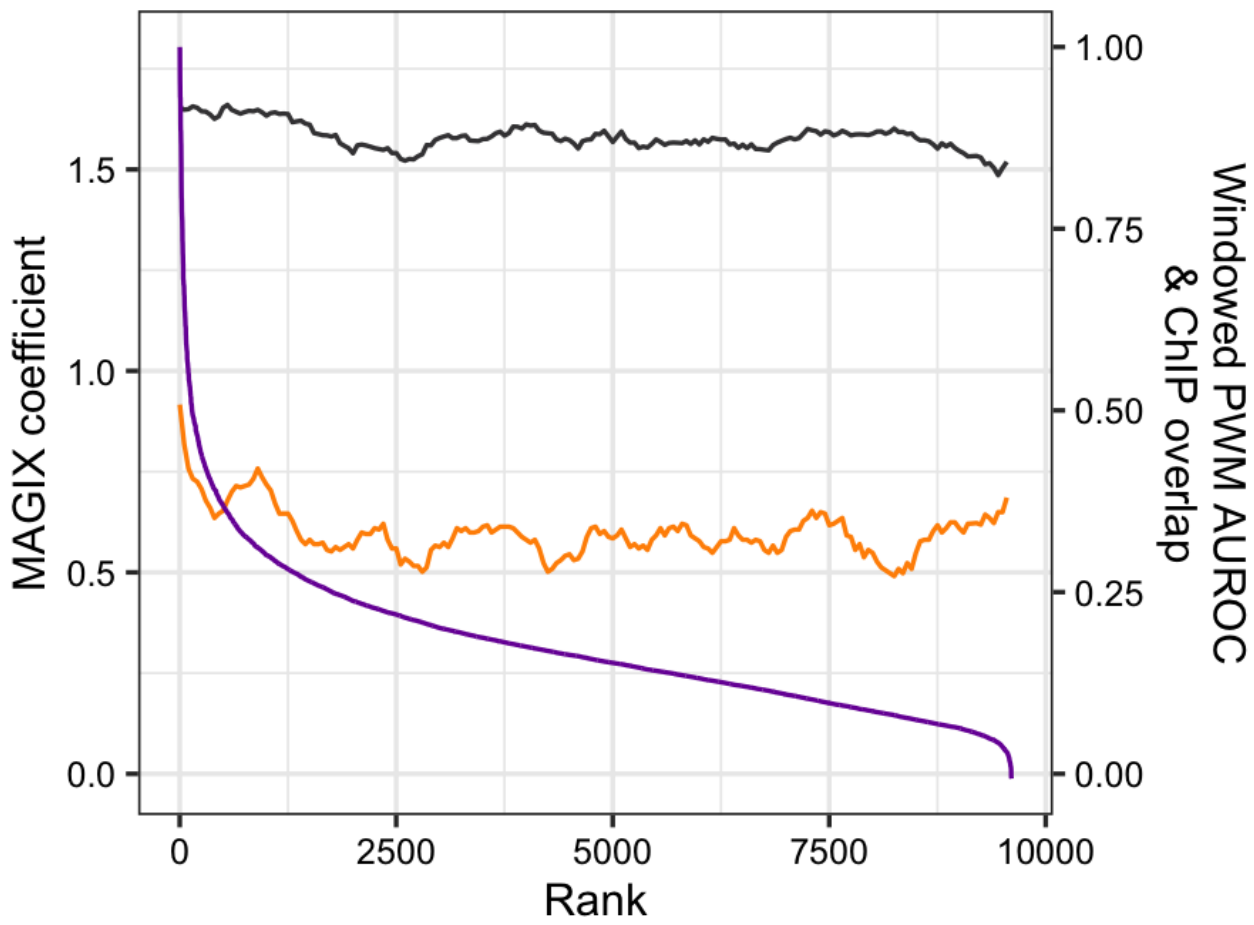

ZBTB8B

Top GRECO-BIT/MEX PWM: PWM002878

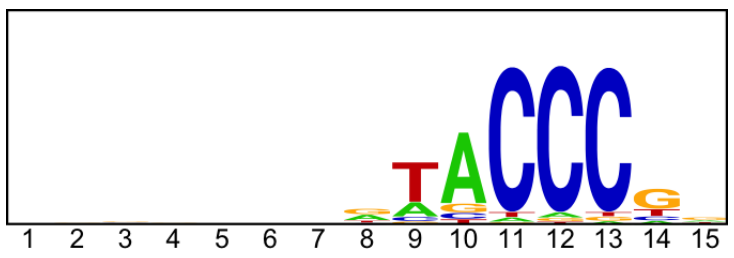

Location of top GRECO-BIT/MEX PWM in MAGIX peaks (up to the top 10,000)

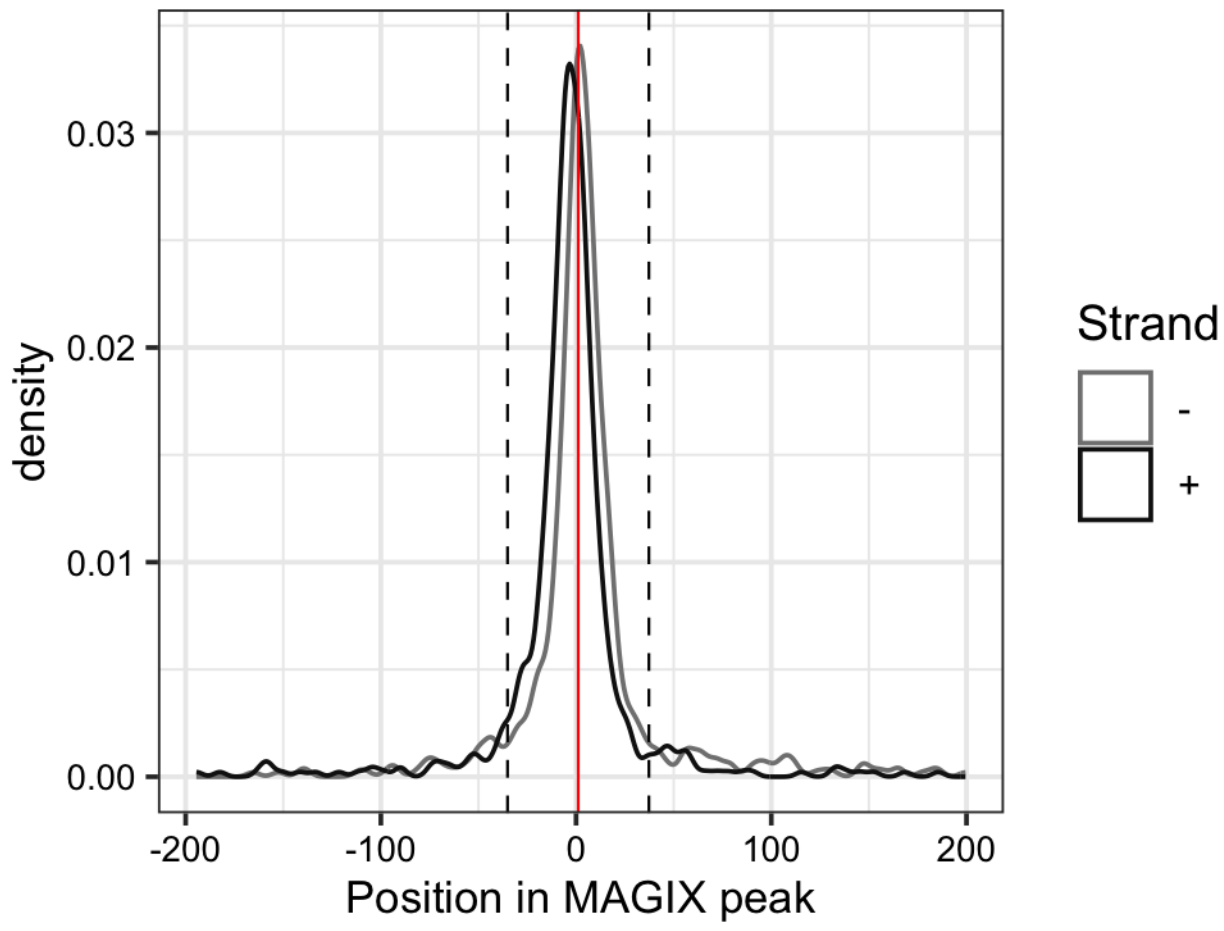

AUROC of top GRECO-BIT/MEX PWM in sliding windows (500 peaks, 50 peak step) along the MAGIX peaks (up to the top 75,000) ranked by the MAGIX enrichment coefficient and fraction of overlapping ChIP peaks in the same window

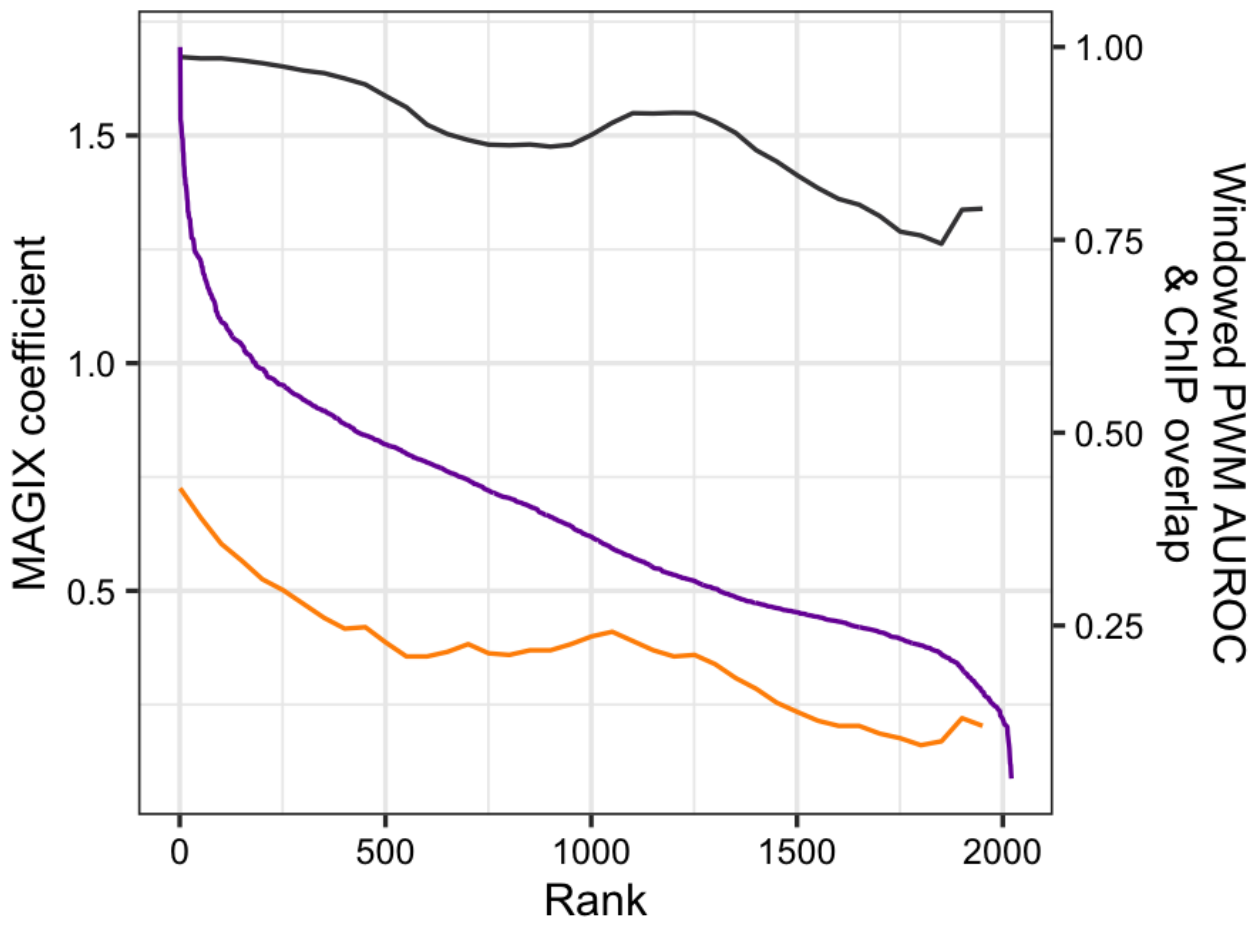

ZFAT.DBD1

Top GRECO-BIT/MEX PWM: PWM002917

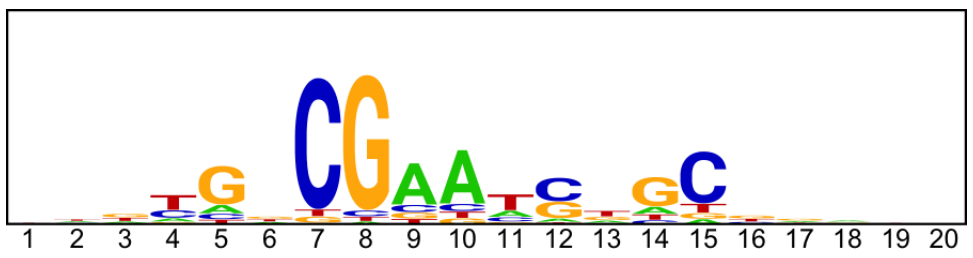

Location of top GRECO-BIT/MEX PWM in MAGIX peaks (up to the top 10,000)

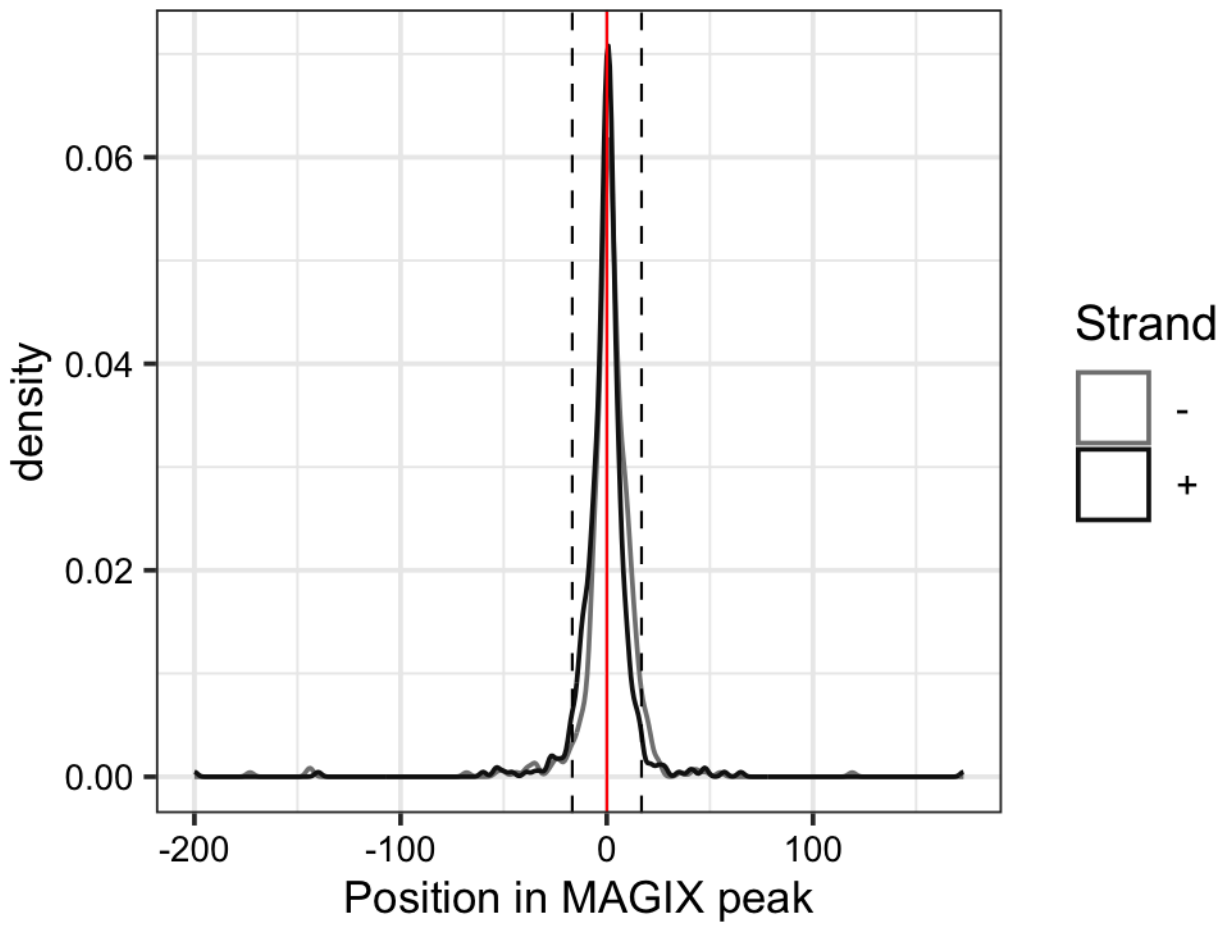

ZFP3

Top GRECO-BIT/MEX PWM: PWM002973

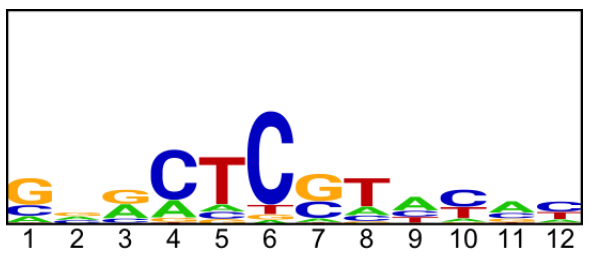

Location of top GRECO-BIT/MEX PWM in MAGIX peaks (up to the top 10,000)

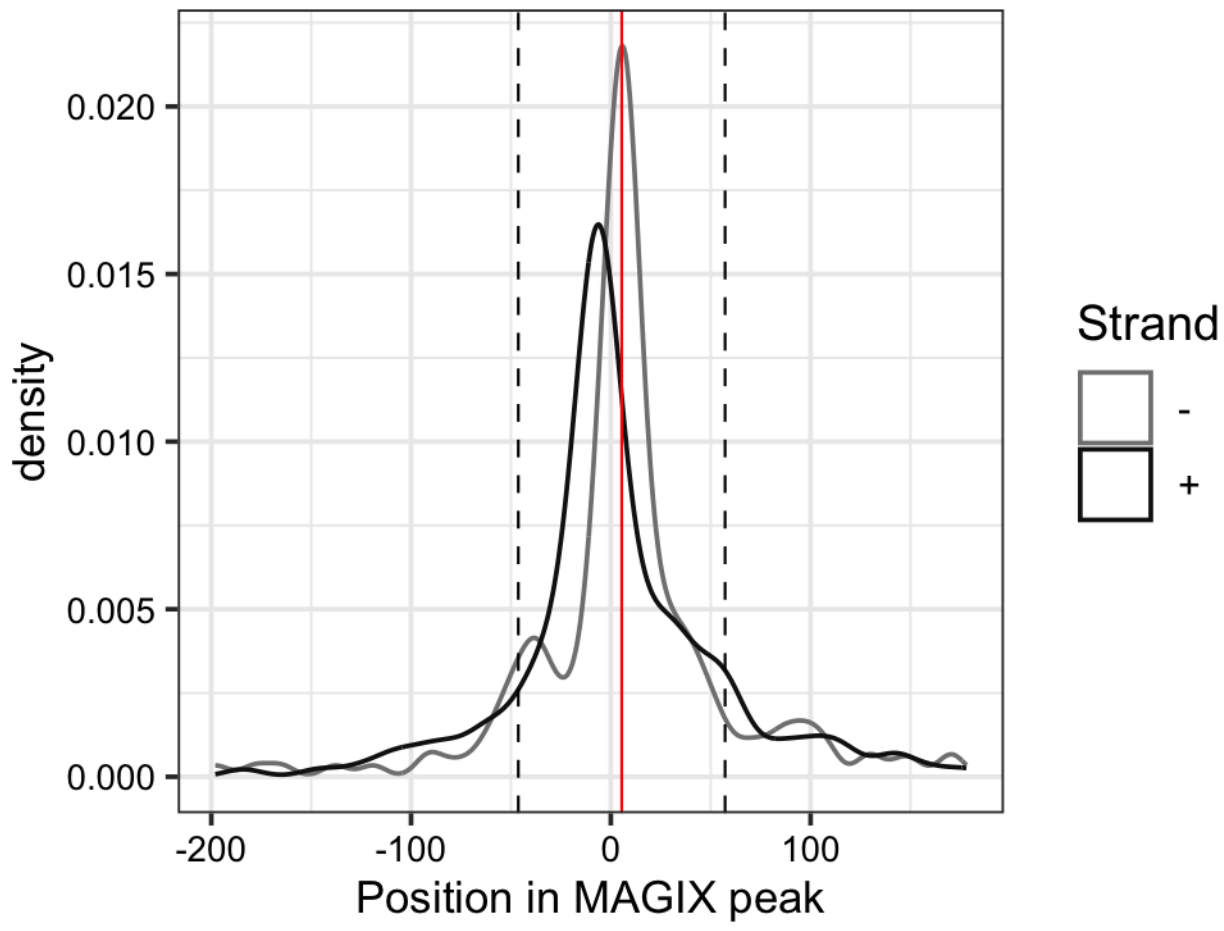

AUROC of top GRECO-BIT/MEX PWM in sliding windows (500 peaks, 50 peak step) along the MAGIX peaks (up to the top 75,000) ranked by the MAGIX enrichment coefficient and fraction of overlapping ChIP peaks in the same window

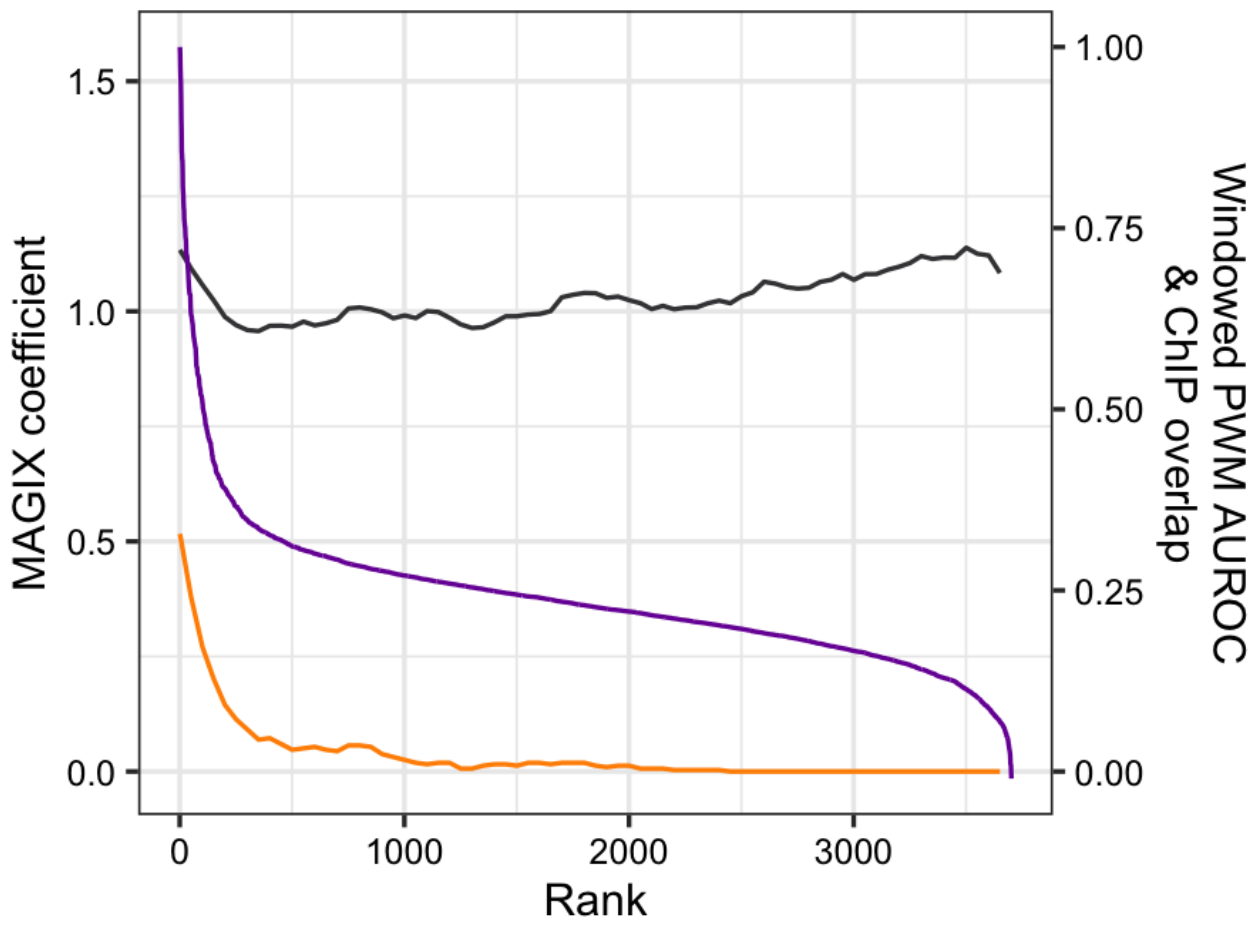

ZFP91

Top GRECO-BIT/MEX PWM: PWM002984

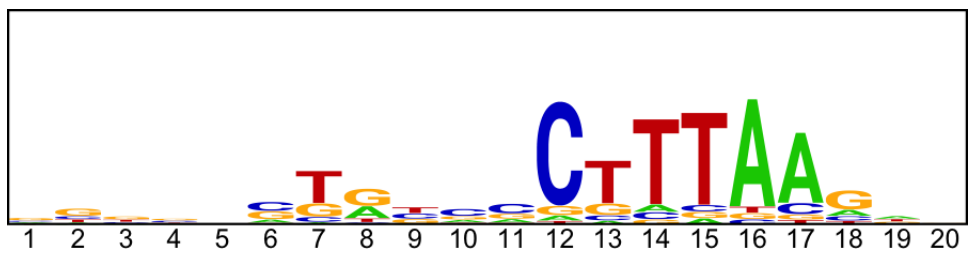

Location of top GRECO-BIT/MEX PWM in MAGIX peaks (up to the top 10,000)

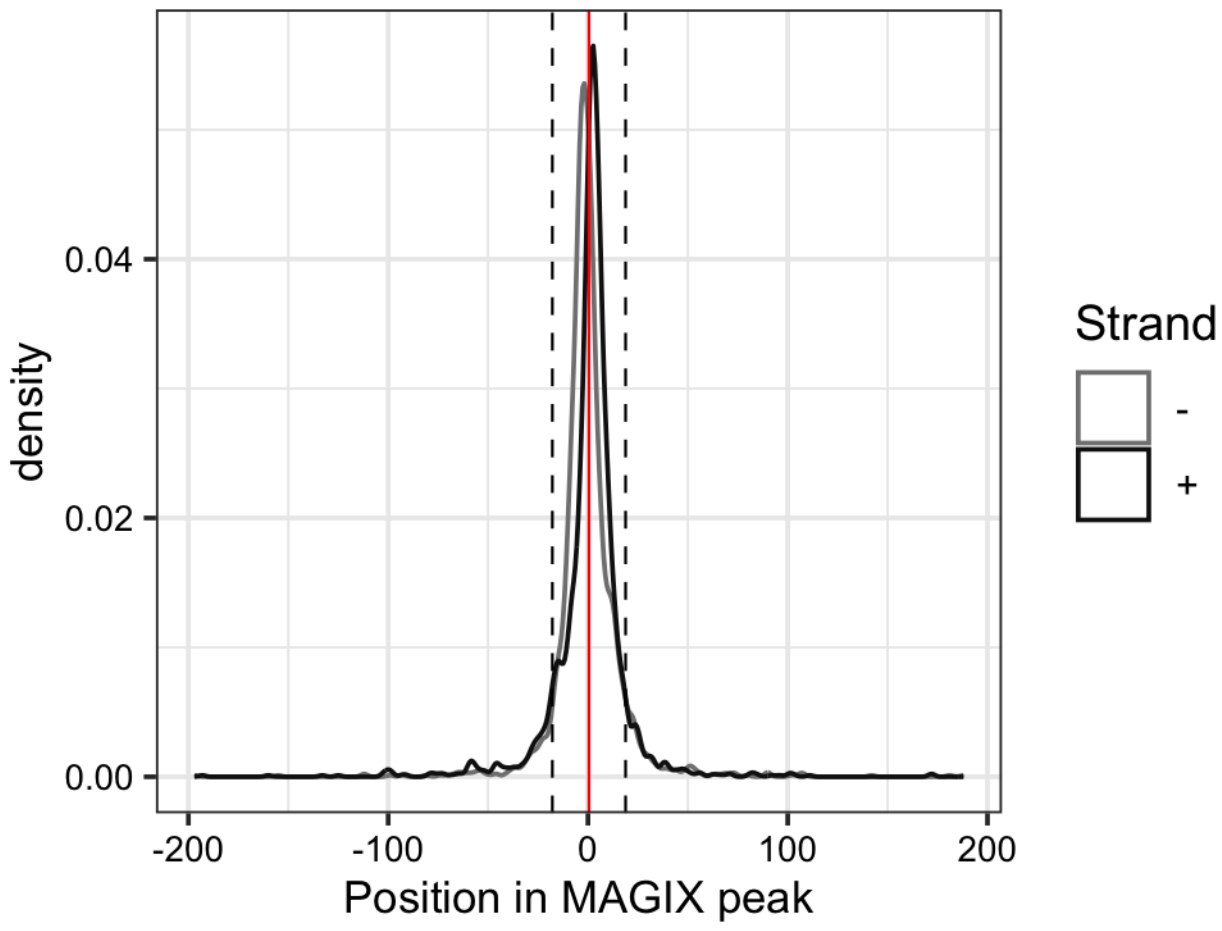

ZFTA

Top GRECO-BIT/MEX PWM: PWM003020

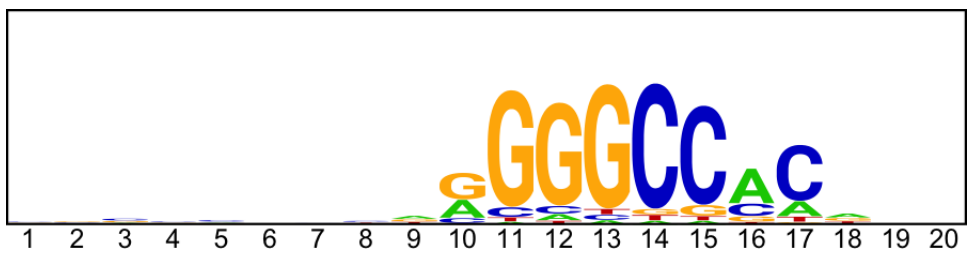

Location of top GRECO-BIT/MEX PWM in MAGIX peaks (up to the top 10,000)

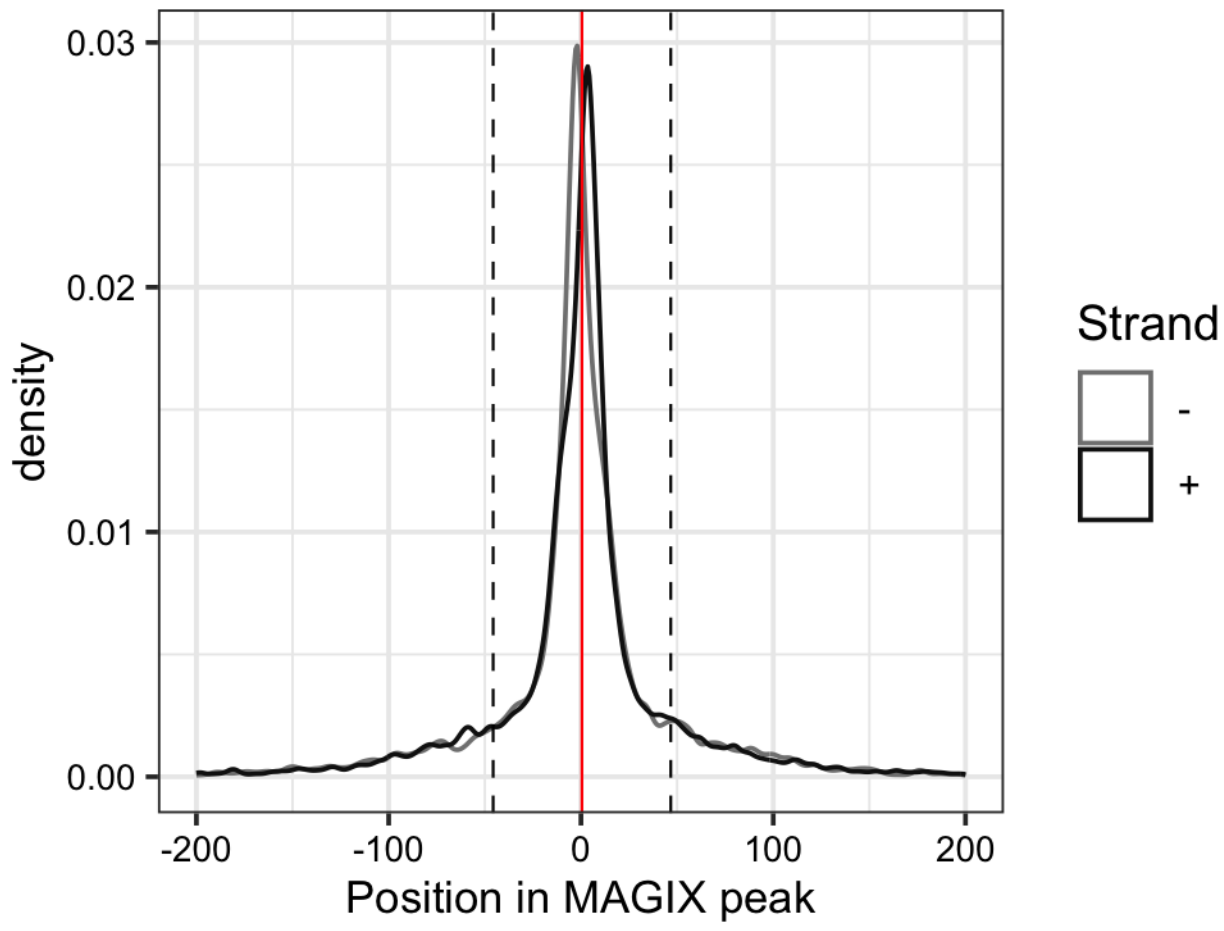

AUROC of top GRECO-BIT/MEX PWM in sliding windows (500 peaks, 50 peak step) along the MAGIX peaks (up to the top 75,000) ranked by the MAGIX enrichment coefficient and fraction of overlapping ChIP peaks in the same window

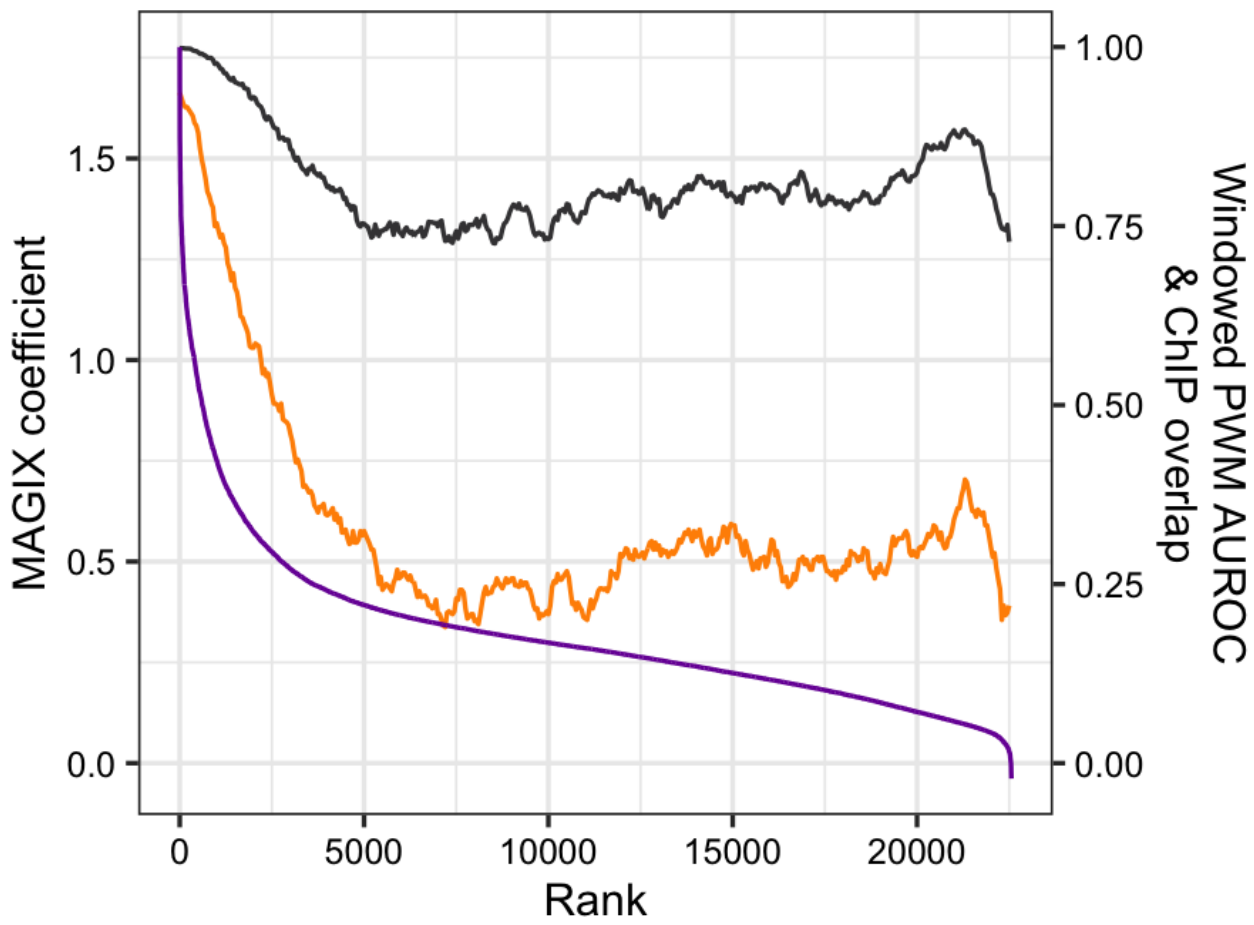

ZGLP1

Top GRECO-BIT/MEX PWM: PWM003053

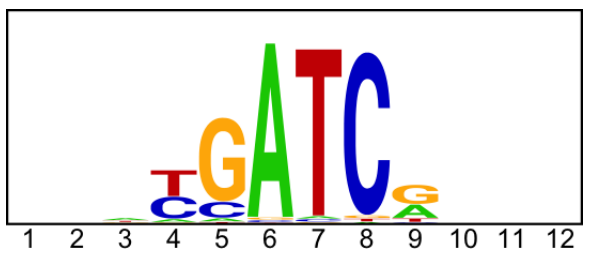

Location of top GRECO-BIT/MEX PWM in MAGIX peaks (up to the top 10,000)

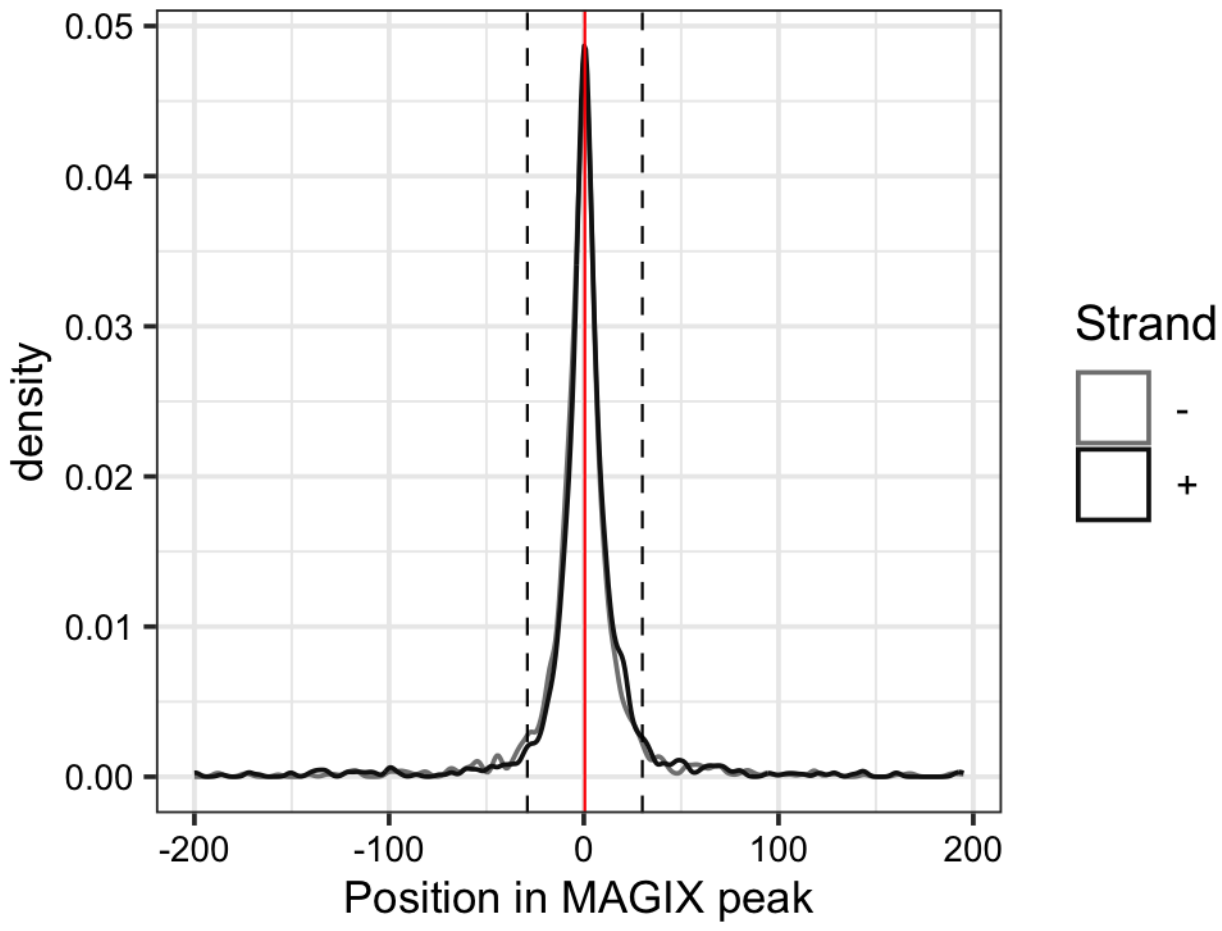

ZIM3

Top GRECO-BIT/MEX PWM: PWM003079

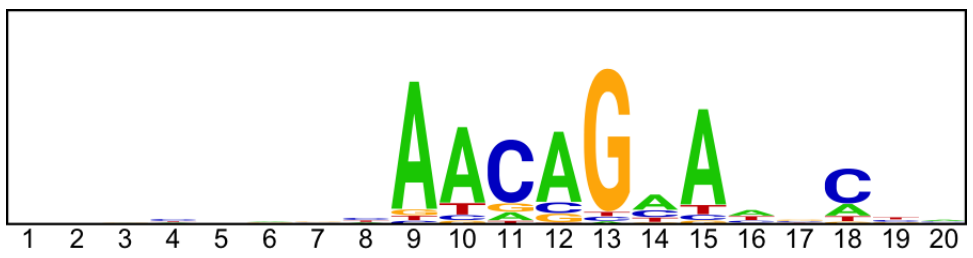

Location of top GRECO-BIT/MEX PWM in MAGIX peaks (up to the top 10,000)

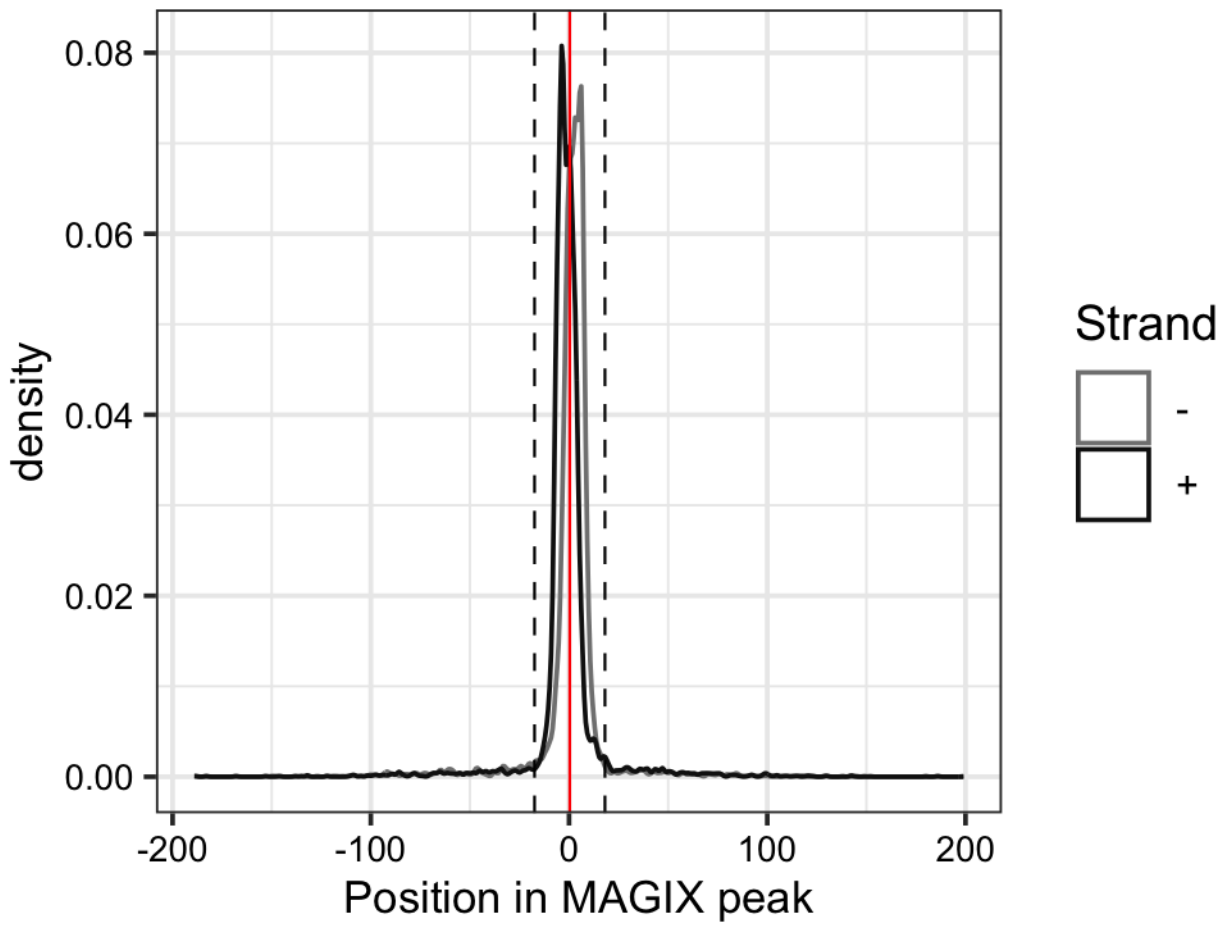

AUROC of top GRECO-BIT/MEX PWM in sliding windows (500 peaks, 50 peak step) along the MAGIX peaks (up to the top 75,000) ranked by the MAGIX enrichment coefficient and fraction of overlapping ChIP peaks in the same window

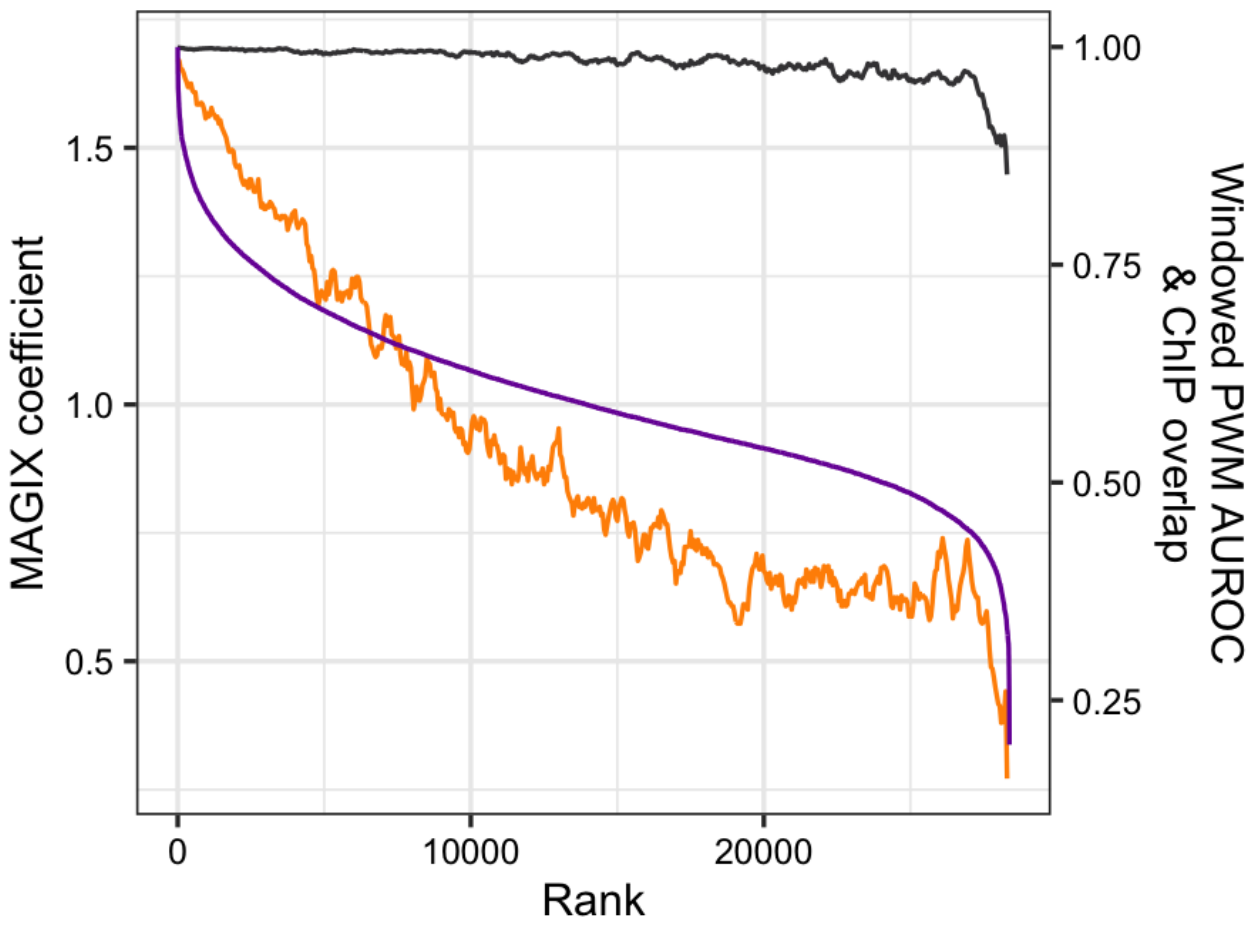

ZKSCAN4

Top GRECO-BIT/MEX PWM: PWM003099

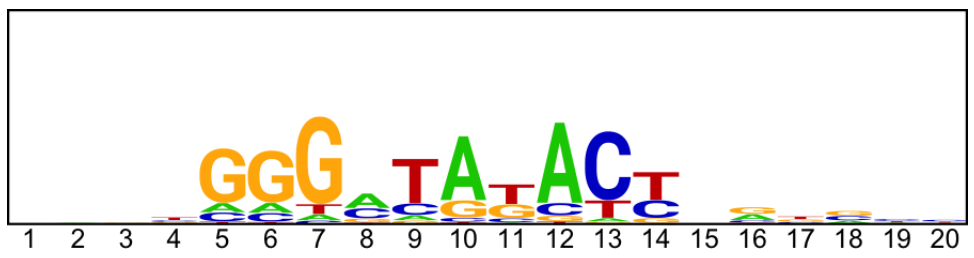

Location of top GRECO-BIT/MEX PWM in MAGIX peaks (up to the top 10,000)

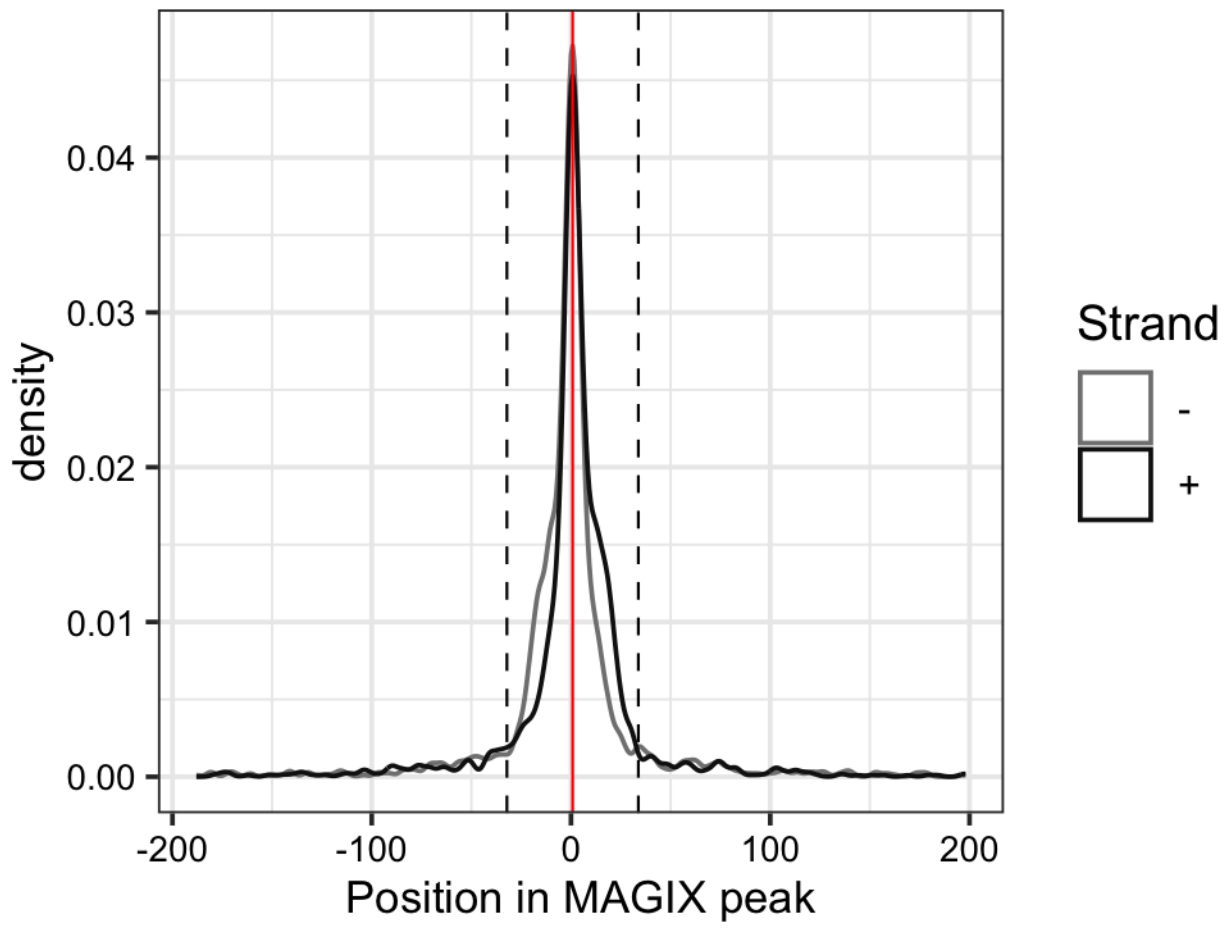

ZNF107.DBD1

Top GRECO-BIT/MEX PWM: PWM003143

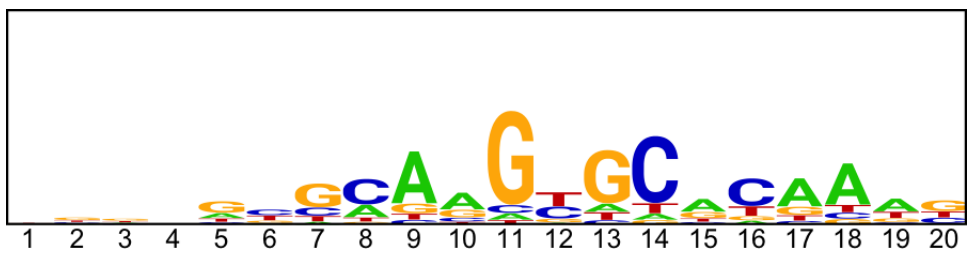

Location of top GRECO-BIT/MEX PWM in MAGIX peaks (up to the top 10,000)

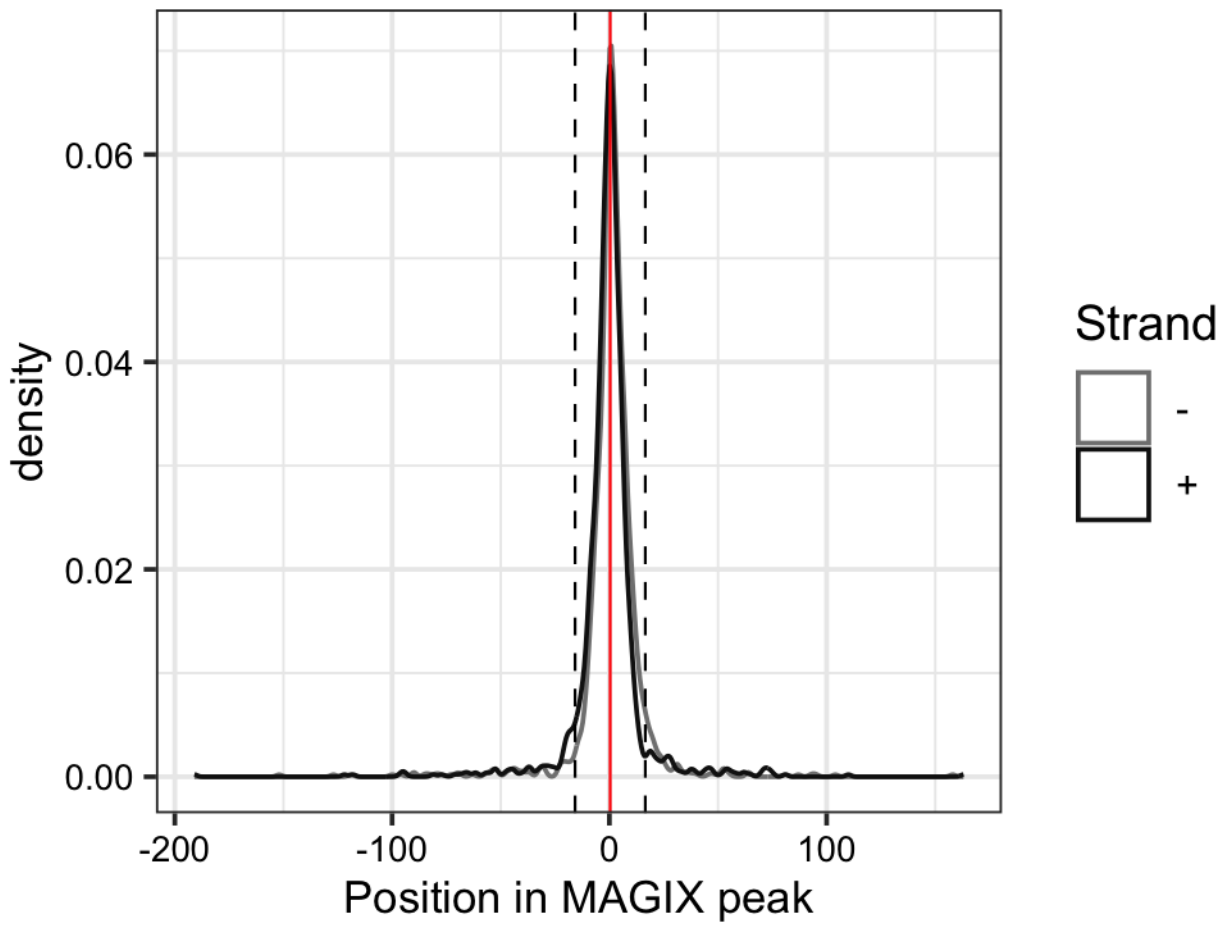

ZNF107.DBD2

Top GRECO-BIT/MEX PWM: PWM003126

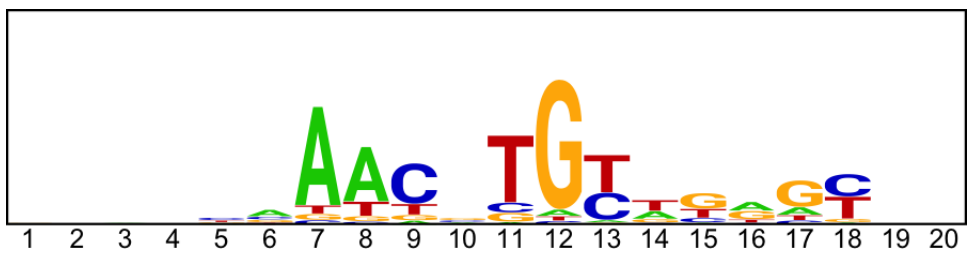

Location of top GRECO-BIT/MEX PWM in MAGIX peaks (up to the top 10,000)

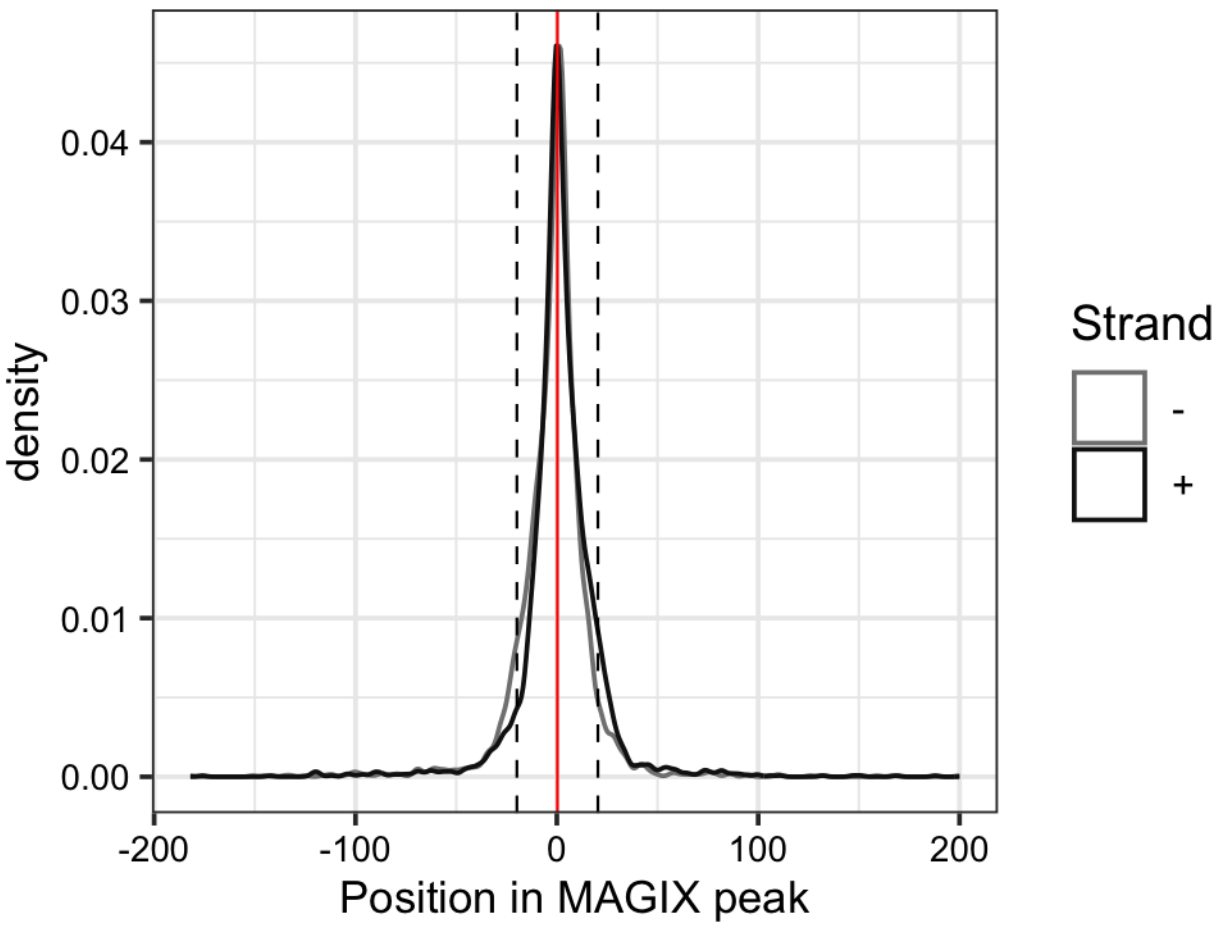

ZNF131

Top GRECO-BIT/MEX PWM: PWM003208

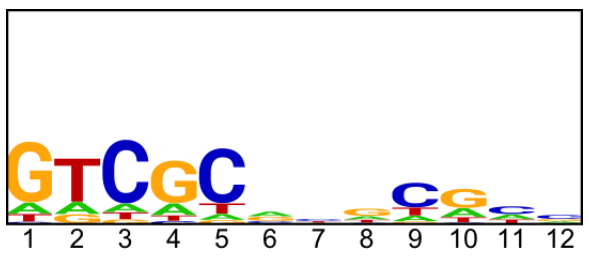

Location of top GRECO-BIT/MEX PWM in MAGIX peaks (up to the top 10,000)

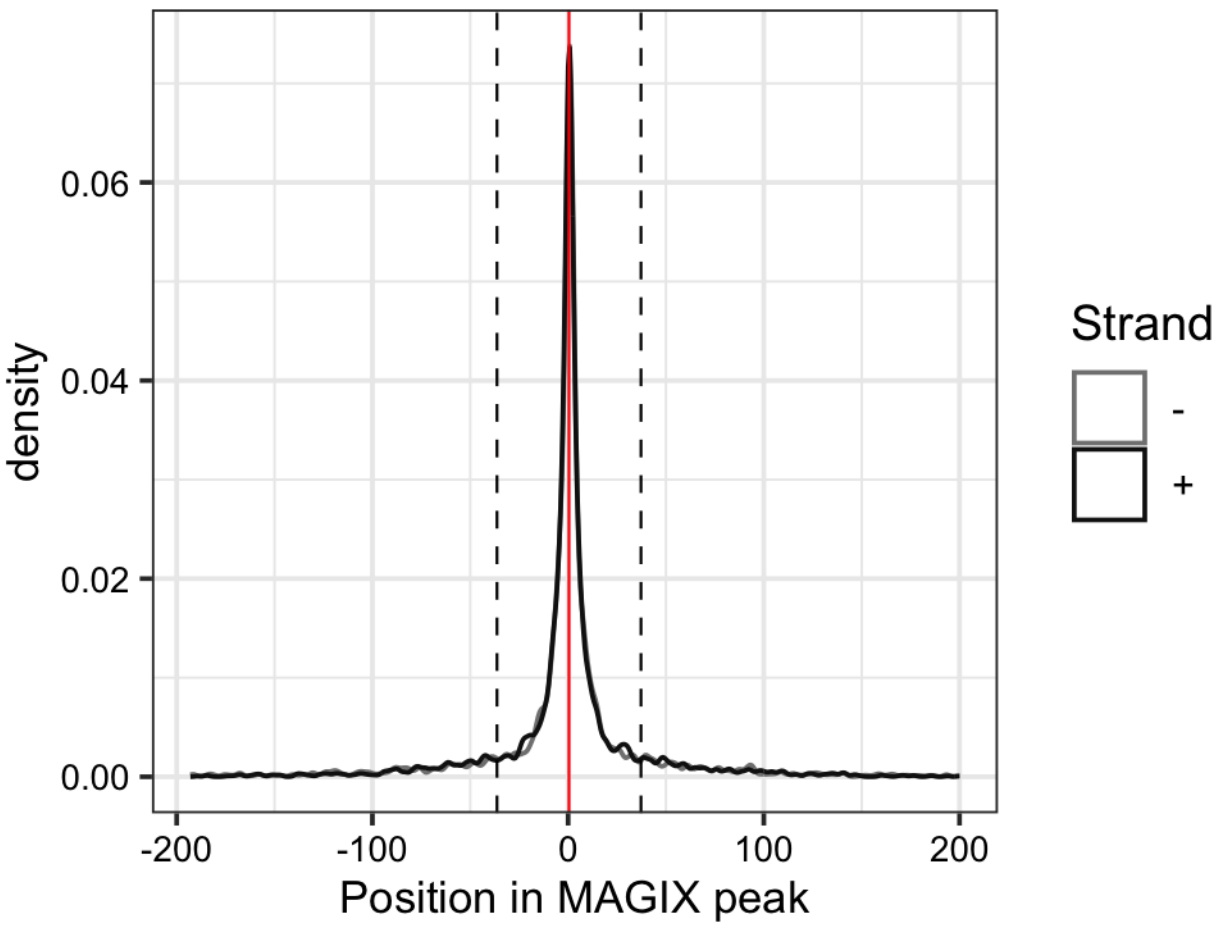

AUROC of top GRECO-BIT/MEX PWM in sliding windows (500 peaks, 50 peak step) along the MAGIX peaks (up to the top 75,000) ranked by the MAGIX enrichment coefficient and fraction of overlapping ChIP peaks in the same window

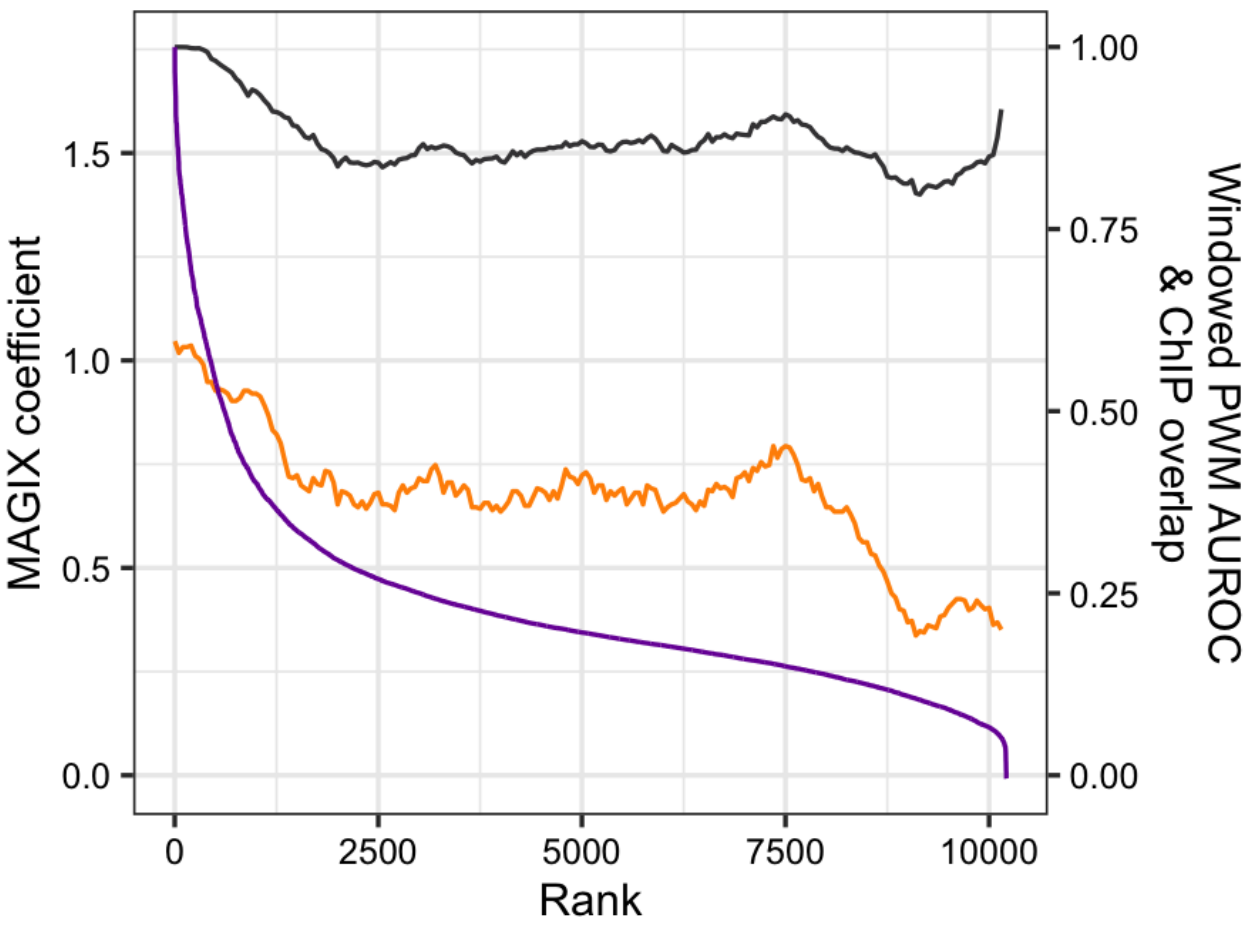

ZNF134

Top GRECO-BIT/MEX PWM: PWM003228

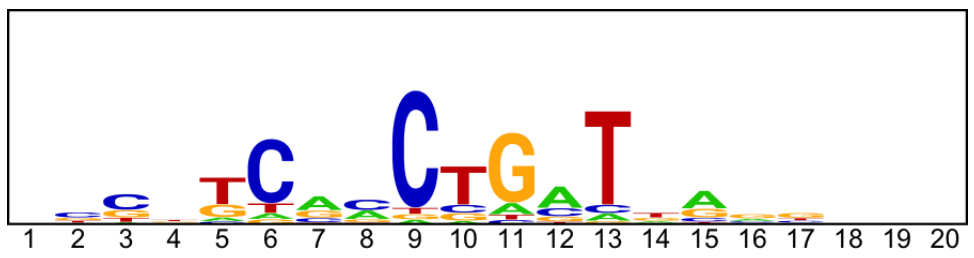

Location of top GRECO-BIT/MEX PWM in MAGIX peaks (up to the top 10,000)

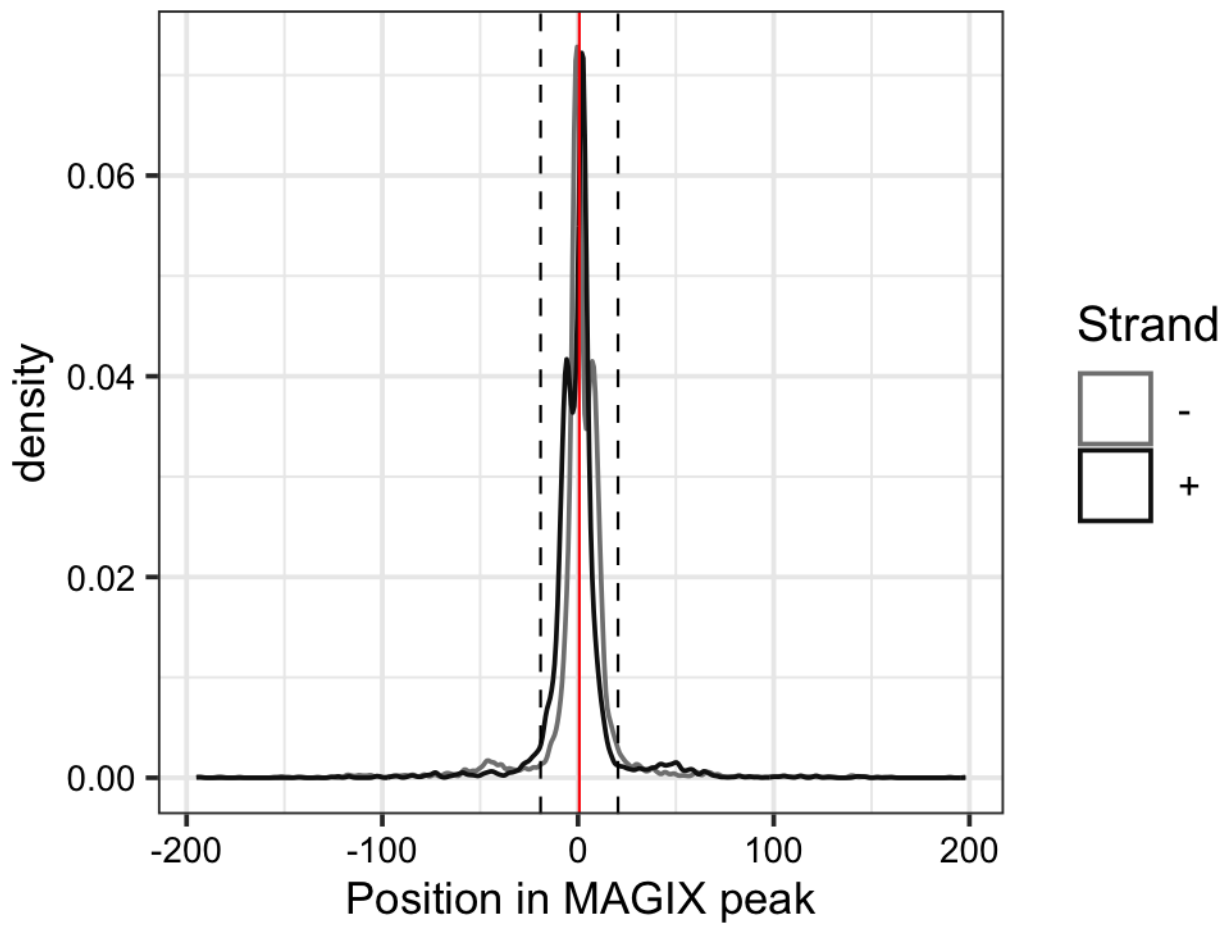

AUROC of top GRECO-BIT/MEX PWM in sliding windows (500 peaks, 50 peak step) along the MAGIX peaks (up to the top 75,000) ranked by the MAGIX enrichment coefficient and fraction of overlapping ChIP peaks in the same window

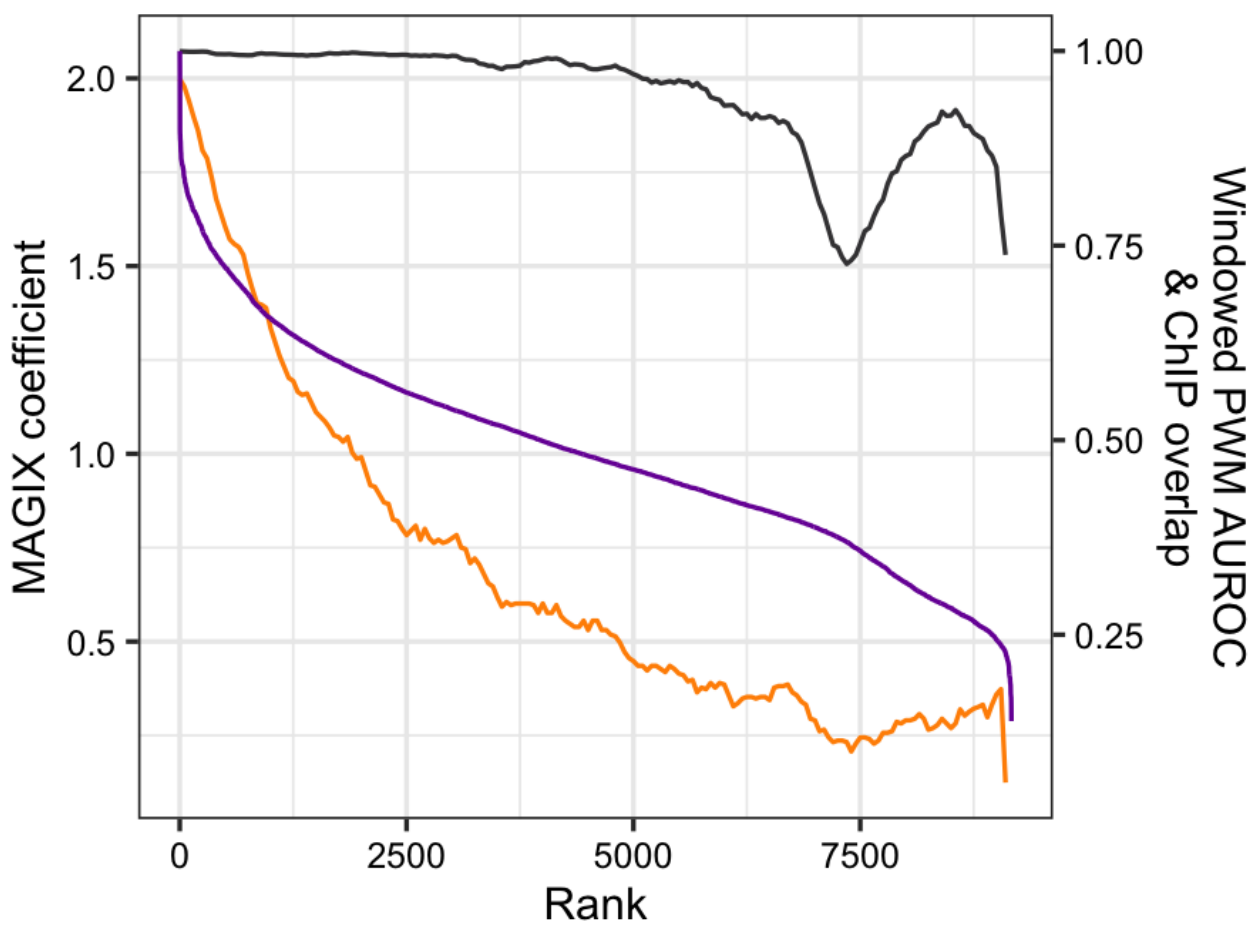

ZNF142.DBD1

Top GRECO-BIT/MEX PWM: PWM003313

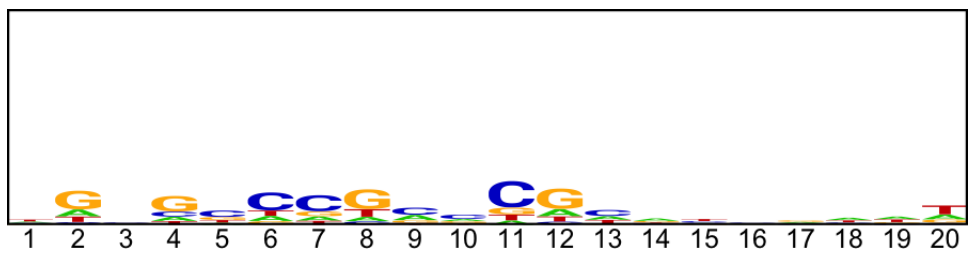

Location of top GRECO-BIT/MEX PWM in MAGIX peaks (up to the top 10,000)

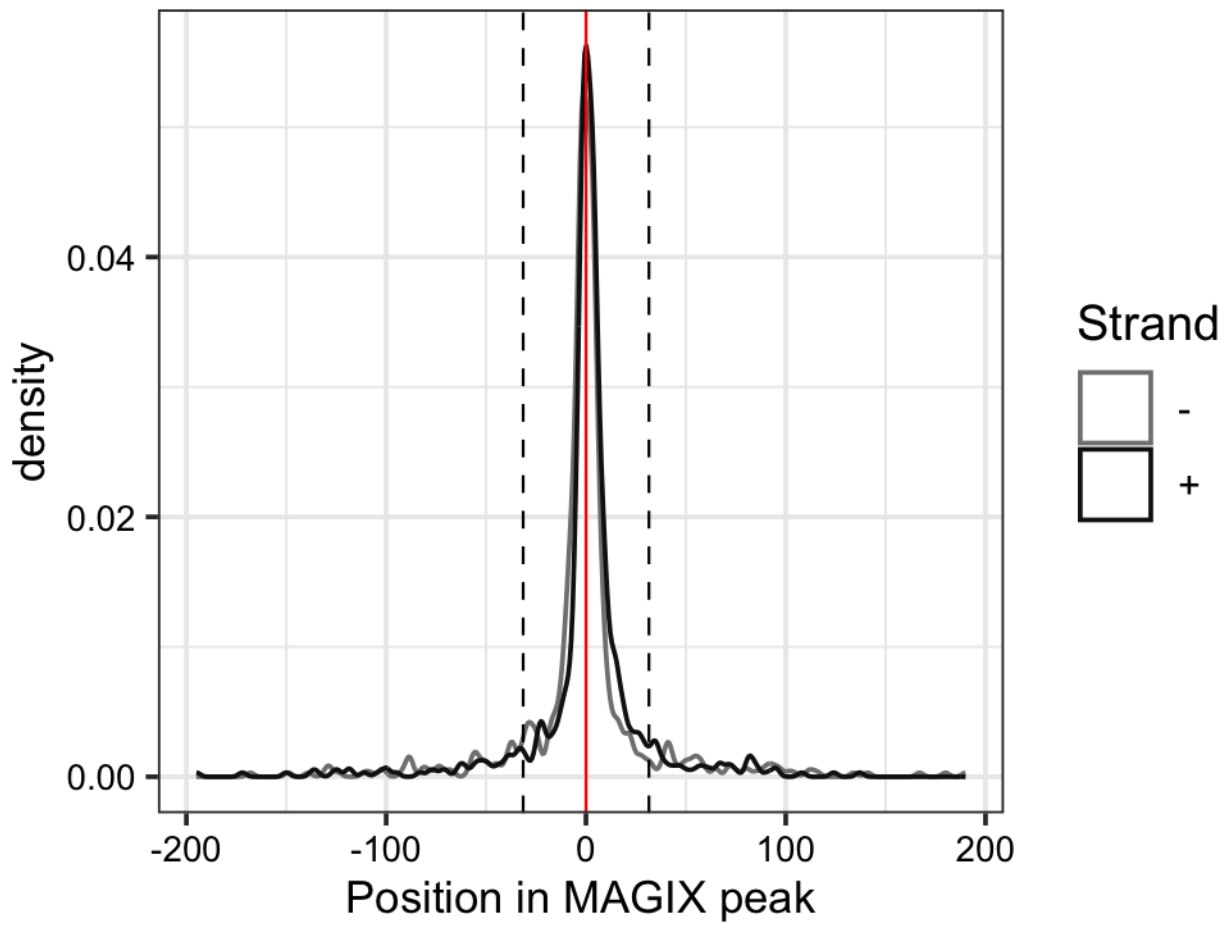

ZNF16

Top GRECO-BIT/MEX PWM: PWM003349

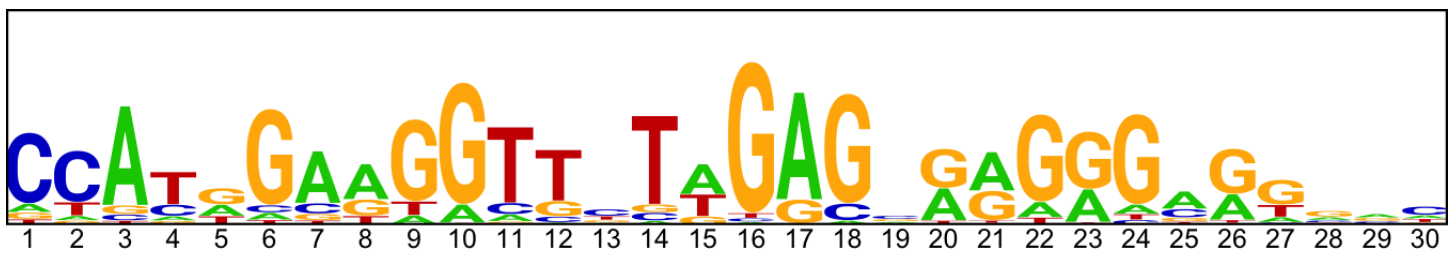

Location of top GRECO-BIT/MEX PWM in MAGIX peaks (up to the top 10,000)

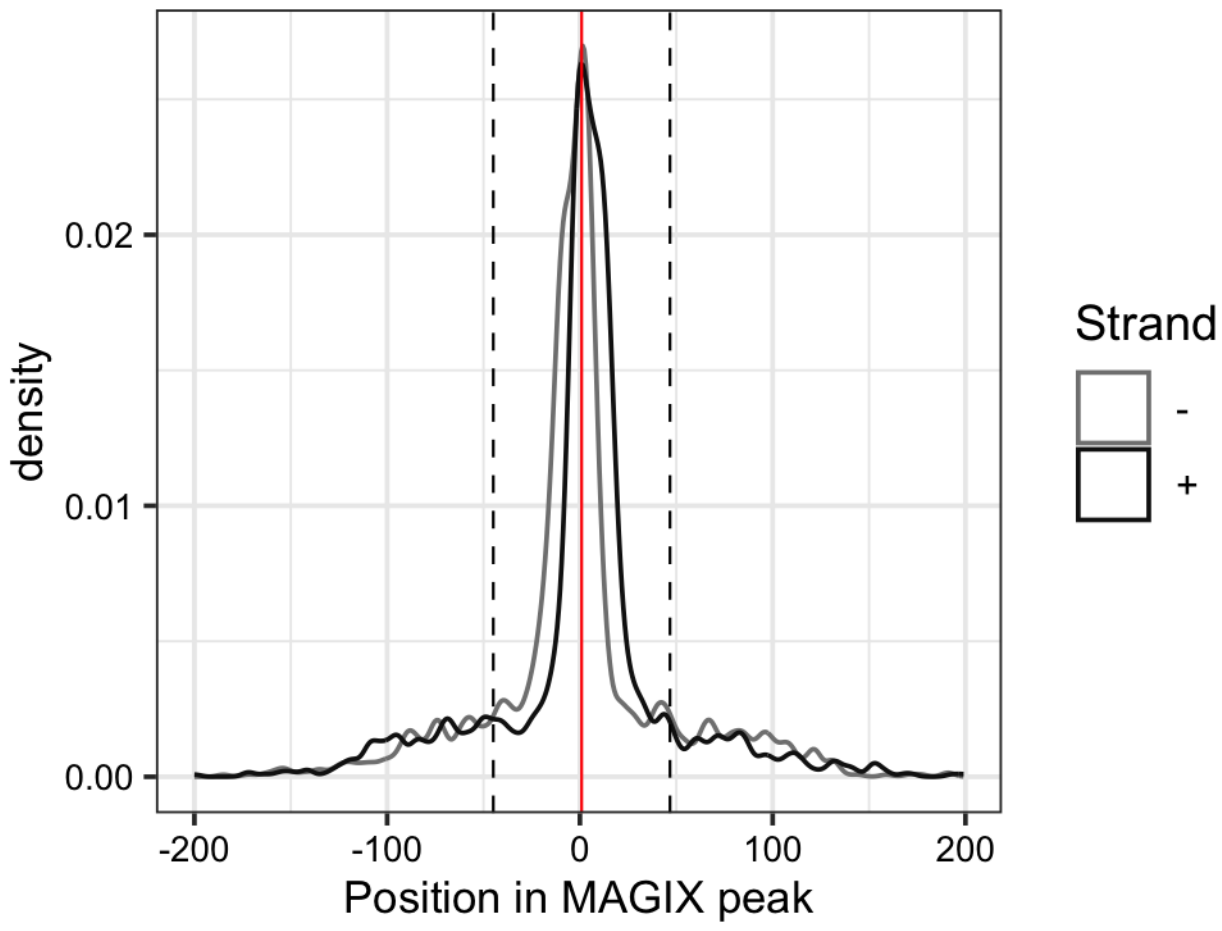

ZNF160

Top GRECO-BIT/MEX PWM: PWM003369

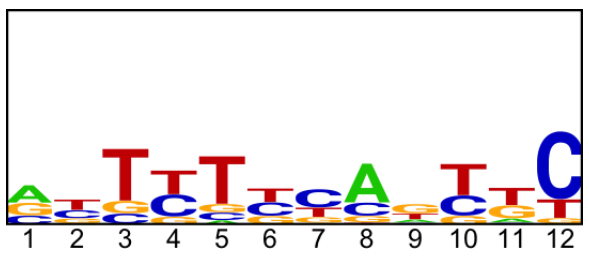

Location of top GRECO-BIT/MEX PWM in MAGIX peaks (up to the top 10,000)

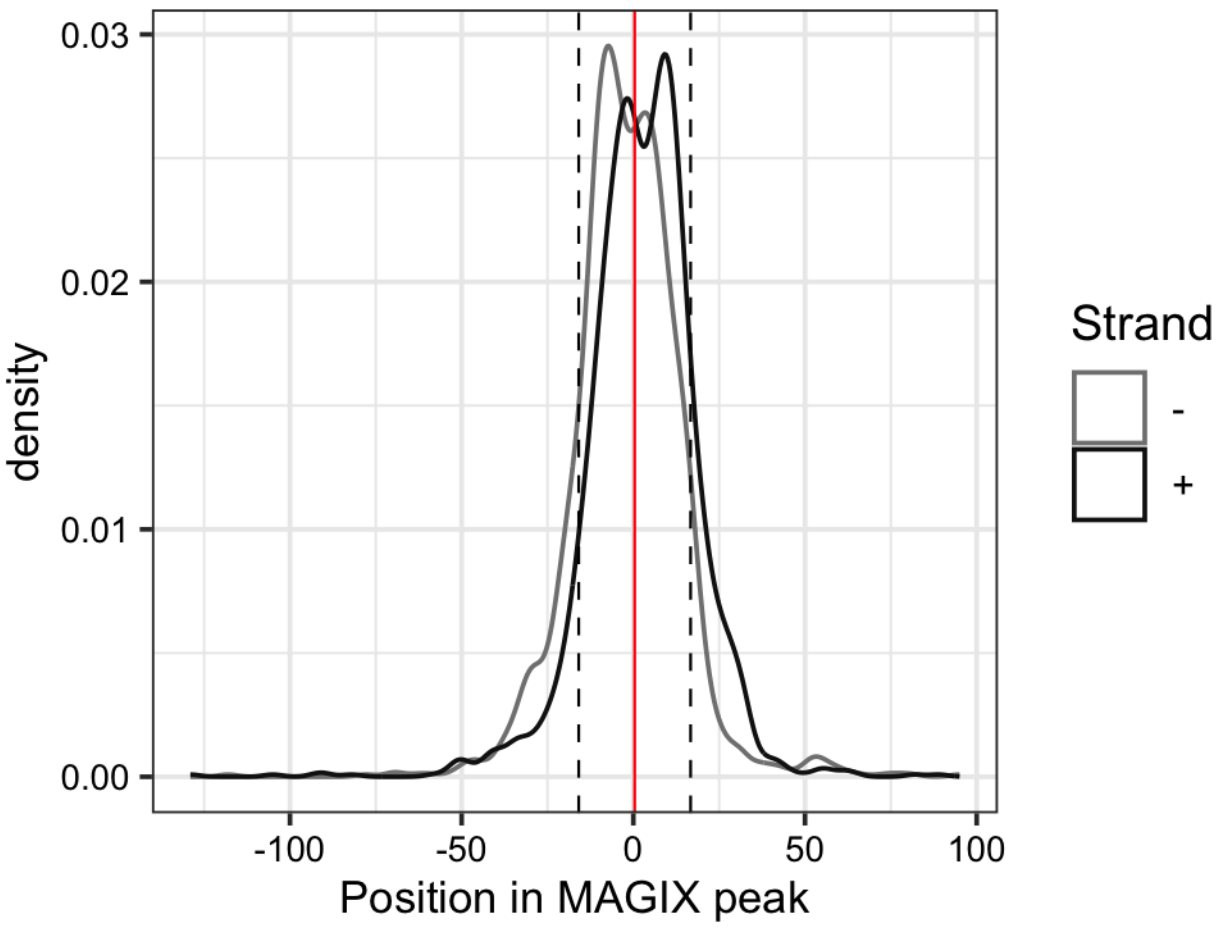

ZNF18

Top GRECO-BIT/MEX PWM: PWM003393

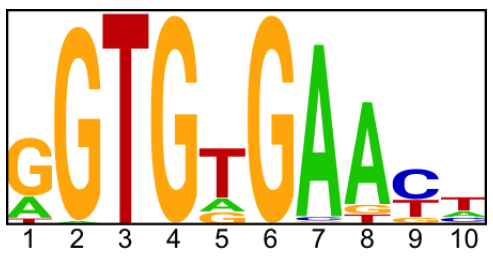

Location of top GRECO-BIT/MEX PWM in MAGIX peaks (up to the top 10,000)

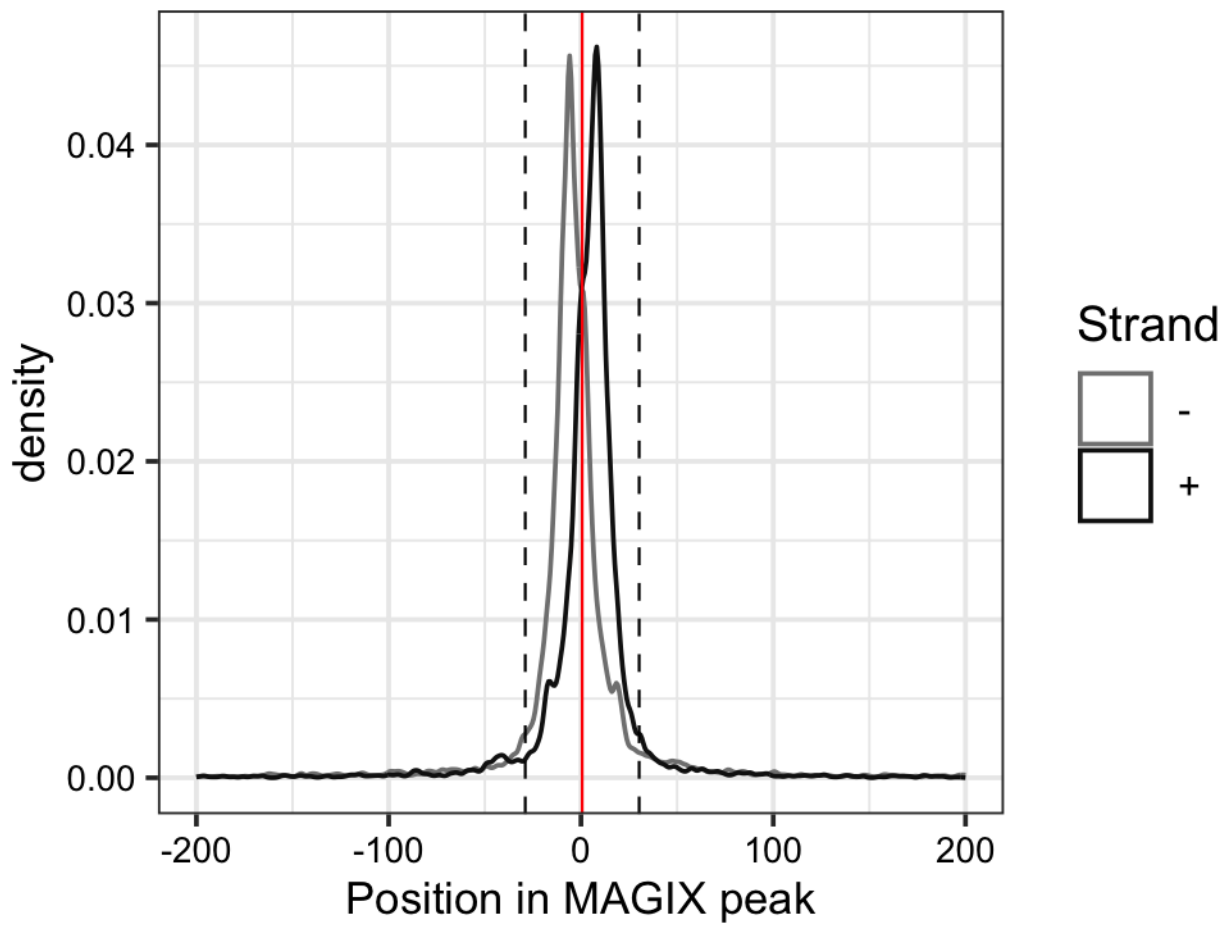

AUROC of top GRECO-BIT/MEX PWM in sliding windows (500 peaks, 50 peak step) along the MAGIX peaks (up to the top 75,000) ranked by the MAGIX enrichment coefficient and fraction of overlapping ChIP peaks in the same window

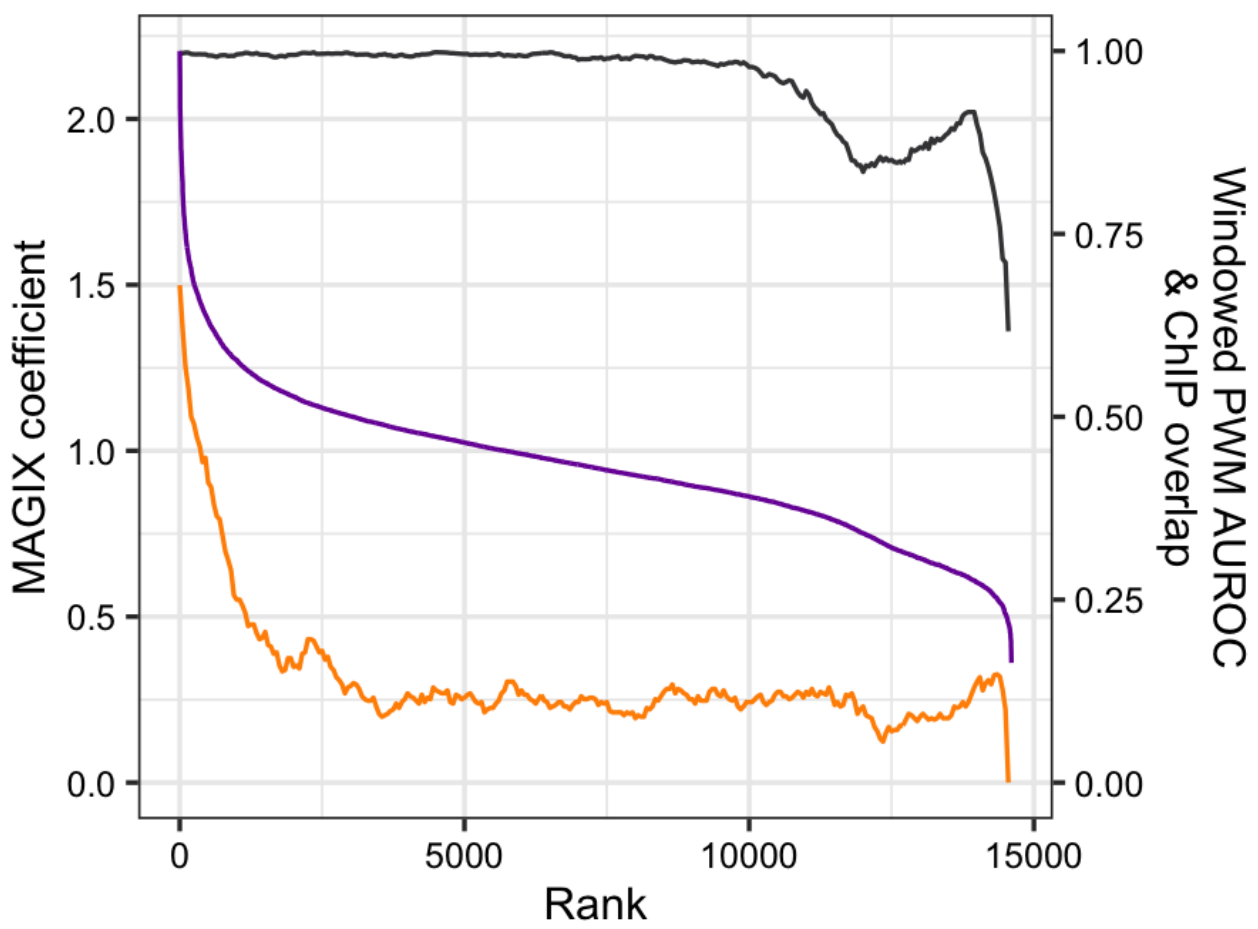

ZNF20

Top GRECO-BIT/MEX PWM: PWM003422

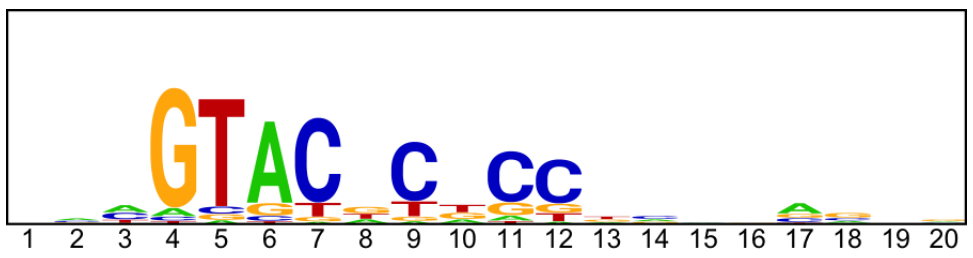

Location of top GRECO-BIT/MEX PWM in MAGIX peaks (up to the top 10,000)

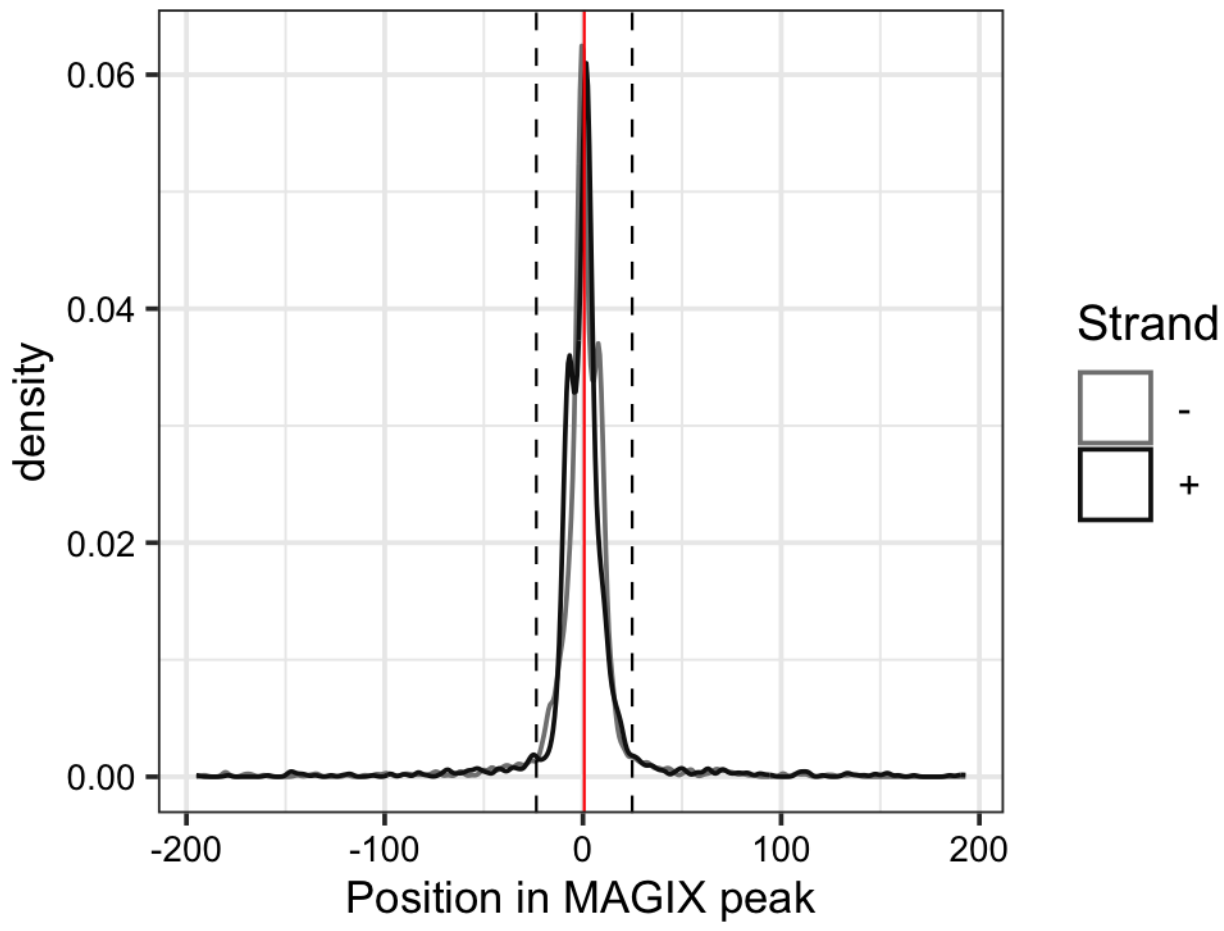

AUROC of top GRECO-BIT/MEX PWM in sliding windows (500 peaks, 50 peak step) along the MAGIX peaks (up to the top 75,000) ranked by the MAGIX enrichment coefficient and fraction of overlapping ChIP peaks in the same window

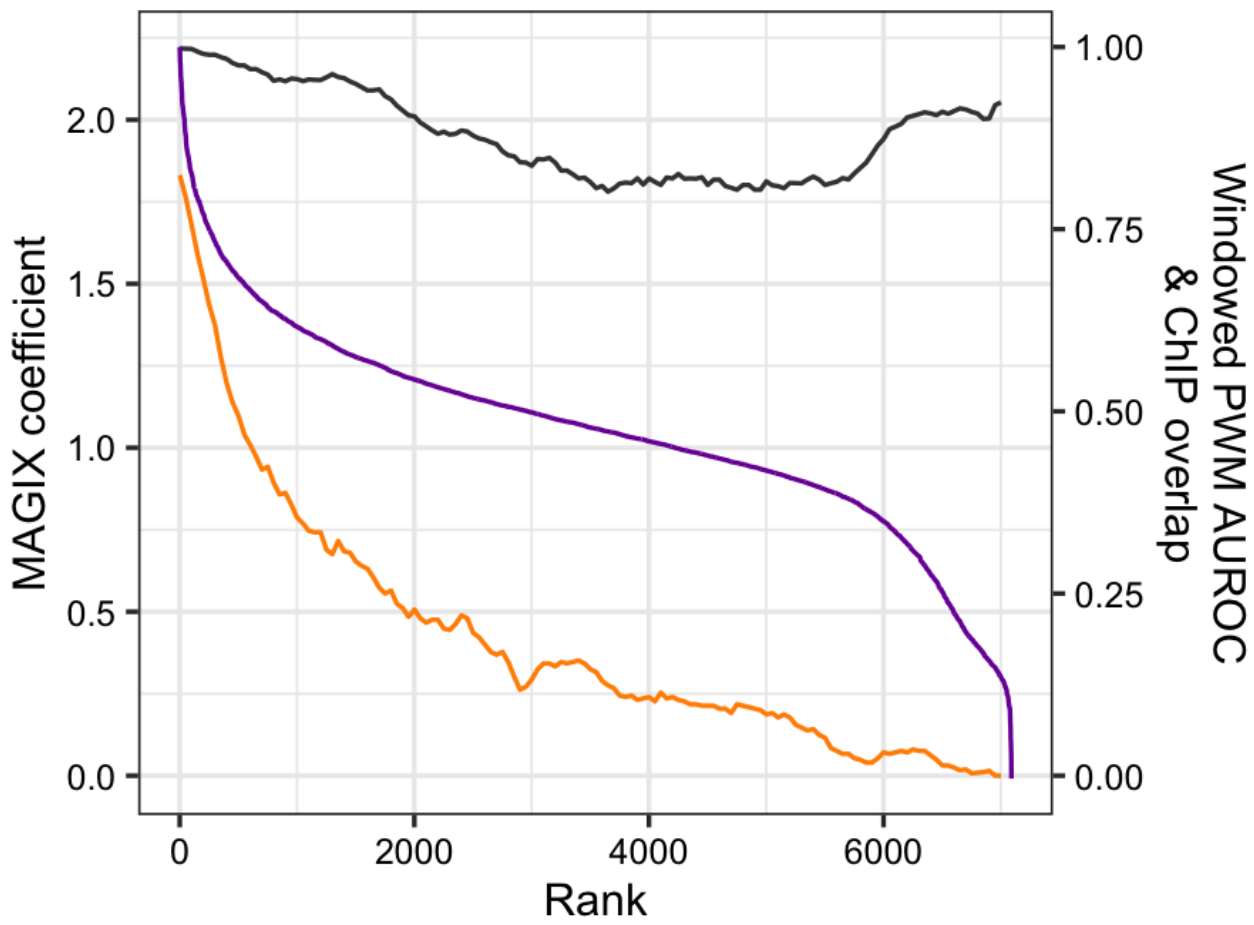

ZNF208.DBD1

Top GRECO-BIT/MEX PWM: PWM124525

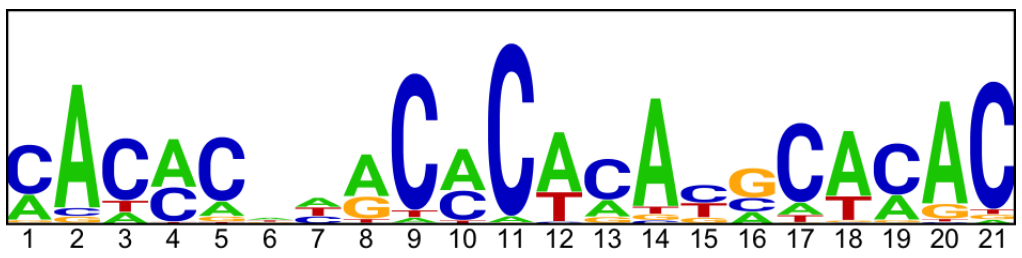

Location of top GRECO-BIT/MEX PWM in MAGIX peaks (up to the top 10,000)

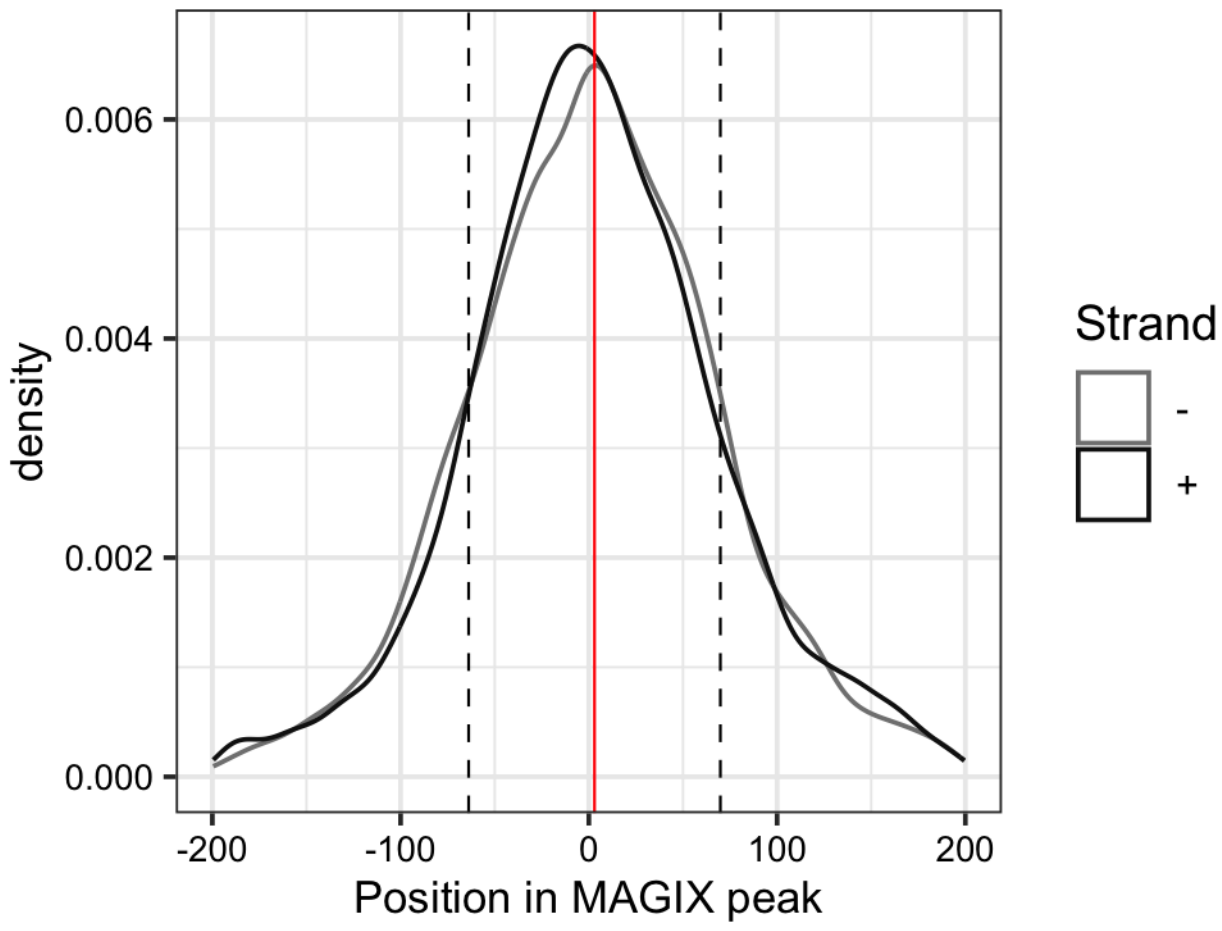

ZNF208.DBD2

Top GRECO-BIT/MEX PWM: PWM003442

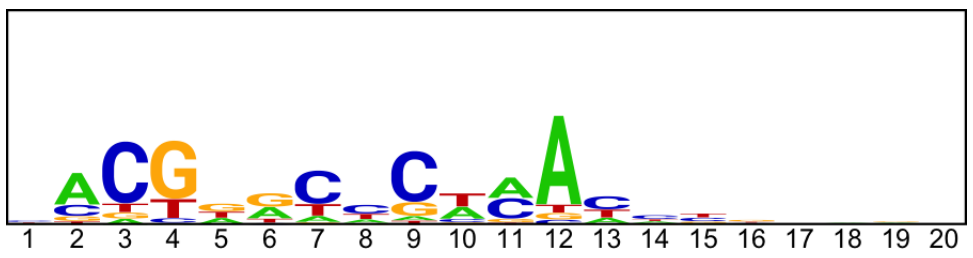

Location of top GRECO-BIT/MEX PWM in MAGIX peaks (up to the top 10,000)

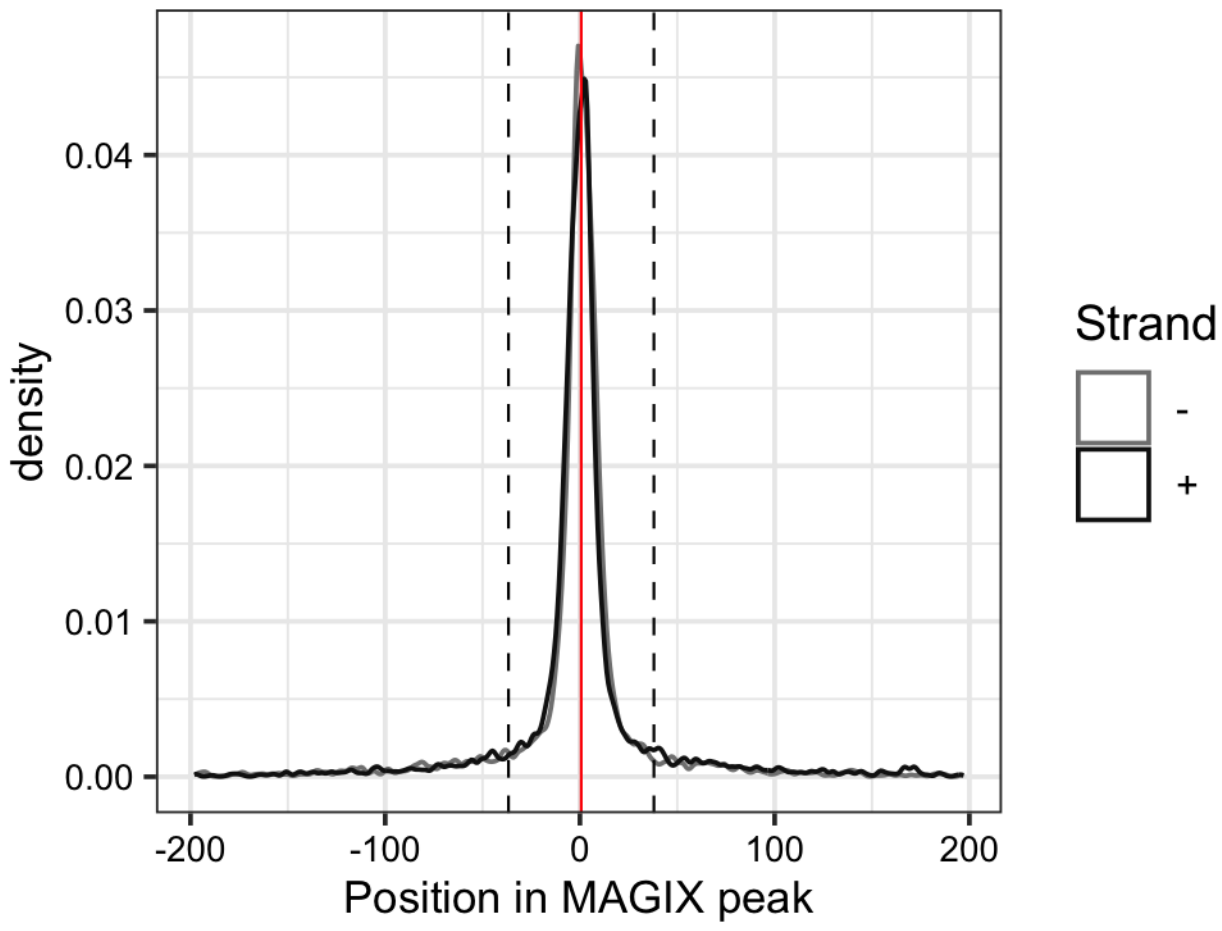

ZNF208.DBD3

Top GRECO-BIT/MEX PWM: PWM003448

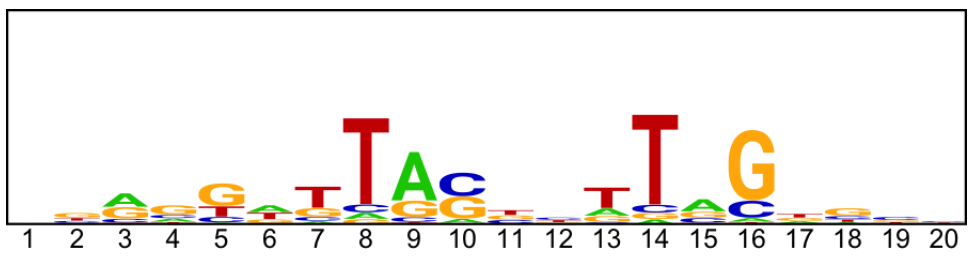

Location of top GRECO-BIT/MEX PWM in MAGIX peaks (up to the top 10,000)

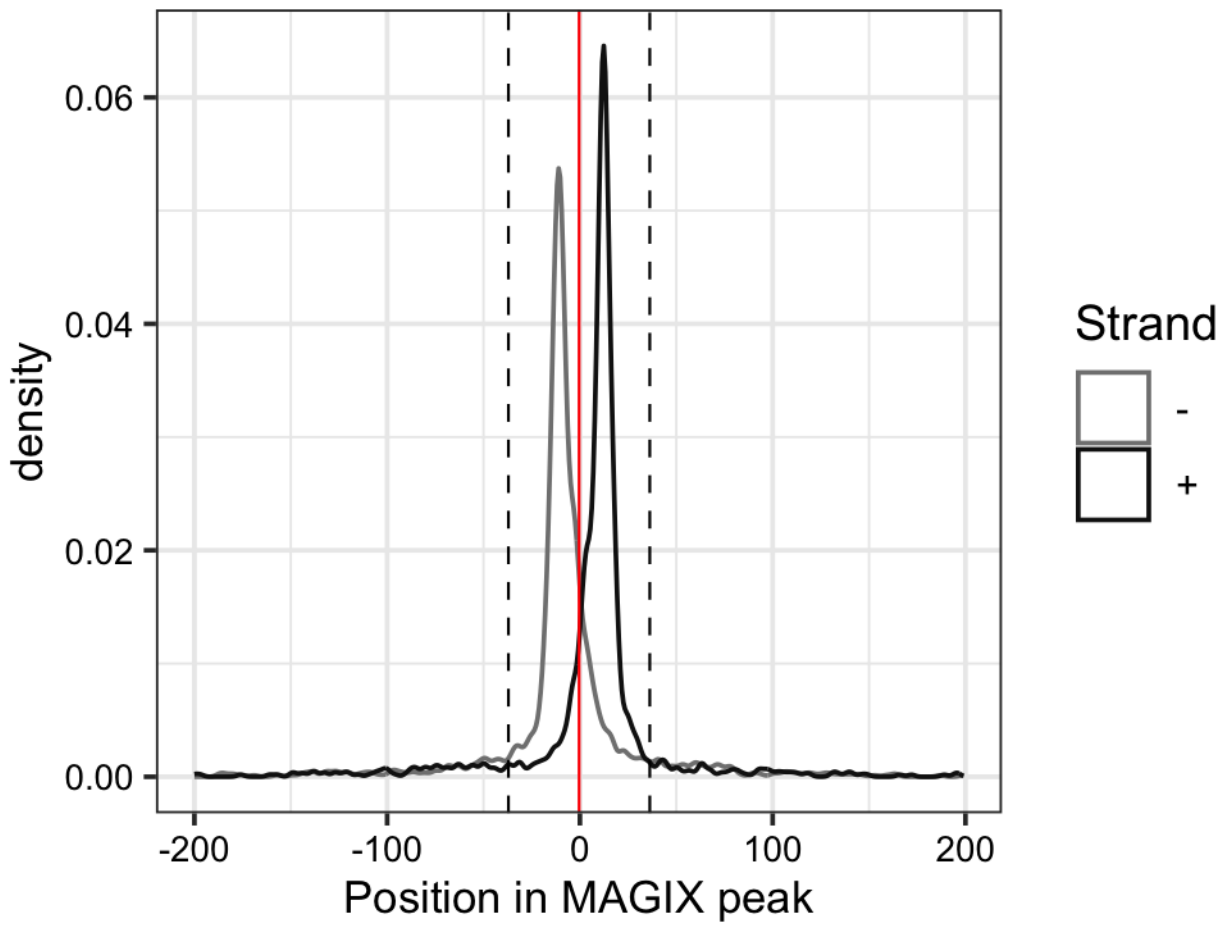

ZNF215

Top GRECO-BIT/MEX PWM: PWM003473

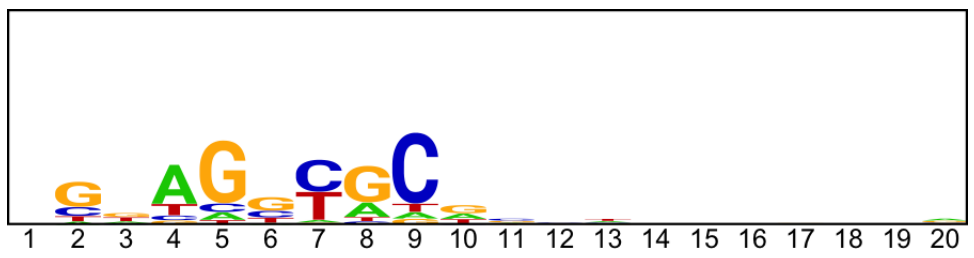

Location of top GRECO-BIT/MEX PWM in MAGIX peaks (up to the top 10,000)

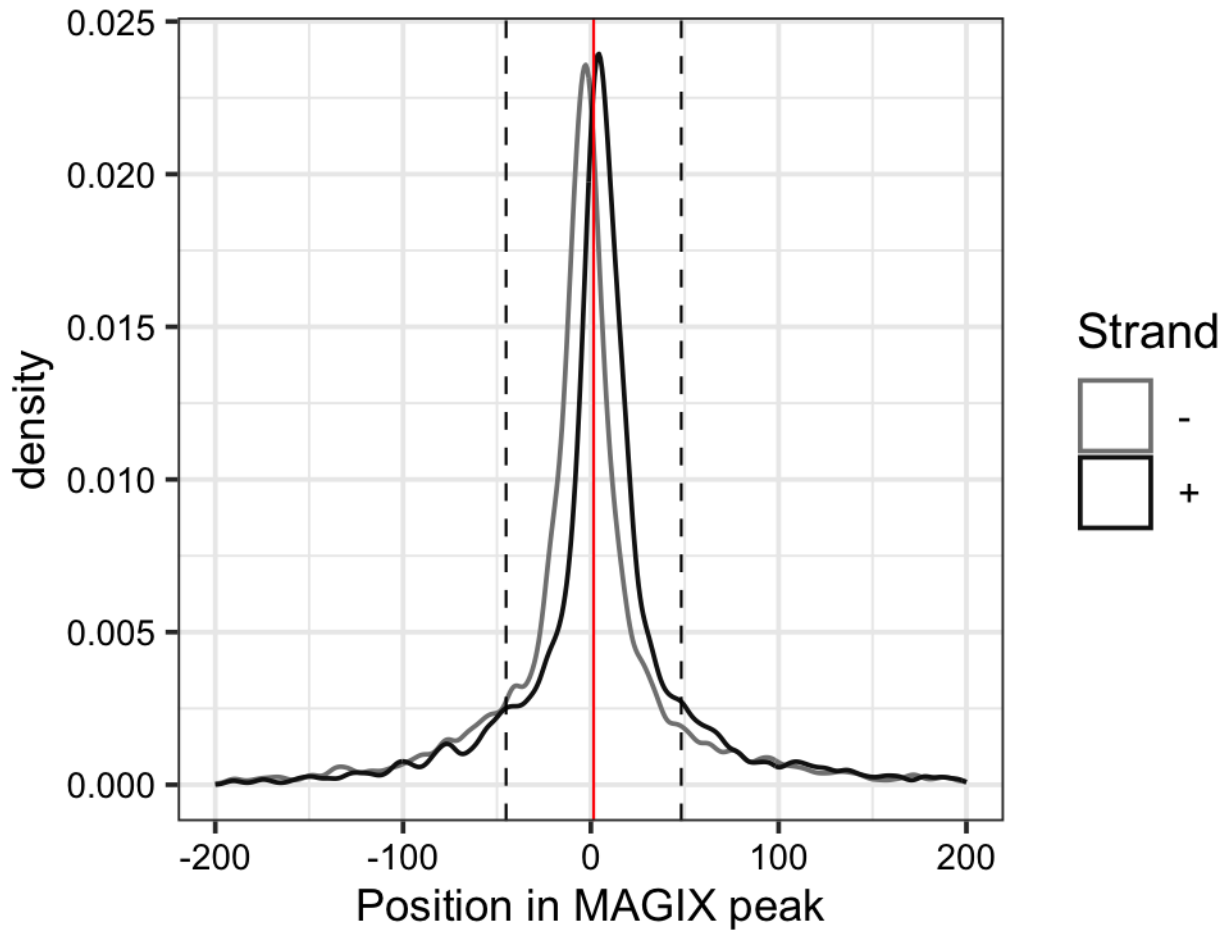

AUROC of top GRECO-BIT/MEX PWM in sliding windows (500 peaks, 50 peak step) along the MAGIX peaks (up to the top 75,000) ranked by the MAGIX enrichment coefficient and fraction of overlapping ChIP peaks in the same window

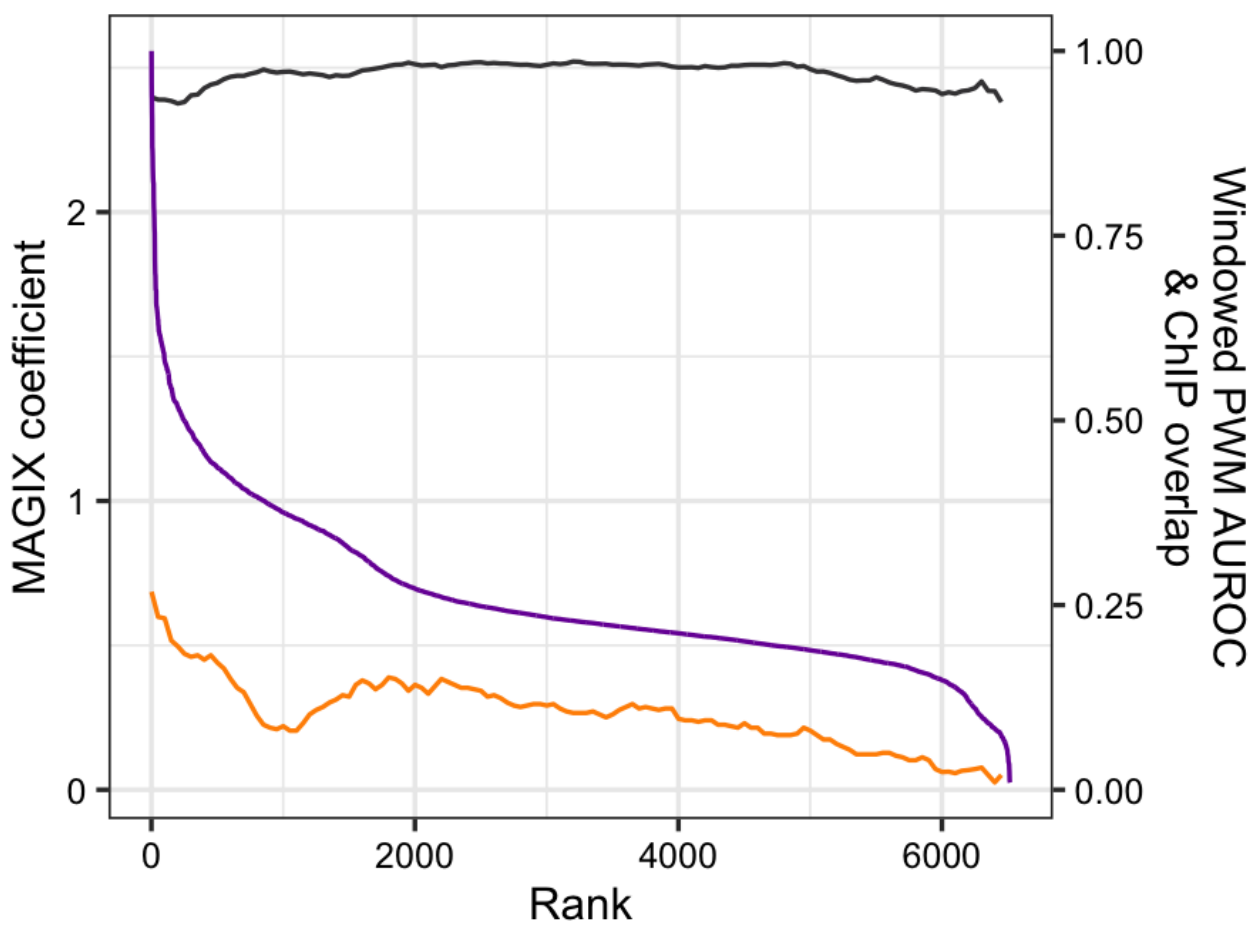

ZNF226

Top GRECO-BIT/MEX PWM: PWM003505

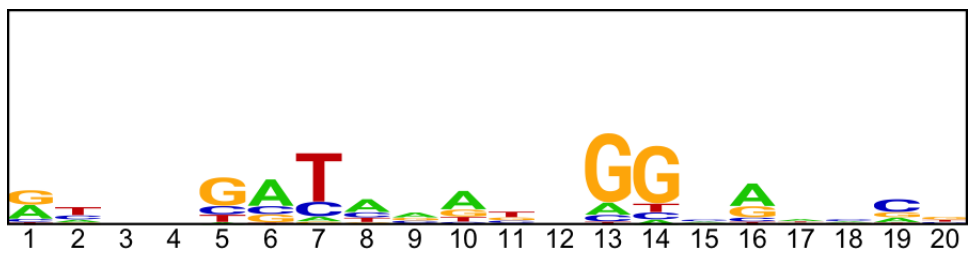

Location of top GRECO-BIT/MEX PWM in MAGIX peaks (up to the top 10,000)

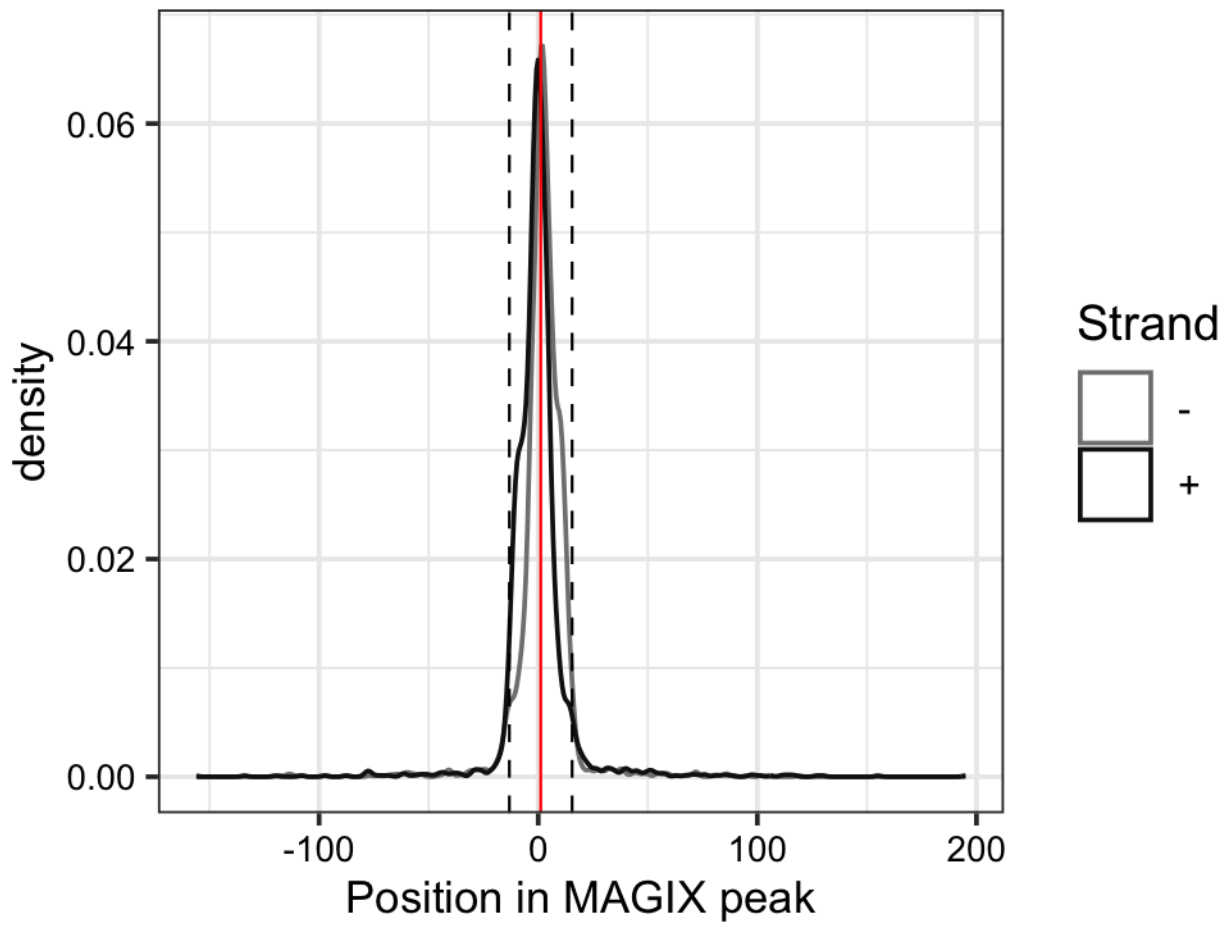

AUROC of top GRECO-BIT/MEX PWM in sliding windows (500 peaks, 50 peak step) along the MAGIX peaks (up to the top 75,000) ranked by the MAGIX enrichment coefficient and fraction of overlapping ChIP peaks in the same window

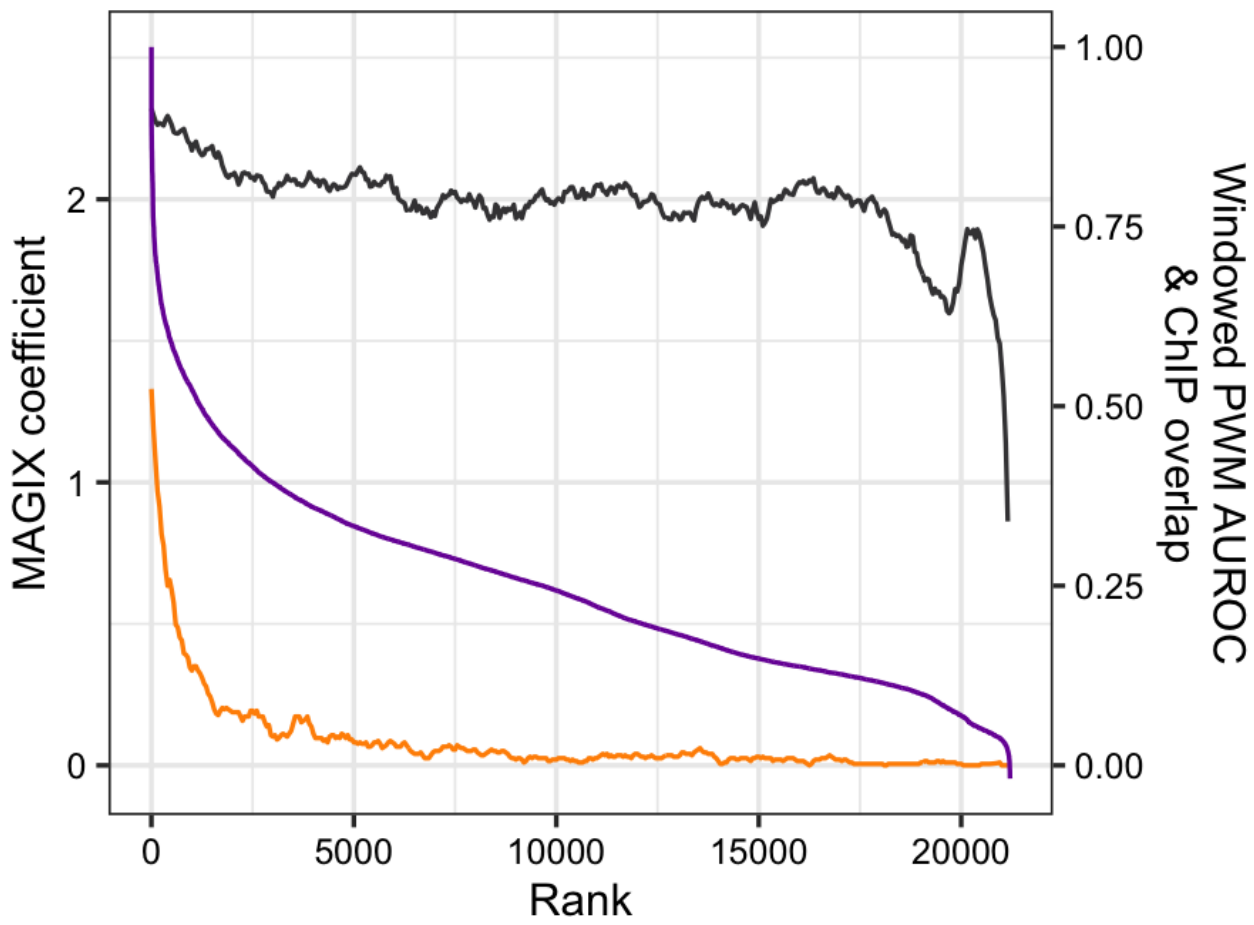

ZNF229

Top GRECO-BIT/MEX PWM: PWM003525

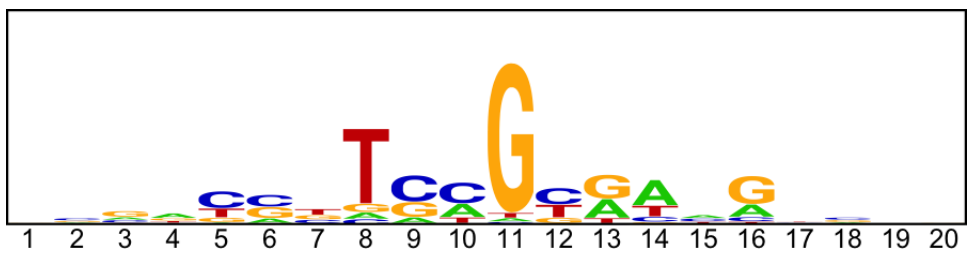

Location of top GRECO-BIT/MEX PWM in MAGIX peaks (up to the top 10,000)

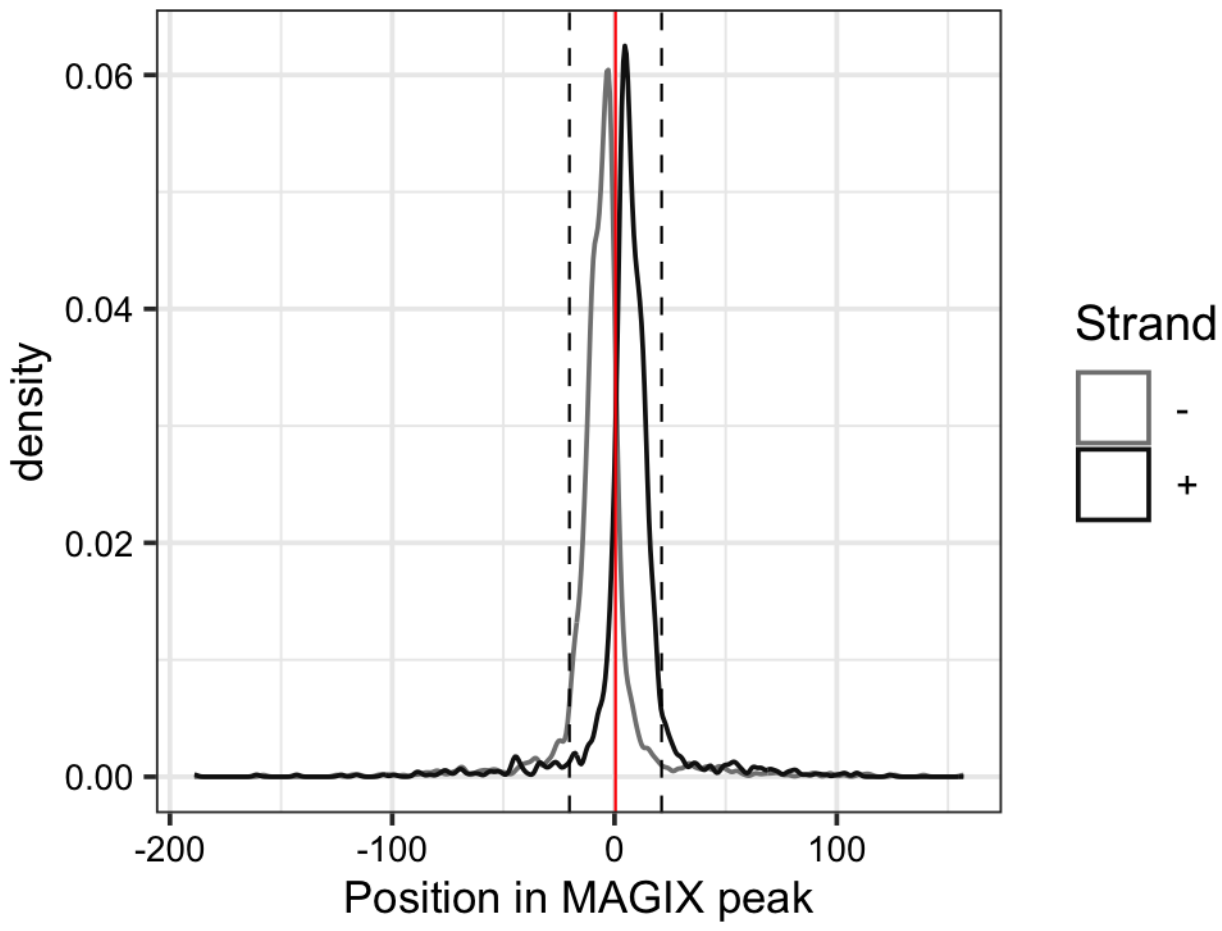

ZNF233

Top GRECO-BIT/MEX PWM: PWM003570

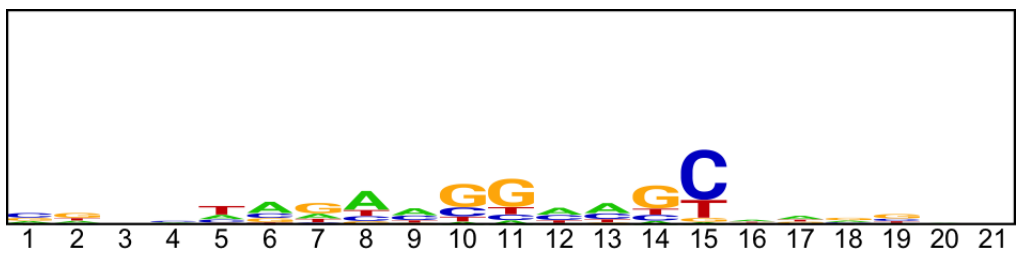

Location of top GRECO-BIT/MEX PWM in MAGIX peaks (up to the top 10,000)

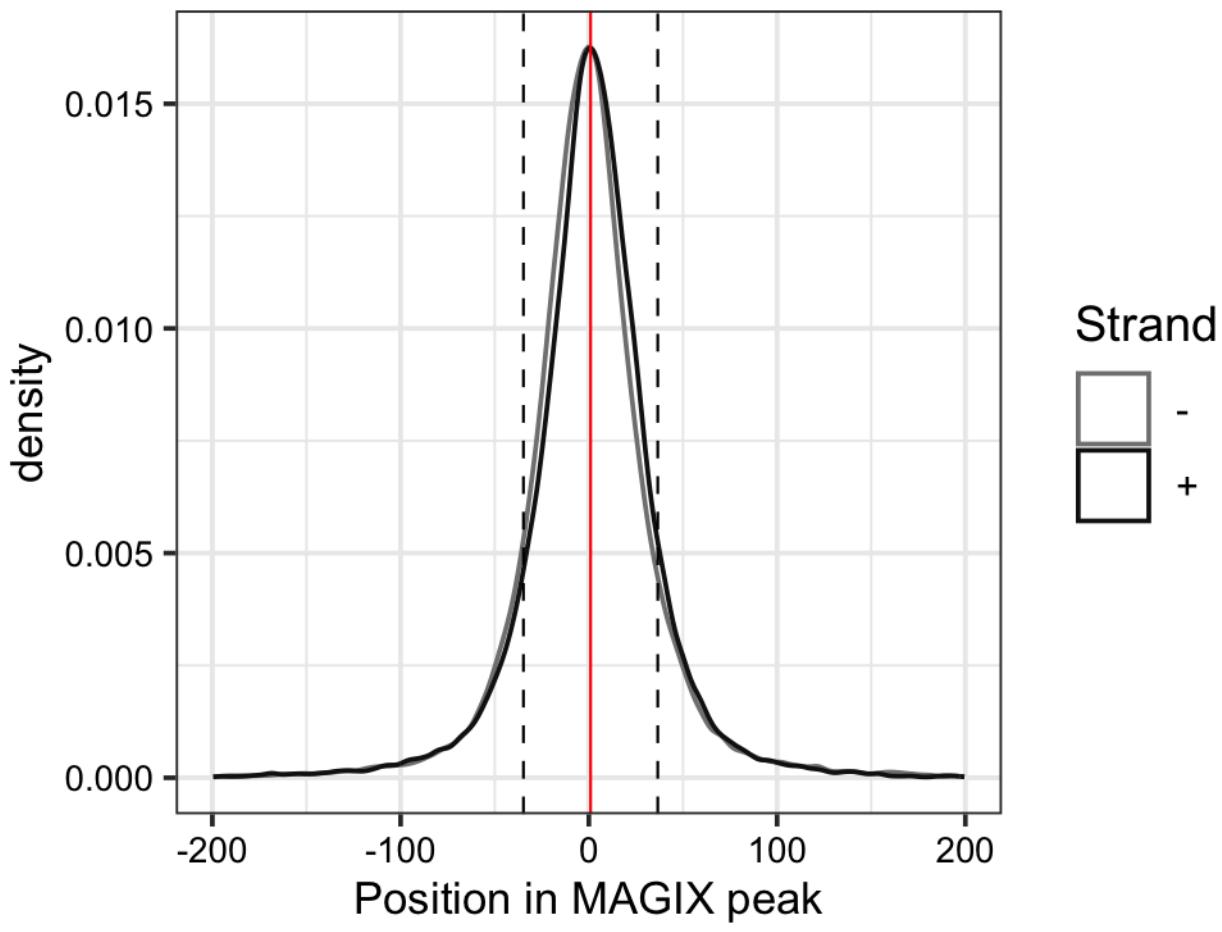

AUROC of top GRECO-BIT/MEX PWM in sliding windows (500 peaks, 50 peak step) along the MAGIX peaks (up to the top 75,000) ranked by the MAGIX enrichment coefficient and fraction of overlapping ChIP peaks in the same window

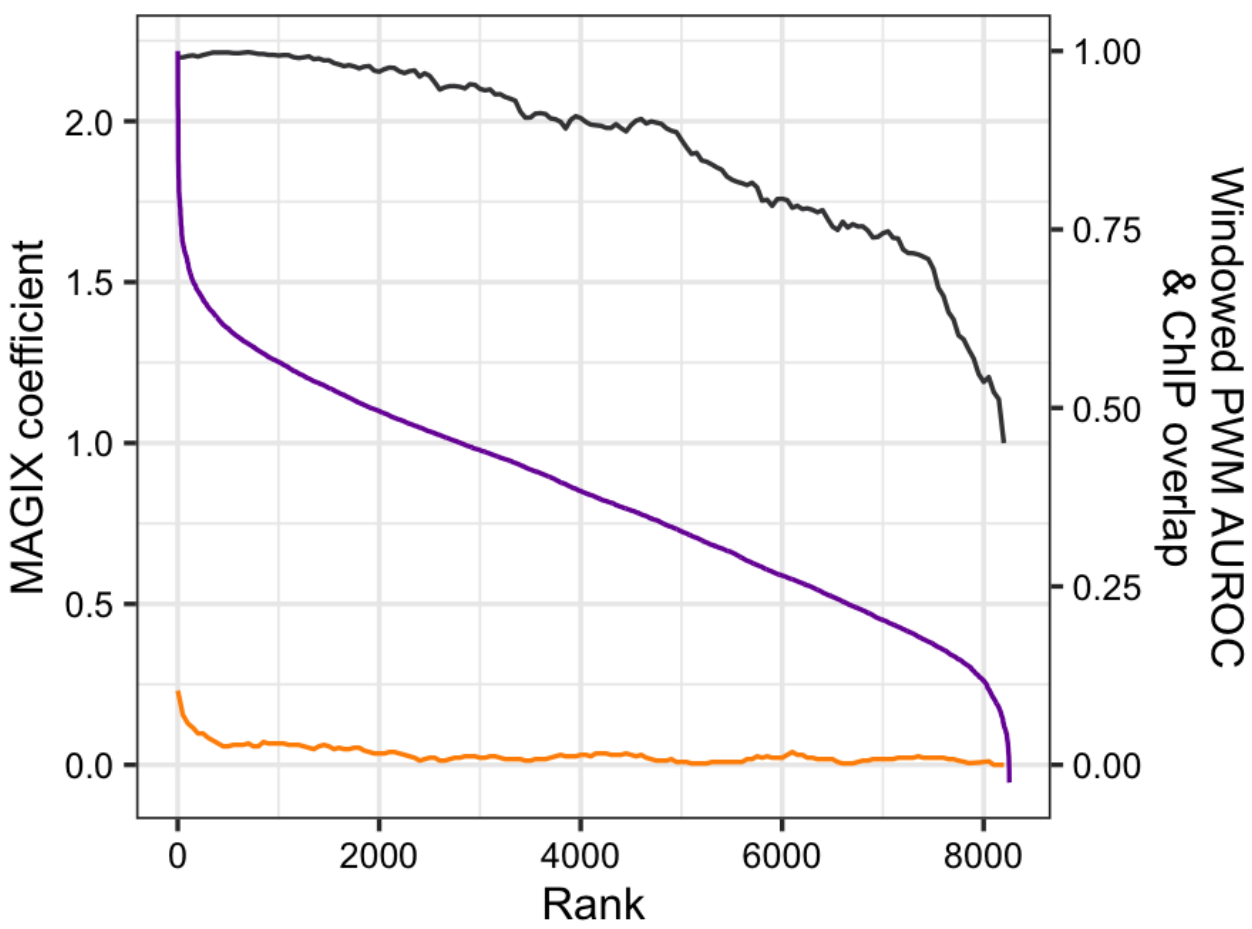

ZNF234

Top GRECO-BIT/MEX PWM: PWM003611

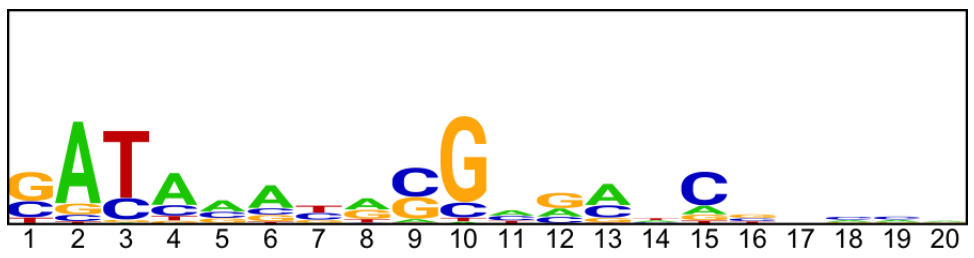

Location of top GRECO-BIT/MEX PWM in MAGIX peaks (up to the top 10,000)

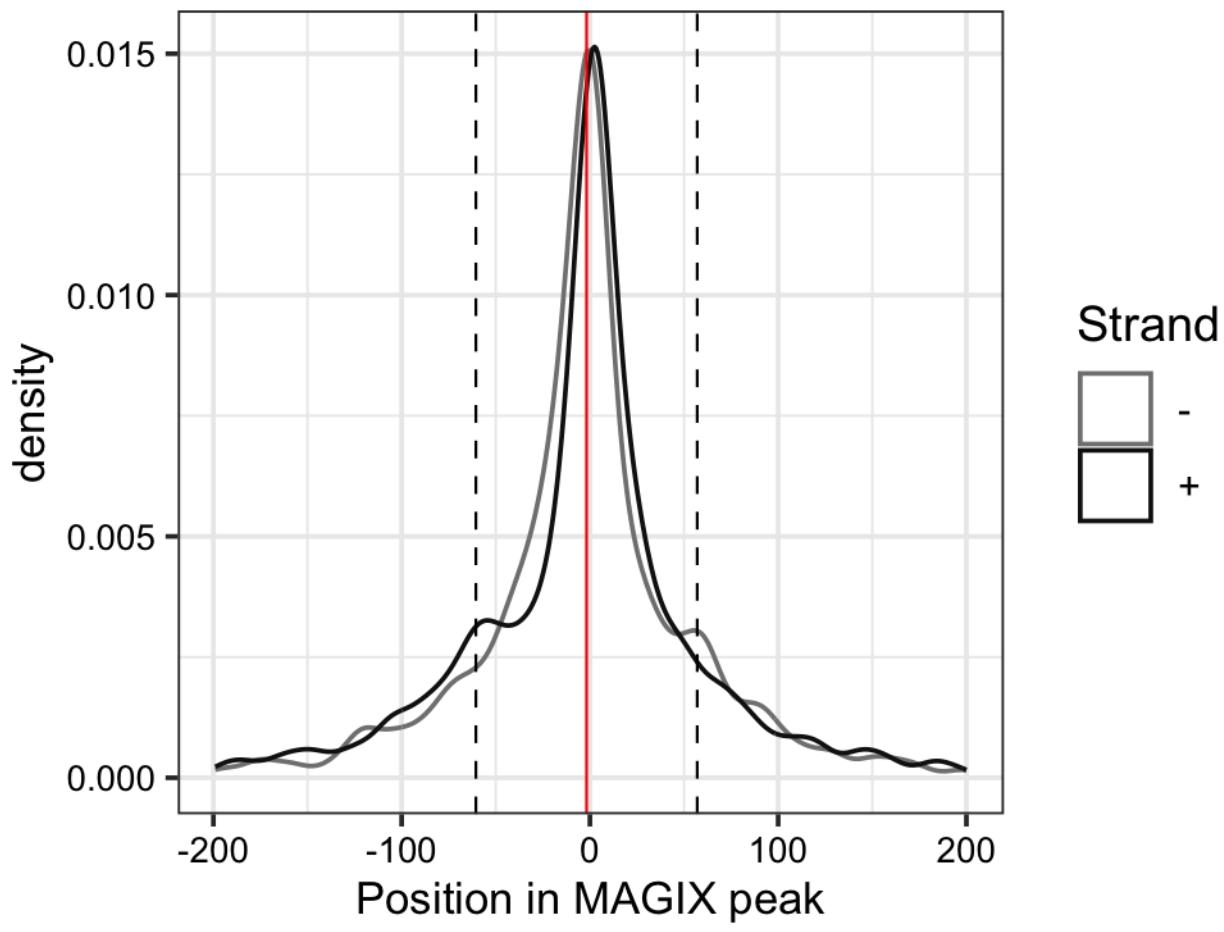

AUROC of top GRECO-BIT/MEX PWM in sliding windows (500 peaks, 50 peak step) along the MAGIX peaks (up to the top 75,000) ranked by the MAGIX enrichment coefficient and fraction of overlapping ChIP peaks in the same window

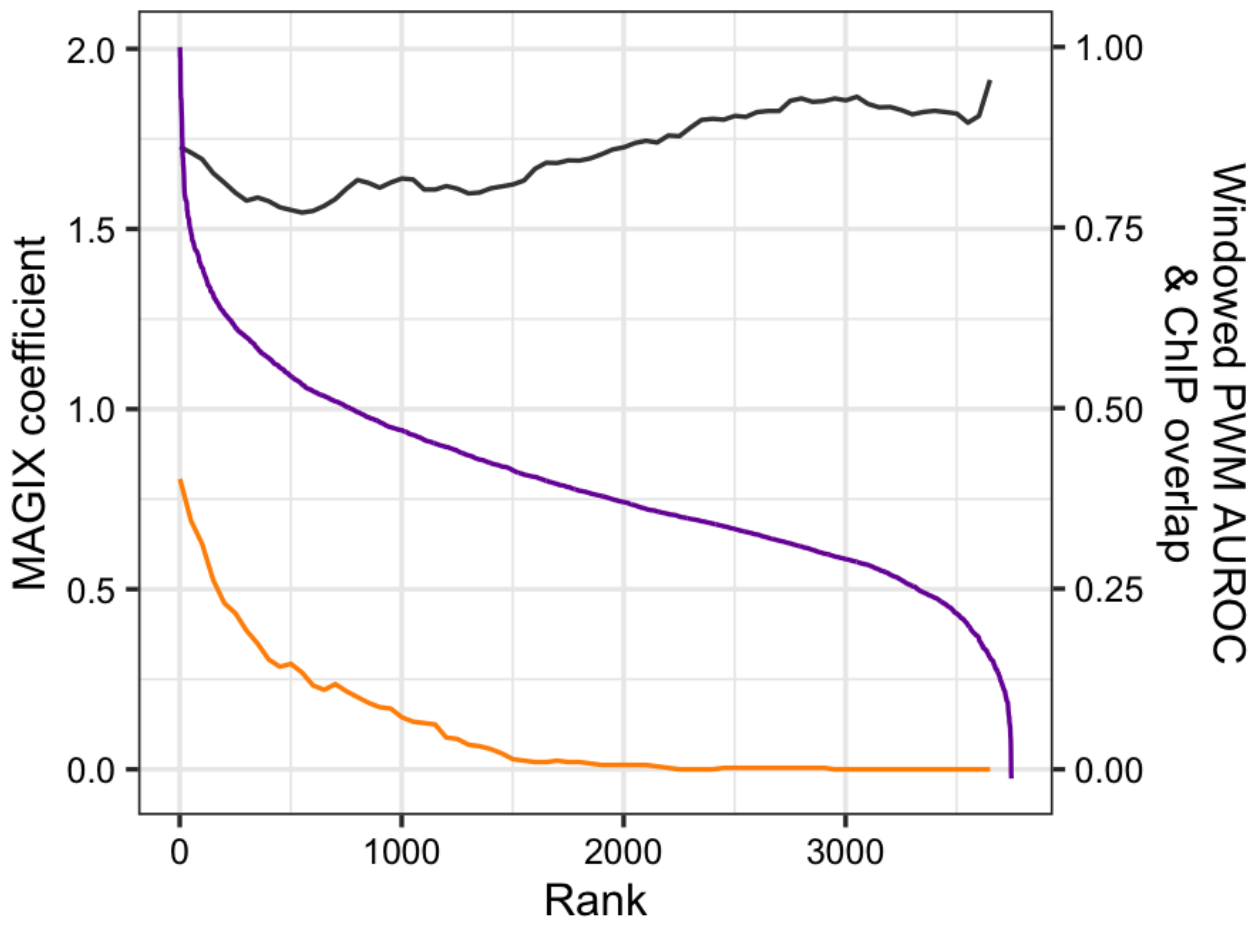

ZNF250

Top GRECO-BIT/MEX PWM: PWM003634

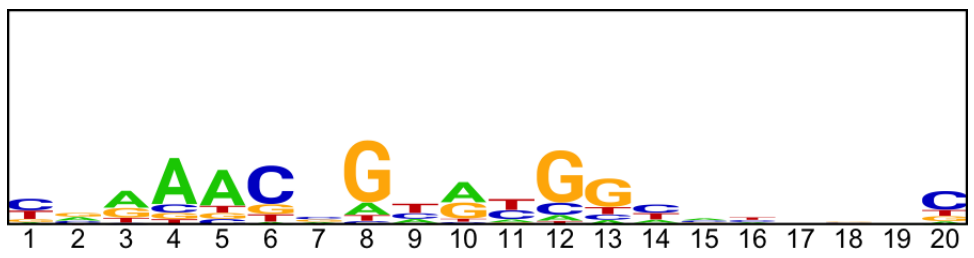

Location of top GRECO-BIT/MEX PWM in MAGIX peaks (up to the top 10,000)

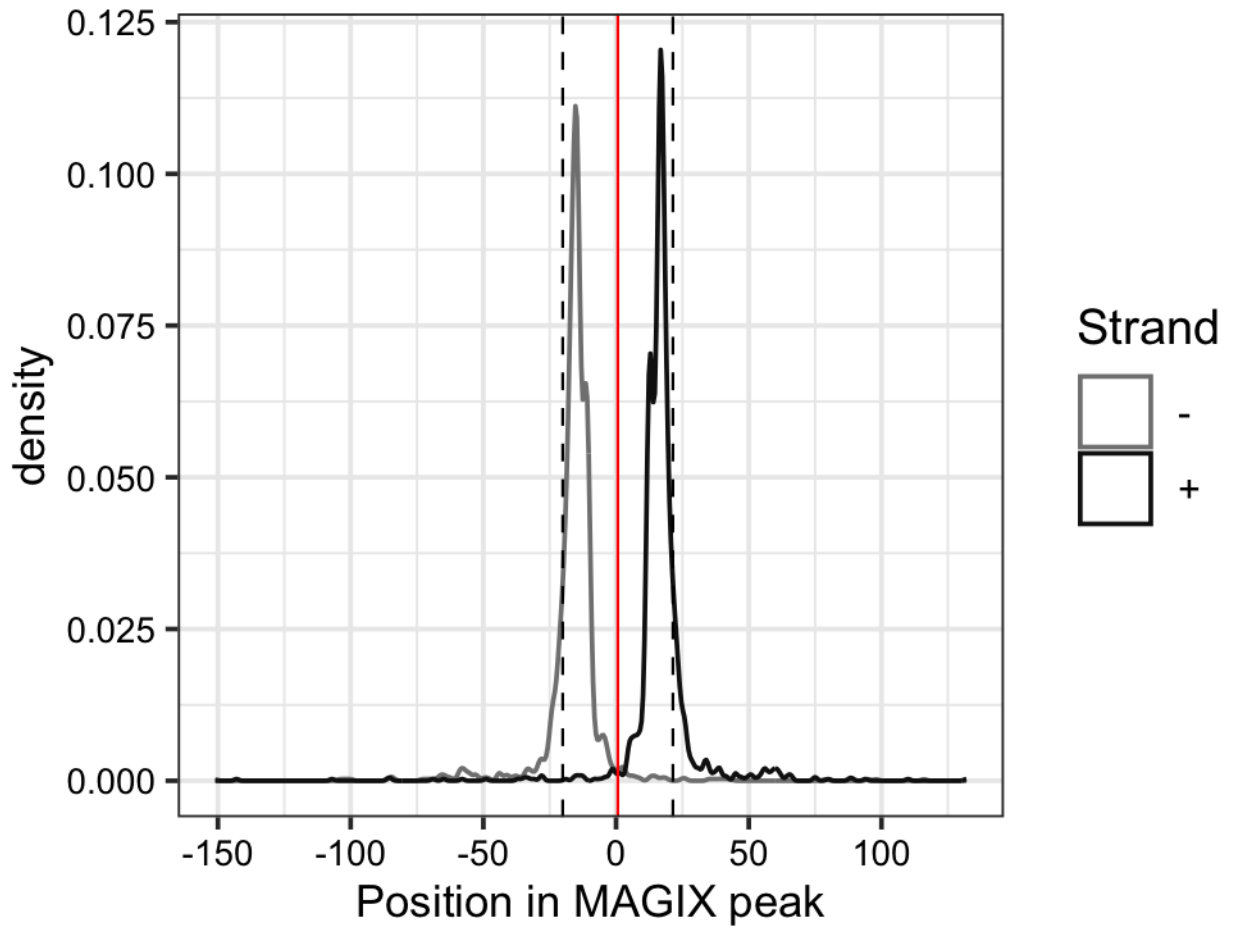

AUROC of top GRECO-BIT/MEX PWM in sliding windows (500 peaks, 50 peak step) along the MAGIX peaks (up to the top 75,000) ranked by the MAGIX enrichment coefficient and fraction of overlapping ChIP peaks in the same window

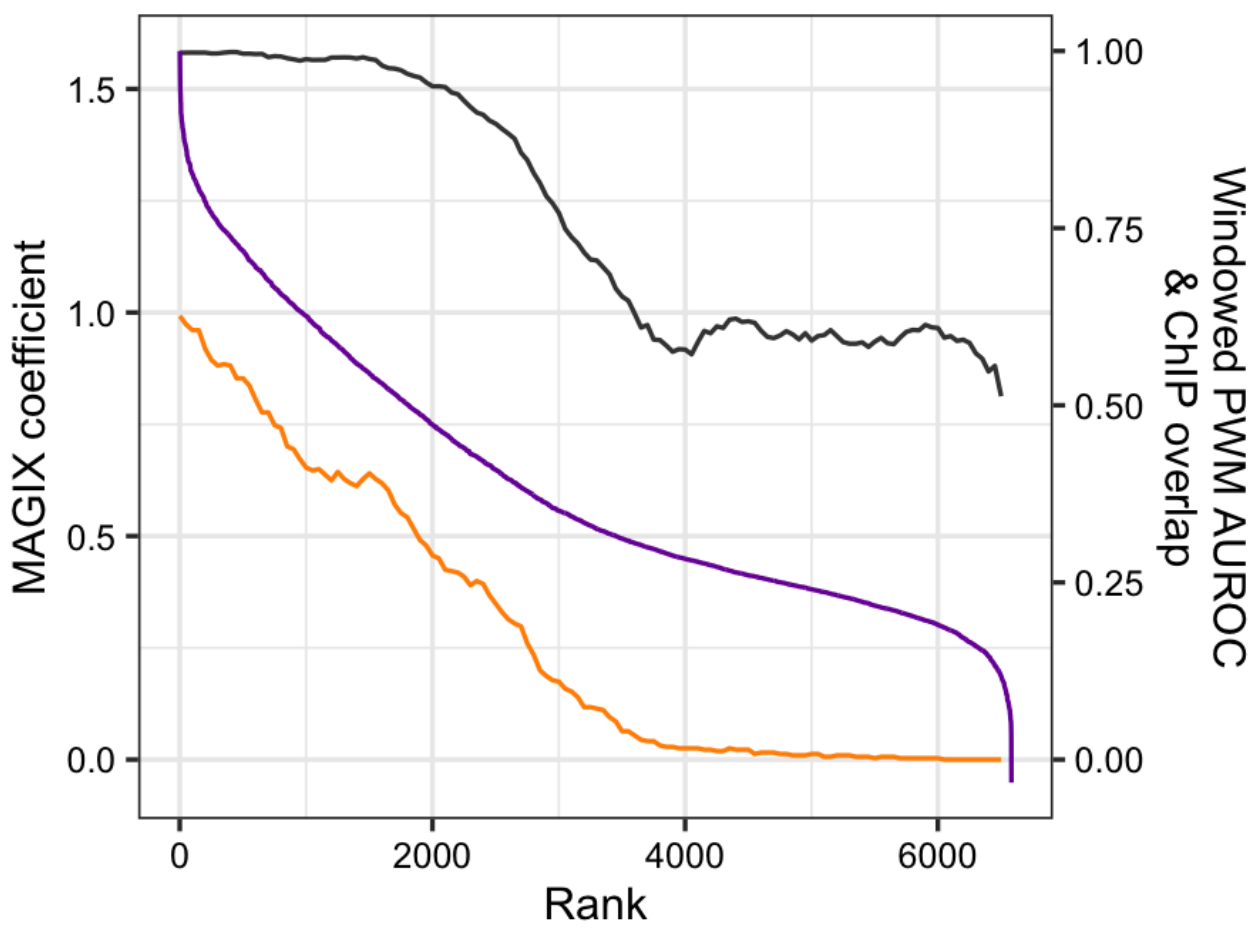

ZNF251

Top GRECO-BIT/MEX PWM: PWM003657

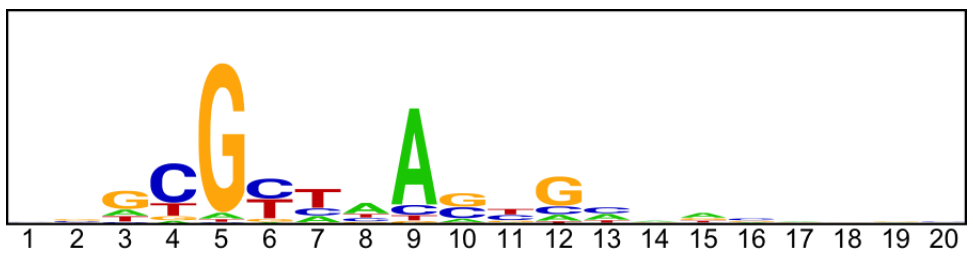

Location of top GRECO-BIT/MEX PWM in MAGIX peaks (up to the top 10,000)

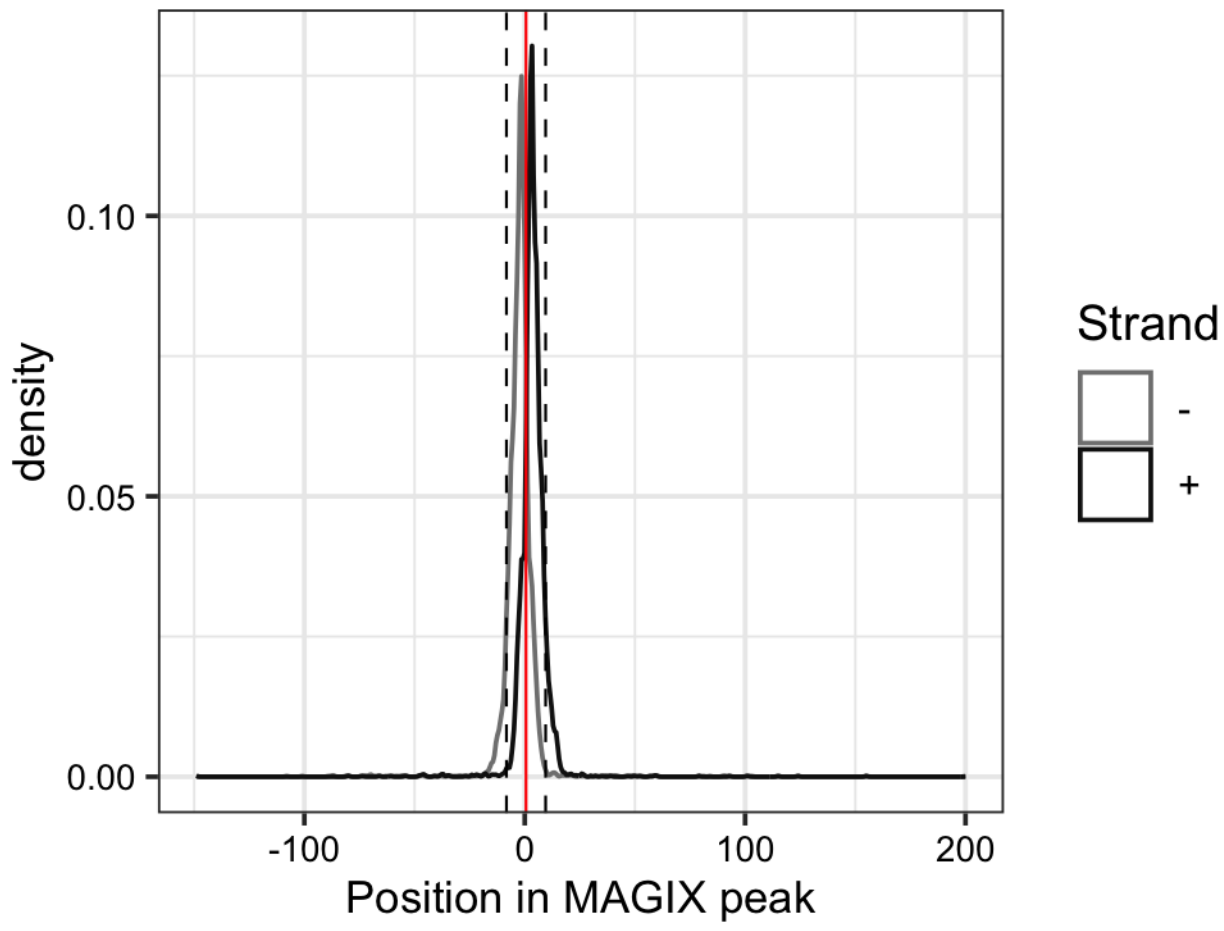

AUROC of top GRECO-BIT/MEX PWM in sliding windows (500 peaks, 50 peak step) along the MAGIX peaks (up to the top 75,000) ranked by the MAGIX enrichment coefficient and fraction of overlapping ChIP peaks in the same window

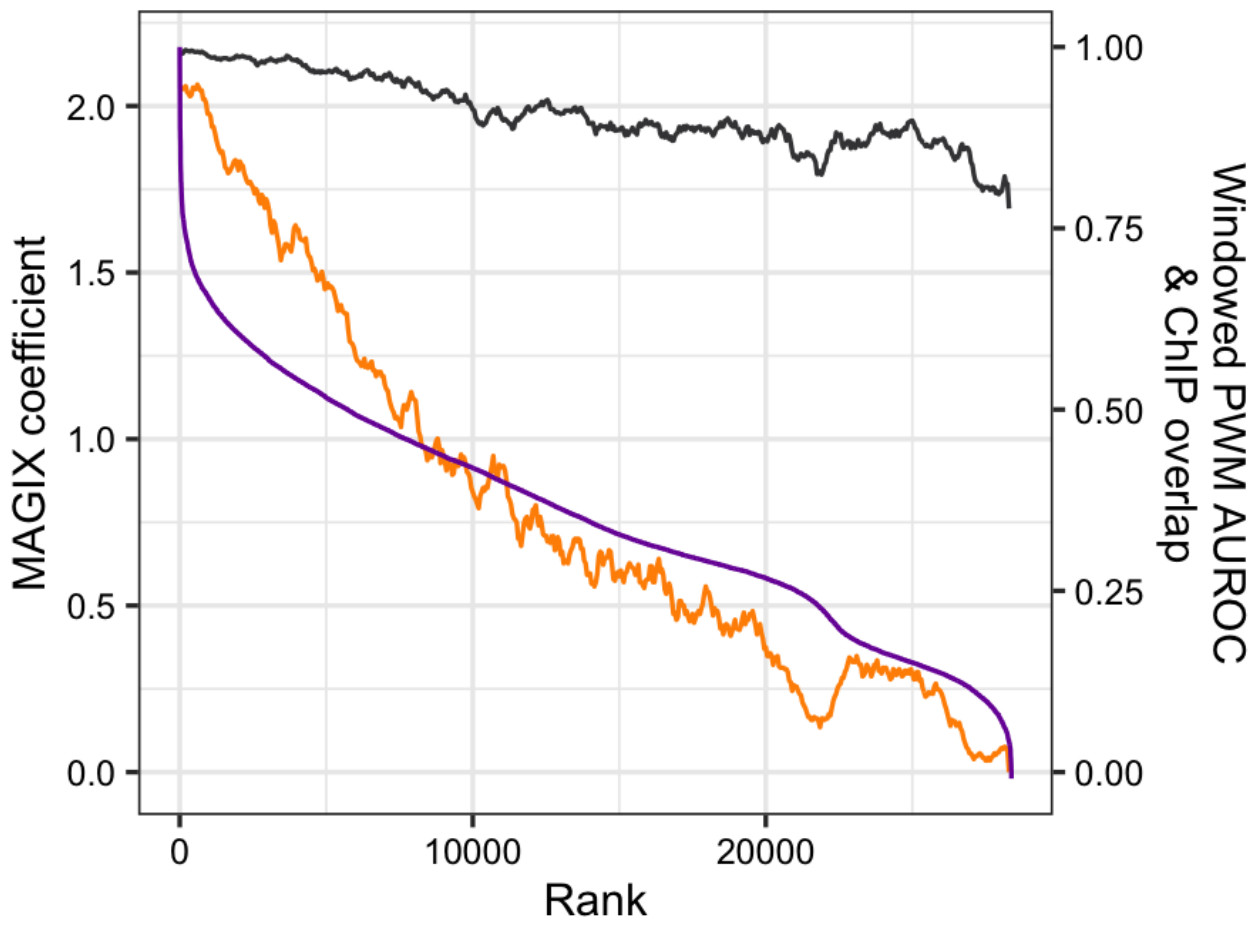

ZNF260

Top GRECO-BIT/MEX PWM: PWM003683

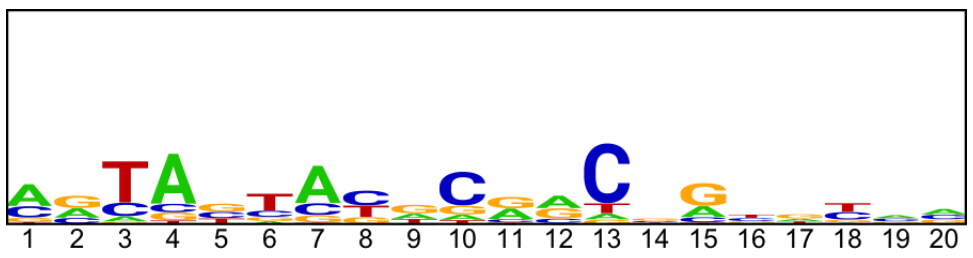

Location of top GRECO-BIT/MEX PWM in MAGIX peaks (up to the top 10,000)

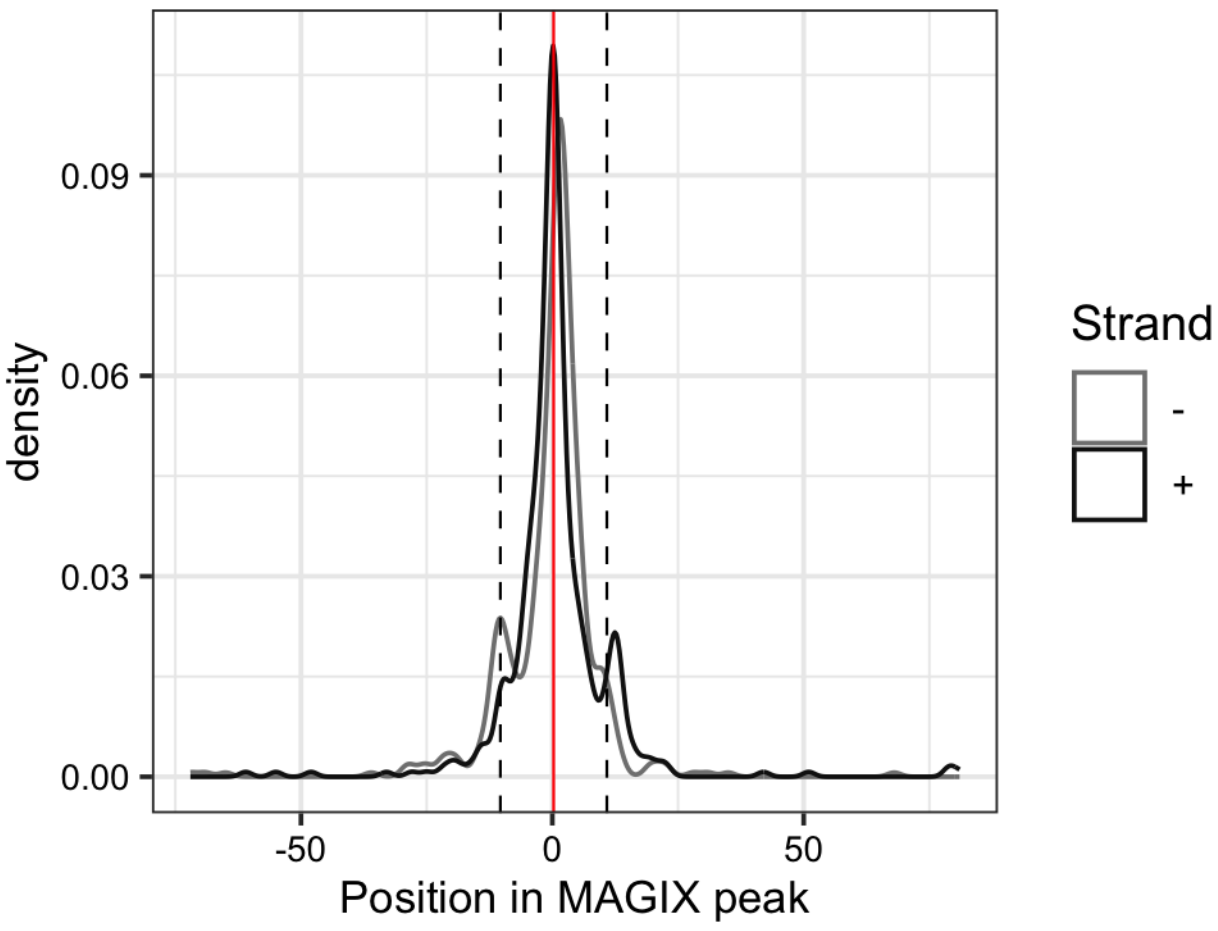

AUROC of top GRECO-BIT/MEX PWM in sliding windows (500 peaks, 50 peak step) along the MAGIX peaks (up to the top 75,000) ranked by the MAGIX enrichment coefficient and fraction of overlapping ChIP peaks in the same window

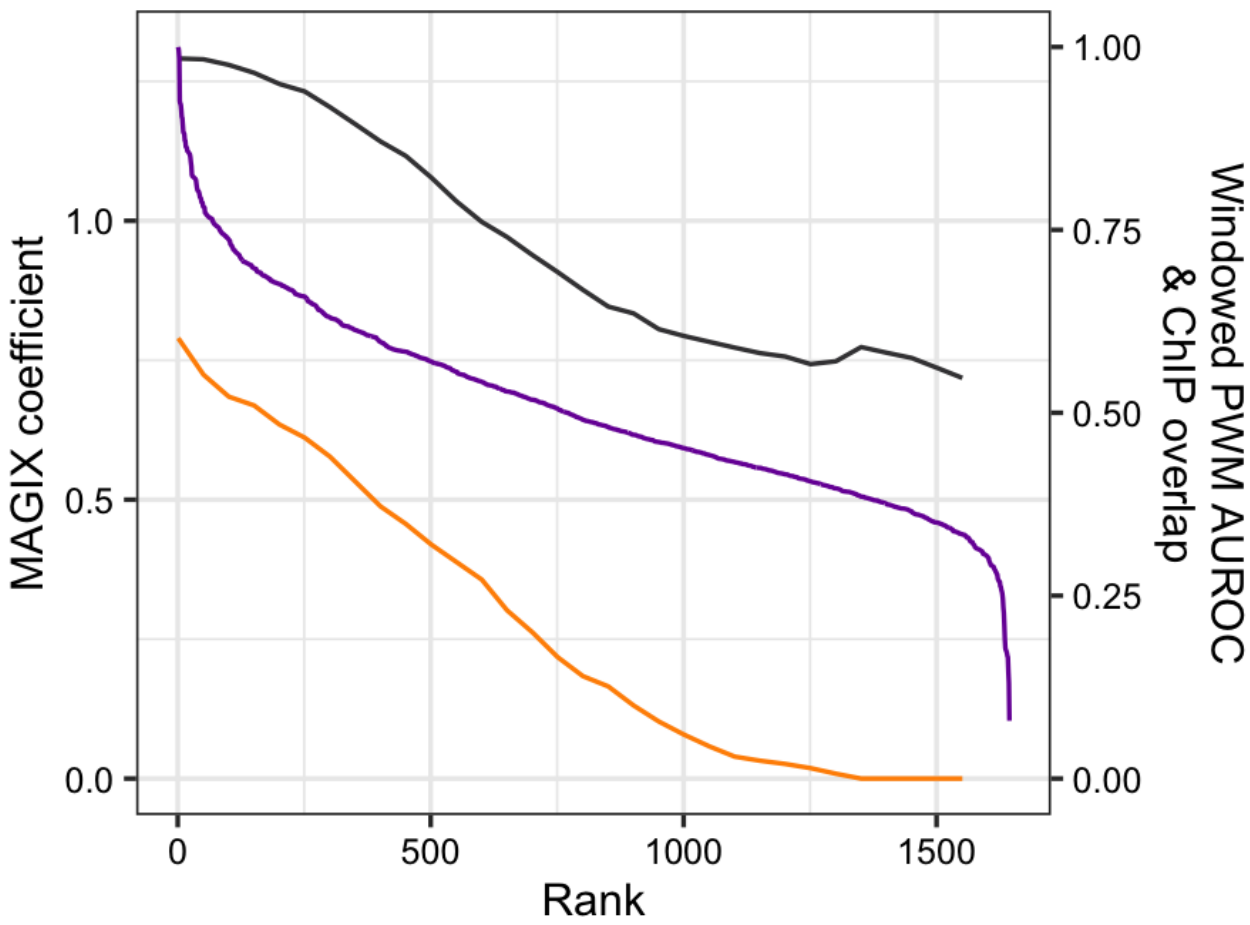

ZNF264

Top GRECO-BIT/MEX PWM: PWM003714

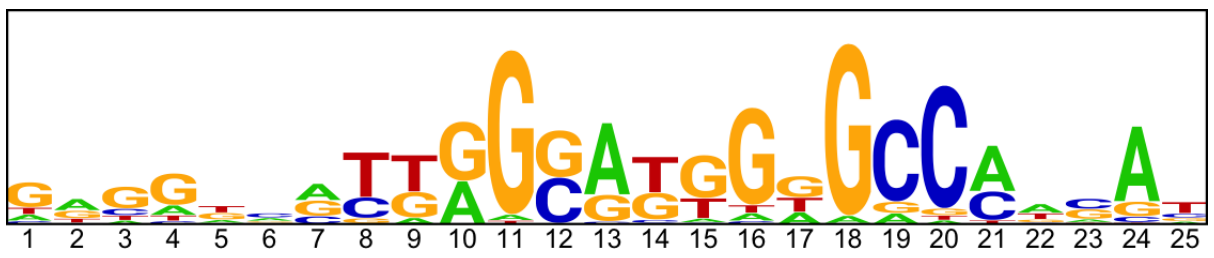

Location of top GRECO-BIT/MEX PWM in MAGIX peaks (up to the top 10,000)

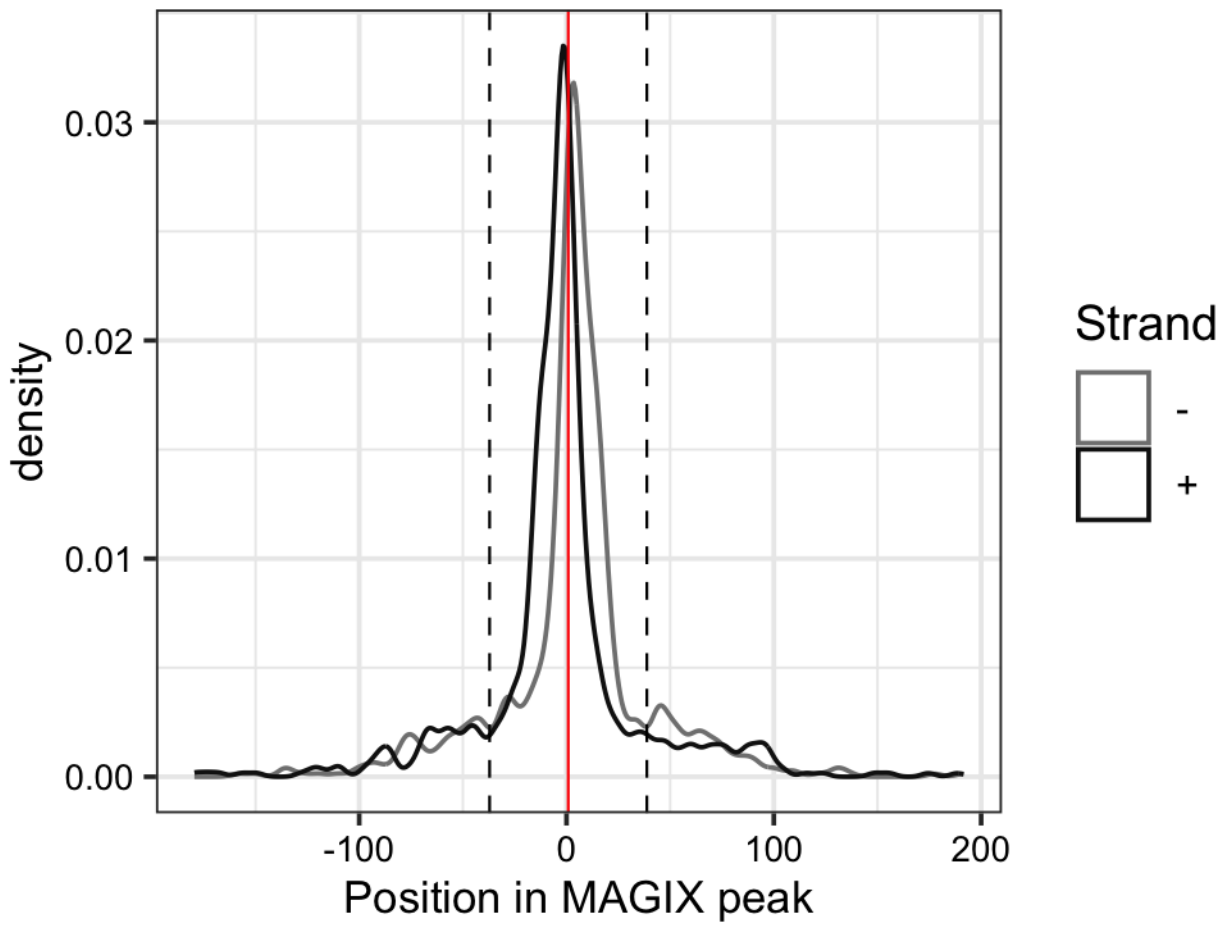

AUROC of top GRECO-BIT/MEX PWM in sliding windows (500 peaks, 50 peak step) along the MAGIX peaks (up to the top 75,000) ranked by the MAGIX enrichment coefficient and fraction of overlapping ChIP peaks in the same window

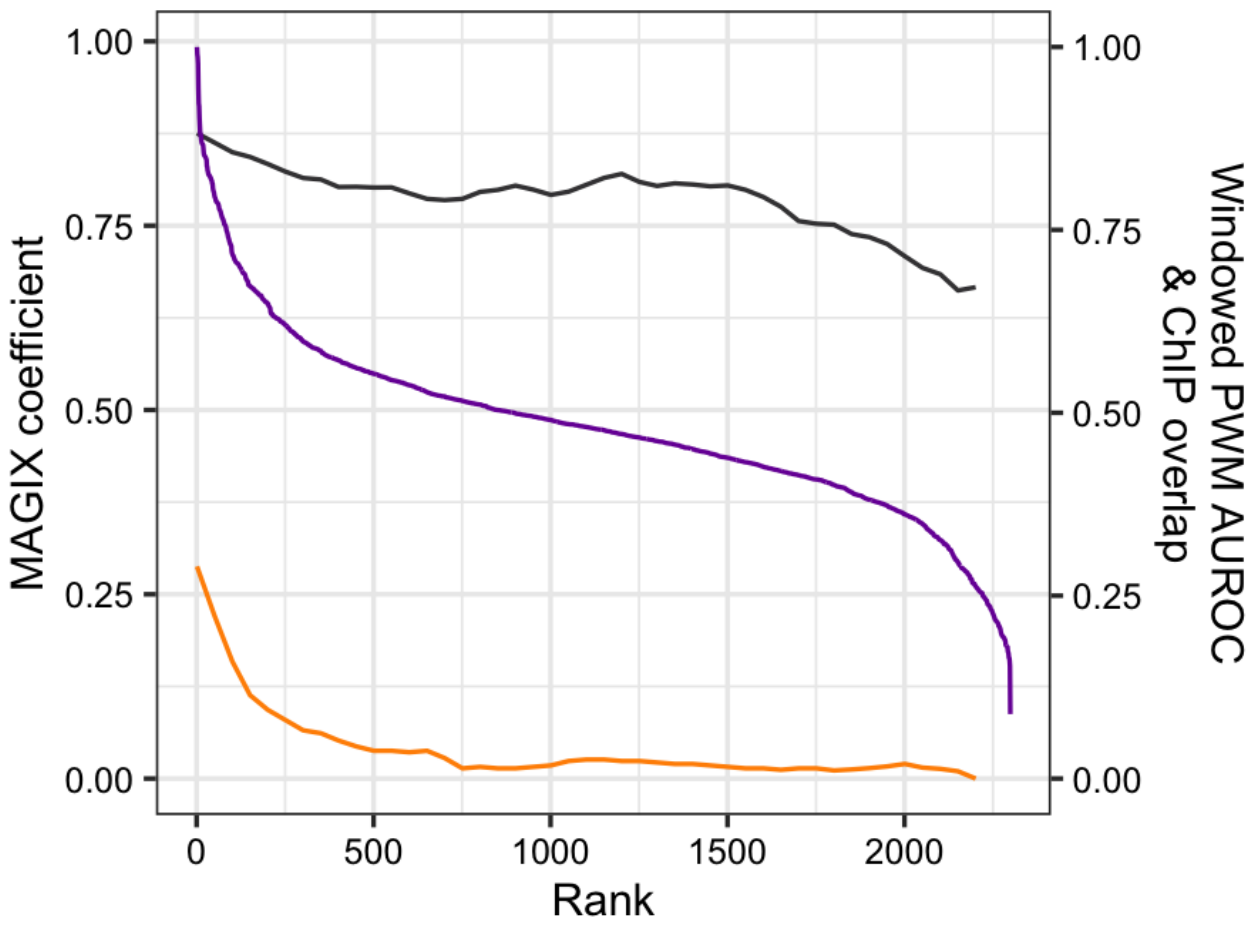

ZNF275

Top GRECO-BIT/MEX PWM: PWM003734

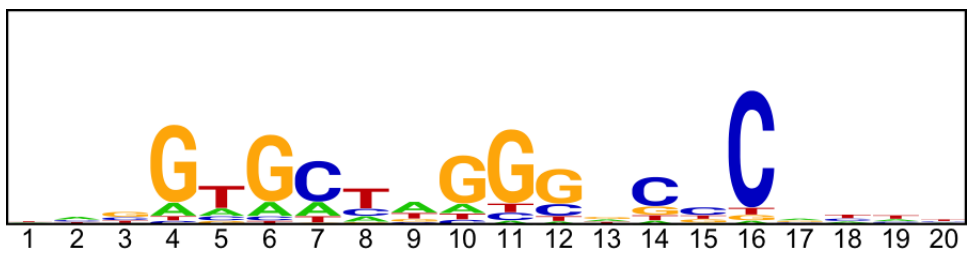

Location of top GRECO-BIT/MEX PWM in MAGIX peaks (up to the top 10,000)

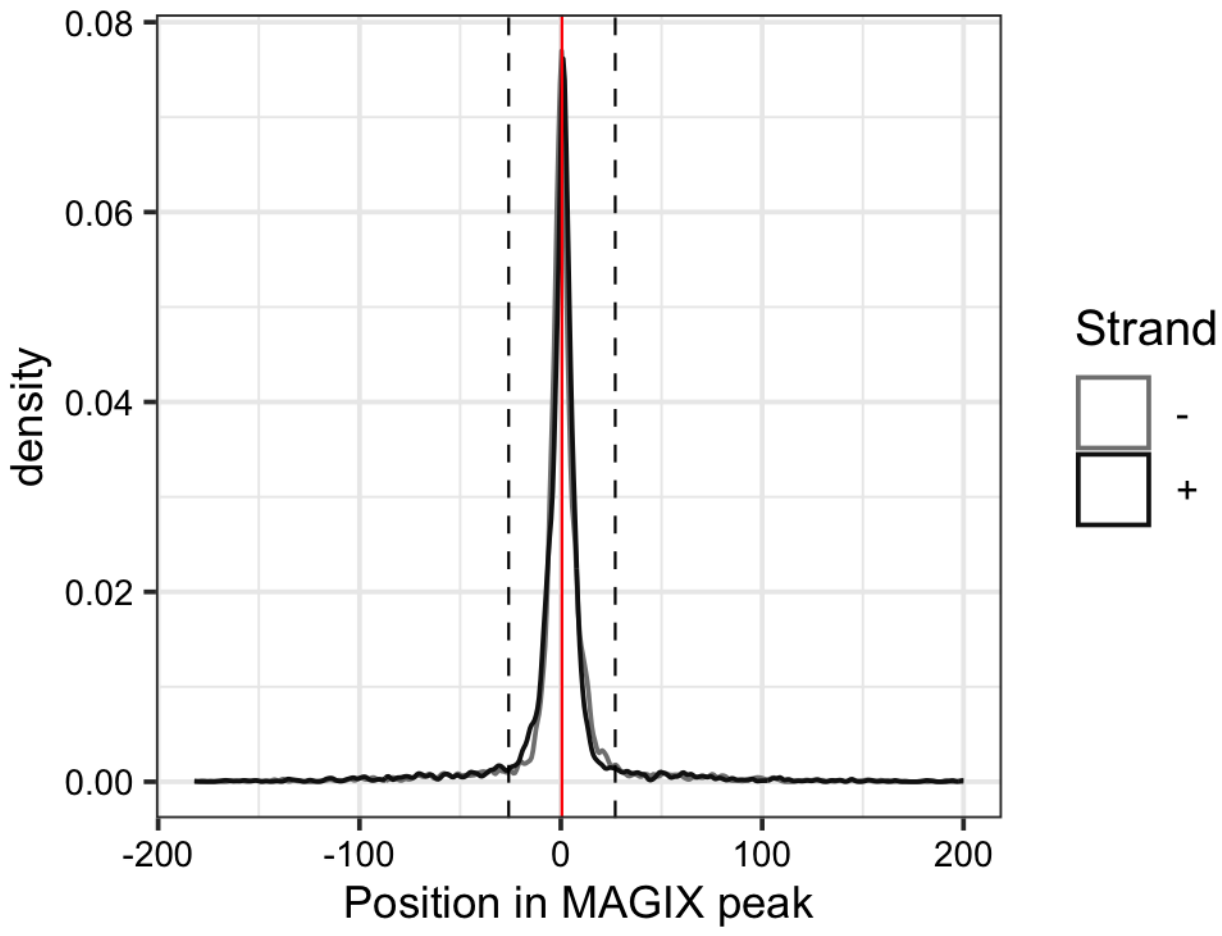

ZNF286B

Top GRECO-BIT/MEX PWM: PWM003759

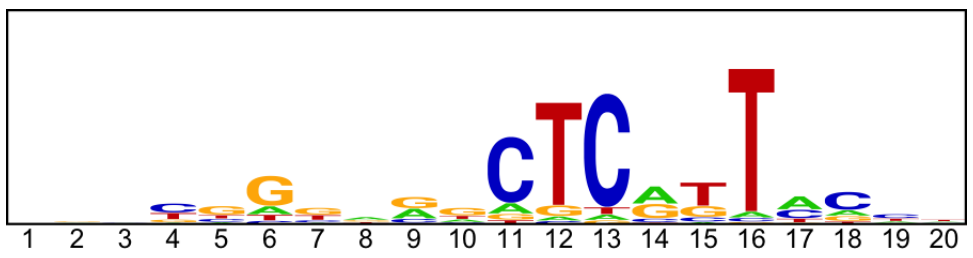

Location of top GRECO-BIT/MEX PWM in MAGIX peaks (up to the top 10,000)

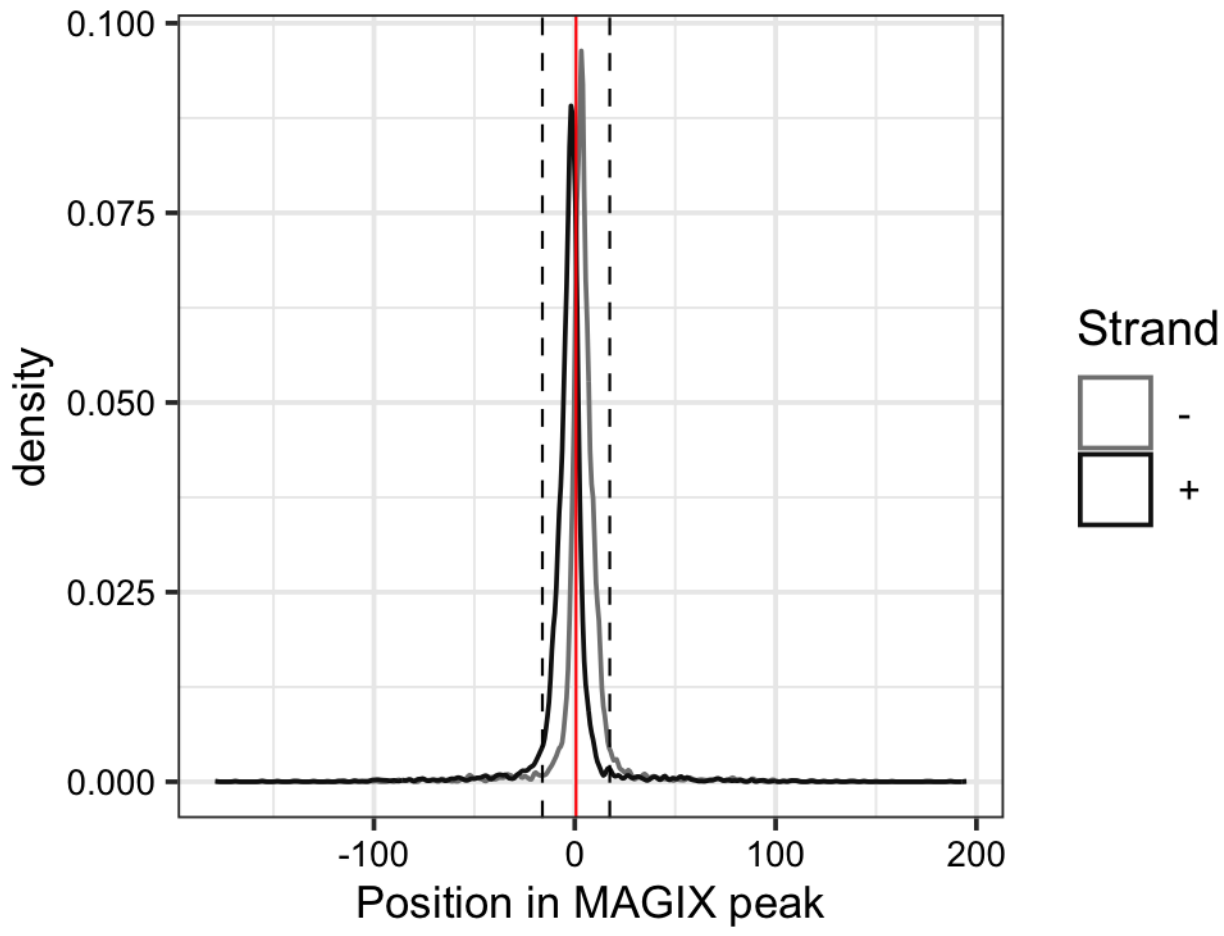

AUROC of top GRECO-BIT/MEX PWM in sliding windows (500 peaks, 50 peak step) along the MAGIX peaks (up to the top 75,000) ranked by the MAGIX enrichment coefficient and fraction of overlapping ChIP peaks in the same window

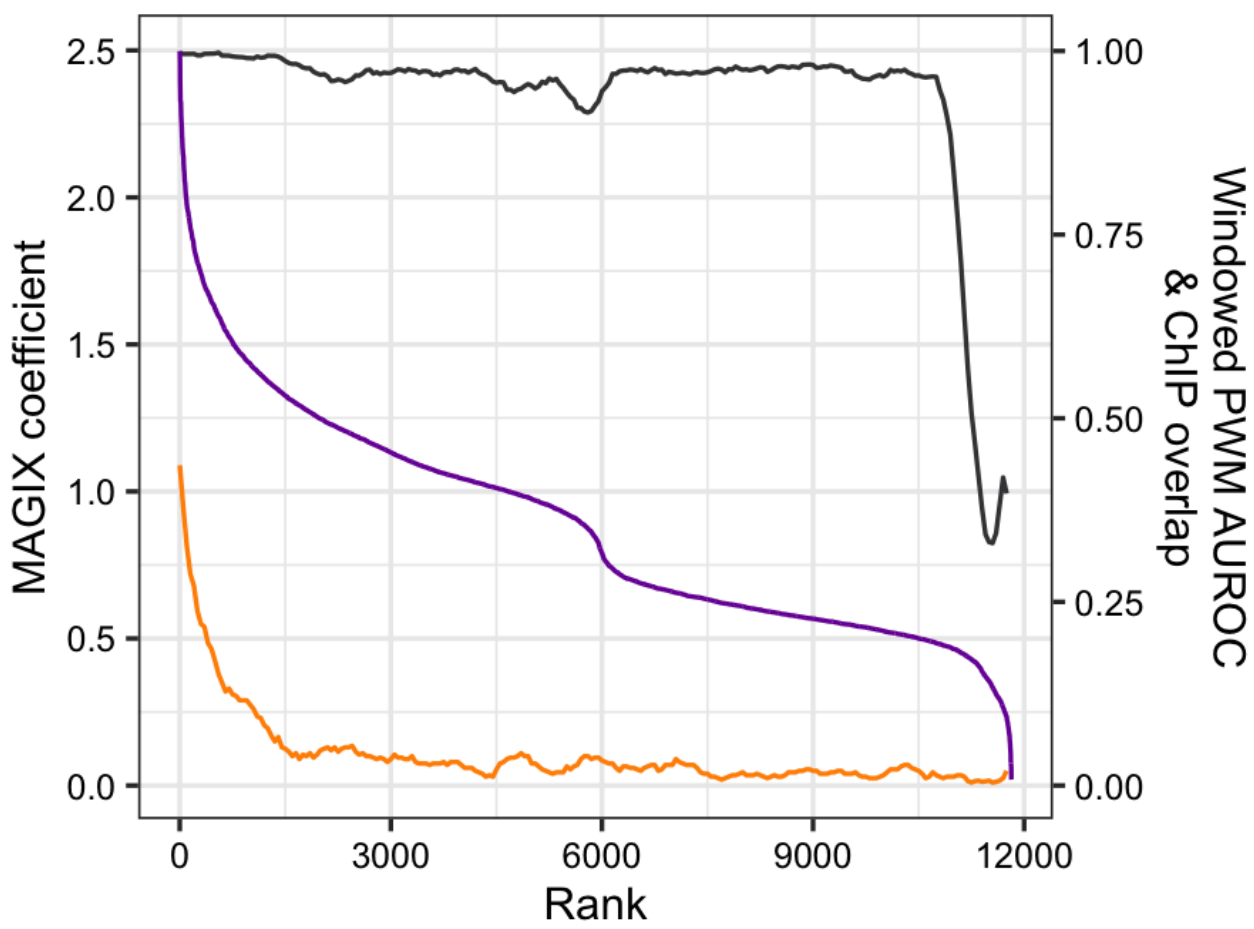

ZNF292.DBD1

Top GRECO-BIT/MEX PWM: PWM003802

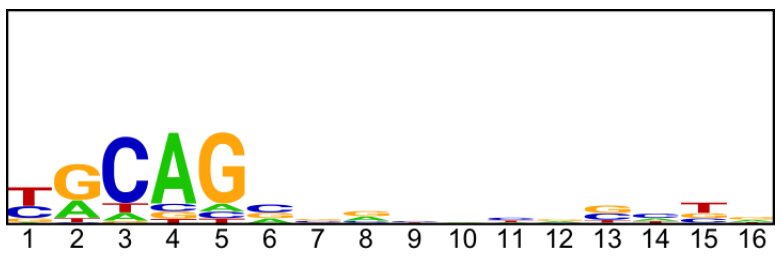

Location of top GRECO-BIT/MEX PWM in MAGIX peaks (up to the top 10,000)

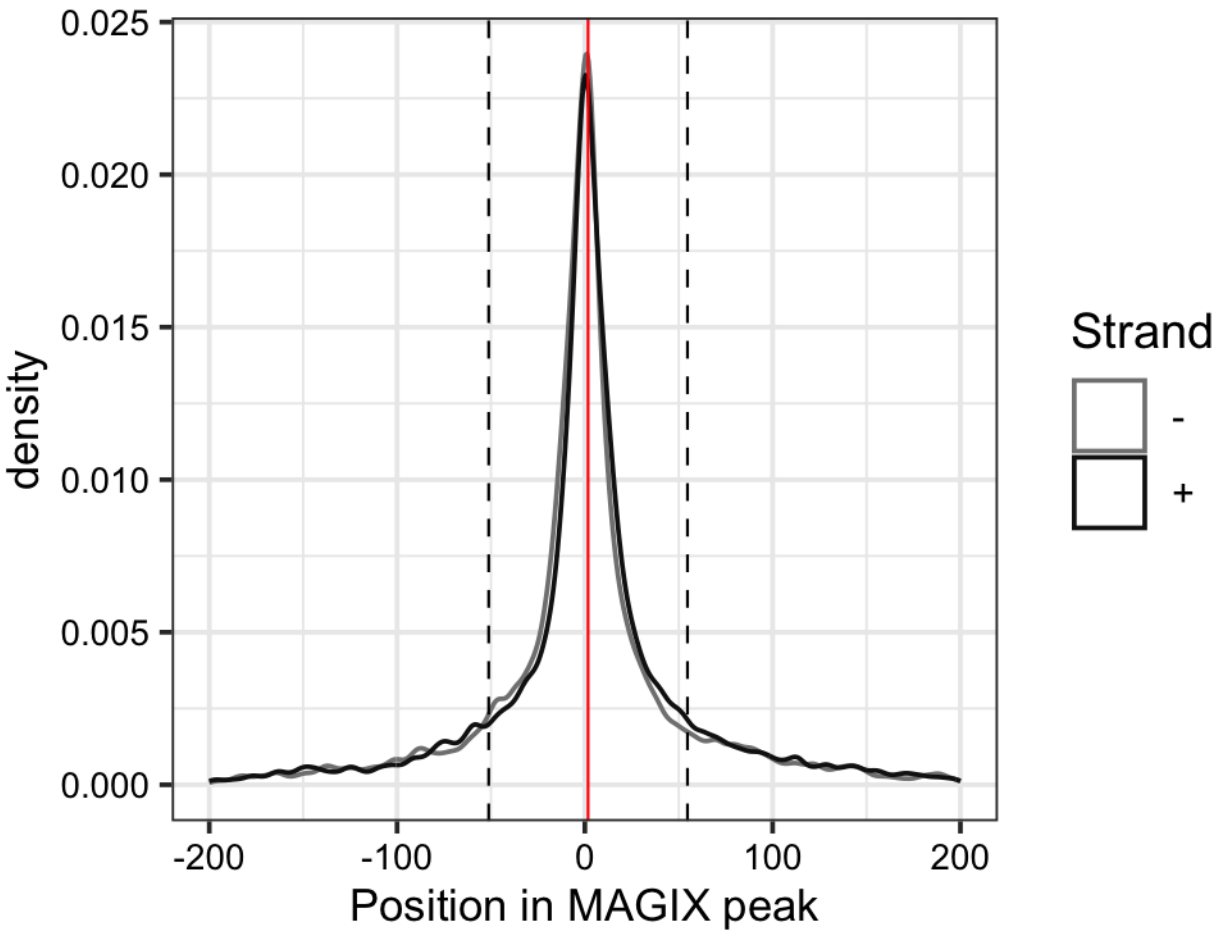

ZNF292.DBD2

Top GRECO-BIT/MEX PWM: PWM003788

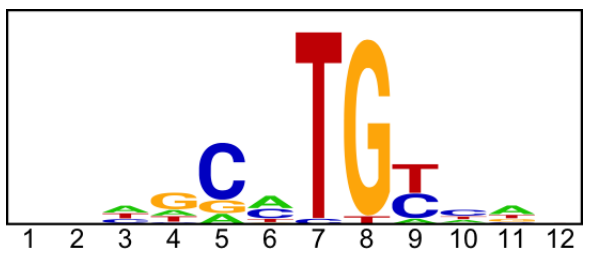

Location of top GRECO-BIT/MEX PWM in MAGIX peaks (up to the top 10,000)

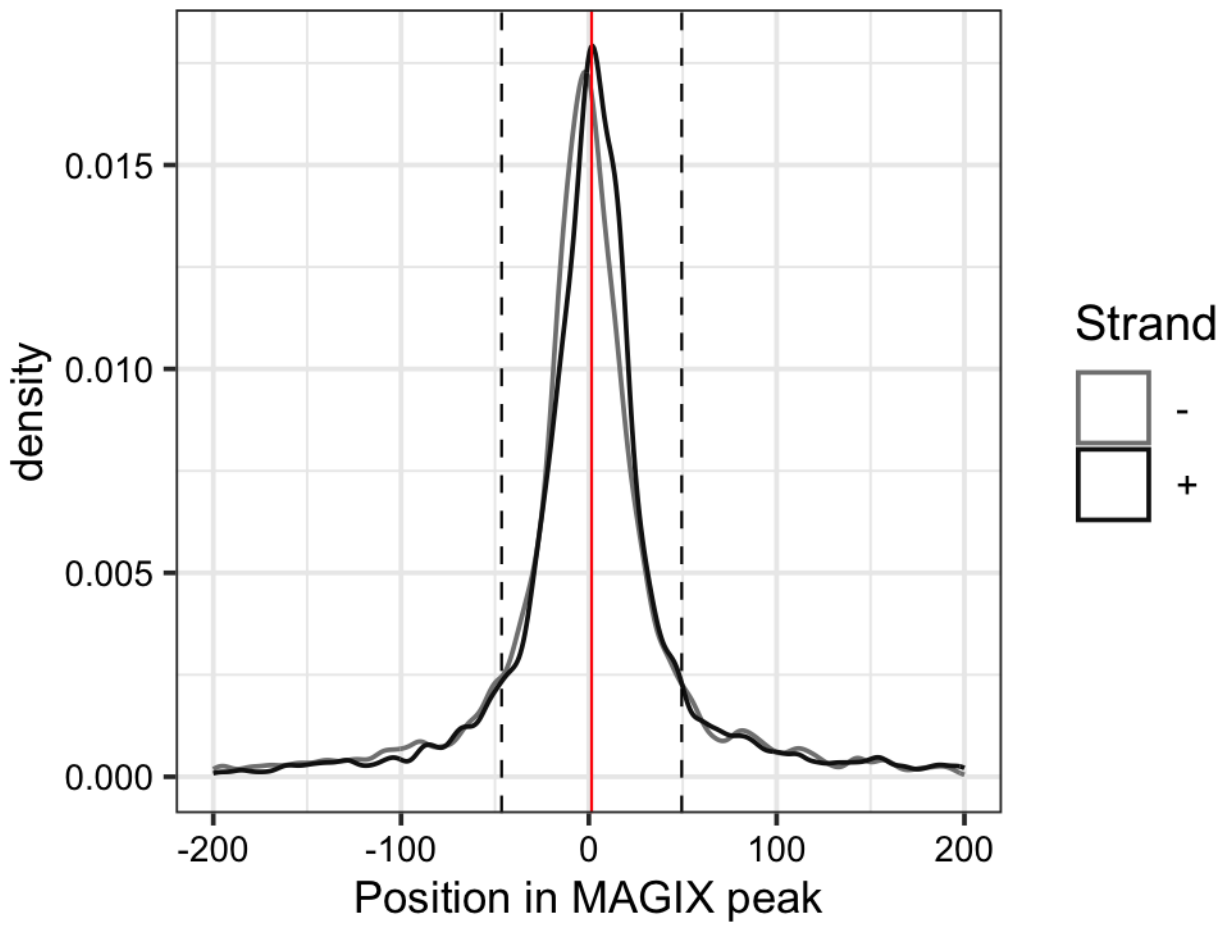

ZNF322

Top GRECO-BIT/MEX PWM: PWM003869

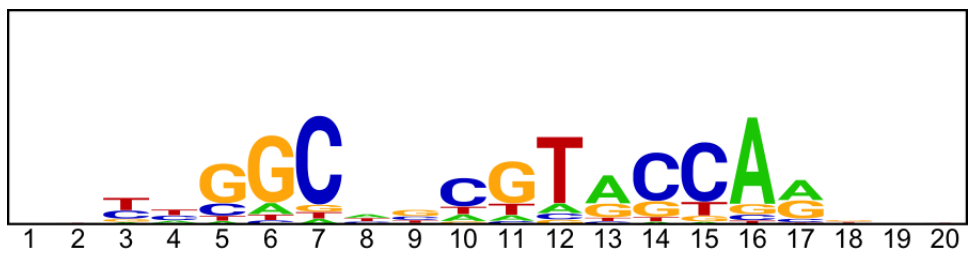

Location of top GRECO-BIT/MEX PWM in MAGIX peaks (up to the top 10,000)

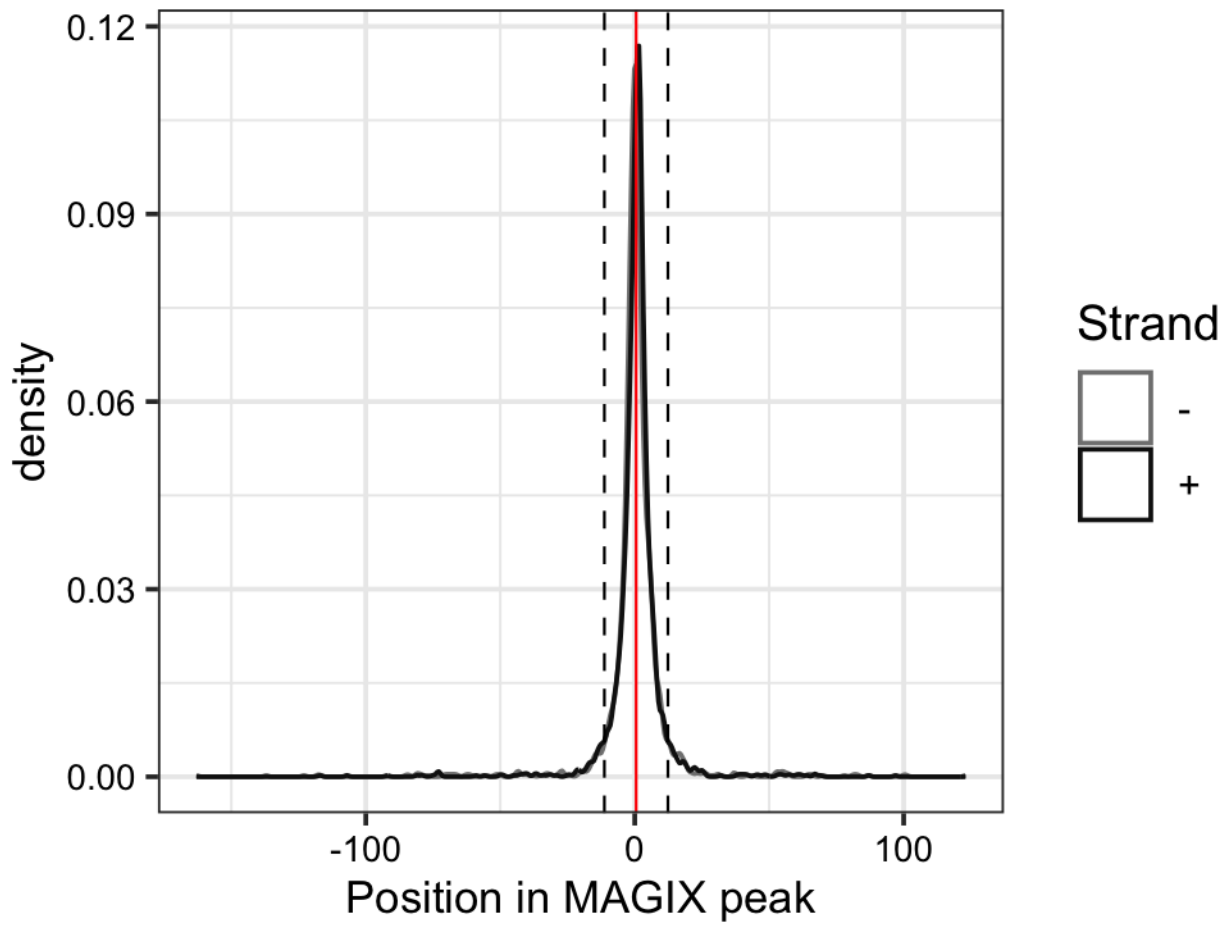

AUROC of top GRECO-BIT/MEX PWM in sliding windows (500 peaks, 50 peak step) along the MAGIX peaks (up to the top 75,000) ranked by the MAGIX enrichment coefficient and fraction of overlapping ChIP peaks in the same window

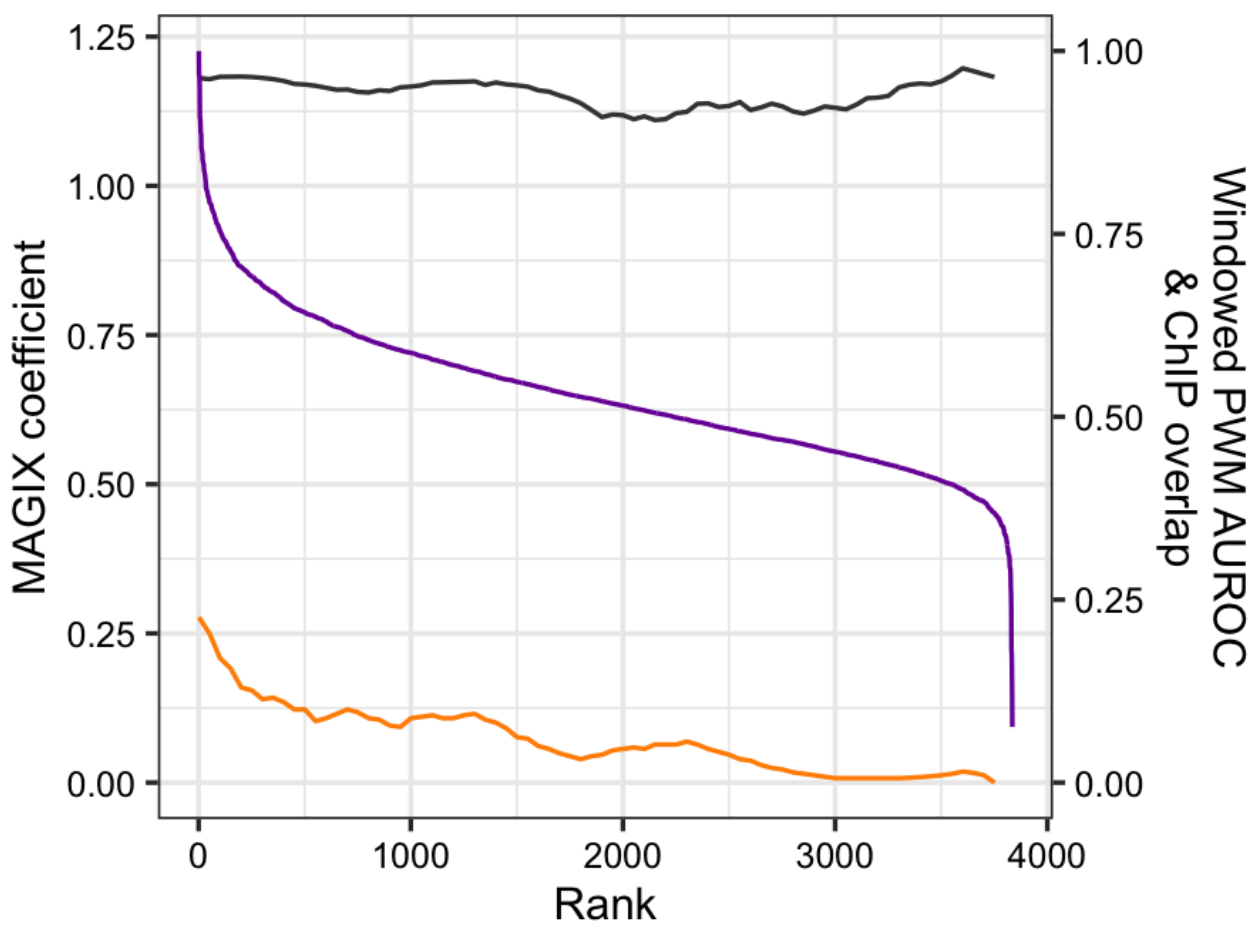

ZNF335.DBD1

Top GRECO-BIT/MEX PWM: PWM003898

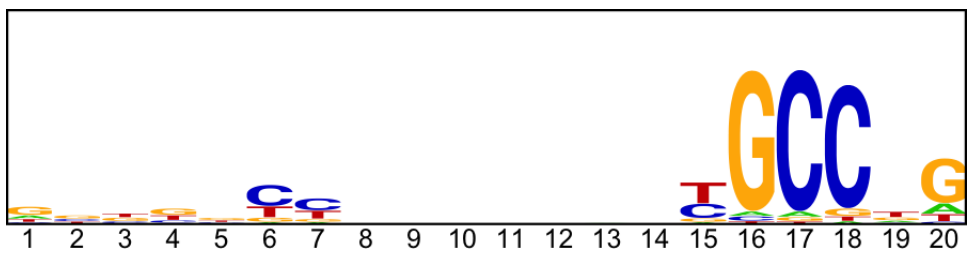

Location of top GRECO-BIT/MEX PWM in MAGIX peaks (up to the top 10,000)

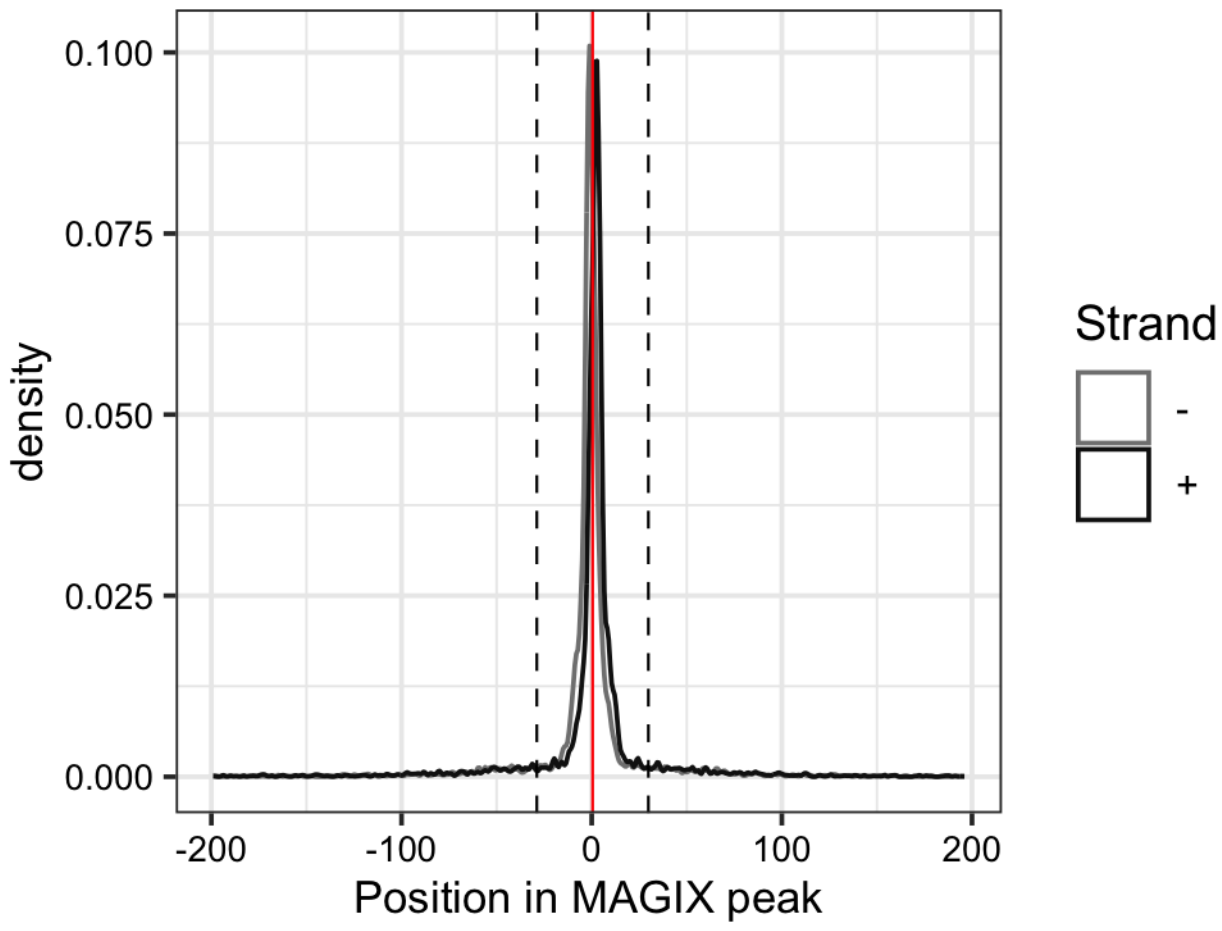

ZNF335.DBD2

Top GRECO-BIT/MEX PWM: PWM003891

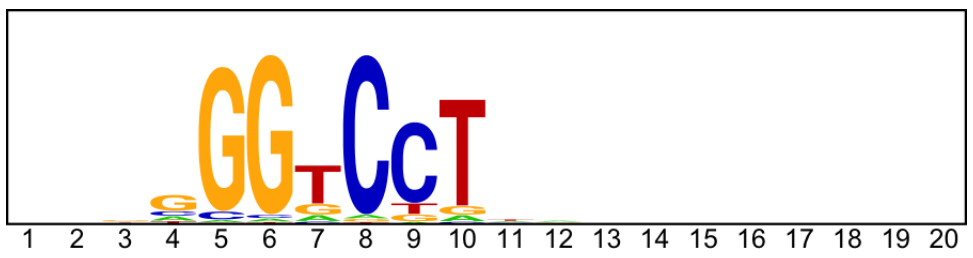

Location of top GRECO-BIT/MEX PWM in MAGIX peaks (up to the top 10,000)

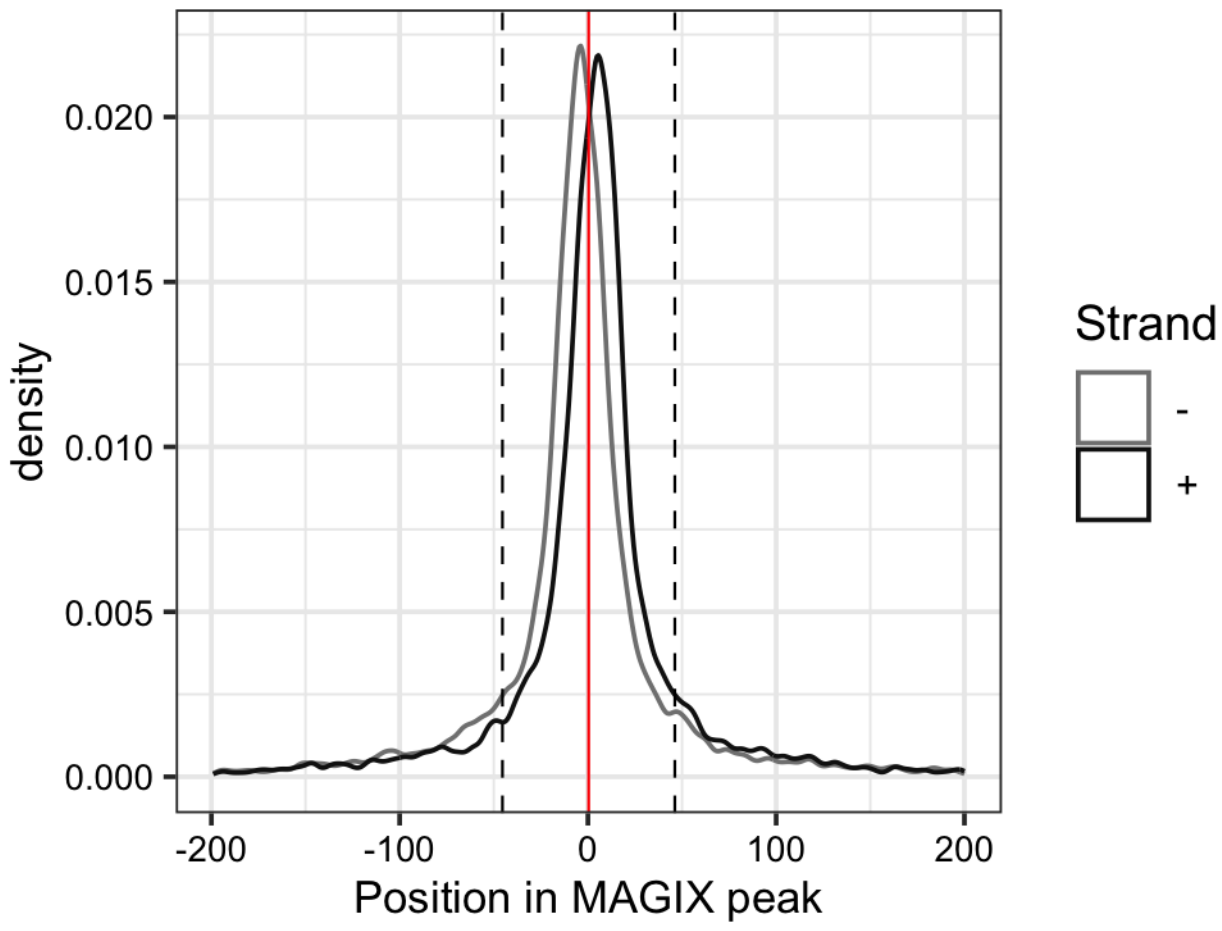

ZNF347

Top GRECO-BIT/MEX PWM: PWM003926

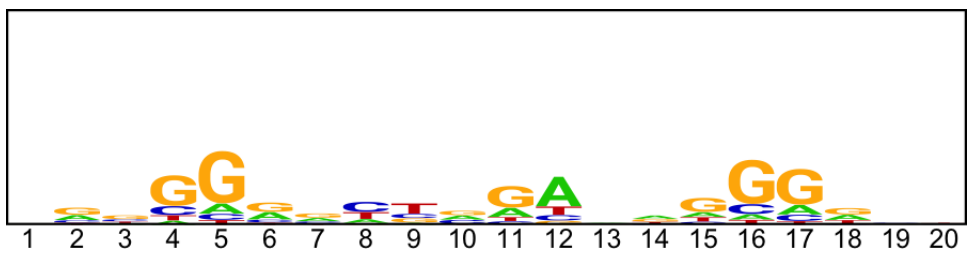

Location of top GRECO-BIT/MEX PWM in MAGIX peaks (up to the top 10,000)

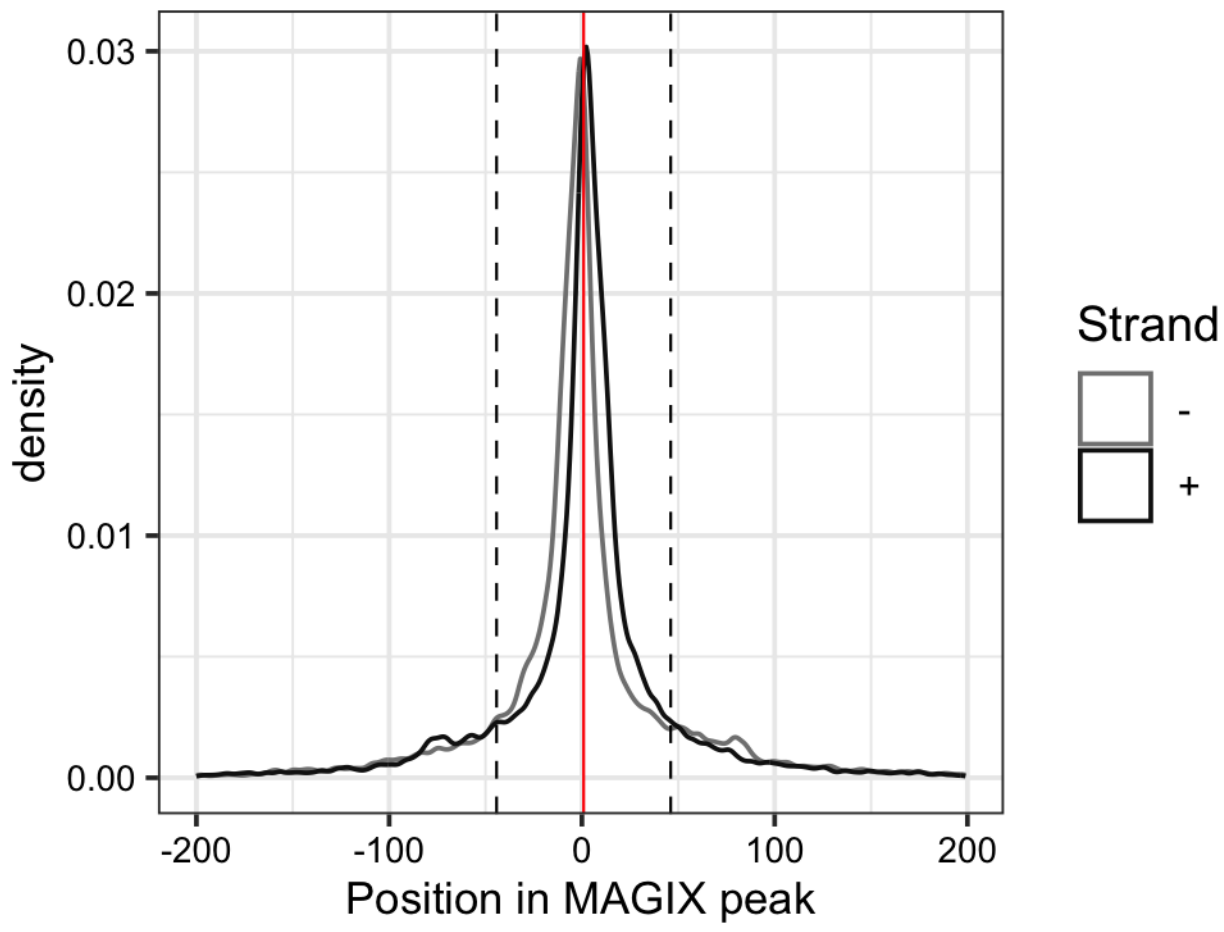

AUROC of top GRECO-BIT/MEX PWM in sliding windows (500 peaks, 50 peak step) along the MAGIX peaks (up to the top 75,000) ranked by the MAGIX enrichment coefficient and fraction of overlapping ChIP peaks in the same window

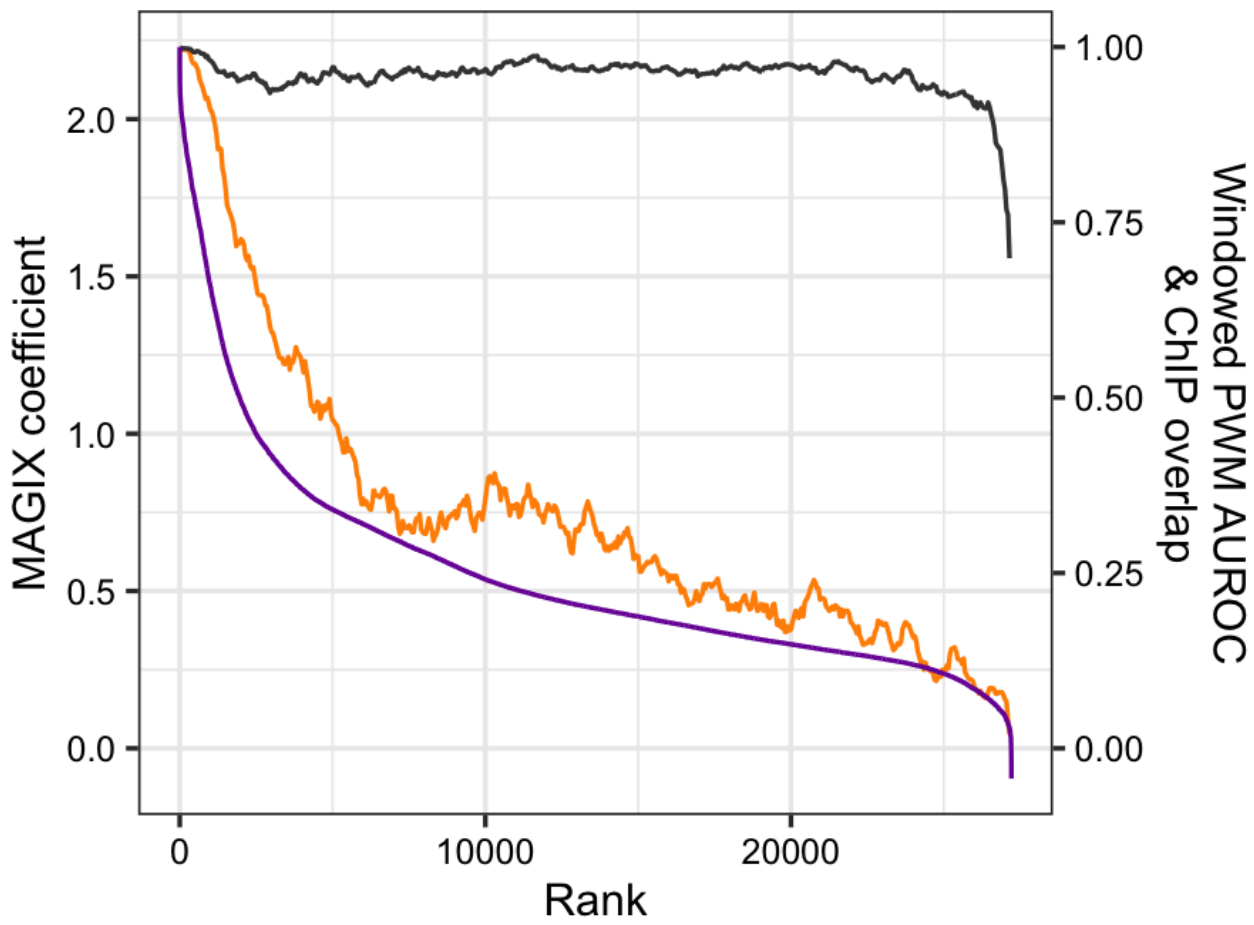

ZNF35

Top GRECO-BIT/MEX PWM: PWM003953

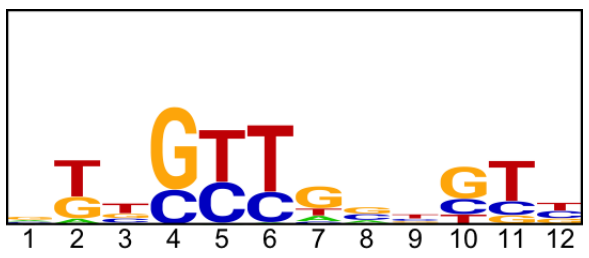

Location of top GRECO-BIT/MEX PWM in MAGIX peaks (up to the top 10,000)

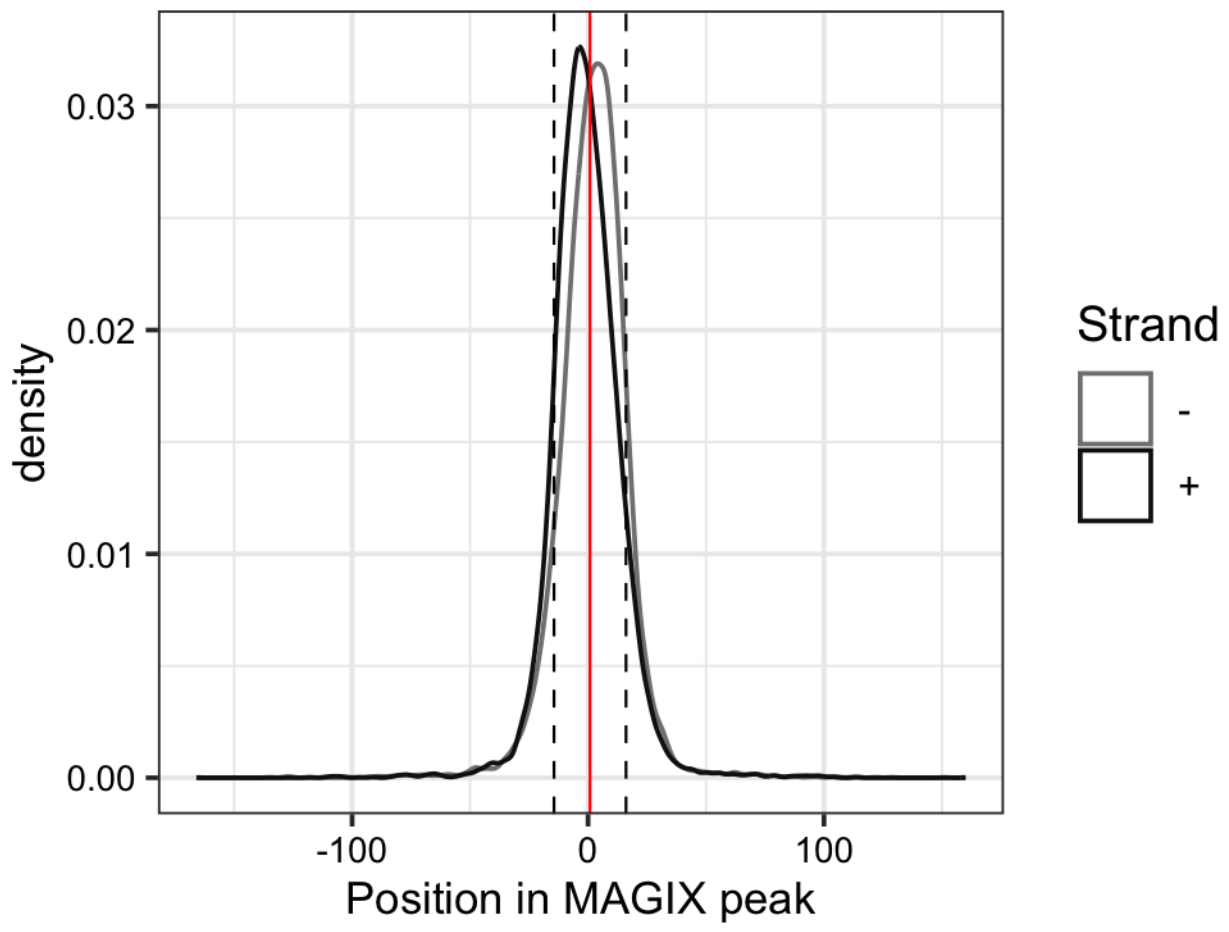

AUROC of top GRECO-BIT/MEX PWM in sliding windows (500 peaks, 50 peak step) along the MAGIX peaks (up to the top 75,000) ranked by the MAGIX enrichment coefficient and fraction of overlapping ChIP peaks in the same window

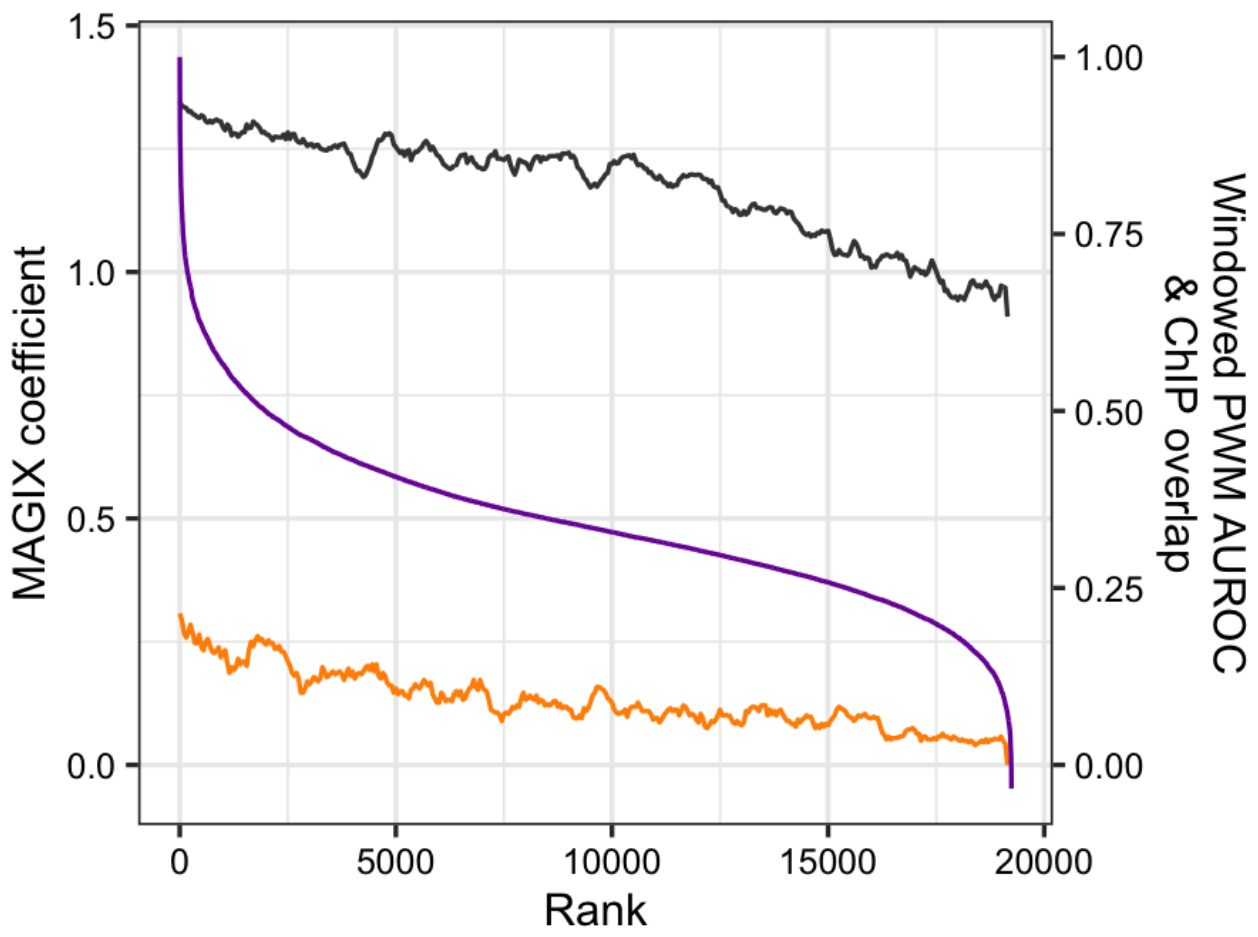

ZNF358

Top GRECO-BIT/MEX PWM: PWM003990

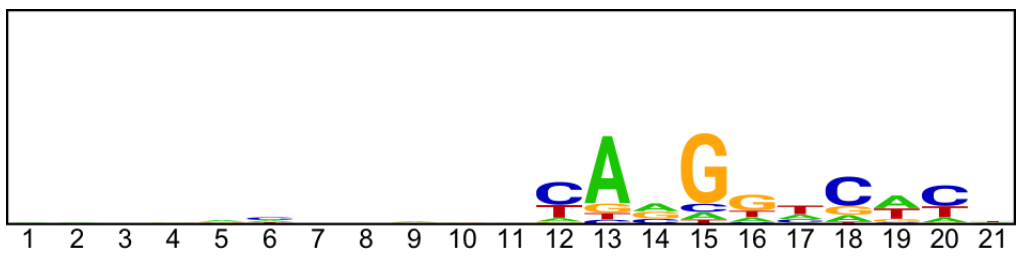

Location of top GRECO-BIT/MEX PWM in MAGIX peaks (up to the top 10,000)

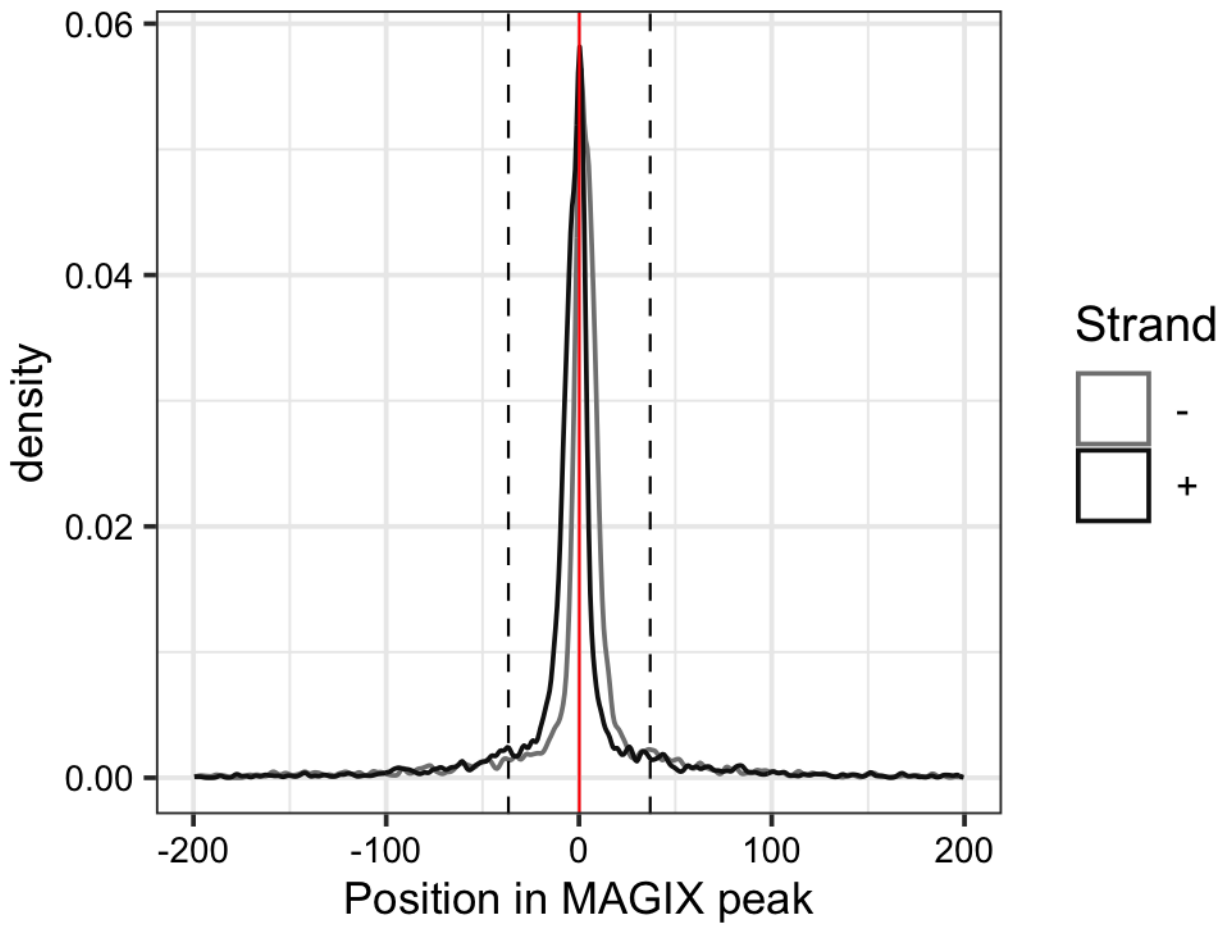

ZNF362

Top GRECO-BIT/MEX PWM: PWM004037

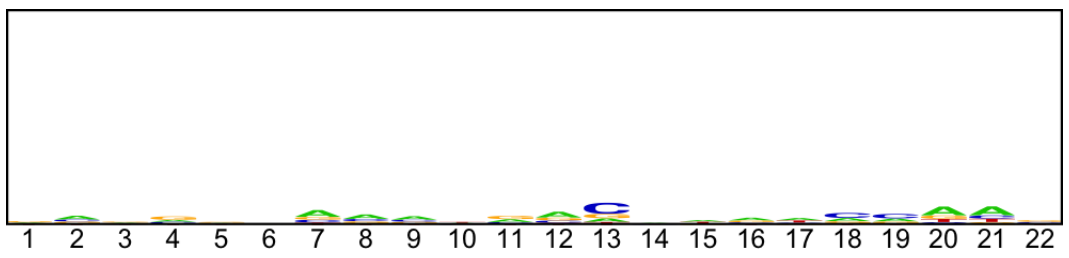

Location of top GRECO-BIT/MEX PWM in MAGIX peaks (up to the top 10,000)

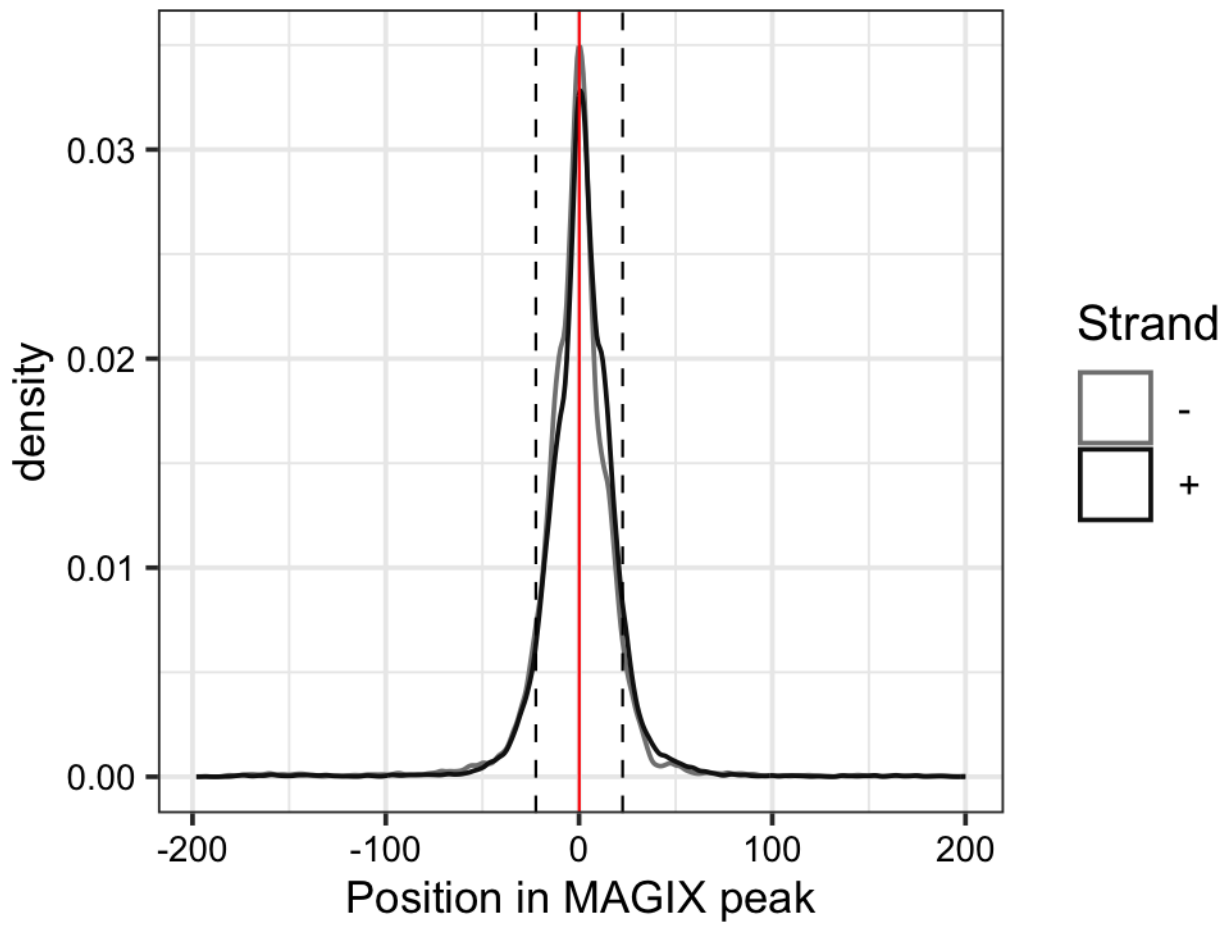

AUROC of top GRECO-BIT/MEX PWM in sliding windows (500 peaks, 50 peak step) along the MAGIX peaks (up to the top 75,000) ranked by the MAGIX enrichment coefficient and fraction of overlapping ChIP peaks in the same window

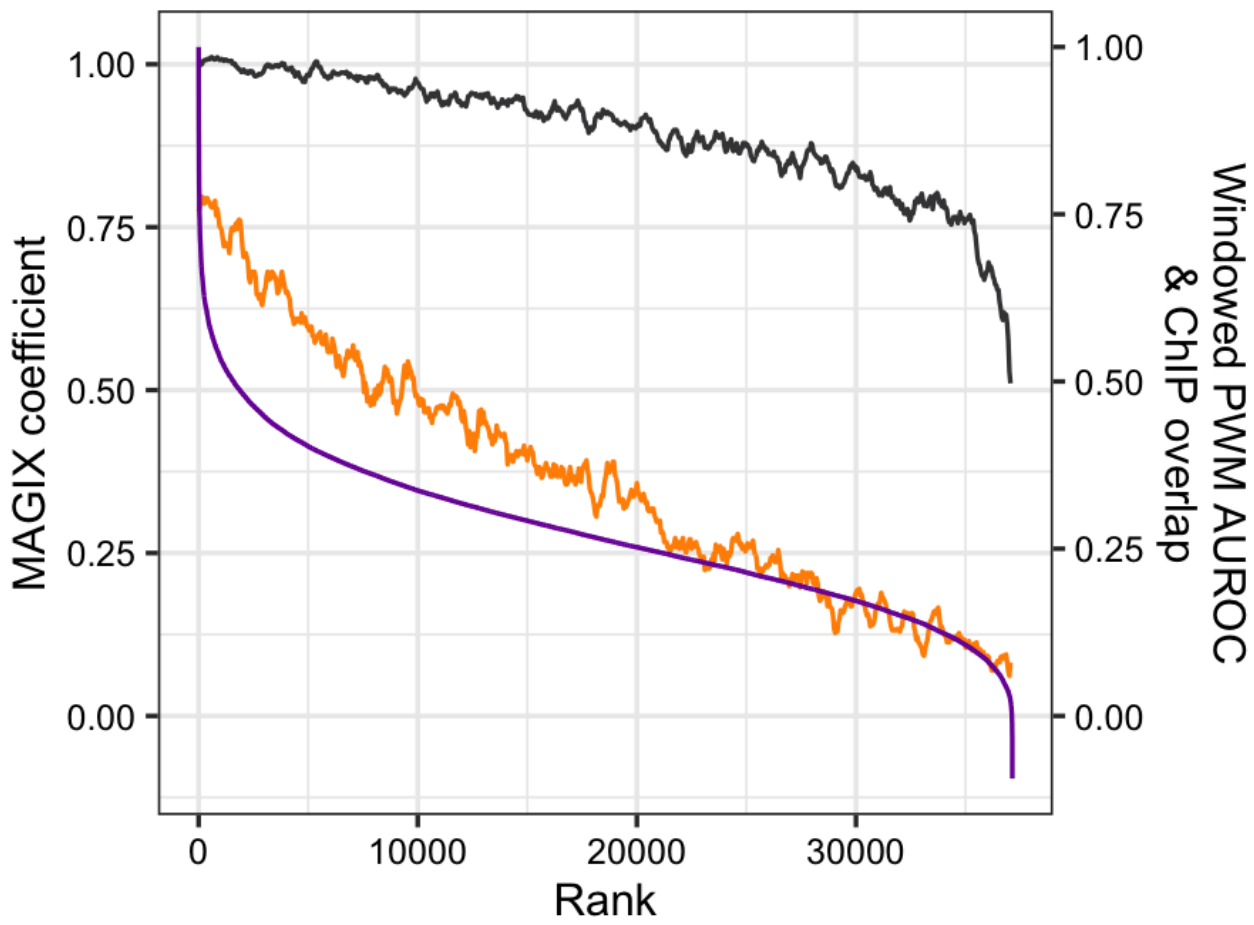

ZNF367

Top GRECO-BIT/MEX PWM: PWM004062

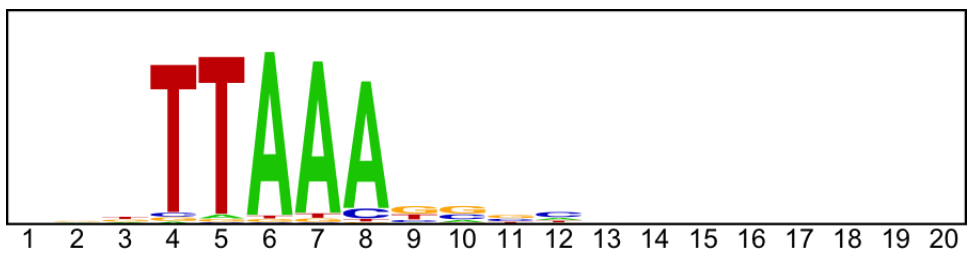

Location of top GRECO-BIT/MEX PWM in MAGIX peaks (up to the top 10,000)

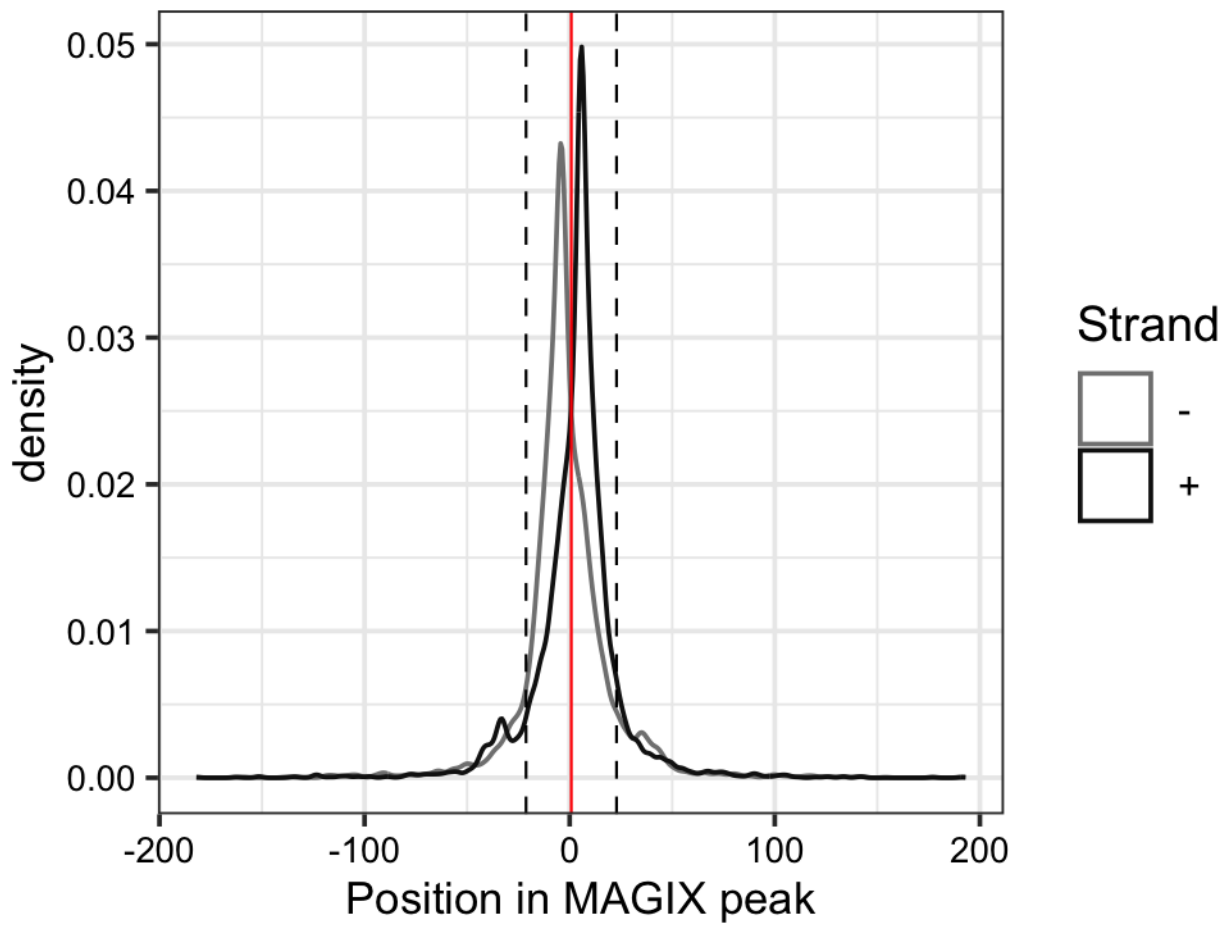

AUROC of top GRECO-BIT/MEX PWM in sliding windows (500 peaks, 50 peak step) along the MAGIX peaks (up to the top 75,000) ranked by the MAGIX enrichment coefficient and fraction of overlapping ChIP peaks in the same window

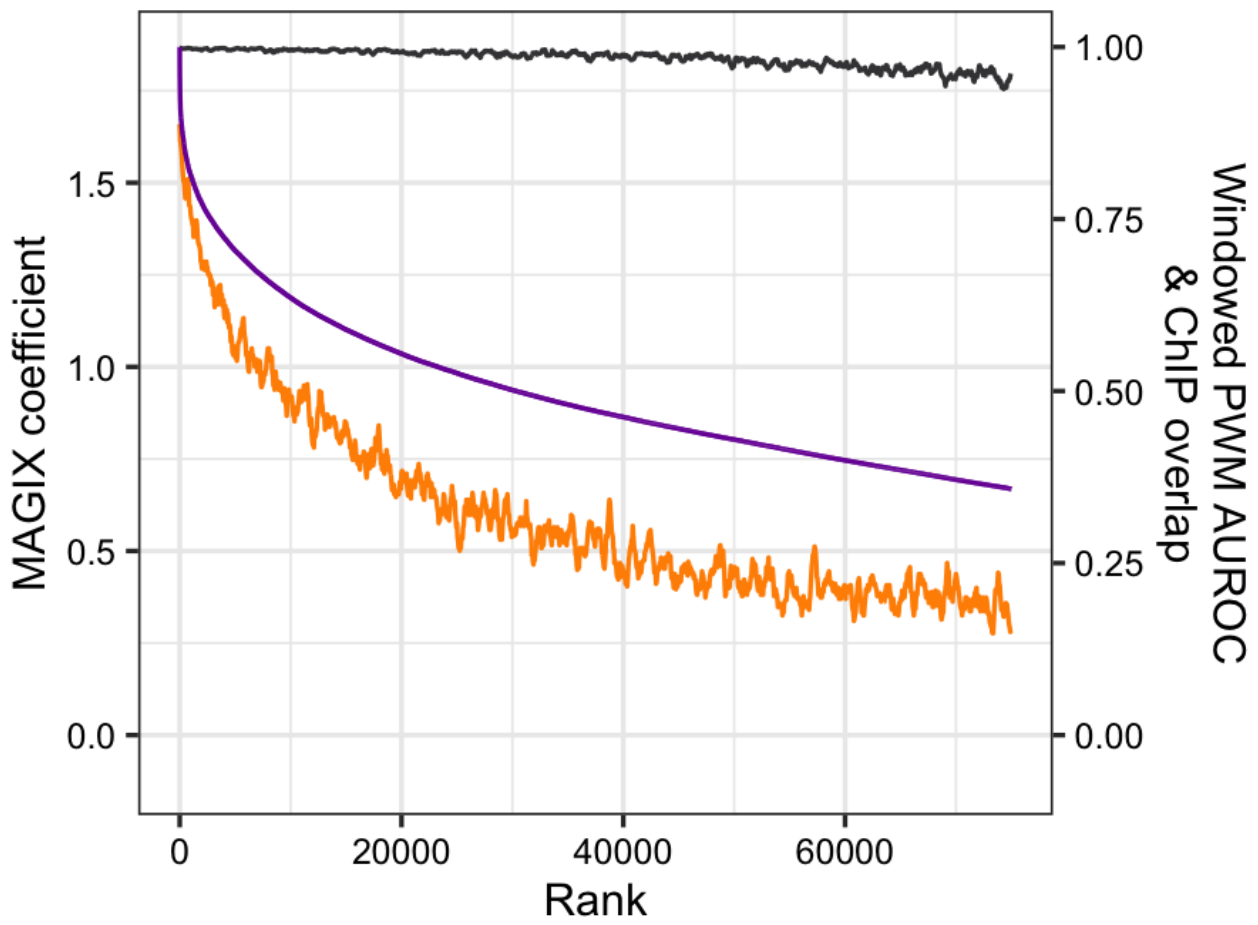

ZNF384

Top GRECO-BIT/MEX PWM: PWM004120

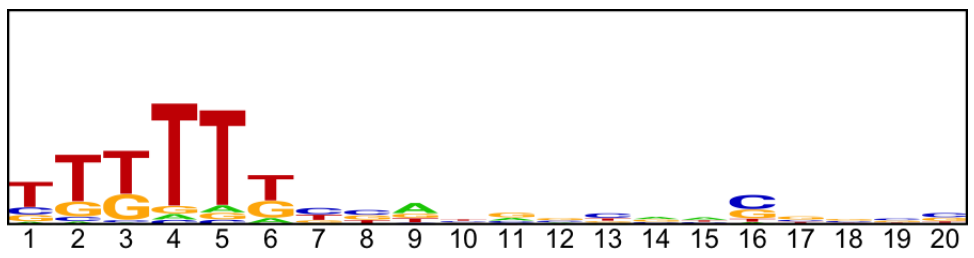

Location of top GRECO-BIT/MEX PWM in MAGIX peaks (up to the top 10,000)

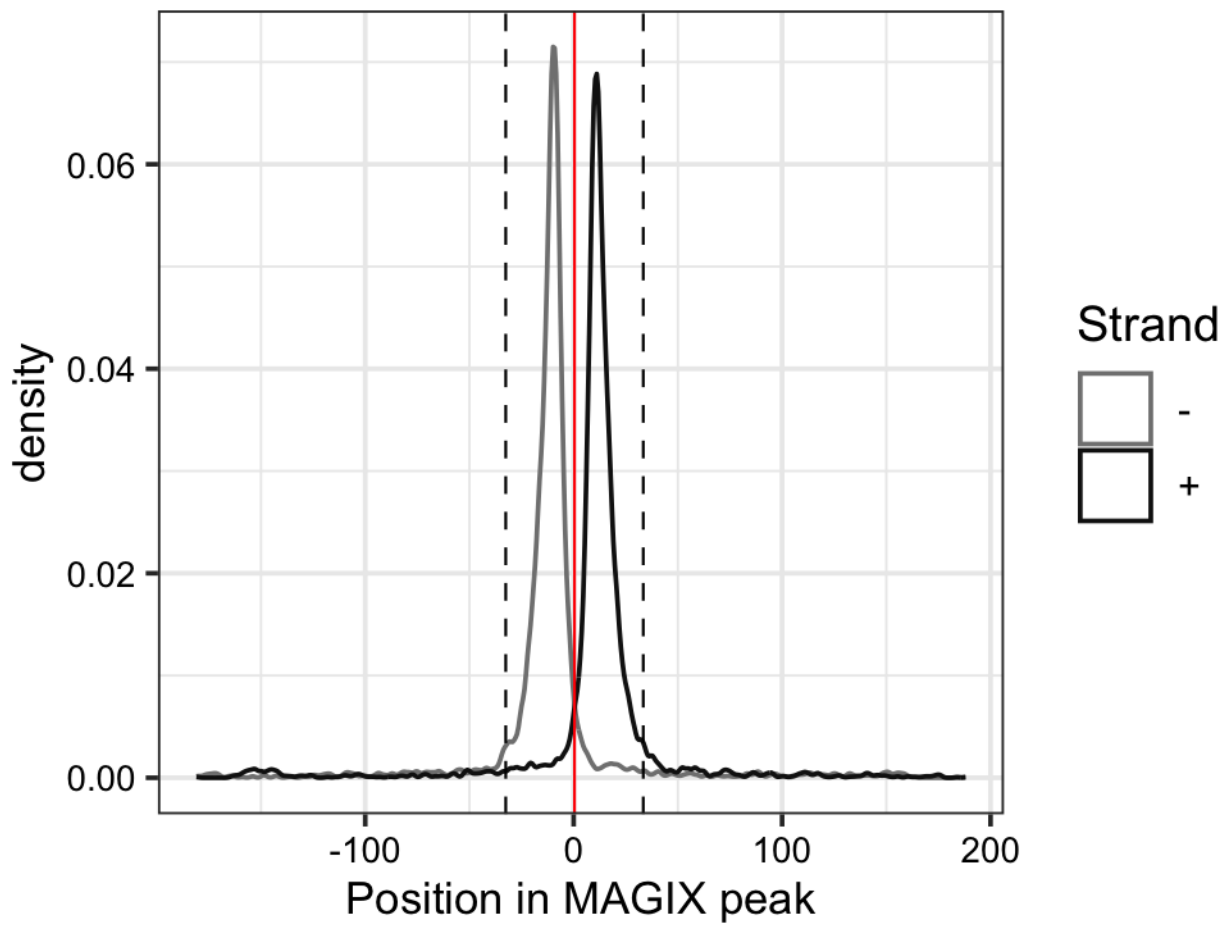

AUROC of top GRECO-BIT/MEX PWM in sliding windows (500 peaks, 50 peak step) along the MAGIX peaks (up to the top 75,000) ranked by the MAGIX enrichment coefficient and fraction of overlapping ChIP peaks in the same window

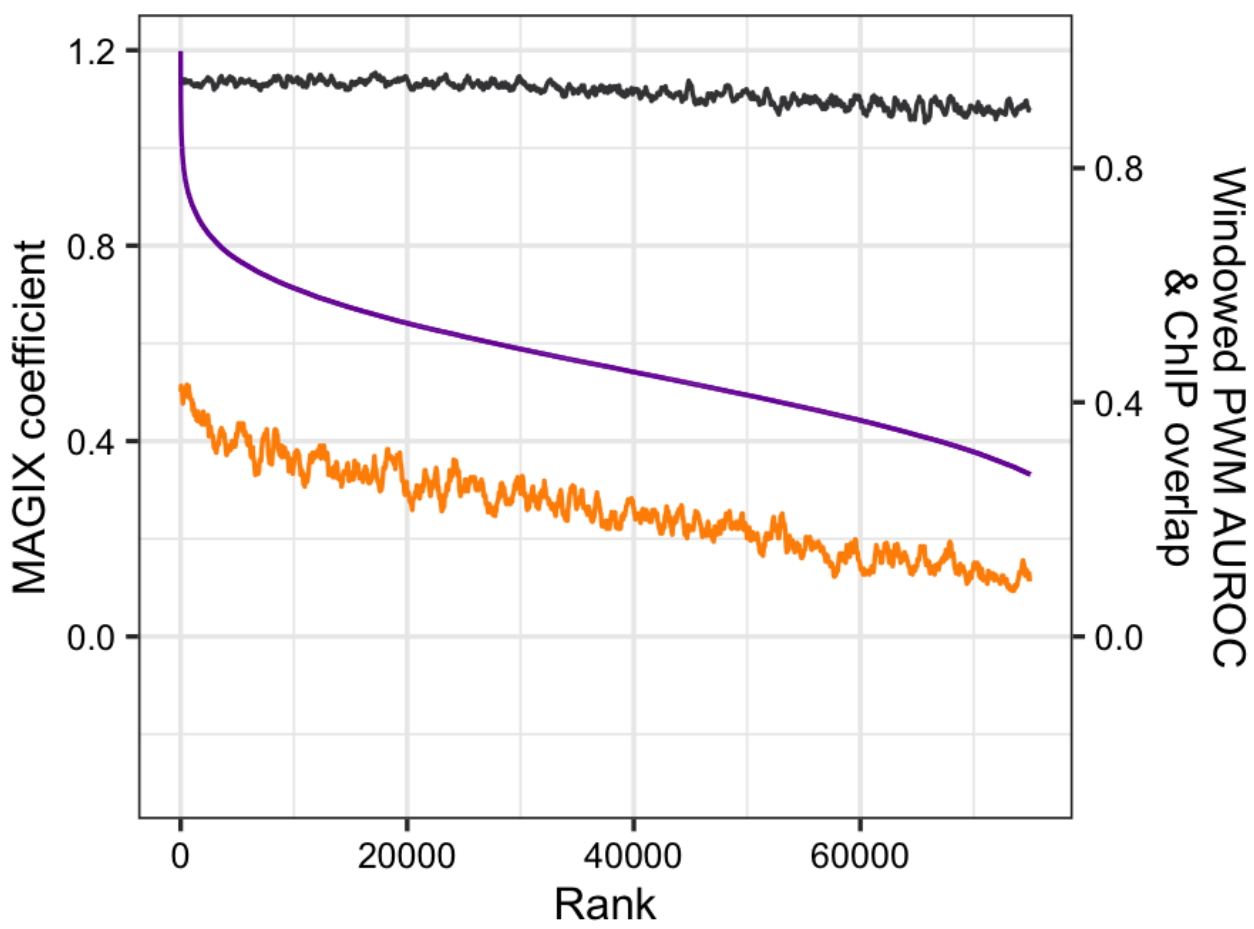

ZNF395

Top GRECO-BIT/MEX PWM: PWM004151

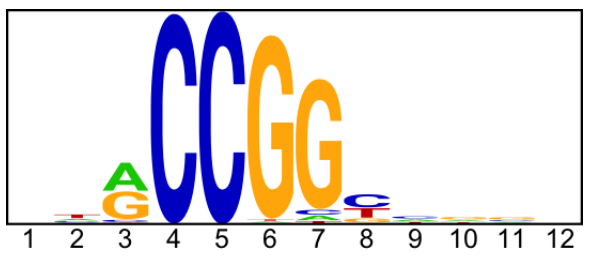

Location of top GRECO-BIT/MEX PWM in MAGIX peaks (up to the top 10,000)

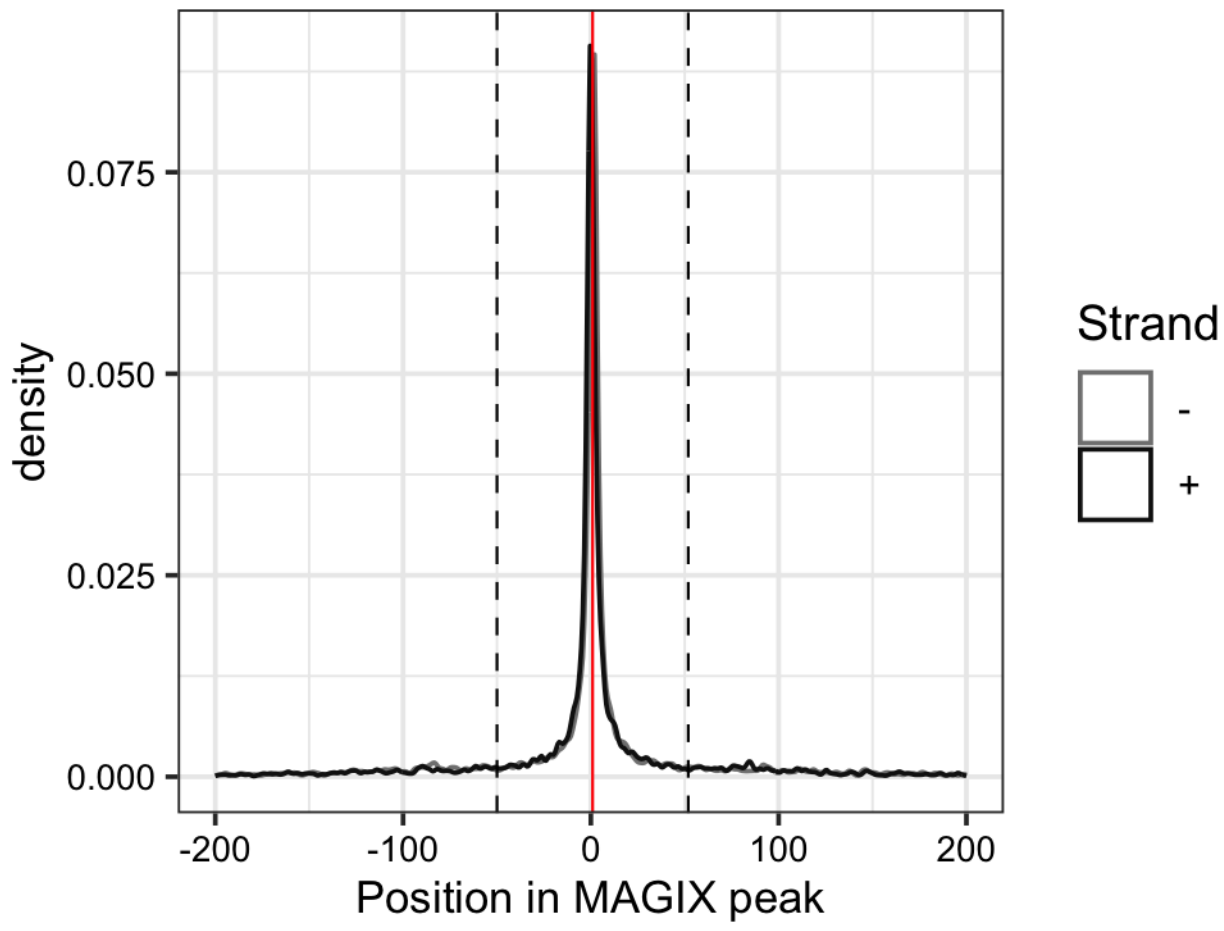

AUROC of top GRECO-BIT/MEX PWM in sliding windows (500 peaks, 50 peak step) along the MAGIX peaks (up to the top 75,000) ranked by the MAGIX enrichment coefficient and fraction of overlapping ChIP peaks in the same window

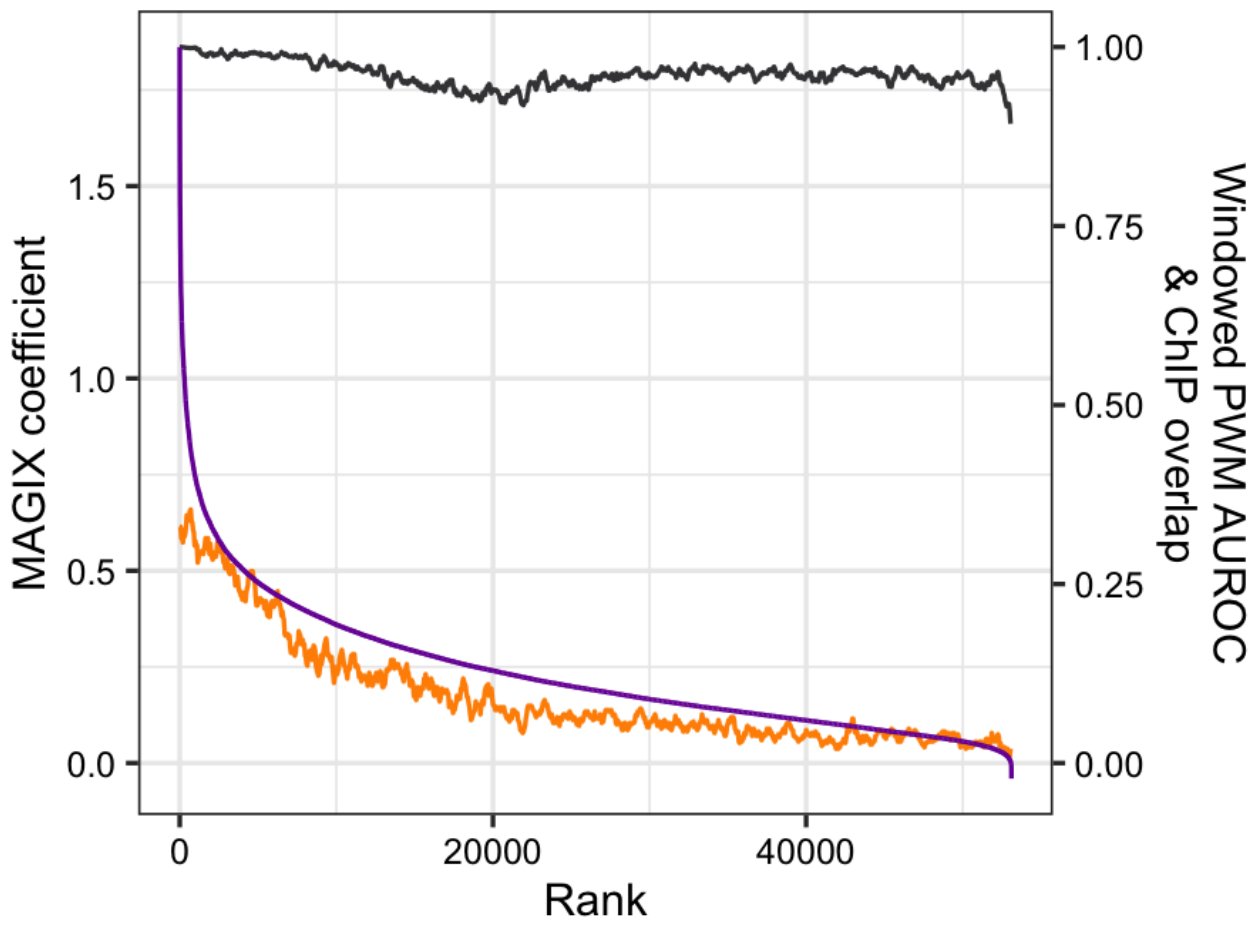

ZNF407

Top GRECO-BIT/MEX PWM: PWM004189

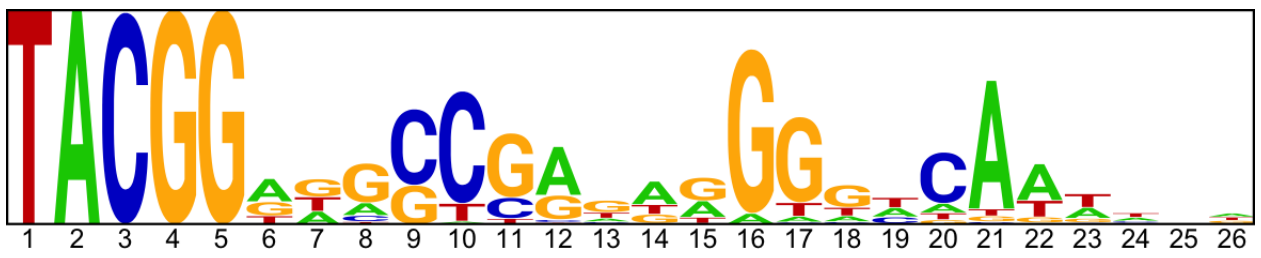

Location of top GRECO-BIT/MEX PWM in MAGIX peaks (up to the top 10,000)

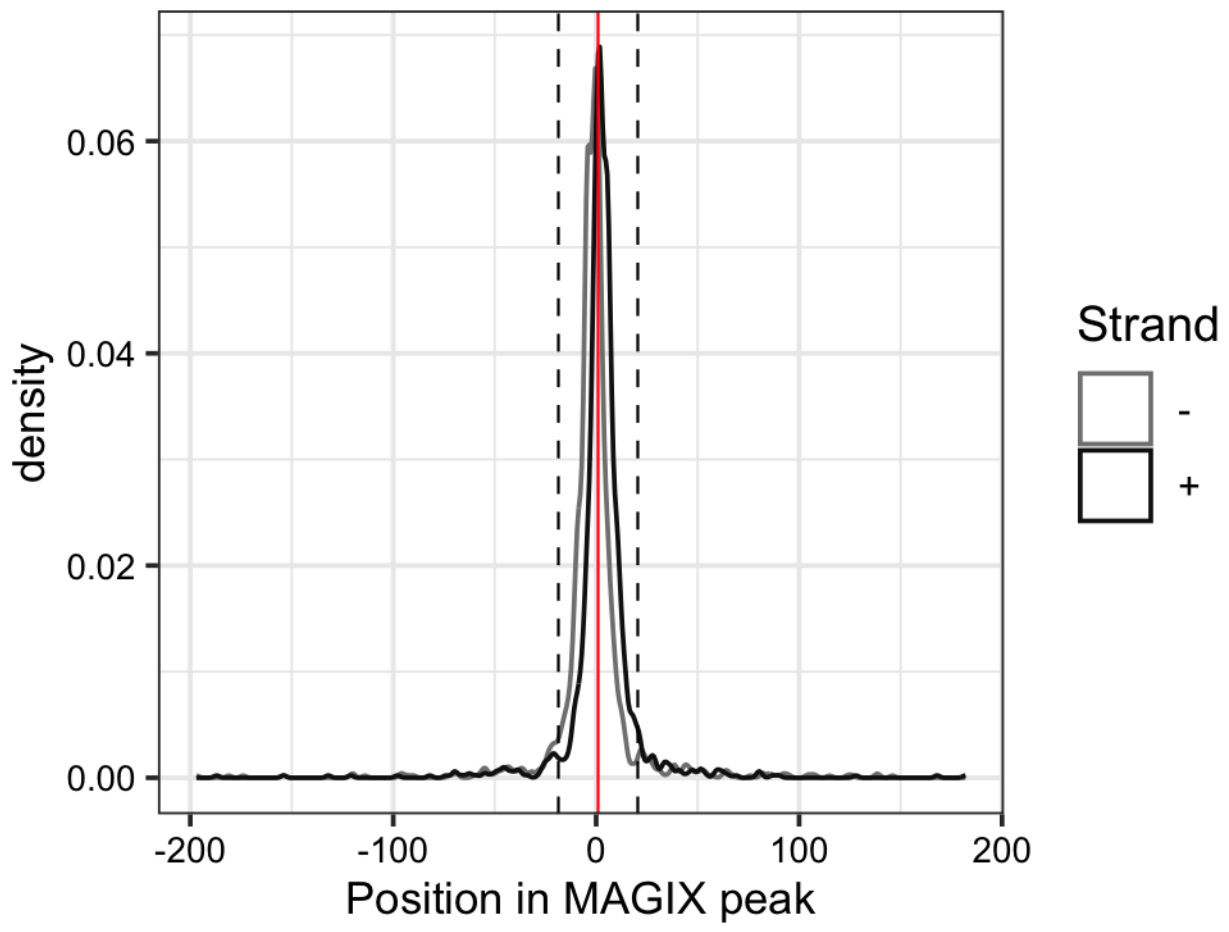

AUROC of top GRECO-BIT/MEX PWM in sliding windows (500 peaks, 50 peak step) along the MAGIX peaks (up to the top 75,000) ranked by the MAGIX enrichment coefficient and fraction of overlapping ChIP peaks in the same window

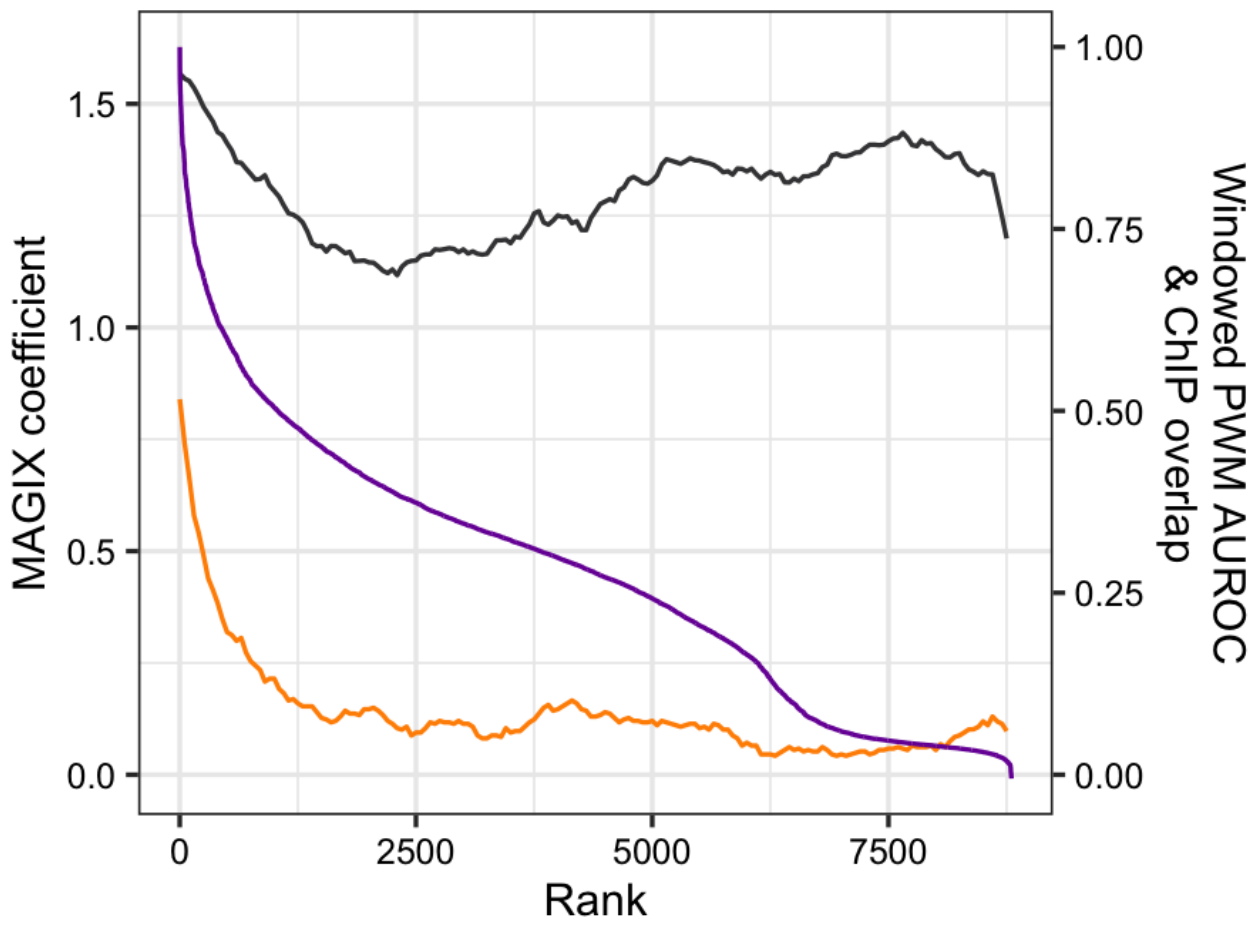

ZNF43.DBD1

Top GRECO-BIT/MEX PWM: PWM004198

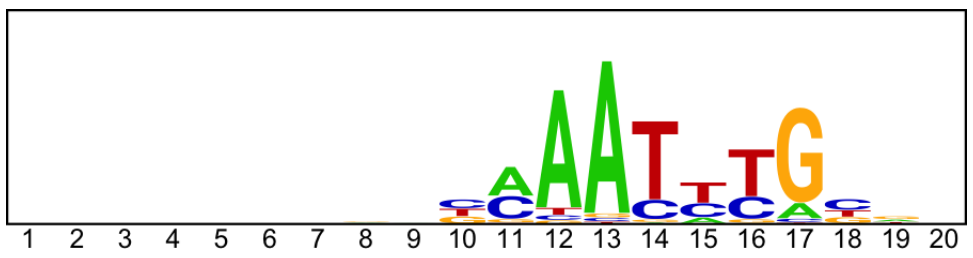

Location of top GRECO-BIT/MEX PWM in MAGIX peaks (up to the top 10,000)

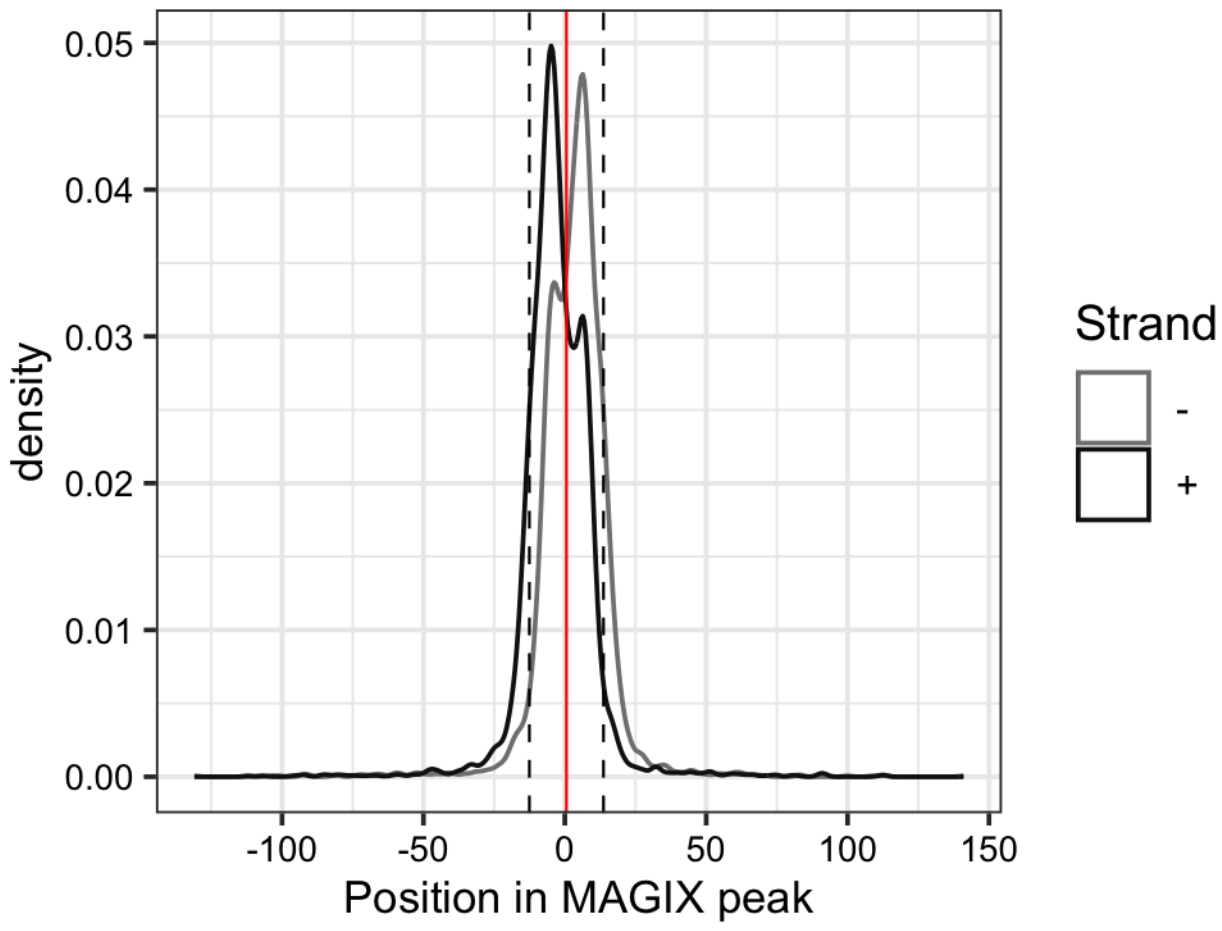

ZNF43.DBD2

Top GRECO-BIT/MEX PWM: PWM004215

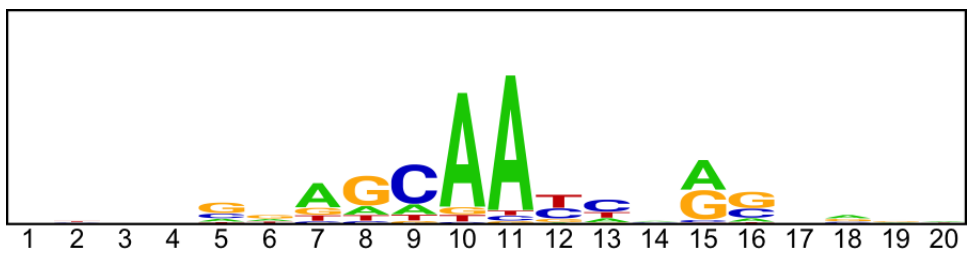

Location of top GRECO-BIT/MEX PWM in MAGIX peaks (up to the top 10,000)

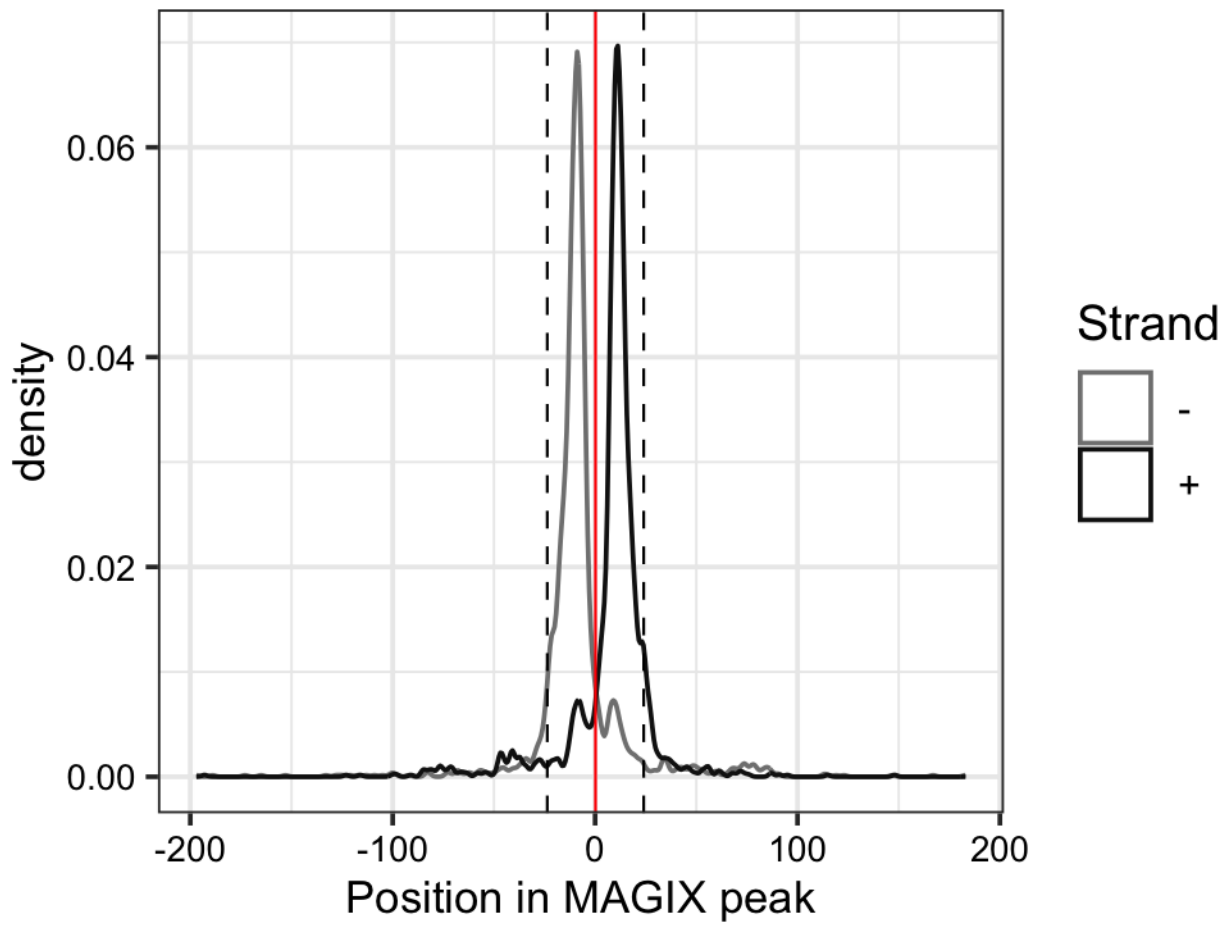

ZNF43.FL

Top GRECO-BIT/MEX PWM: PWM004214

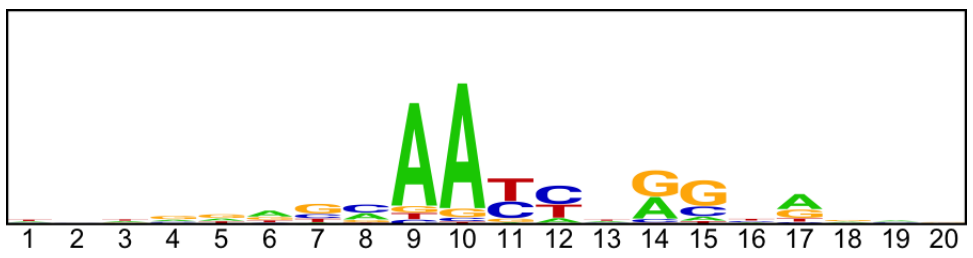

Location of top GRECO-BIT/MEX PWM in MAGIX peaks (up to the top 10,000)

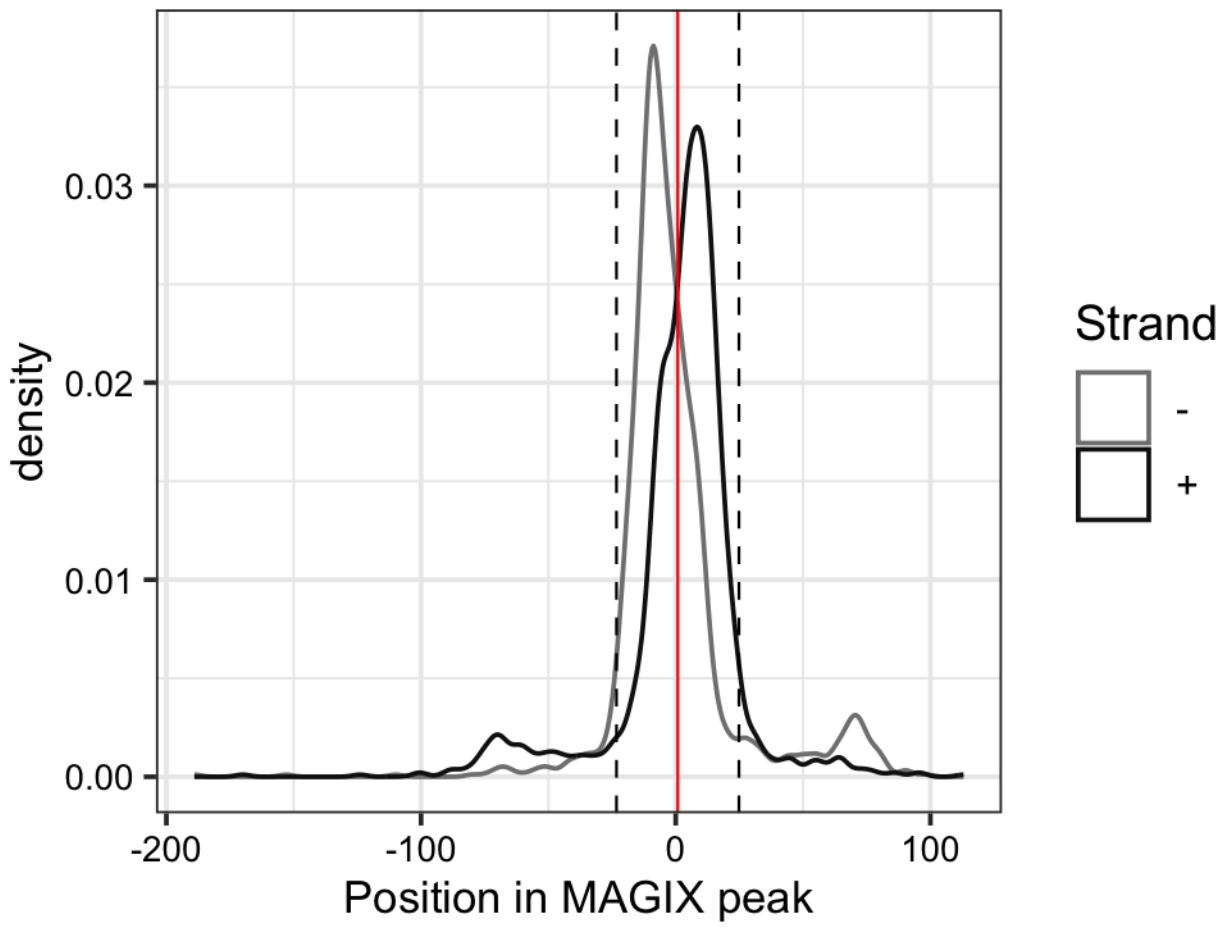

AUROC of top GRECO-BIT/MEX PWM in sliding windows (500 peaks, 50 peak step) along the MAGIX peaks (up to the top 75,000) ranked by the MAGIX enrichment coefficient and fraction of overlapping ChIP peaks in the same window

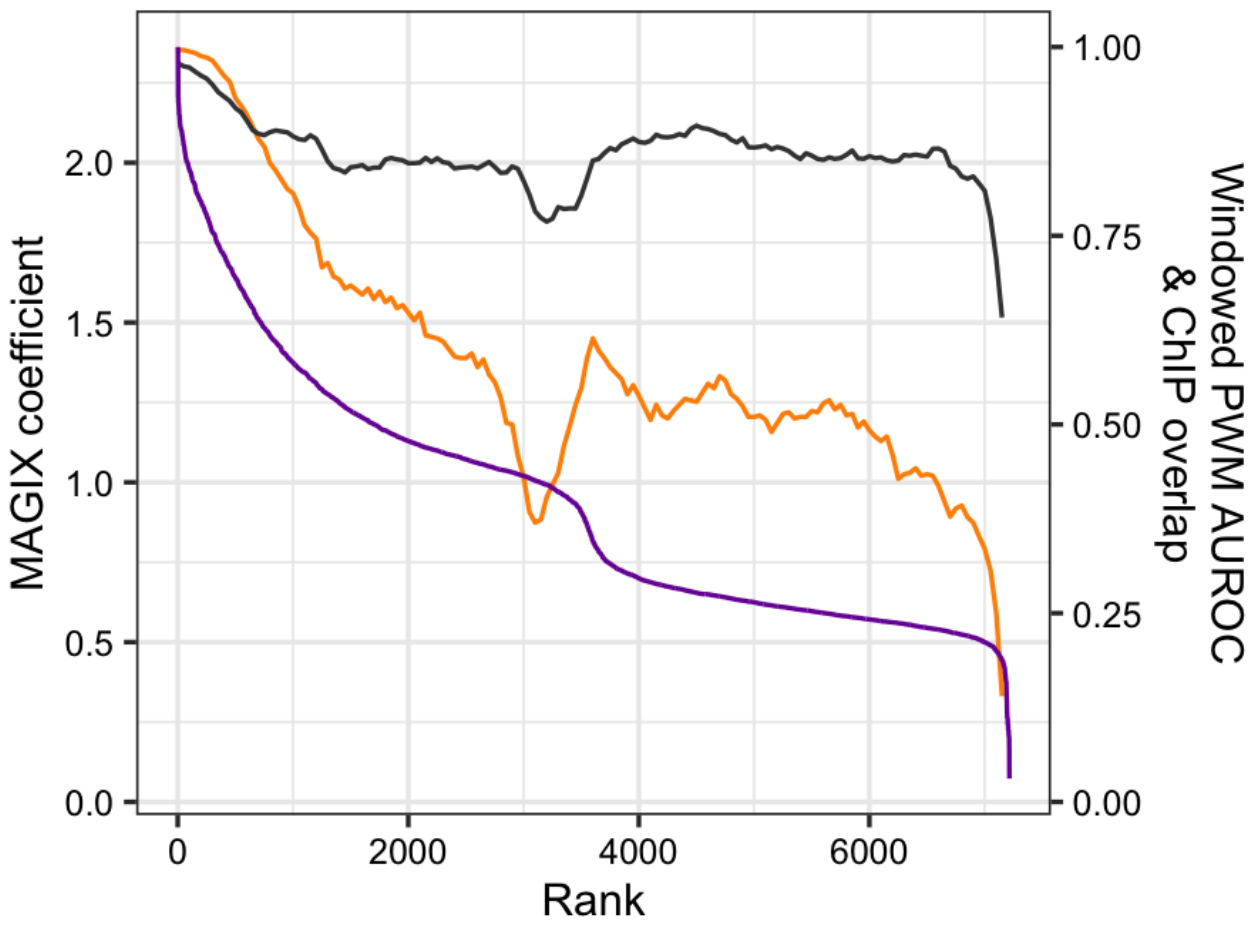

ZNF436

Top GRECO-BIT/MEX PWM: PWM004243

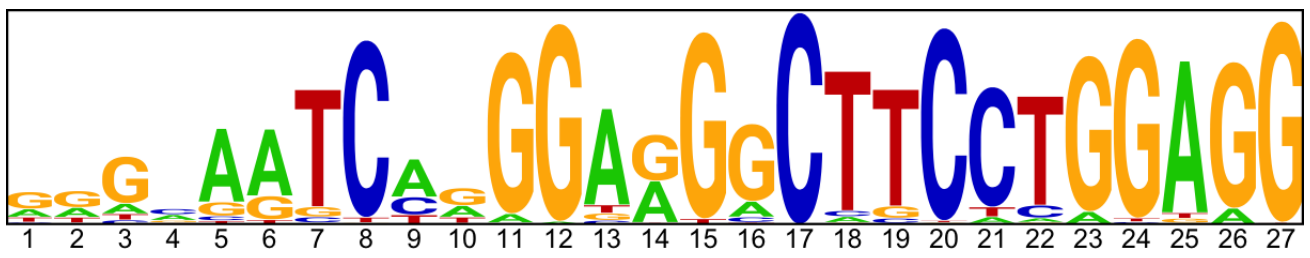

Location of top GRECO-BIT/MEX PWM in MAGIX peaks (up to the top 10,000)

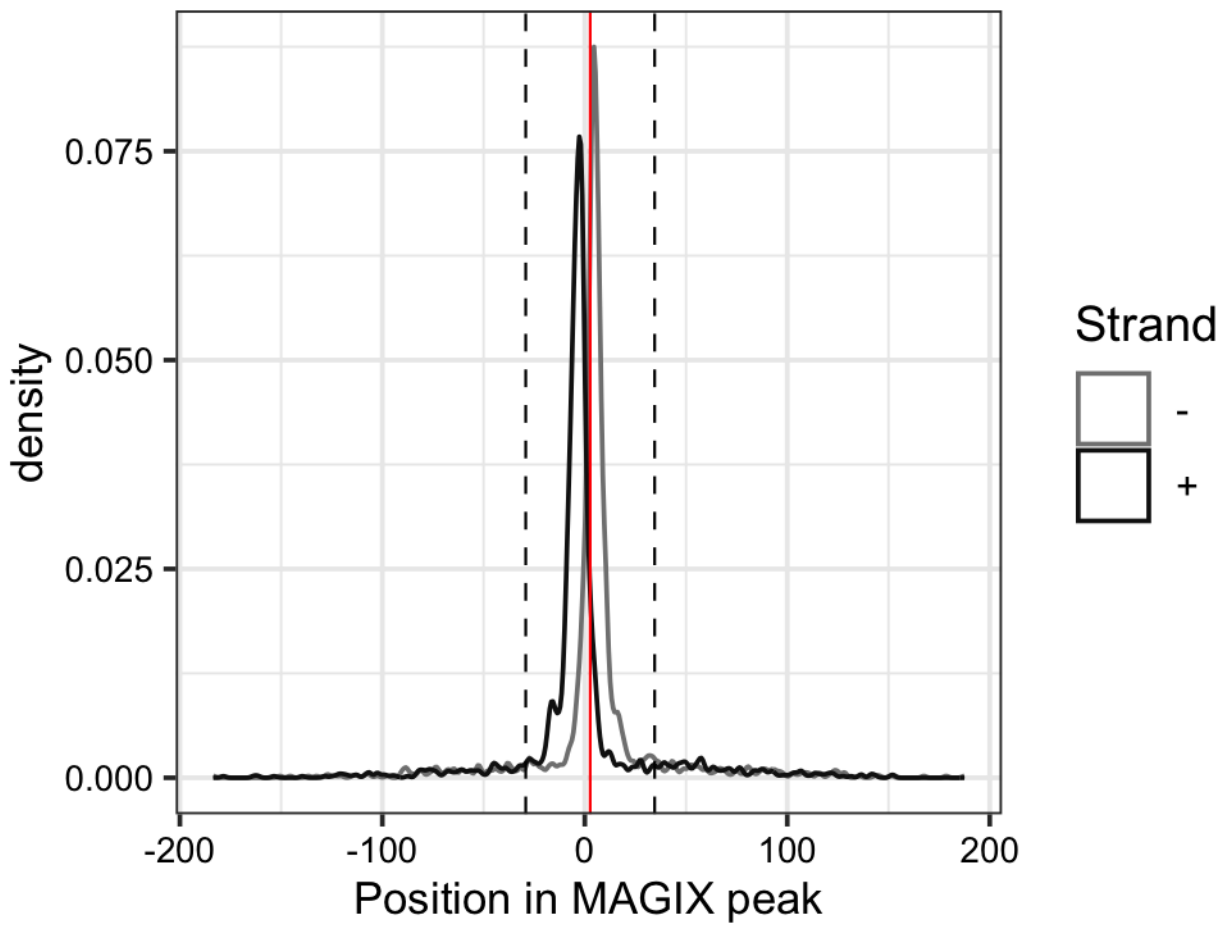

AUROC of top GRECO-BIT/MEX PWM in sliding windows (500 peaks, 50 peak step) along the MAGIX peaks (up to the top 75,000) ranked by the MAGIX enrichment coefficient and fraction of overlapping ChIP peaks in the same window

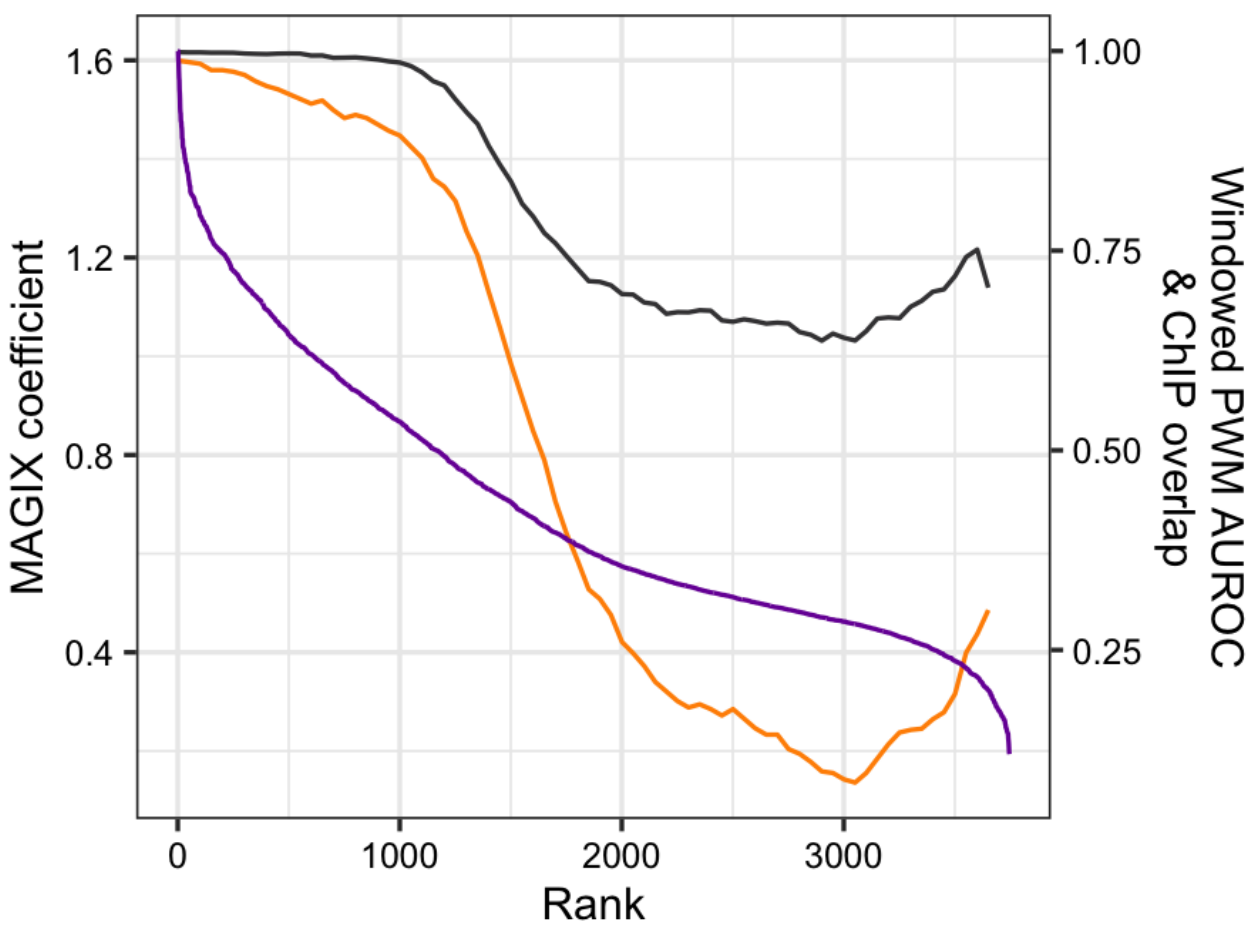

ZNF470

Top GRECO-BIT/MEX PWM: PWM004282

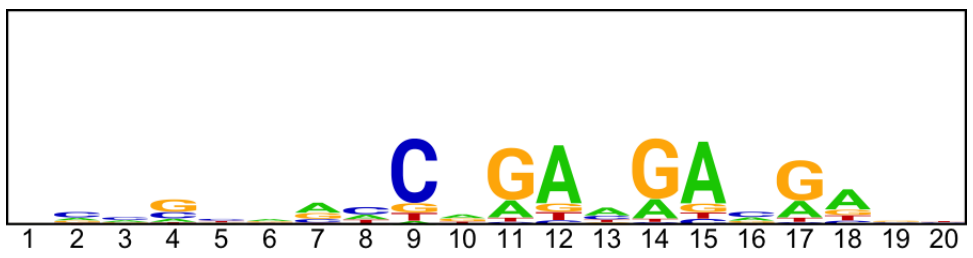

Location of top GRECO-BIT/MEX PWM in MAGIX peaks (up to the top 10,000)

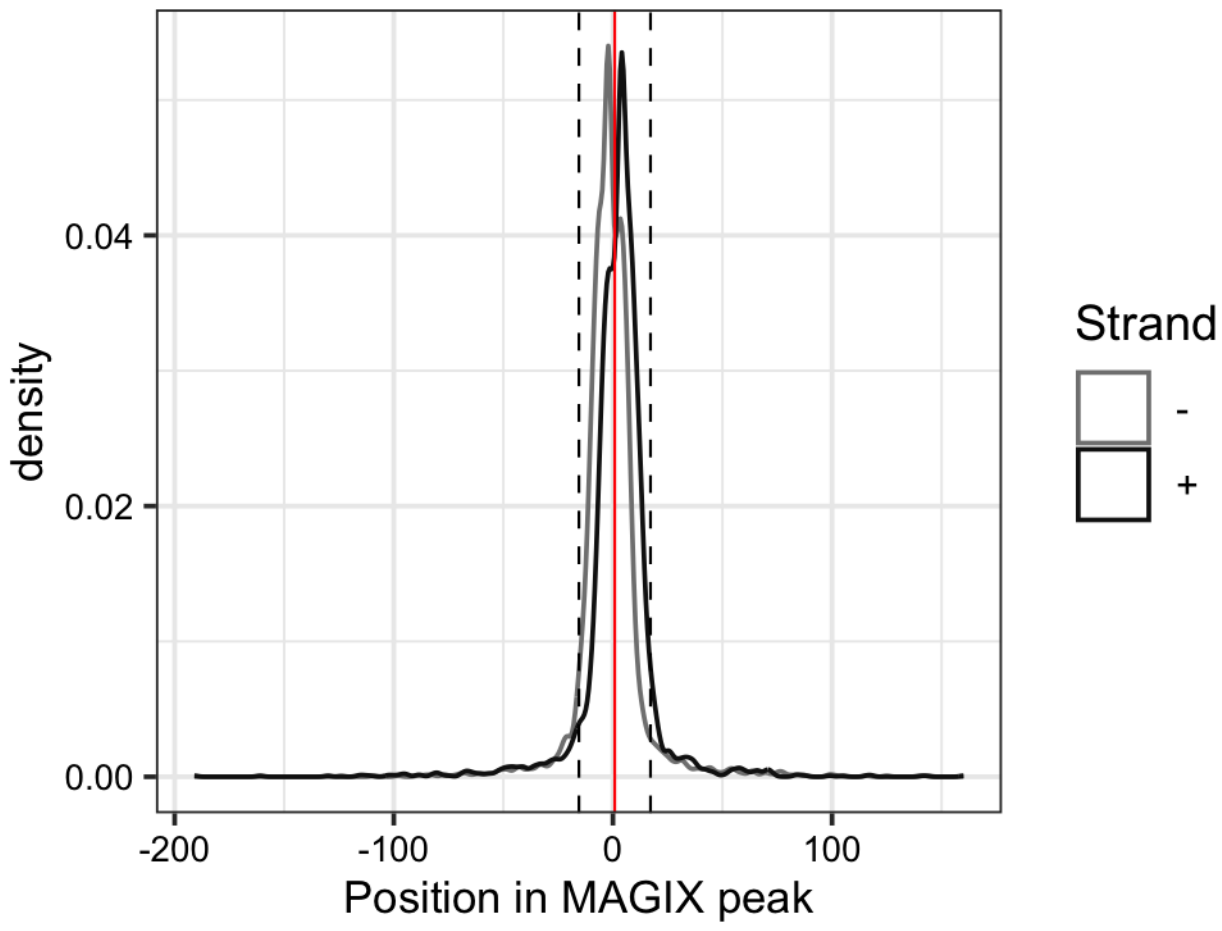

AUROC of top GRECO-BIT/MEX PWM in sliding windows (500 peaks, 50 peak step) along the MAGIX peaks (up to the top 75,000) ranked by the MAGIX enrichment coefficient and fraction of overlapping ChIP peaks in the same window

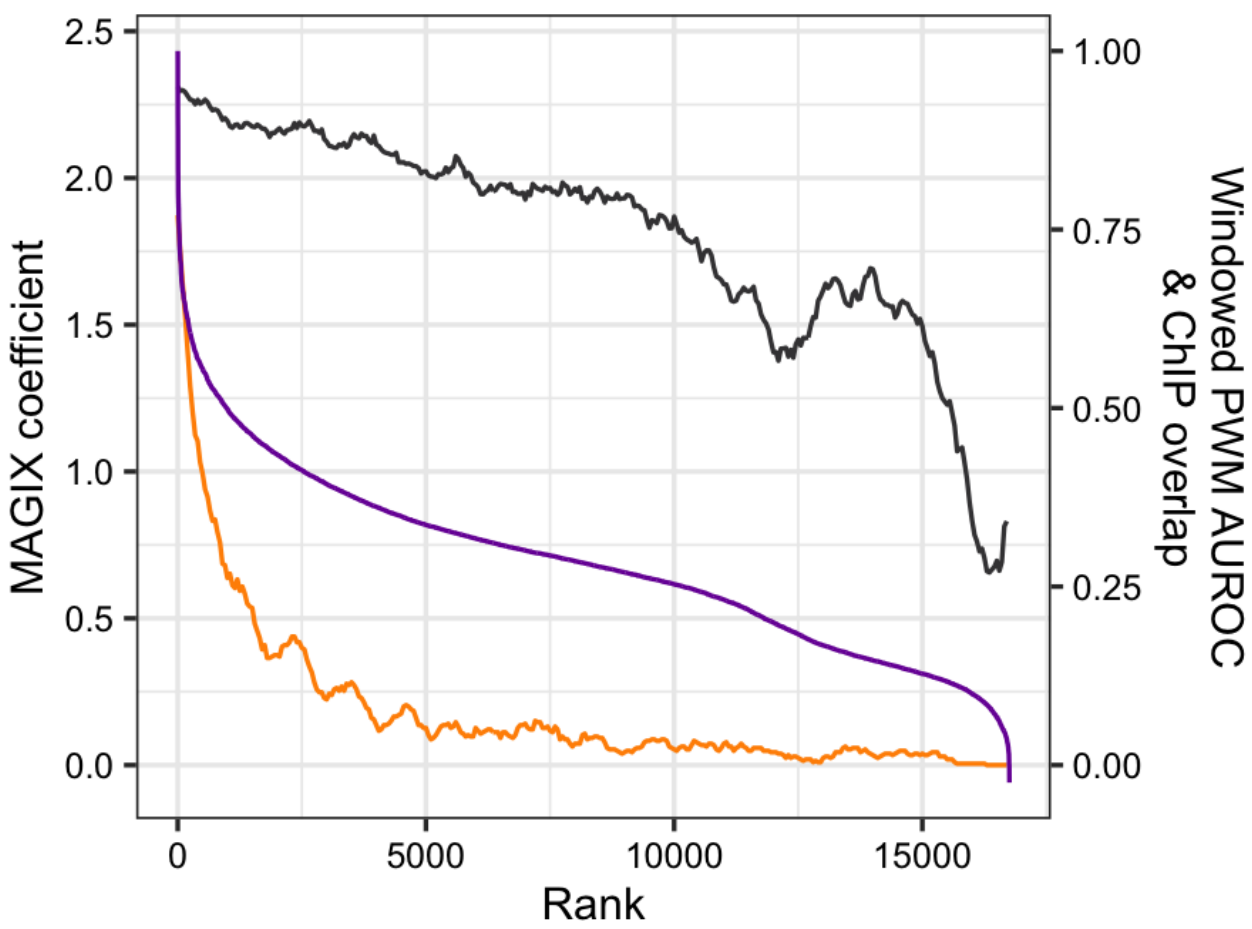

ZNF471

Top GRECO-BIT/MEX PWM: PWM004337

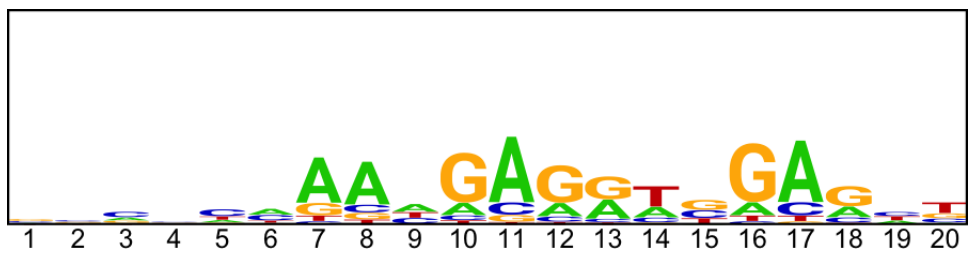

Location of top GRECO-BIT/MEX PWM in MAGIX peaks (up to the top 10,000)

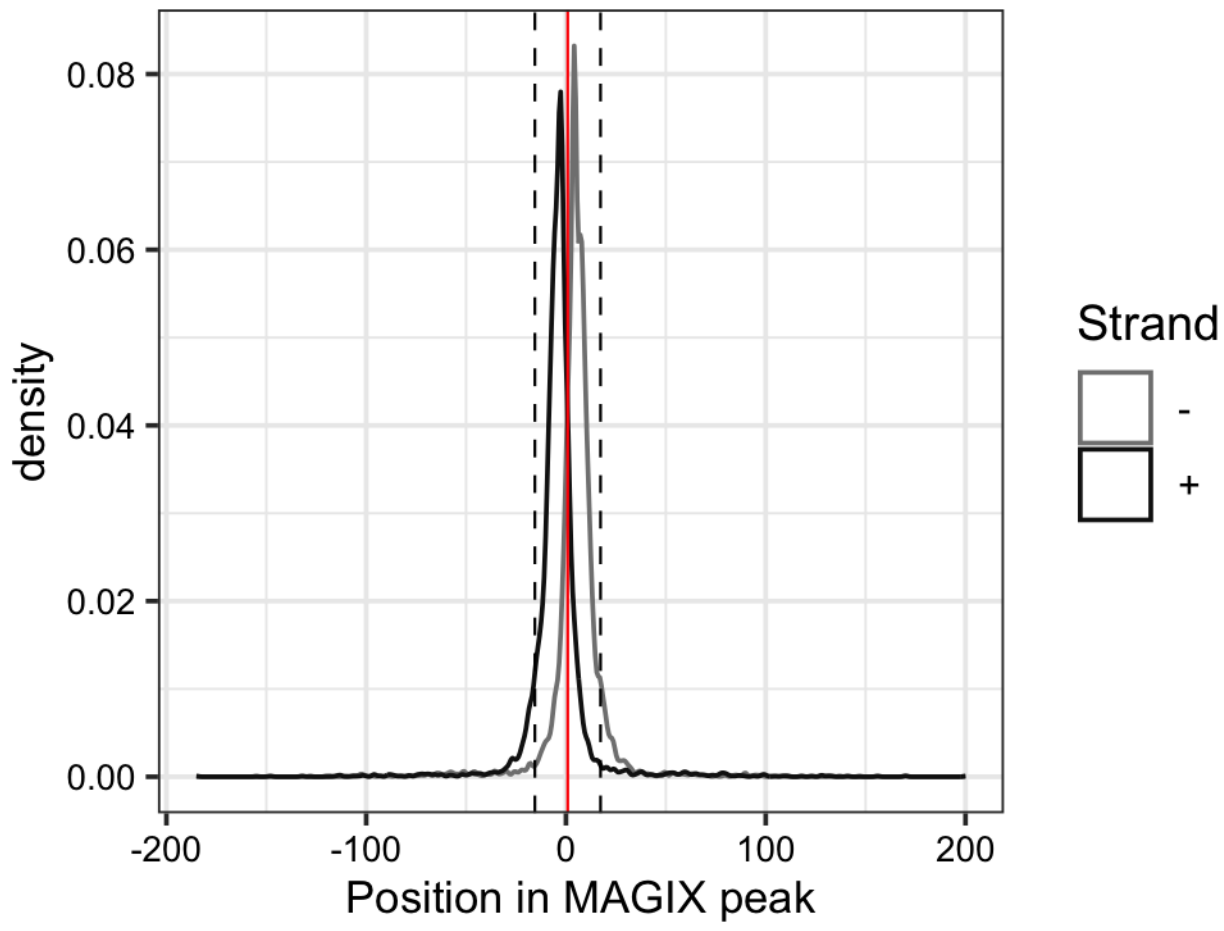

AUROC of top GRECO-BIT/MEX PWM in sliding windows (500 peaks, 50 peak step) along the MAGIX peaks (up to the top 75,000) ranked by the MAGIX enrichment coefficient and fraction of overlapping ChIP peaks in the same window

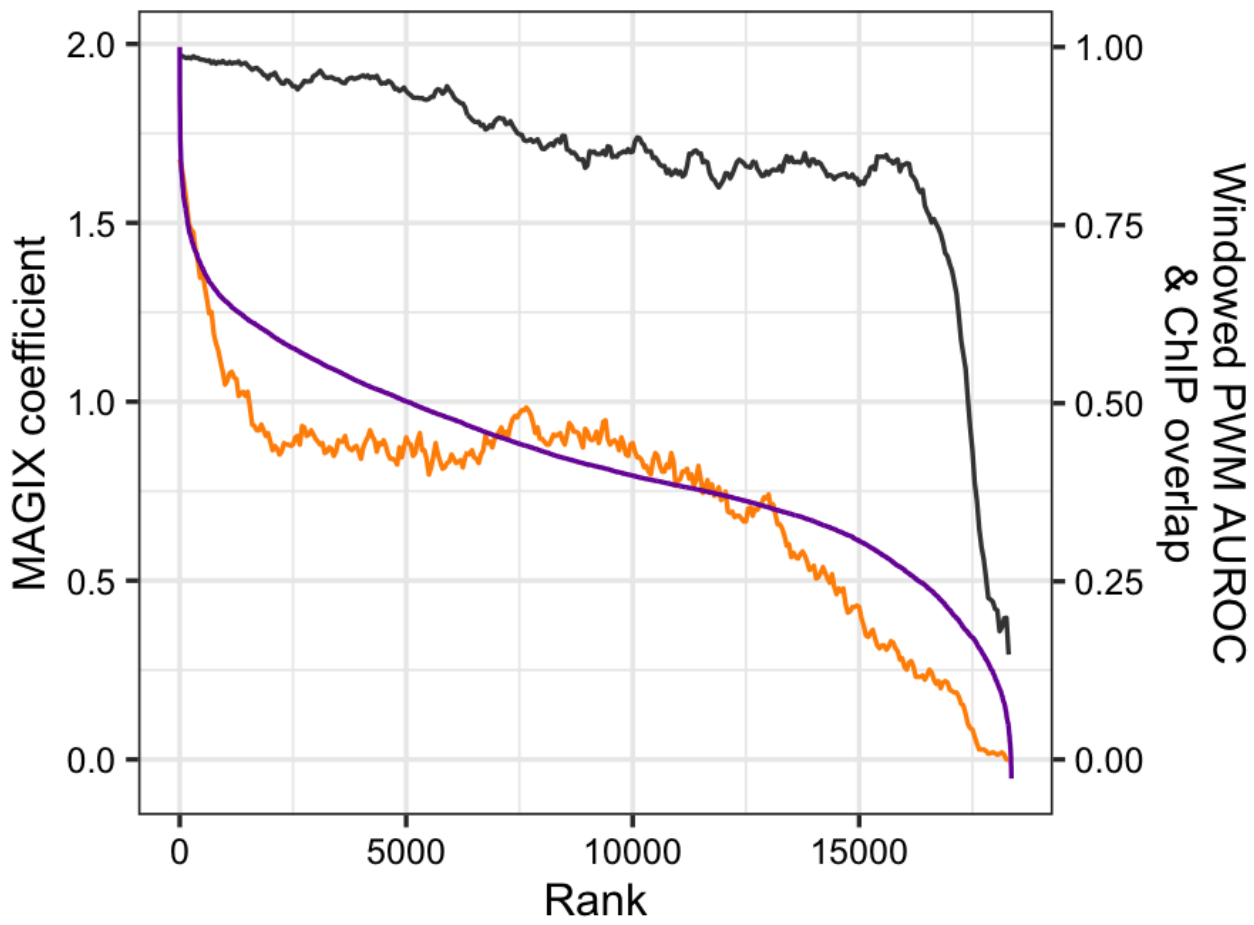

ZNF48

Top GRECO-BIT/MEX PWM: PWM004344

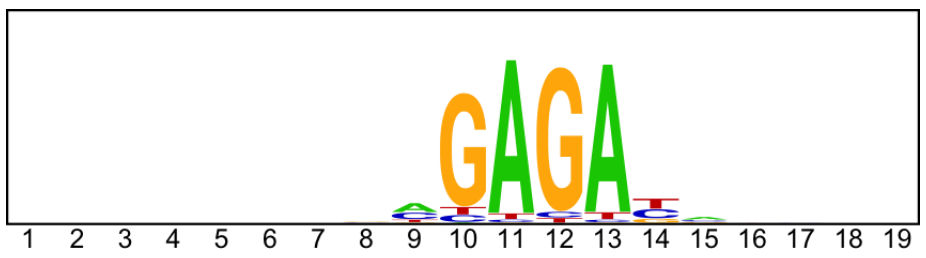

Location of top GRECO-BIT/MEX PWM in MAGIX peaks (up to the top 10,000)

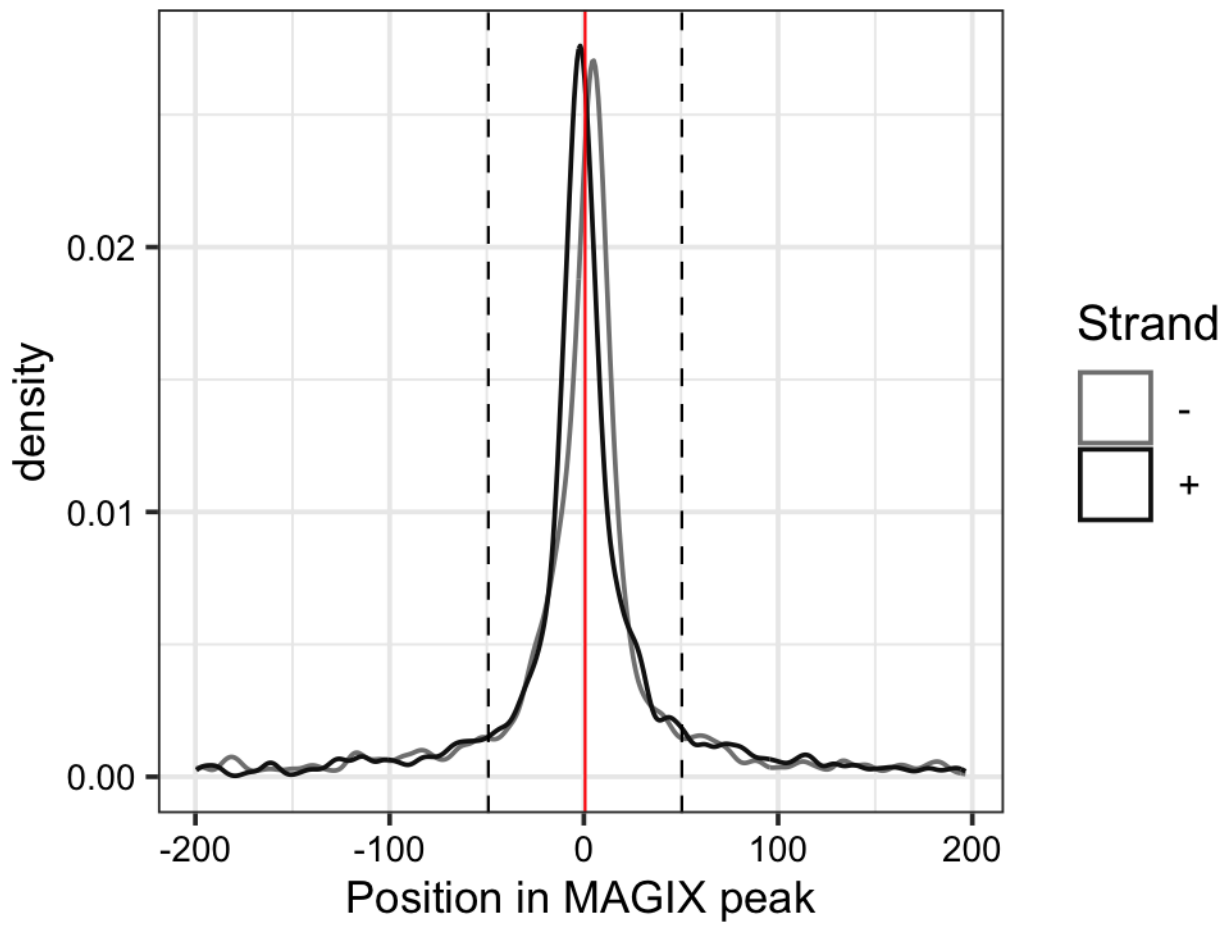

AUROC of top GRECO-BIT/MEX PWM in sliding windows (500 peaks, 50 peak step) along the MAGIX peaks (up to the top 75,000) ranked by the MAGIX enrichment coefficient and fraction of overlapping ChIP peaks in the same window

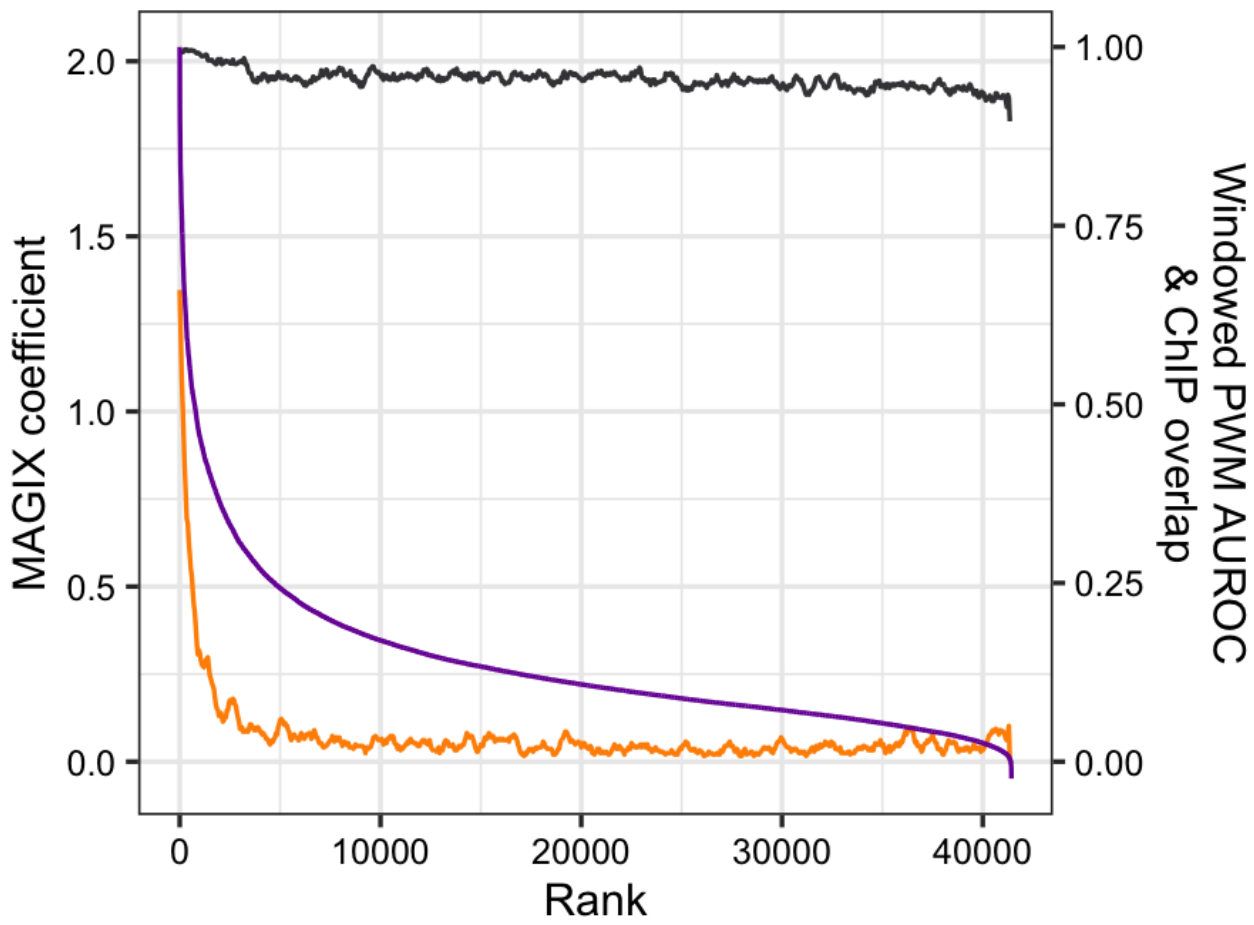

ZNF493.DBD2

Top GRECO-BIT/MEX PWM: PWM004415

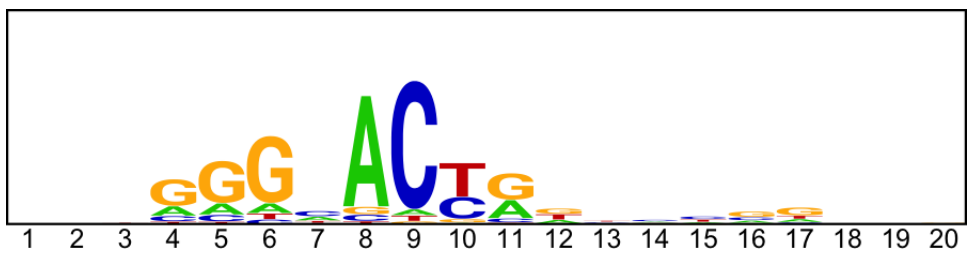

Location of top GRECO-BIT/MEX PWM in MAGIX peaks (up to the top 10,000)

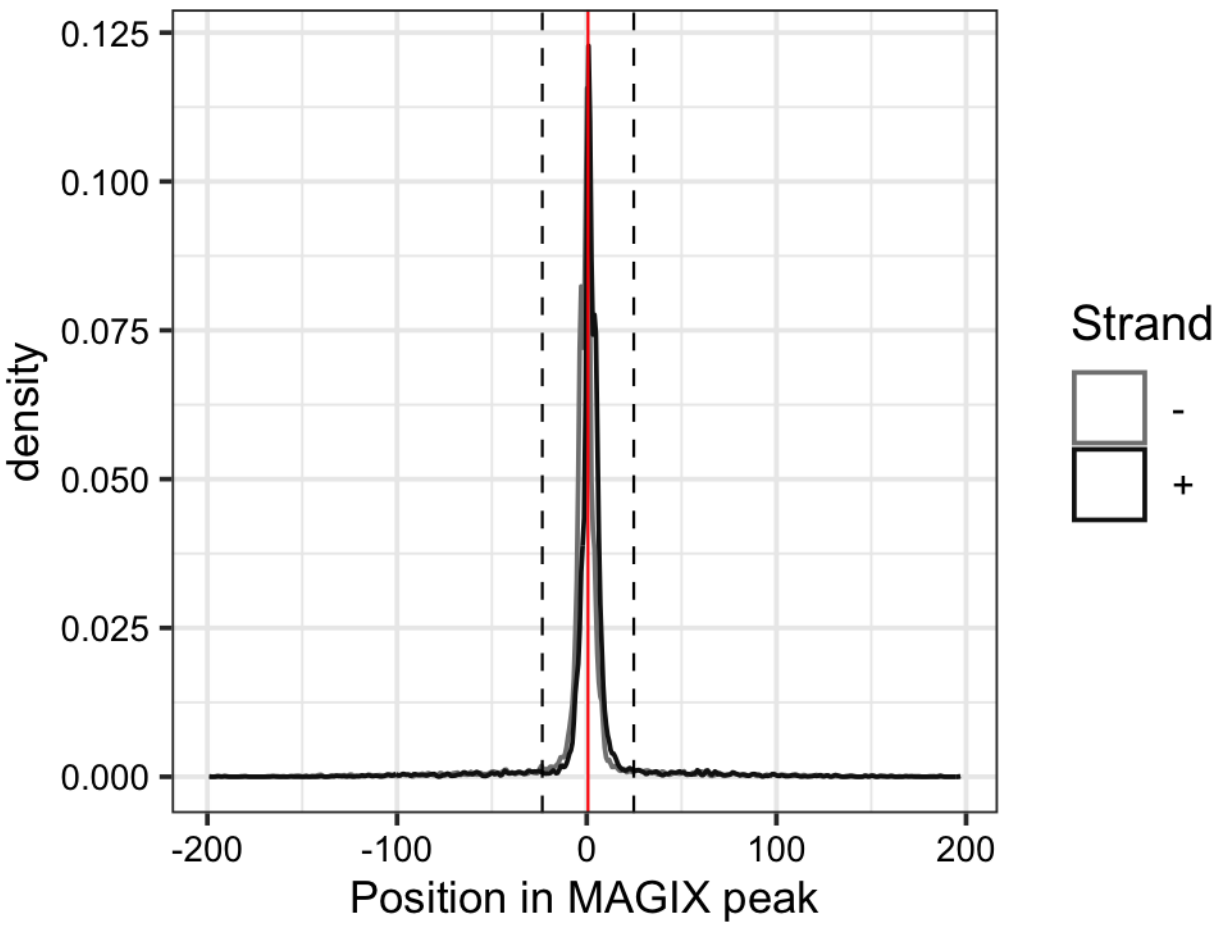

ZNF493.FL

Top GRECO-BIT/MEX PWM: PWM004411

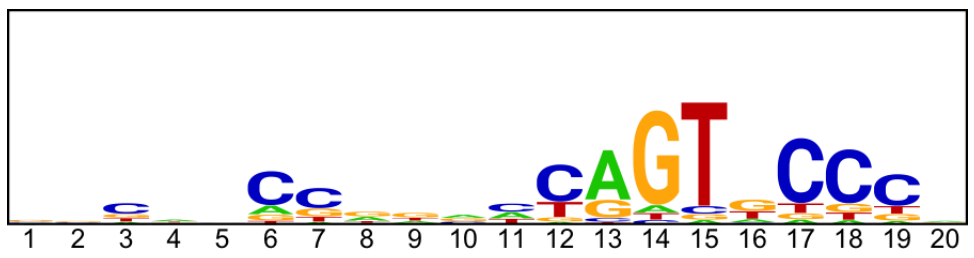

Location of top GRECO-BIT/MEX PWM in MAGIX peaks (up to the top 10,000)

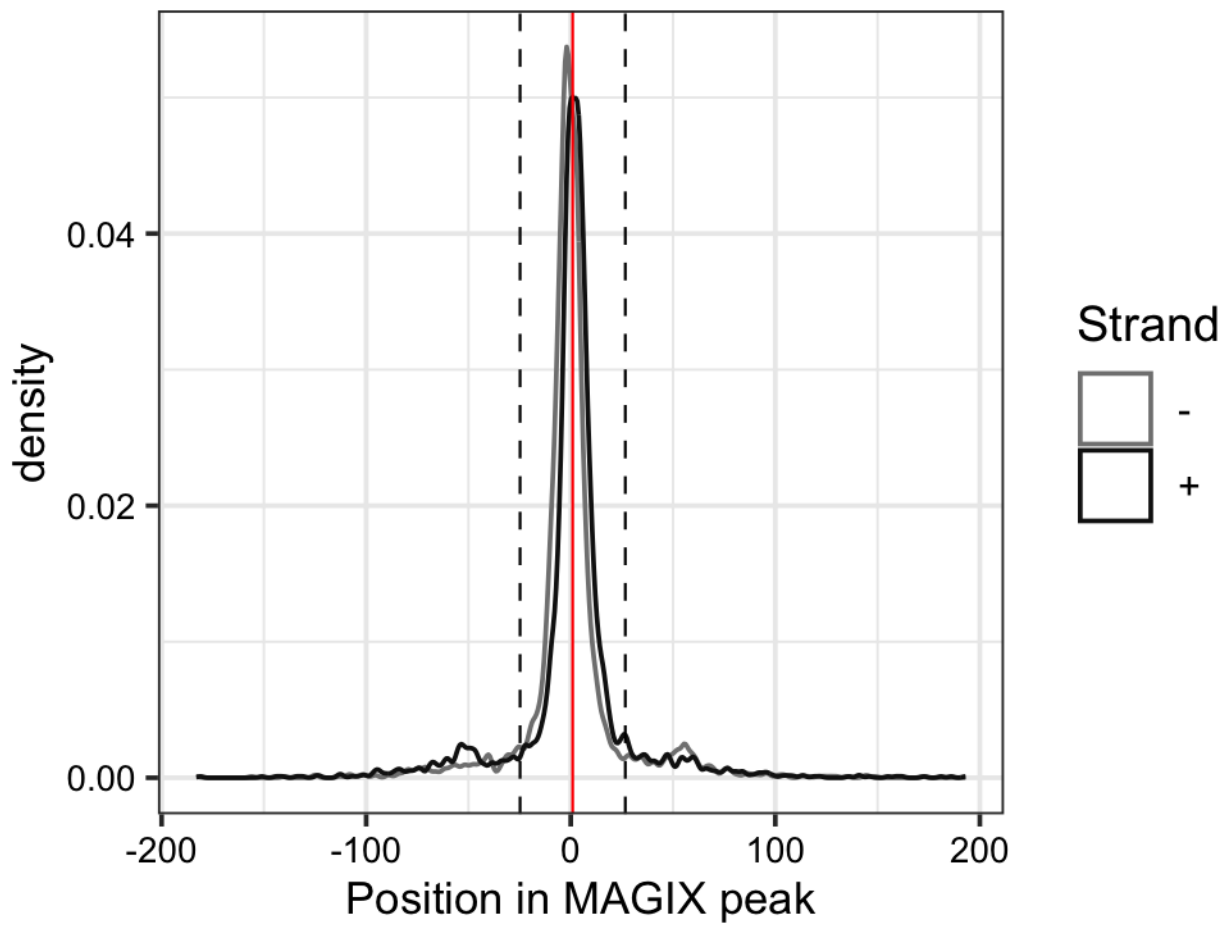

AUROC of top GRECO-BIT/MEX PWM in sliding windows (500 peaks, 50 peak step) along the MAGIX peaks (up to the top 75,000) ranked by the MAGIX enrichment coefficient and fraction of overlapping ChIP peaks in the same window

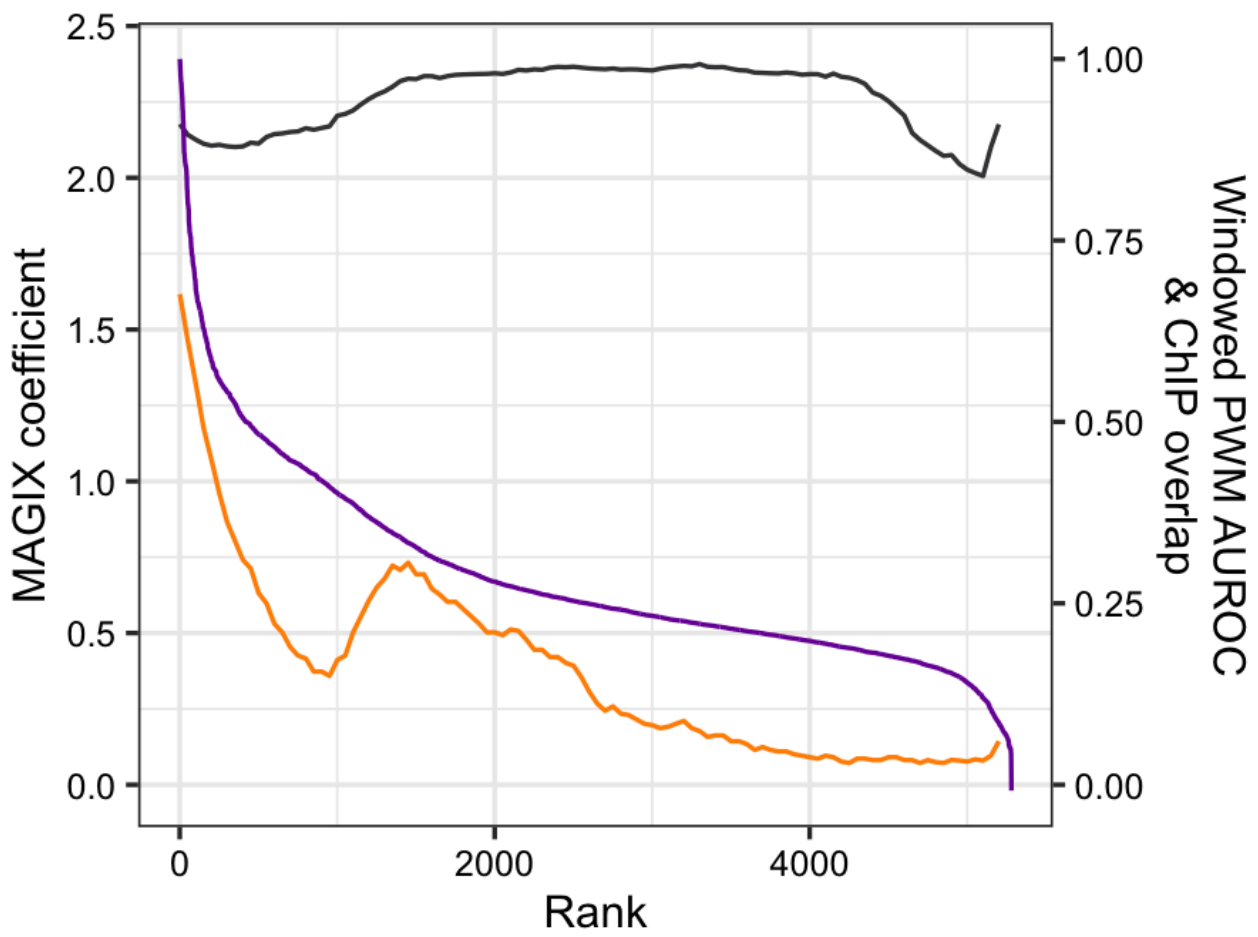

ZNF497

Top GRECO-BIT/MEX PWM: PWM004430

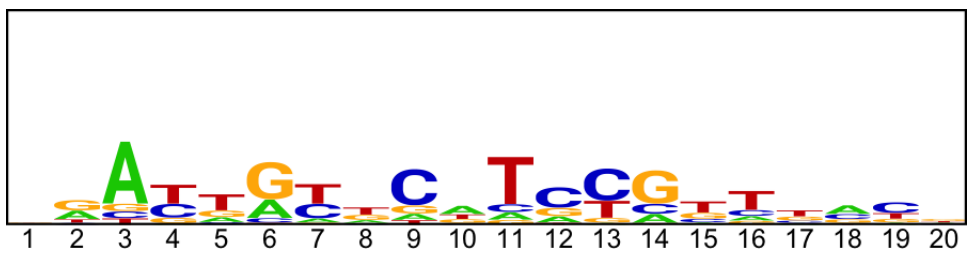

Location of top GRECO-BIT/MEX PWM in MAGIX peaks (up to the top 10,000)

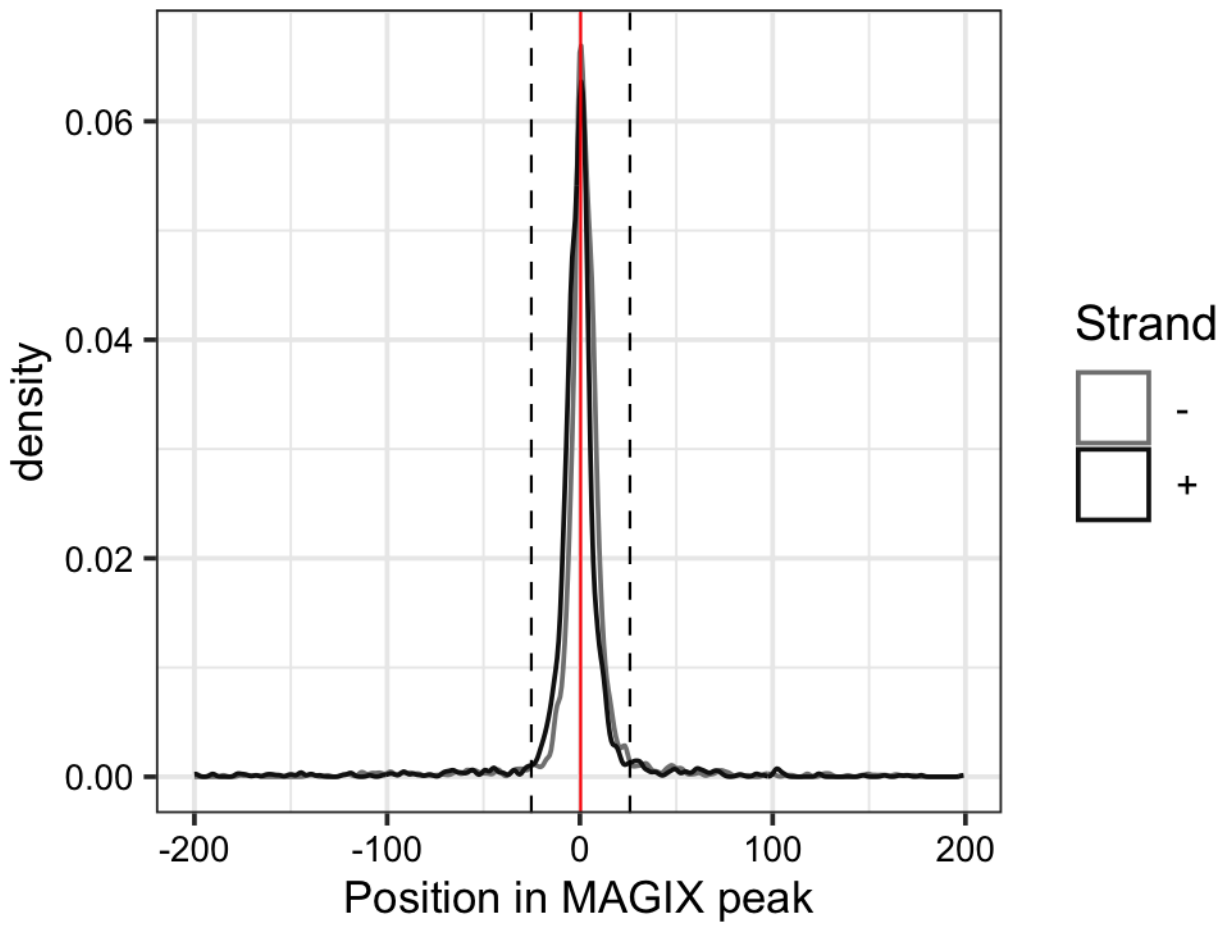

ZNF500

Top GRECO-BIT/MEX PWM: PWM004463

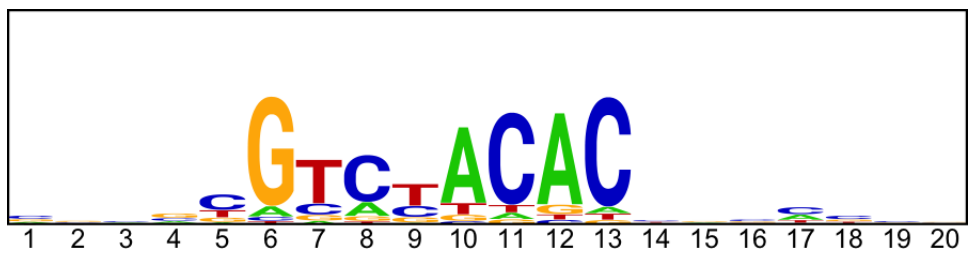

Location of top GRECO-BIT/MEX PWM in MAGIX peaks (up to the top 10,000)

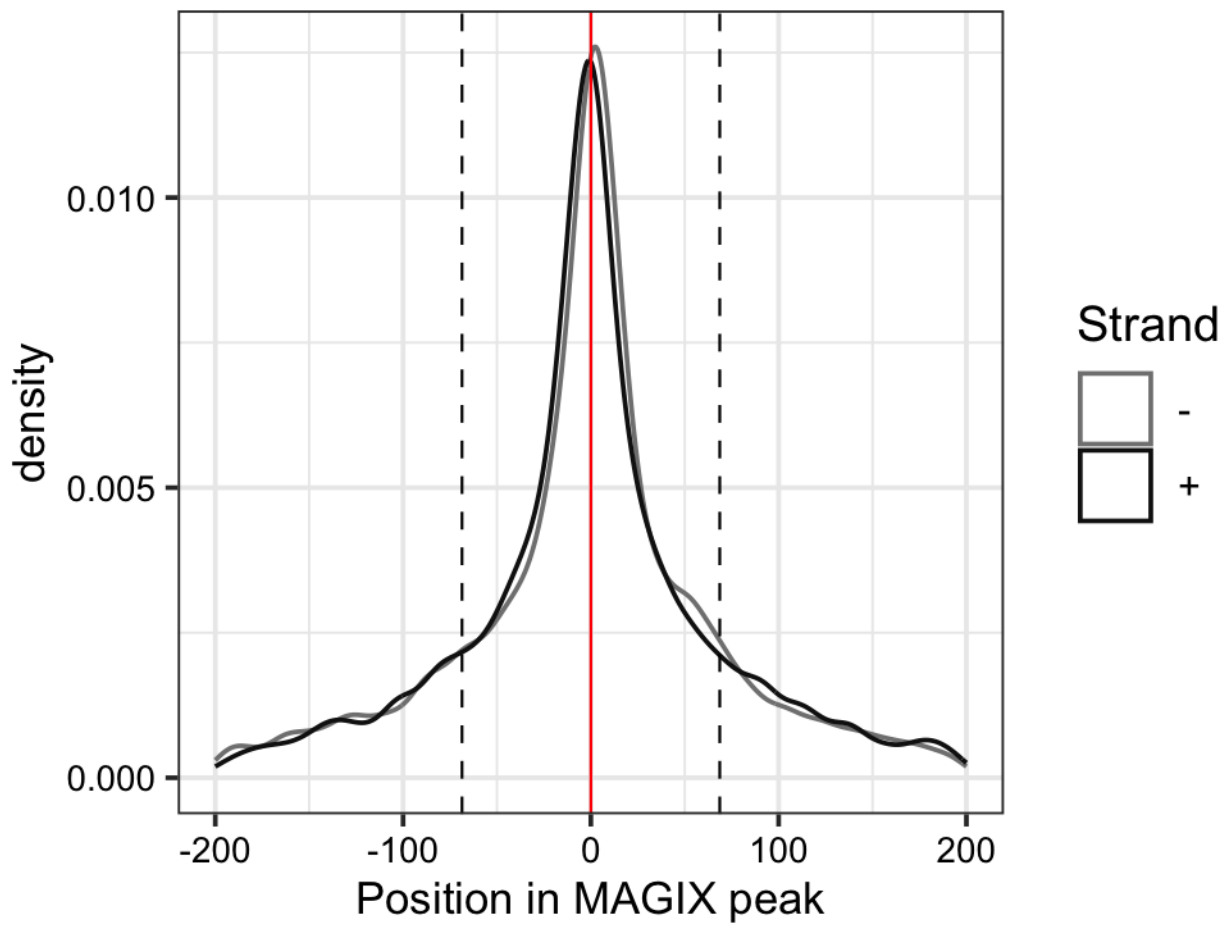

AUROC of top GRECO-BIT/MEX PWM in sliding windows (500 peaks, 50 peak step) along the MAGIX peaks (up to the top 75,000) ranked by the MAGIX enrichment coefficient and fraction of overlapping ChIP peaks in the same window

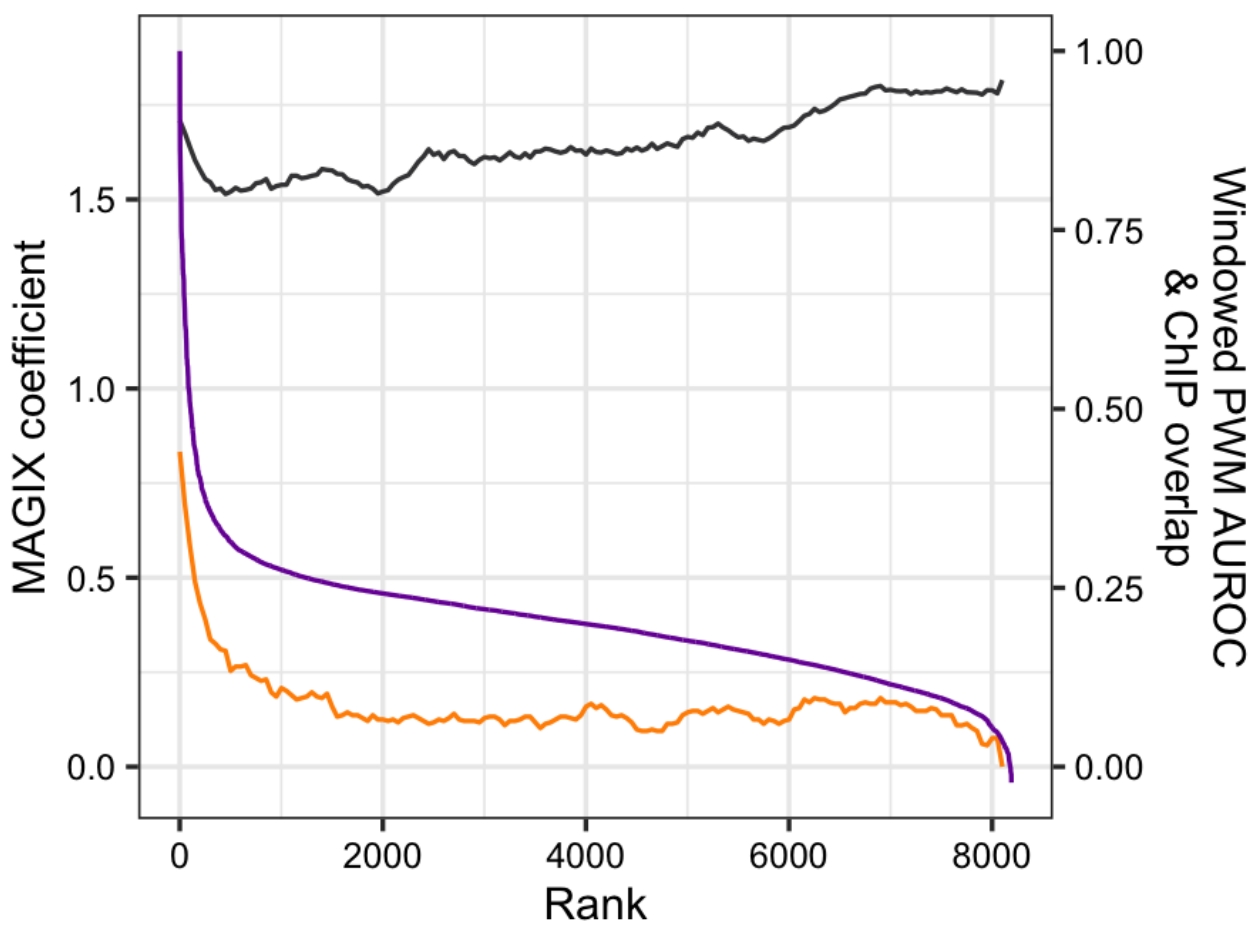

ZNF510

Top GRECO-BIT/MEX PWM: PWM004519

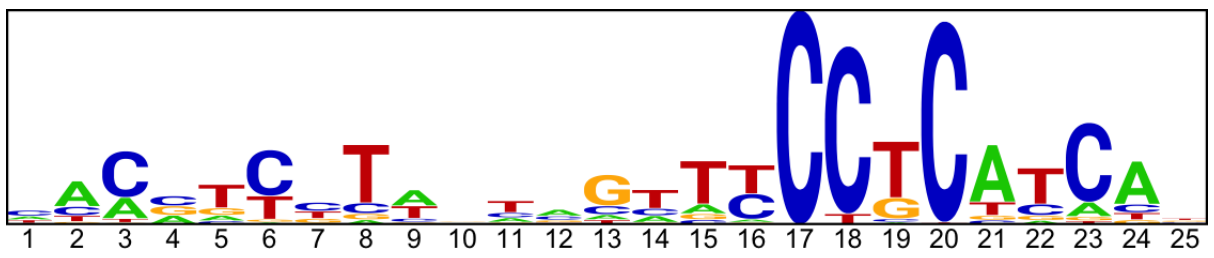

Location of top GRECO-BIT/MEX PWM in MAGIX peaks (up to the top 10,000)

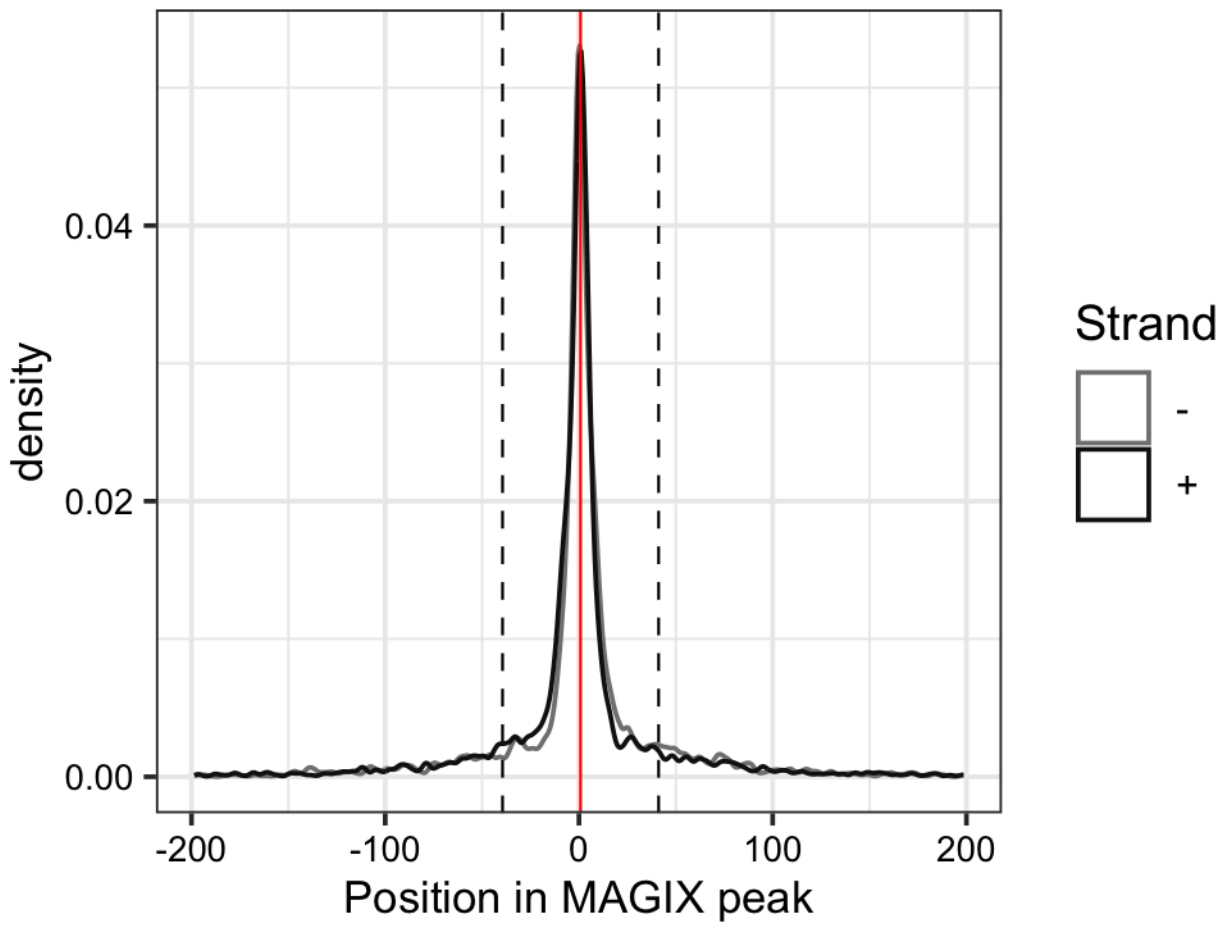

AUROC of top GRECO-BIT/MEX PWM in sliding windows (500 peaks, 50 peak step) along the MAGIX peaks (up to the top 75,000) ranked by the MAGIX enrichment coefficient and fraction of overlapping ChIP peaks in the same window

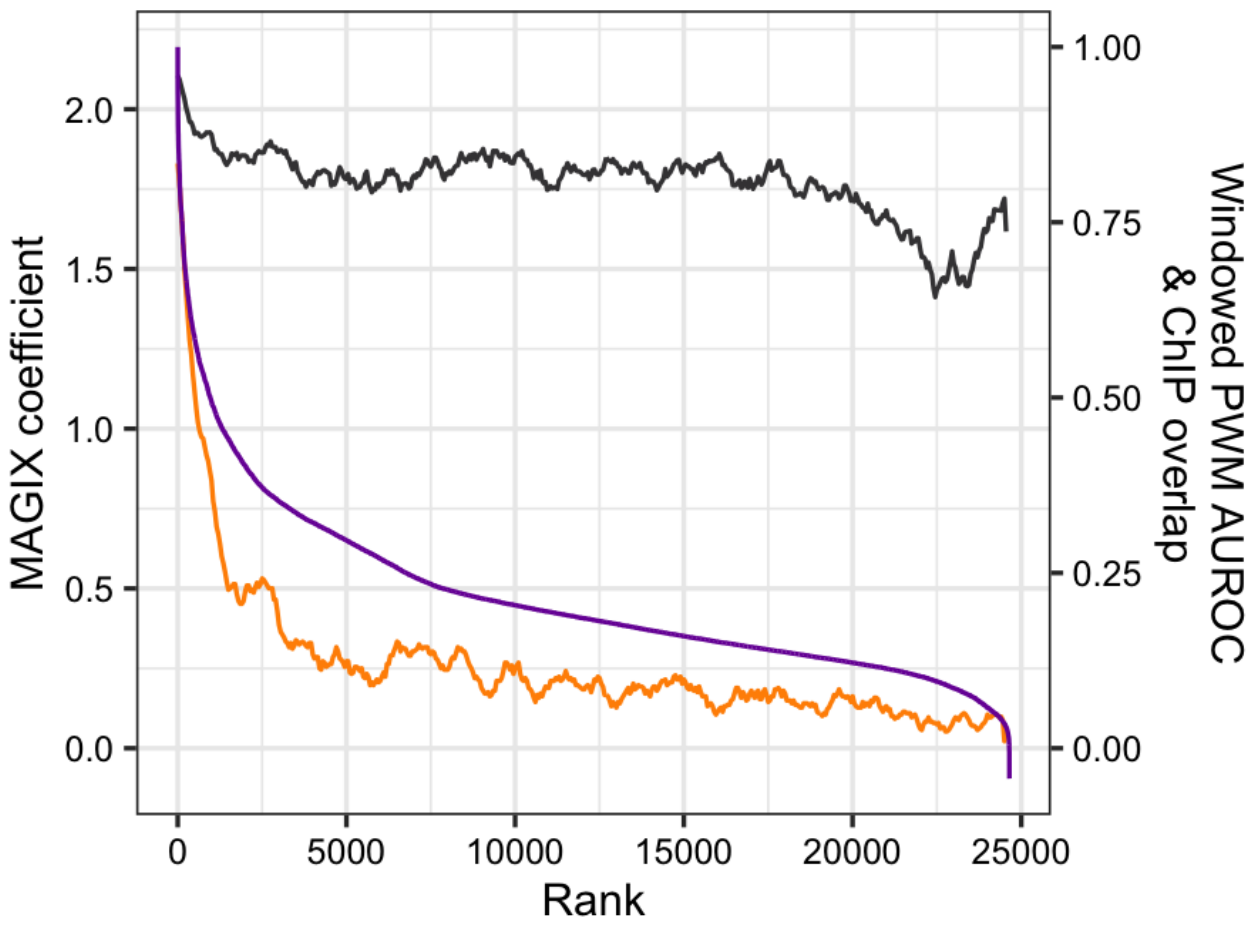

ZNF518B

Top GRECO-BIT/MEX PWM: PWM004578

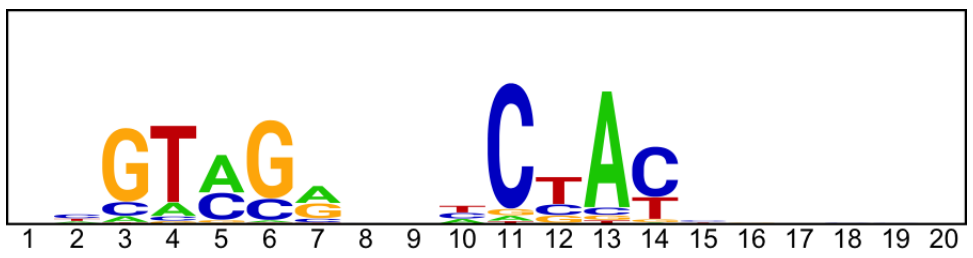

Location of top GRECO-BIT/MEX PWM in MAGIX peaks (up to the top 10,000)

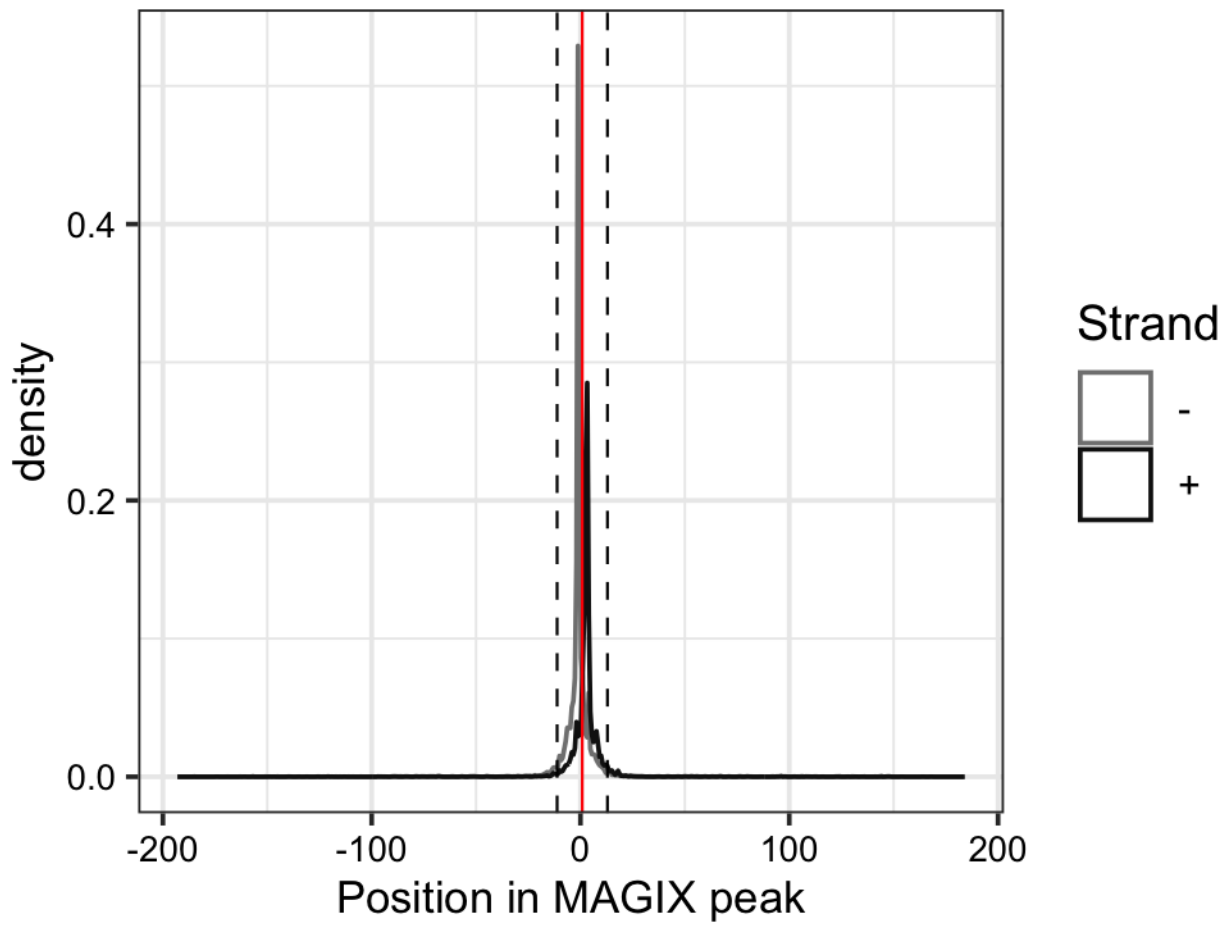

AUROC of top GRECO-BIT/MEX PWM in sliding windows (500 peaks, 50 peak step) along the MAGIX peaks (up to the top 75,000) ranked by the MAGIX enrichment coefficient and fraction of overlapping ChIP peaks in the same window

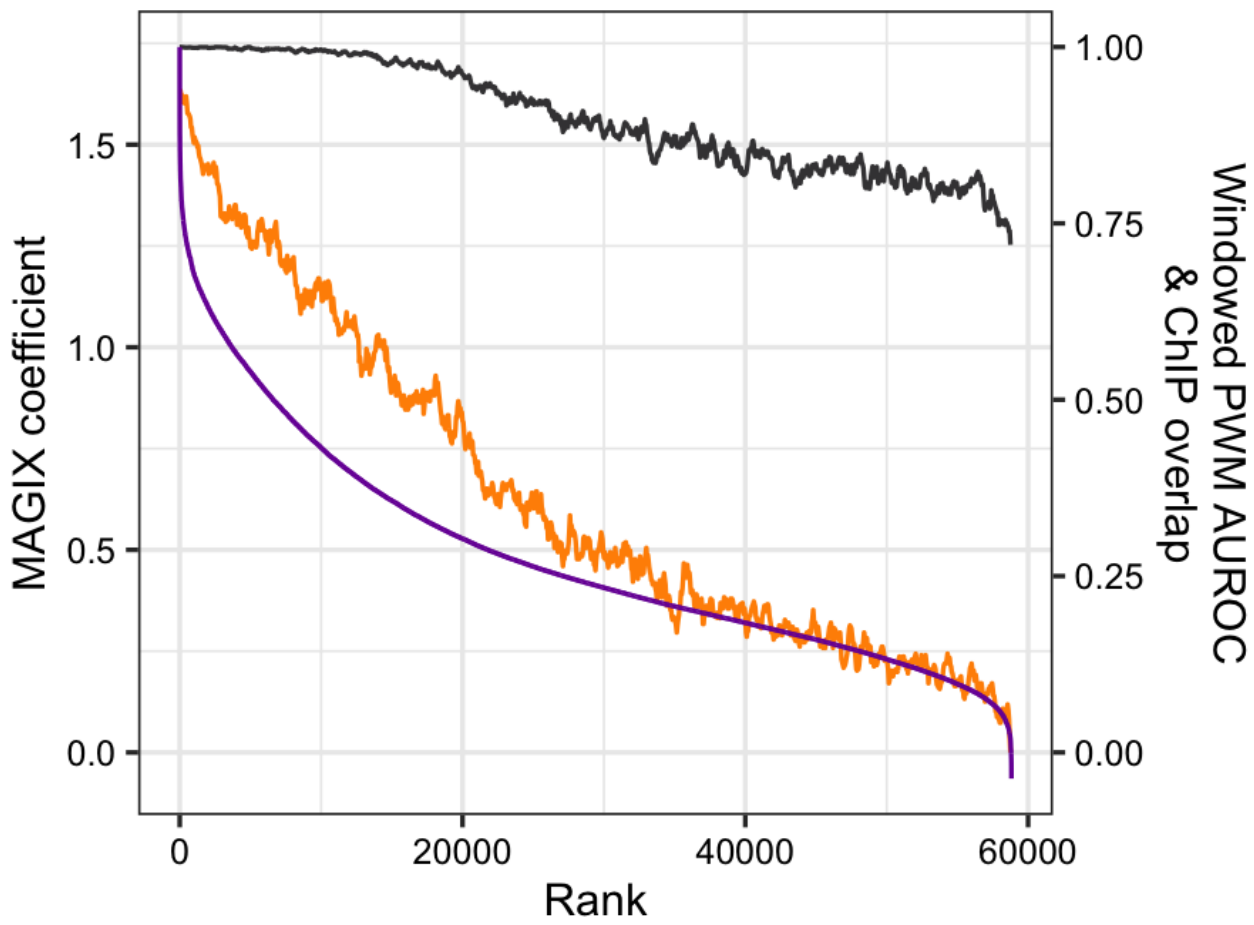

ZNF532.DBD2

Top GRECO-BIT/MEX PWM: PWM004588

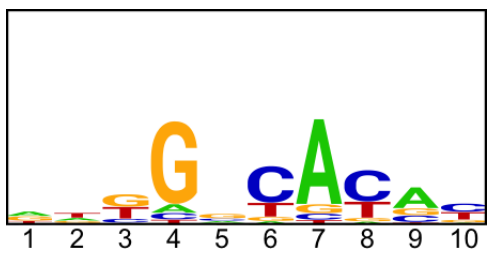

Location of top GRECO-BIT/MEX PWM in MAGIX peaks (up to the top 10,000)

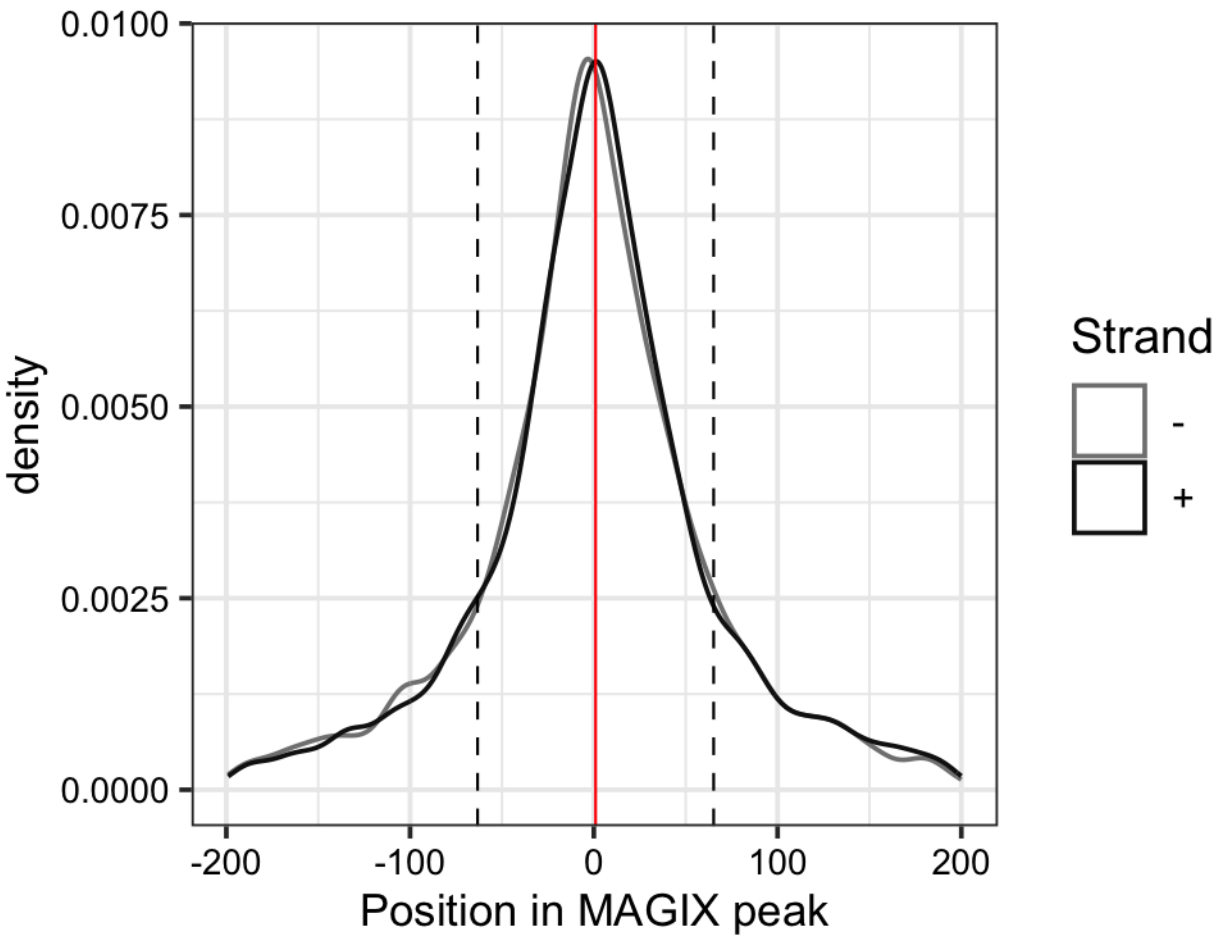

ZNF536.DBD2

Top GRECO-BIT/MEX PWM: PWM004608

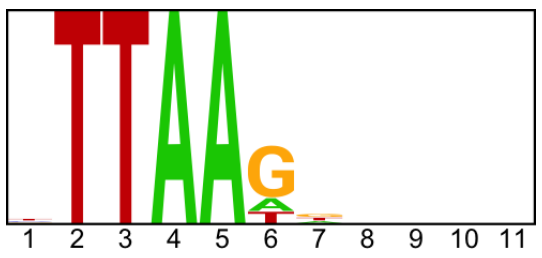

Location of top GRECO-BIT/MEX PWM in MAGIX peaks (up to the top 10,000)

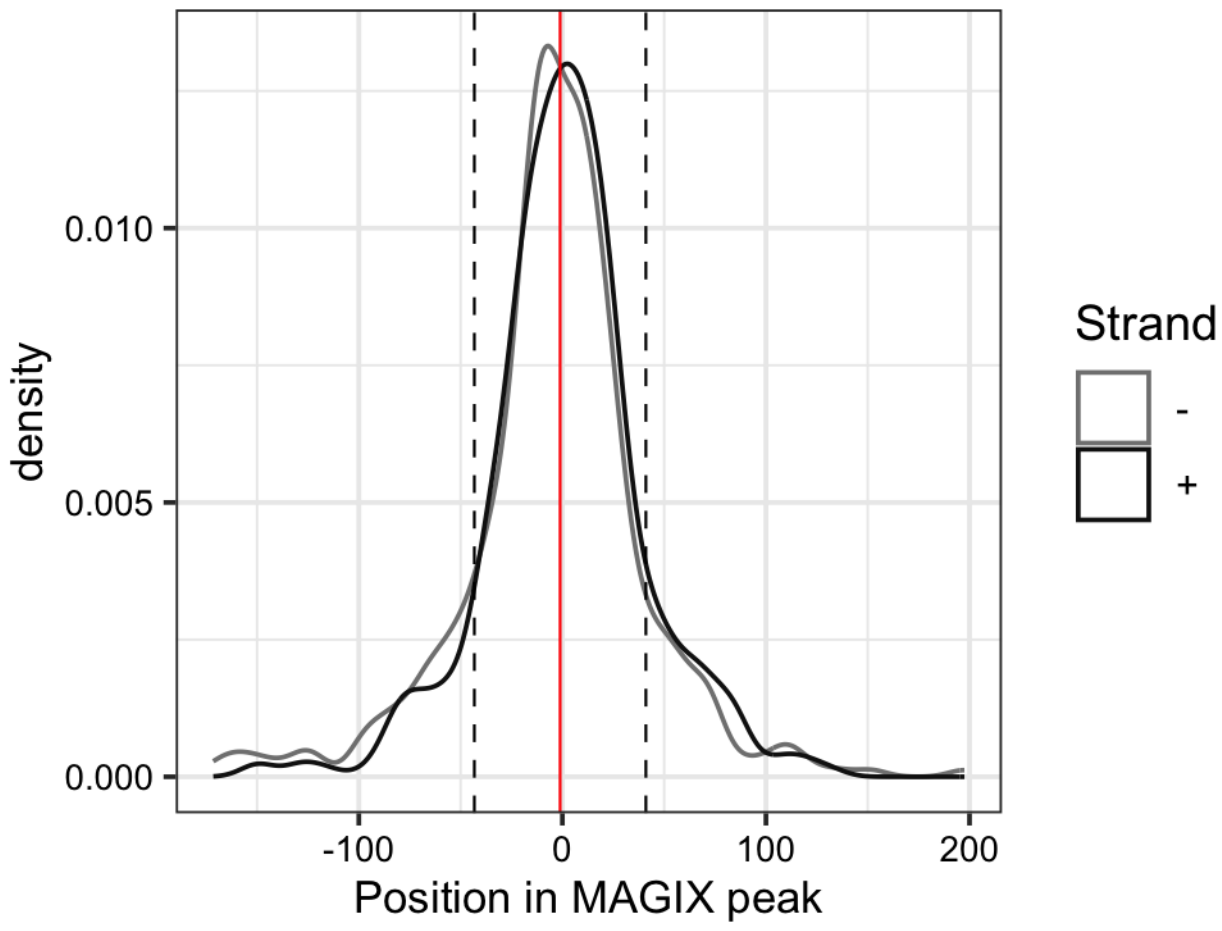

ZNF551

Top GRECO-BIT/MEX PWM: PWM004658

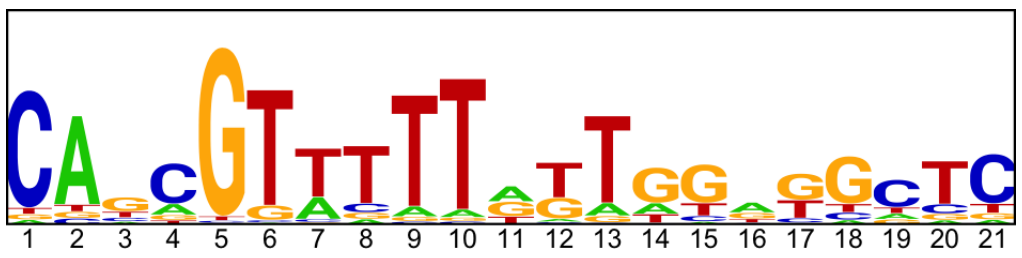

Location of top GRECO-BIT/MEX PWM in MAGIX peaks (up to the top 10,000)

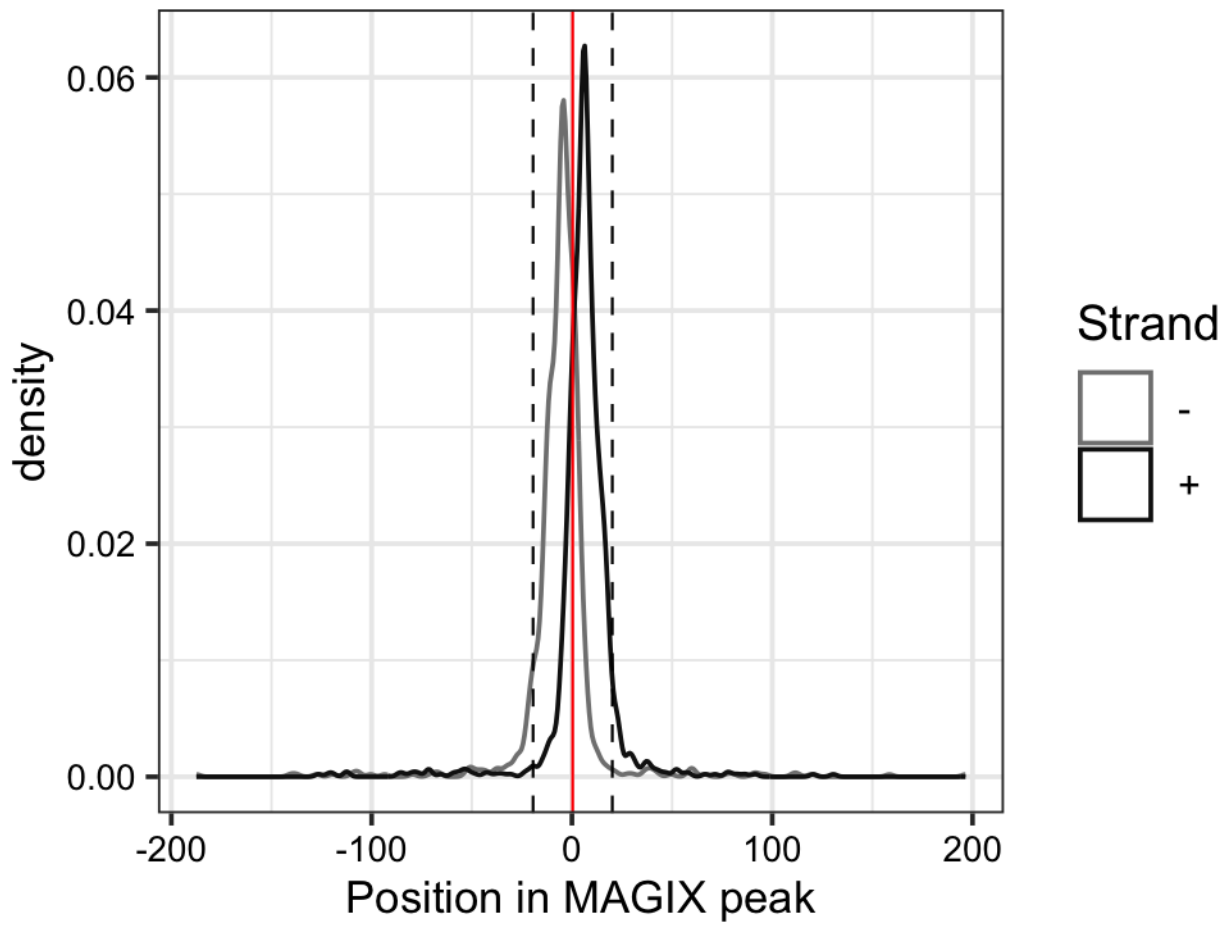

AUROC of top GRECO-BIT/MEX PWM in sliding windows (500 peaks, 50 peak step) along the MAGIX peaks (up to the top 75,000) ranked by the MAGIX enrichment coefficient and fraction of overlapping ChIP peaks in the same window

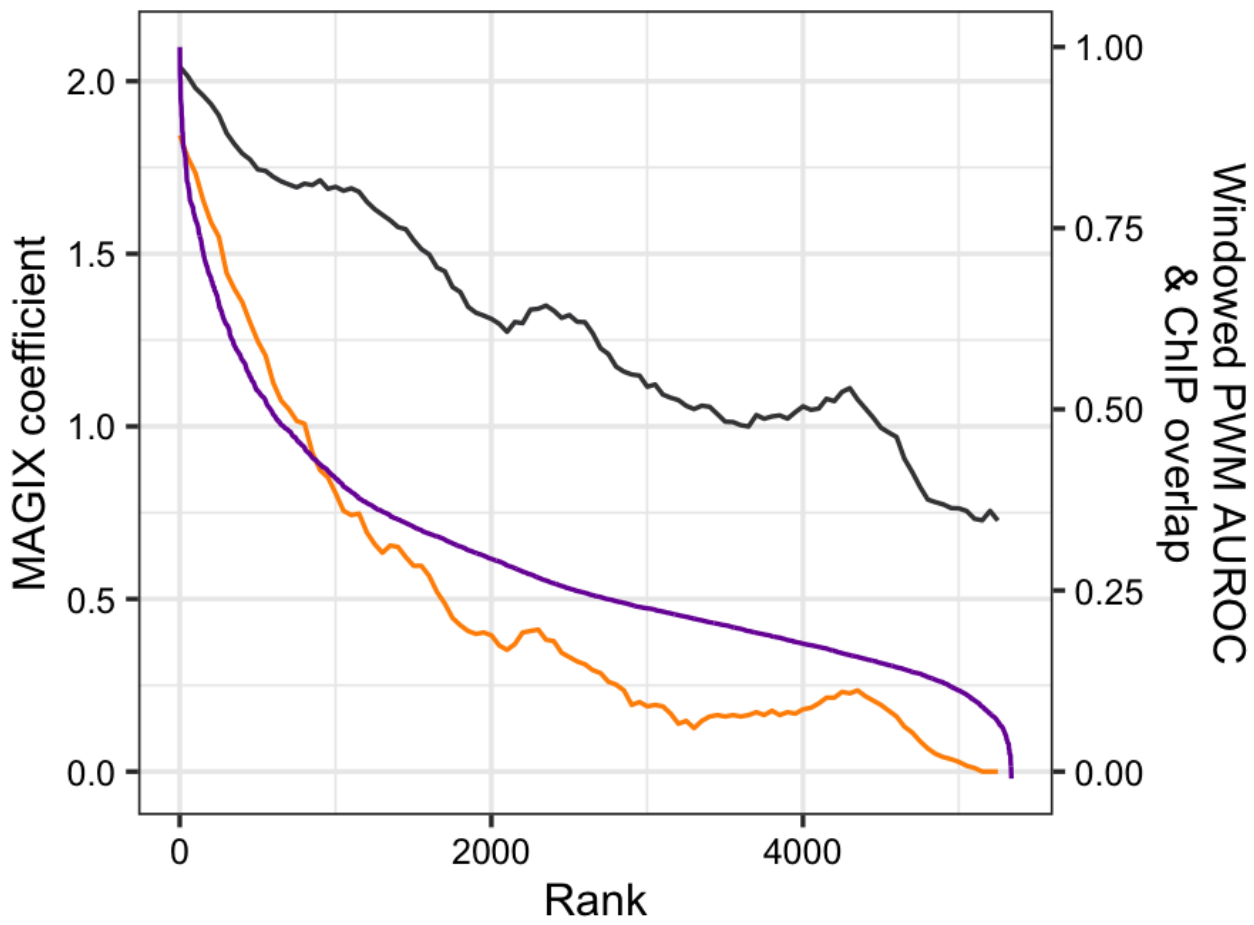

ZNF568

Top GRECO-BIT/MEX PWM: PWM004669

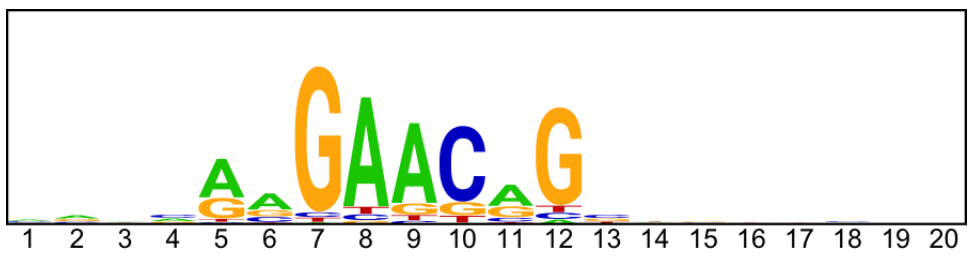

Location of top GRECO-BIT/MEX PWM in MAGIX peaks (up to the top 10,000)

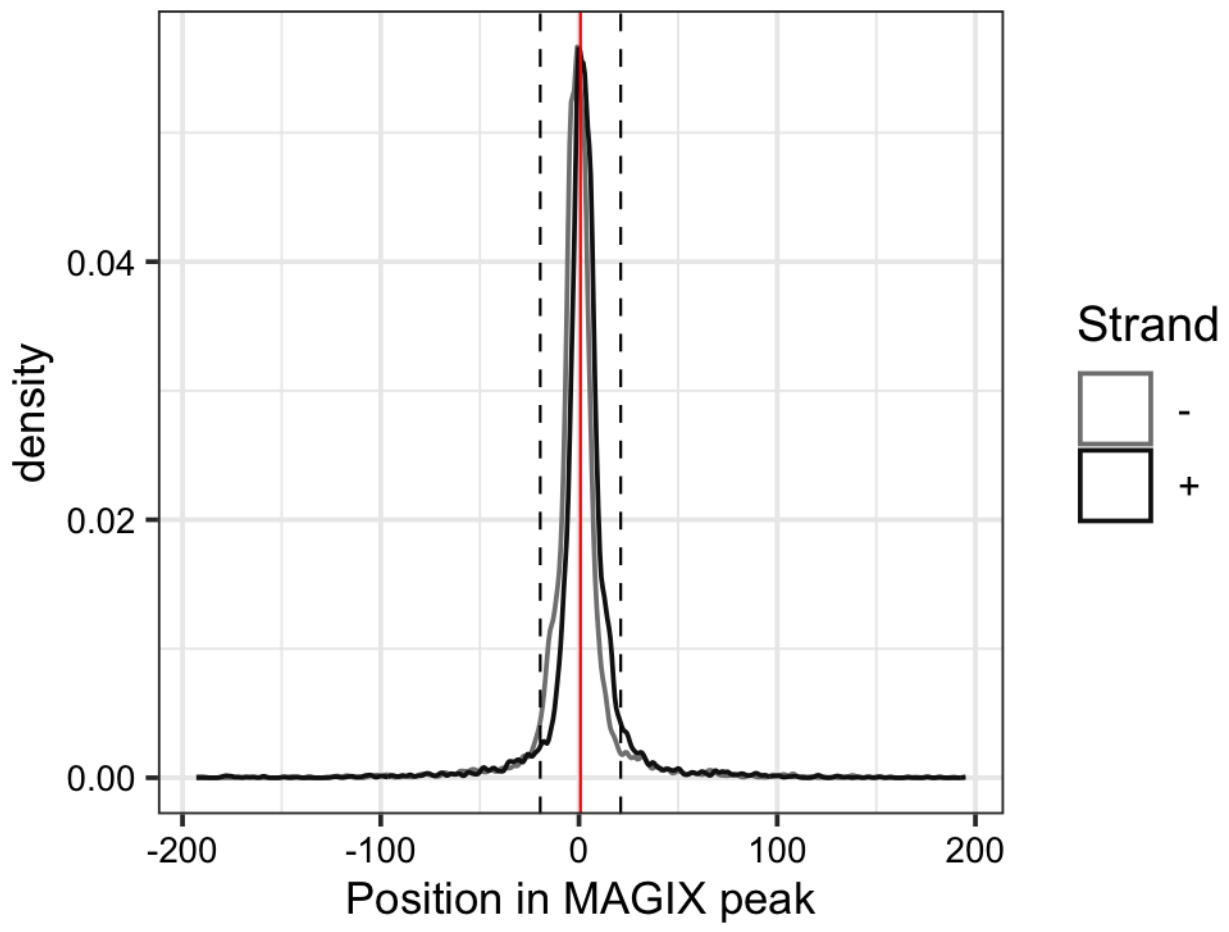

ZNF569

Top GRECO-BIT/MEX PWM: PWM004689

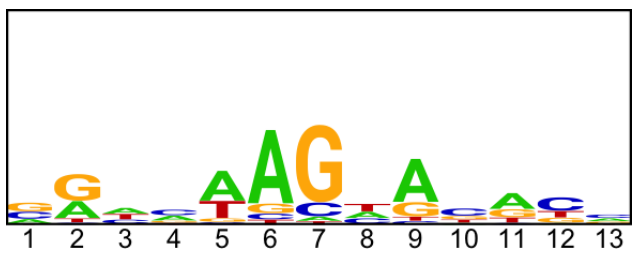

Location of top GRECO-BIT/MEX PWM in MAGIX peaks (up to the top 10,000)

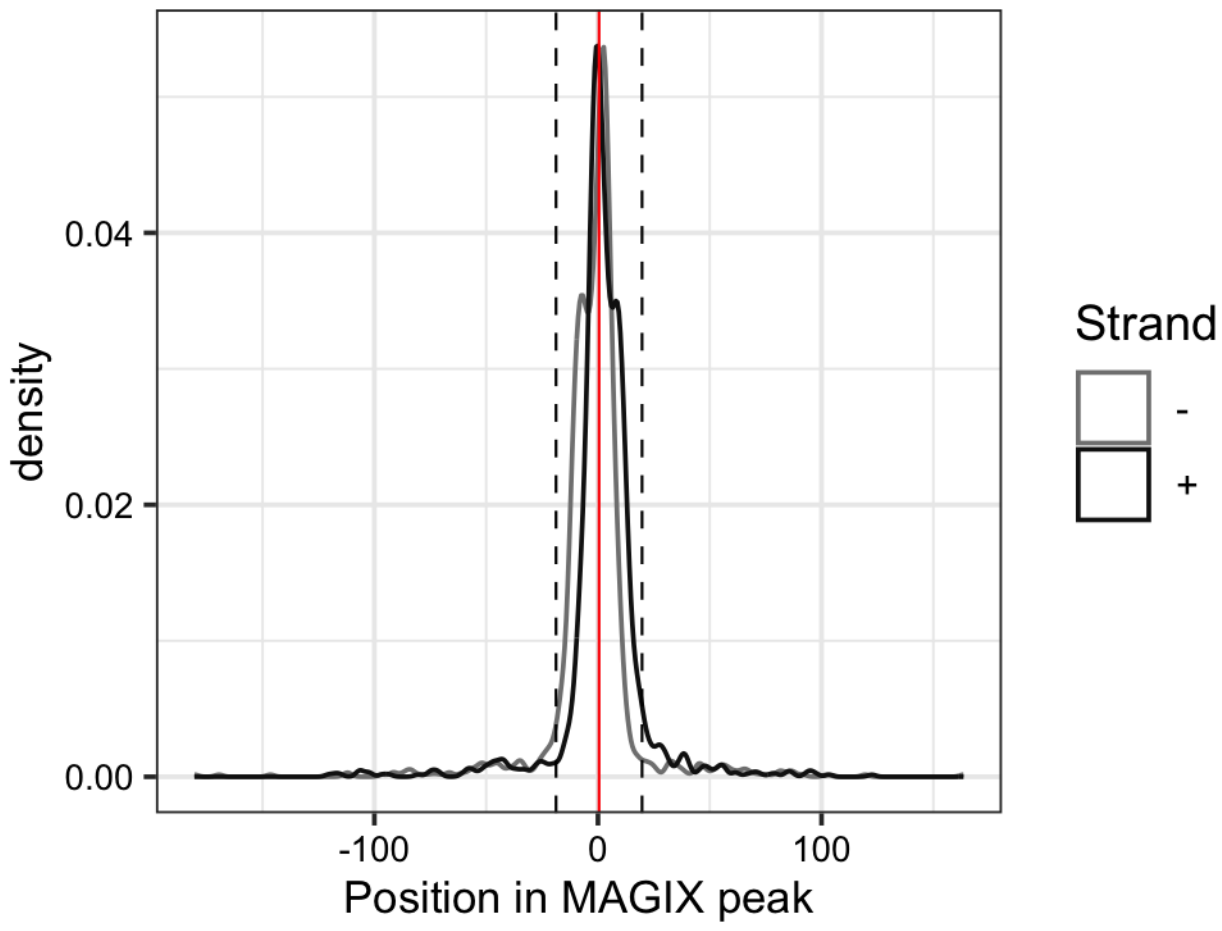

ZNF57

Top GRECO-BIT/MEX PWM: PWM004716

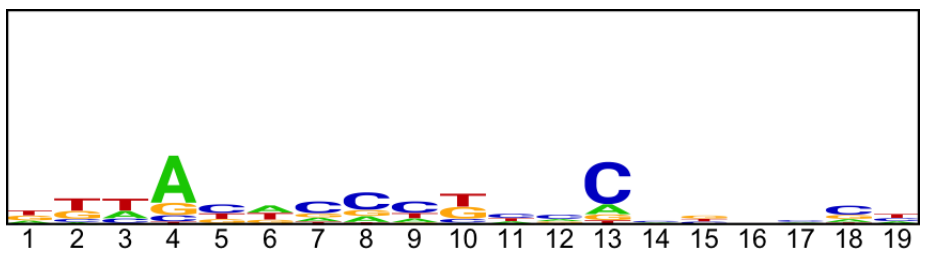

Location of top GRECO-BIT/MEX PWM in MAGIX peaks (up to the top 10,000)

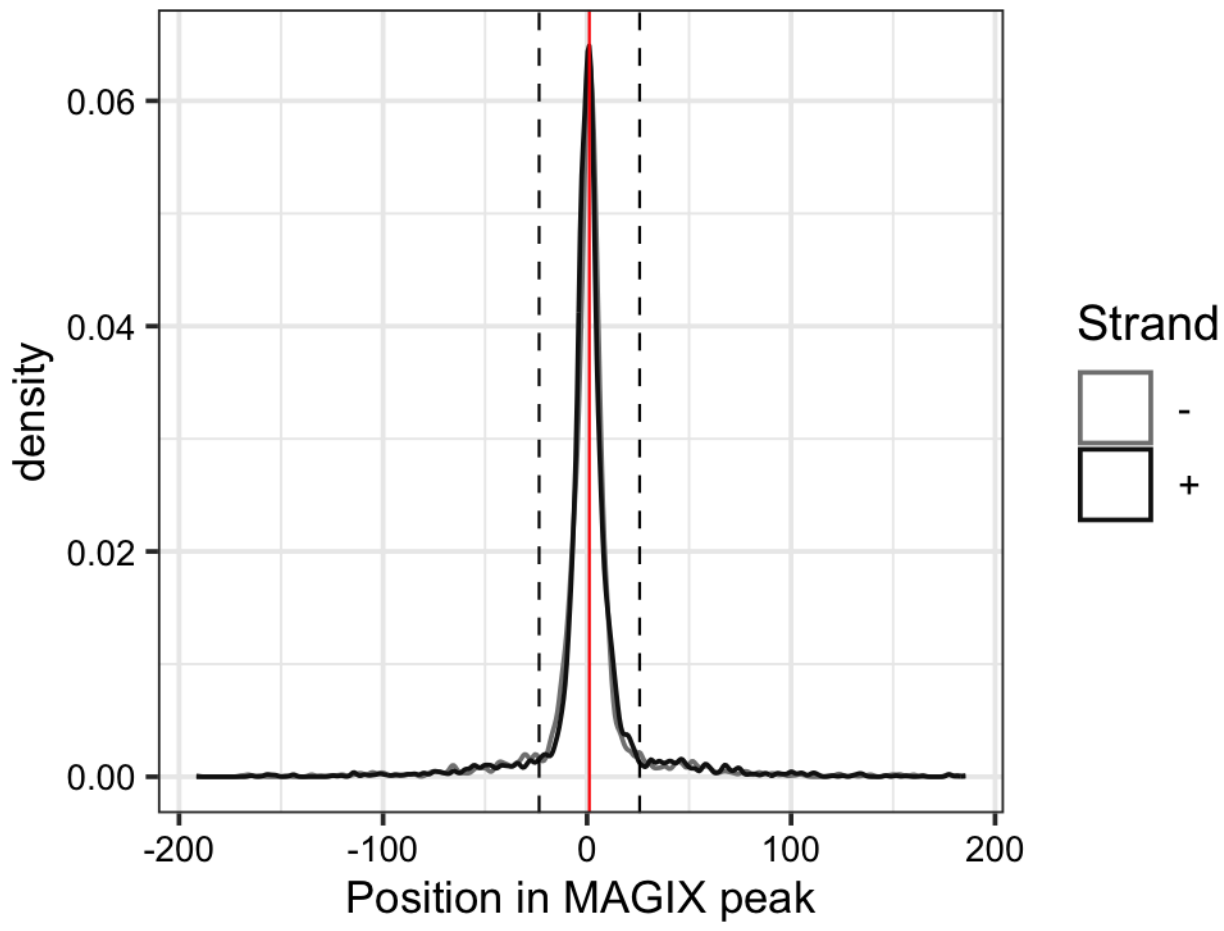

AUROC of top GRECO-BIT/MEX PWM in sliding windows (500 peaks, 50 peak step) along the MAGIX peaks (up to the top 75,000) ranked by the MAGIX enrichment coefficient and fraction of overlapping ChIP peaks in the same window

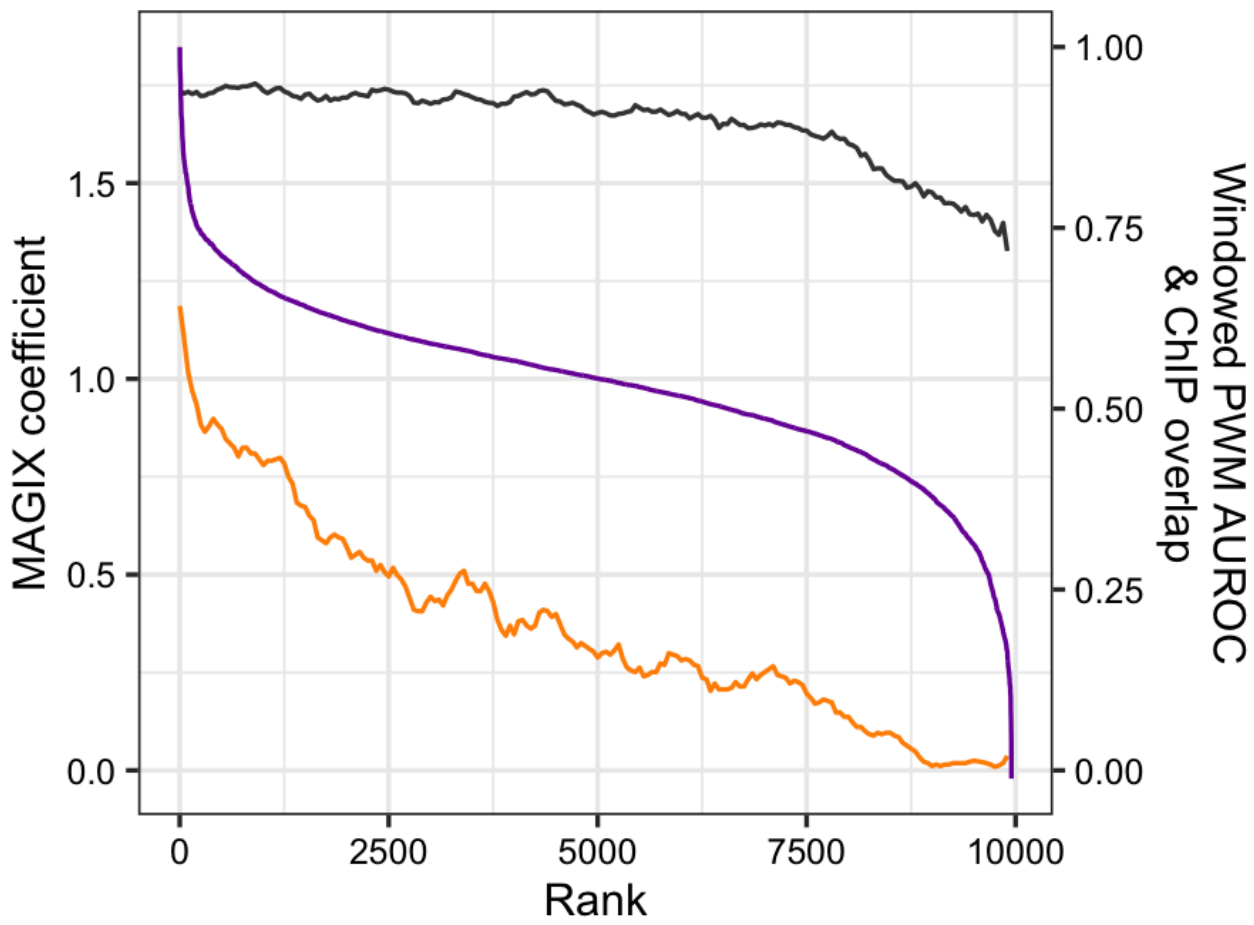

ZNF575

Top GRECO-BIT/MEX PWM: PWM004745

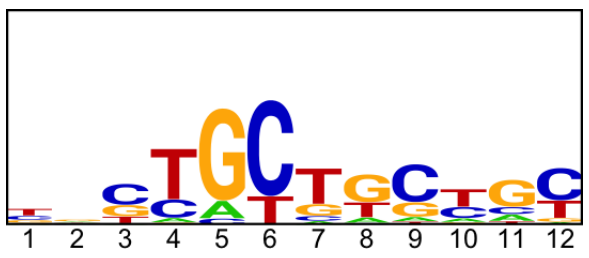

Location of top GRECO-BIT/MEX PWM in MAGIX peaks (up to the top 10,000)

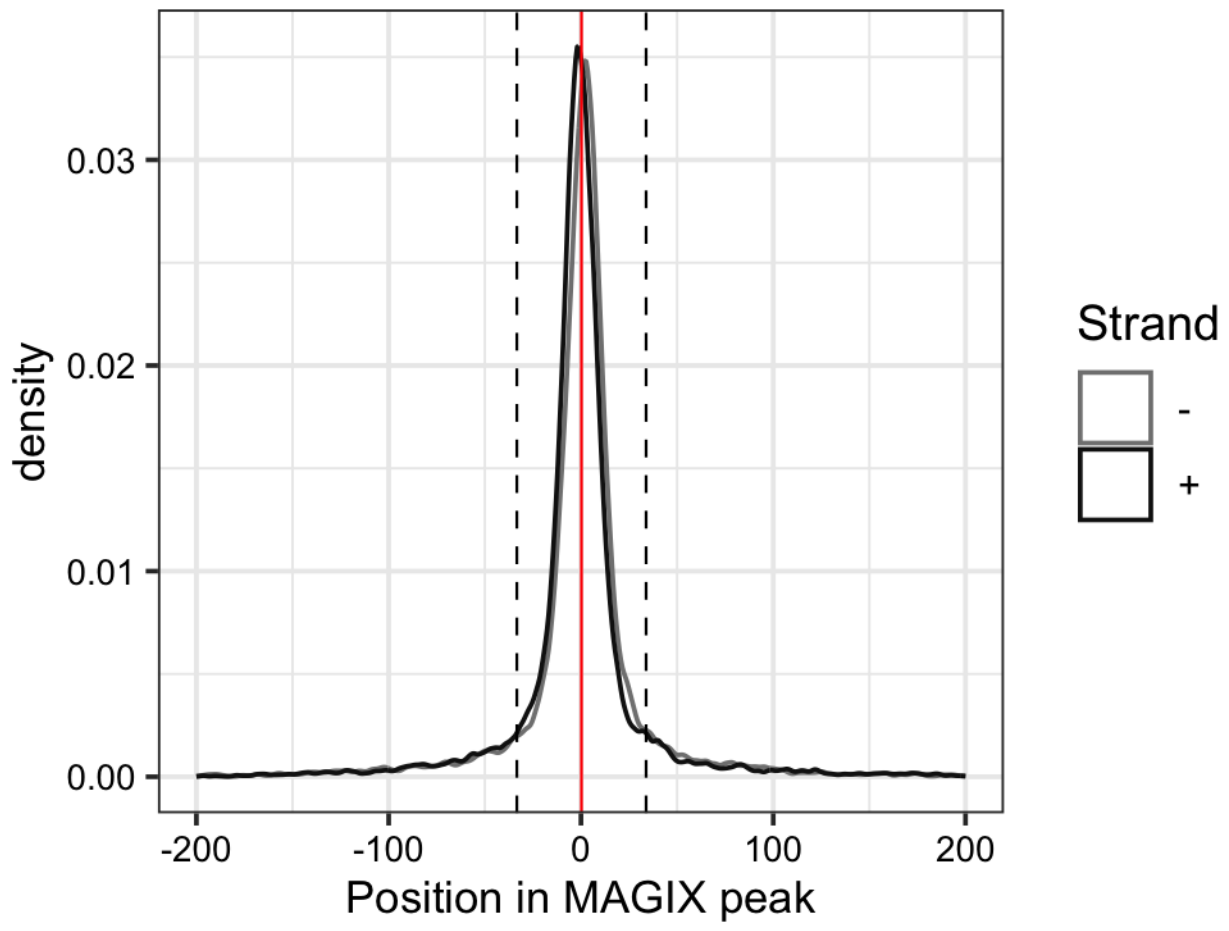

AUROC of top GRECO-BIT/MEX PWM in sliding windows (500 peaks, 50 peak step) along the MAGIX peaks (up to the top 75,000) ranked by the MAGIX enrichment coefficient and fraction of overlapping ChIP peaks in the same window

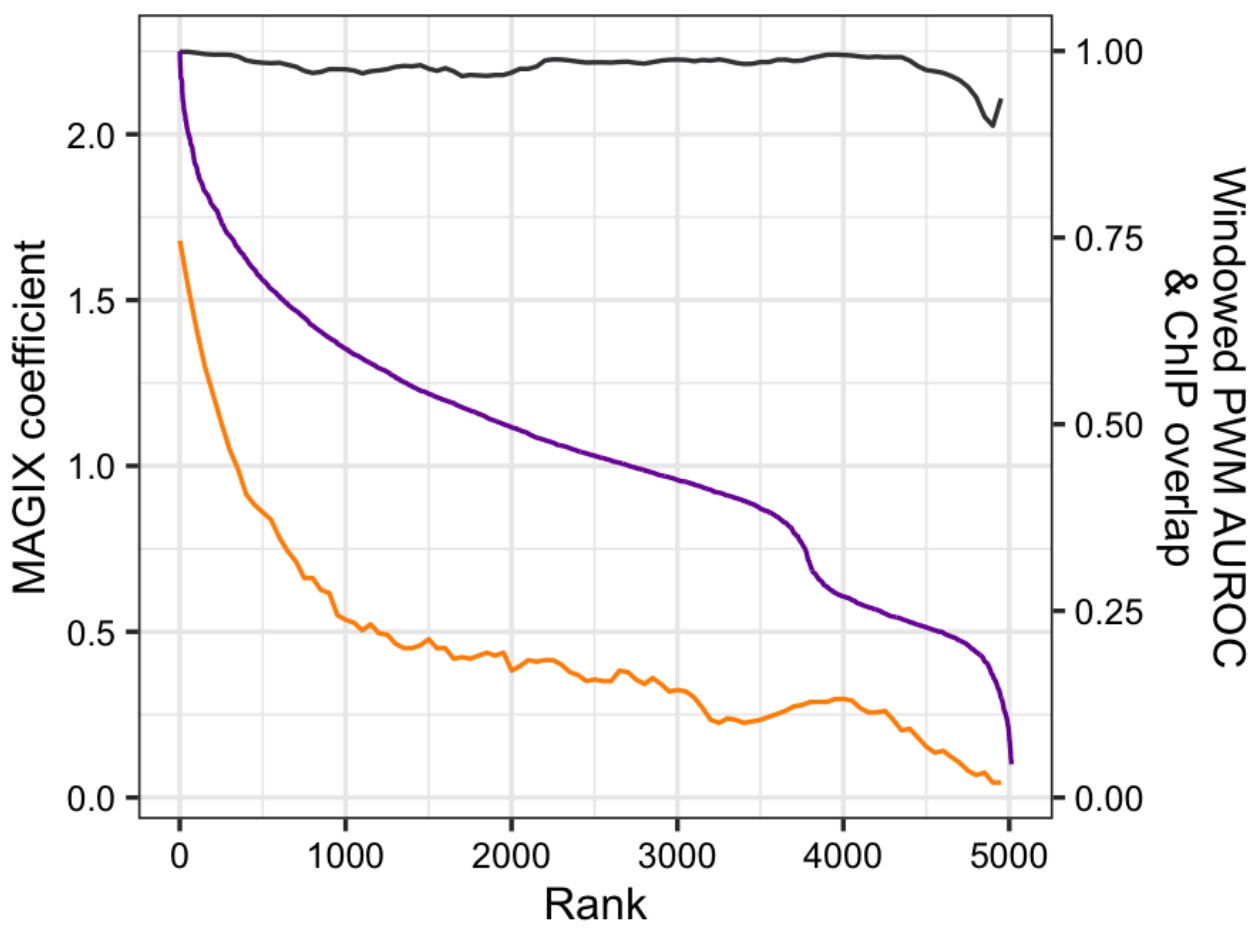

ZNF587B

Top GRECO-BIT/MEX PWM: PWM004785

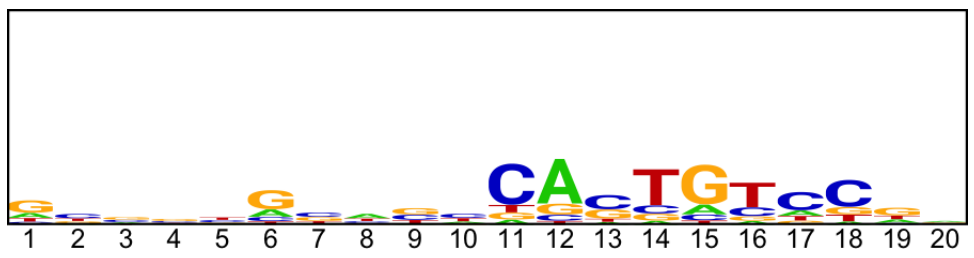

Location of top GRECO-BIT/MEX PWM in MAGIX peaks (up to the top 10,000)

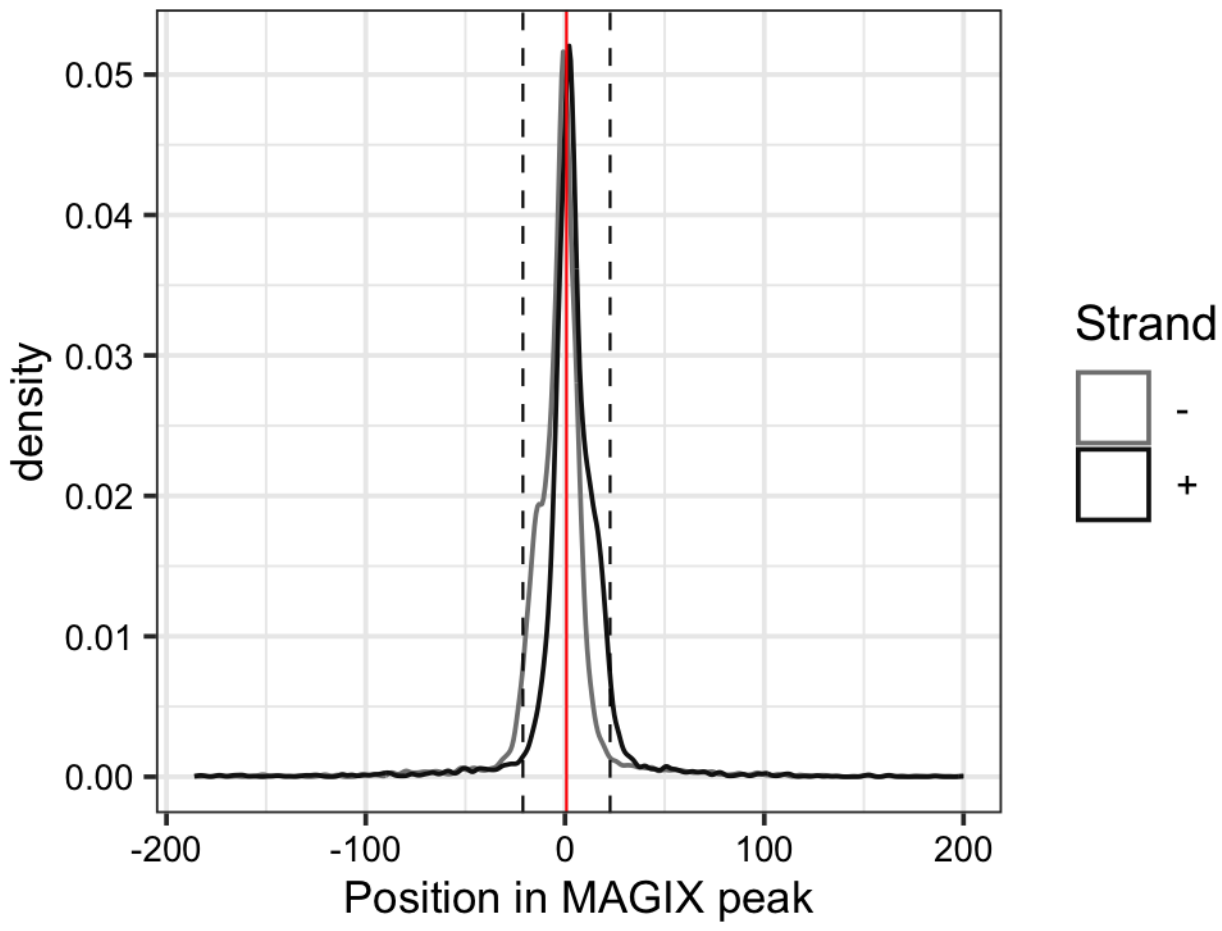

ZNF596

Top GRECO-BIT/MEX PWM: PWM004806

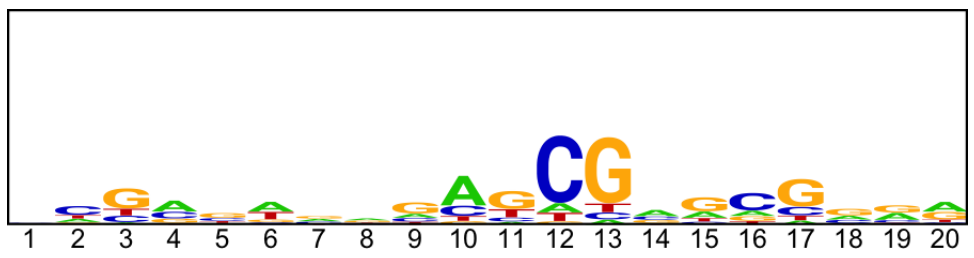

Location of top GRECO-BIT/MEX PWM in MAGIX peaks (up to the top 10,000)

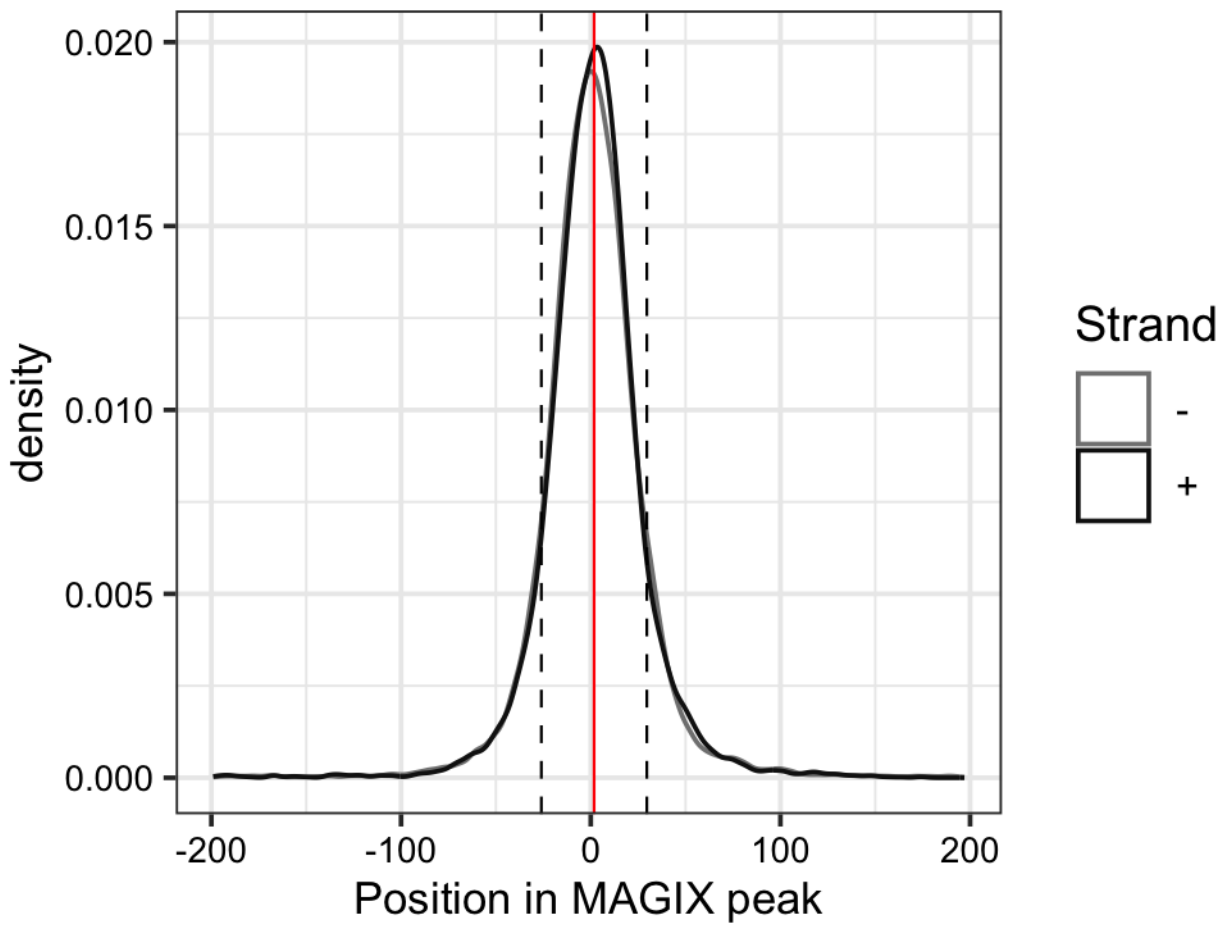

AUROC of top GRECO-BIT/MEX PWM in sliding windows (500 peaks, 50 peak step) along the MAGIX peaks (up to the top 75,000) ranked by the MAGIX enrichment coefficient and fraction of overlapping ChIP peaks in the same window

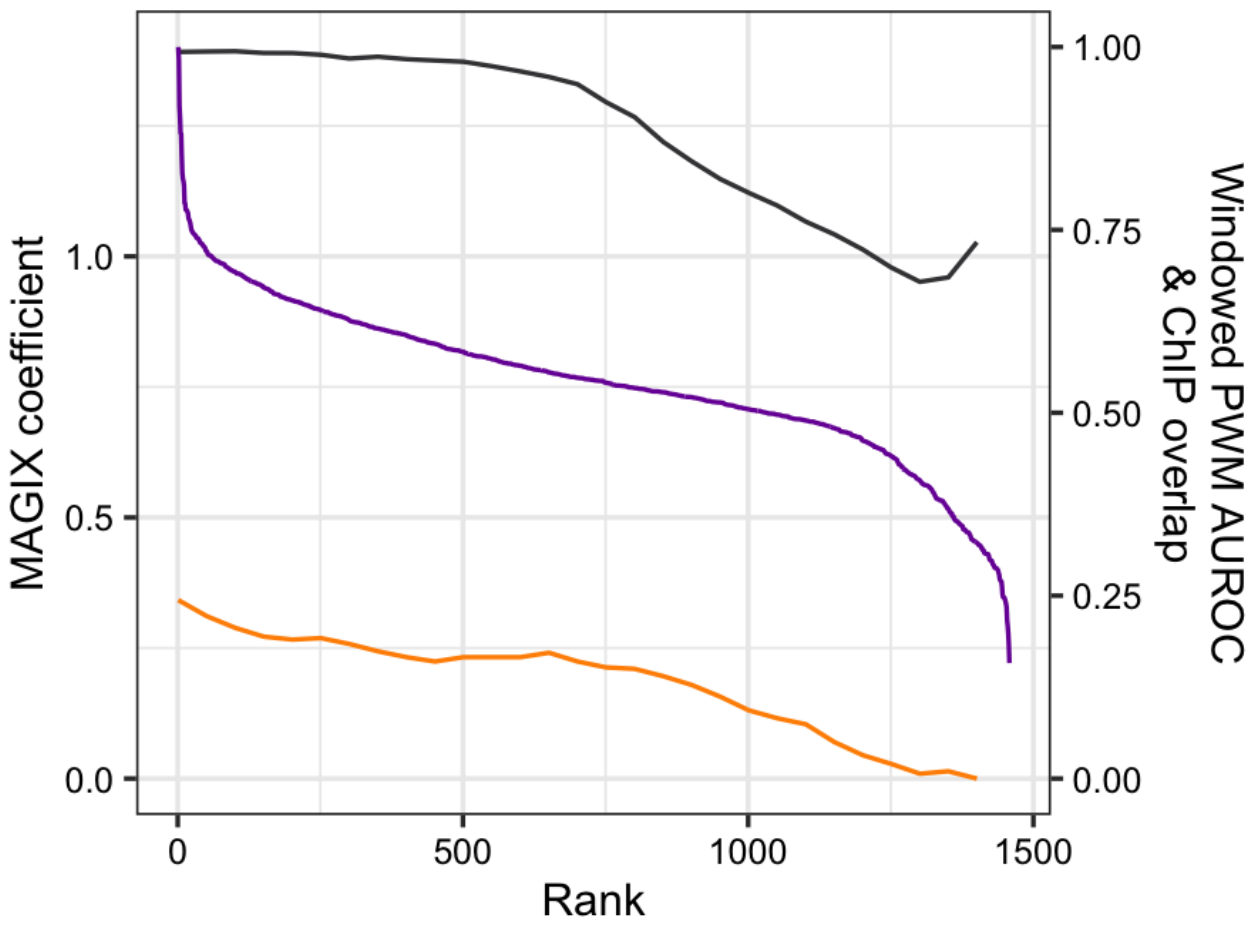

ZNF606

Top GRECO-BIT/MEX PWM: PWM004837

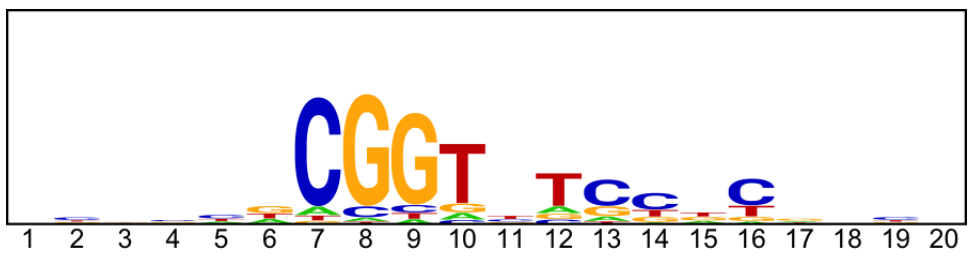

Location of top GRECO-BIT/MEX PWM in MAGIX peaks (up to the top 10,000)

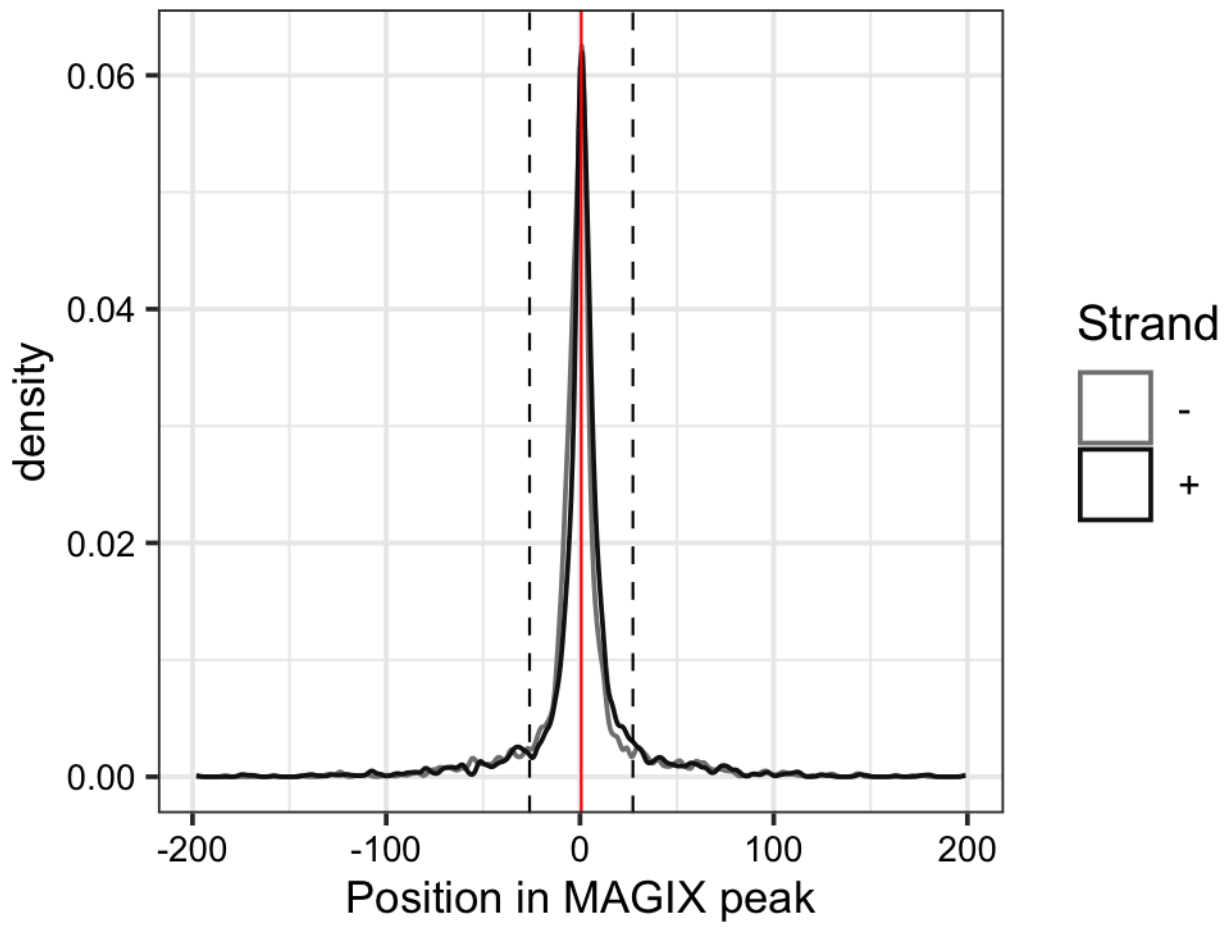

AUROC of top GRECO-BIT/MEX PWM in sliding windows (500 peaks, 50 peak step) along the MAGIX peaks (up to the top 75,000) ranked by the MAGIX enrichment coefficient and fraction of overlapping ChIP peaks in the same window

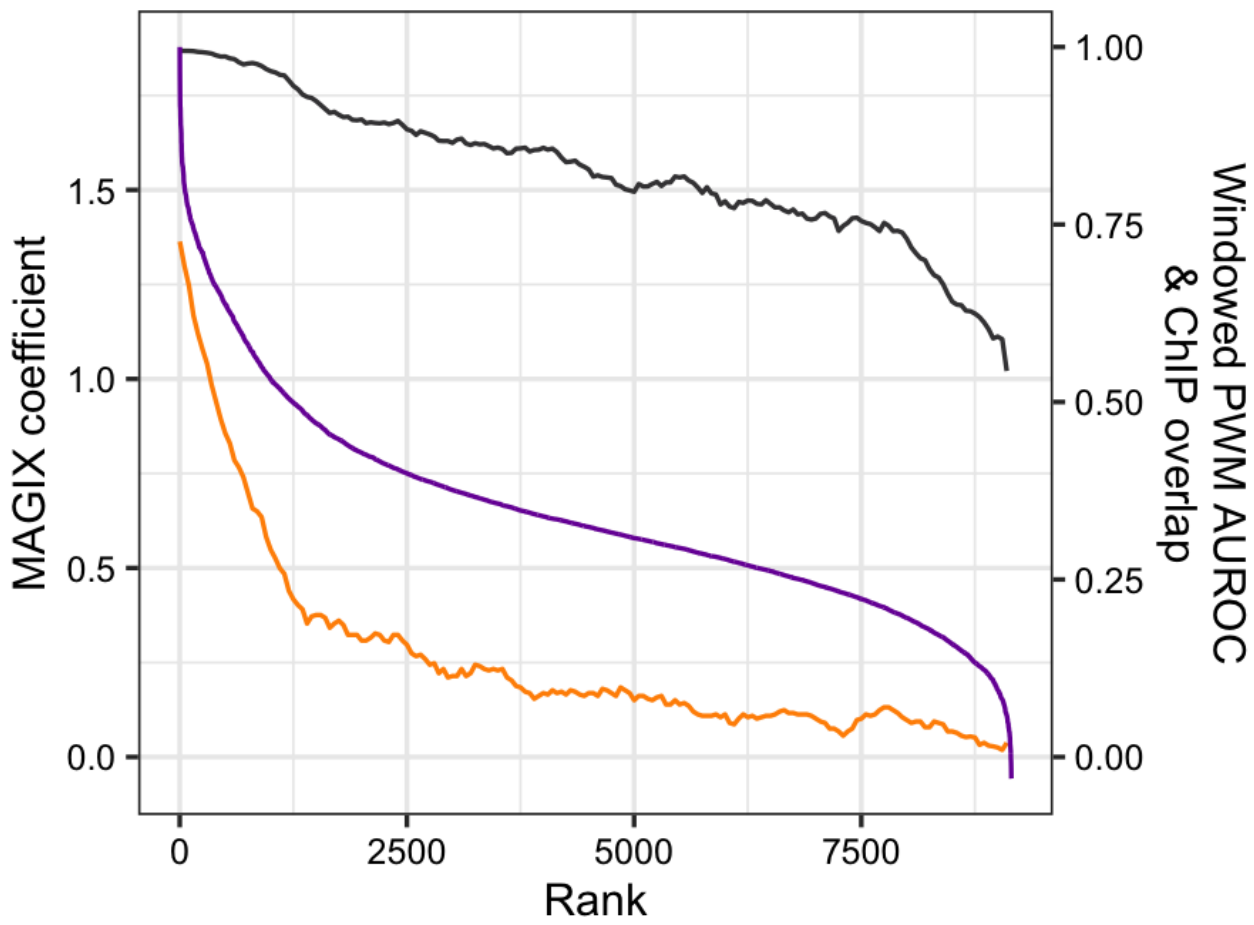

ZNF618

Top GRECO-BIT/MEX PWM: PWM004873

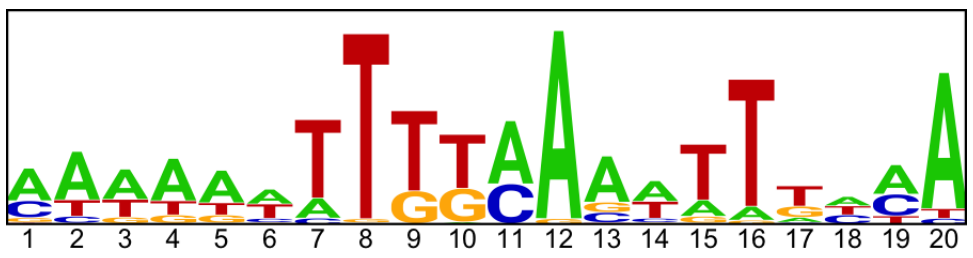

Location of top GRECO-BIT/MEX PWM in MAGIX peaks (up to the top 10,000)

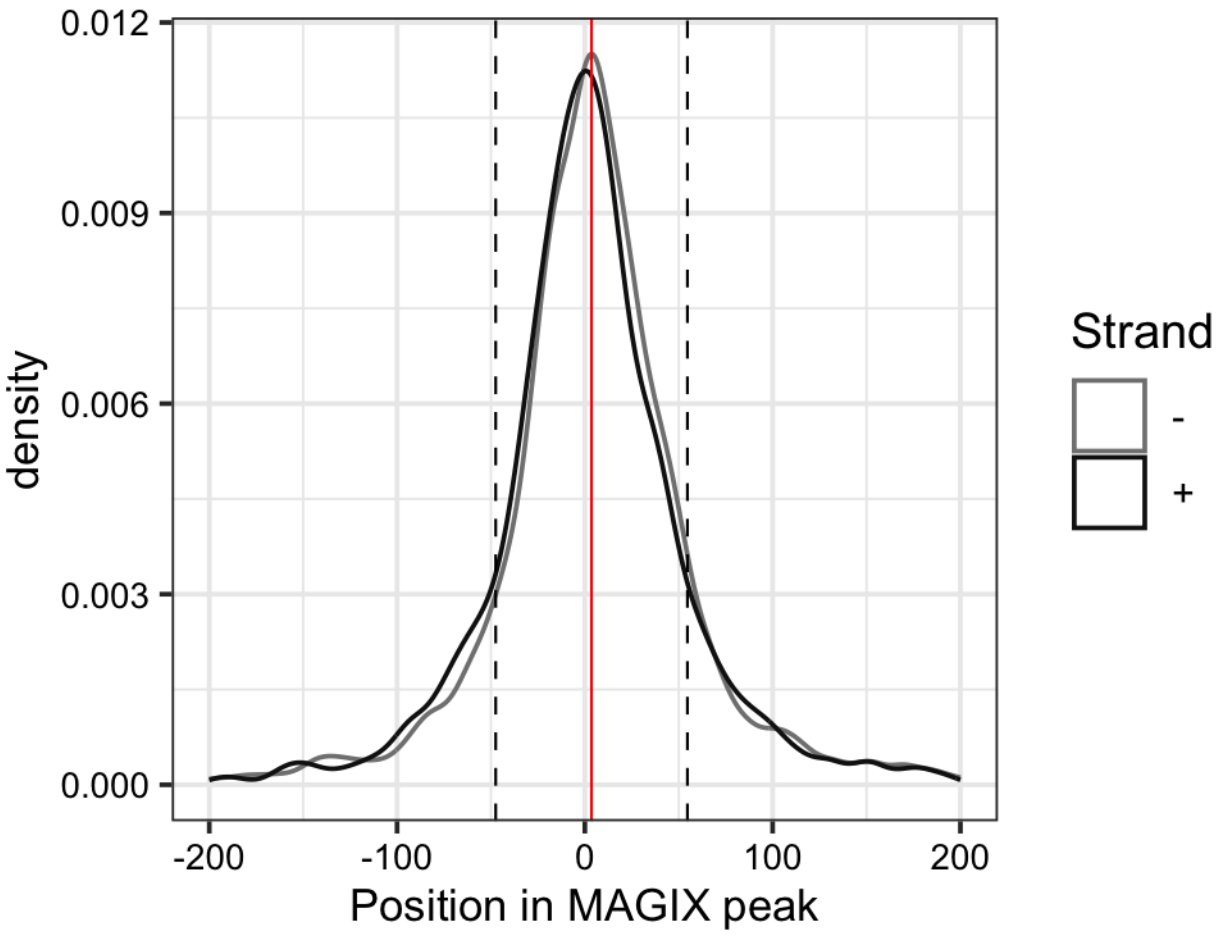

ZNF623

Top GRECO-BIT/MEX PWM: PWM004915

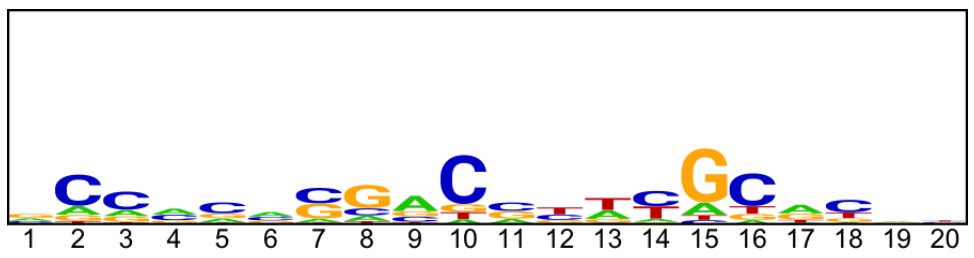

Location of top GRECO-BIT/MEX PWM in MAGIX peaks (up to the top 10,000)

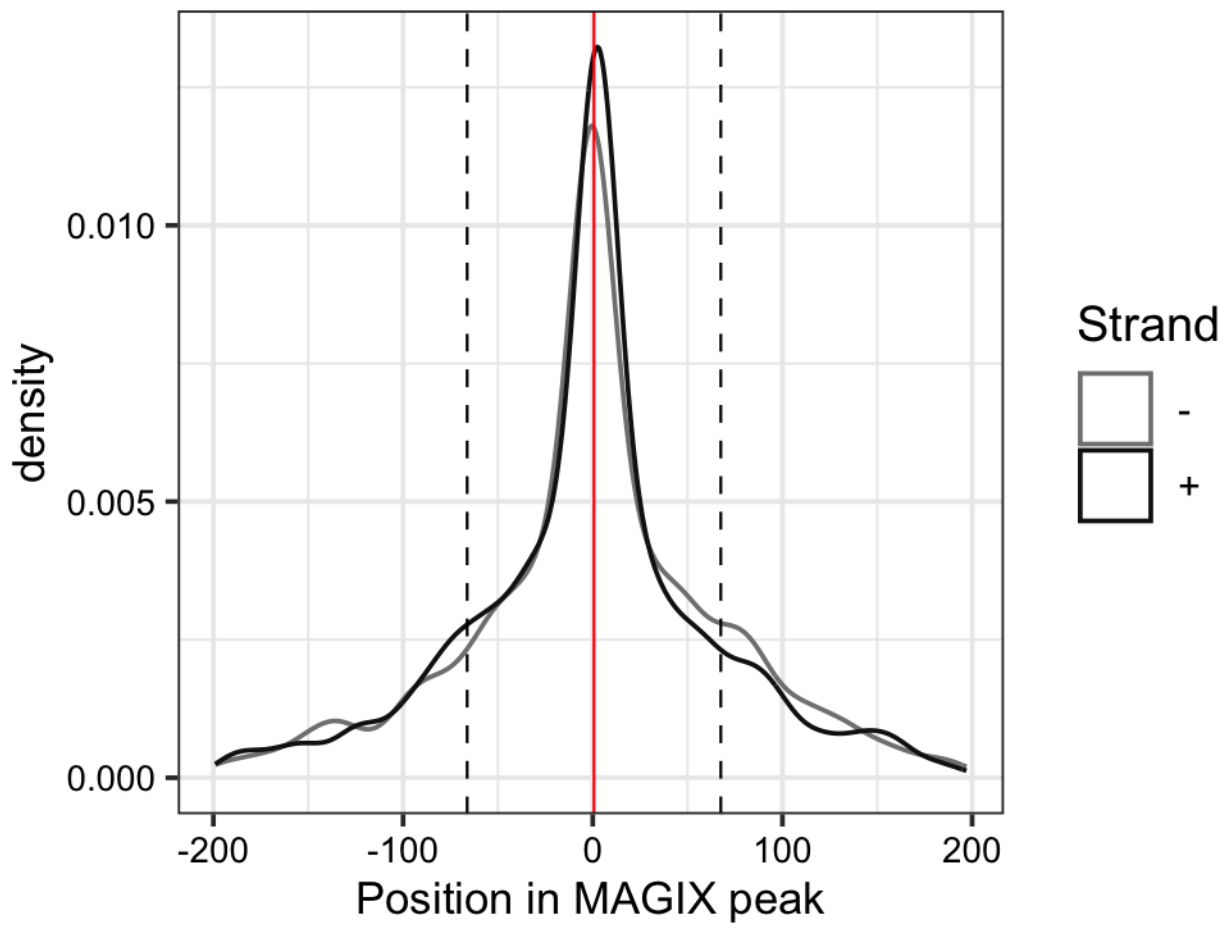

AUROC of top GRECO-BIT/MEX PWM in sliding windows (500 peaks, 50 peak step) along the MAGIX peaks (up to the top 75,000) ranked by the MAGIX enrichment coefficient and fraction of overlapping ChIP peaks in the same window

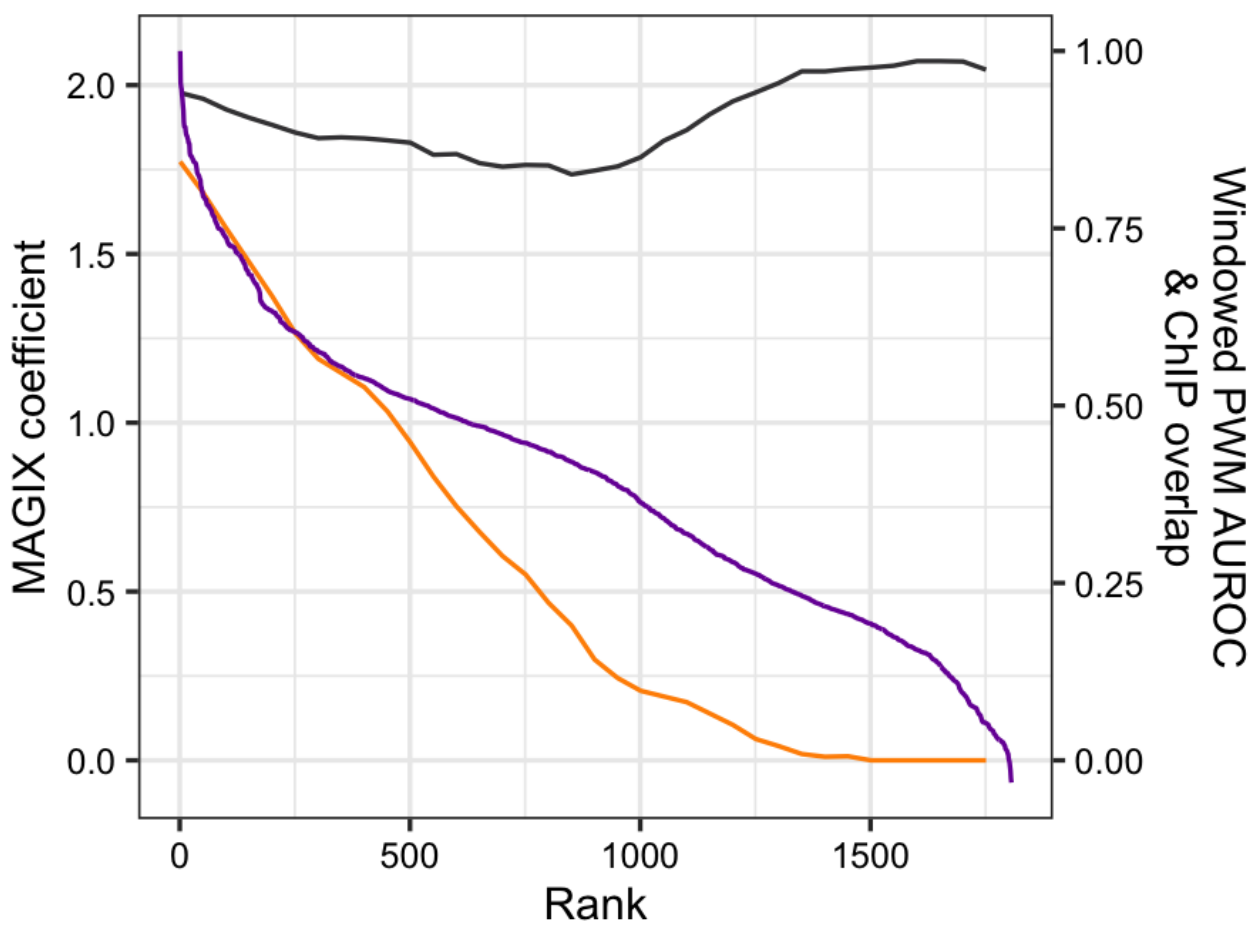

ZNF646.DBD1

Top GRECO-BIT/MEX PWM: PWM163370

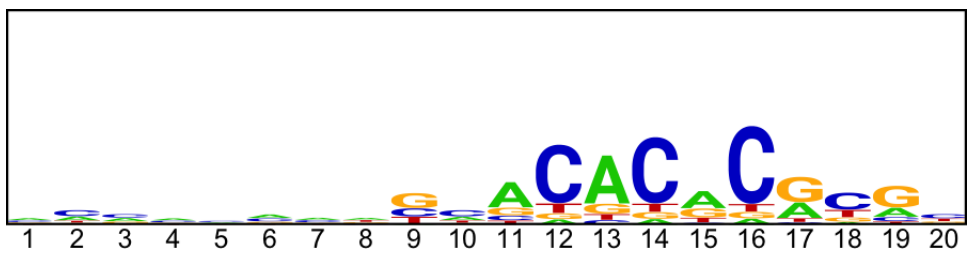

Location of top GRECO-BIT/MEX PWM in MAGIX peaks (up to the top 10,000)

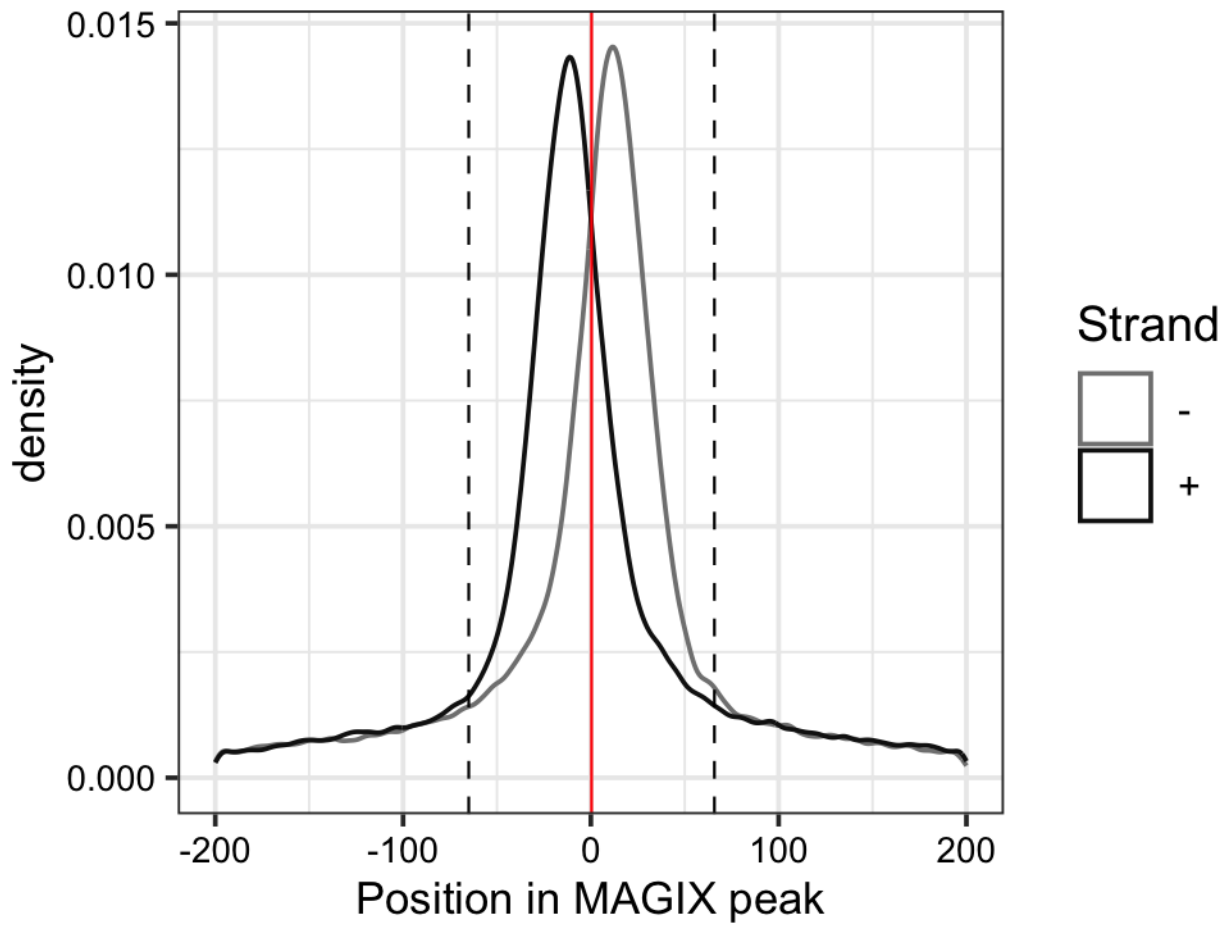

ZNF646.DBD2

Top GRECO-BIT/MEX PWM: PWM163643

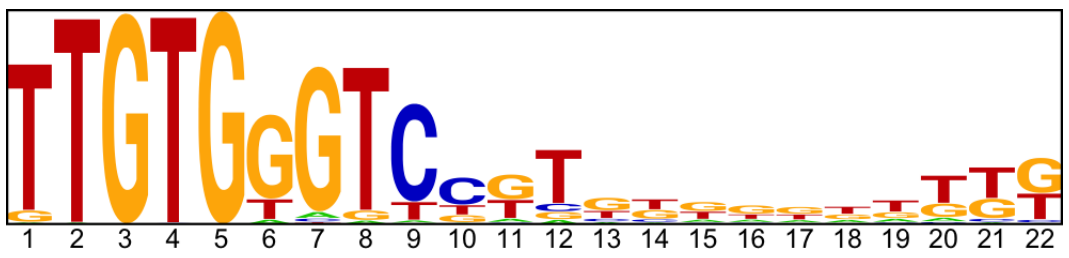

Location of top GRECO-BIT/MEX PWM in MAGIX peaks (up to the top 10,000)

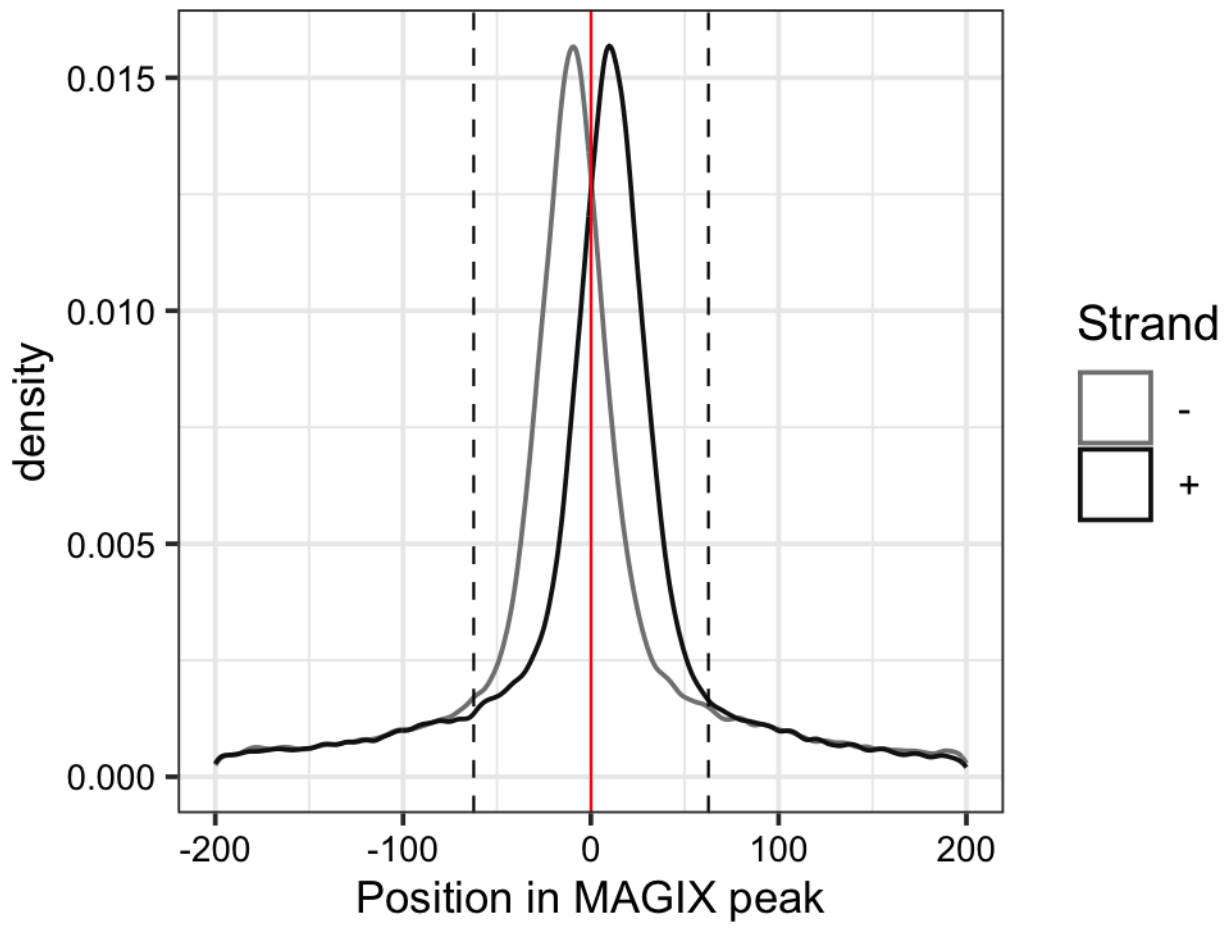

ZNF646.DBD3

Top GRECO-BIT/MEX PWM: PWM004958

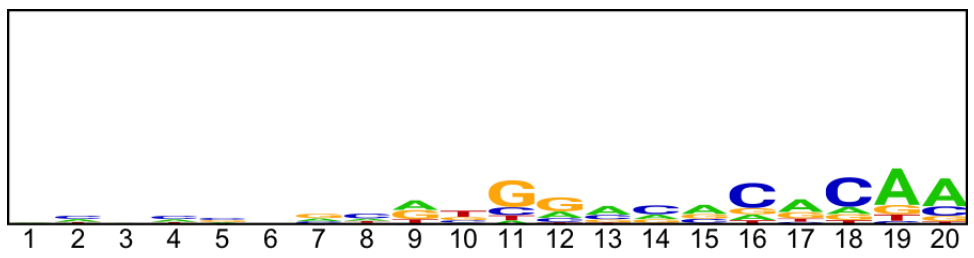

Location of top GRECO-BIT/MEX PWM in MAGIX peaks (up to the top 10,000)

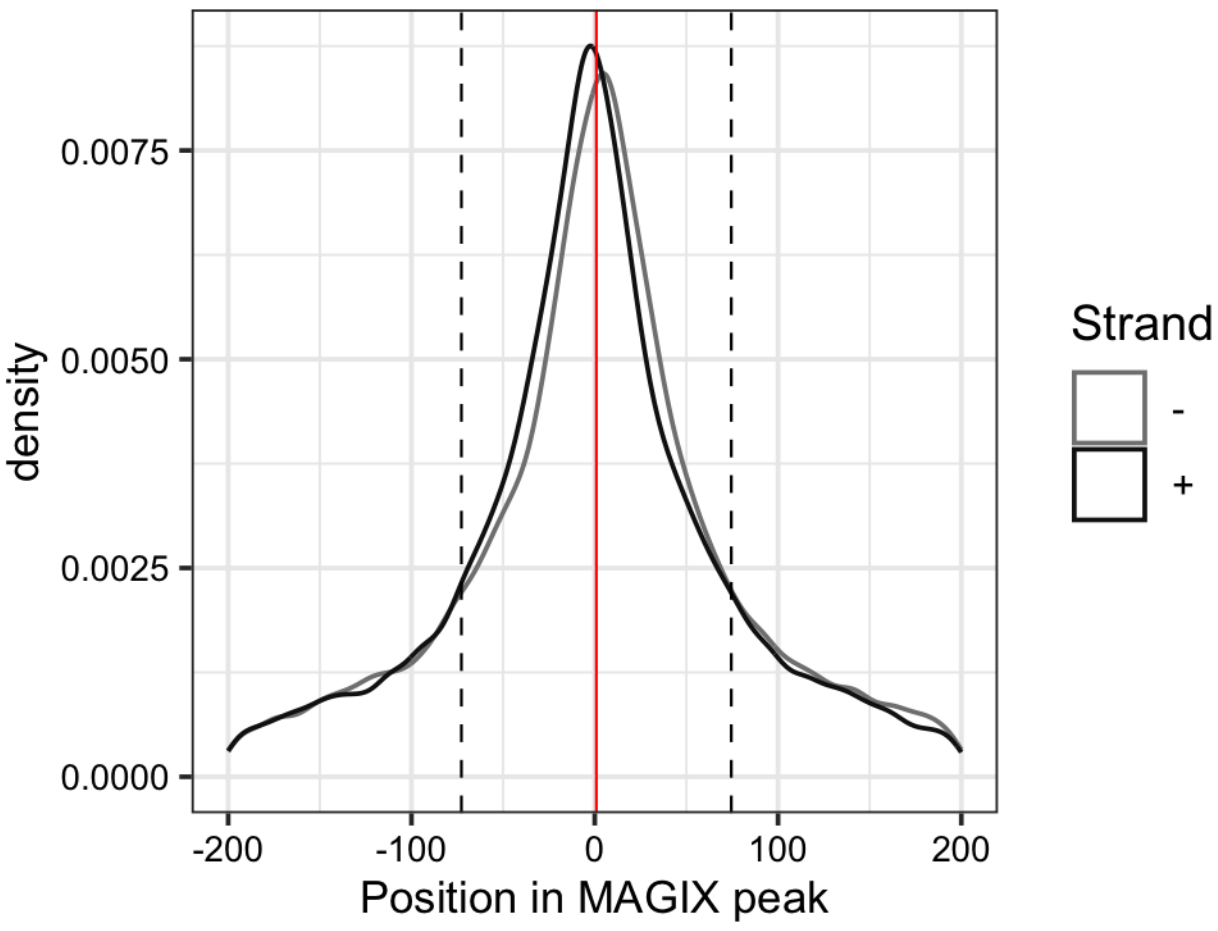

ZNF648

Top GRECO-BIT/MEX PWM: PWM004990

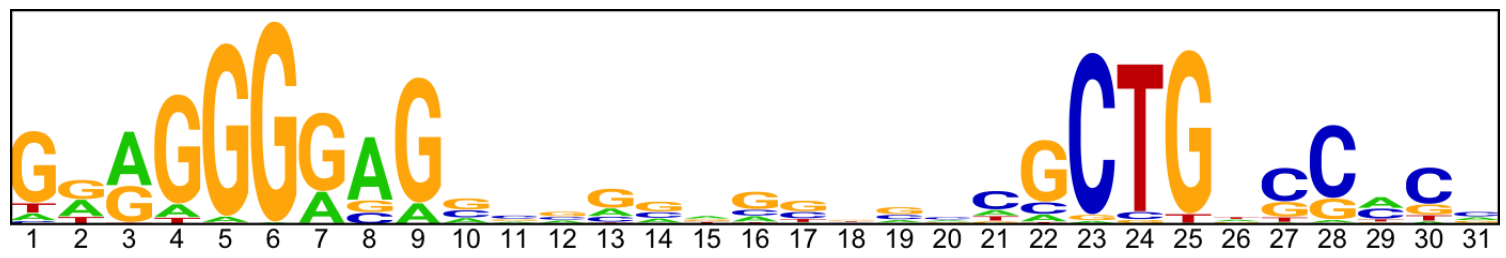

Location of top GRECO-BIT/MEX PWM in MAGIX peaks (up to the top 10,000)

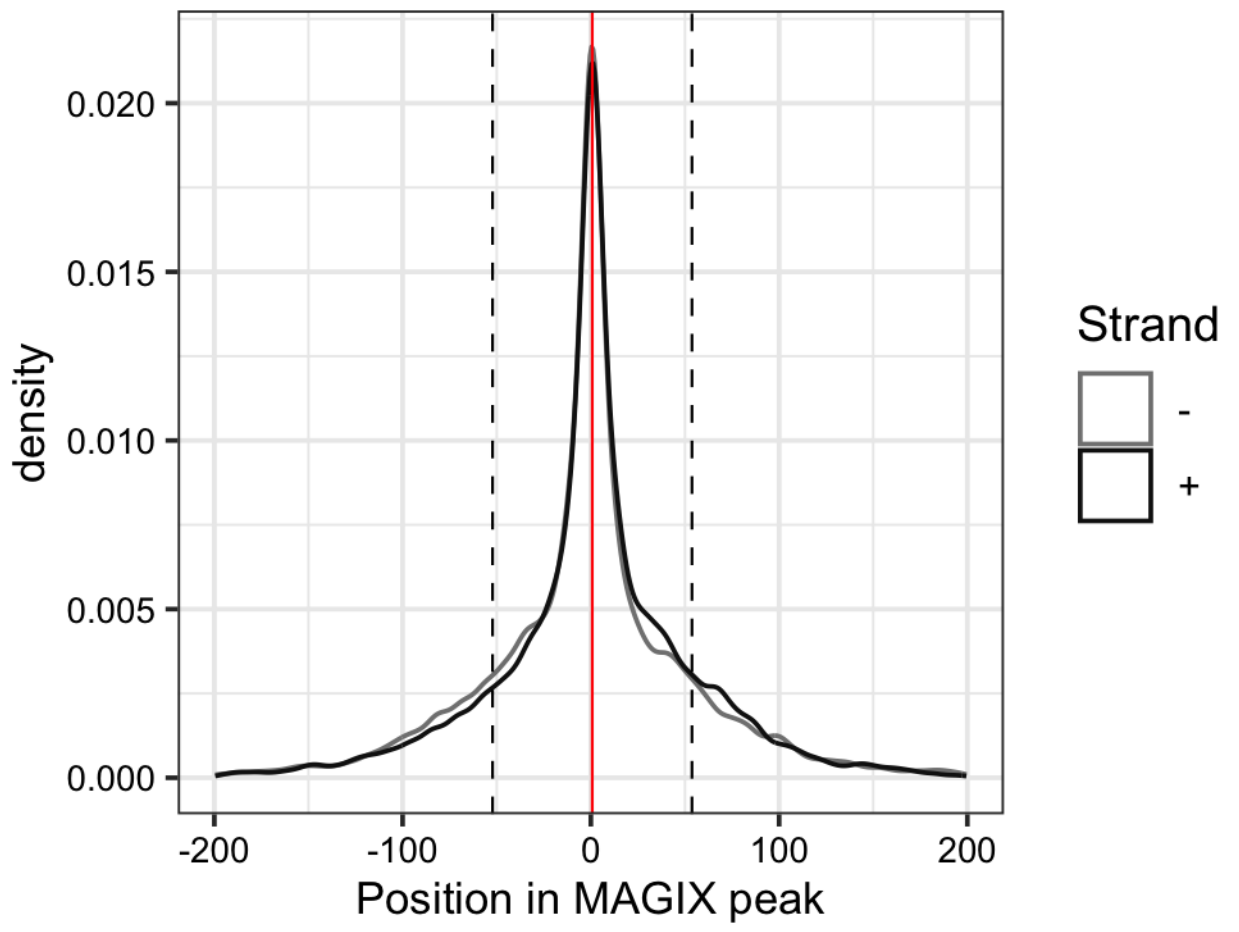

AUROC of top GRECO-BIT/MEX PWM in sliding windows (500 peaks, 50 peak step) along the MAGIX peaks (up to the top 75,000) ranked by the MAGIX enrichment coefficient and fraction of overlapping ChIP peaks in the same window

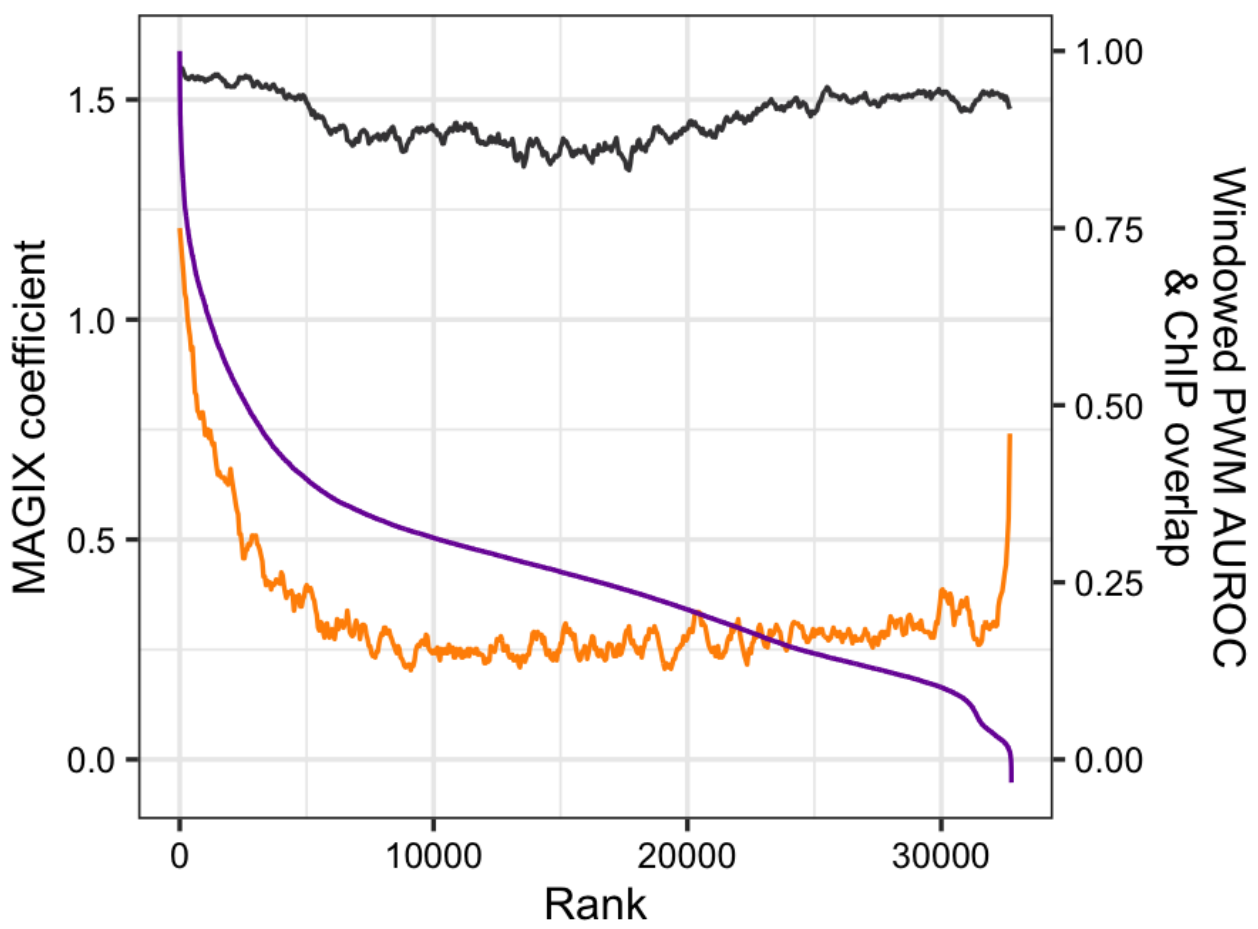

ZNF66

Top GRECO-BIT/MEX PWM: PWM005025

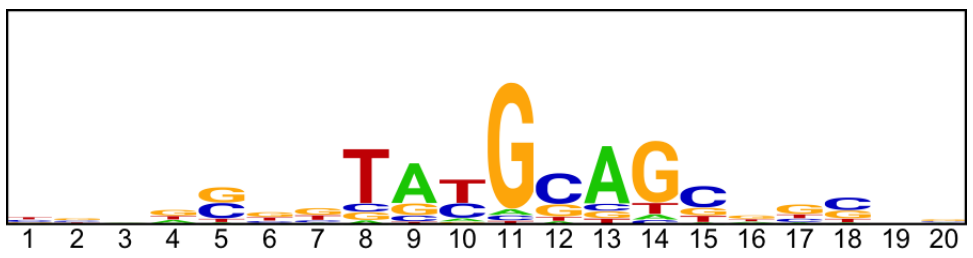

Location of top GRECO-BIT/MEX PWM in MAGIX peaks (up to the top 10,000)

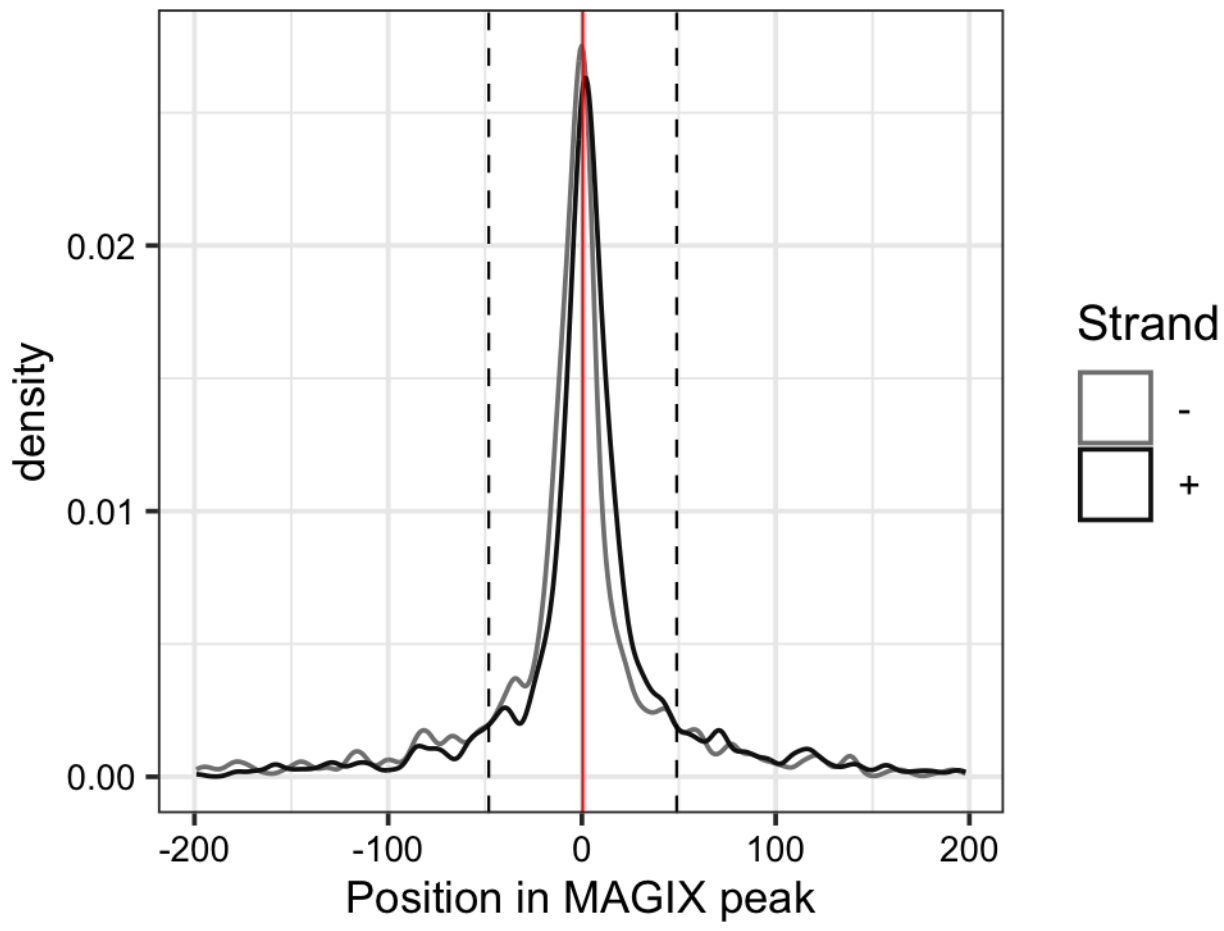

AUROC of top GRECO-BIT/MEX PWM in sliding windows (500 peaks, 50 peak step) along the MAGIX peaks (up to the top 75,000) ranked by the MAGIX enrichment coefficient and fraction of overlapping ChIP peaks in the same window

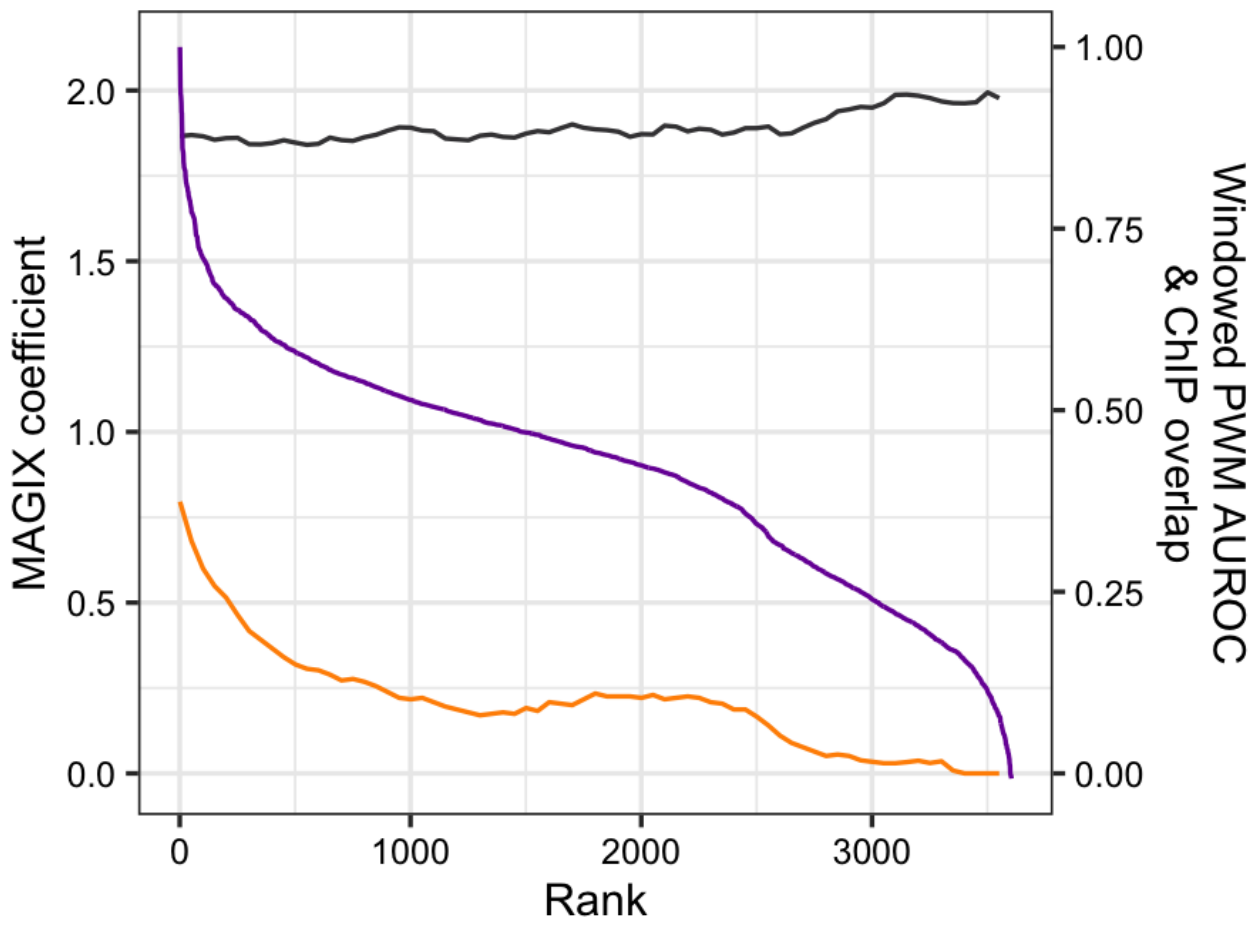

ZNF665

Top GRECO-BIT/MEX PWM: PWM005065

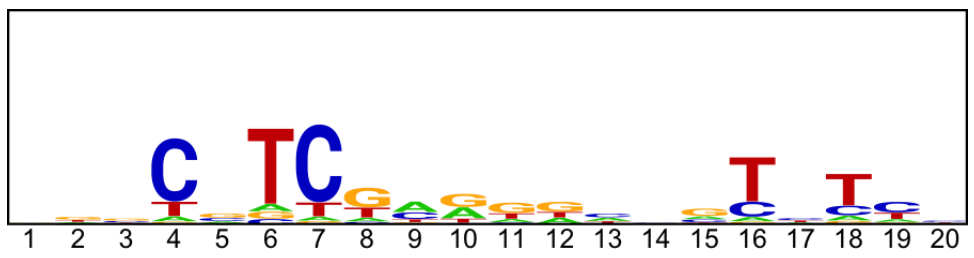

Location of top GRECO-BIT/MEX PWM in MAGIX peaks (up to the top 10,000)

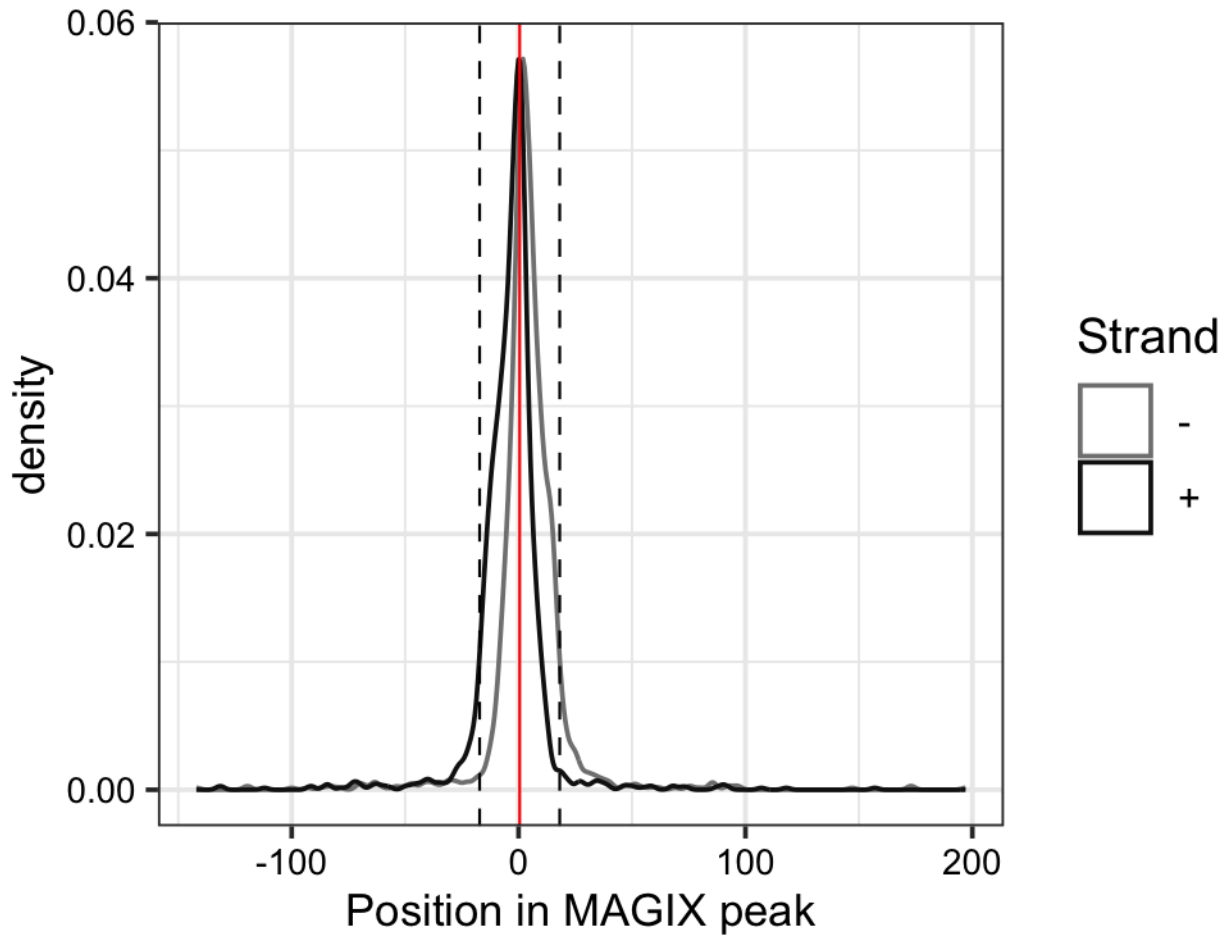

AUROC of top GRECO-BIT/MEX PWM in sliding windows (500 peaks, 50 peak step) along the MAGIX peaks (up to the top 75,000) ranked by the MAGIX enrichment coefficient and fraction of overlapping ChIP peaks in the same window

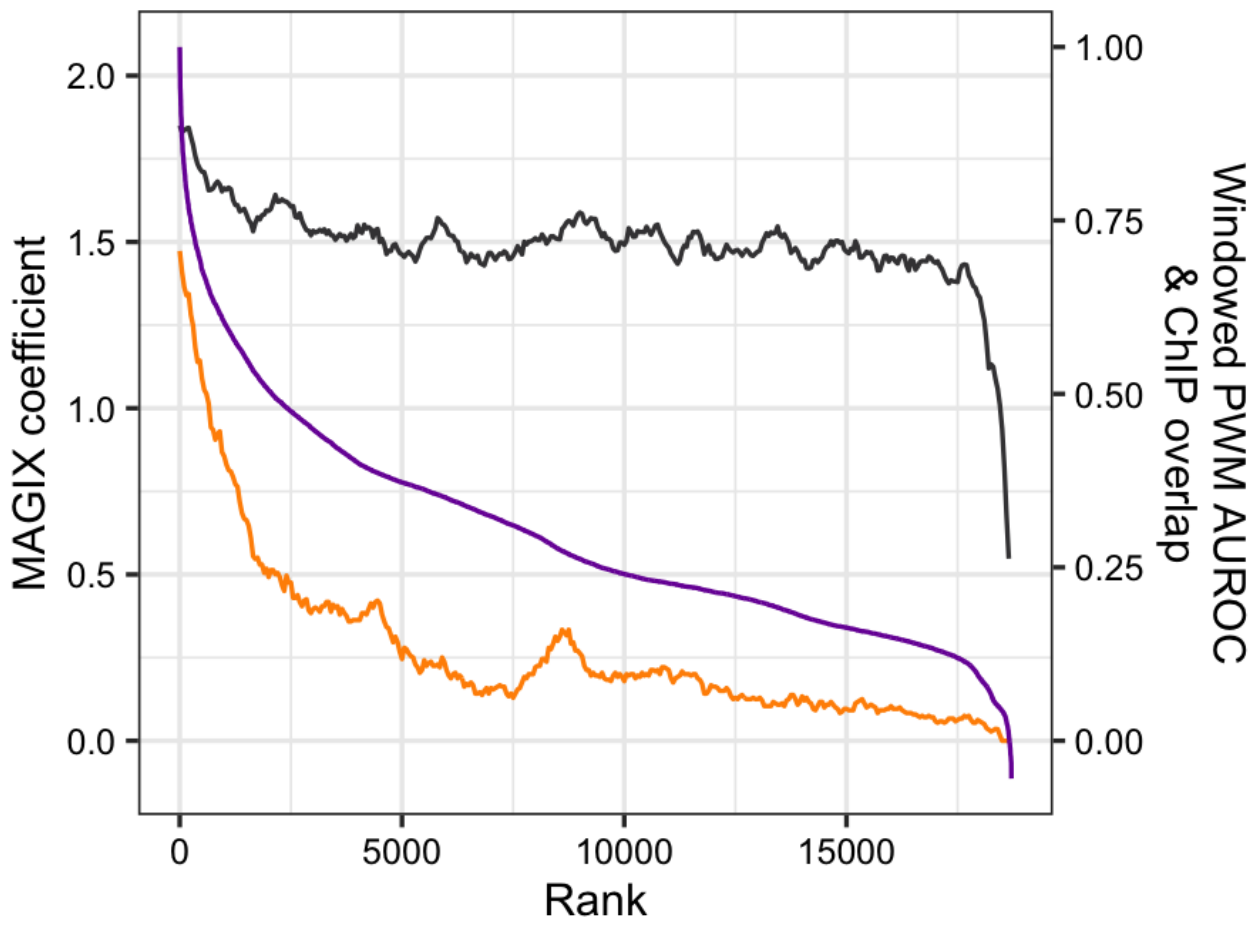

ZNF668.DBD1

Top GRECO-BIT/MEX PWM: PWM005082

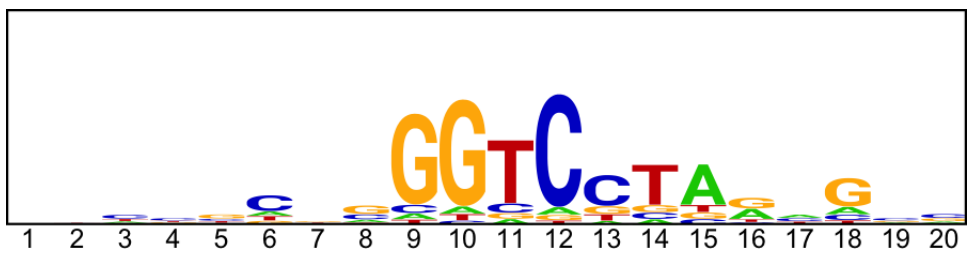

Location of top GRECO-BIT/MEX PWM in MAGIX peaks (up to the top 10,000)

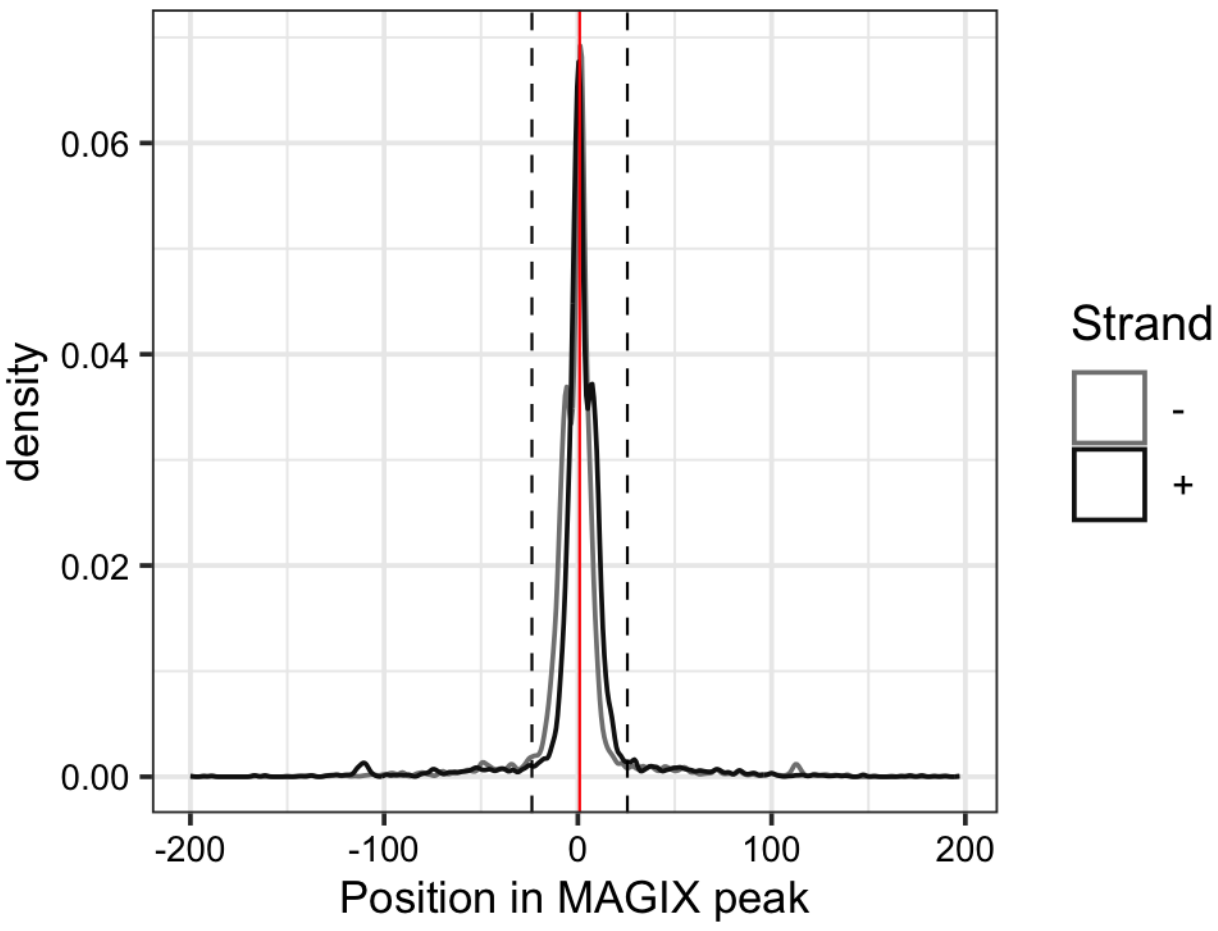

ZNF668.FL

Top GRECO-BIT/MEX PWM: PWM005084

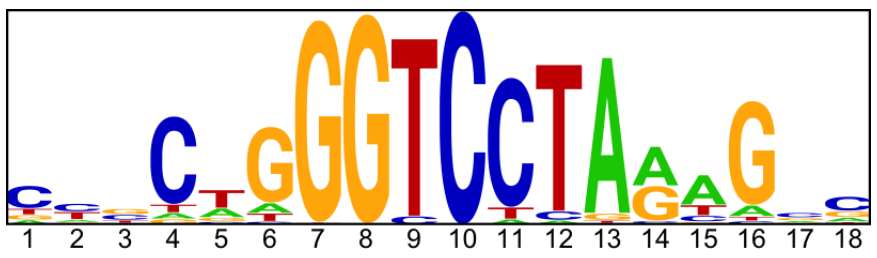

Location of top GRECO-BIT/MEX PWM in MAGIX peaks (up to the top 10,000)

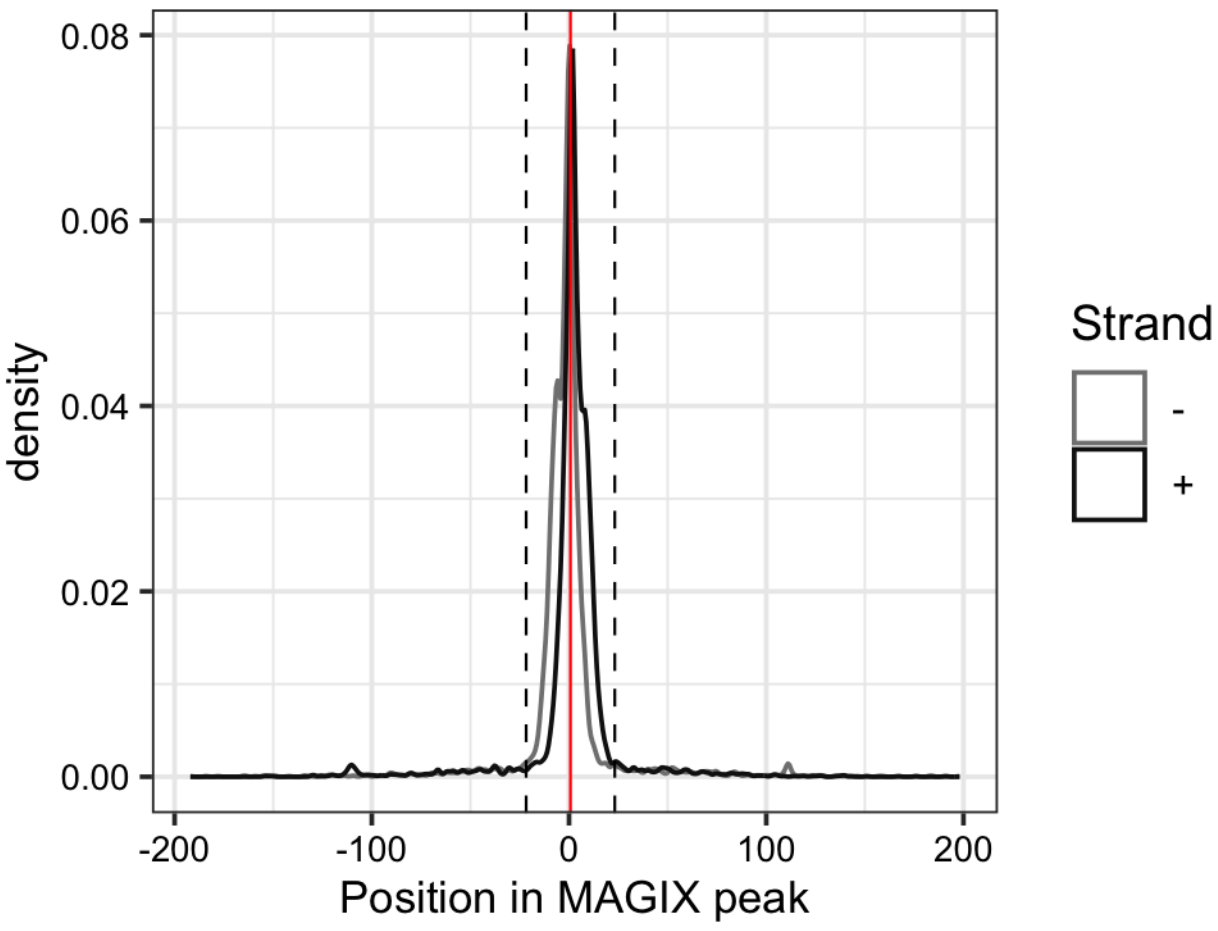

ZNF672

Top GRECO-BIT/MEX PWM: PWM005120

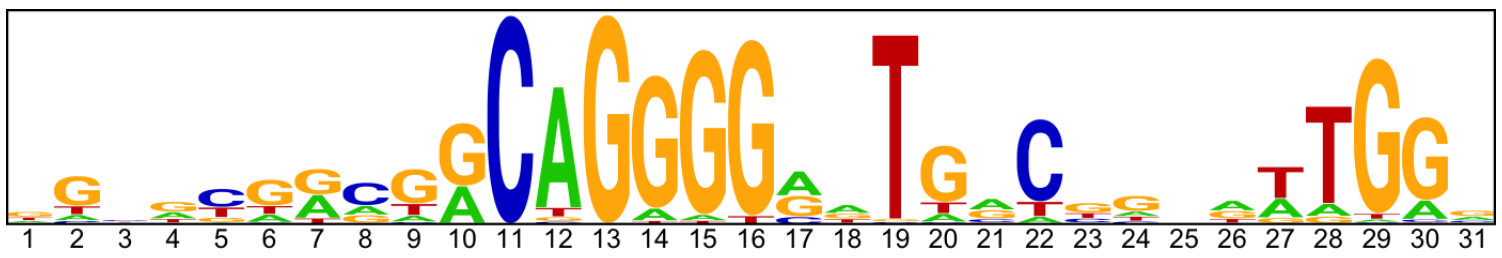

Location of top GRECO-BIT/MEX PWM in MAGIX peaks (up to the top 10,000)

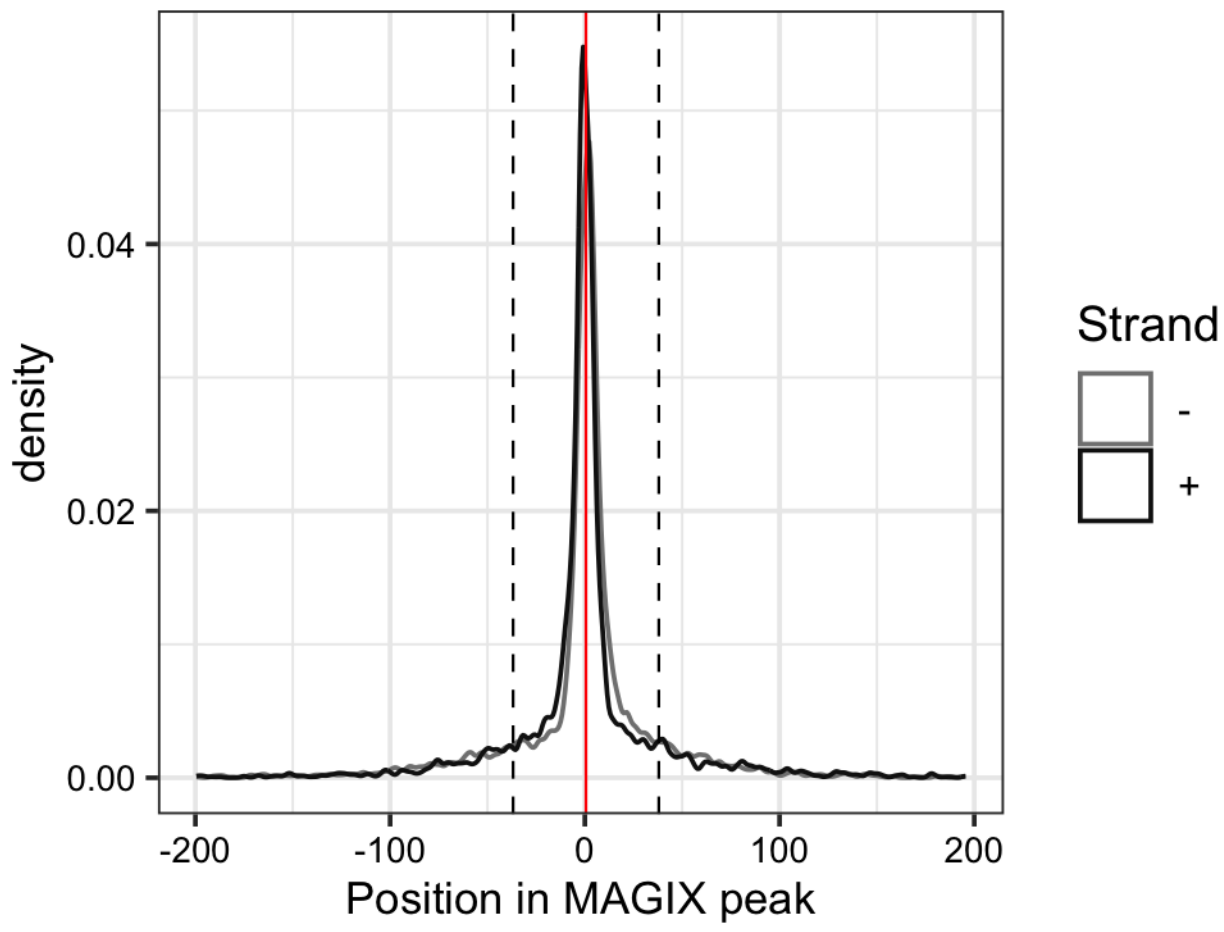

AUROC of top GRECO-BIT/MEX PWM in sliding windows (500 peaks, 50 peak step) along the MAGIX peaks (up to the top 75,000) ranked by the MAGIX enrichment coefficient and fraction of overlapping ChIP peaks in the same window

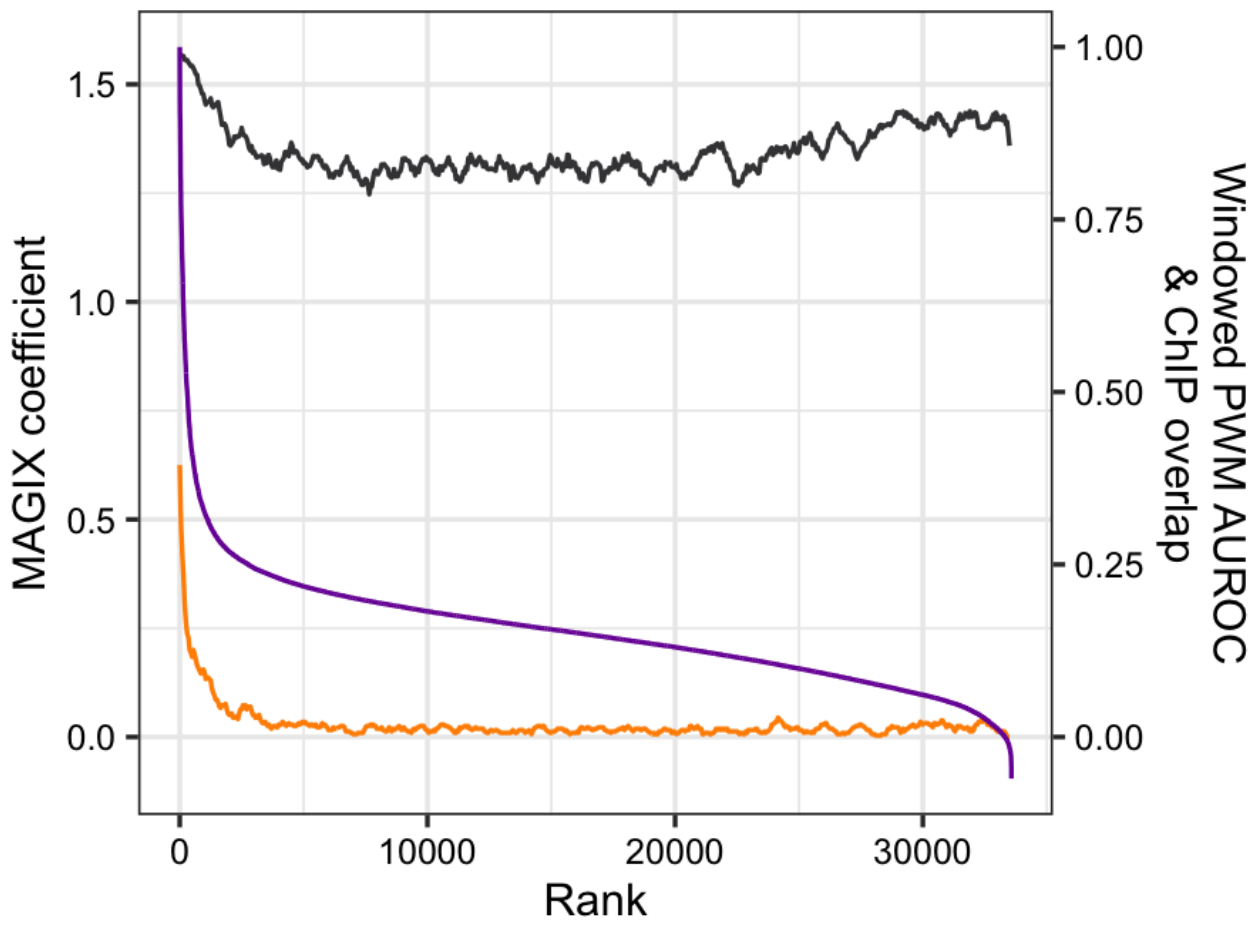

ZNF676

Top GRECO-BIT/MEX PWM: PWM005154

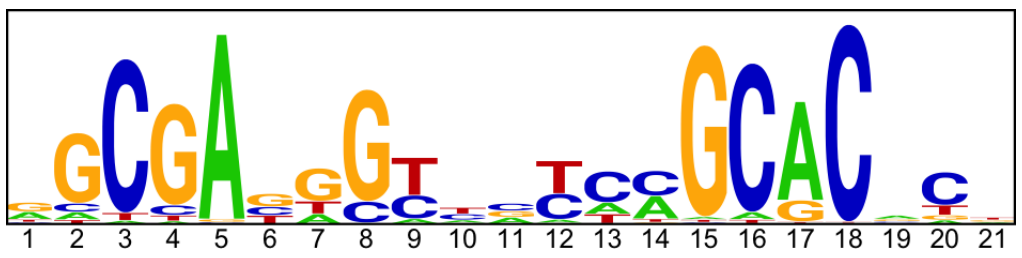

Location of top GRECO-BIT/MEX PWM in MAGIX peaks (up to the top 10,000)

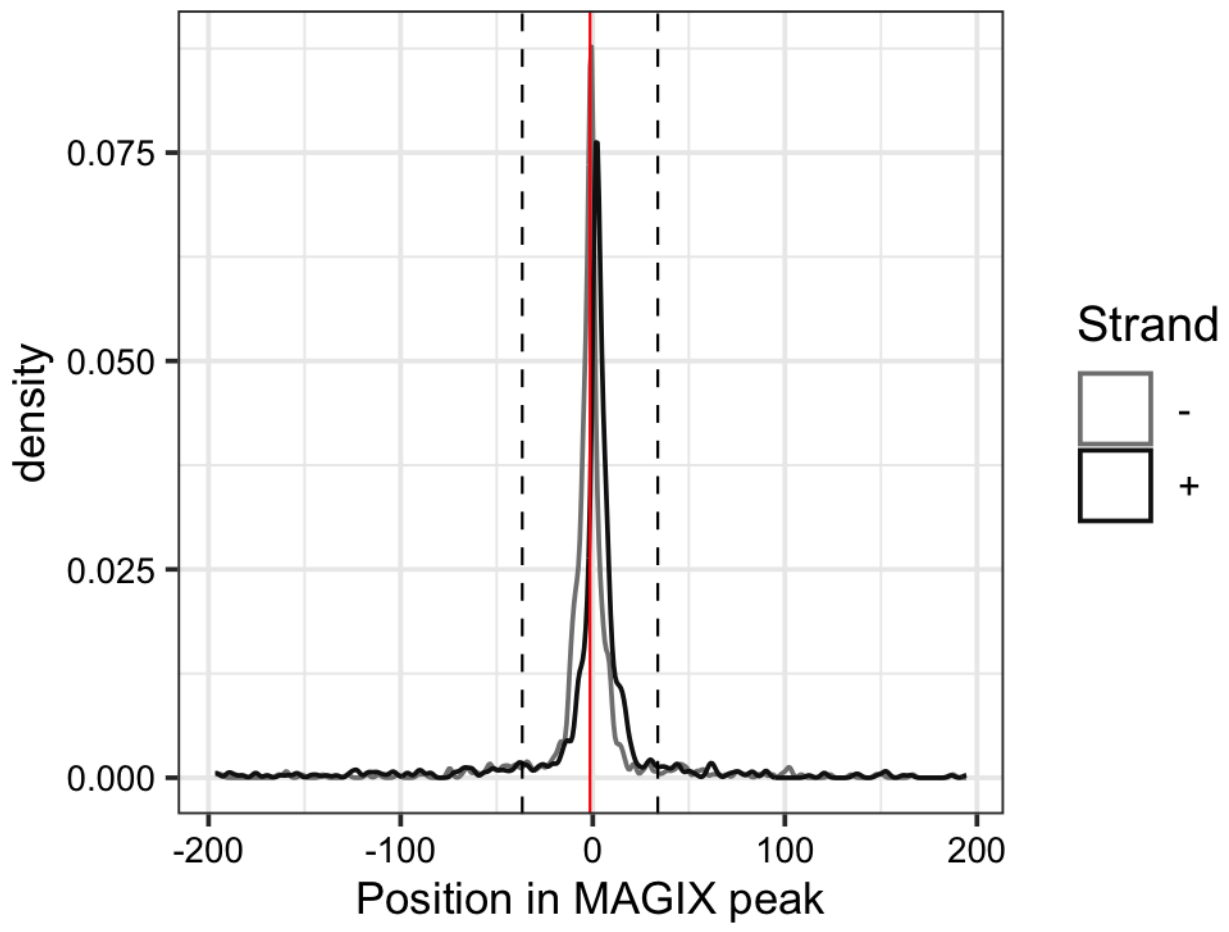

AUROC of top GRECO-BIT/MEX PWM in sliding windows (500 peaks, 50 peak step) along the MAGIX peaks (up to the top 75,000) ranked by the MAGIX enrichment coefficient and fraction of overlapping ChIP peaks in the same window

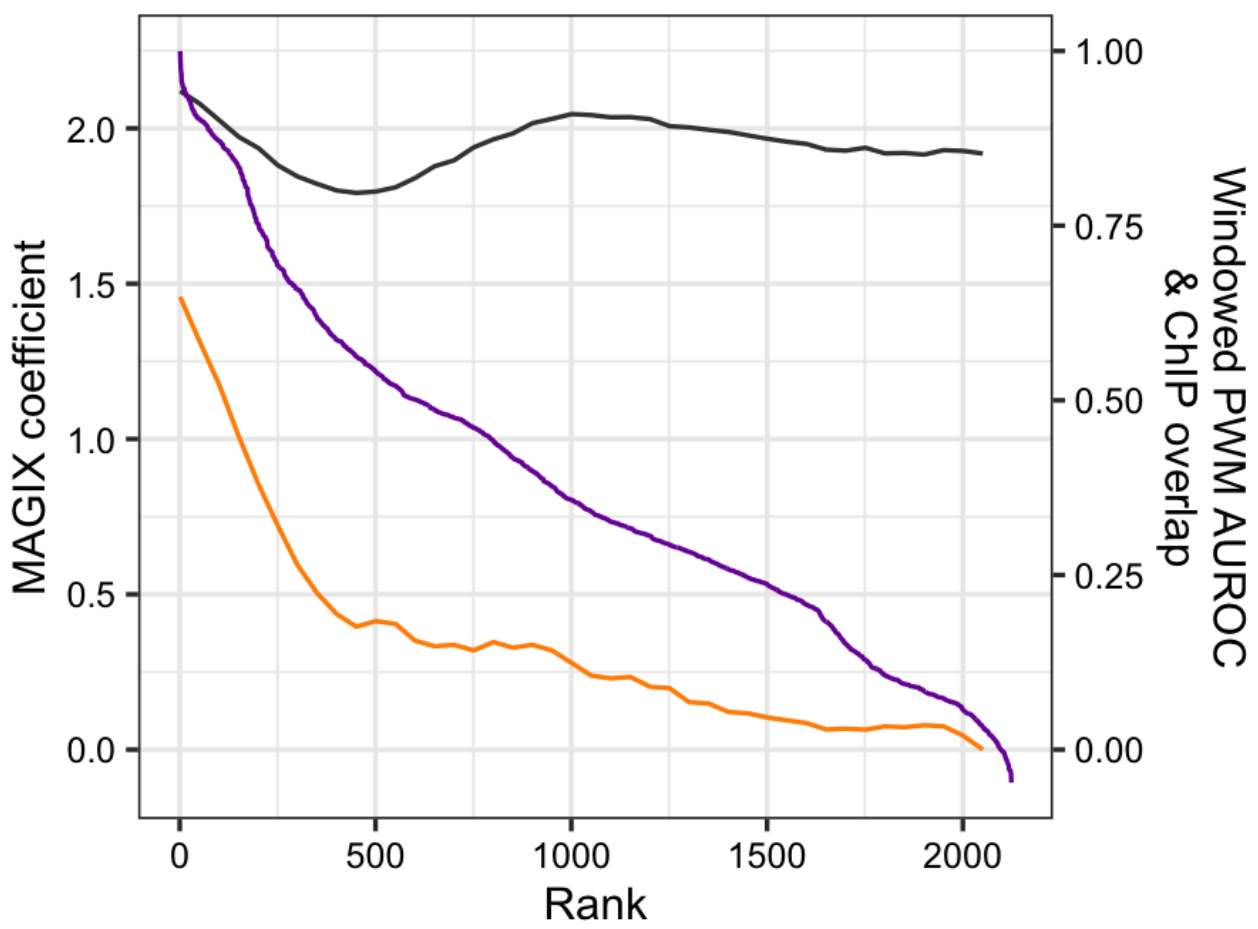

ZNF678

Top GRECO-BIT/MEX PWM: PWM005176

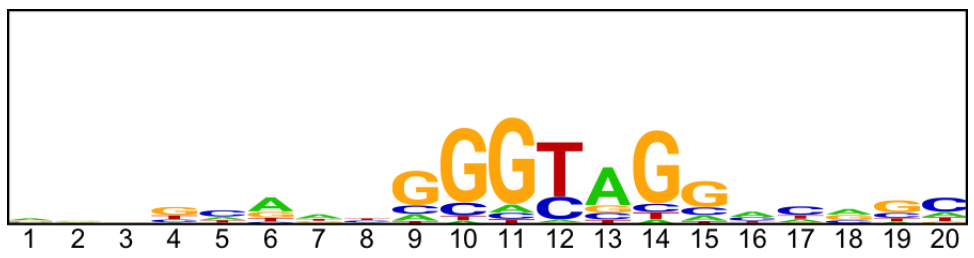

Location of top GRECO-BIT/MEX PWM in MAGIX peaks (up to the top 10,000)

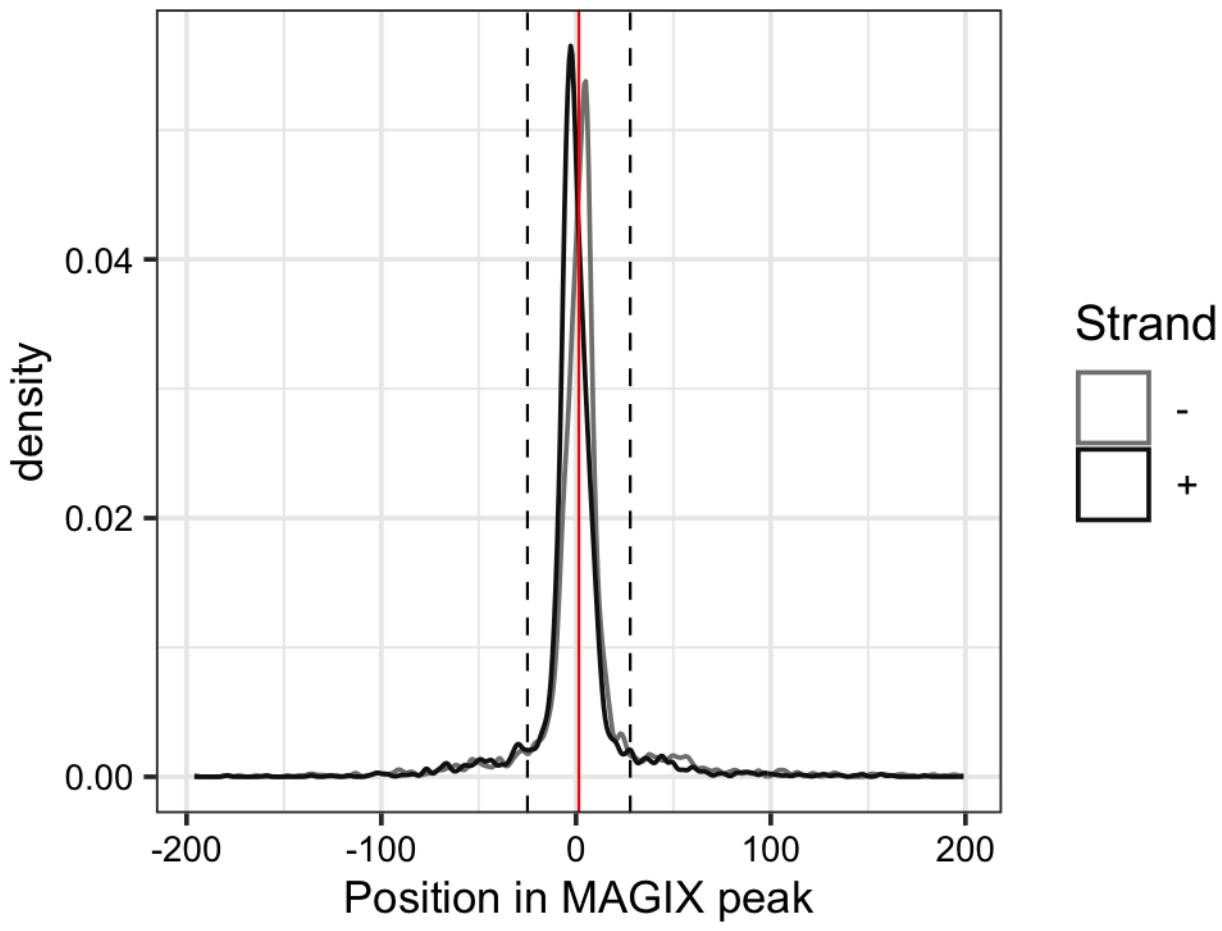

AUROC of top GRECO-BIT/MEX PWM in sliding windows (500 peaks, 50 peak step) along the MAGIX peaks (up to the top 75,000) ranked by the MAGIX enrichment coefficient and fraction of overlapping ChIP peaks in the same window

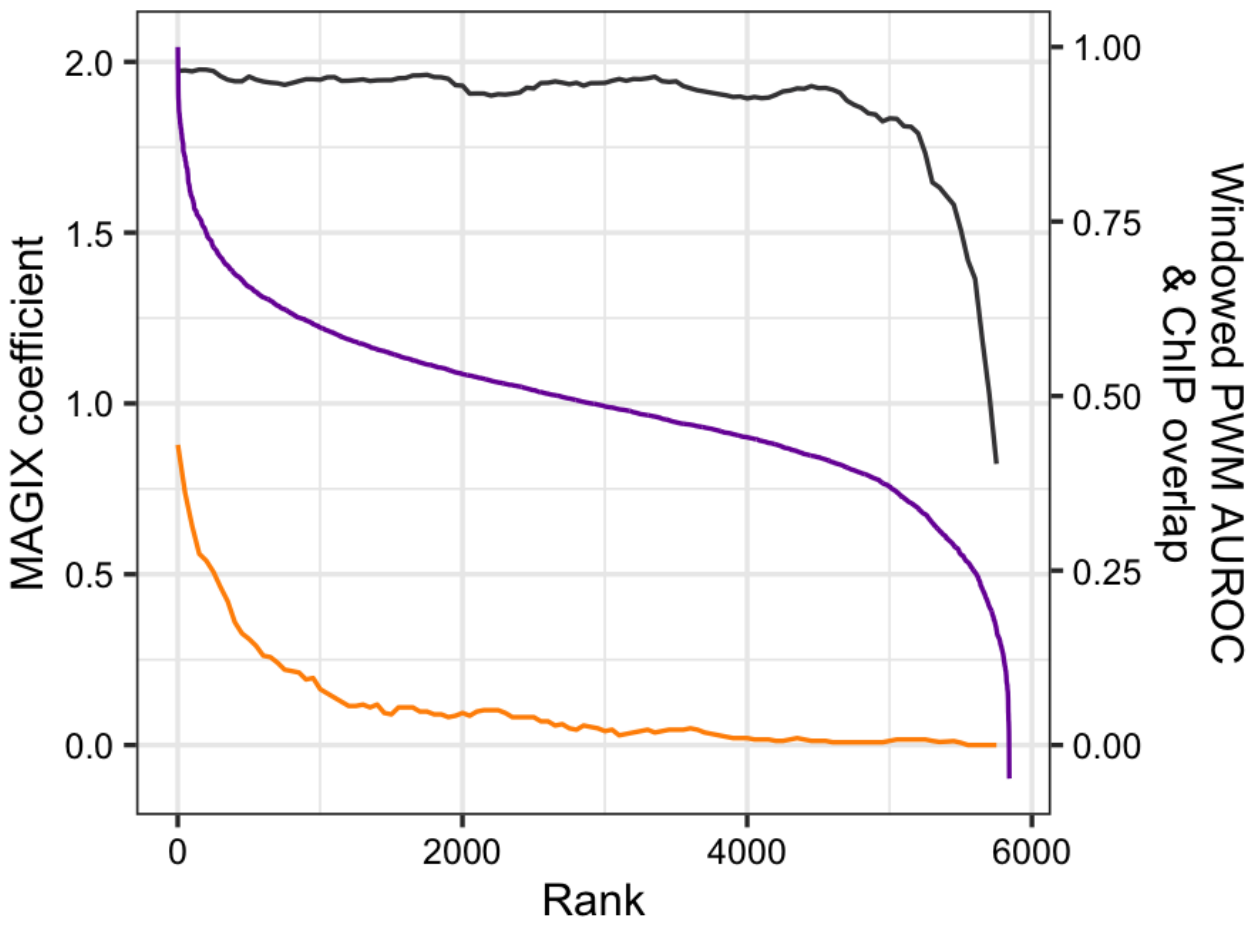

ZNF683

Top GRECO-BIT/MEX PWM: PWM005199

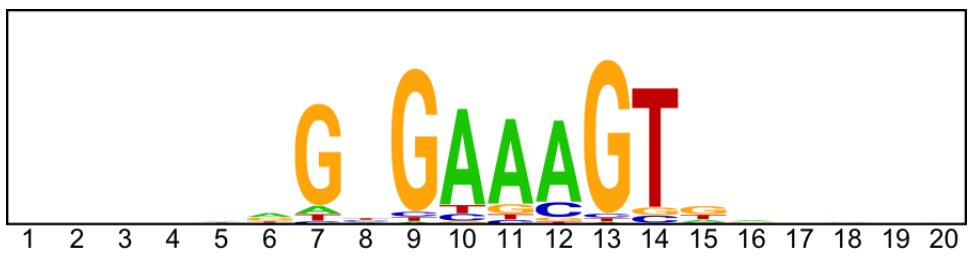

Location of top GRECO-BIT/MEX PWM in MAGIX peaks (up to the top 10,000)

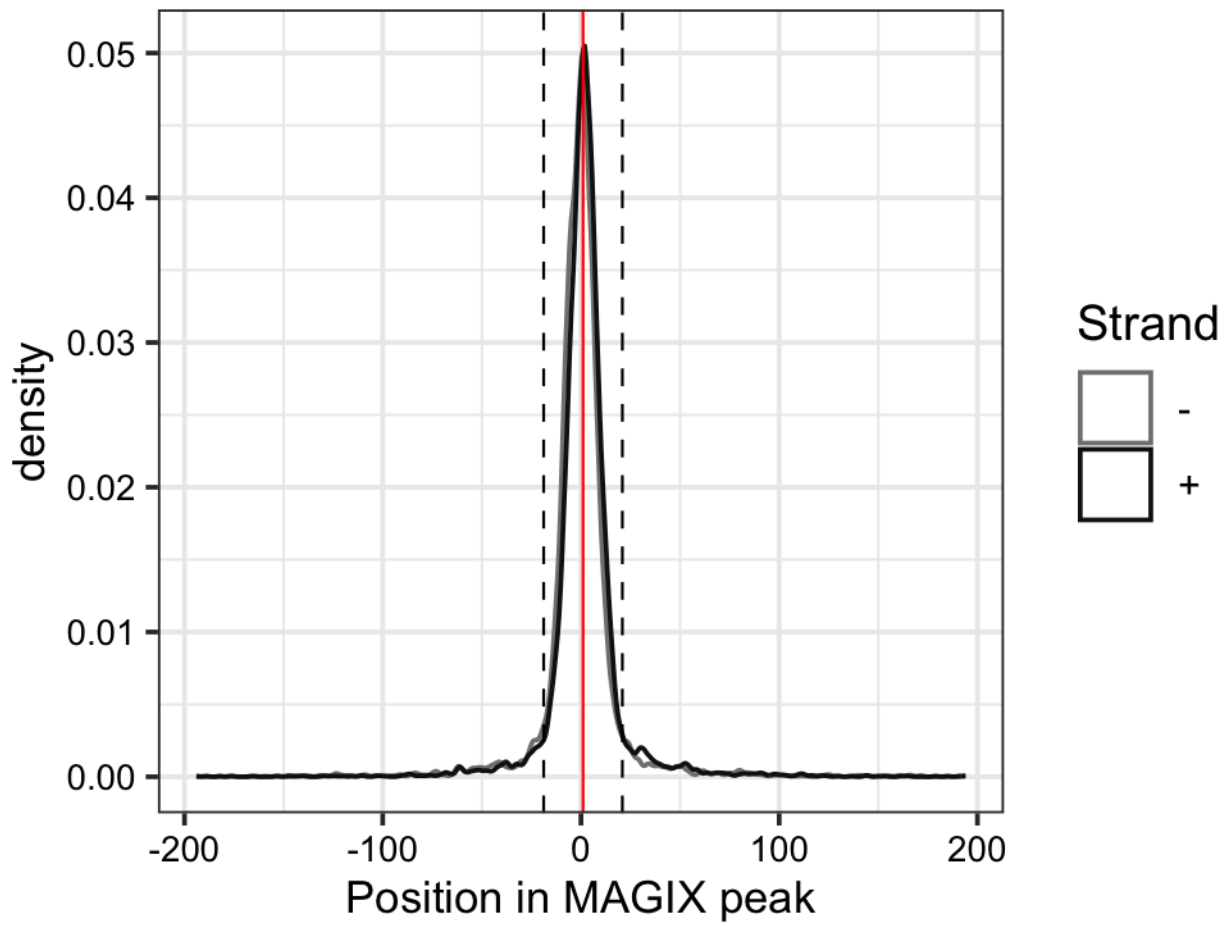

AUROC of top GRECO-BIT/MEX PWM in sliding windows (500 peaks, 50 peak step) along the MAGIX peaks (up to the top 75,000) ranked by the MAGIX enrichment coefficient and fraction of overlapping ChIP peaks in the same window

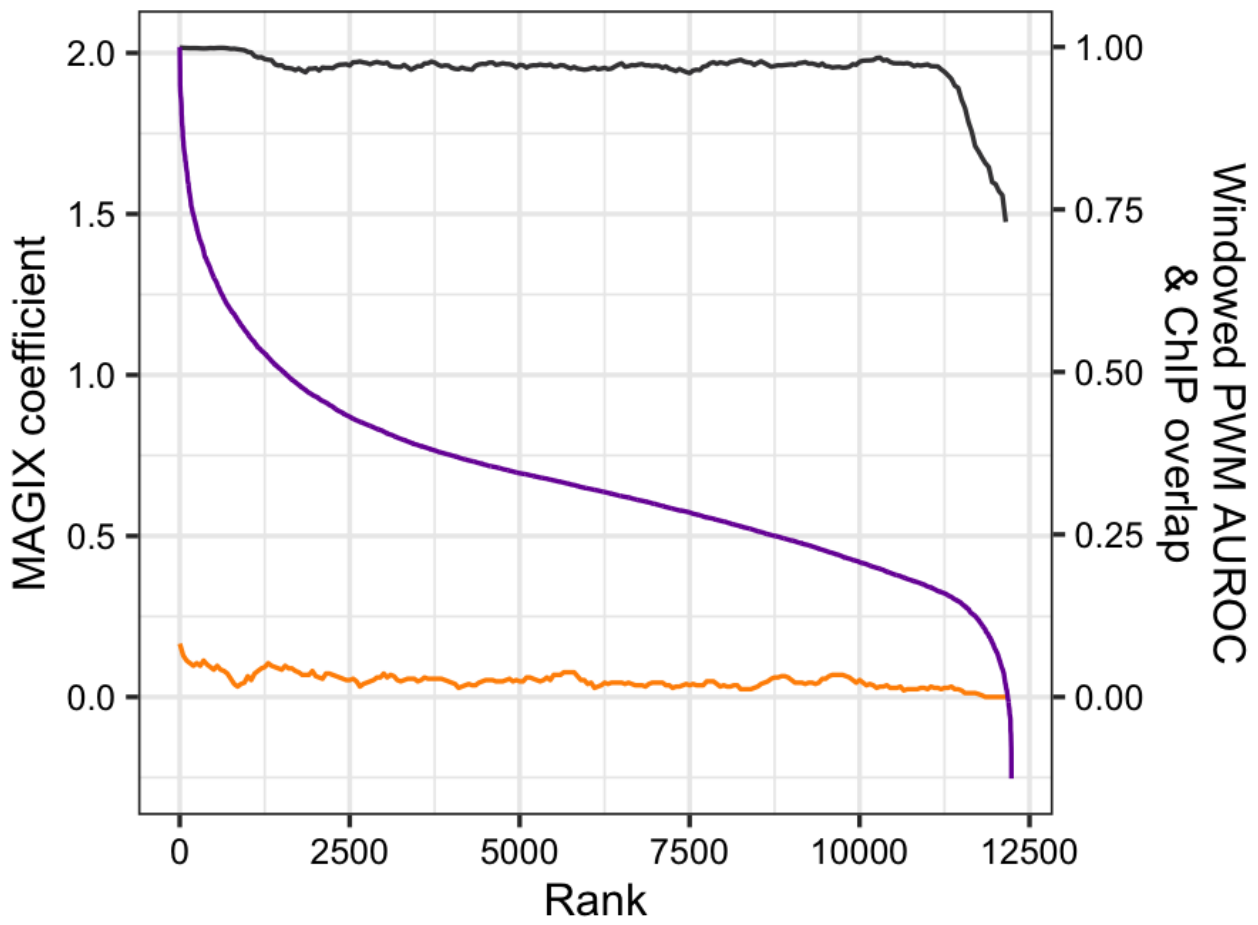

ZNF689

Top GRECO-BIT/MEX PWM: PWM005247

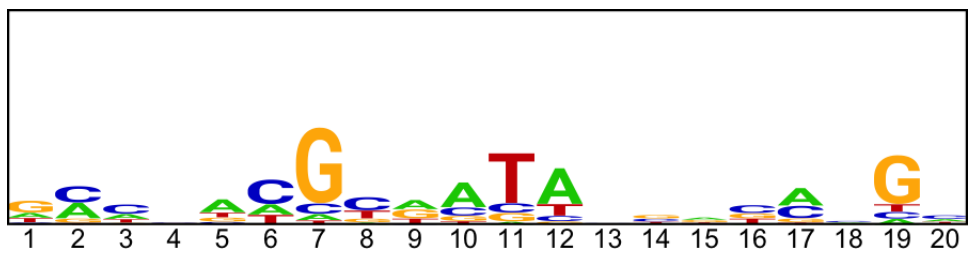

Location of top GRECO-BIT/MEX PWM in MAGIX peaks (up to the top 10,000)

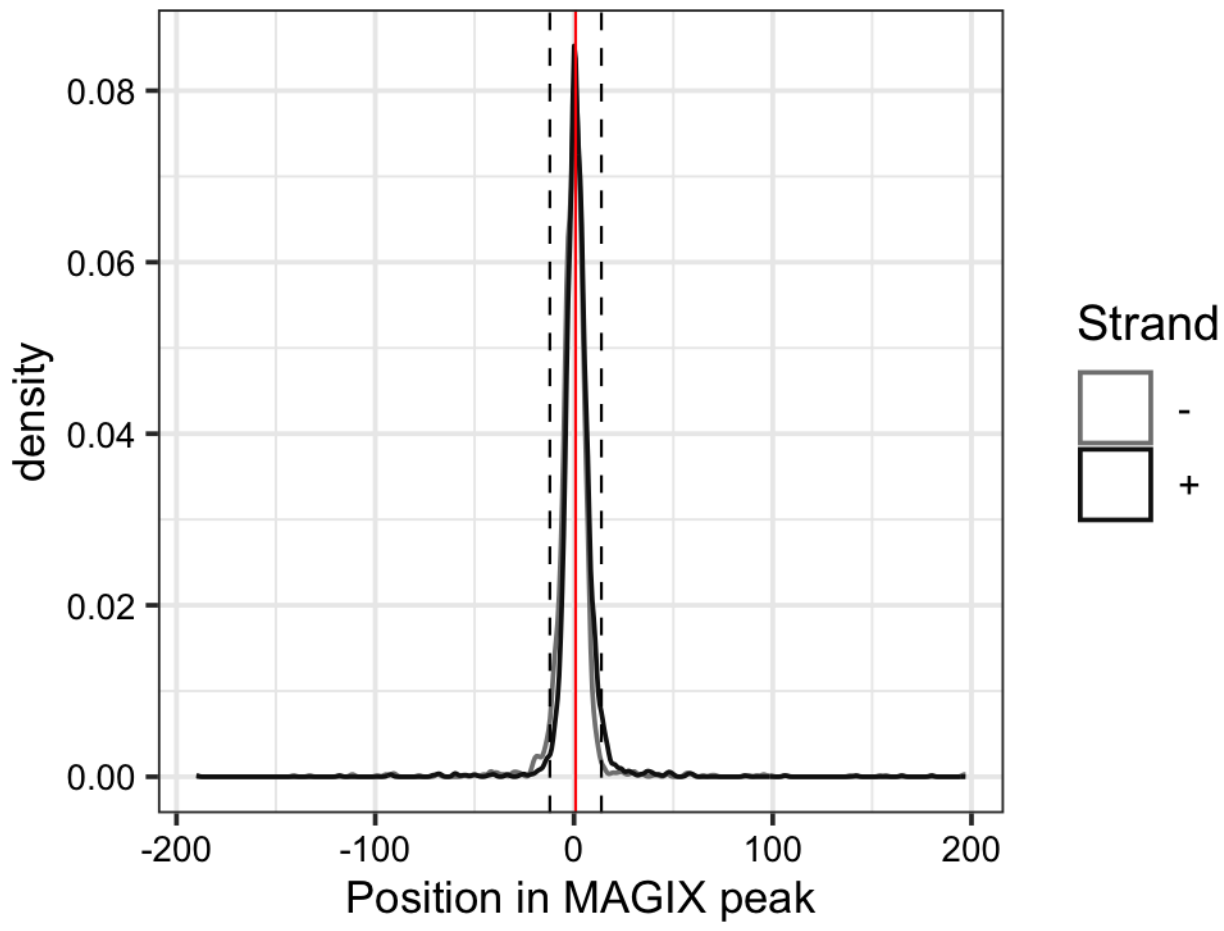

AUROC of top GRECO-BIT/MEX PWM in sliding windows (500 peaks, 50 peak step) along the MAGIX peaks (up to the top 75,000) ranked by the MAGIX enrichment coefficient and fraction of overlapping ChIP peaks in the same window

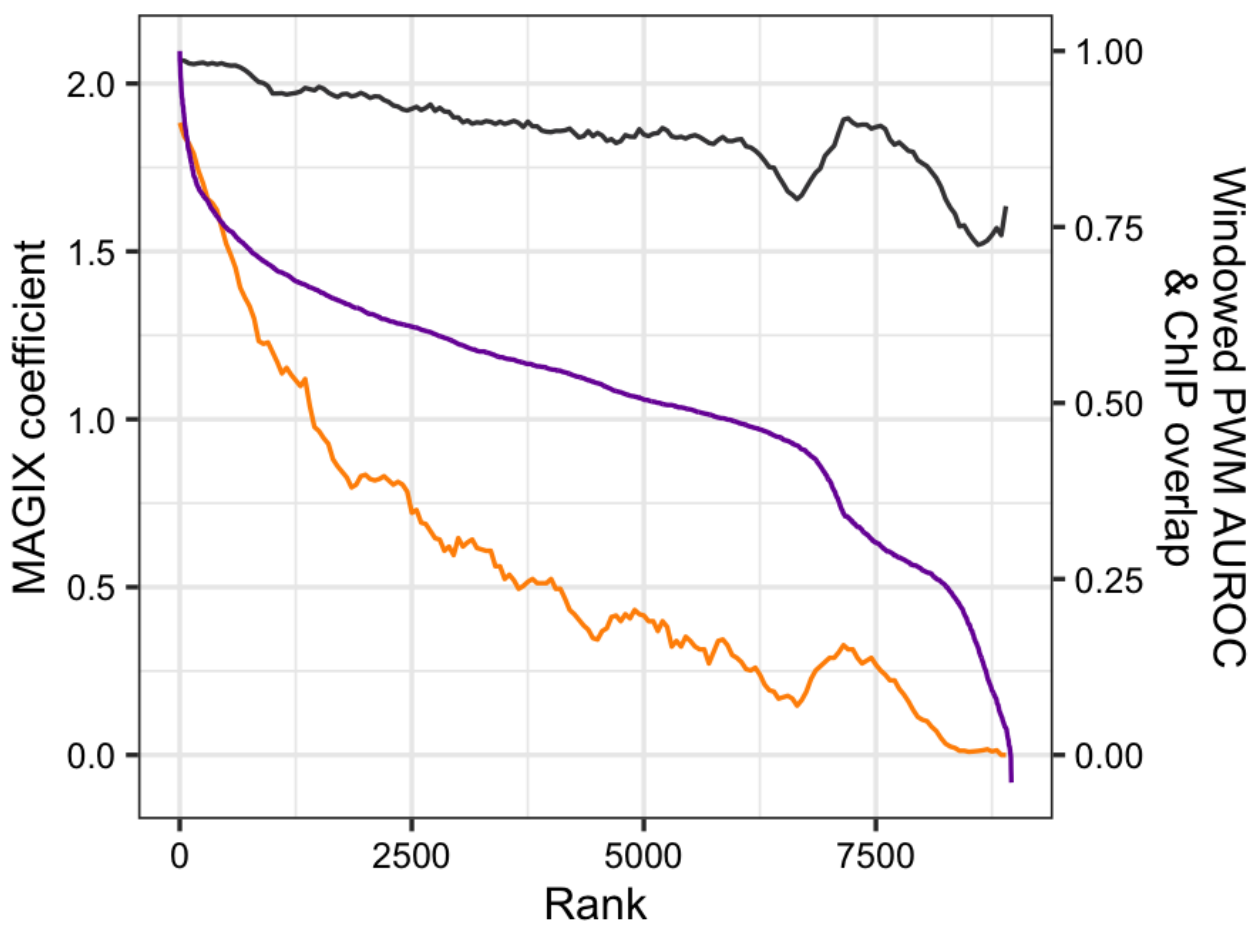

ZNF696

Top GRECO-BIT/MEX PWM: PWM005274

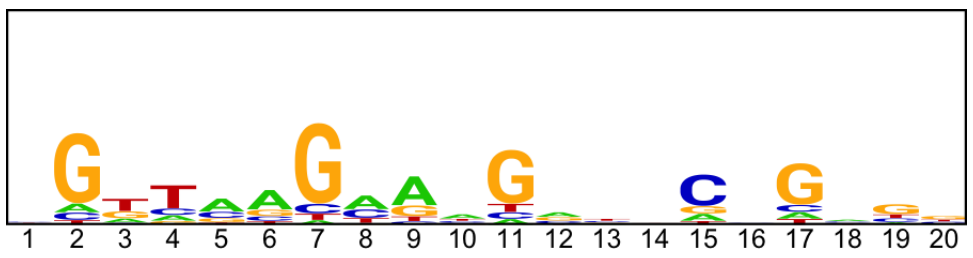

Location of top GRECO-BIT/MEX PWM in MAGIX peaks (up to the top 10,000)

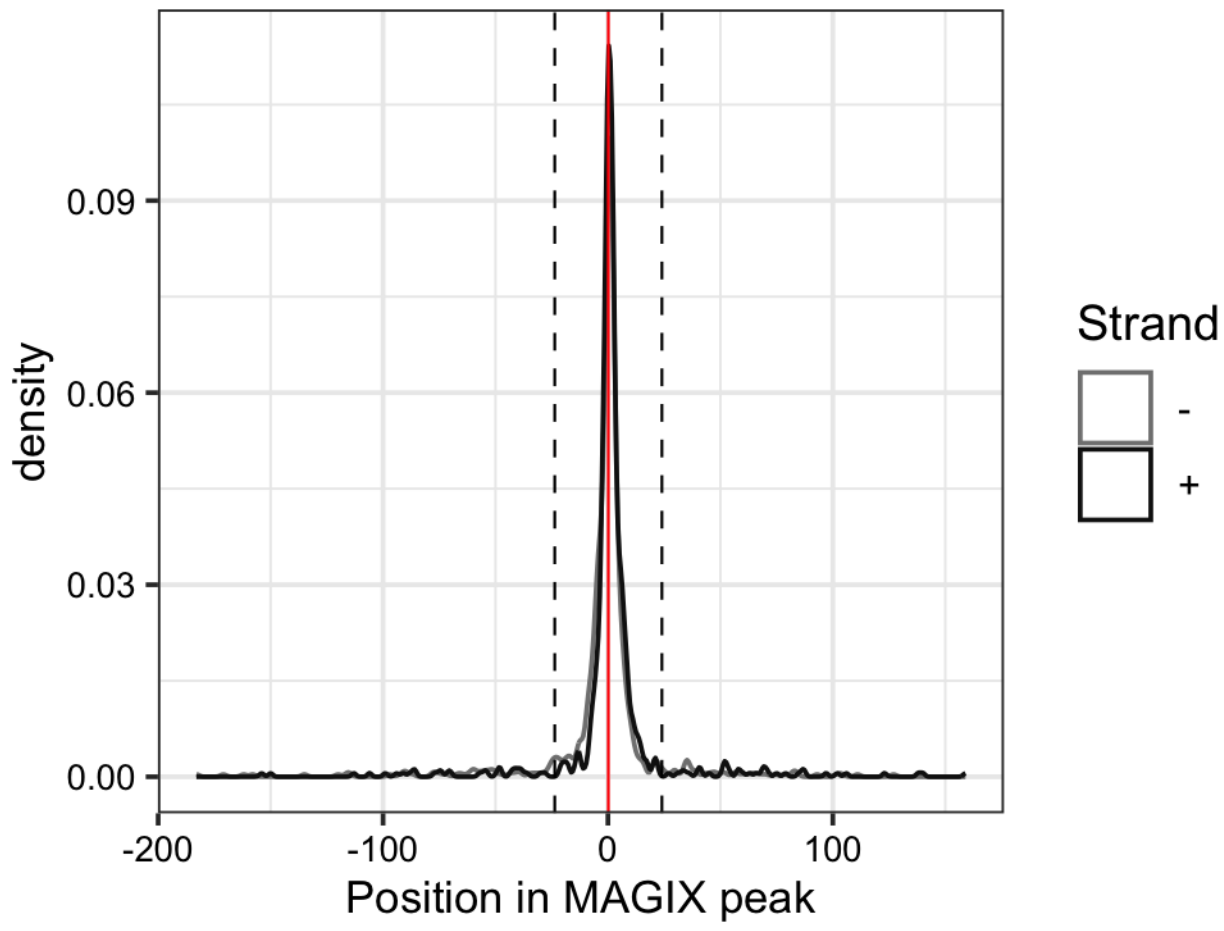

AUROC of top GRECO-BIT/MEX PWM in sliding windows (500 peaks, 50 peak step) along the MAGIX peaks (up to the top 75,000) ranked by the MAGIX enrichment coefficient and fraction of overlapping ChIP peaks in the same window

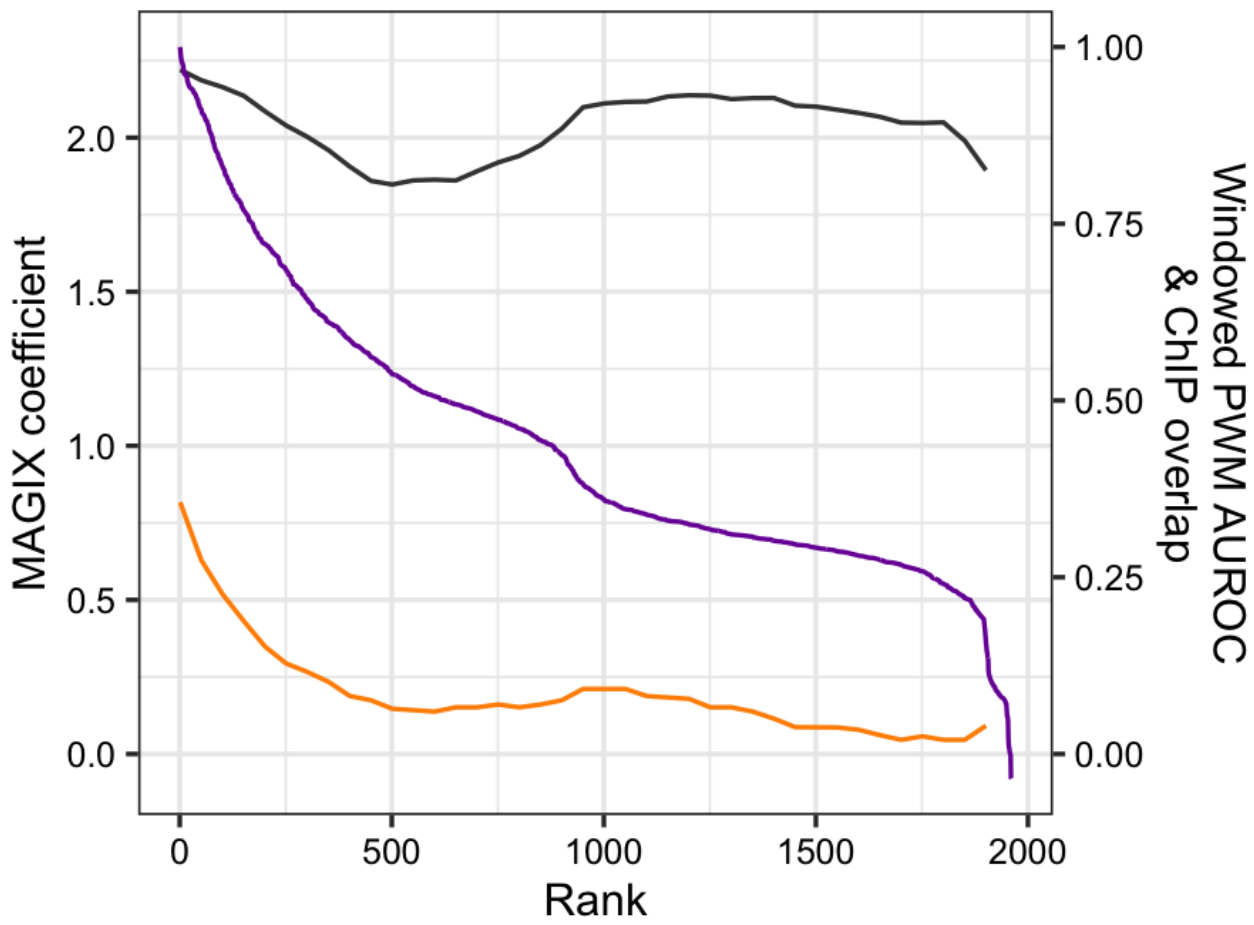

ZNF699

Top GRECO-BIT/MEX PWM: PWM005313

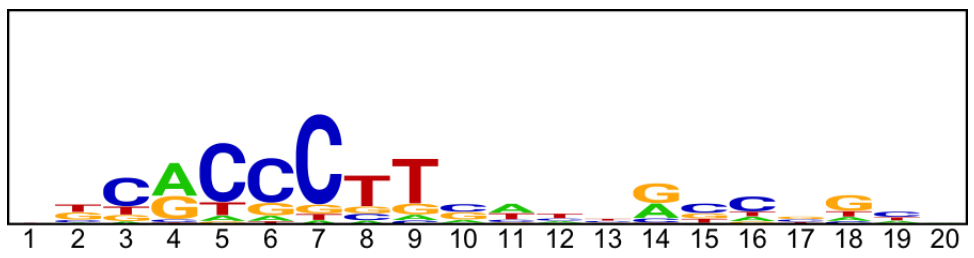

Location of top GRECO-BIT/MEX PWM in MAGIX peaks (up to the top 10,000)

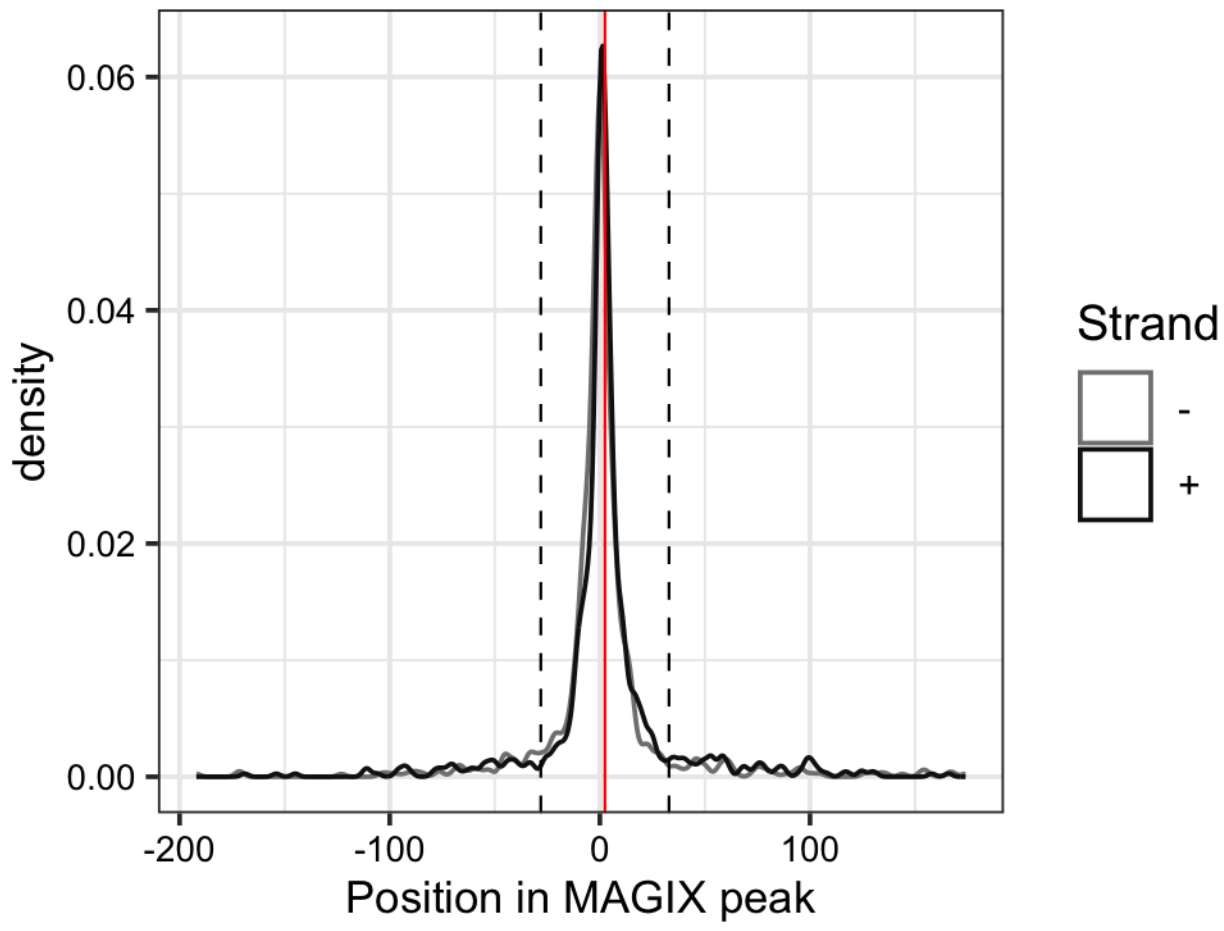

AUROC of top GRECO-BIT/MEX PWM in sliding windows (500 peaks, 50 peak step) along the MAGIX peaks (up to the top 75,000) ranked by the MAGIX enrichment coefficient and fraction of overlapping ChIP peaks in the same window

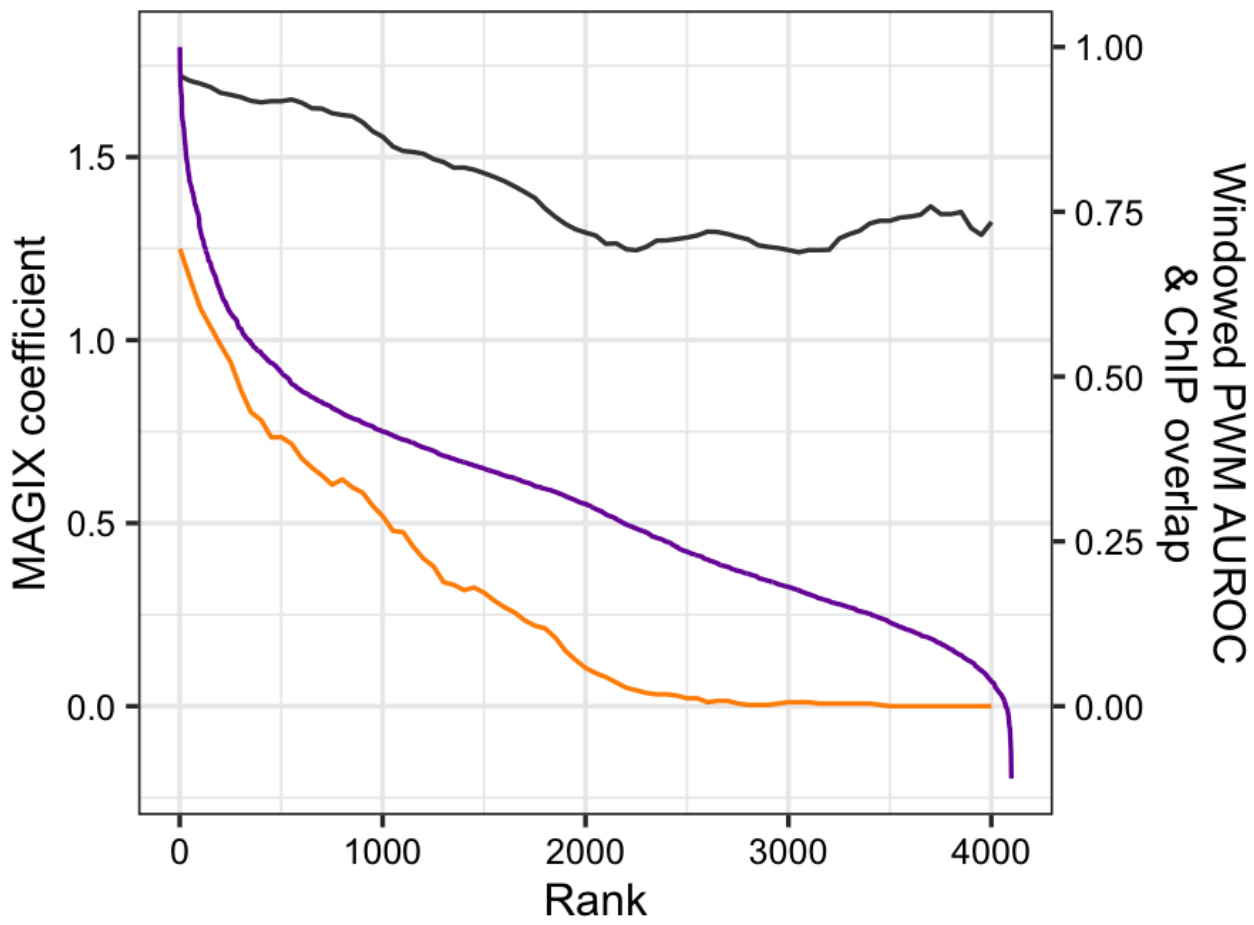

ZNF70

Top GRECO-BIT/MEX PWM: PWM005343

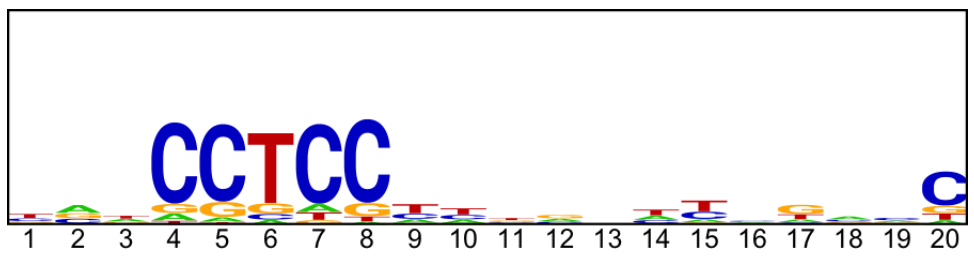

Location of top GRECO-BIT/MEX PWM in MAGIX peaks (up to the top 10,000)

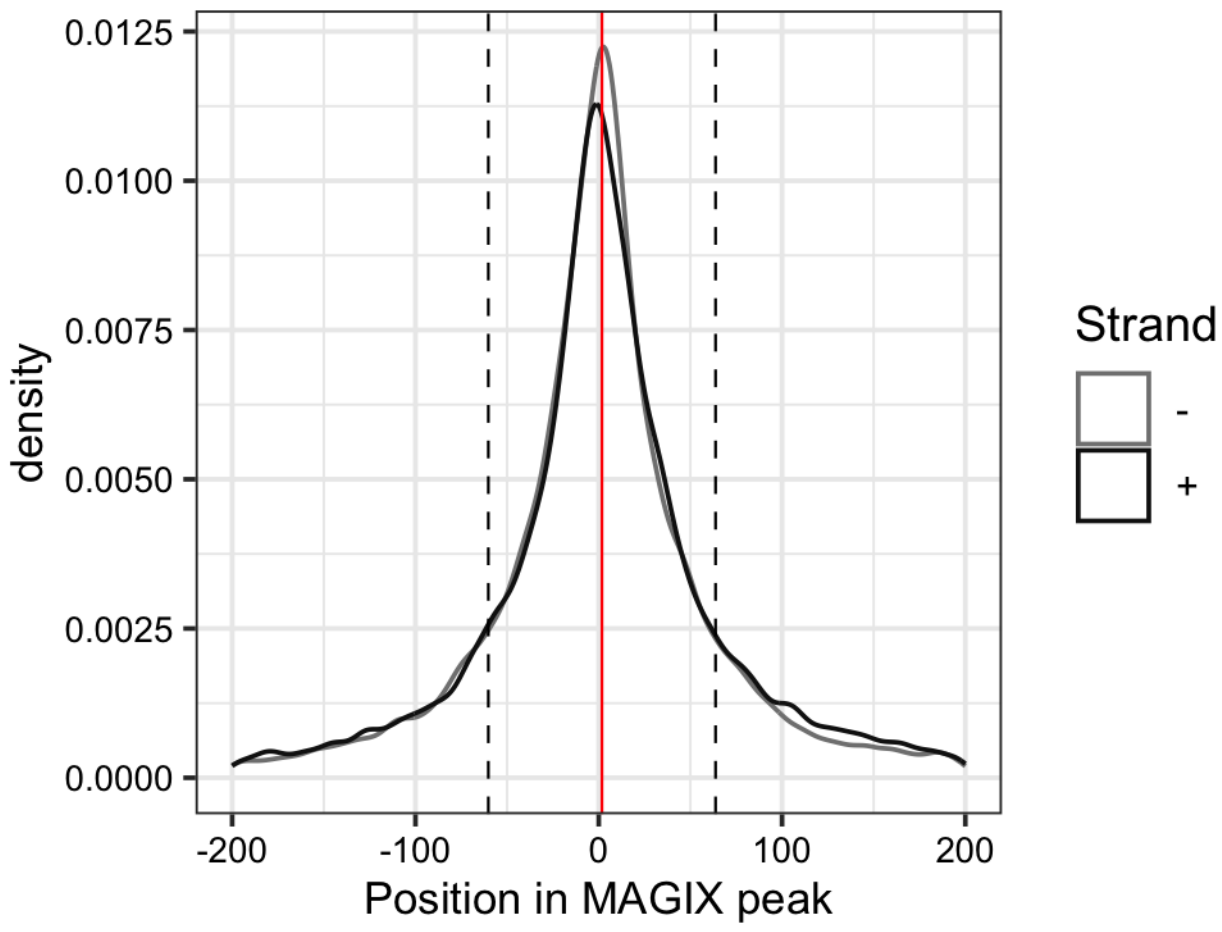

AUROC of top GRECO-BIT/MEX PWM in sliding windows (500 peaks, 50 peak step) along the MAGIX peaks (up to the top 75,000) ranked by the MAGIX enrichment coefficient and fraction of overlapping ChIP peaks in the same window

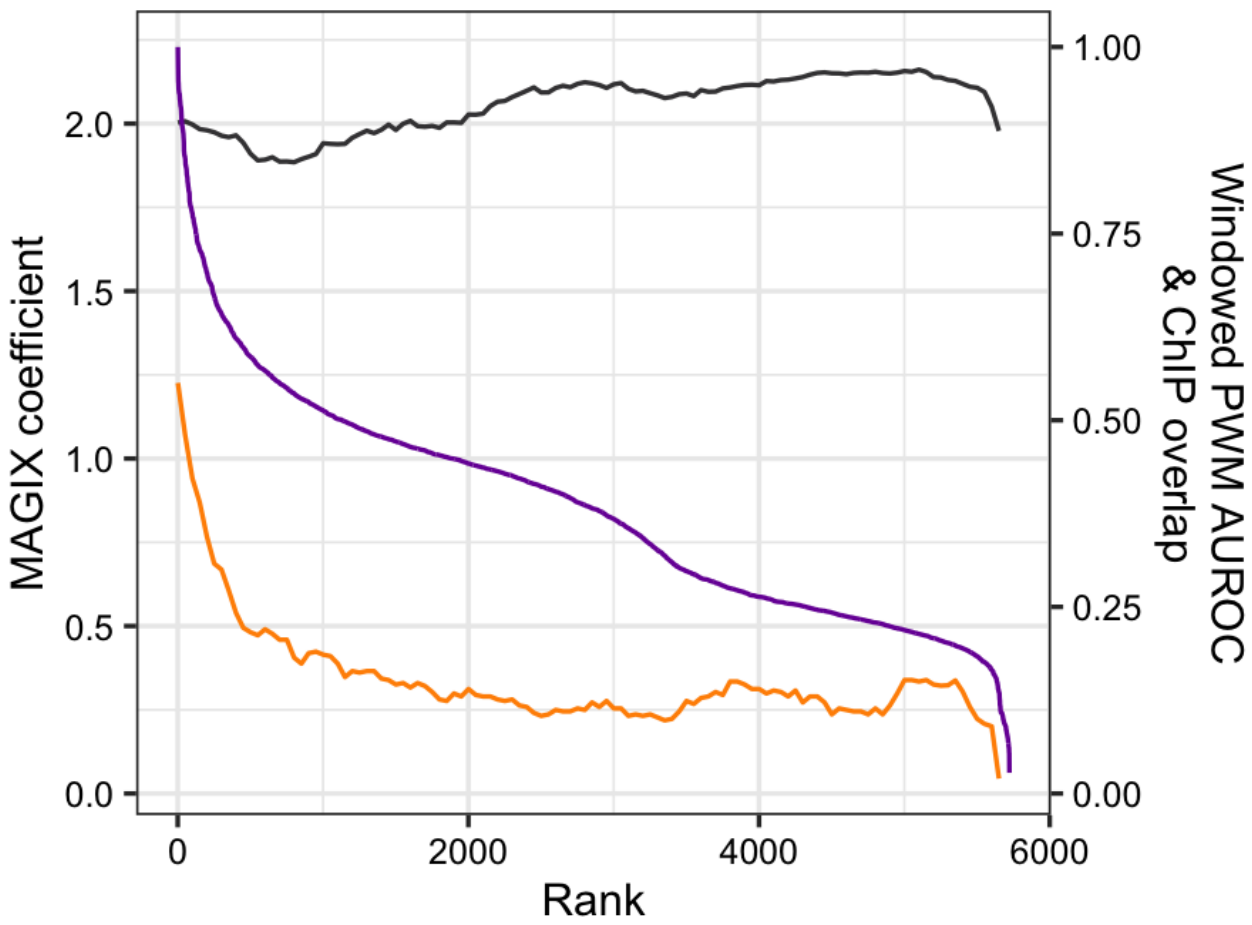

ZNF700.DBD2

Top GRECO-BIT/MEX PWM: PWM005386

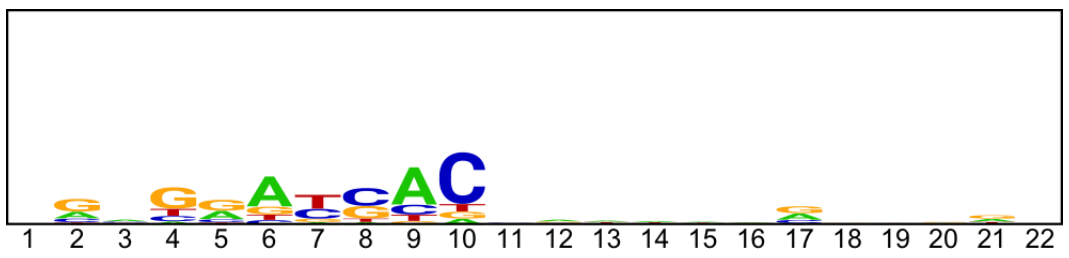

Location of top GRECO-BIT/MEX PWM in MAGIX peaks (up to the top 10,000)

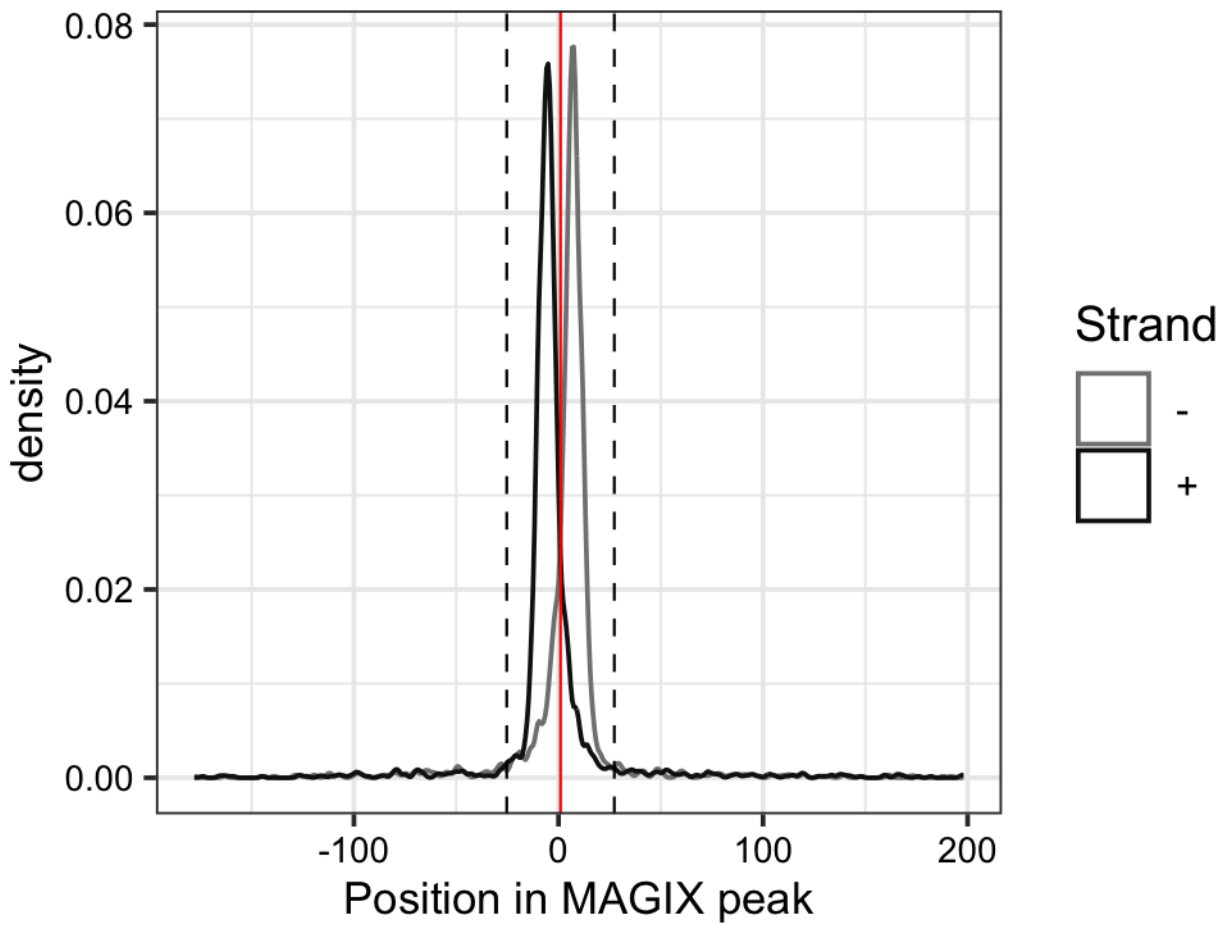

ZNF721.DBD1

Top GRECO-BIT/MEX PWM: PWM005501

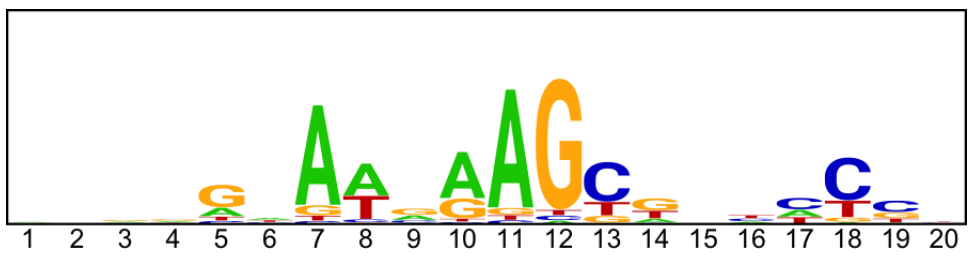

Location of top GRECO-BIT/MEX PWM in MAGIX peaks (up to the top 10,000)

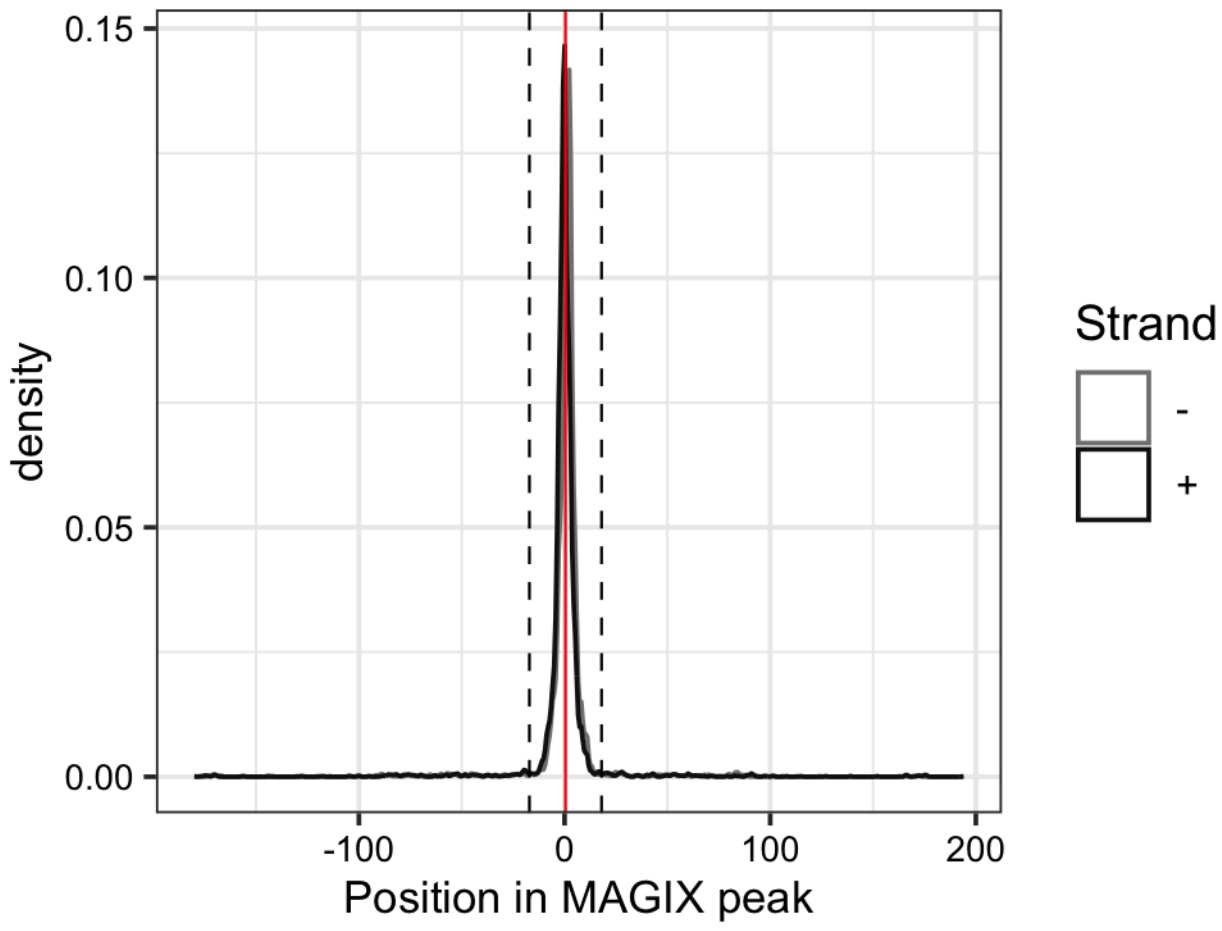

ZNF721.DBD2

Top GRECO-BIT/MEX PWM: PWM005498

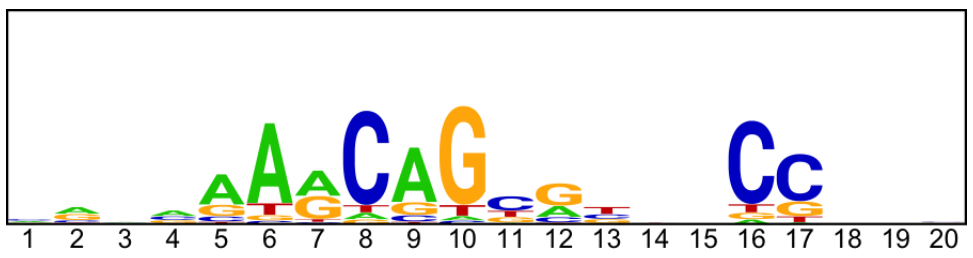

Location of top GRECO-BIT/MEX PWM in MAGIX peaks (up to the top 10,000)

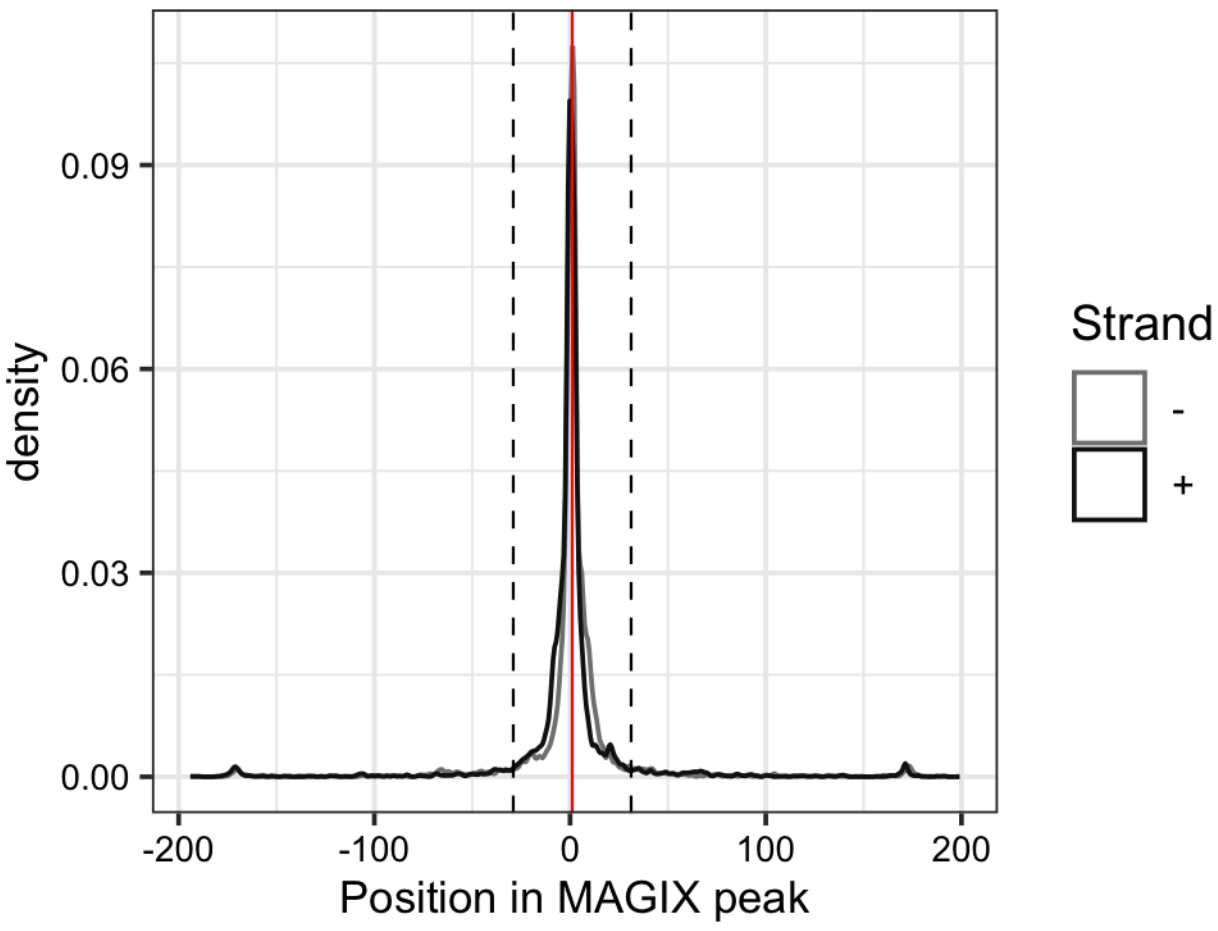

ZNF721.FL

Top GRECO-BIT/MEX PWM: PWM005515

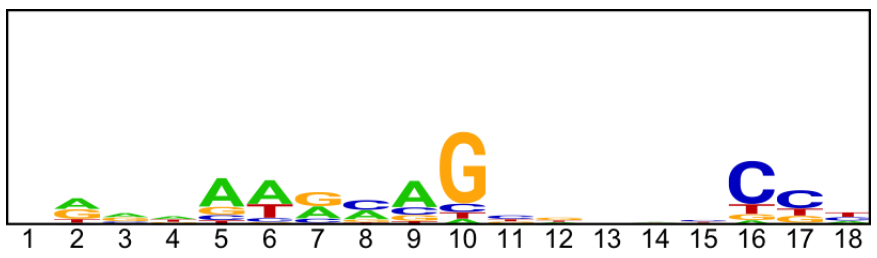

Location of top GRECO-BIT/MEX PWM in MAGIX peaks (up to the top 10,000)

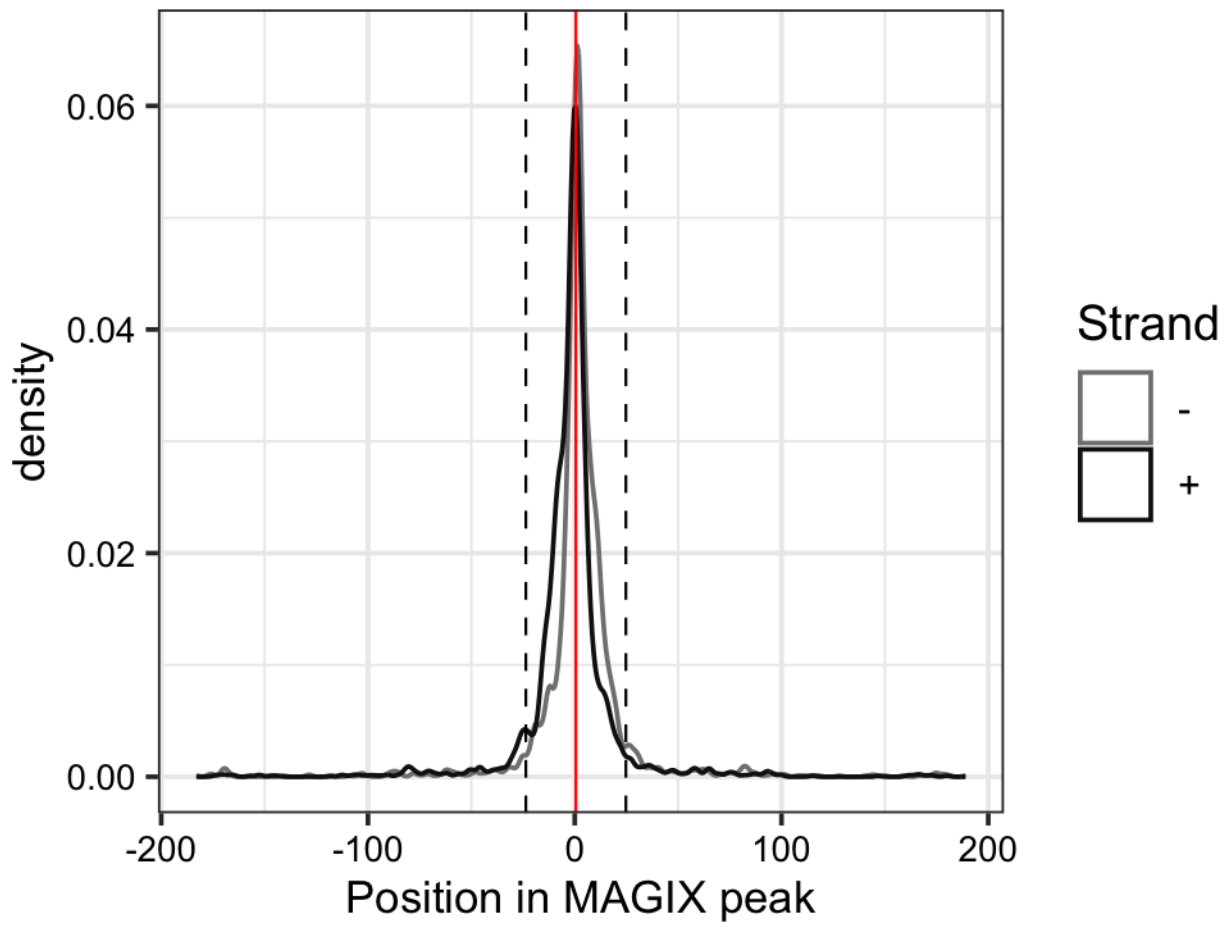

AUROC of top GRECO-BIT/MEX PWM in sliding windows (500 peaks, 50 peak step) along the MAGIX peaks (up to the top 75,000) ranked by the MAGIX enrichment coefficient and fraction of overlapping ChIP peaks in the same window

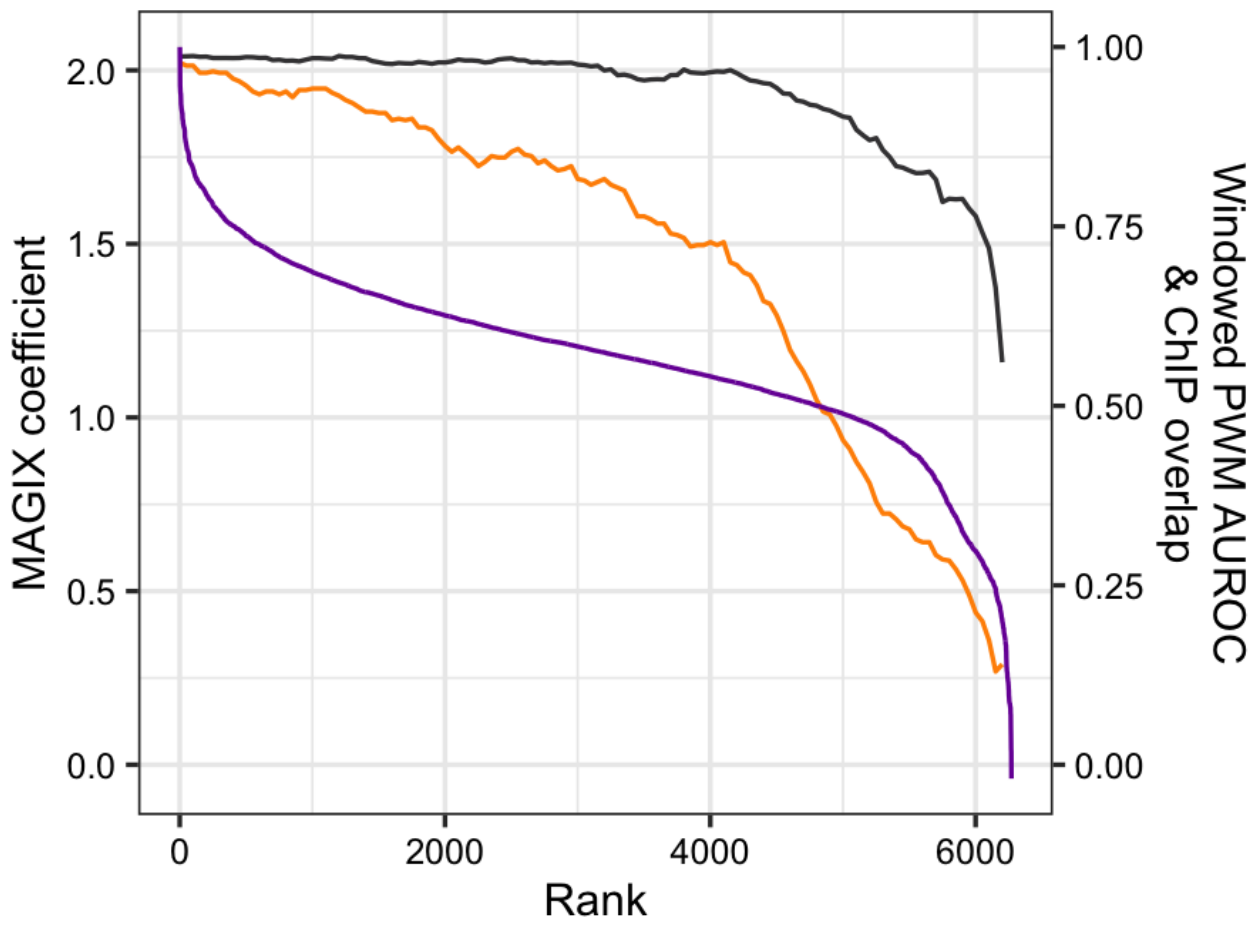

ZNF724

Top GRECO-BIT/MEX PWM: PWM005525

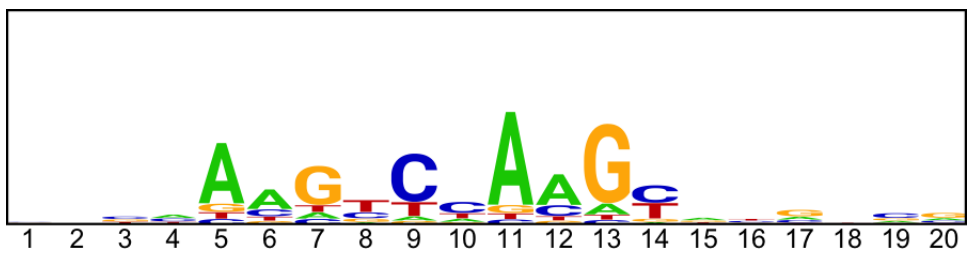

Location of top GRECO-BIT/MEX PWM in MAGIX peaks (up to the top 10,000)

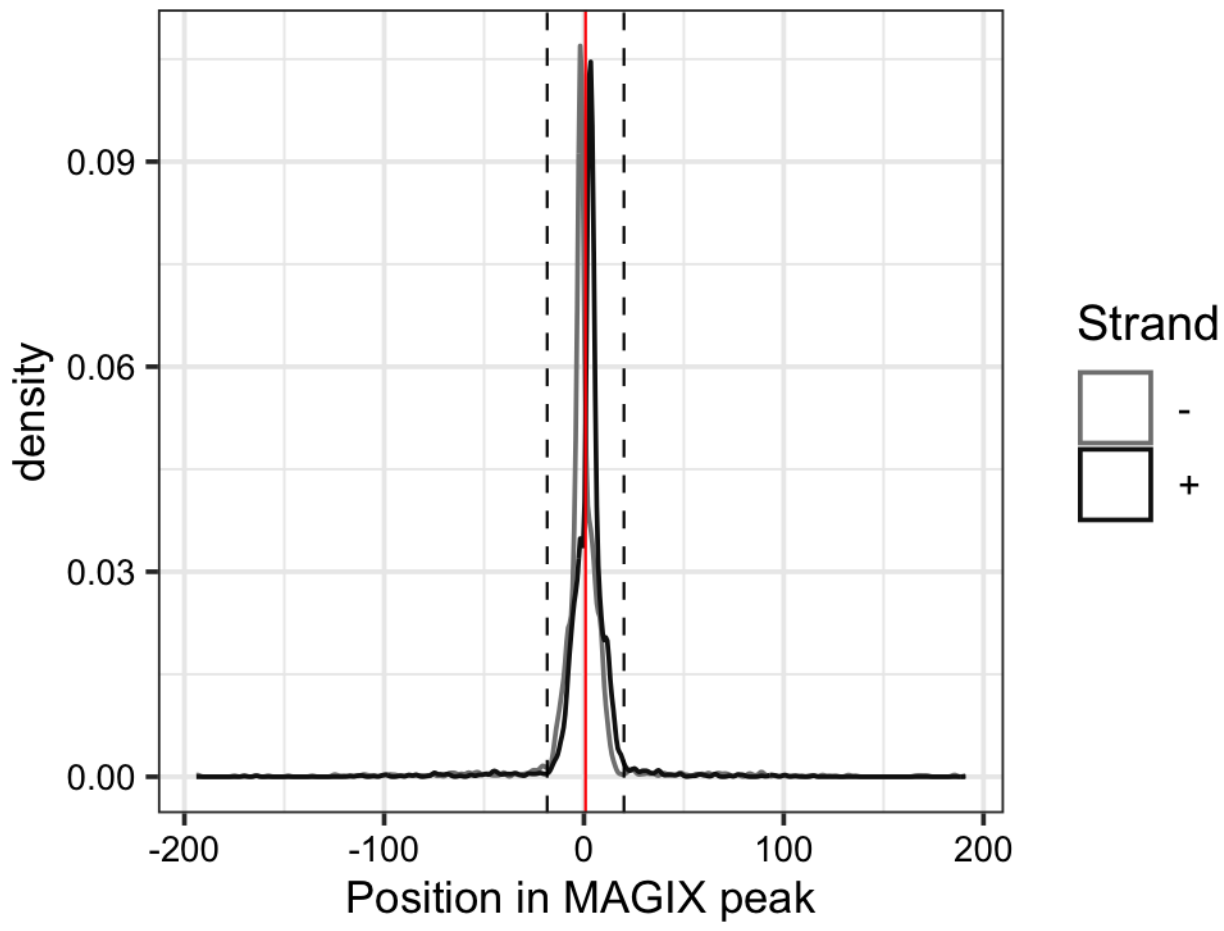

AUROC of top GRECO-BIT/MEX PWM in sliding windows (500 peaks, 50 peak step) along the MAGIX peaks (up to the top 75,000) ranked by the MAGIX enrichment coefficient and fraction of overlapping ChIP peaks in the same window

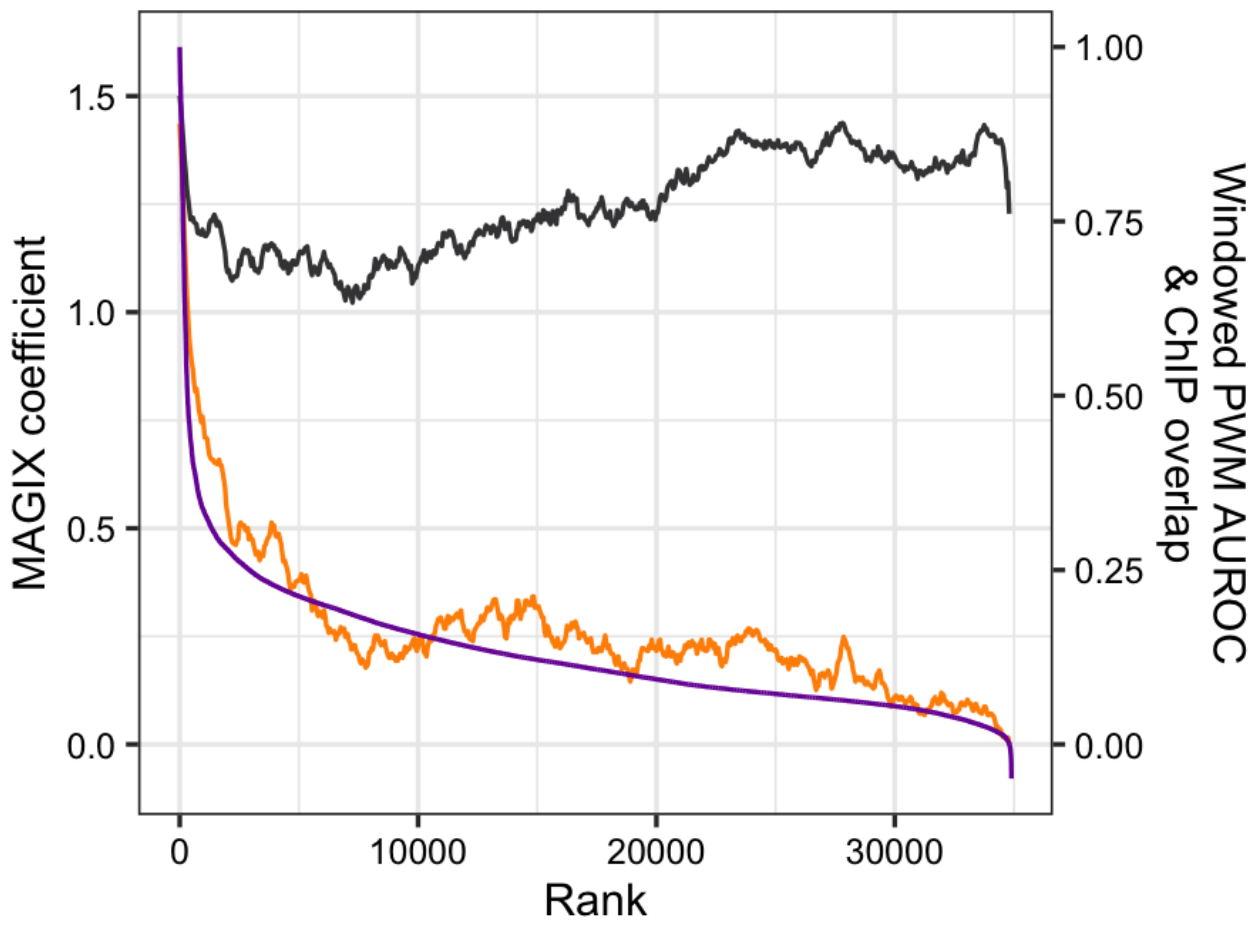

ZNF726.DBD2

Top GRECO-BIT/MEX PWM: PWM005557

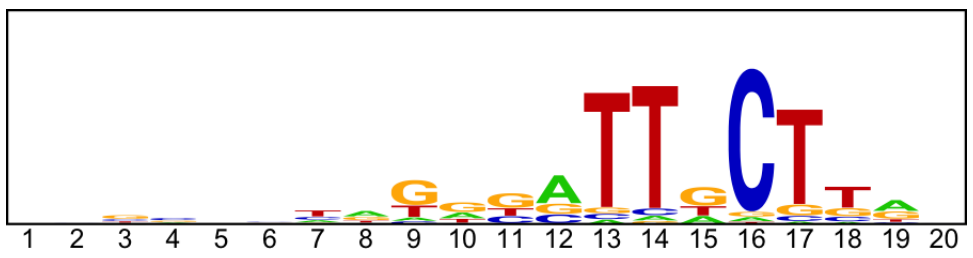

Location of top GRECO-BIT/MEX PWM in MAGIX peaks (up to the top 10,000)

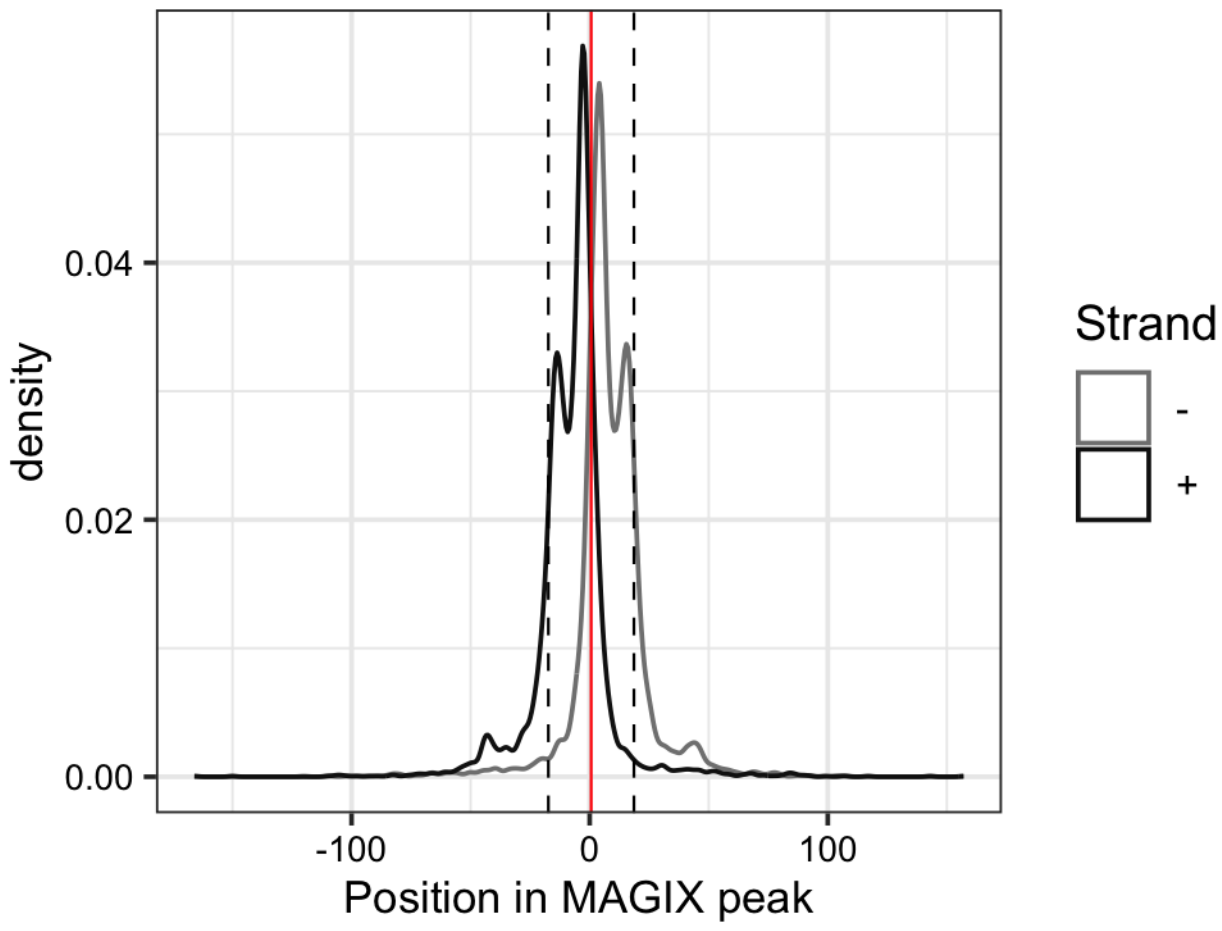

ZNF726.FL

Top GRECO-BIT/MEX PWM: PWM005561

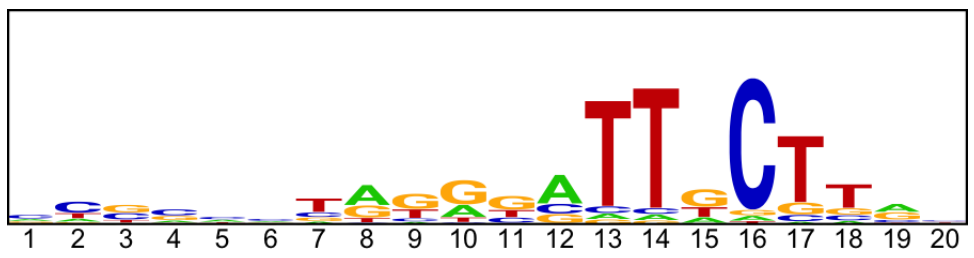

Location of top GRECO-BIT/MEX PWM in MAGIX peaks (up to the top 10,000)

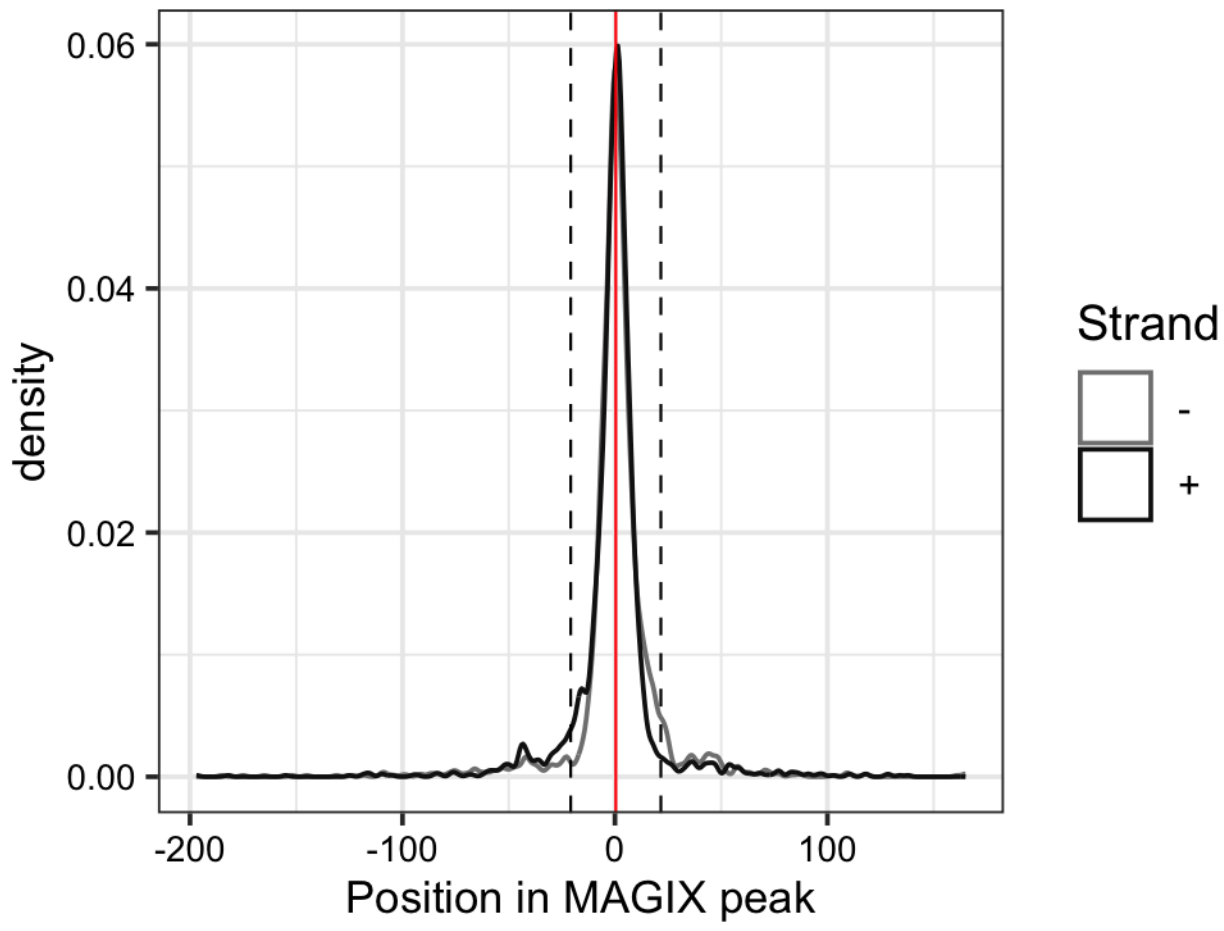

AUROC of top GRECO-BIT/MEX PWM in sliding windows (500 peaks, 50 peak step) along the MAGIX peaks (up to the top 75,000) ranked by the MAGIX enrichment coefficient and fraction of overlapping ChIP peaks in the same window

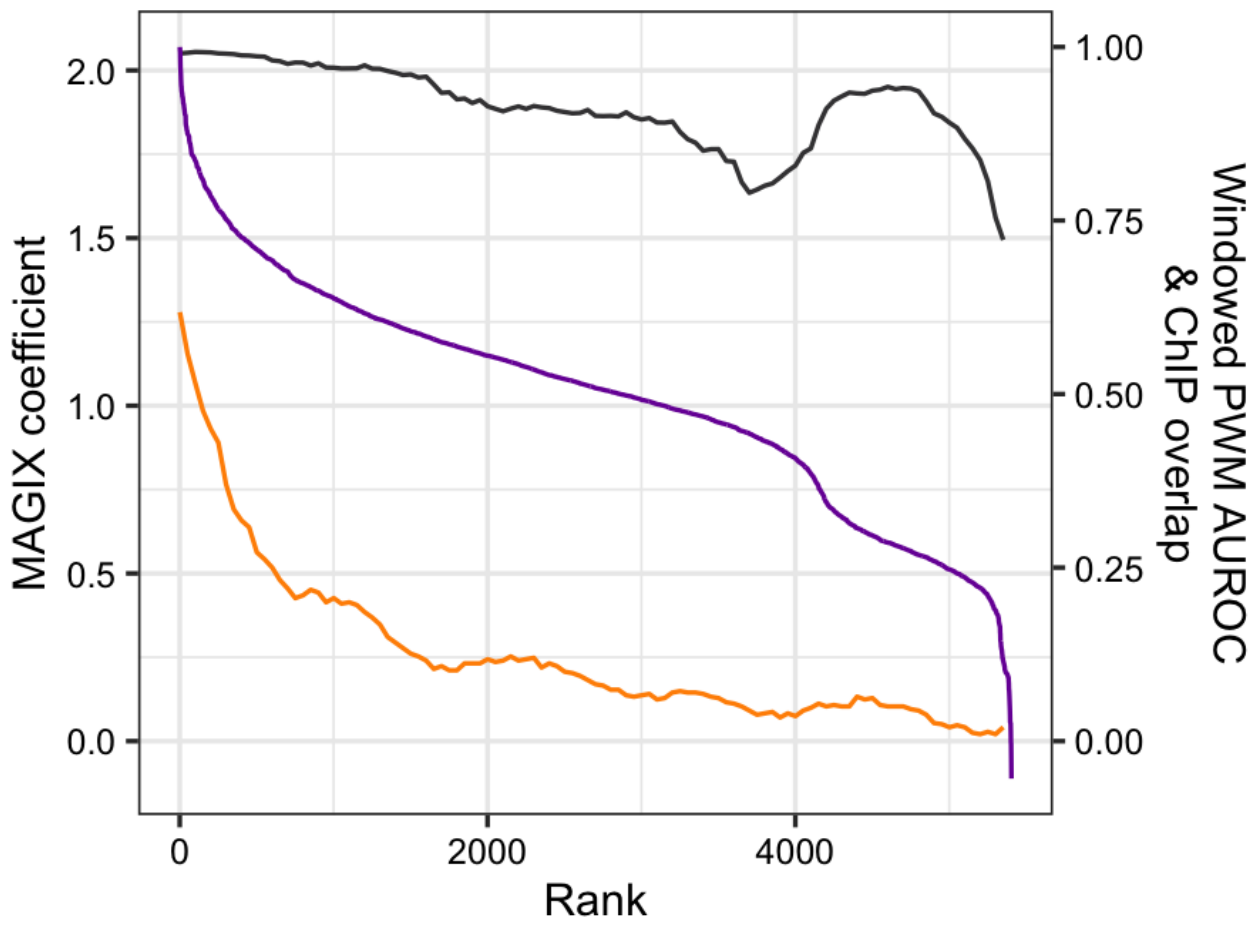

ZNF728

Top GRECO-BIT/MEX PWM: PWM005595

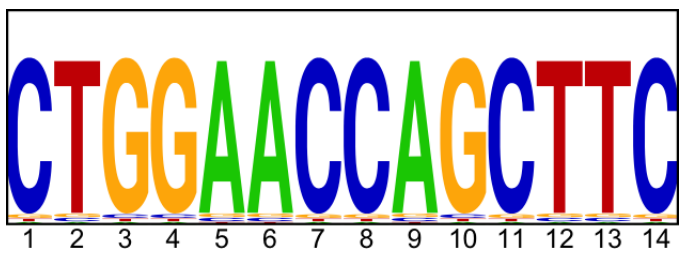

Location of top GRECO-BIT/MEX PWM in MAGIX peaks (up to the top 10,000)

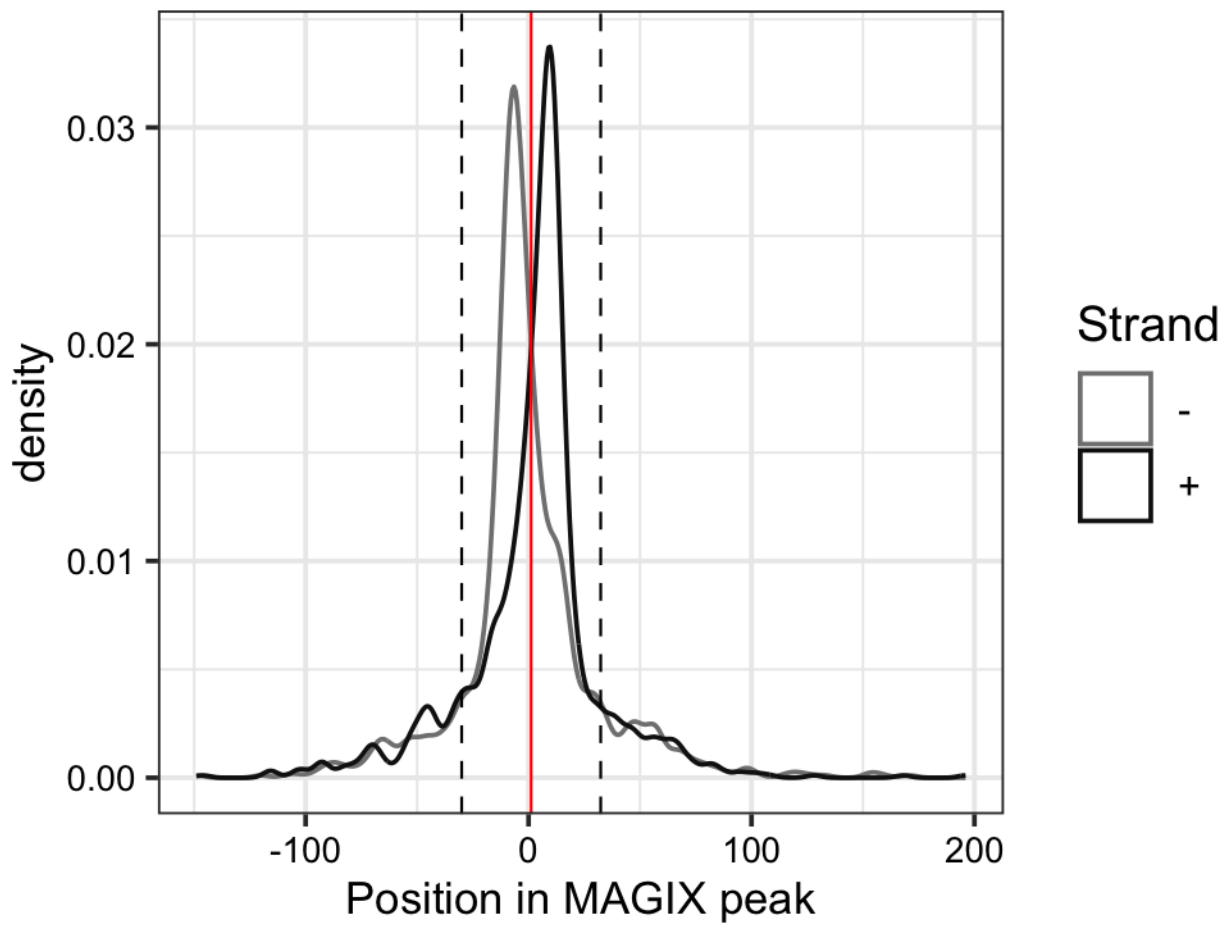

AUROC of top GRECO-BIT/MEX PWM in sliding windows (500 peaks, 50 peak step) along the MAGIX peaks (up to the top 75,000) ranked by the MAGIX enrichment coefficient and fraction of overlapping ChIP peaks in the same window

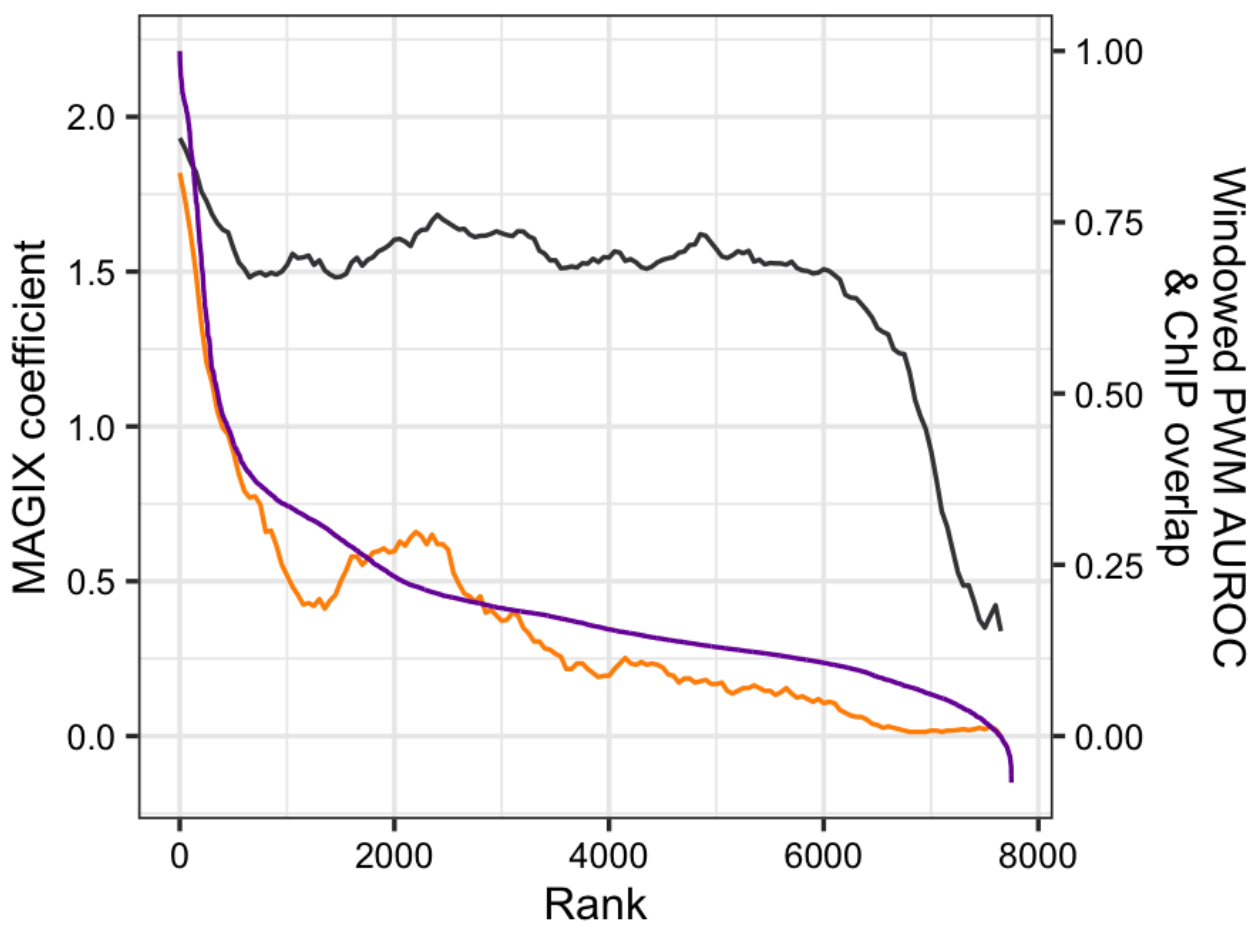

ZNF729.DBD1

Top GRECO-BIT/MEX PWM: PWM180359

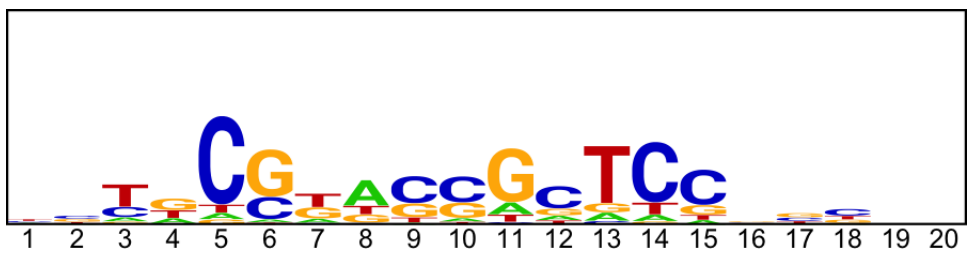

Location of top GRECO-BIT/MEX PWM in MAGIX peaks (up to the top 10,000)

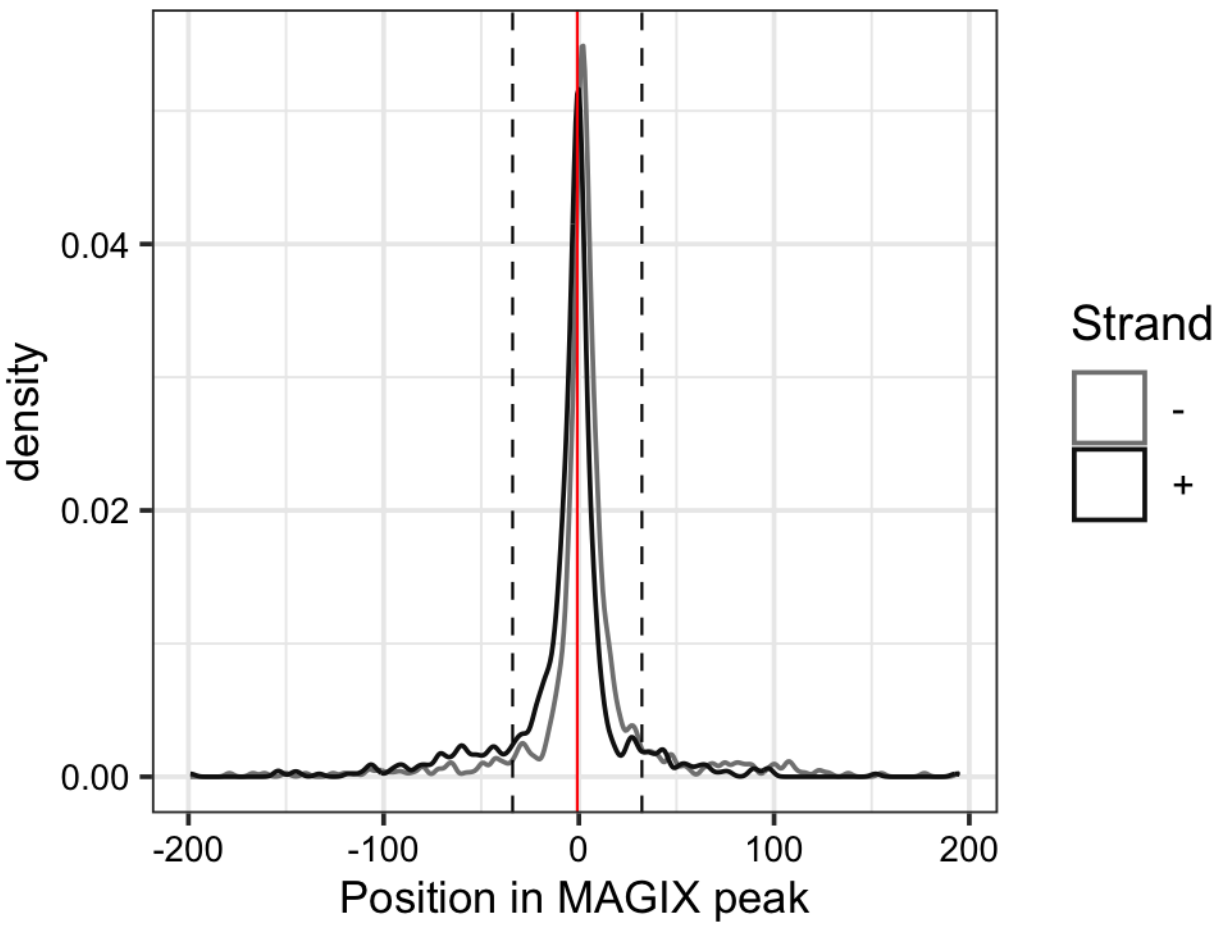

ZNF729.DBD2

Top GRECO-BIT/MEX PWM: PWM180029

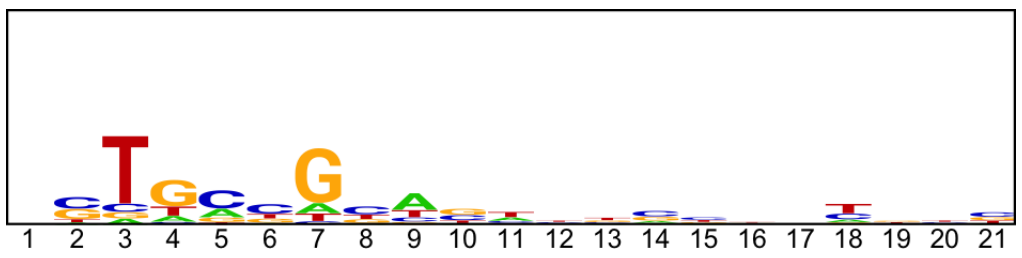

Location of top GRECO-BIT/MEX PWM in MAGIX peaks (up to the top 10,000)

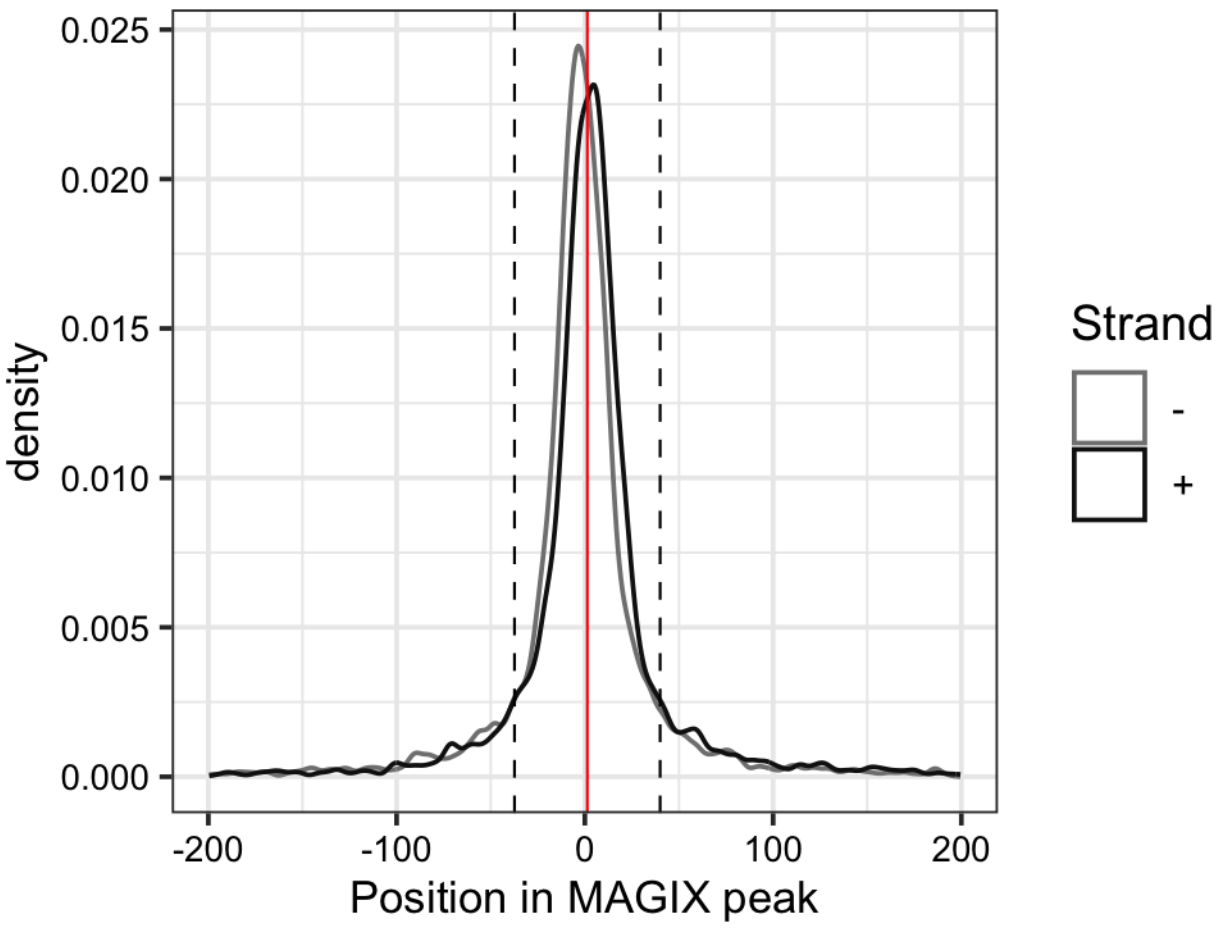

ZNF729.DBD3

Top GRECO-BIT/MEX PWM: PWM005604

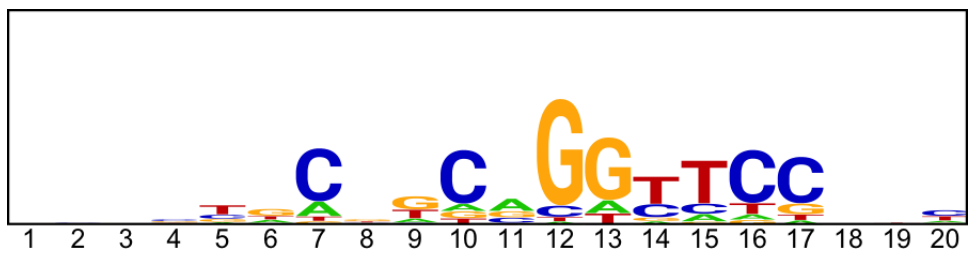

Location of top GRECO-BIT/MEX PWM in MAGIX peaks (up to the top 10,000)

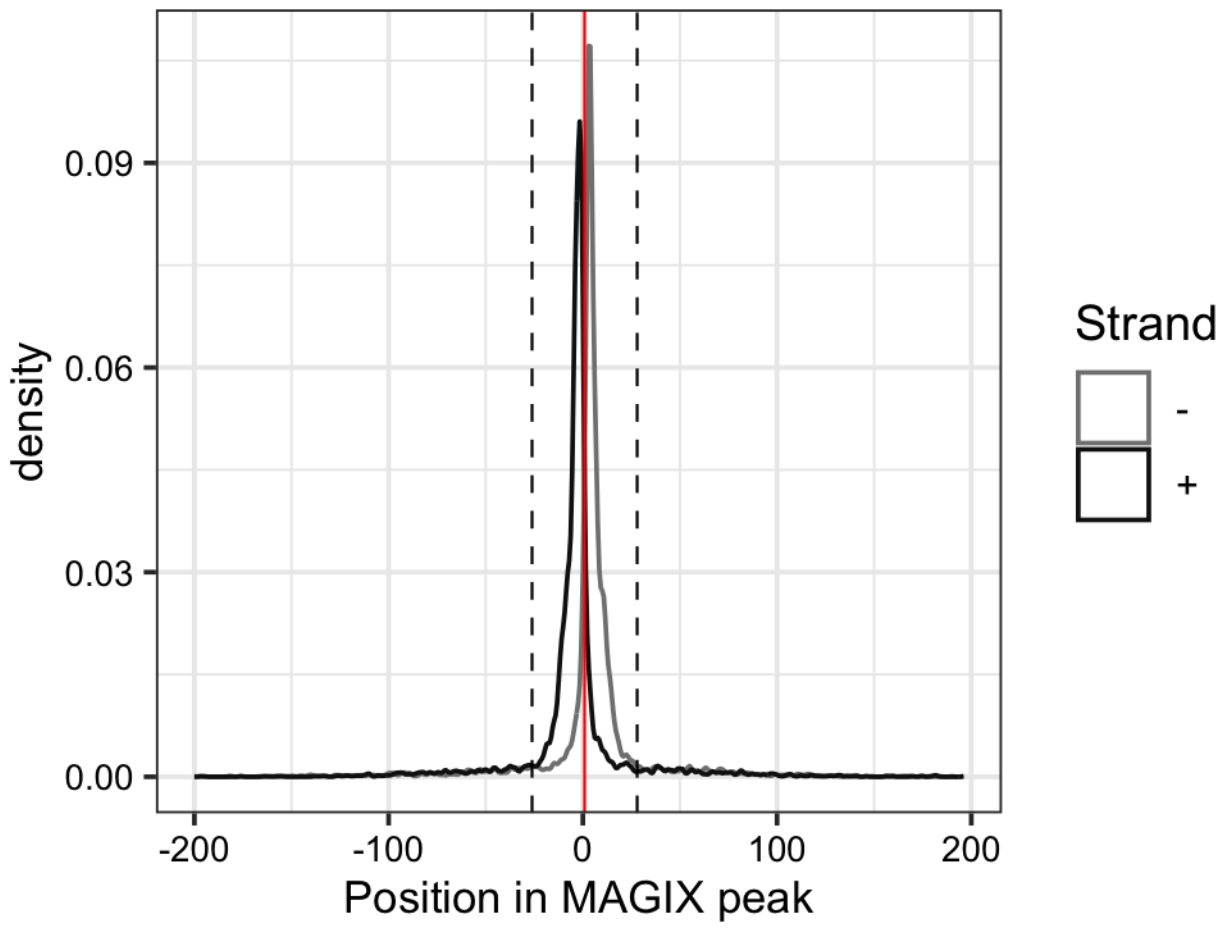

ZNF732

Top GRECO-BIT/MEX PWM: PWM005631

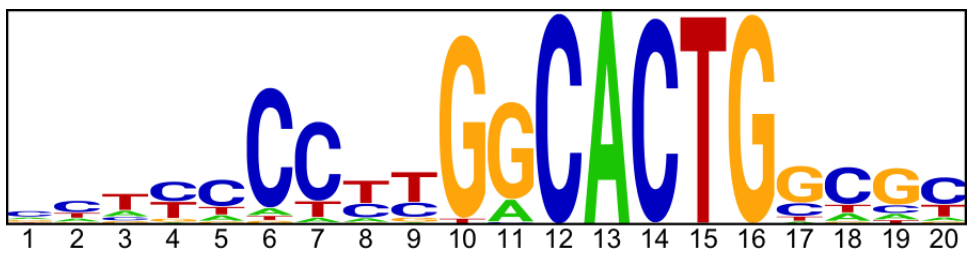

Location of top GRECO-BIT/MEX PWM in MAGIX peaks (up to the top 10,000)

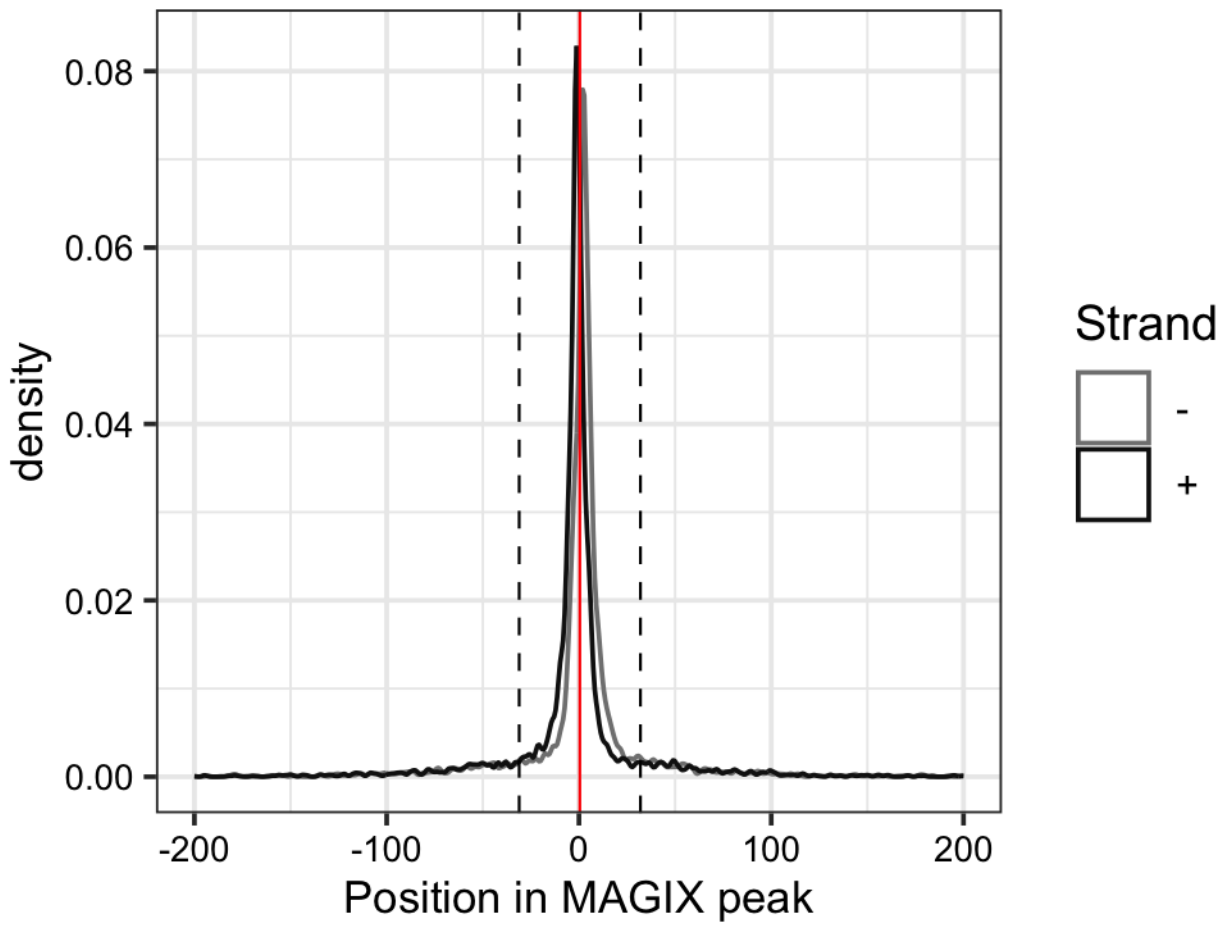

AUROC of top GRECO-BIT/MEX PWM in sliding windows (500 peaks, 50 peak step) along the MAGIX peaks (up to the top 75,000) ranked by the MAGIX enrichment coefficient and fraction of overlapping ChIP peaks in the same window

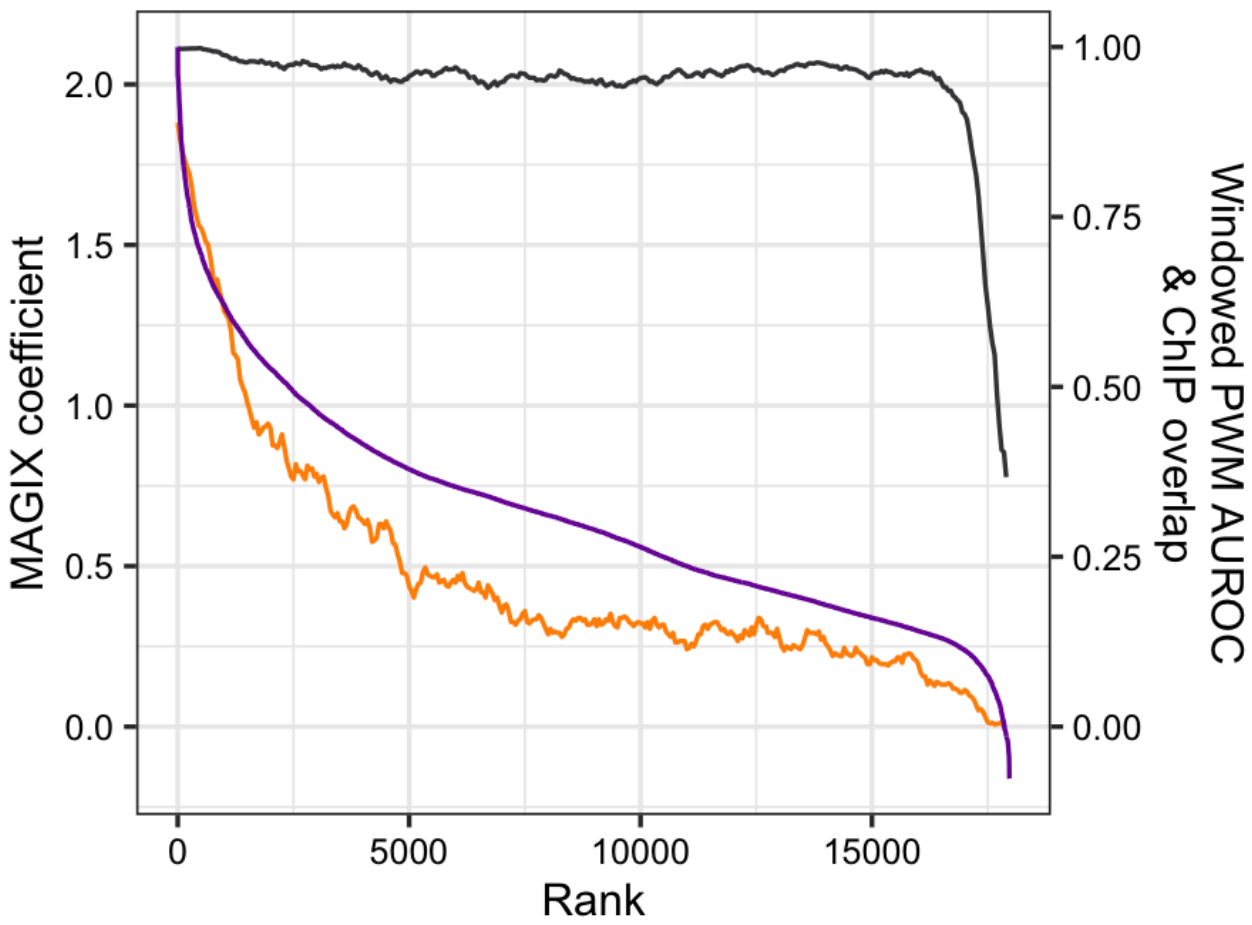

ZNF746

Top GRECO-BIT/MEX PWM: PWM005659

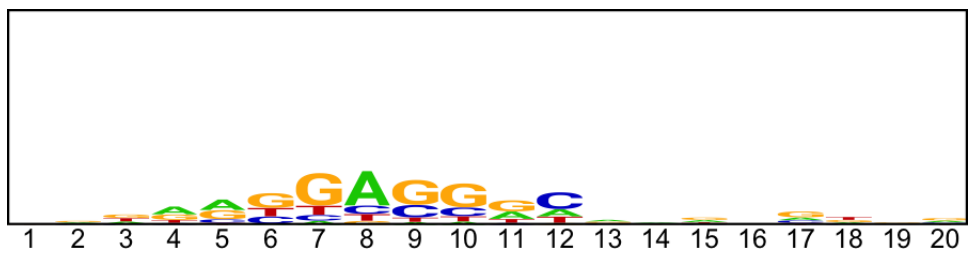

Location of top GRECO-BIT/MEX PWM in MAGIX peaks (up to the top 10,000)

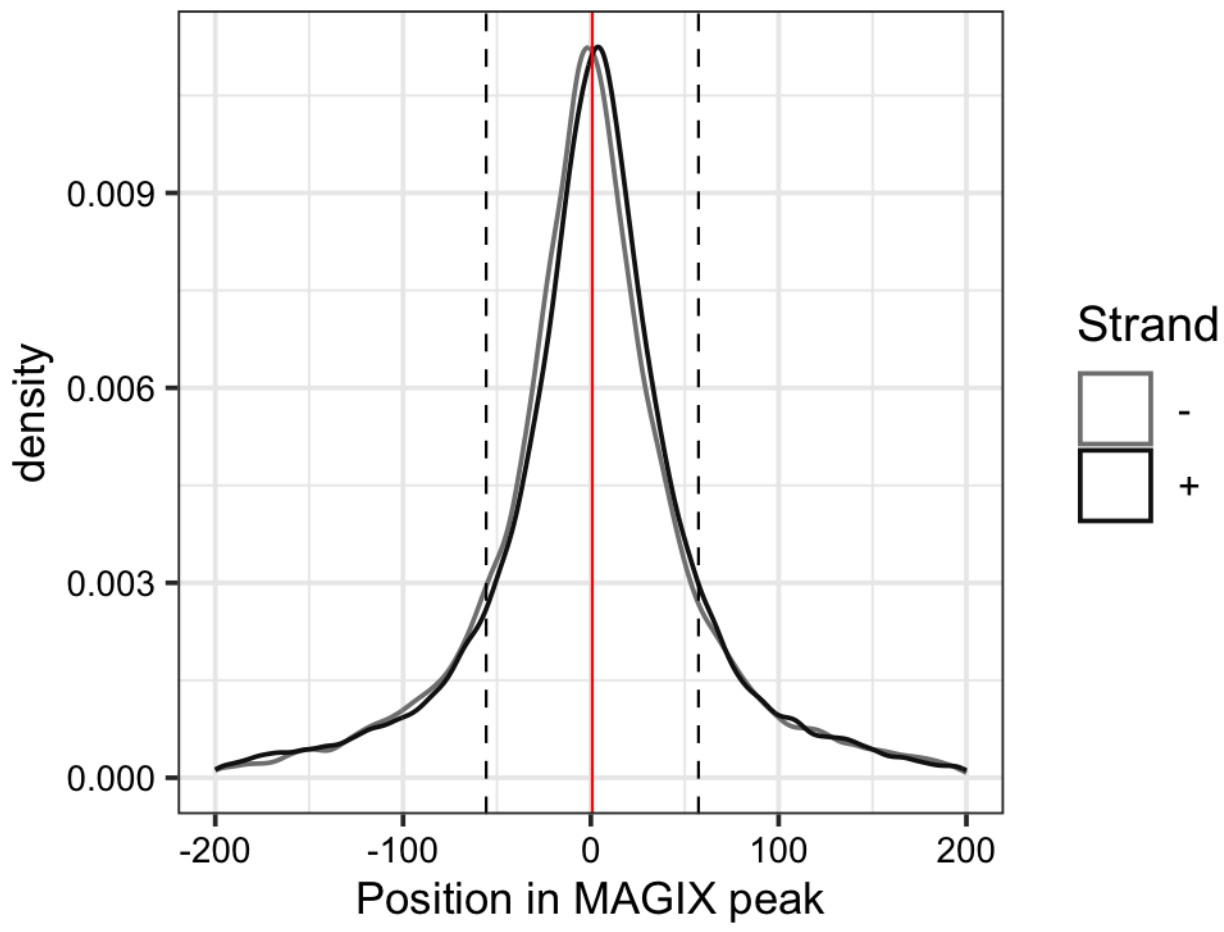

AUROC of top GRECO-BIT/MEX PWM in sliding windows (500 peaks, 50 peak step) along the MAGIX peaks (up to the top 75,000) ranked by the MAGIX enrichment coefficient and fraction of overlapping ChIP peaks in the same window

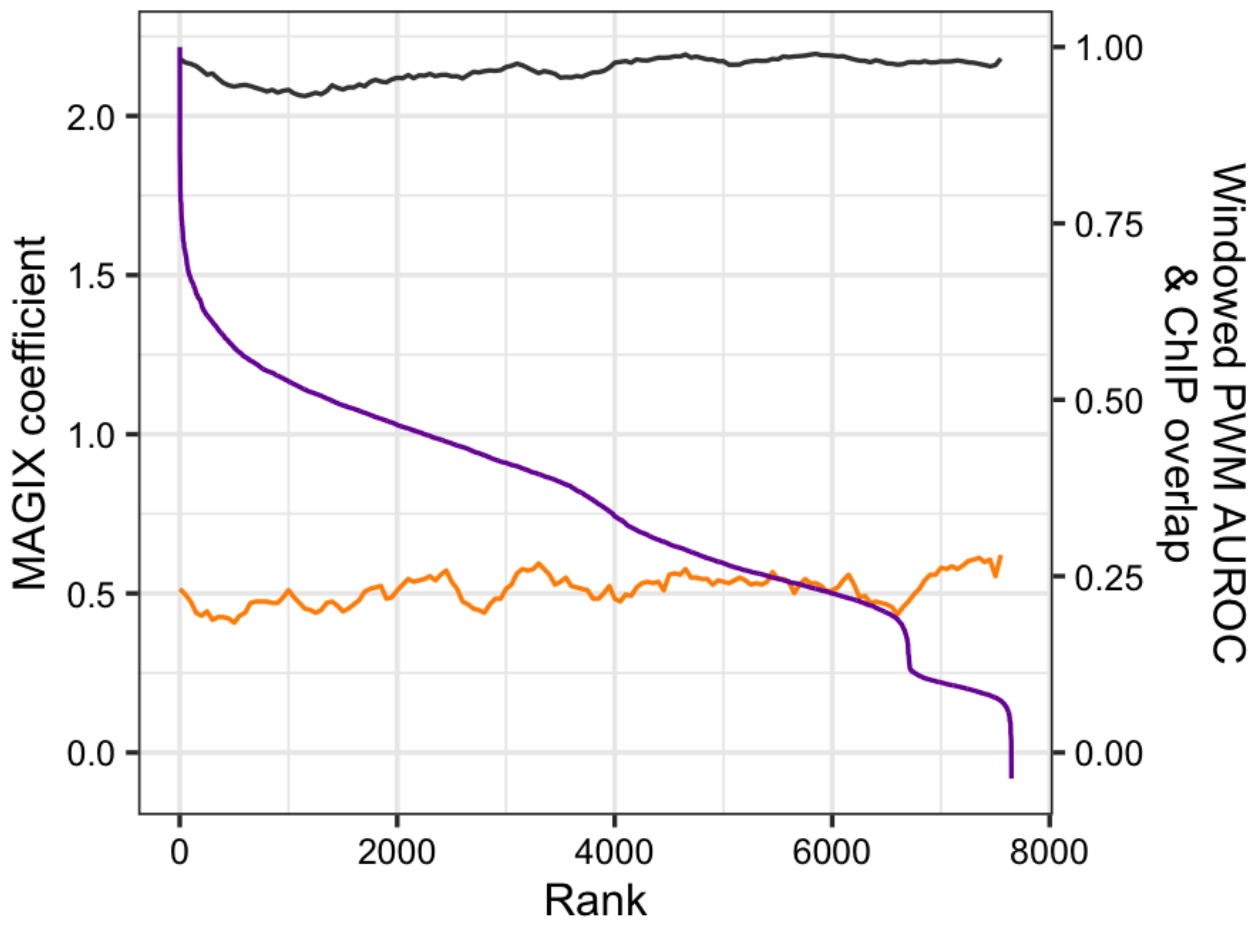

ZNF770

Top GRECO-BIT/MEX PWM: PWM005690

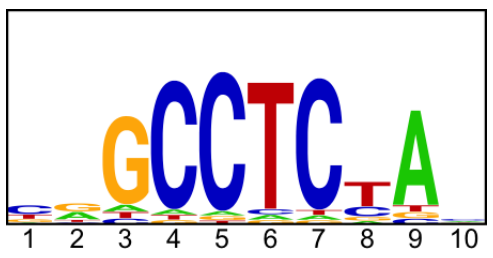

Location of top GRECO-BIT/MEX PWM in MAGIX peaks (up to the top 10,000)

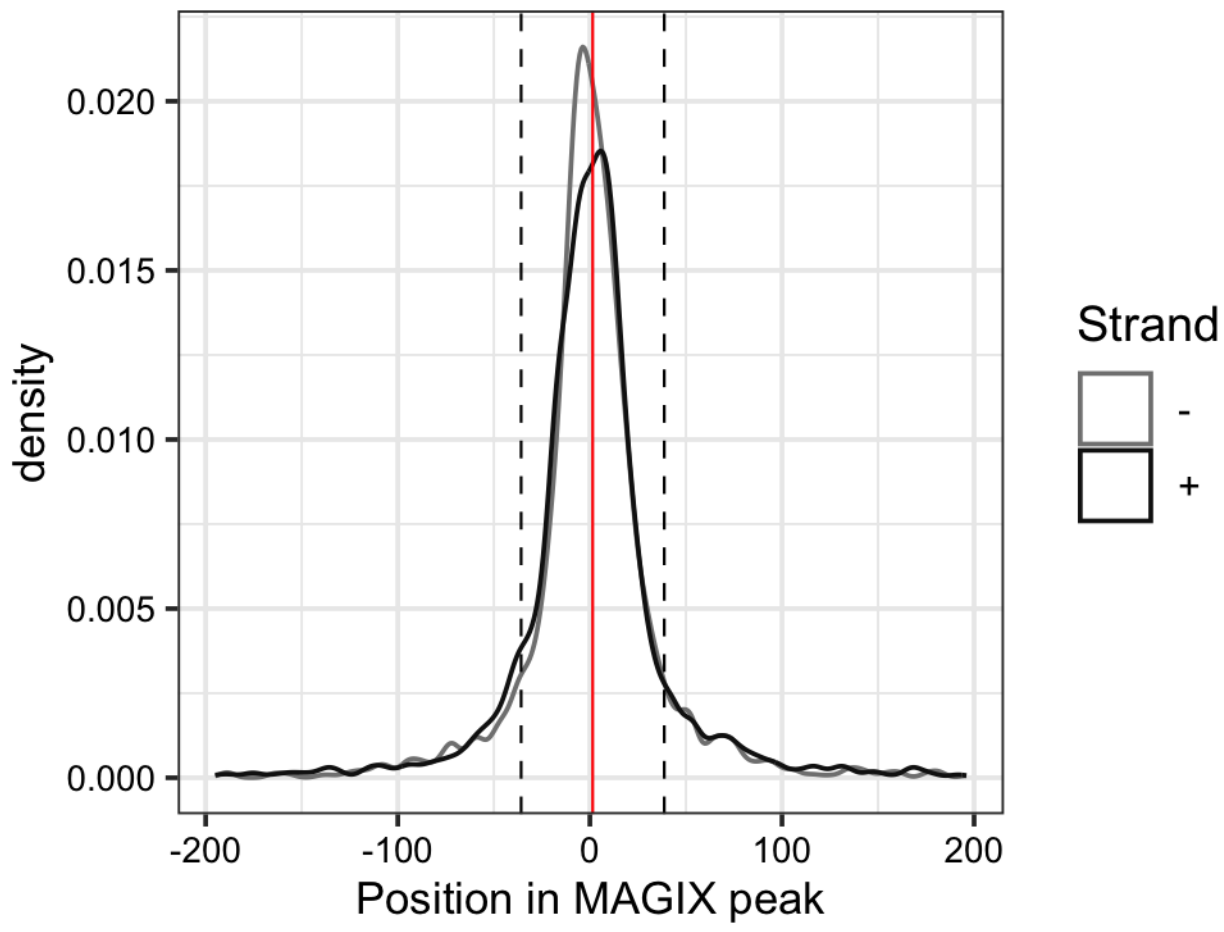

AUROC of top GRECO-BIT/MEX PWM in sliding windows (500 peaks, 50 peak step) along the MAGIX peaks (up to the top 75,000) ranked by the MAGIX enrichment coefficient and fraction of overlapping ChIP peaks in the same window

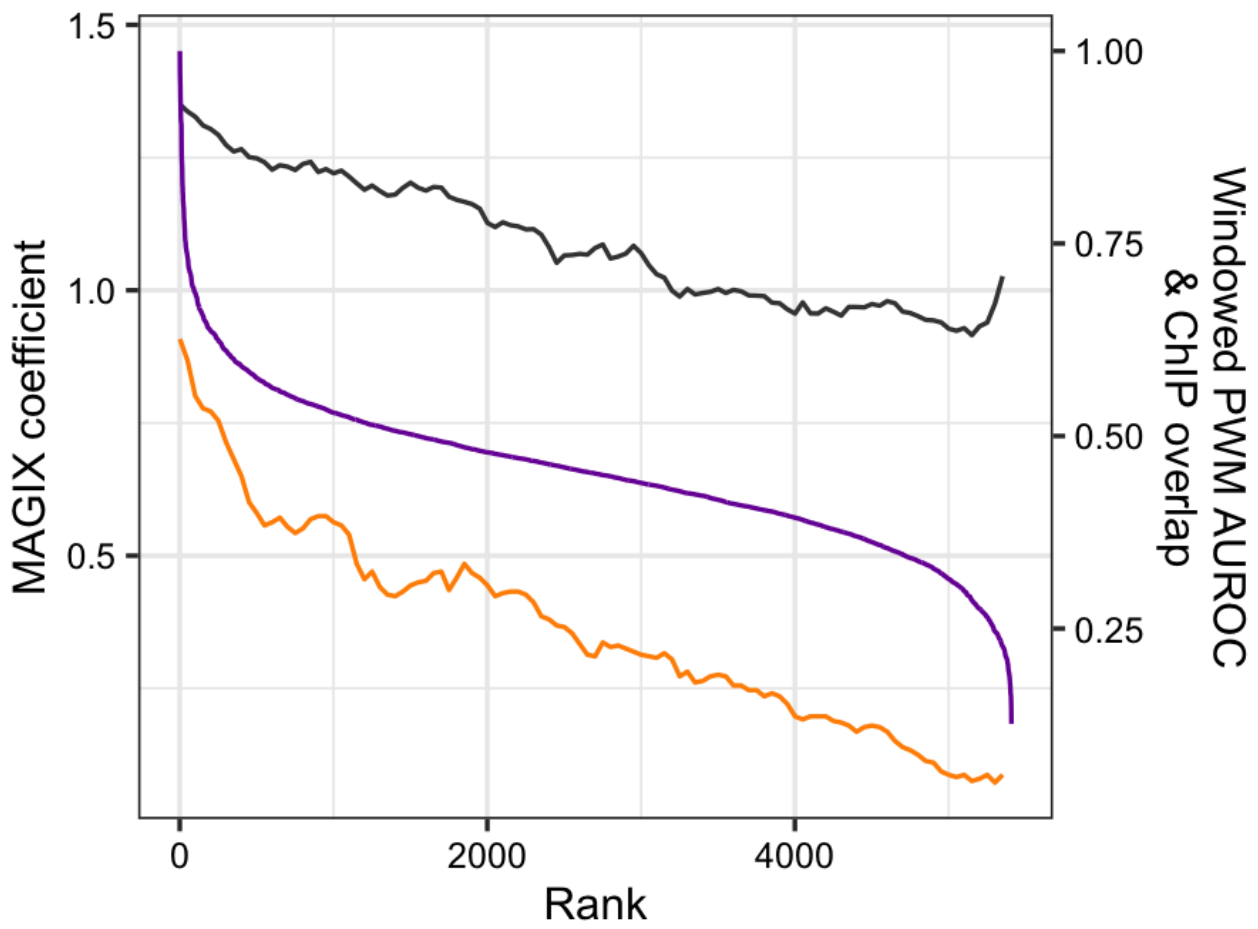

ZNF772

Top GRECO-BIT/MEX PWM: PWM005715

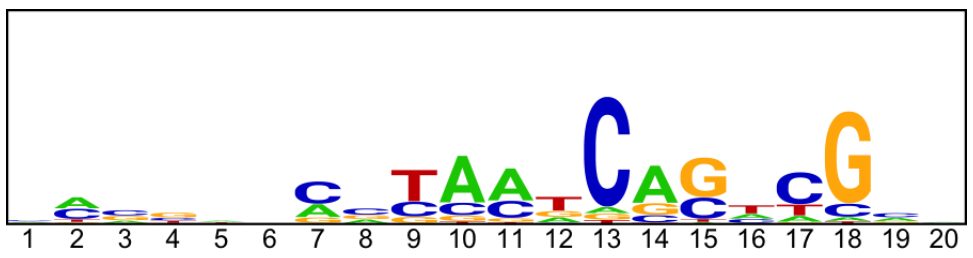

Location of top GRECO-BIT/MEX PWM in MAGIX peaks (up to the top 10,000)

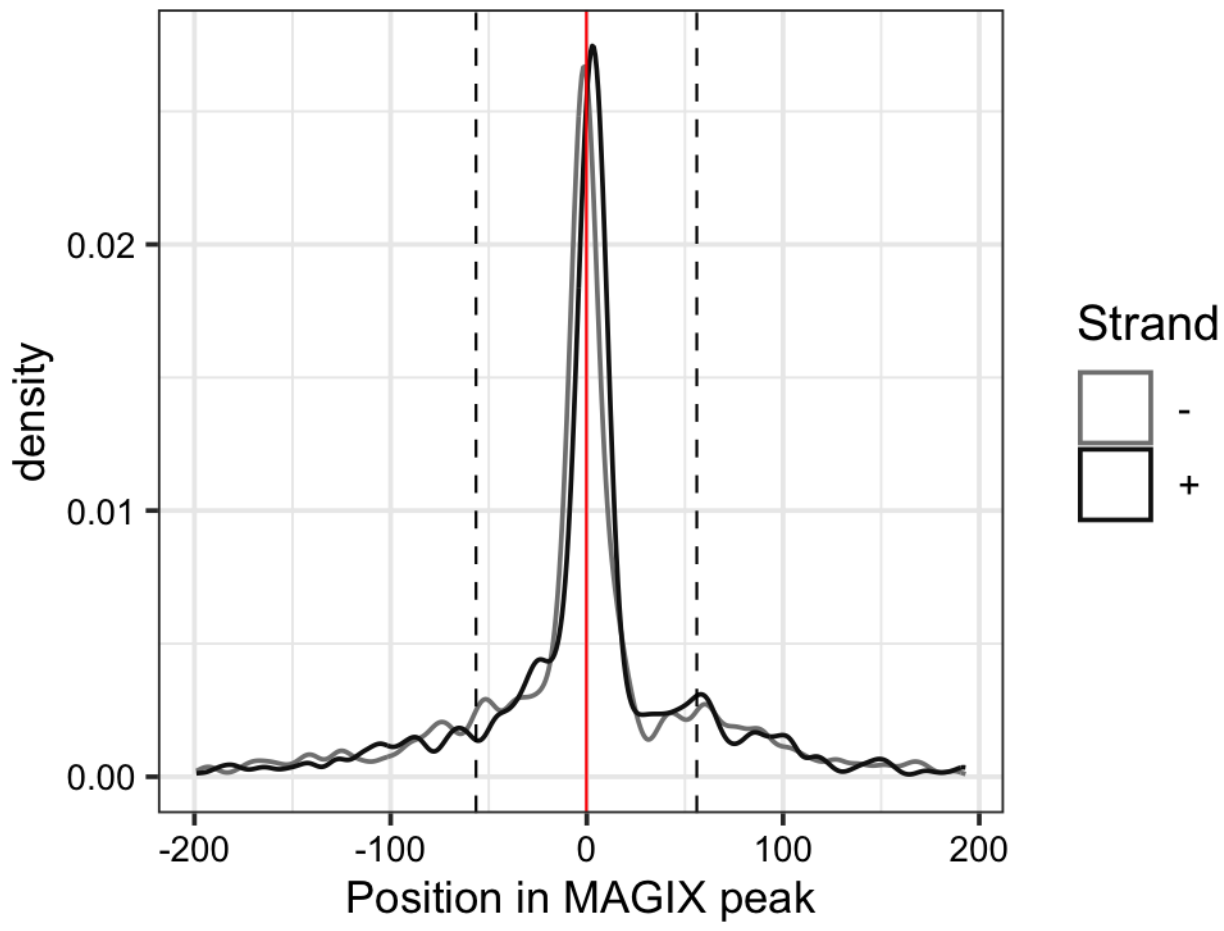

AUROC of top GRECO-BIT/MEX PWM in sliding windows (500 peaks, 50 peak step) along the MAGIX peaks (up to the top 75,000) ranked by the MAGIX enrichment coefficient and fraction of overlapping ChIP peaks in the same window

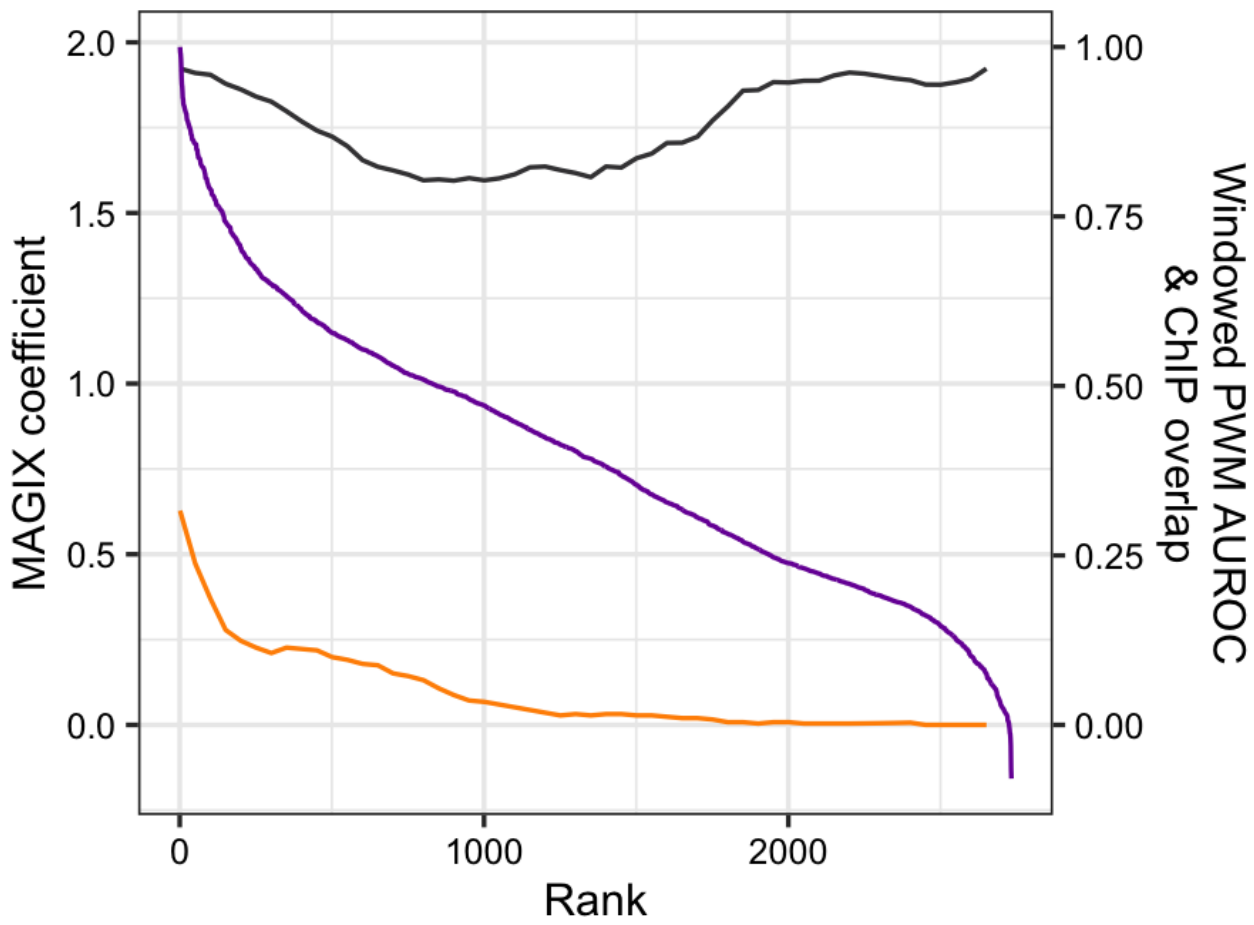

ZNF773

Top GRECO-BIT/MEX PWM: PWM005749

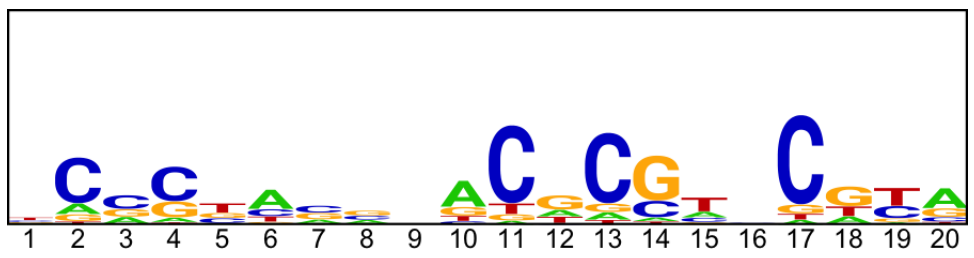

Location of top GRECO-BIT/MEX PWM in MAGIX peaks (up to the top 10,000)

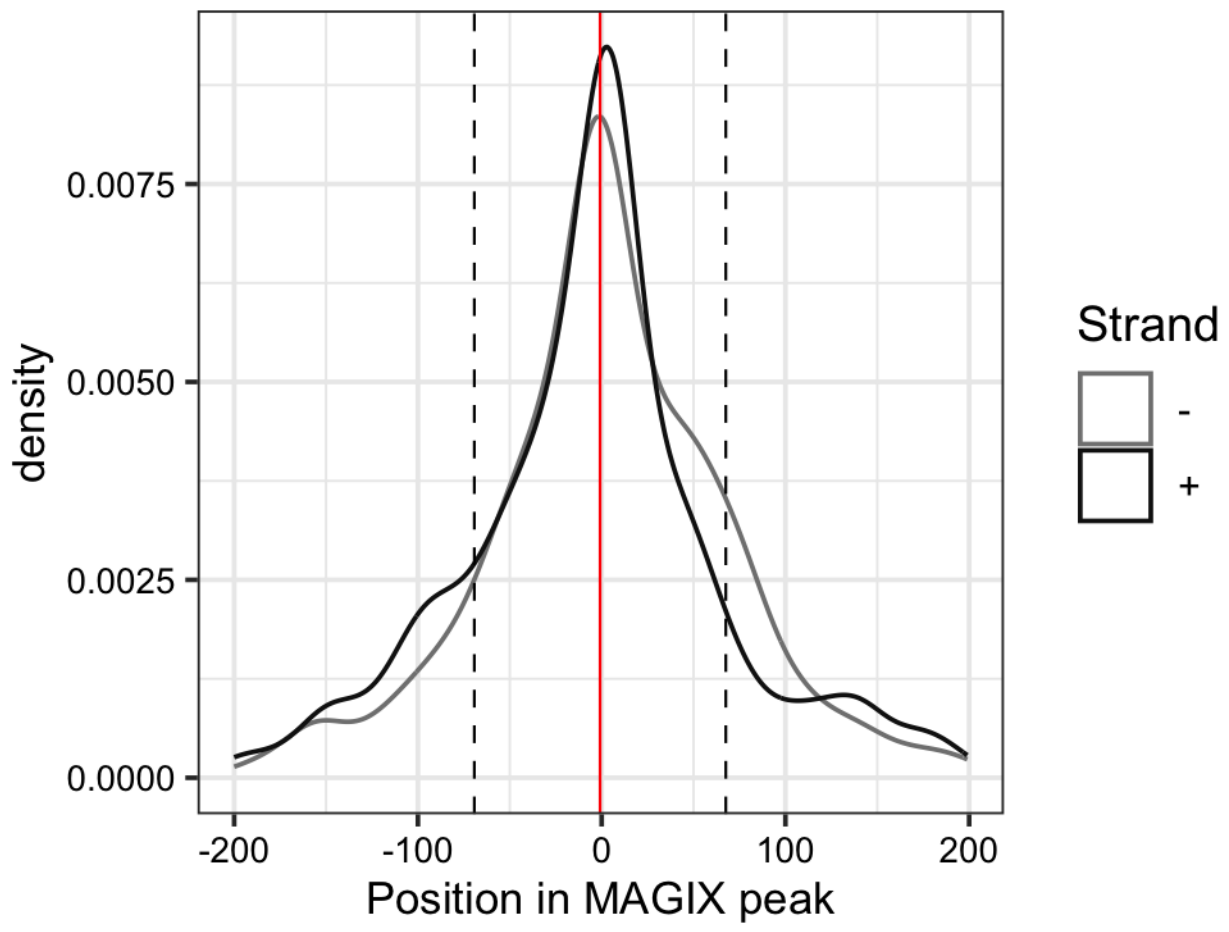

AUROC of top GRECO-BIT/MEX PWM in sliding windows (500 peaks, 50 peak step) along the MAGIX peaks (up to the top 75,000) ranked by the MAGIX enrichment coefficient and fraction of overlapping ChIP peaks in the same window

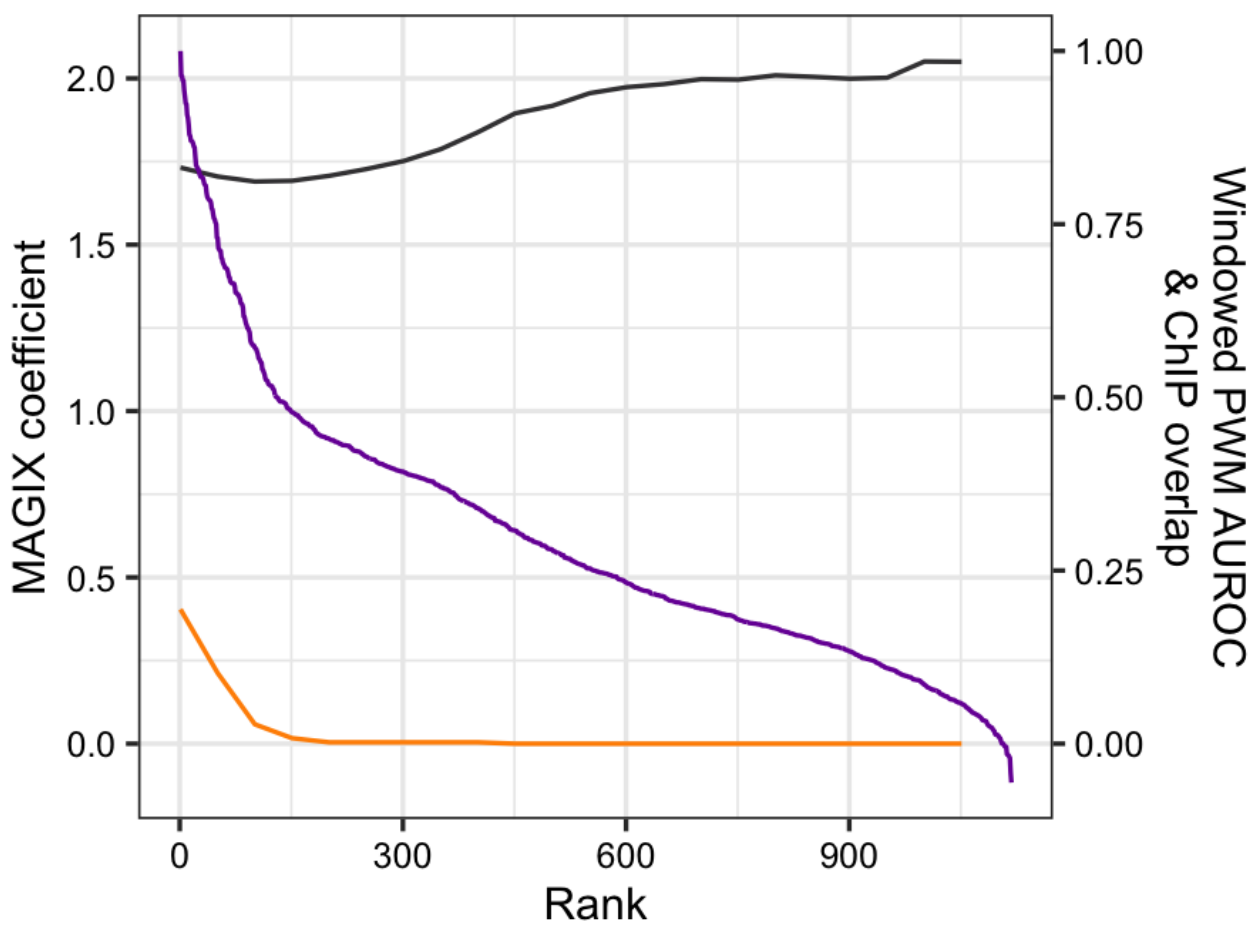

ZNF775

Top GRECO-BIT/MEX PWM: PWM005771

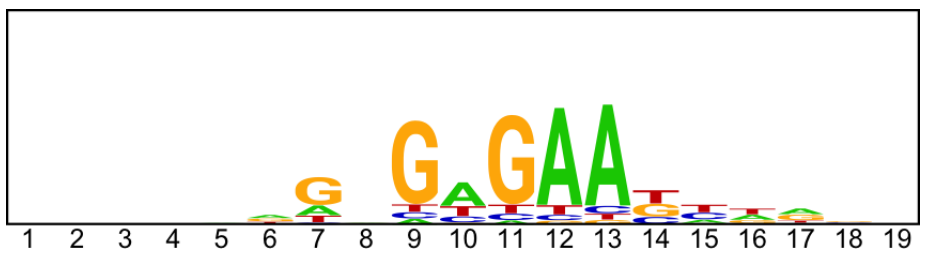

Location of top GRECO-BIT/MEX PWM in MAGIX peaks (up to the top 10,000)

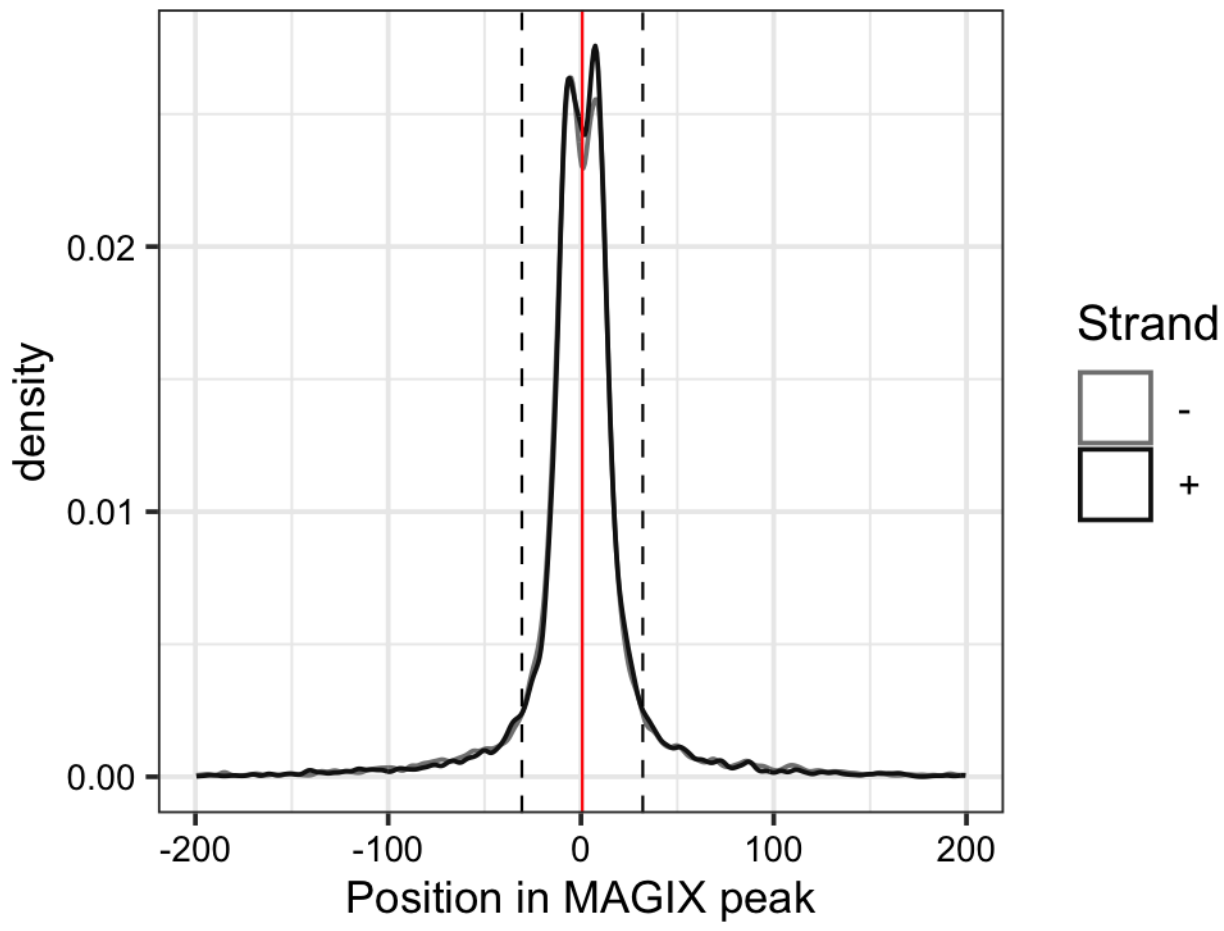

AUROC of top GRECO-BIT/MEX PWM in sliding windows (500 peaks, 50 peak step) along the MAGIX peaks (up to the top 75,000) ranked by the MAGIX enrichment coefficient and fraction of overlapping ChIP peaks in the same window

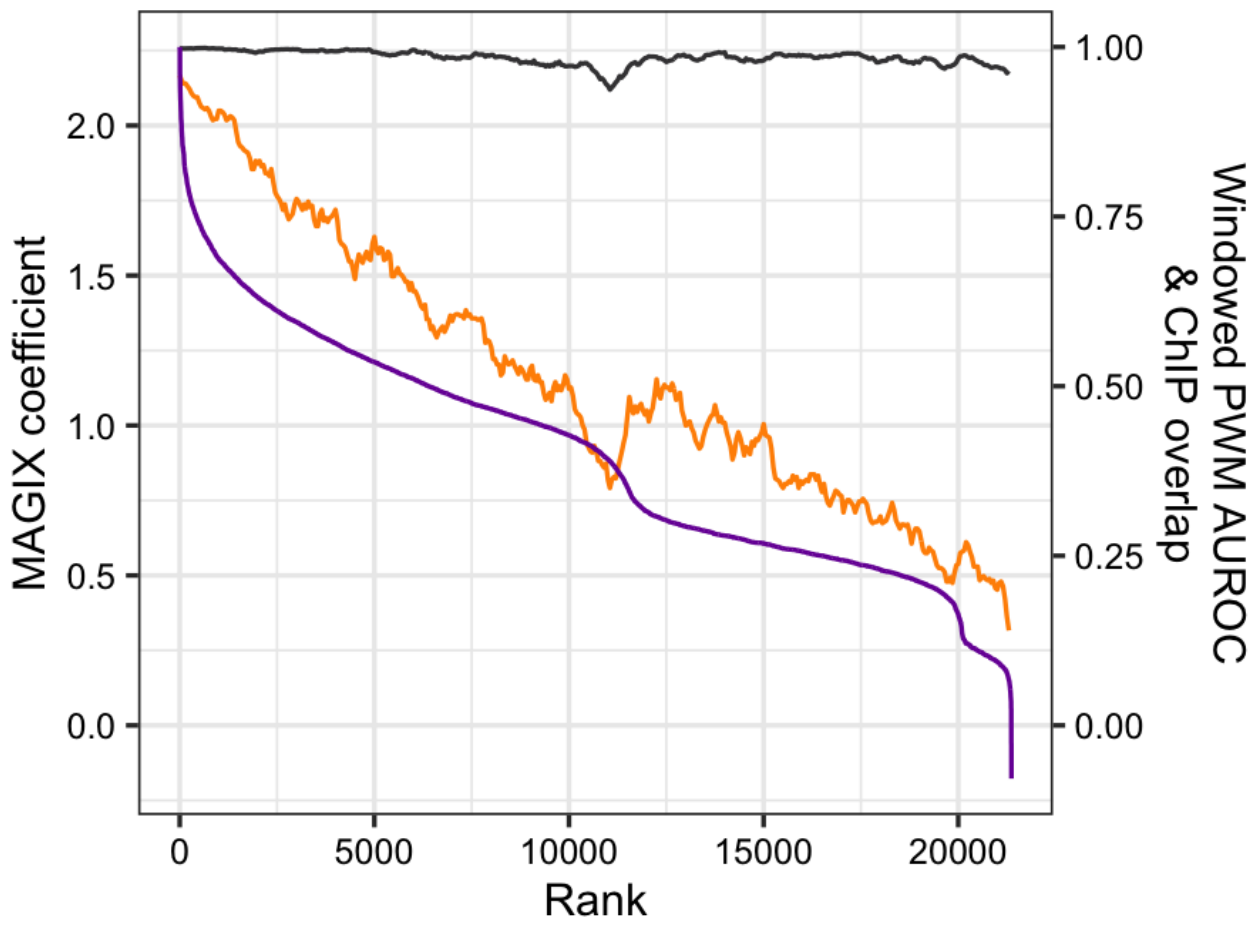

ZNF780B.DBD1

Top GRECO-BIT/MEX PWM: PWM005795

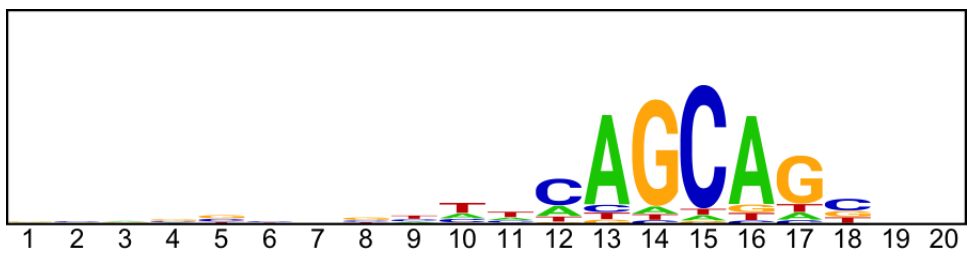

Location of top GRECO-BIT/MEX PWM in MAGIX peaks (up to the top 10,000)

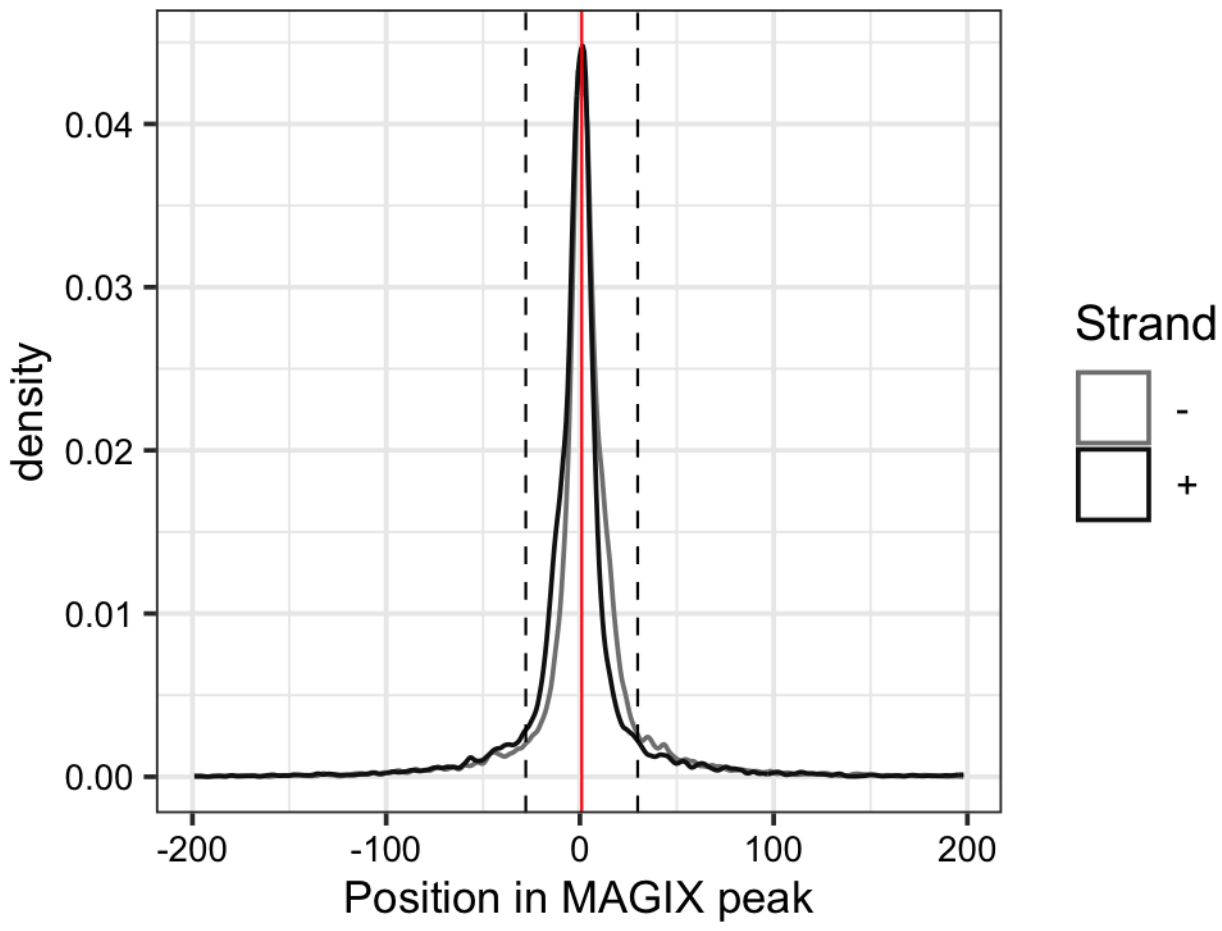

ZNF780B.FL

Top GRECO-BIT/MEX PWM: PWM005796

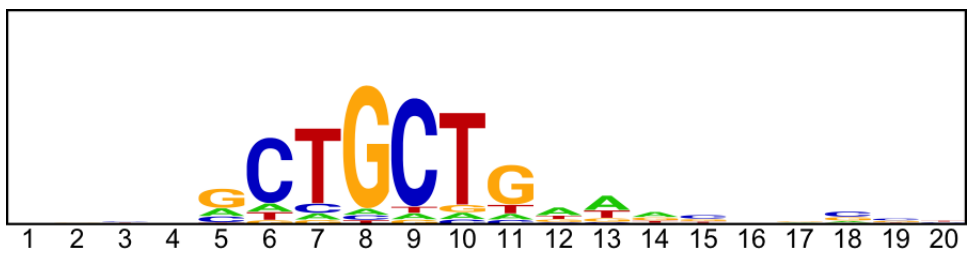

Location of top GRECO-BIT/MEX PWM in MAGIX peaks (up to the top 10,000)

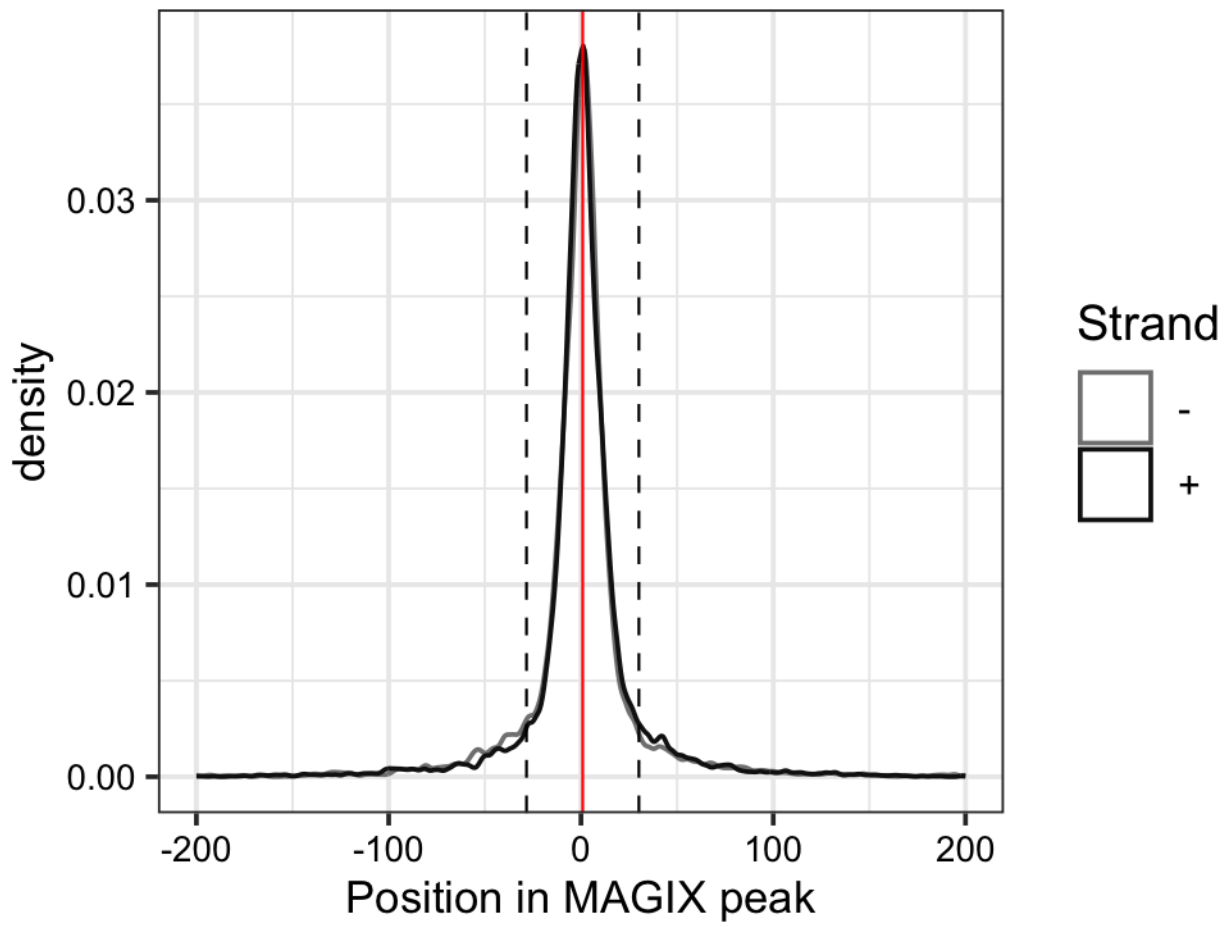

AUROC of top GRECO-BIT/MEX PWM in sliding windows (500 peaks, 50 peak step) along the MAGIX peaks (up to the top 75,000) ranked by the MAGIX enrichment coefficient and fraction of overlapping ChIP peaks in the same window

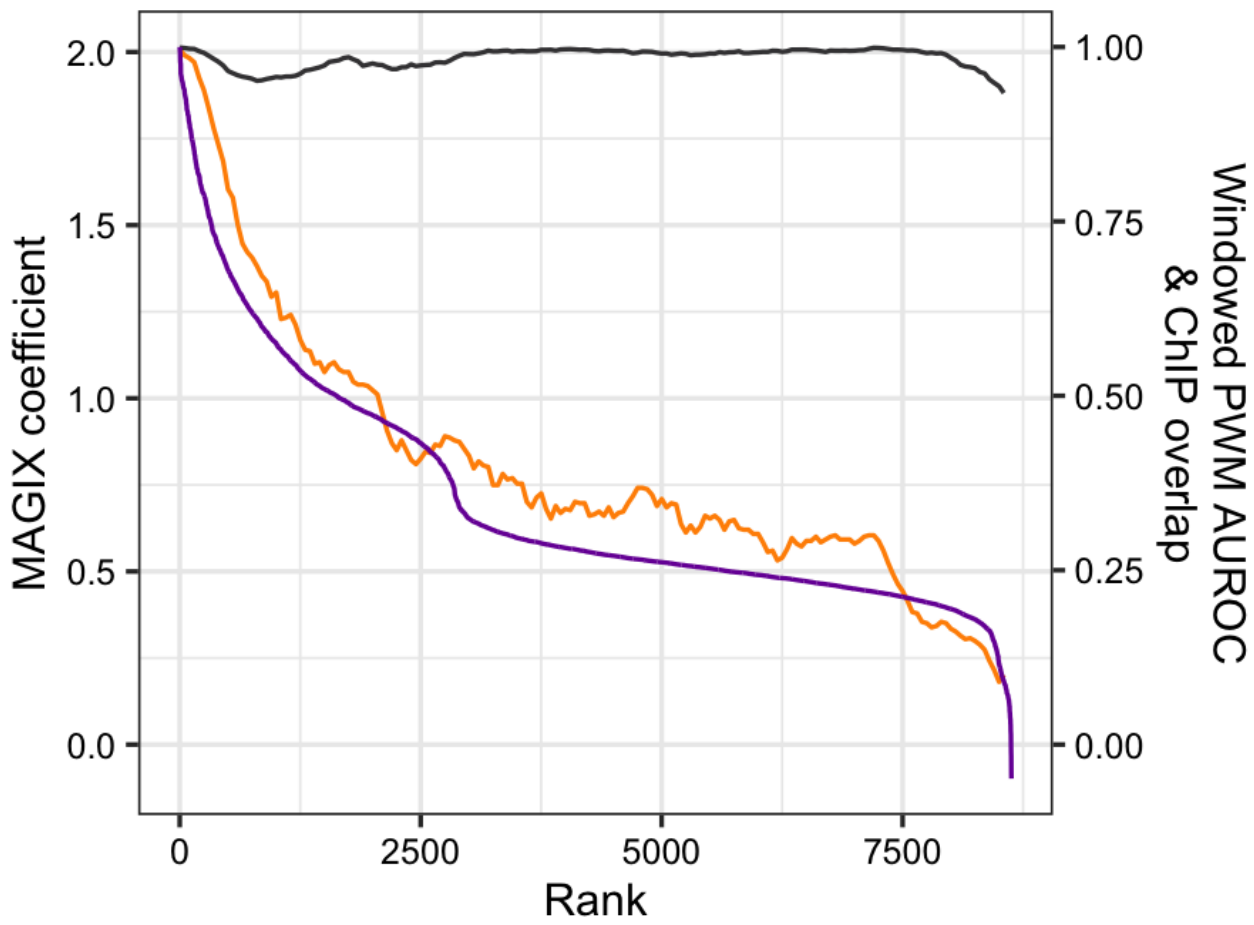

ZNF800

Top GRECO-BIT/MEX PWM: PWM005848

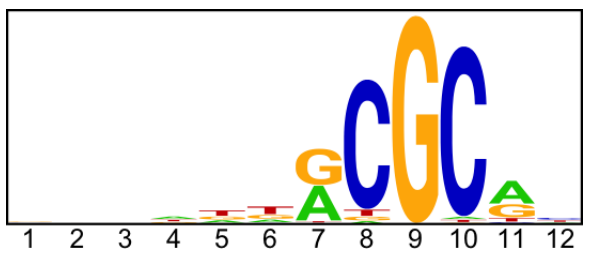

Location of top GRECO-BIT/MEX PWM in MAGIX peaks (up to the top 10,000)

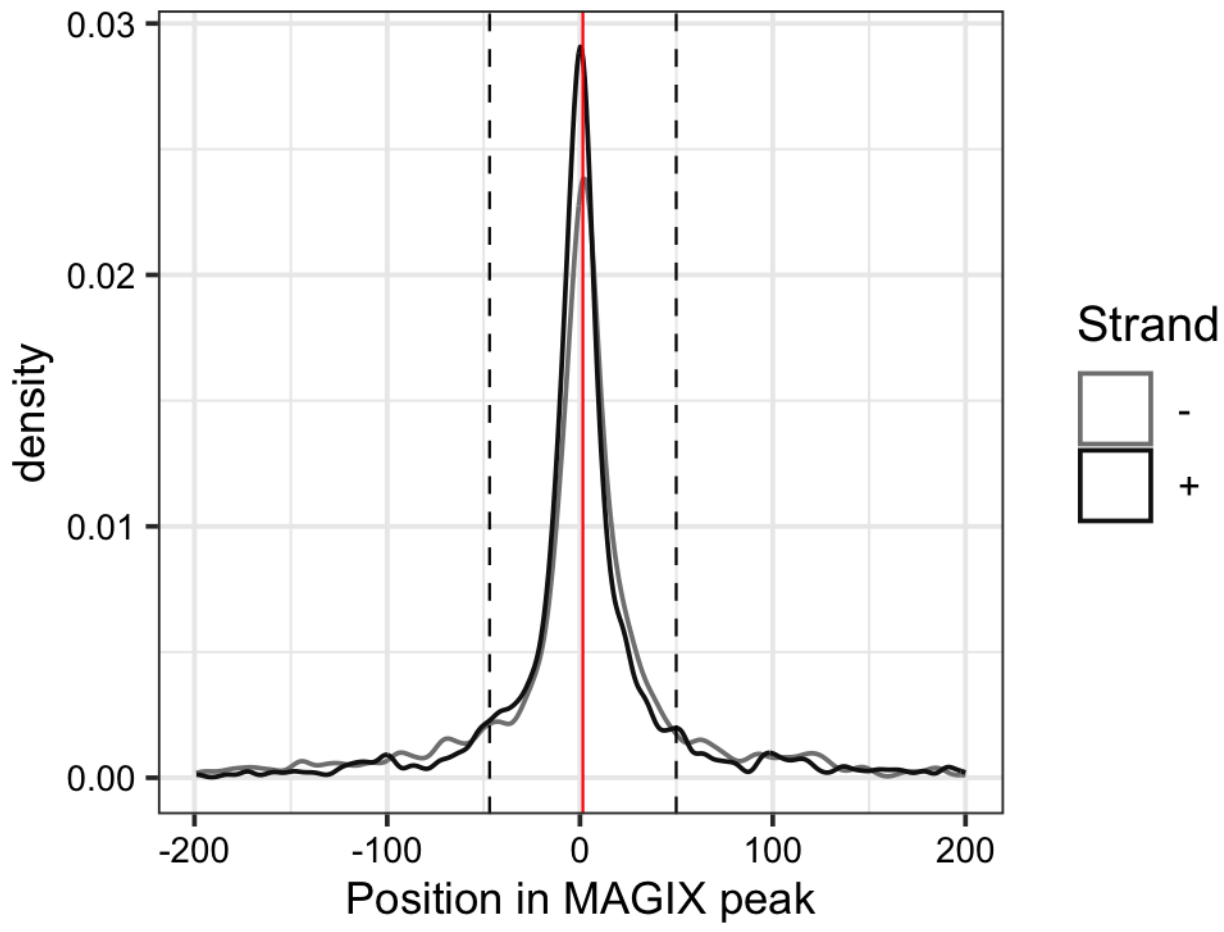

AUROC of top GRECO-BIT/MEX PWM in sliding windows (500 peaks, 50 peak step) along the MAGIX peaks (up to the top 75,000) ranked by the MAGIX enrichment coefficient and fraction of overlapping ChIP peaks in the same window

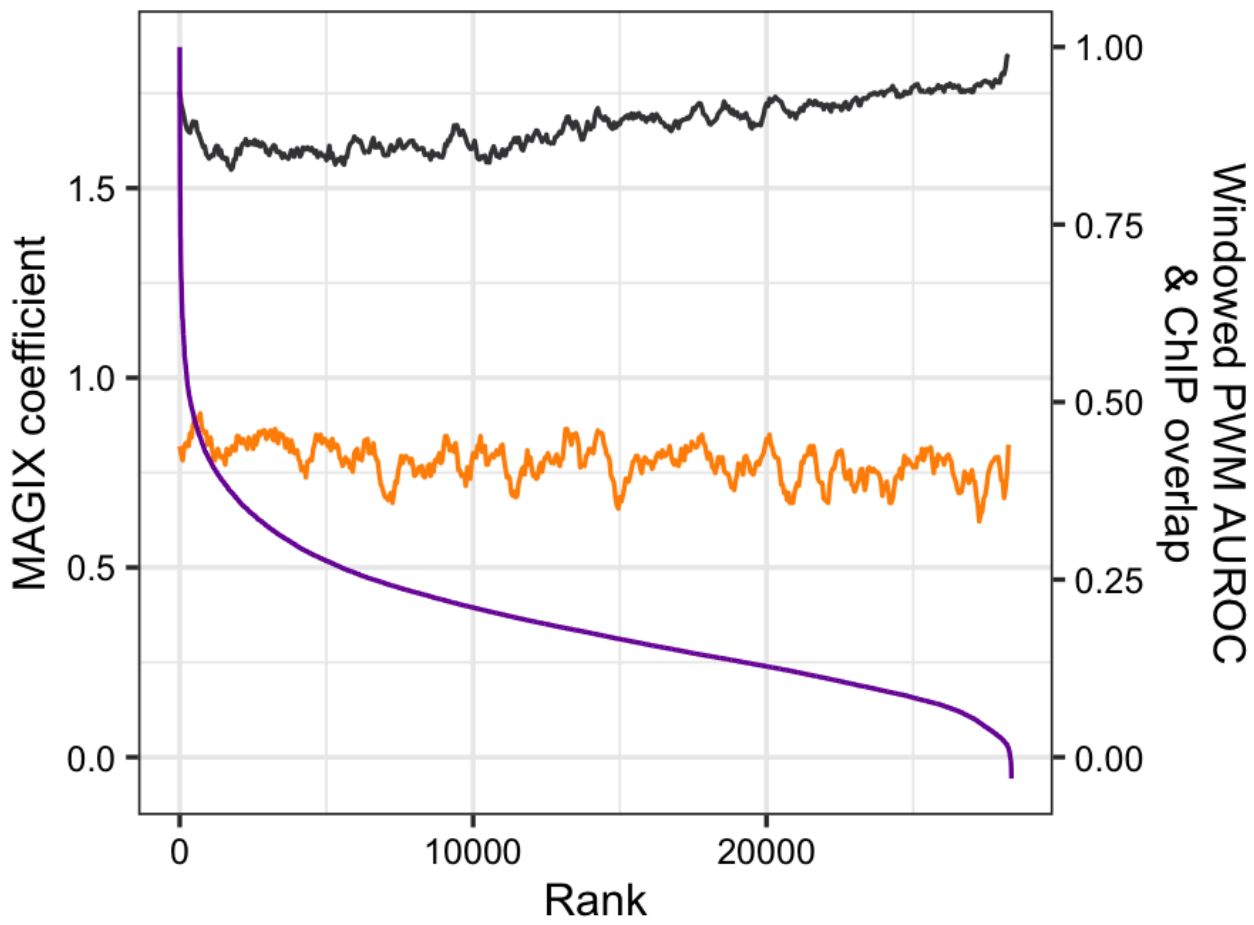

ZNF813

Top GRECO-BIT/MEX PWM: PWM005866

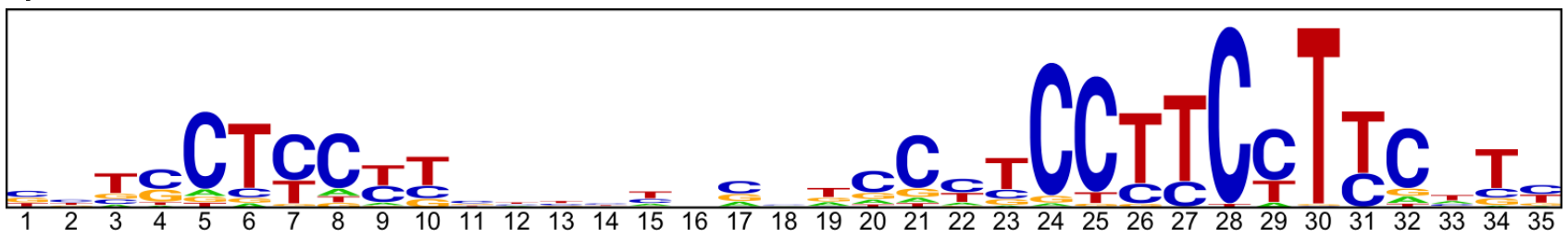

Location of top GRECO-BIT/MEX PWM in MAGIX peaks (up to the top 10,000)

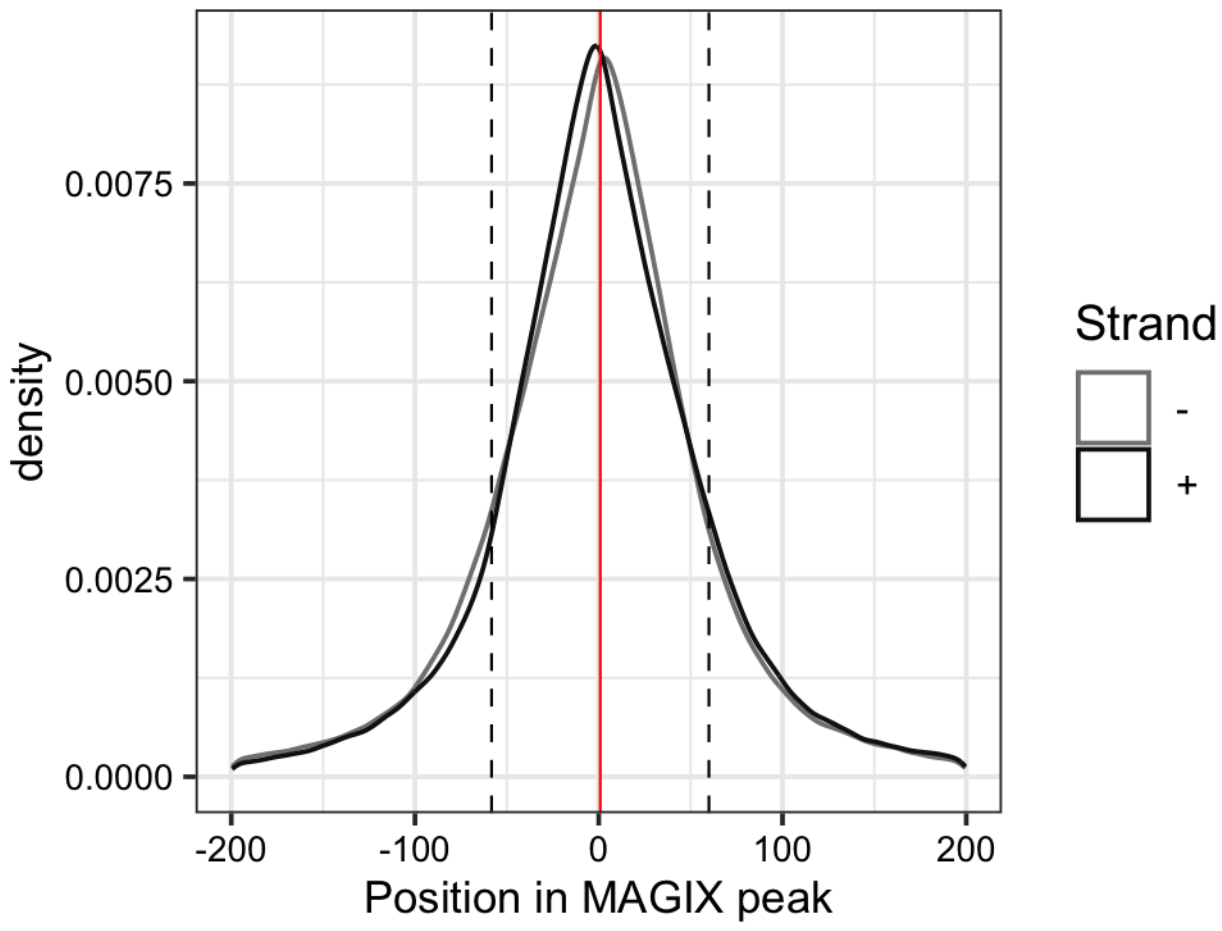

ZNF814

Top GRECO-BIT/MEX PWM: PWM005898

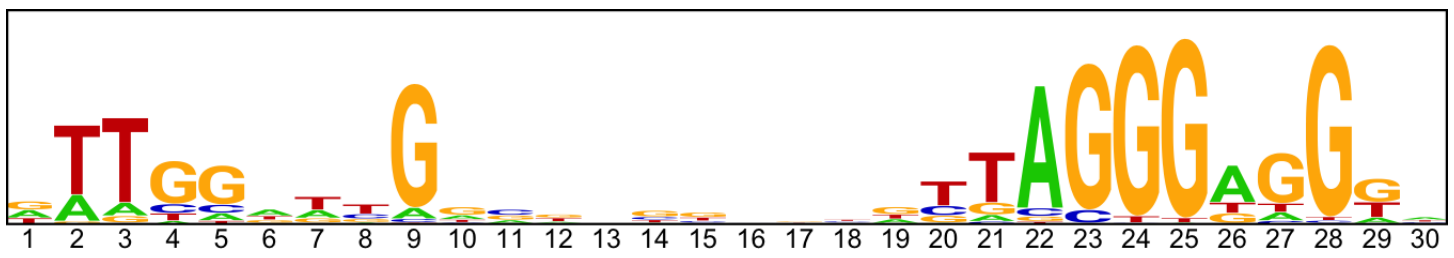

Location of top GRECO-BIT/MEX PWM in MAGIX peaks (up to the top 10,000)

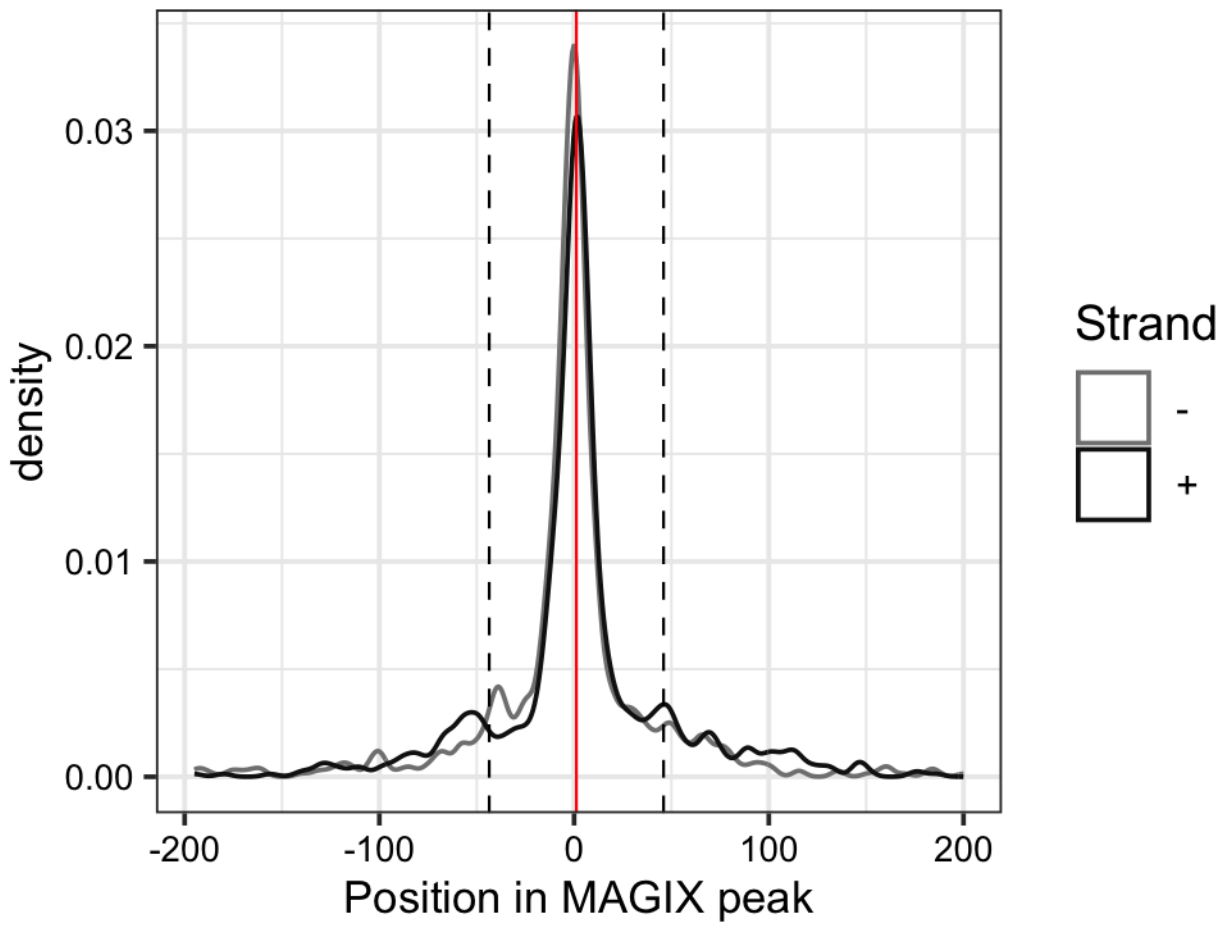

AUROC of top GRECO-BIT/MEX PWM in sliding windows (500 peaks, 50 peak step) along the MAGIX peaks (up to the top 75,000) ranked by the MAGIX enrichment coefficient and fraction of overlapping ChIP peaks in the same window

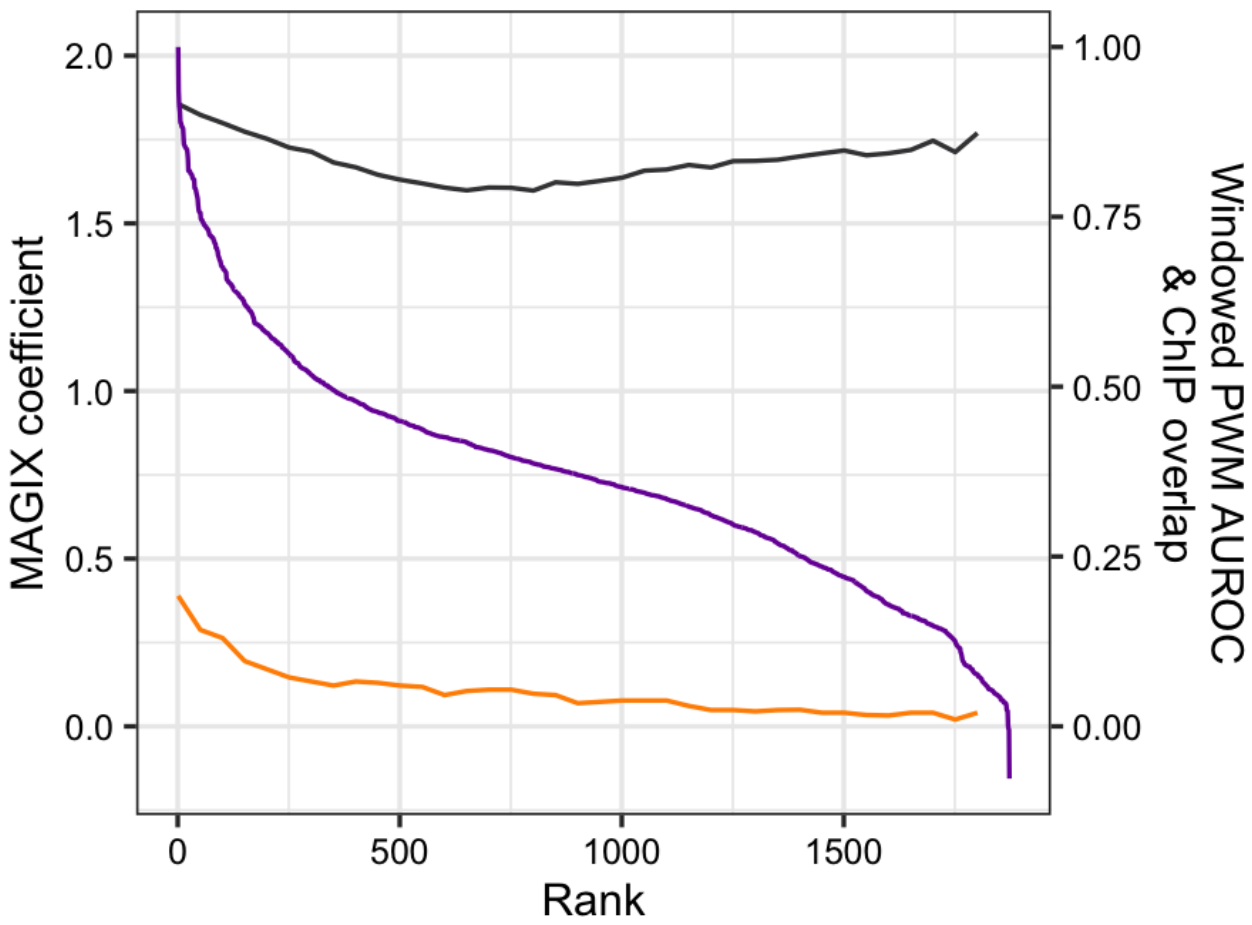

ZNF827.DBD1

Top GRECO-BIT/MEX PWM: PWM005918

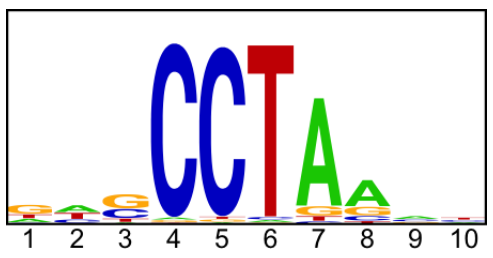

Location of top GRECO-BIT/MEX PWM in MAGIX peaks (up to the top 10,000)

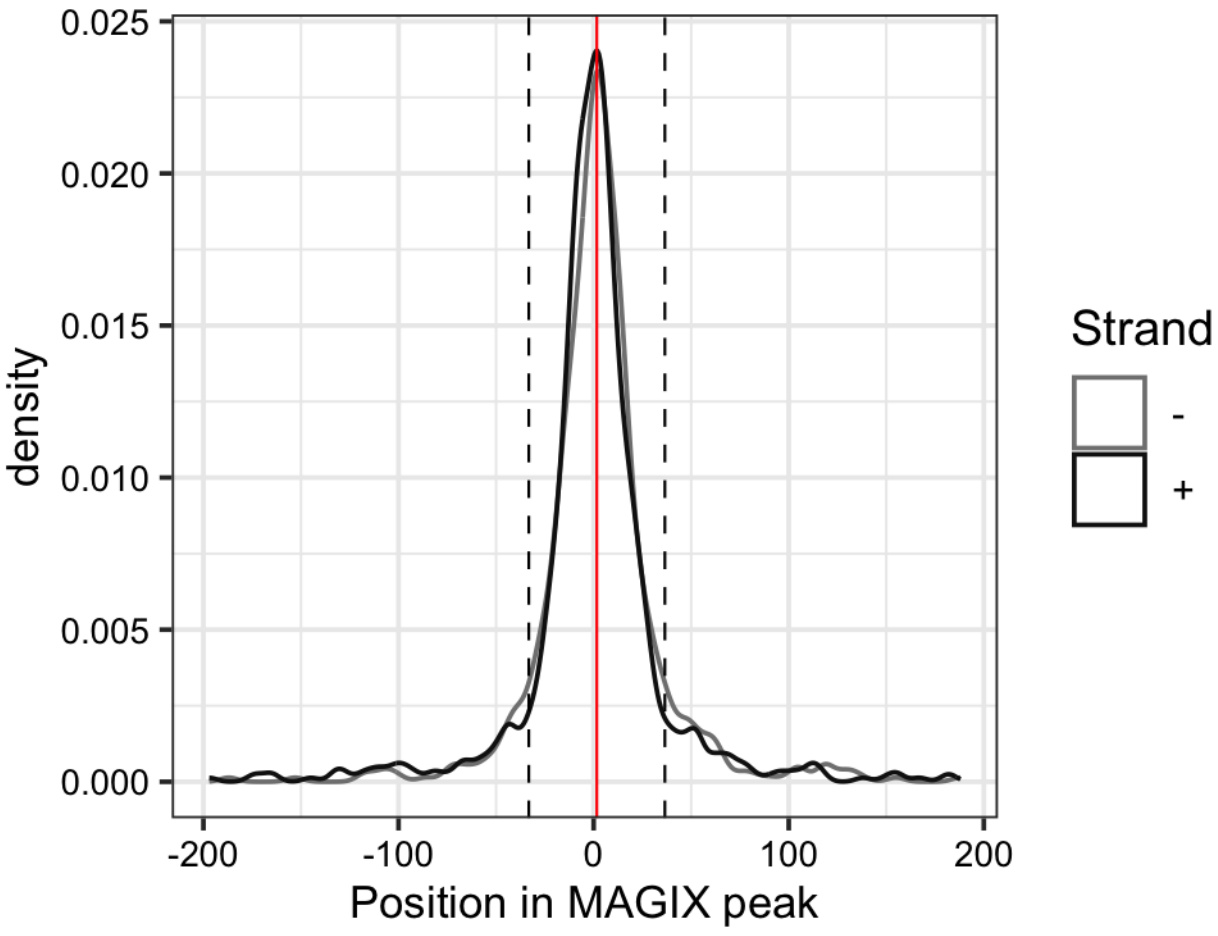

ZNF83

Top GRECO-BIT/MEX PWM: PWM005938

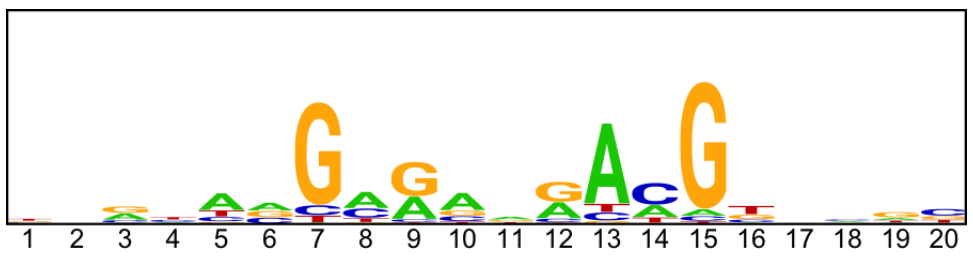

Location of top GRECO-BIT/MEX PWM in MAGIX peaks (up to the top 10,000)

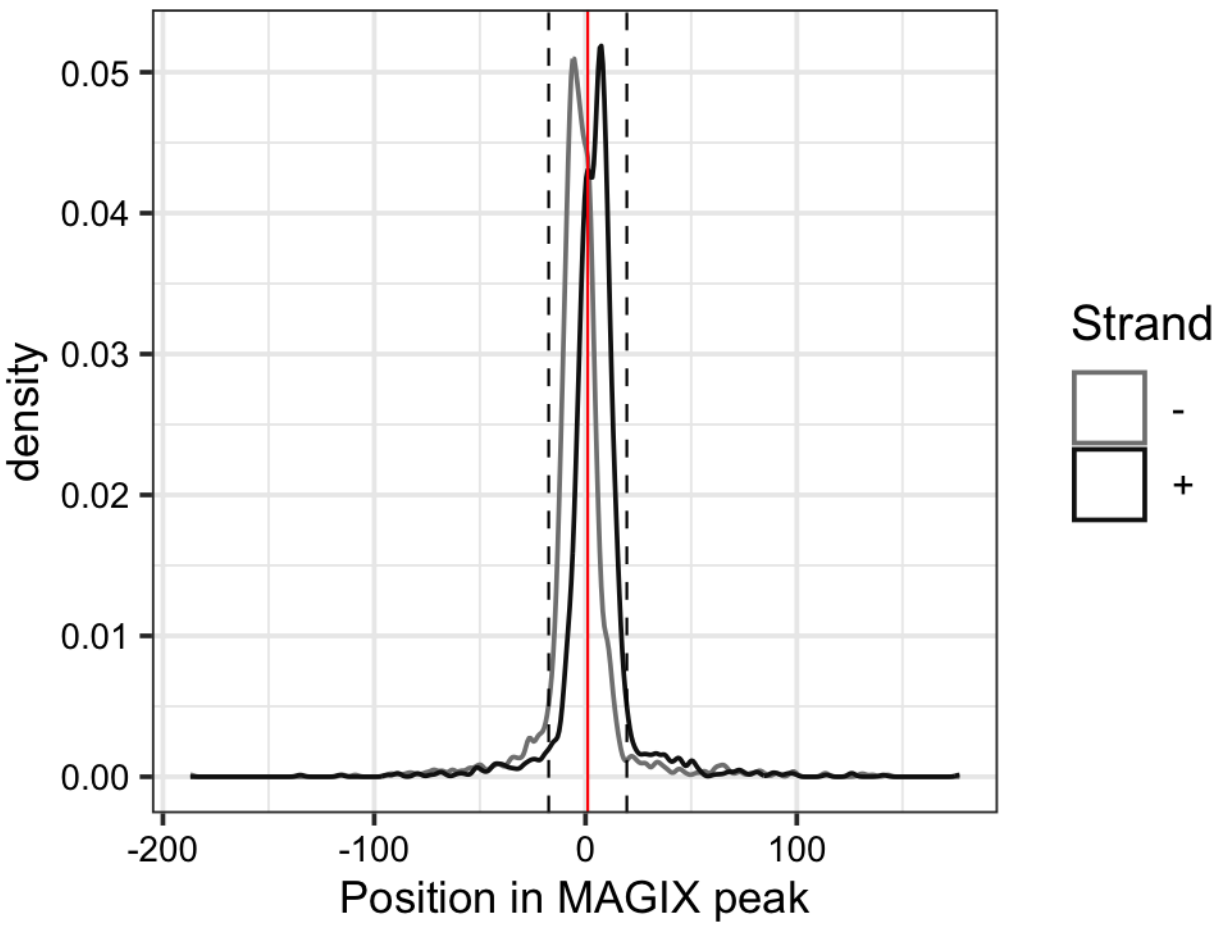

ZNF831

Top GRECO-BIT/MEX PWM: PWM005969

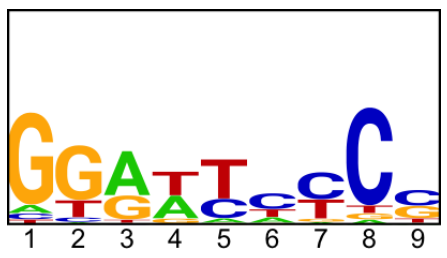

Location of top GRECO-BIT/MEX PWM in MAGIX peaks (up to the top 10,000)

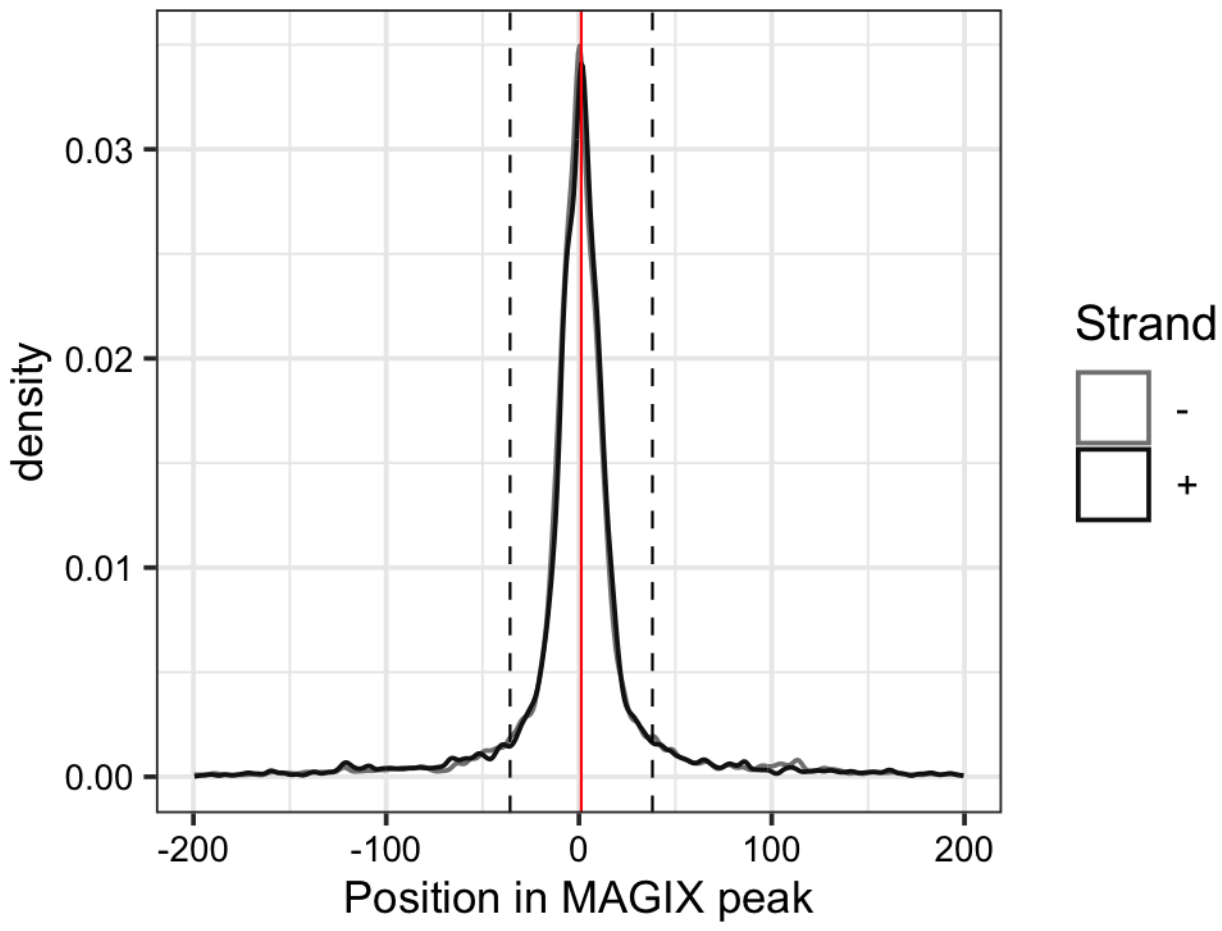

AUROC of top GRECO-BIT/MEX PWM in sliding windows (500 peaks, 50 peak step) along the MAGIX peaks (up to the top 75,000) ranked by the MAGIX enrichment coefficient and fraction of overlapping ChIP peaks in the same window

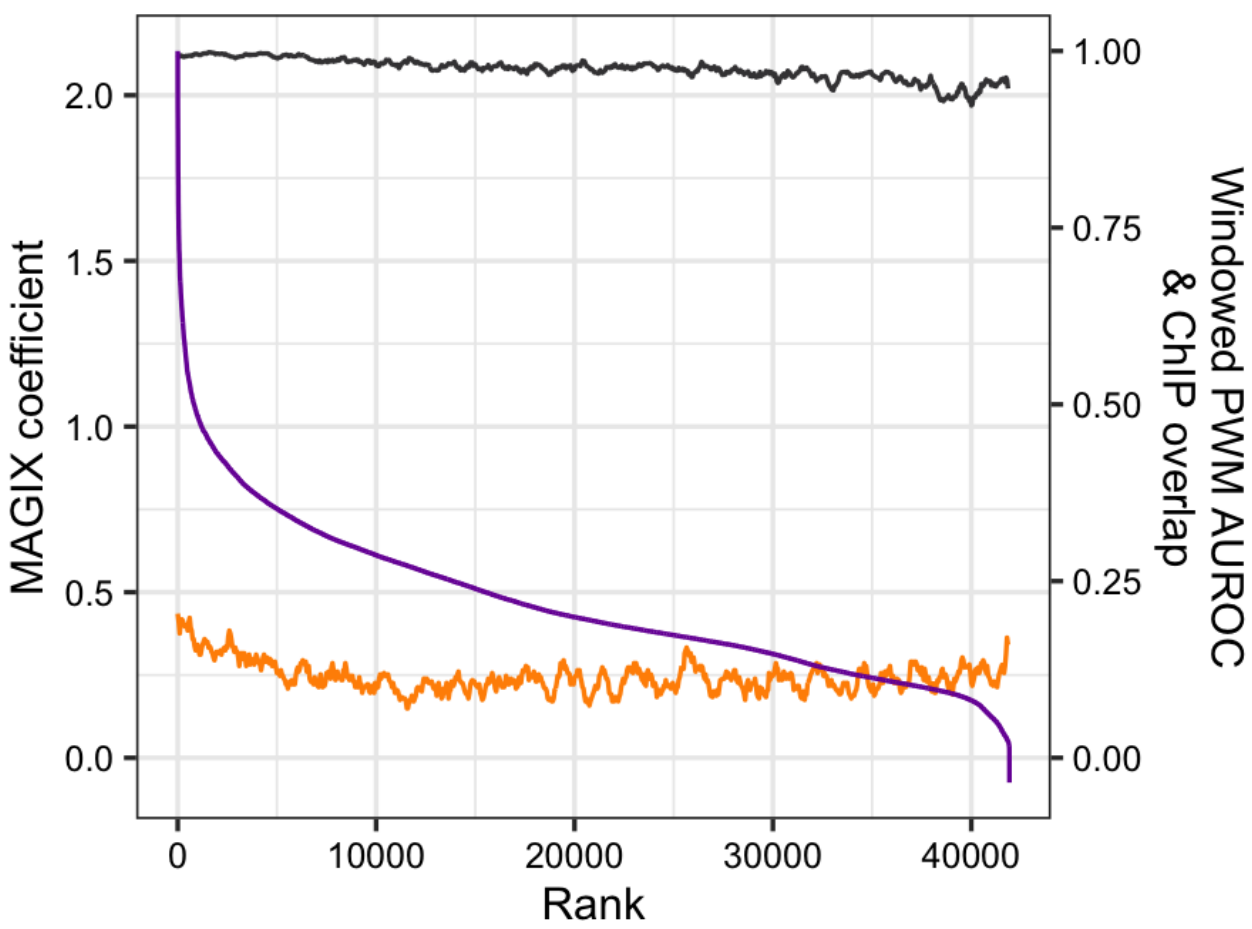

ZNF836.DBD1

Top GRECO-BIT/MEX PWM: PWM190008

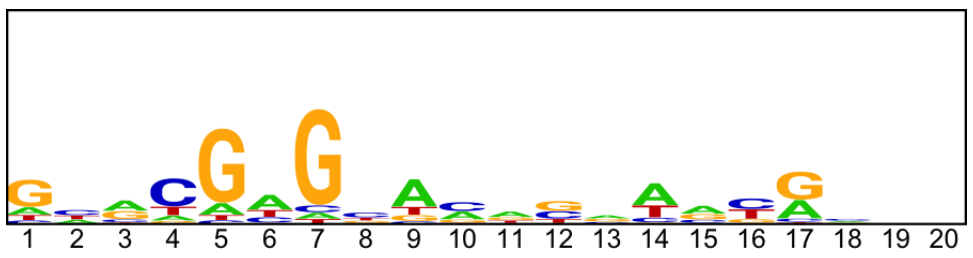

Location of top GRECO-BIT/MEX PWM in MAGIX peaks (up to the top 10,000)

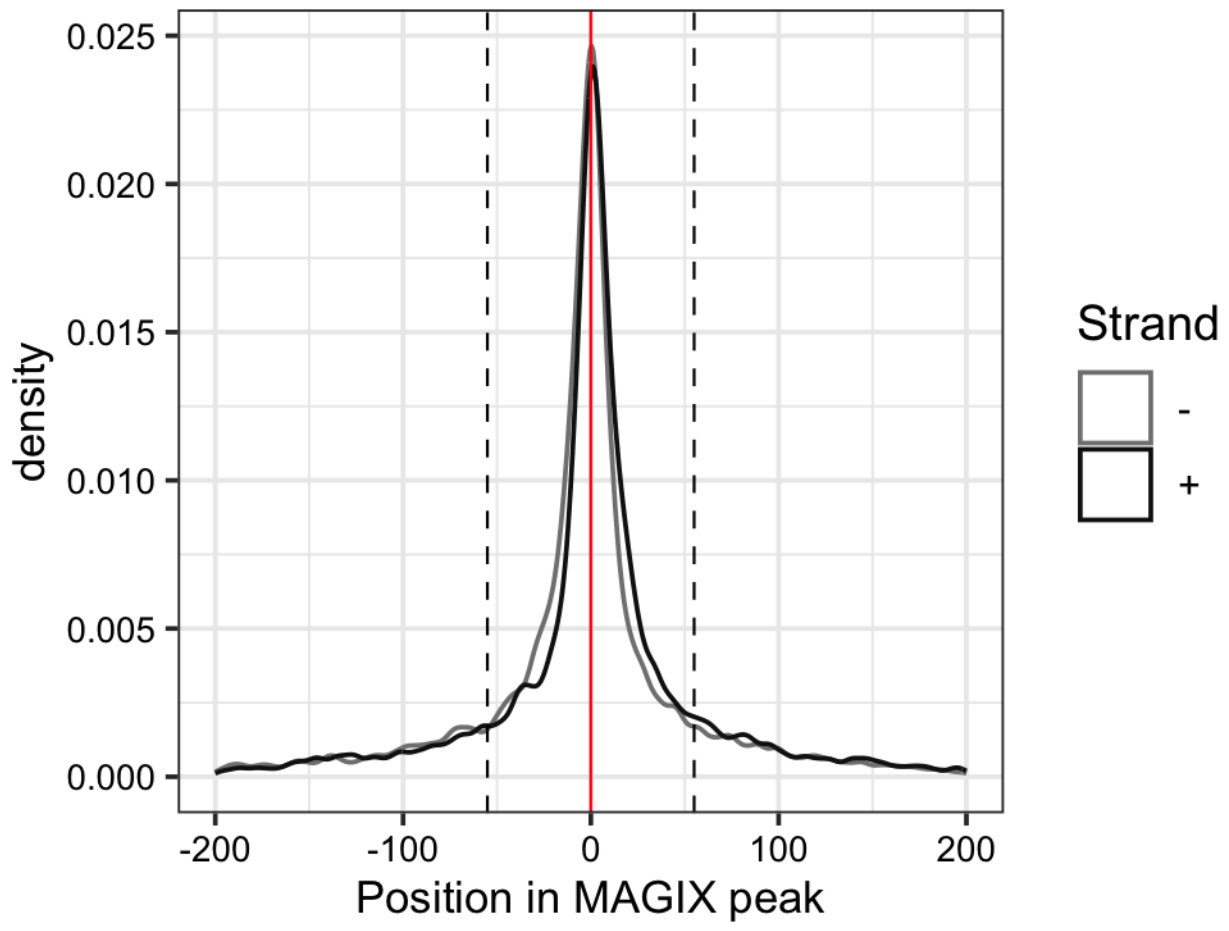

ZNF836.DBD2

Top GRECO-BIT/MEX PWM: PWM006000

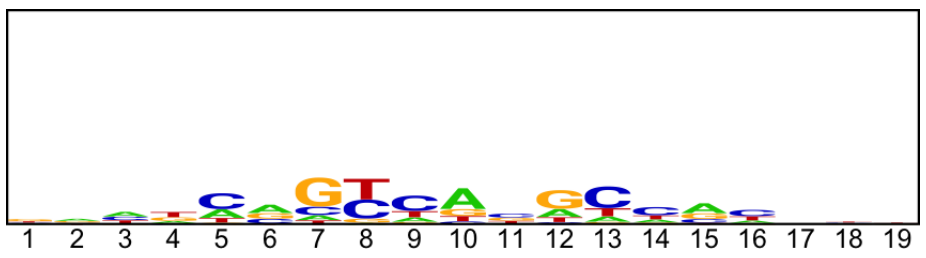

Location of top GRECO-BIT/MEX PWM in MAGIX peaks (up to the top 10,000)

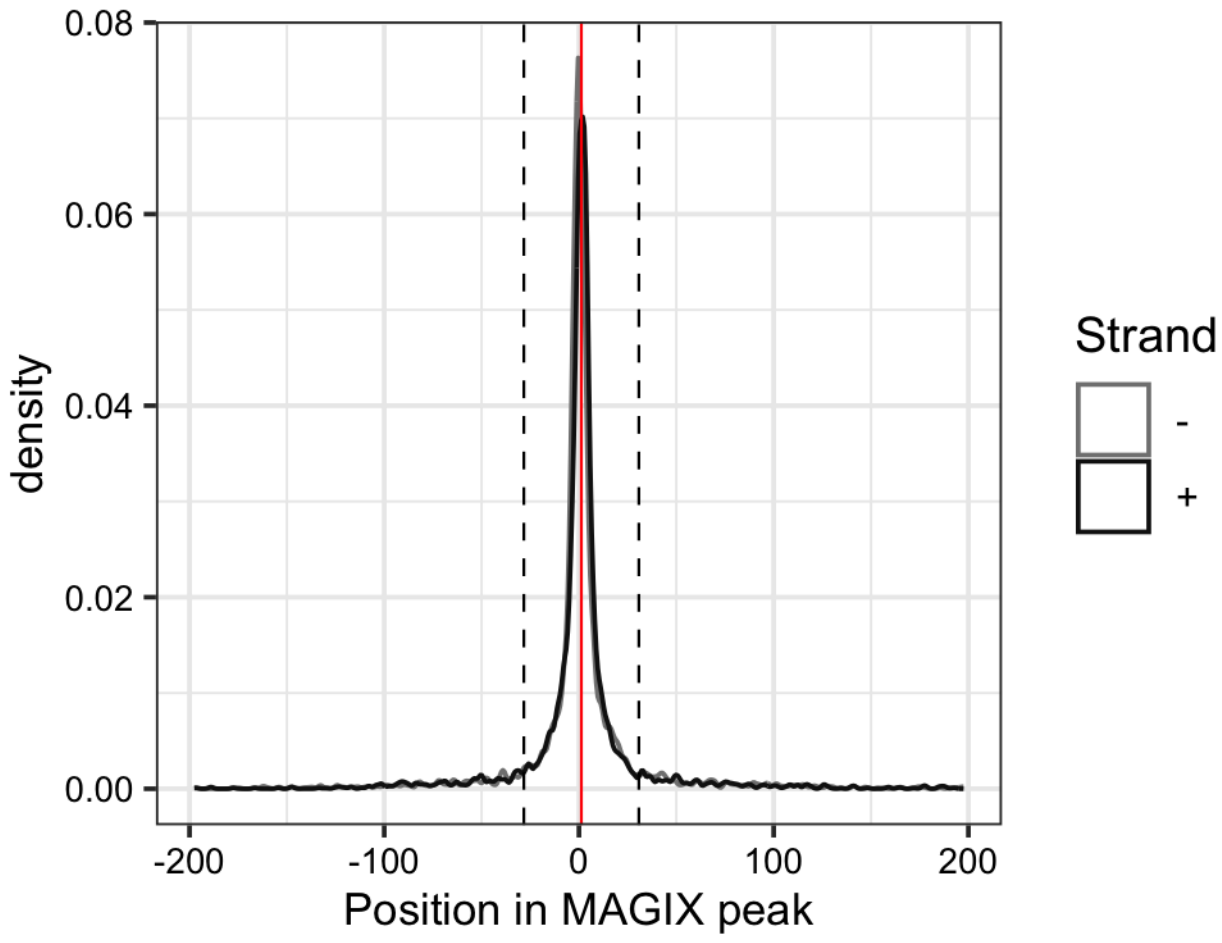

ZNF836.FL

Top GRECO-BIT/MEX PWM: PWM006005

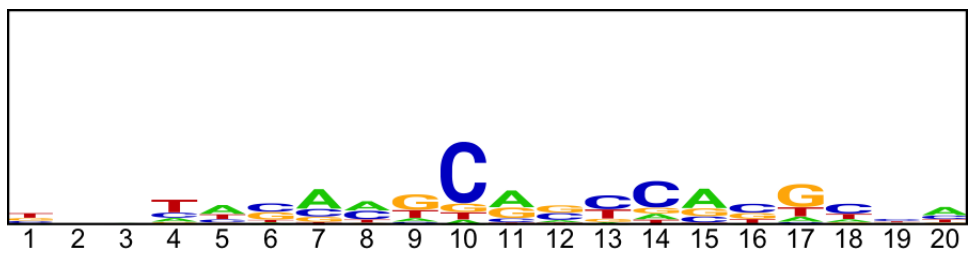

Location of top GRECO-BIT/MEX PWM in MAGIX peaks (up to the top 10,000)

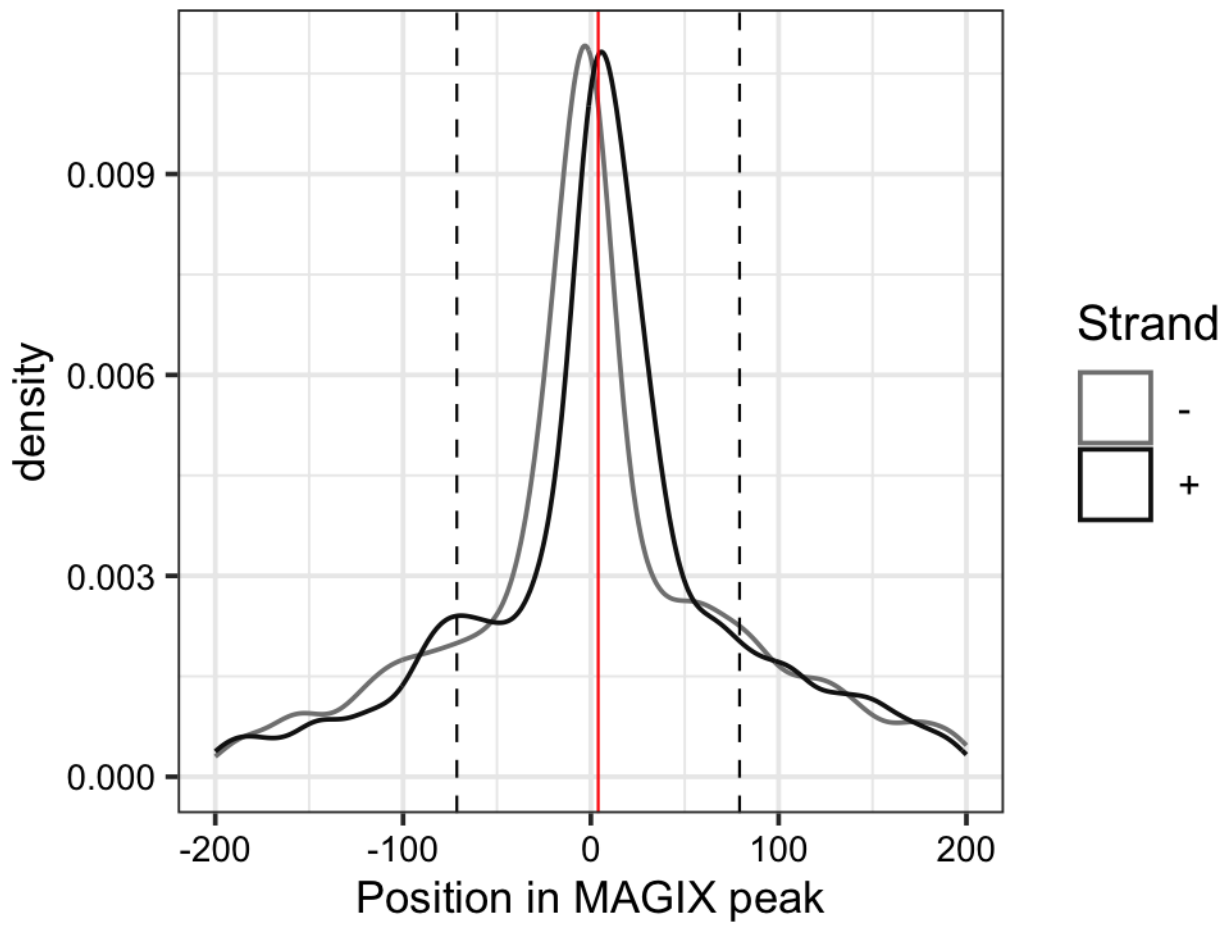

AUROC of top GRECO-BIT/MEX PWM in sliding windows (500 peaks, 50 peak step) along the MAGIX peaks (up to the top 75,000) ranked by the MAGIX enrichment coefficient and fraction of overlapping ChIP peaks in the same window

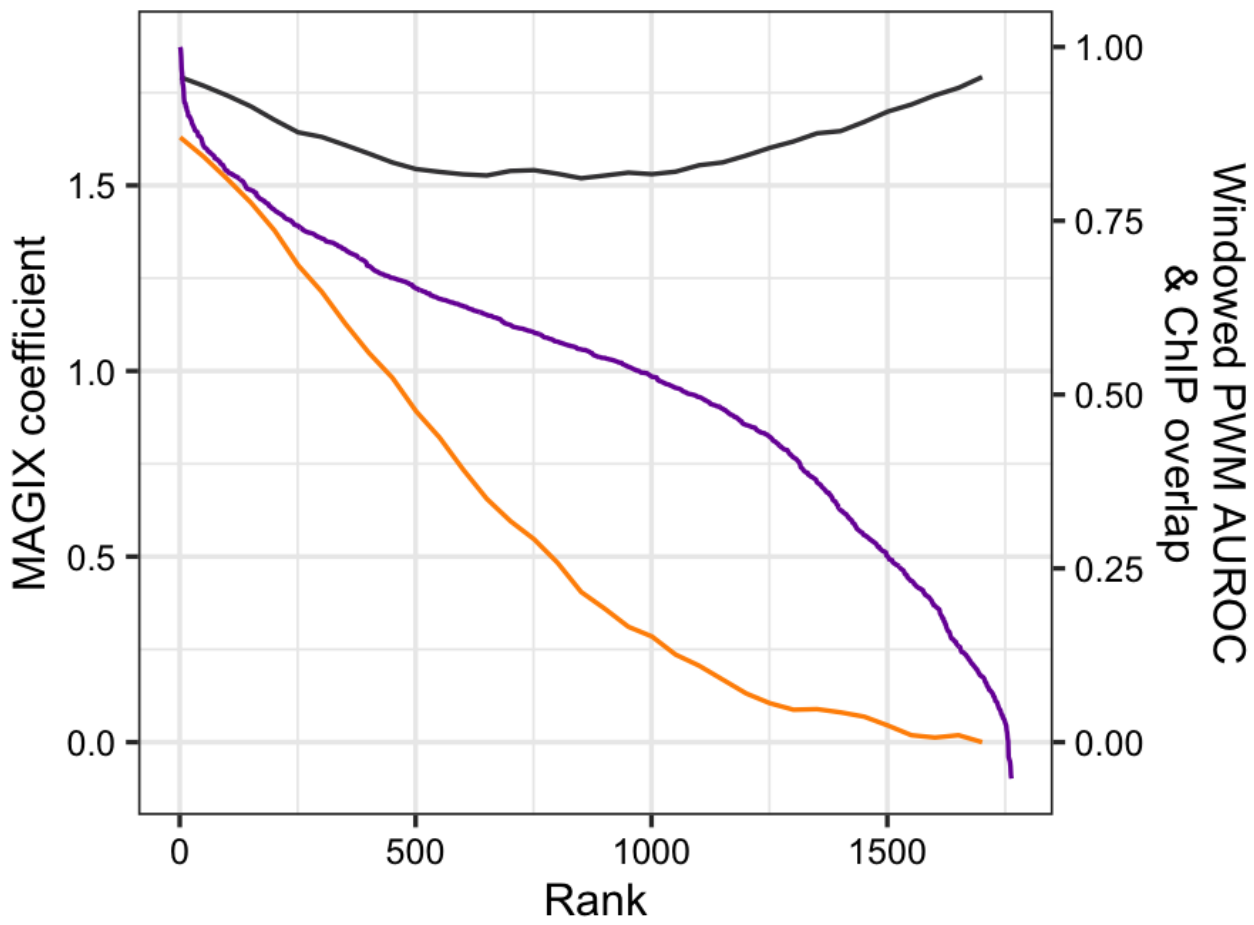

ZNF841.DBD2

Top GRECO-BIT/MEX PWM: PWM006038

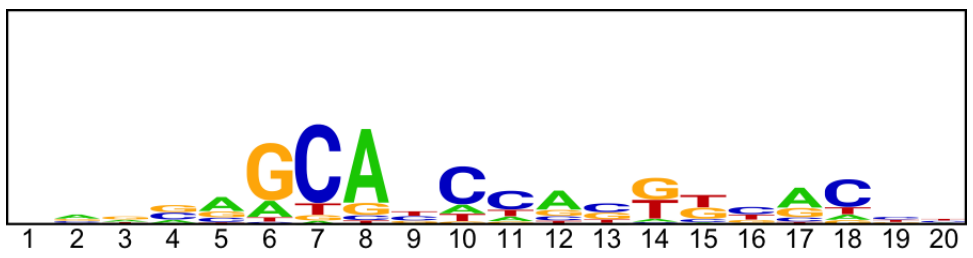

Location of top GRECO-BIT/MEX PWM in MAGIX peaks (up to the top 10,000)

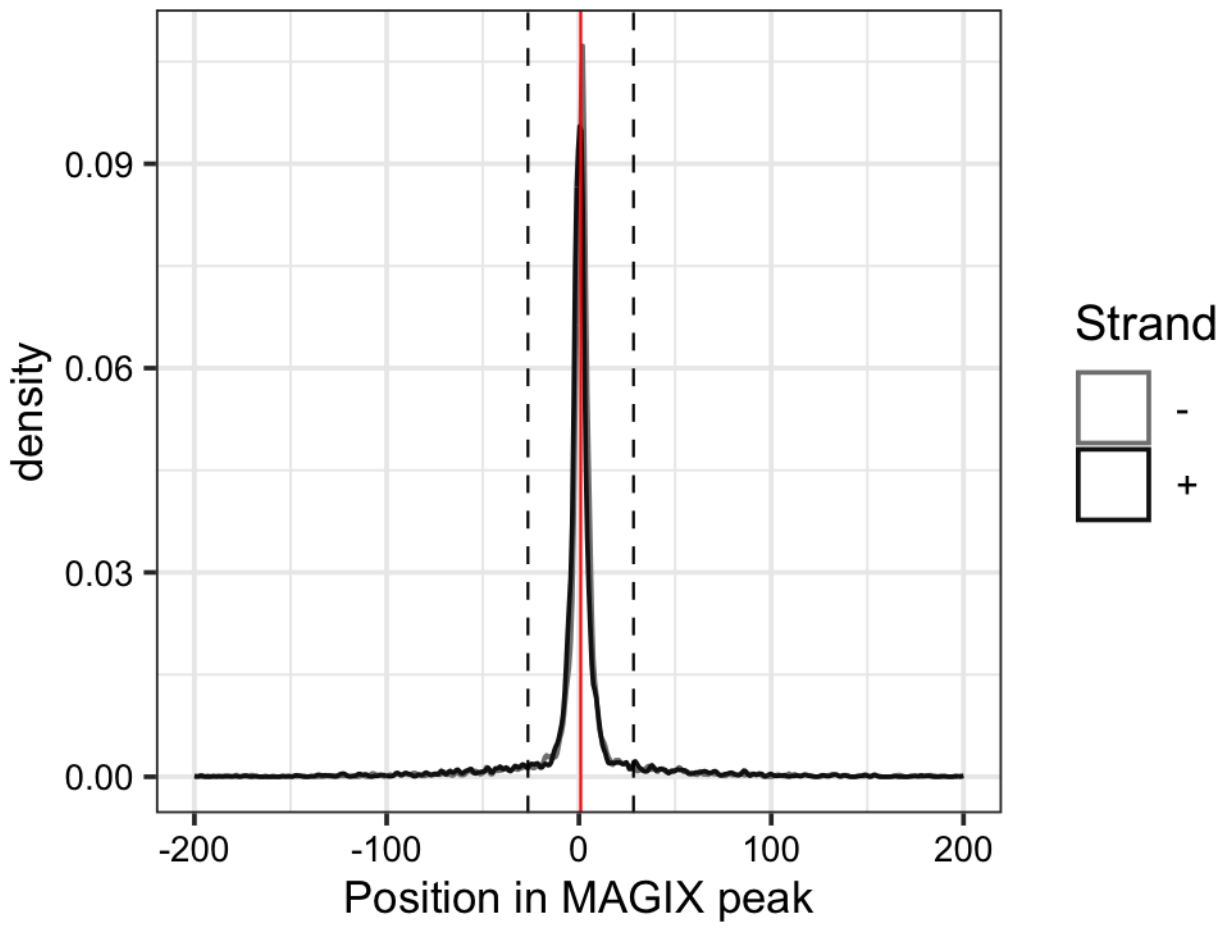

ZNF841.FL

Top GRECO-BIT/MEX PWM: PWM006030

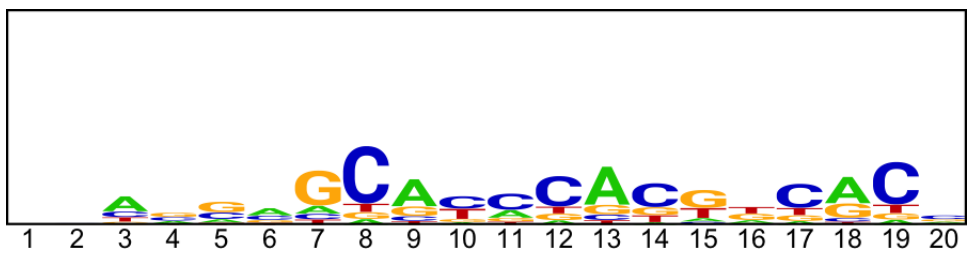

Location of top GRECO-BIT/MEX PWM in MAGIX peaks (up to the top 10,000)

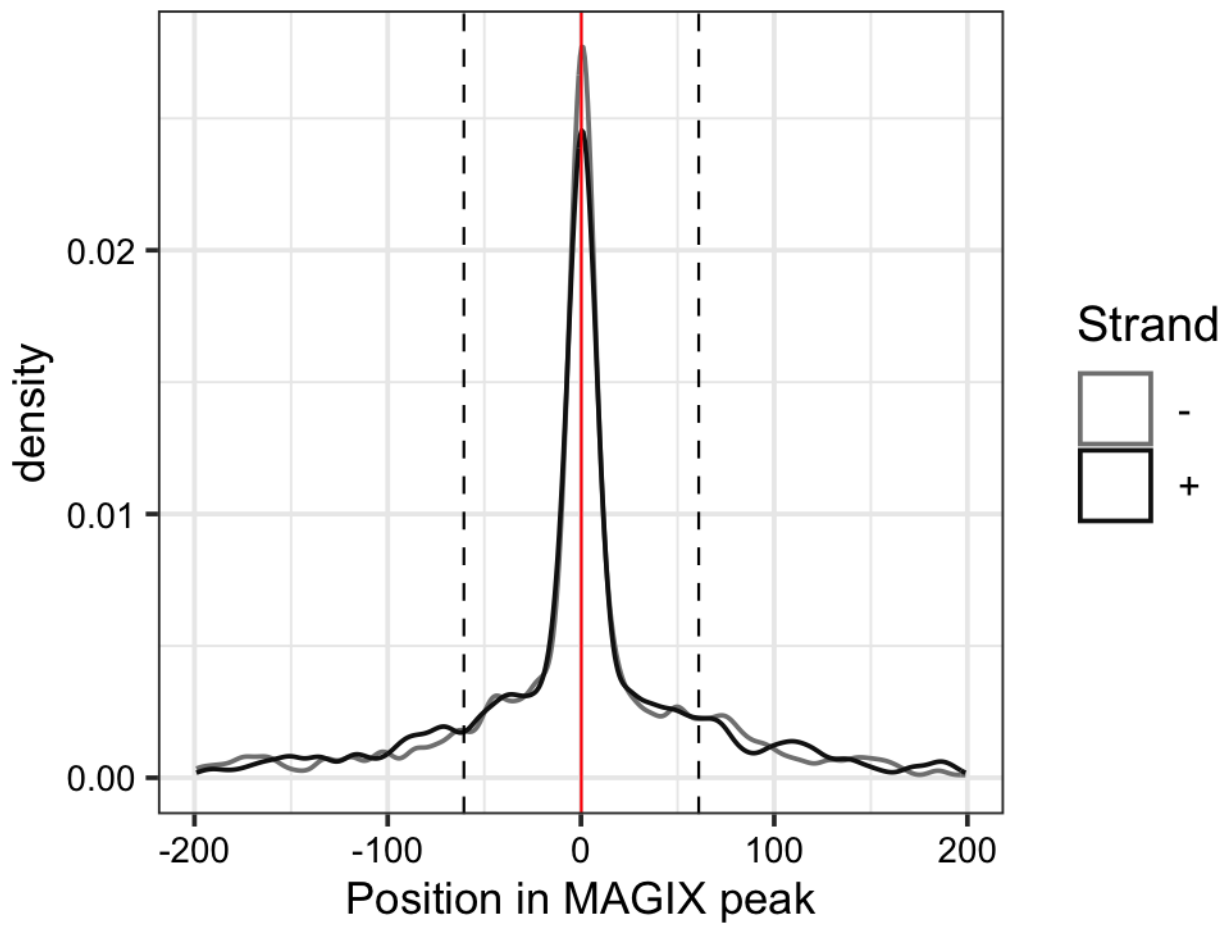

AUROC of top GRECO-BIT/MEX PWM in sliding windows (500 peaks, 50 peak step) along the MAGIX peaks (up to the top 75,000) ranked by the MAGIX enrichment coefficient and fraction of overlapping ChIP peaks in the same window

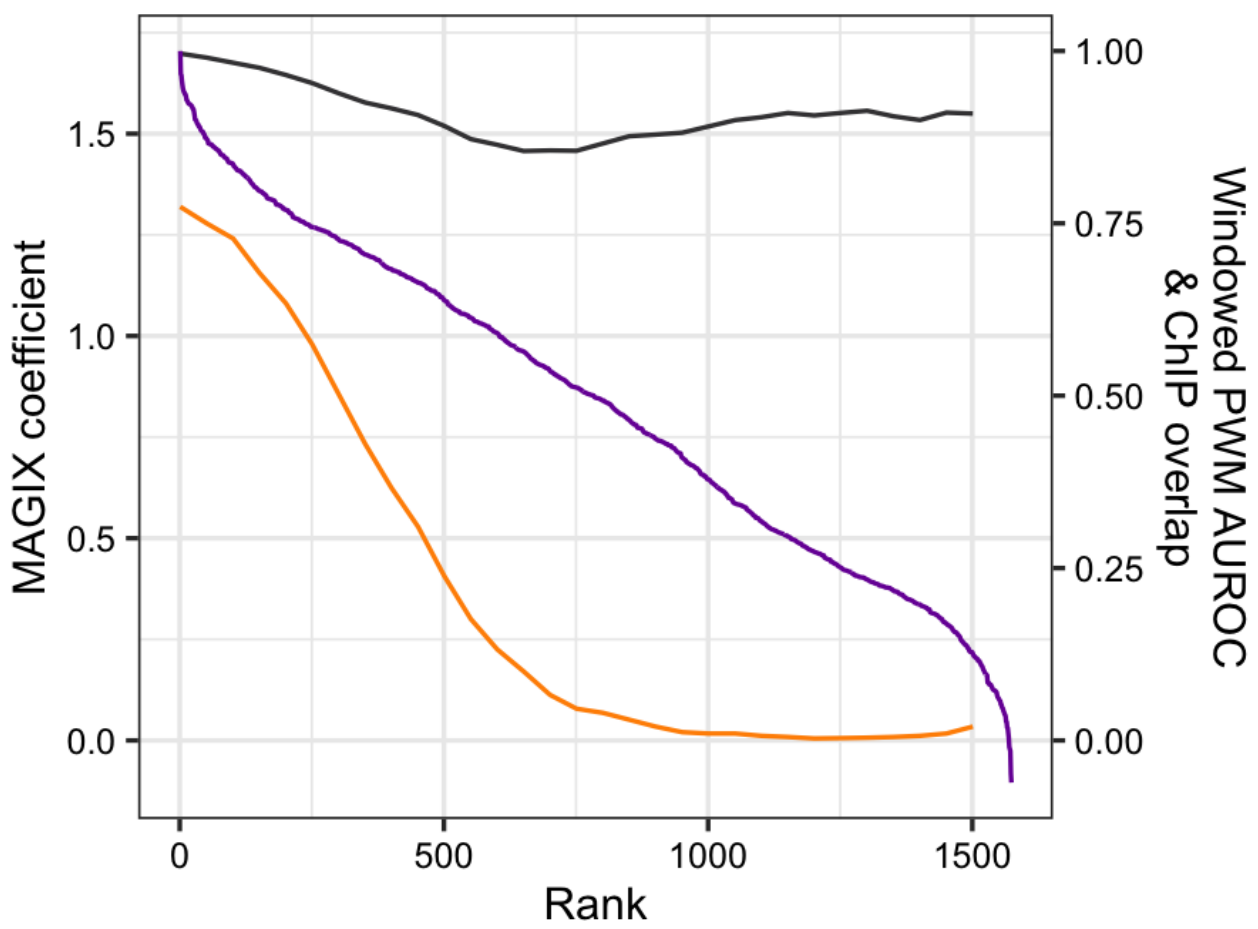

ZNF845.DBD1

Top GRECO-BIT/MEX PWM: PWM192799

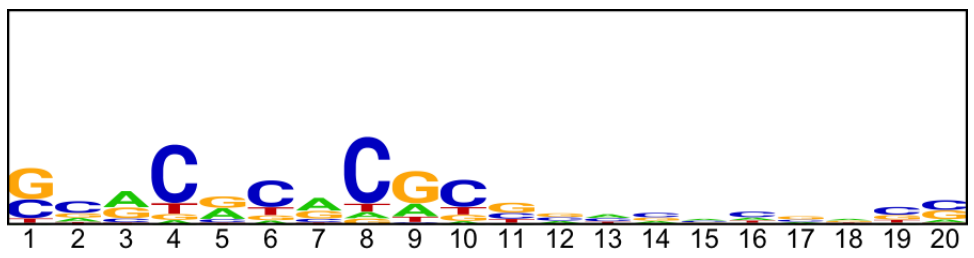

Location of top GRECO-BIT/MEX PWM in MAGIX peaks (up to the top 10,000)

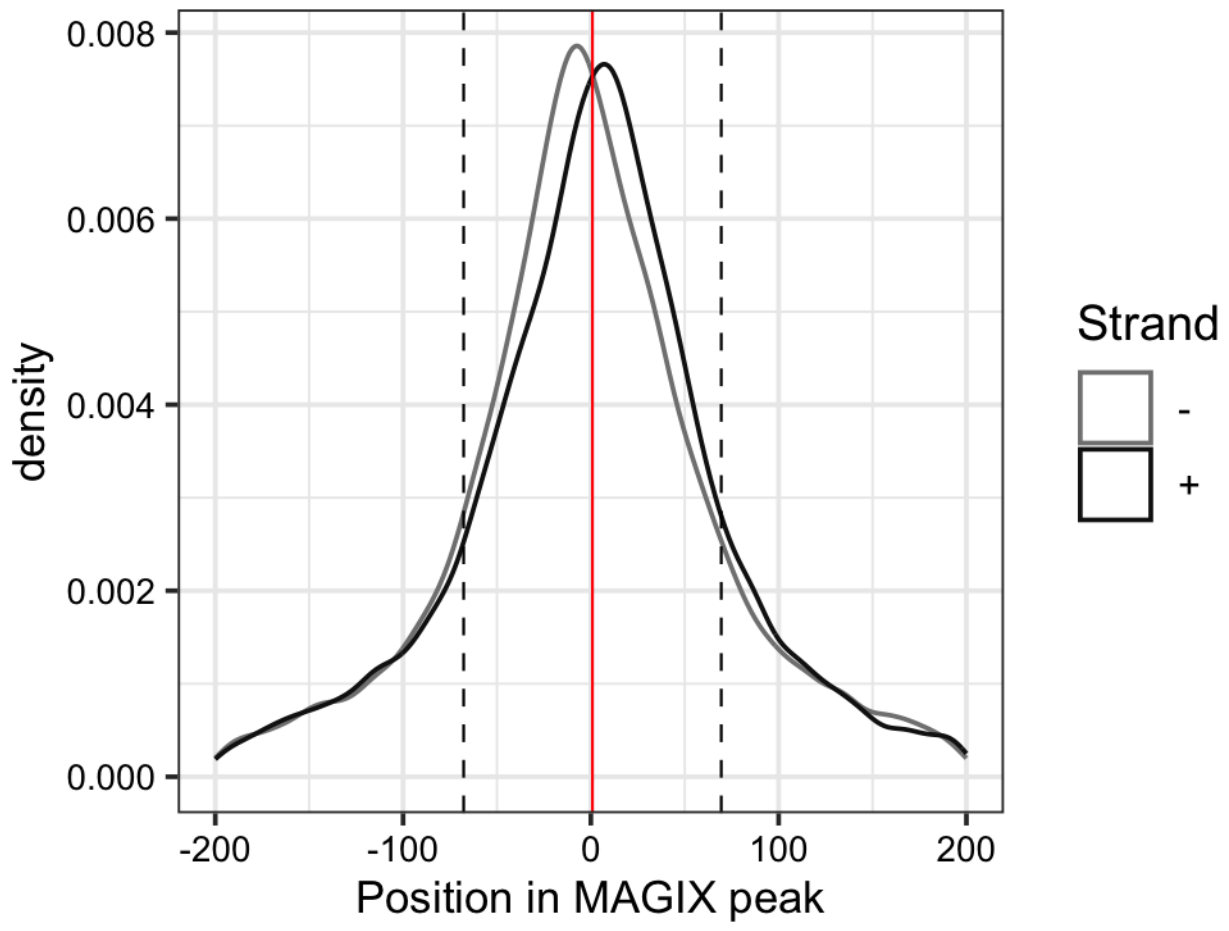

ZNF845.DBD2

Top GRECO-BIT/MEX PWM: PWM006060

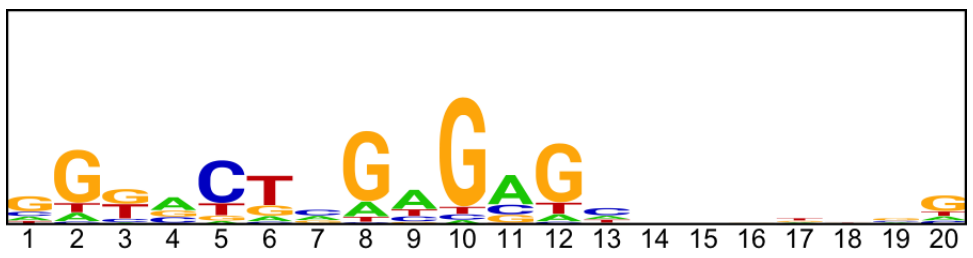

Location of top GRECO-BIT/MEX PWM in MAGIX peaks (up to the top 10,000)

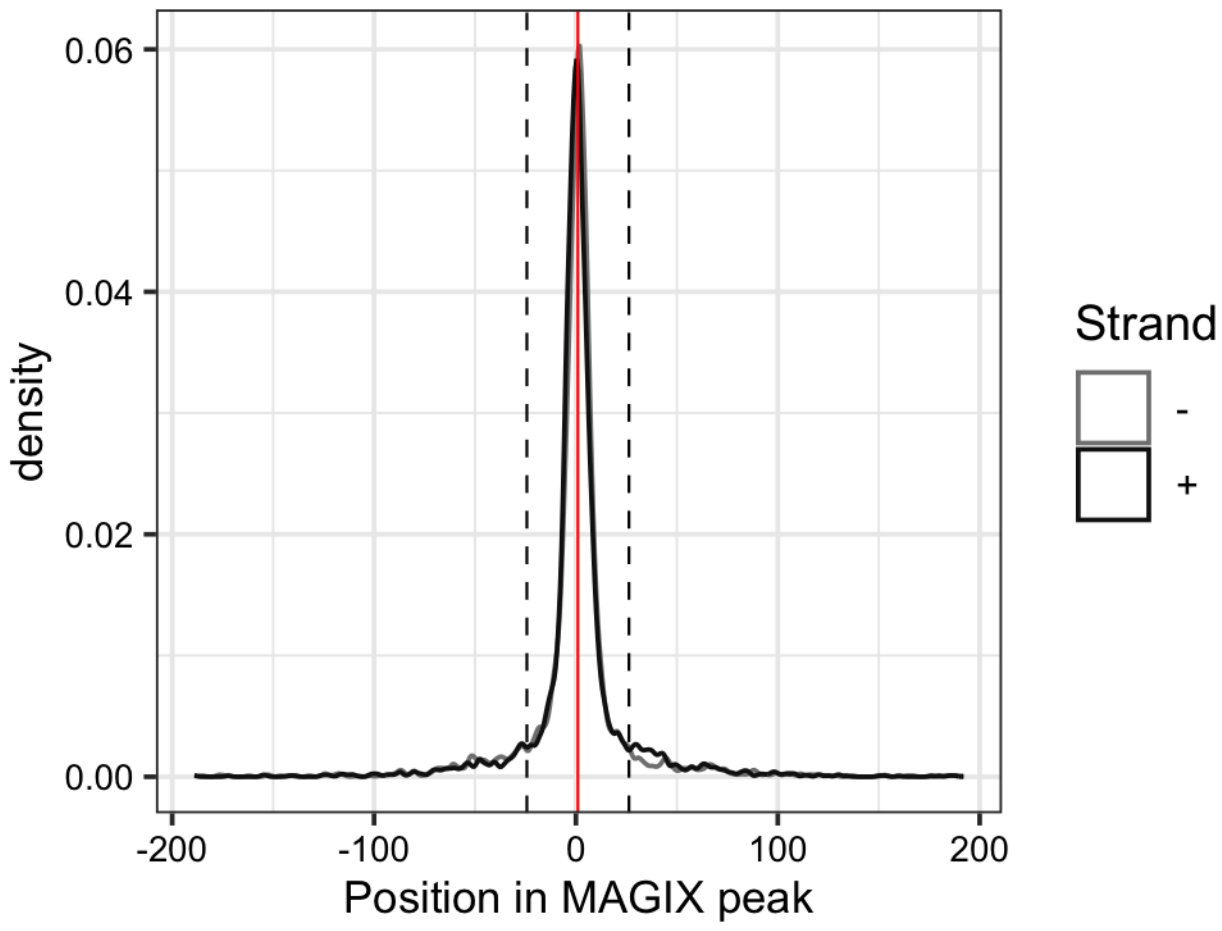

ZNF850.DBD2

Top GRECO-BIT/MEX PWM: PWM006084

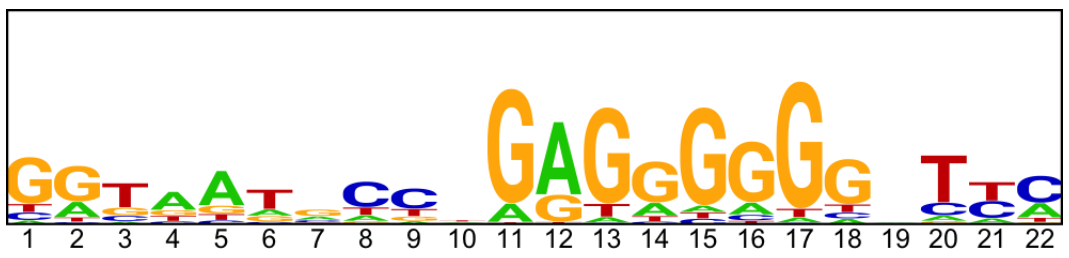

Location of top GRECO-BIT/MEX PWM in MAGIX peaks (up to the top 10,000)

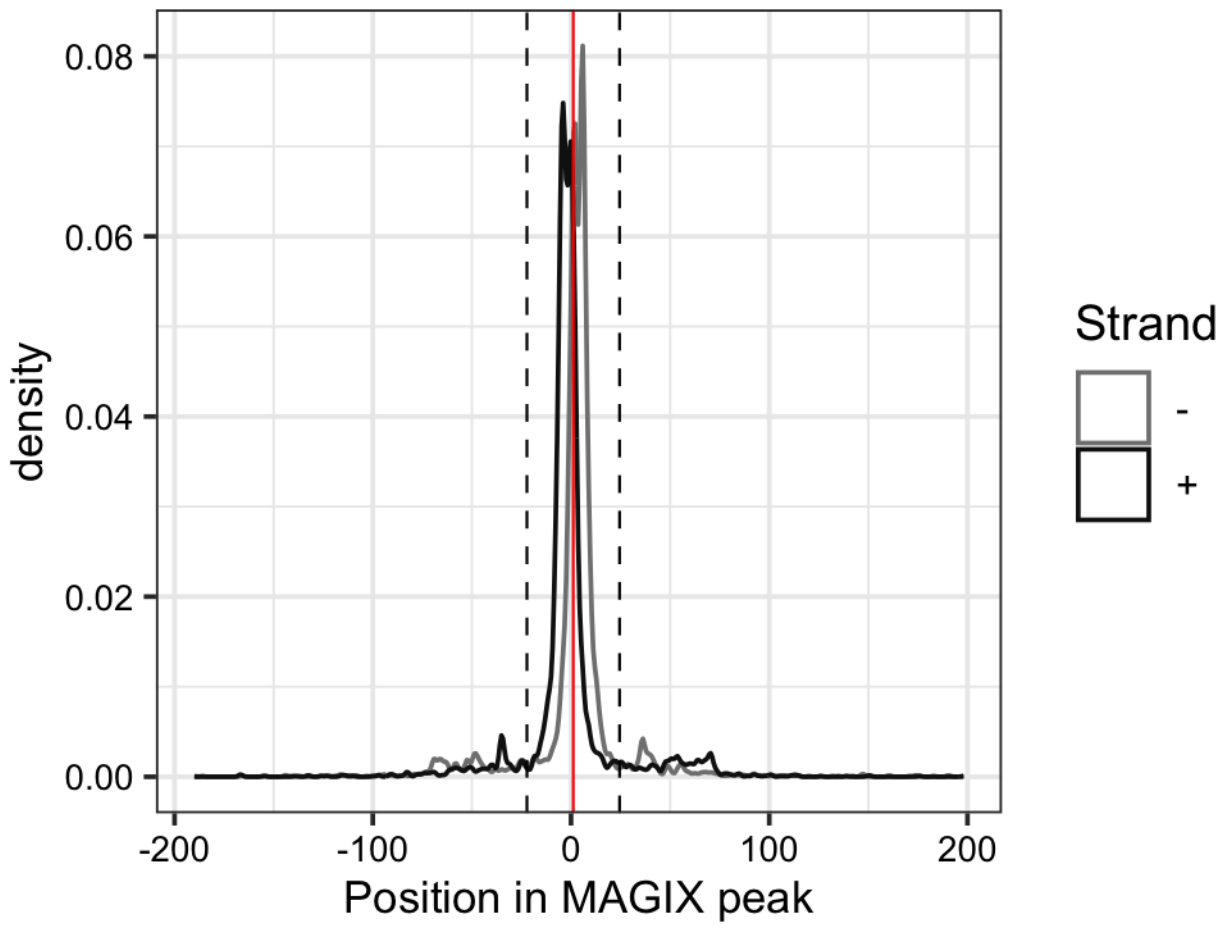

ZNF853

Top GRECO-BIT/MEX PWM: PWM006104

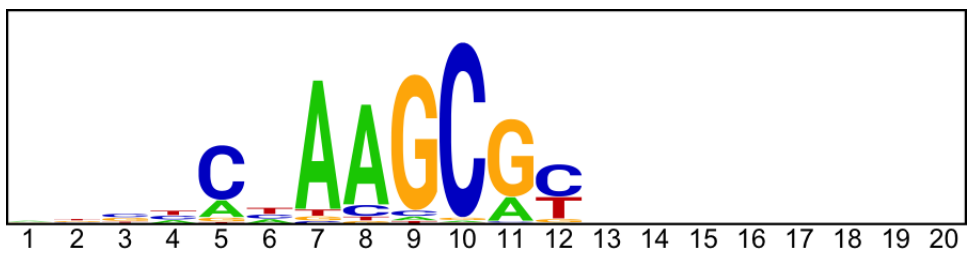

Location of top GRECO-BIT/MEX PWM in MAGIX peaks (up to the top 10,000)

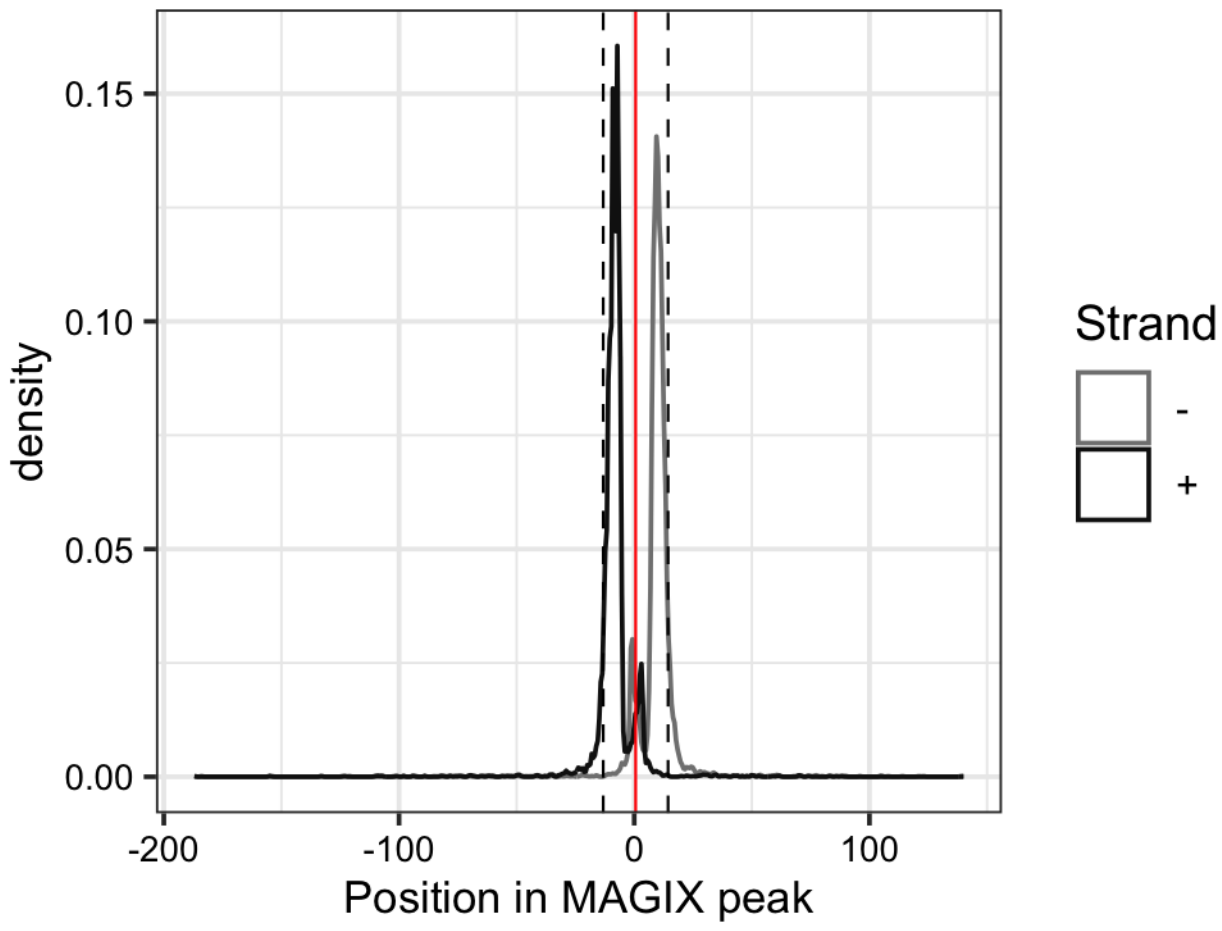

ZNF865.DBD1

Top GRECO-BIT/MEX PWM: PWM006141

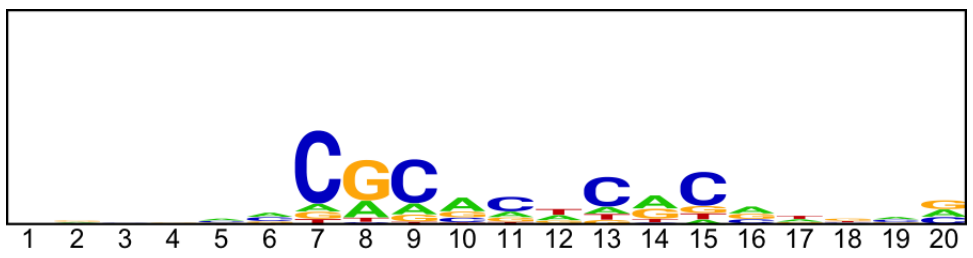

Location of top GRECO-BIT/MEX PWM in MAGIX peaks (up to the top 10,000)

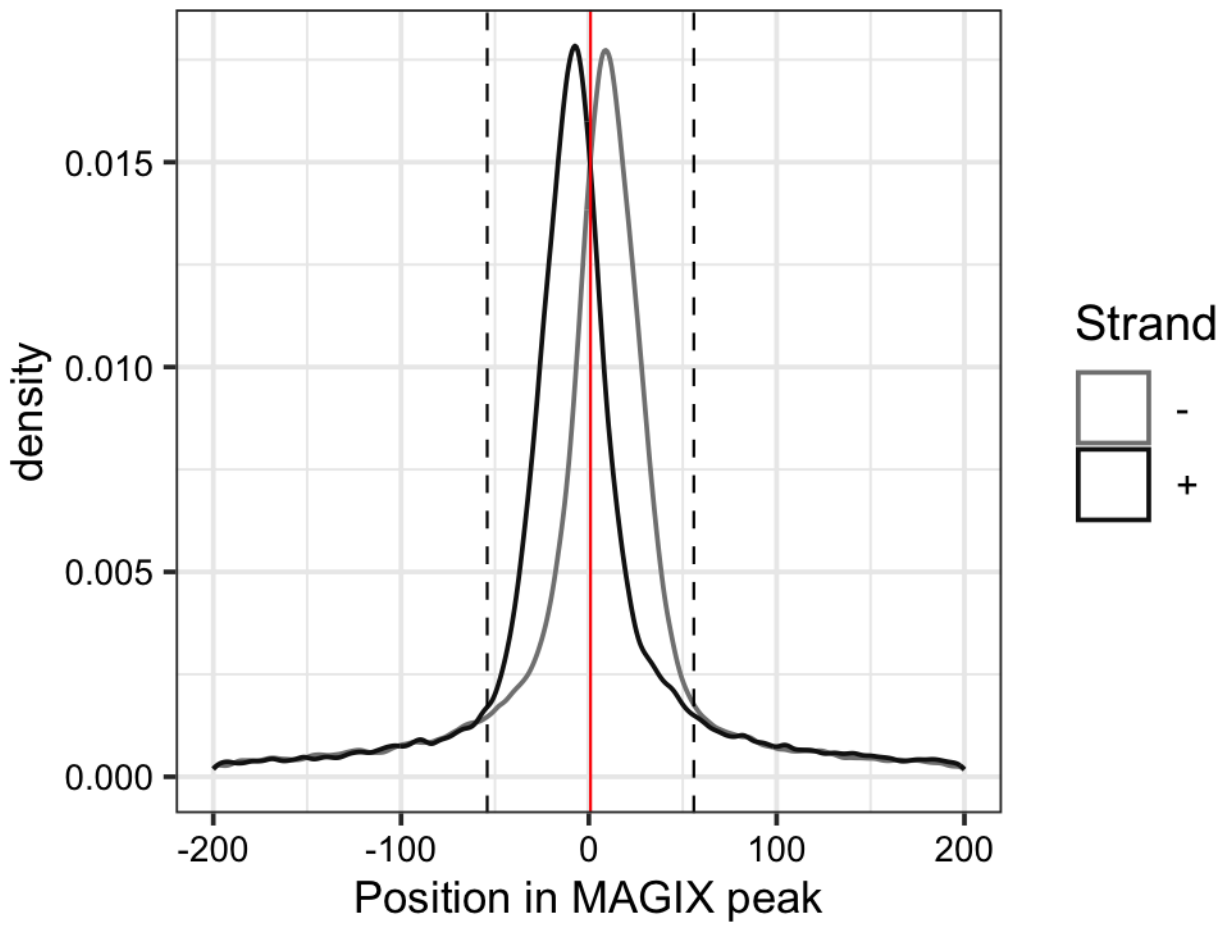

ZNF865.DBD2

Top GRECO-BIT/MEX PWM: PWM006139

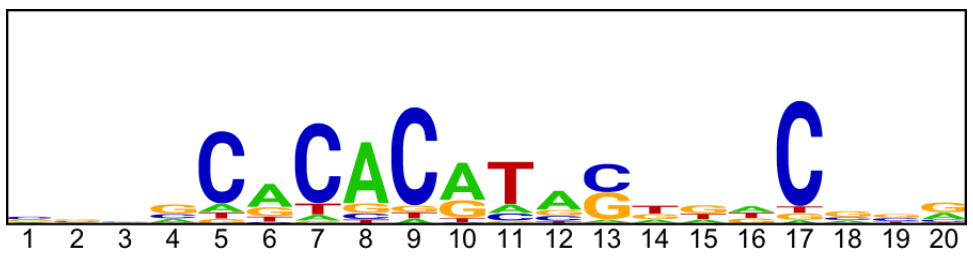

Location of top GRECO-BIT/MEX PWM in MAGIX peaks (up to the top 10,000)

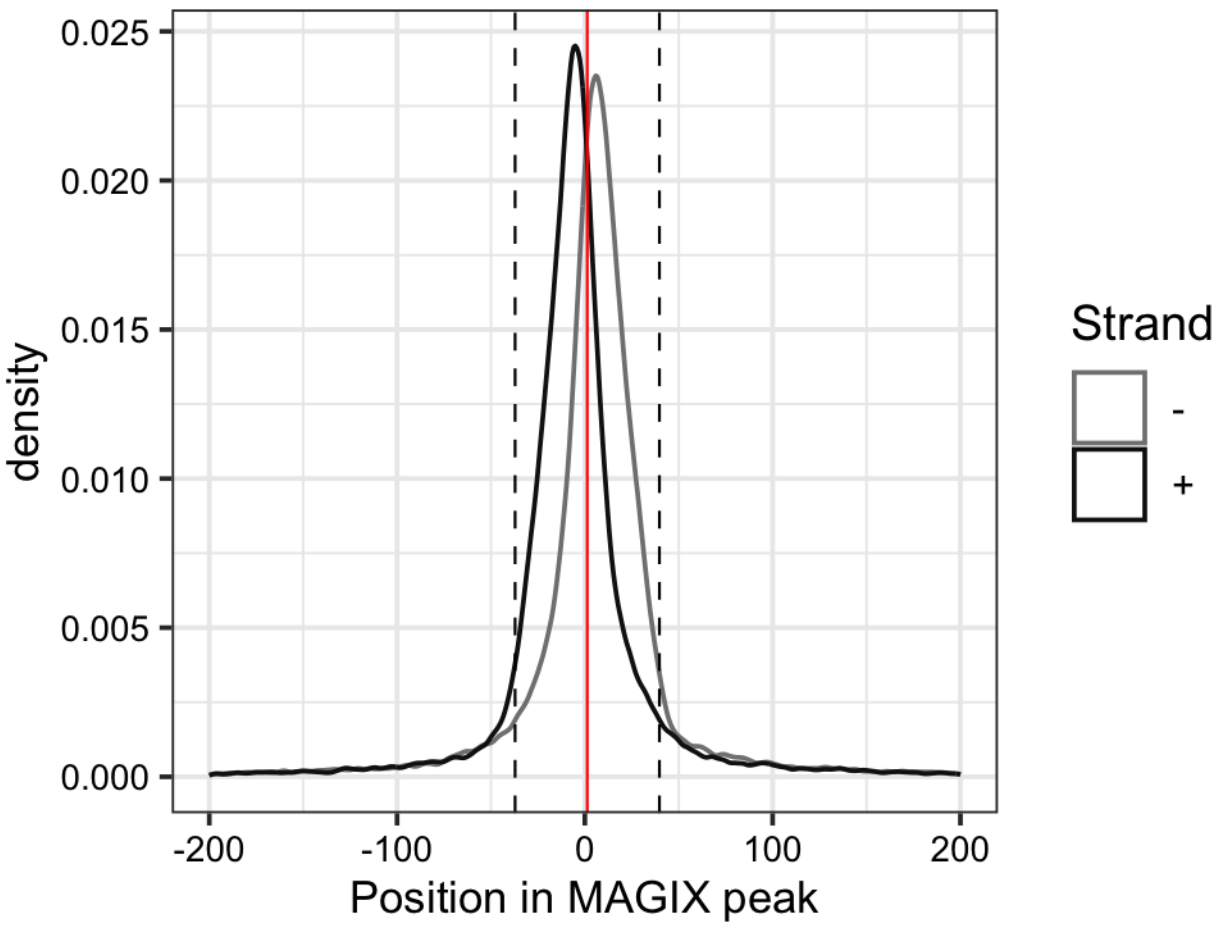

ZSCAN2

Top GRECO-BIT/MEX PWM: PWM006244

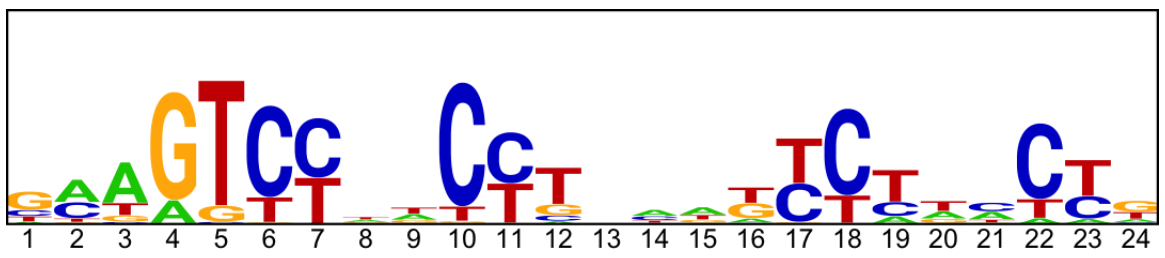

Location of top GRECO-BIT/MEX PWM in MAGIX peaks (up to the top 10,000)

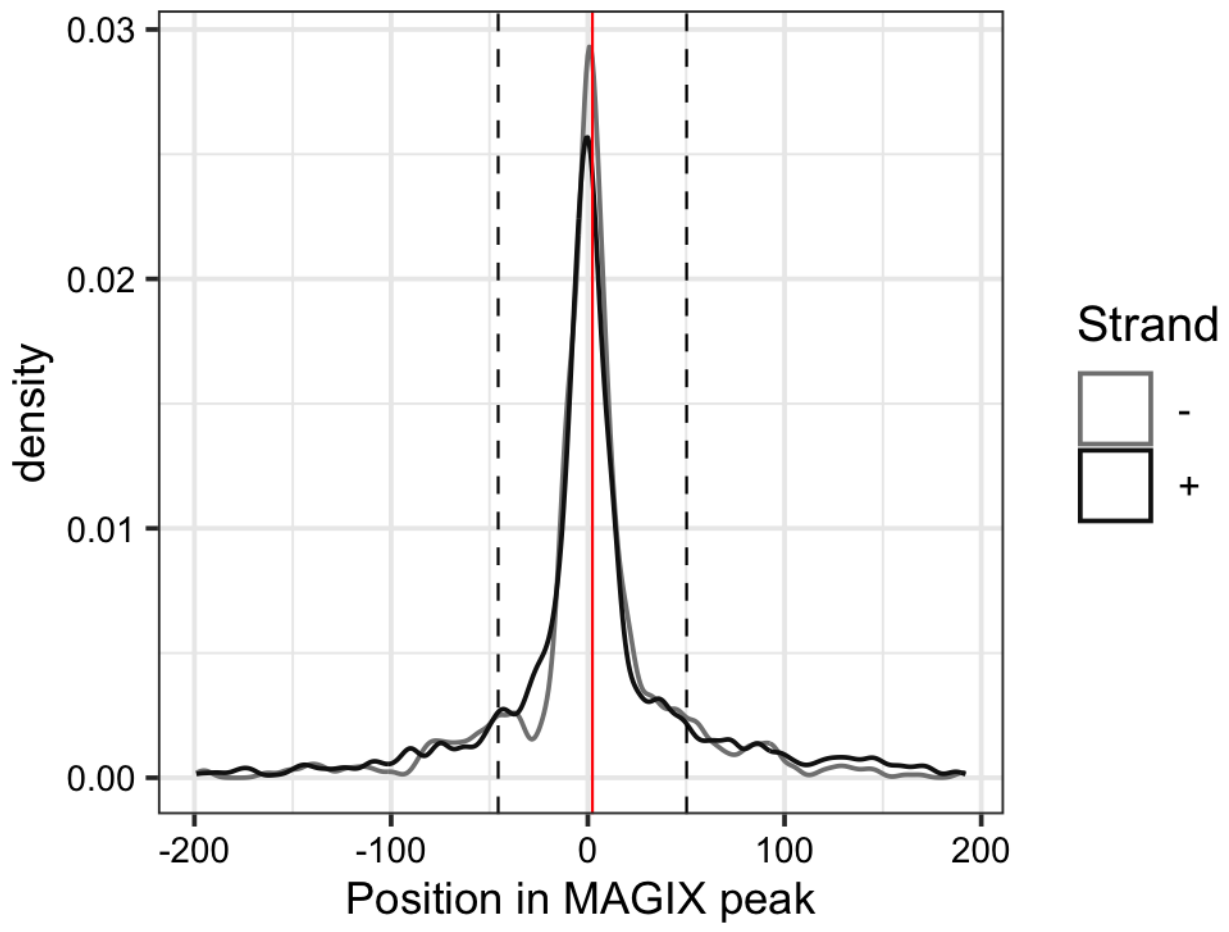

AUROC of top GRECO-BIT/MEX PWM in sliding windows (500 peaks, 50 peak step) along the MAGIX peaks (up to the top 75,000) ranked by the MAGIX enrichment coefficient and fraction of overlapping ChIP peaks in the same window

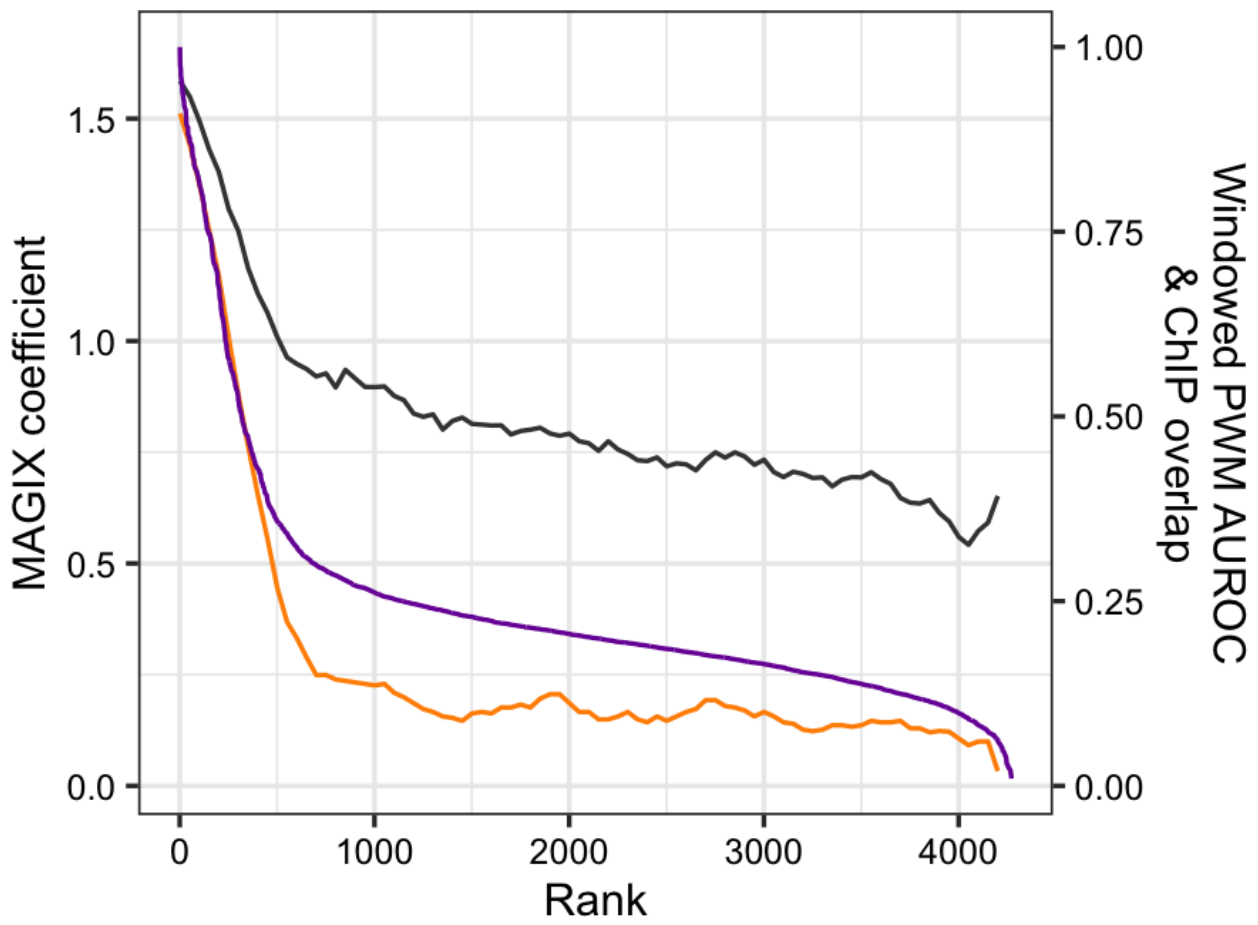

ZSCAN25

Top GRECO-BIT/MEX PWM: PWM006291

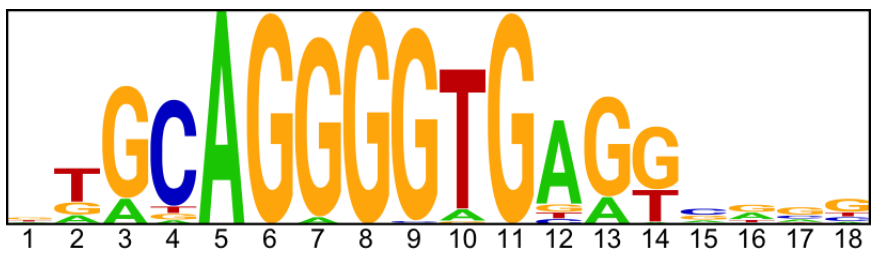

Location of top GRECO-BIT/MEX PWM in MAGIX peaks (up to the top 10,000)

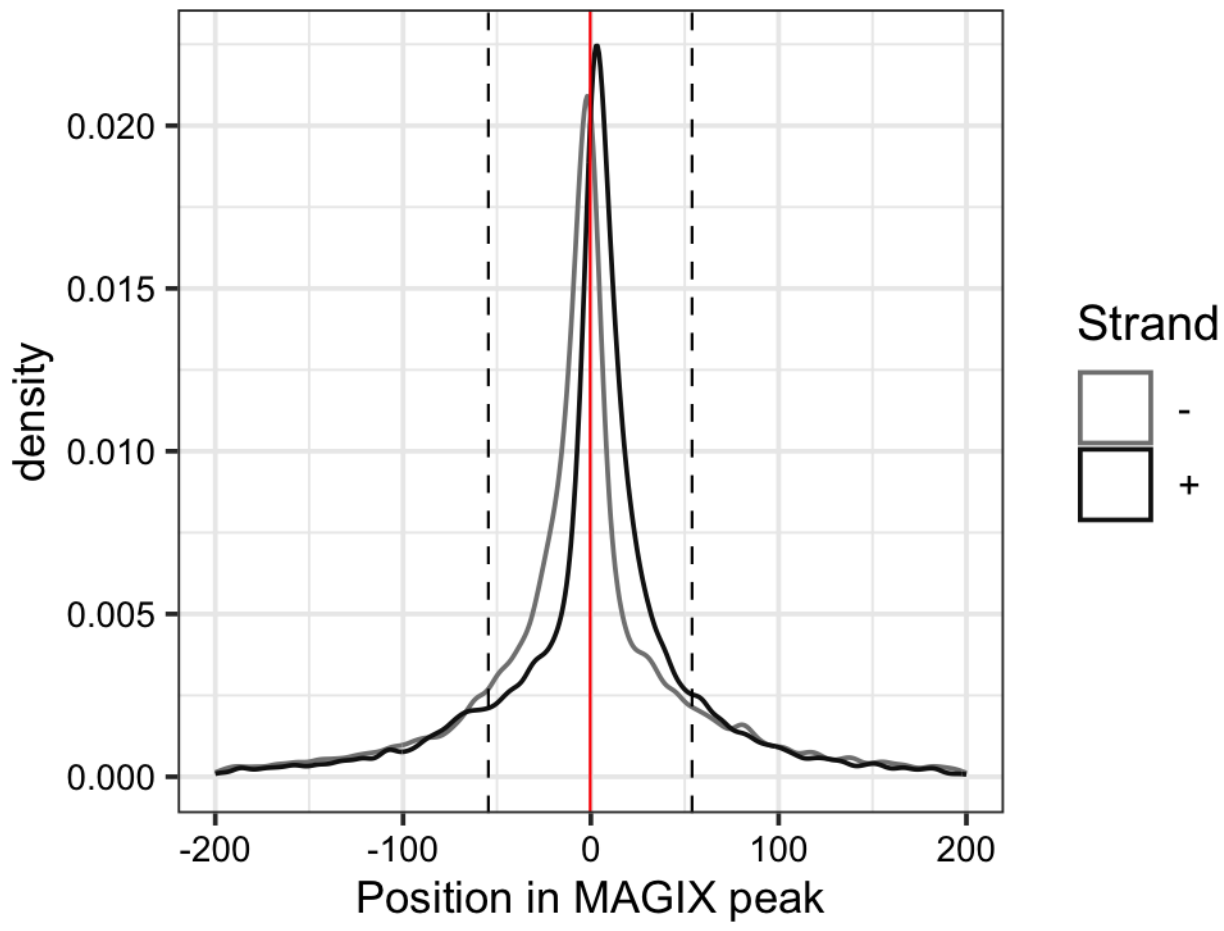

AUROC of top GRECO-BIT/MEX PWM in sliding windows (500 peaks, 50 peak step) along the MAGIX peaks (up to the top 75,000) ranked by the MAGIX enrichment coefficient and fraction of overlapping ChIP peaks in the same window

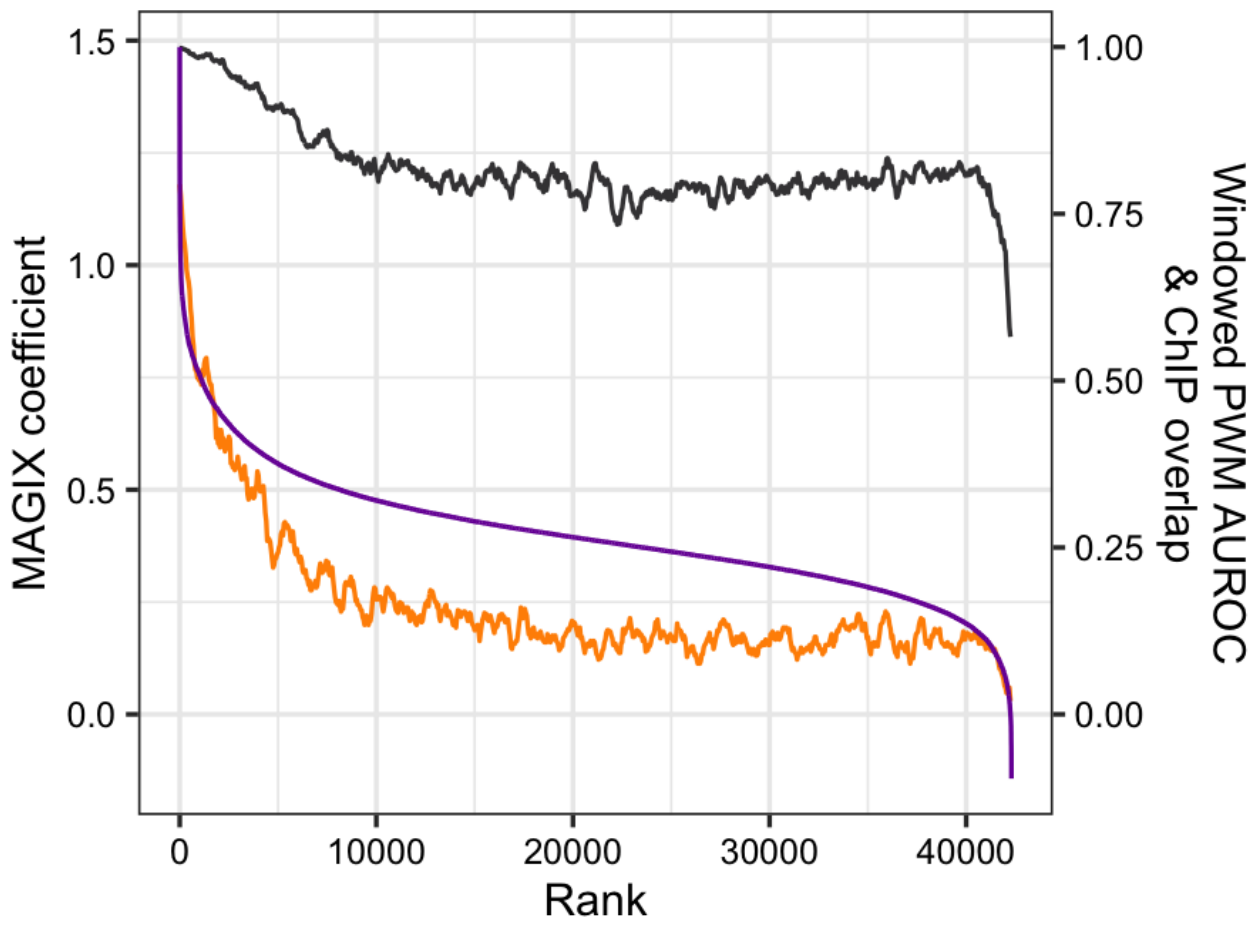

ZSCAN4

Top GRECO-BIT/MEX PWM: PWM006306

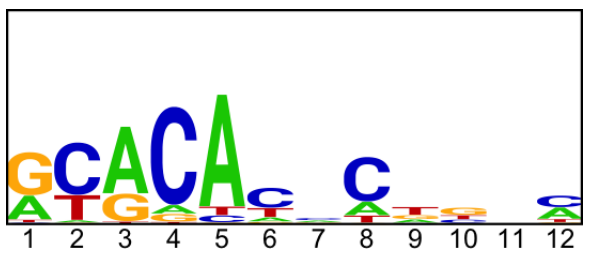

Location of top GRECO-BIT/MEX PWM in MAGIX peaks (up to the top 10,000)

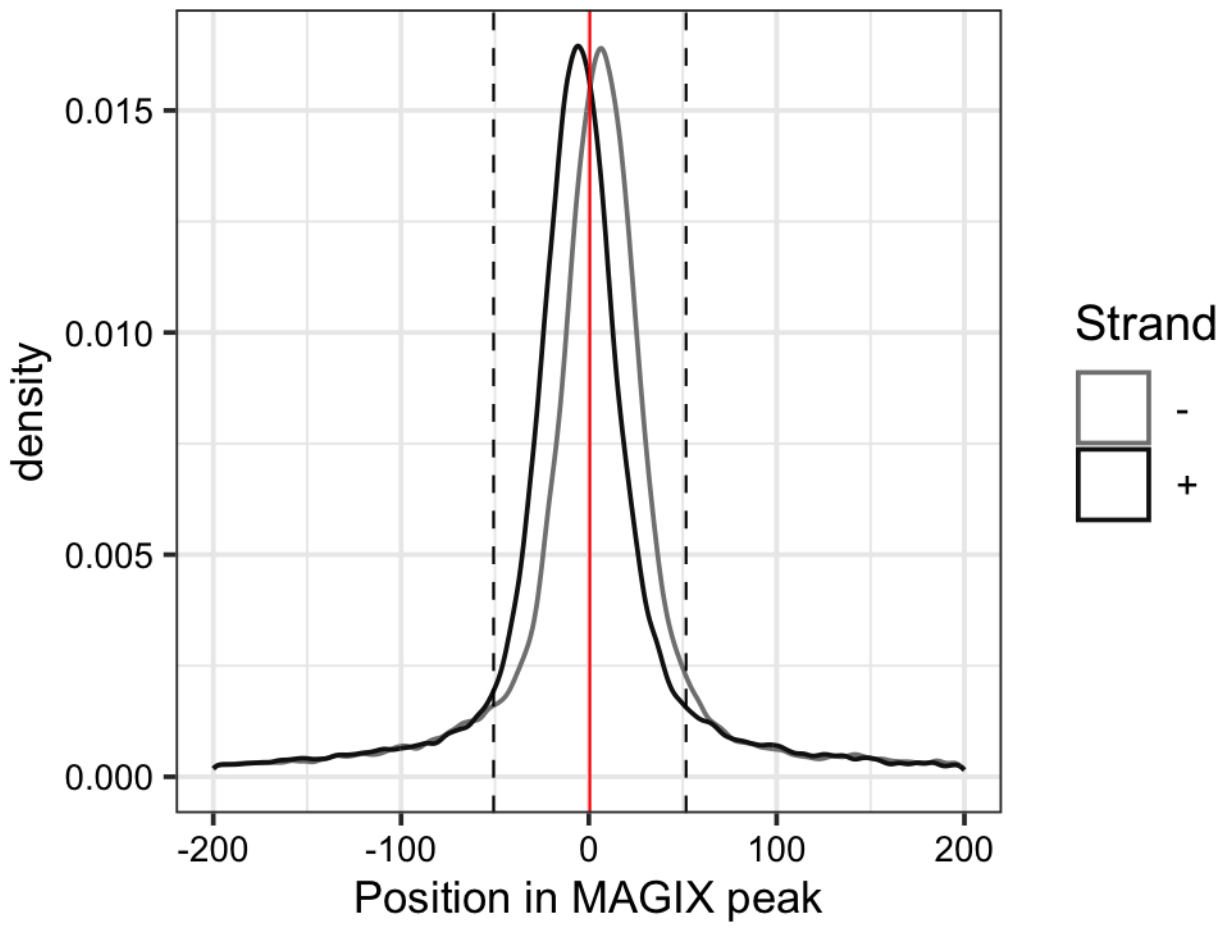

Supplement: Supplementary file 2 [file 41592_2026_3177_MOESM17_ESM.pdf]
