# Supplementary material for: GHT-SELEX demonstrates unexpectedly high intrinsic sequence specificity and complex DNA binding of many human transcription factors
Source: Nat Methods. 2026 Aug 5;23(9):1775–85. doi: 10.1038/s41592-026-03177-9 (PMC13541613; doi:10.1038/s41592-026-03177-9)

Enrichment of k-mers between HT-SELEX replicate experiments

This document displays plots of k-mers enriched in experiments for the same TF expressed in different or same expression system and describes what is the most likely explanation for differences seen in kmers. See **Table S6** for details of each pairwise comparison.

C and G rich sequences
There is a fairly common tendency for low level poly-C/poly-G sequences to be enriched in one sample but not the other. This outcome is however uncorrelated with the protein expression system, or with any other experimental variable. The initial HT-SELEX pool it is biased towards the same sequences, presumably due to bias in the commercial DNA synthesis. Examples of this are visible for example in comparison on page: 4,

Enrichment of sequences that are complementary to Illumina flanking regions
In this artifact the experiment enriches sequences that have partial matches to constant regions that are used in the Illumina sequencing. We do not know the mechanism behind this but it is likely derived from selection artifact of mis-hybridized ligands and occurs due to presence of partially single stranded DNA in the experiments. A common kmer like this is ACACGACGC. This is clearly visible for example in experiment pair on page: 26, where it enriches alongside genuine target site for DACH1.

Enrichment of mono- and homodimeric binding sites for FLI1
FLI1, a positive control, is known to bind both as mono- and homodimers, and selection pressure for them differs based on protein concentration as higher concentration of TF will drive enrichment of homodimer (See Jolma et al. 2013, PMID: 23332764); we observe what appears to be differential abundance of the two modes in different experiments. An example of an experiment pair with similar enrichment for both monomeric and dimeric FLI1 target sites is on page 33 with example 9-mers being ACCGGAAGT (monomer) and CGGATATCC (dimer). This differential enrichment is visible in comparisons on pages: 33, 37, 38 and 39.

Enrichment of aptameric partially single stranded DNA molecules in experiments for SOX-proteins
SOX proteins can bind both doublestranded DNA and short aptamers formed from partially single stranded DNA and this is visible in several experiments for SRY, SOX15 and SOX2 proteins. select commonly sequences that are likely to be stem-loop DNA aptamers formed from partially single stranded DNA (also previously described in Jolma et al. 2013), because the bases have symmetric dependencies on the opposite sides of the motif. An example of a commonly enriched aptameric 9-mer is AATGACATT whereas GAACAATGG is a commonly entriched 9-mer with a double stranded DNA target site. This differential enrichment is visible in experiment comparisons on pages: SOX15: 140, 143, 145 and 146; SOX2: 148, 150, 151, 153, 154 and 156; SRY: 178, 180 and 181

CAMTA1

Lysate vs Lysate, same construct

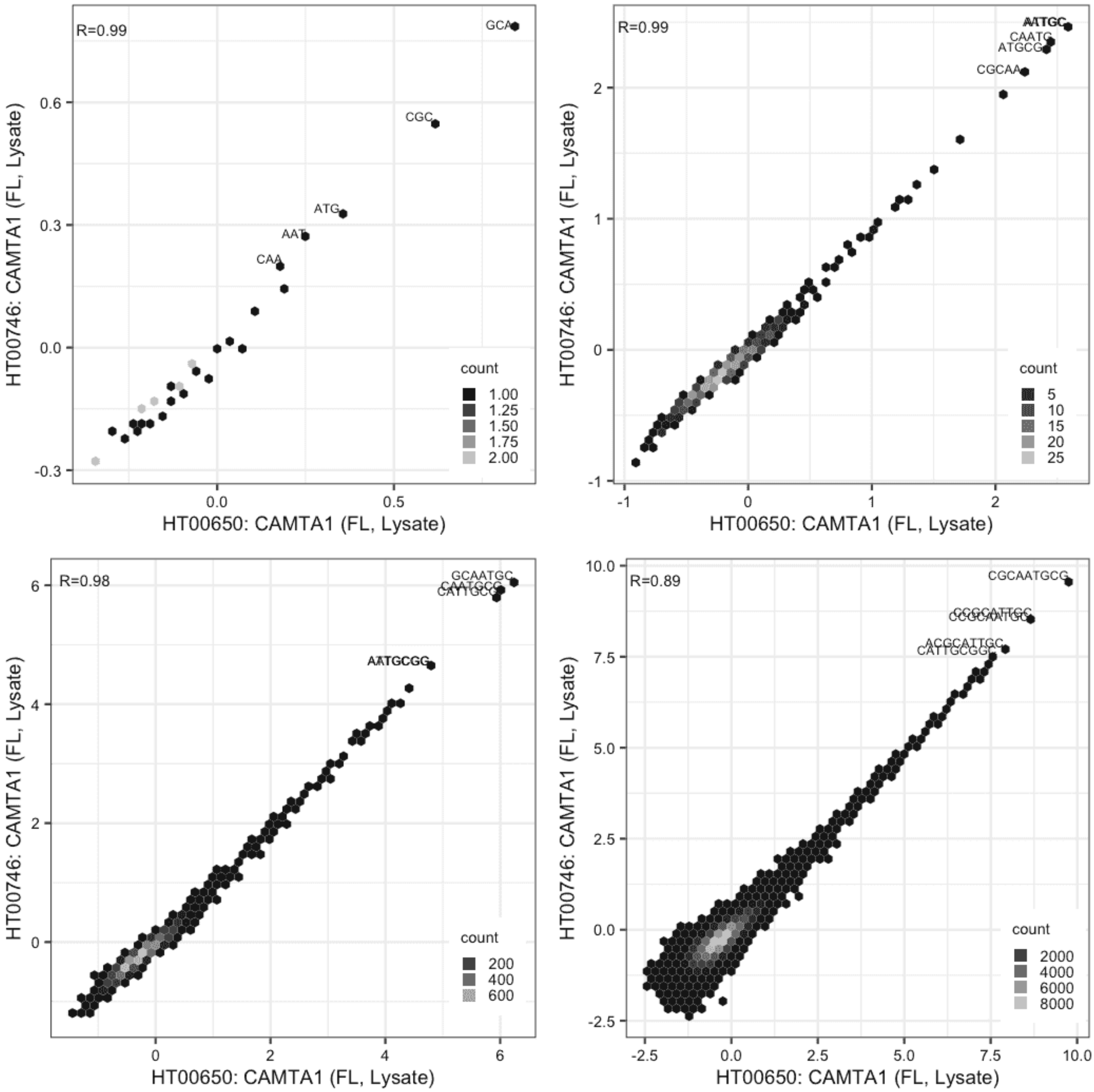

CAMTA1

Lysate vs GFPIVT, different constructs

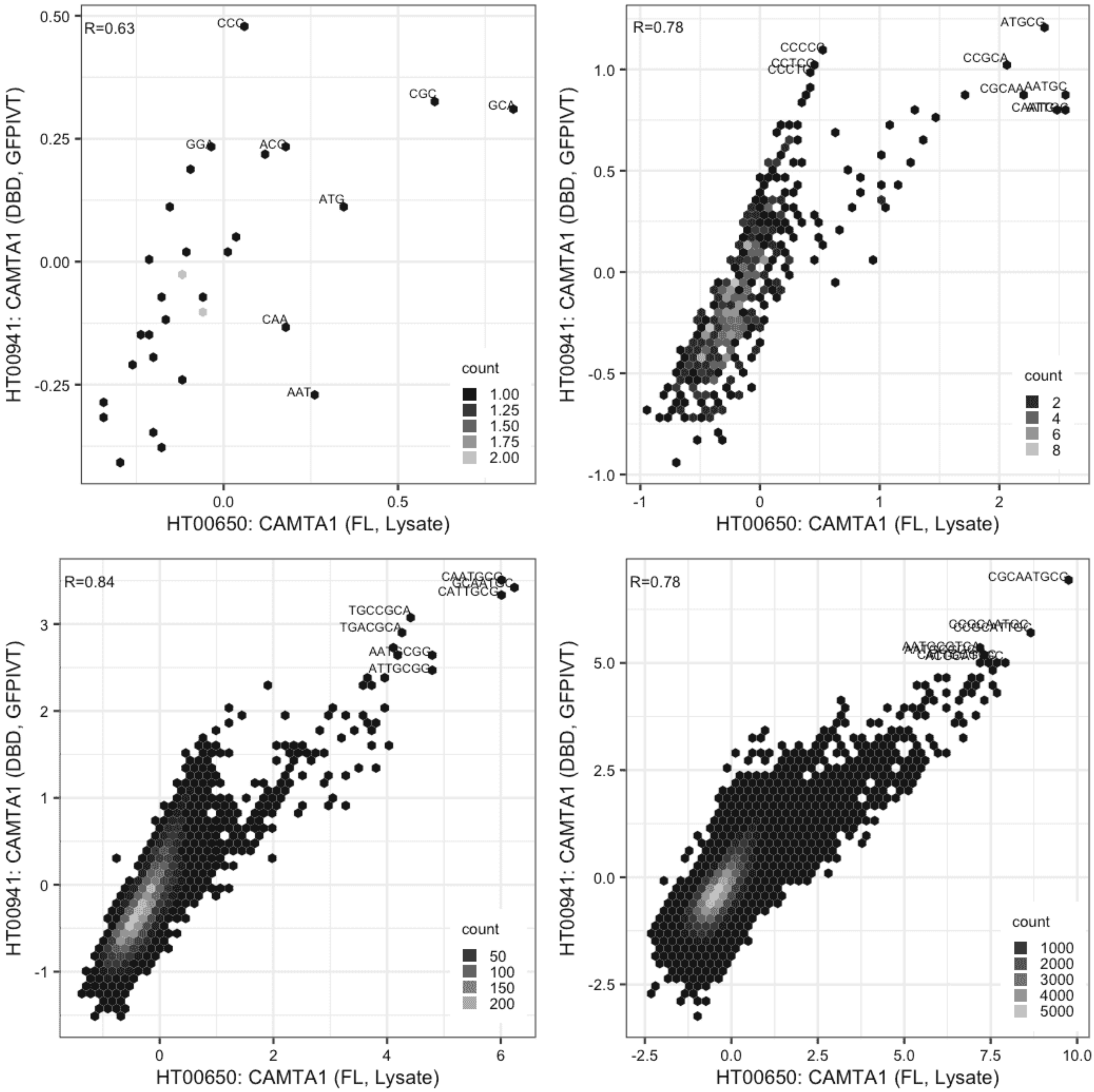

CAMTA1

Lysate vs GFPIVT, different constructs

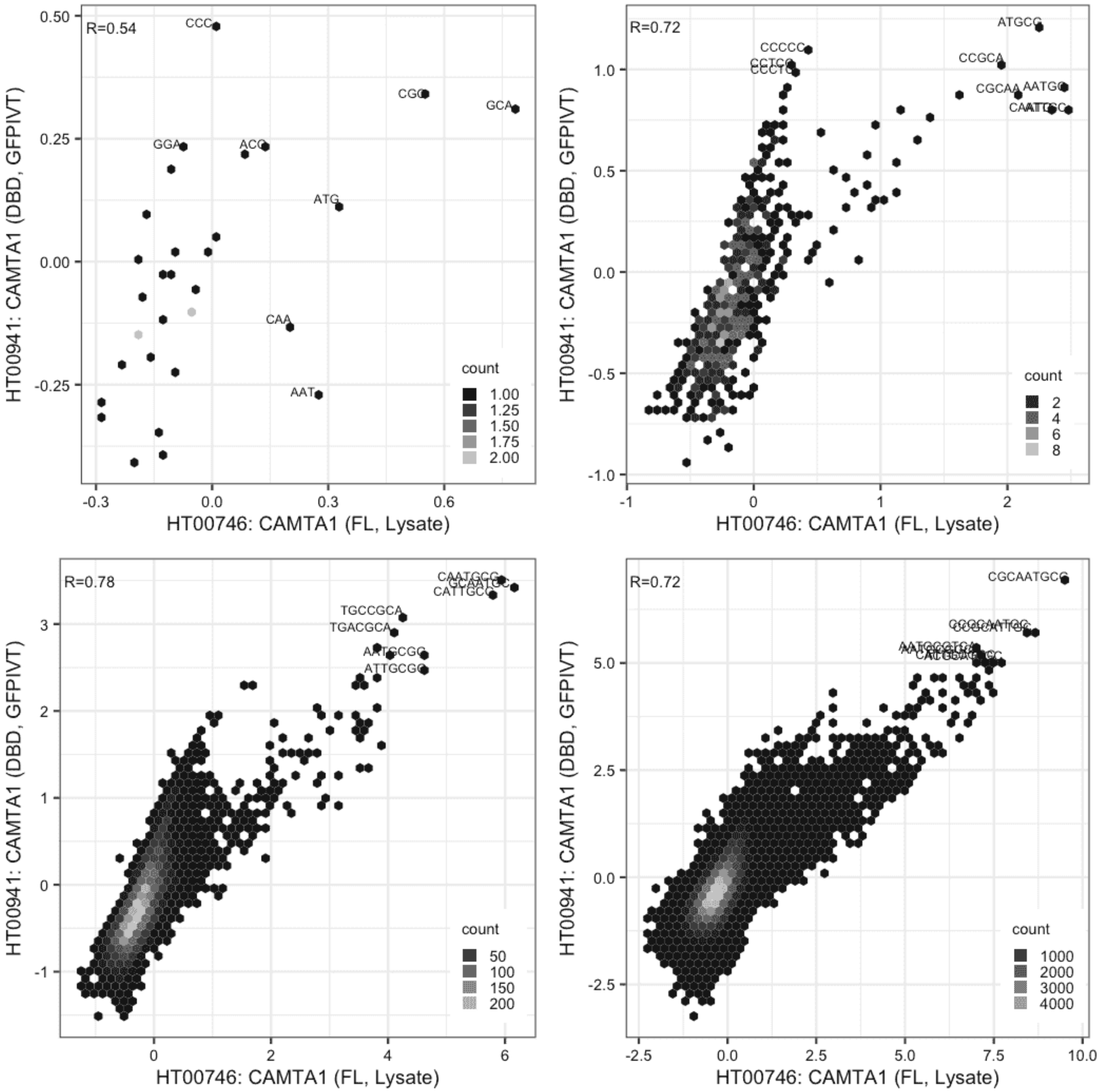

CASZ1

Lysate vs Lysate, same construct

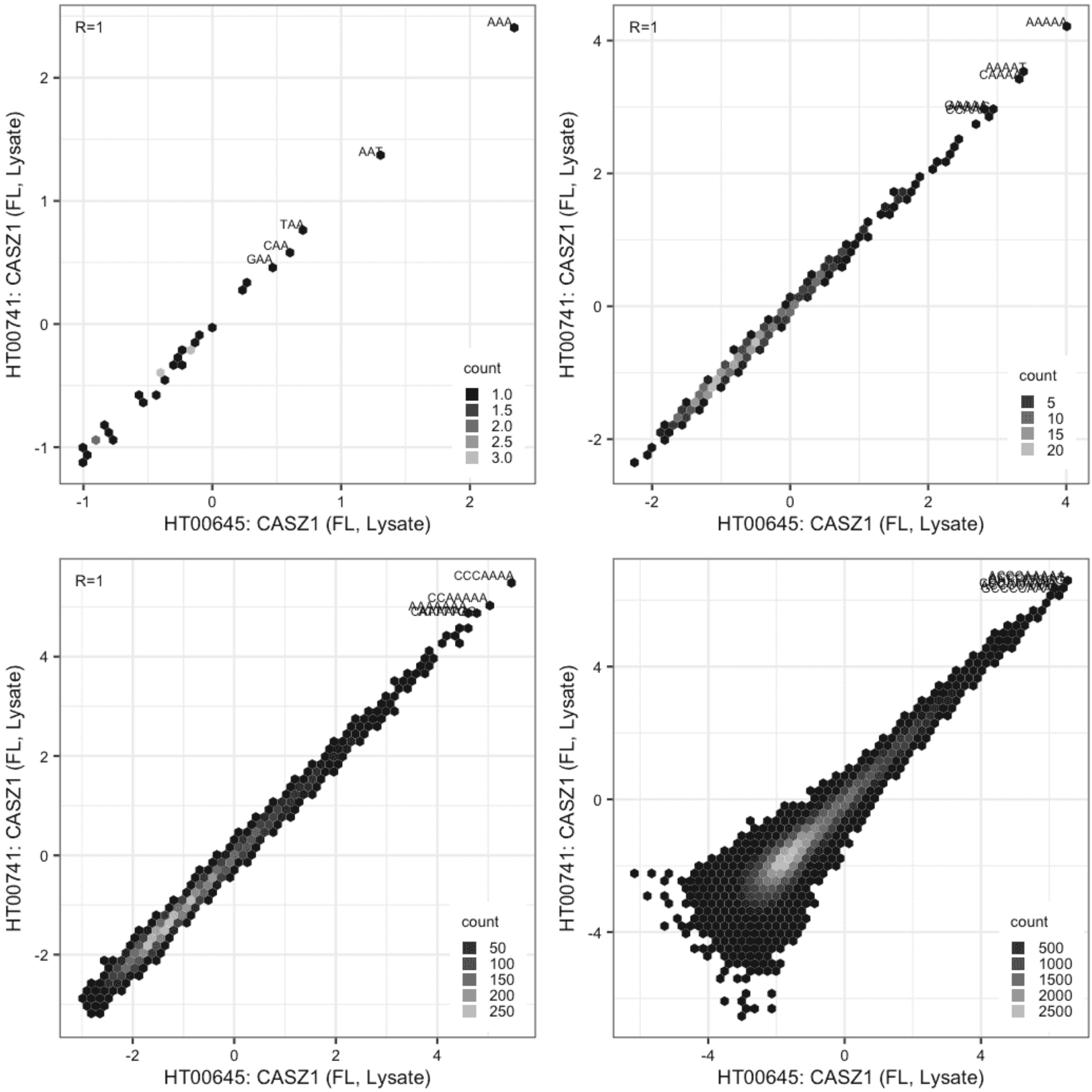

CASZ1

Lysate vs GFPIVT, same construct

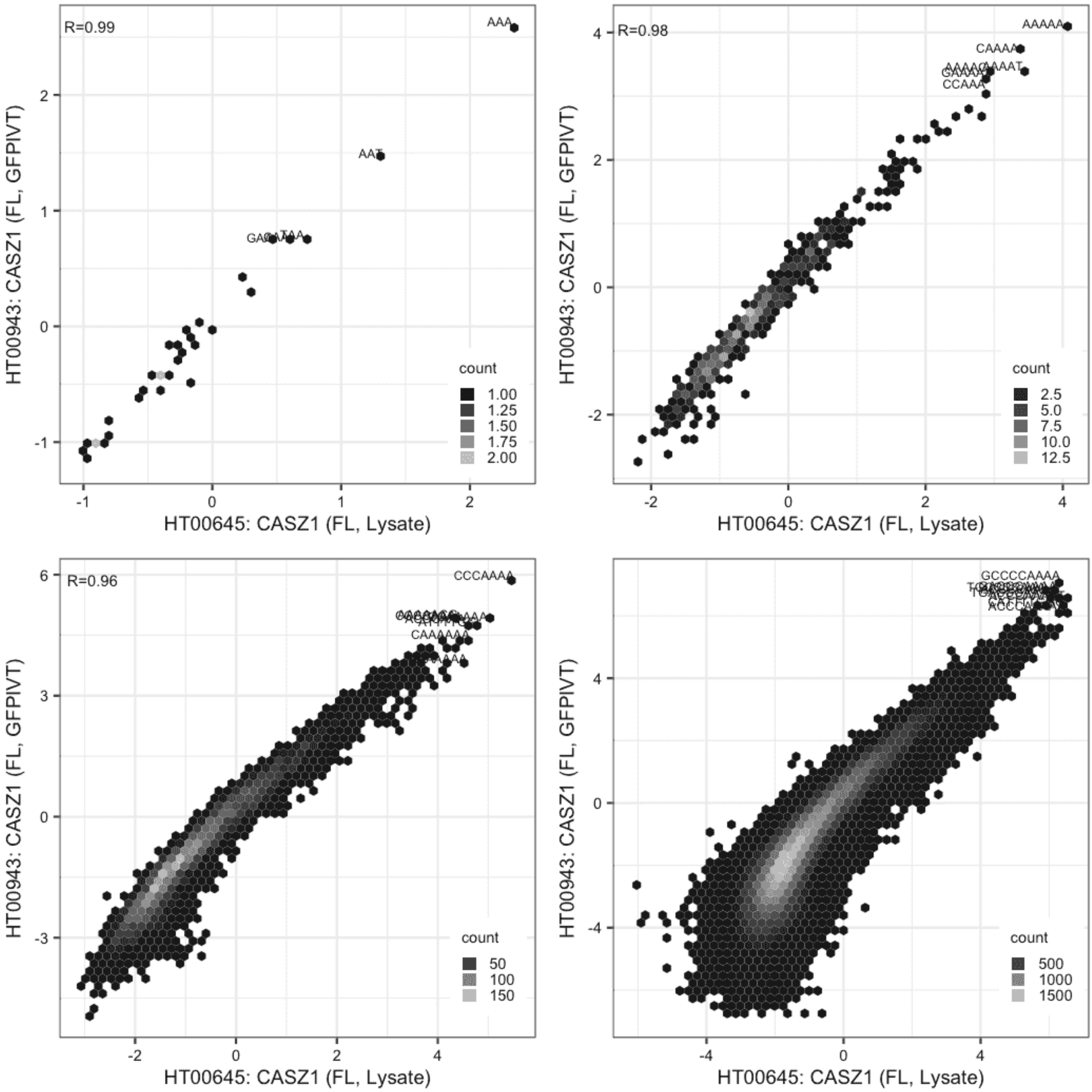

CASZ1

Lysate vs GFPIVT, same construct

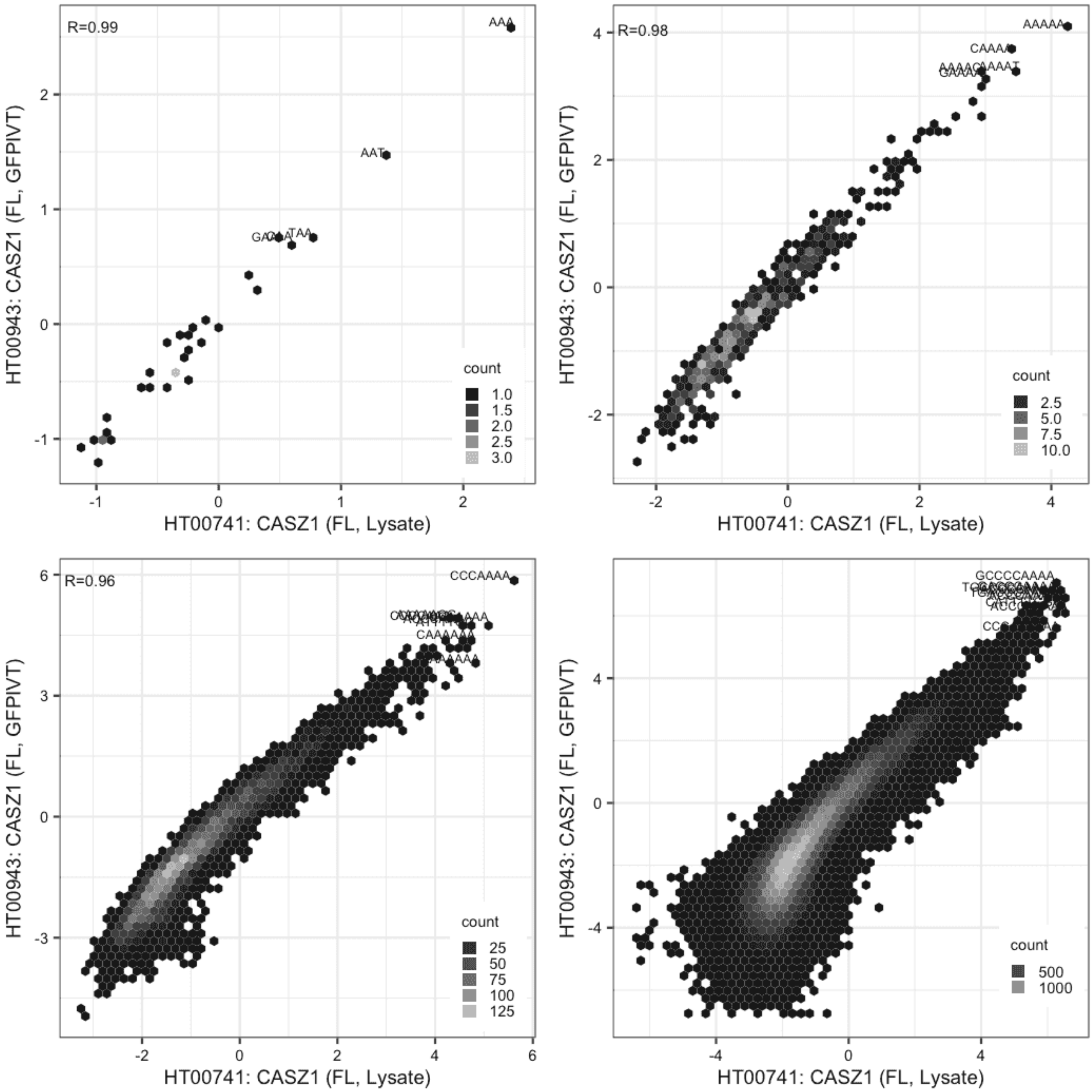

CREB3L3

IVT vs GFPIVT, different constructs

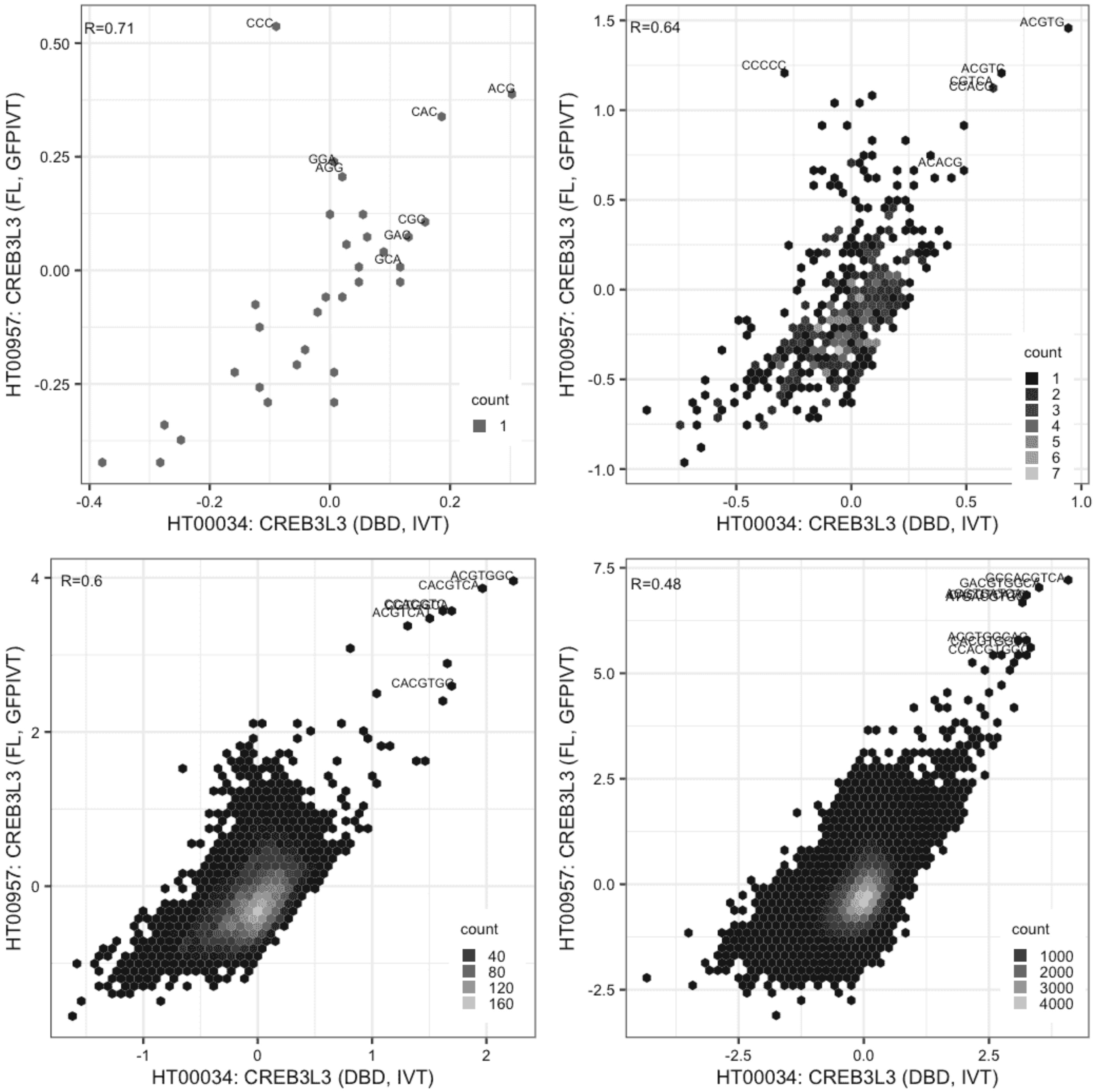

CREB3L3

Lysate vs IVT, different constructs

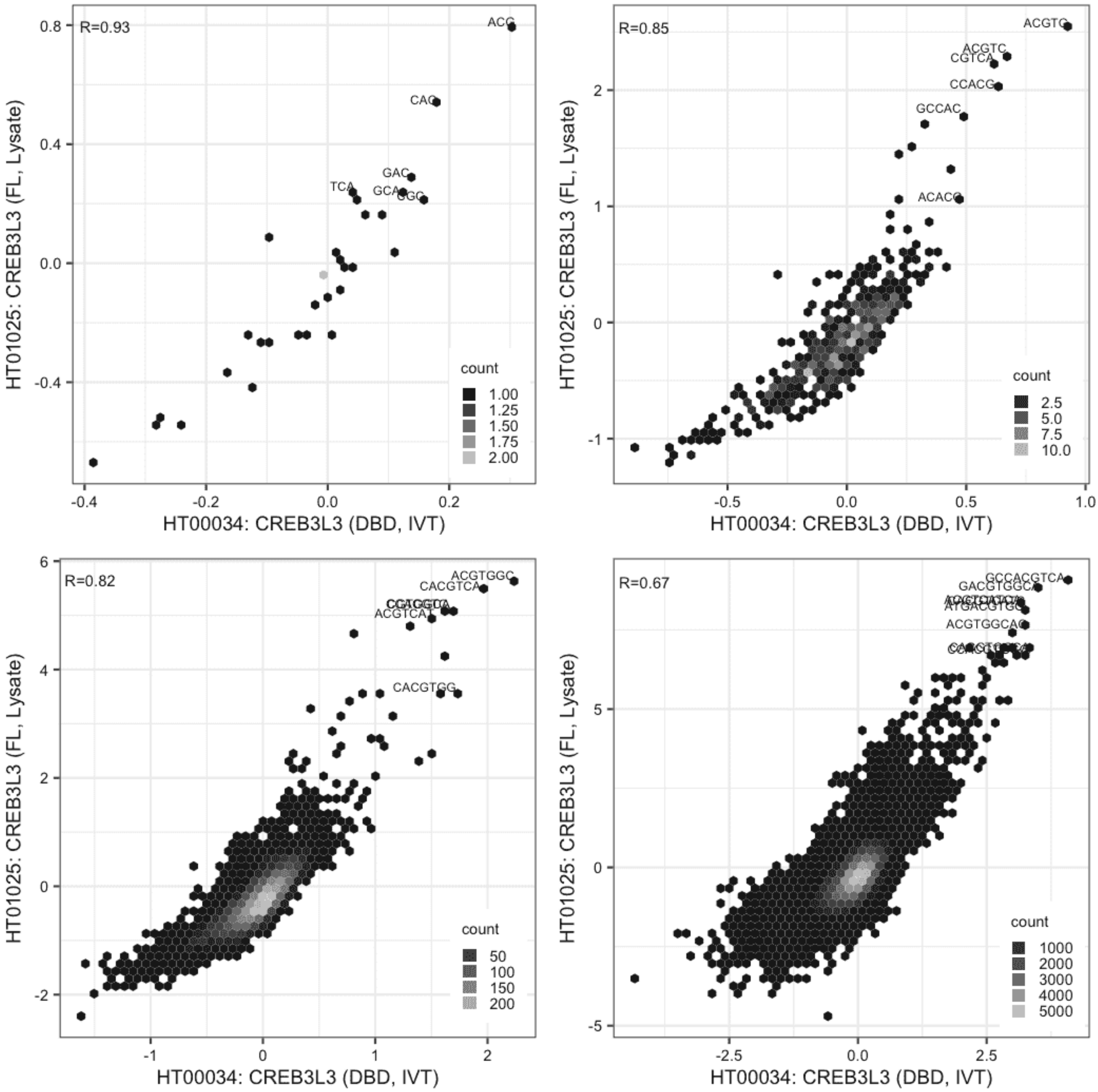

CREB3L3

Lysate vs IVT, different constructs

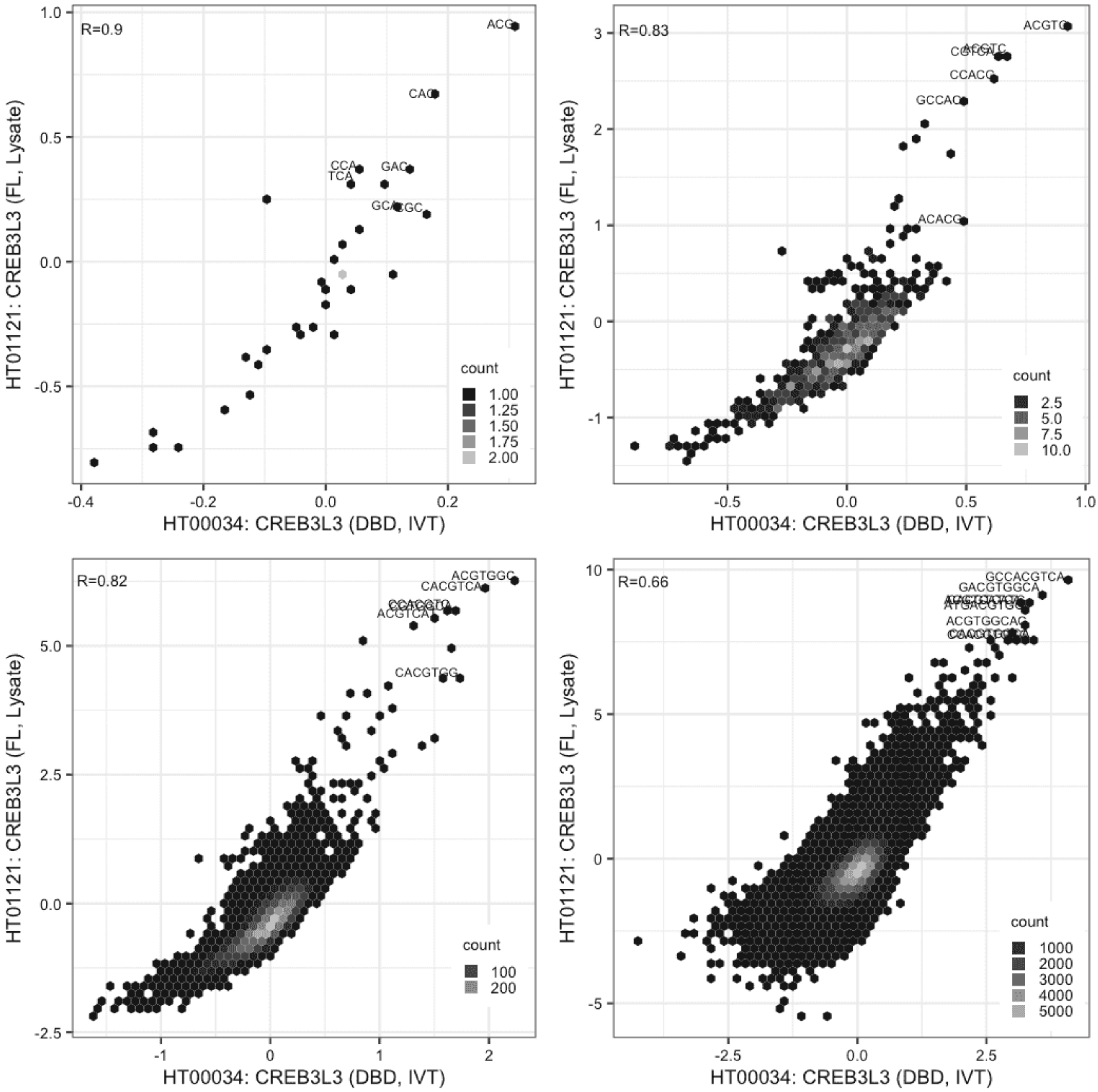

CREB3L3

Lysate vs GFPIVT, same construct

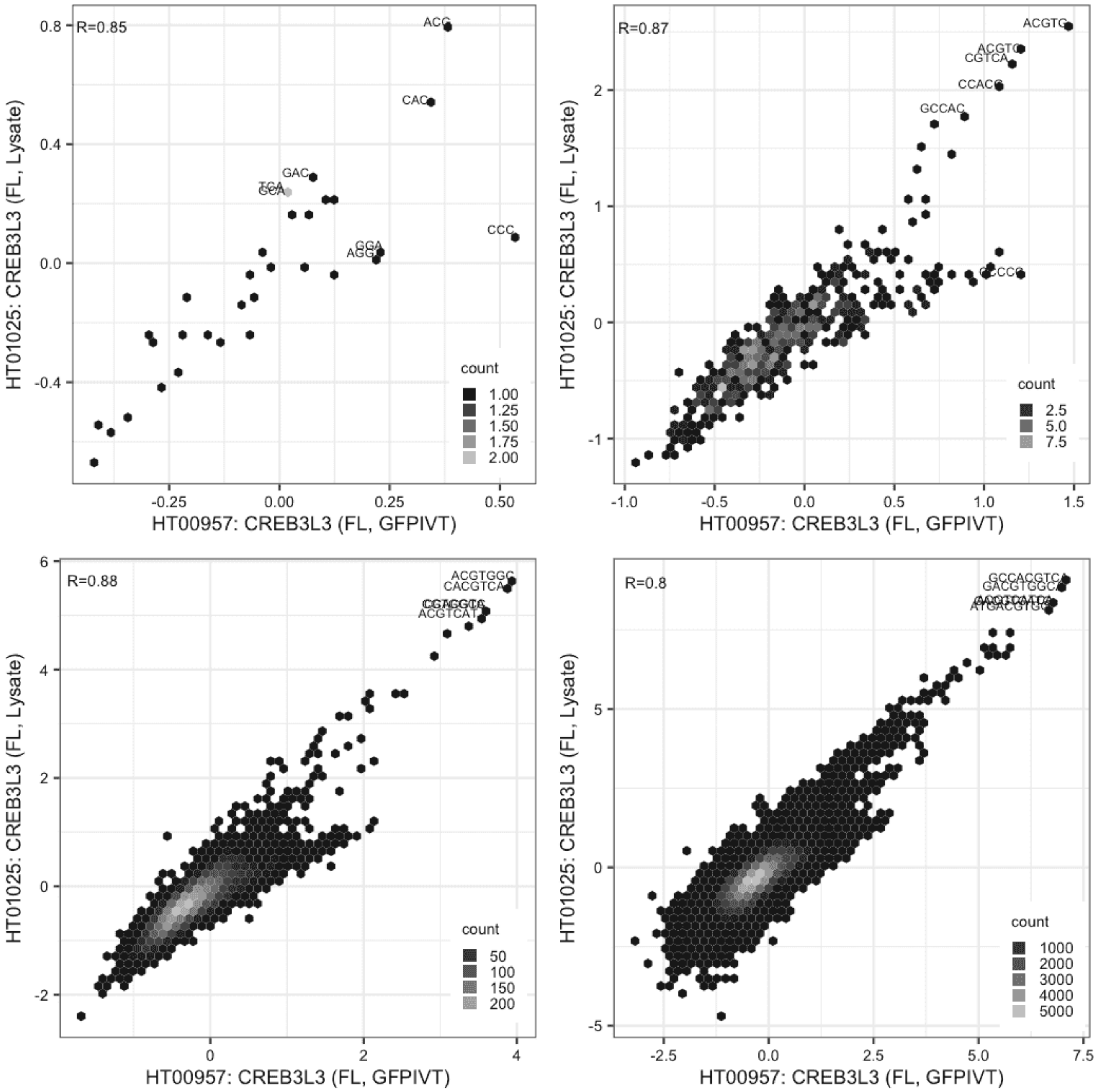

CREB3L3

Lysate vs GFPIVT, same construct

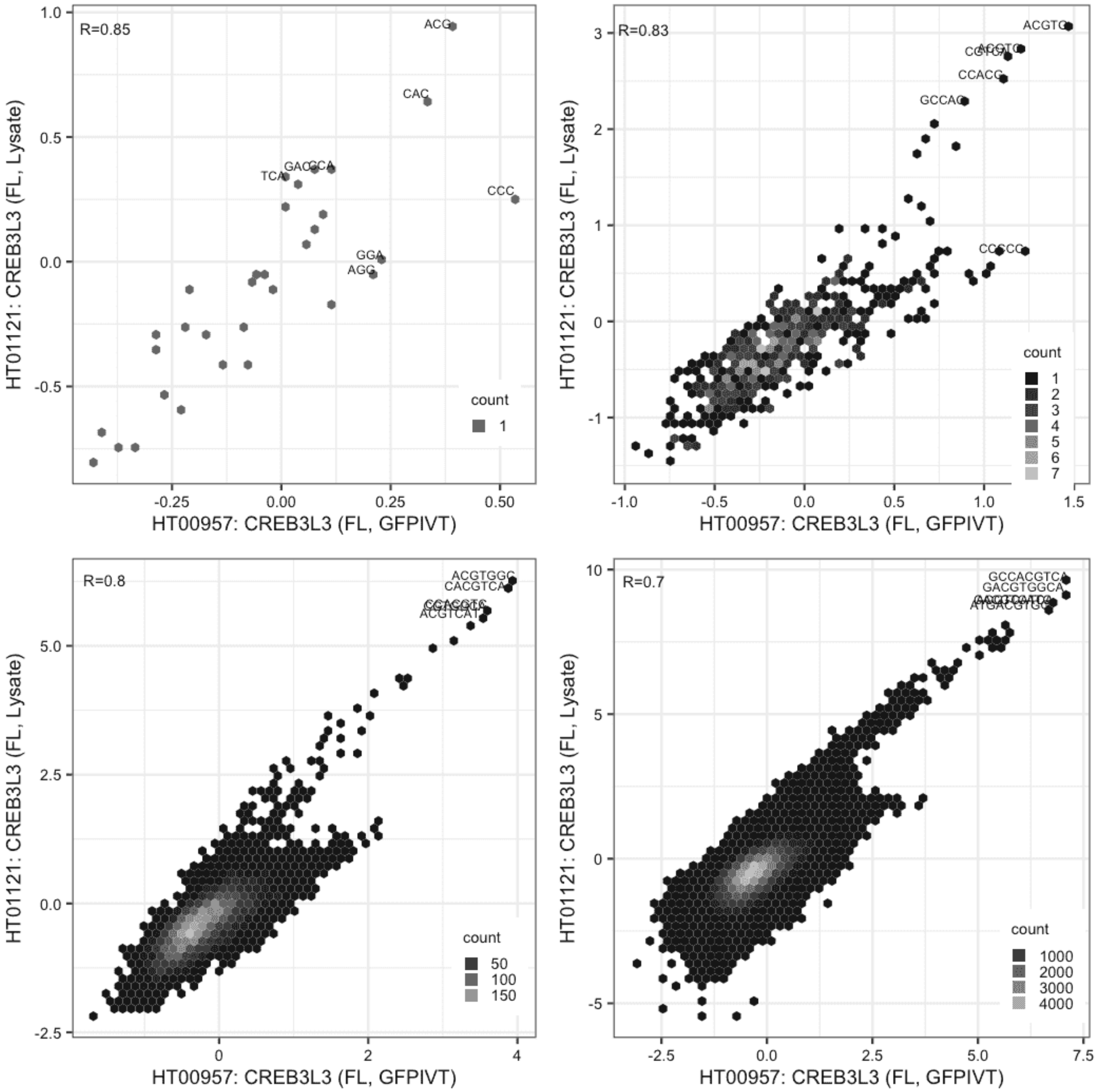

CREB3L3

Lysate vs Lysate, same construct

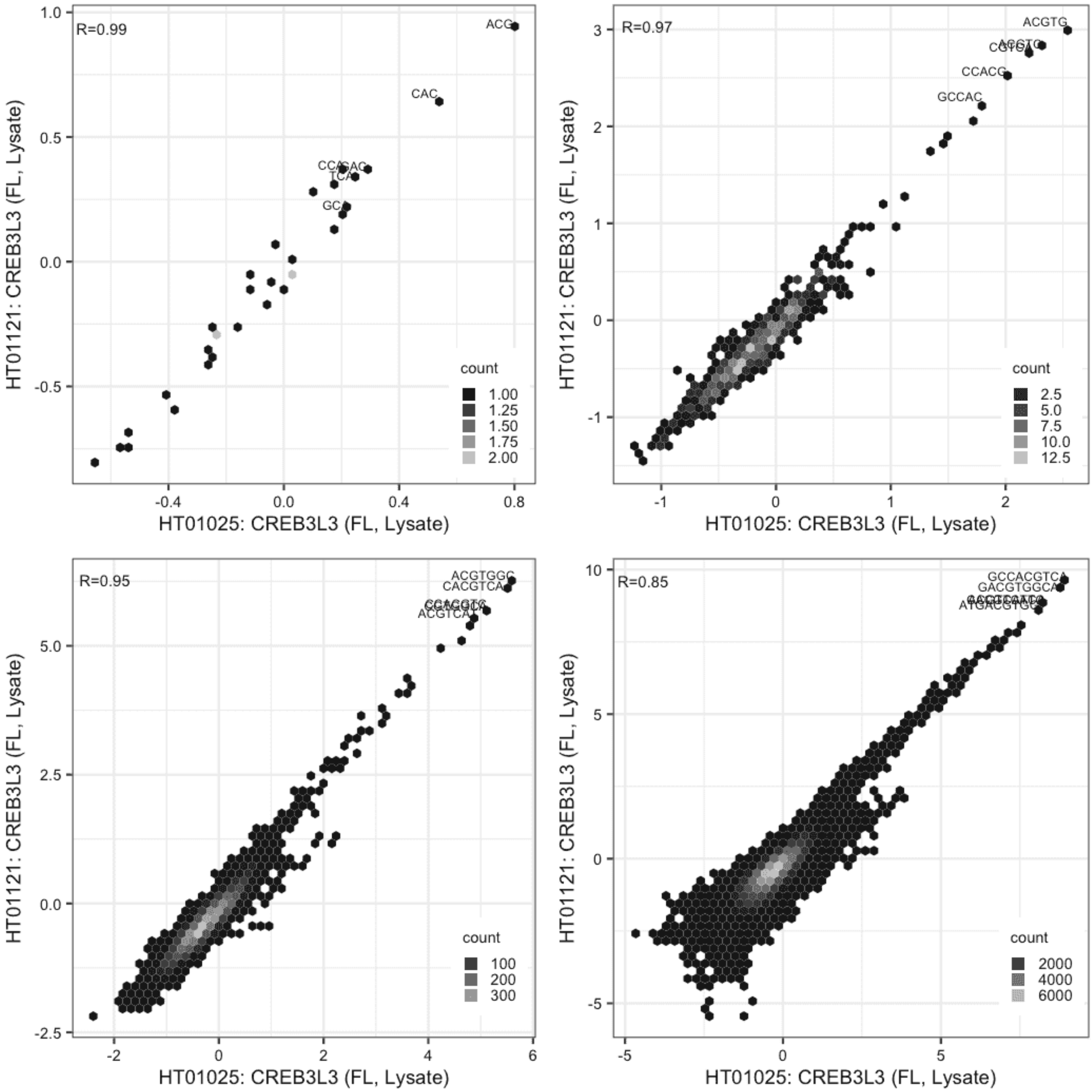

CXXC4

IVT vs IVT, different constructs

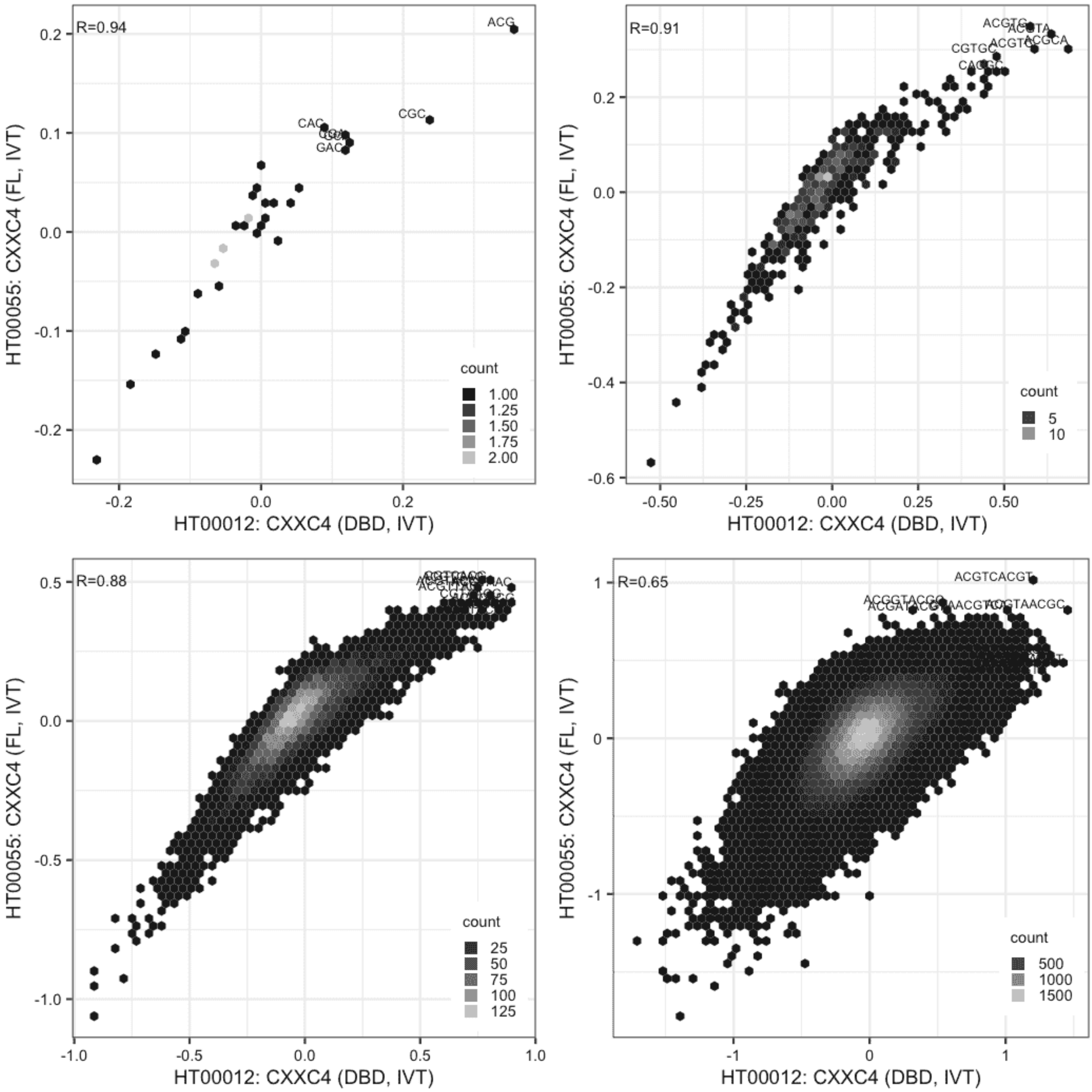

CXXC4

Lysate vs IVT, different constructs

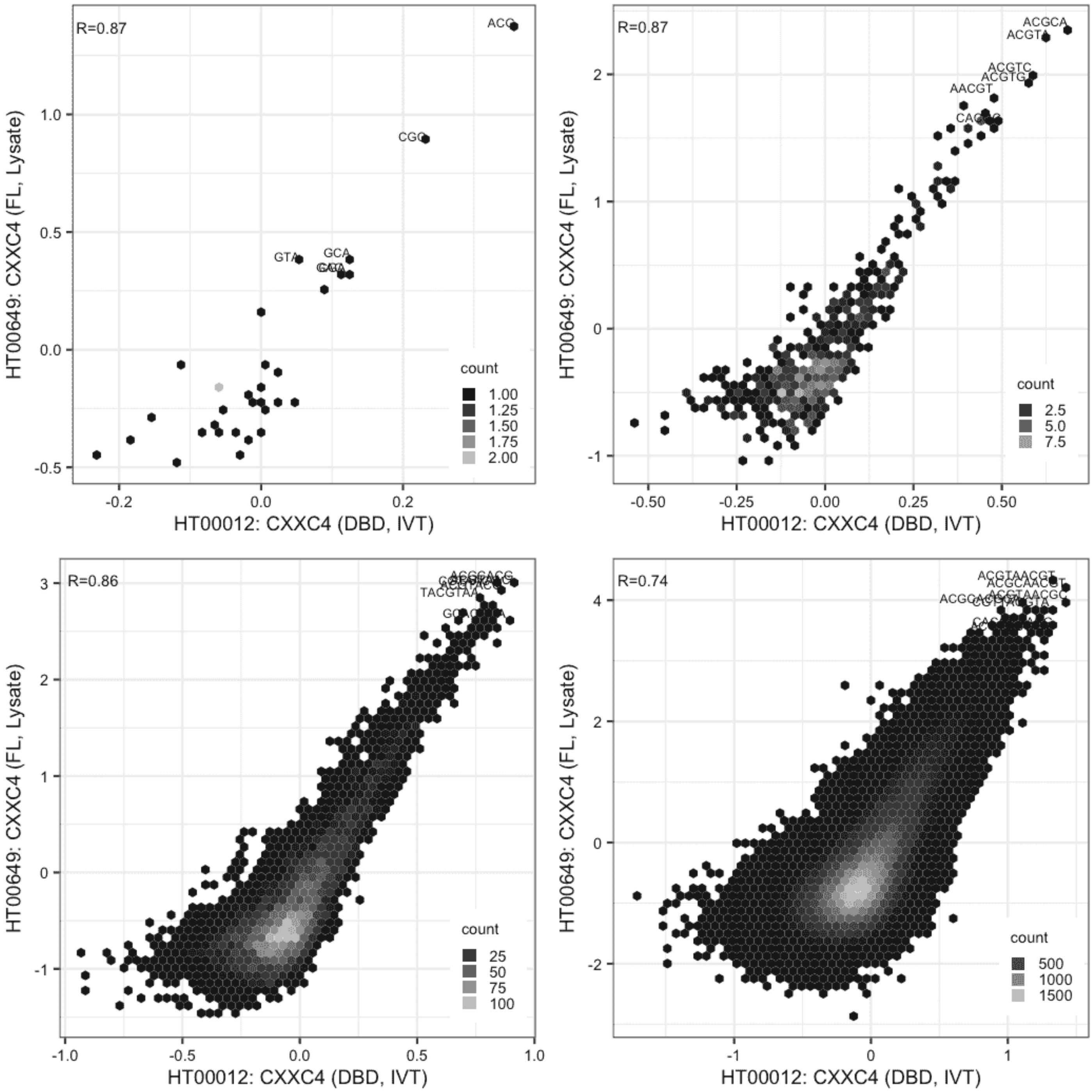

CXXC4

Lysate vs IVT, different constructs

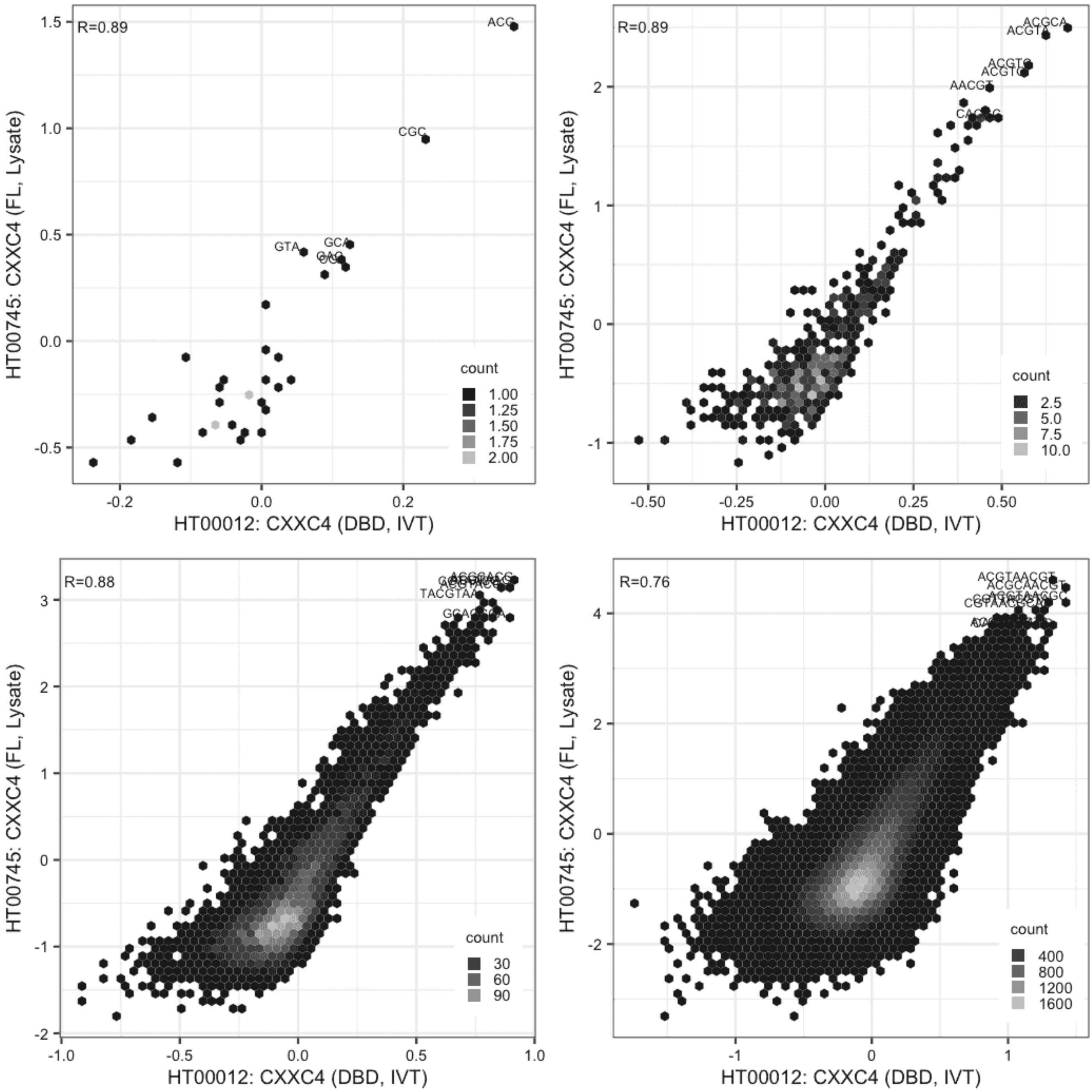

CXXC4

IVT vs GFPIVT, different constructs

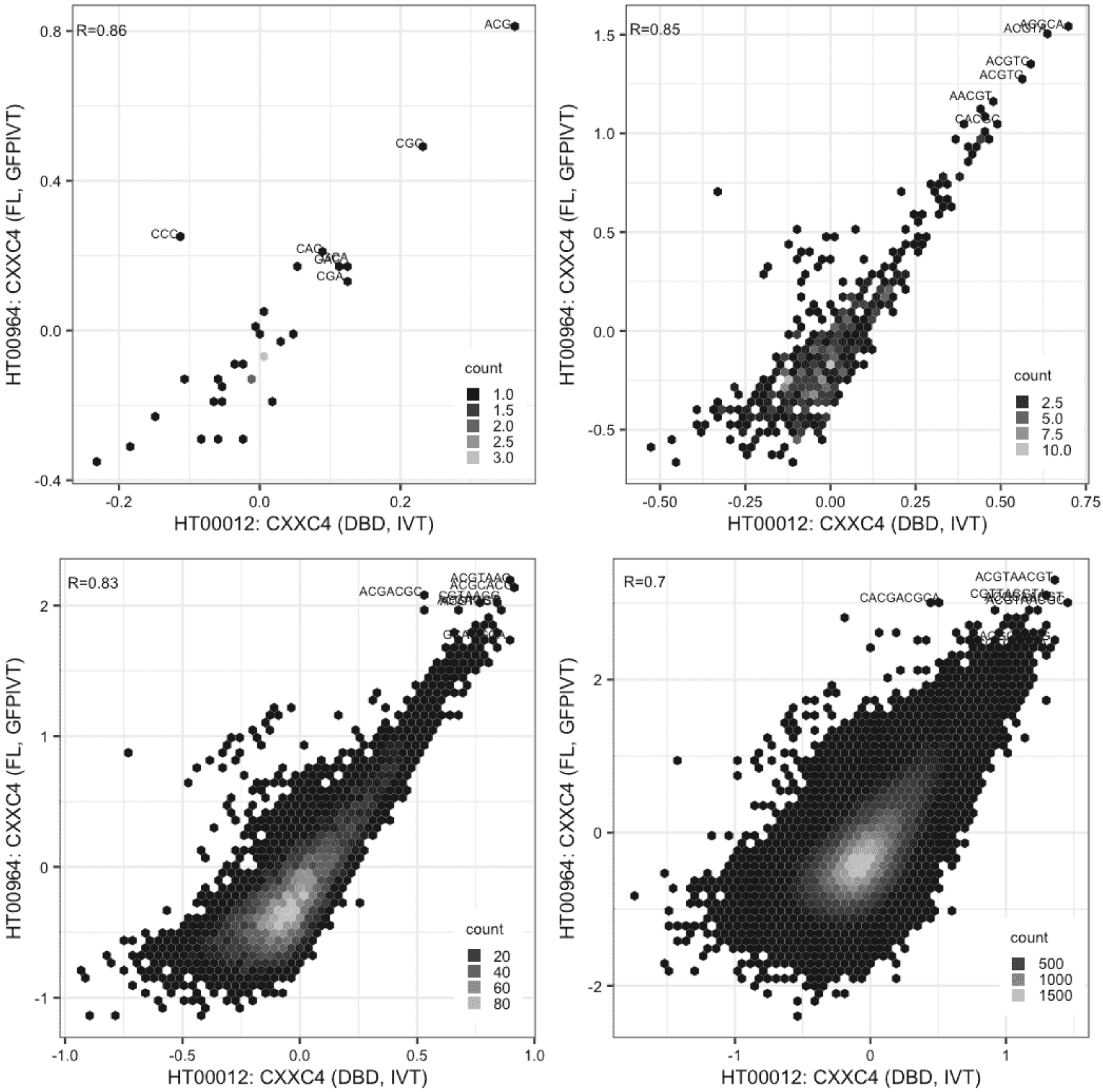

CXXC4

Lysate vs IVT, same construct

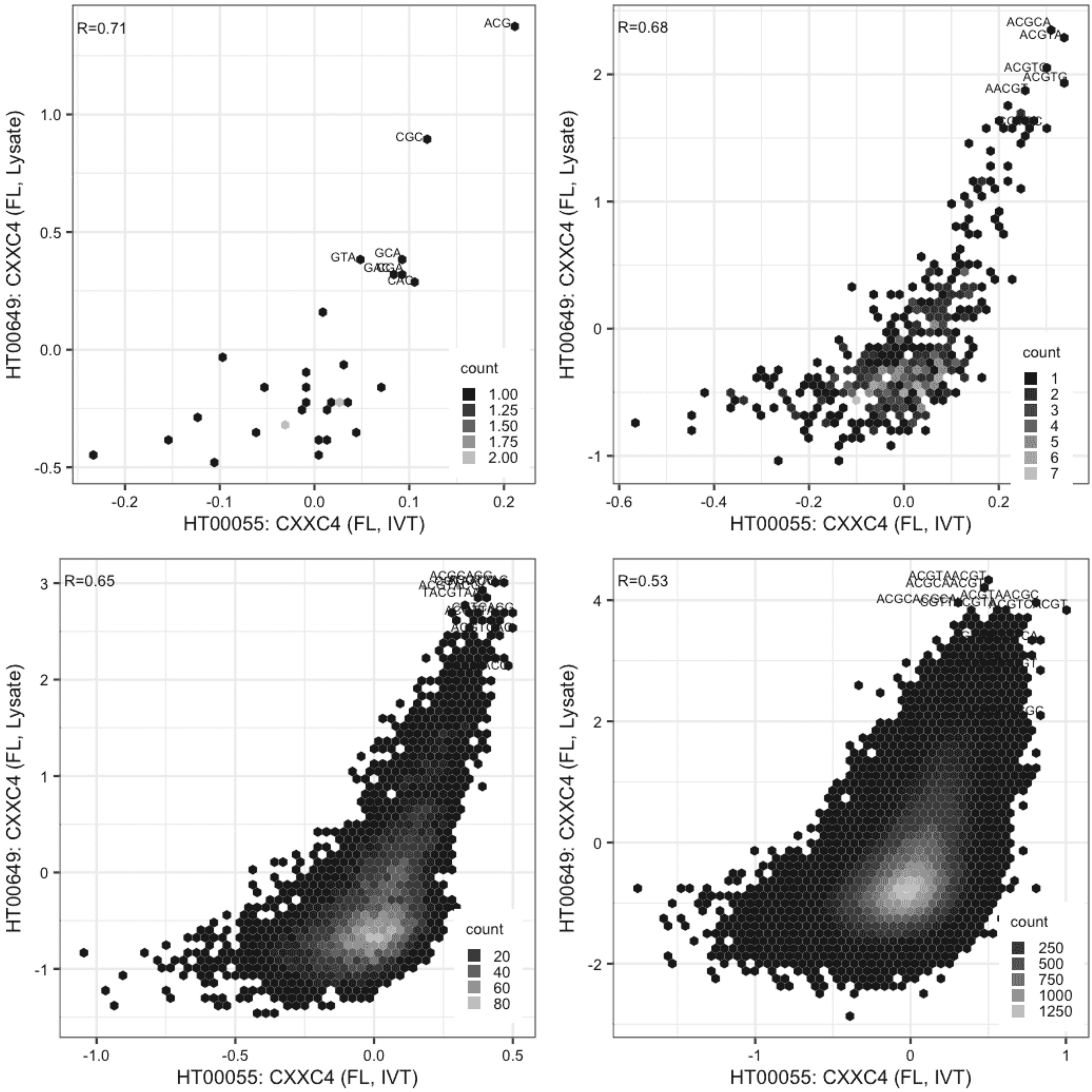

CXXC4

Lysate vs IVT, same construct

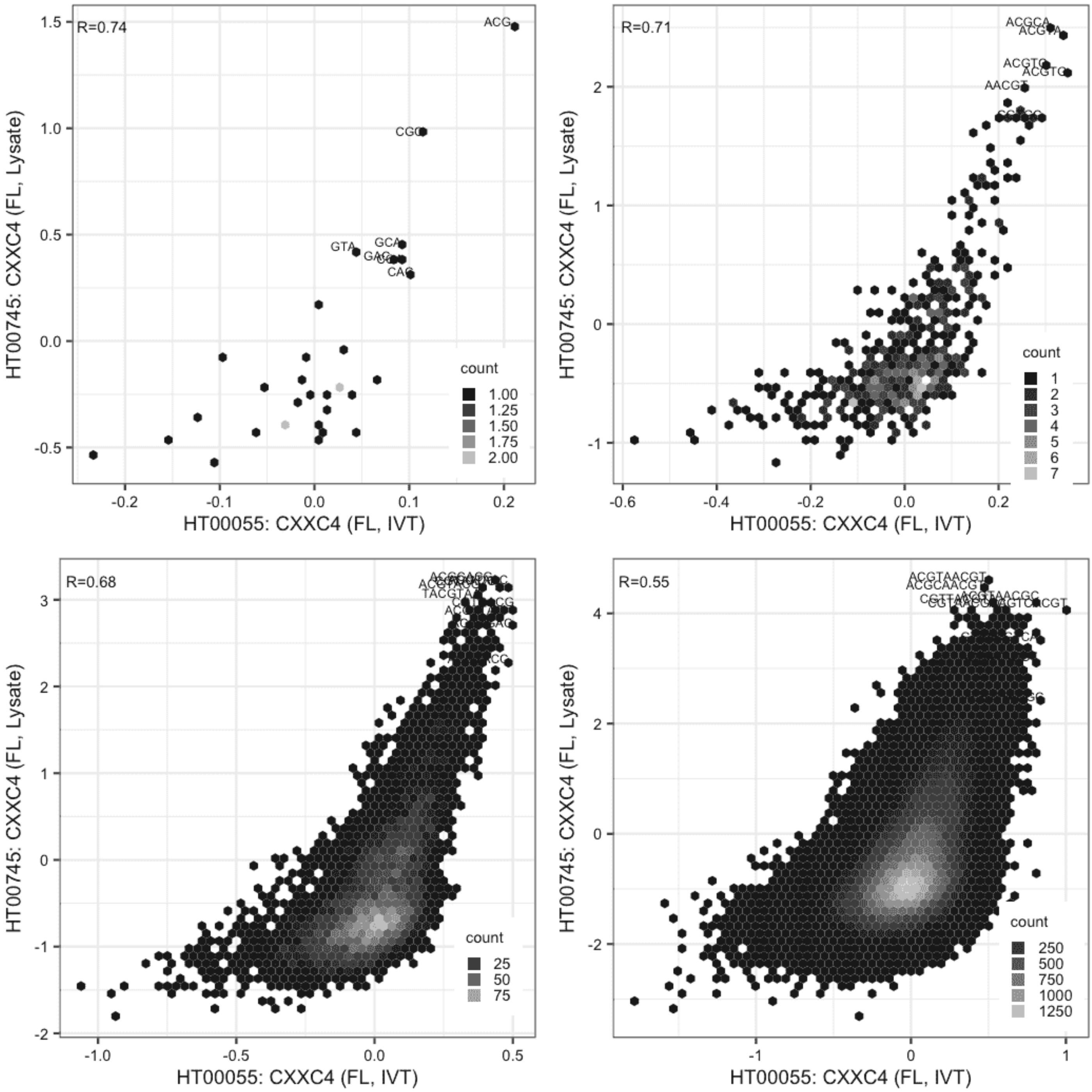

CXXC4

IVT vs GFPIVT, same construct

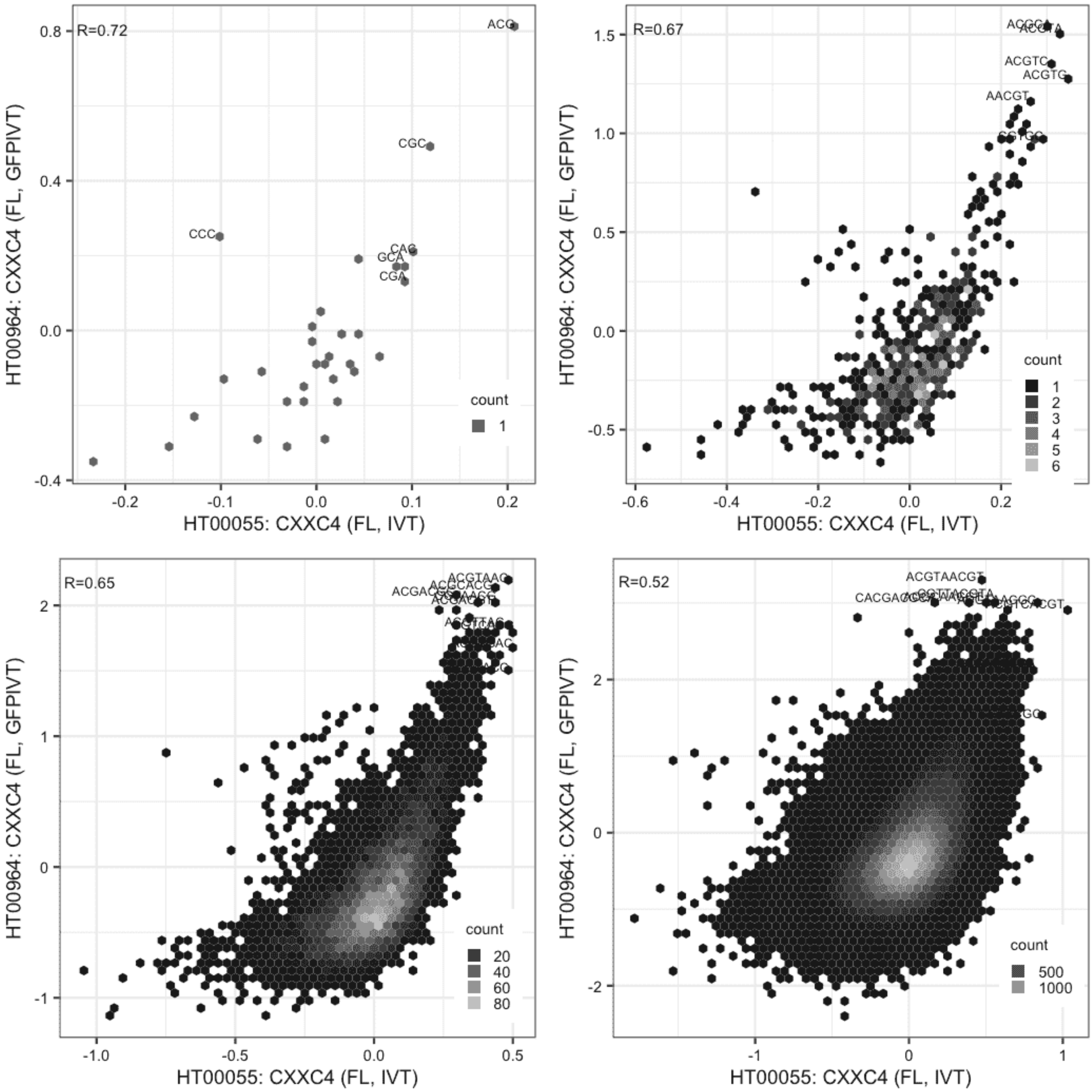

CXXC4

Lysate vs Lysate, same construct

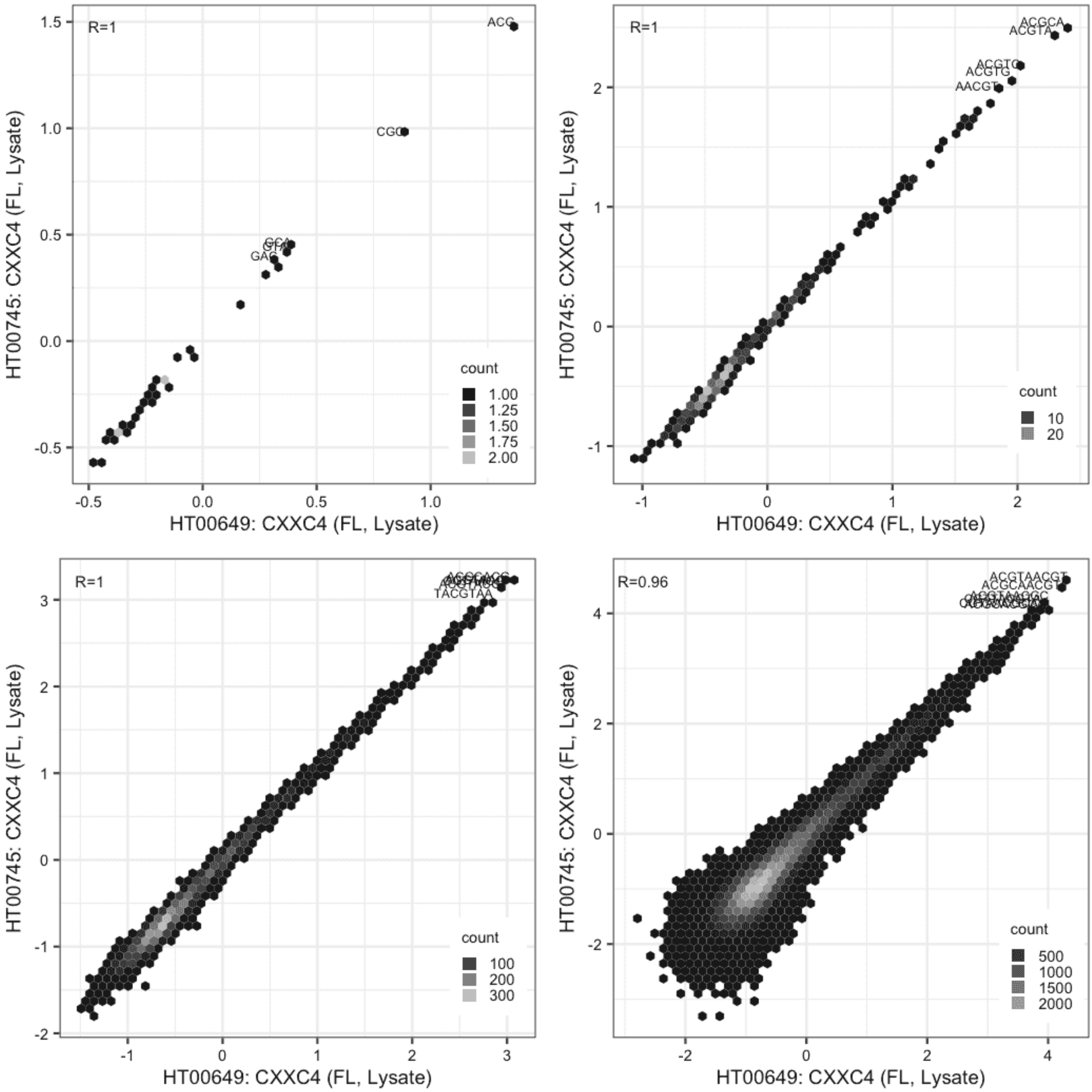

CXXC4

Lysate vs GFPIVT, same construct

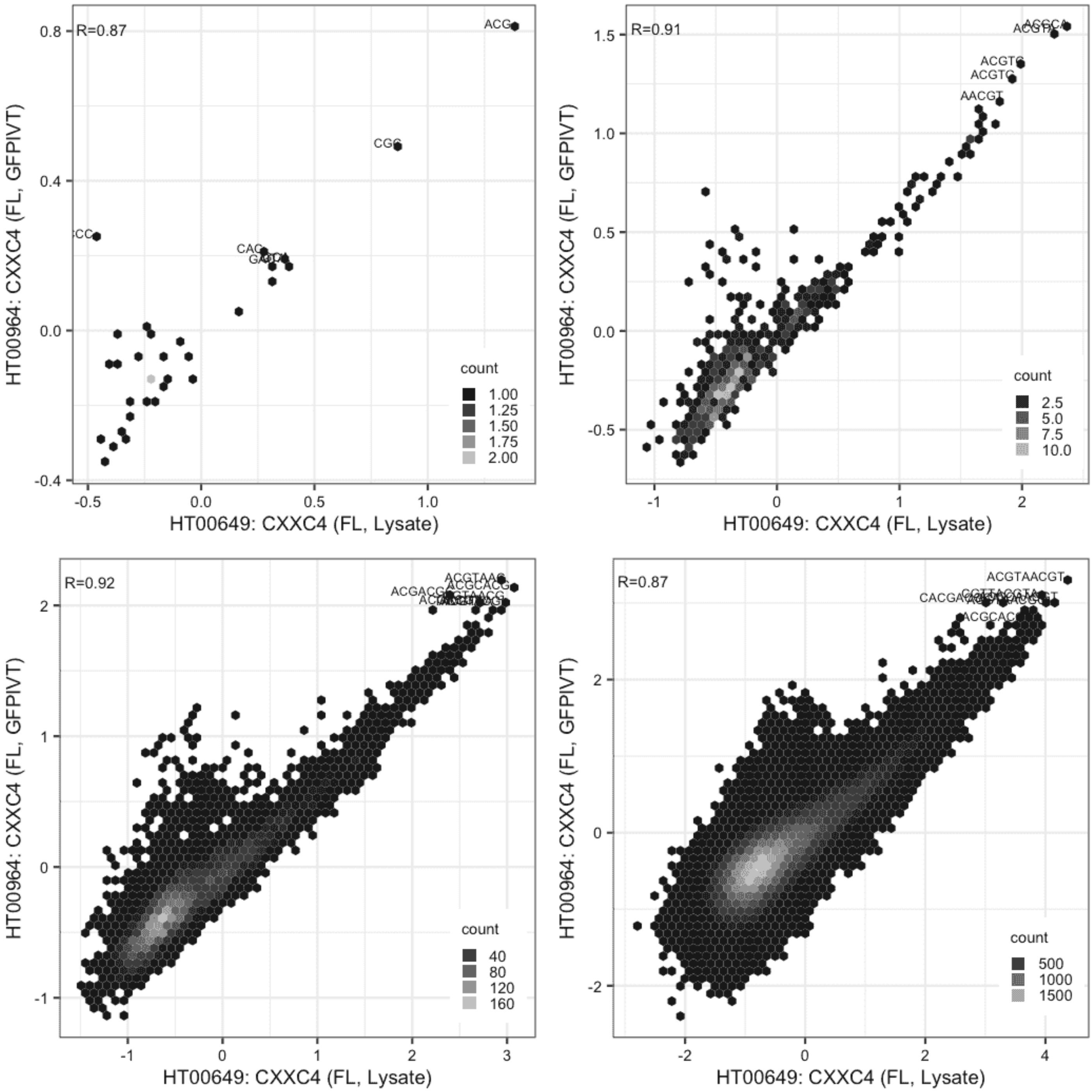

CXXC4

Lysate vs GFPIVT, same construct

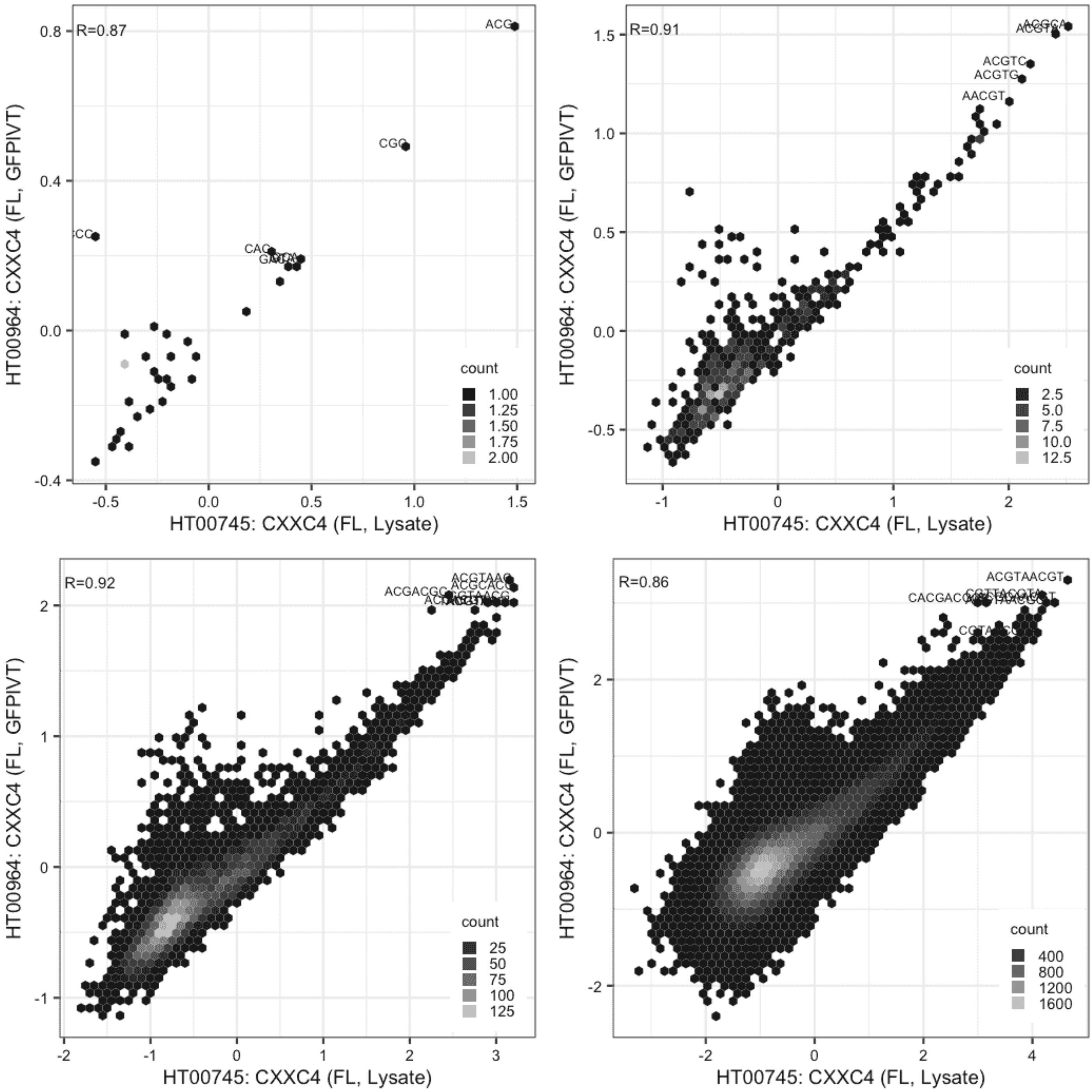

DACH1

Lysate vs GFPIVT, different constructs

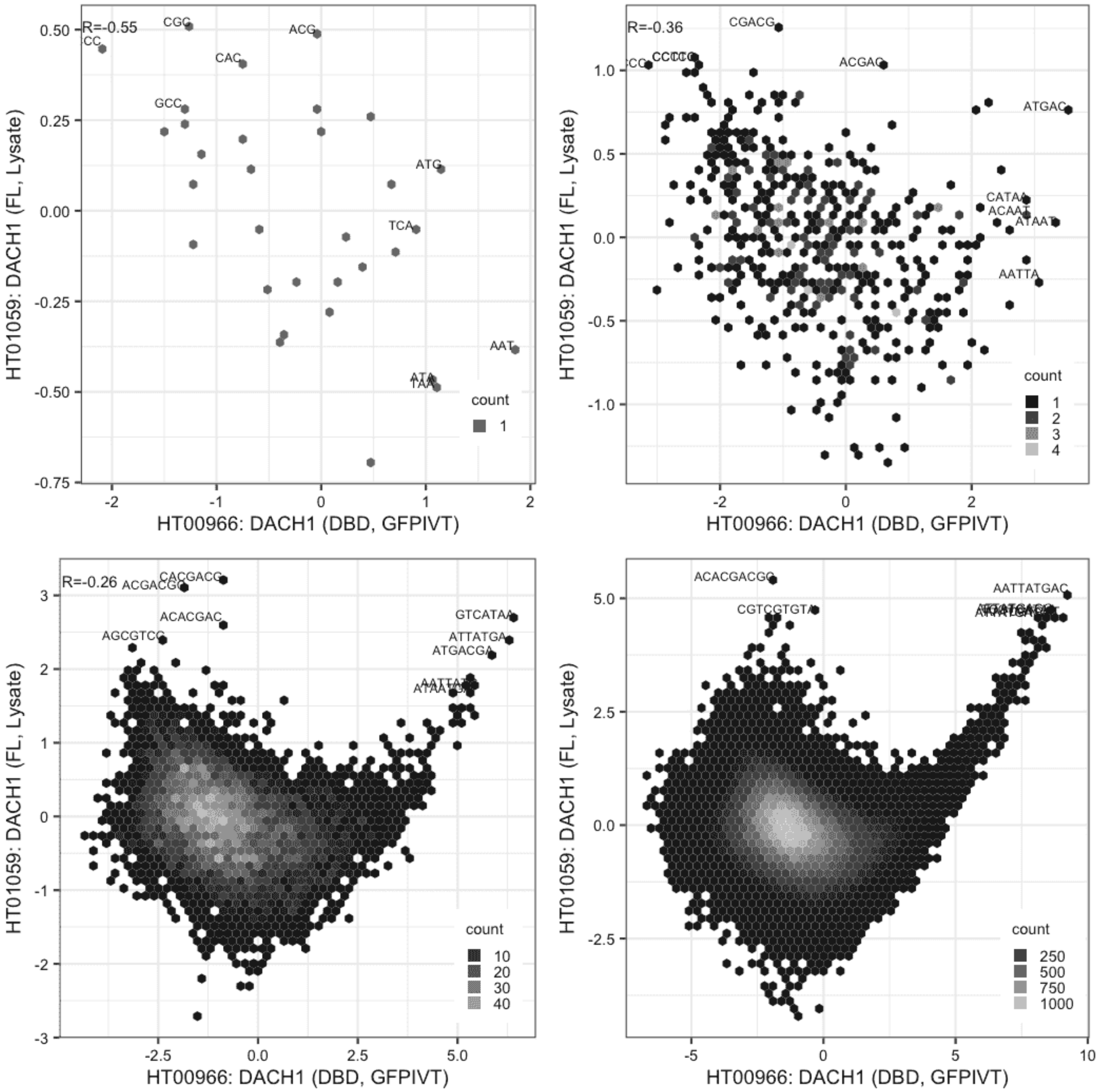

DACH1

Lysate vs GFPIVT, different constructs

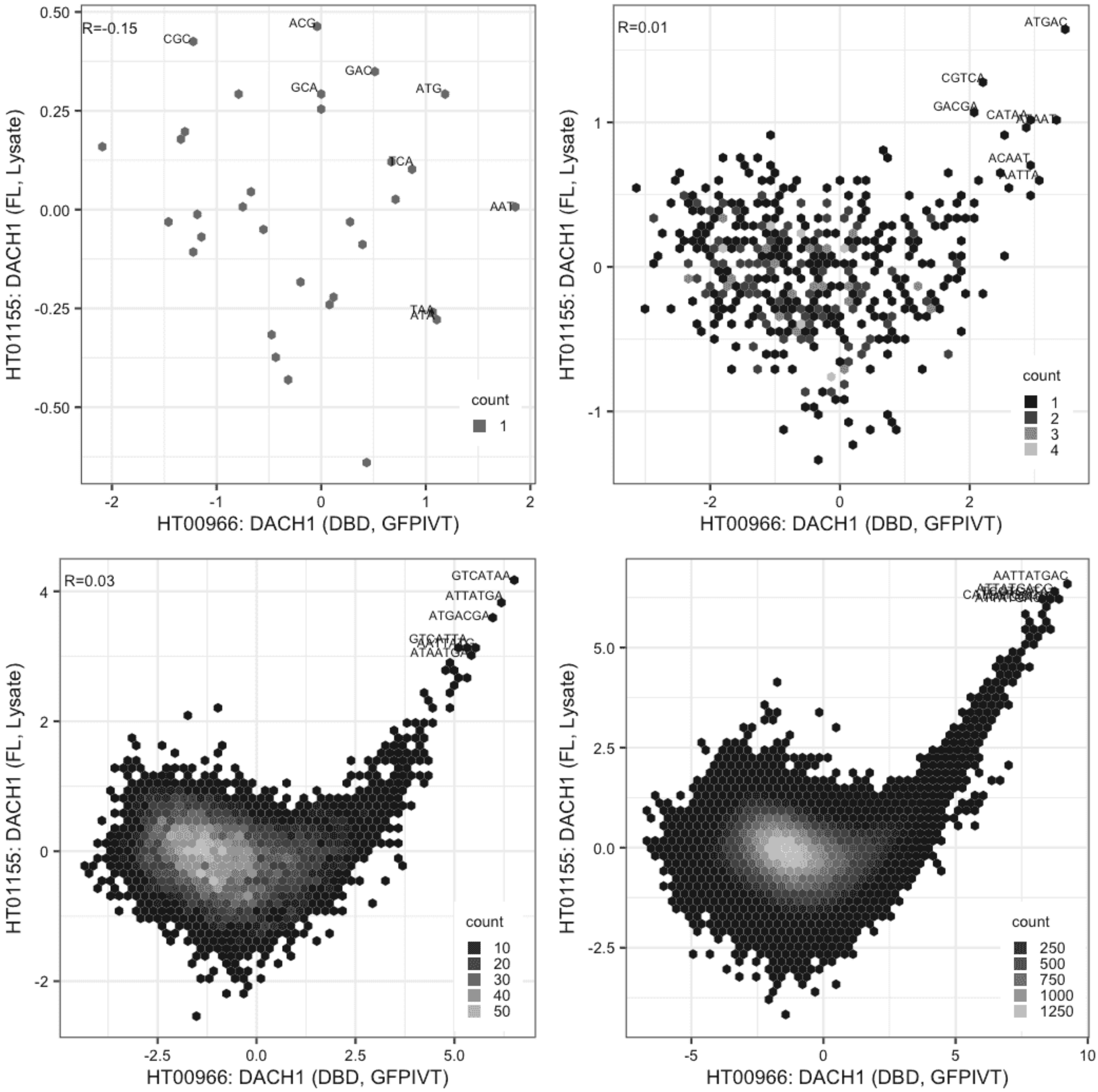

DACH1

Lysate vs Lysate, same construct

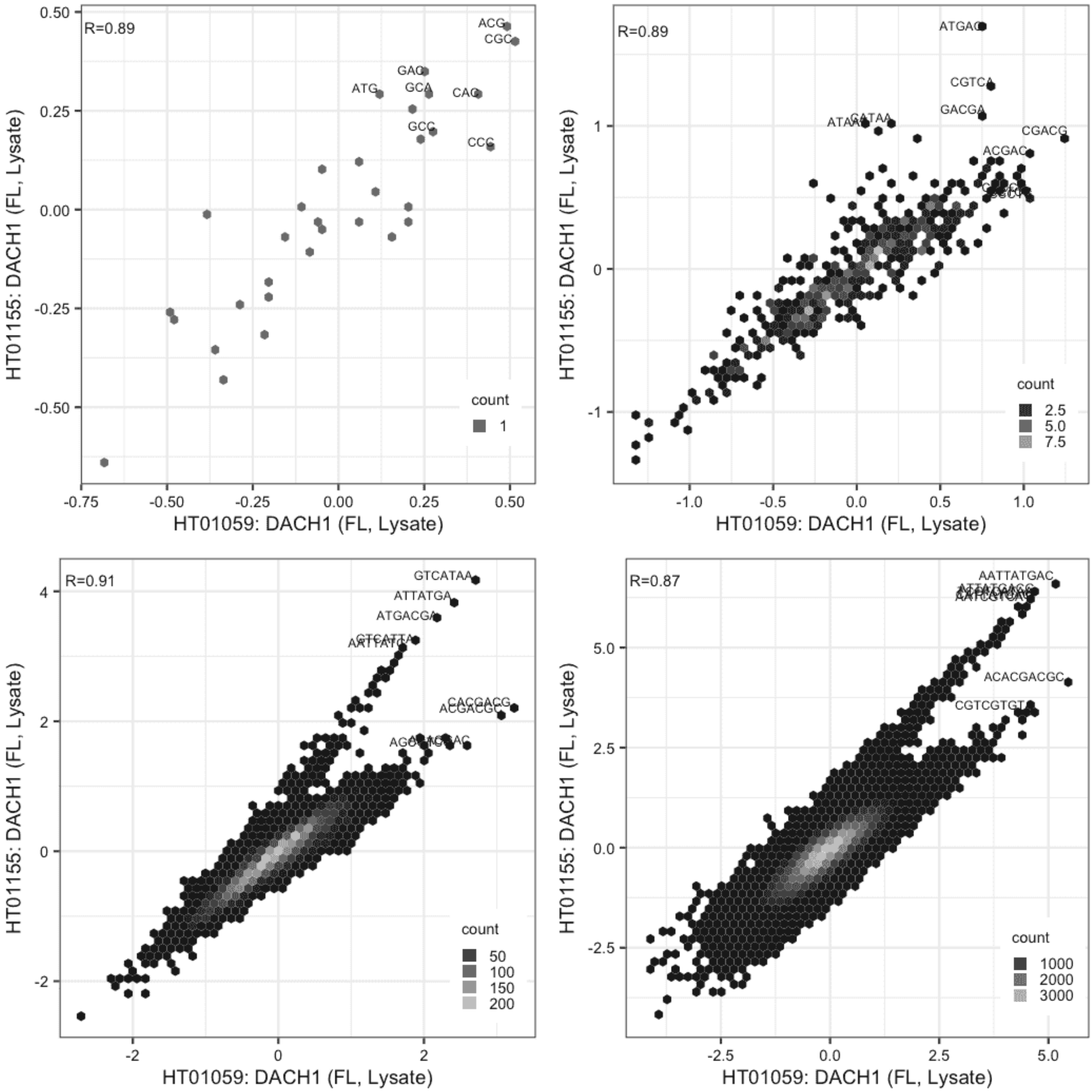

DMTF1

Lysate vs GFPIVT, same construct

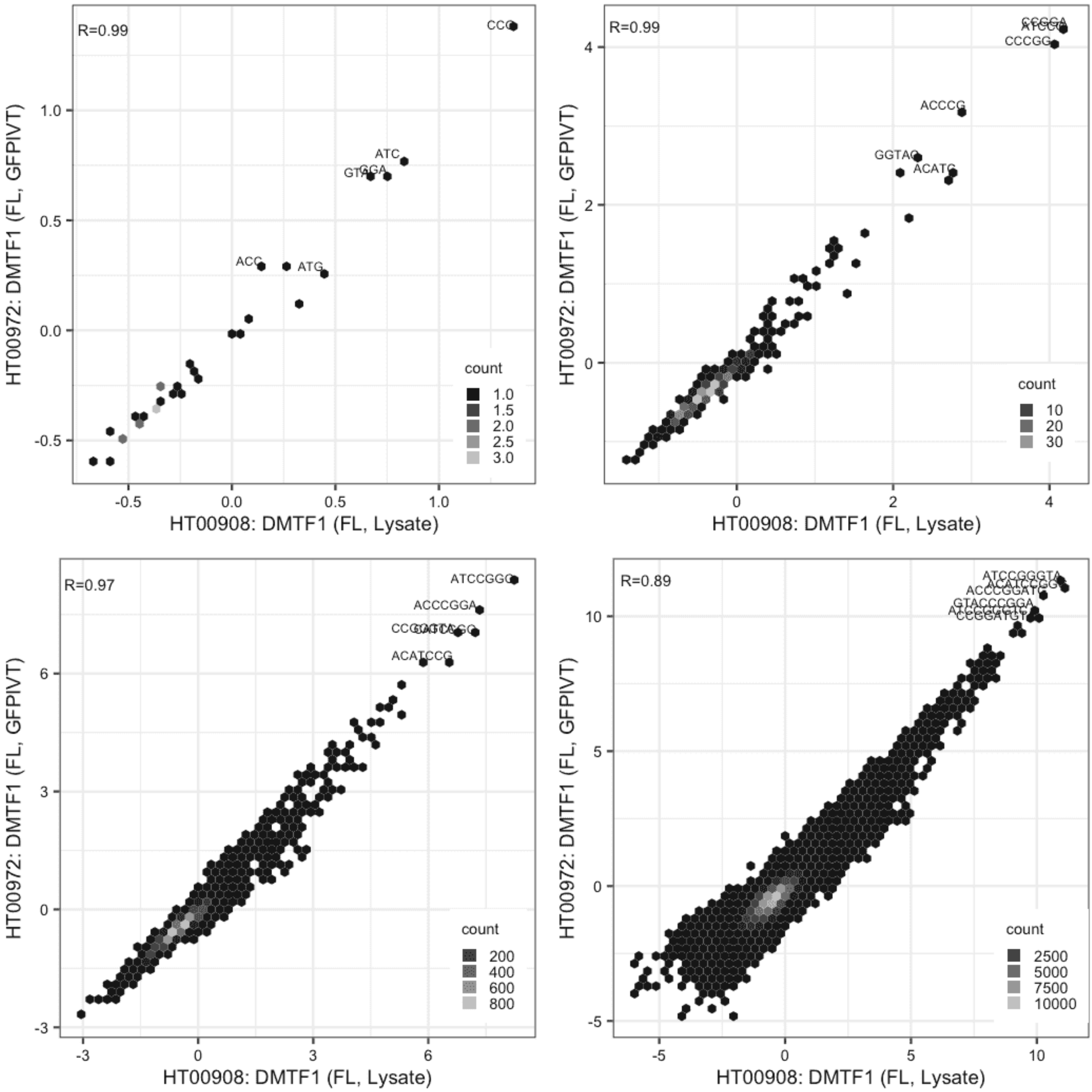

DNTTIP1

Lysate vs GFPIVT, same construct

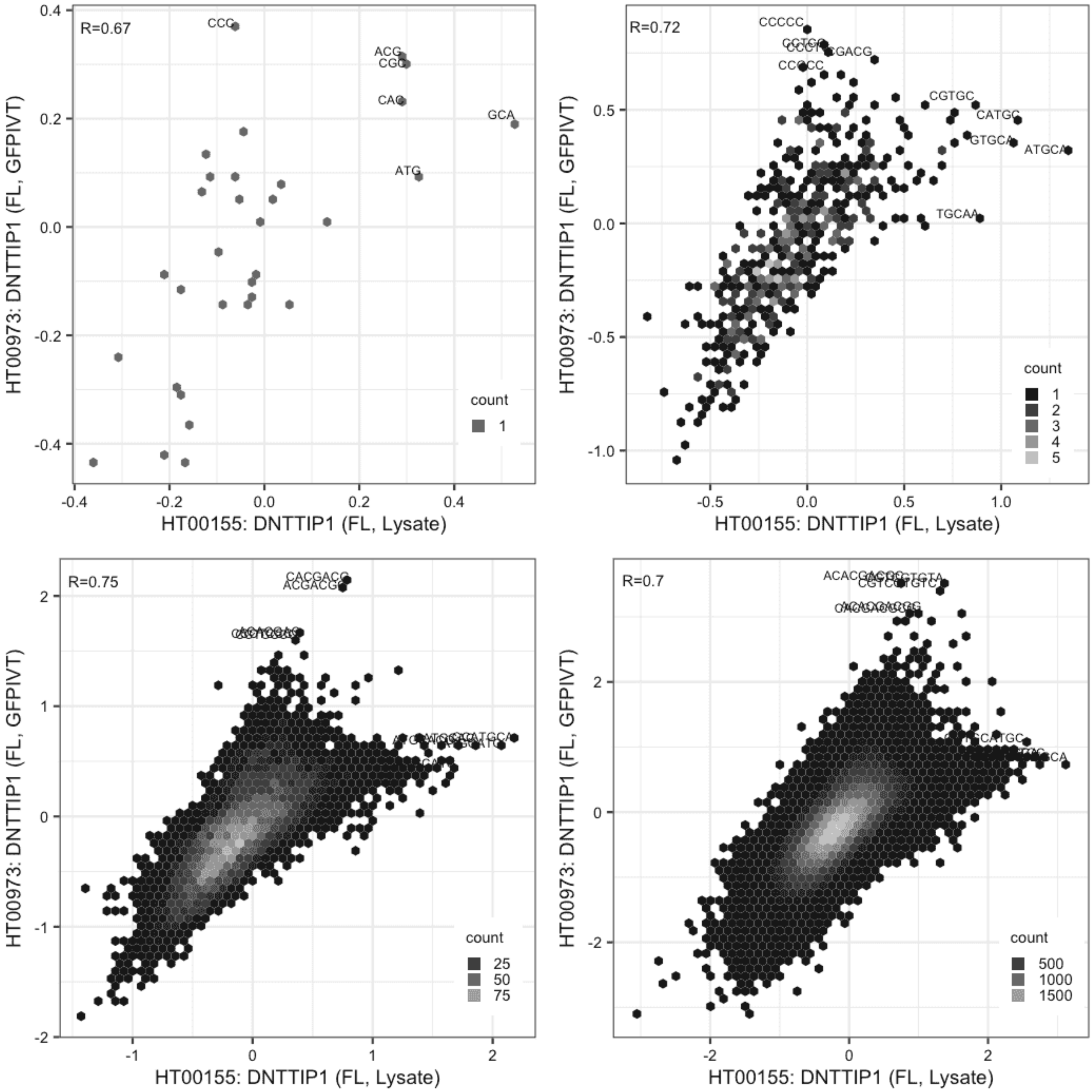

ELF3

Lysate vs GFPIVT, same construct

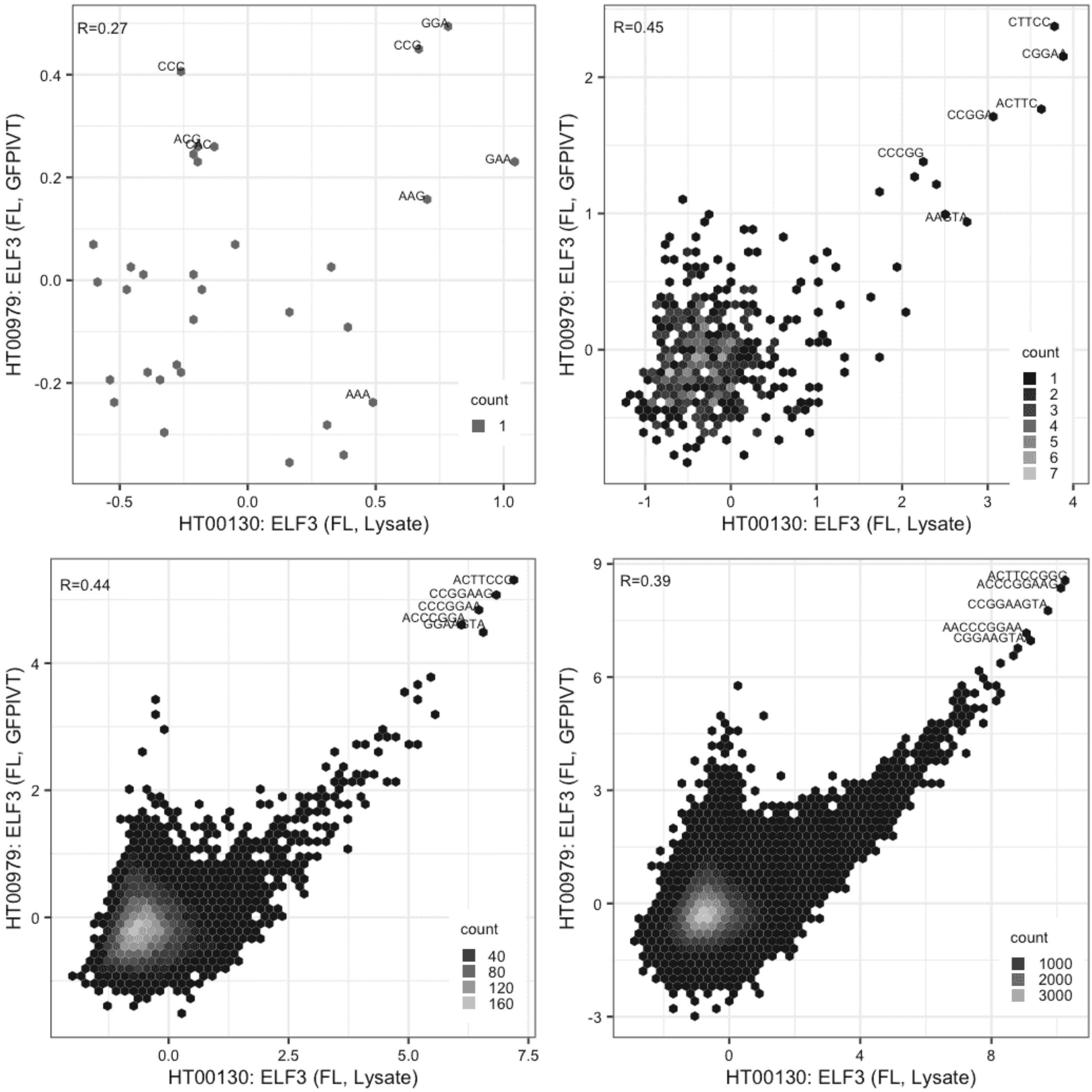

FBXL19

Lysate vs IVT, different constructs

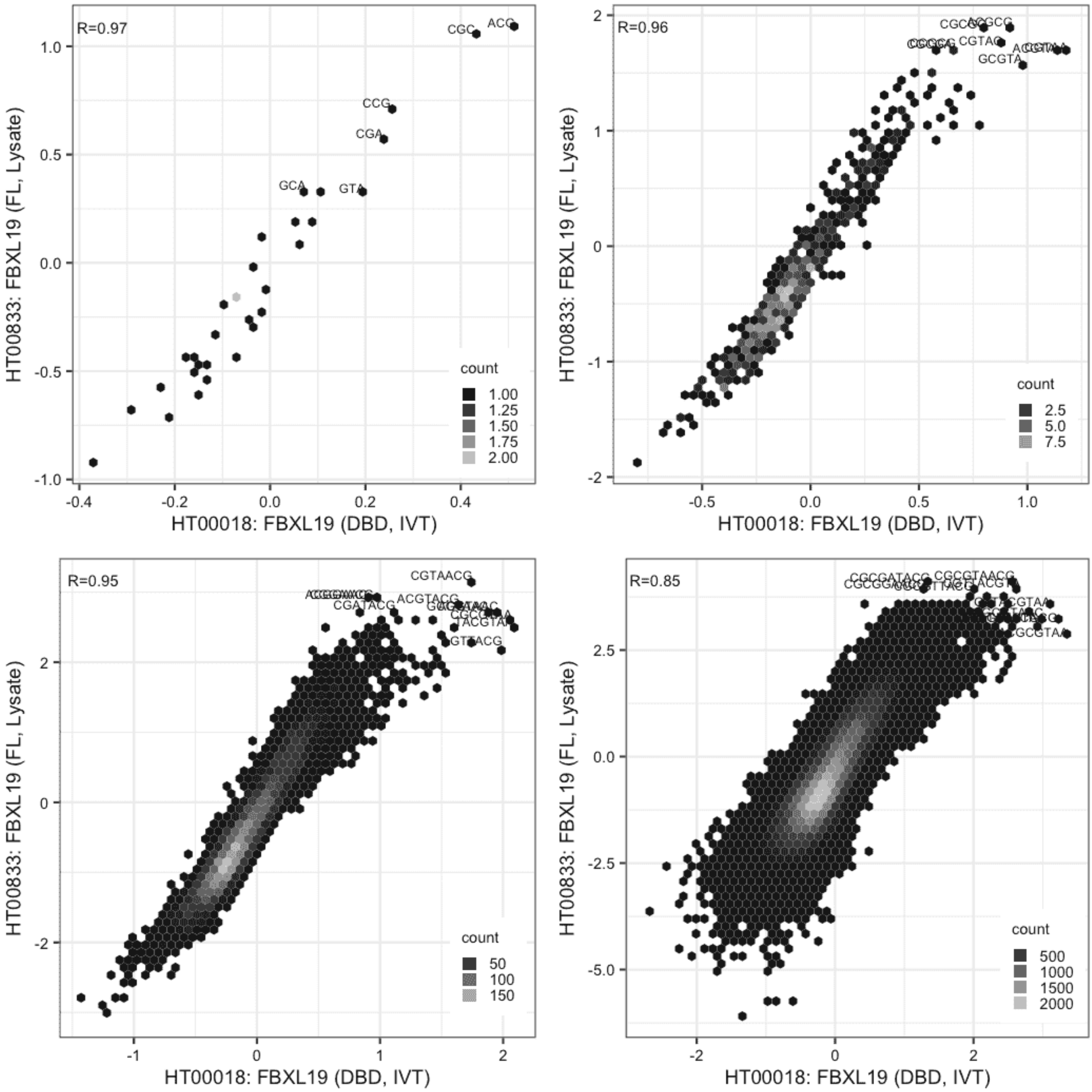

FBXL19

IVT vs GFPIVT, same construct

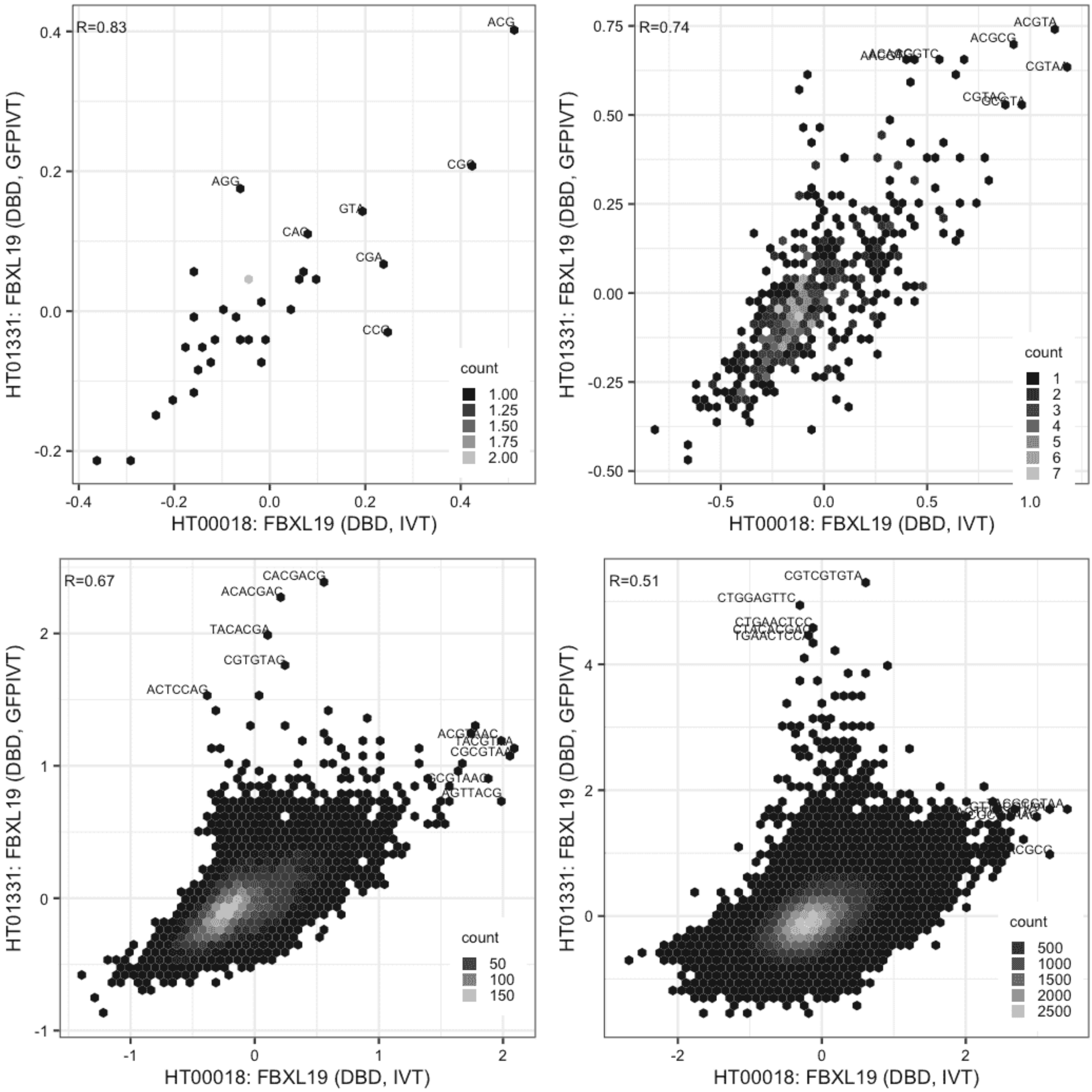

FBXL19

Lysate vs GFPIVT, different constructs

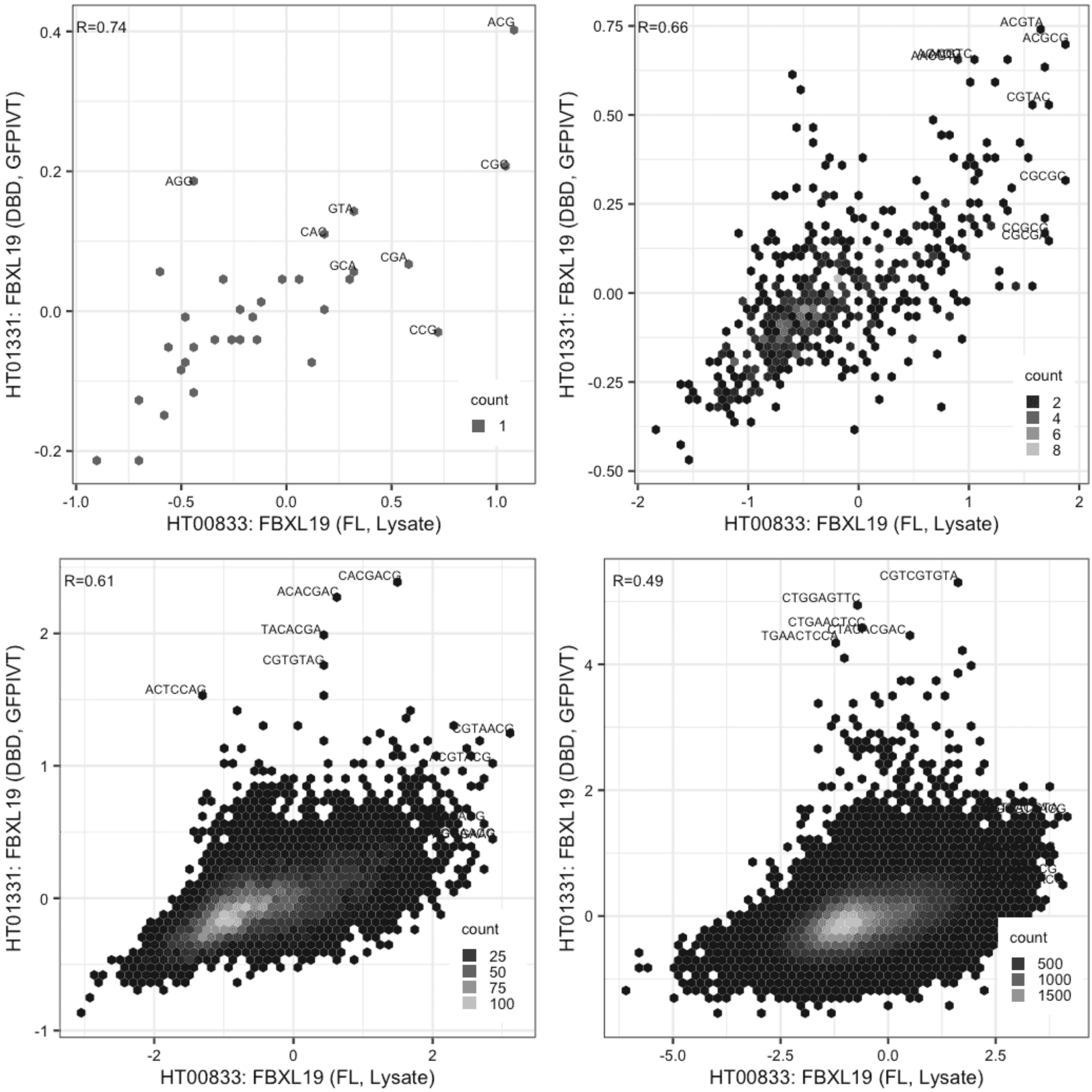

FLI1

IVT vs IVT, same construct

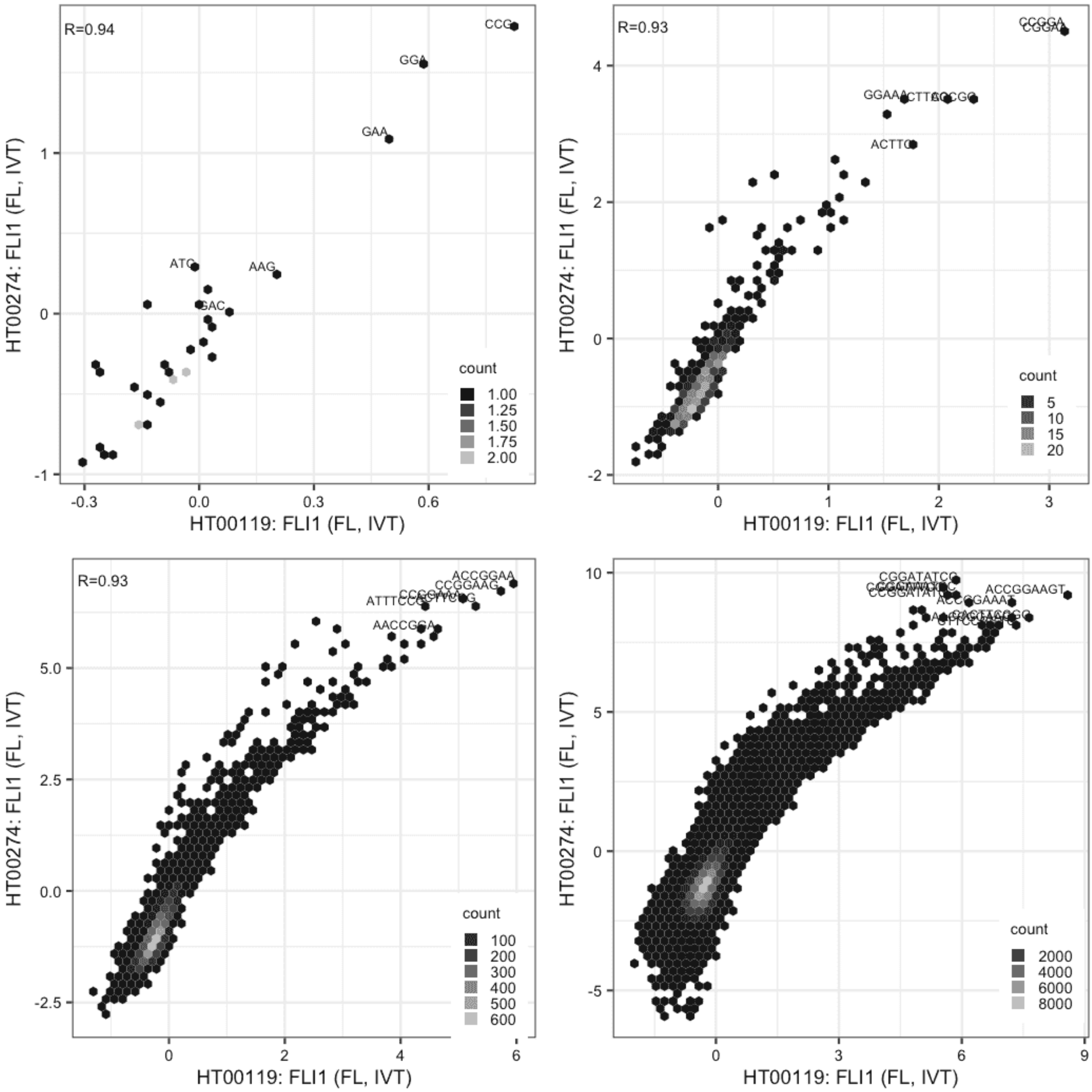

FLI1

Lysate vs IVT, same construct

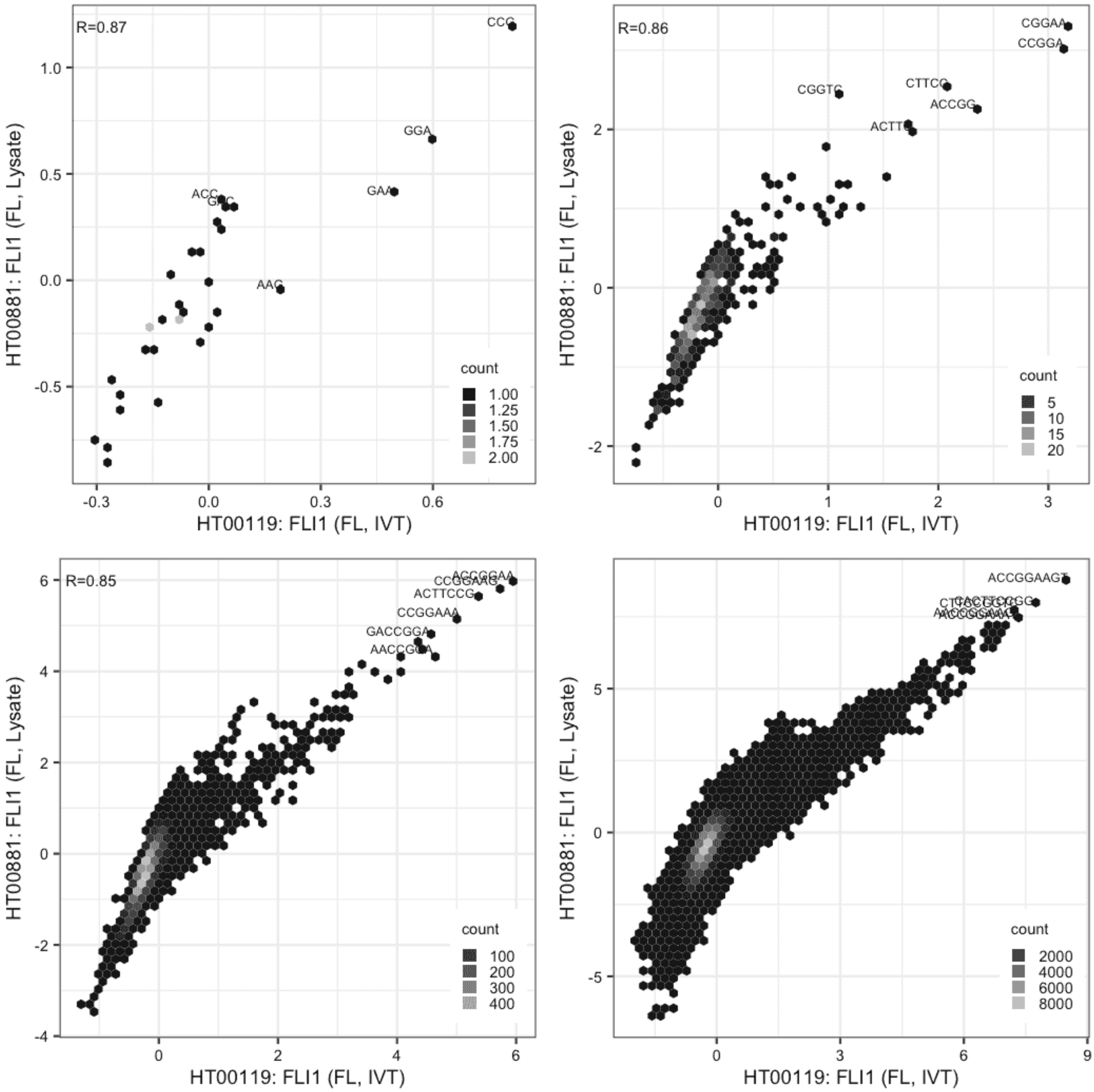

FLI1

Lysate vs IVT, same construct

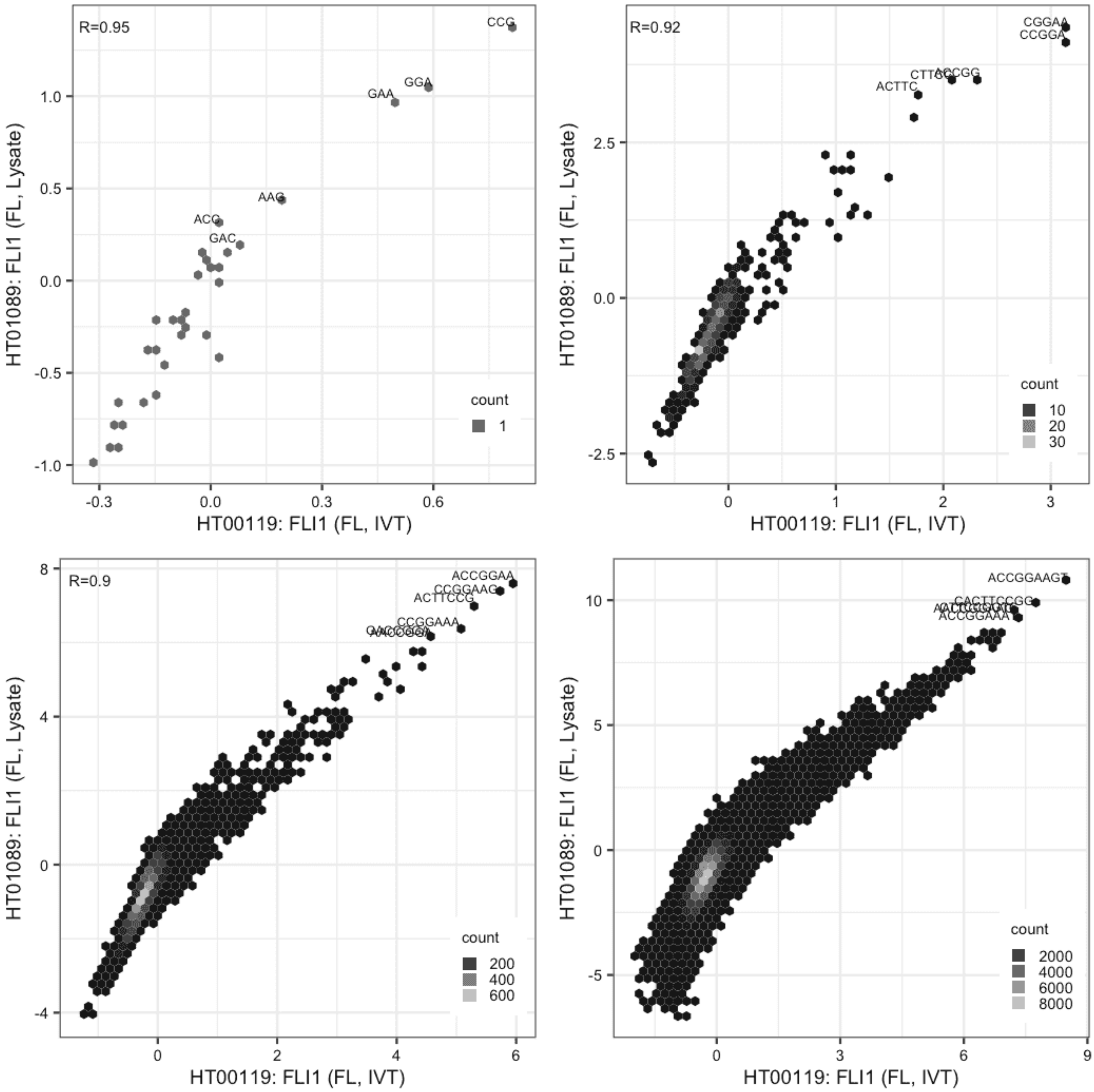

FLI1

Lysate vs IVT, same construct

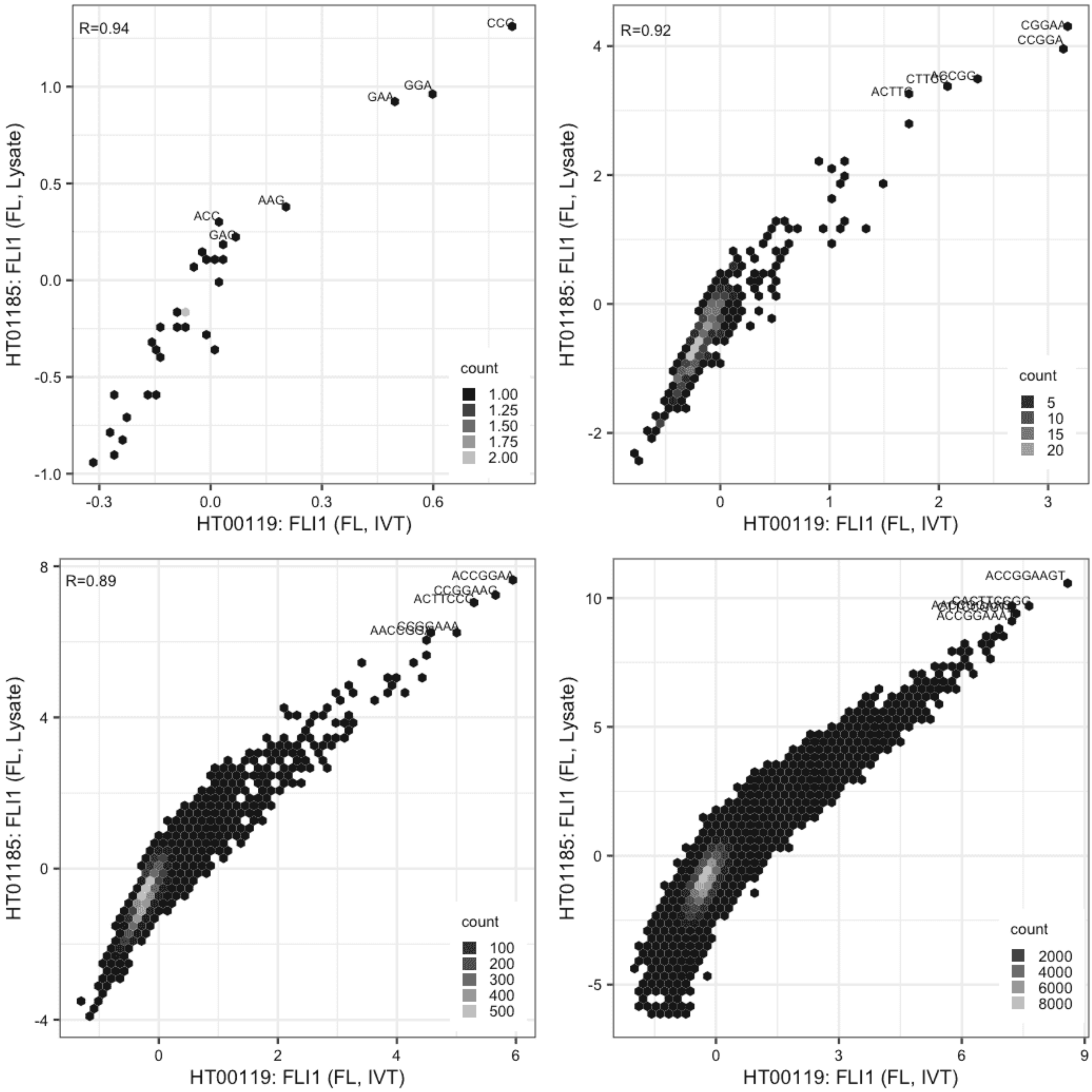

FLI1

Lysate vs IVT, same construct

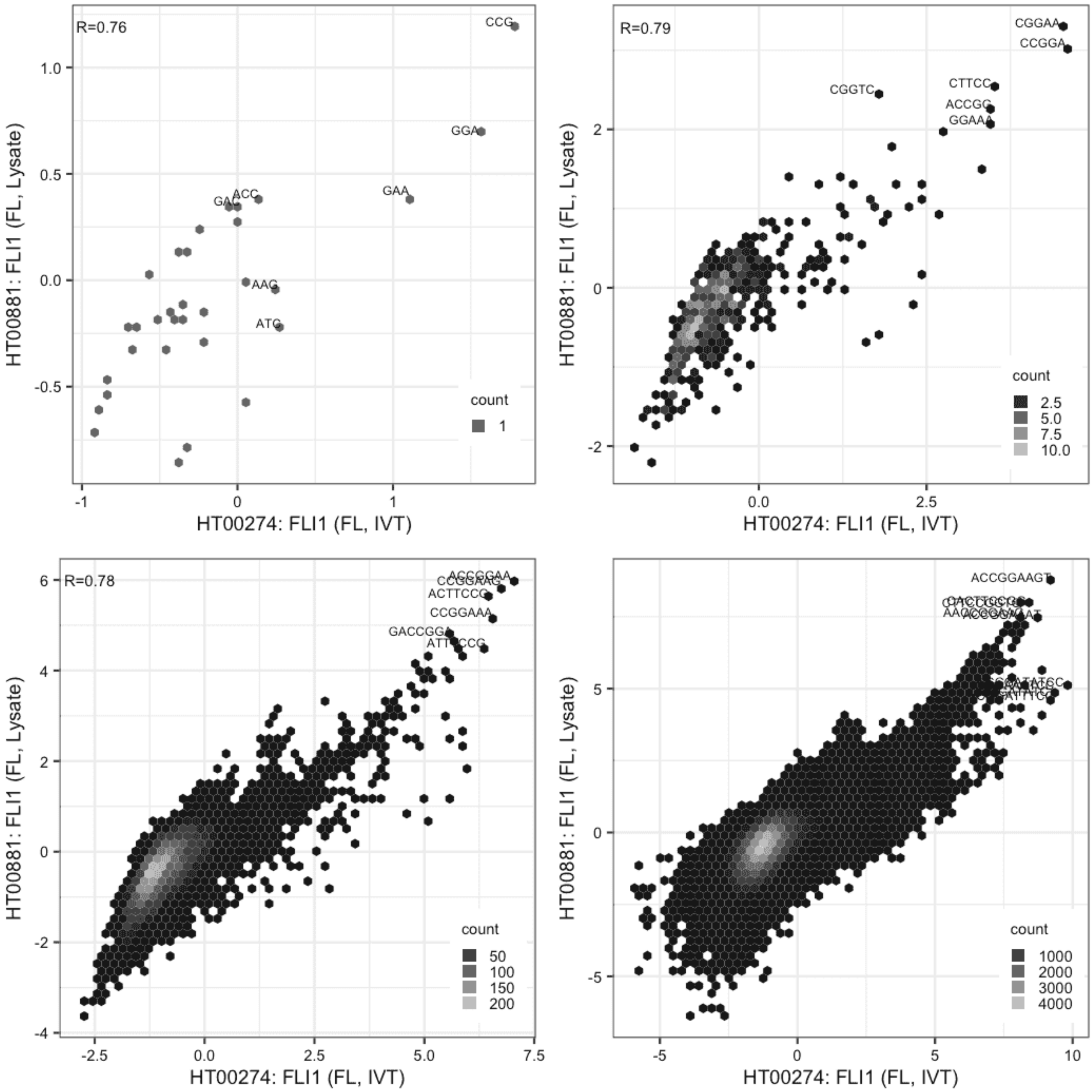

FLI1

Lysate vs IVT, same construct

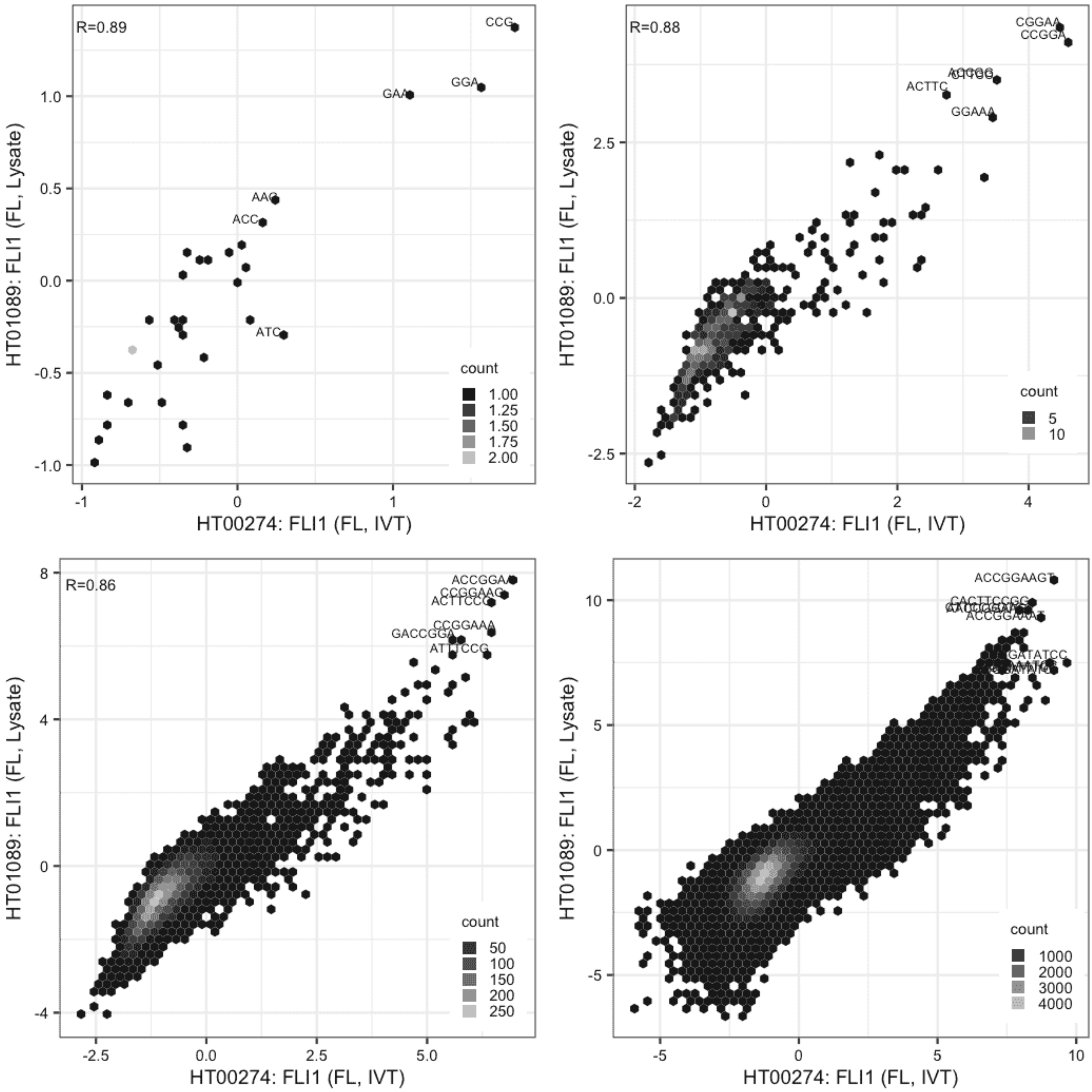

FLI1

Lysate vs IVT, same construct

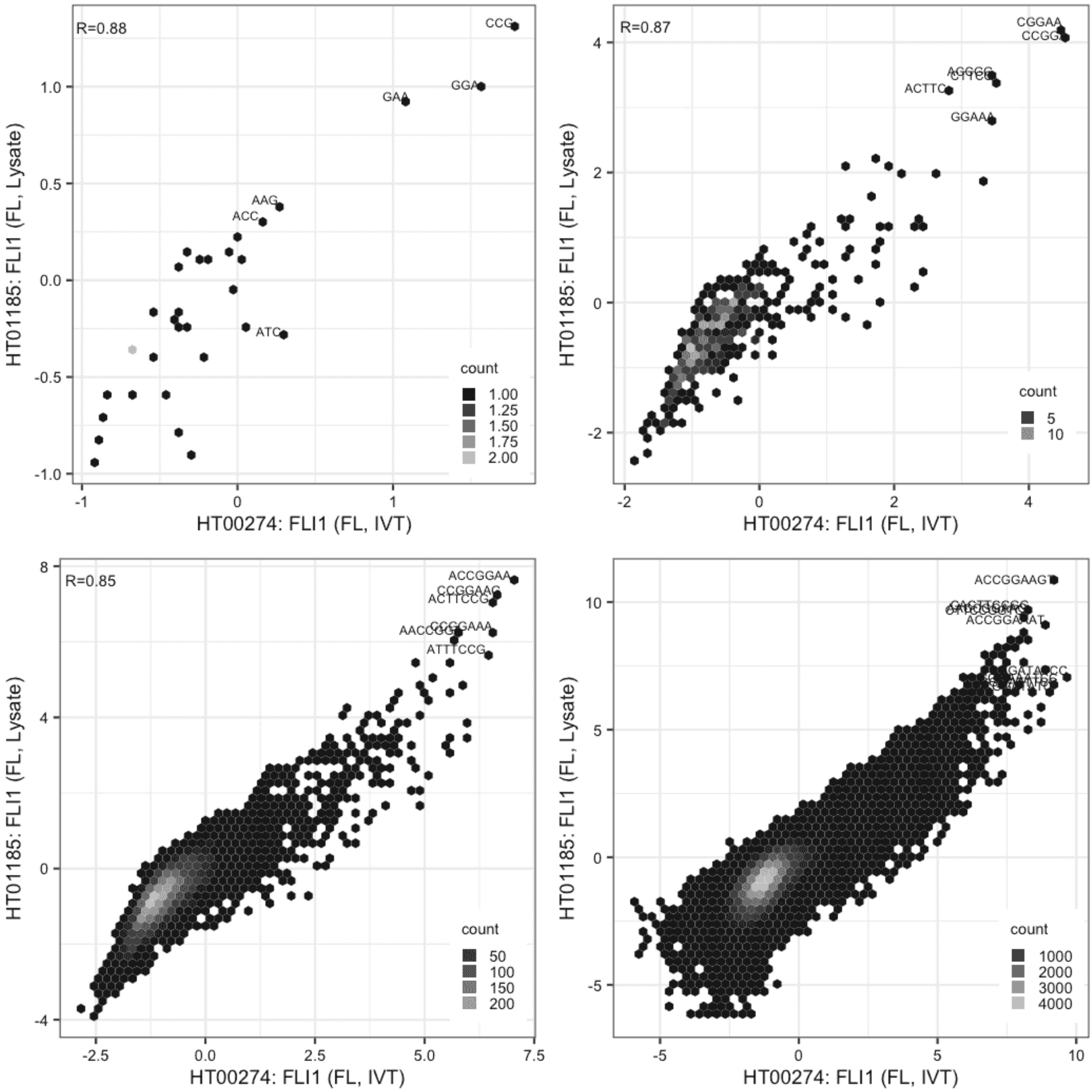

FLI1

Lysate vs Lysate, same construct

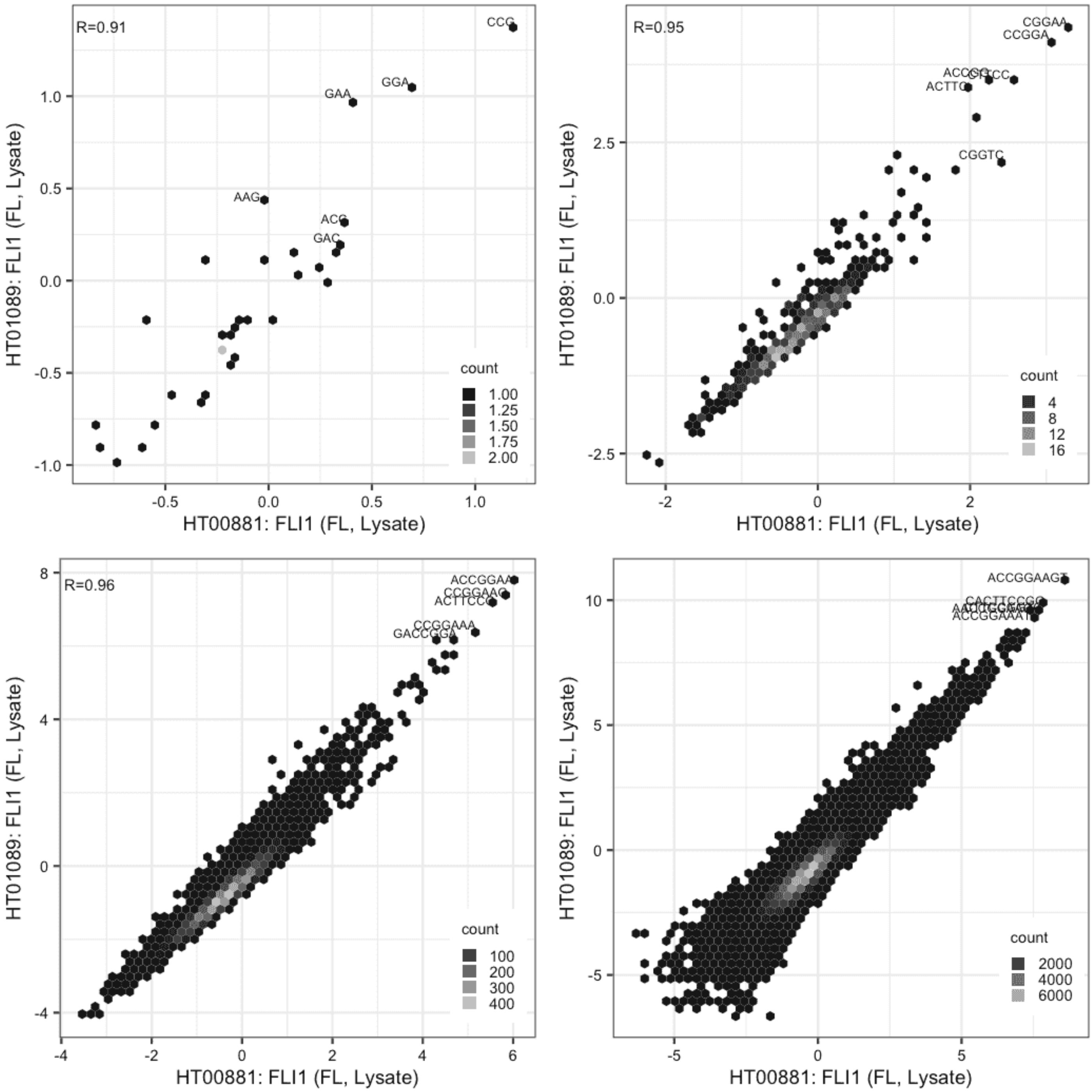

FLI1

Lysate vs Lysate, same construct

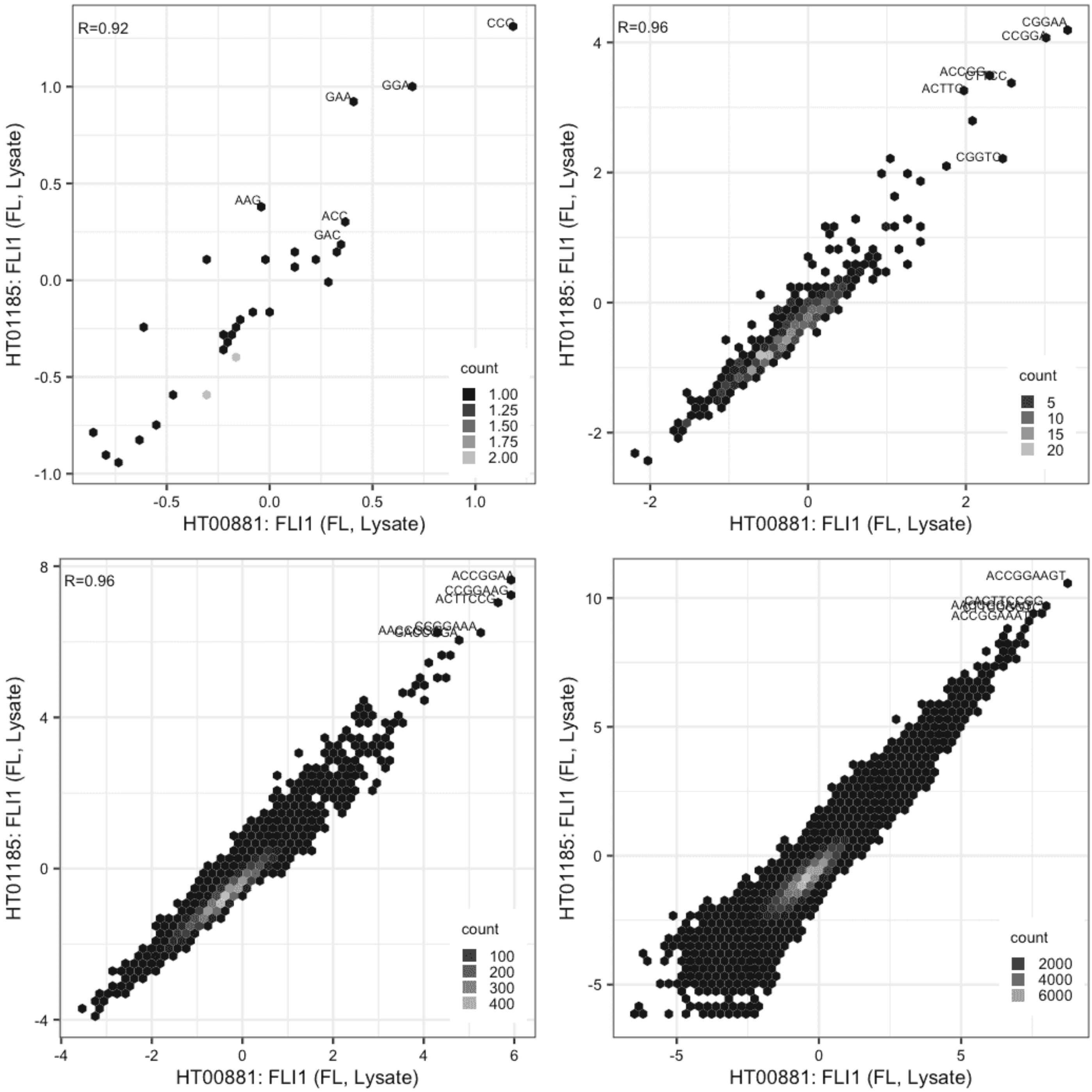

FLI1

Lysate vs Lysate, same construct

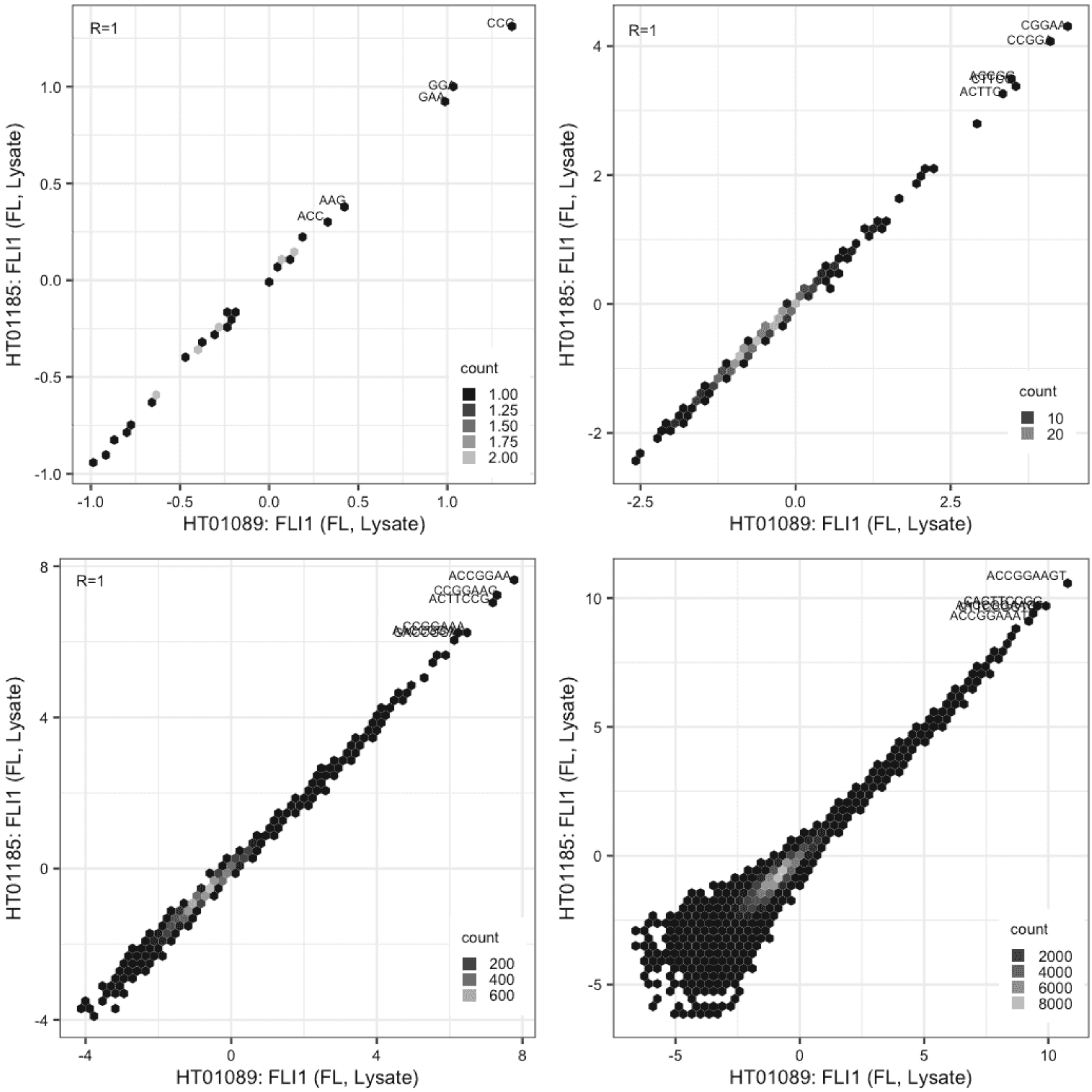

FOSL2

Lysate vs IVT, same construct

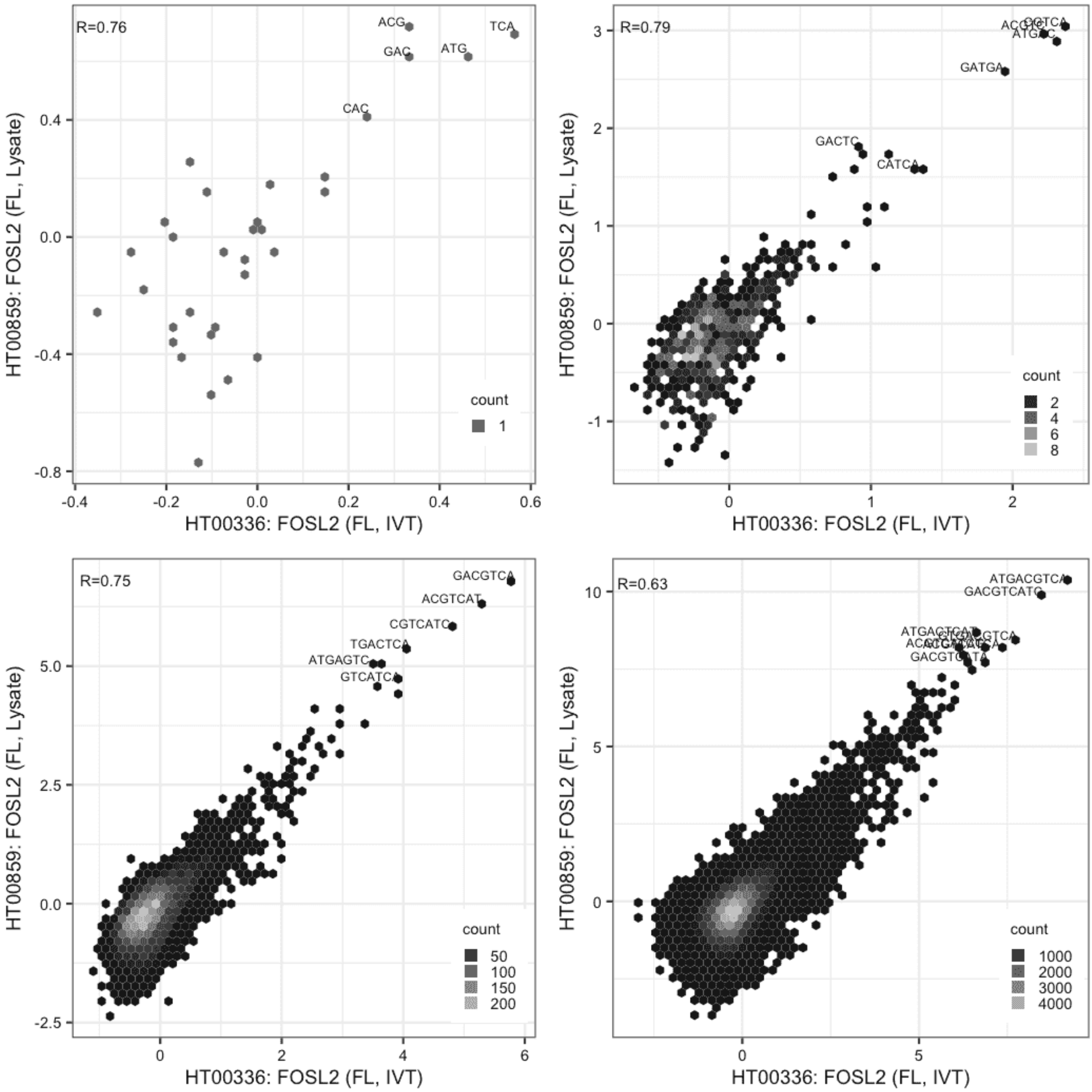

GABPA

Lysate vs IVT, same construct

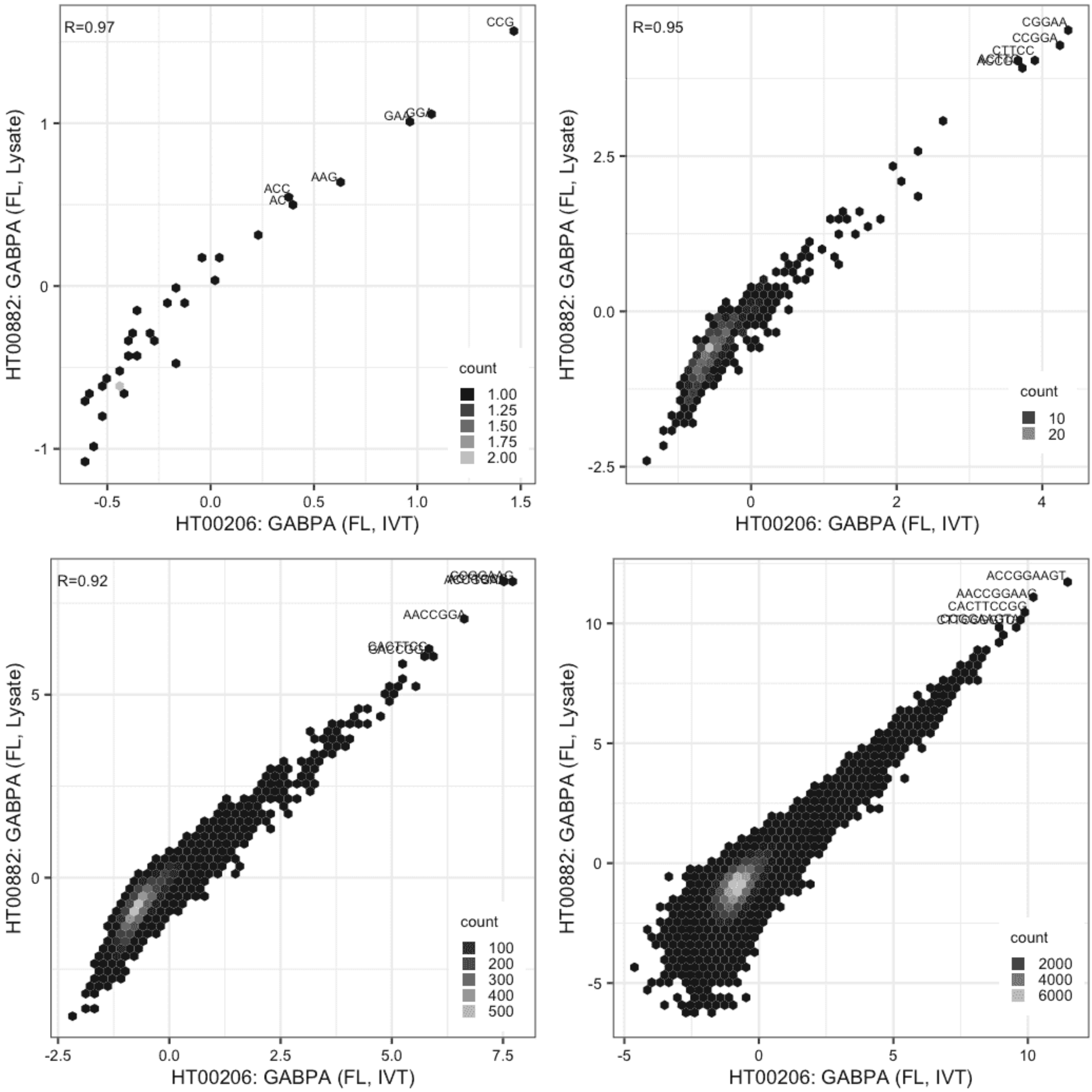

GCM1

Lysate vs IVT, same construct

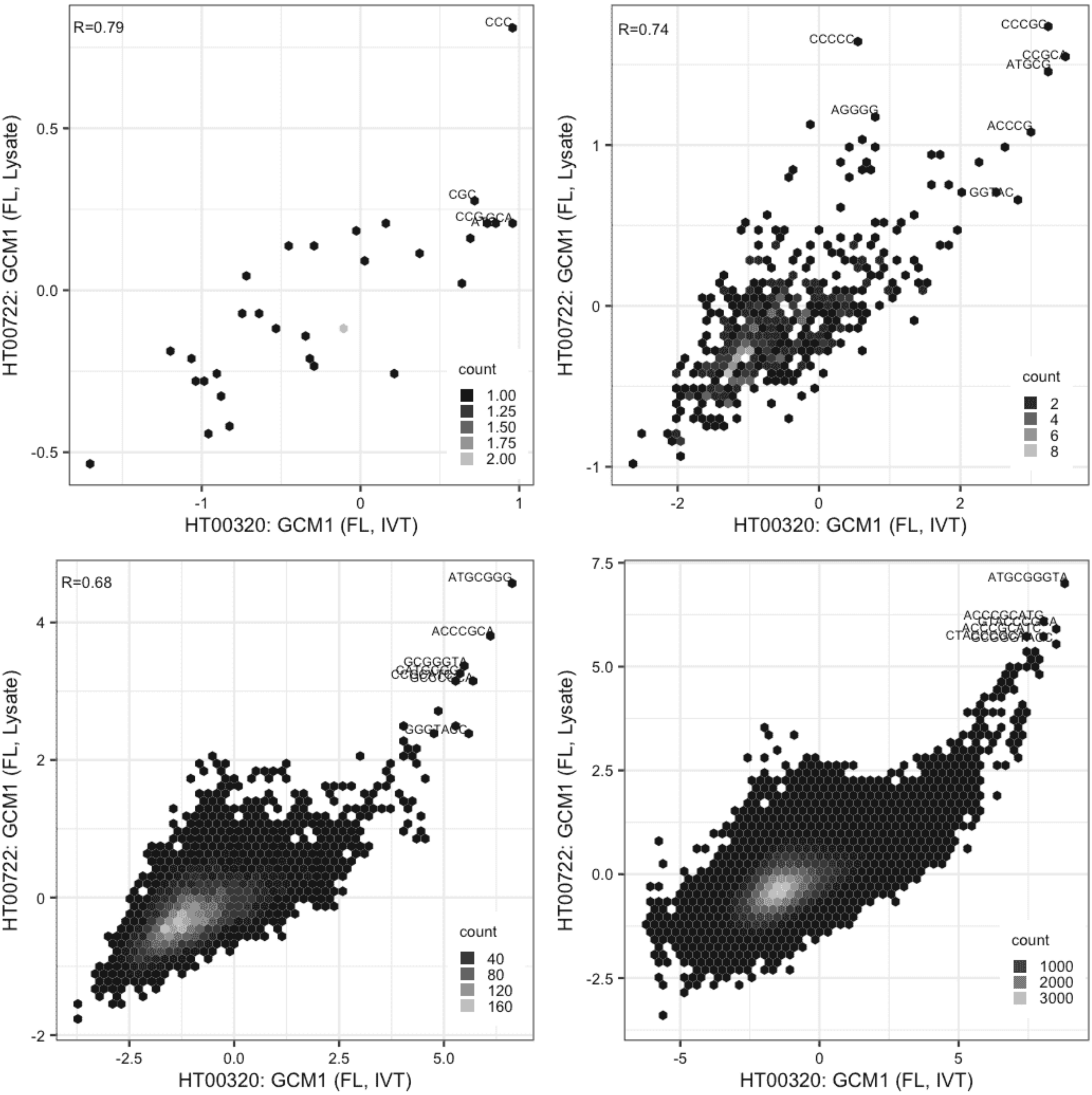

GCM1

Lysate vs IVT, same construct

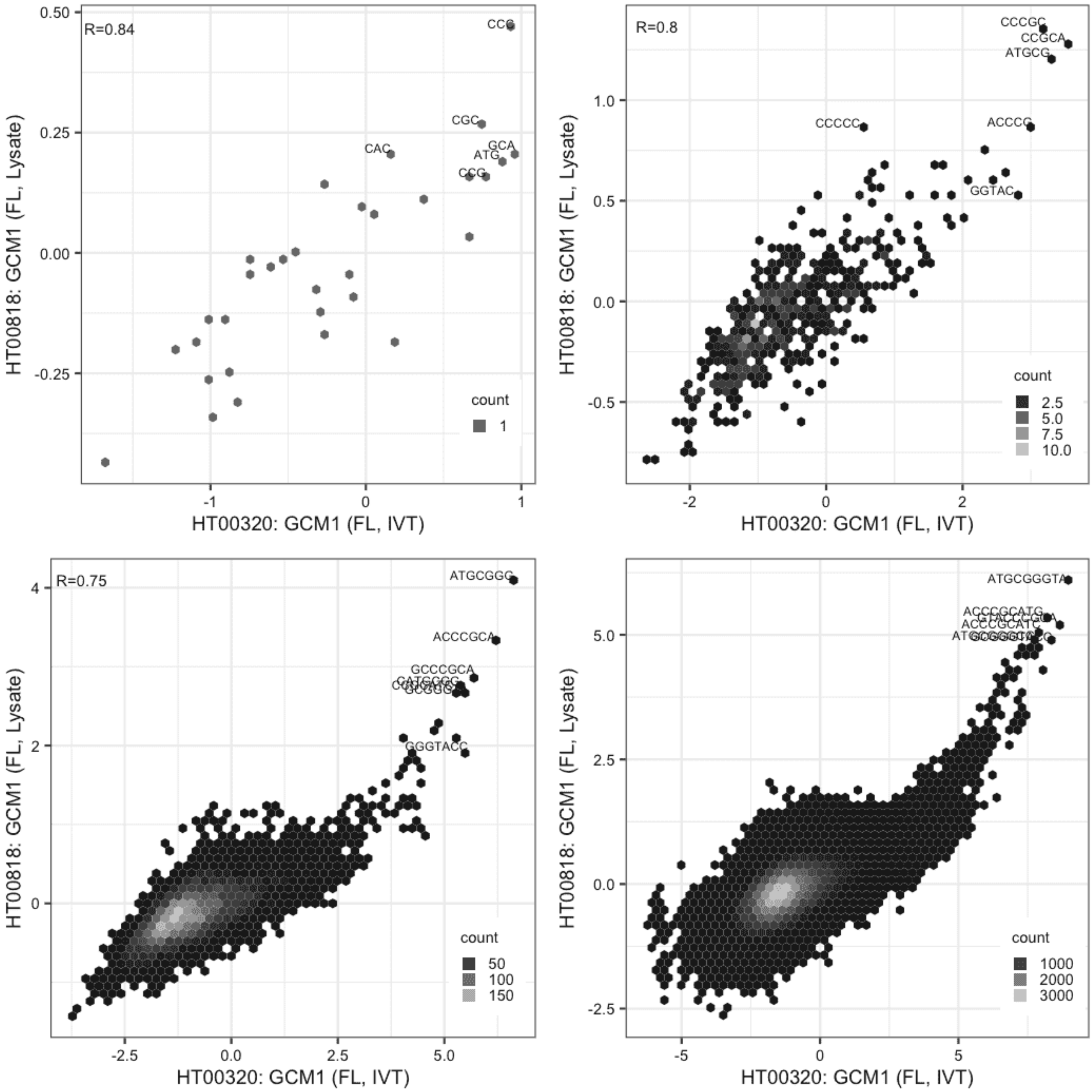

GCM1

Lysate vs Lysate, same construct

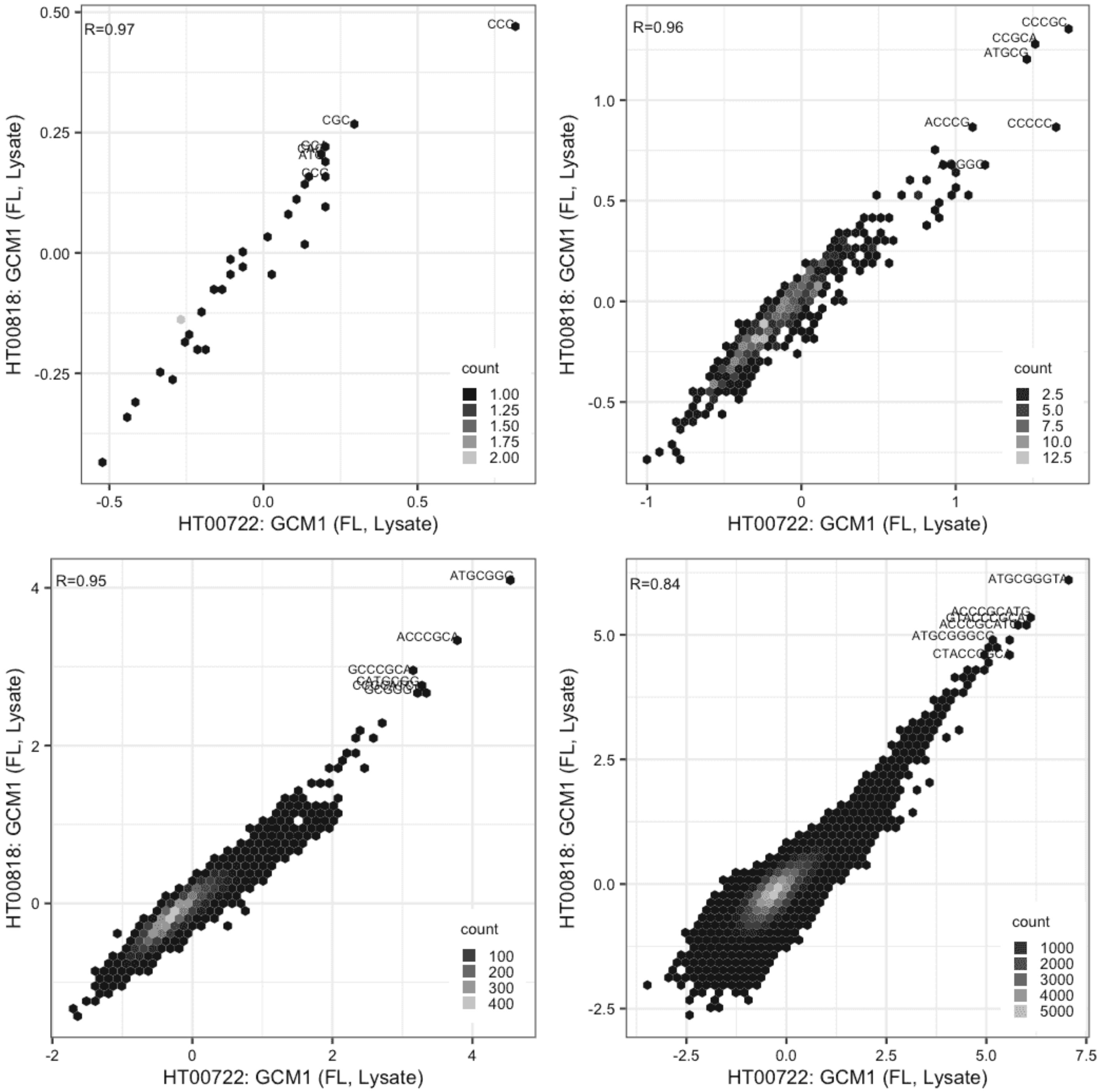

JRK

Lysate vs IVT, different constructs

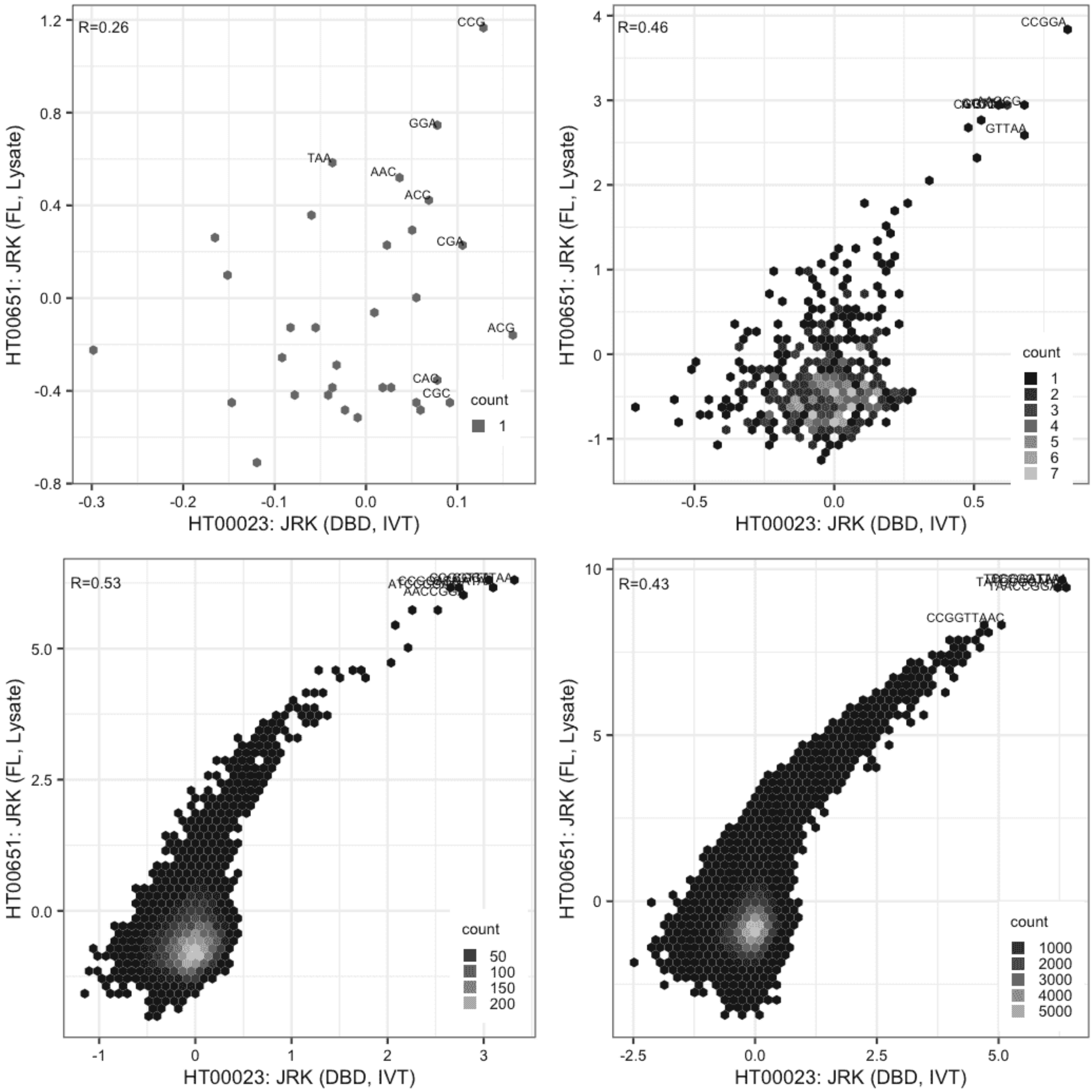

JRK

Lysate vs IVT, different constructs

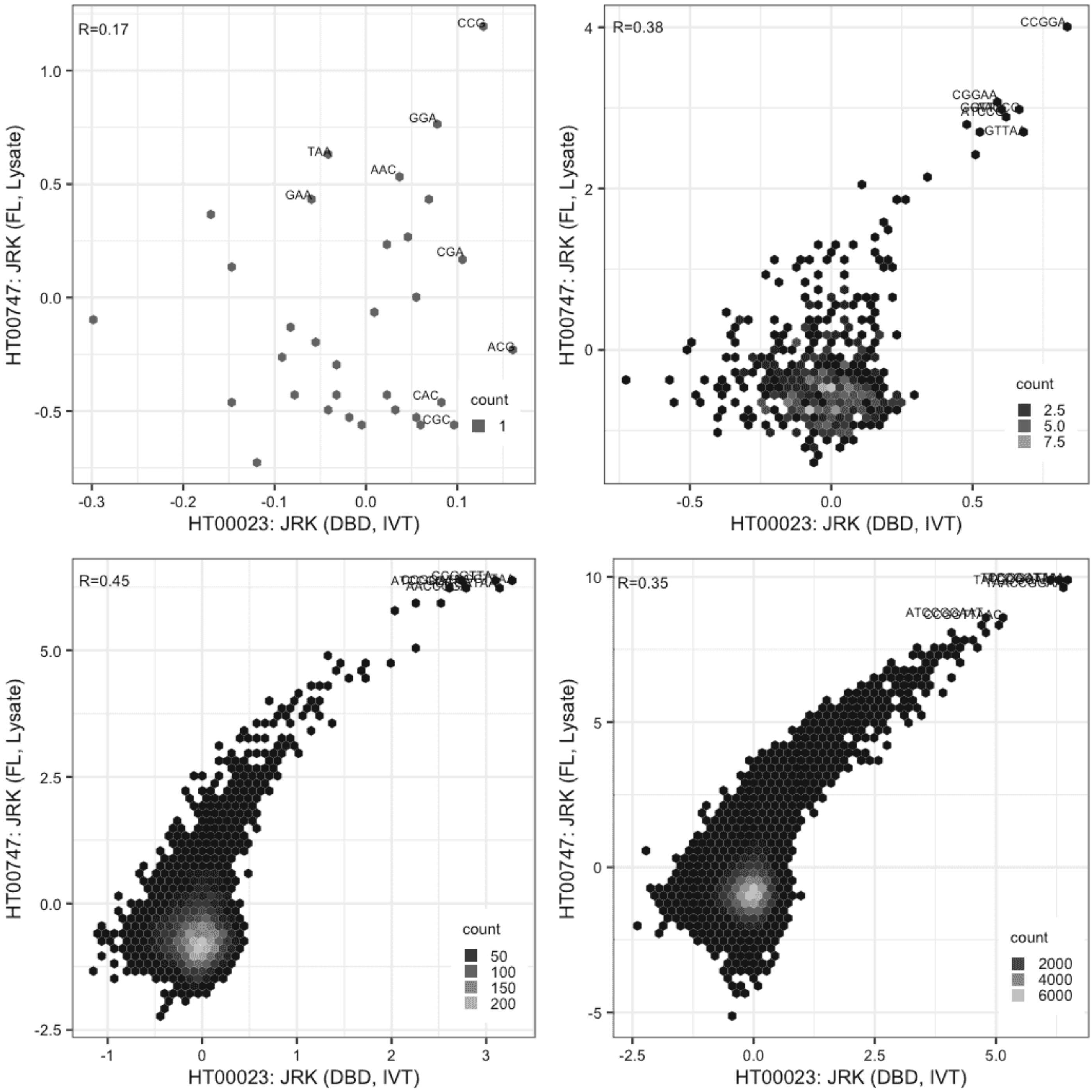

JRK

IVT vs GFPIVT, same construct

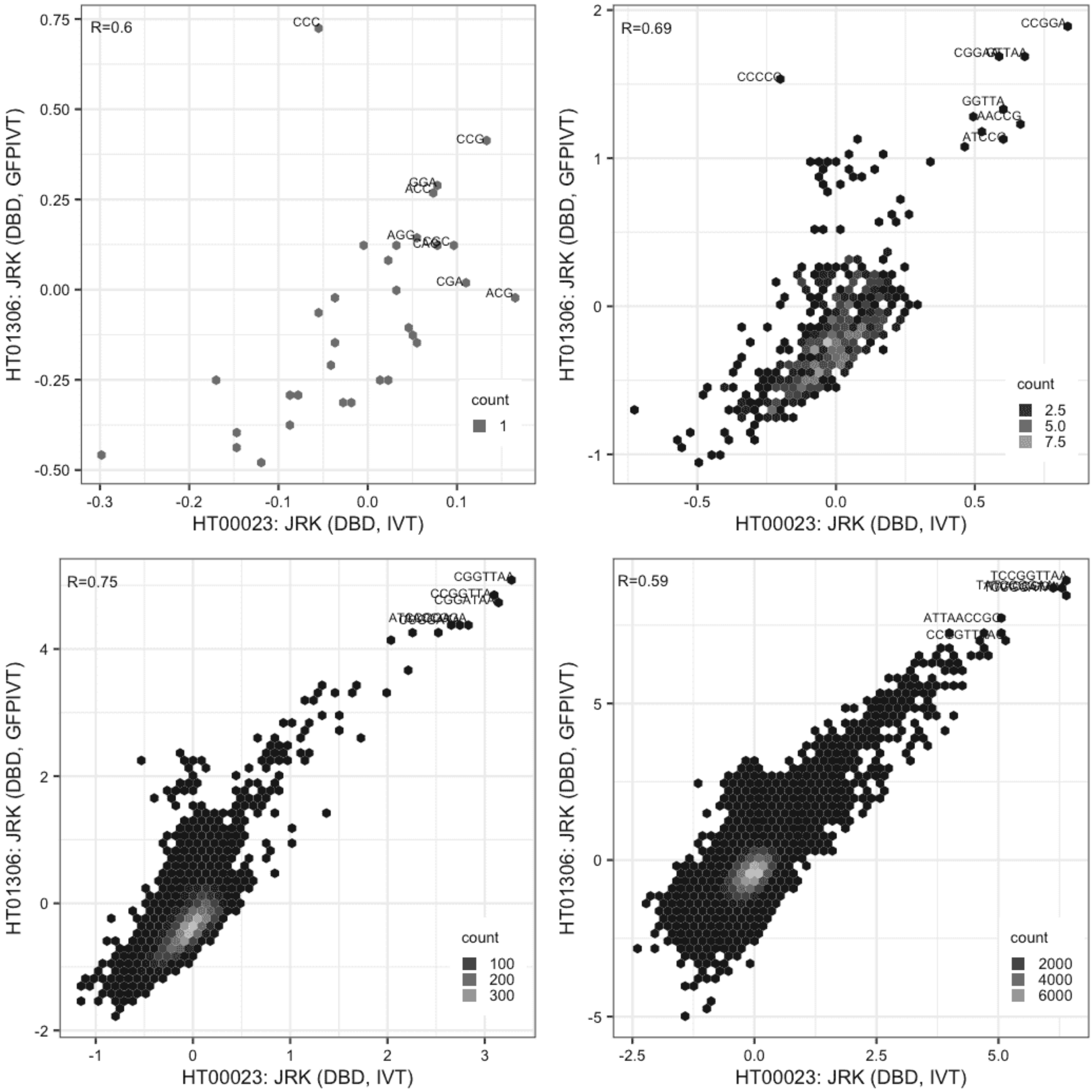

JRK

IVT vs GFPIVT, different constructs

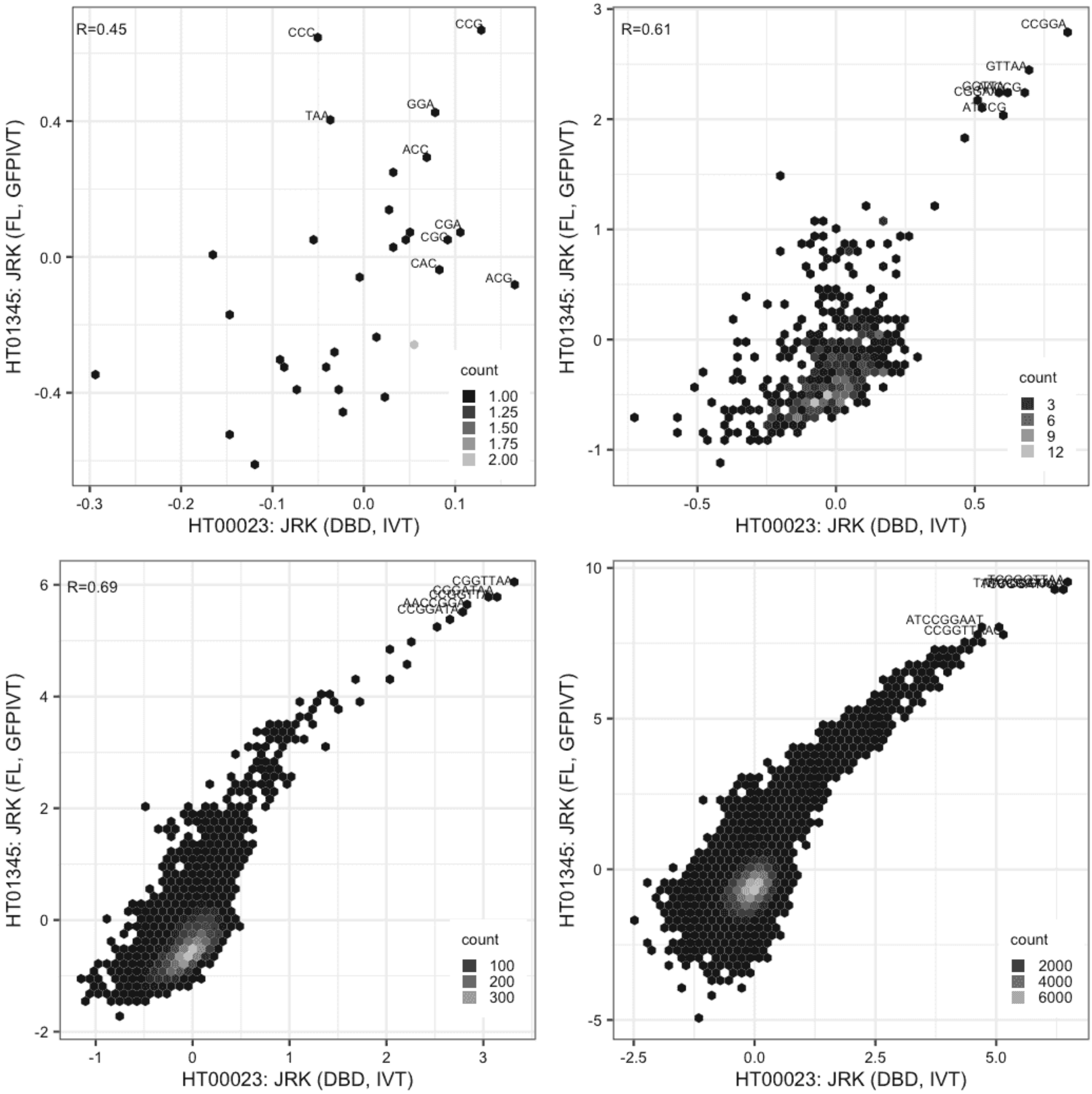

JRK

Lysate vs Lysate, same construct

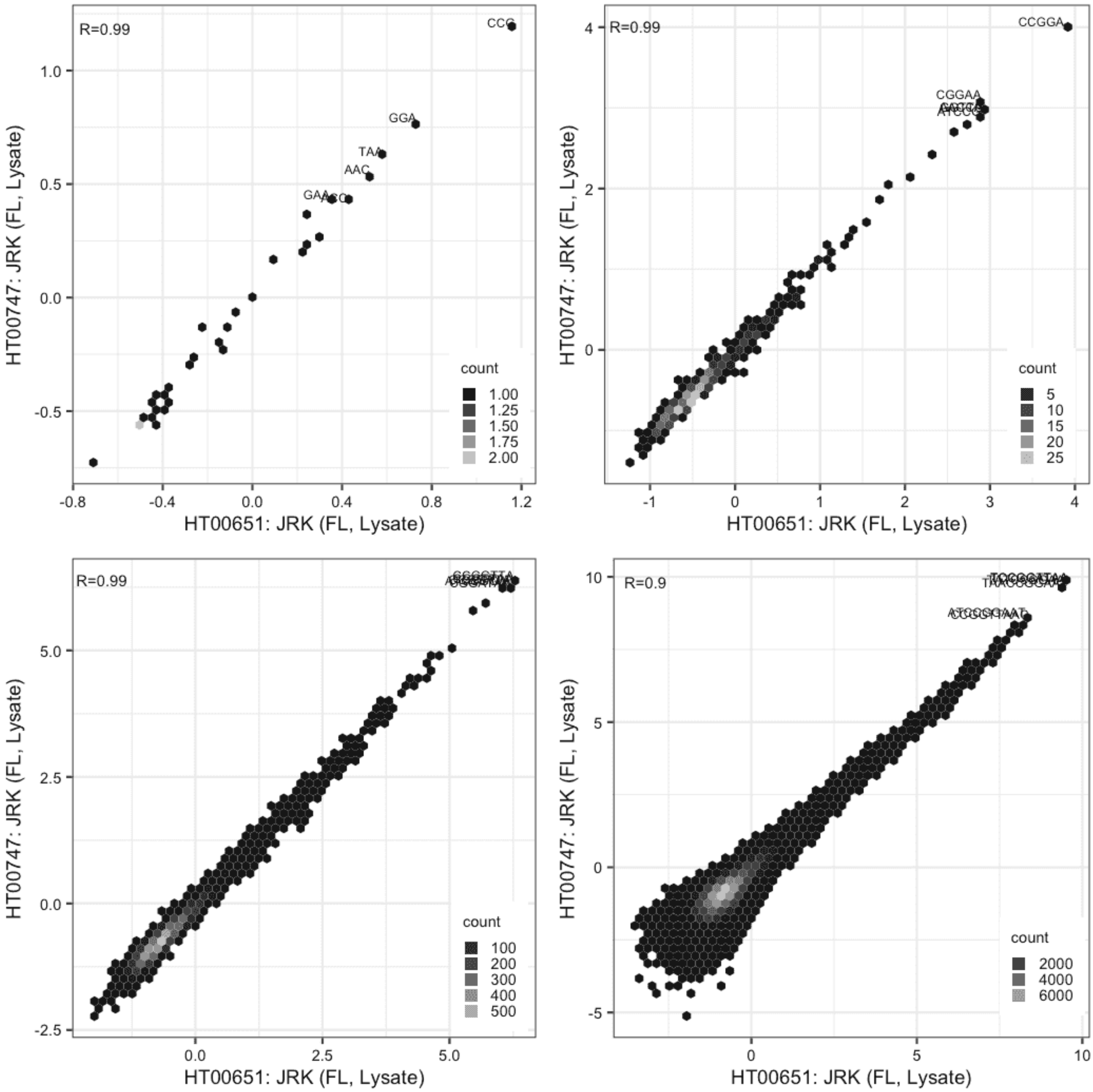

JRK

Lysate vs GFPIVT, different constructs

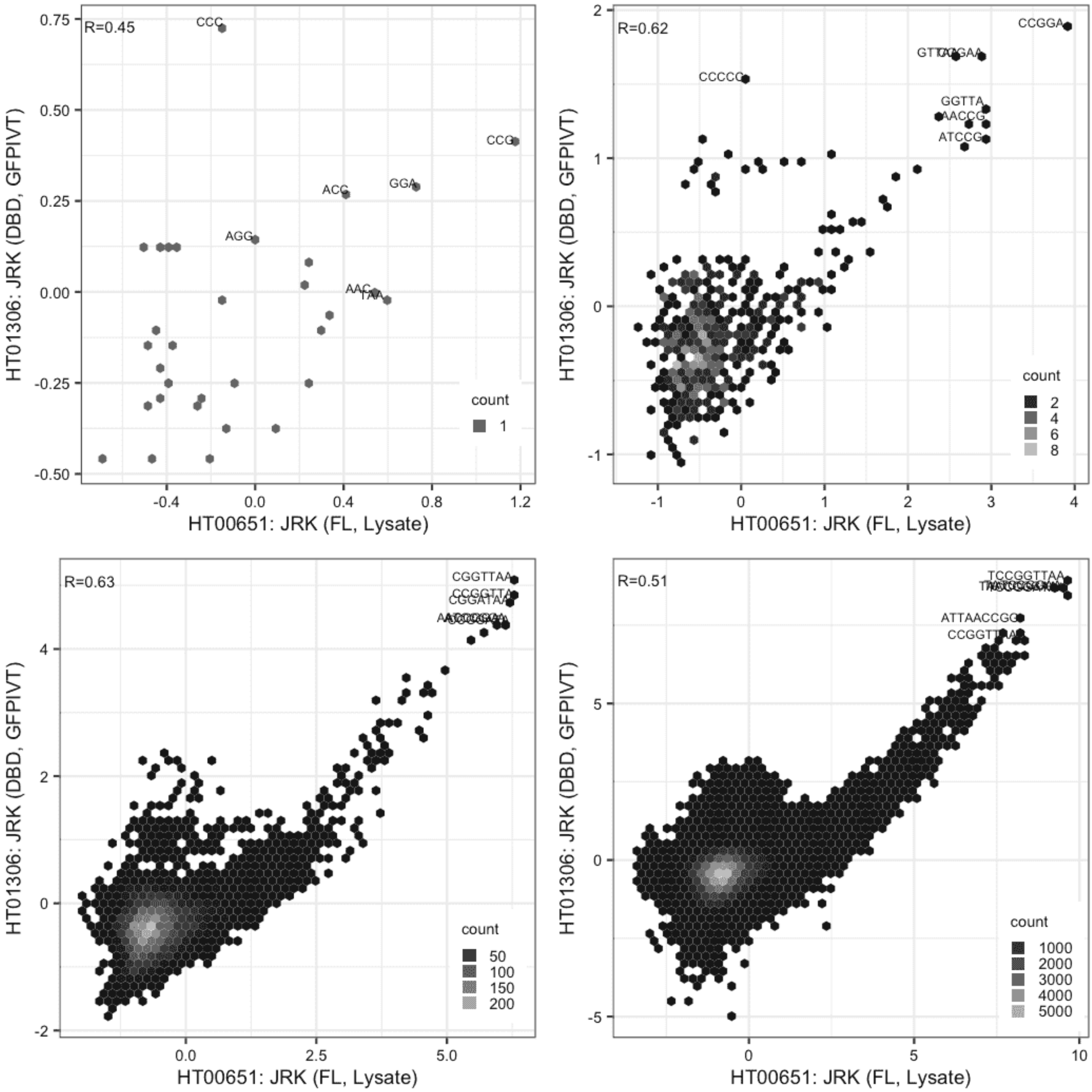

JRK

Lysate vs GFPIVT, same construct

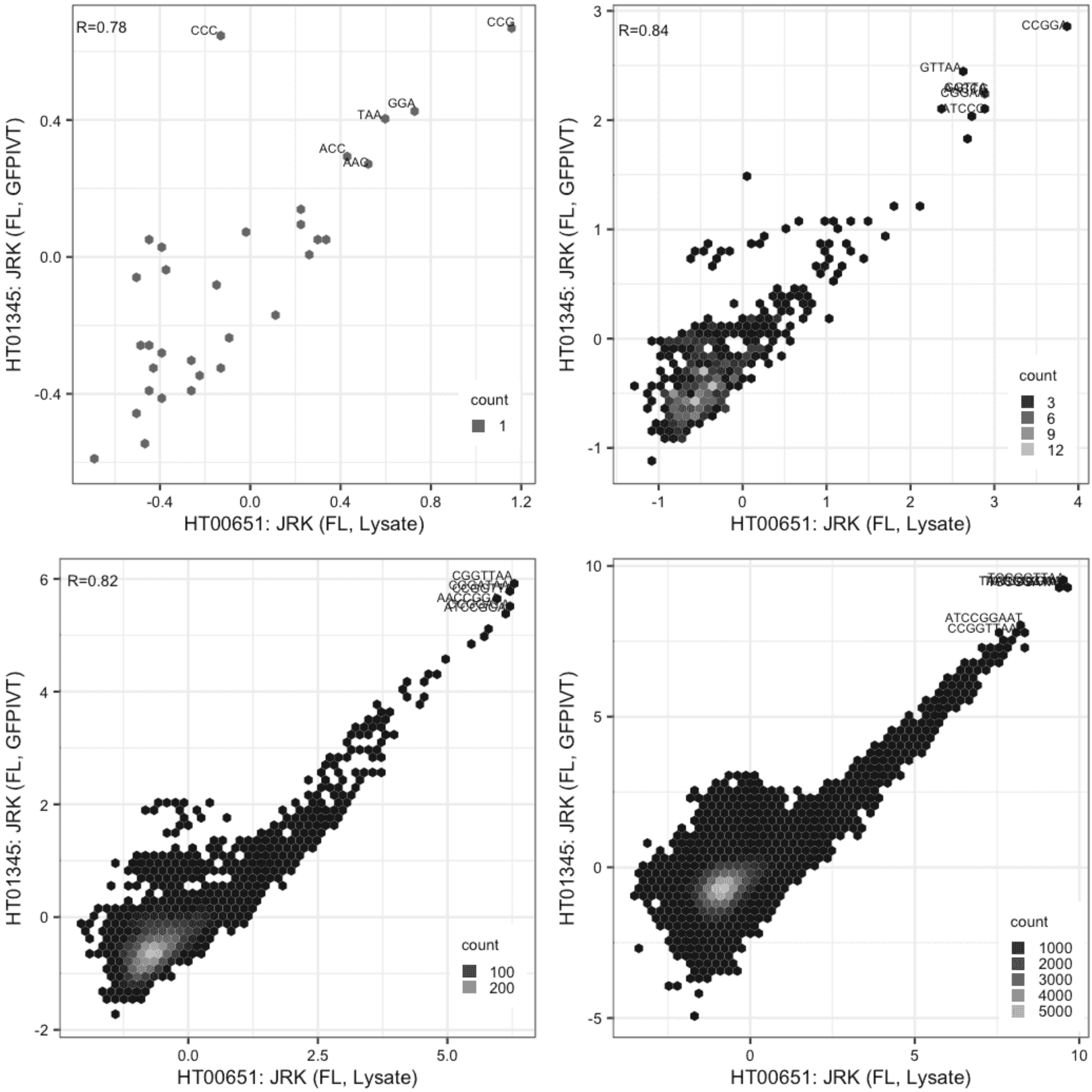

JRK

Lysate vs GFPIVT, different constructs

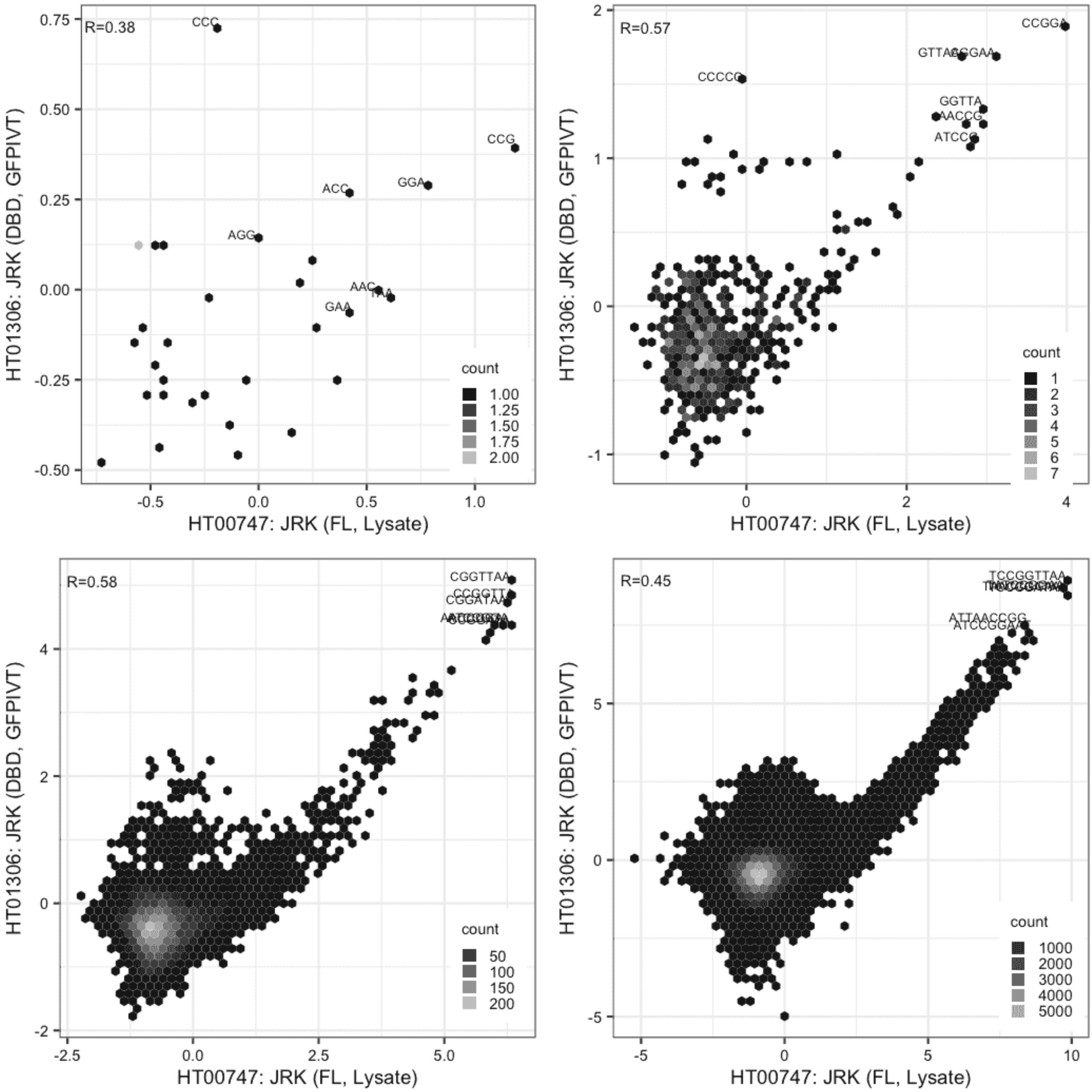

JRK

Lysate vs GFPIVT, same construct

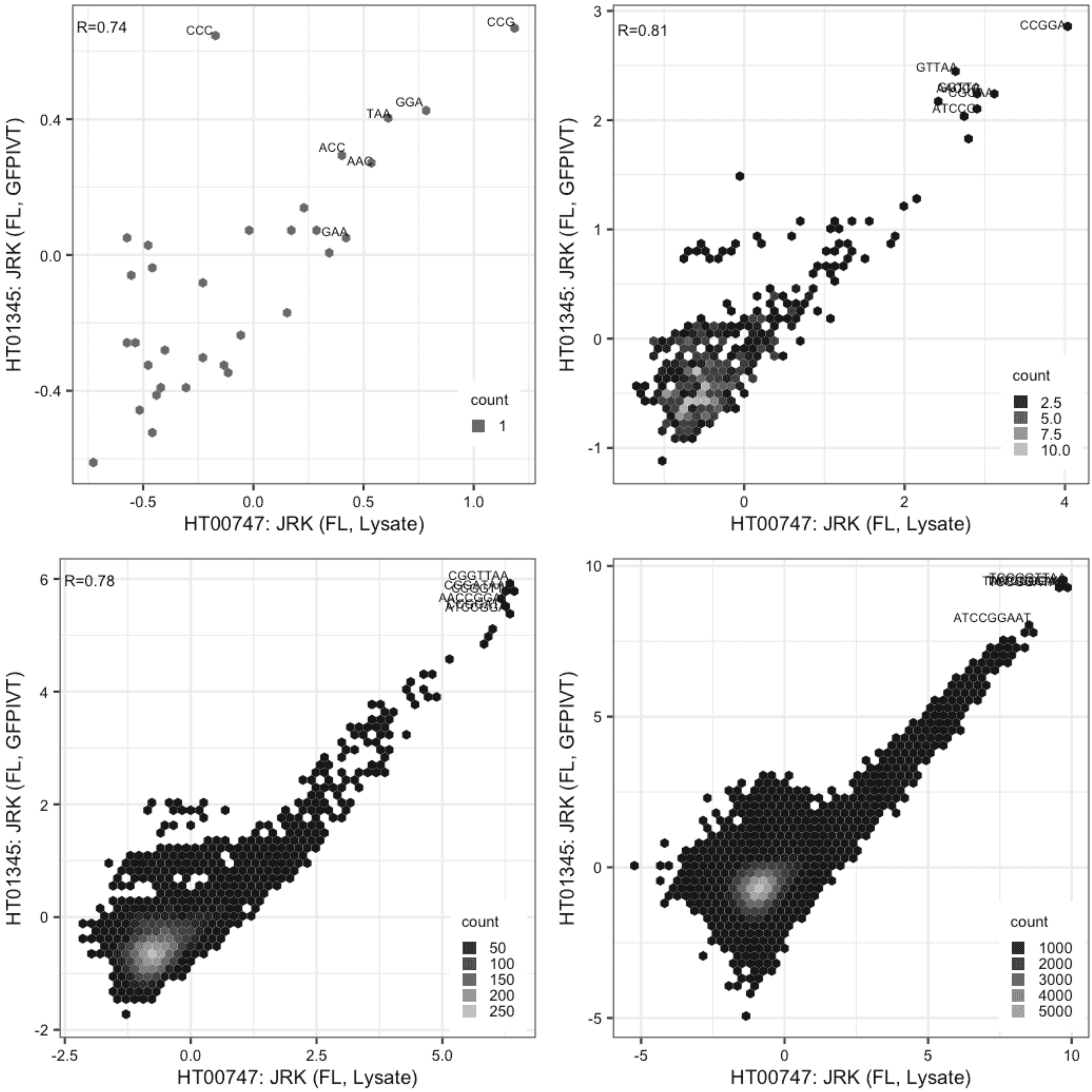

JRK

GFPIVT vs GFPIVT, different constructs

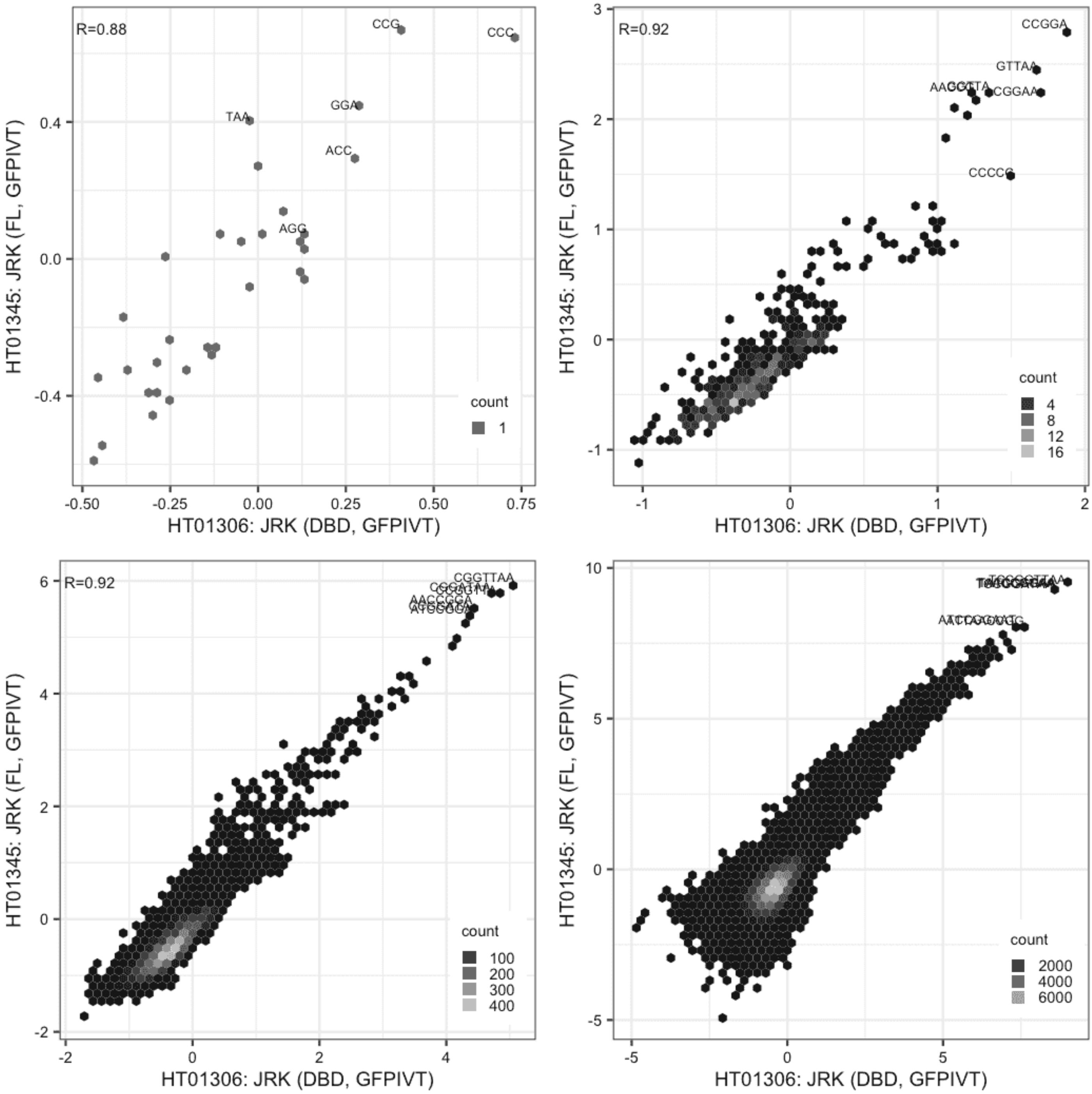

KDM2A

Lysate vs GFPIVT, different constructs

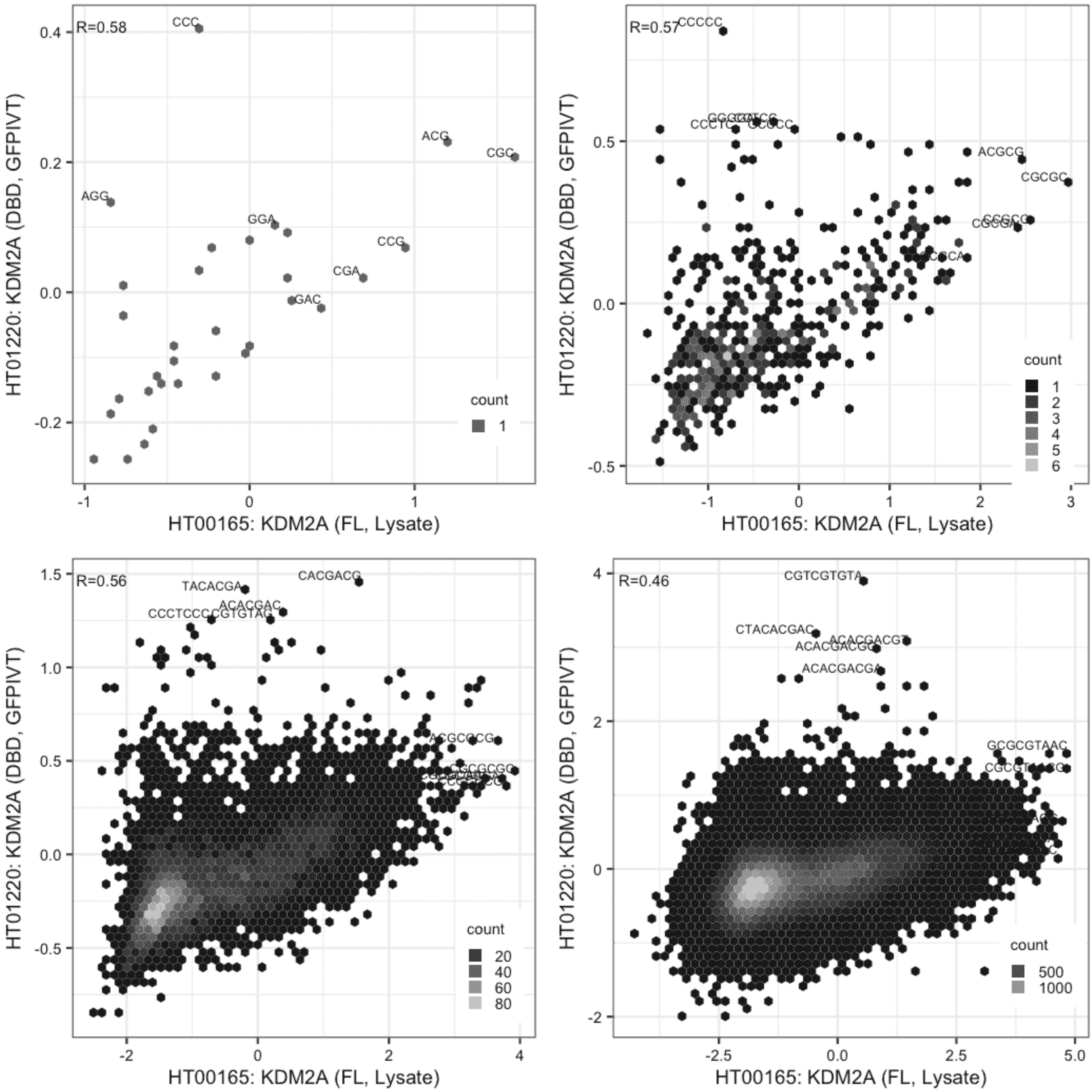

LEF1

Lysate vs IVT, same construct

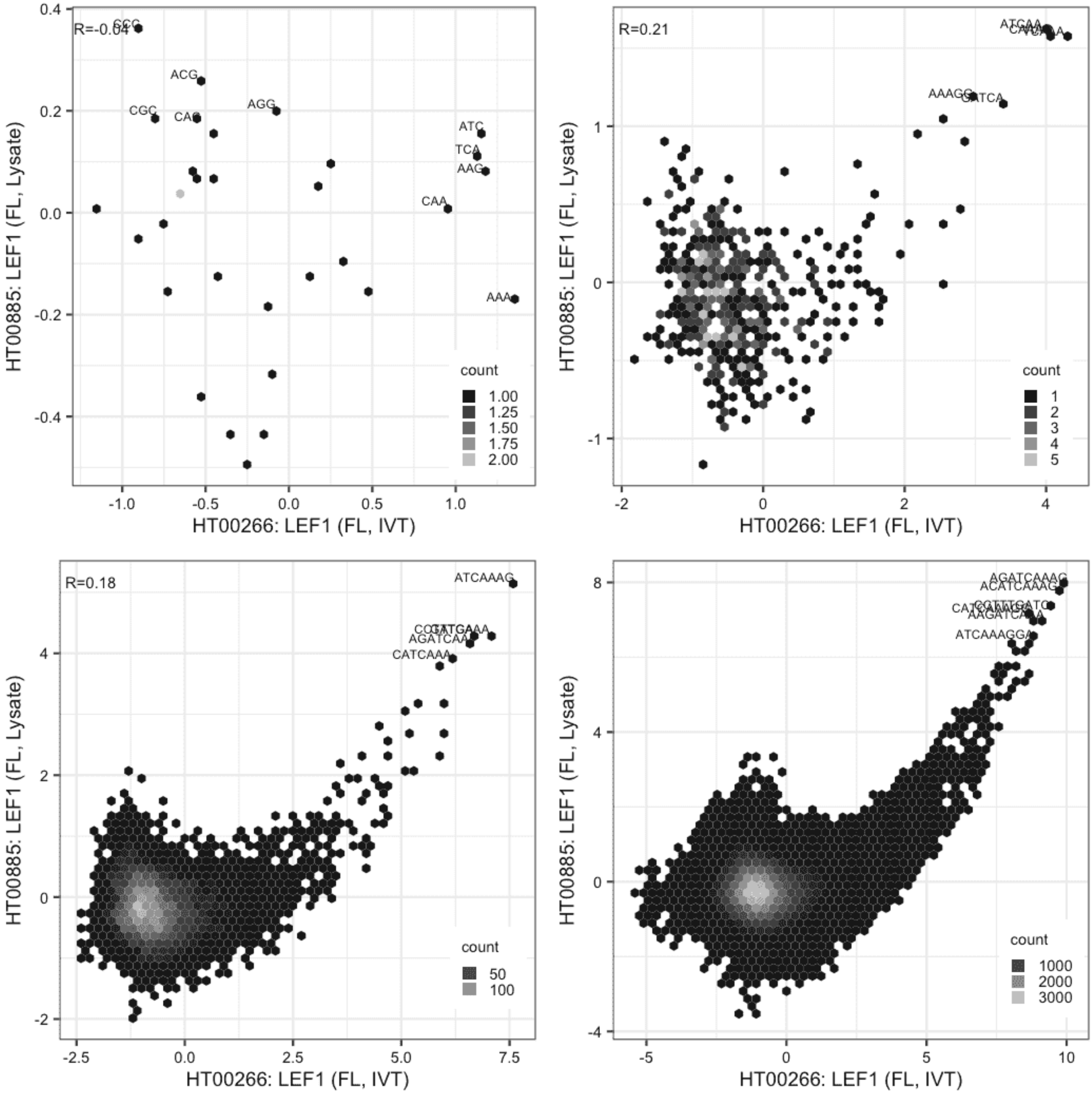

LEUTX

Lysate vs Lysate, same construct

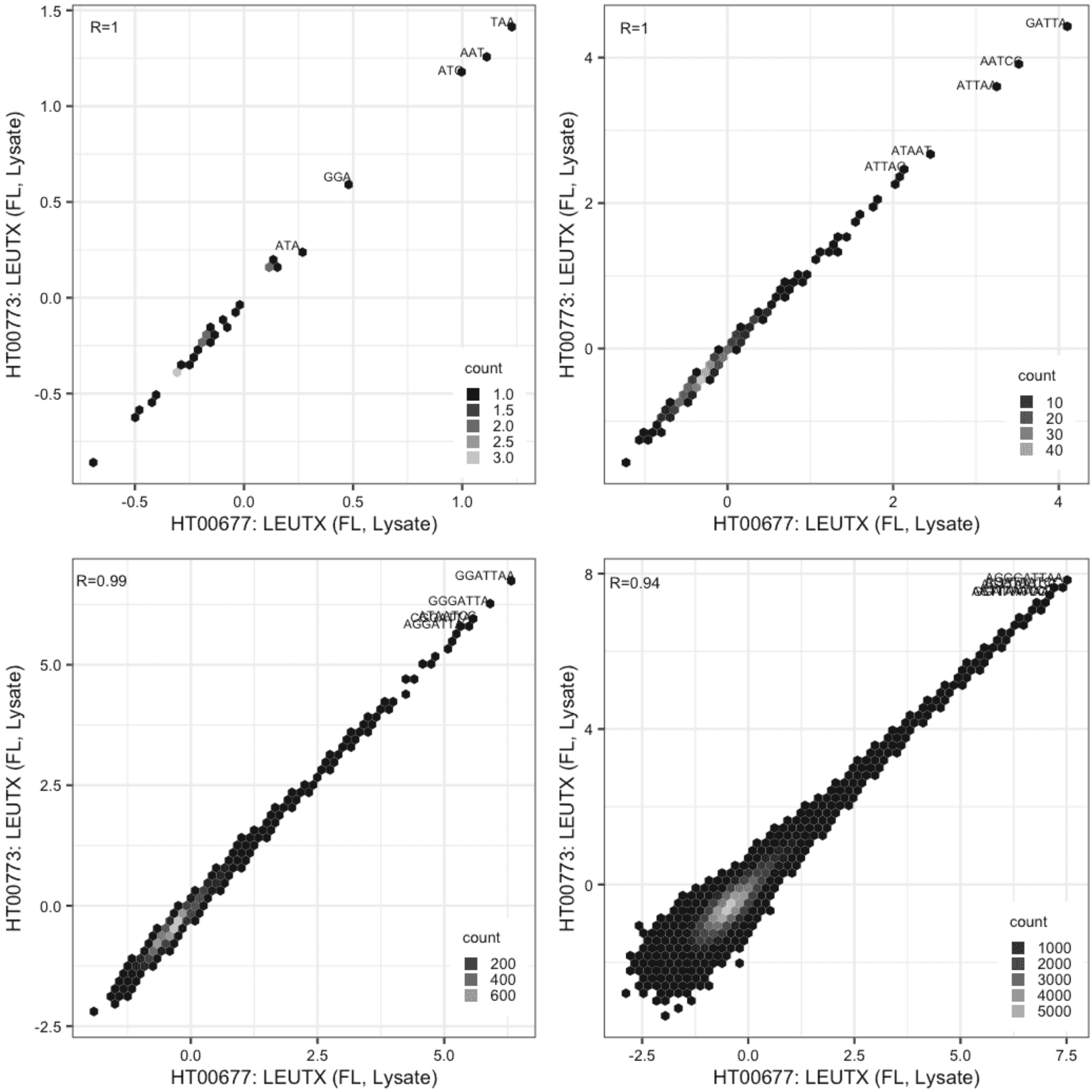

LEUTX

Lysate vs GFPIVT, same construct

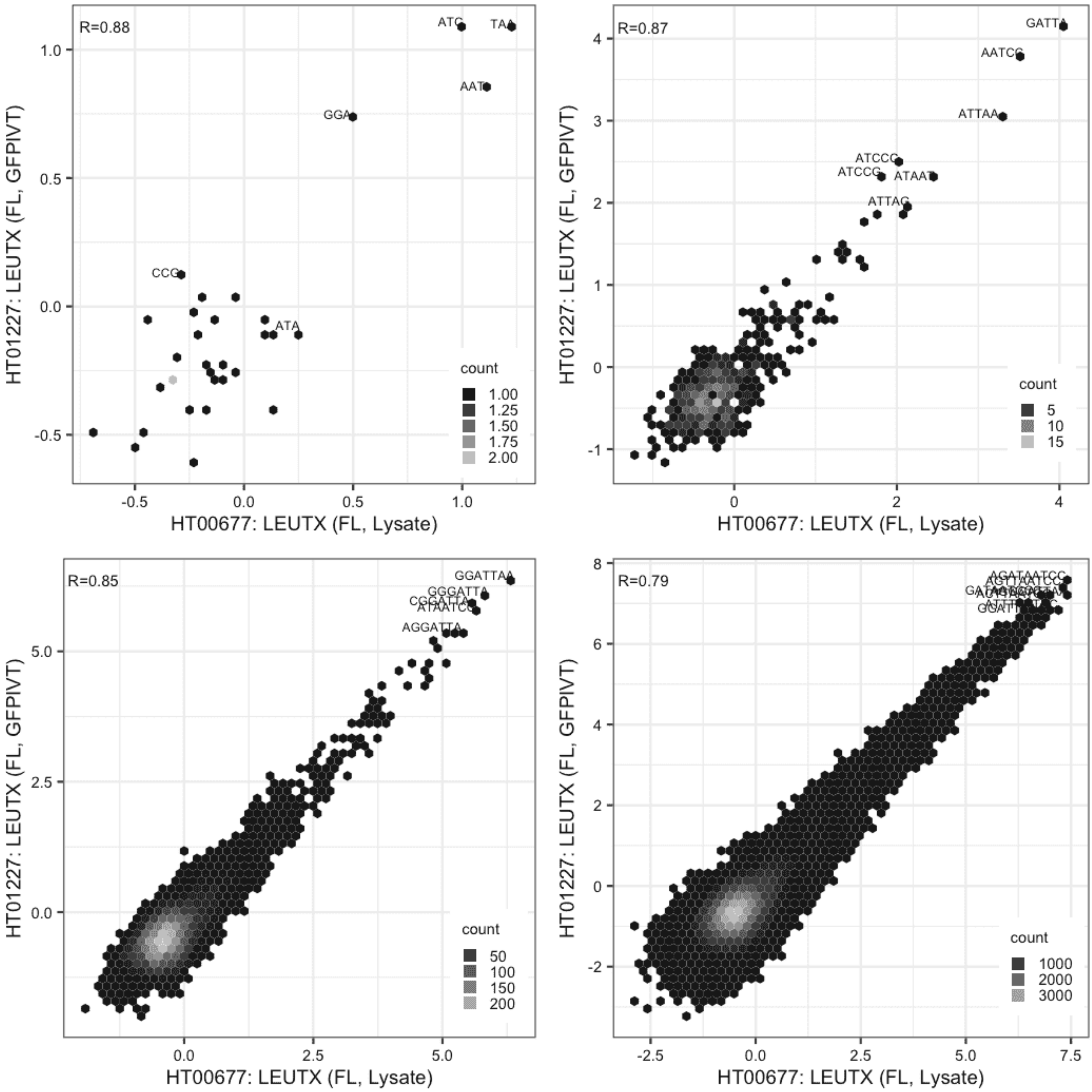

LEUTX

Lysate vs GFPIVT, same construct

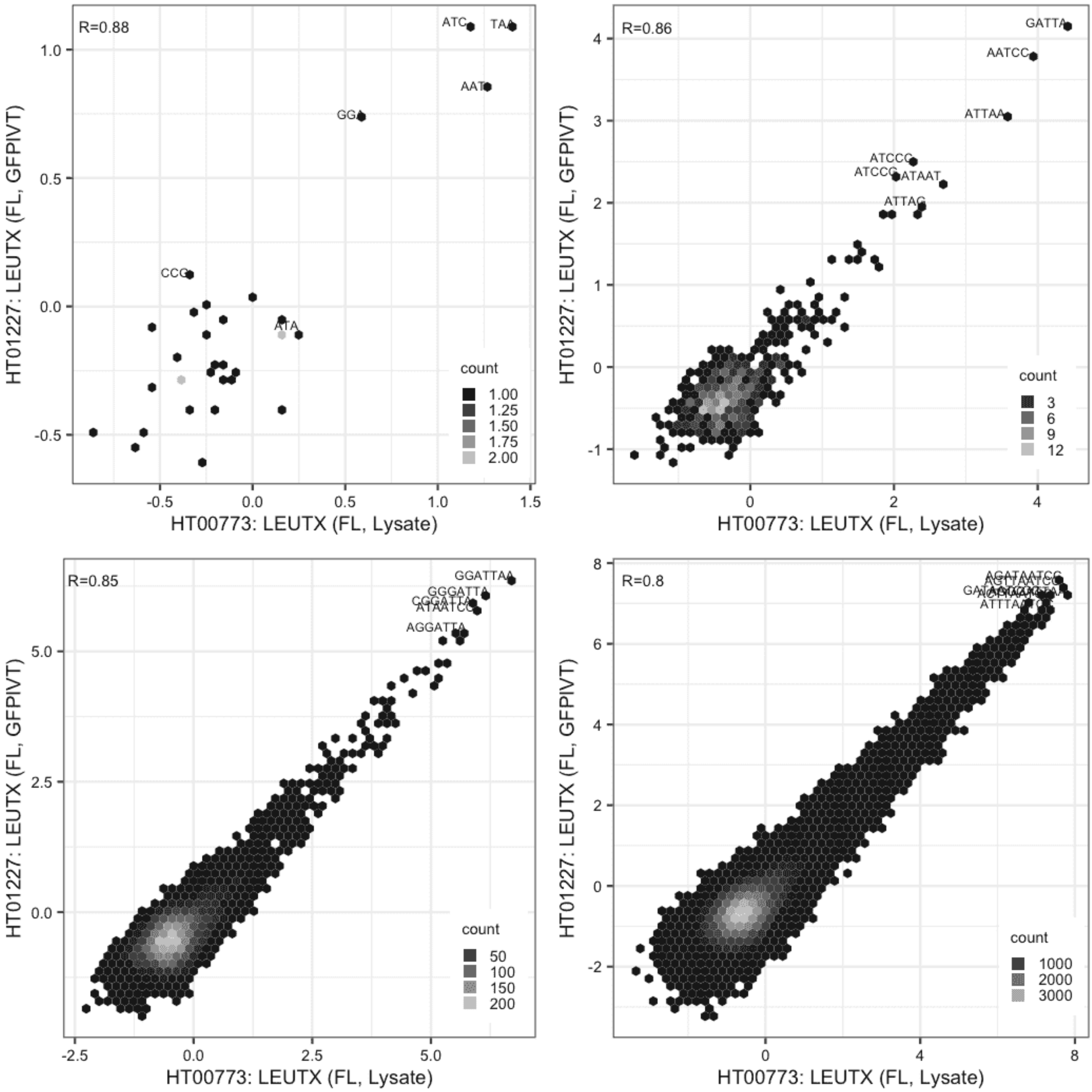

MAX

Lysate vs IVT, same construct

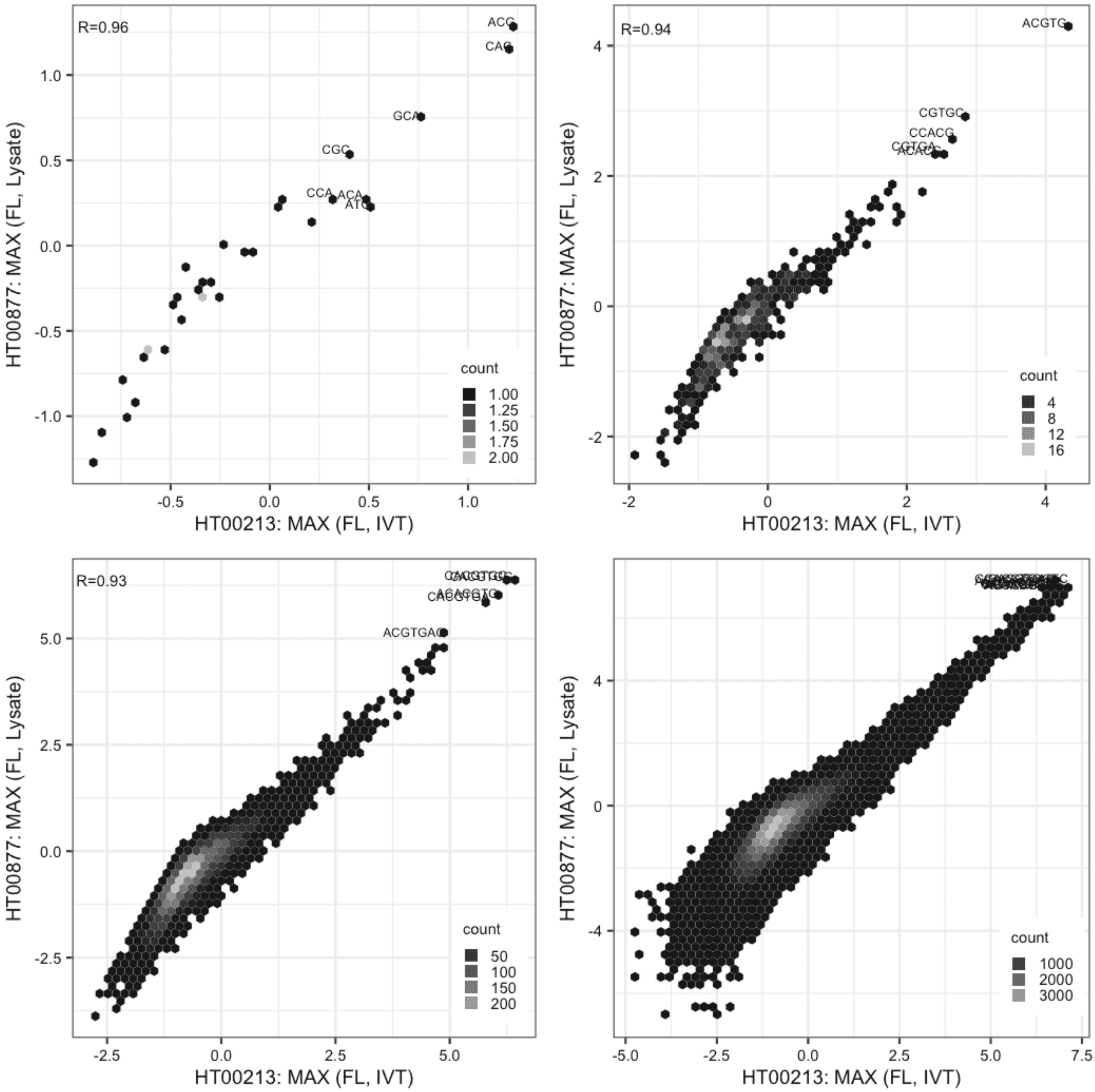

MAX

Lysate vs IVT, same construct

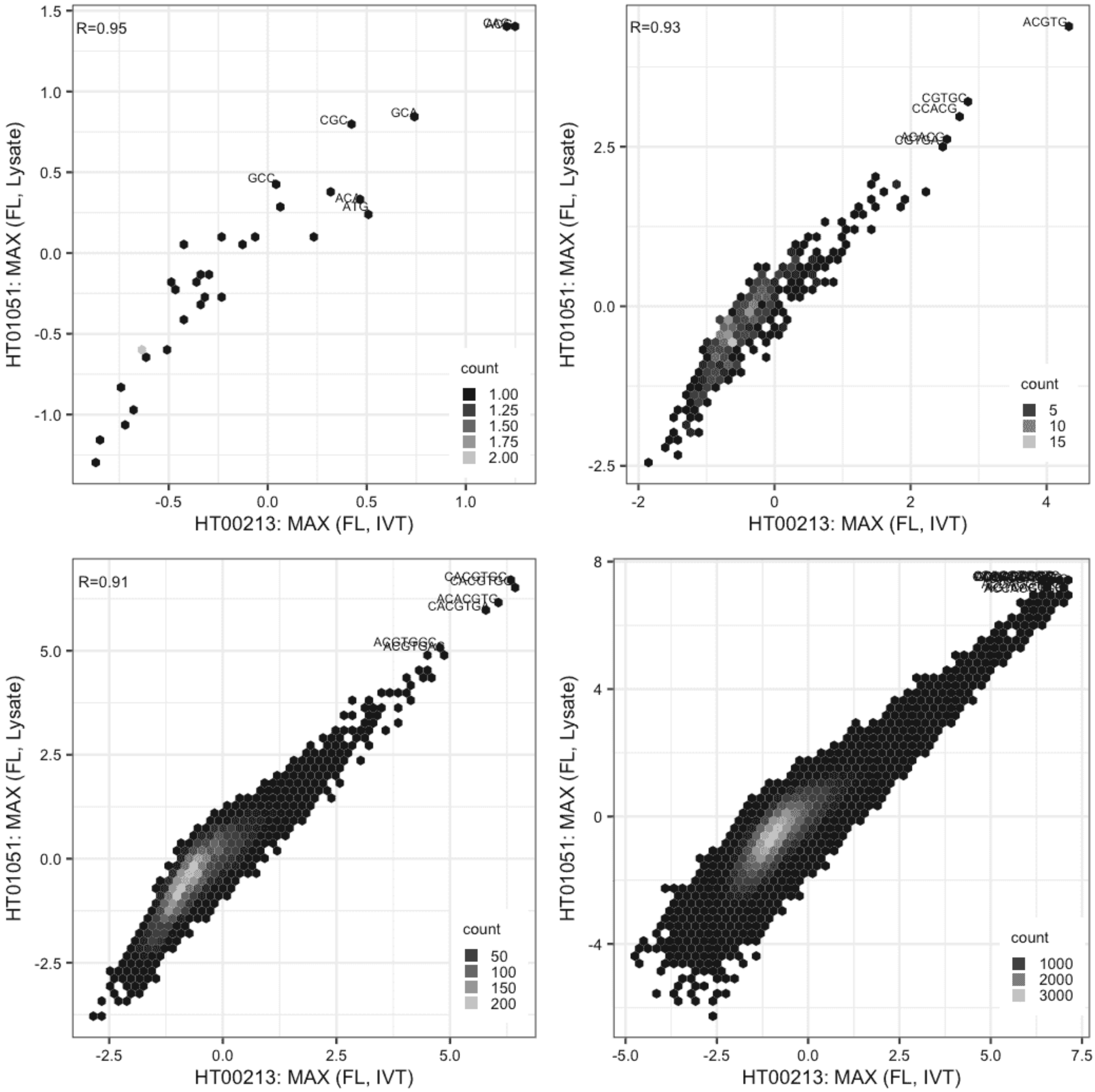

MAX

Lysate vs IVT, same construct

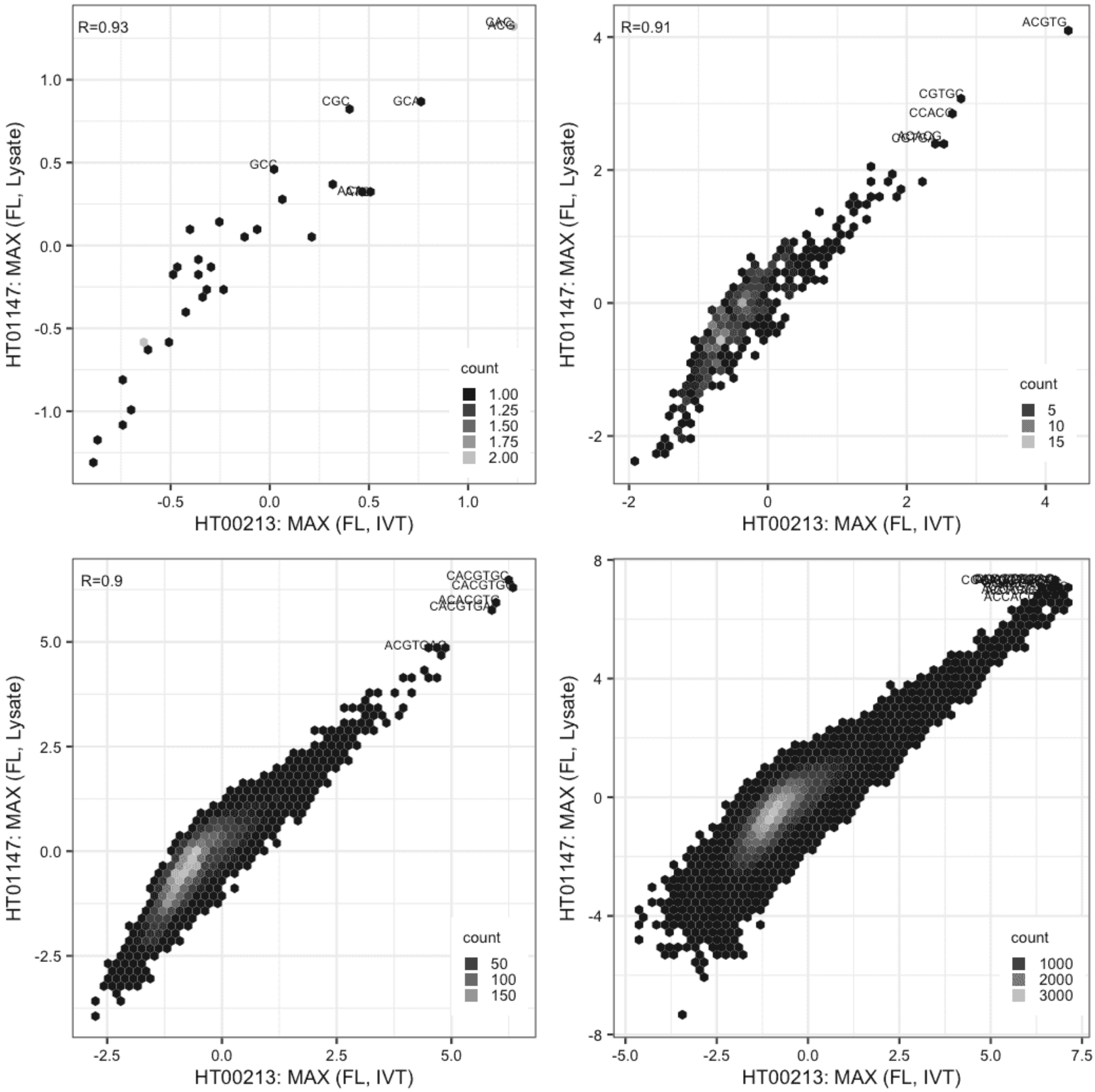

MAX

Lysate vs Lysate, same construct

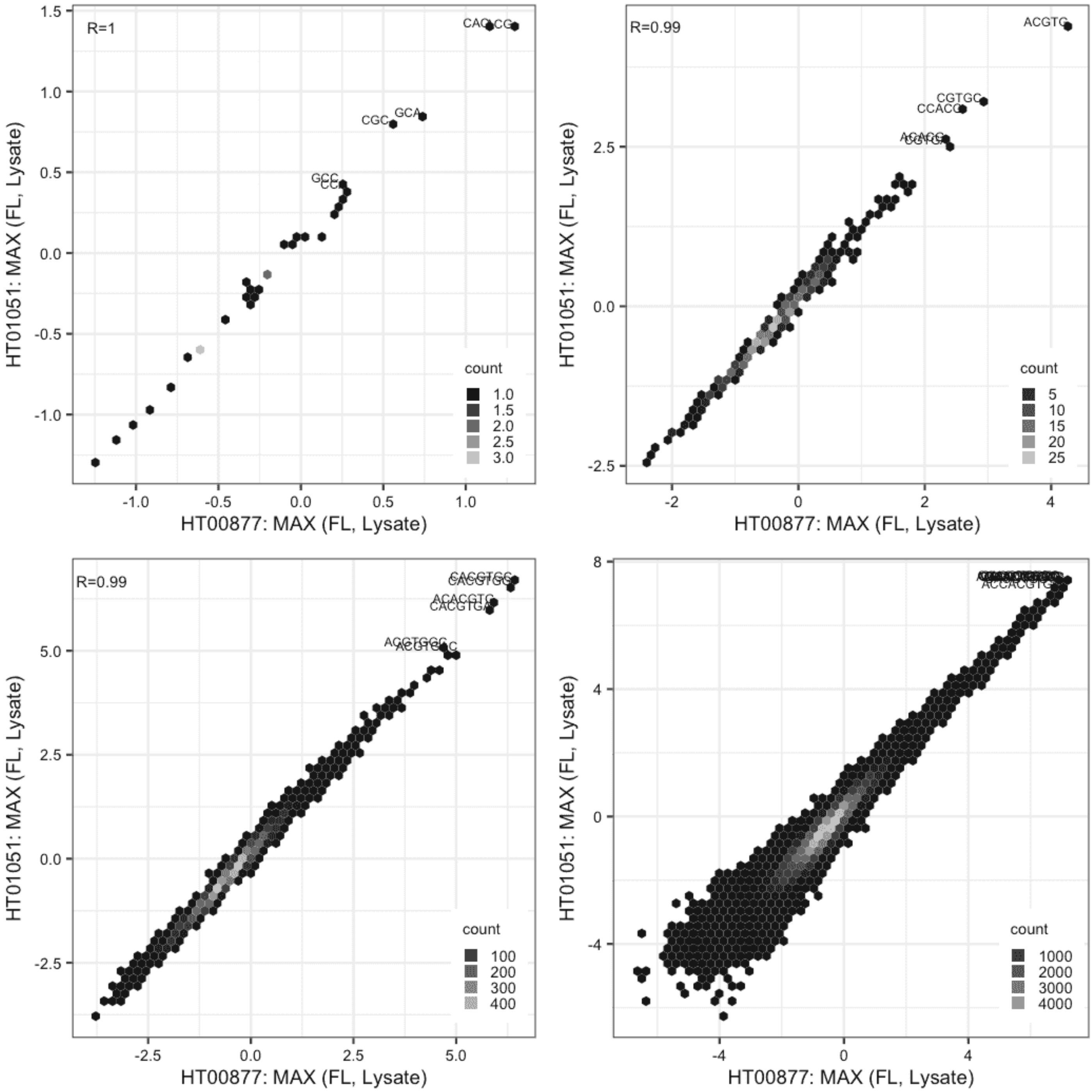

MAX

Lysate vs Lysate, same construct

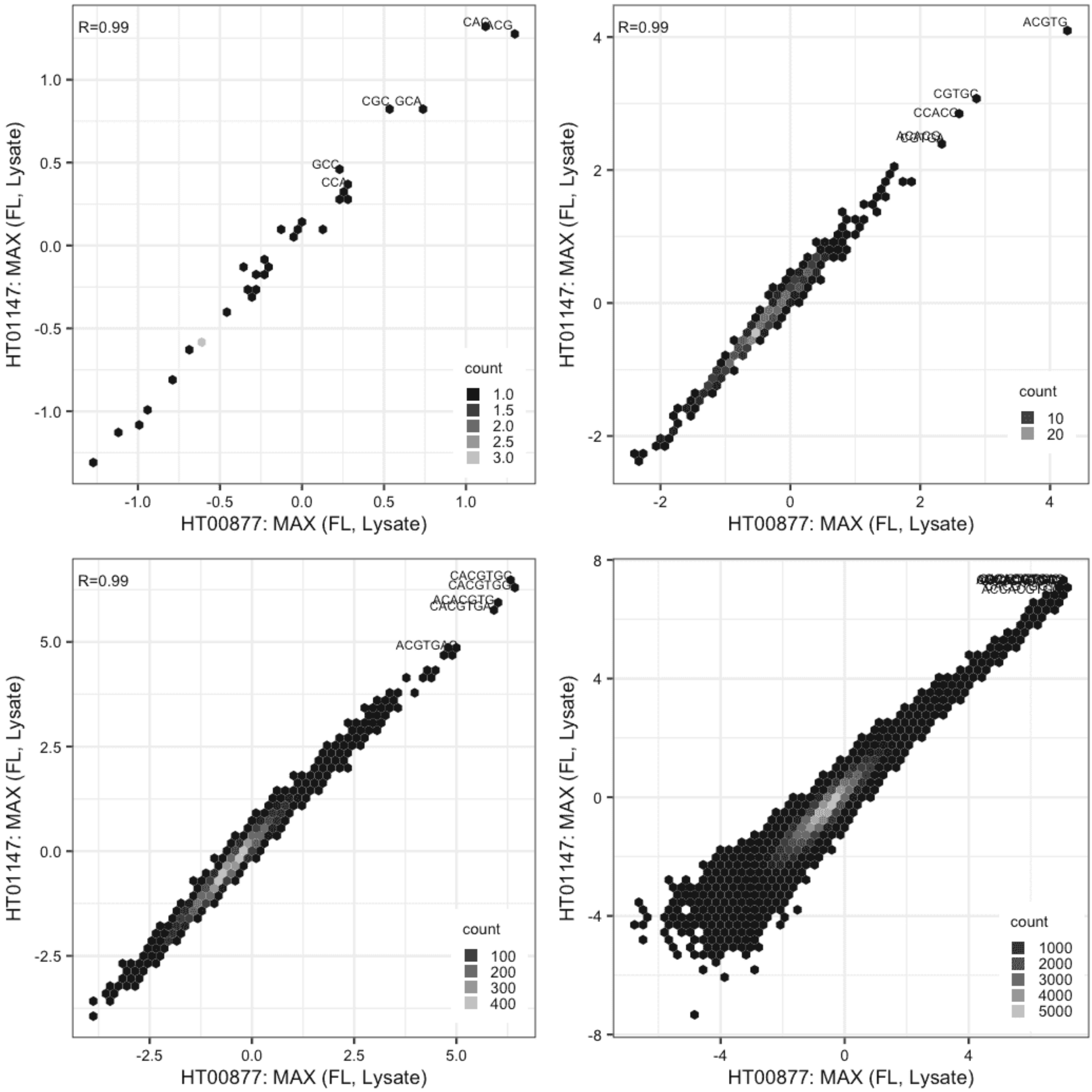

MAX

Lysate vs Lysate, same construct

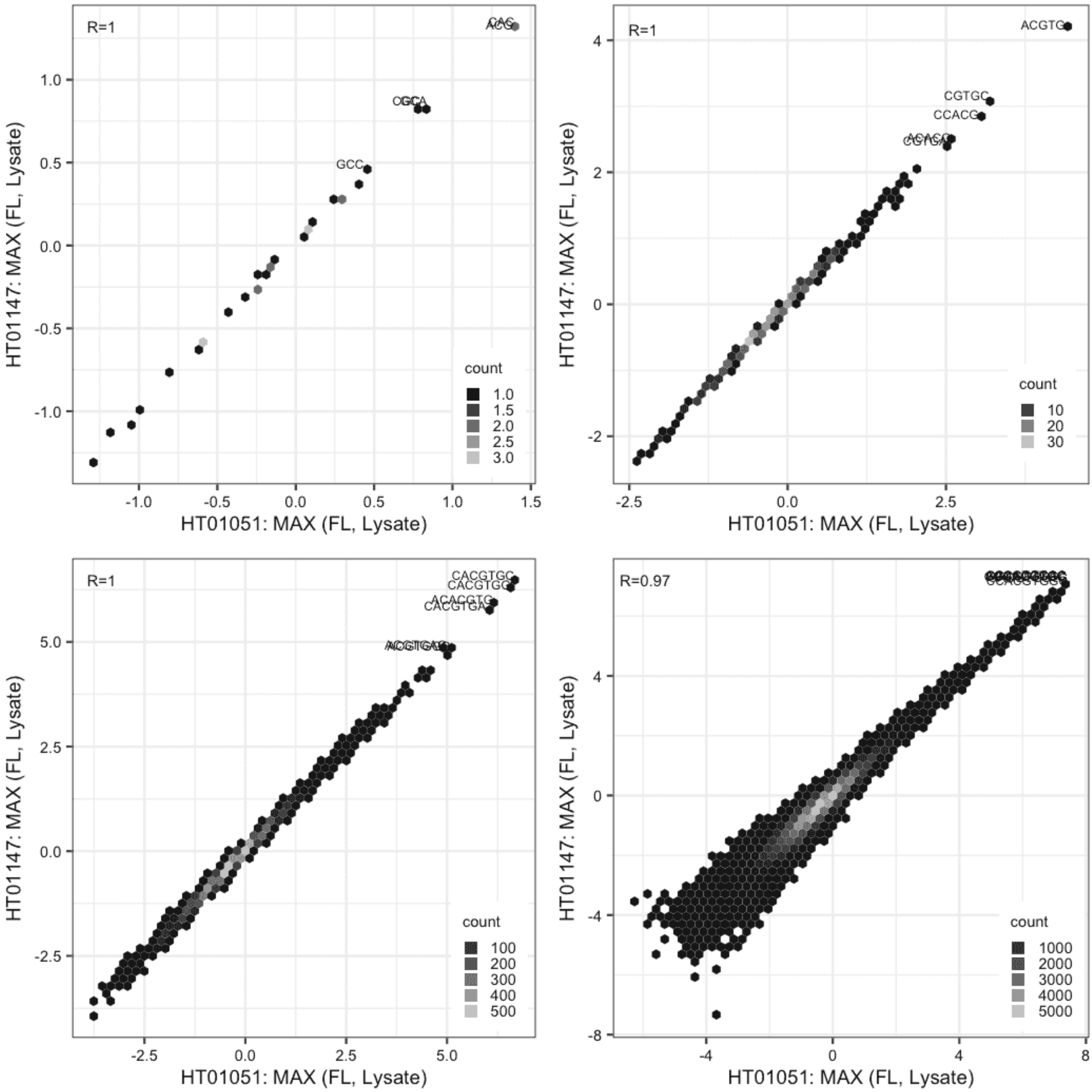

MKX

IVT vs IVT, different constructs

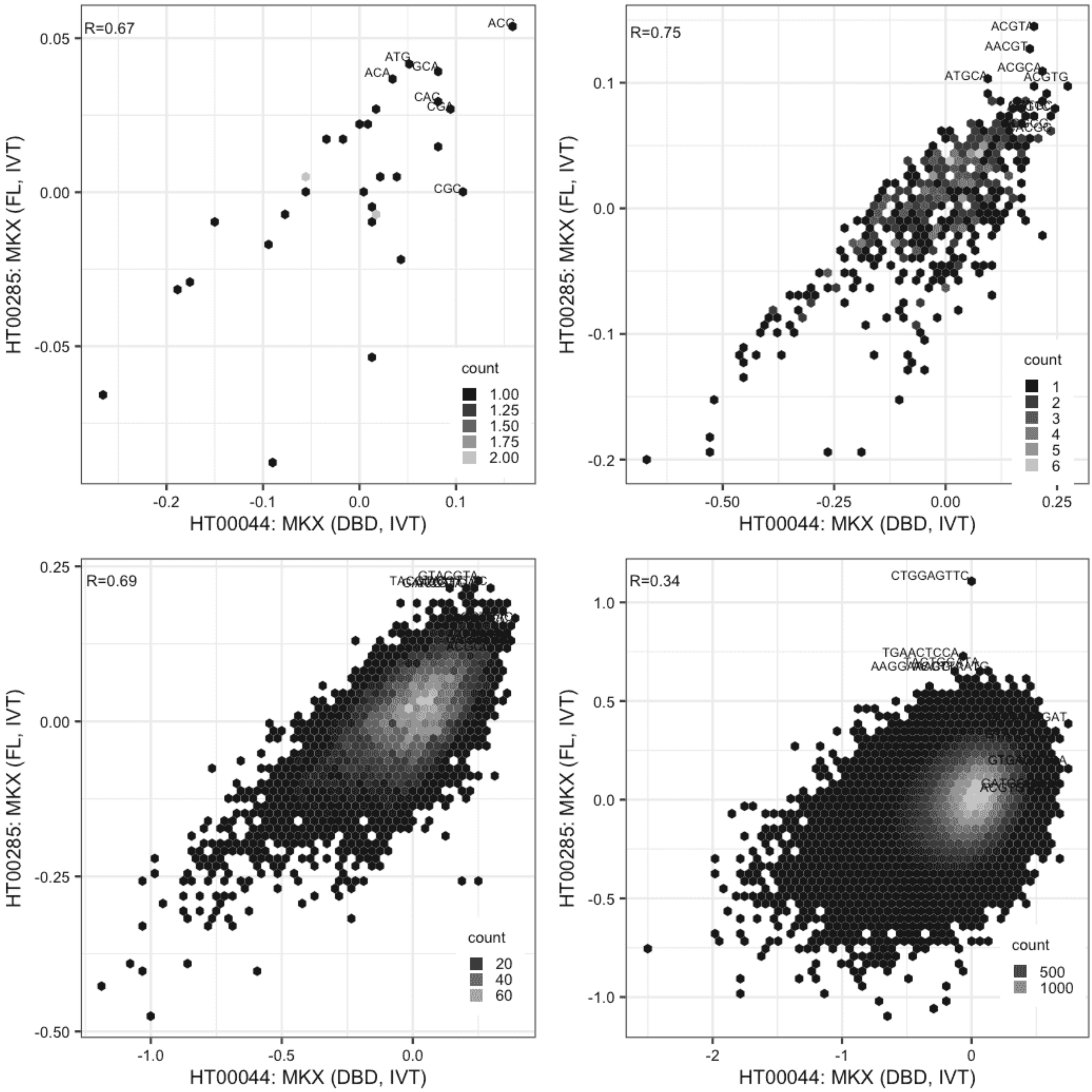

MKX

Lysate vs IVT, different constructs

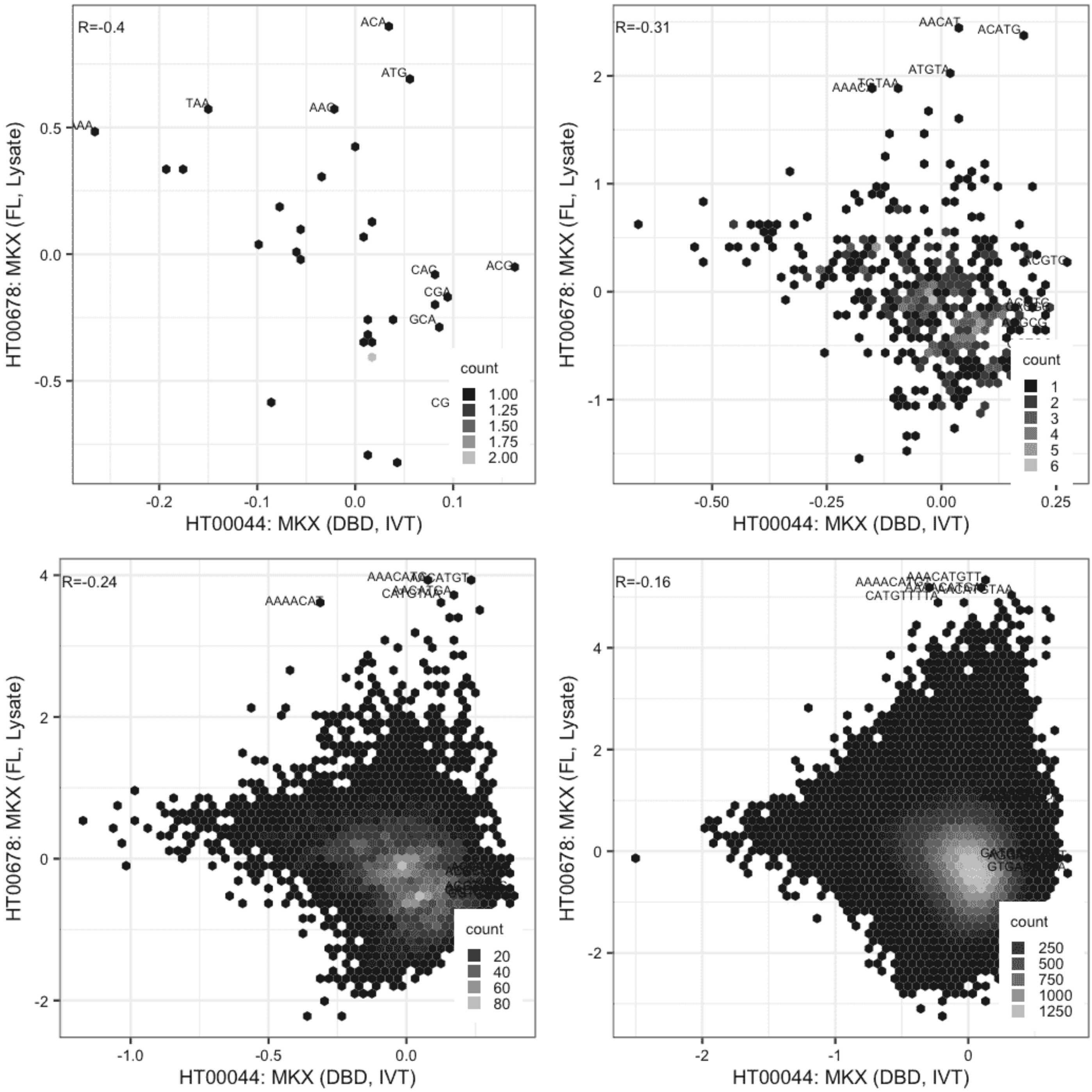

MKX

Lysate vs IVT, different constructs

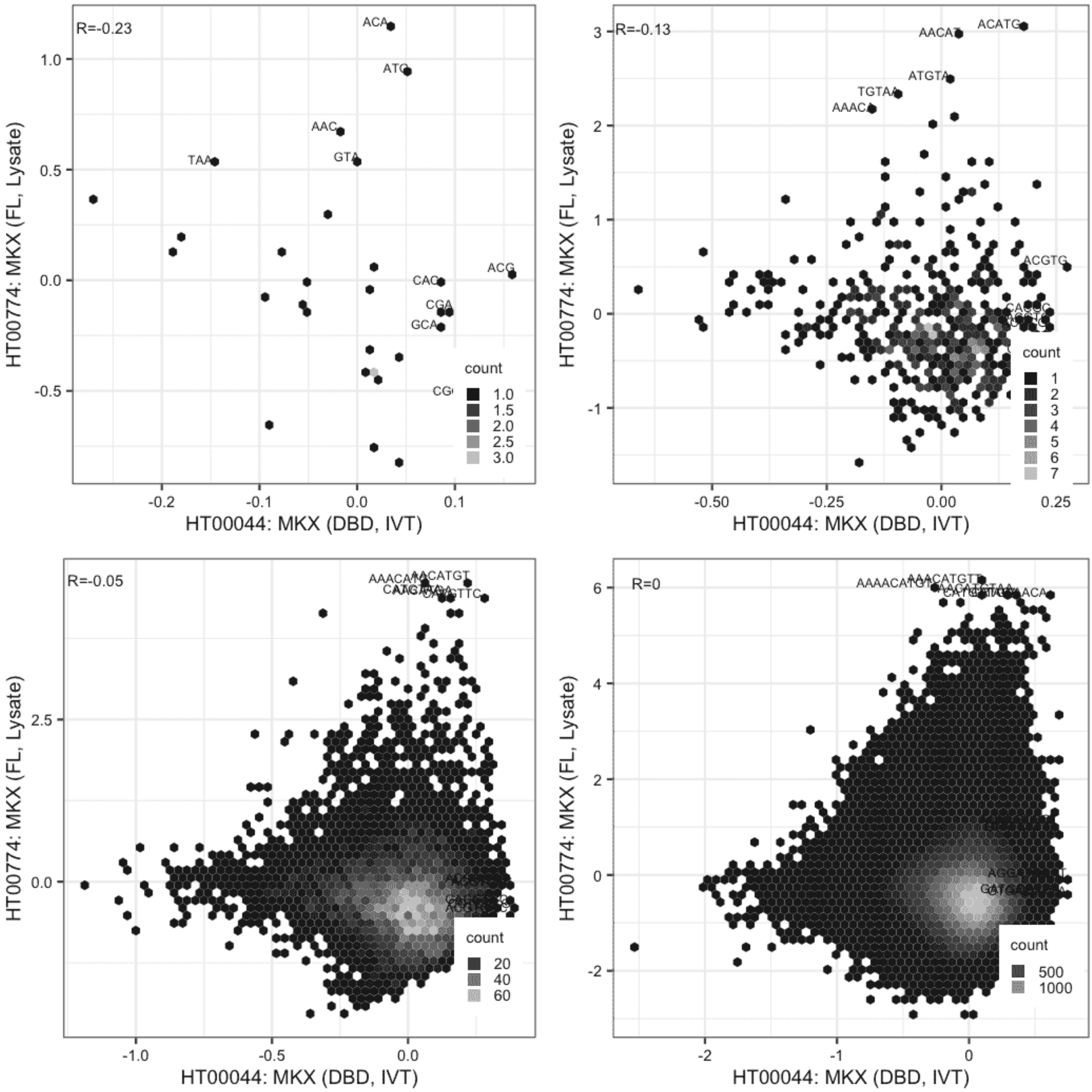

MKX

IVT vs GFPIVT, different constructs

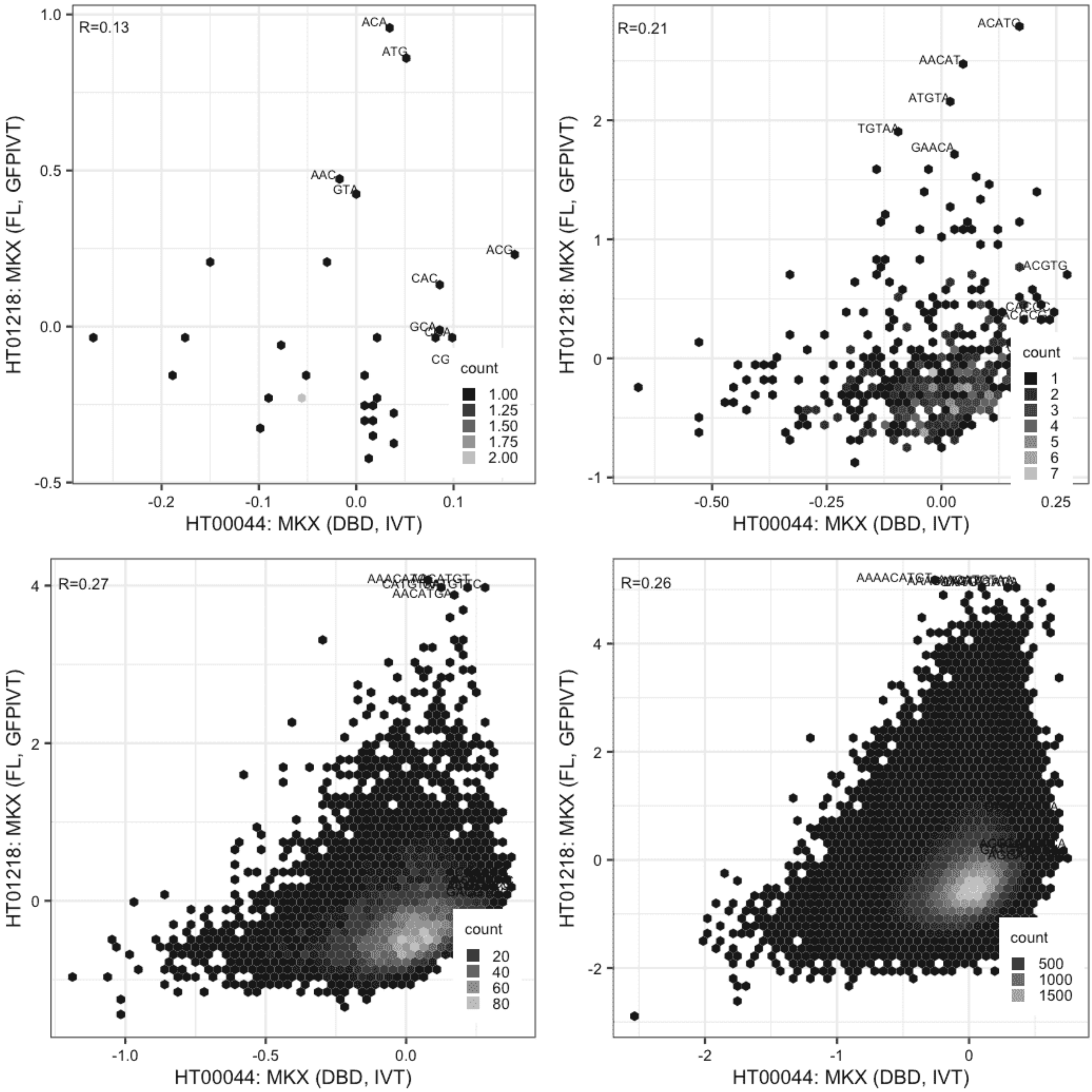

MKX

Lysate vs IVT, same construct

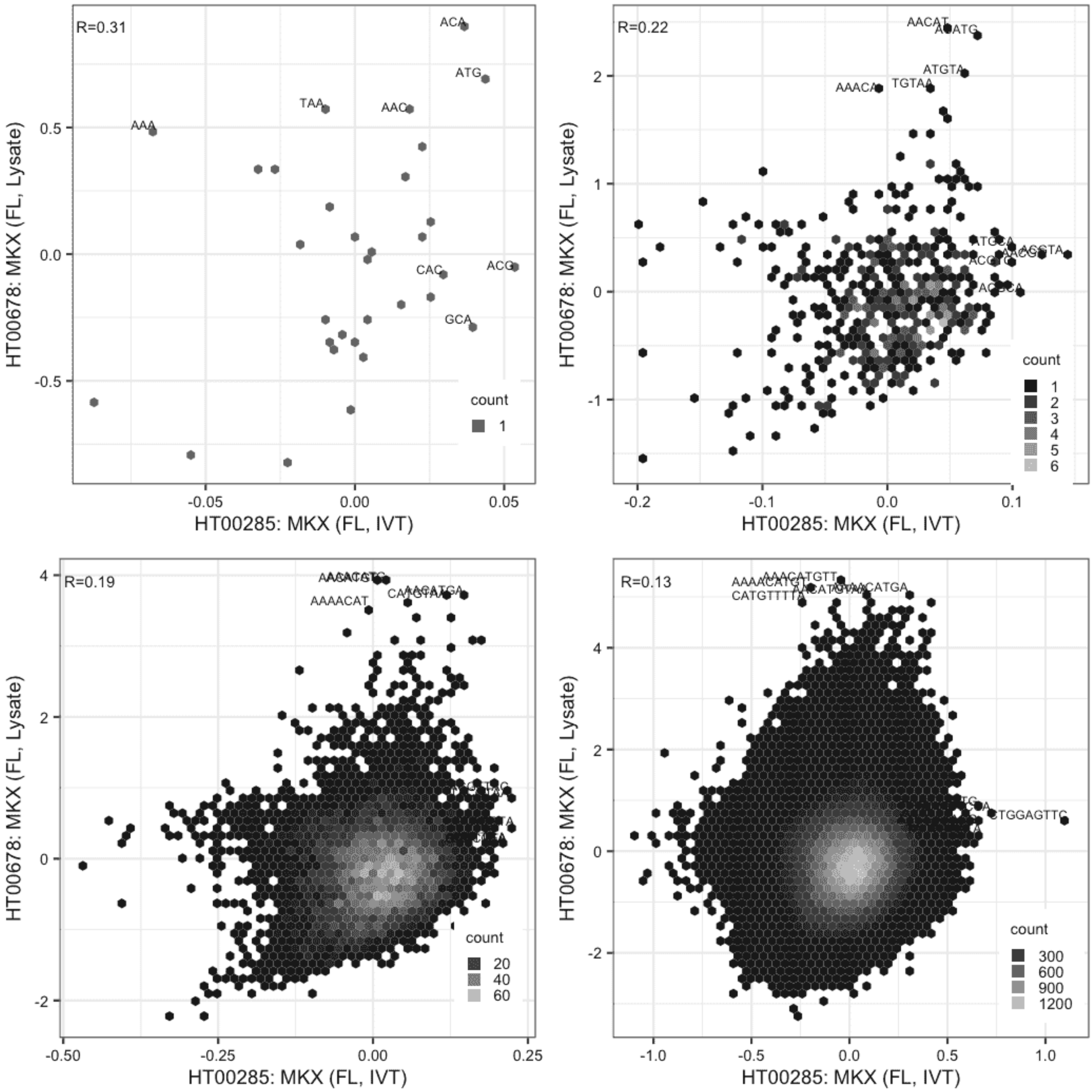

MKX

Lysate vs IVT, same construct

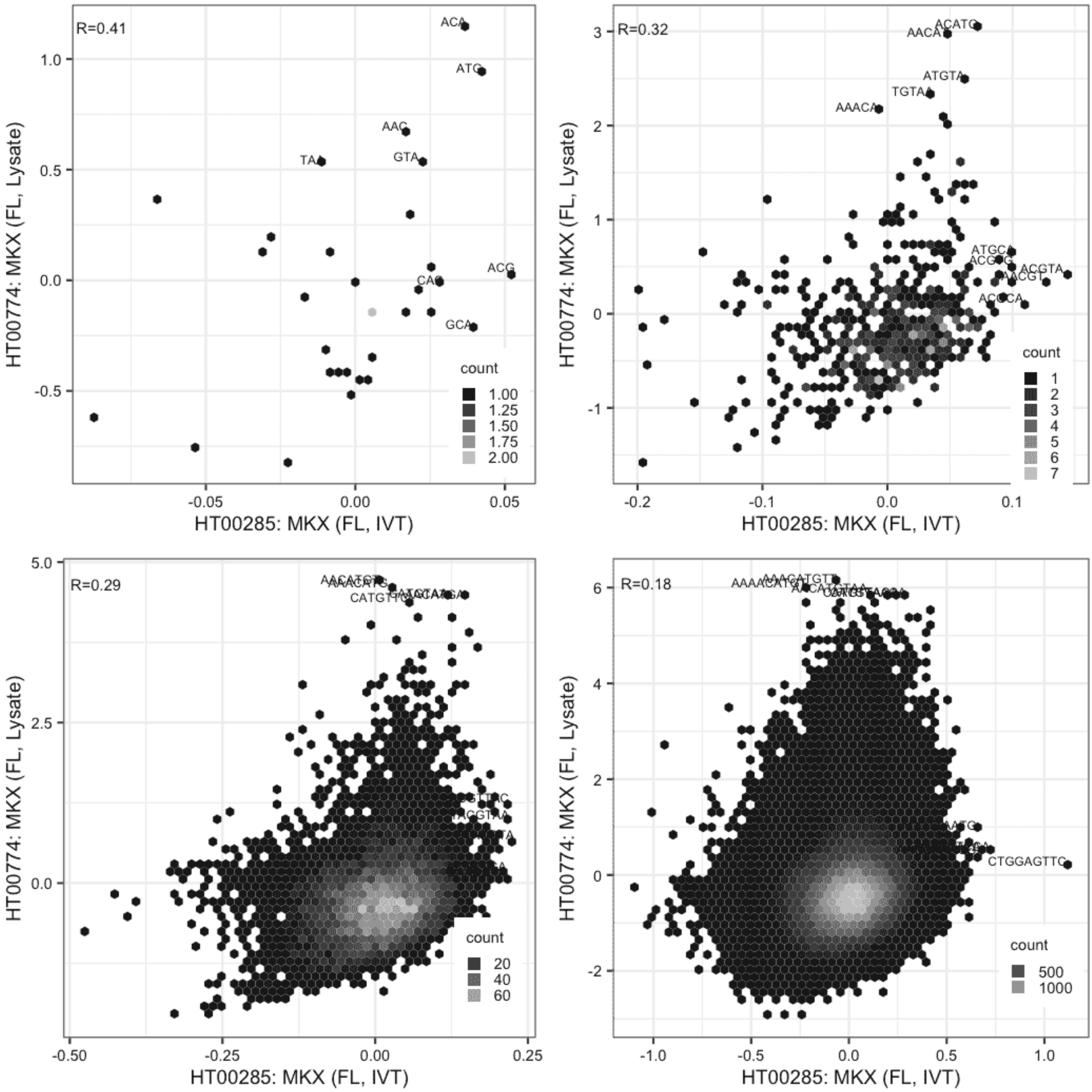

MKX

IVT vs GFPIVT, same construct

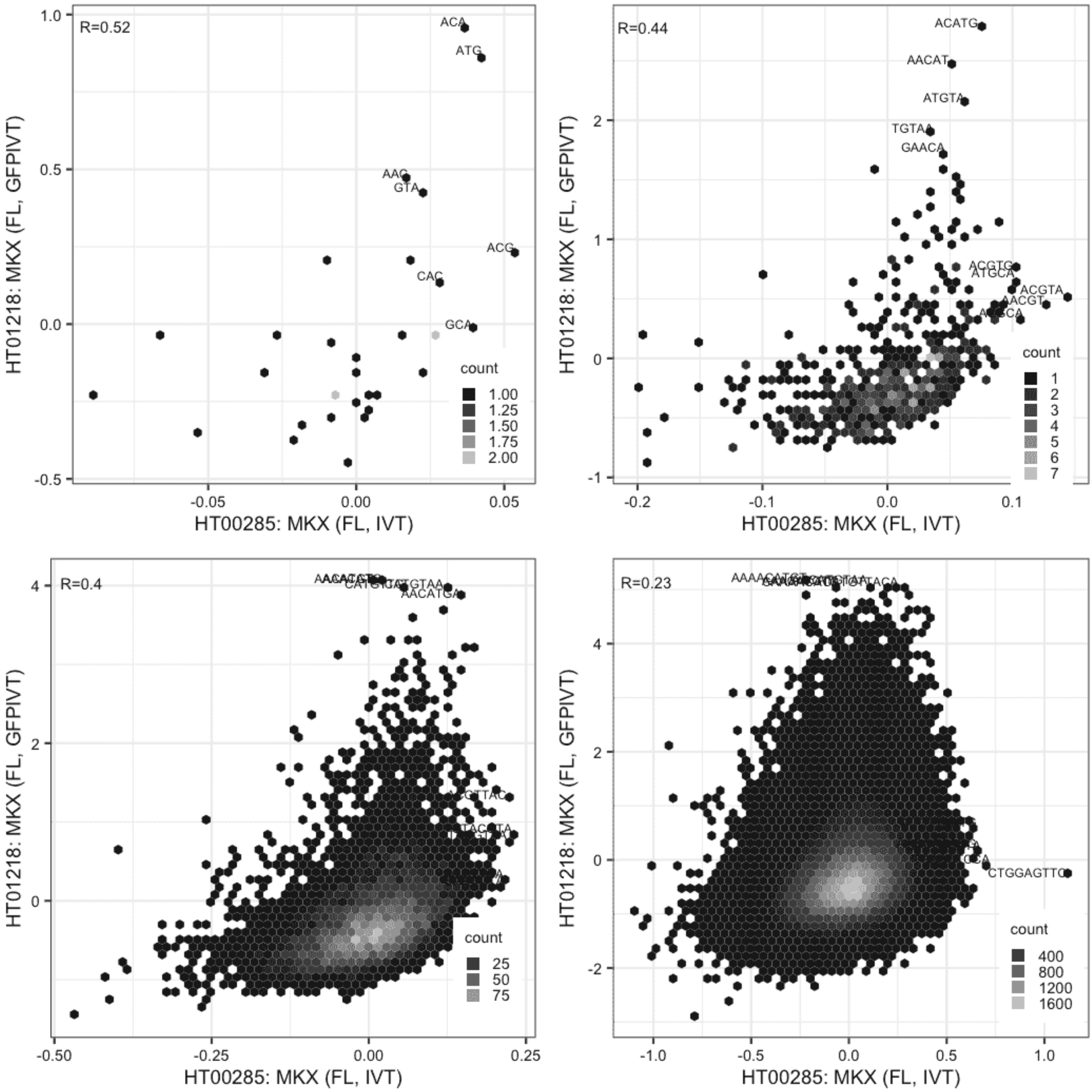

MKX

Lysate vs Lysate, same construct

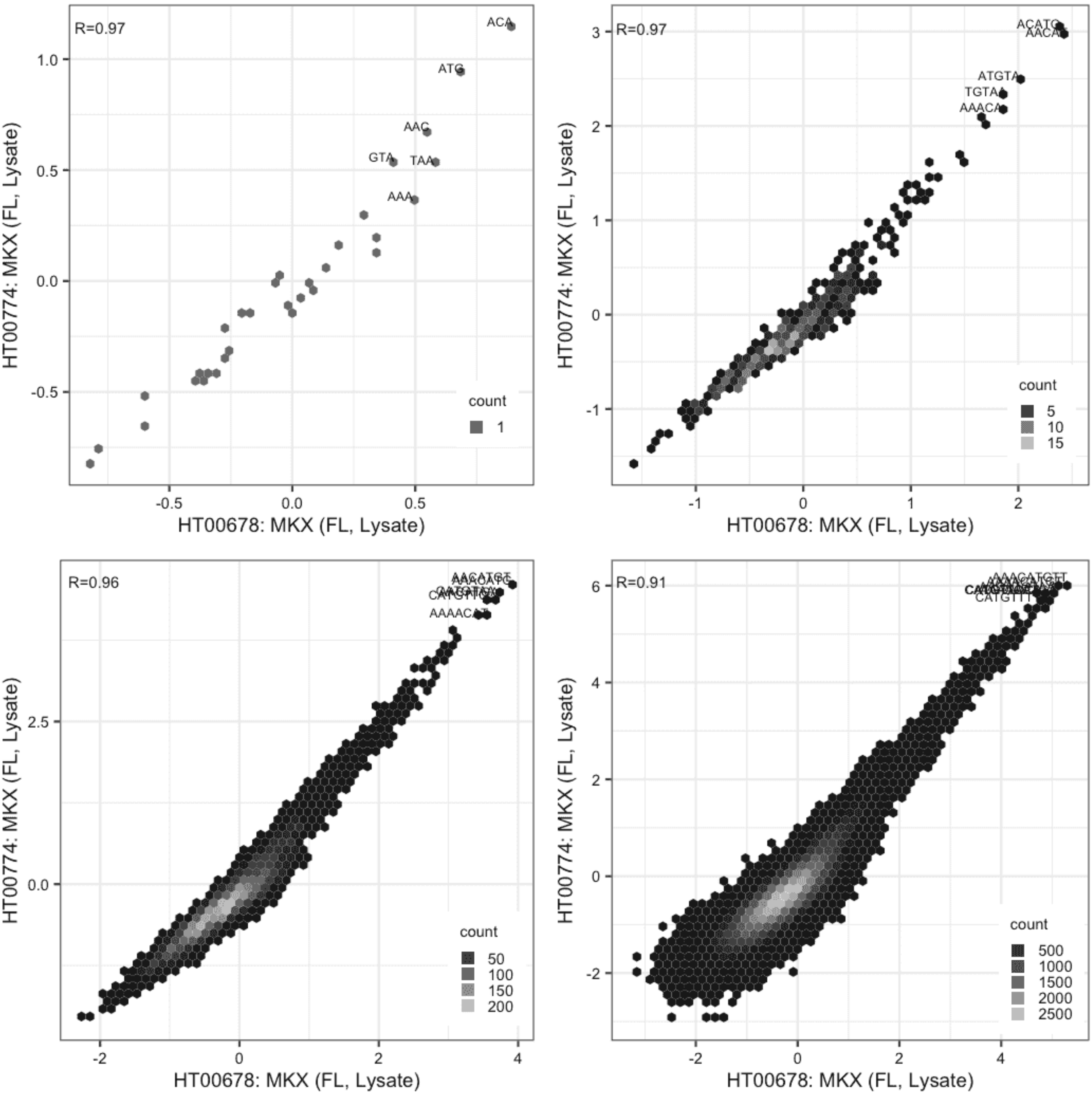

MKX

Lysate vs GFPIVT, same construct

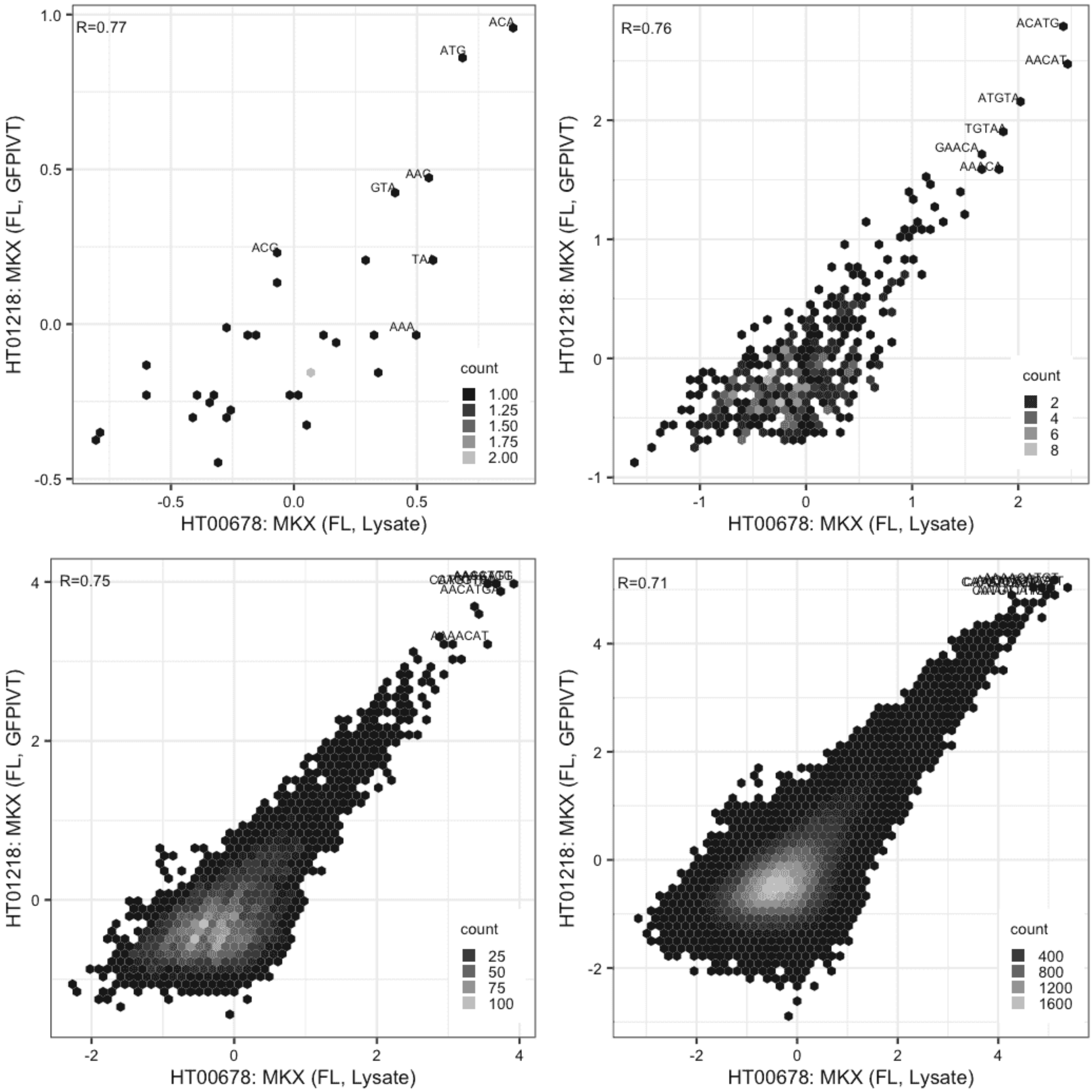

MKX

Lysate vs GFPIVT, same construct

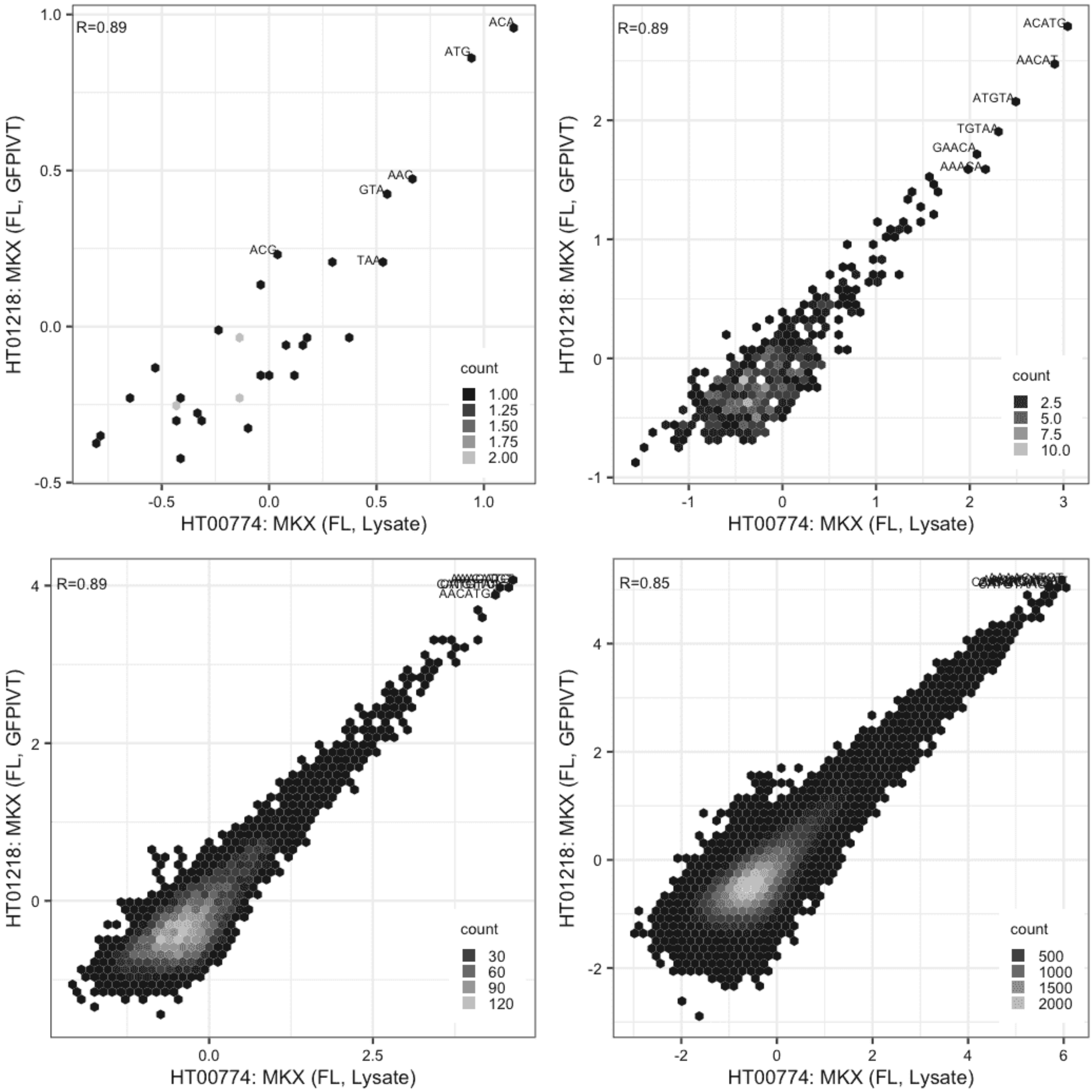

MSANTD1

Lysate vs IVT, different constructs

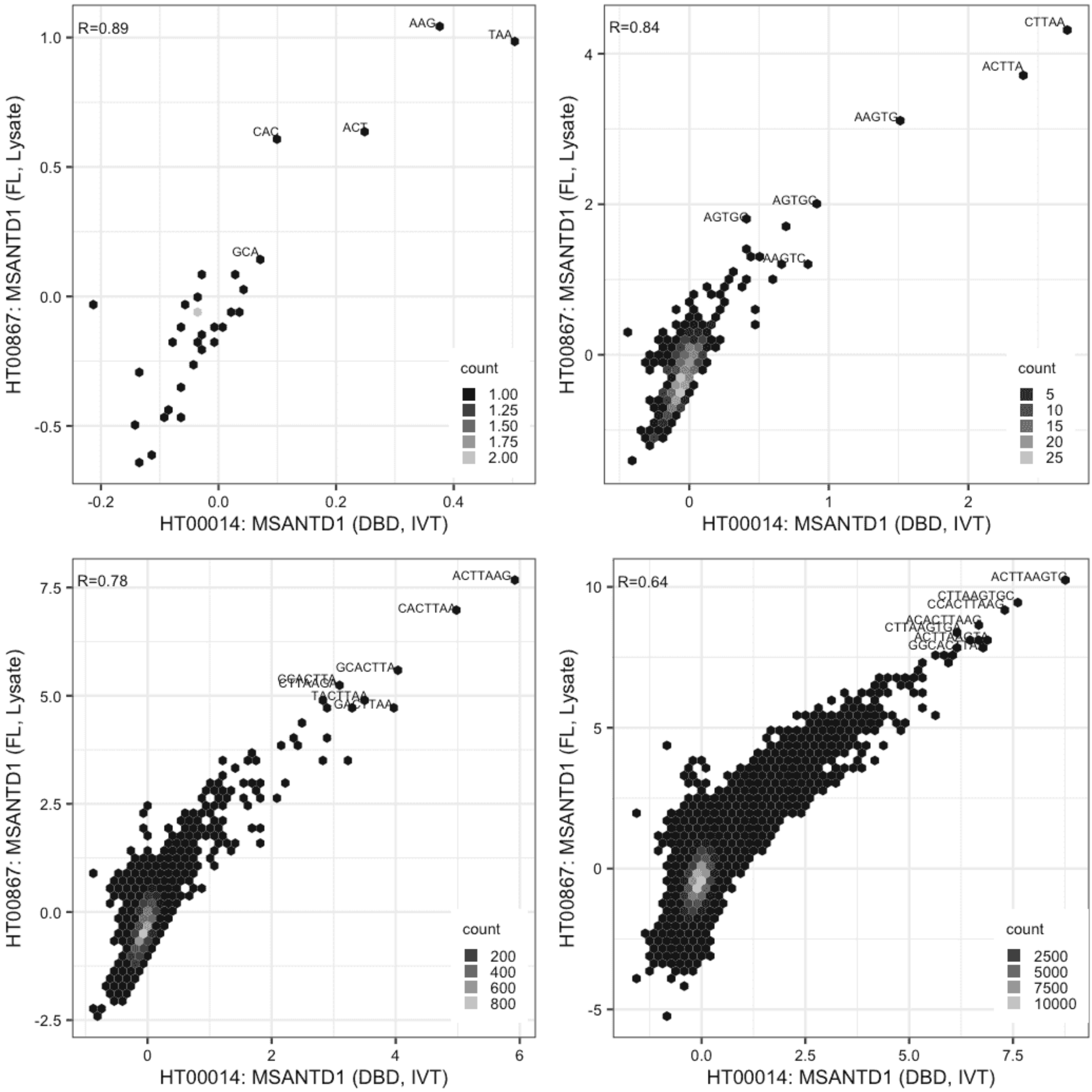

MSANTD1

IVT vs GFPIVT, different constructs

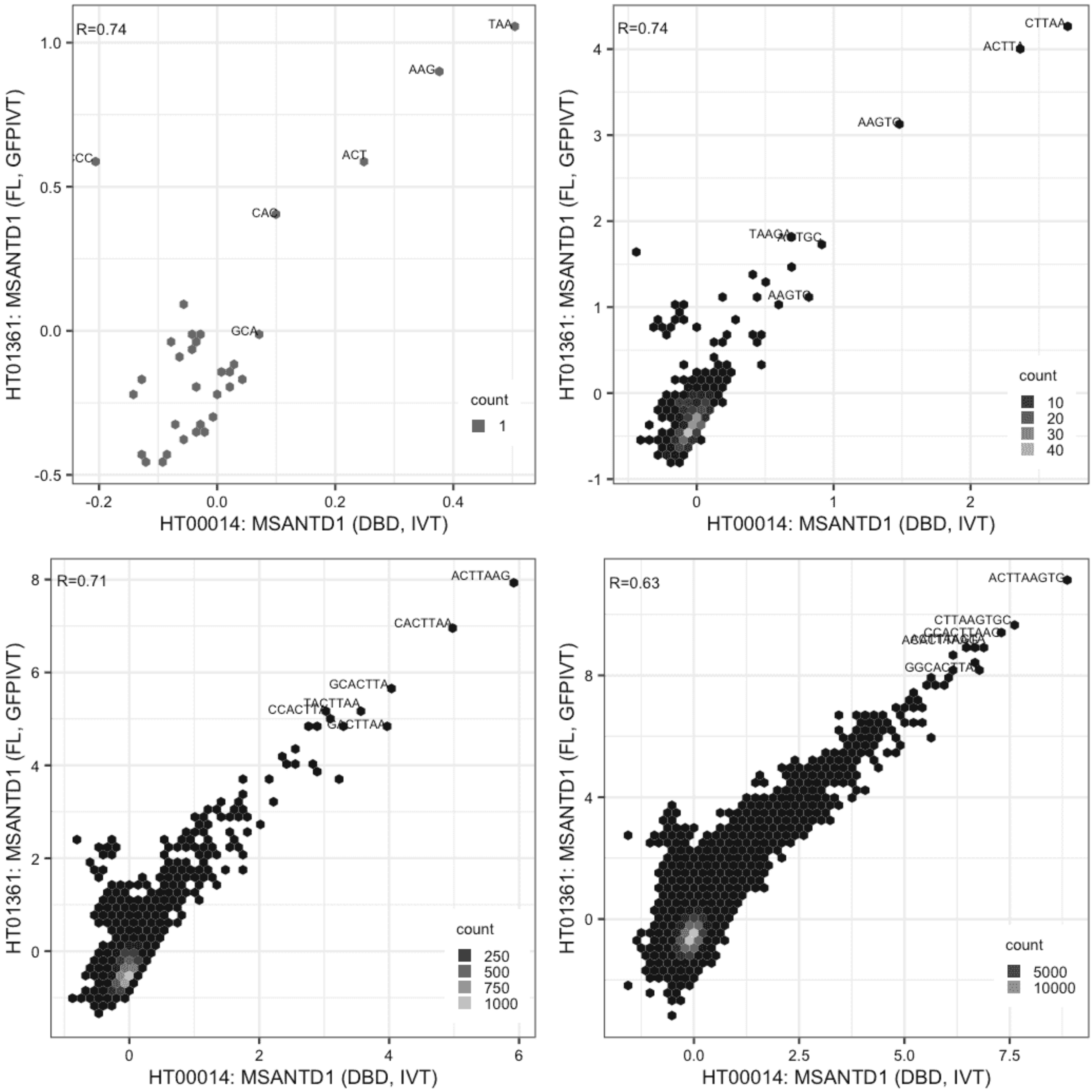

MSANTD1

Lysate vs GFPIVT, same construct

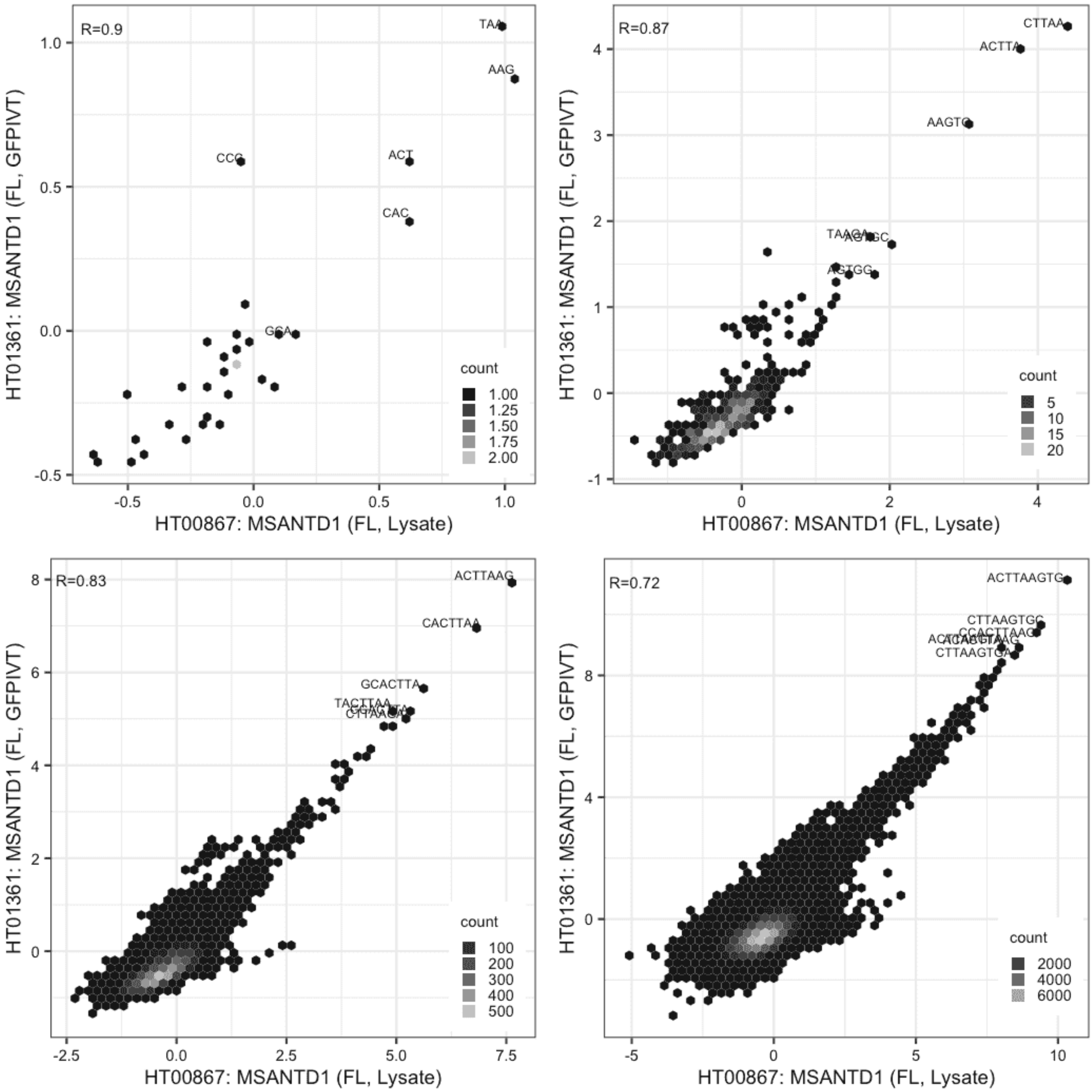

MSANTD4

Lysate vs GFPIVT, same construct

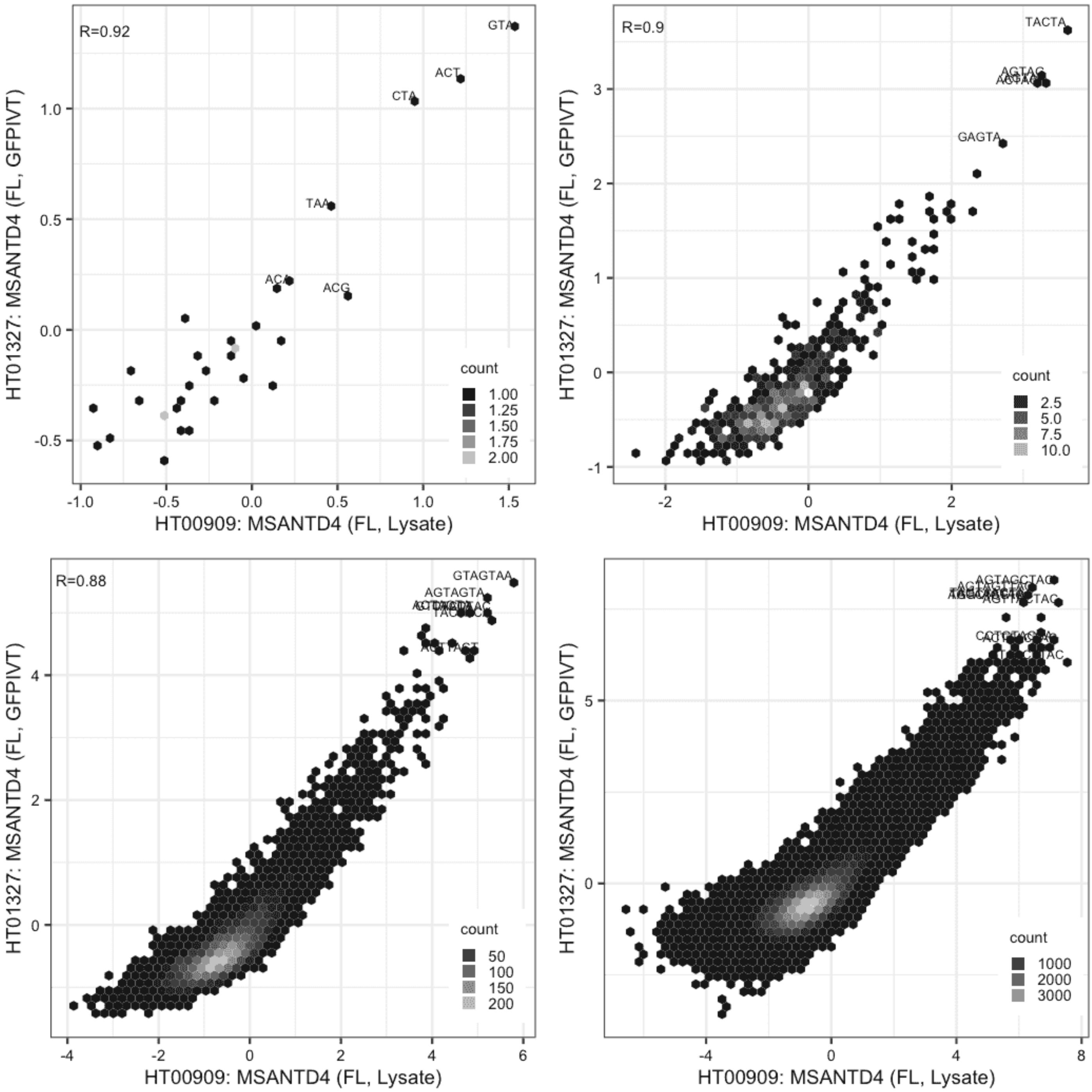

MYF6

Lysate vs IVT, same construct

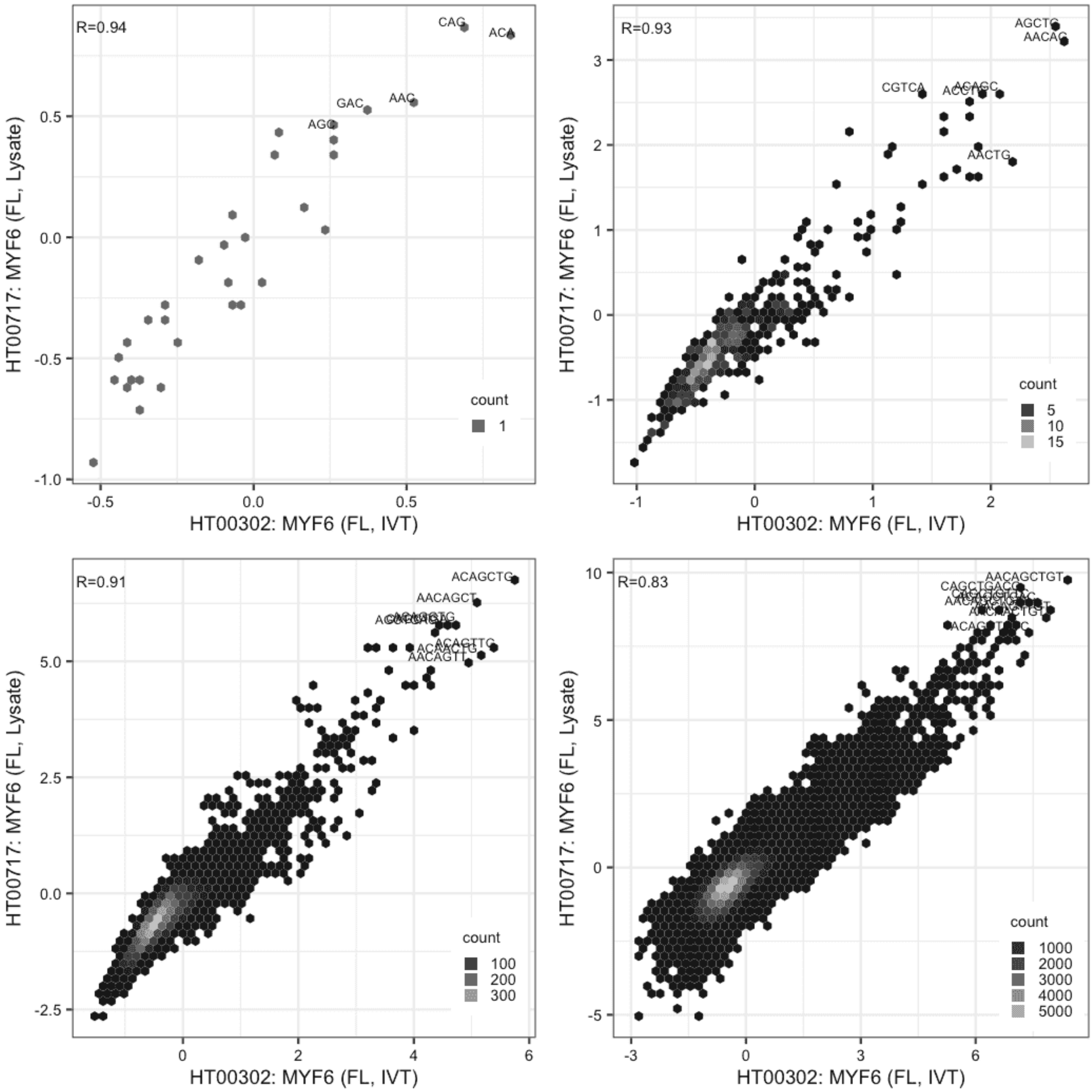

MYF6

Lysate vs IVT, same construct

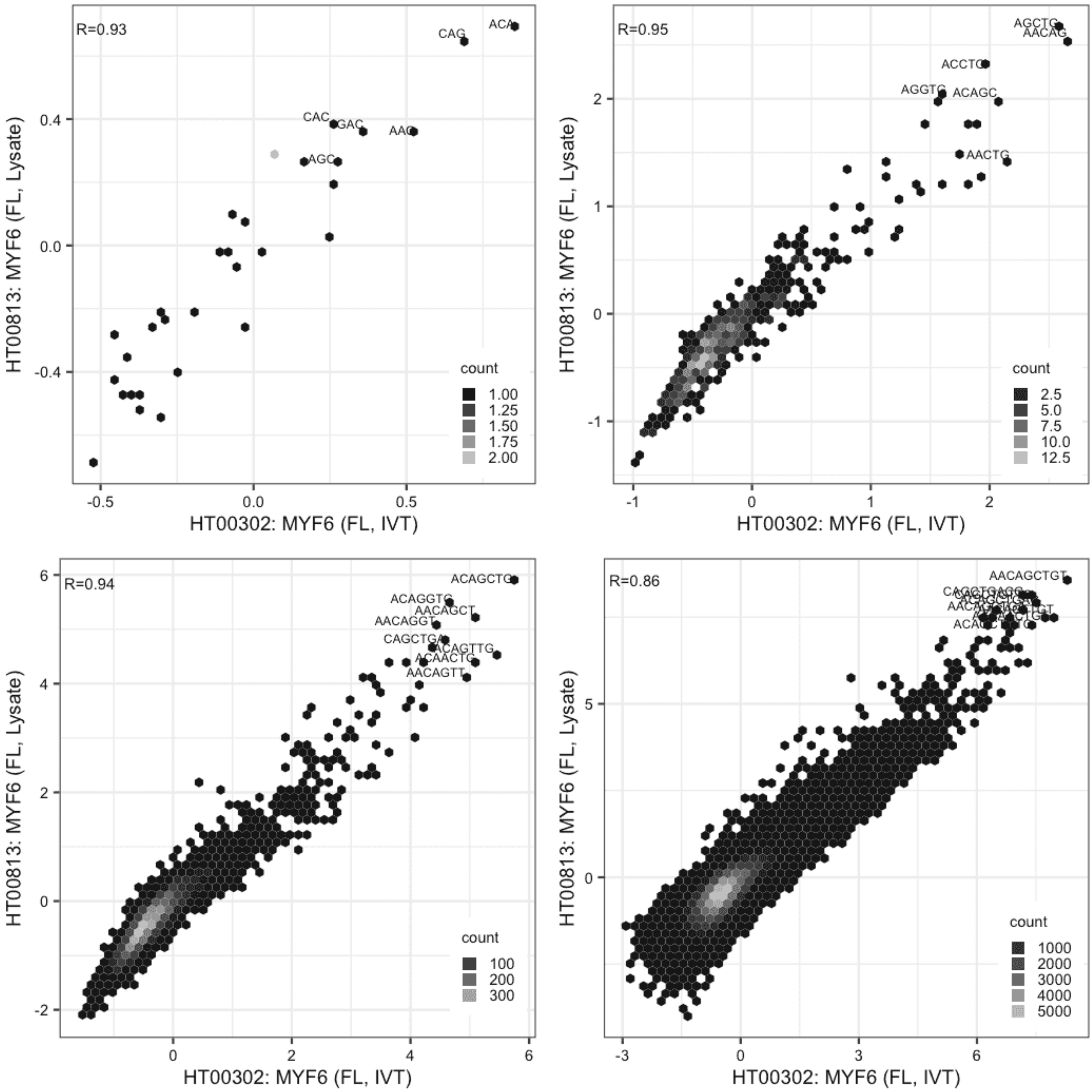

MYF6

Lysate vs Lysate, same construct

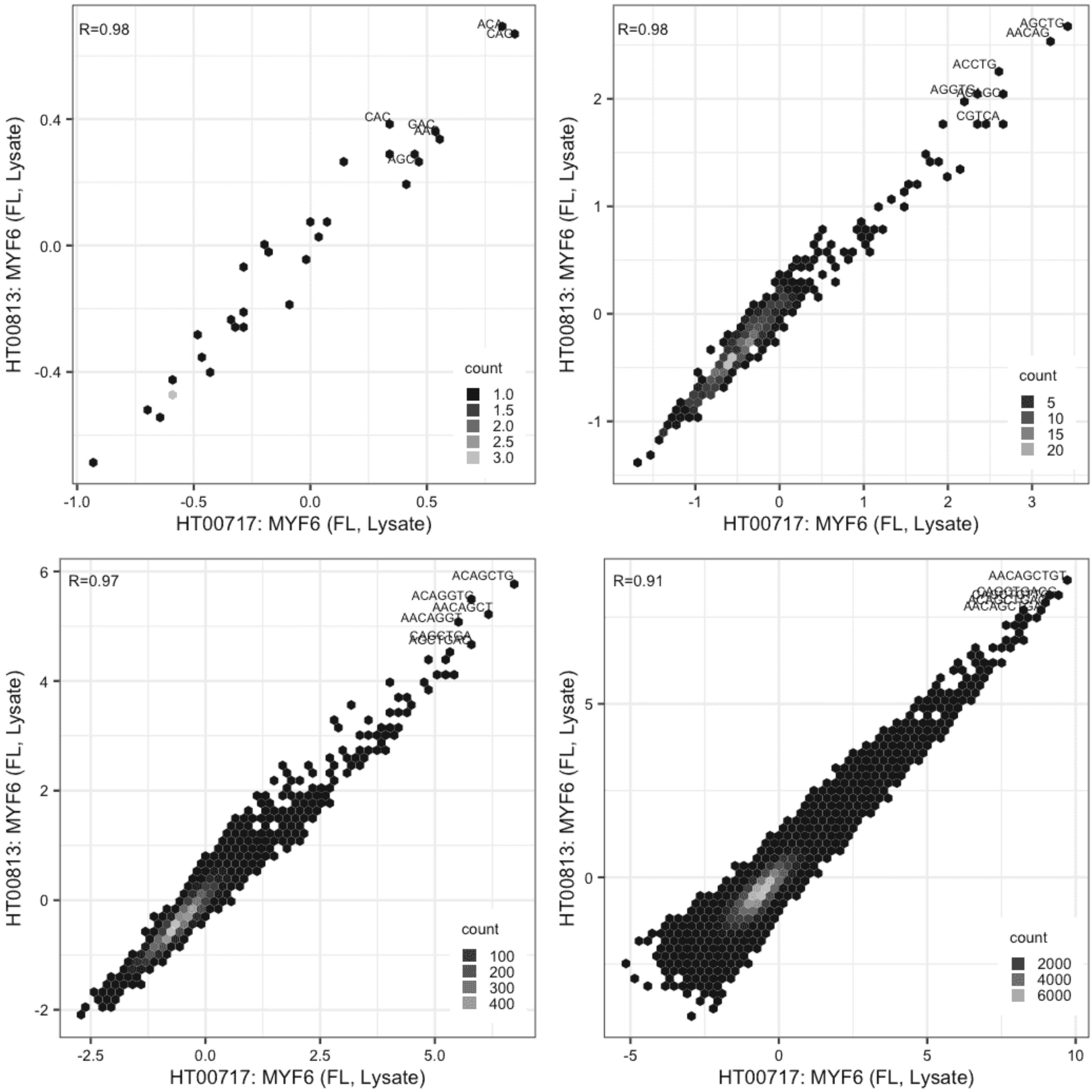

MYPOP

IVT vs IVT, different constructs

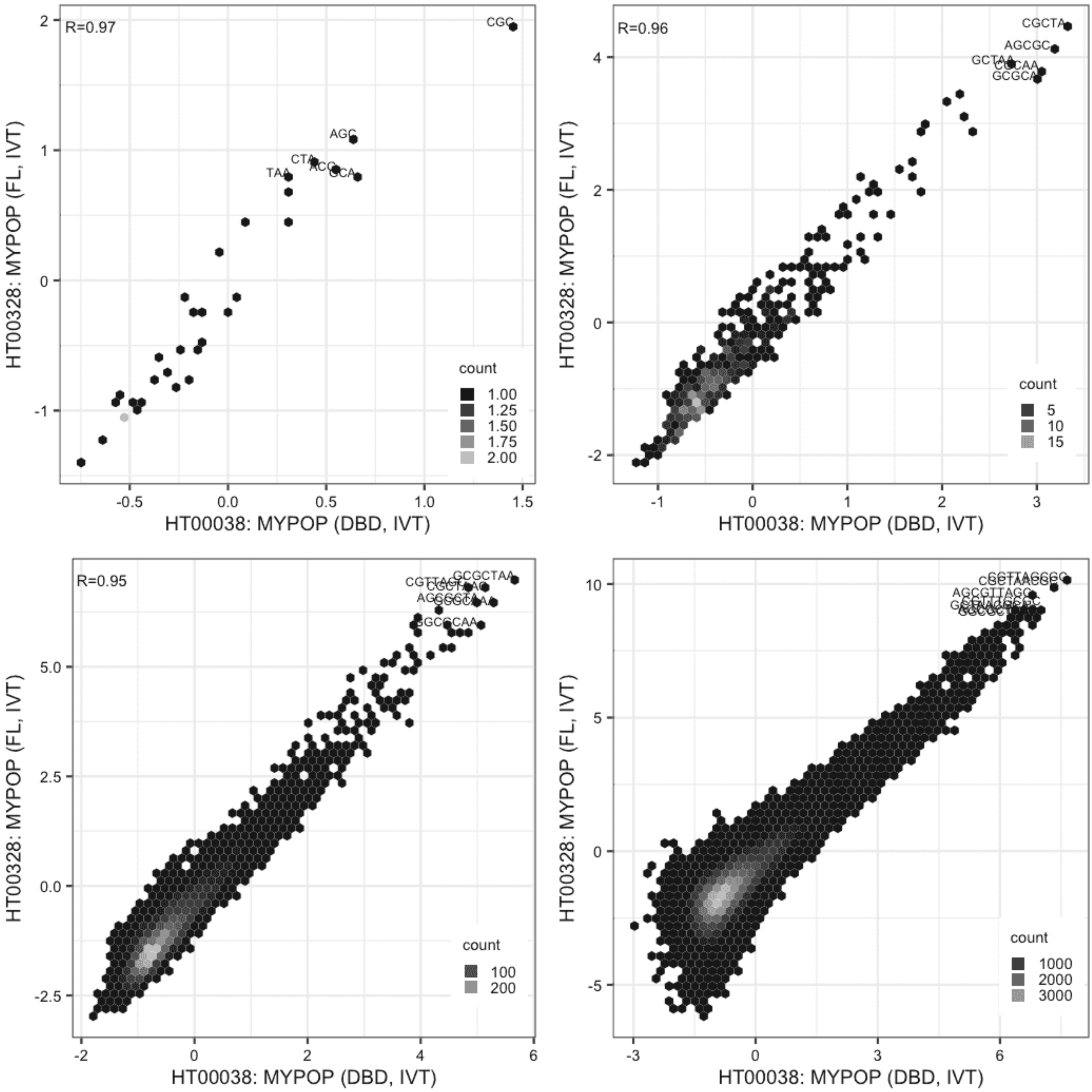

MYPOP

Lysate vs IVT, different constructs

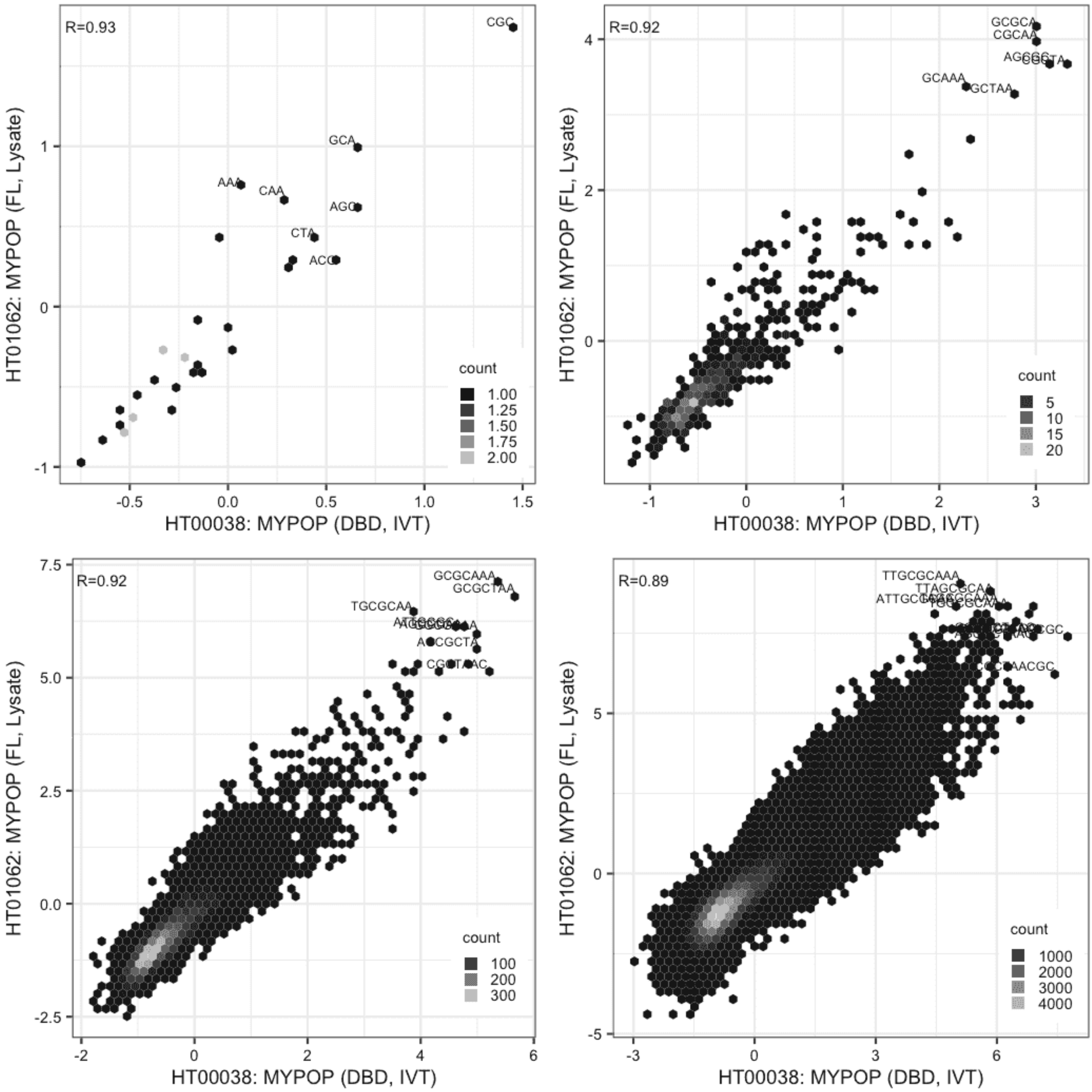

MYPOP

Lysate vs IVT, different constructs

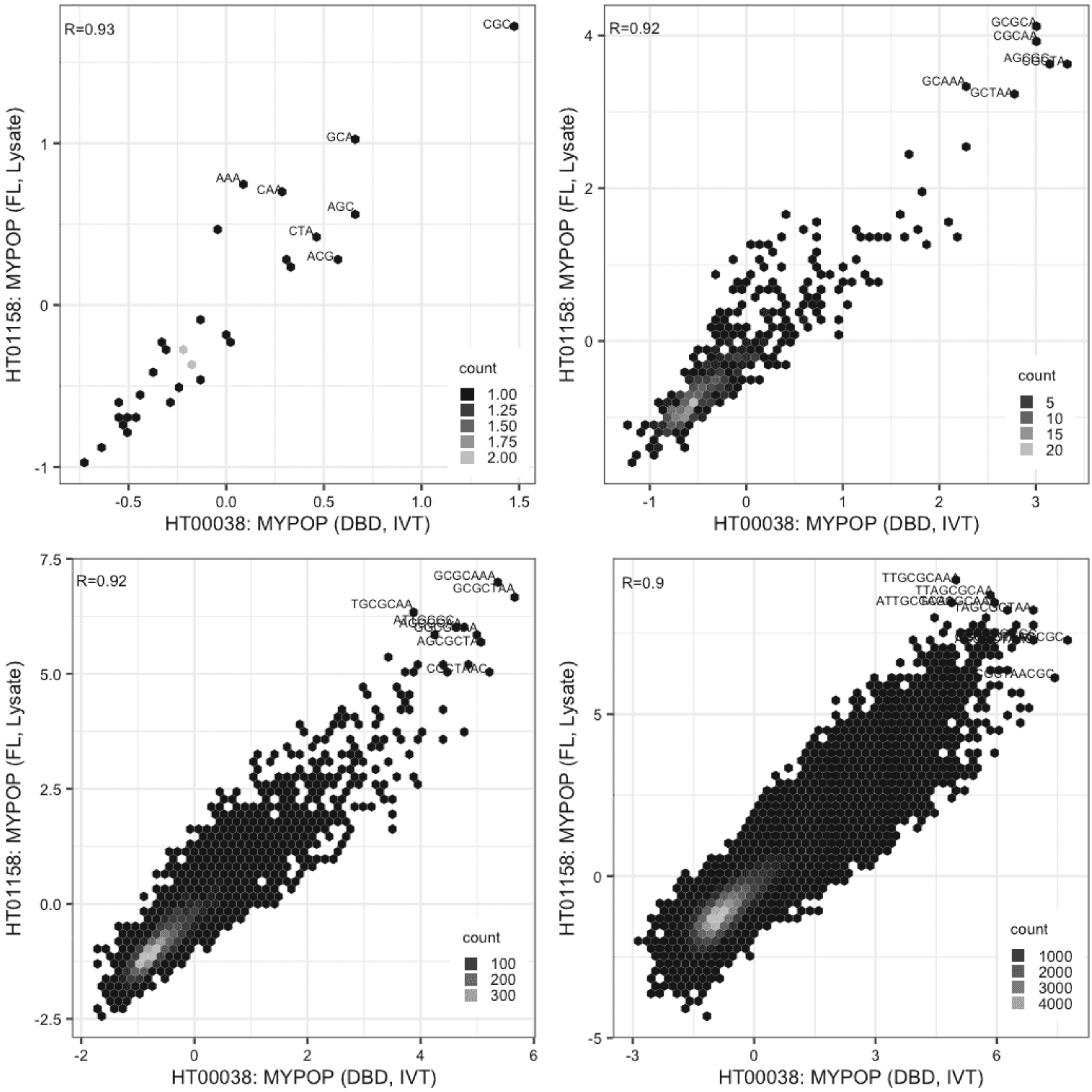

MYPOP

IVT vs GFPIVT, different constructs

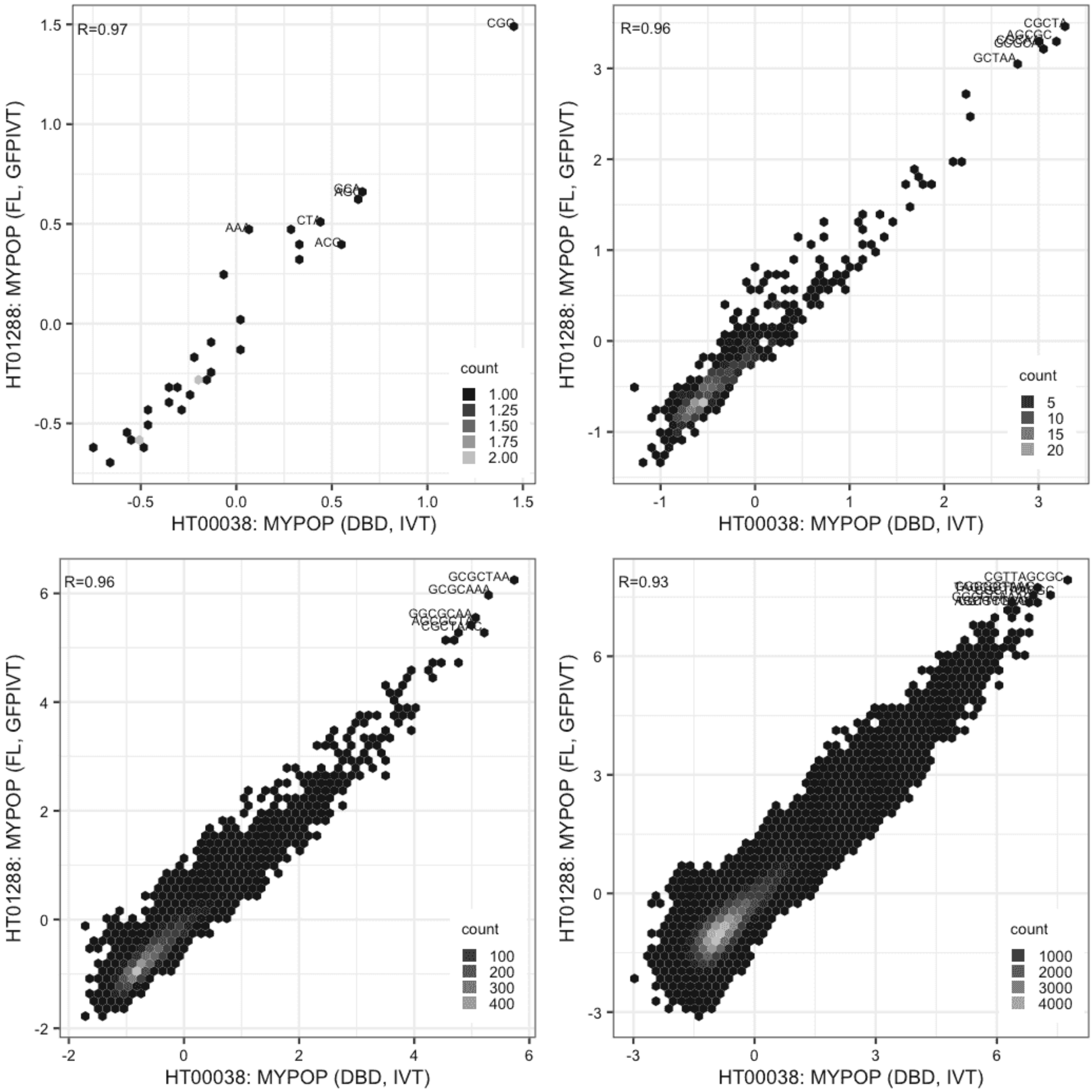

MYPOP

Lysate vs IVT, same construct

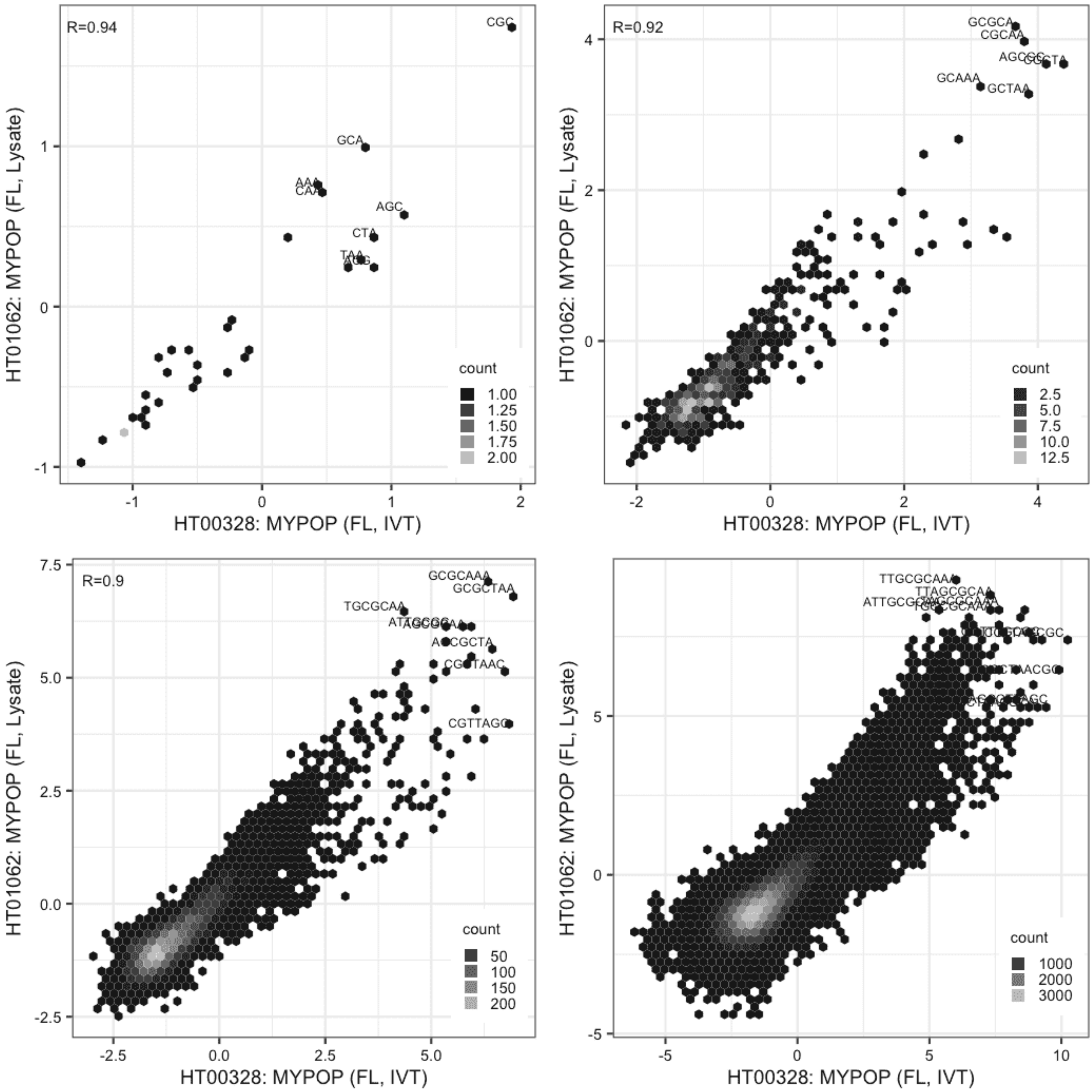

MYPOP

Lysate vs IVT, same construct

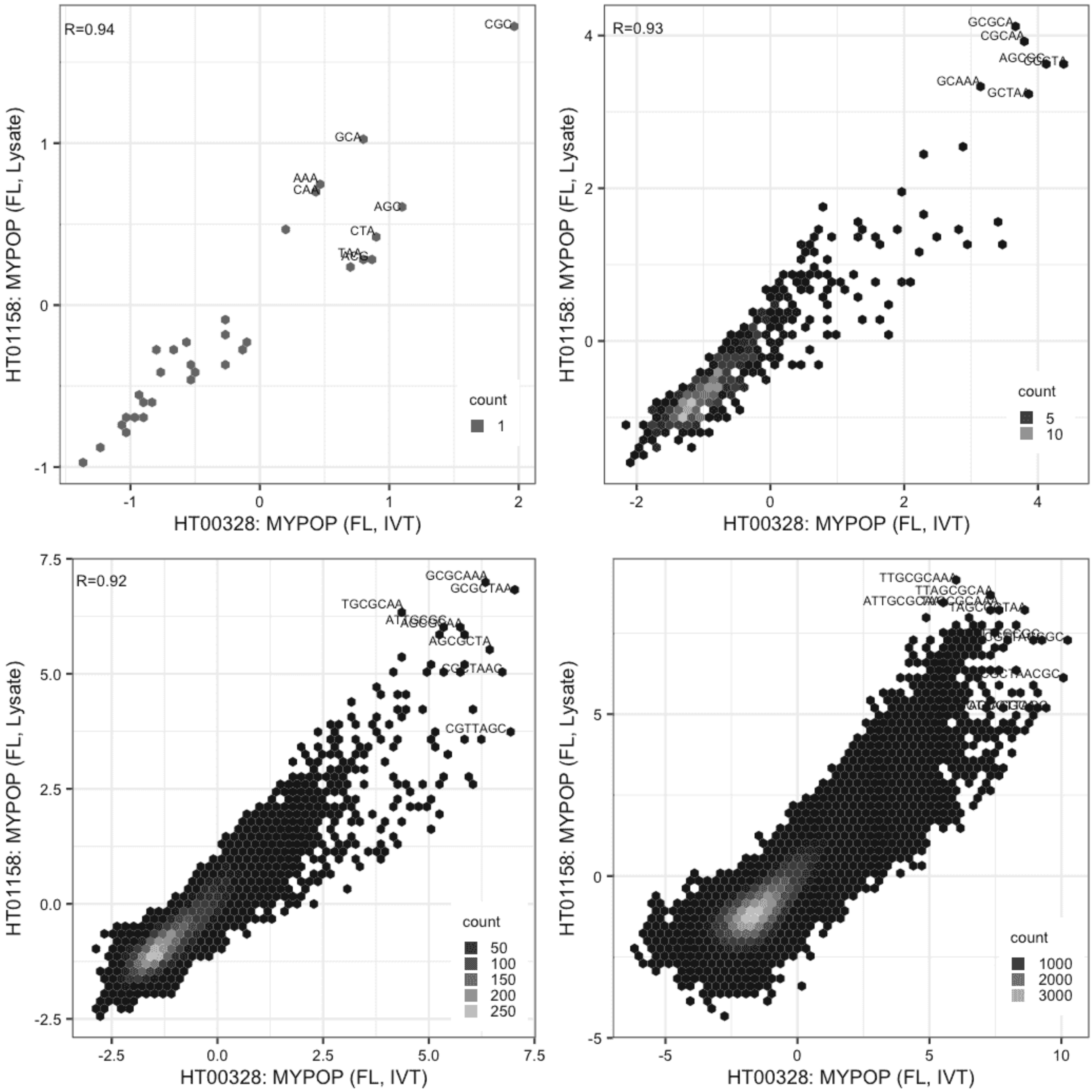

MYPOP

IVT vs GFPIVT, same construct

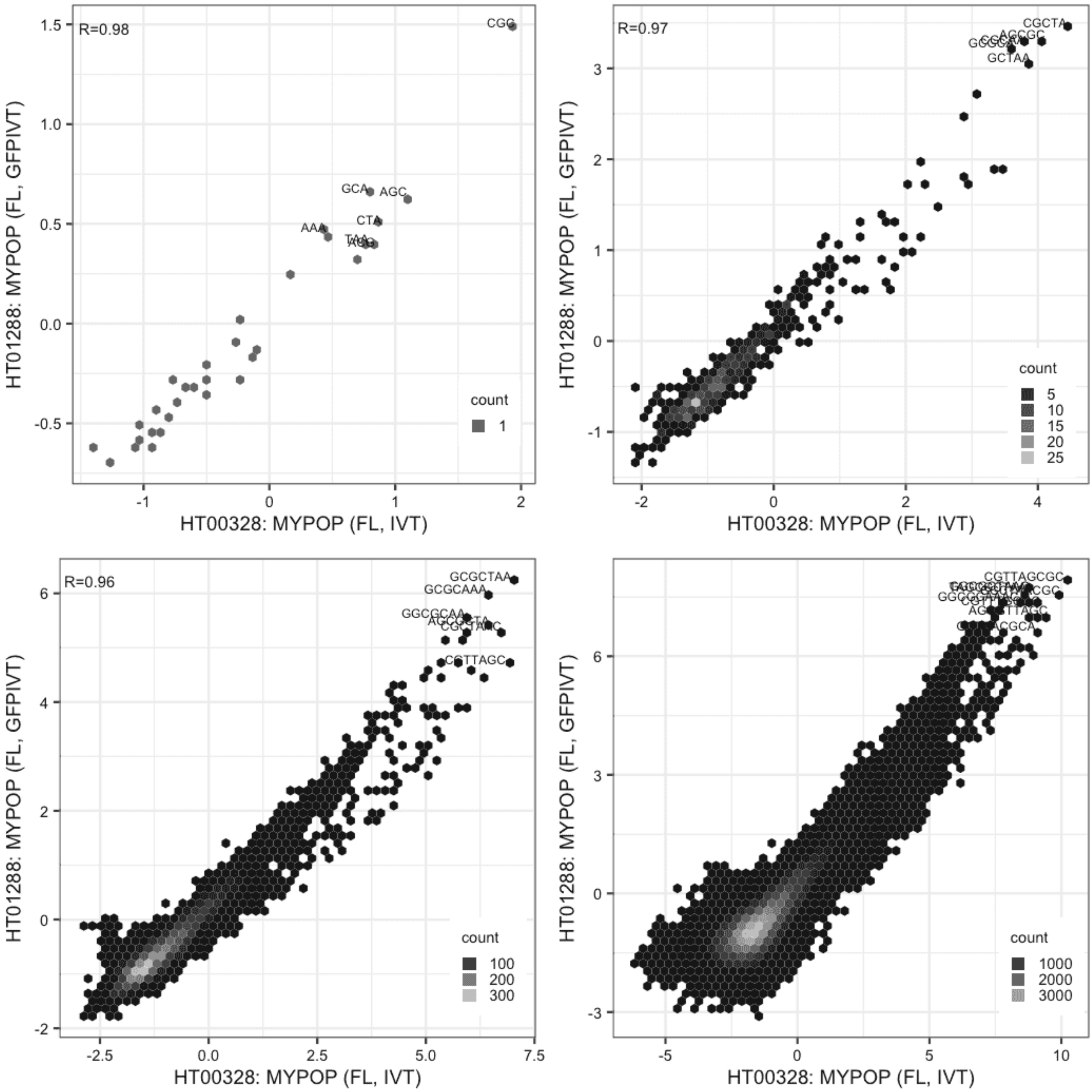

MYPOP

Lysate vs Lysate, same construct

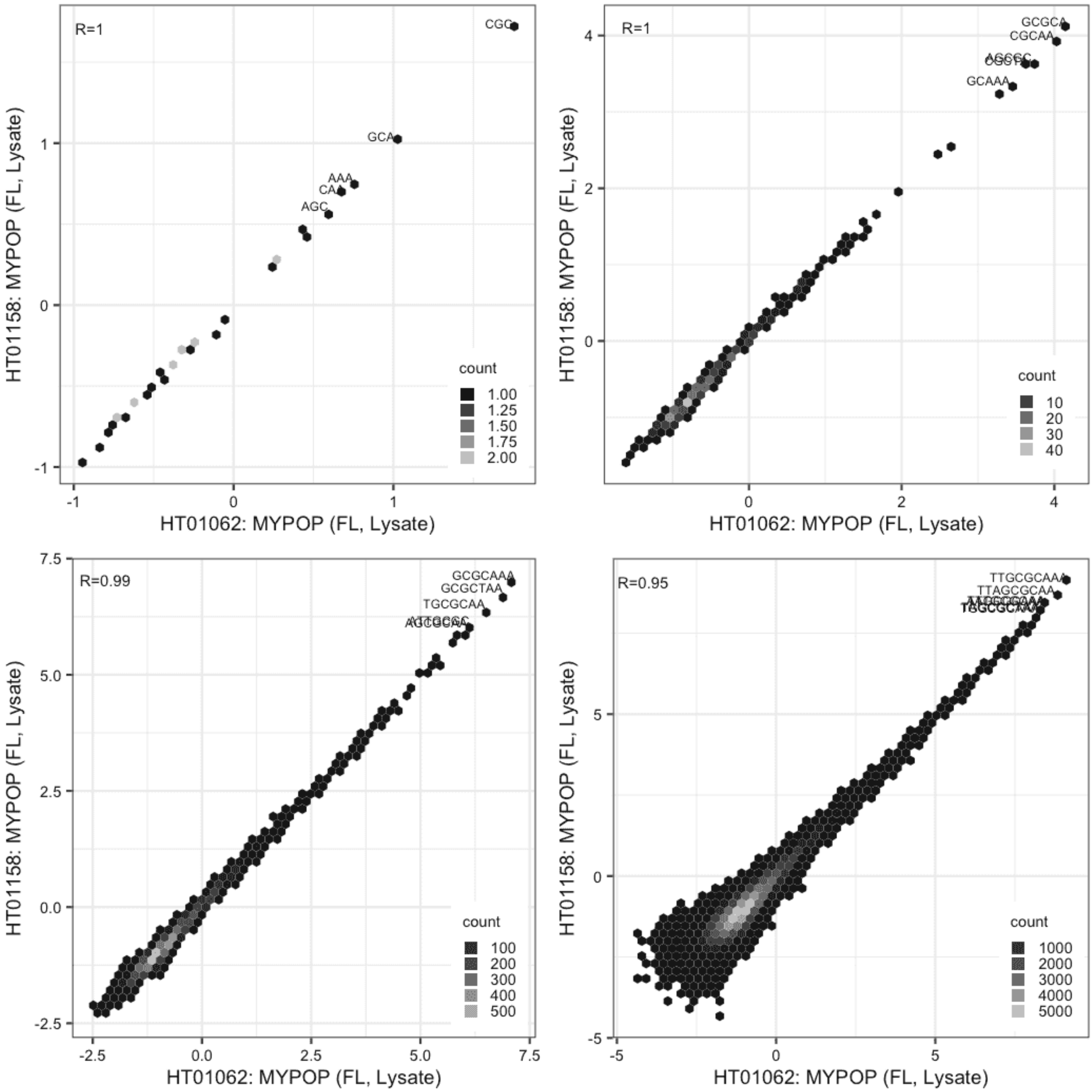

MYPOP

Lysate vs GFPIVT, same construct

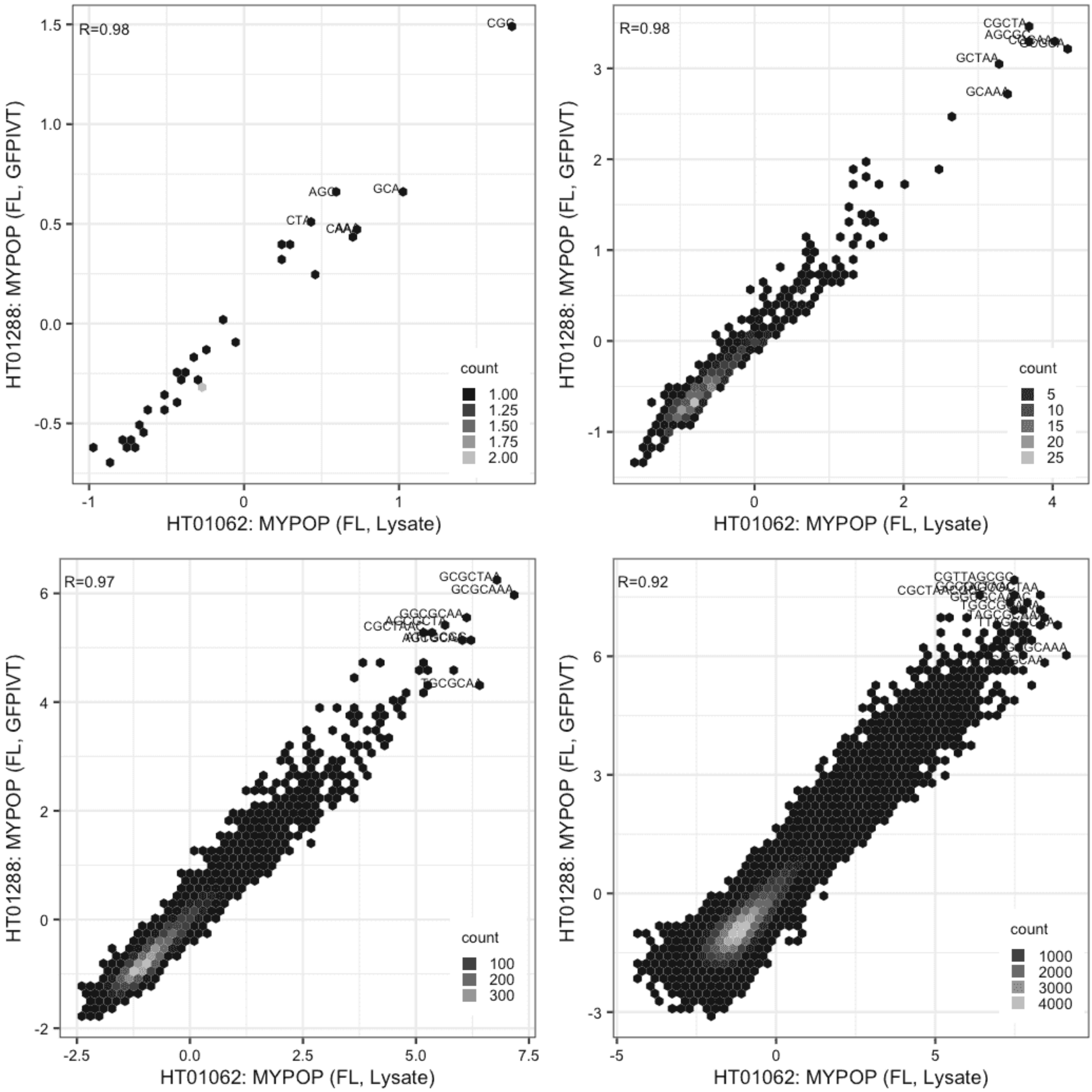

MYPOP

Lysate vs GFPIVT, same construct

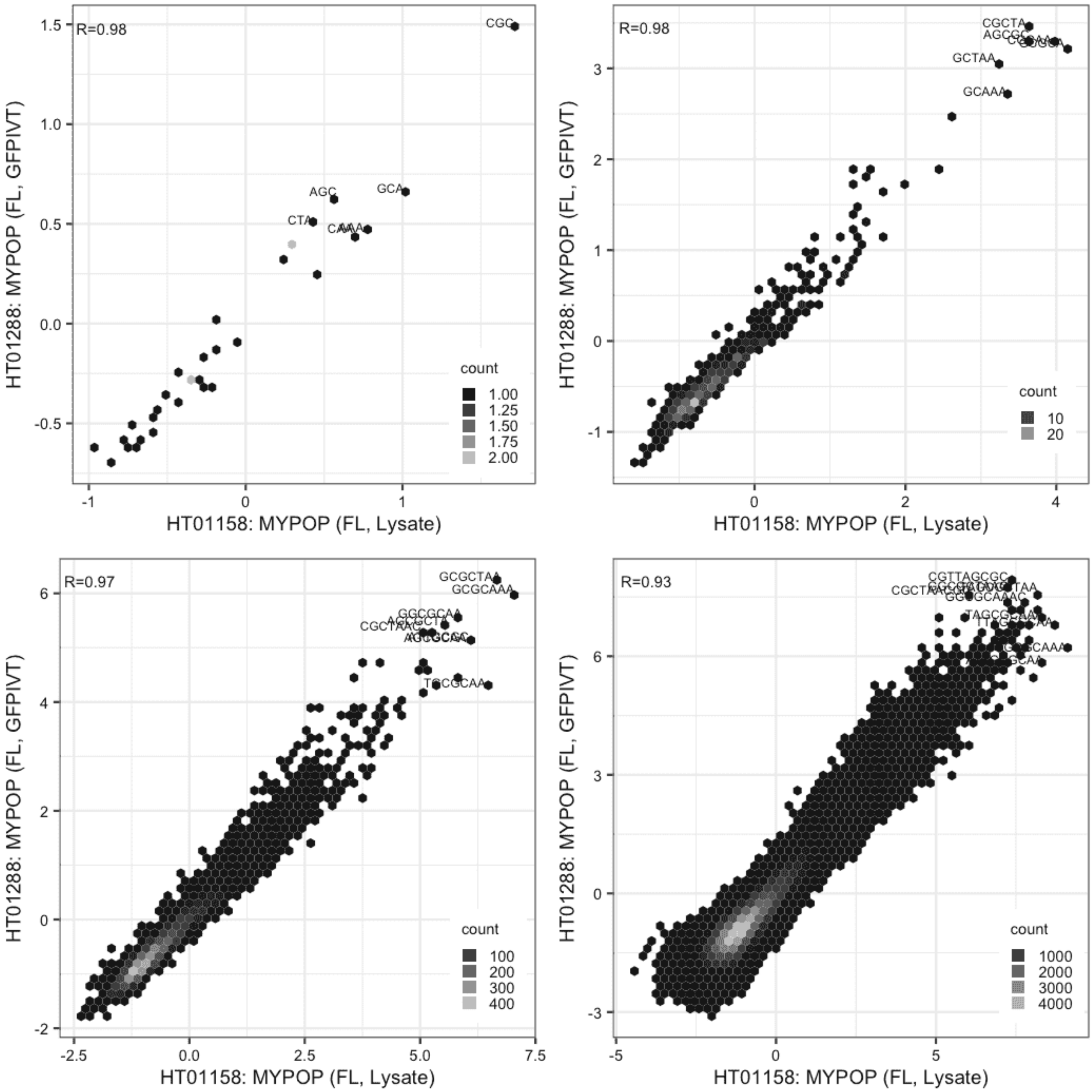

NACC2

Lysate vs IVT, different constructs

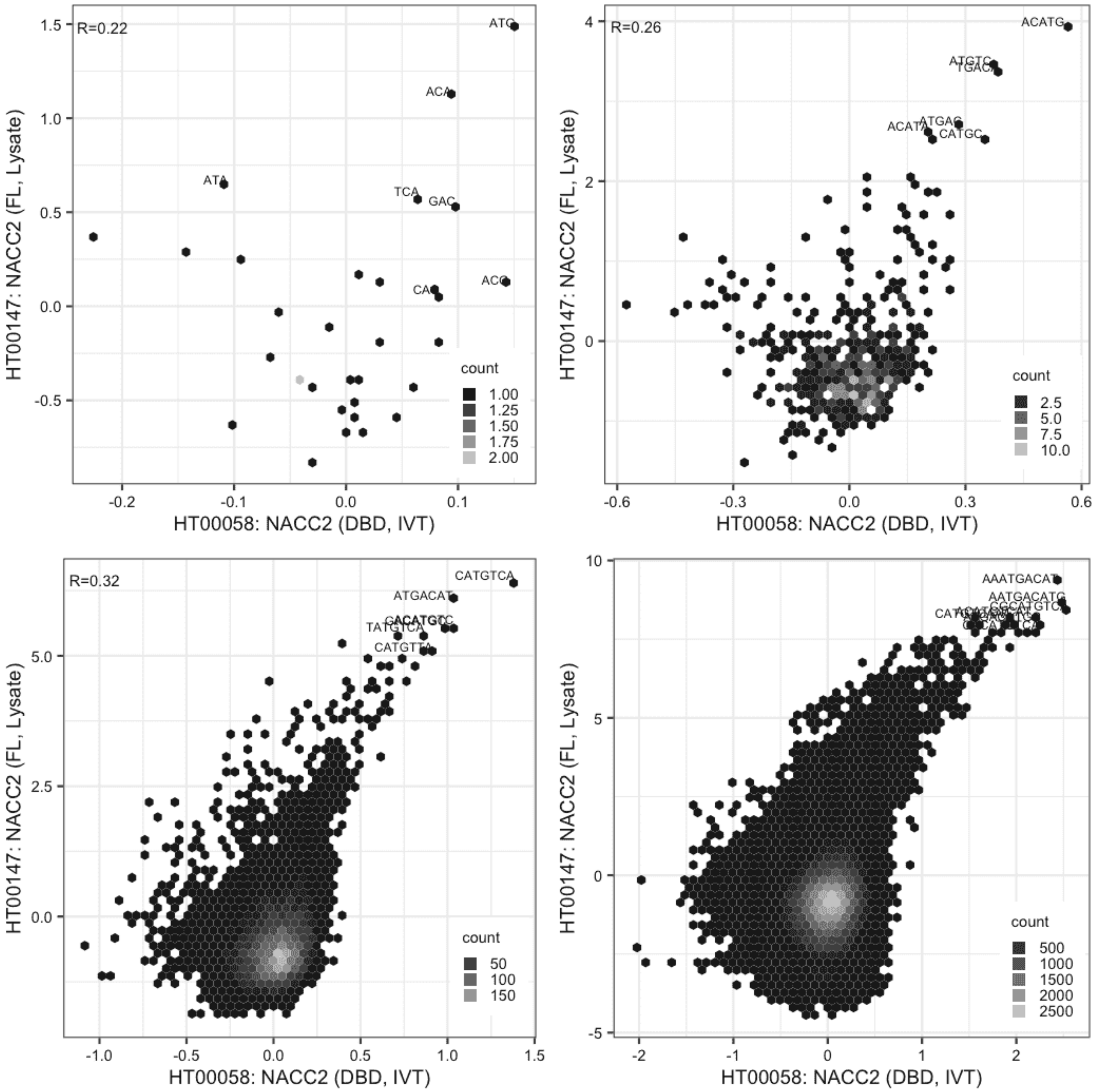

NACC2

IVT vs IVT, different constructs

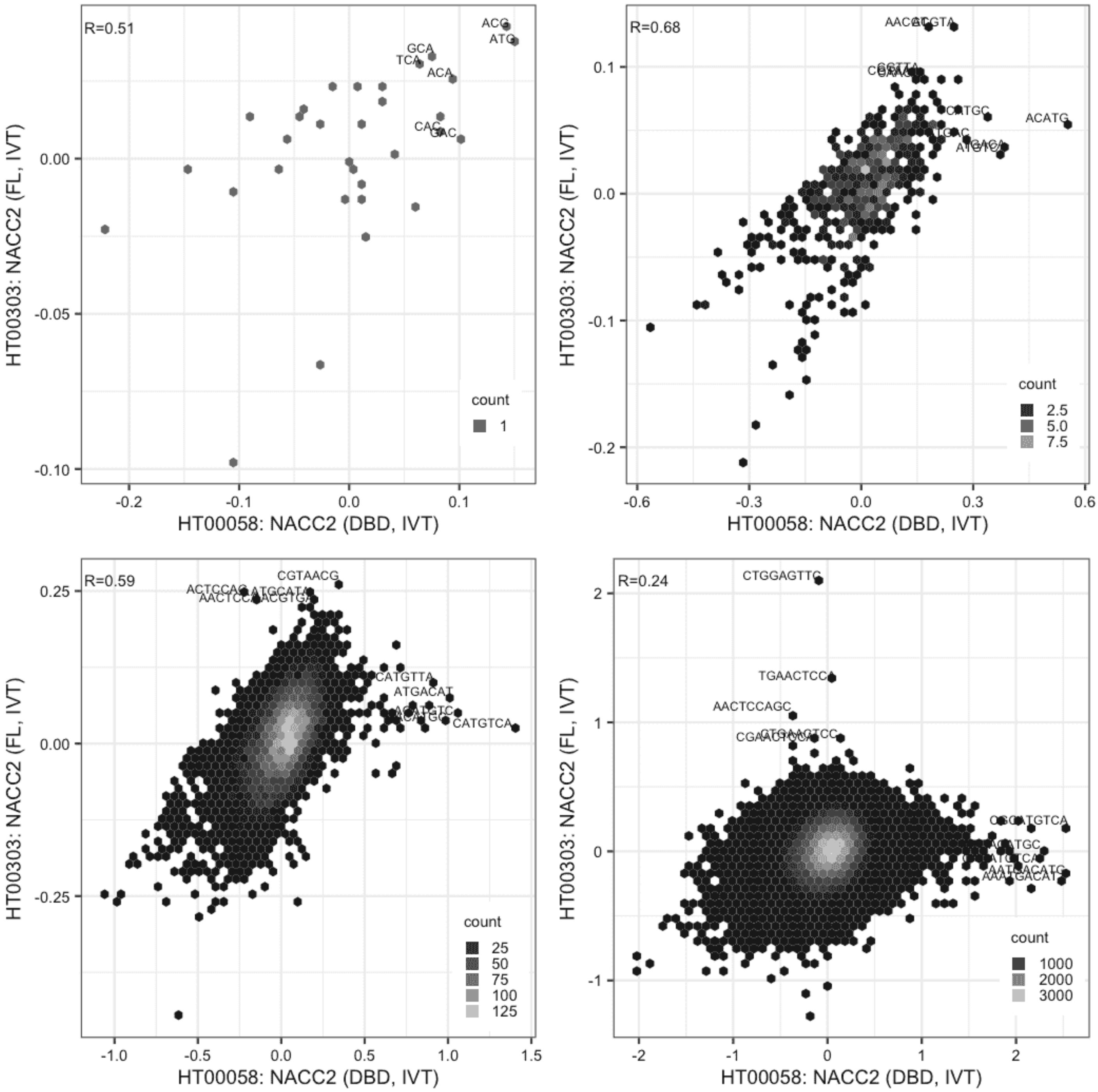

NACC2

IVT vs GFPIVT, same construct

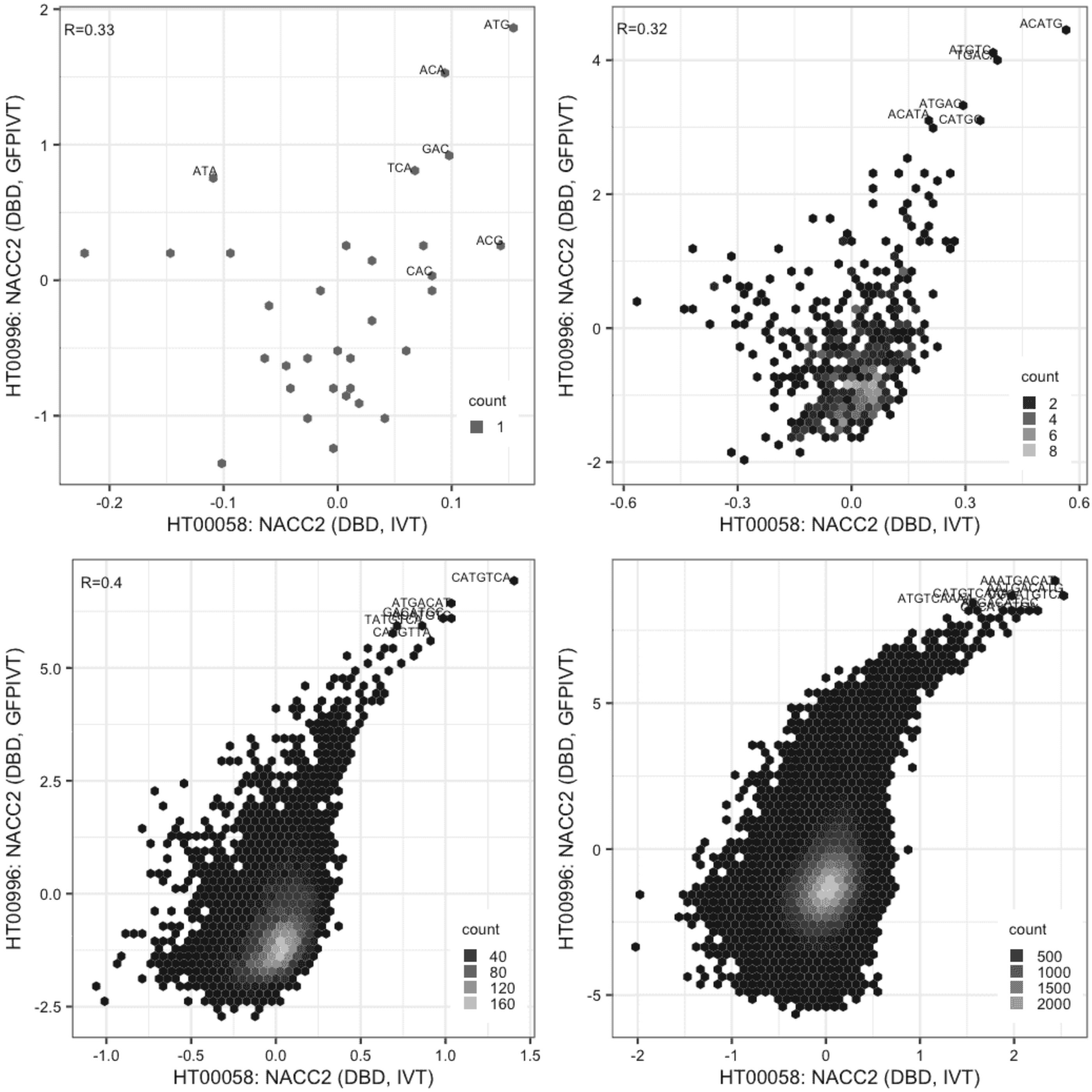

NACC2

Lysate vs IVT, same construct

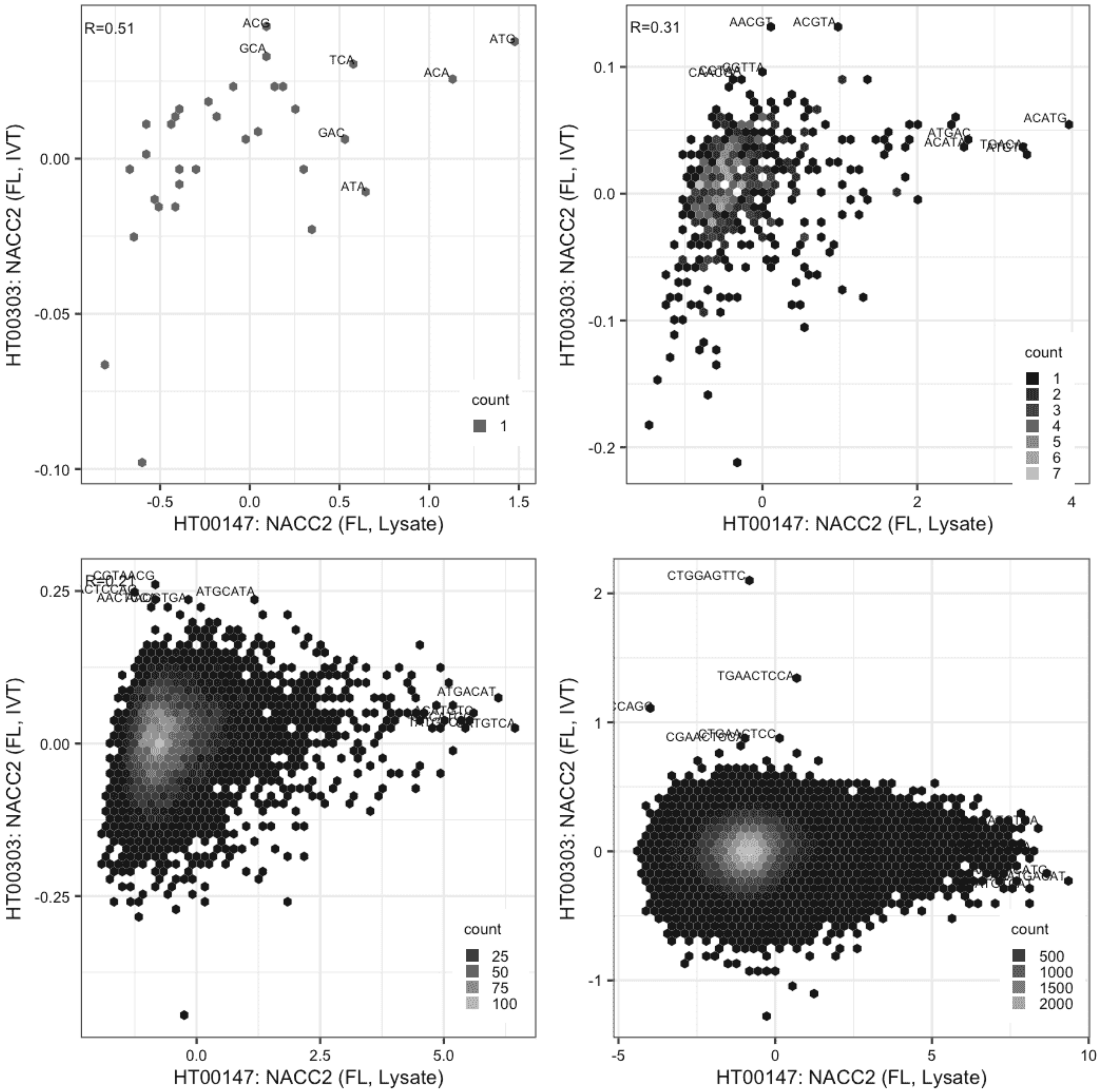

NACC2

Lysate vs GFPIVT, different constructs

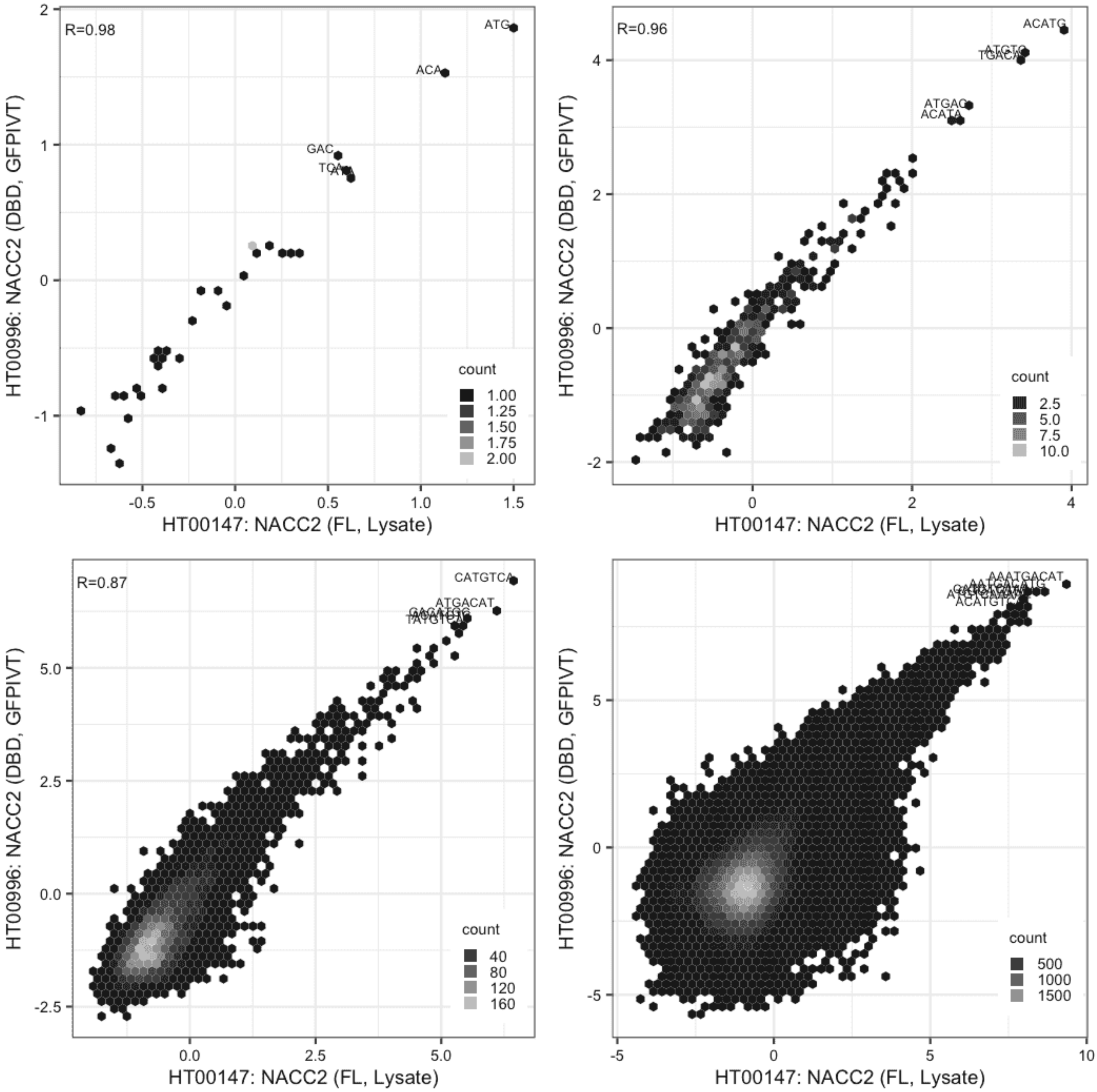

NACC2

IVT vs GFPIVT, different constructs

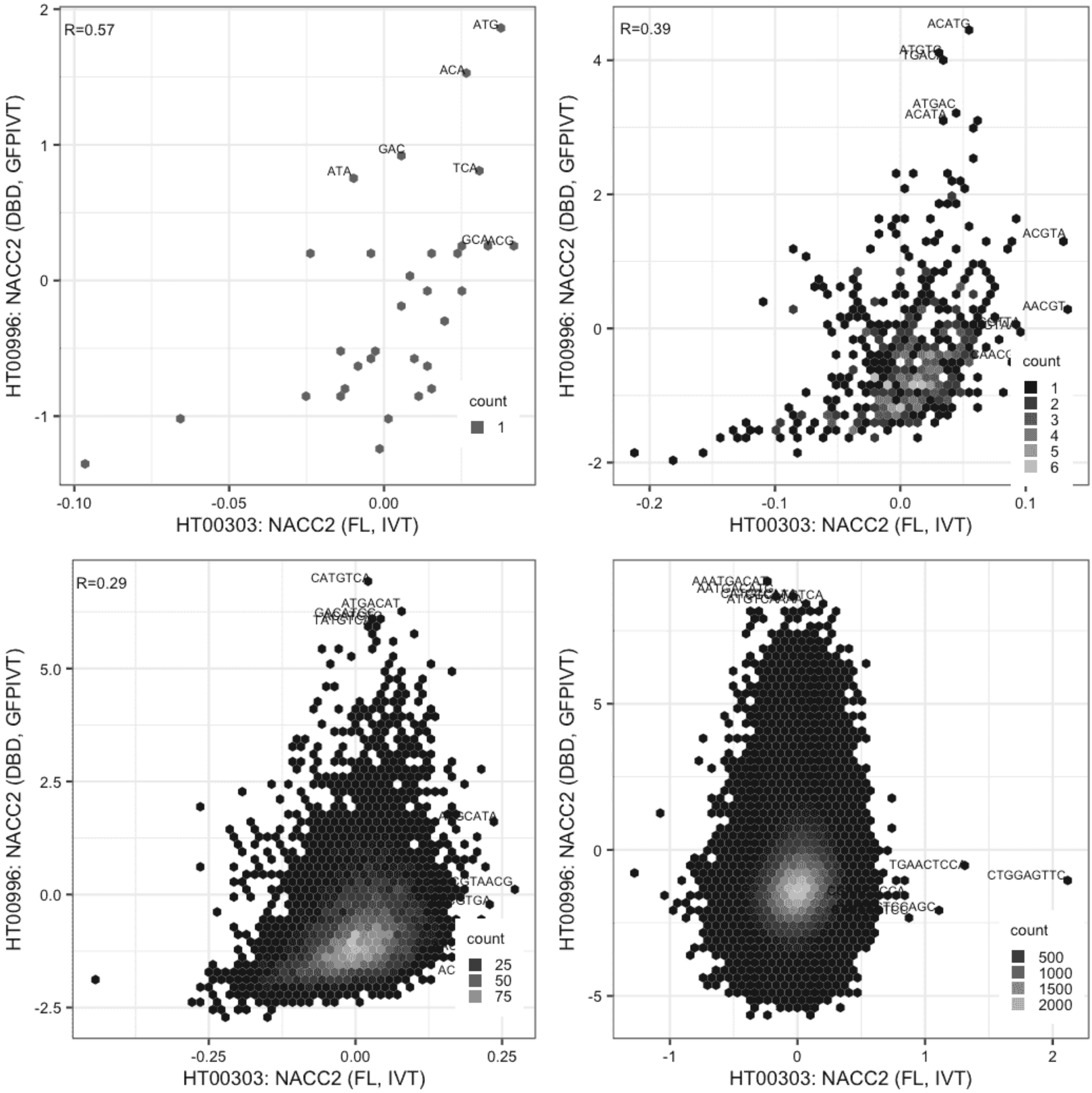

NFKB1

Lysate vs IVT, same construct

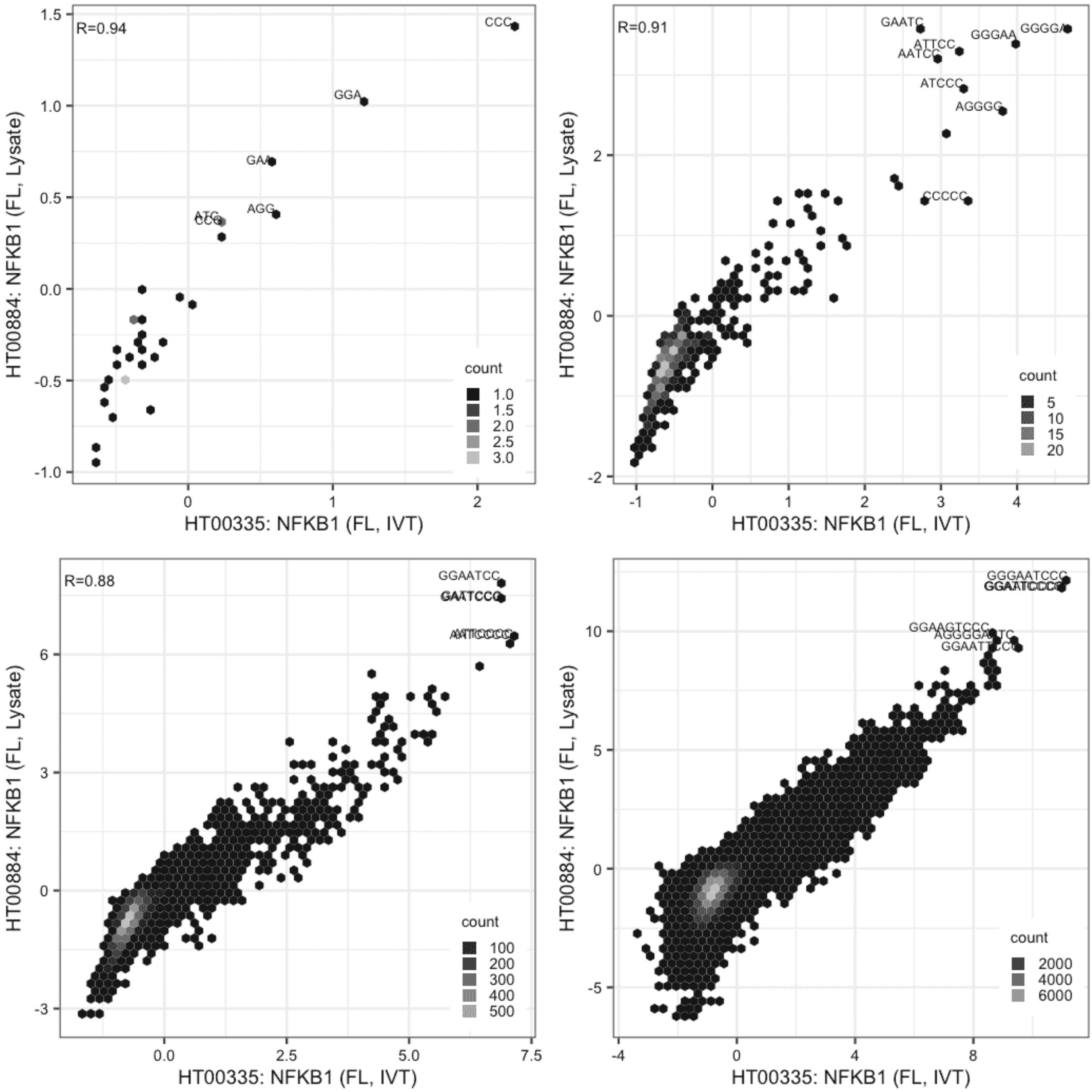

NR1H4

Lysate vs IVT, same construct

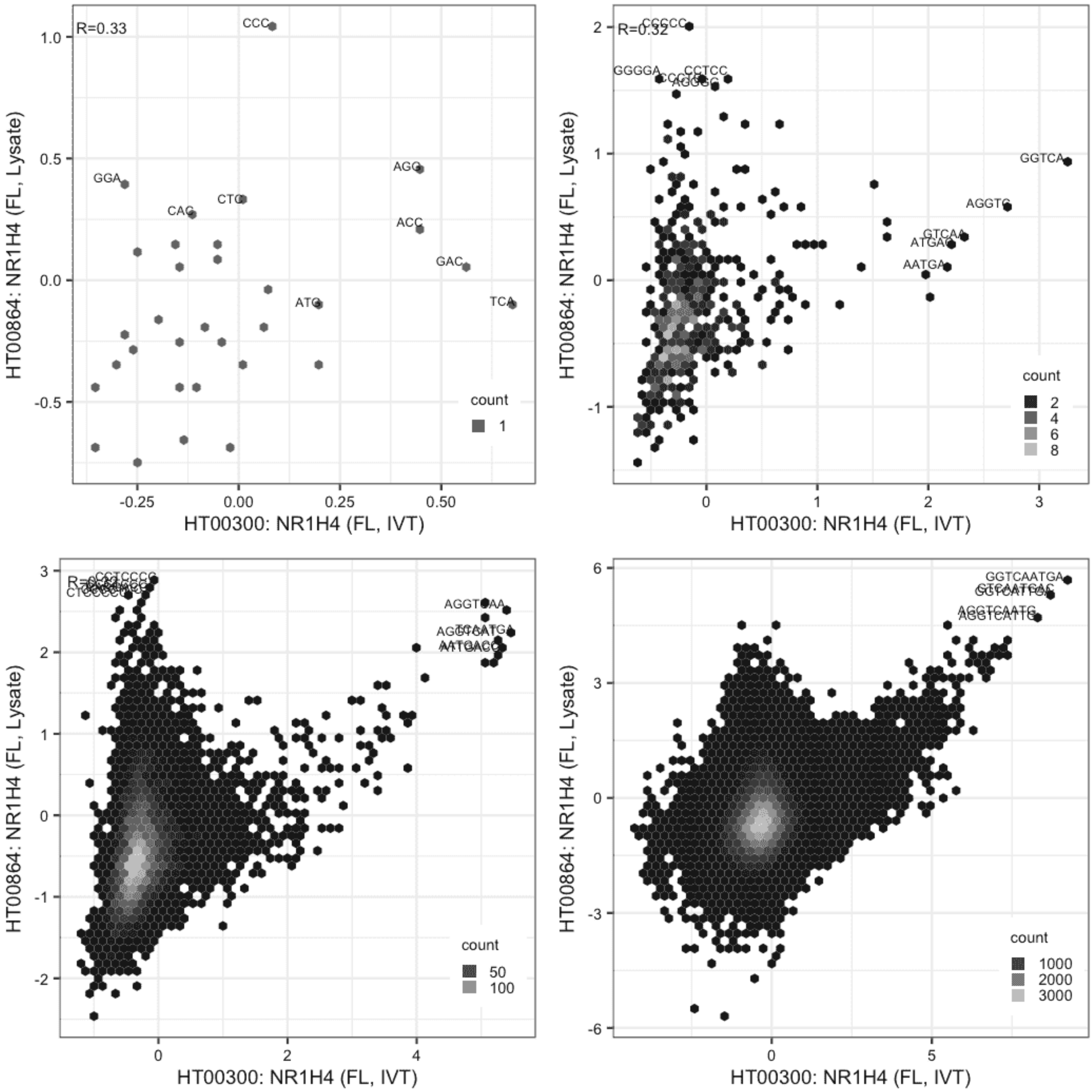

PAX7

Lysate vs IVT, same construct

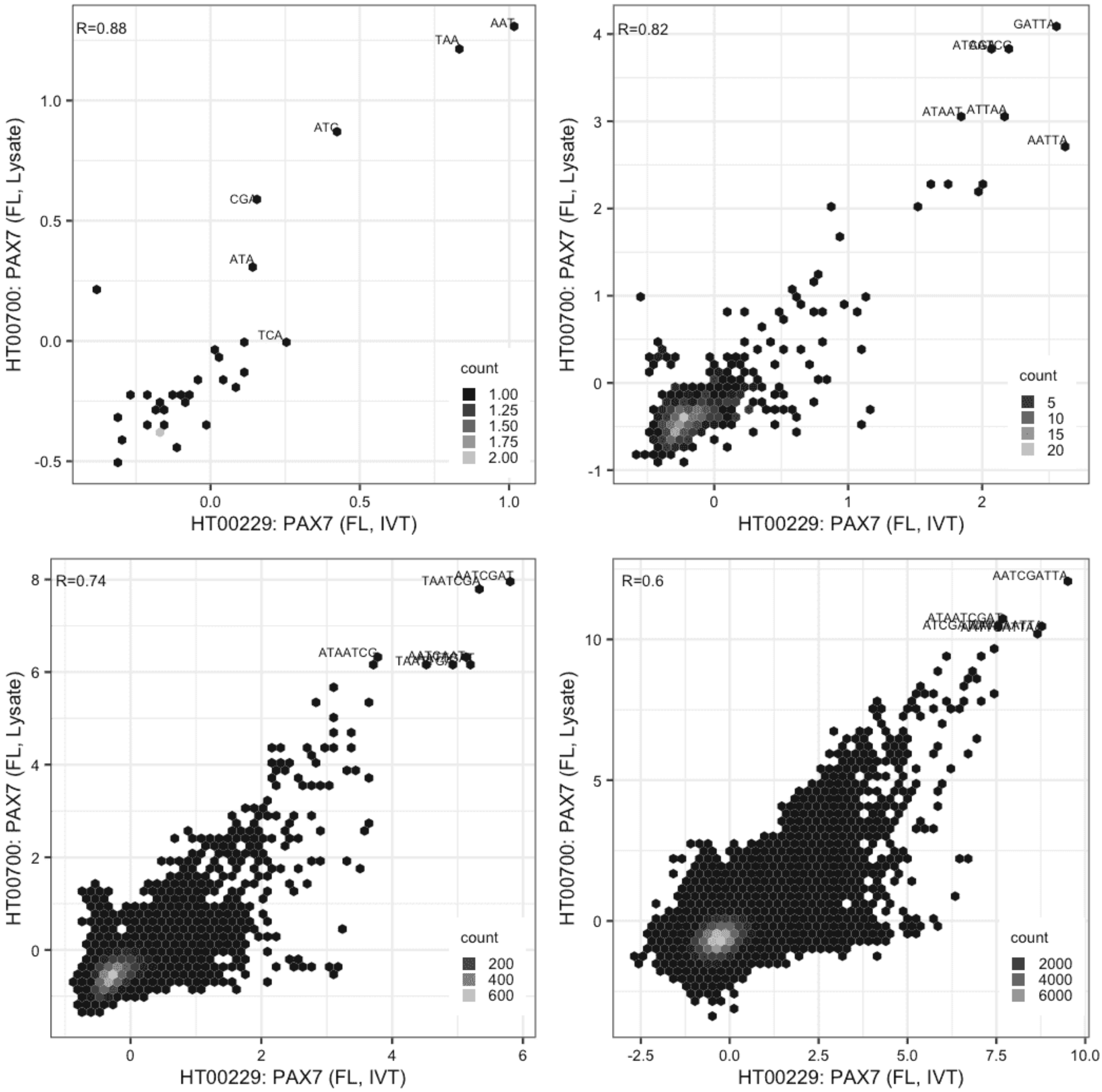

PAX7

Lysate vs IVT, same construct

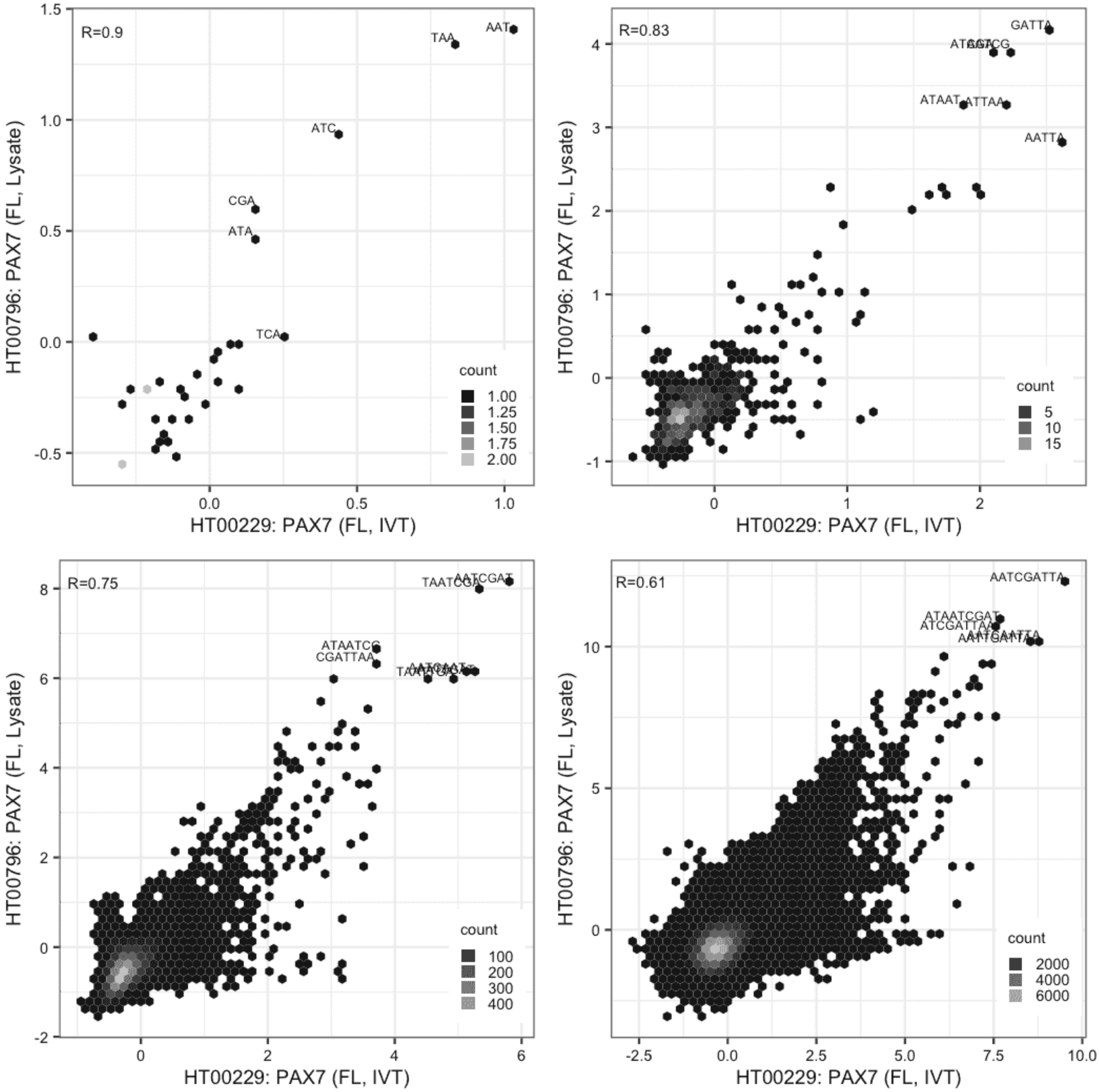

PAX7

Lysate vs Lysate, same construct

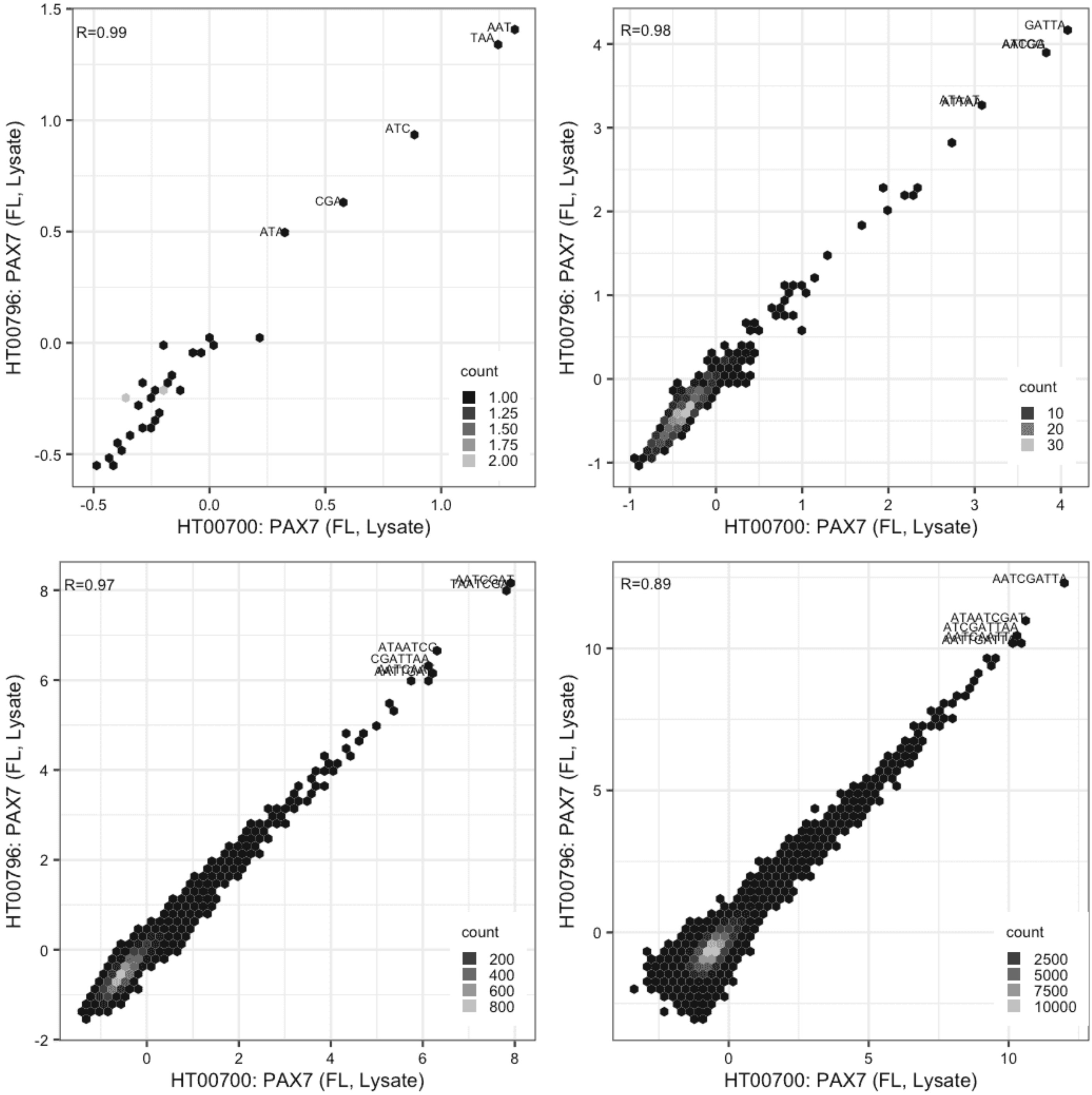

POGK

Lysate vs IVT, different constructs

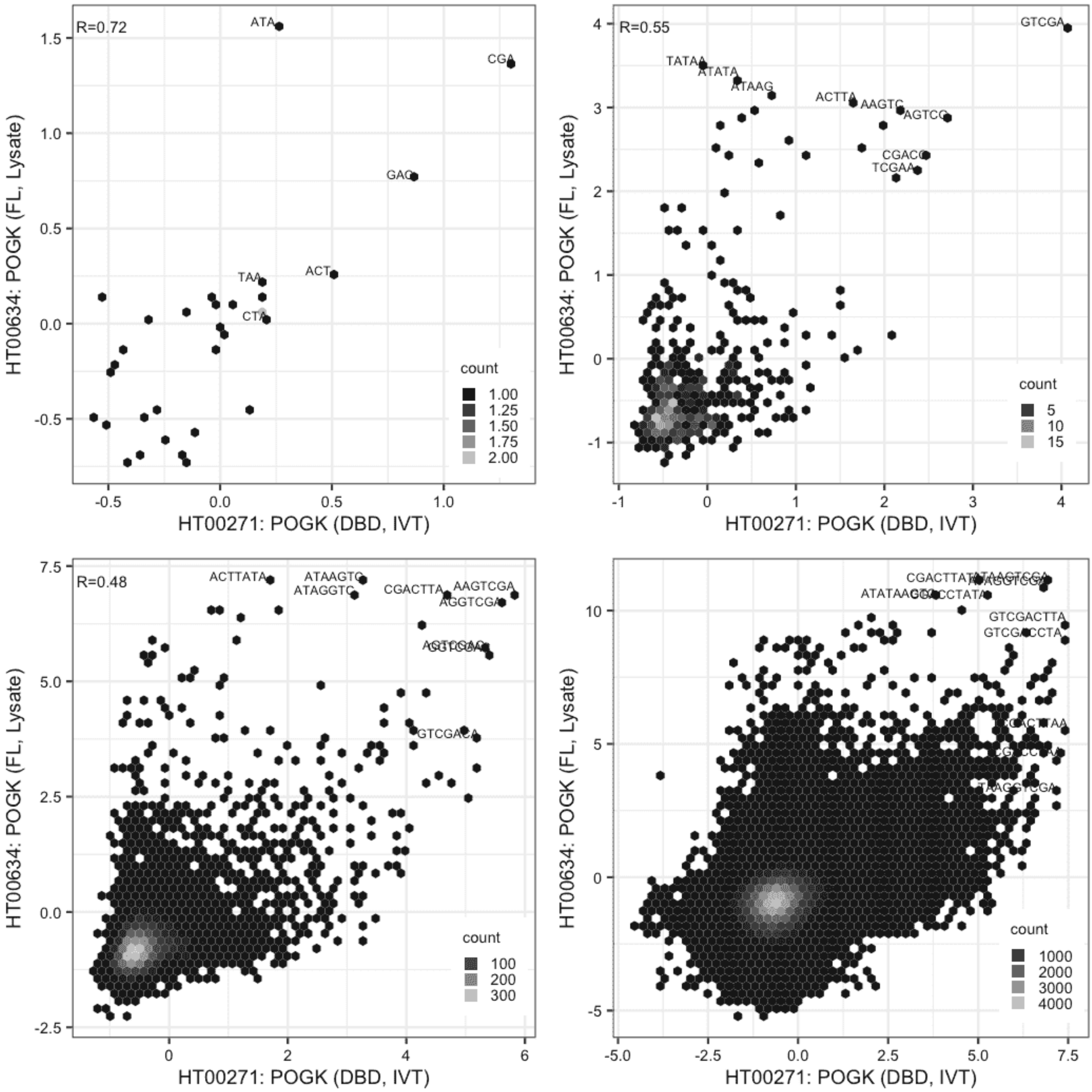

PRDM10

Lysate vs Lysate, same construct

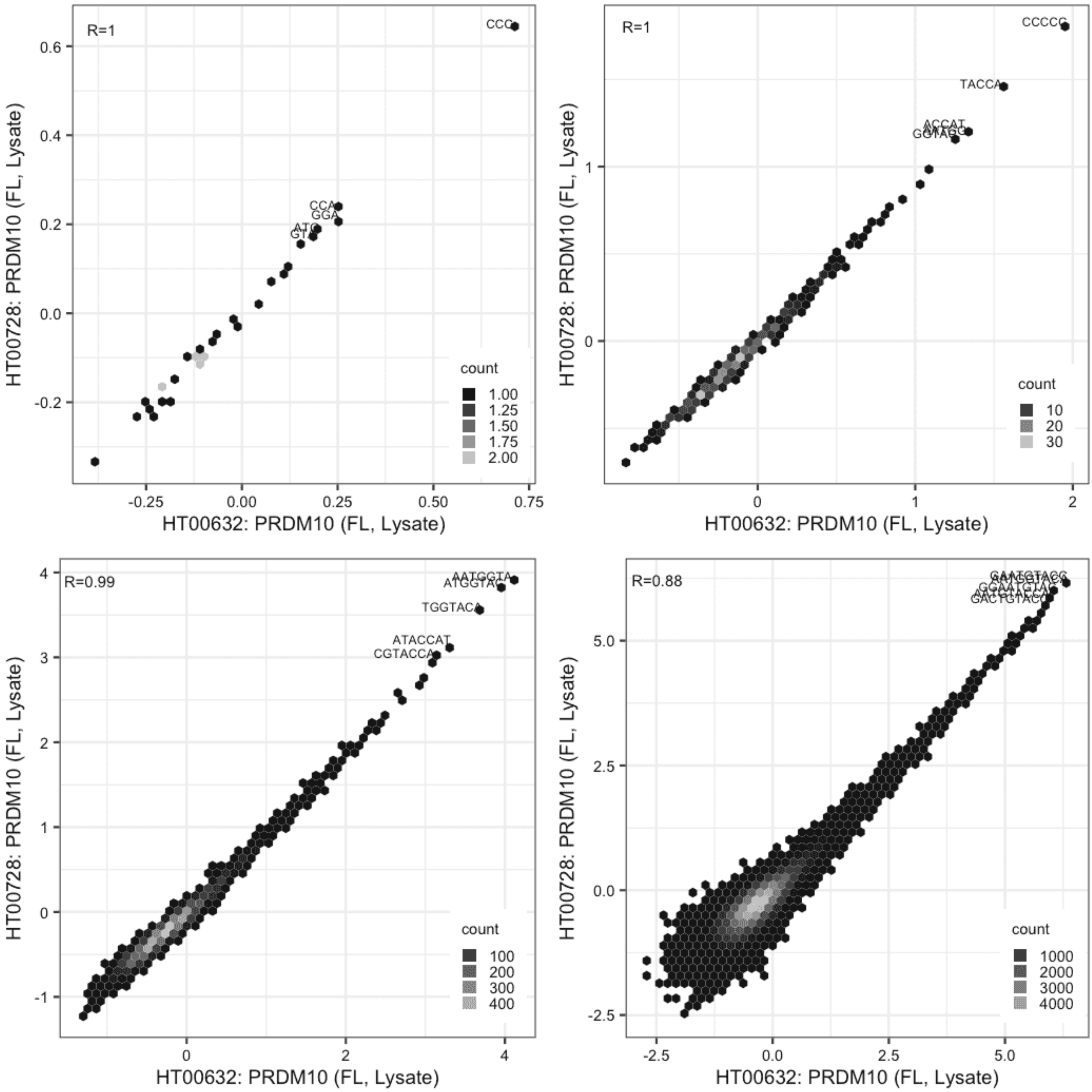

PRDM10

Lysate vs GFPIVT, different constructs

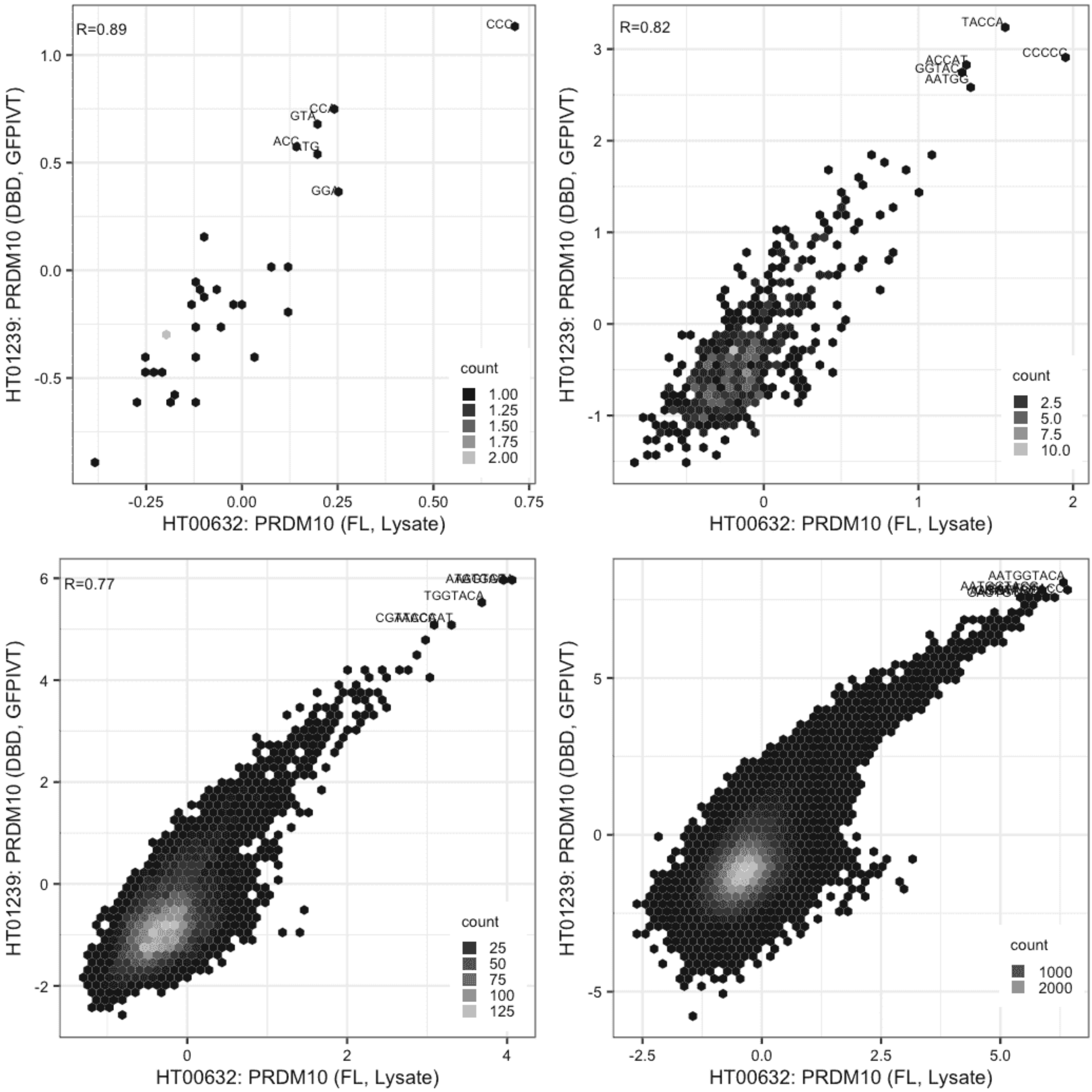

PRDM10

Lysate vs GFPIVT, different constructs

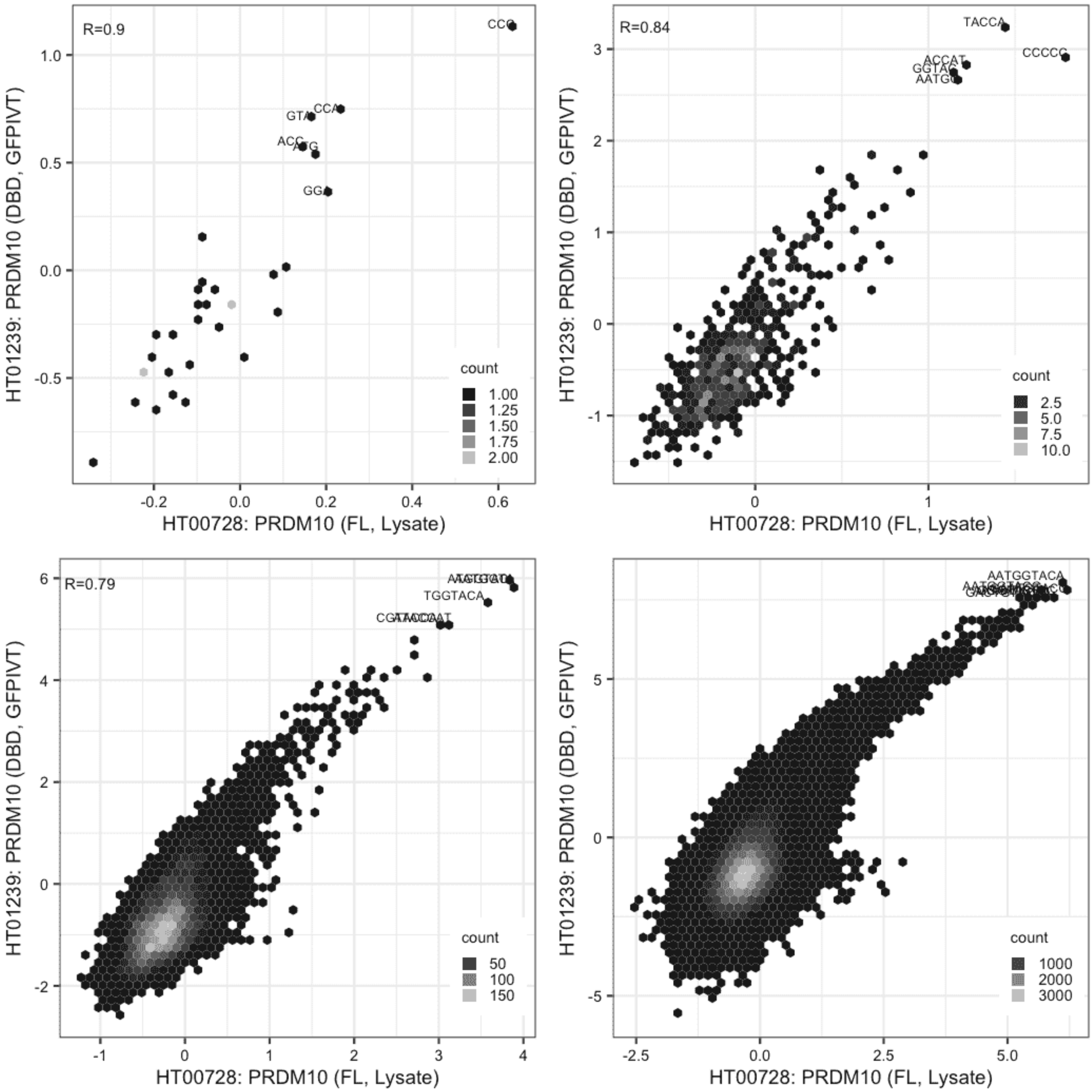

PRDM13

Lysate vs IVT, different constructs

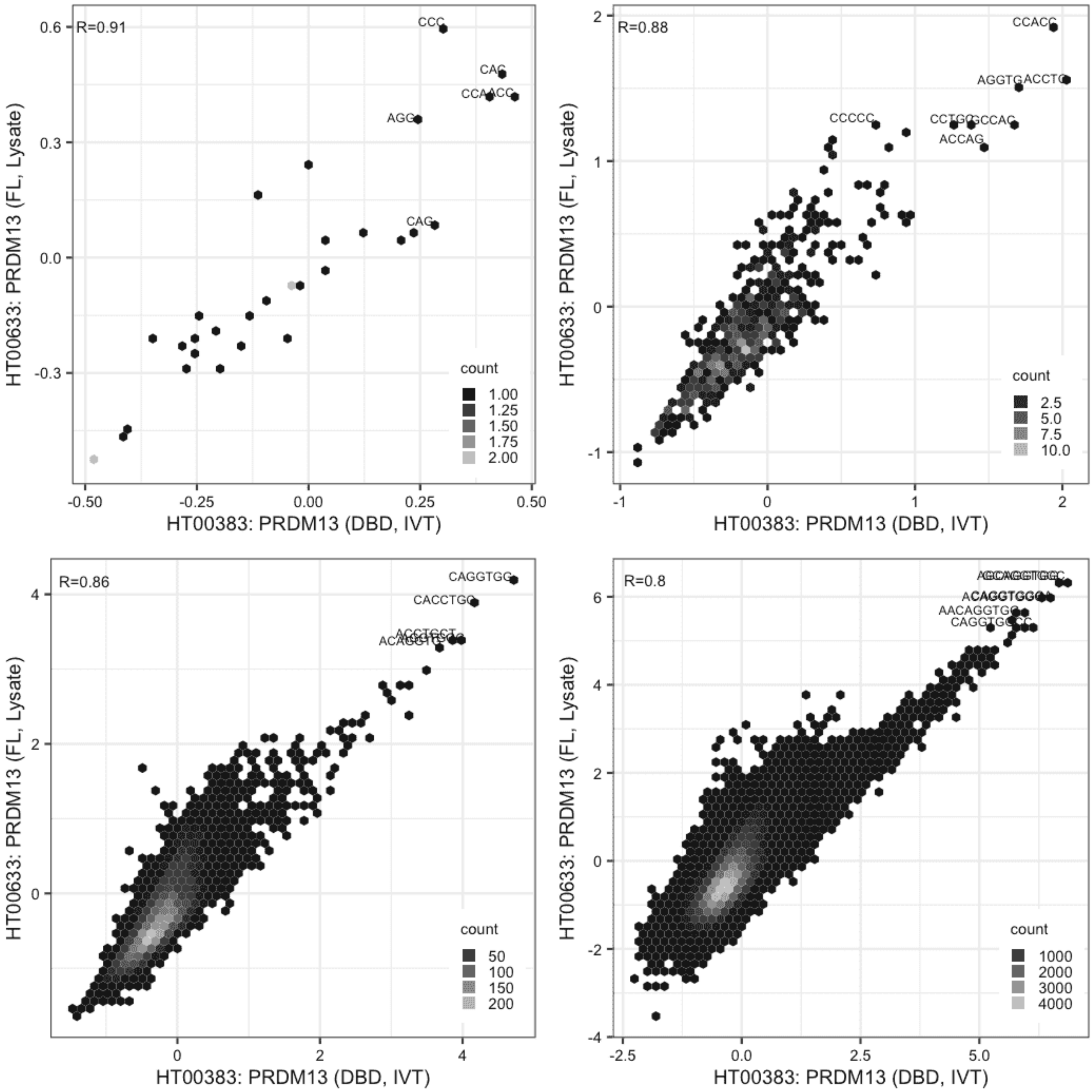

PRDM13

Lysate vs IVT, different constructs

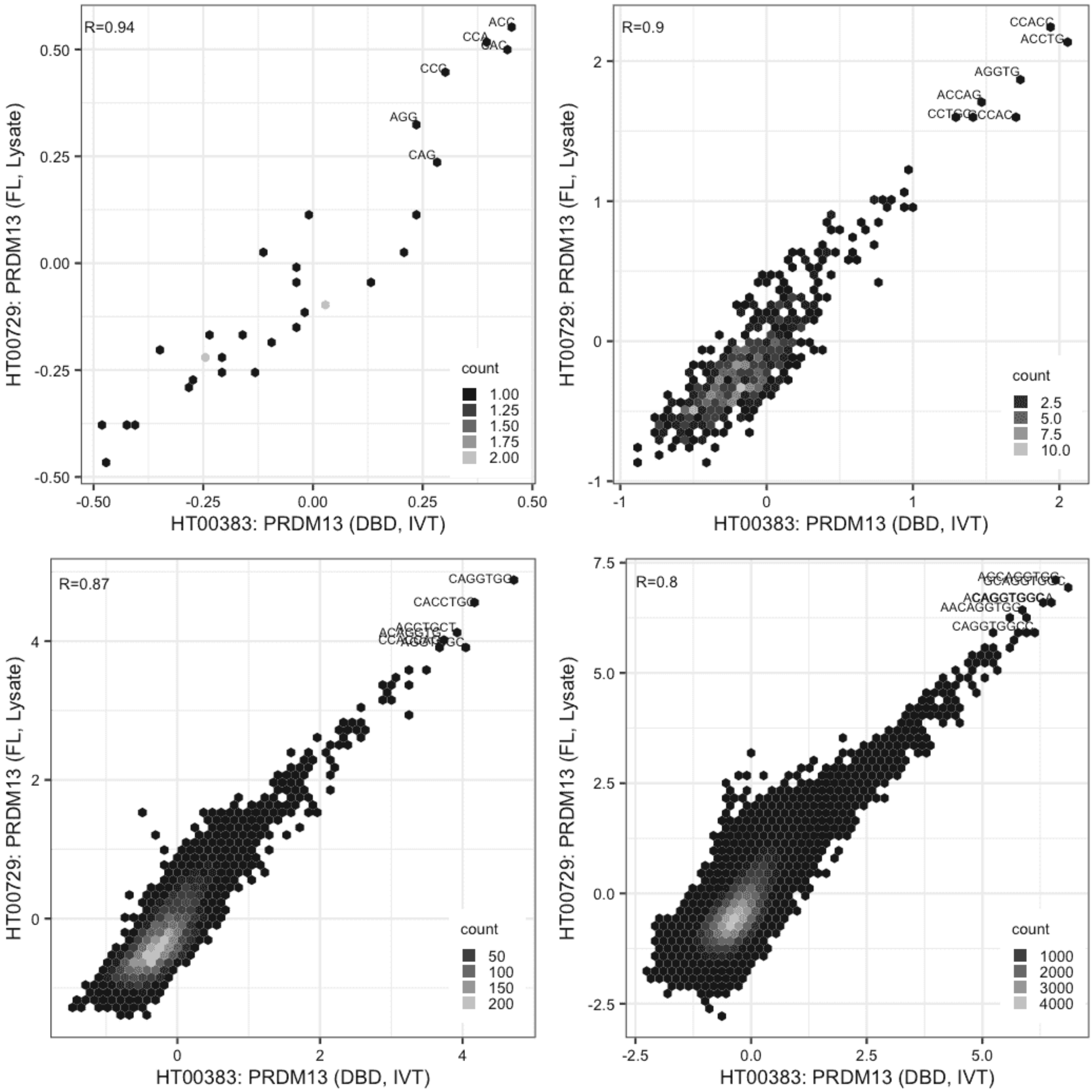

PRDM13

IVT vs GFPIVT, same construct

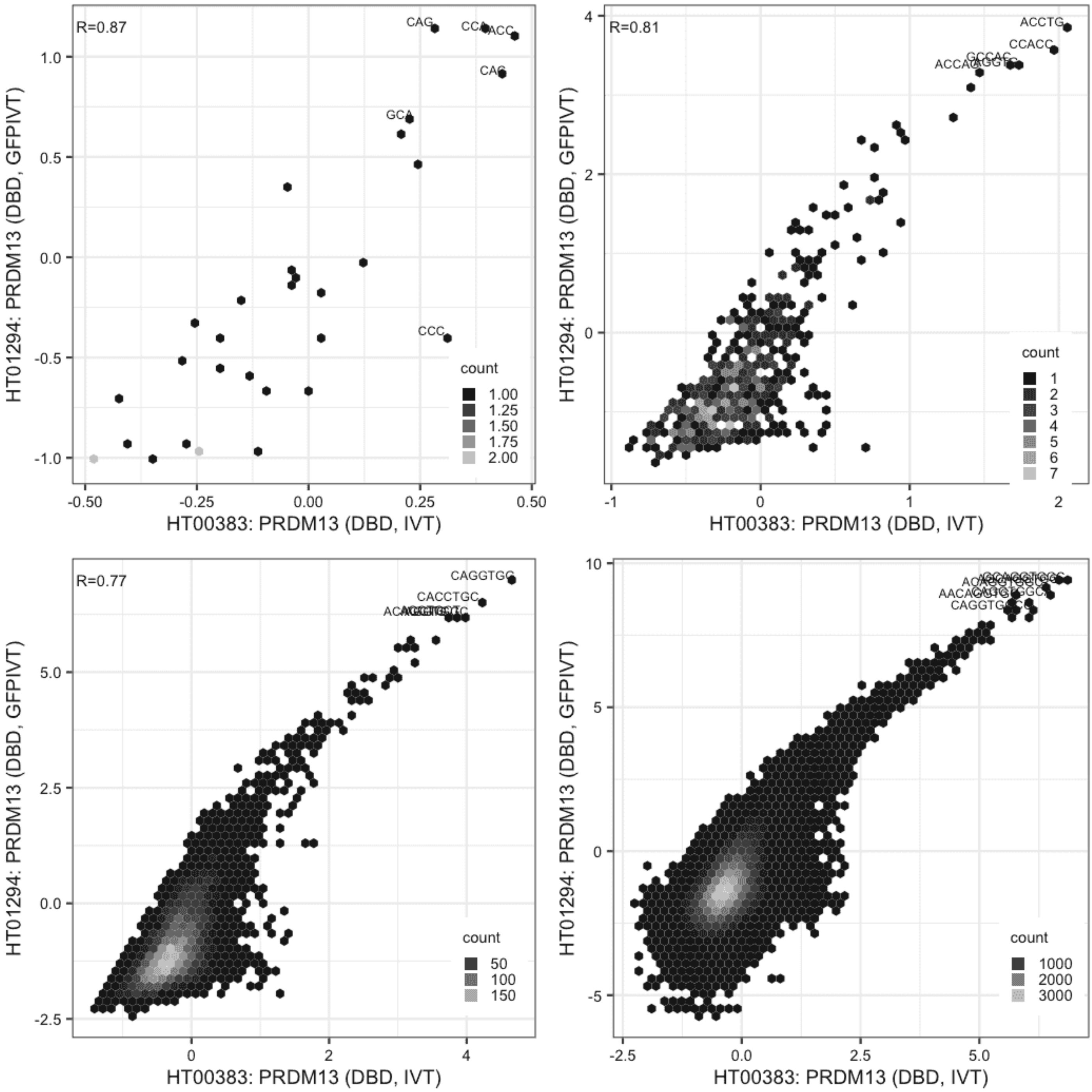

PRDM13

IVT vs GFPIVT, different constructs

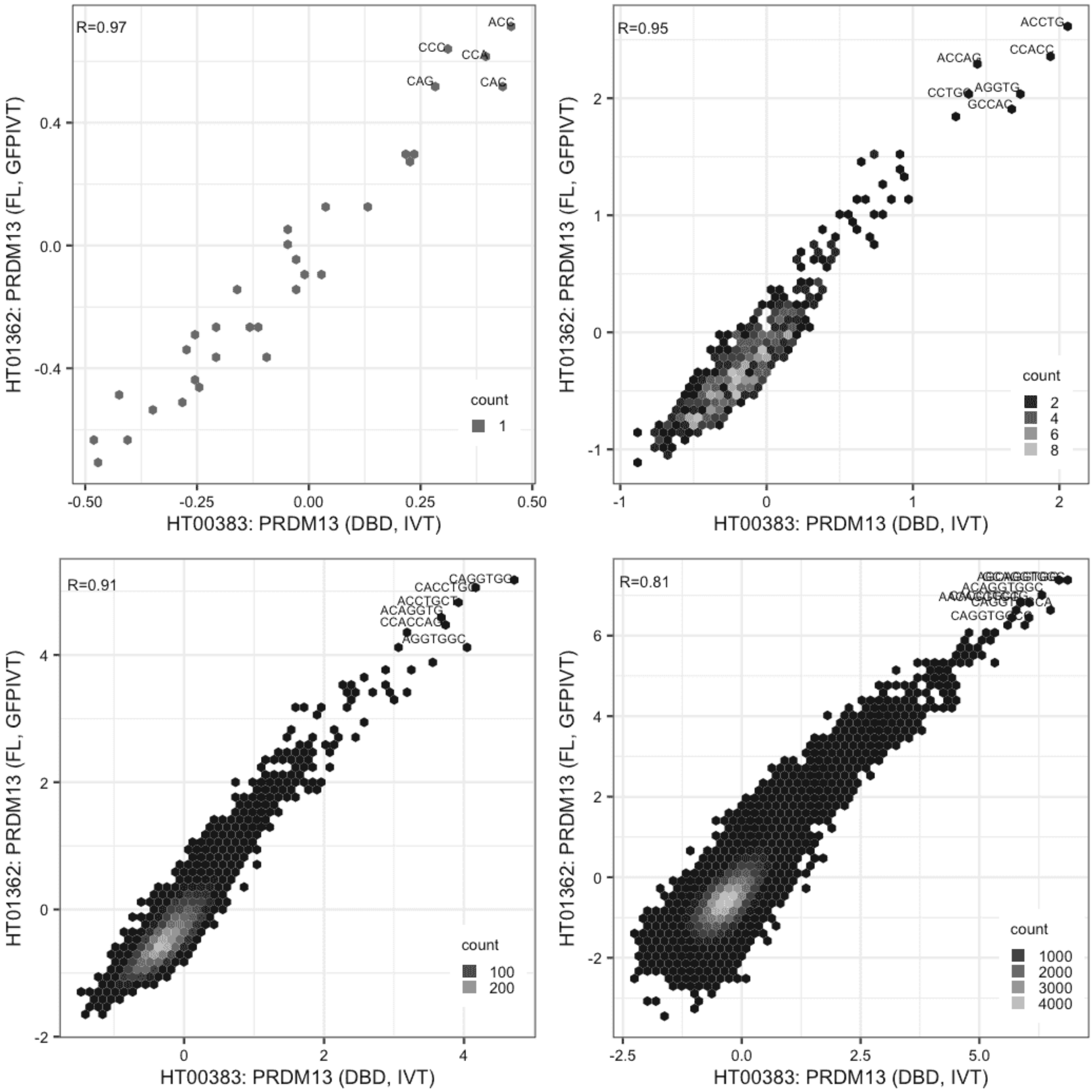

PRDM13

Lysate vs Lysate, same construct

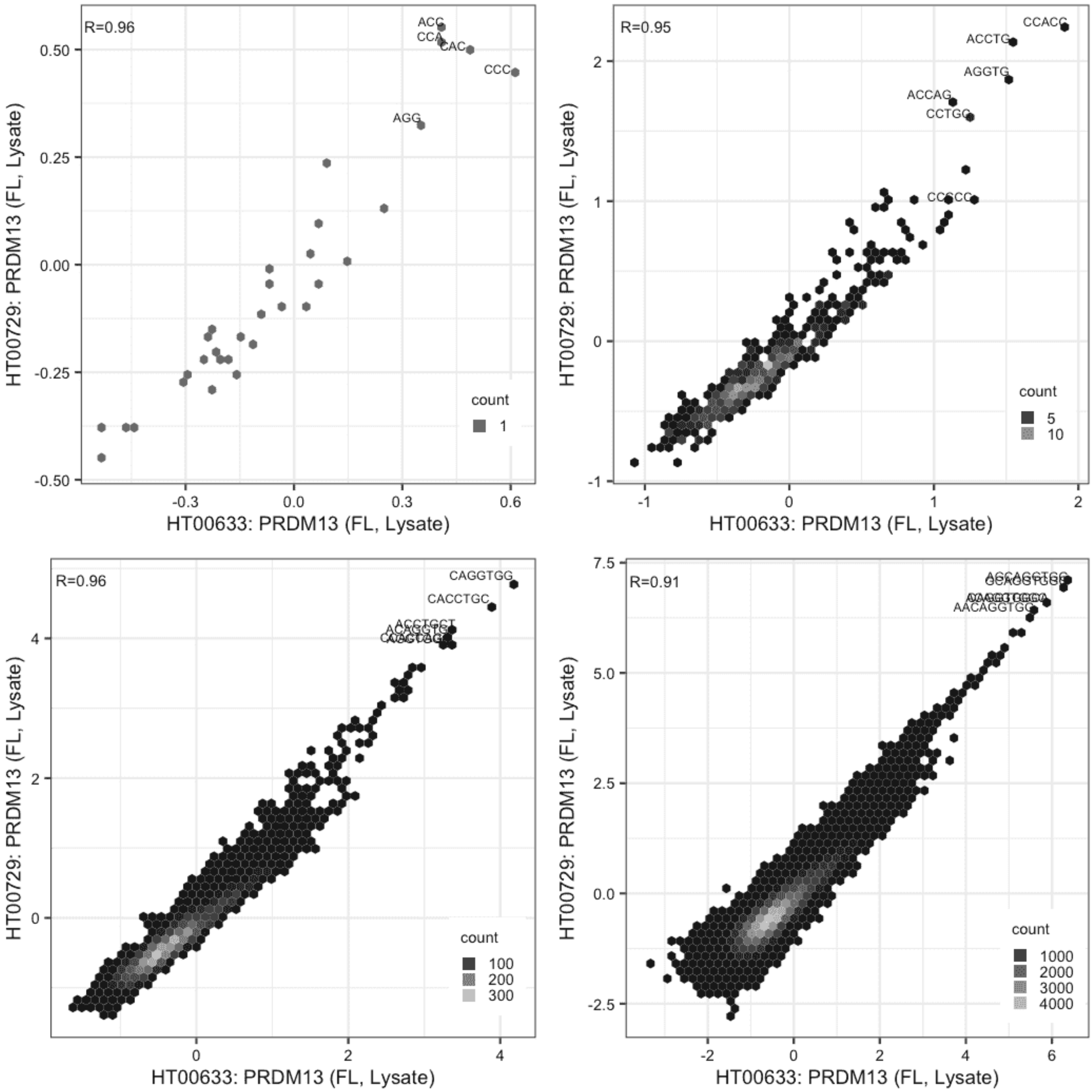

PRDM13

Lysate vs GFPIVT, different constructs

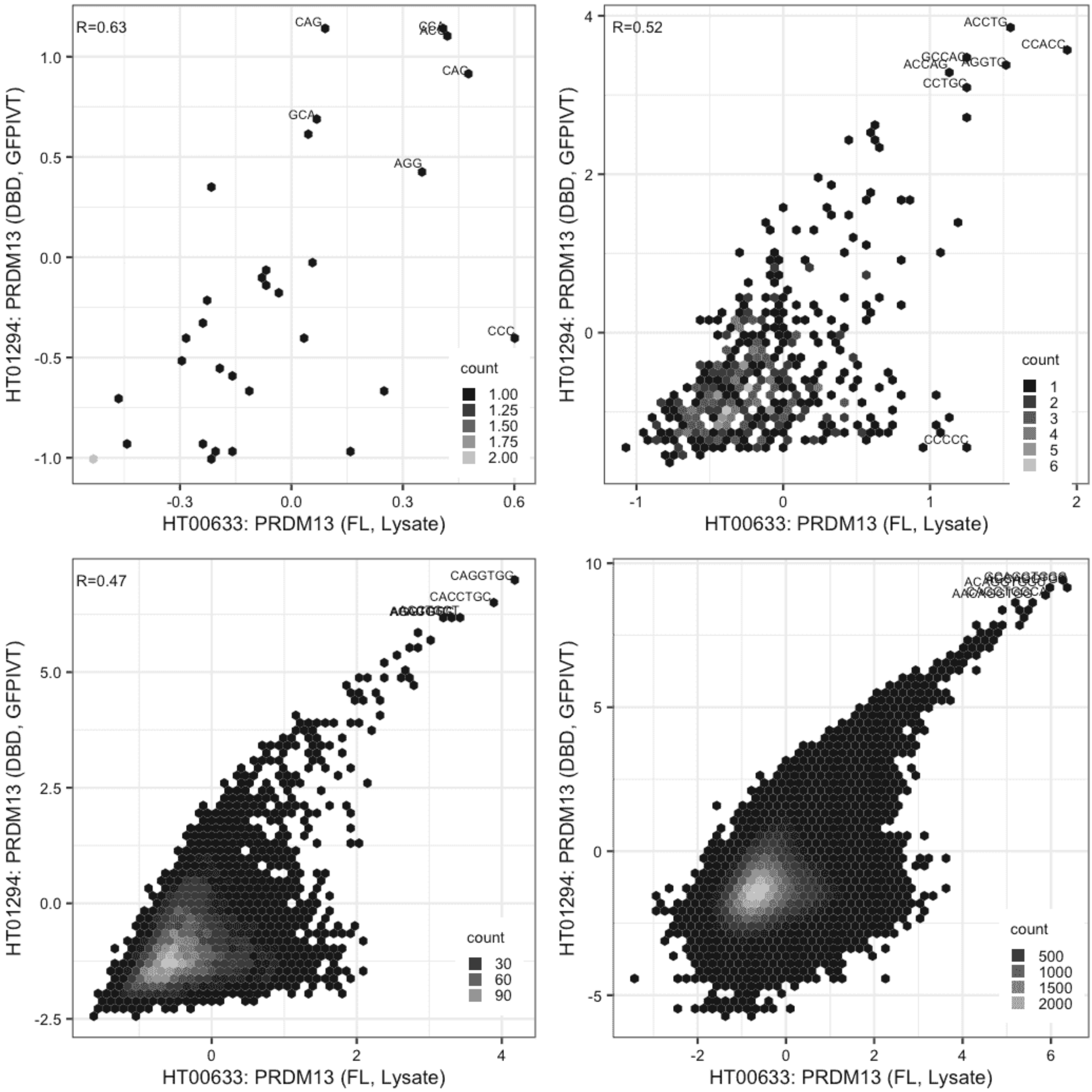

PRDM13

Lysate vs GFPIVT, same construct

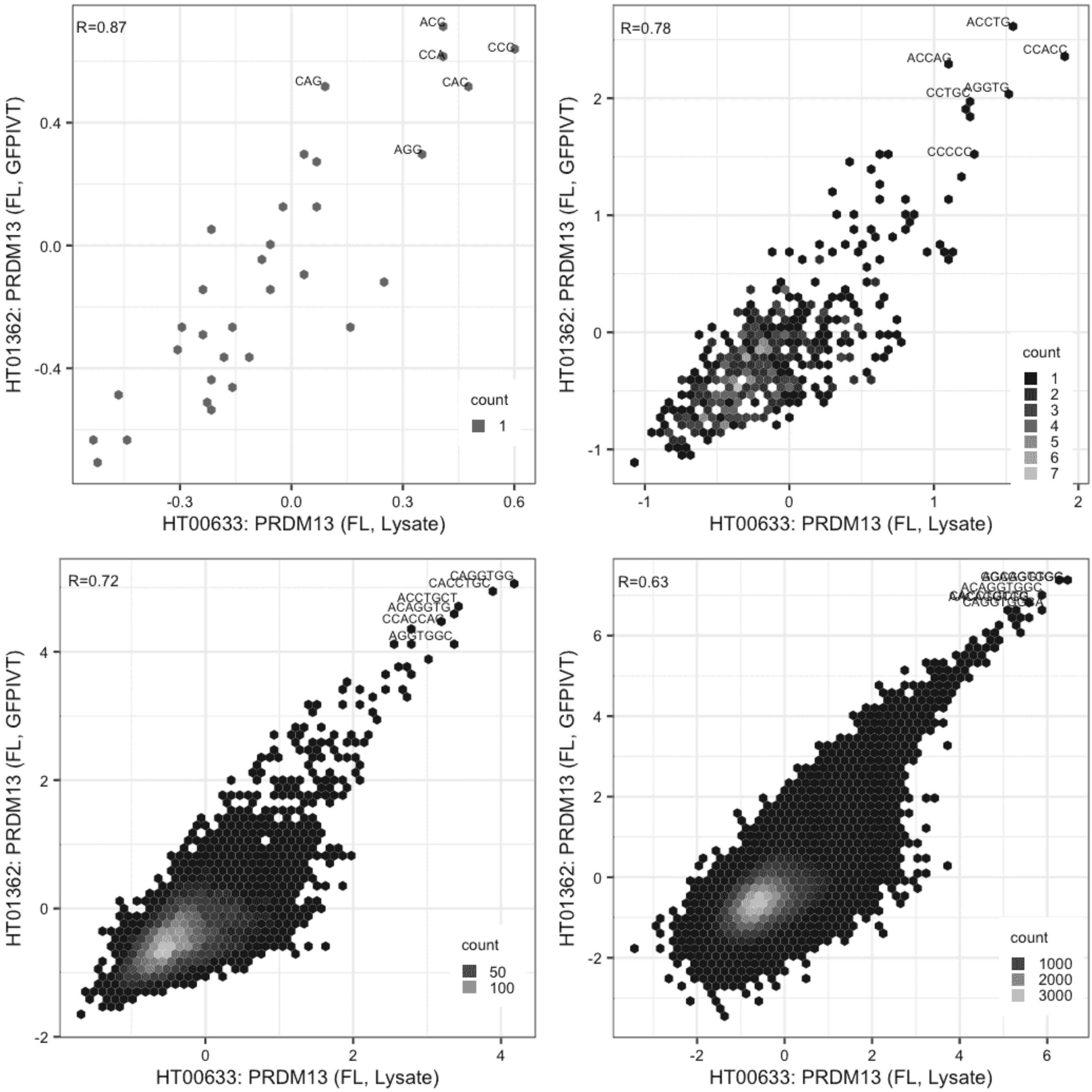

PRDM13

Lysate vs GFPIVT, different constructs

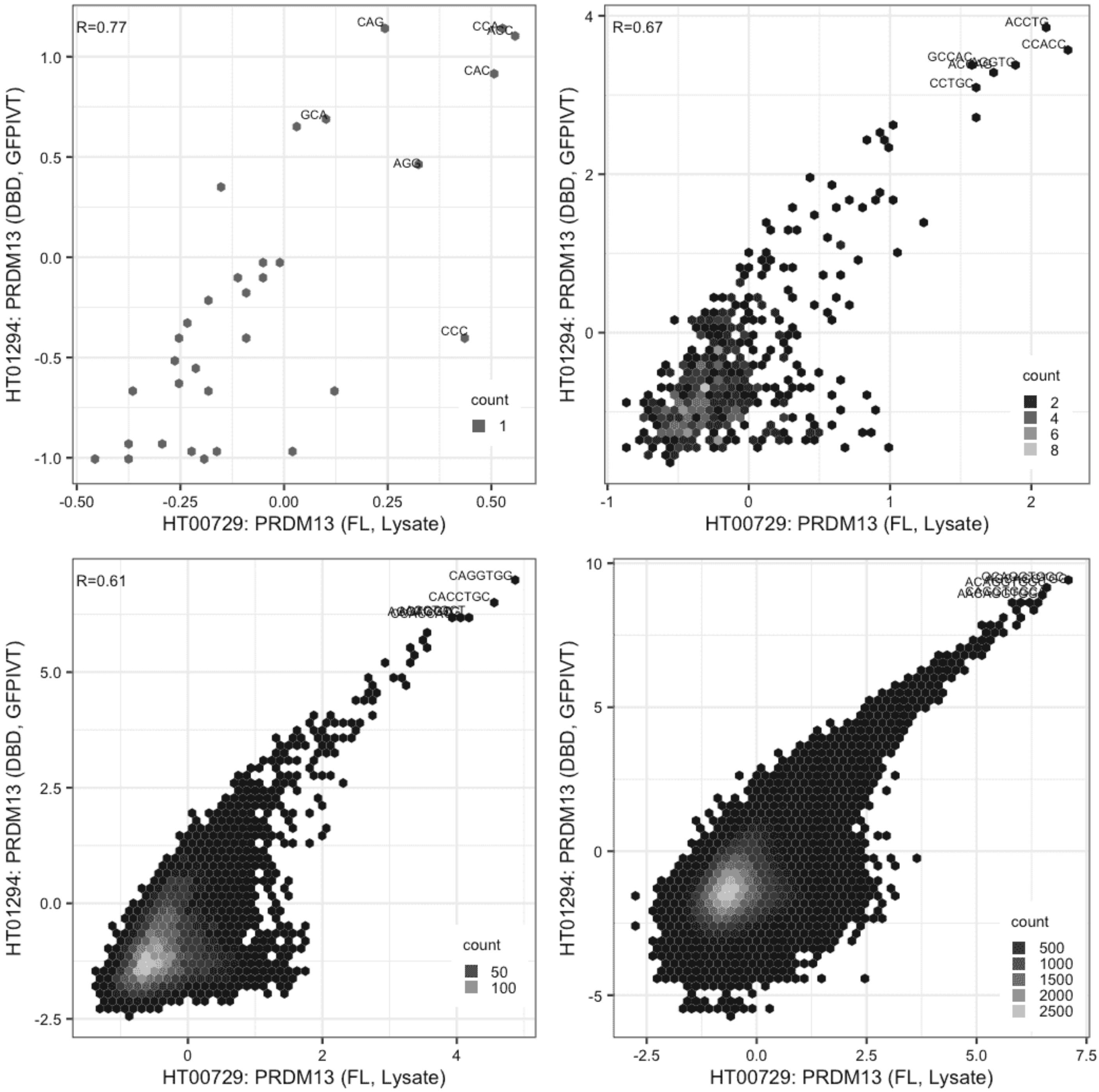

PRDM13

Lysate vs GFPIVT, same construct

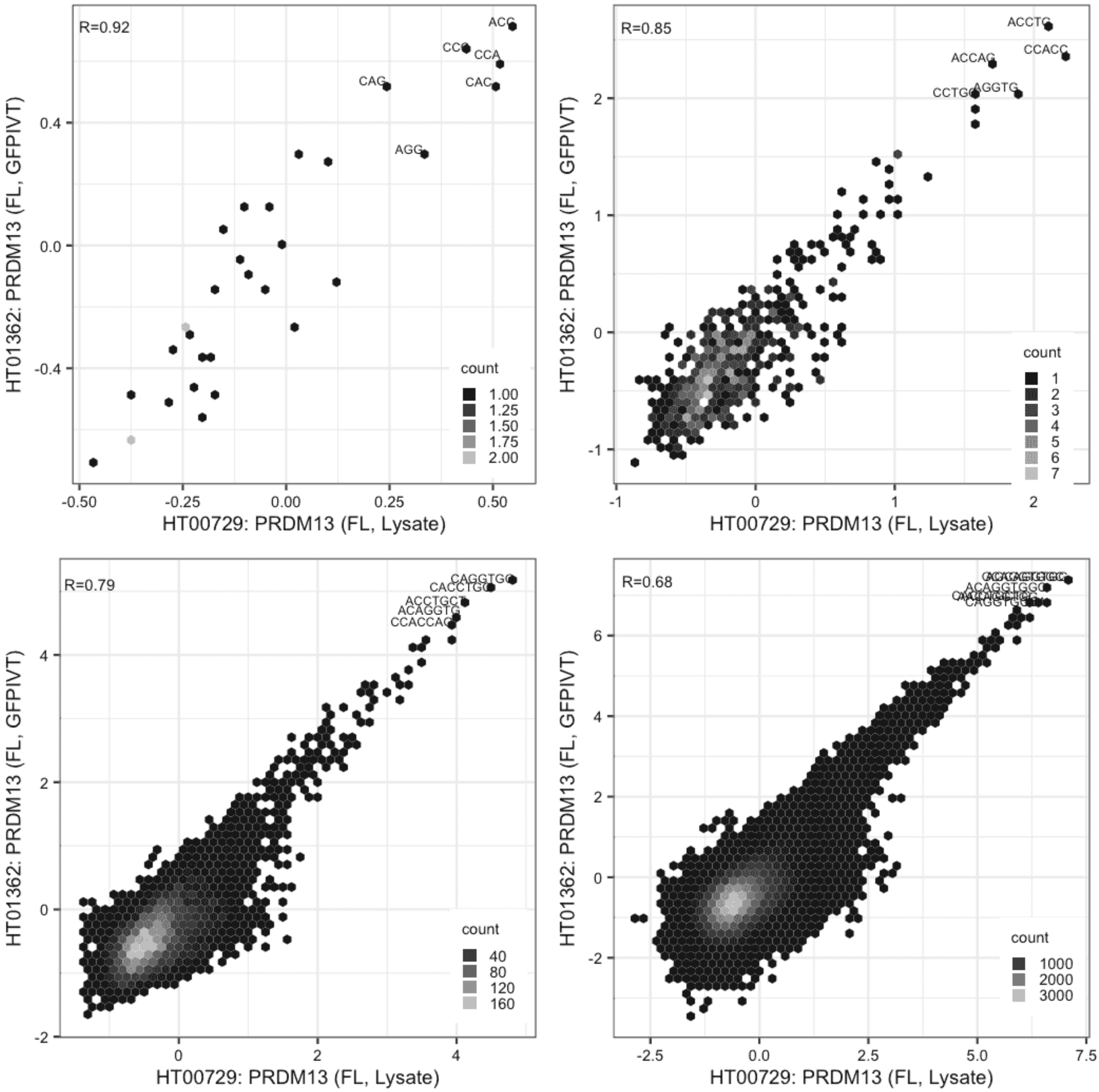

PRDM13

GFPIVT vs GFPIVT, different constructs

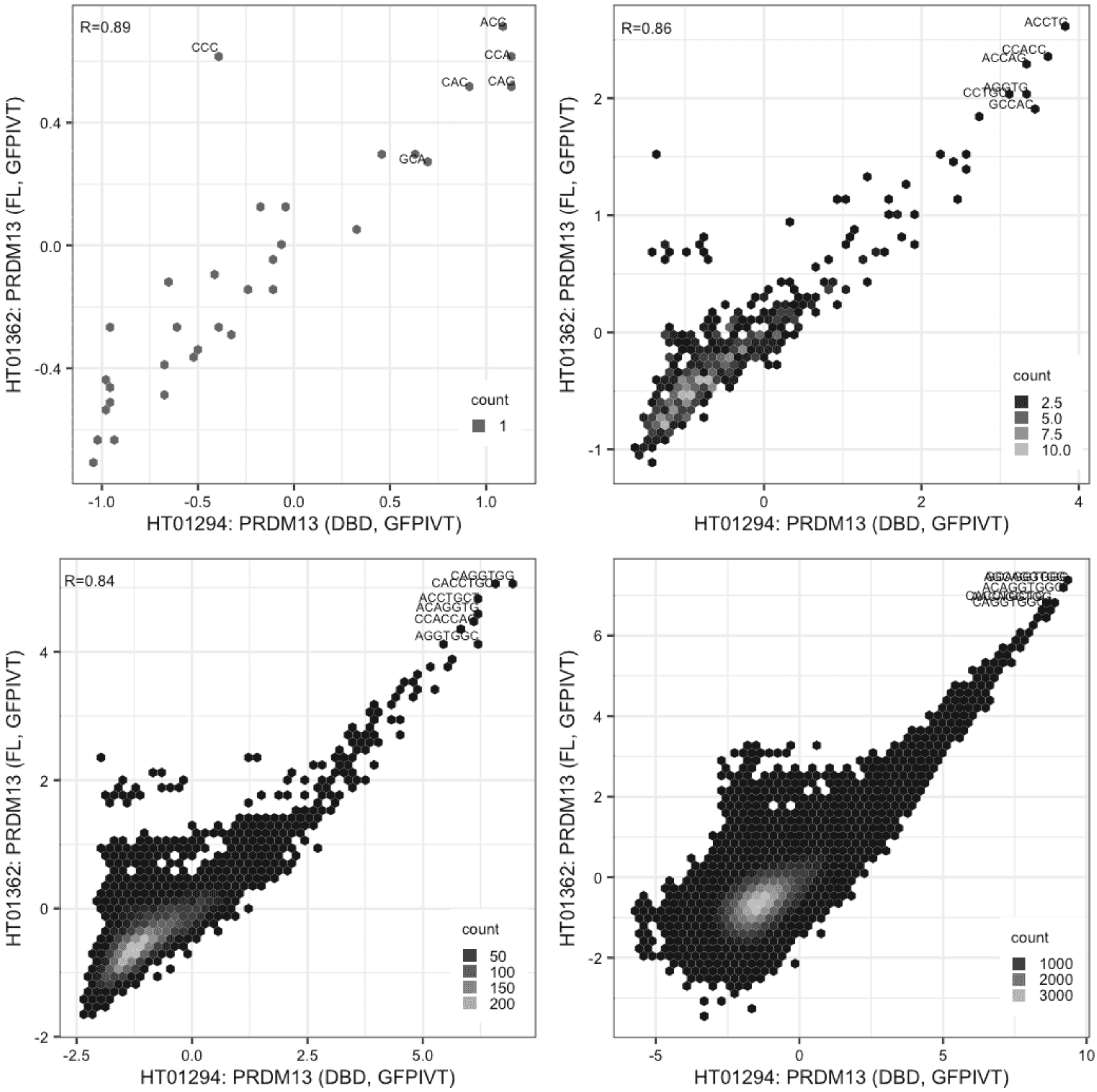

PRDM5

Lysate vs Lysate, same construct

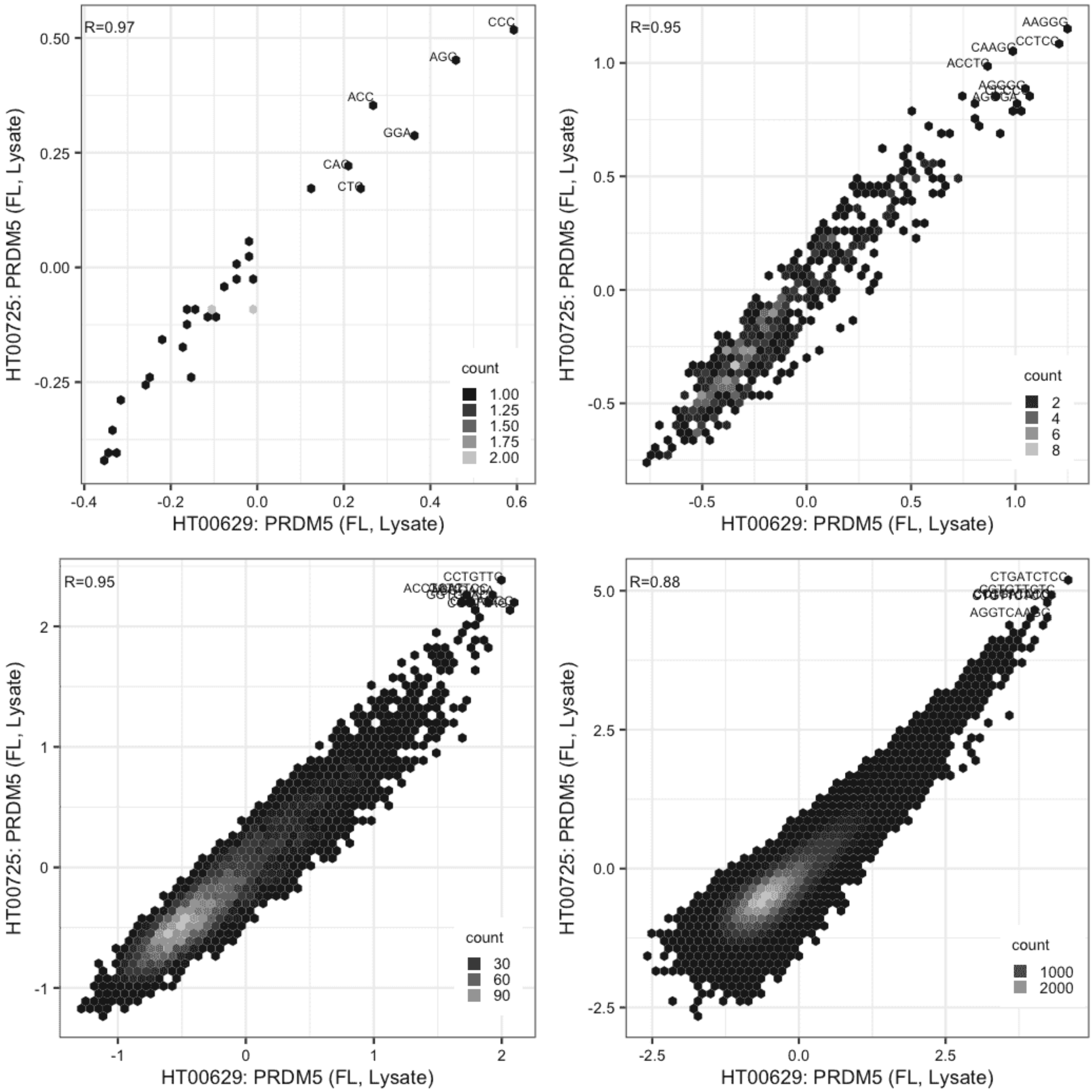

PRDM5

Lysate vs GFPIVT, same construct

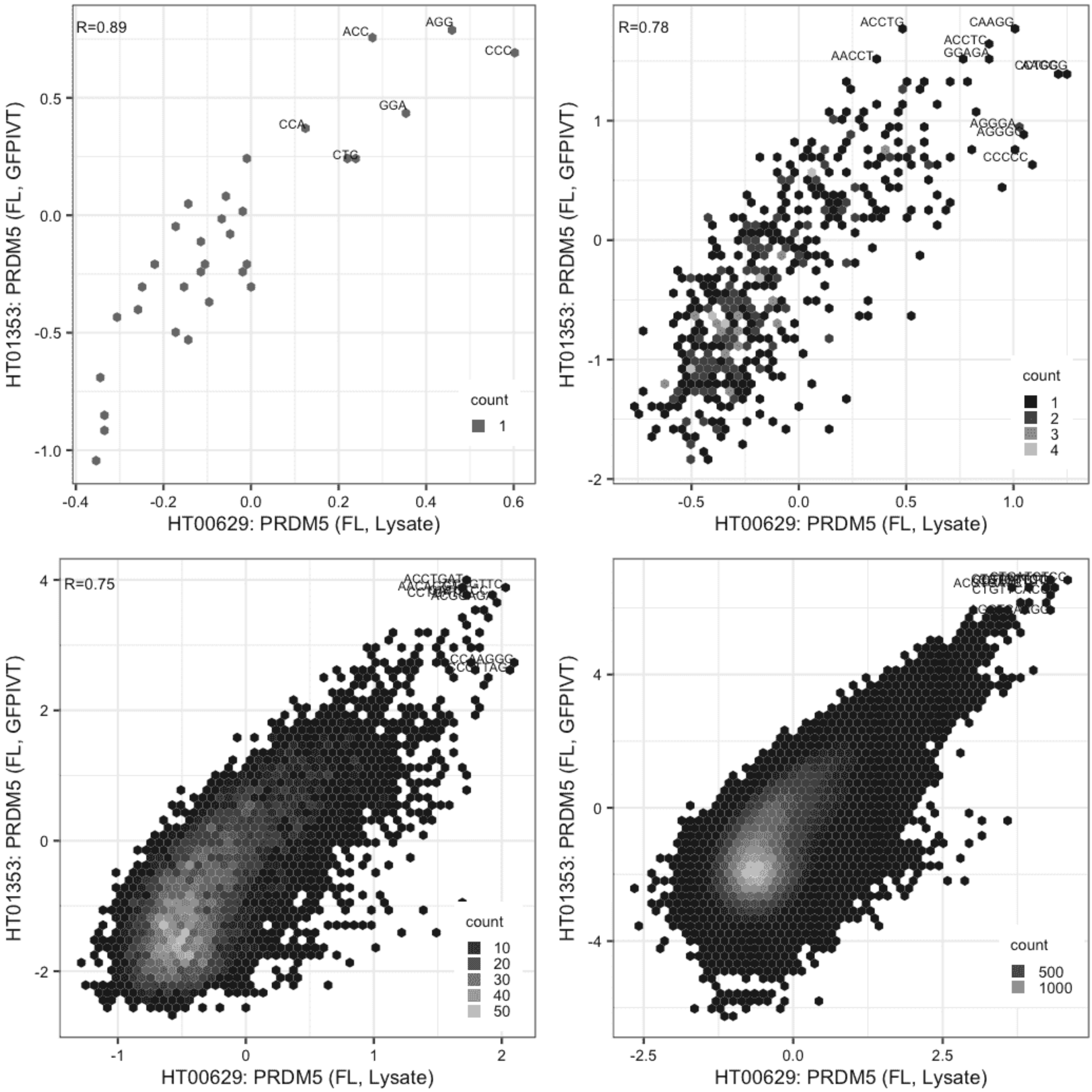

PRDM5

Lysate vs GFPIVT, different constructs

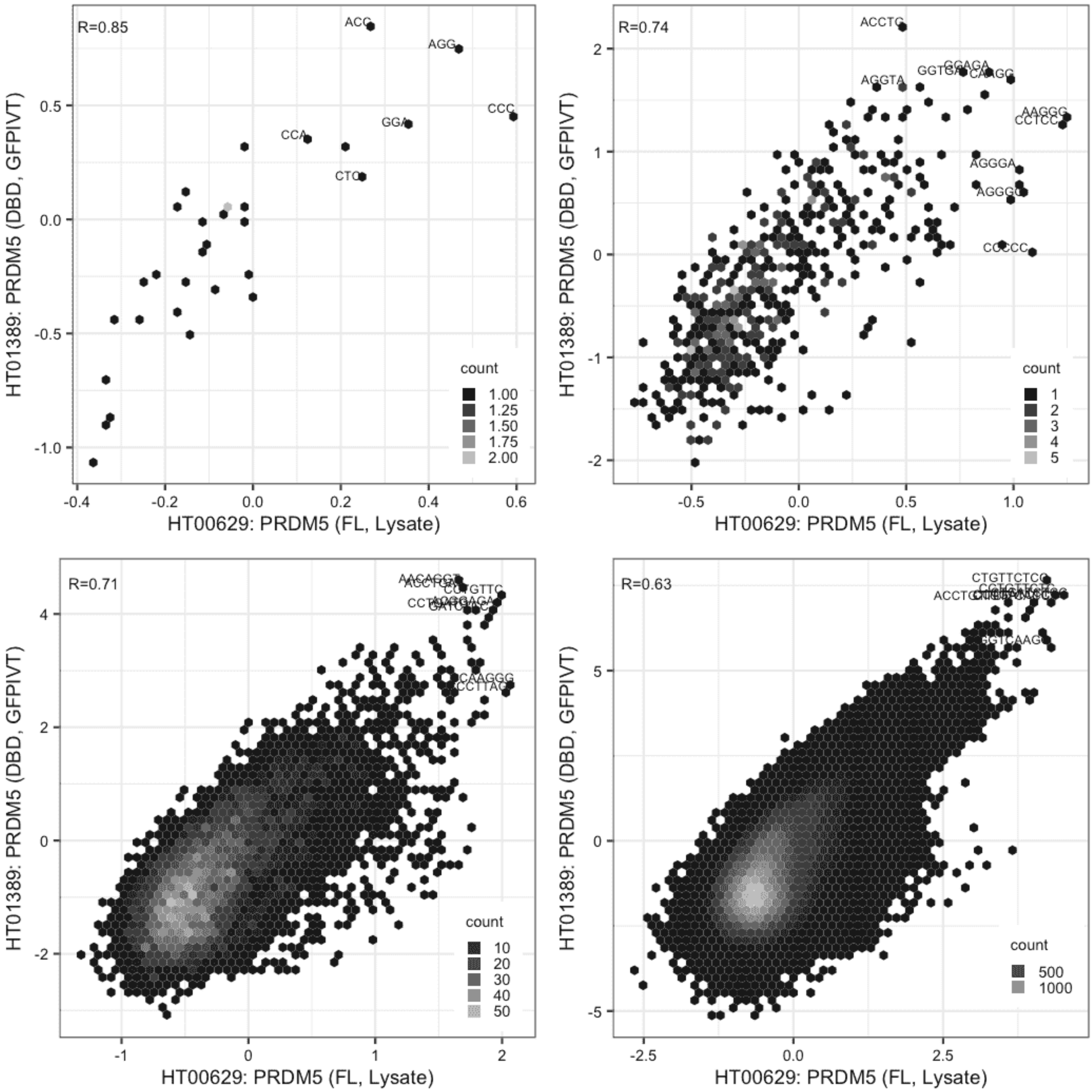

PRDM5

Lysate vs GFPIVT, same construct

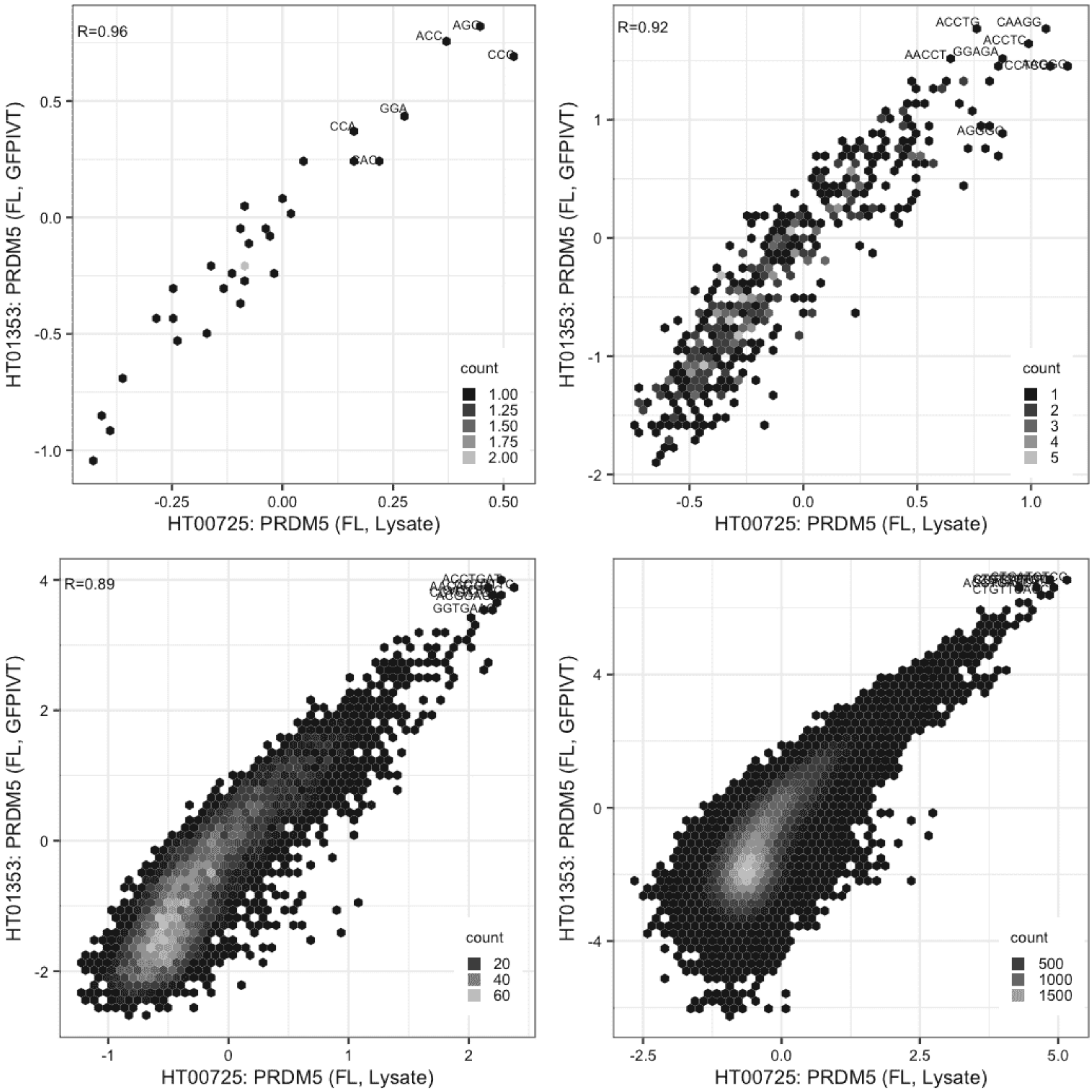

PRDM5

Lysate vs GFPIVT, different constructs

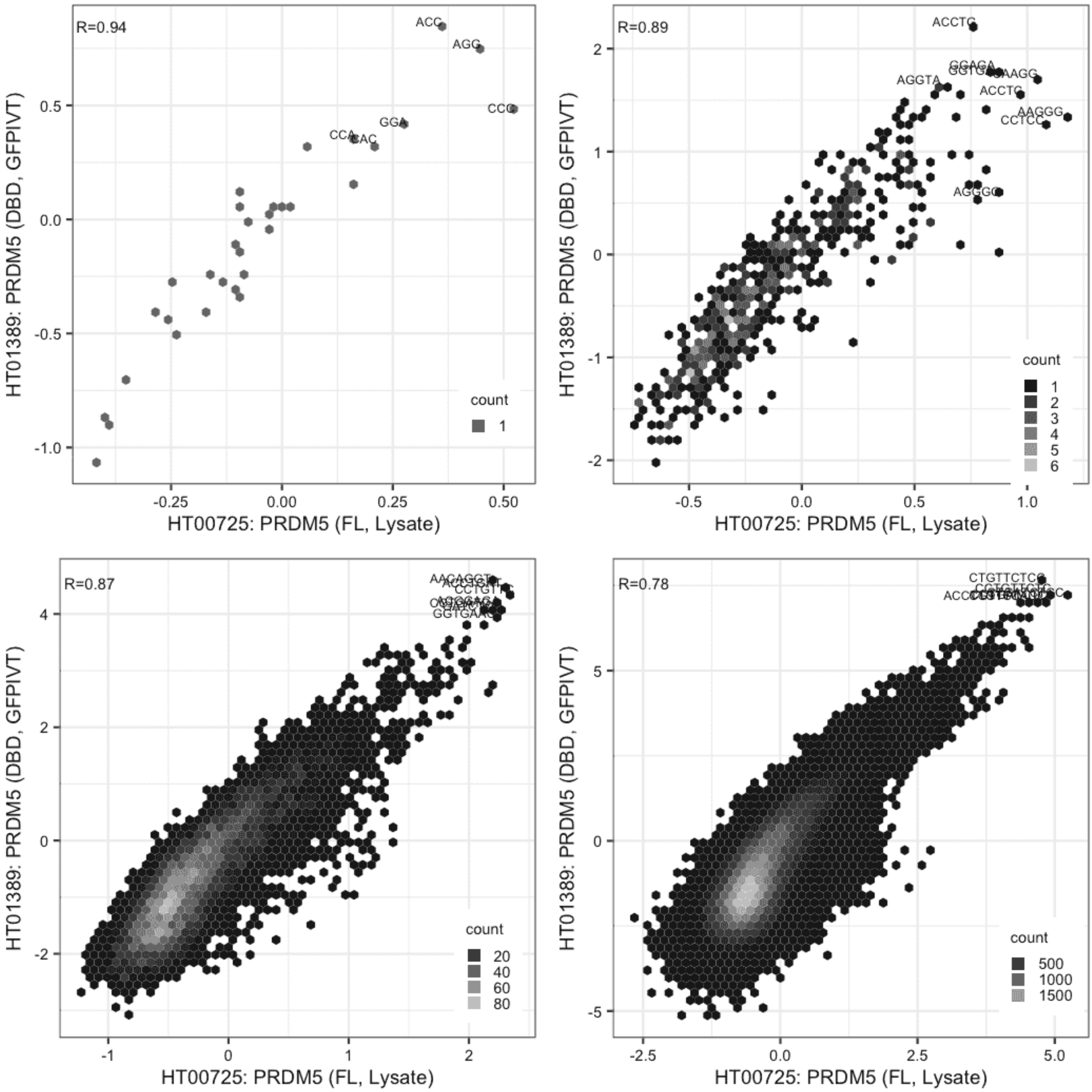

PRDM5

GFPIVT vs GFPIVT, different constructs

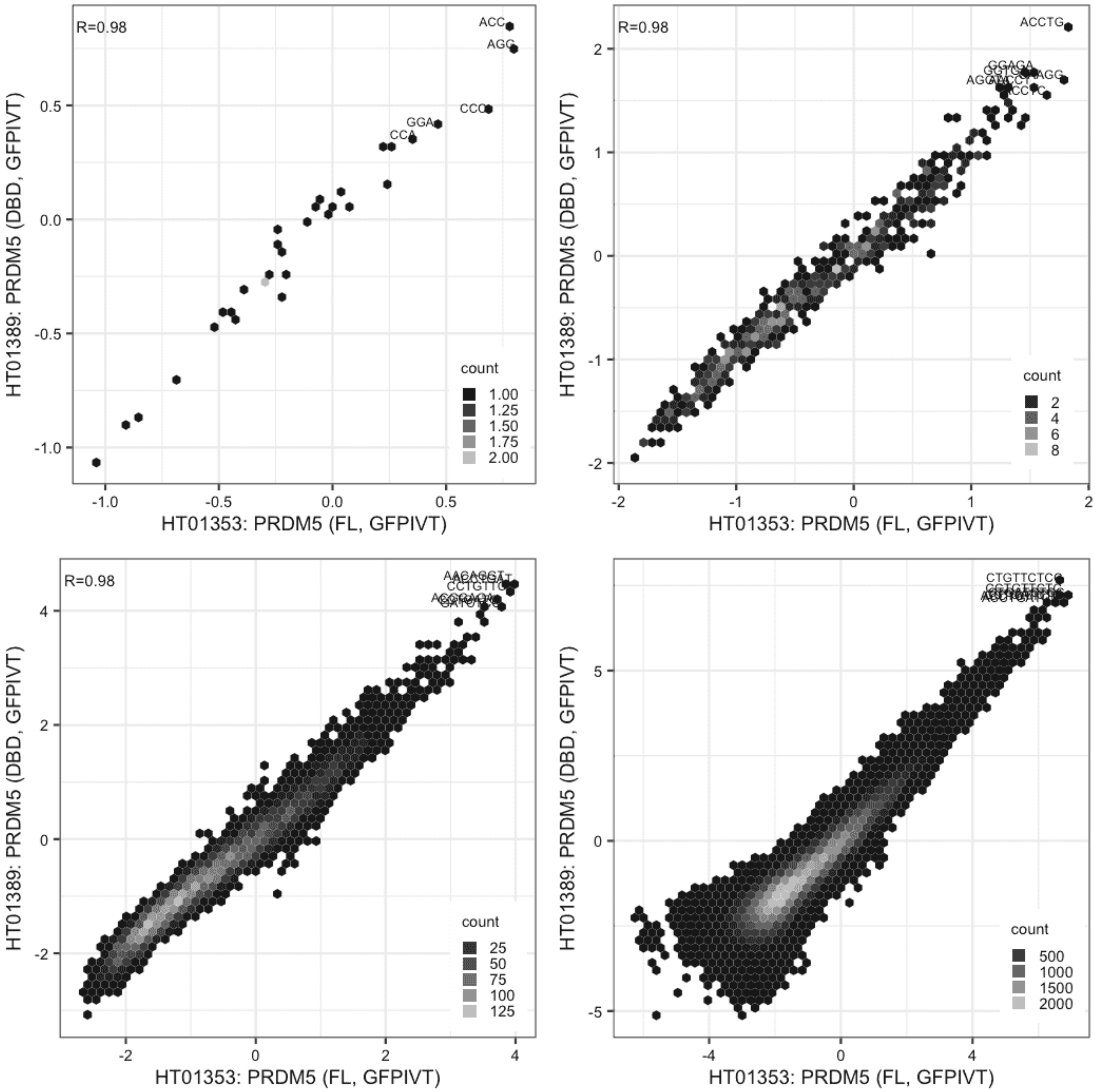

RARA

Lysate vs IVT, same construct

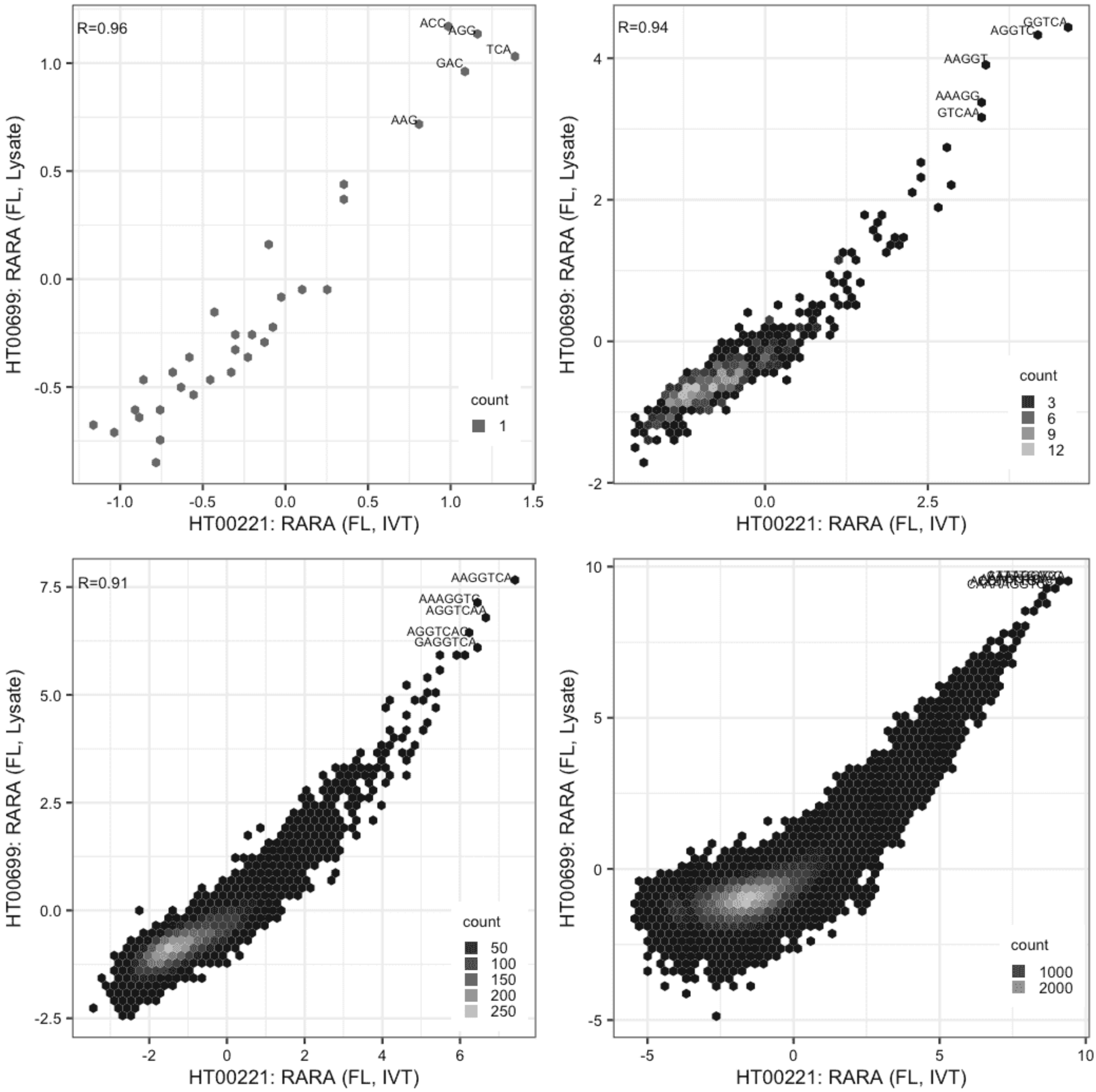

RARA

Lysate vs IVT, same construct

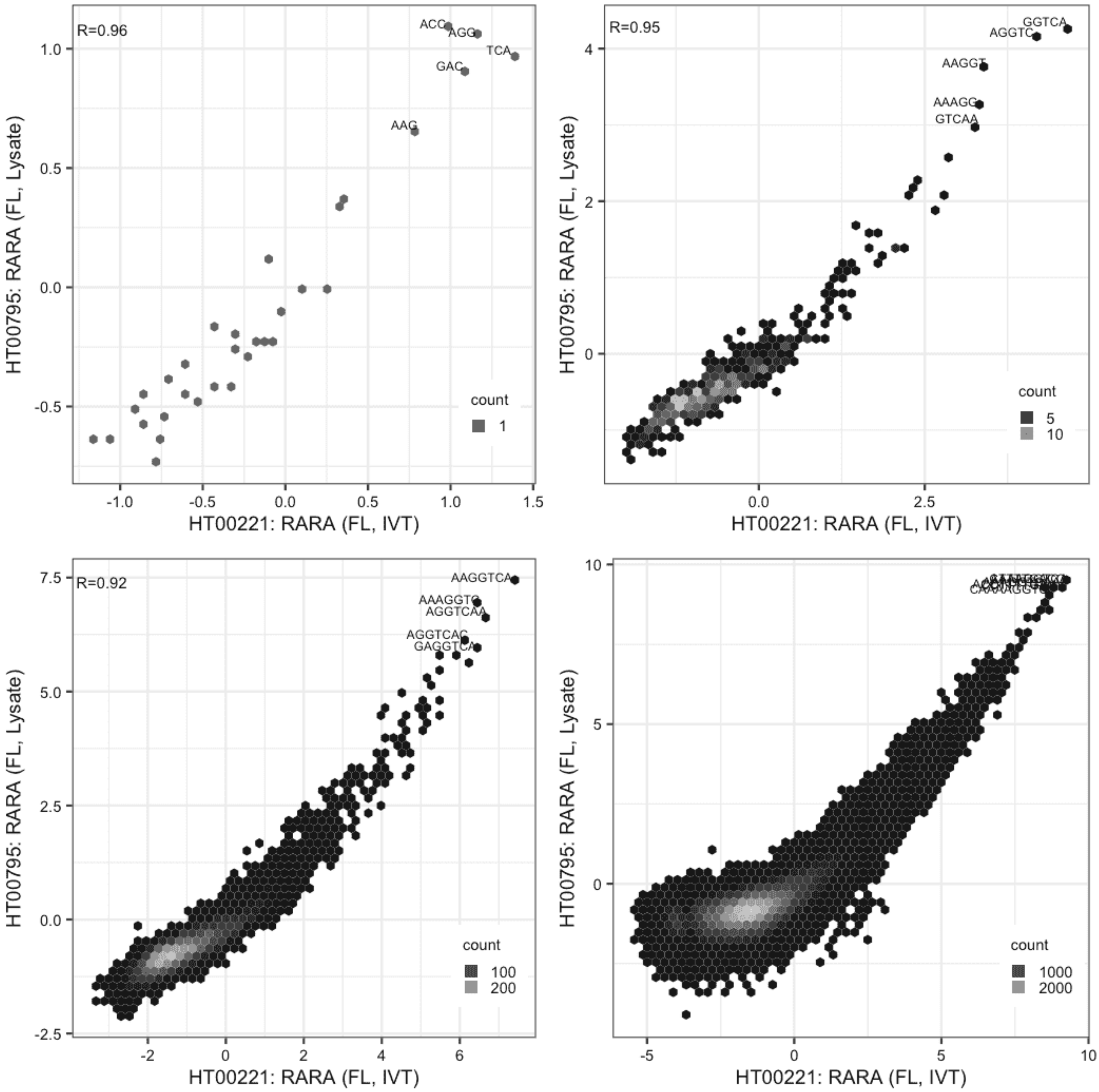

RARA

Lysate vs Lysate, same construct

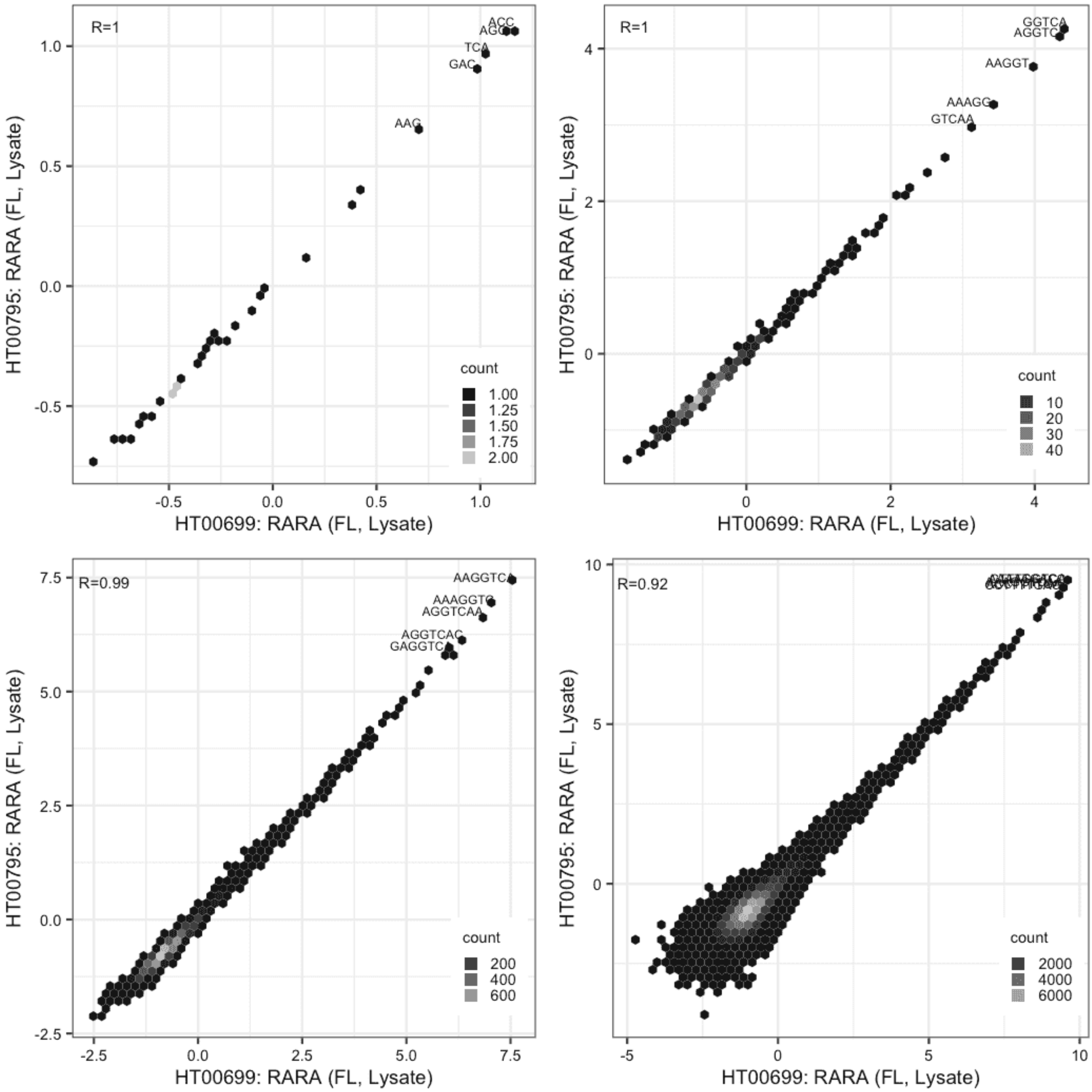

RORB

Lysate vs IVT, same construct

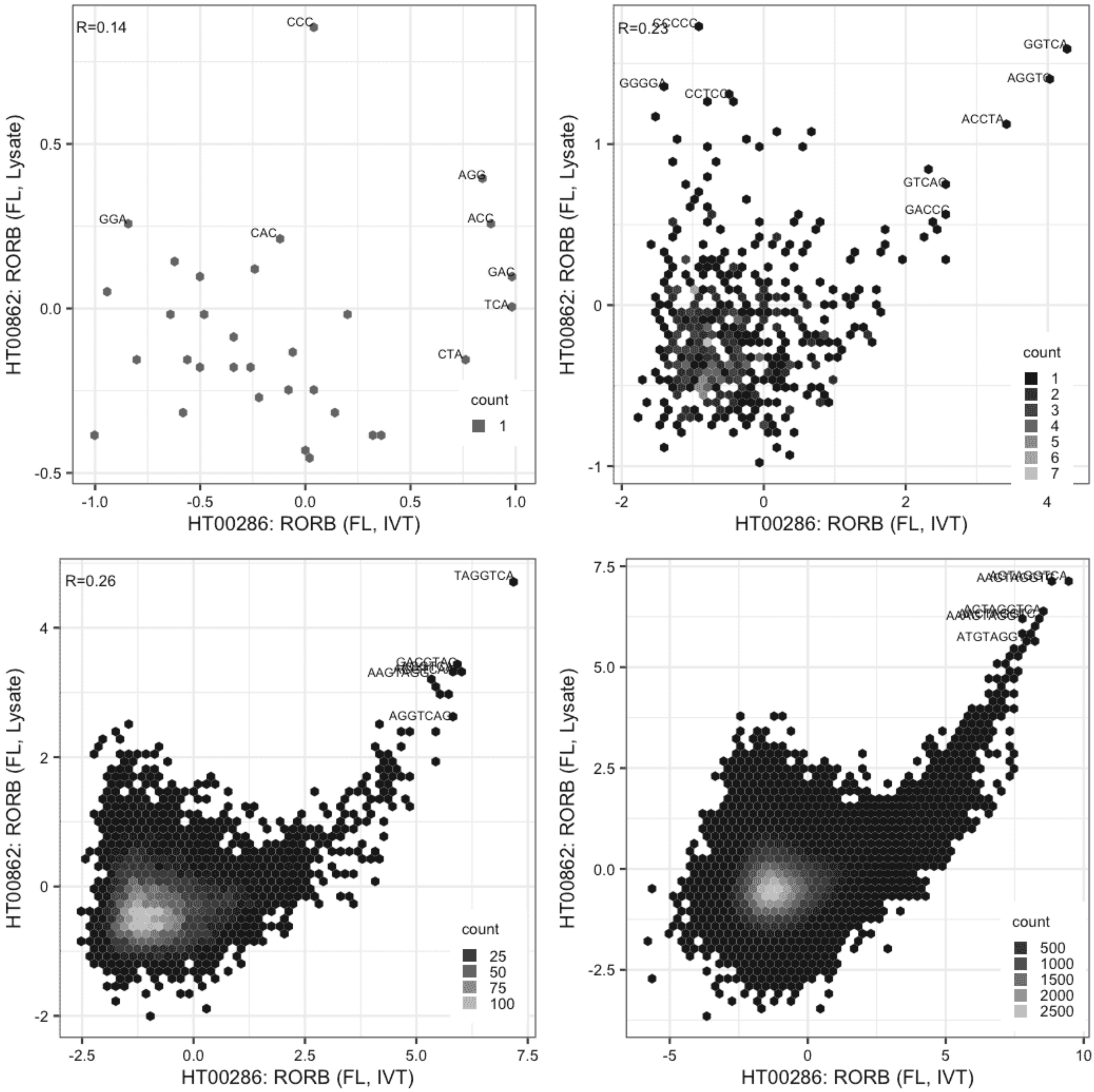

RXRA

IVT vs IVT, same construct

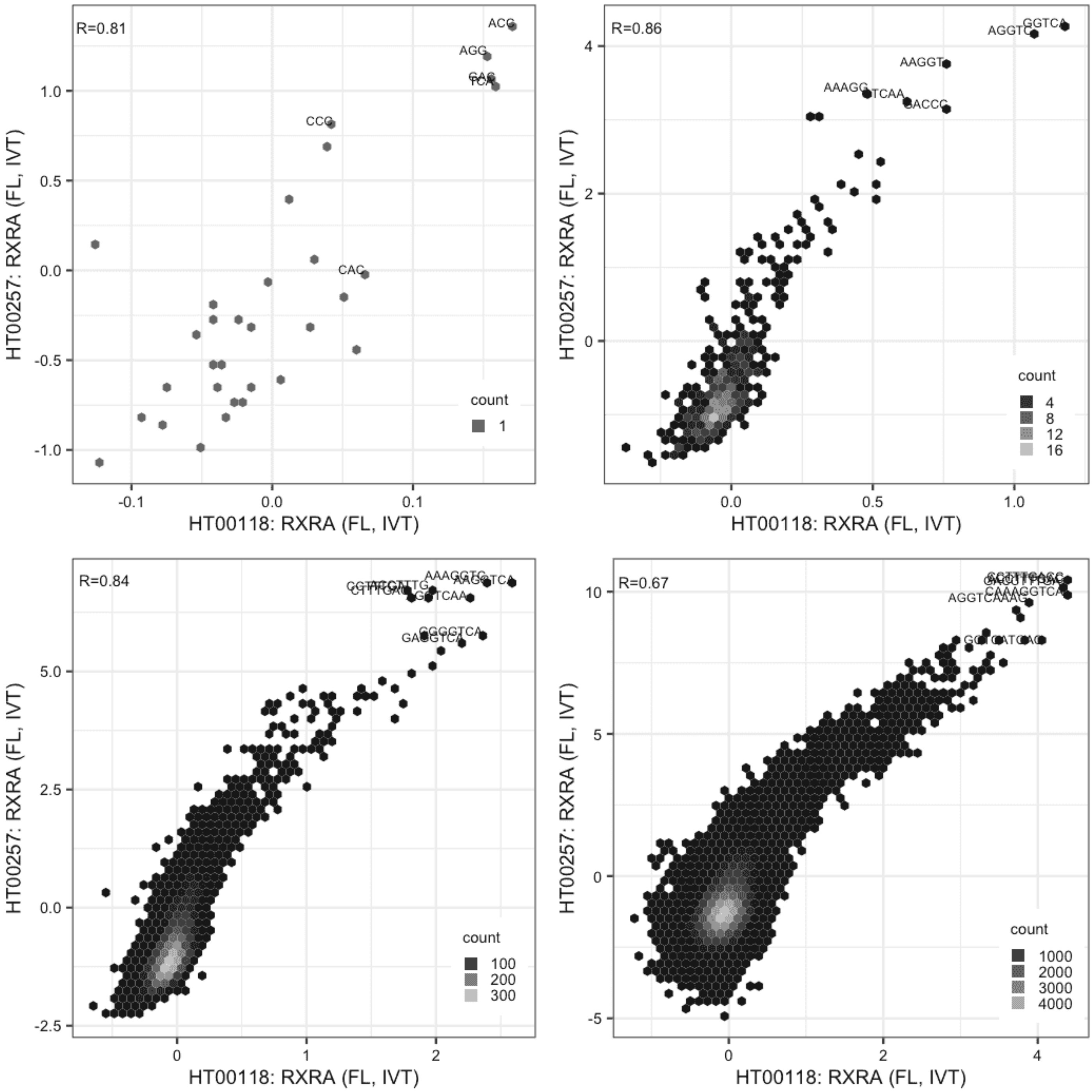

RXRA

Lysate vs IVT, same construct

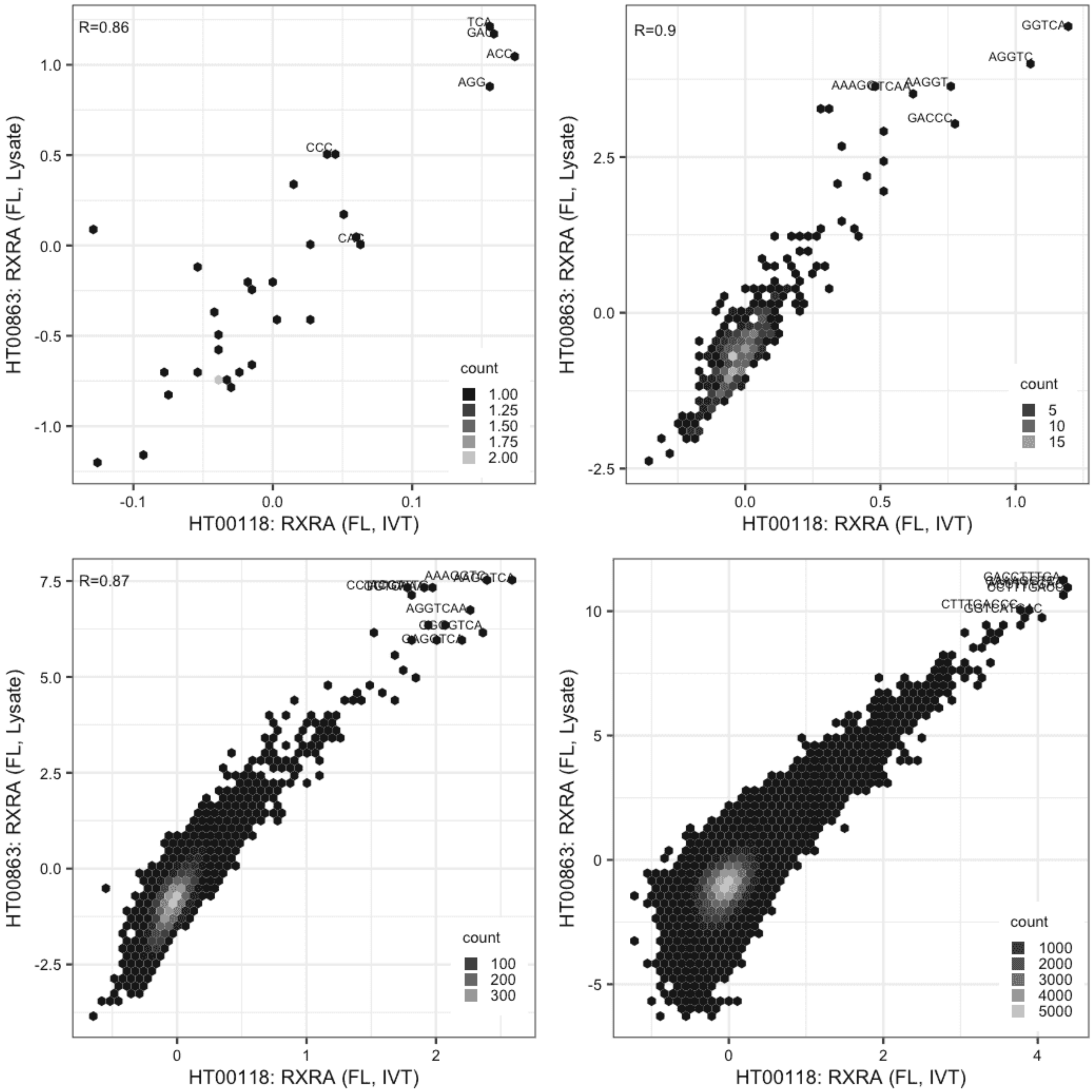

RXRA

Lysate vs IVT, same construct

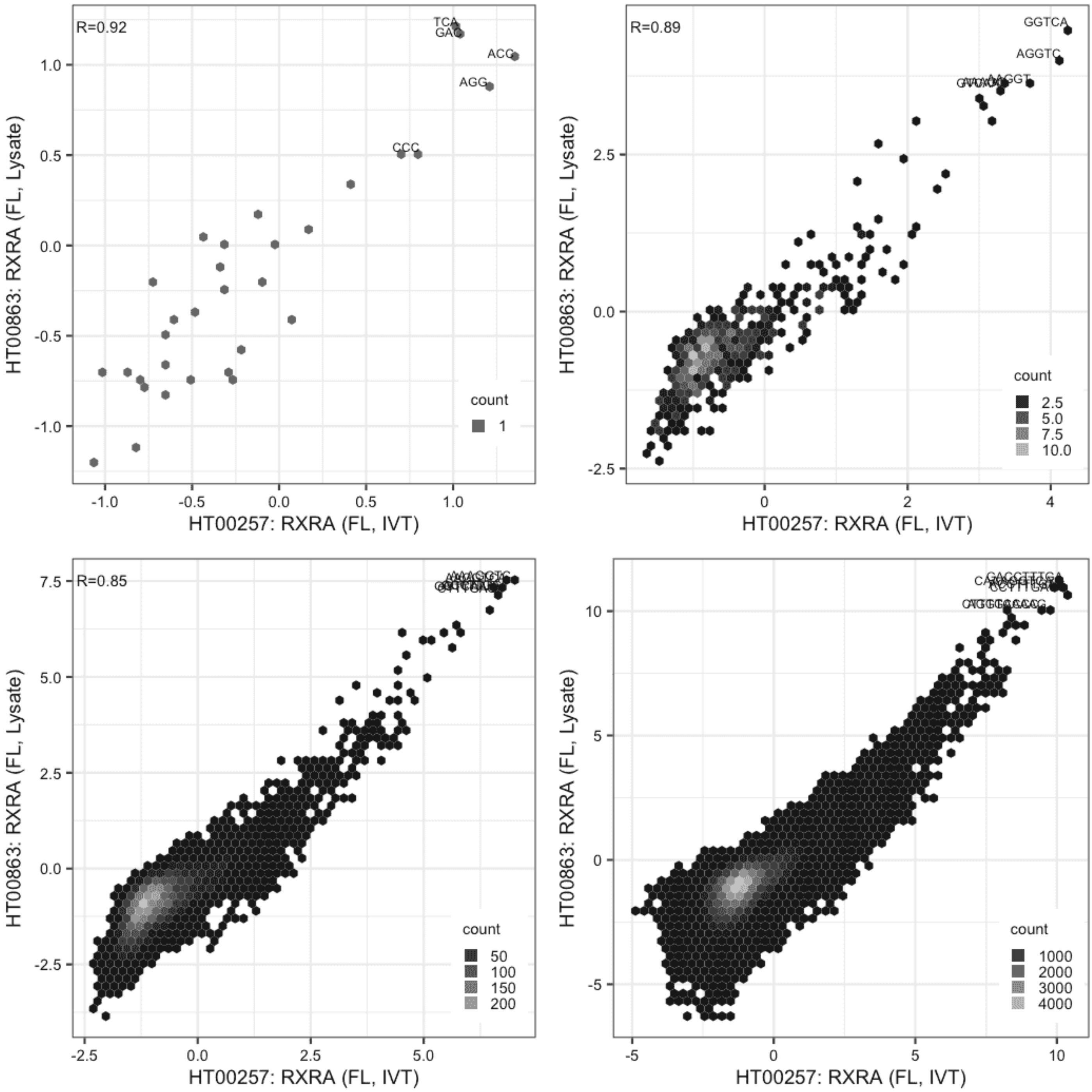

SLC2A4RG

Lysate vs IVT, different constructs

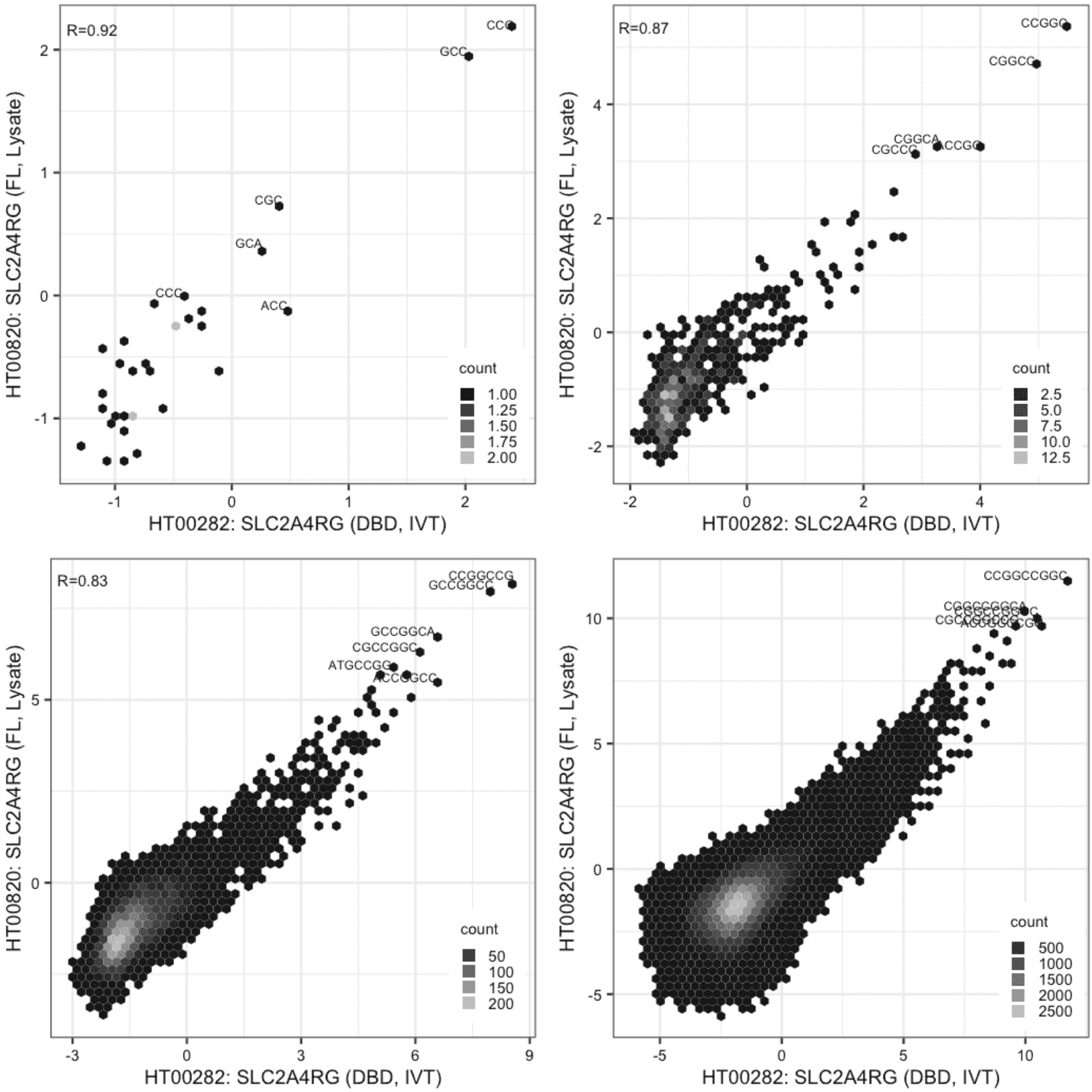

SLC2A4RG

IVT vs GFPIVT, different constructs

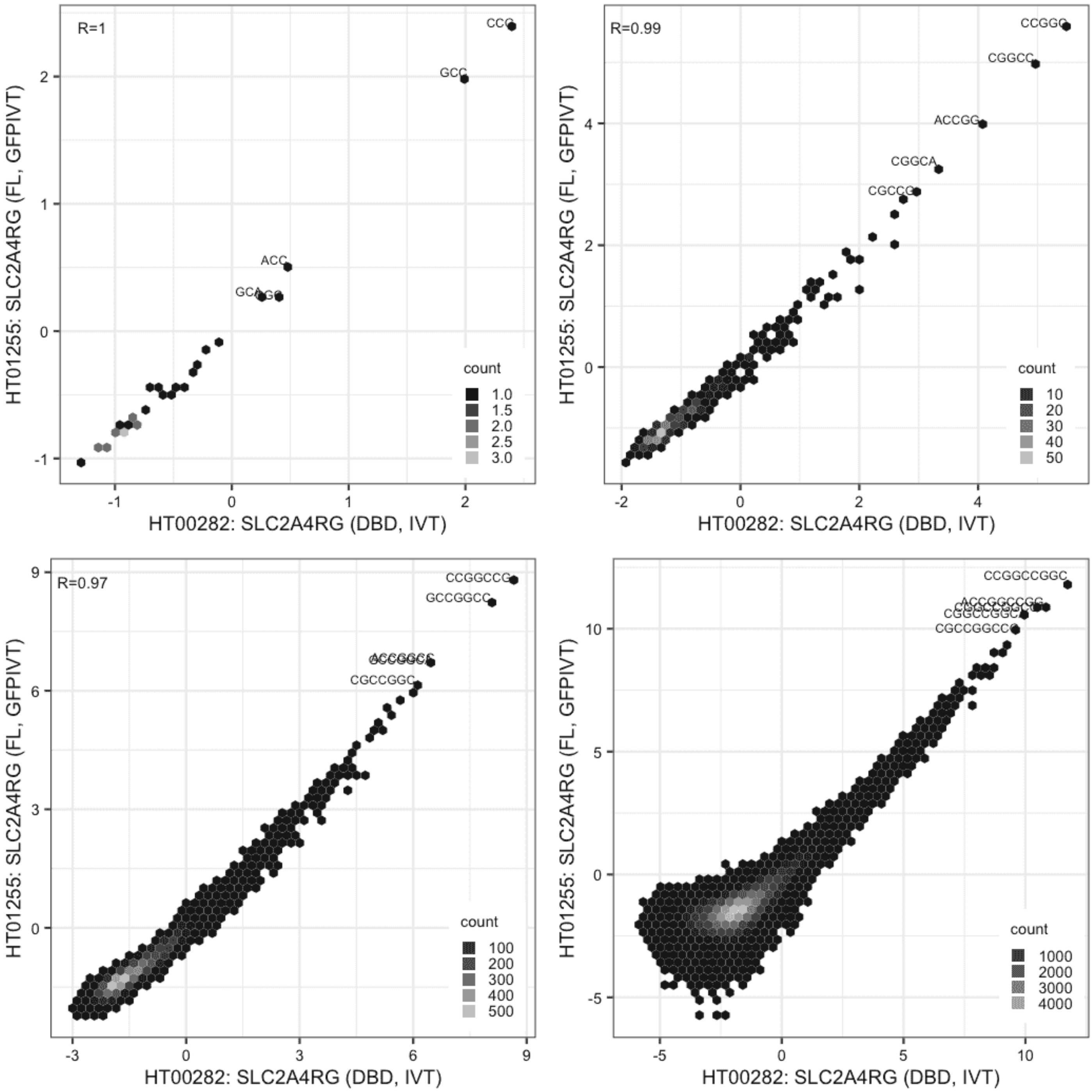

SLC2A4RG

Lysate vs GFPIVT, same construct

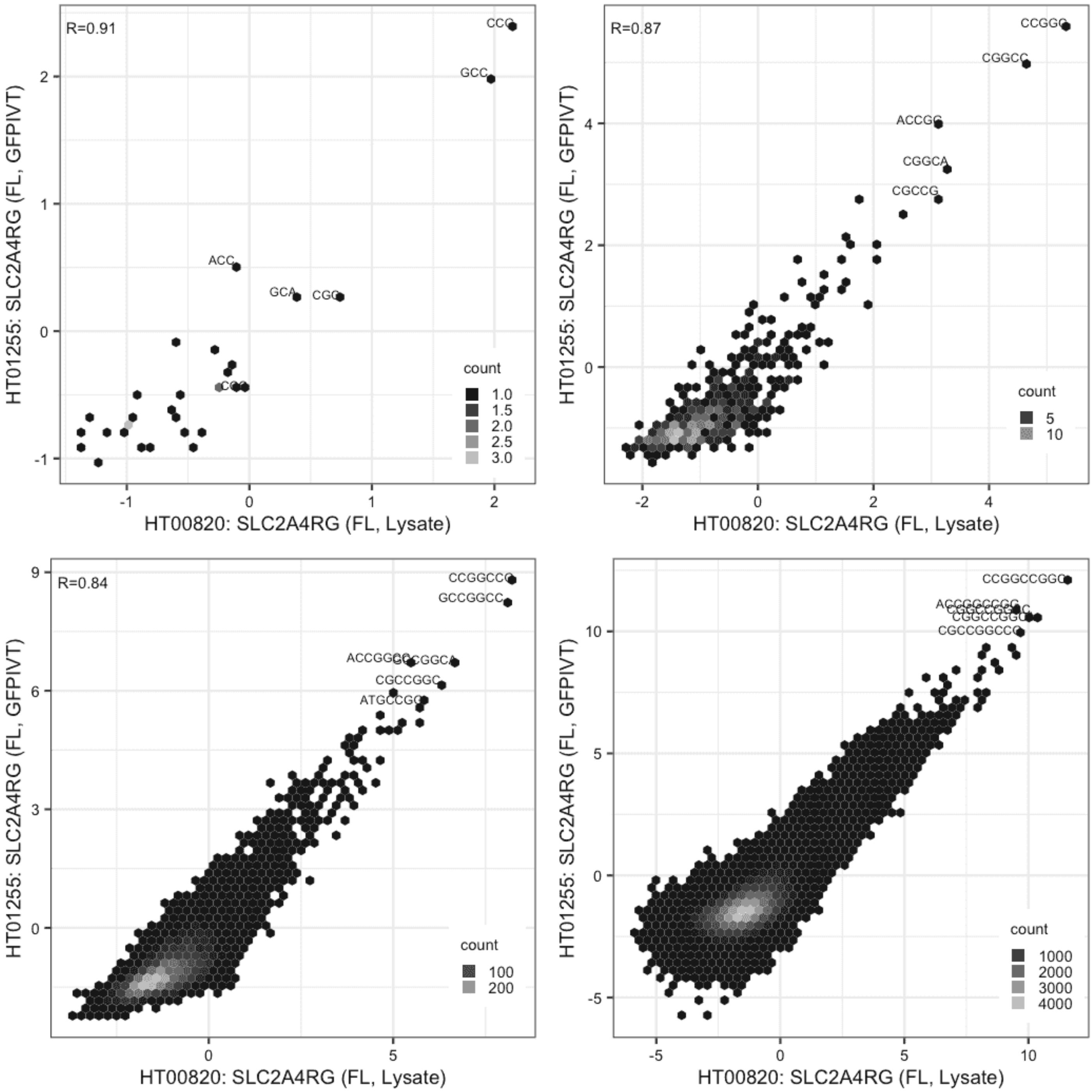

SOX15

Lysate vs IVT, same construct

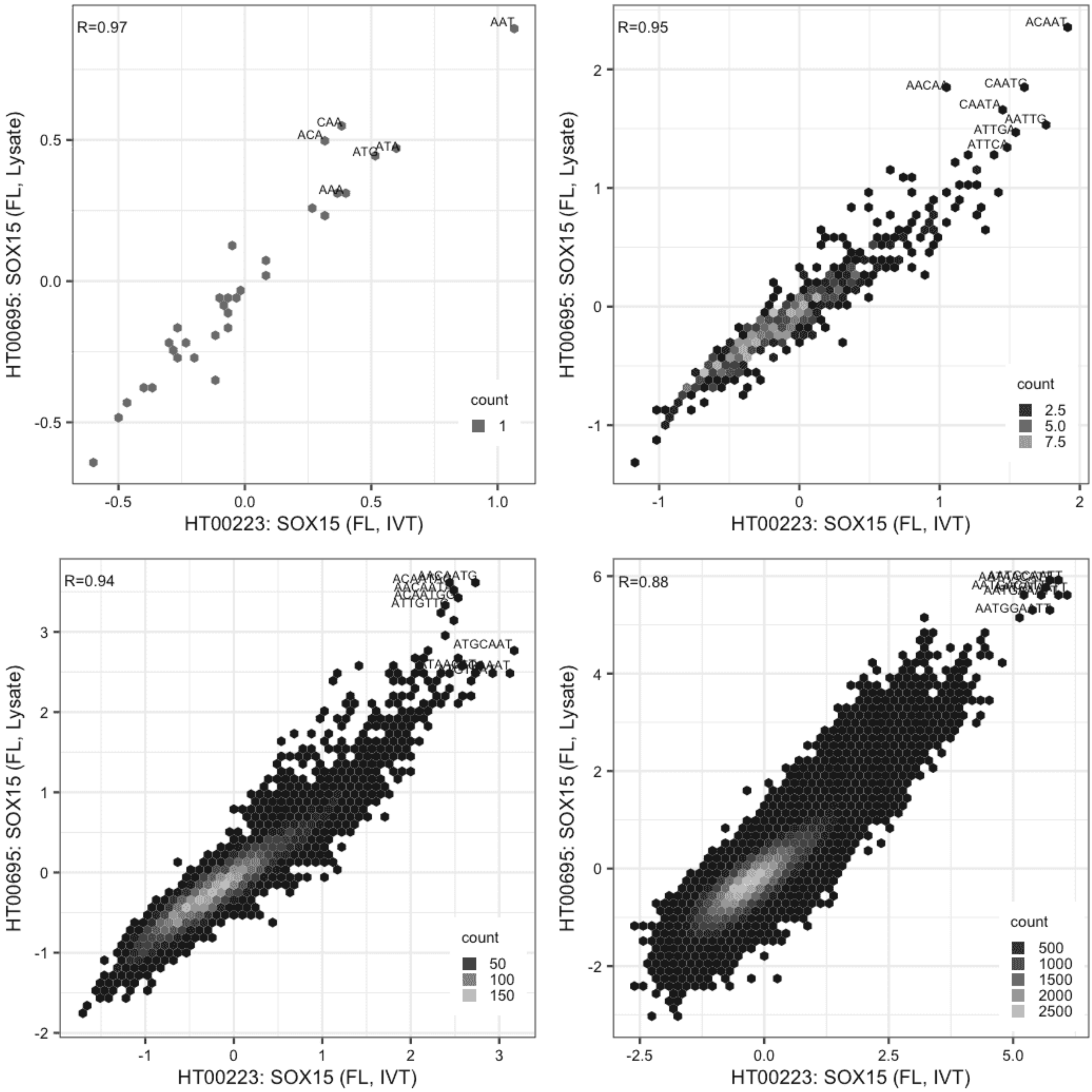

SOX15

Lysate vs IVT, same construct

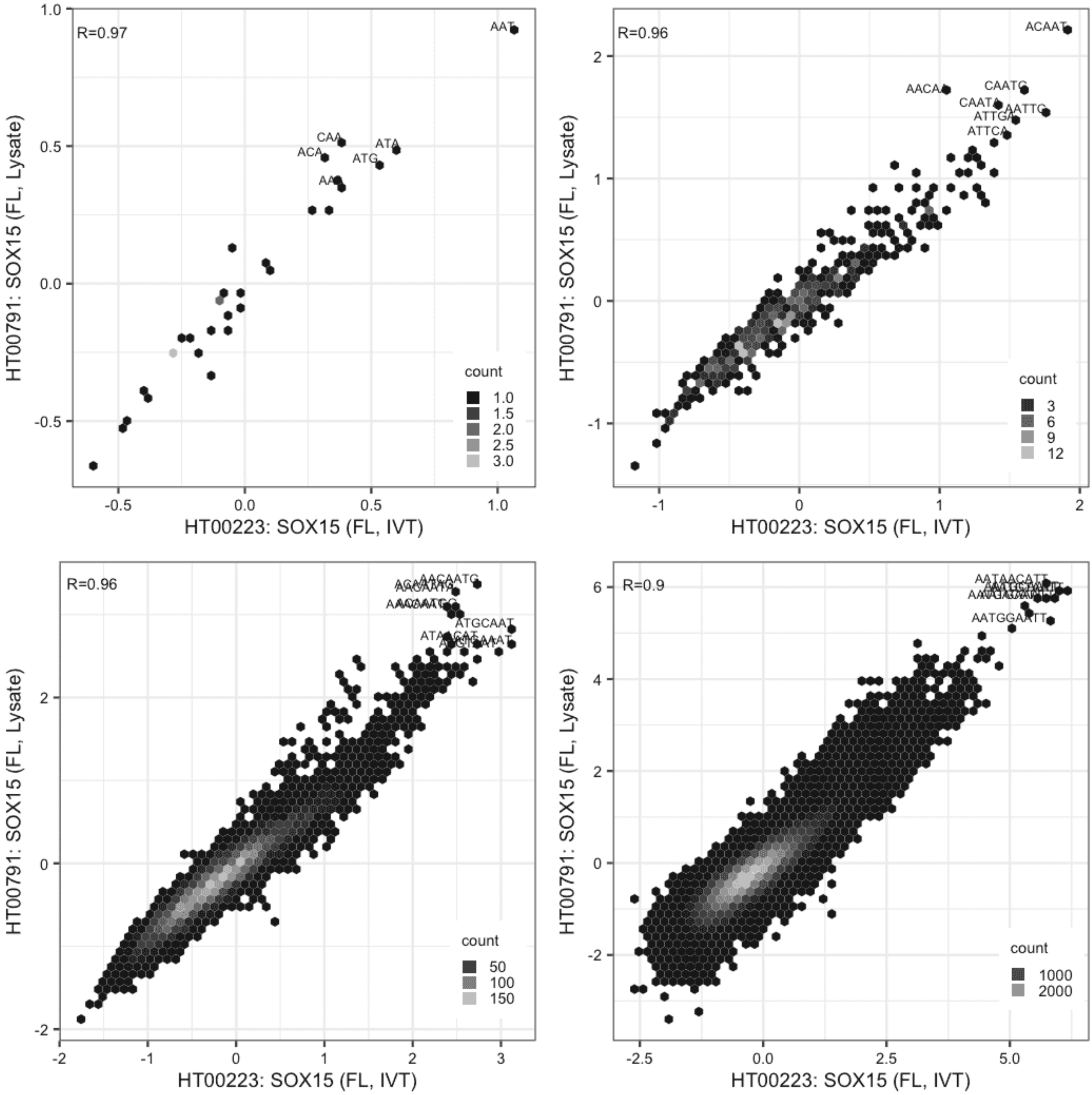

SOX15

Lysate vs IVT, same construct

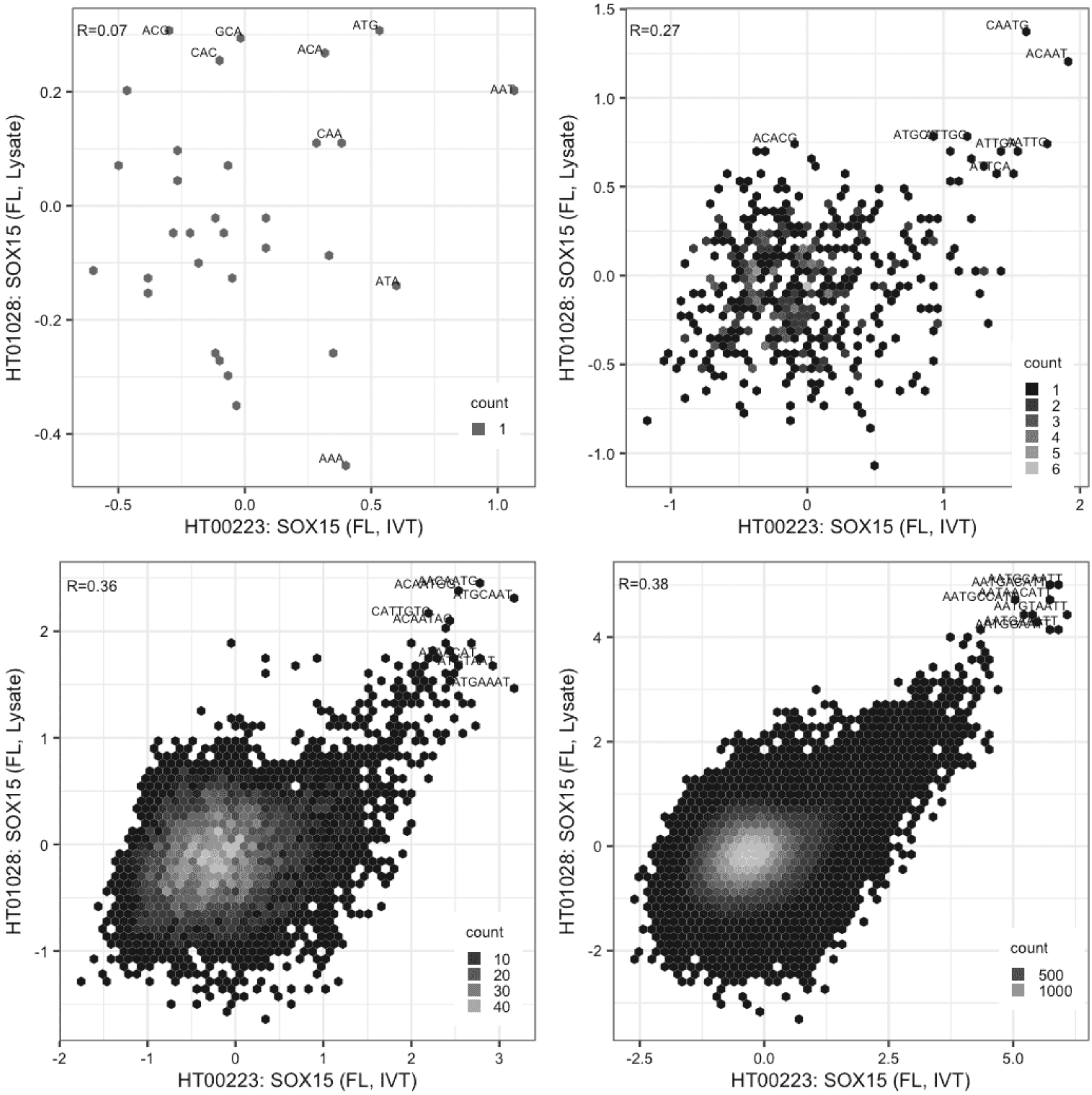

SOX15

Lysate vs IVT, same construct

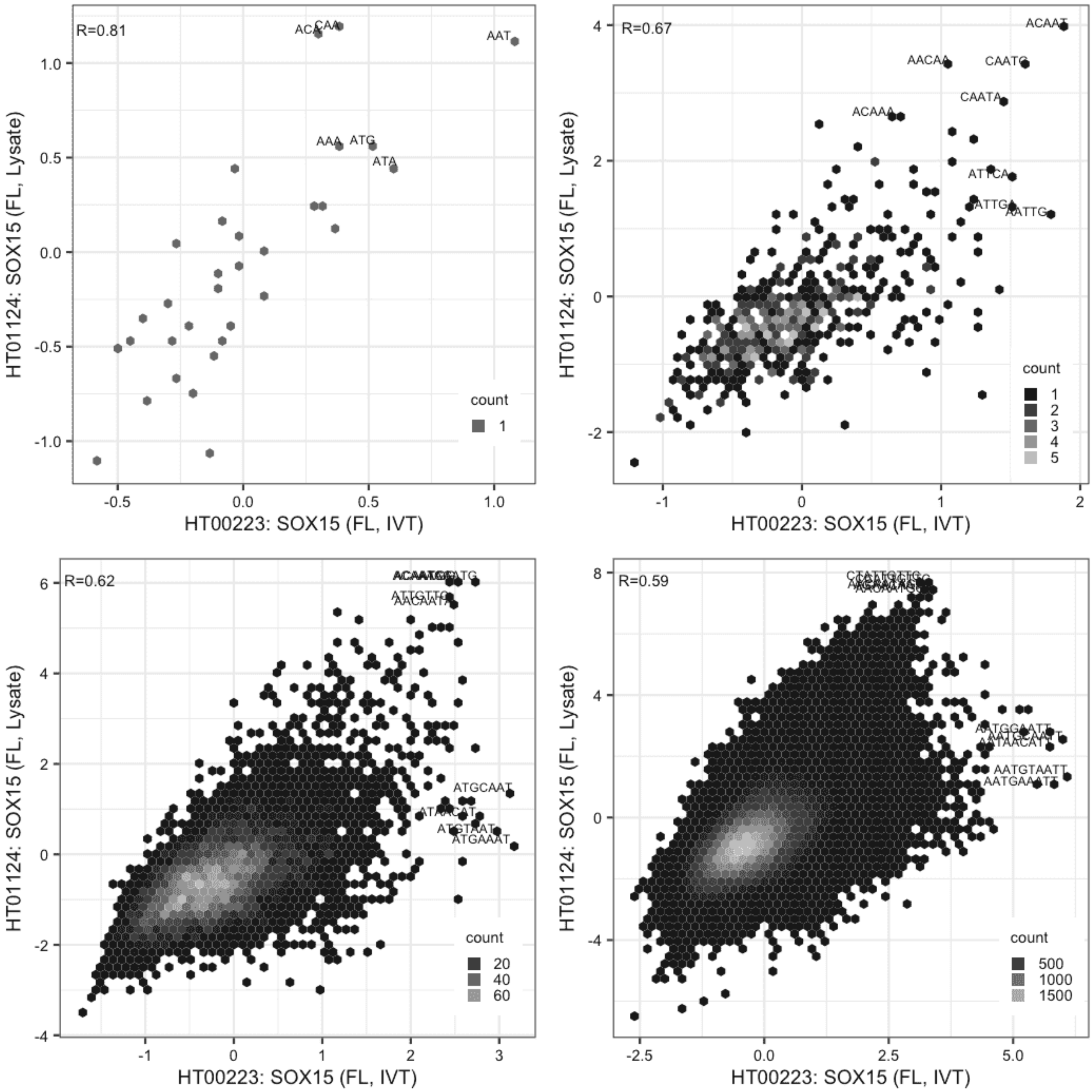

SOX15

Lysate vs Lysate, same construct

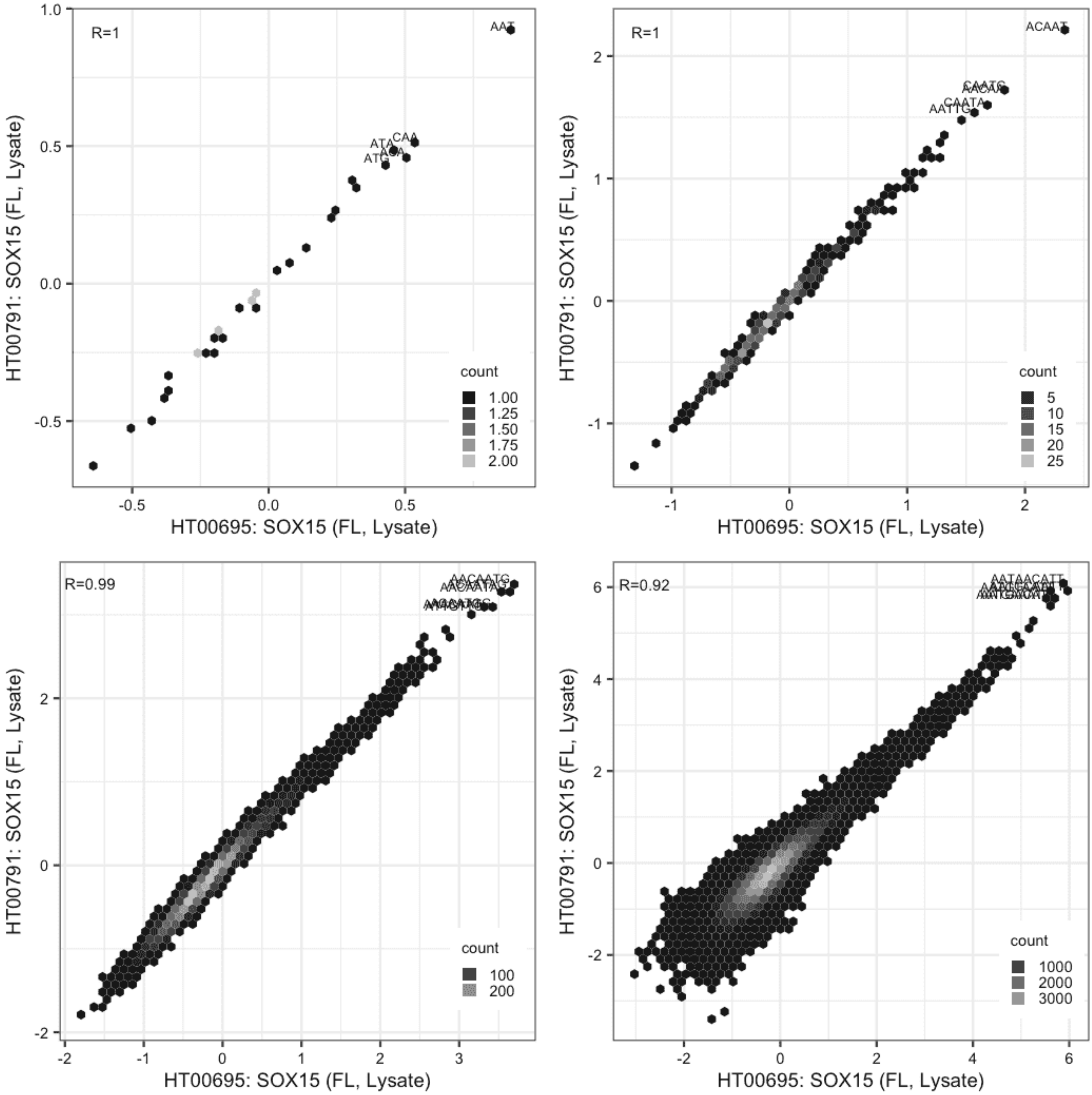

SOX15

Lysate vs Lysate, same construct

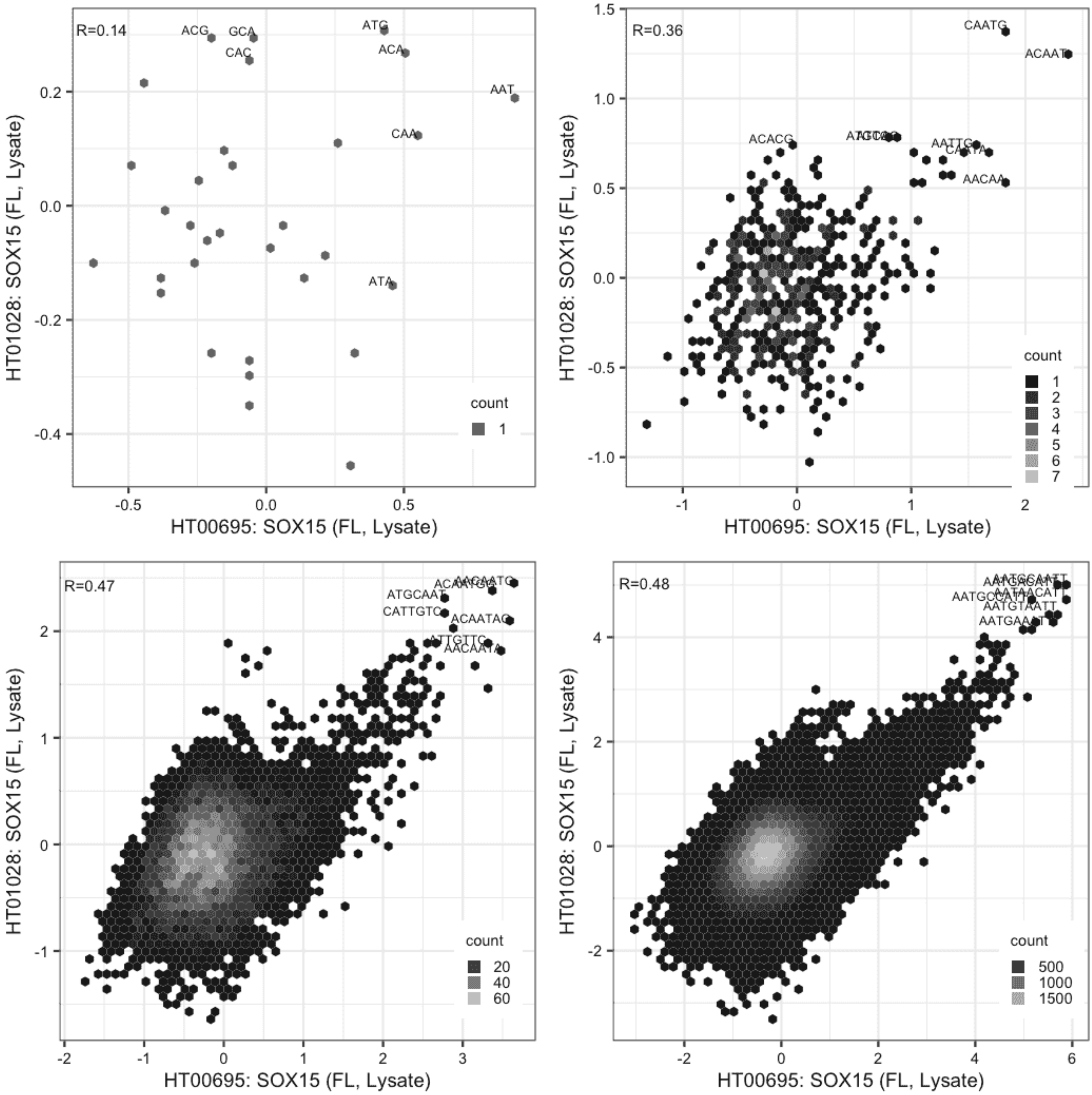

SOX15

Lysate vs Lysate, same construct

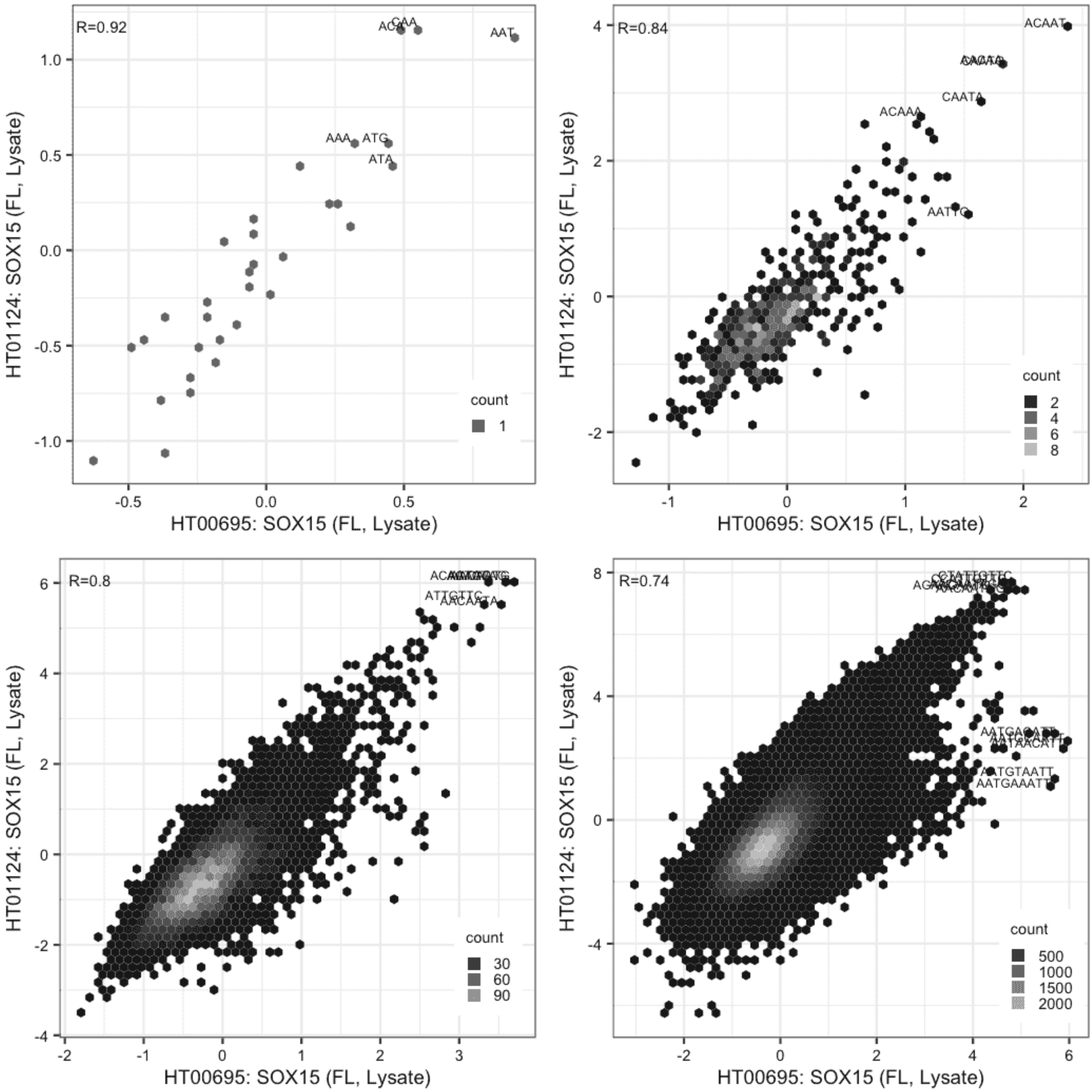

SOX15

Lysate vs Lysate, same construct

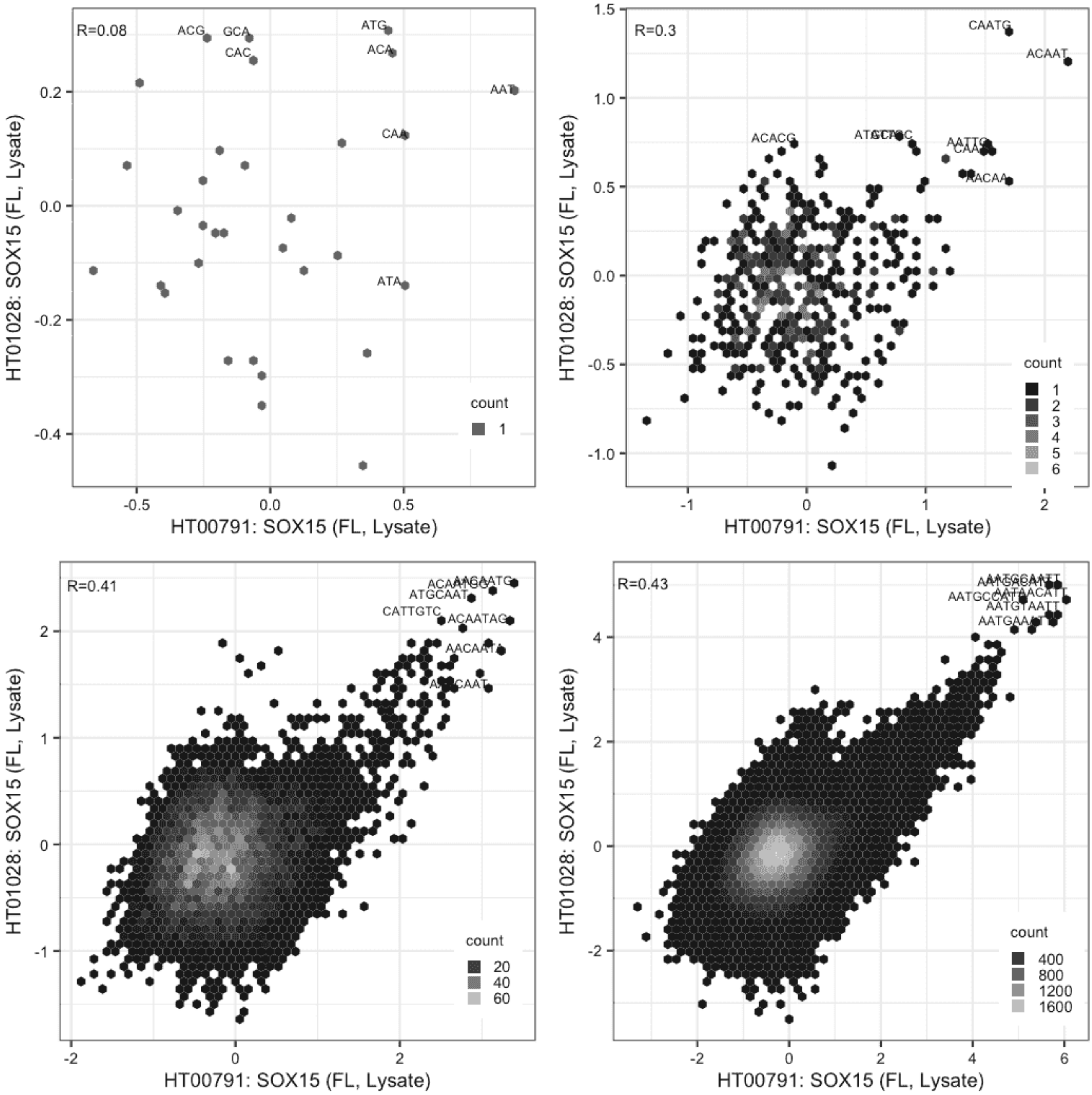

SOX15

Lysate vs Lysate, same construct

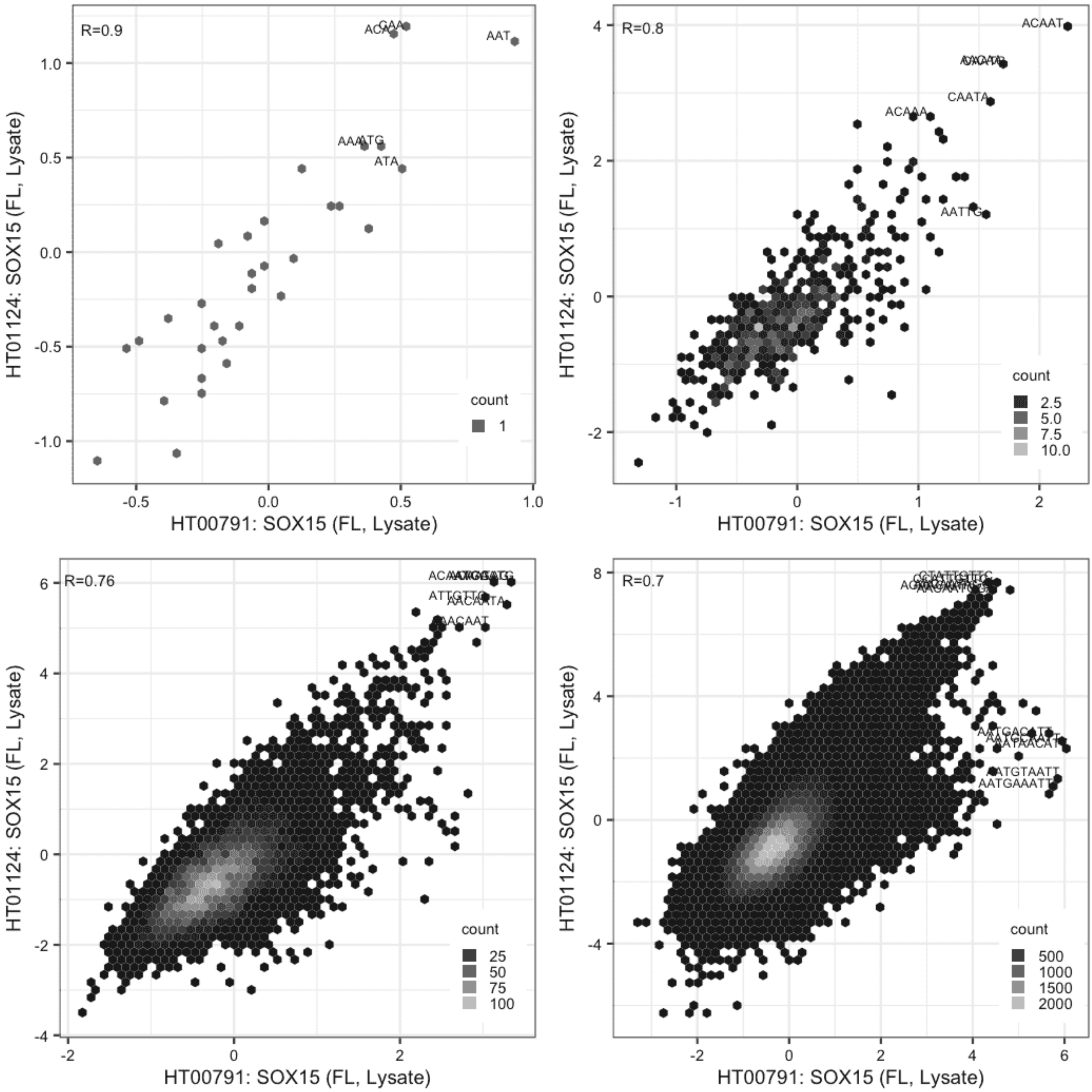

SOX15

Lysate vs Lysate, same construct

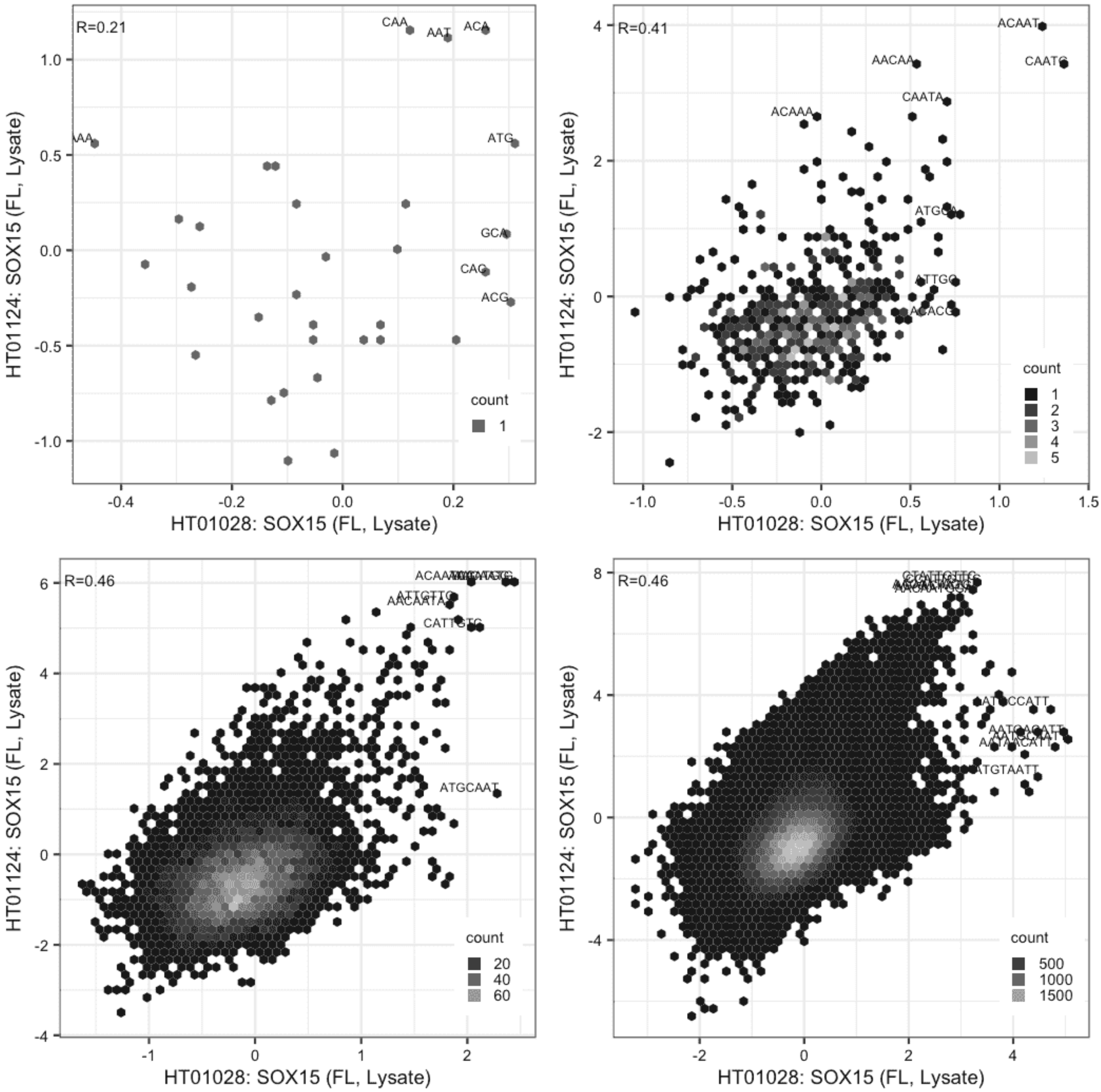

SOX2

IVT vs IVT, same construct

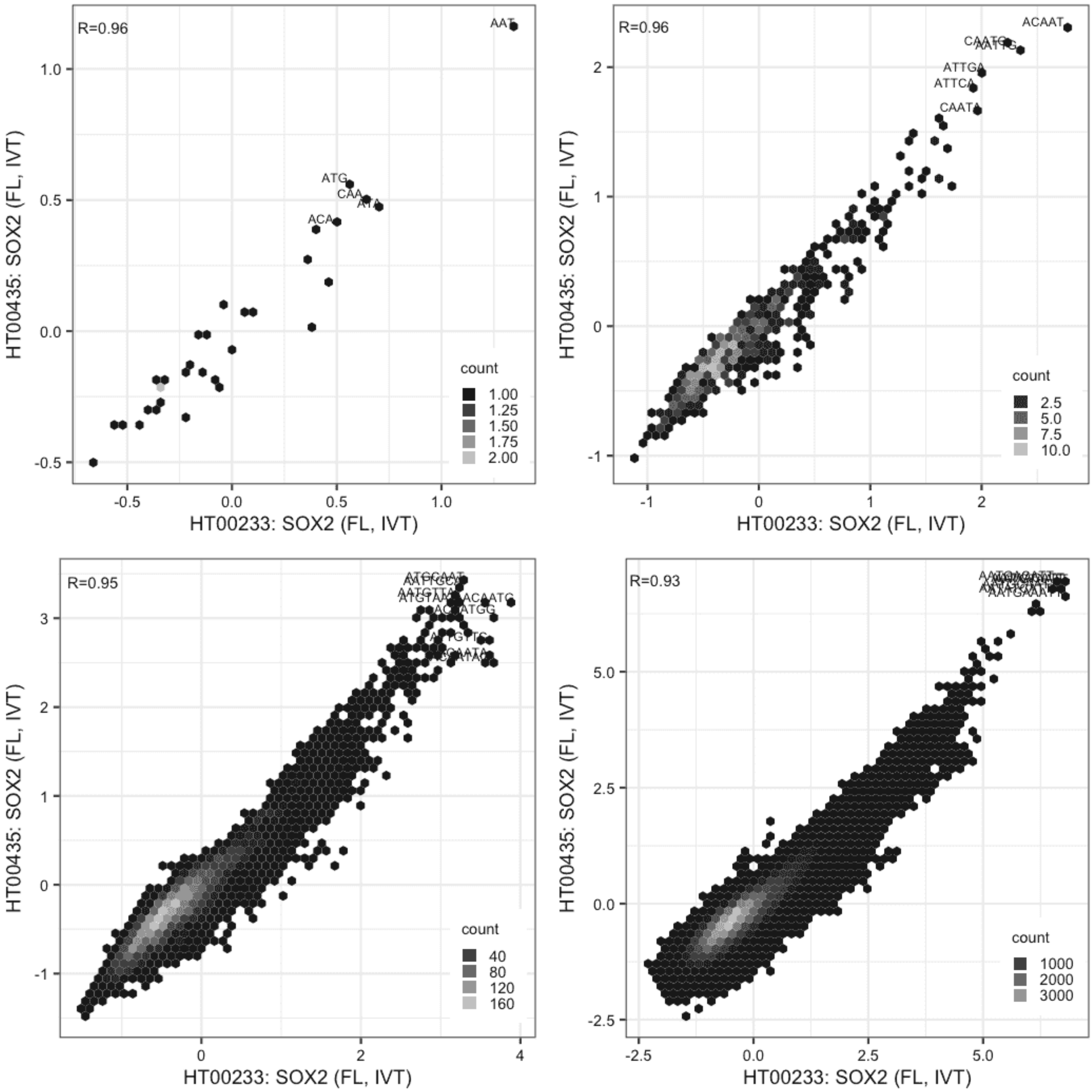

SOX2

Lysate vs IVT, same construct

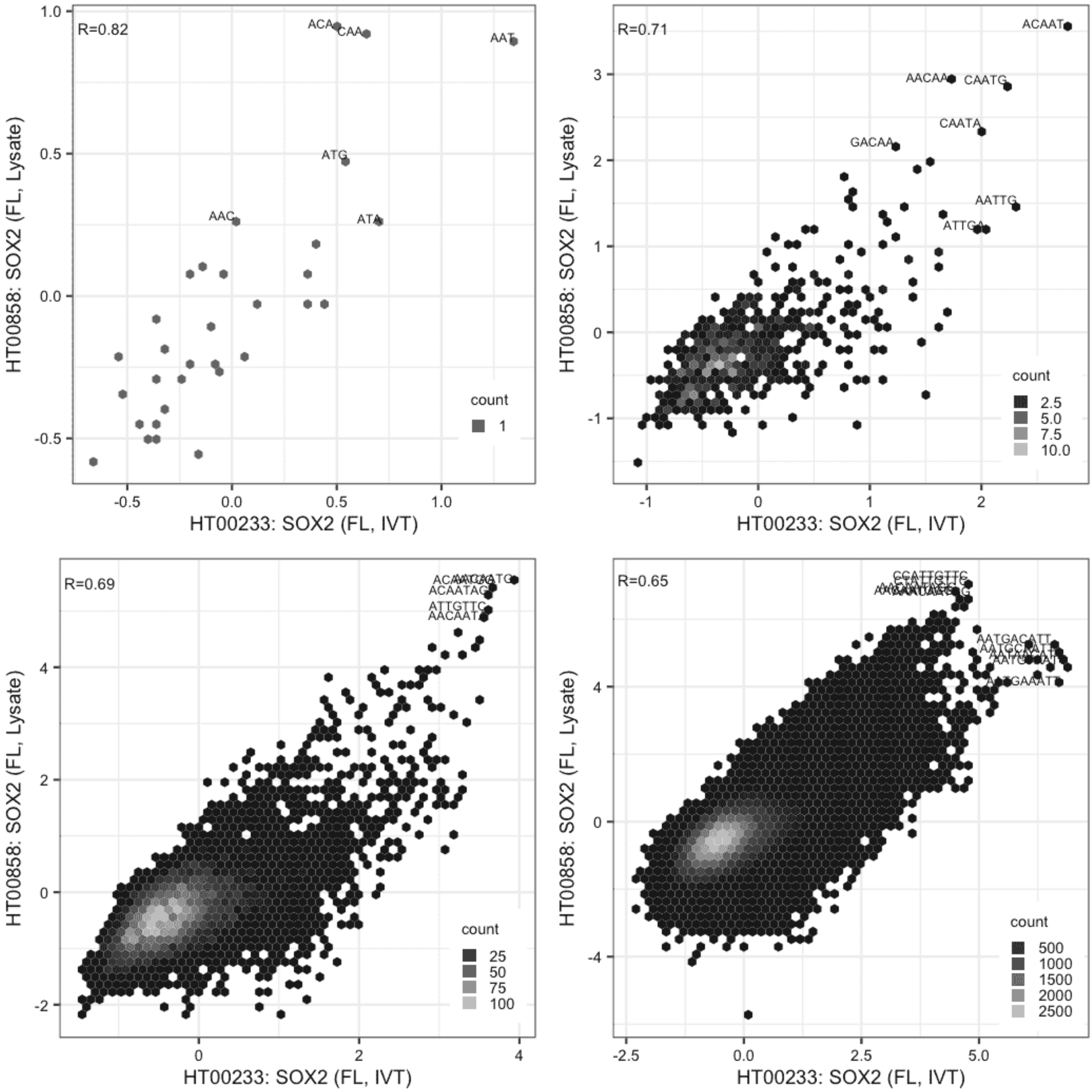

SOX2

Lysate vs IVT, same construct

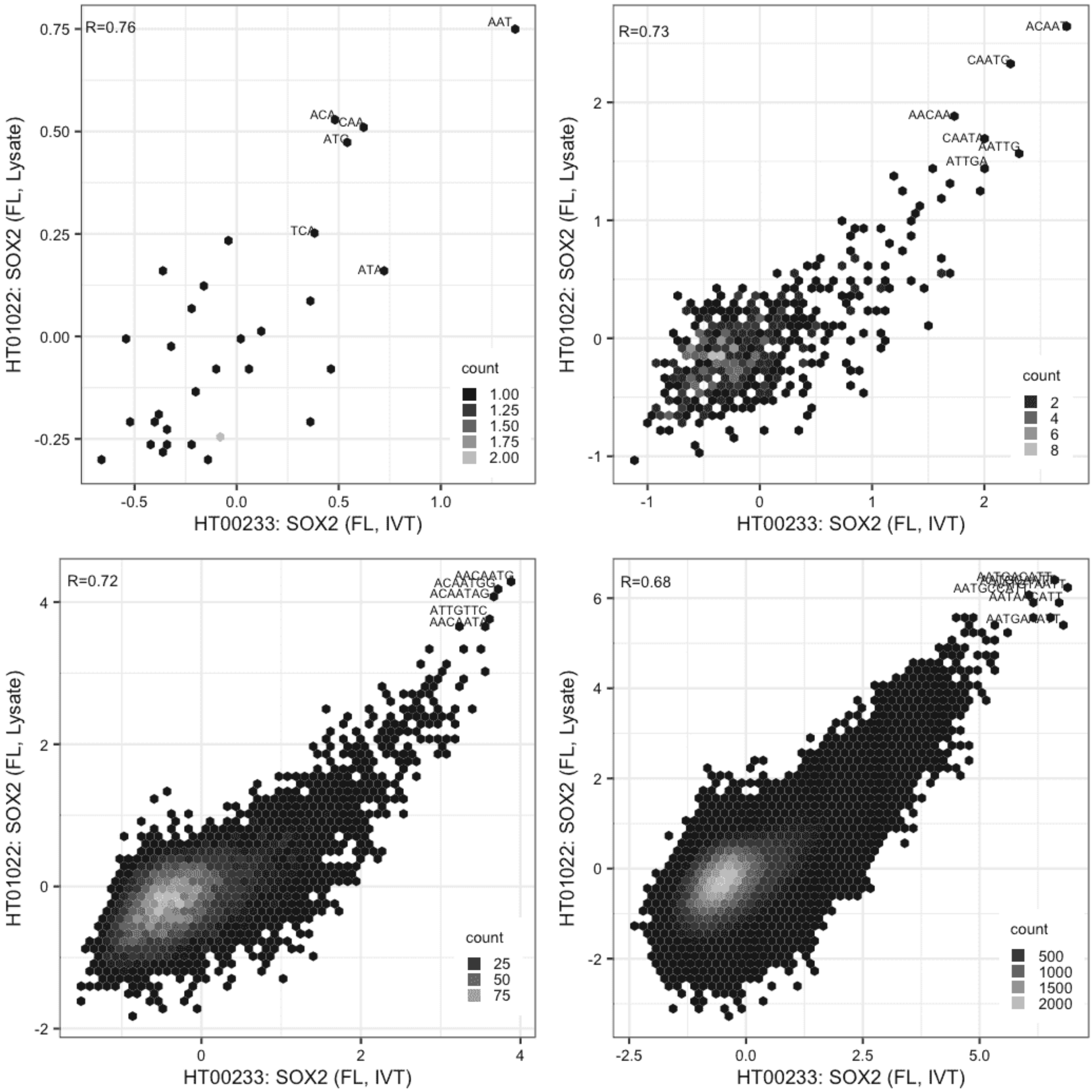

SOX2

Lysate vs IVT, same construct

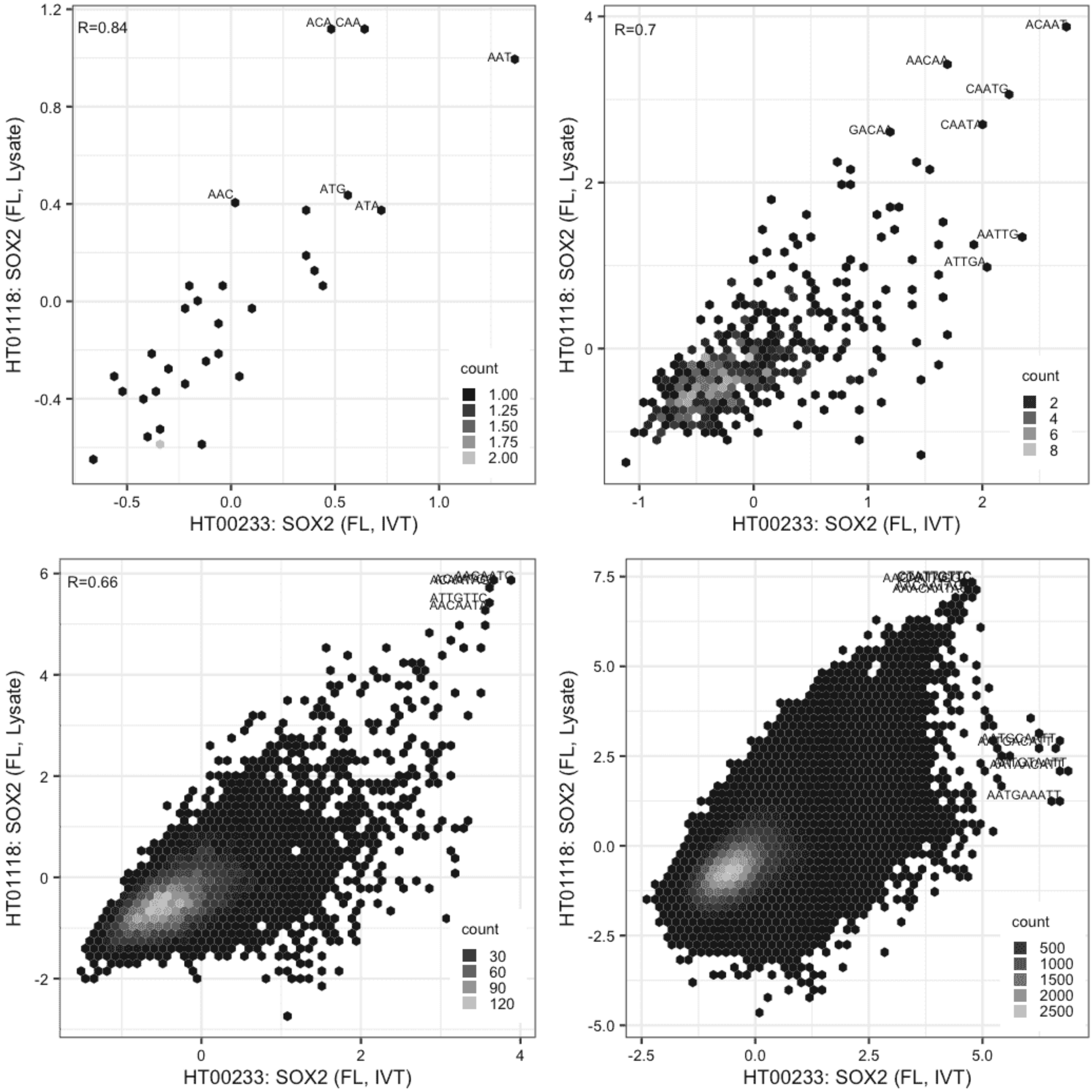

SOX2

Lysate vs IVT, same construct

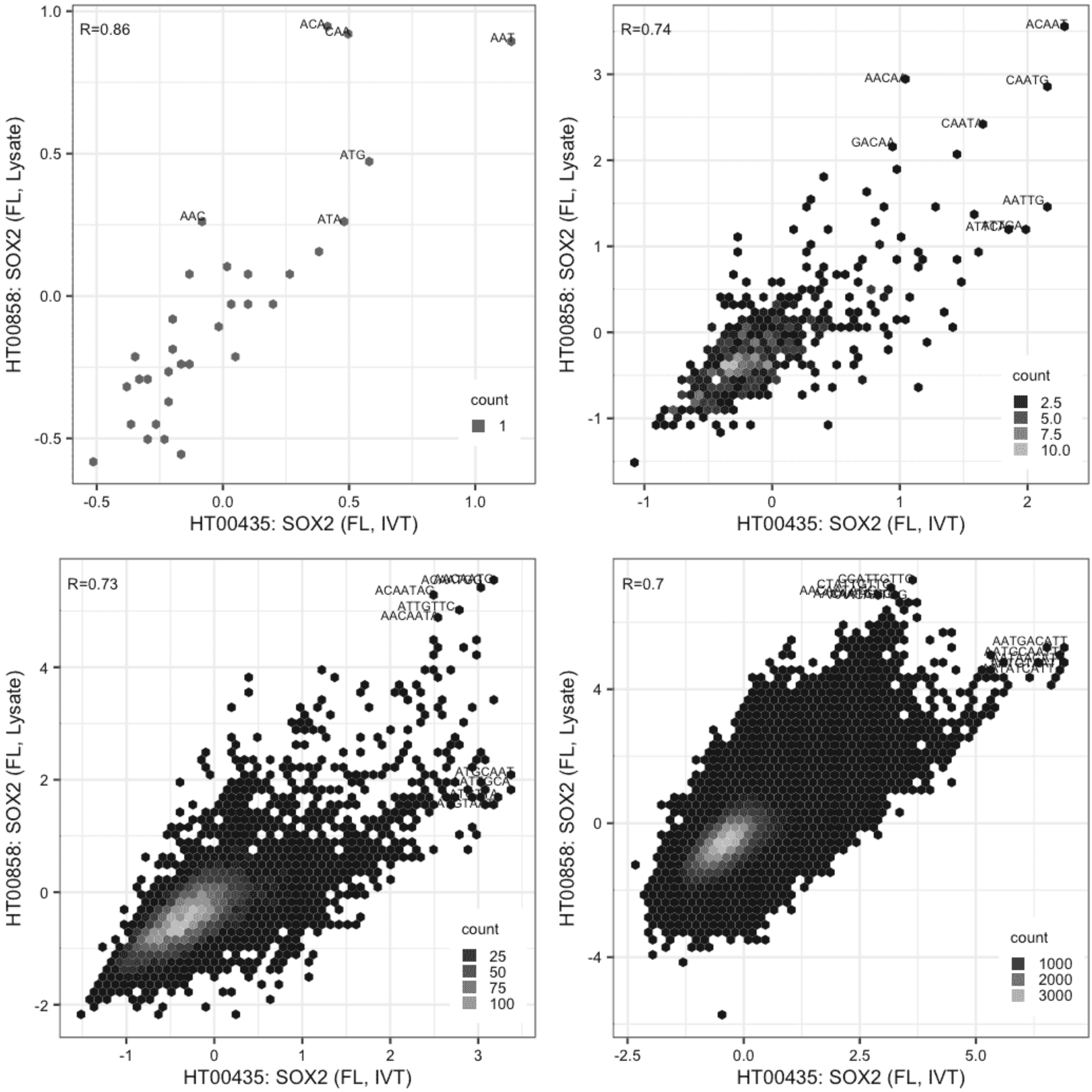

SOX2

Lysate vs IVT, same construct

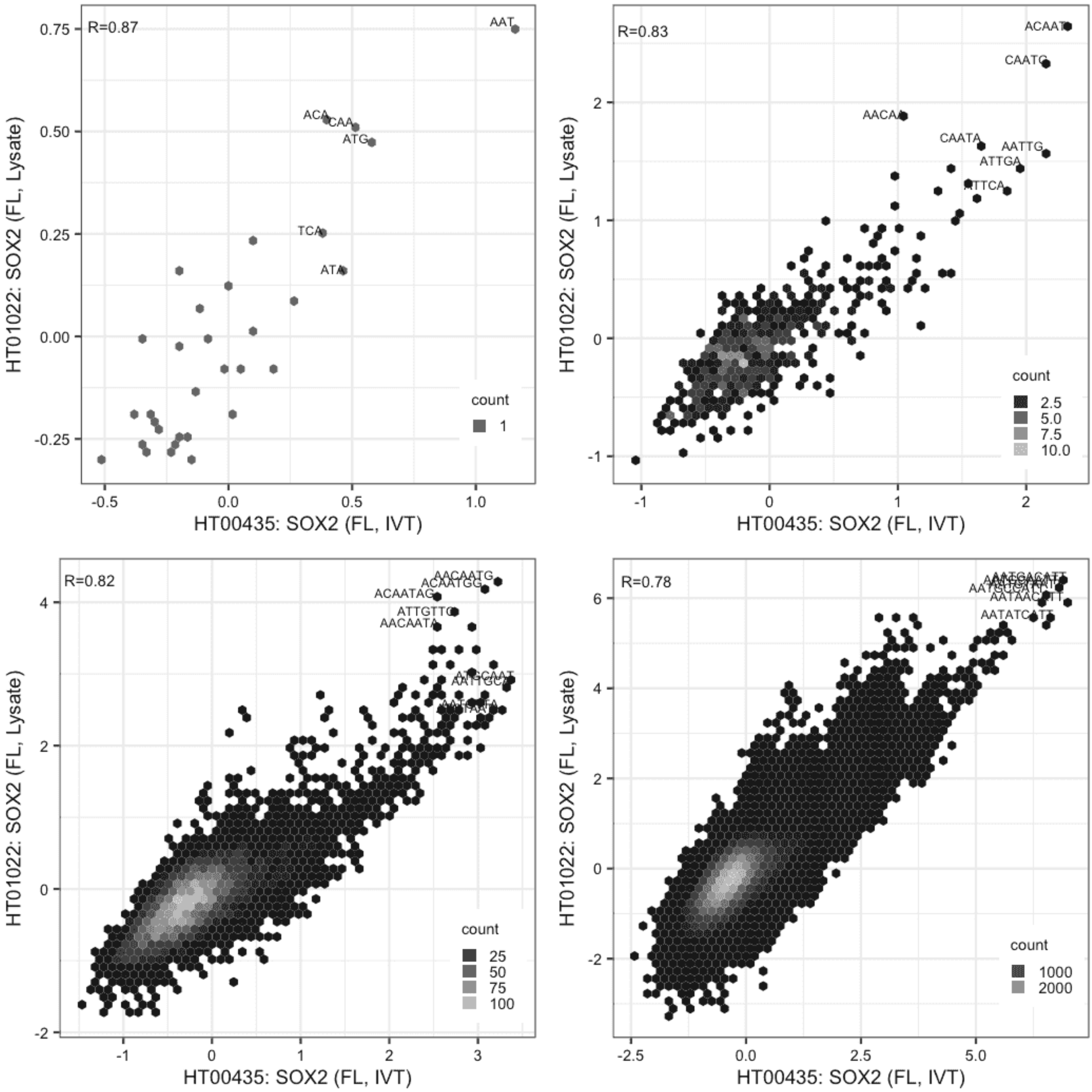

SOX2

Lysate vs IVT, same construct

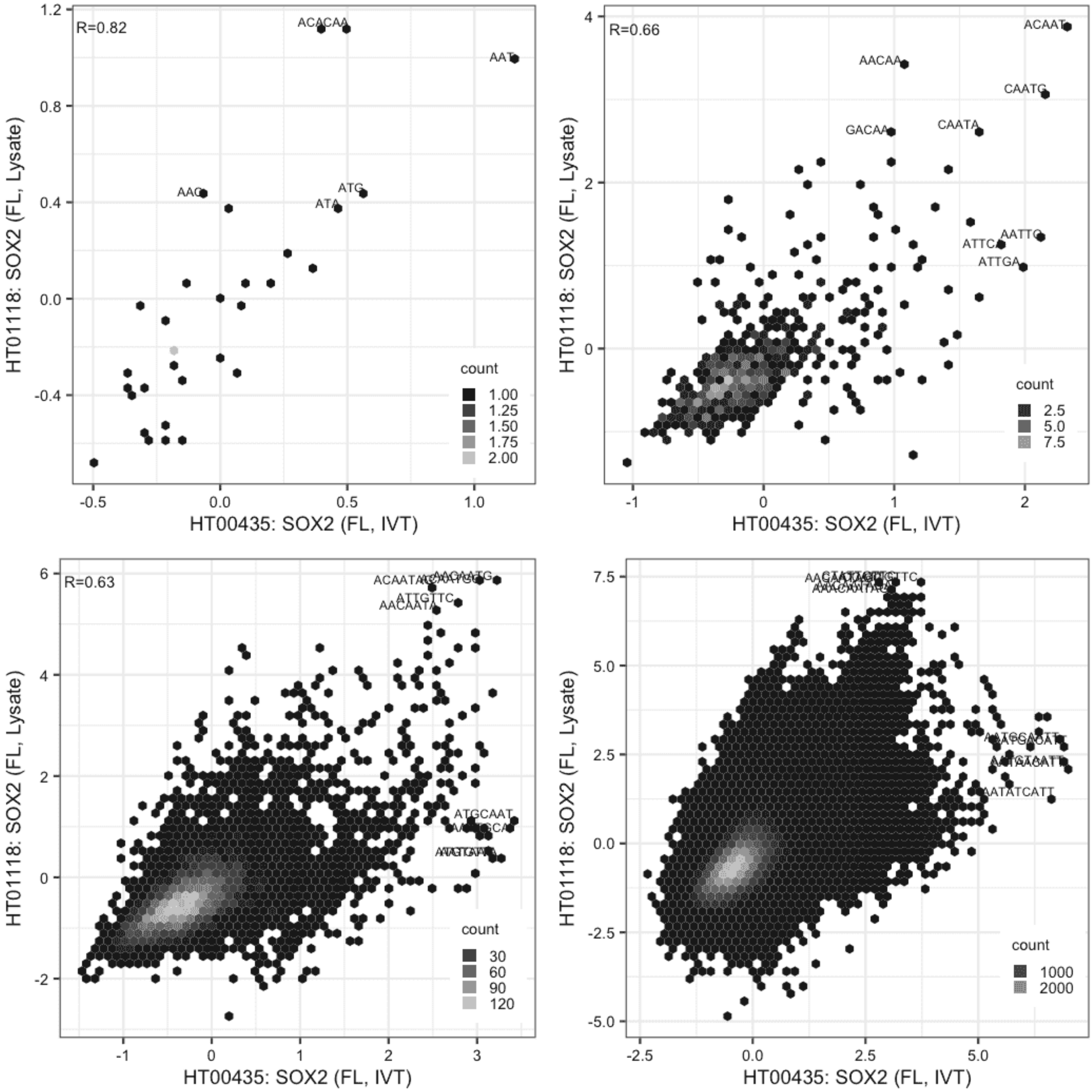

SOX2

Lysate vs Lysate, same construct

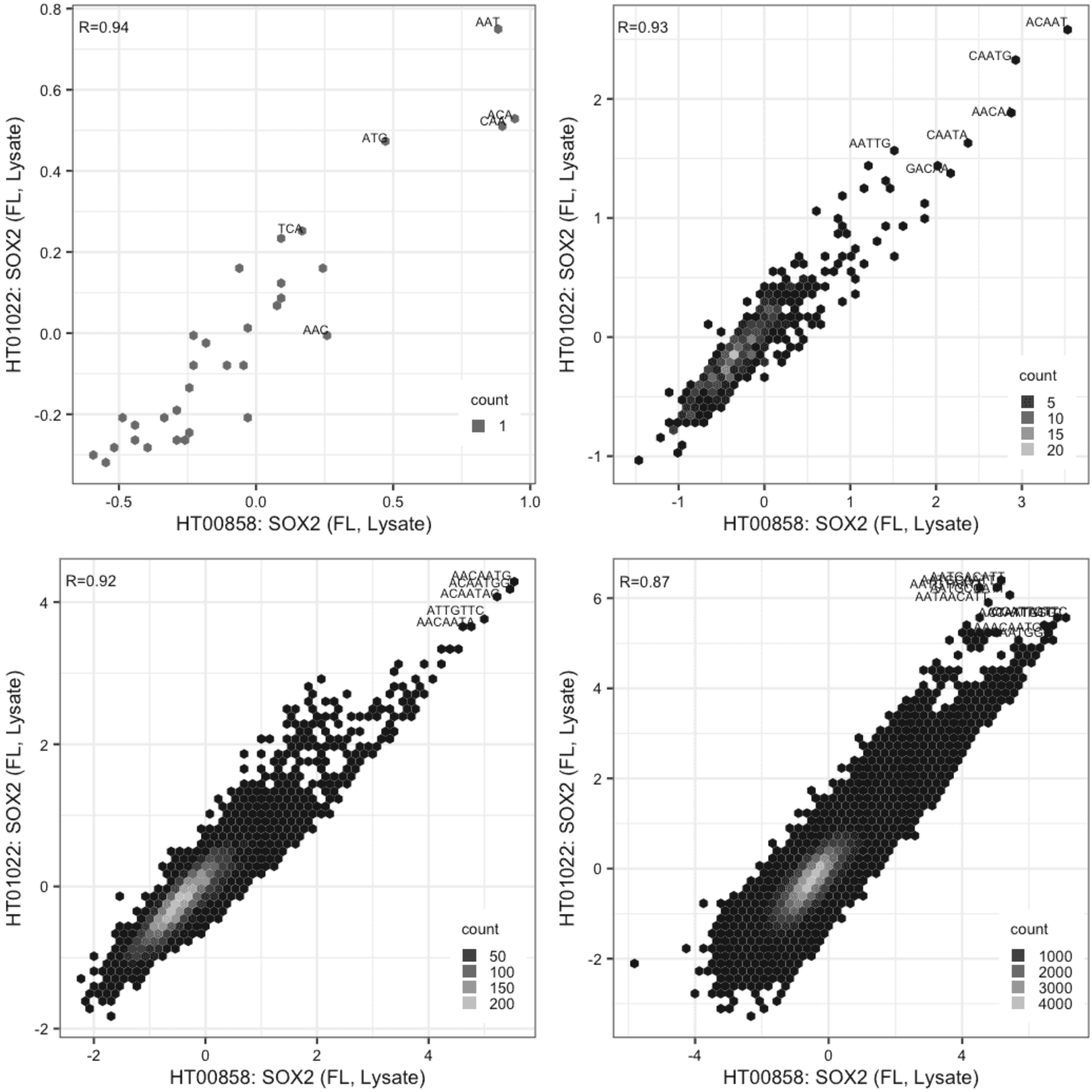

SOX2

Lysate vs Lysate, same construct

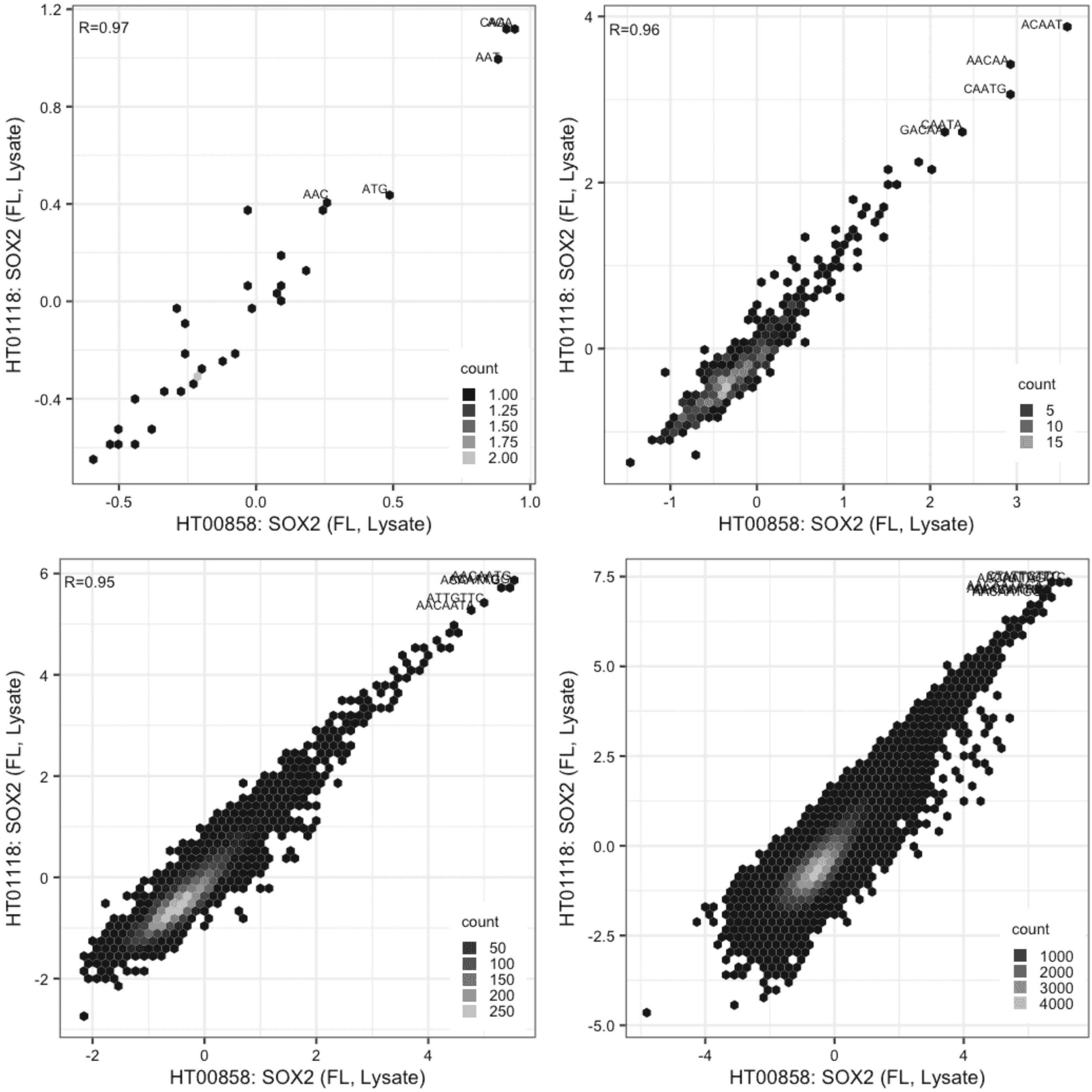

SOX2

Lysate vs Lysate, same construct

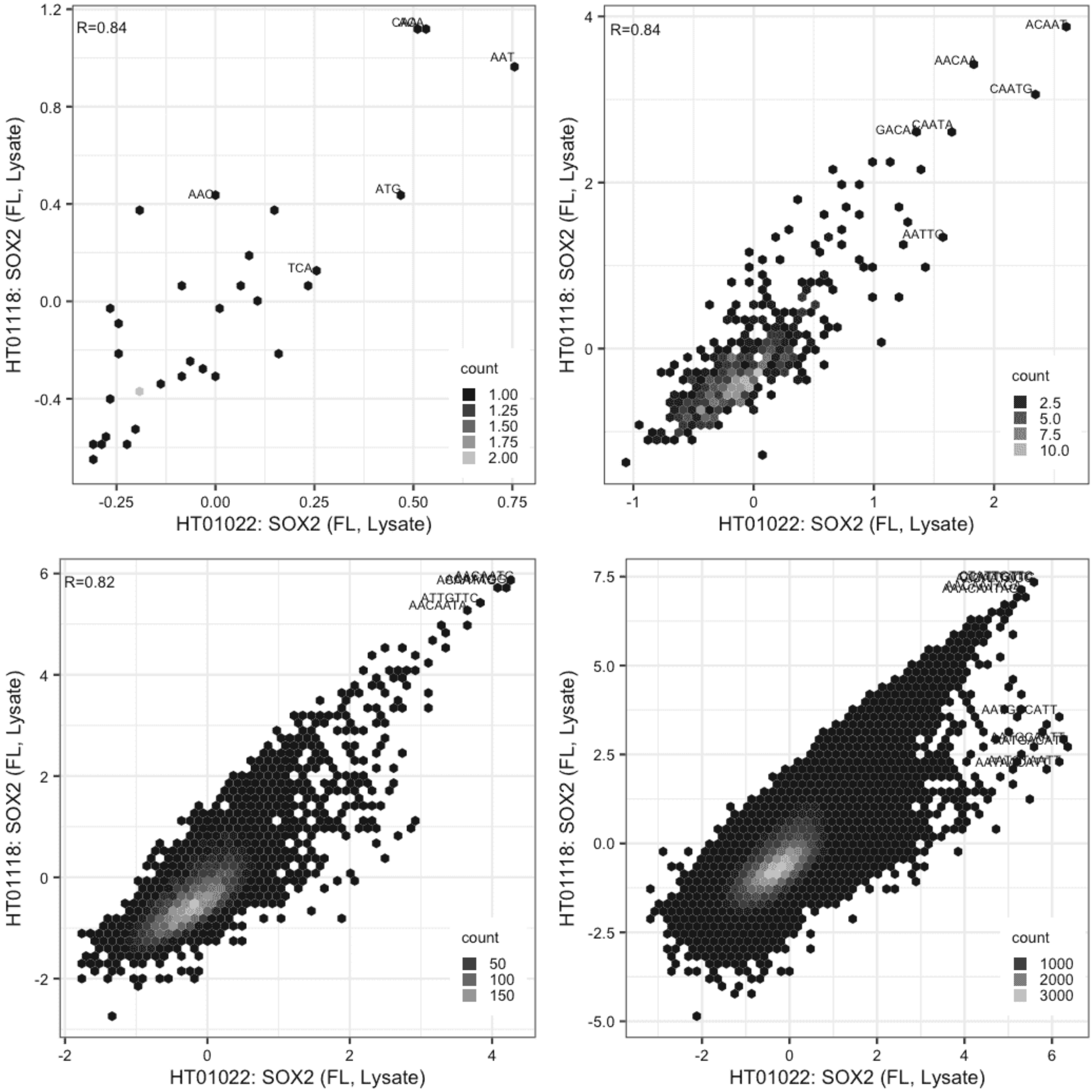

SP140L

IVT vs IVT, different constructs

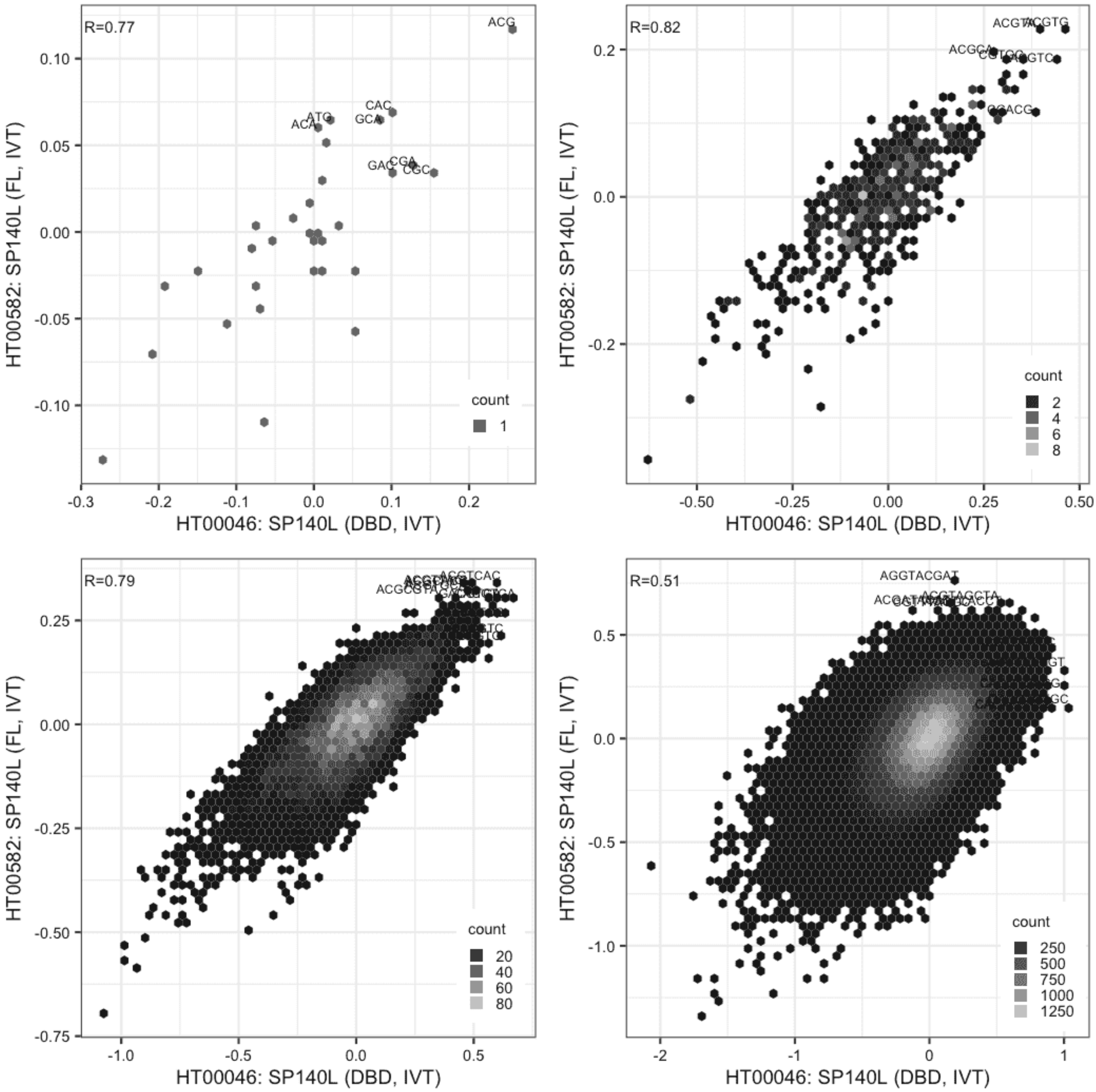

SP140L

Lysate vs IVT, different constructs

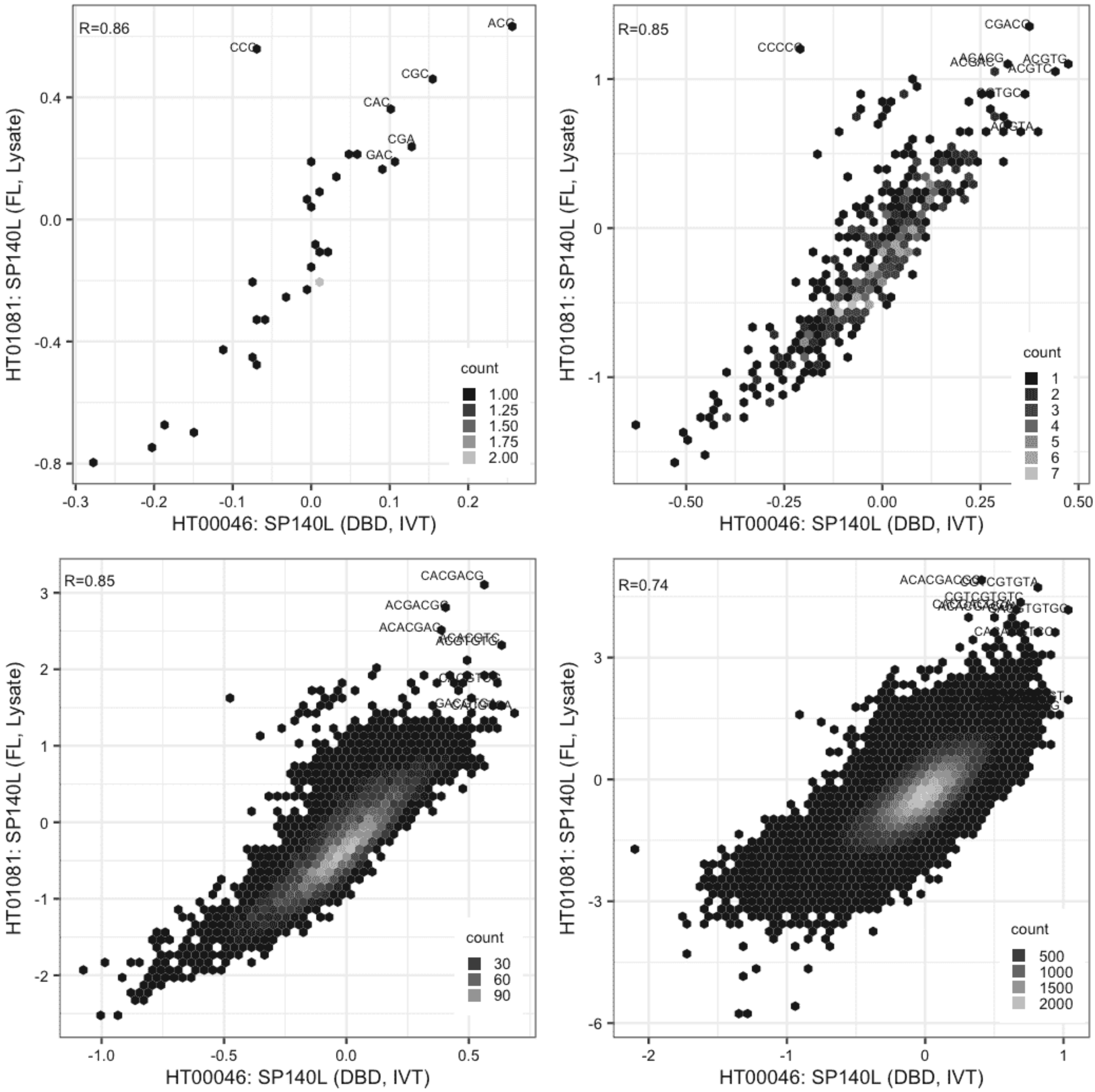

SP140L

Lysate vs IVT, different constructs

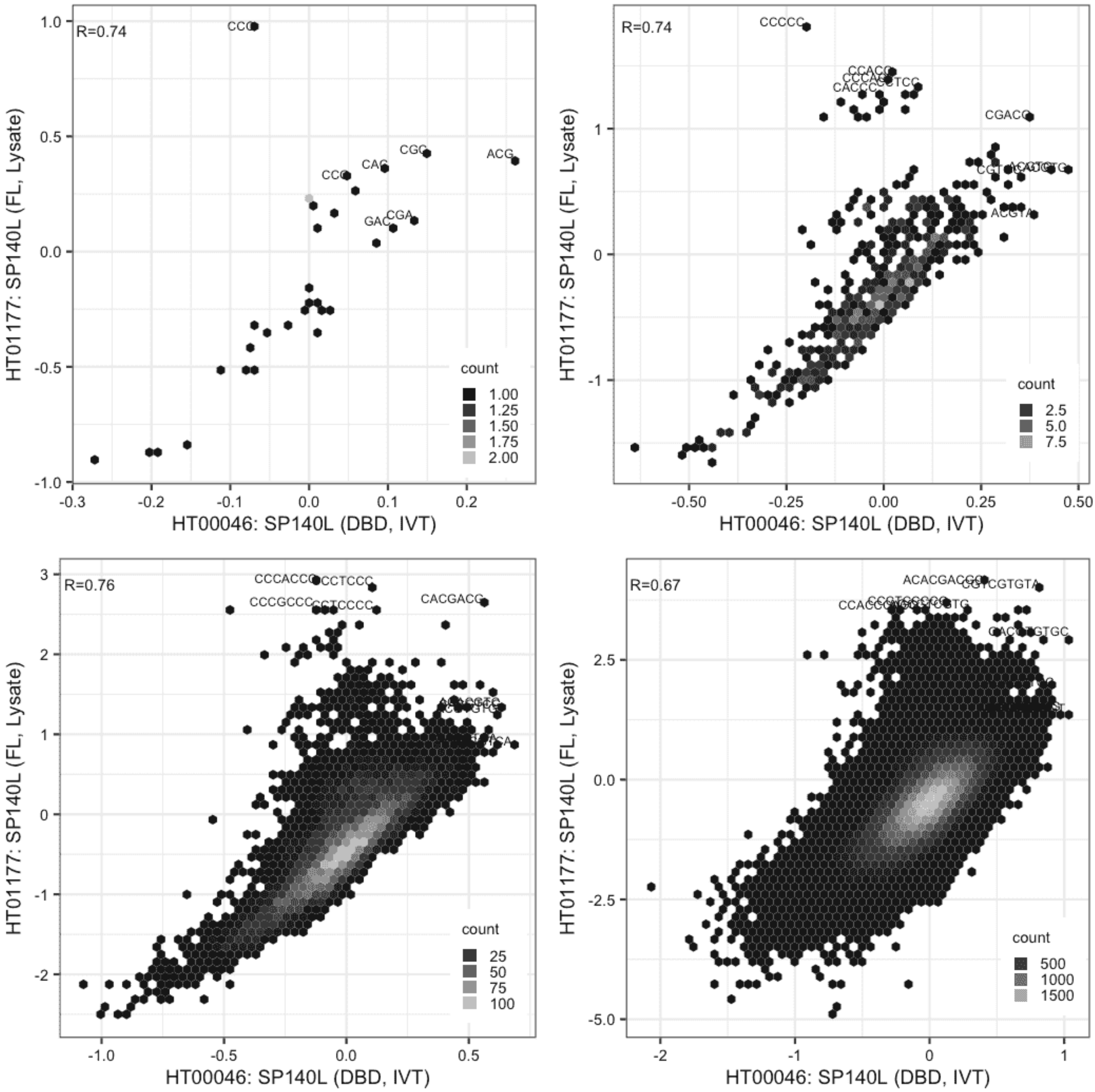

SP140L

IVT vs GFPIVT, different constructs

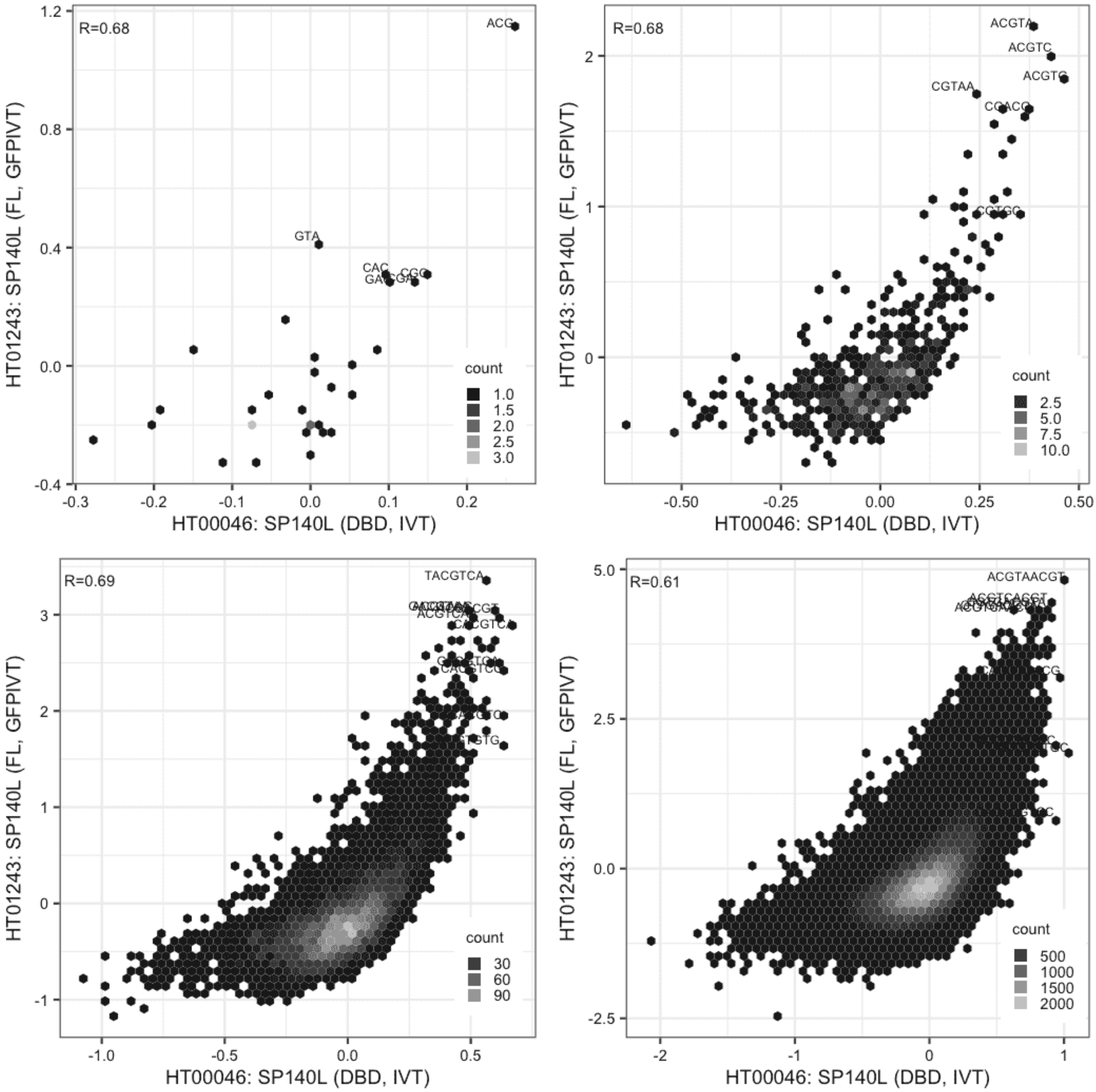

SP140L

IVT vs GFPIVT, same construct

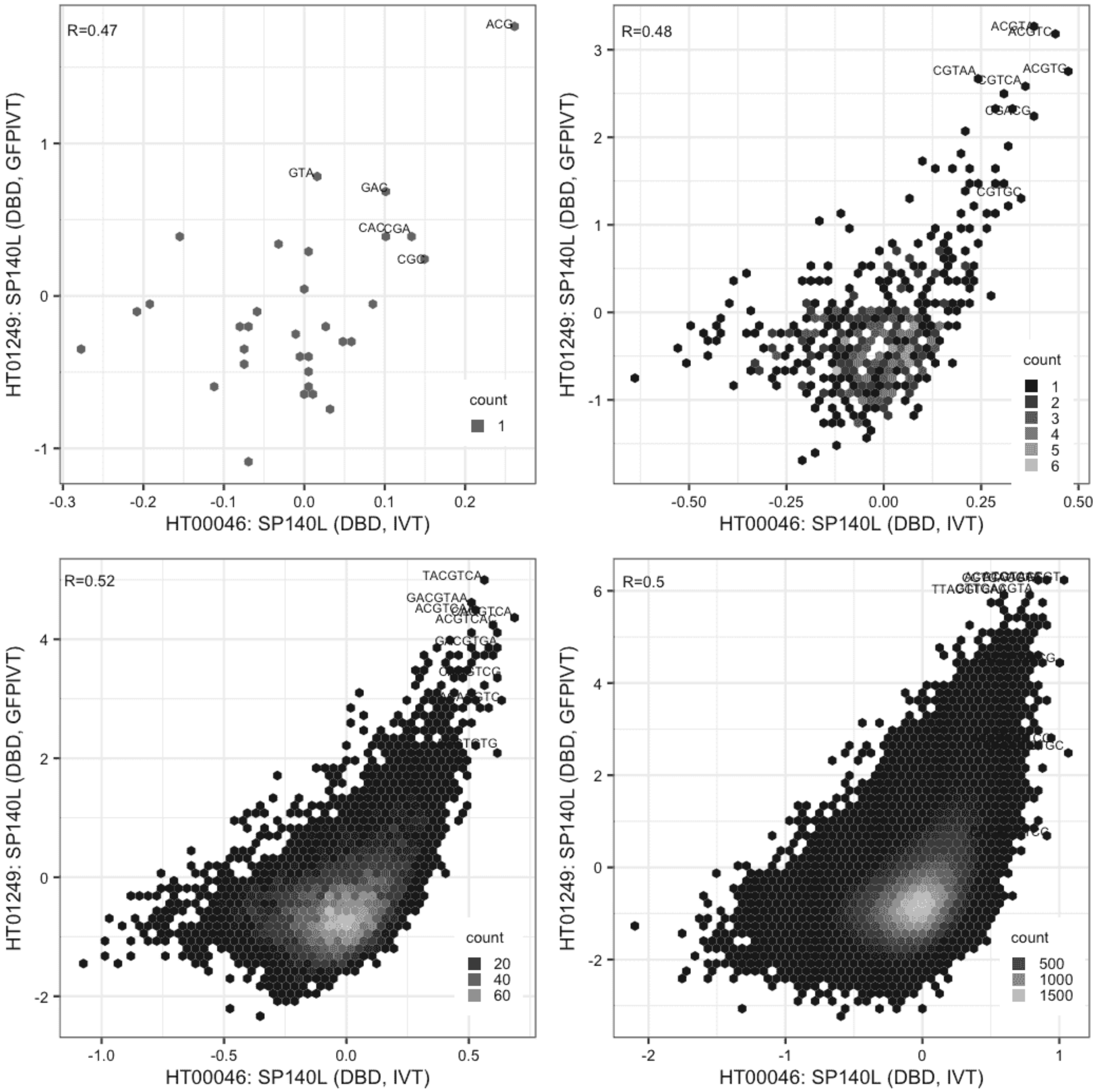

SP140L

Lysate vs IVT, same construct

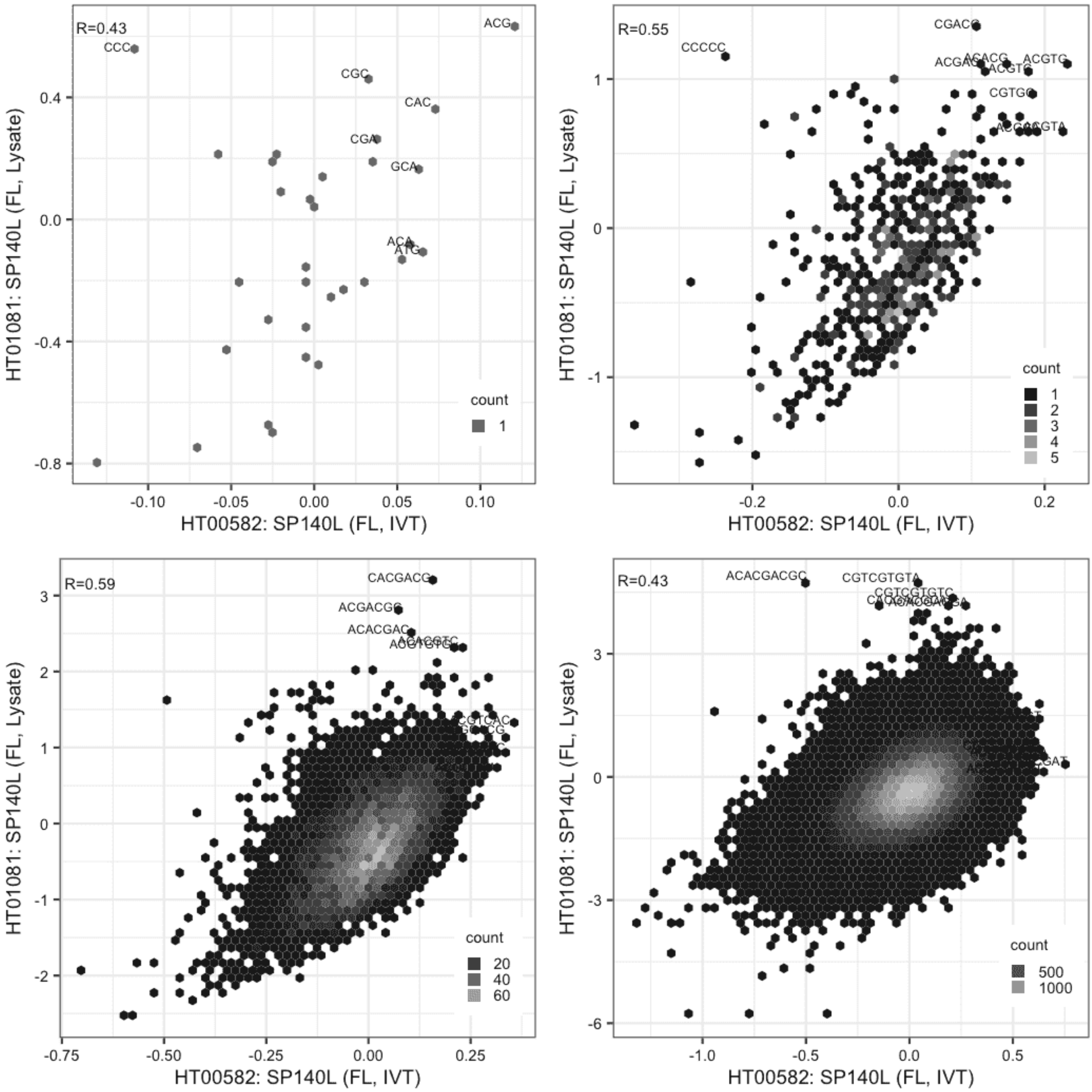

SP140L

Lysate vs IVT, same construct

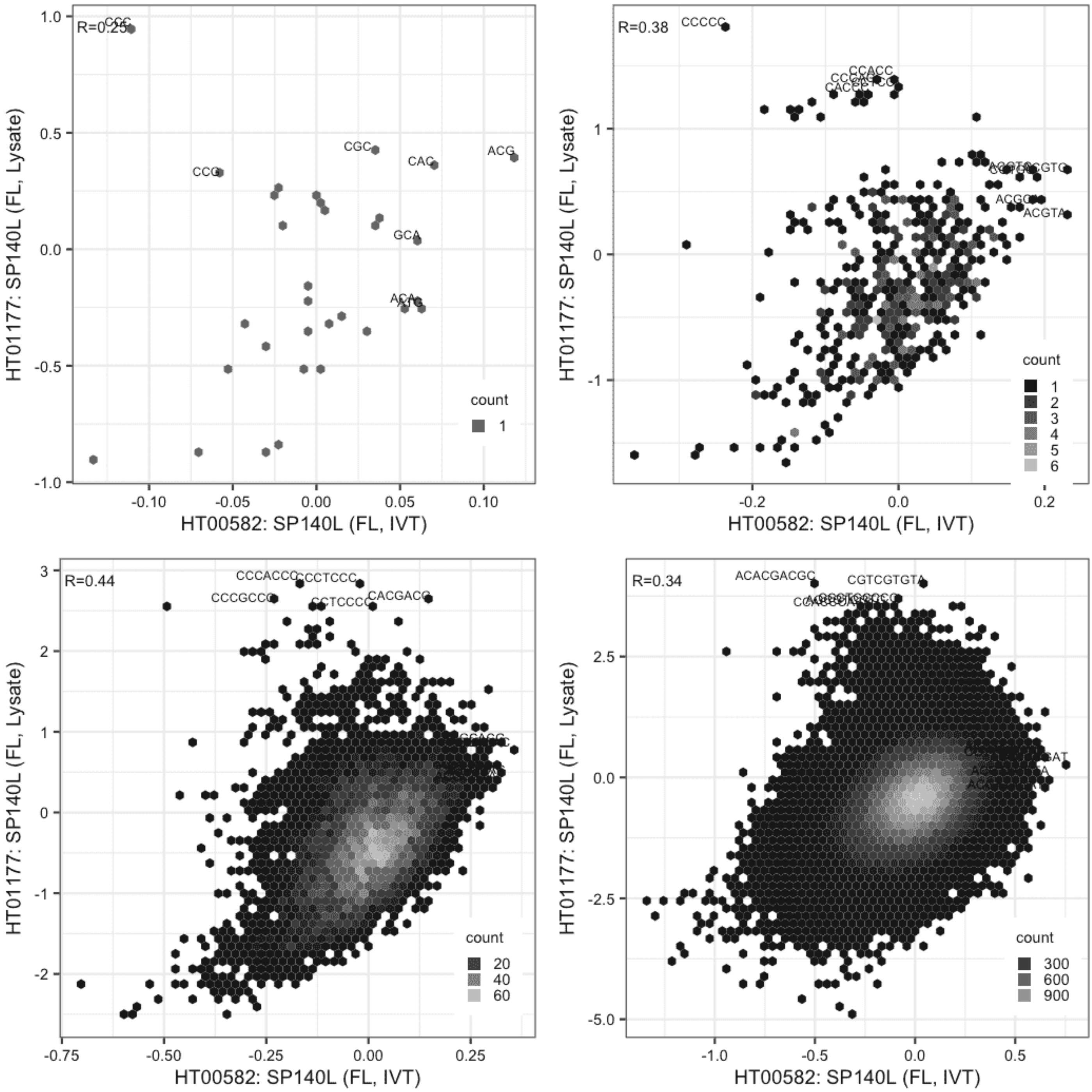

SP140L

IVT vs GFPIVT, same construct

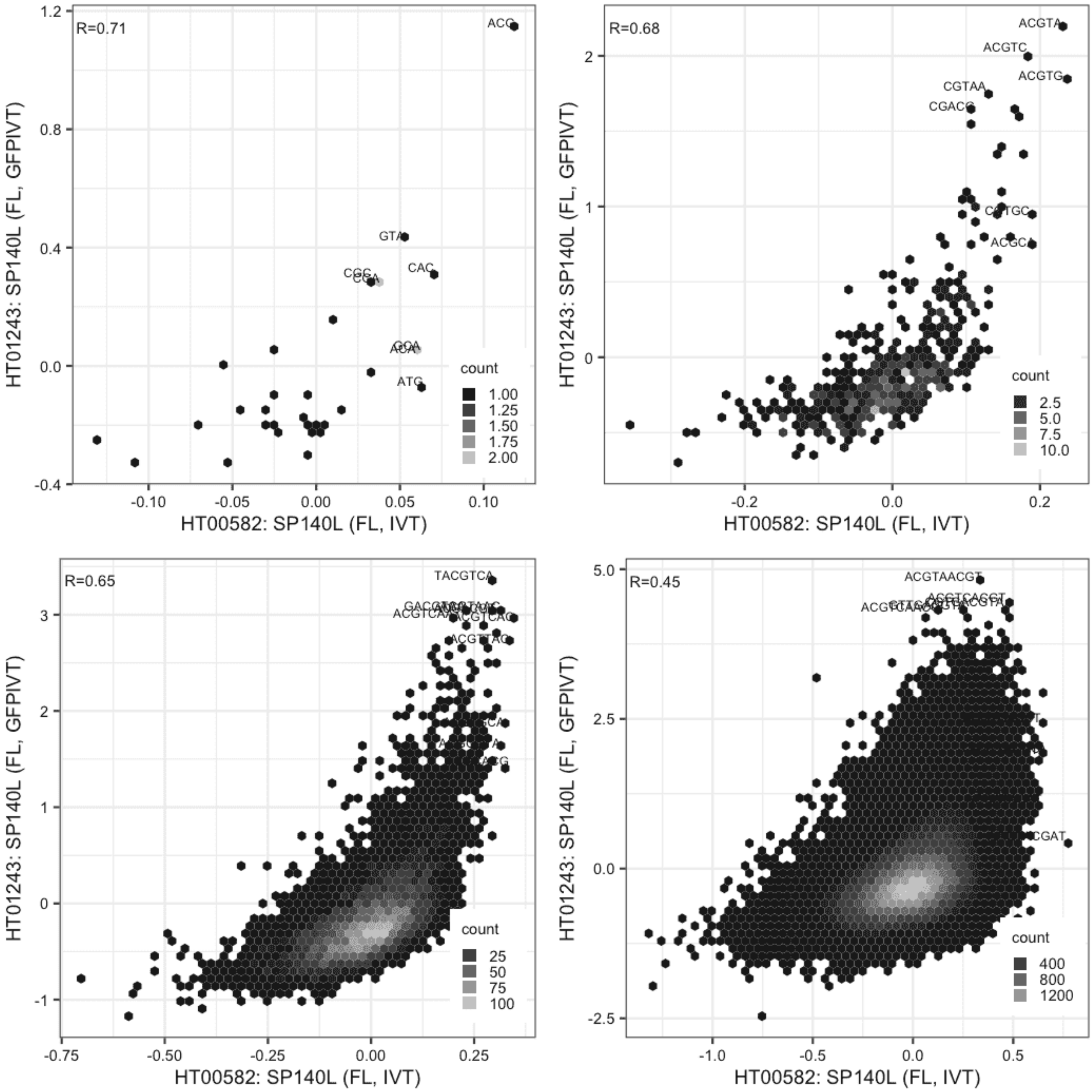

SP140L

IVT vs GFPIVT, different constructs

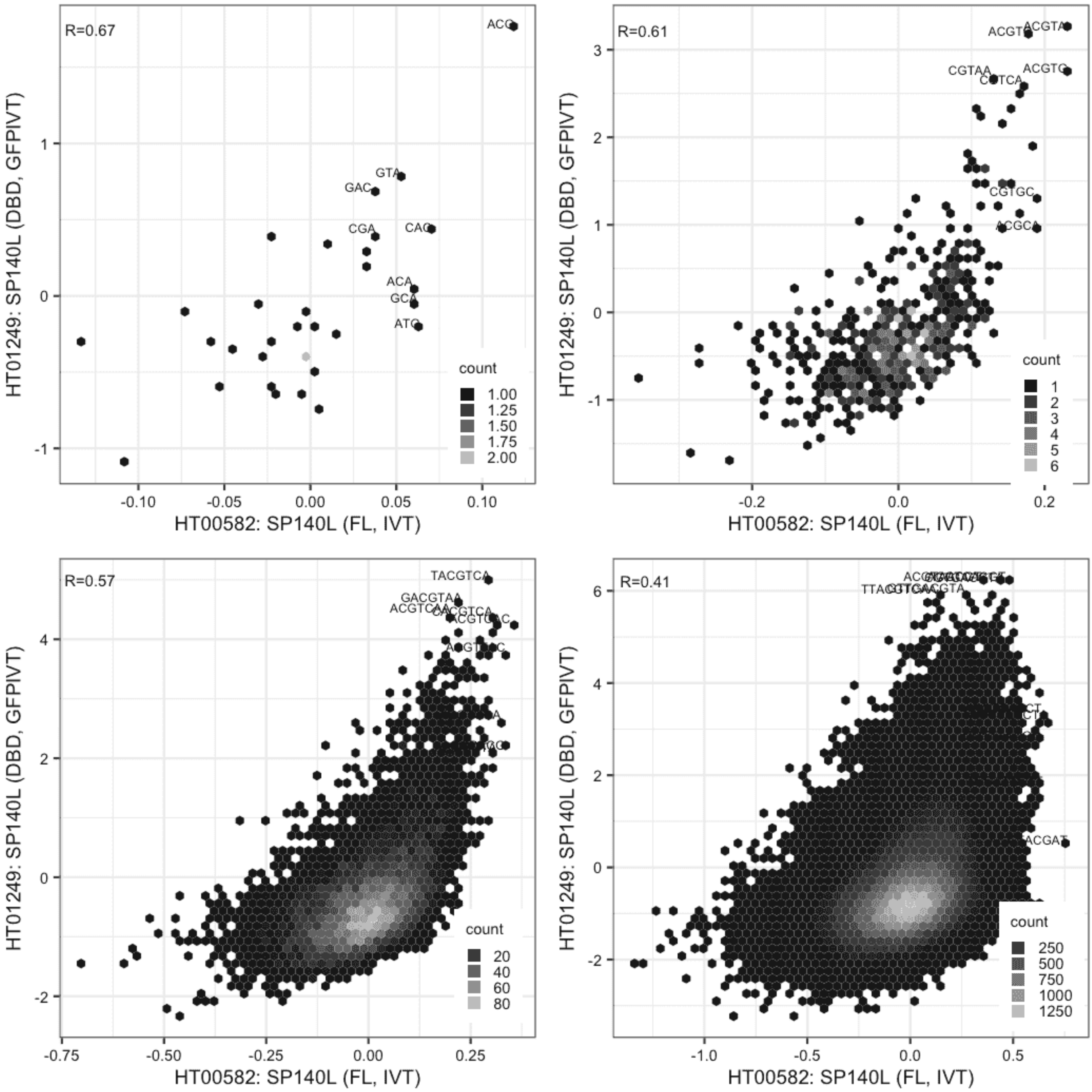

SP140L

Lysate vs Lysate, same construct

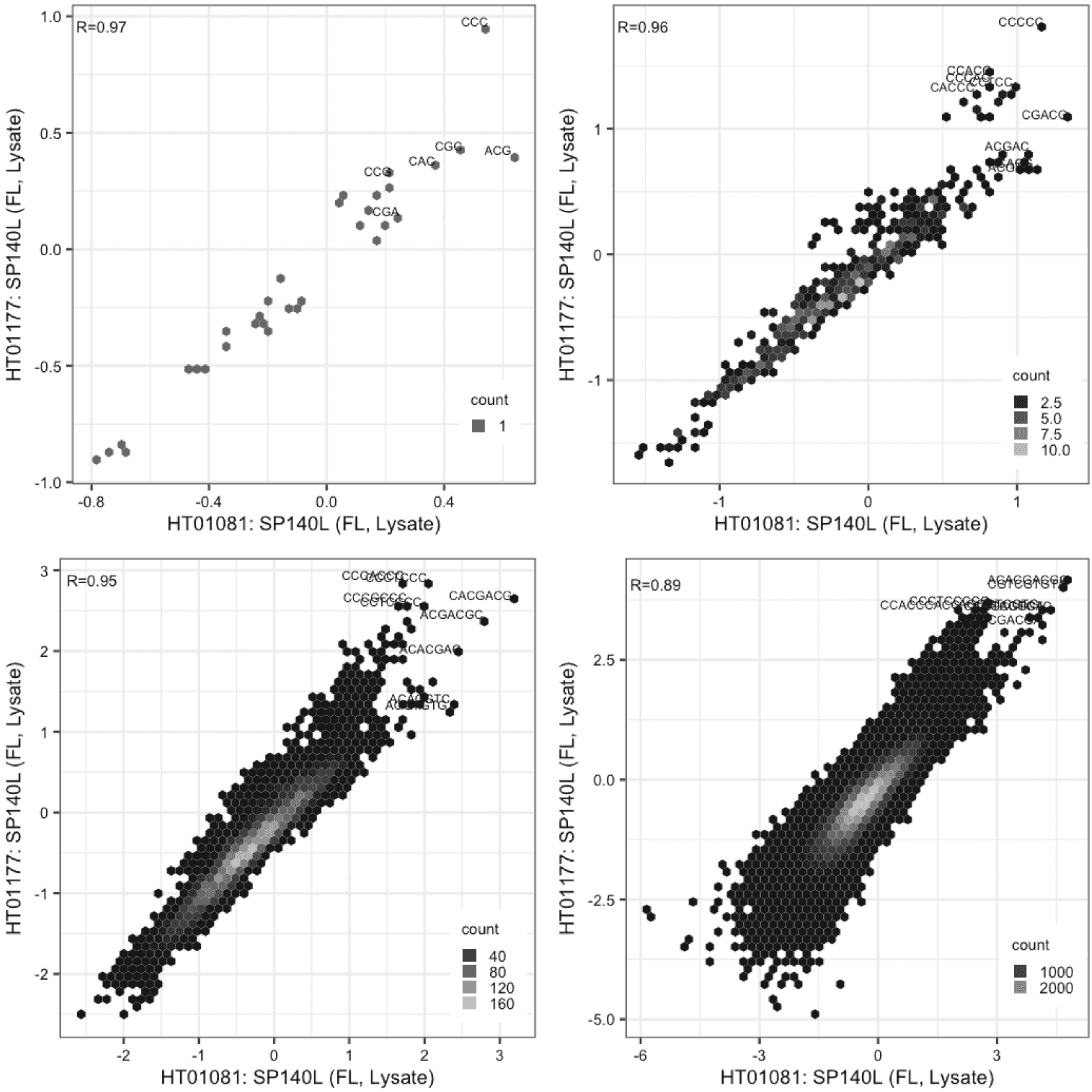

SP140L

Lysate vs GFPIVT, same construct

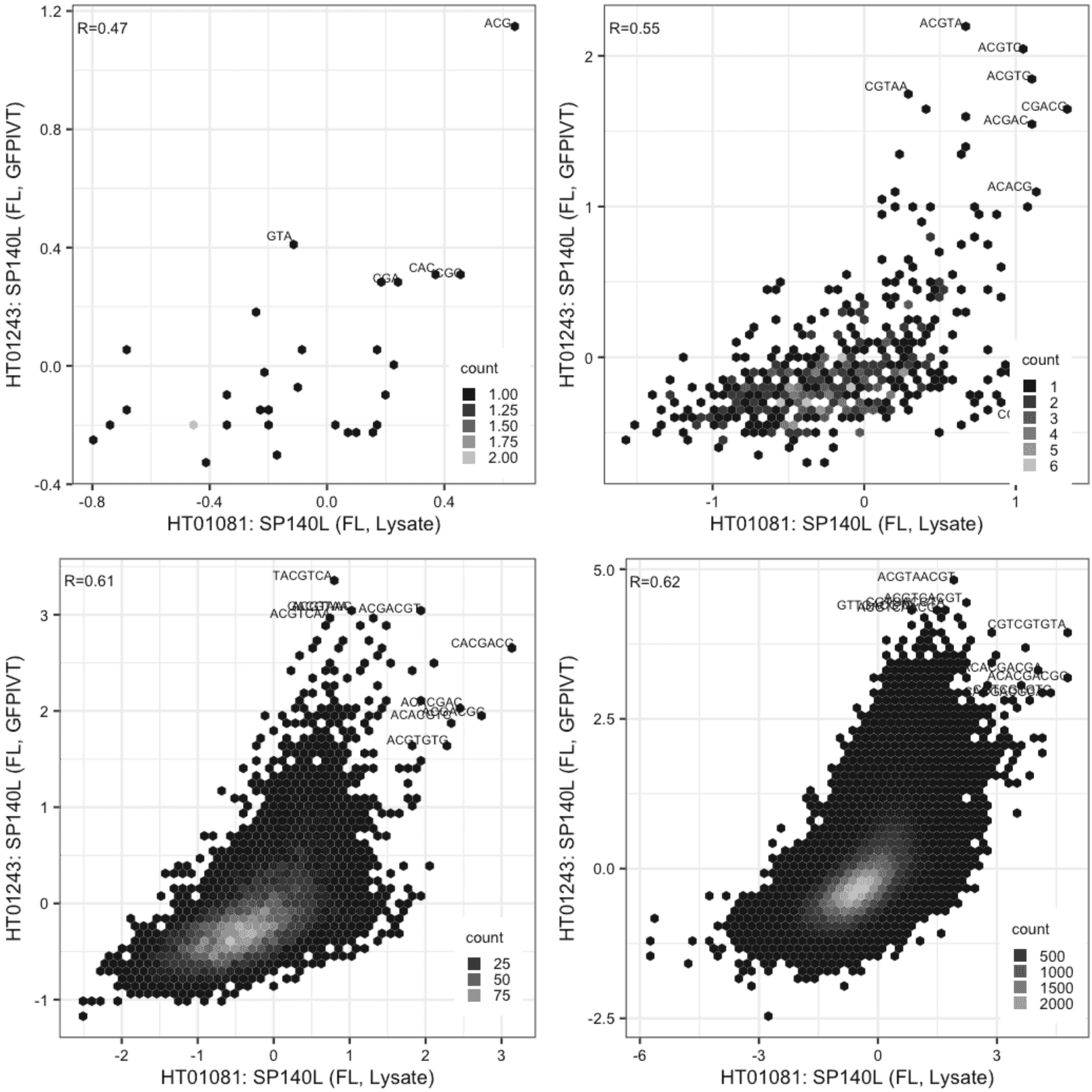

SP140L

Lysate vs GFPIVT, different constructs

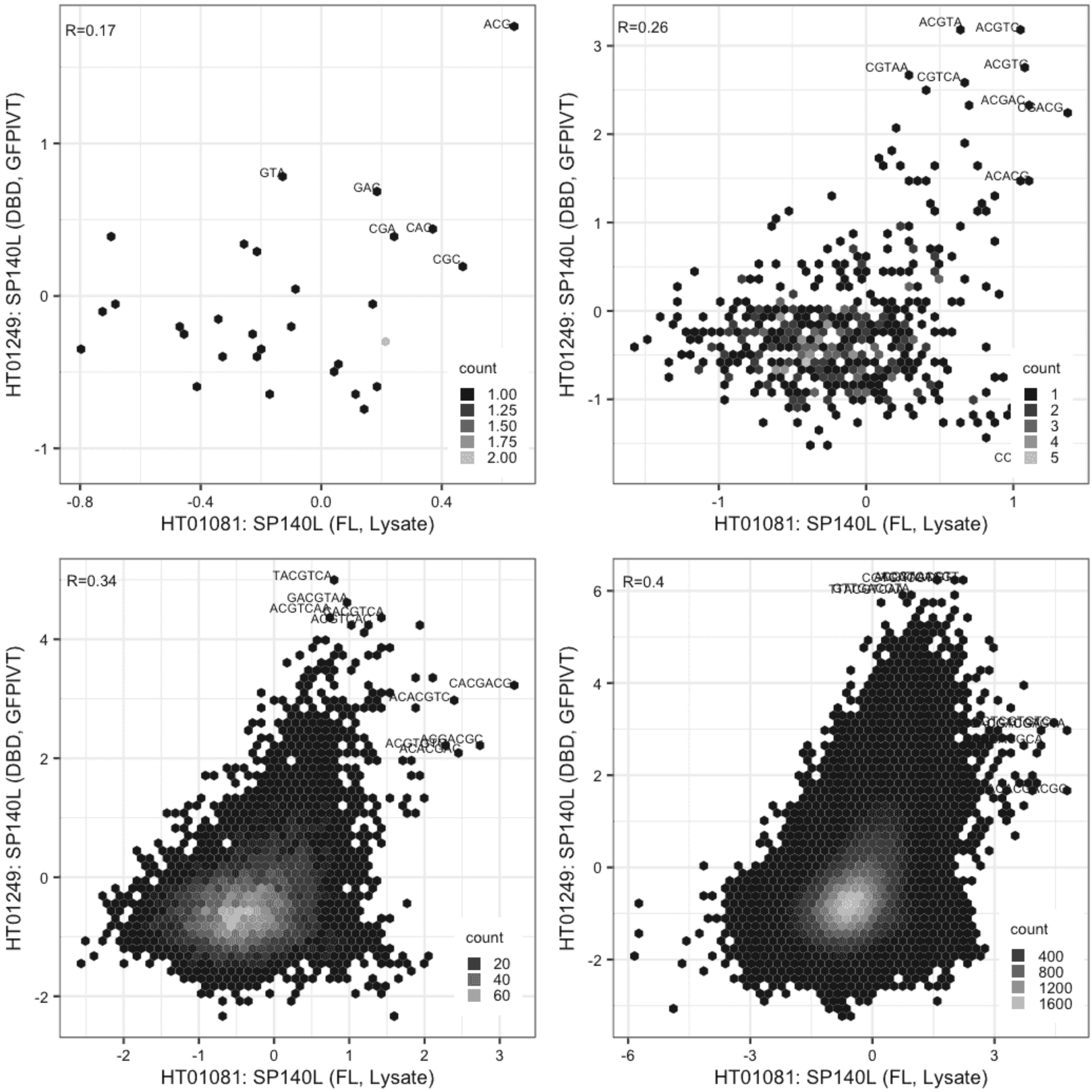

SP140L

Lysate vs GFPIVT, same construct

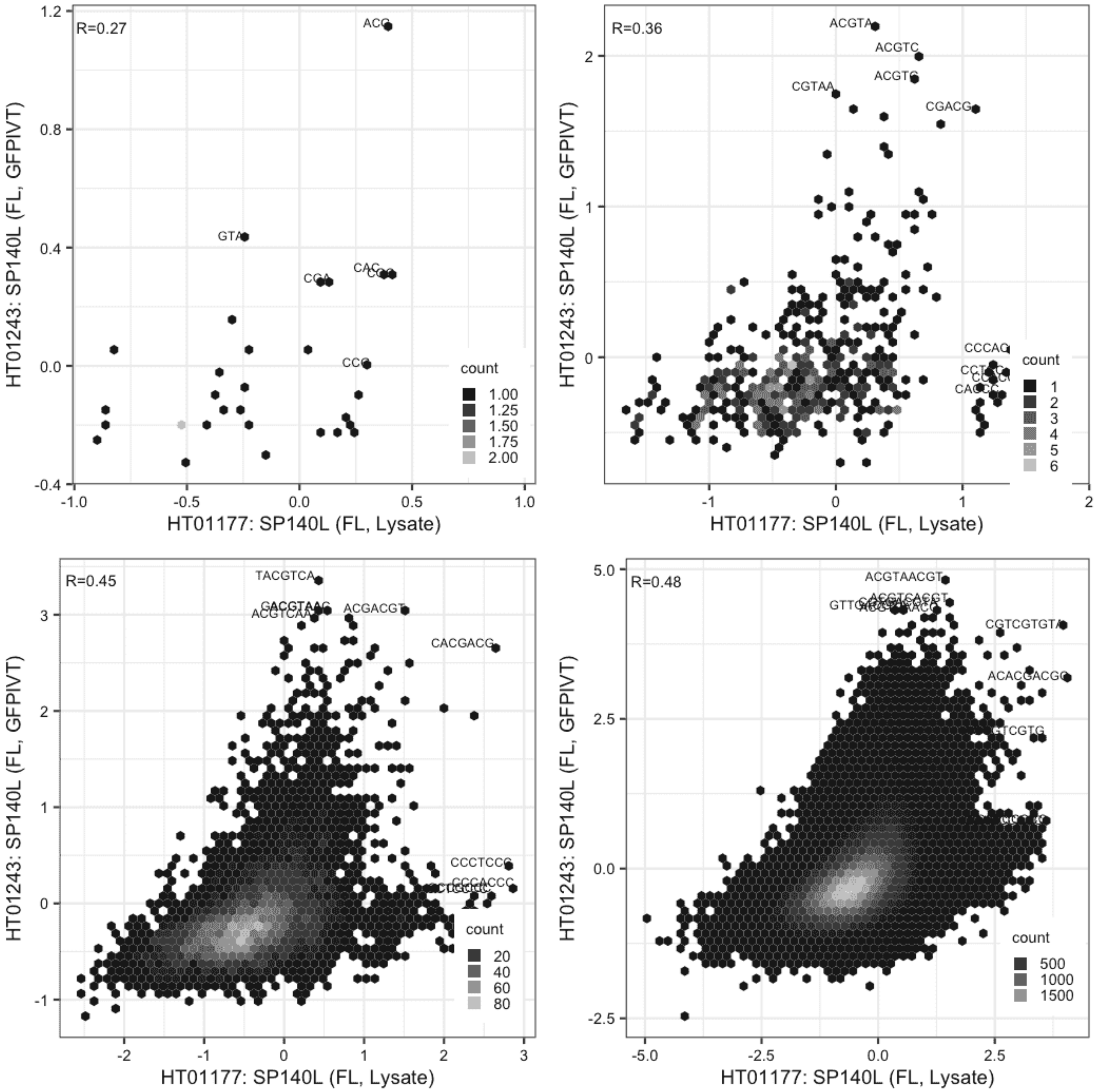

SP140L

Lysate vs GFPIVT, different constructs

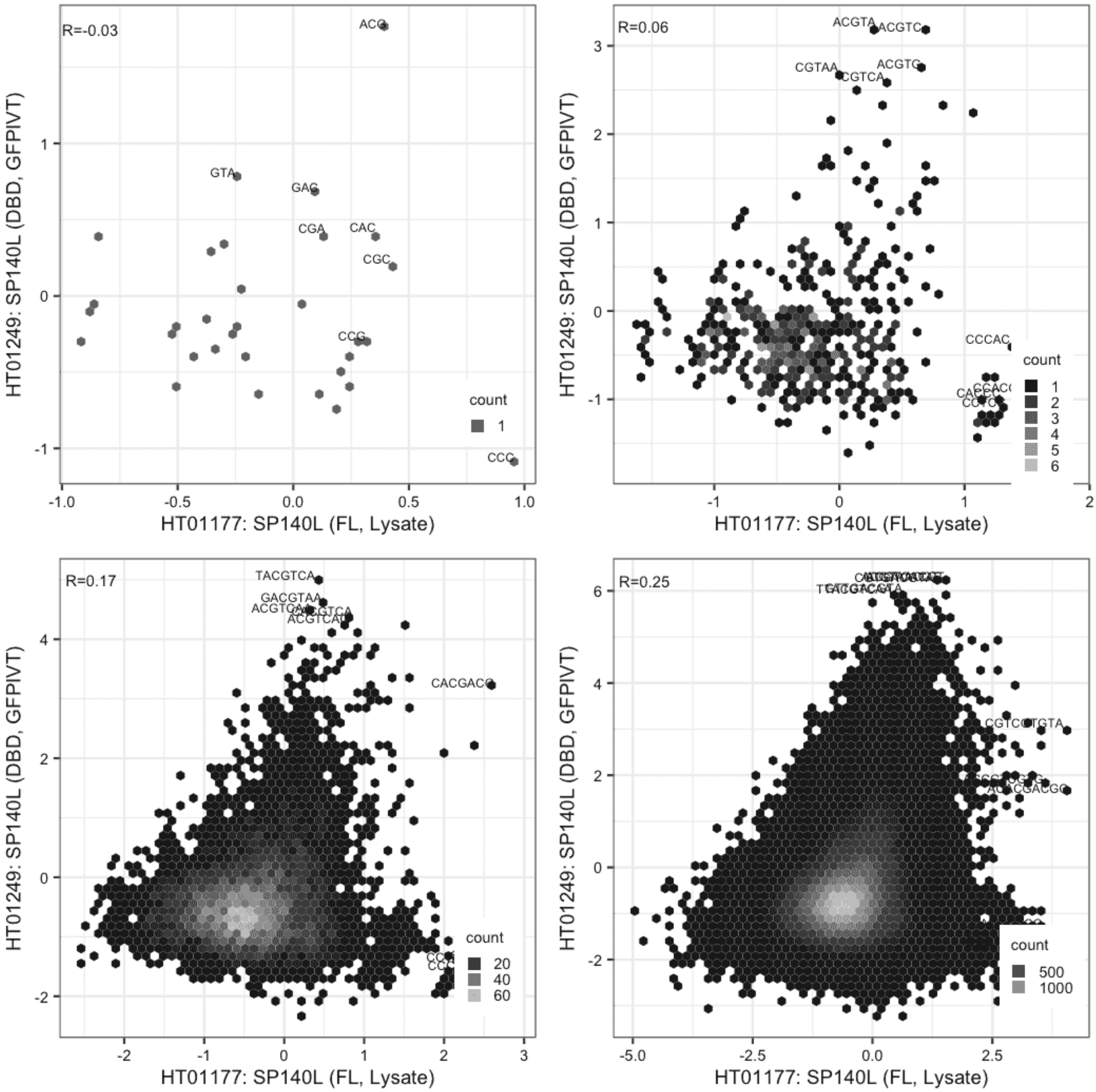

SP140L

GFPIVT vs GFPIVT, different constructs

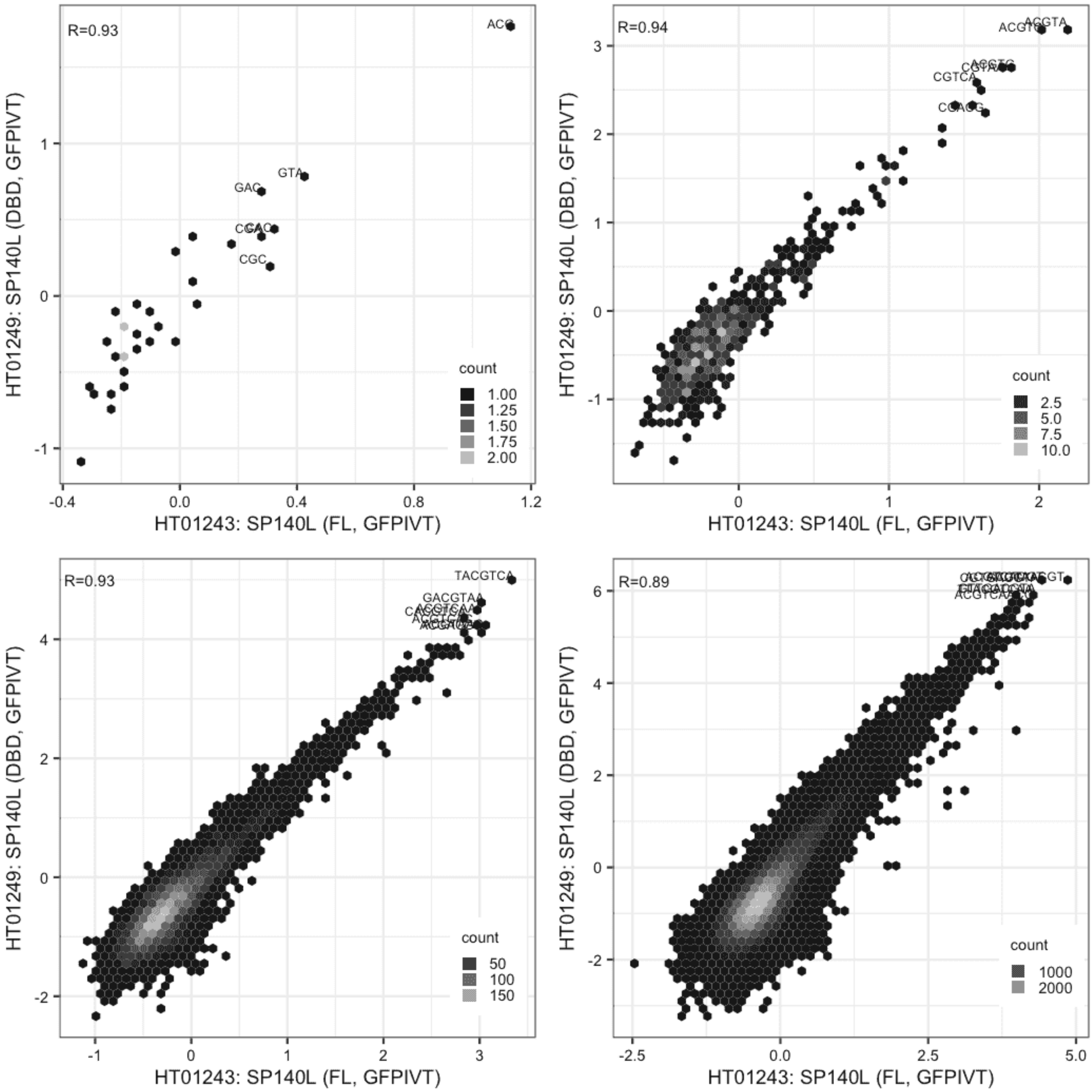

SRY

IVT vs IVT, same construct

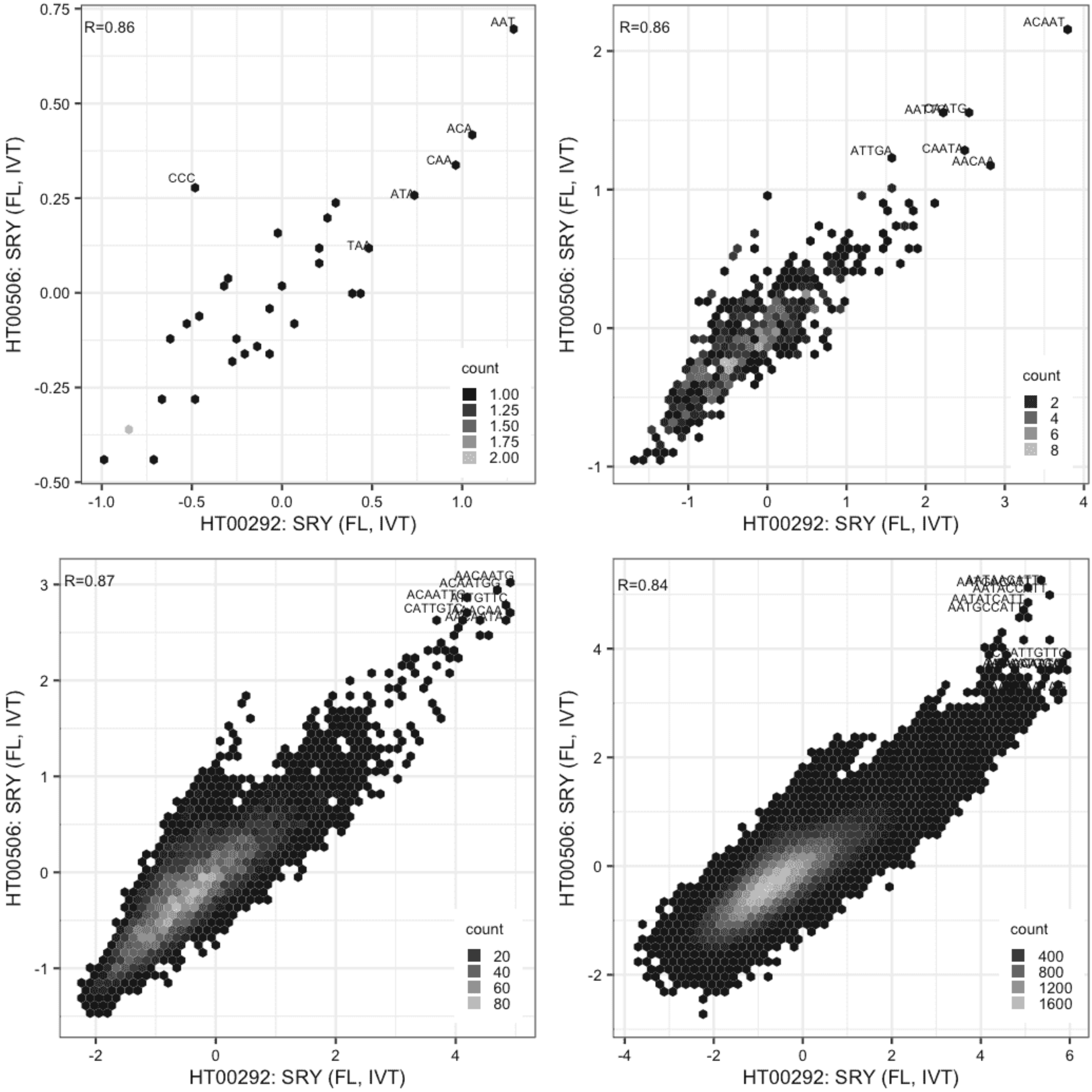

SRY

Lysate vs IVT, same construct

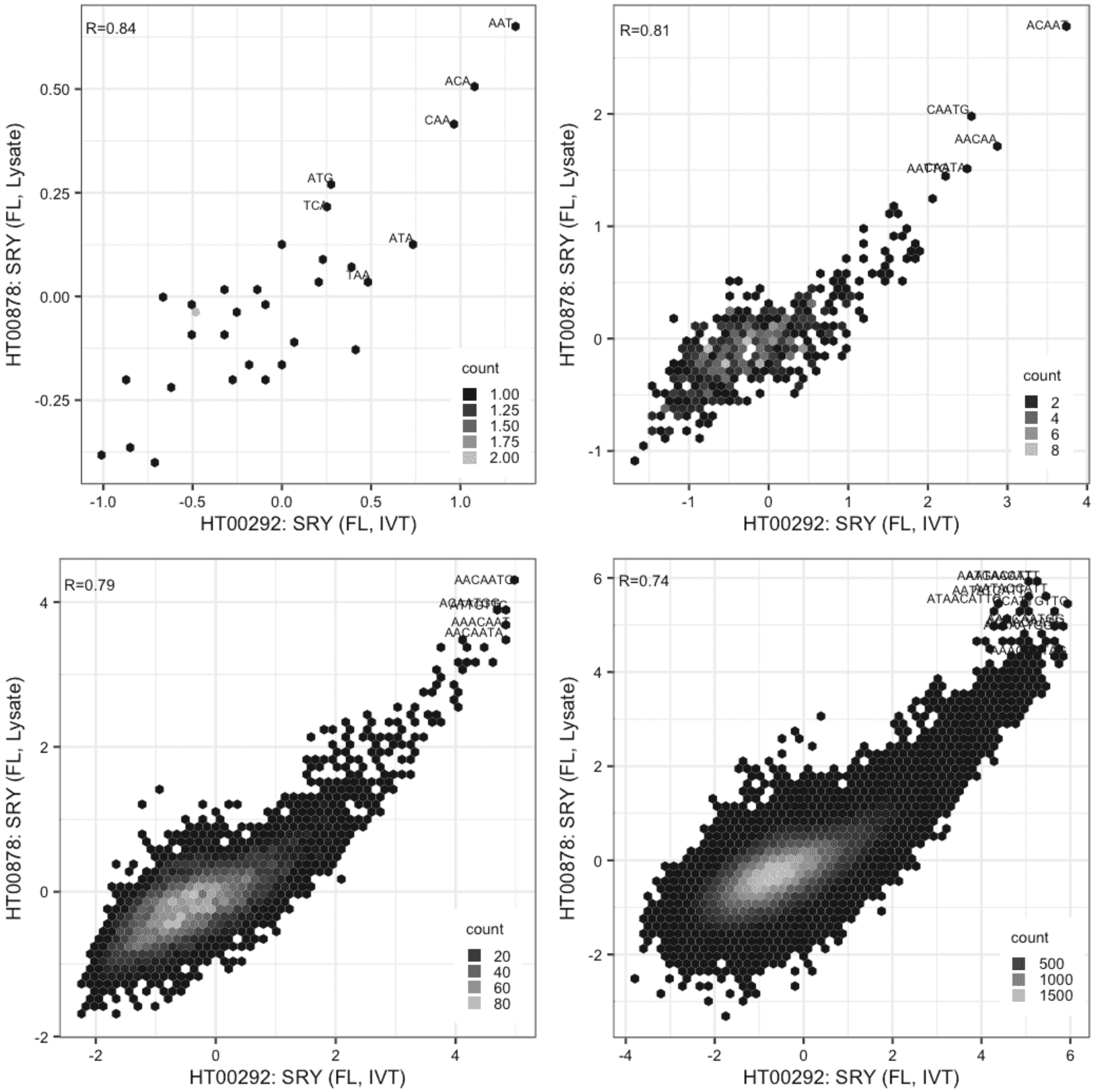

SRY

Lysate vs IVT, same construct

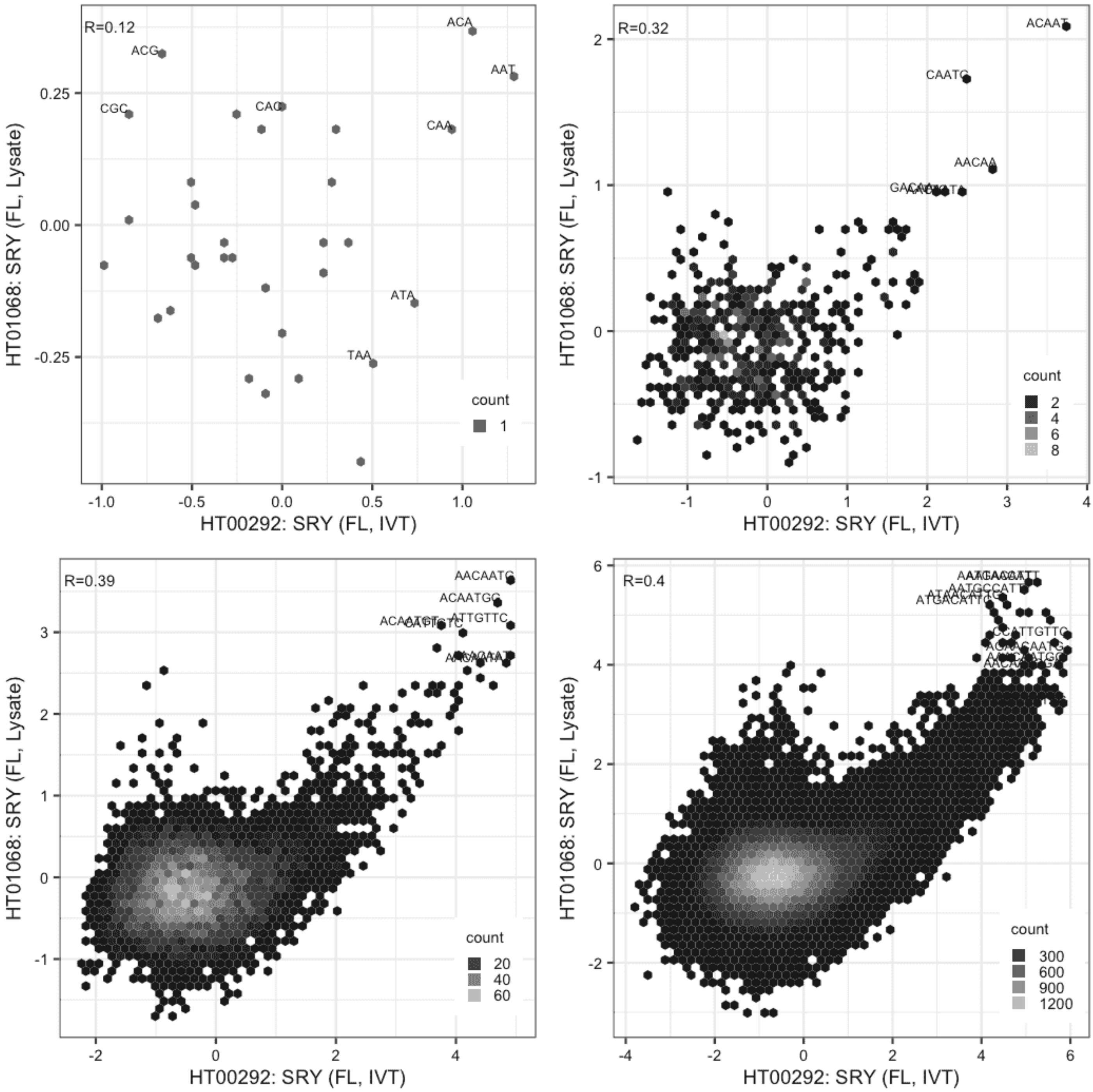

SRY

Lysate vs IVT, same construct

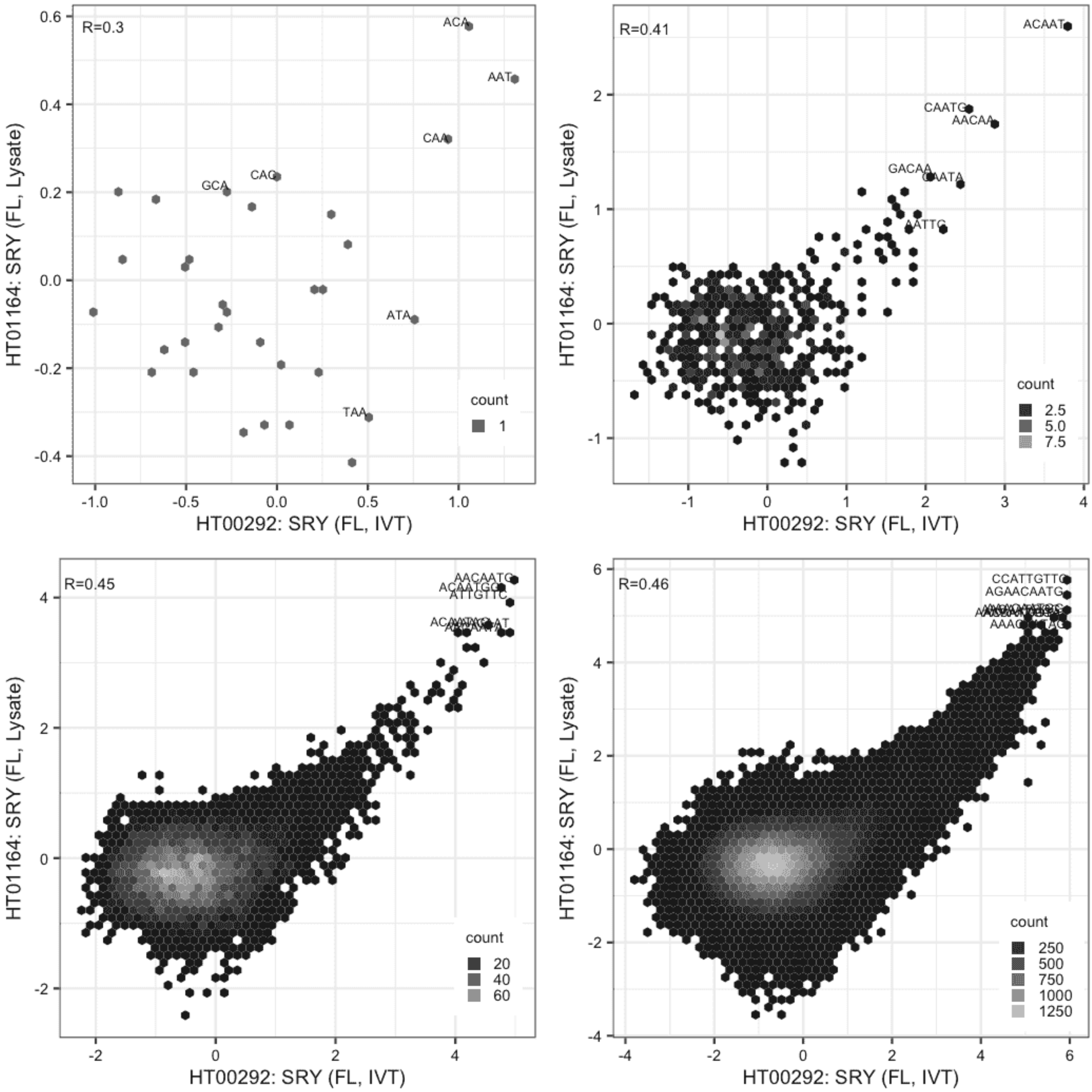

SRY

Lysate vs IVT, same construct

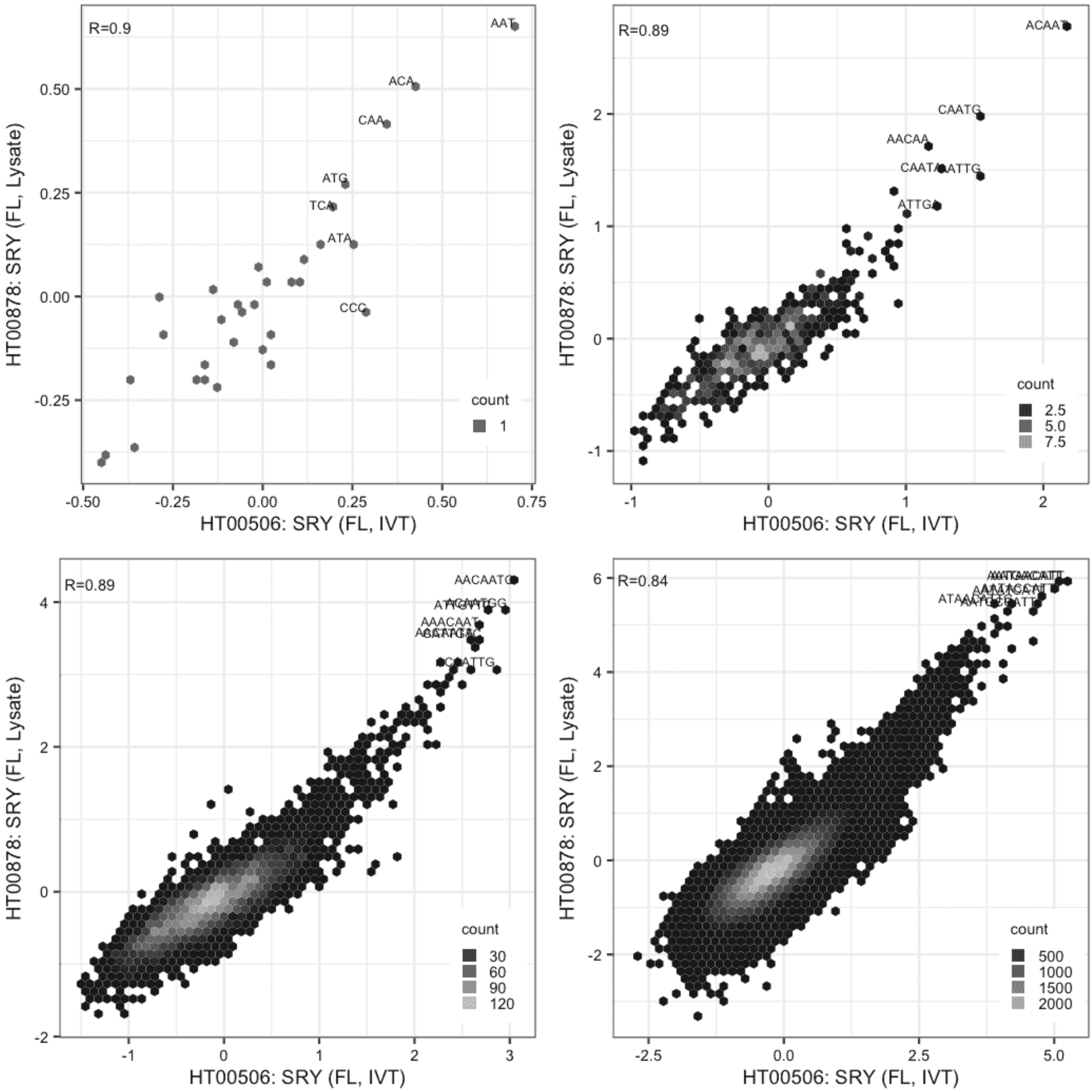

SRY

Lysate vs IVT, same construct

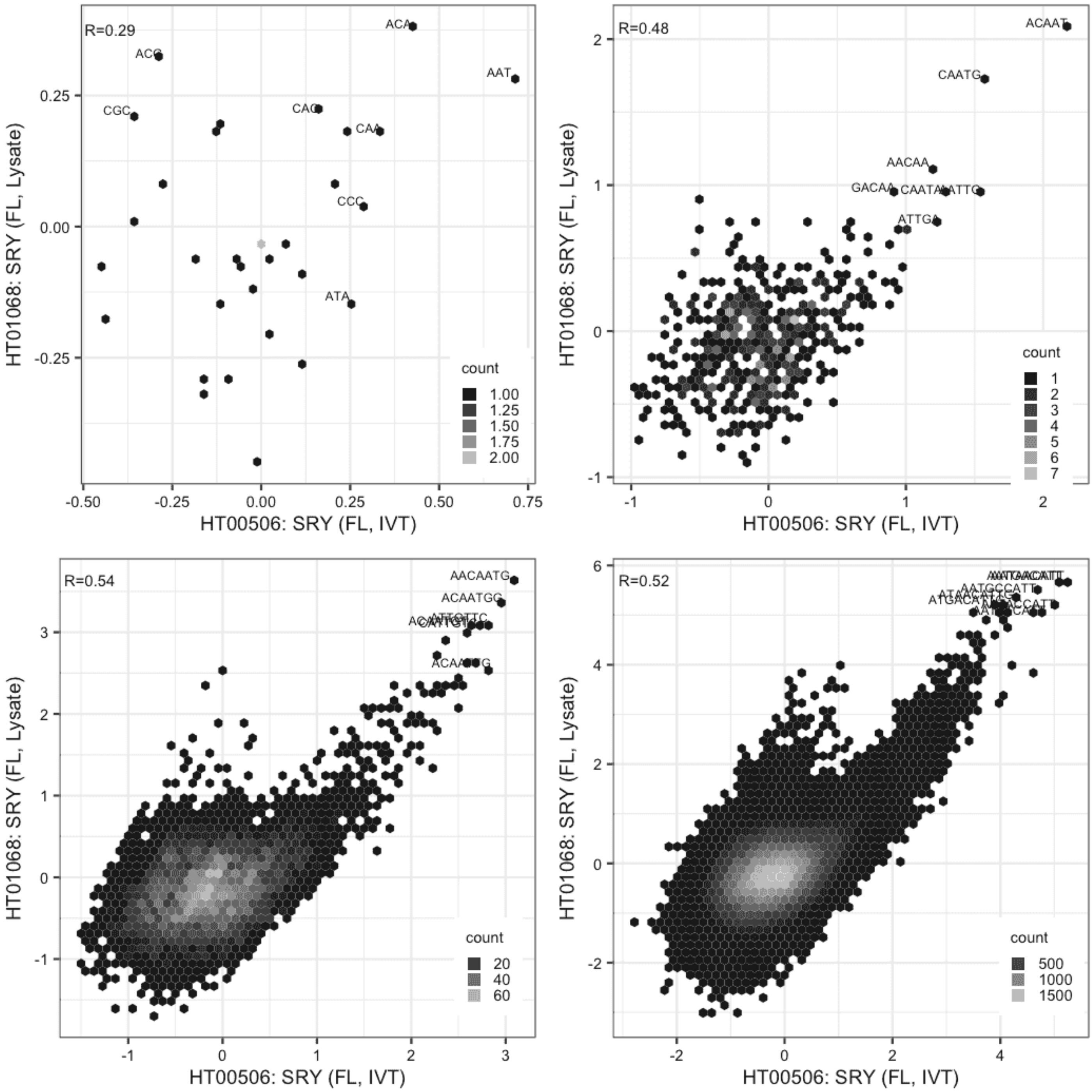

SRY

Lysate vs IVT, same construct

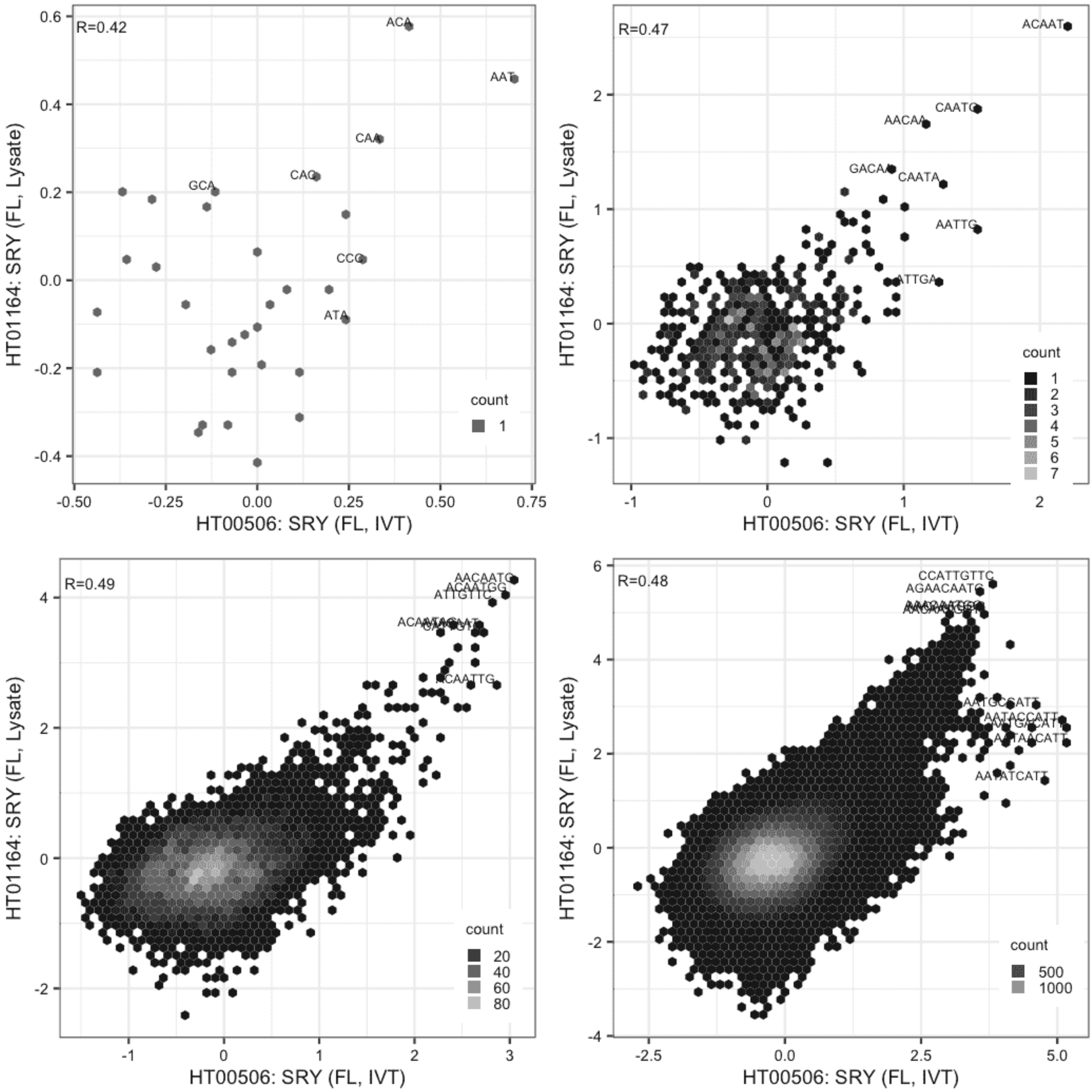

SRY

Lysate vs Lysate, same construct

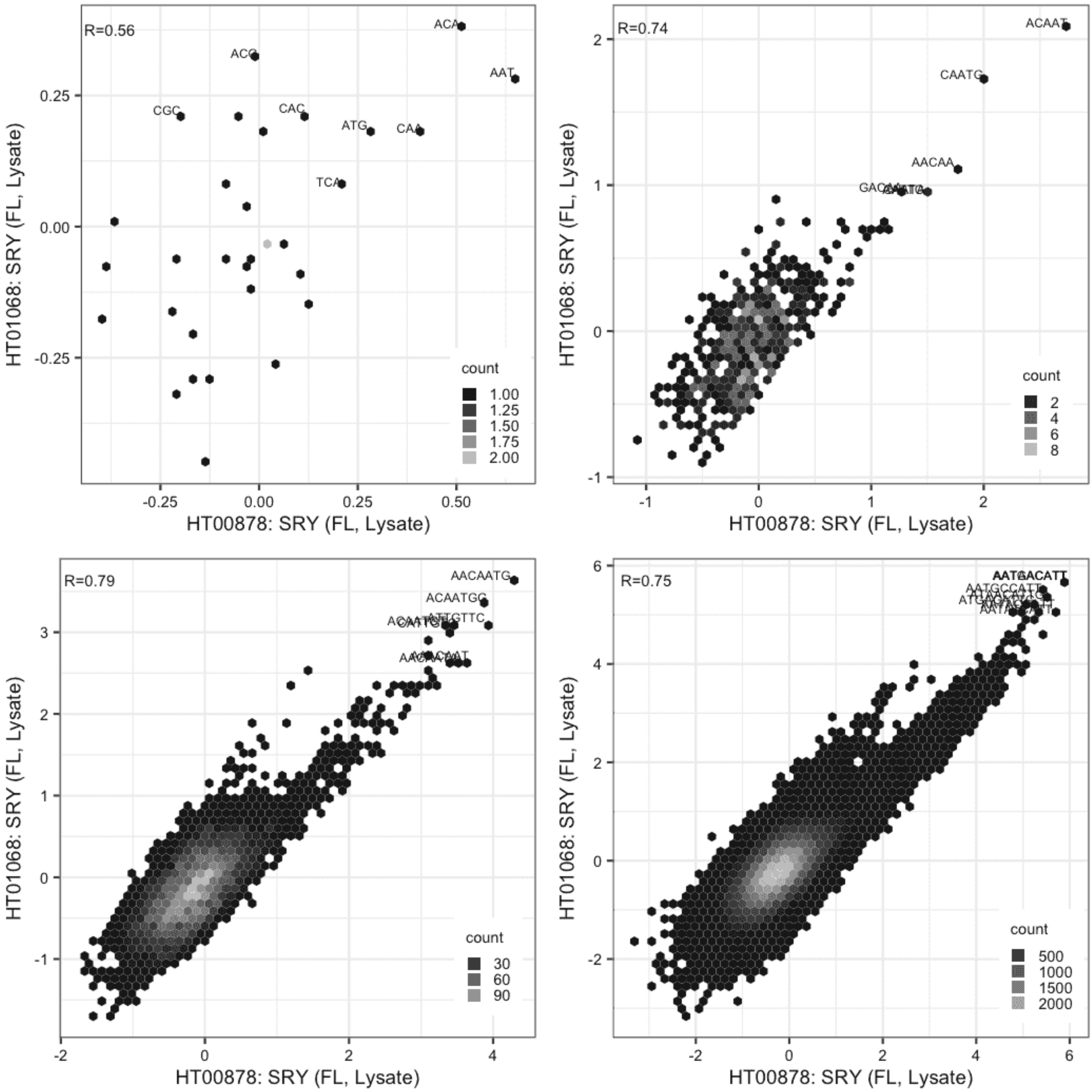

SRY

Lysate vs Lysate, same construct

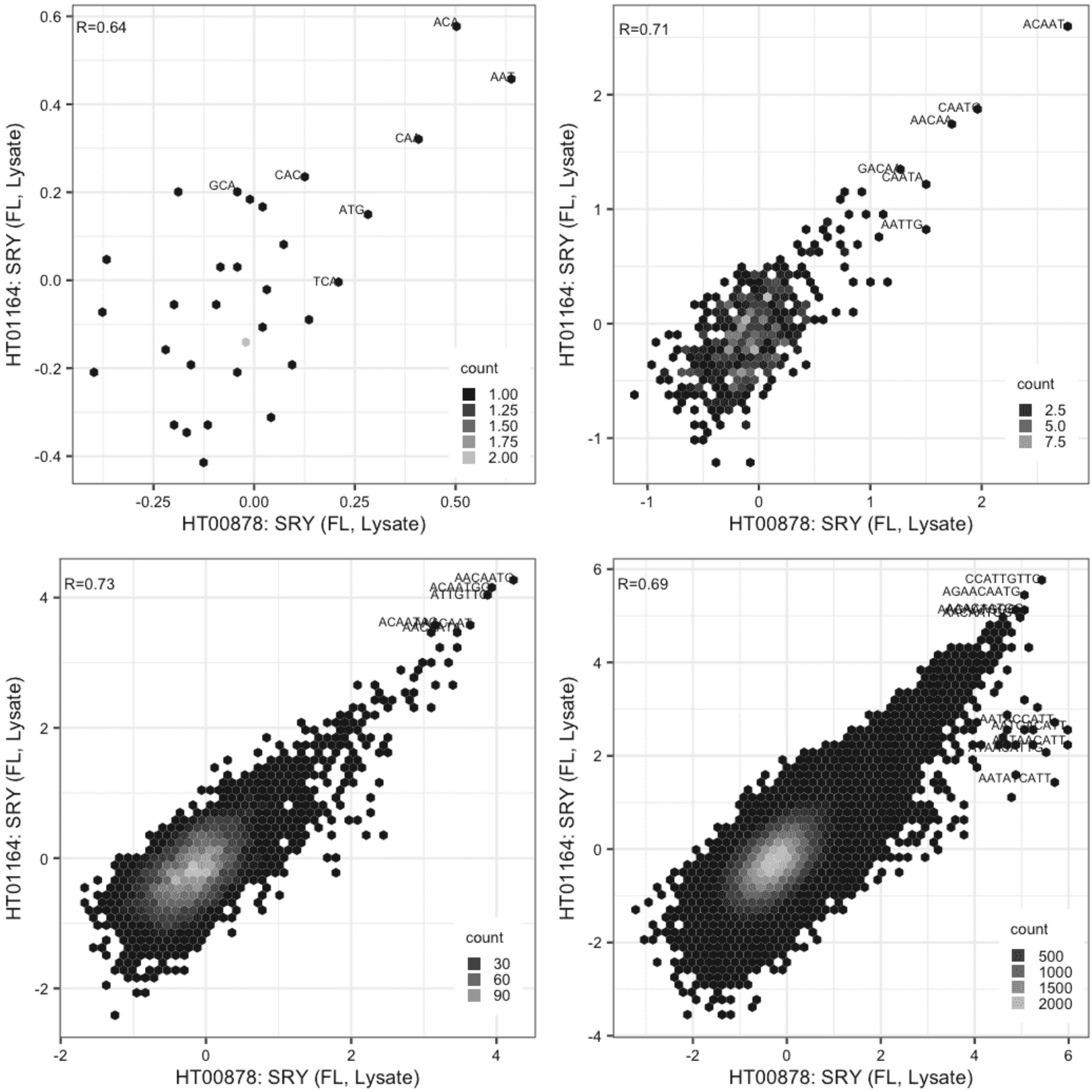

SRY

Lysate vs Lysate, same construct

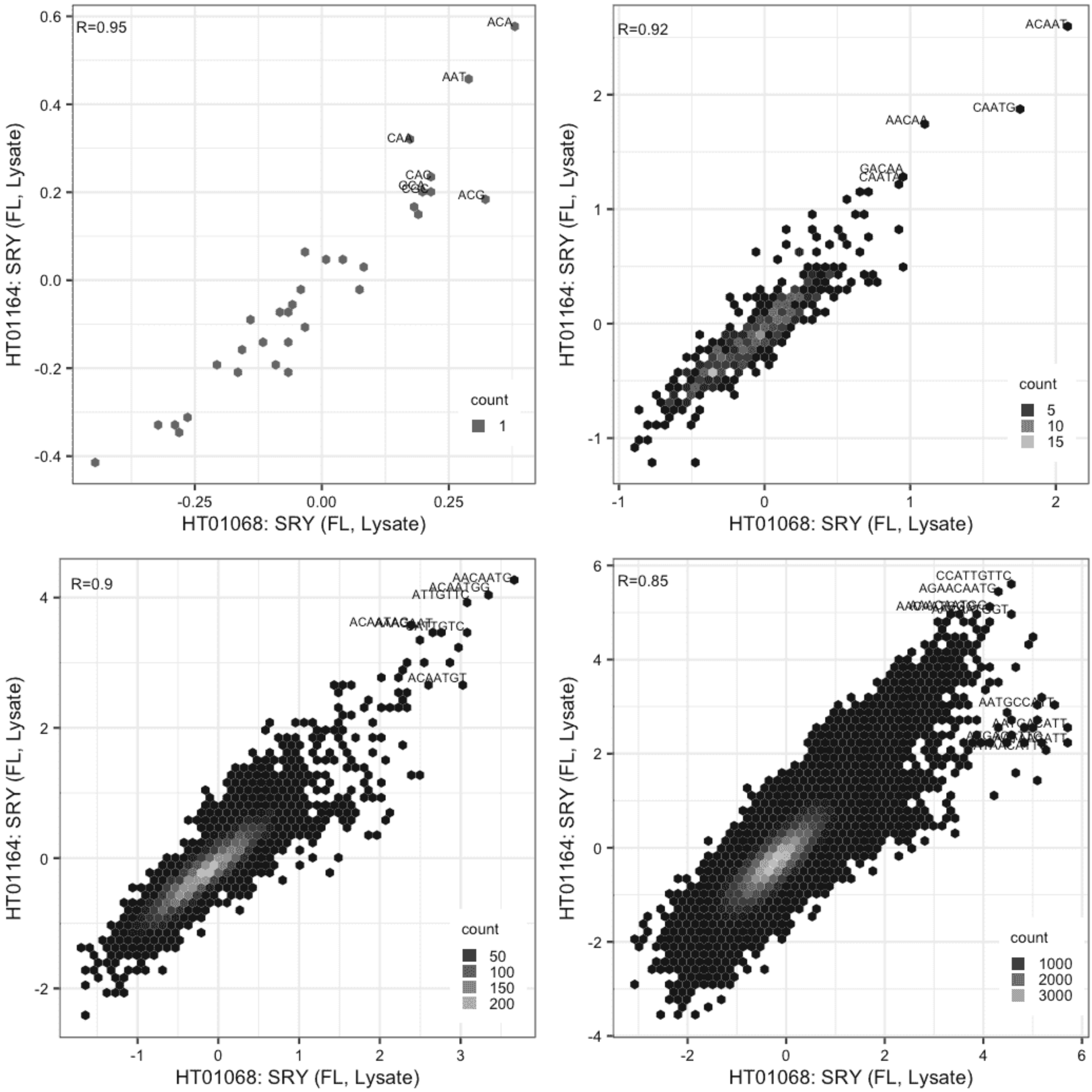

TET3

IVT vs GFPIVT, same construct

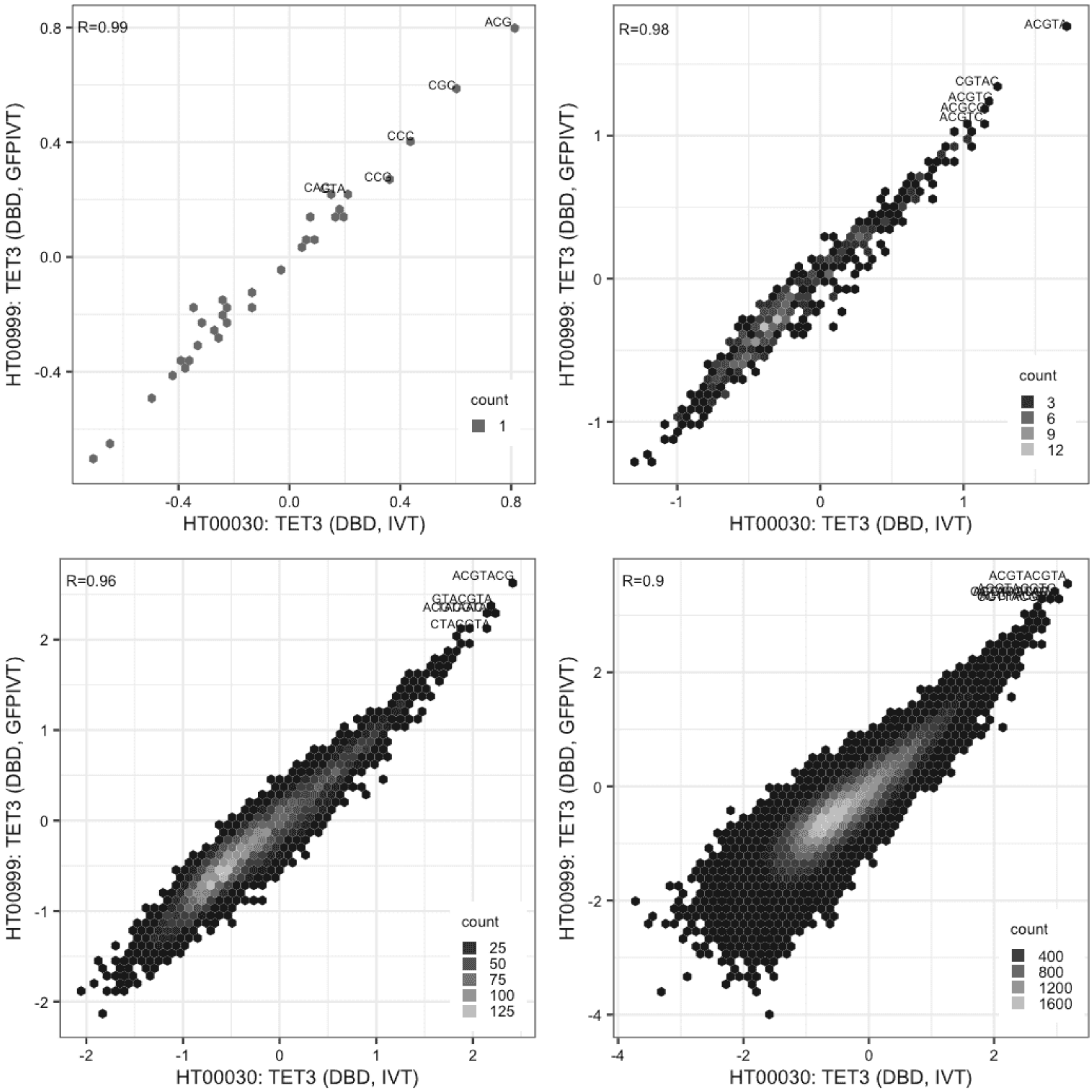

TET3

Lysate vs IVT, different constructs

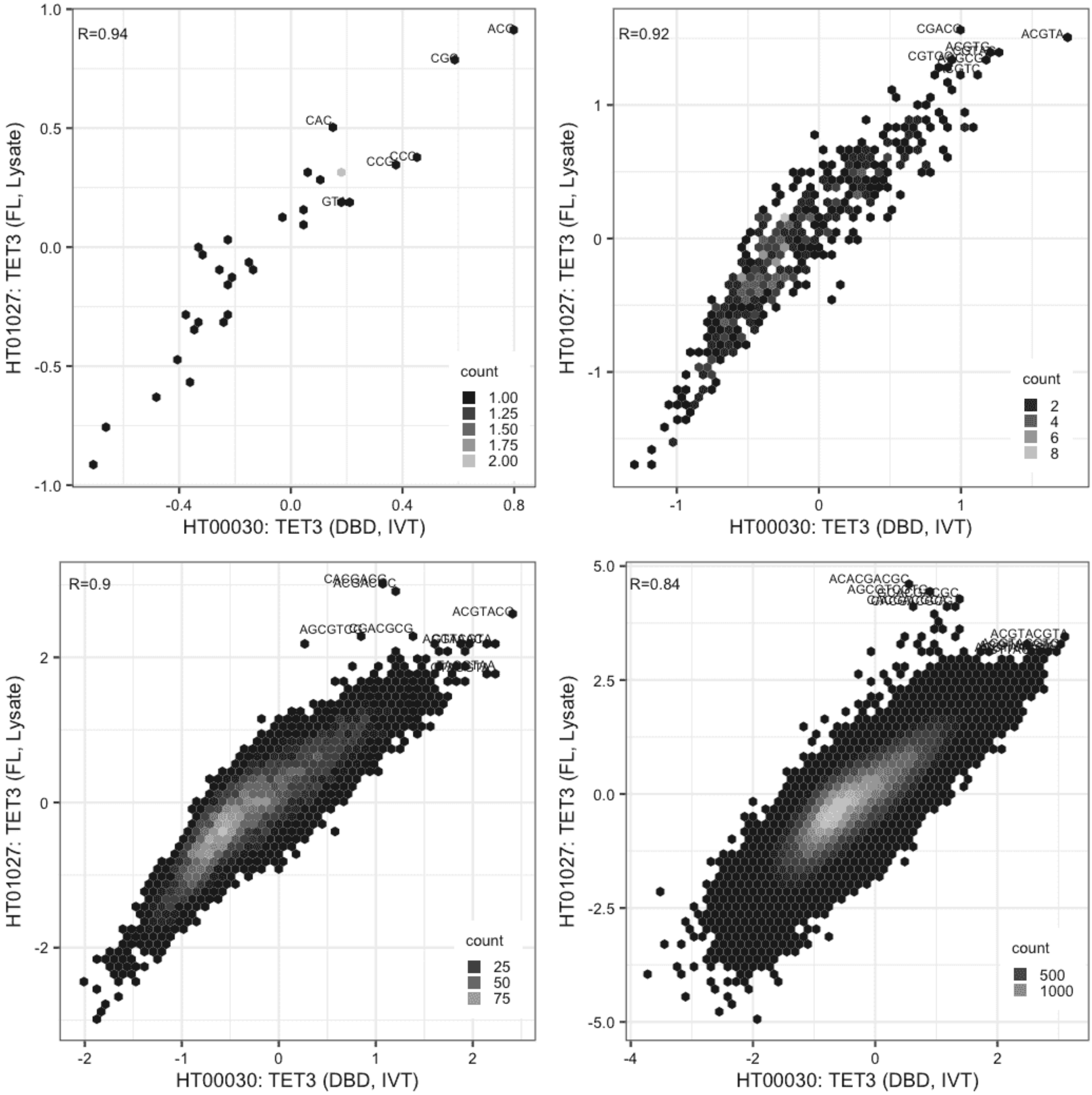

TET3

Lysate vs IVT, different constructs

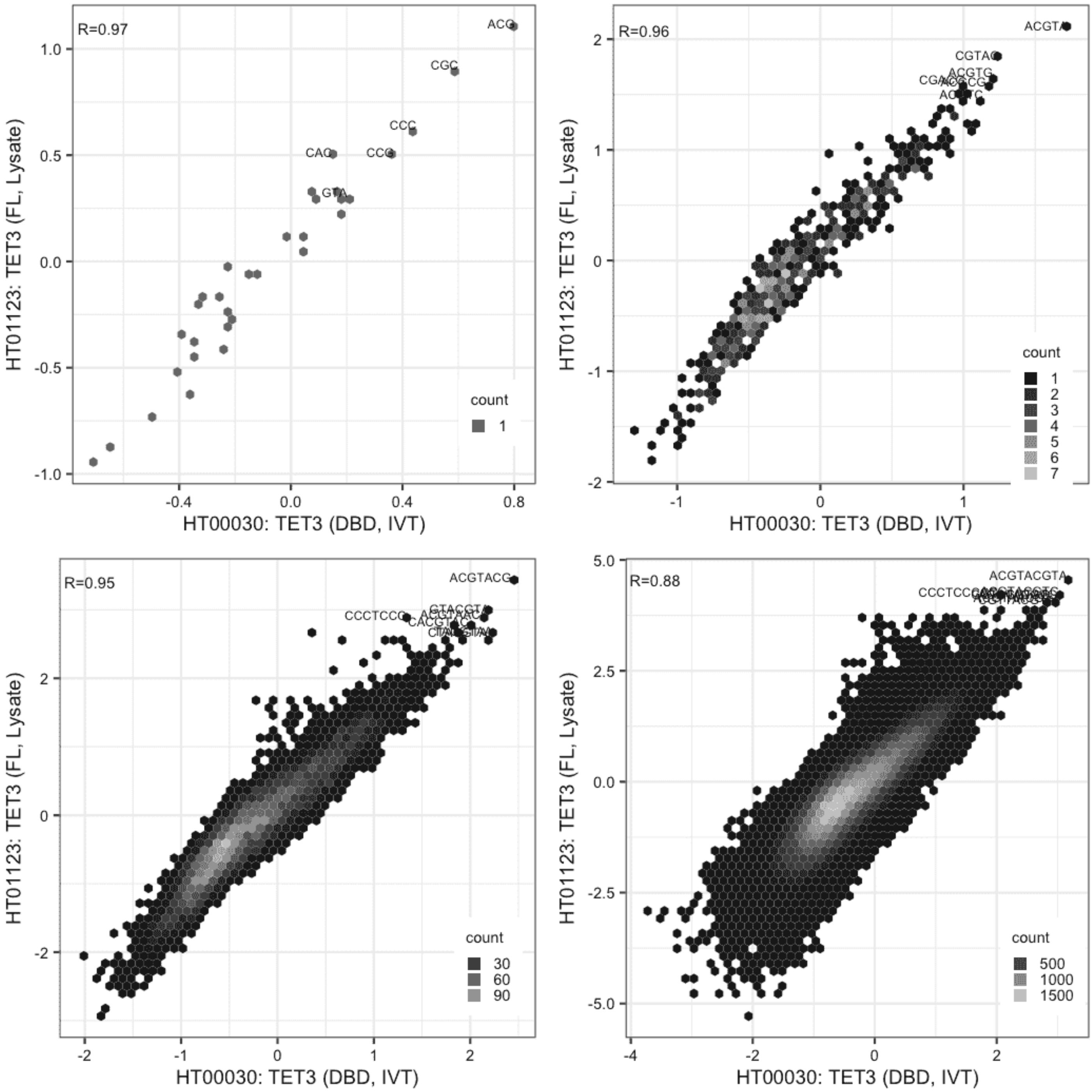

TET3

Lysate vs GFPIVT, different constructs

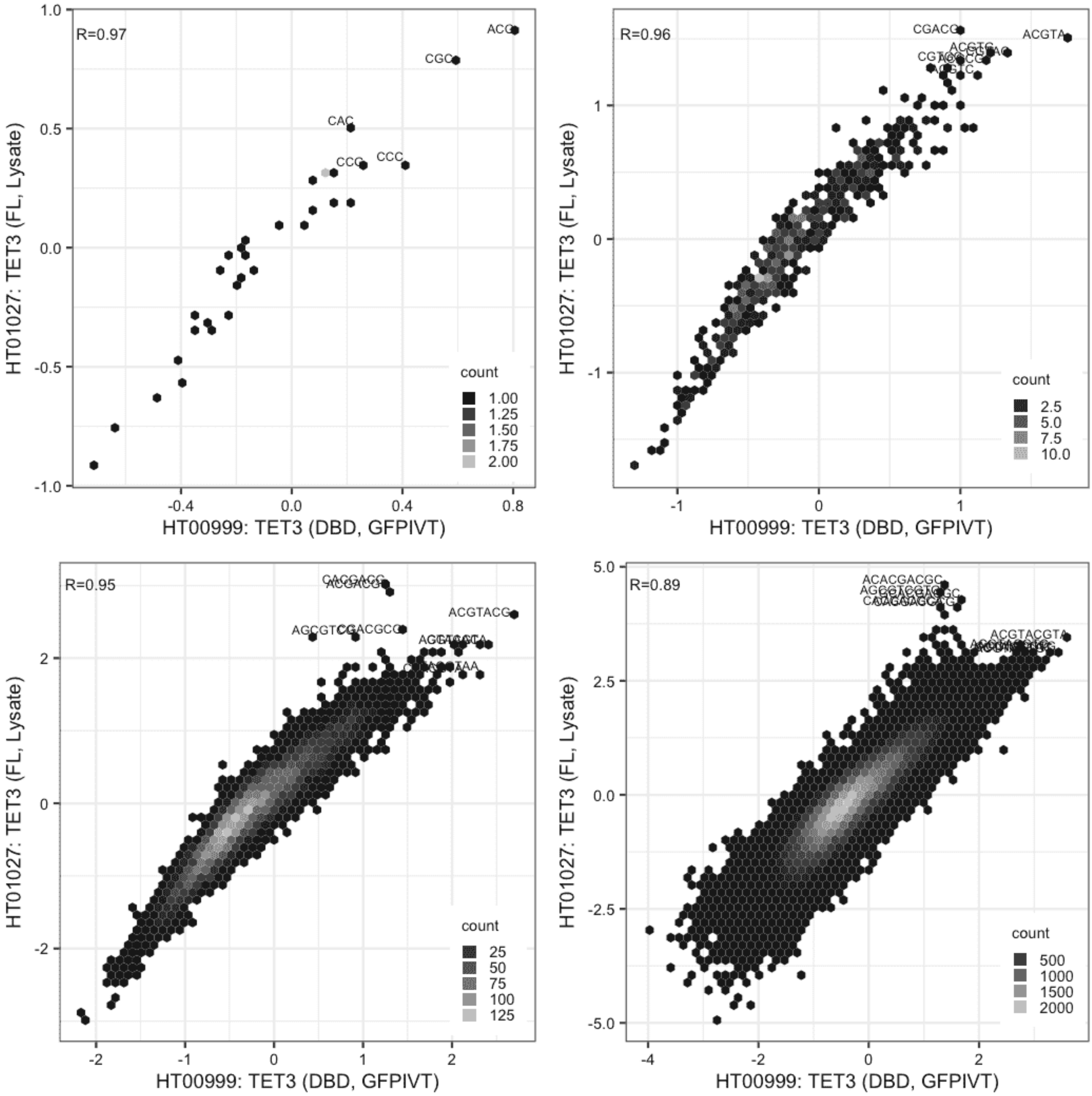

TET3

Lysate vs GFPIVT, different constructs

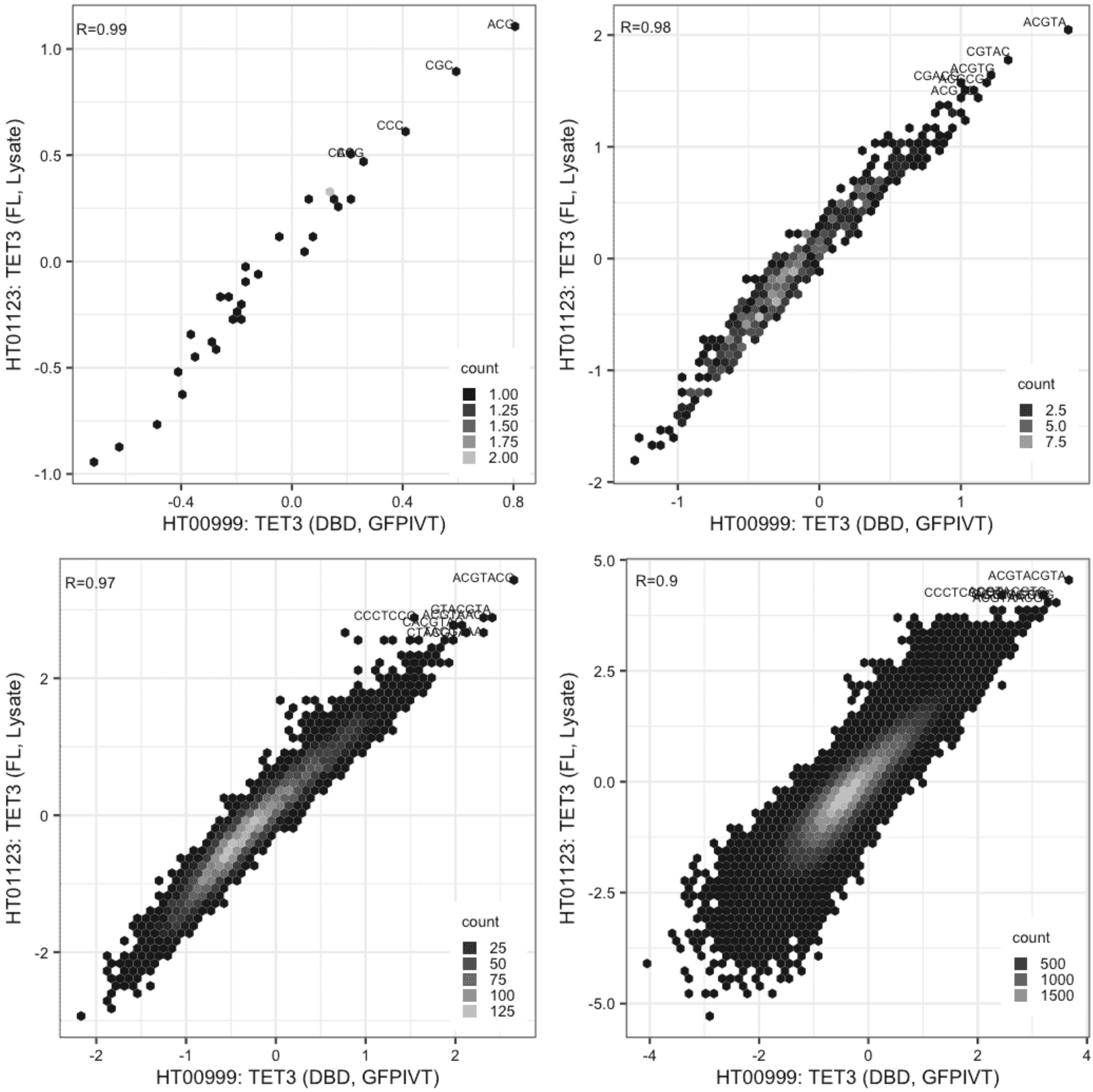

TET3

Lysate vs Lysate, same construct

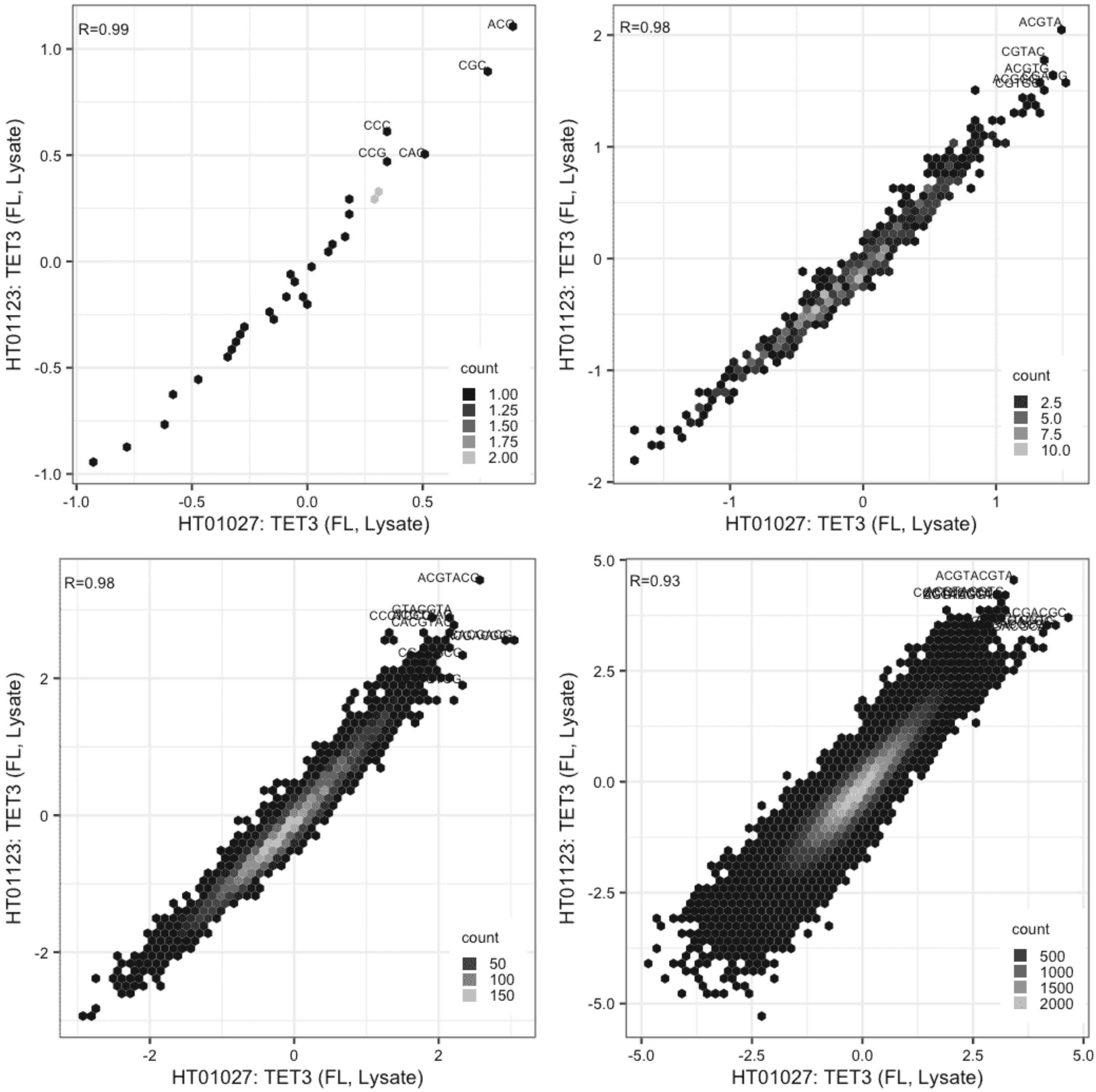

TIGD3

Lysate vs Lysate, same construct

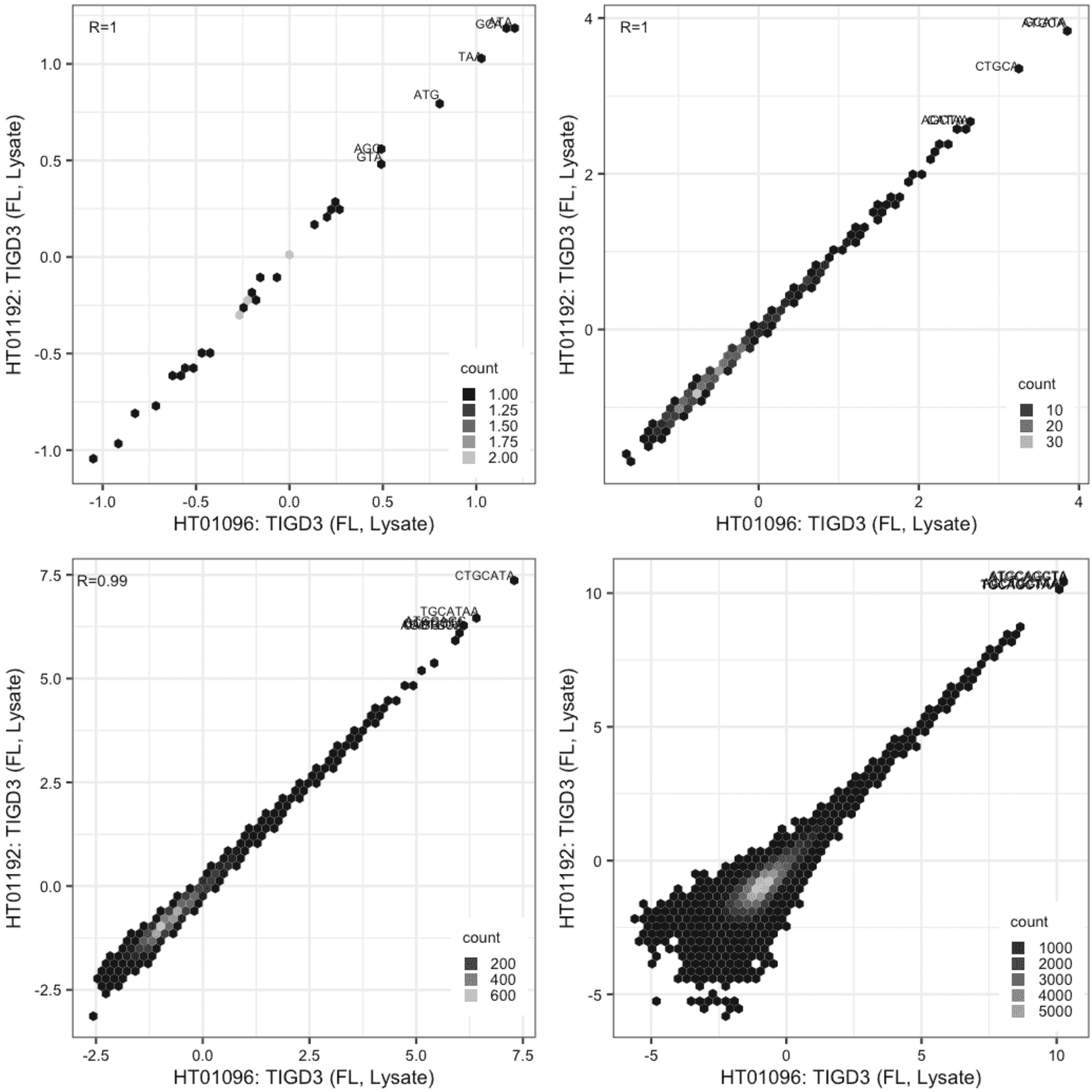

TIGD3

Lysate vs GFPIVT, same construct

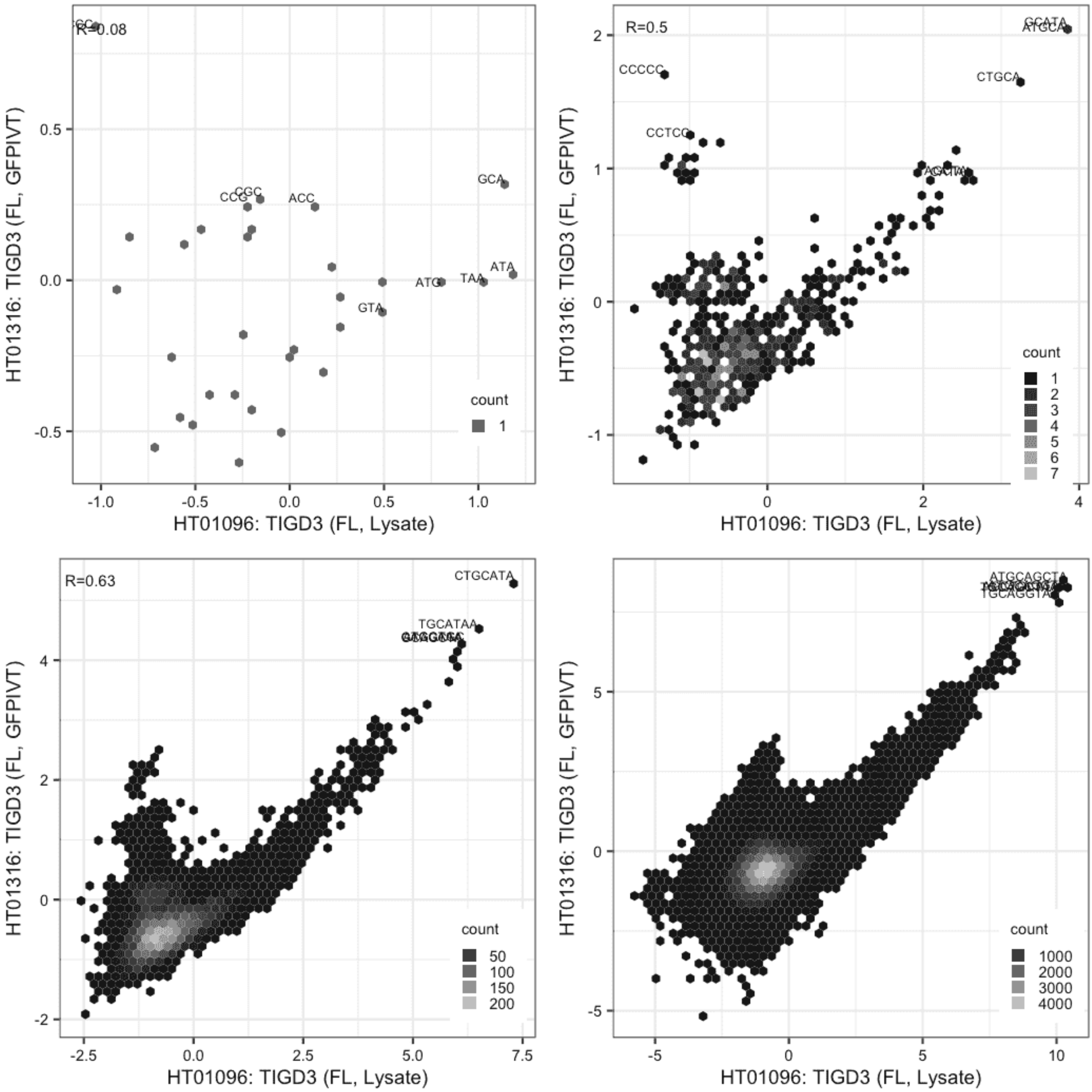

TIGD3

Lysate vs GFPIVT, same construct

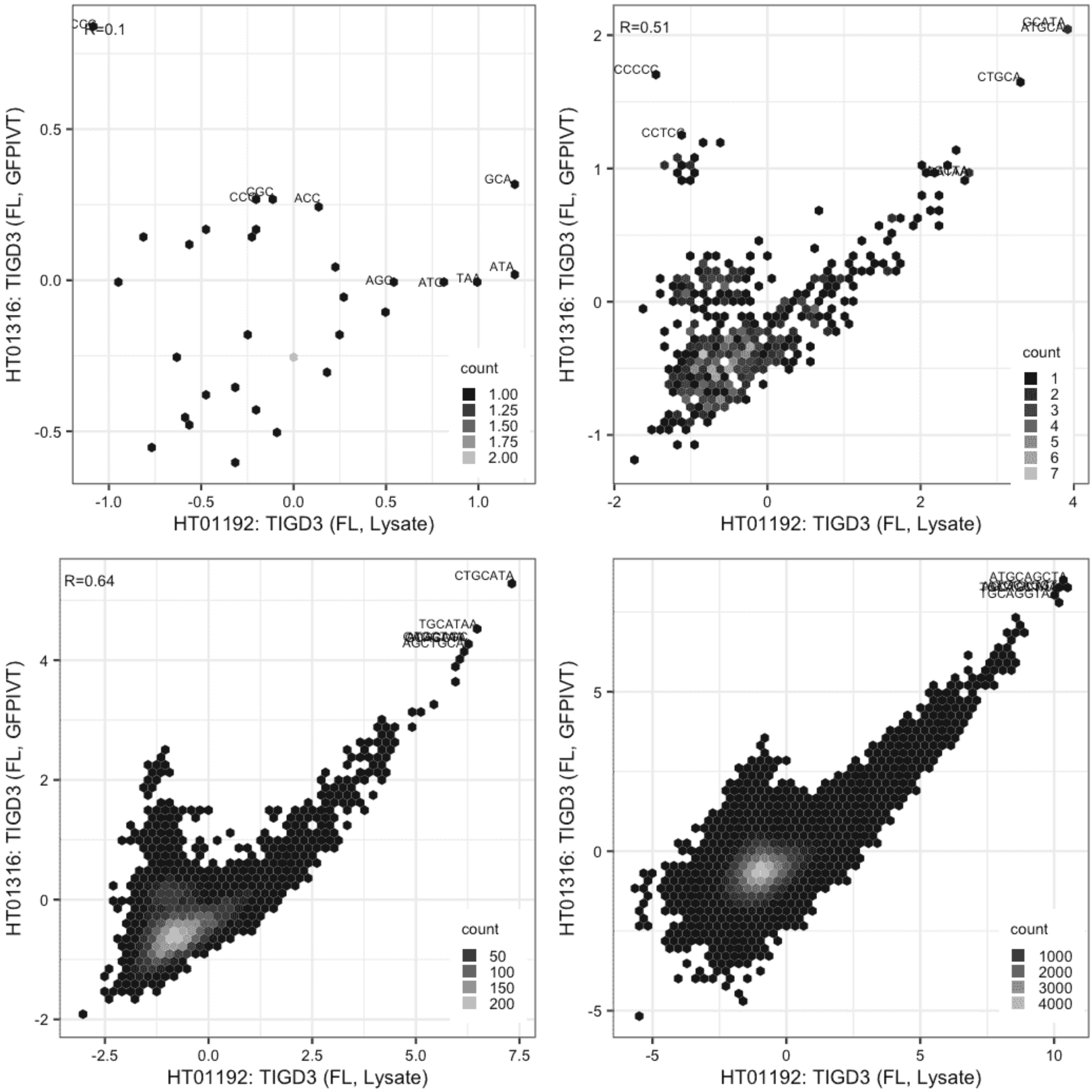

TIGD4

IVT vs GFPIVT, same construct

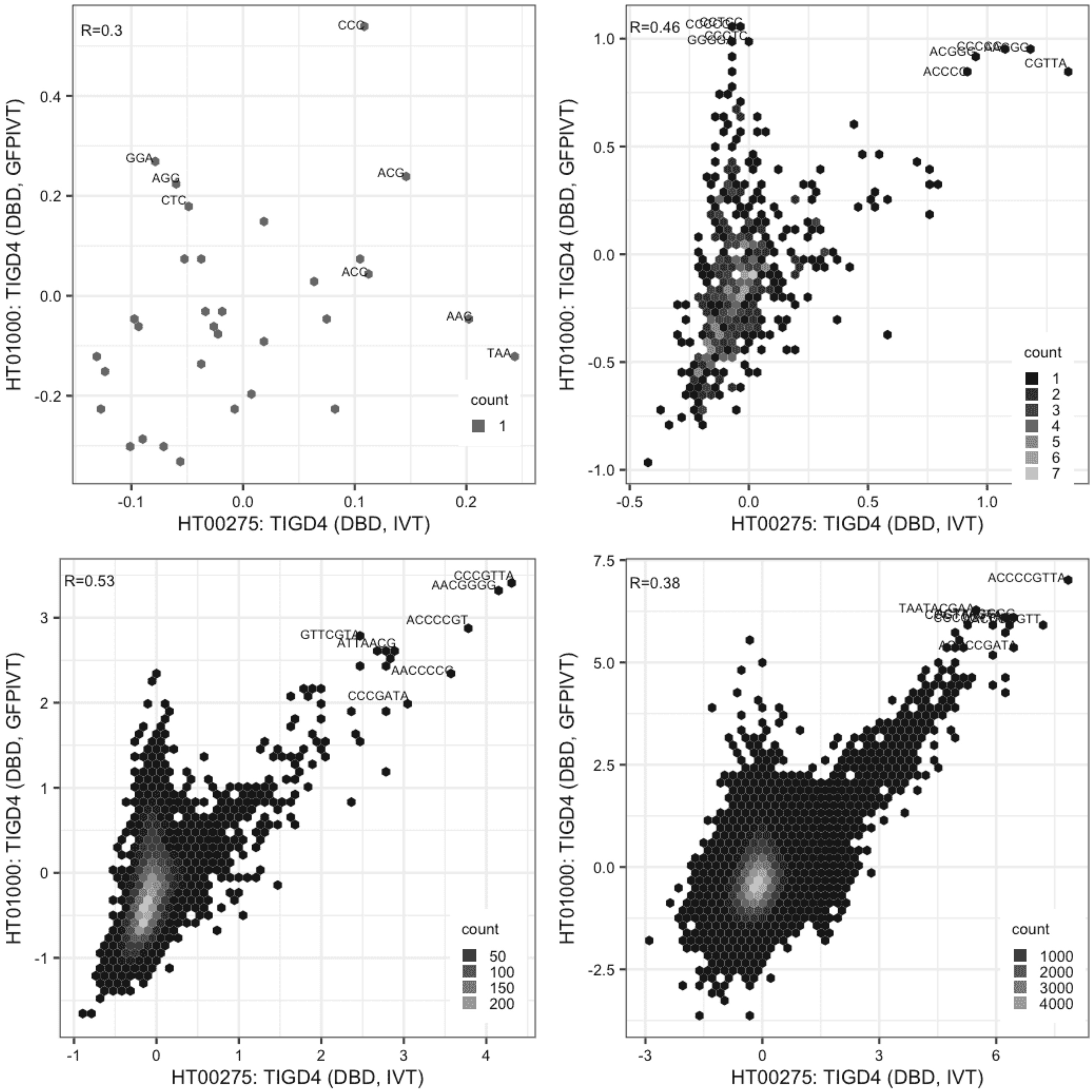

TIGD4

Lysate vs IVT, different constructs

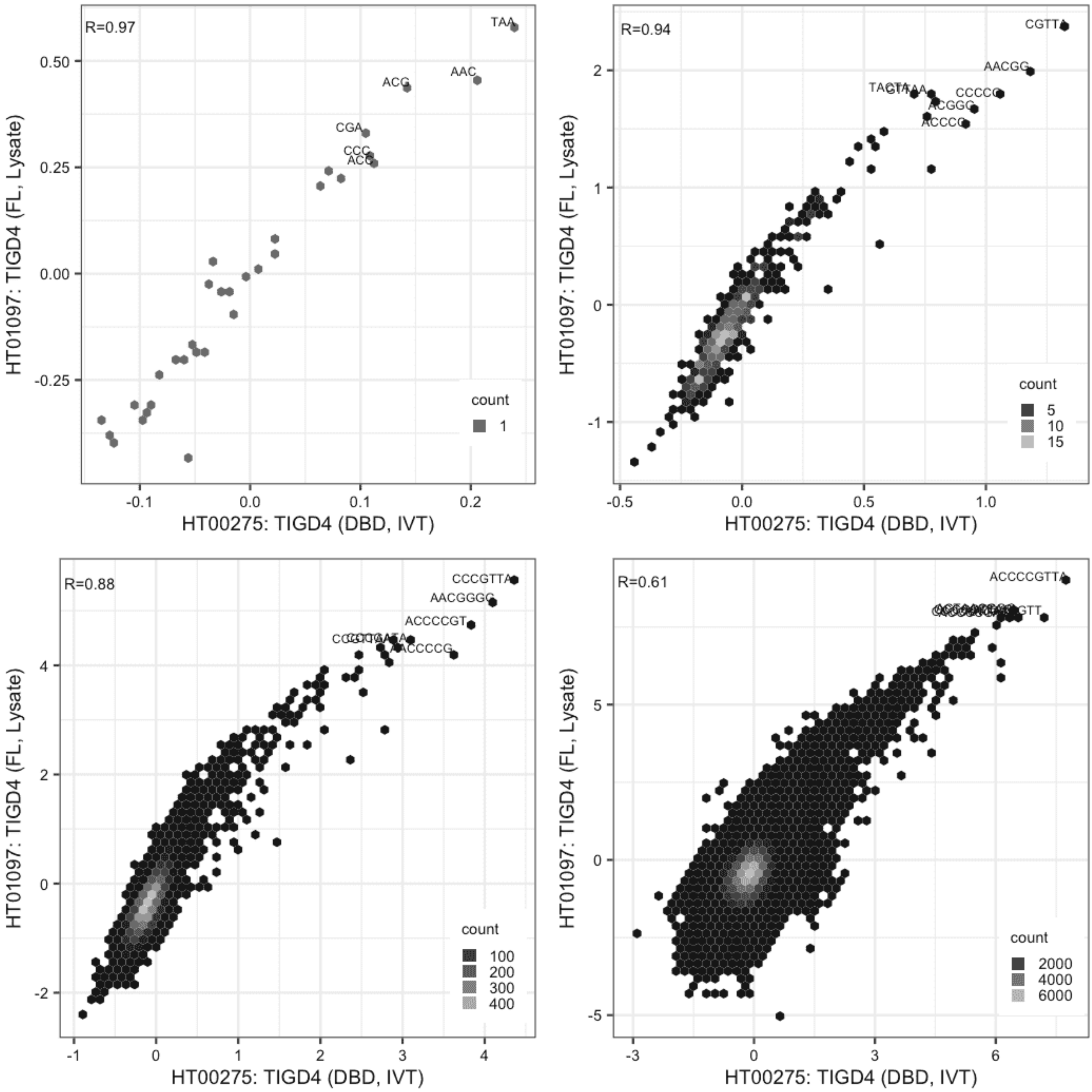

TIGD4

Lysate vs IVT, different constructs

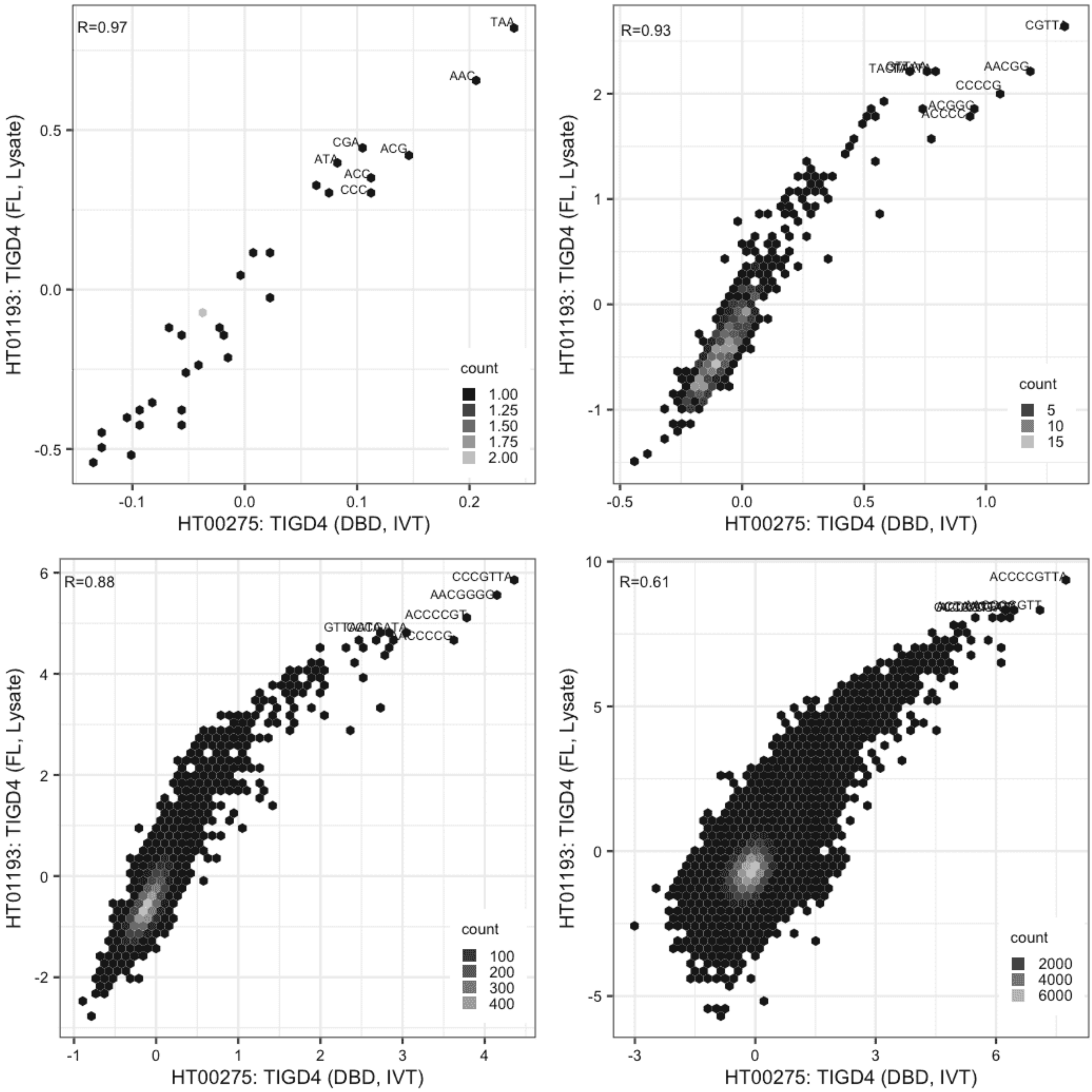

TIGD4

IVT vs GFPIVT, different constructs

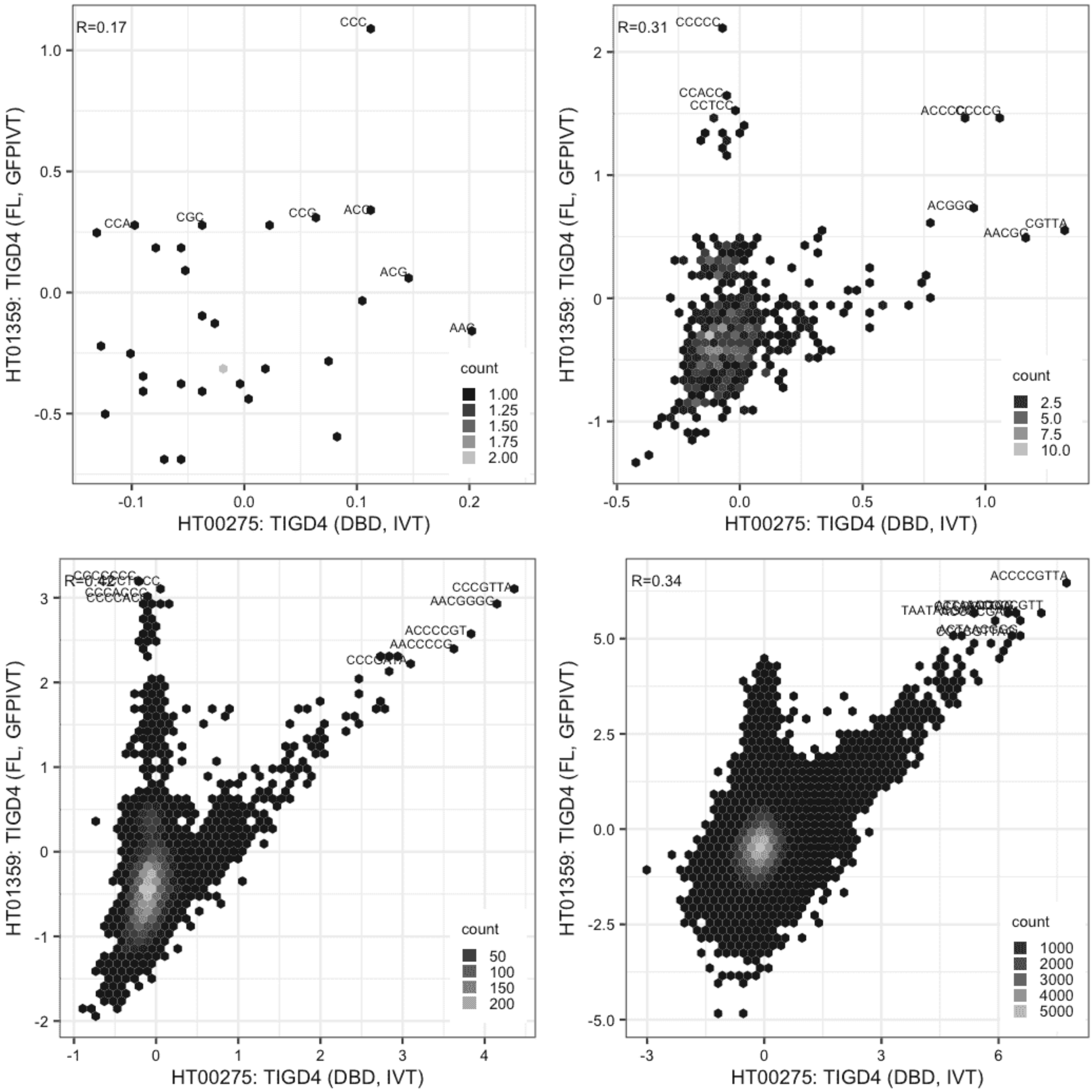

TIGD4

Lysate vs GFPIVT, different constructs

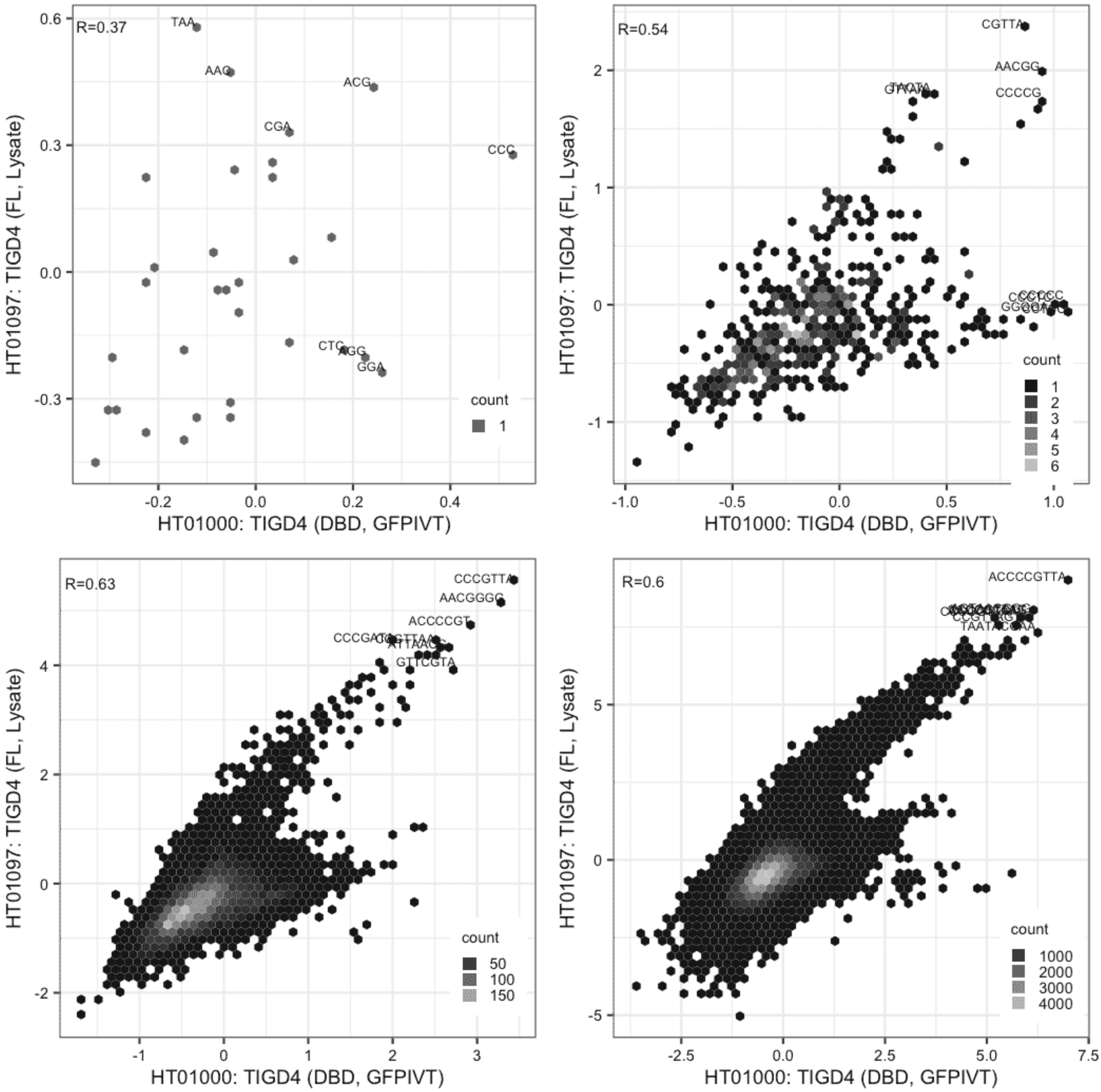

TIGD4

Lysate vs GFPIVT, different constructs

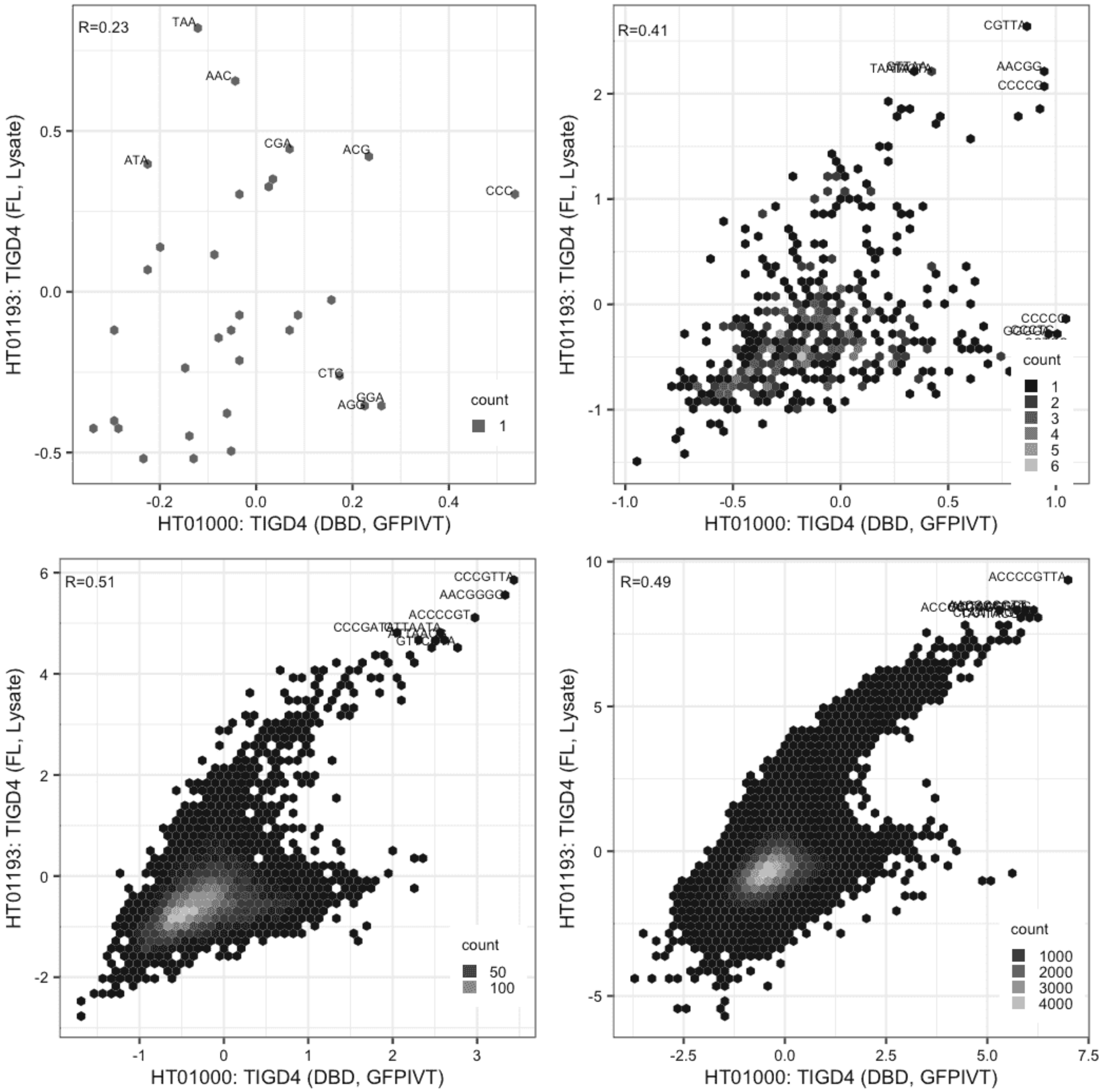

TIGD4

GFPIVT vs GFPIVT, different constructs

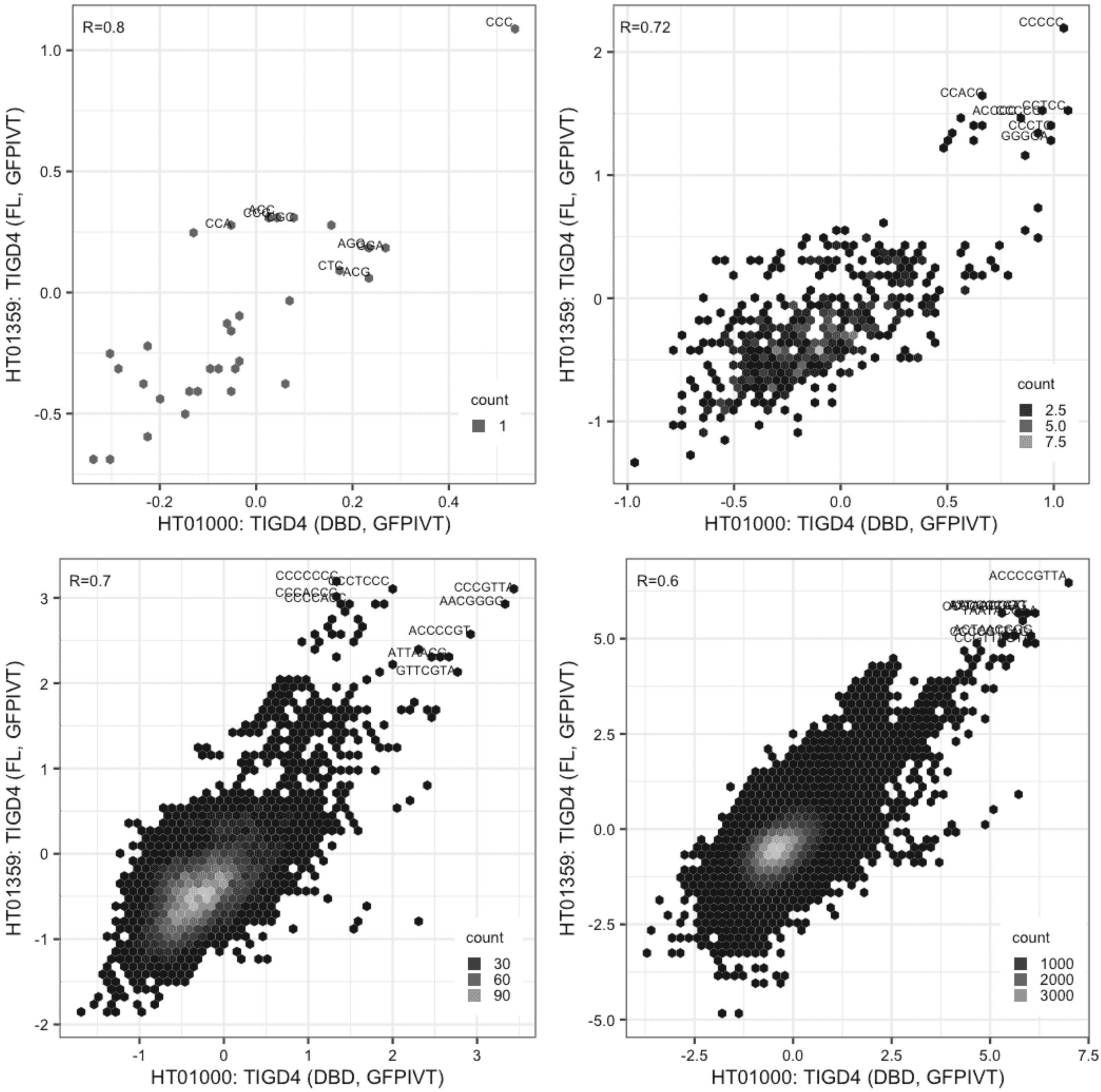

TIGD4

Lysate vs Lysate, same construct

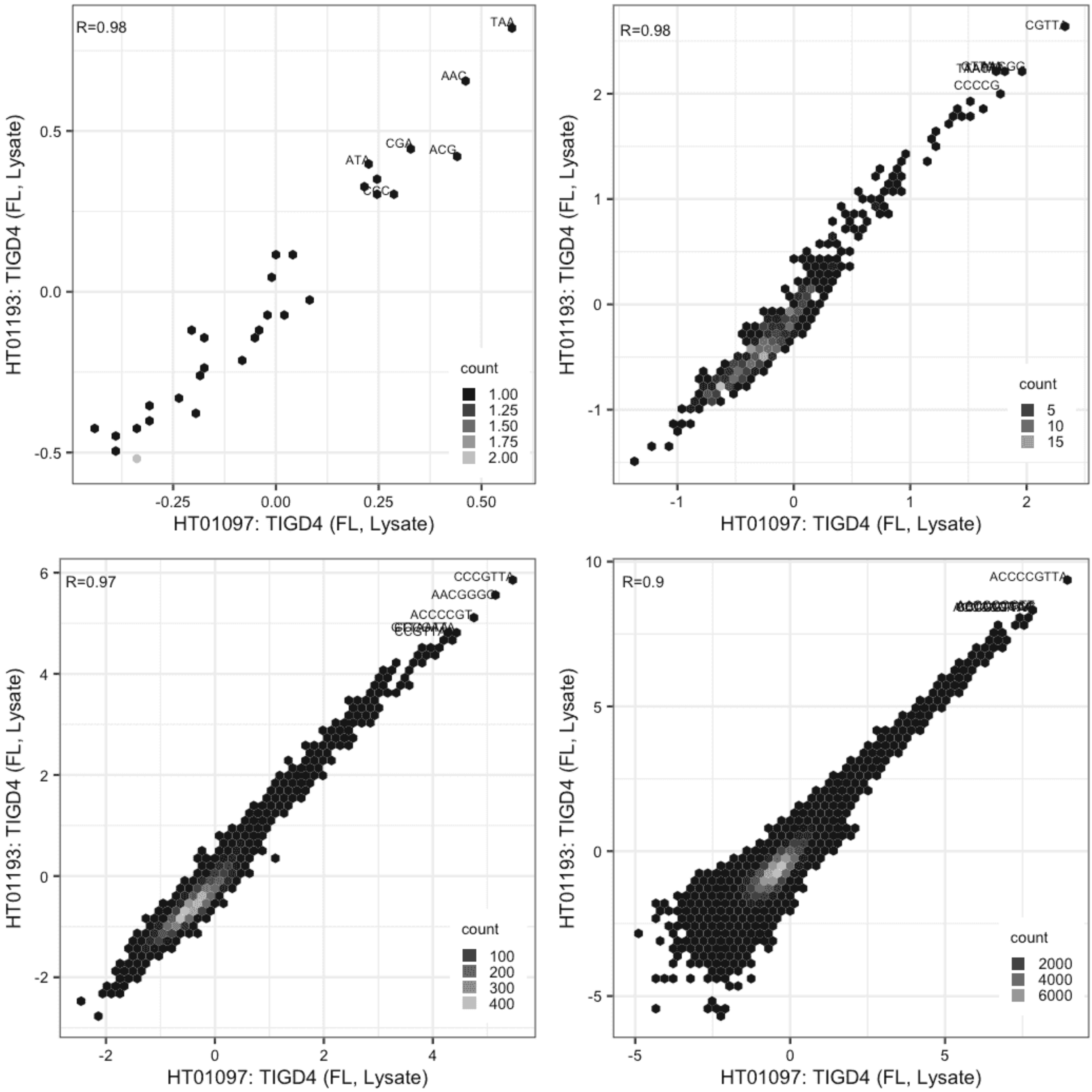

TIGD4

Lysate vs GFPIVT, same construct

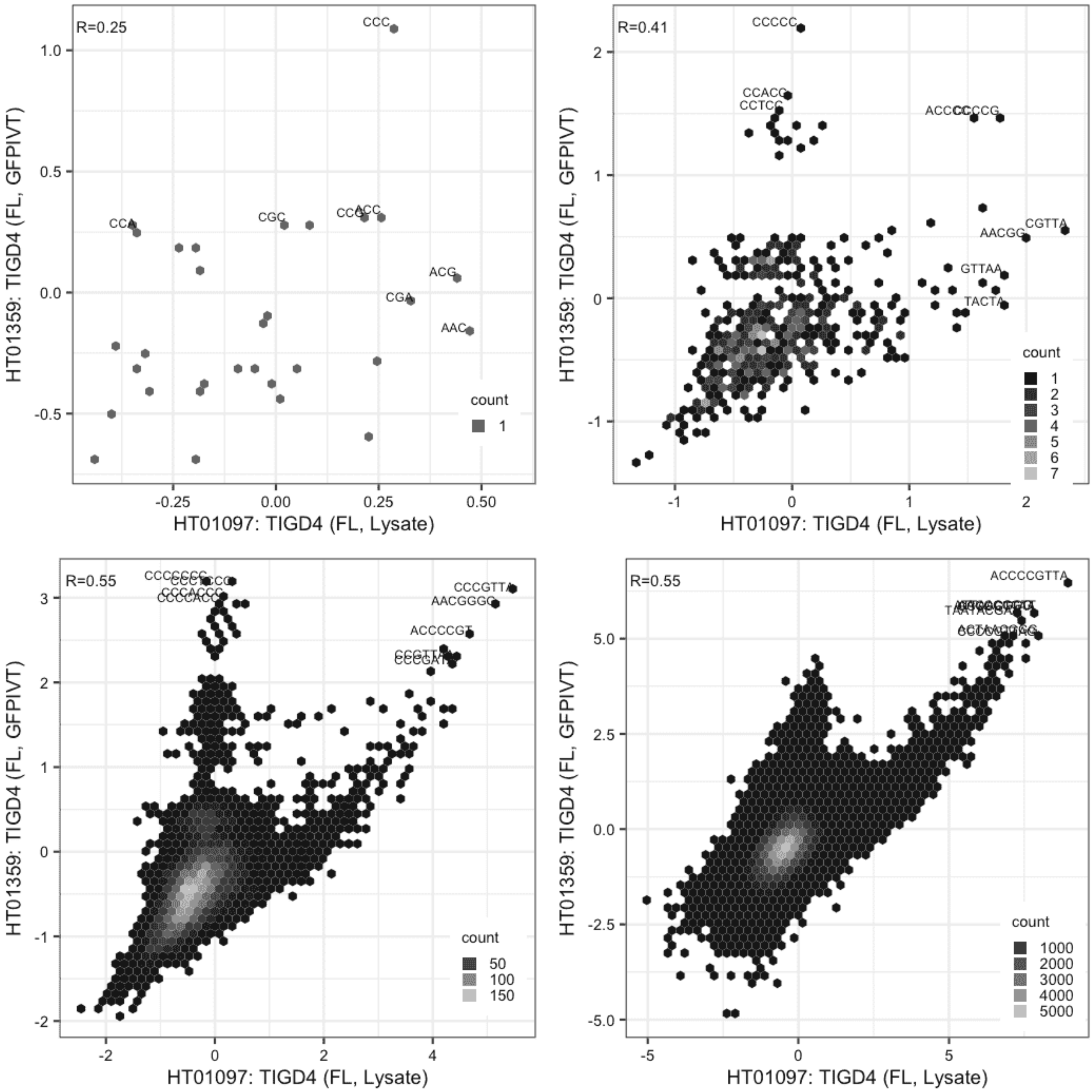

TIGD4

Lysate vs GFPIVT, same construct

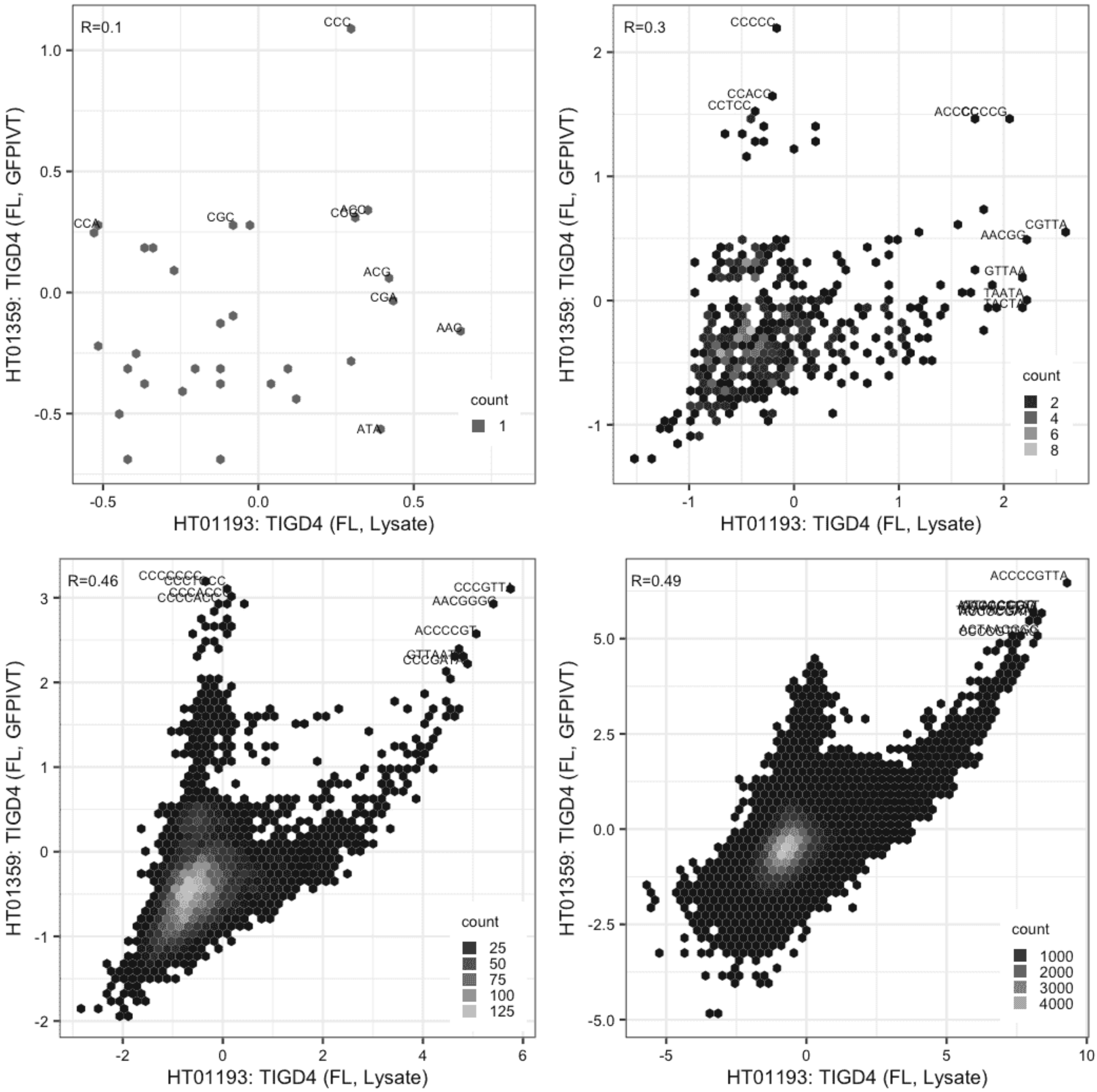

TIGD5

IVT vs GFPIVT, same construct

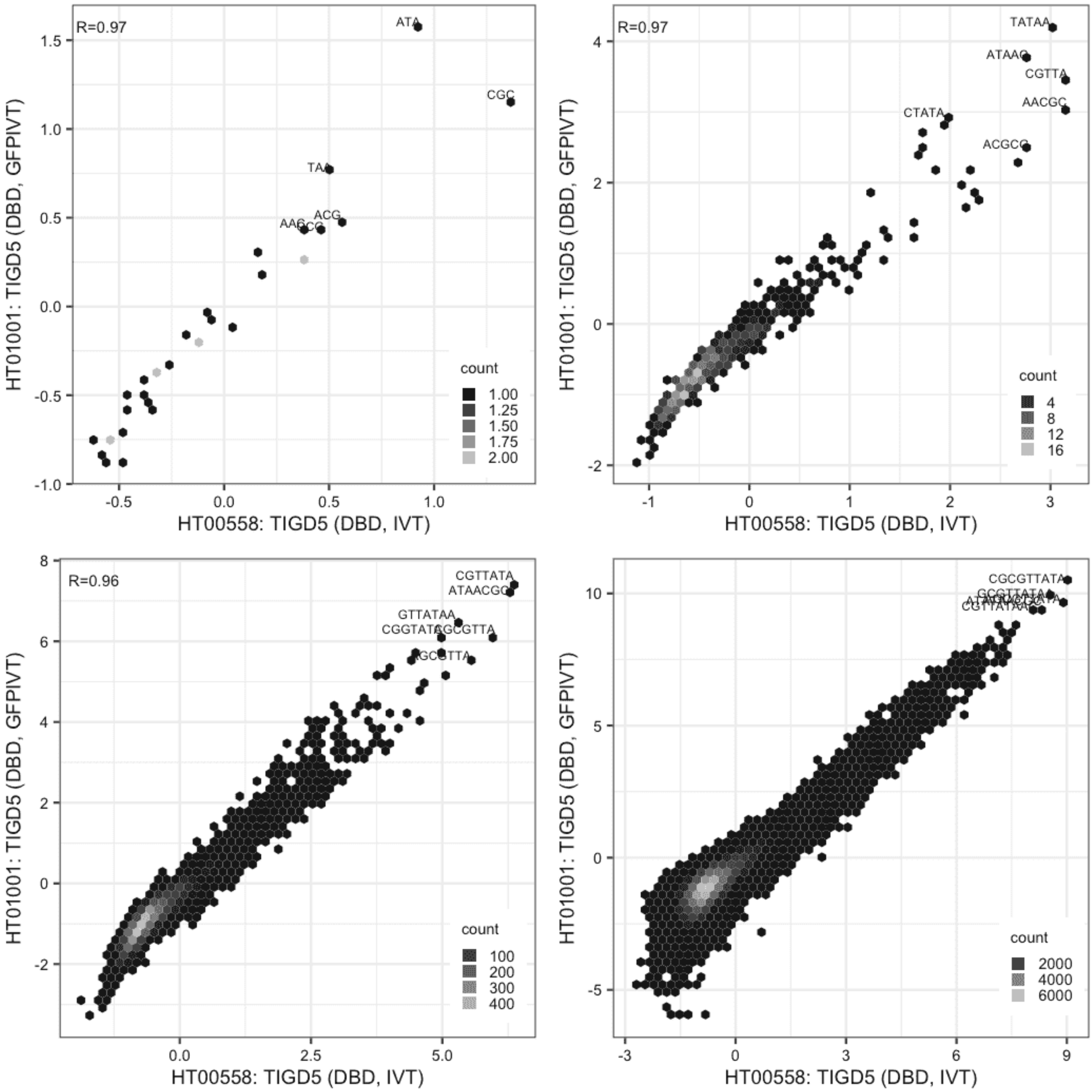

TIGD5

Lysate vs IVT, different constructs

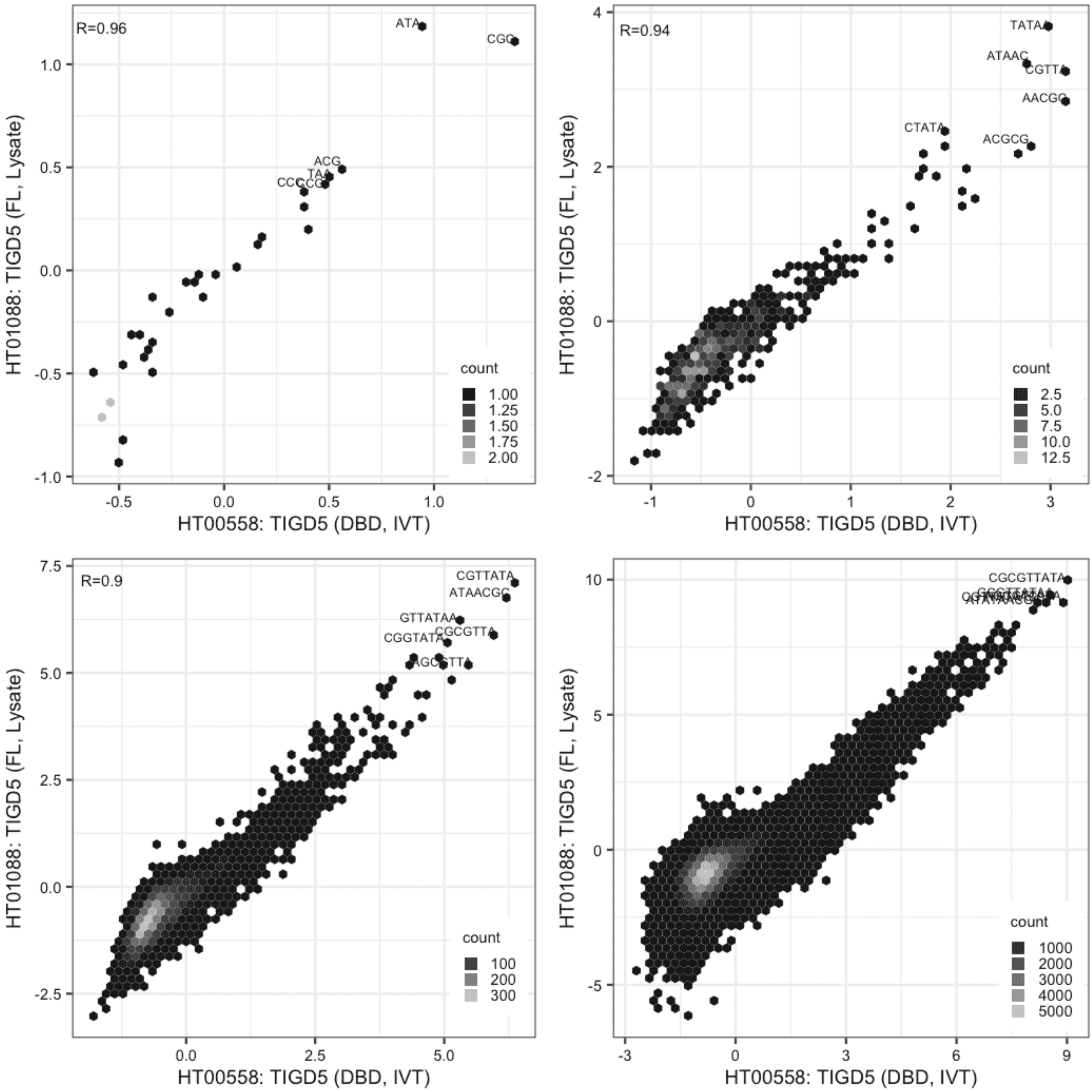

TIGD5

Lysate vs IVT, different constructs

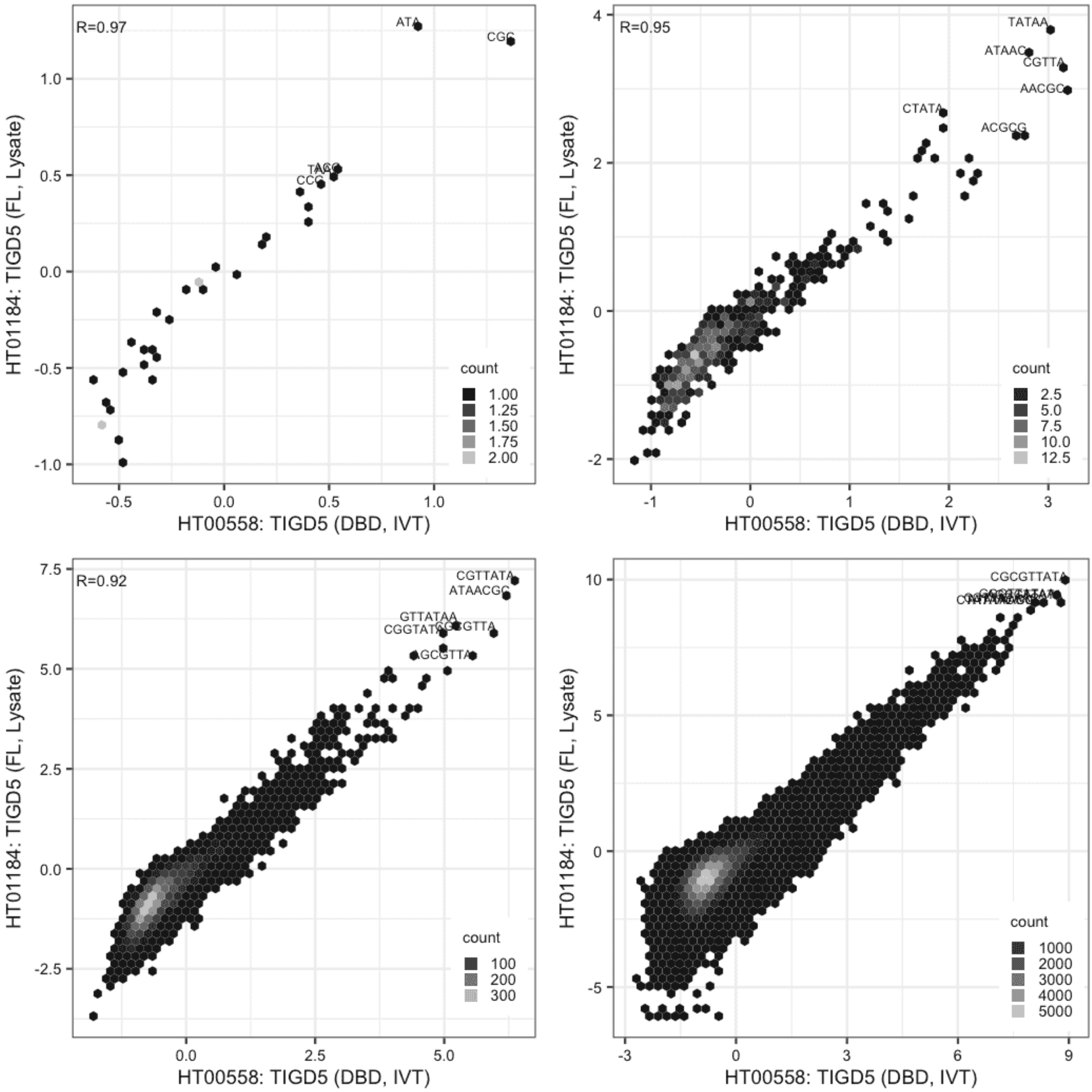

TIGD5

IVT vs GFPIVT, different constructs

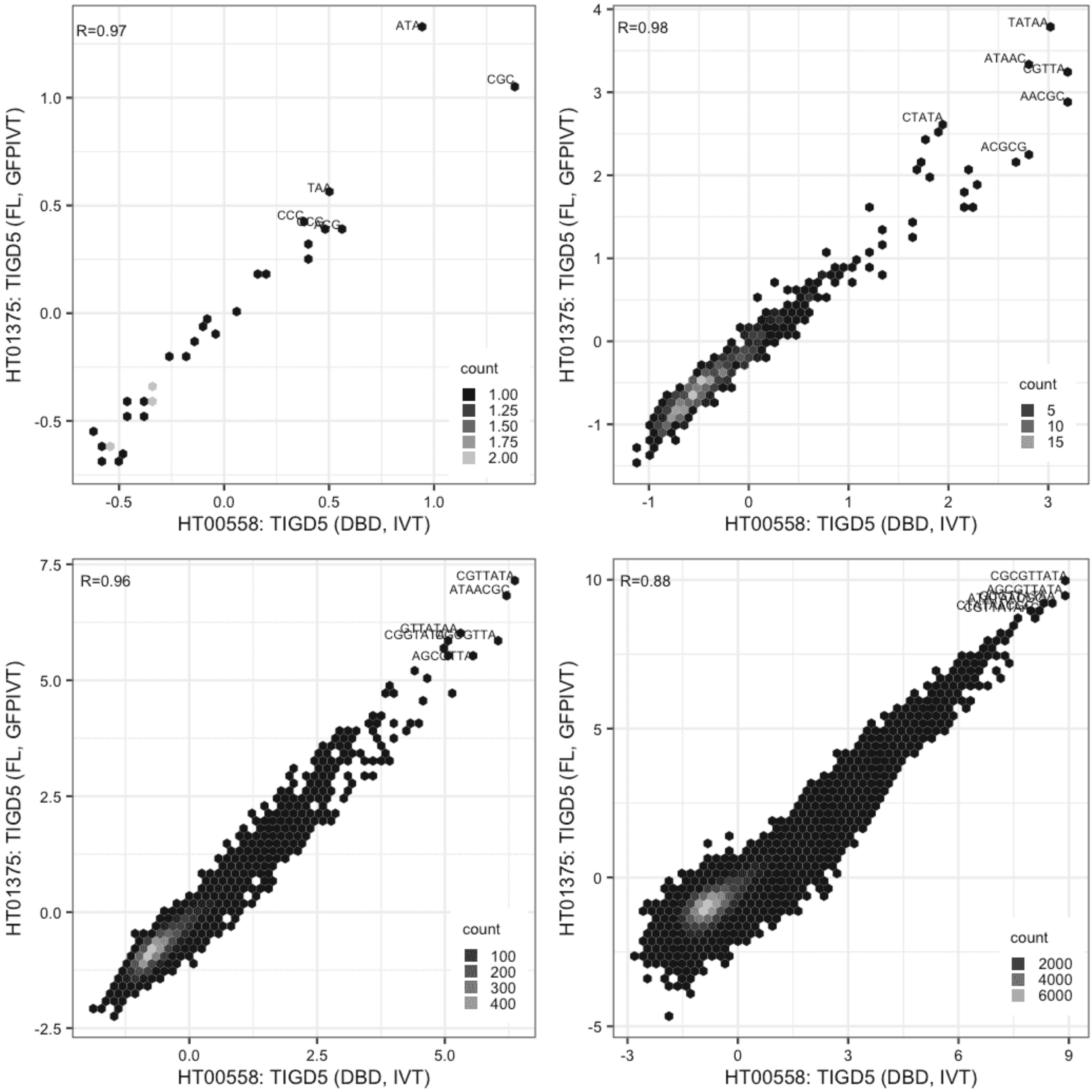

TIGD5

Lysate vs GFPIVT, different constructs

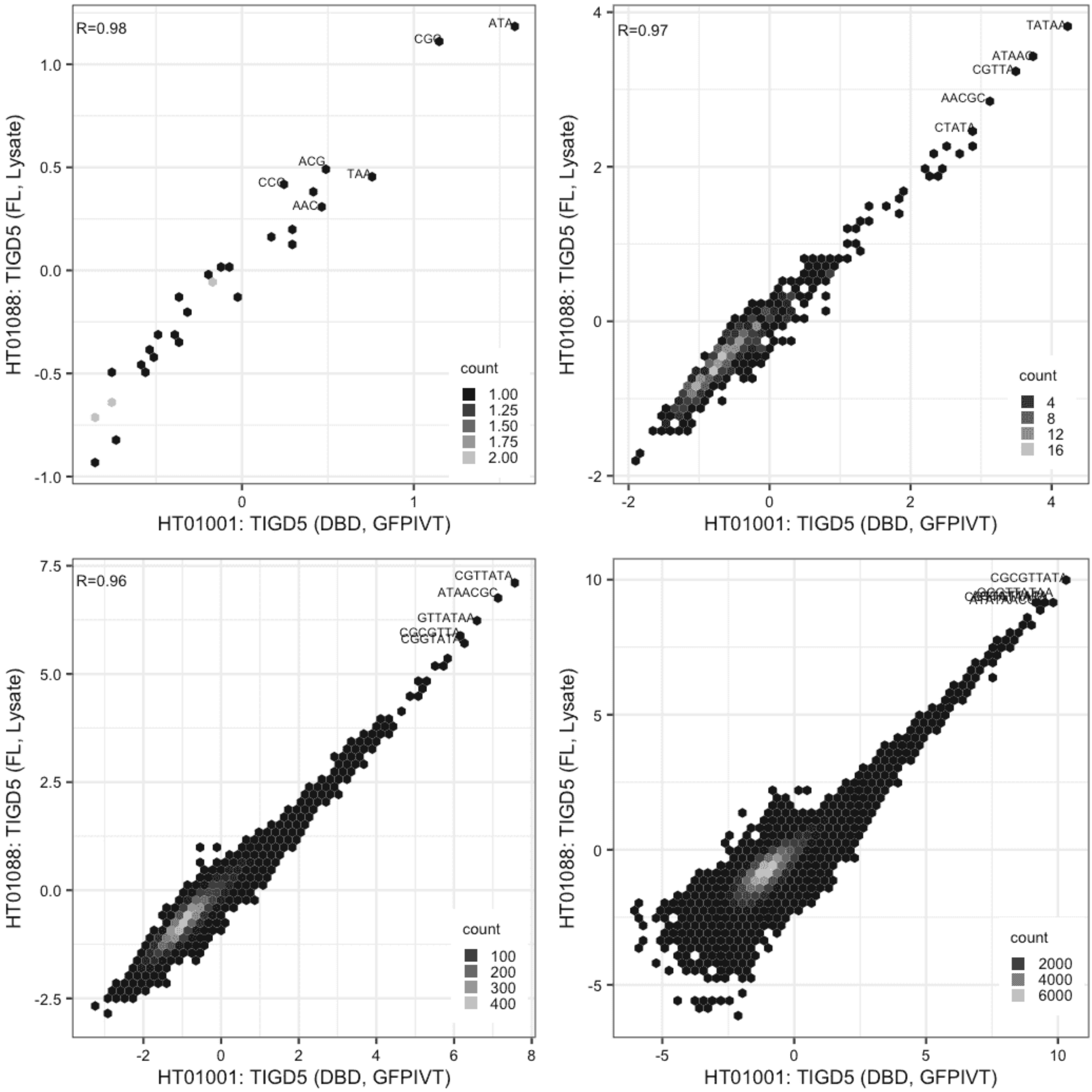

TIGD5

Lysate vs GFPIVT, different constructs

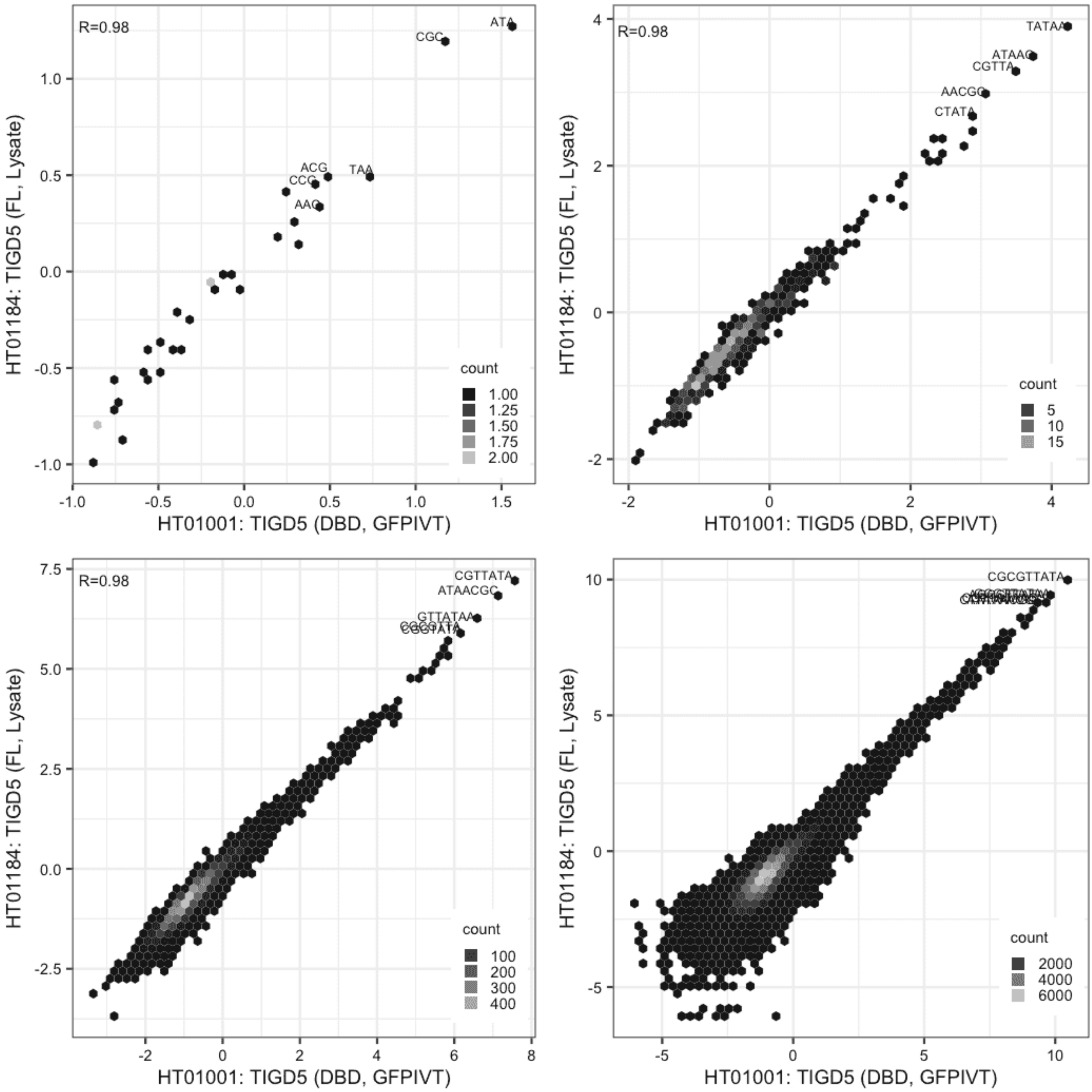

TIGD5

GFPIVT vs GFPIVT, different constructs

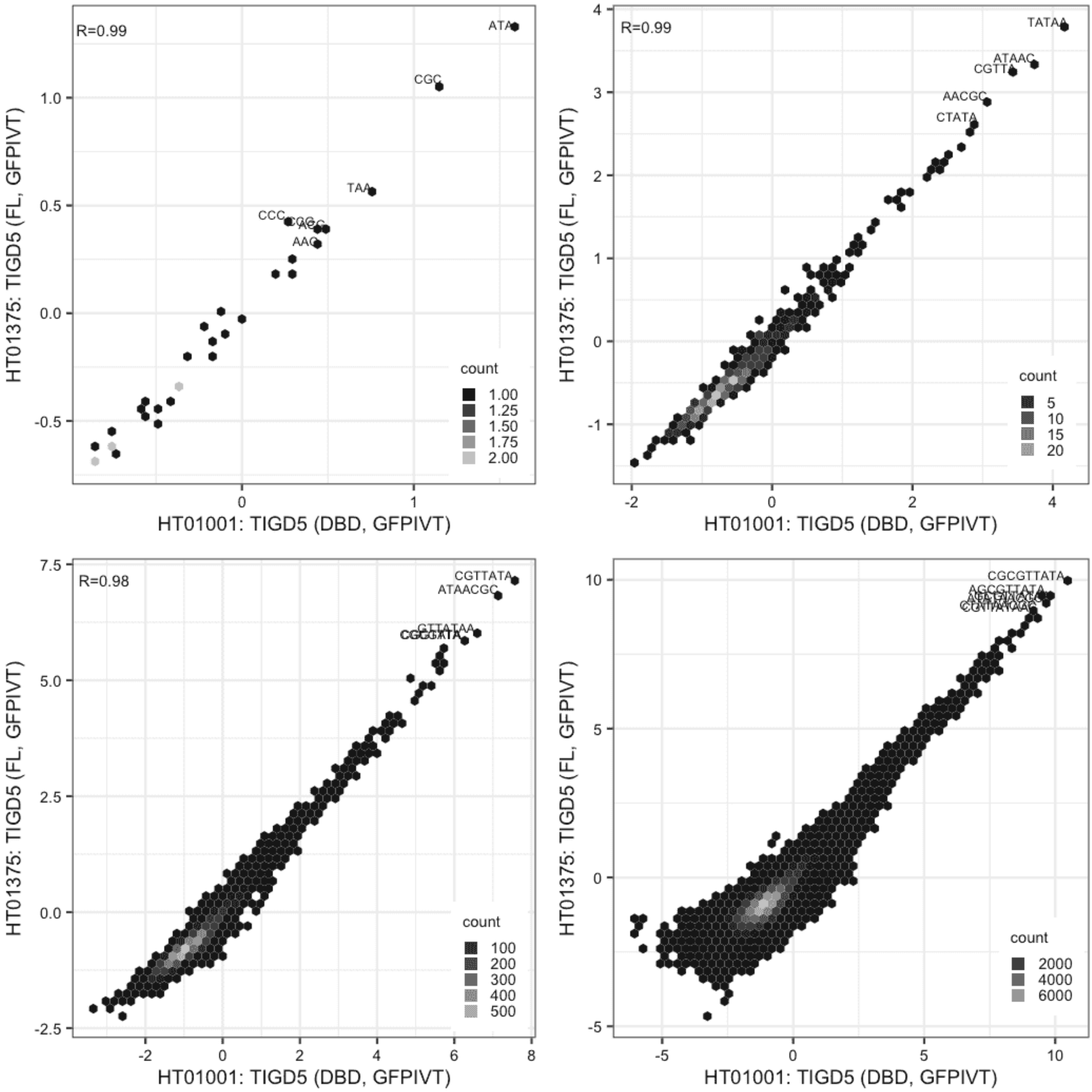

TIGD5

Lysate vs Lysate, same construct

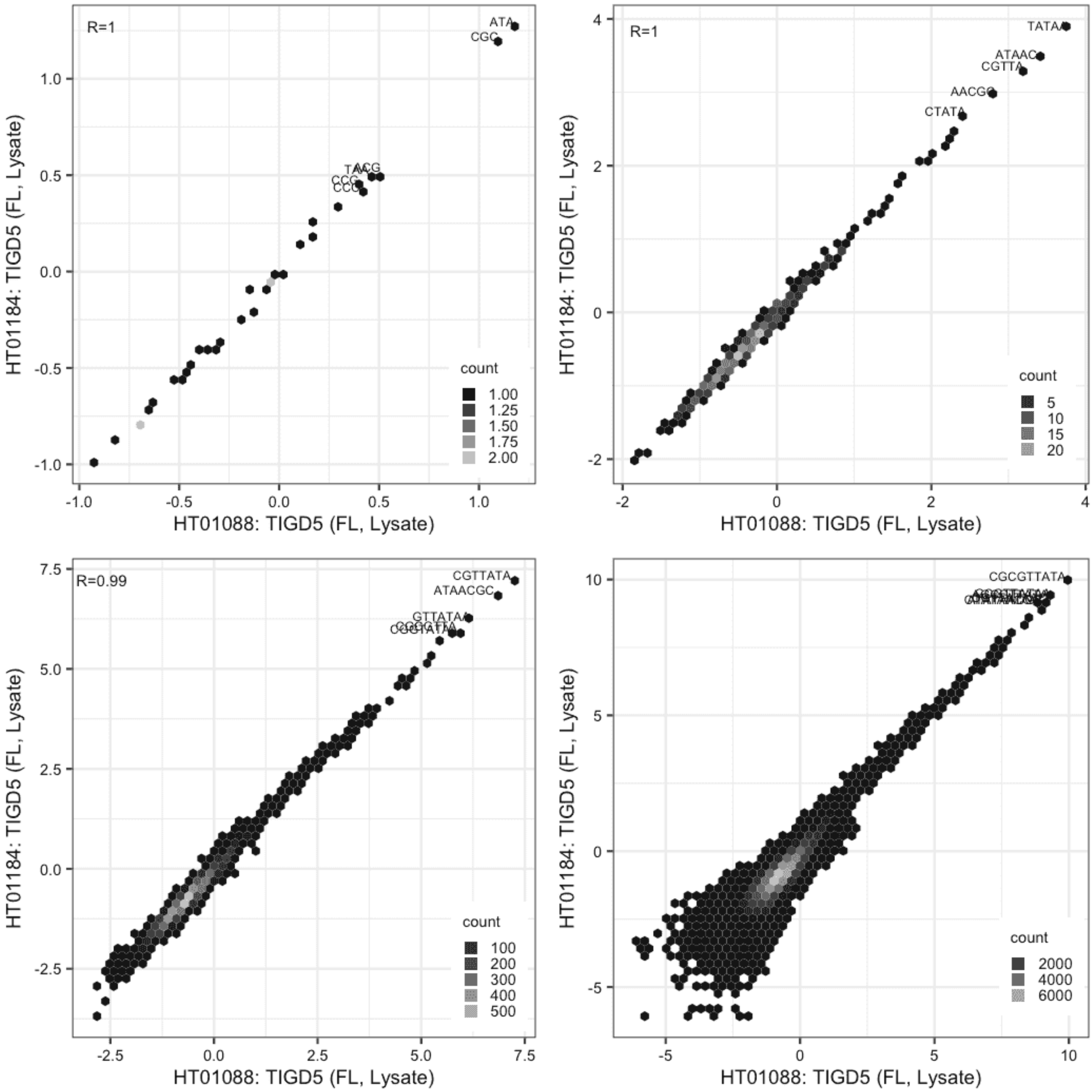

TIGD5

Lysate vs GFPIVT, same construct

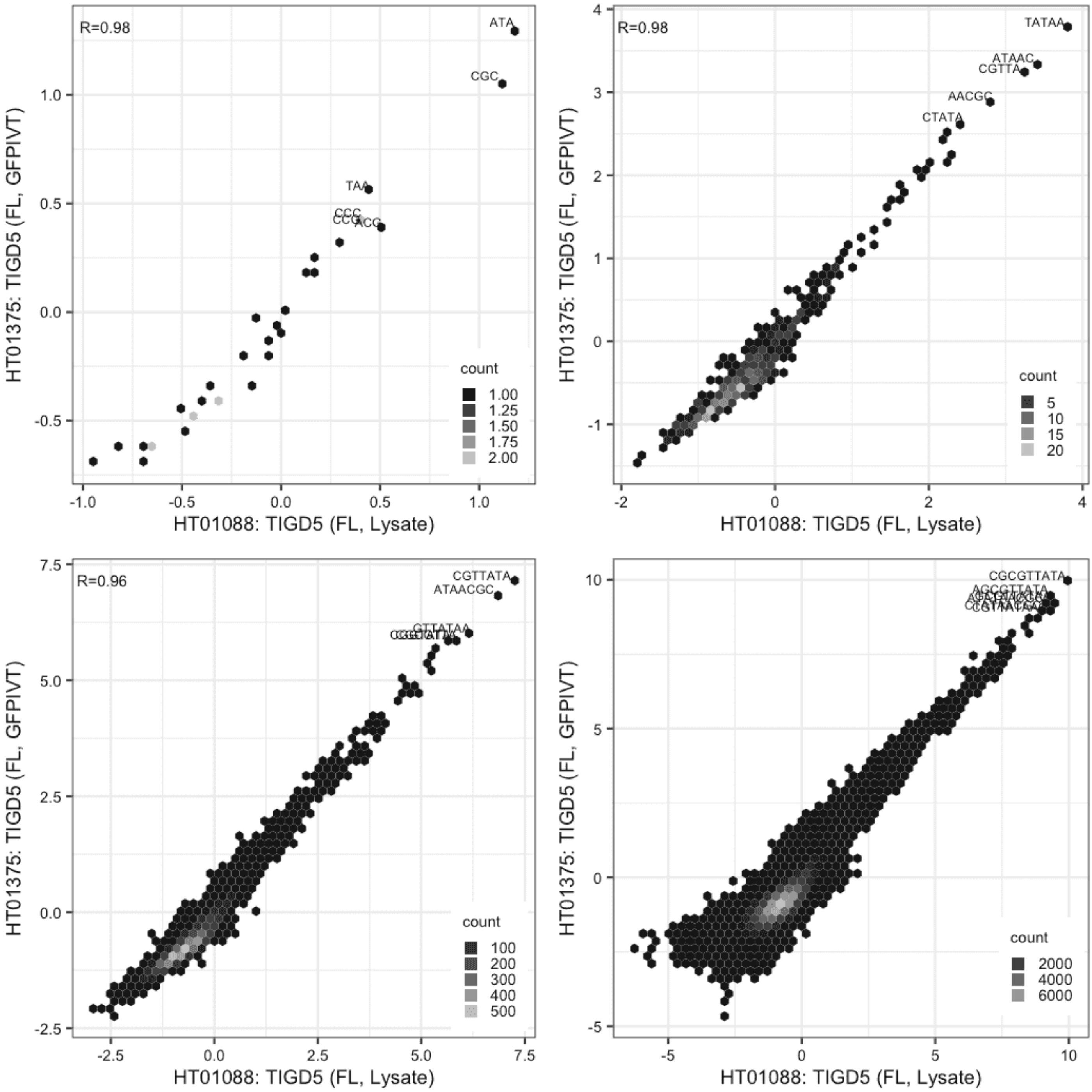

TIGD5

Lysate vs GFPIVT, same construct

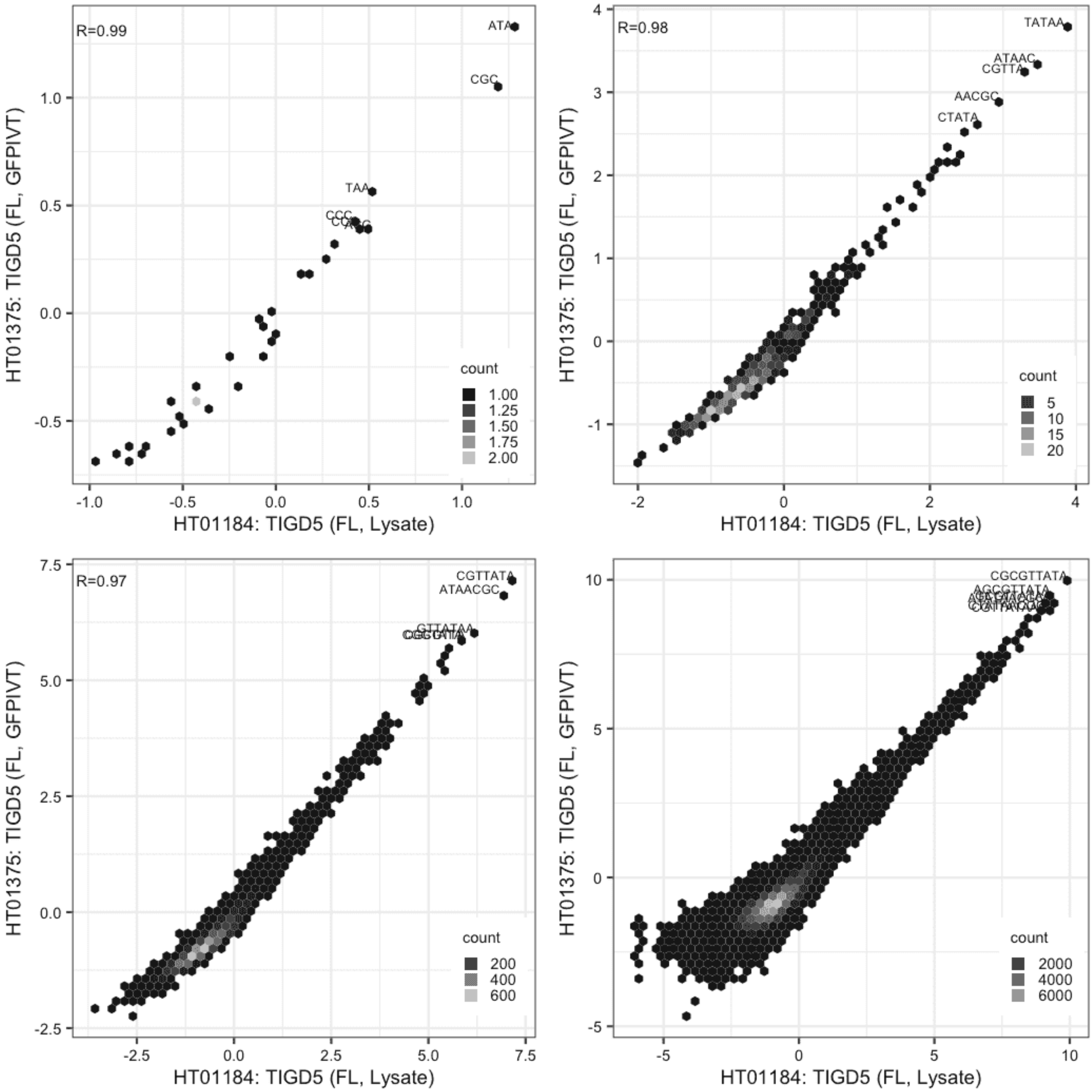

TPRX1

Lysate vs IVT, different constructs

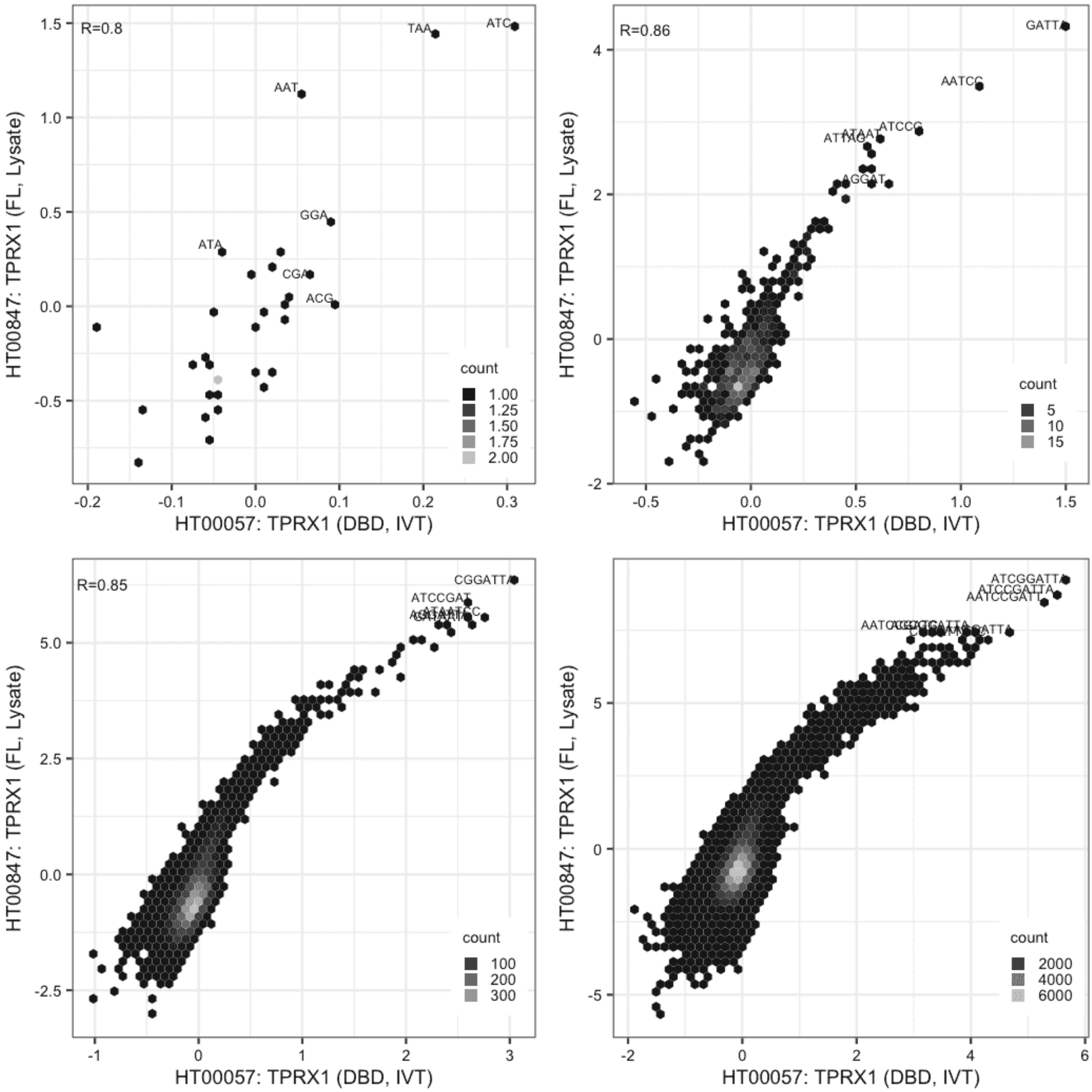

TPRX1

IVT vs GFPIVT, same construct

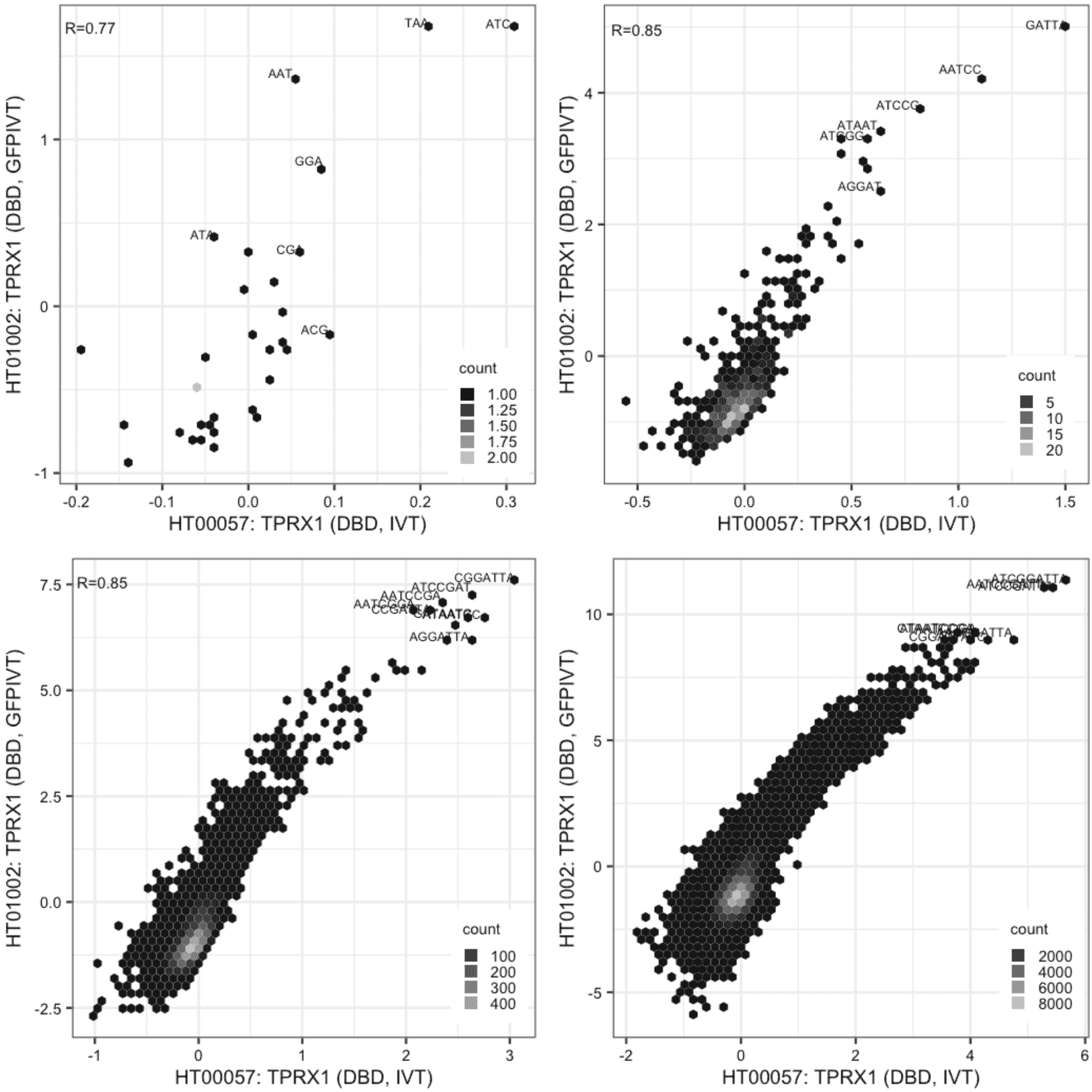

TPRX1

IVT vs GFPIVT, different constructs

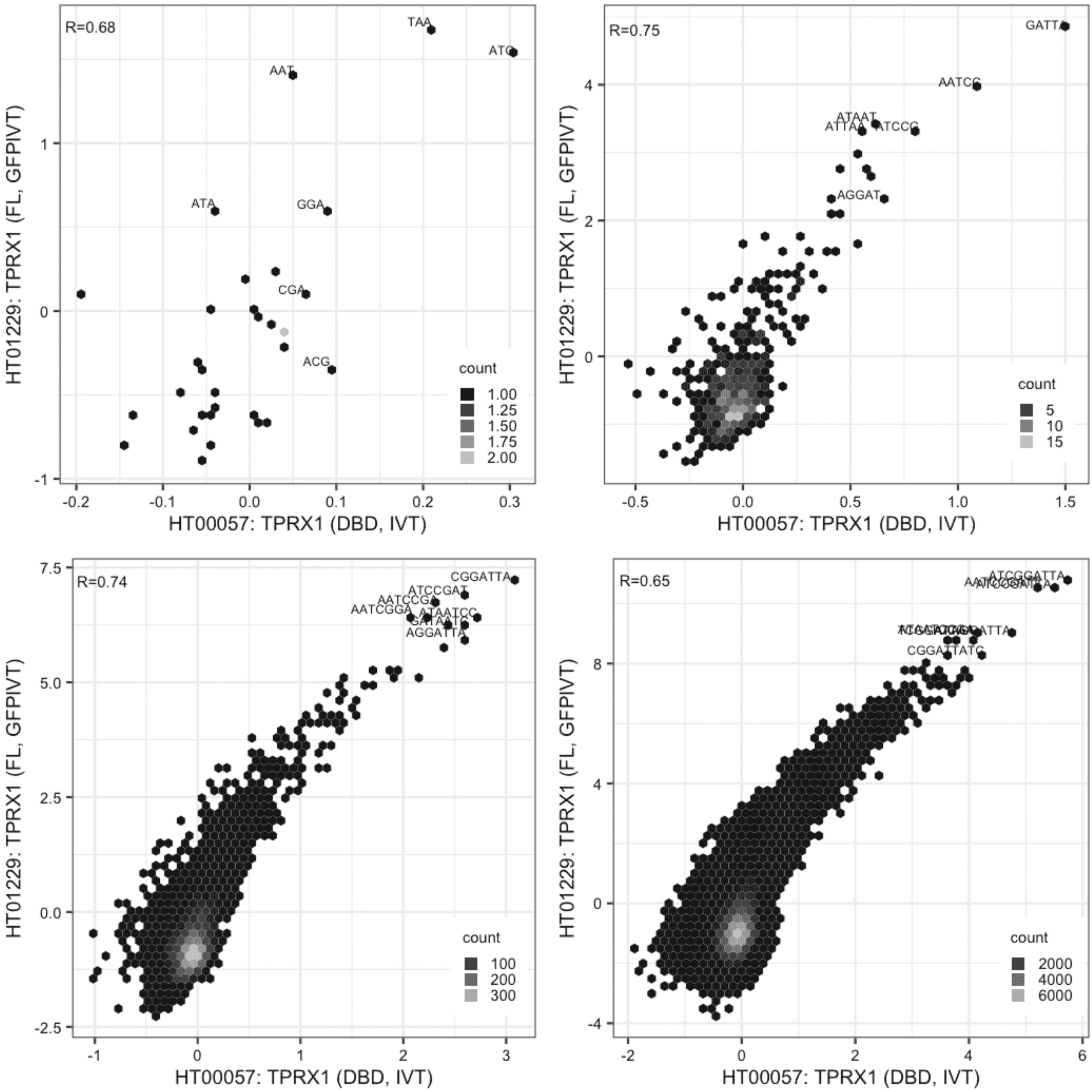

TPRX1

Lysate vs GFPIVT, different constructs

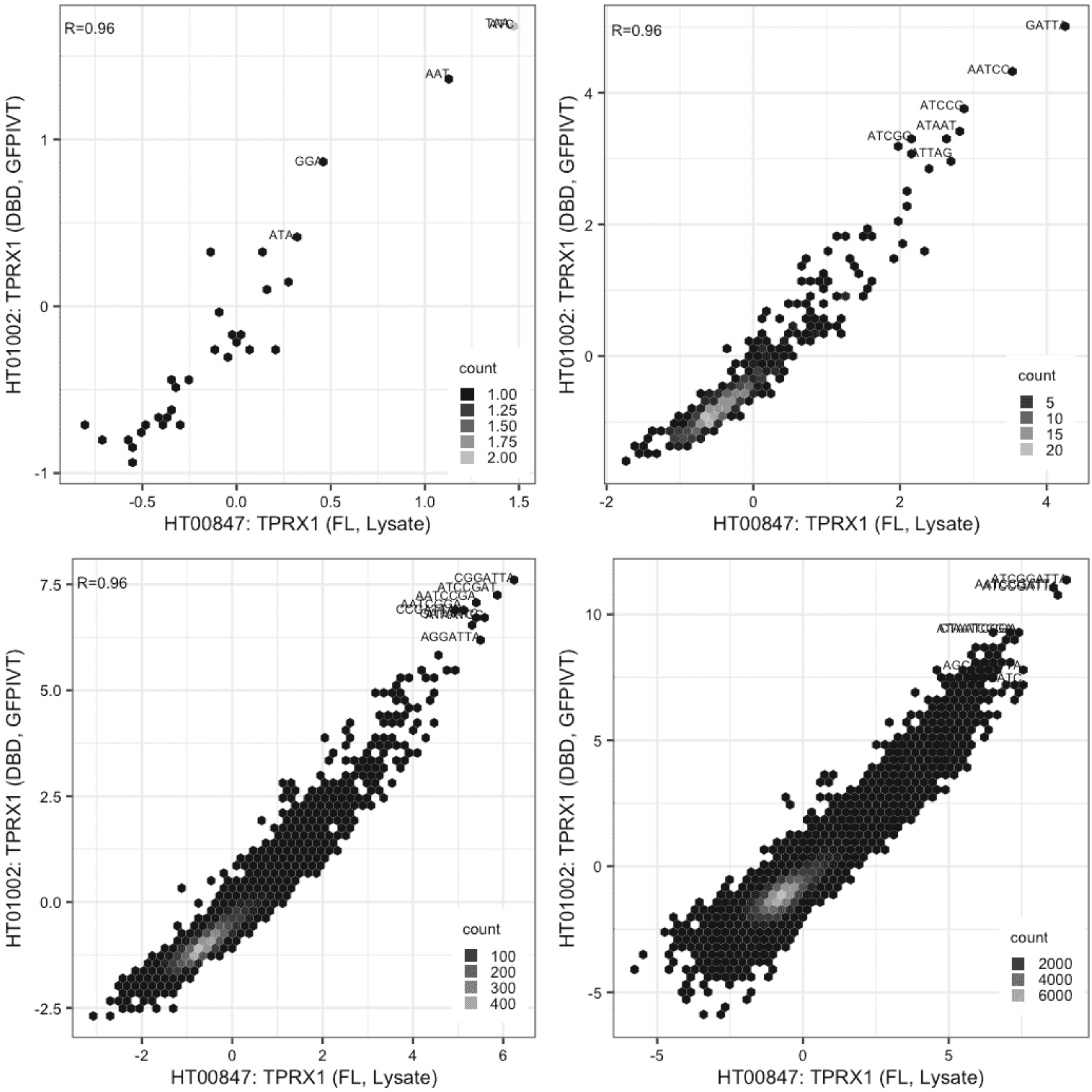

TPRX1

Lysate vs GFPIVT, same construct

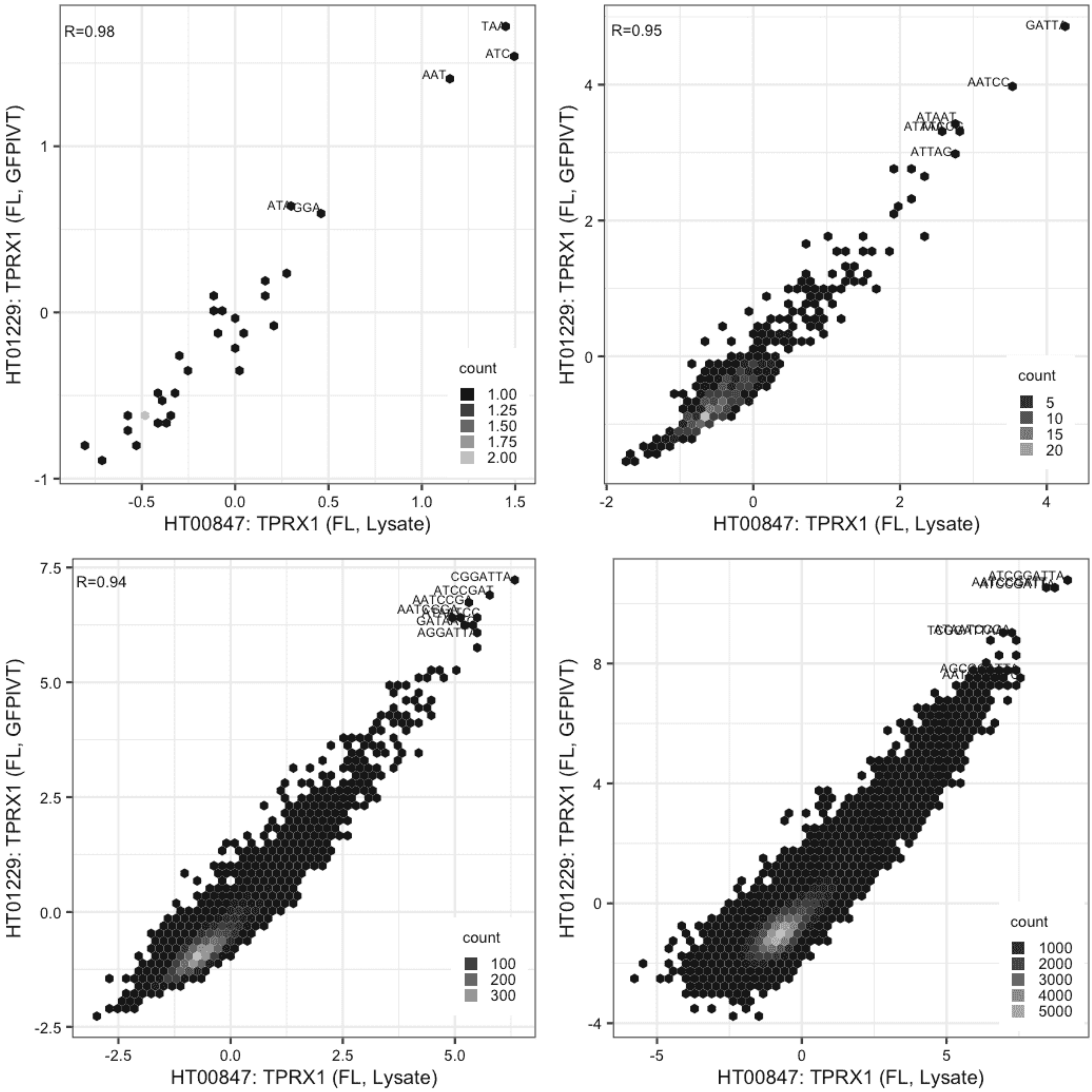

TPRX1

GFPIVT vs GFPIVT, different constructs

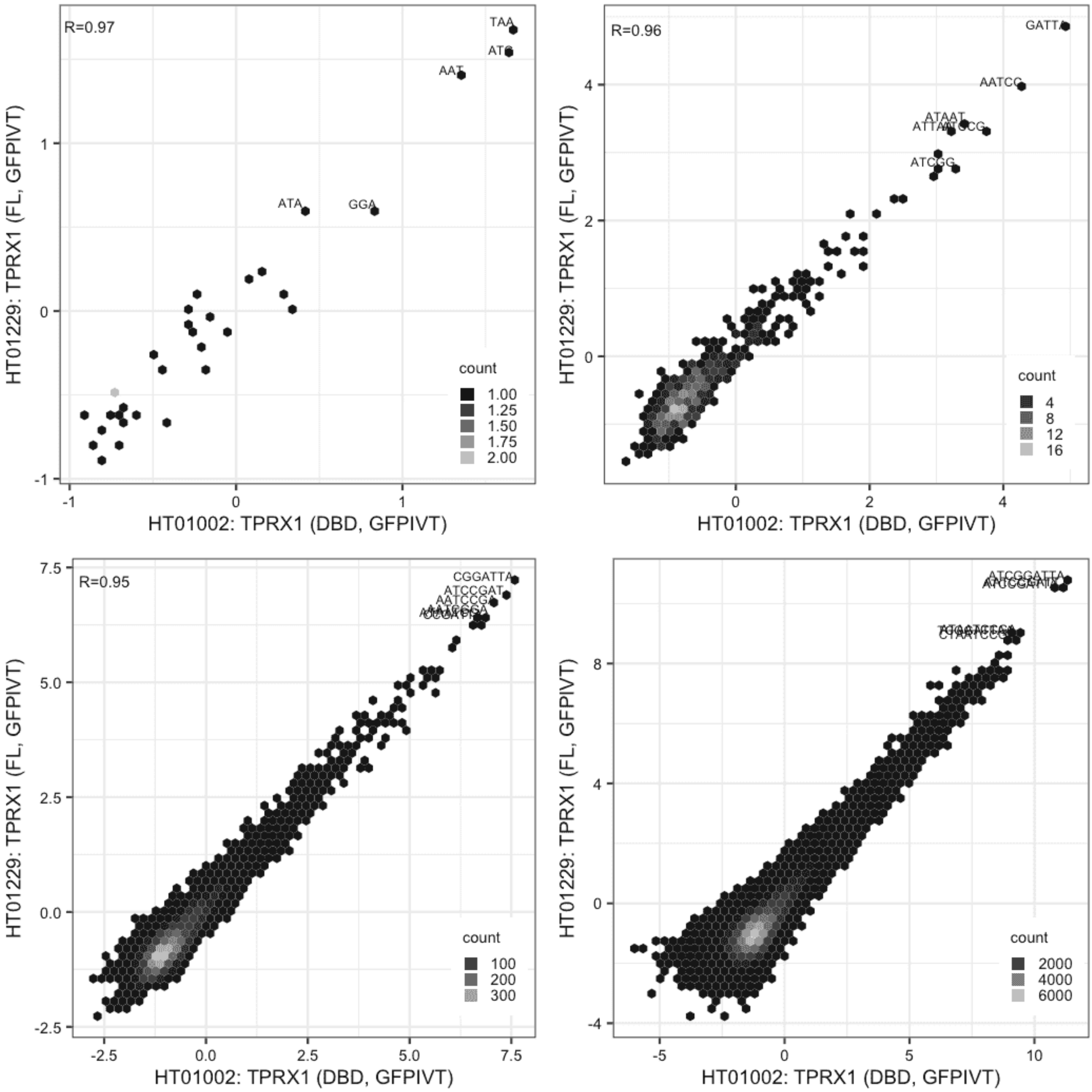

USF3

Lysate vs IVT, different constructs

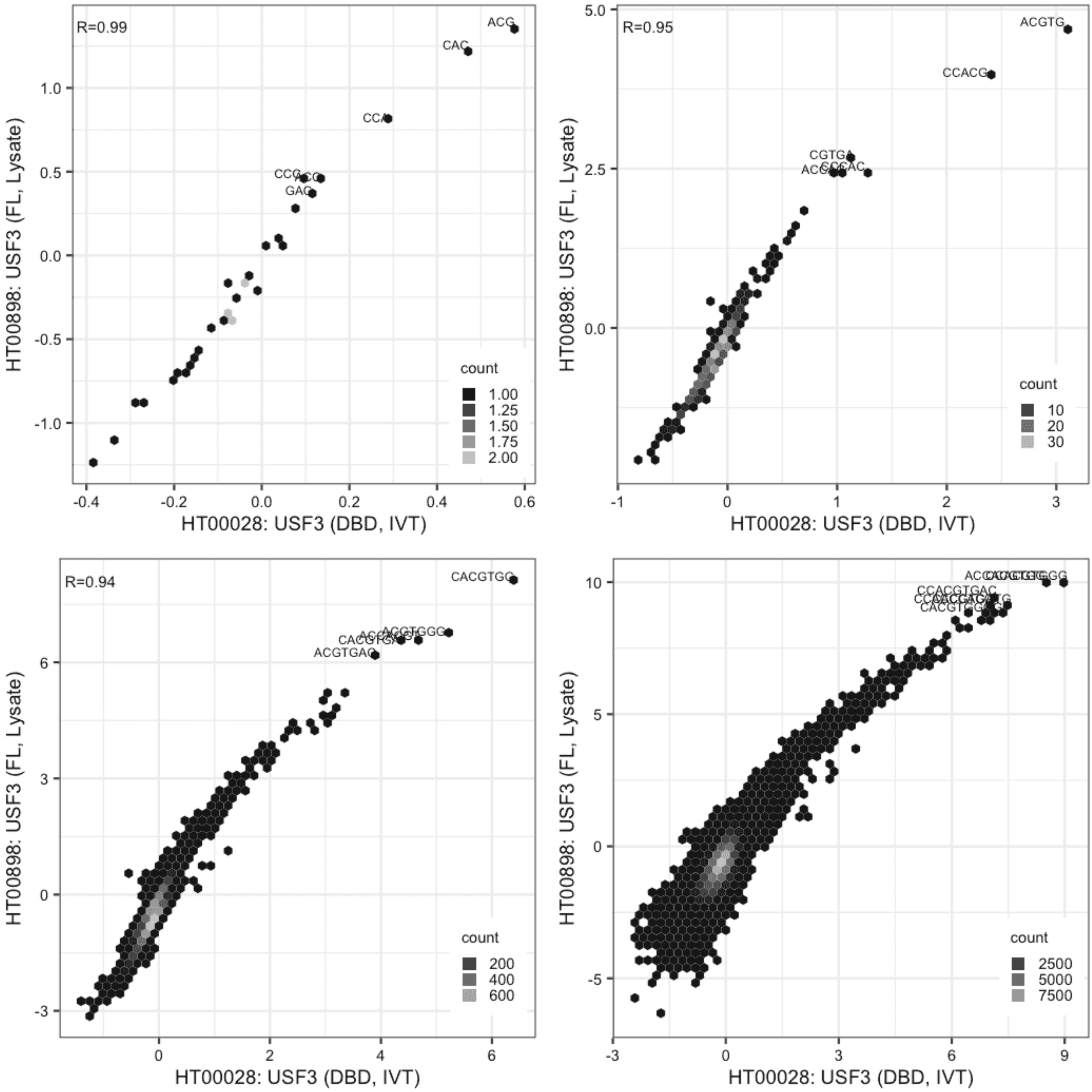

USF3

IVT vs GFPIVT, same construct

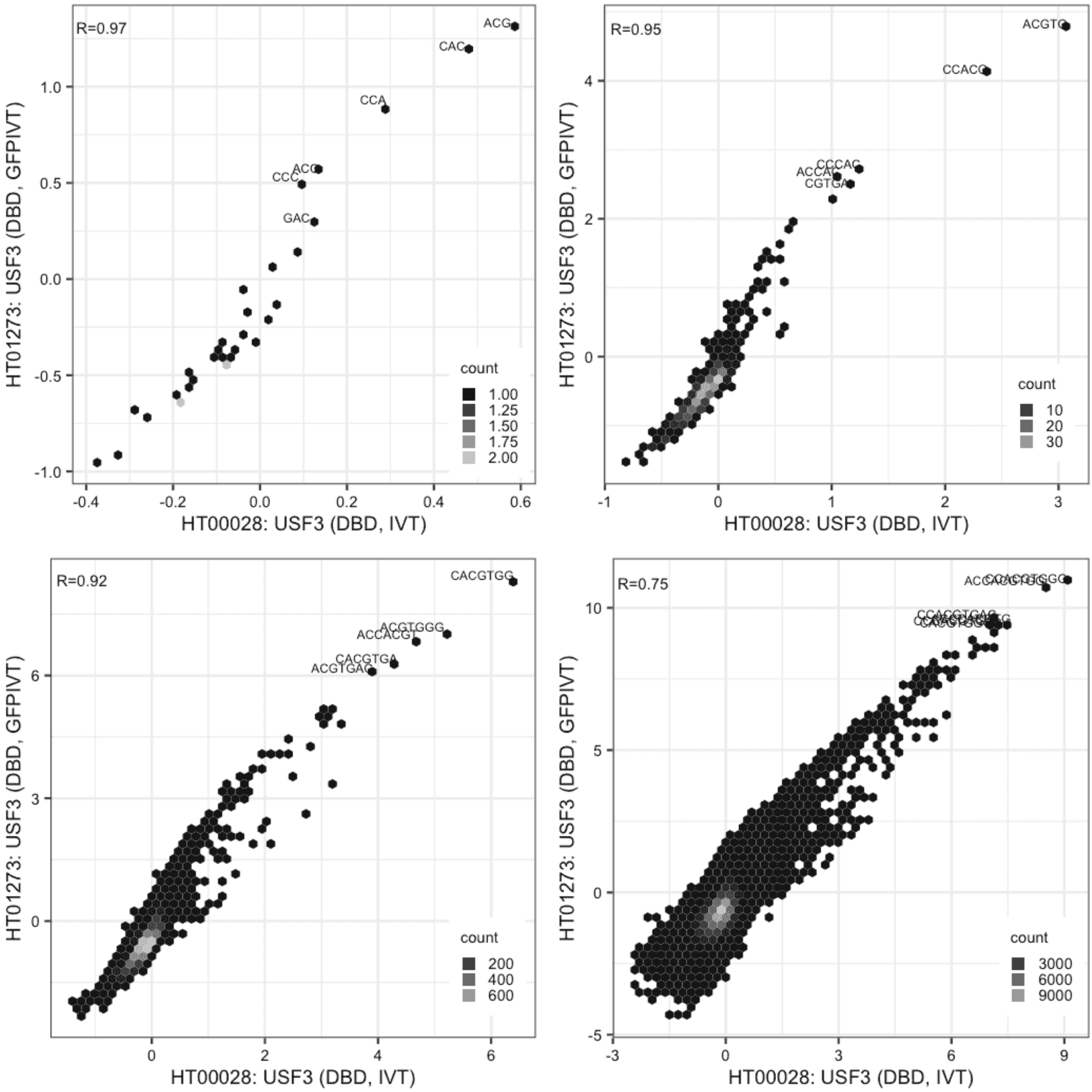

USF3

Lysate vs GFPIVT, different constructs

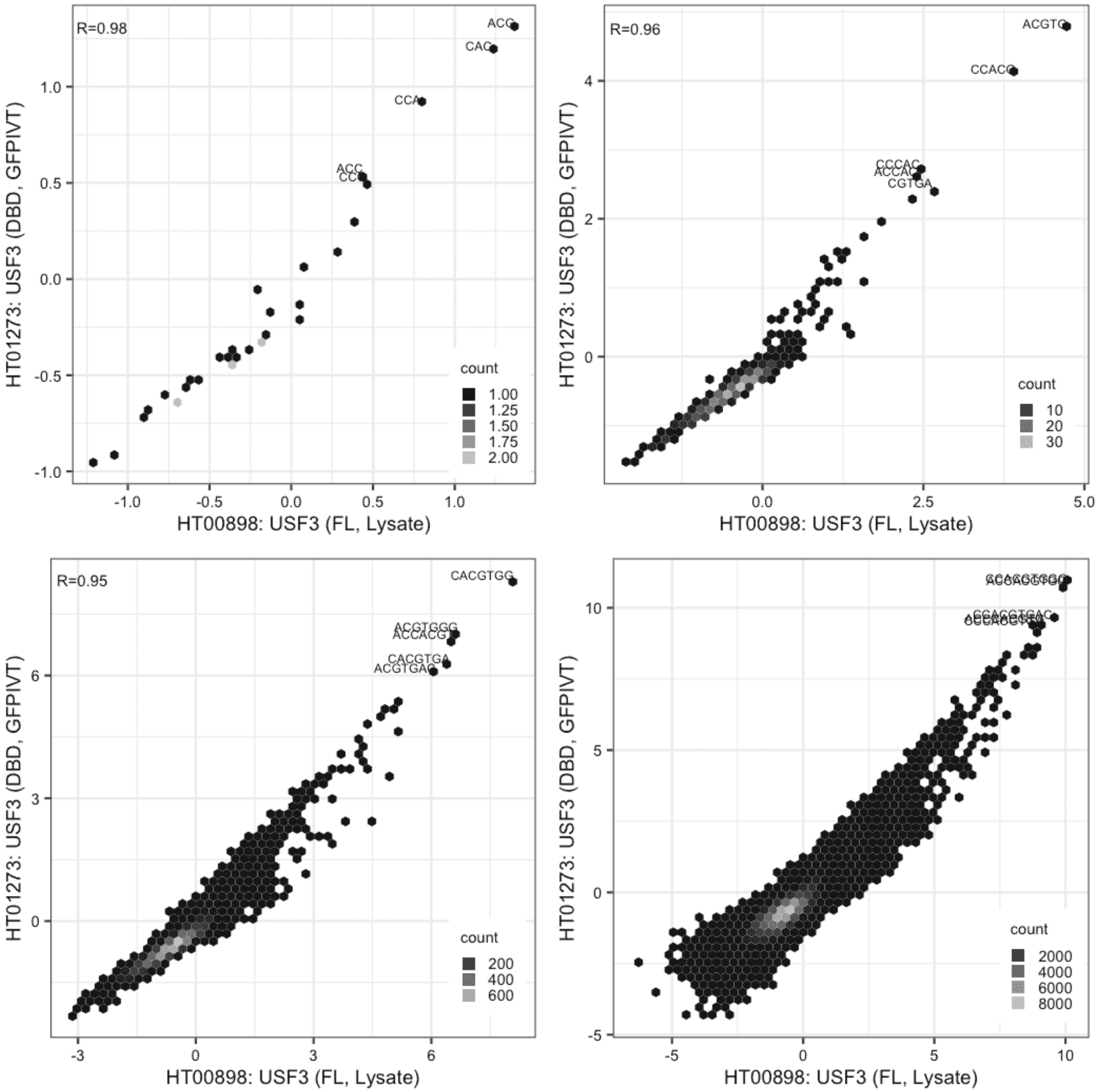

VDR

IVT vs IVT, same construct

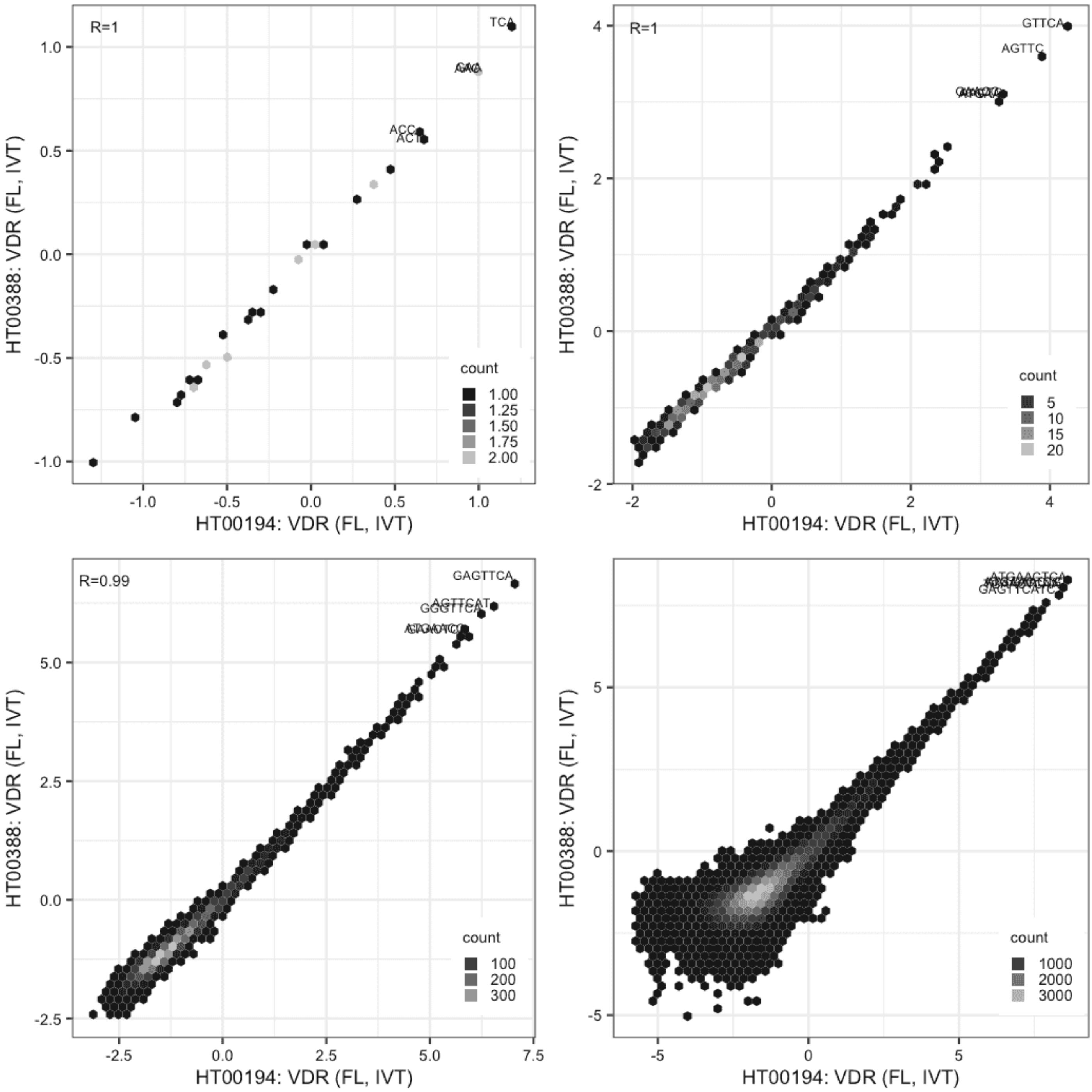

VDR

Lysate vs IVT, same construct

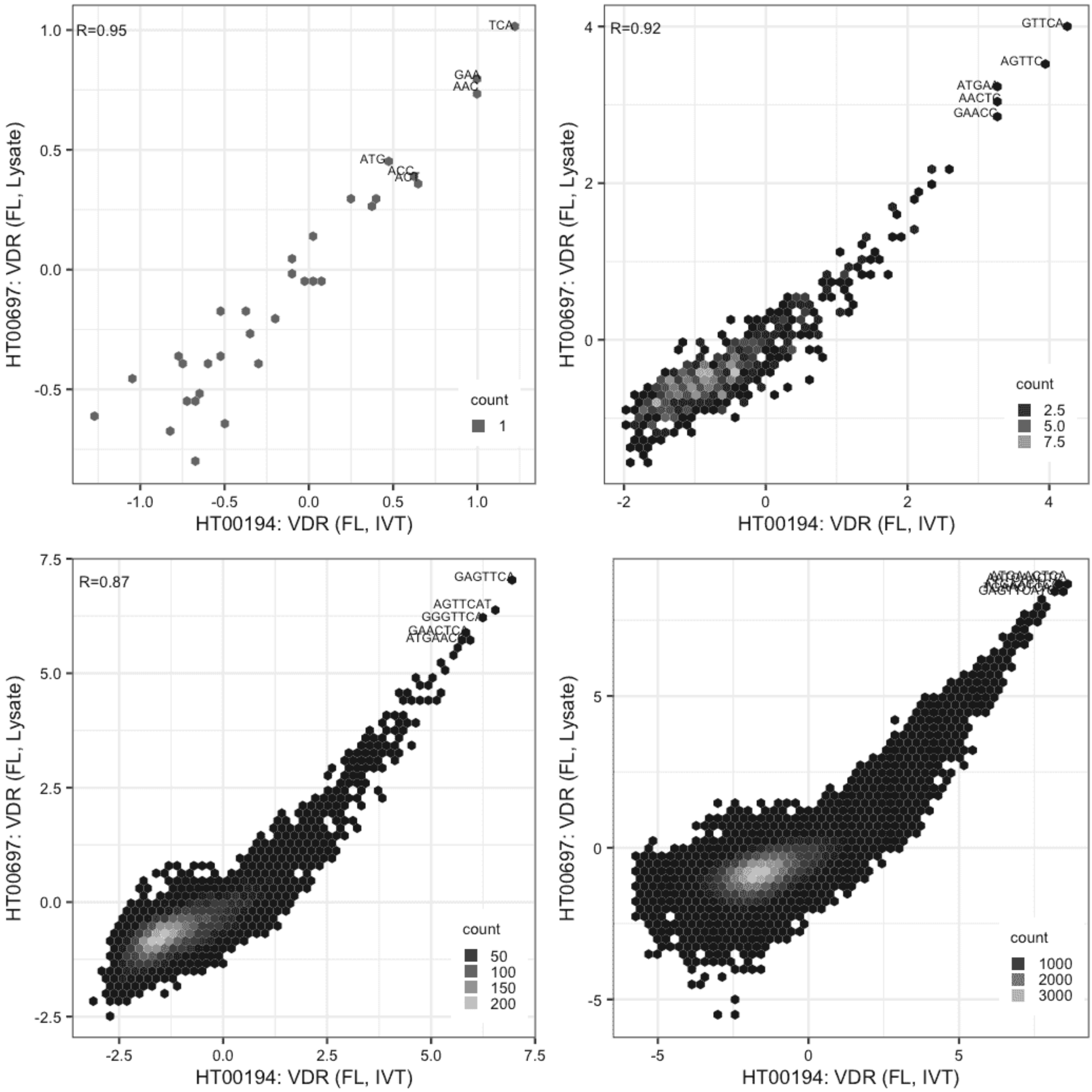

VDR

Lysate vs IVT, same construct

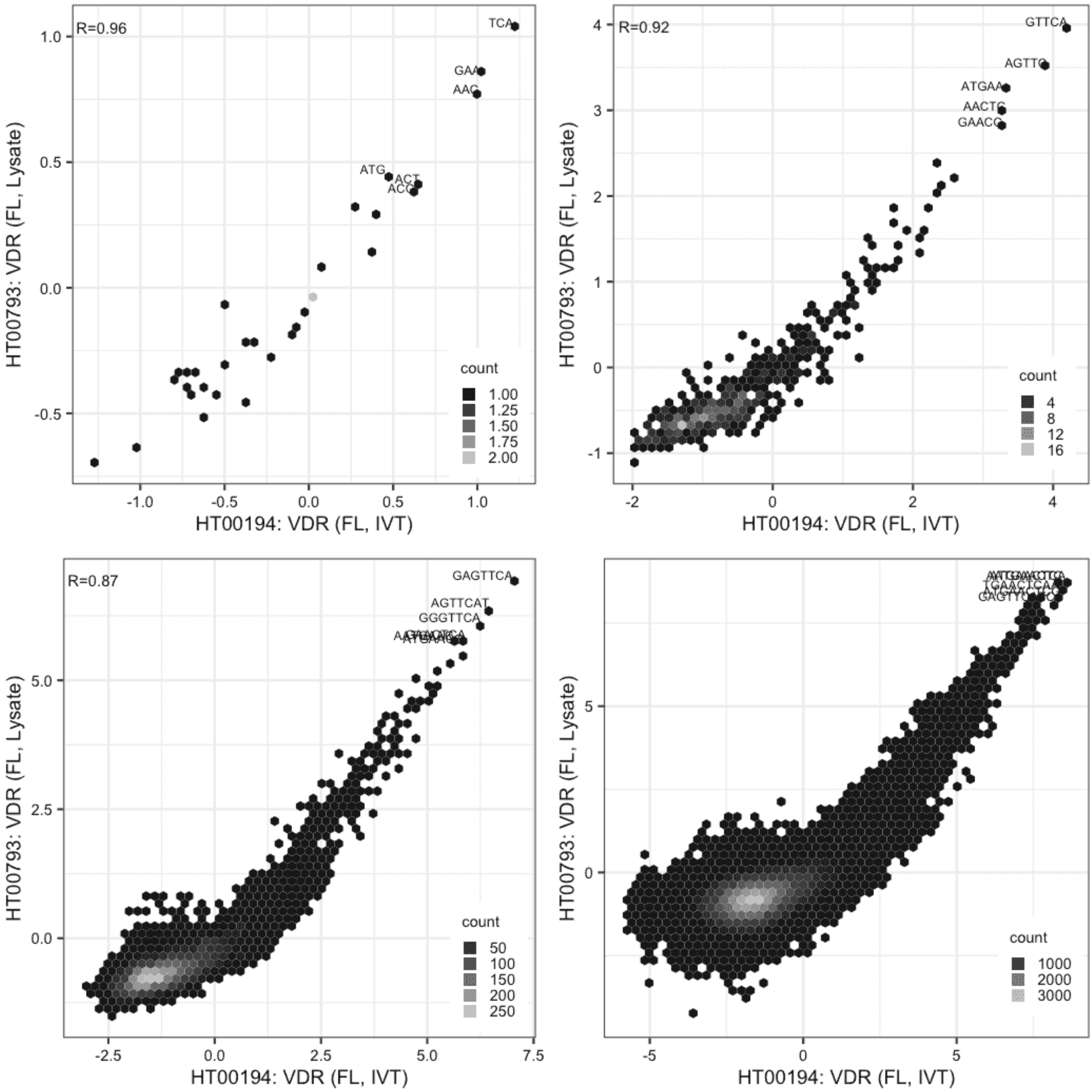

VDR

Lysate vs IVT, same construct

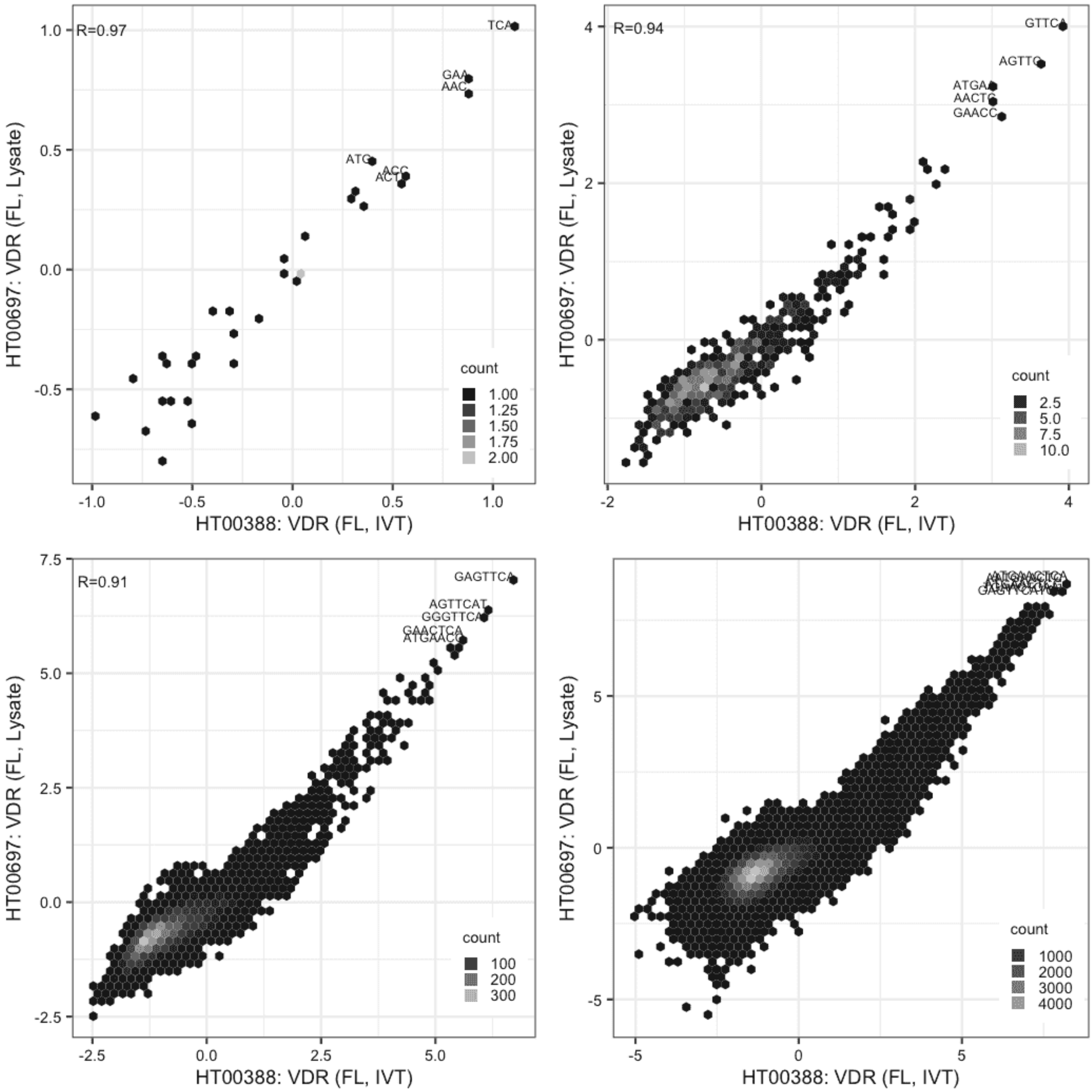

VDR

Lysate vs IVT, same construct

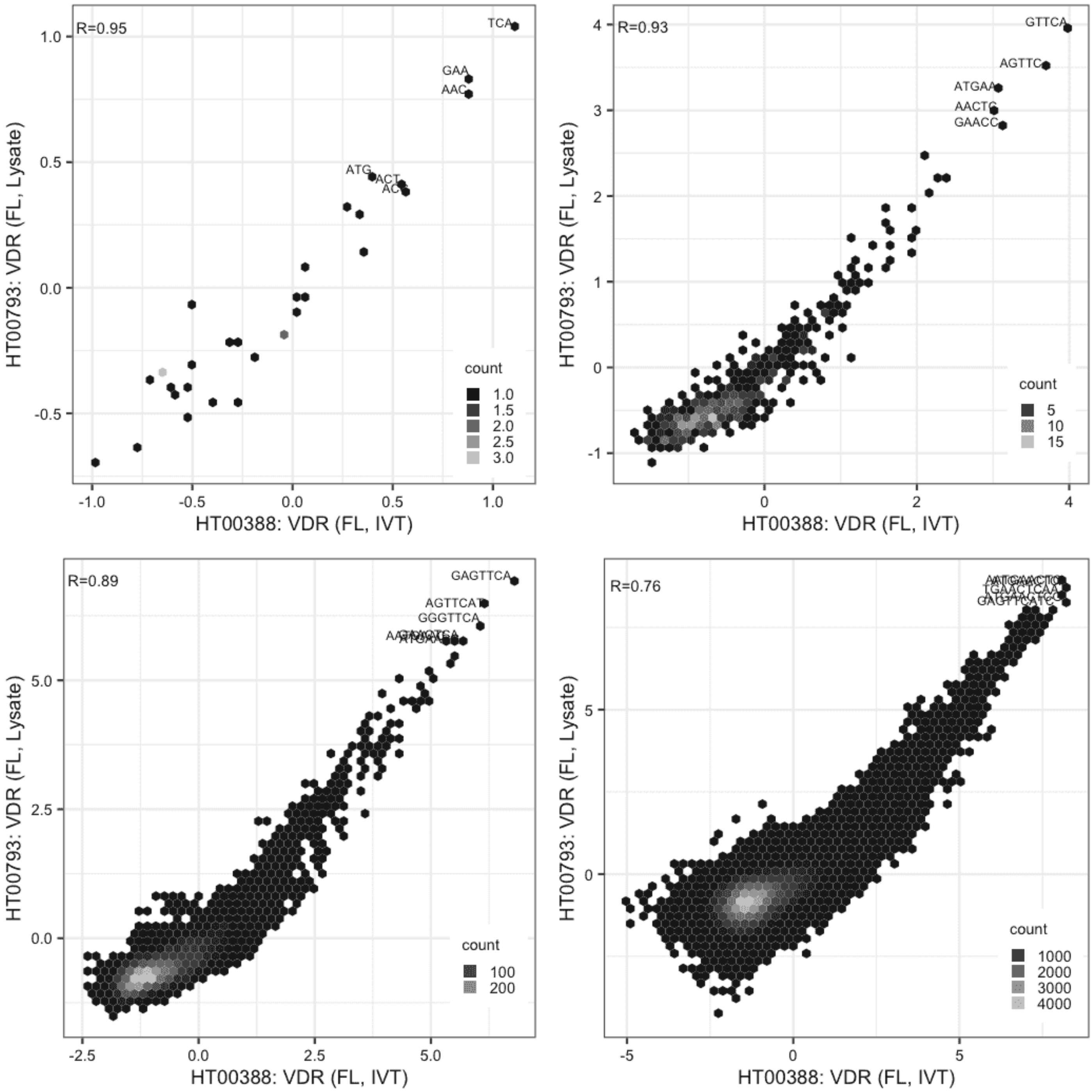

VDR

Lysate vs Lysate, same construct

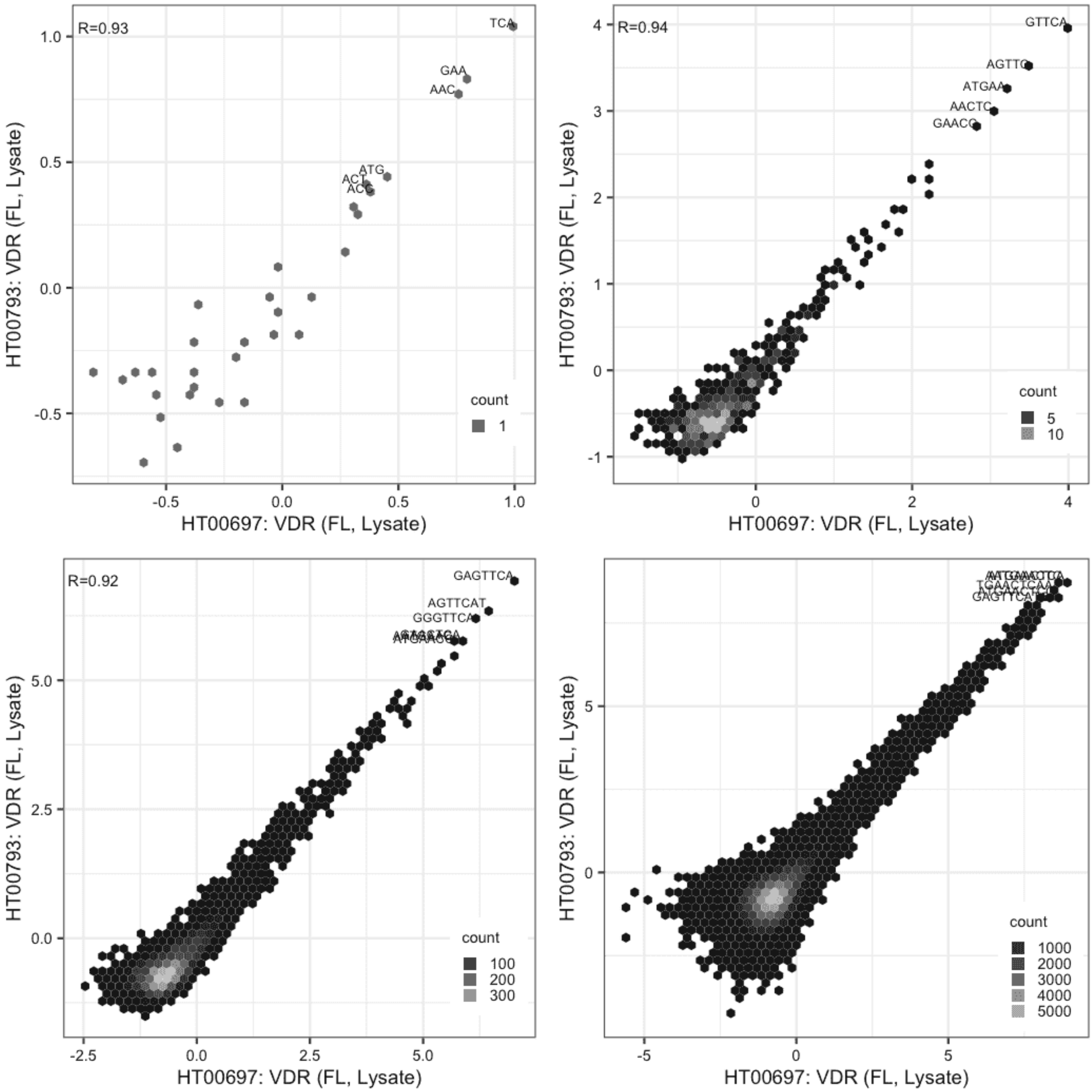

ZBED2

Lysate vs GFPIVT, same construct

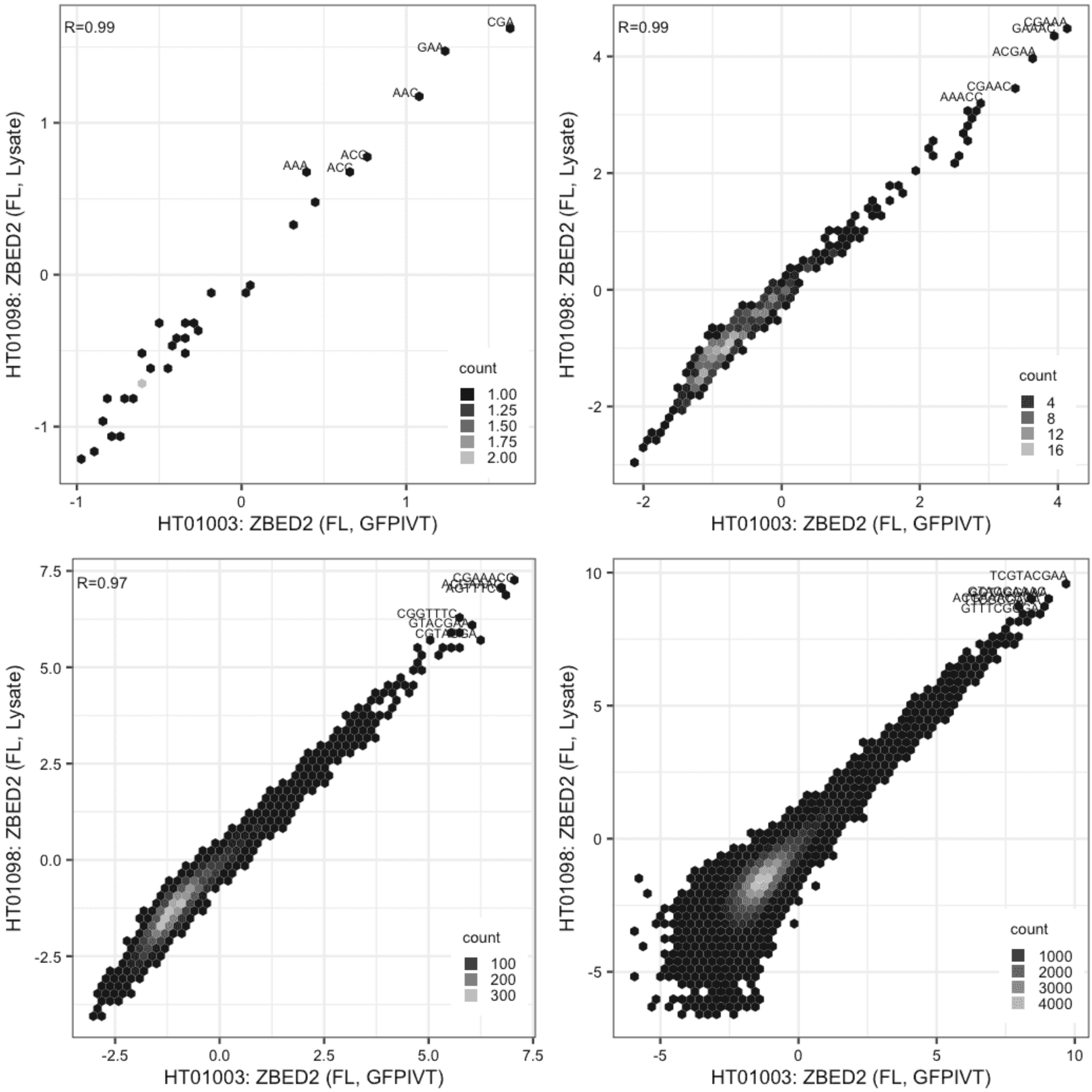

ZBED2

Lysate vs GFPIVT, same construct

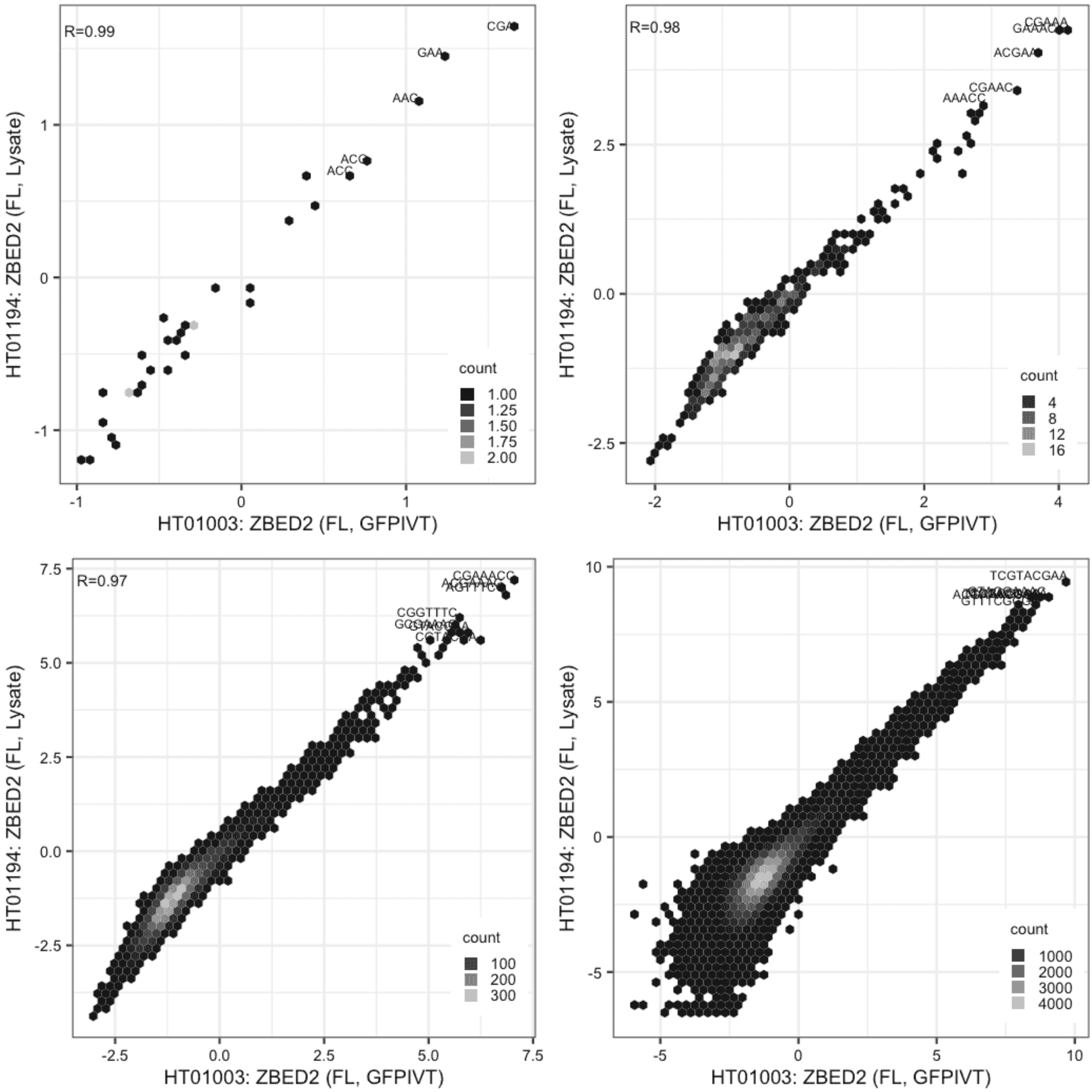

ZBED2

Lysate vs Lysate, same construct

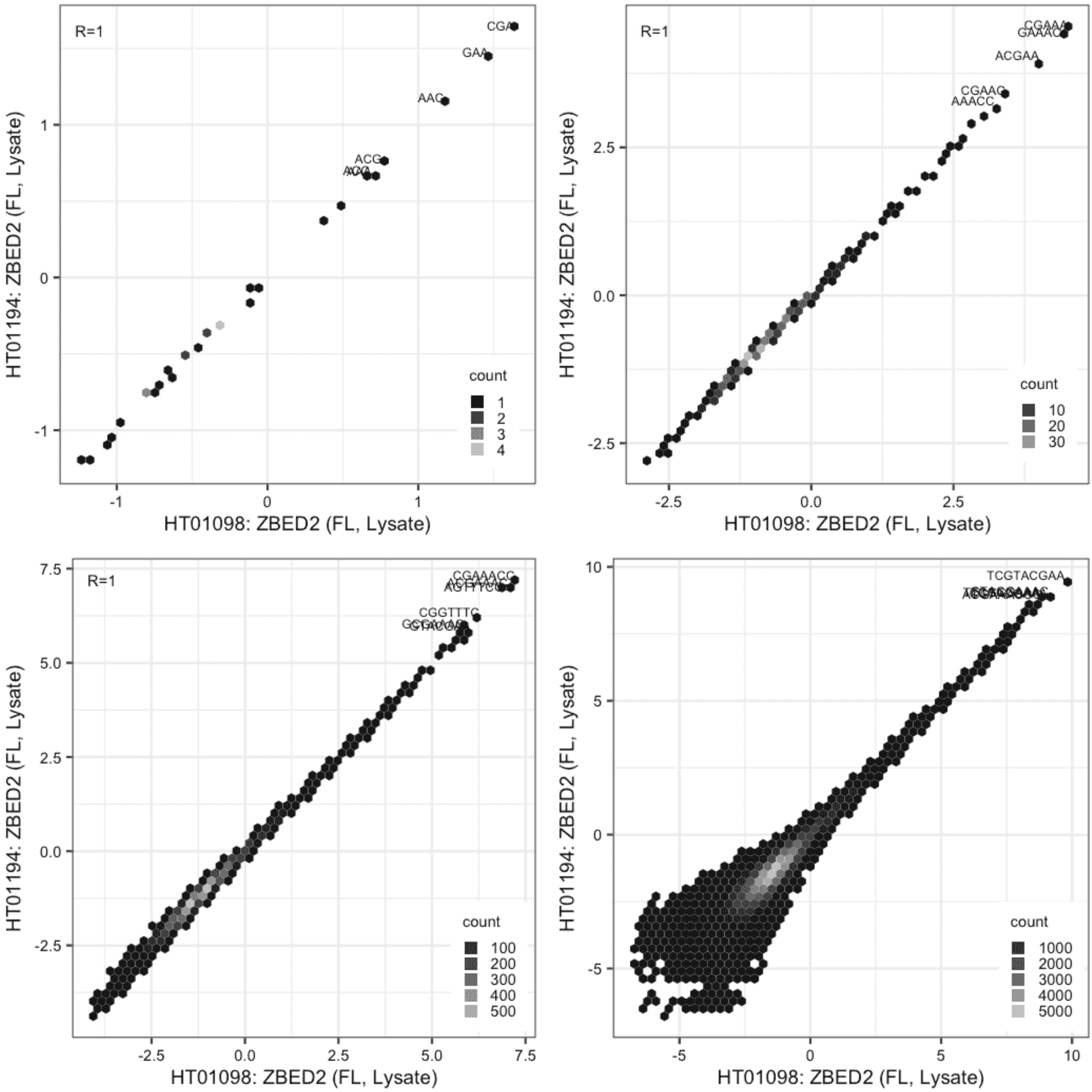

ZBED5

IVT vs GFPIVT, same construct

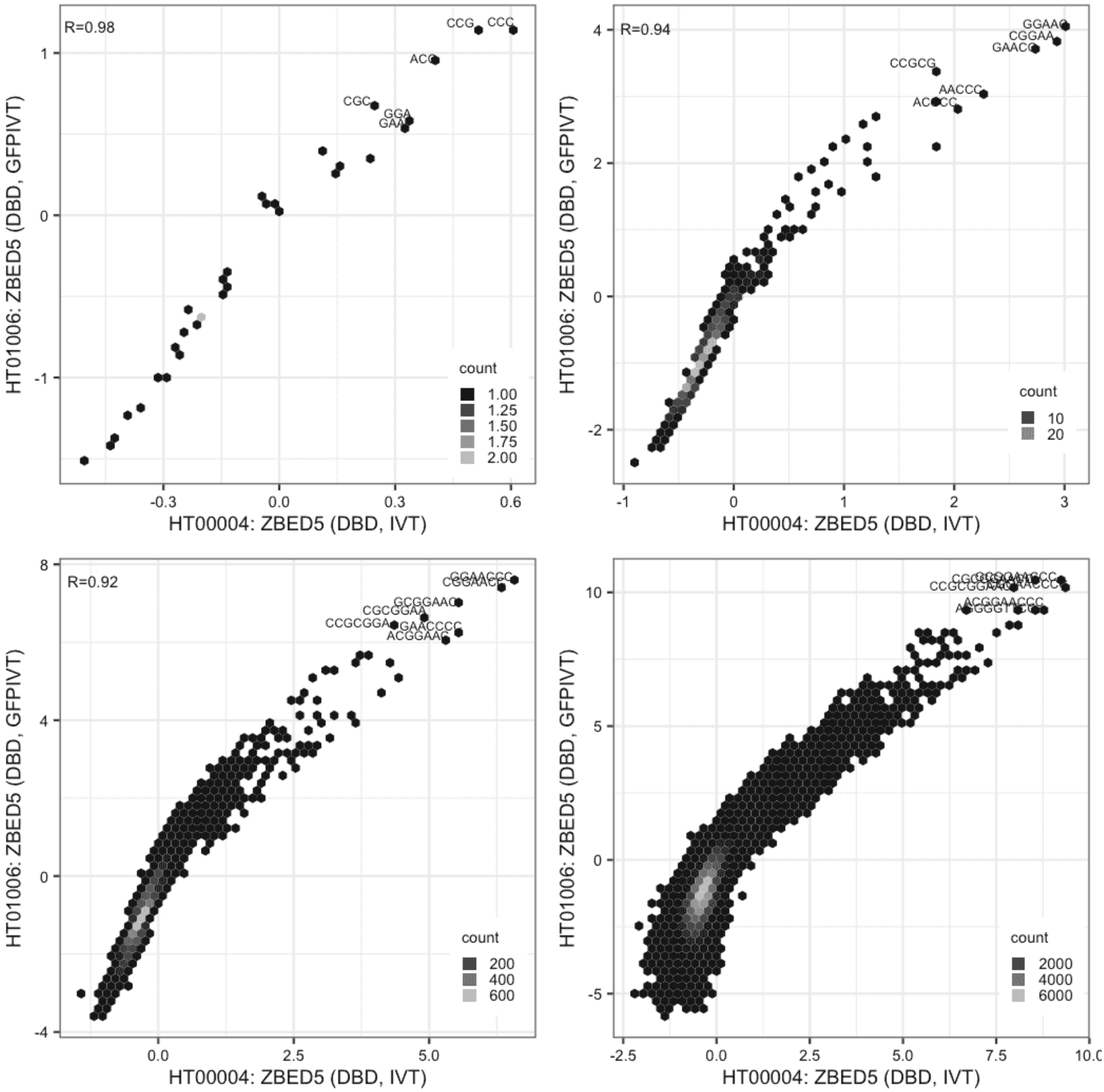

ZBED5

Lysate vs IVT, different constructs

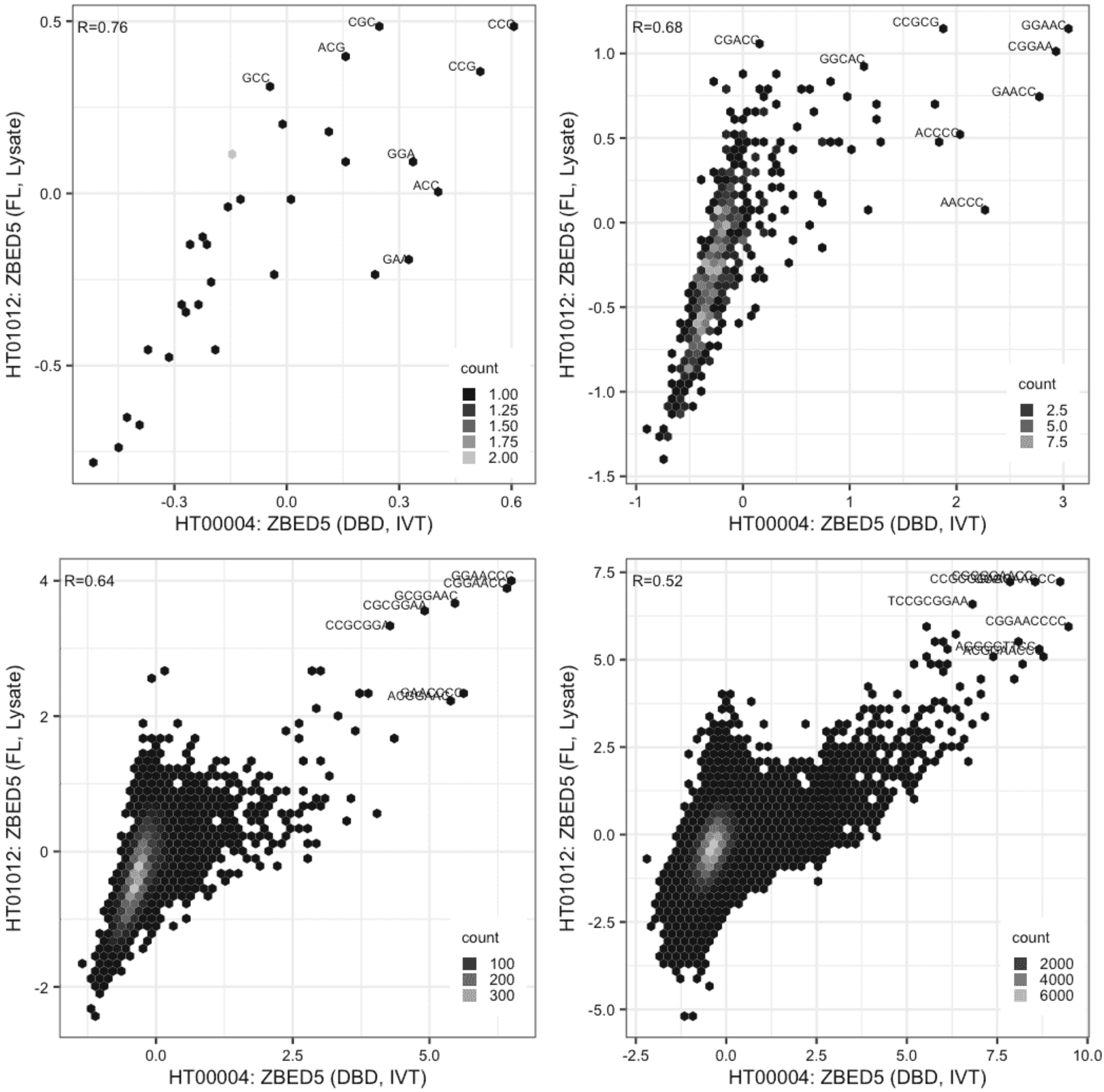

ZBED5

Lysate vs IVT, different constructs

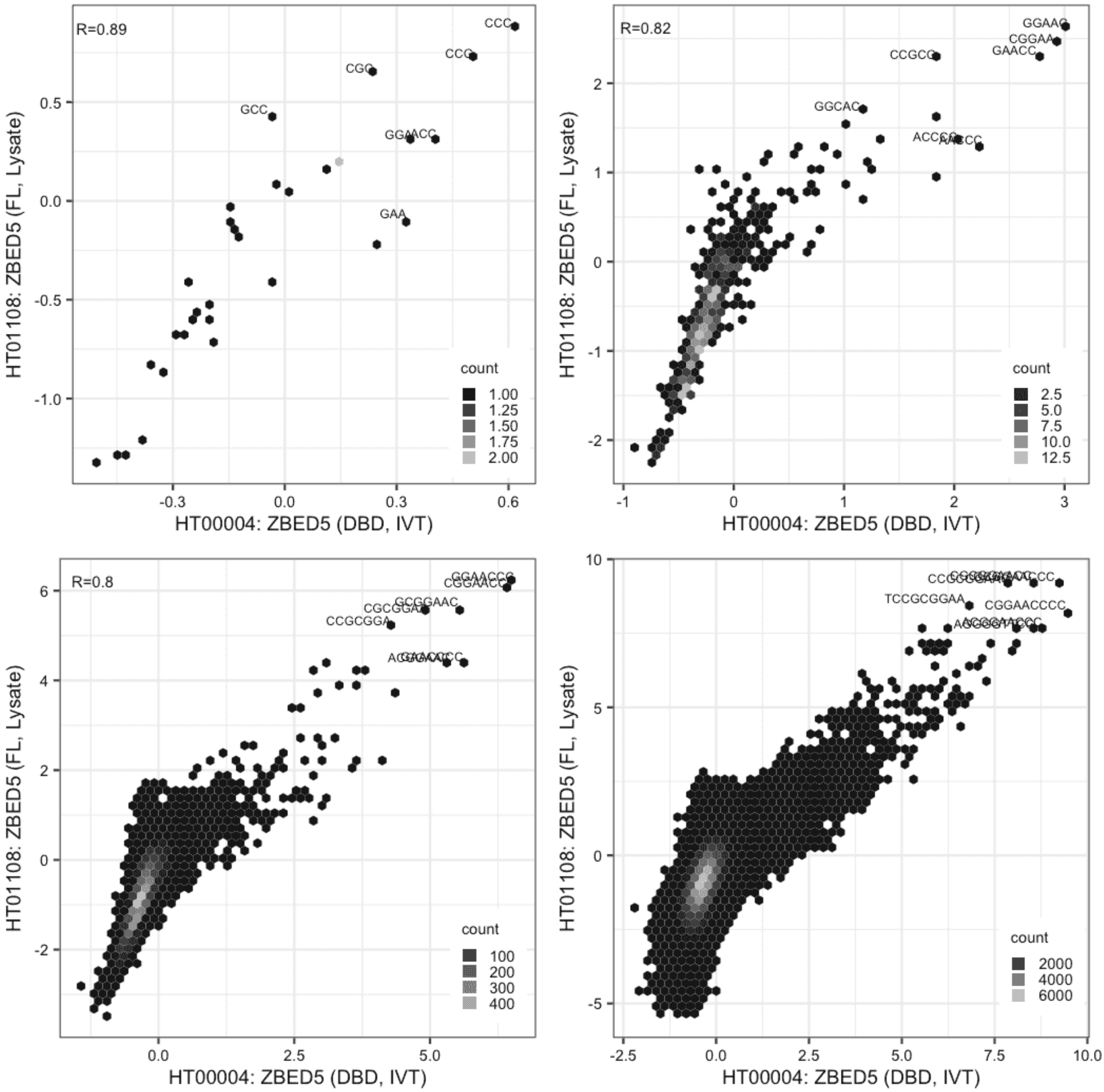

ZBED5

IVT vs GFPIVT, different constructs

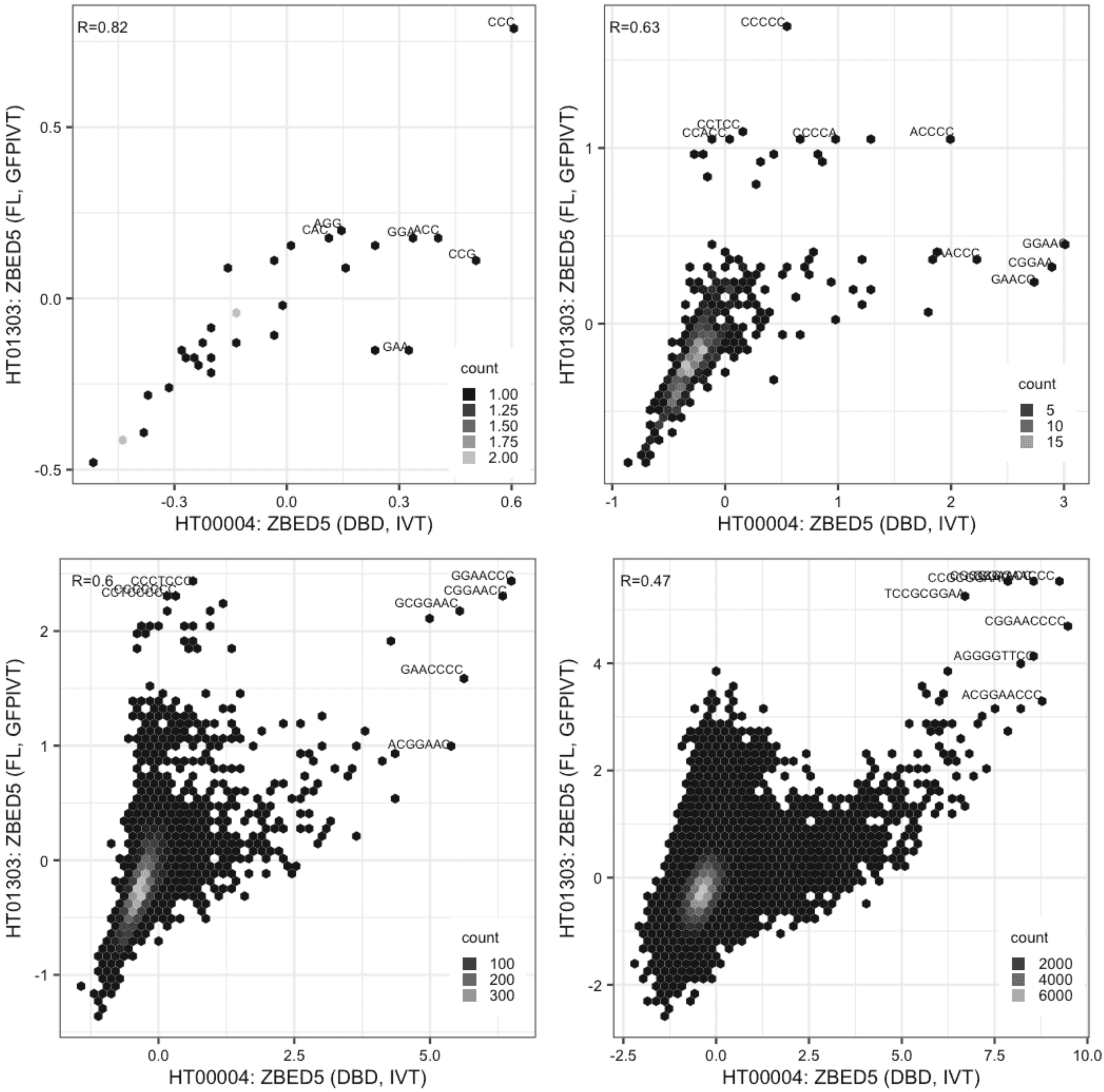

ZBED5

Lysate vs GFPIVT, different constructs

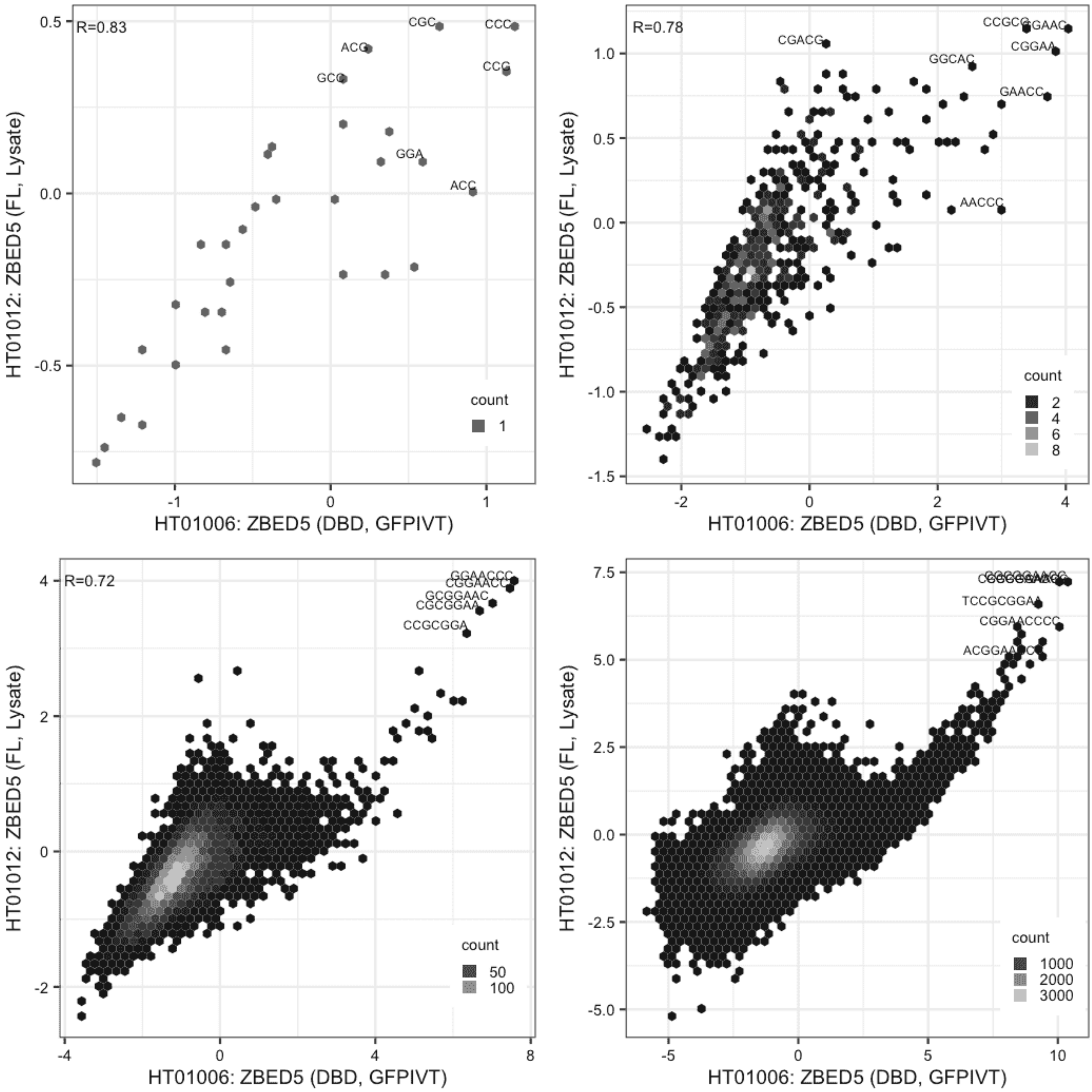

ZBED5

Lysate vs GFPIVT, different constructs

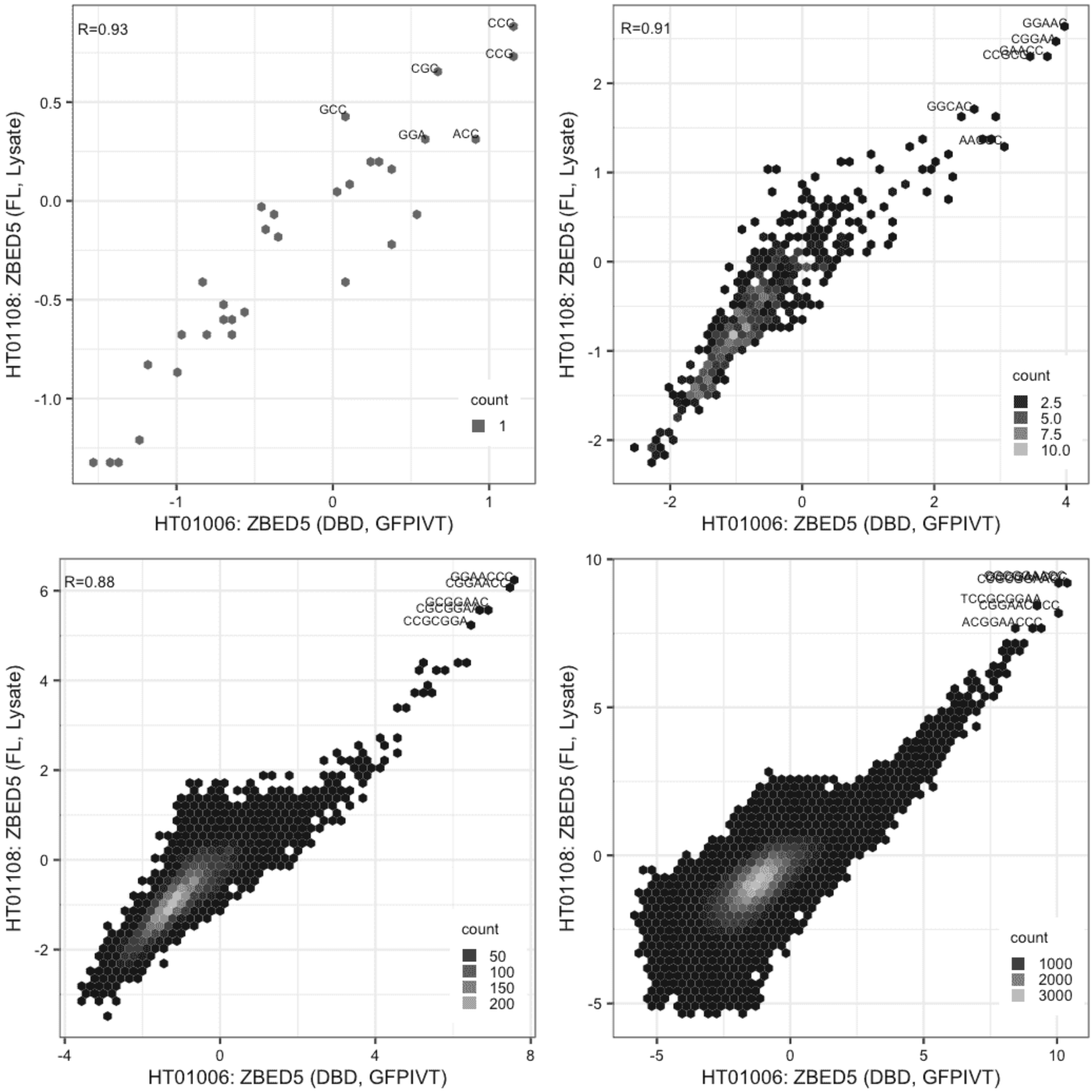

ZBED5

GFPIVT vs GFPIVT, different constructs

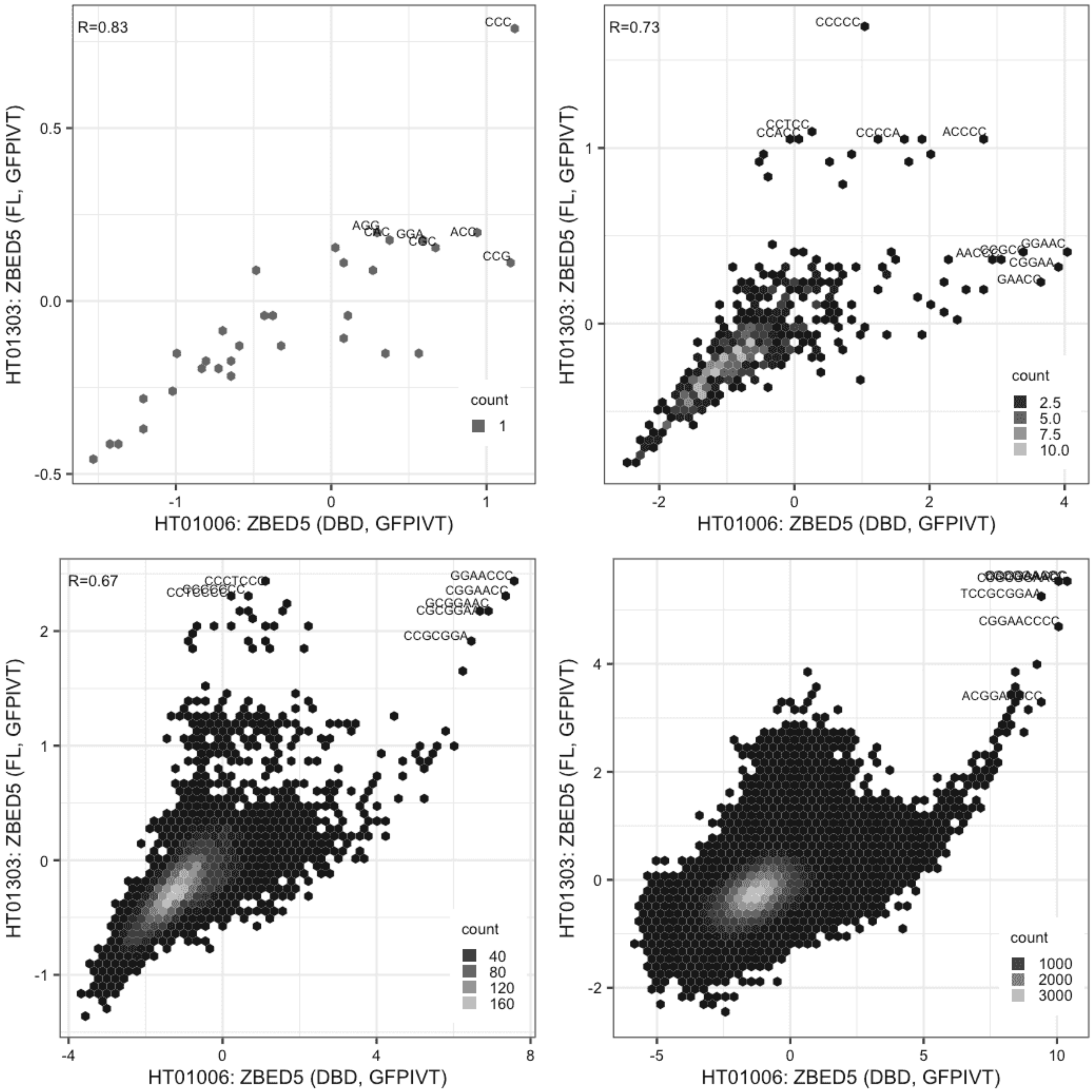

ZBED5

Lysate vs Lysate, same construct

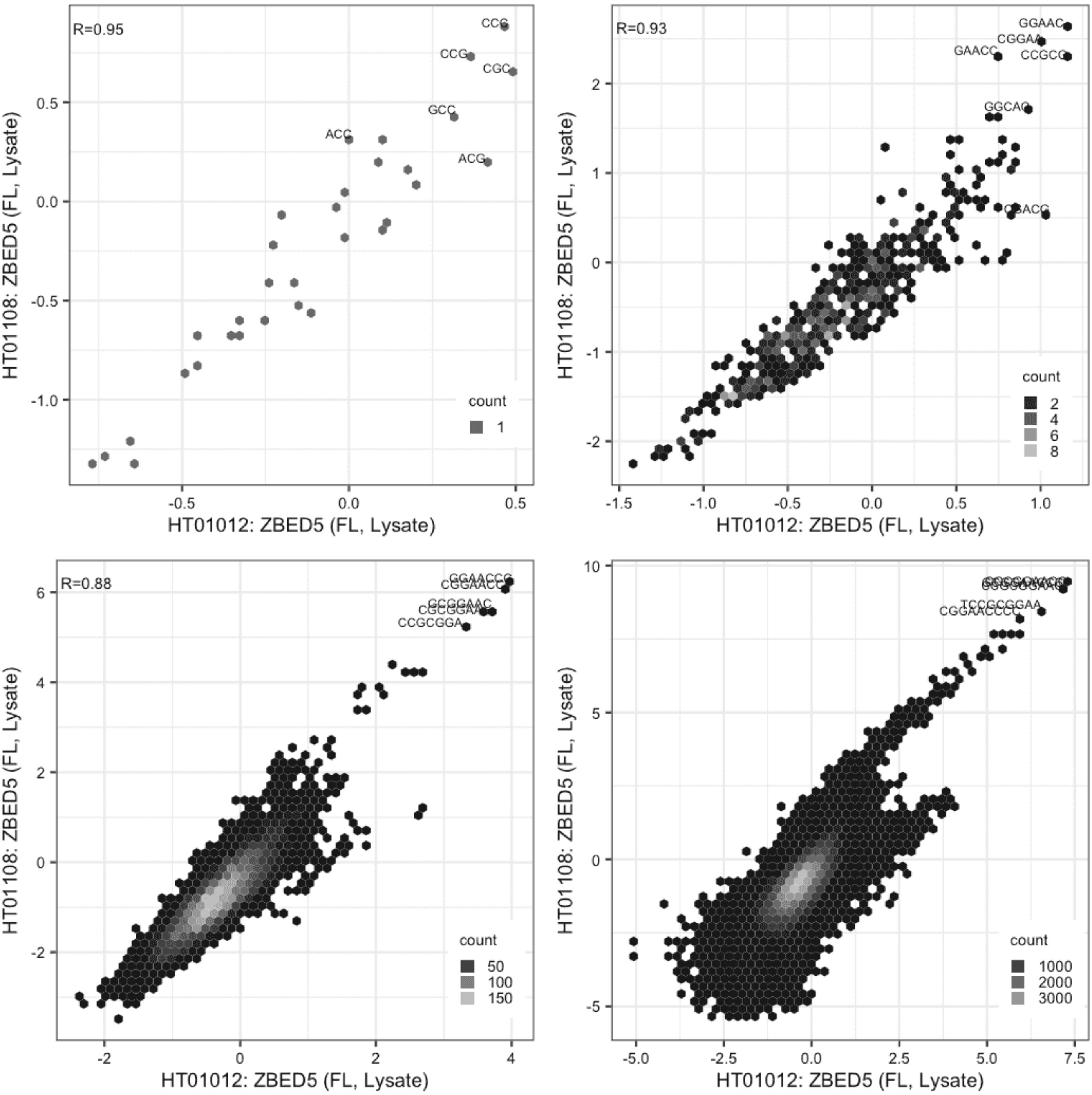

ZBED5

Lysate vs GFPIVT, same construct

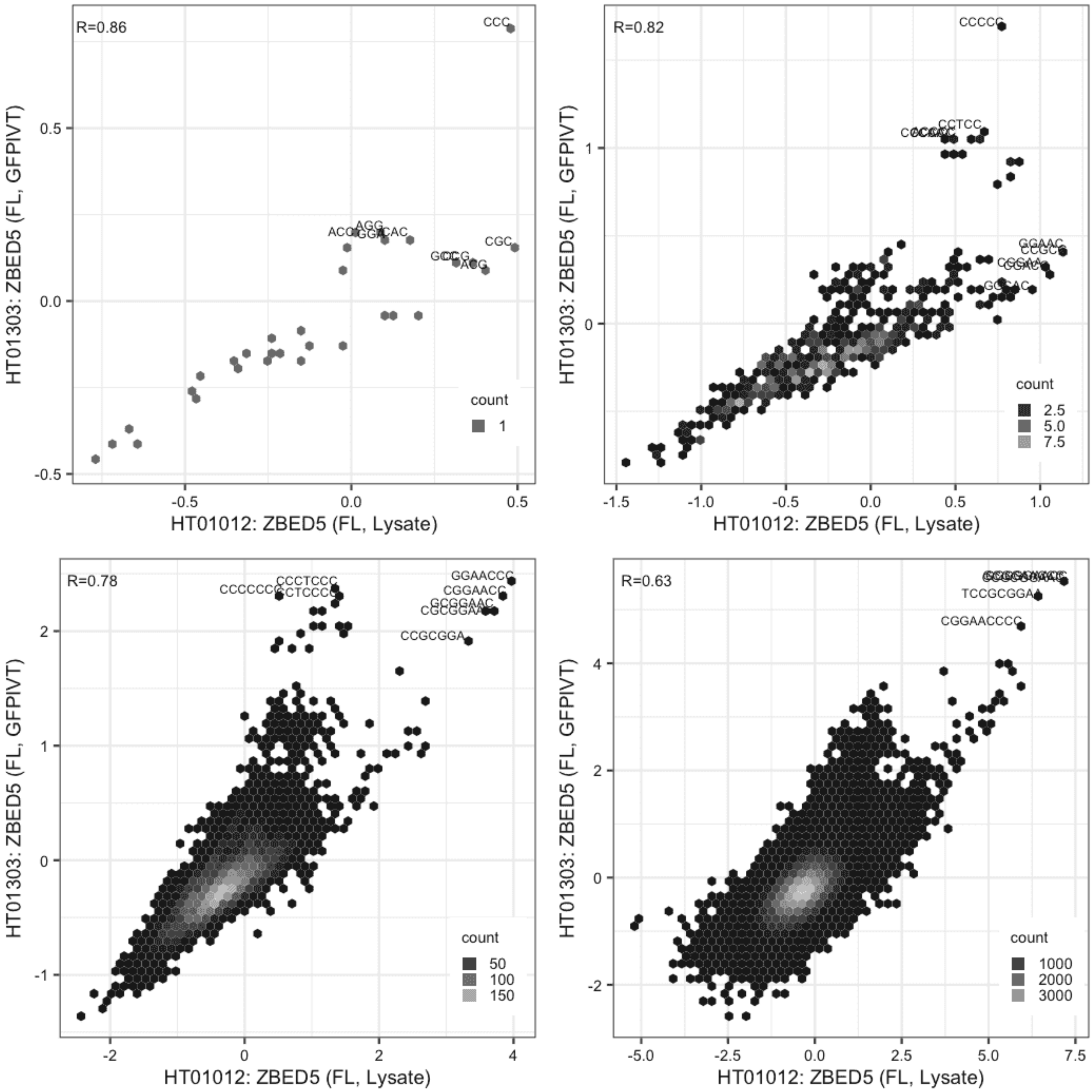

ZBED5

Lysate vs GFPIVT, same construct

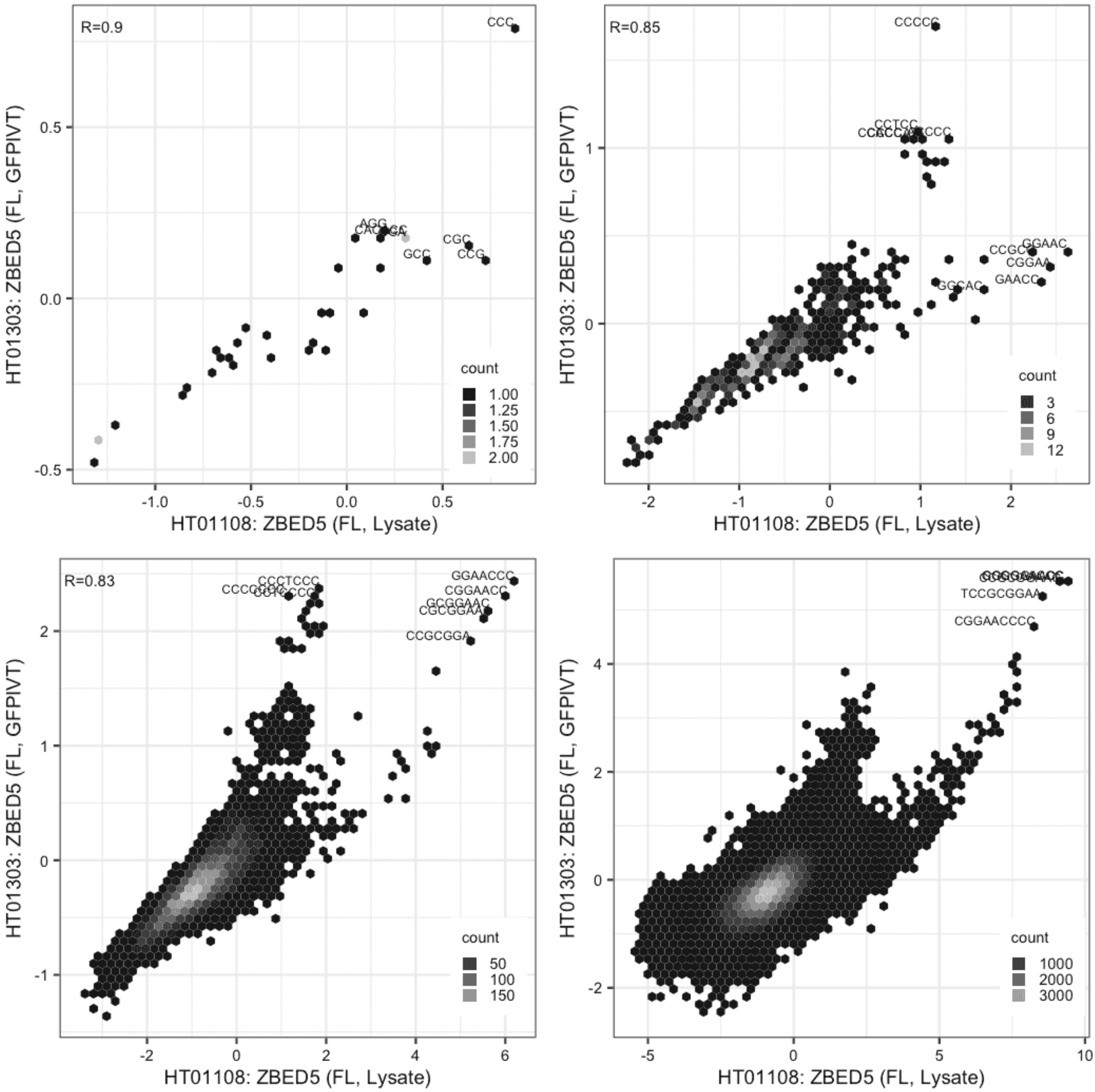

ZBTB47

Lysate vs Lysate, same construct

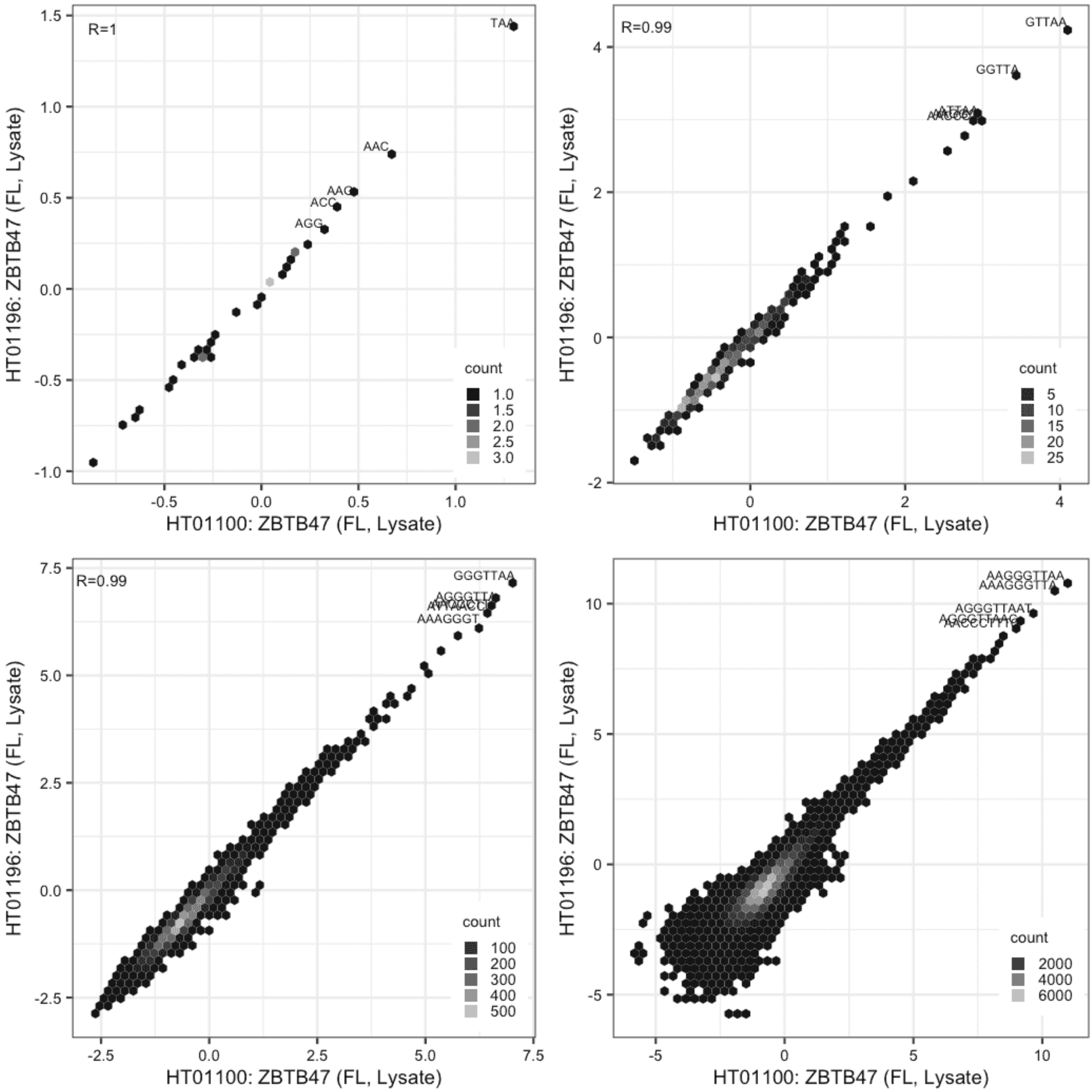

ZBTB47

Lysate vs GFPIVT, same construct

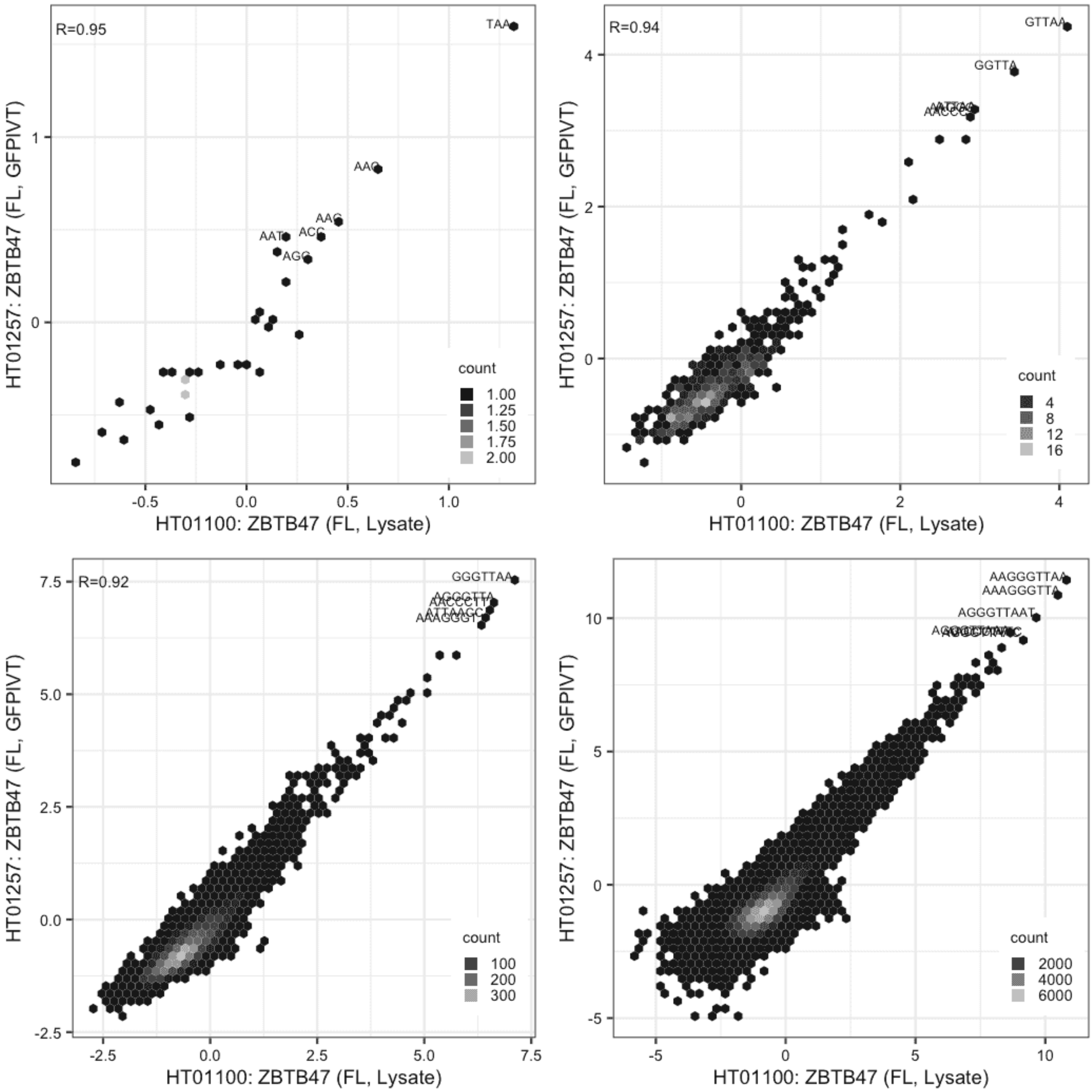

ZBTB47

Lysate vs GFPIVT, different constructs

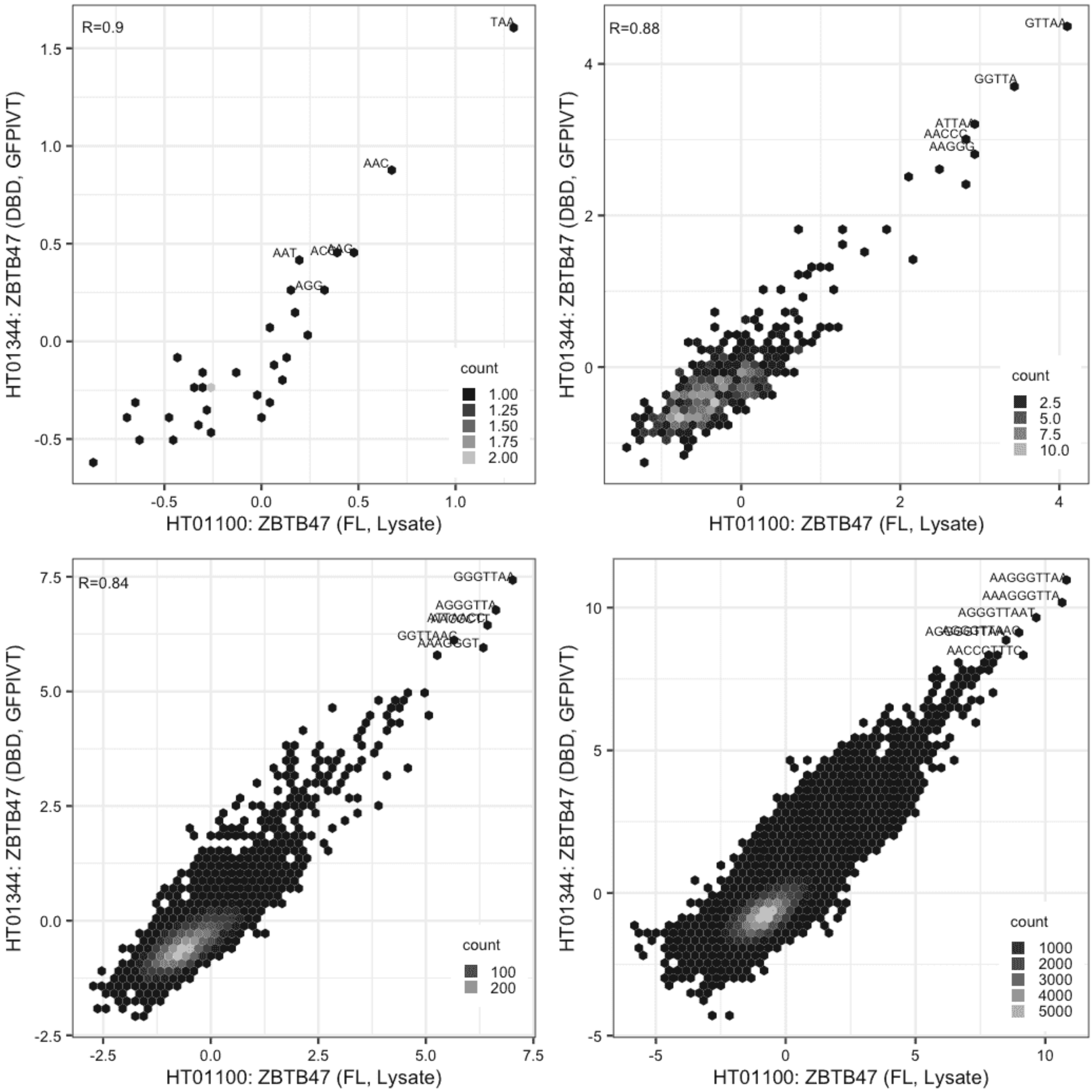

ZBTB47

Lysate vs GFPIVT, same construct

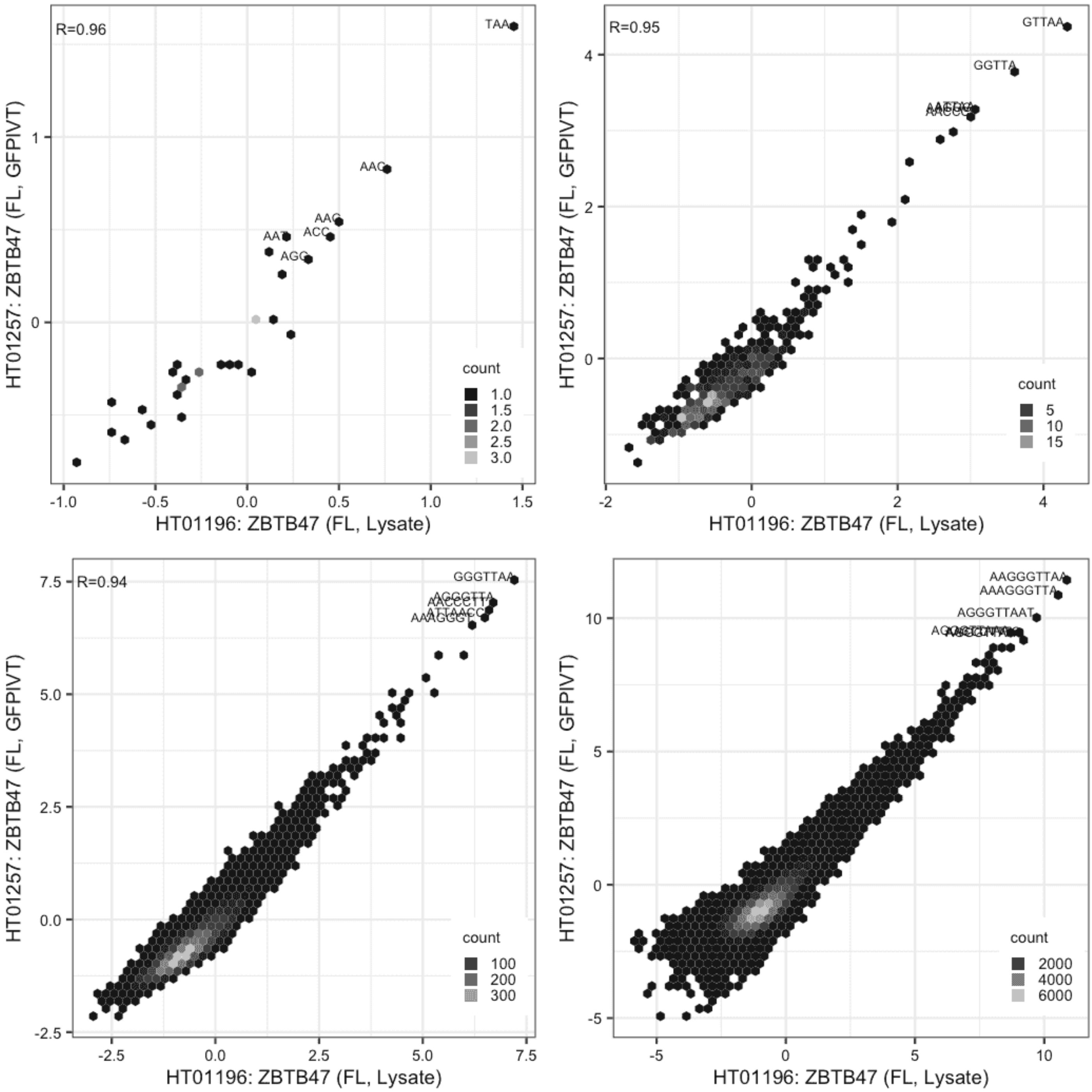

ZBTB47

Lysate vs GFPIVT, different constructs

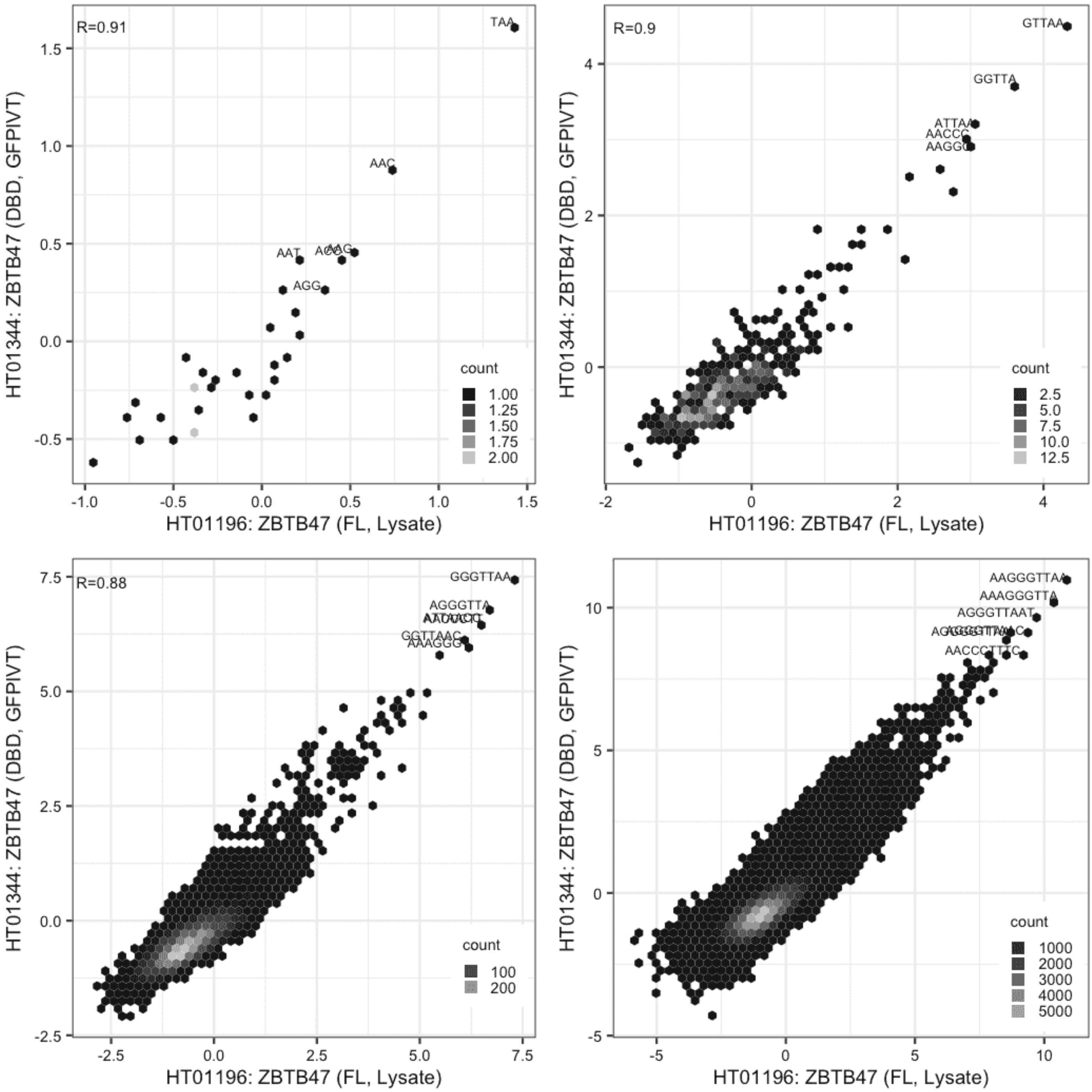

ZBTB47

GFPIVT vs GFPIVT, different constructs

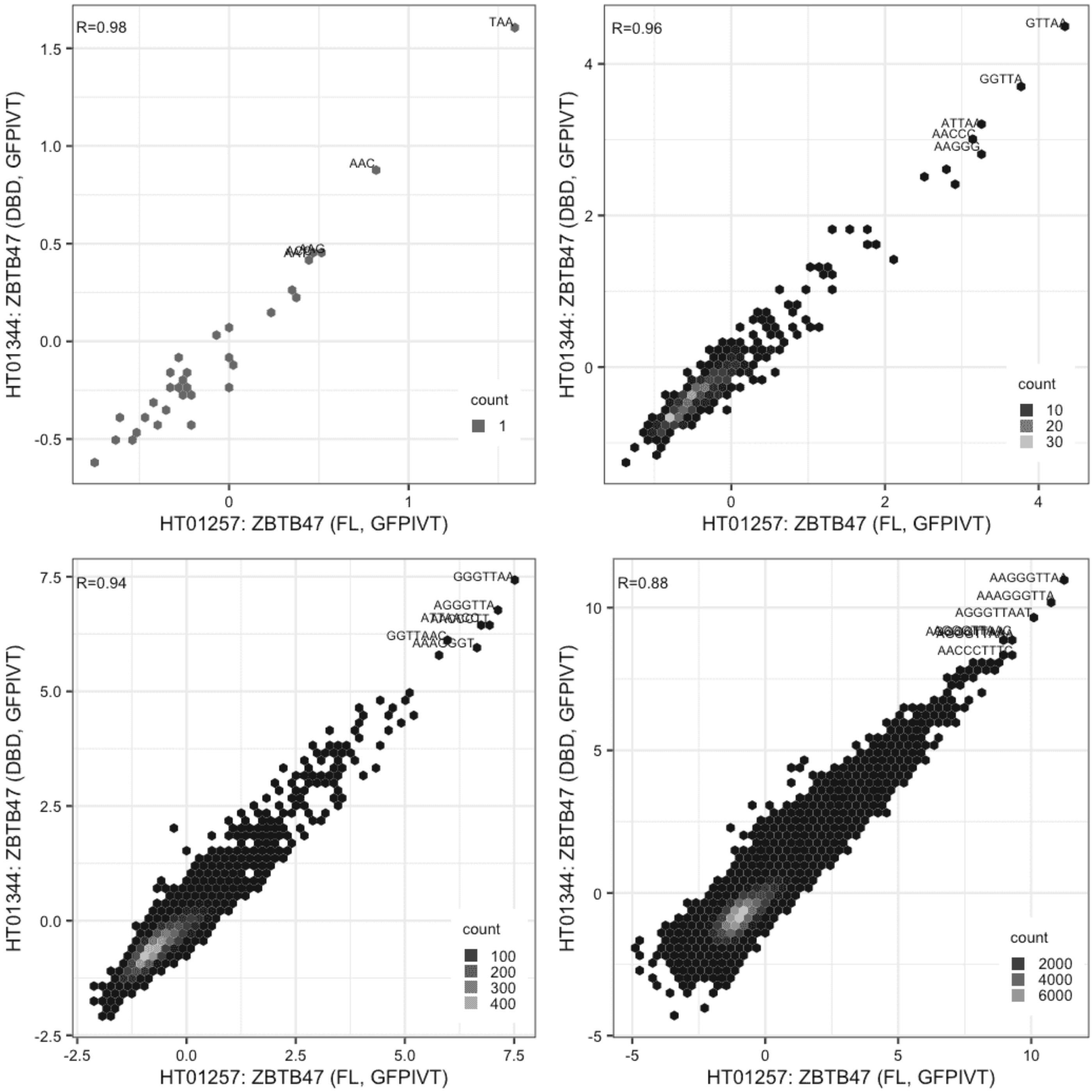

ZBTB8A

Lysate vs IVT, different constructs

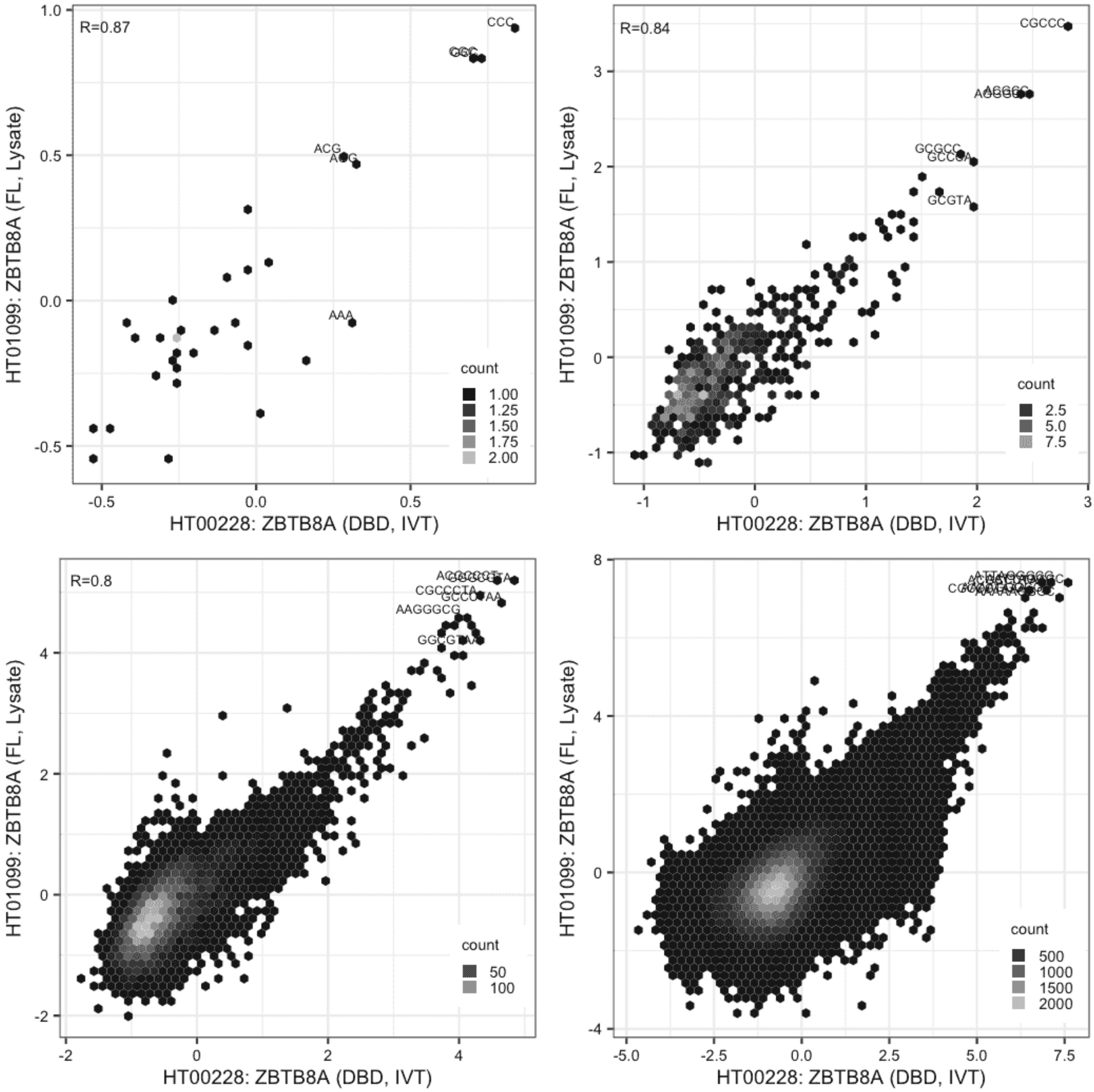

ZBTB8A

Lysate vs IVT, different constructs

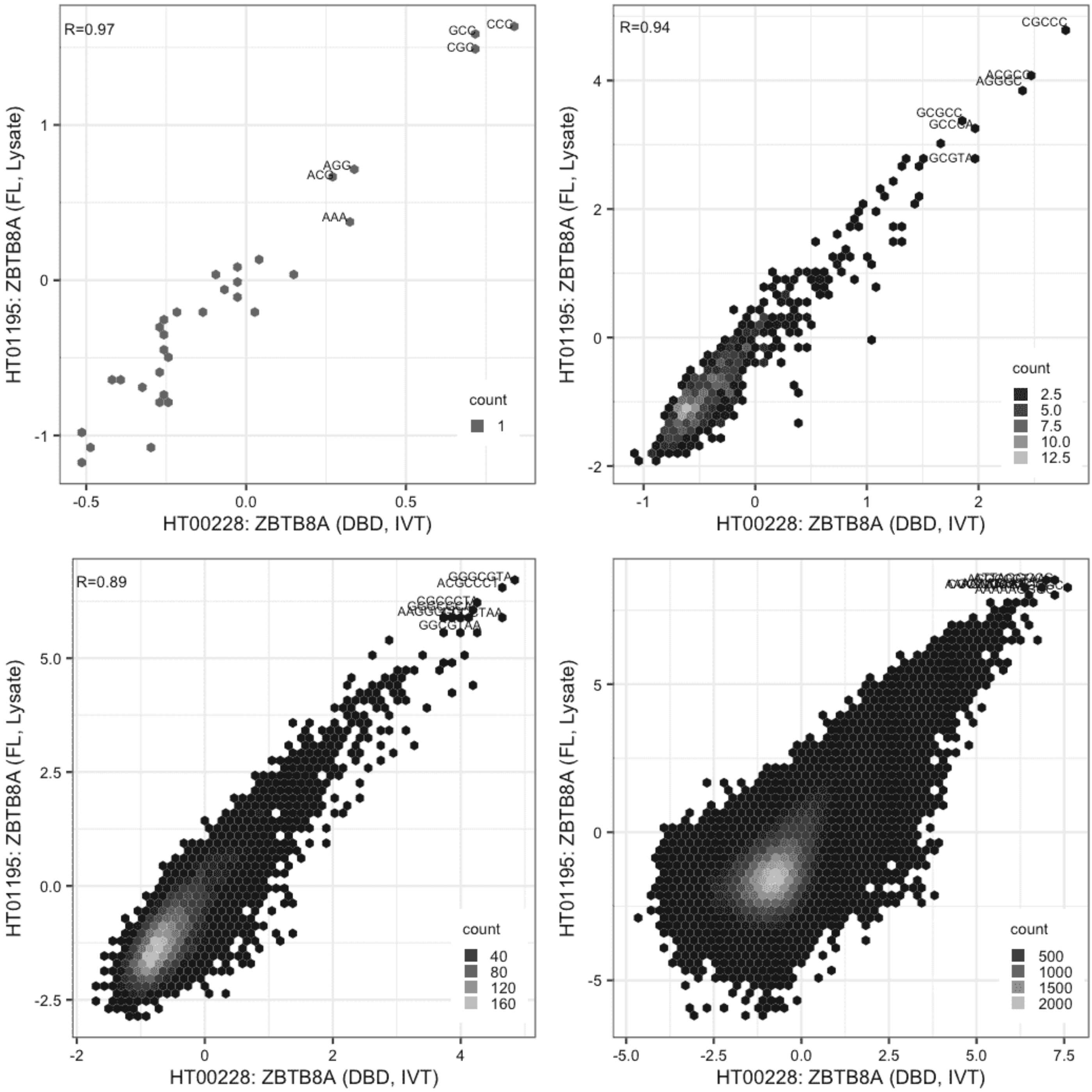

ZBTB8A

Lysate vs Lysate, same construct

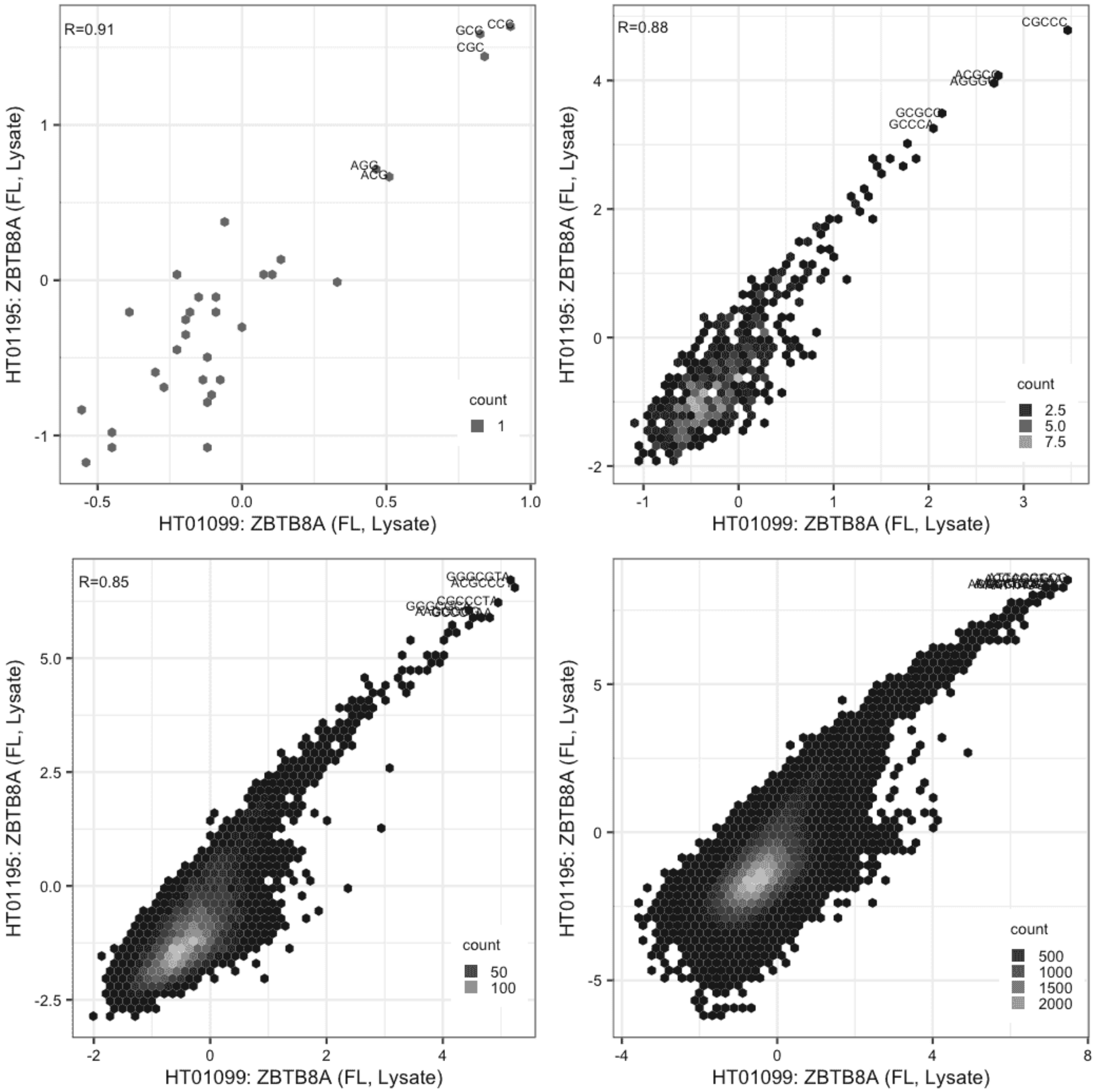

ZBTB8B

Lysate vs IVT, different constructs

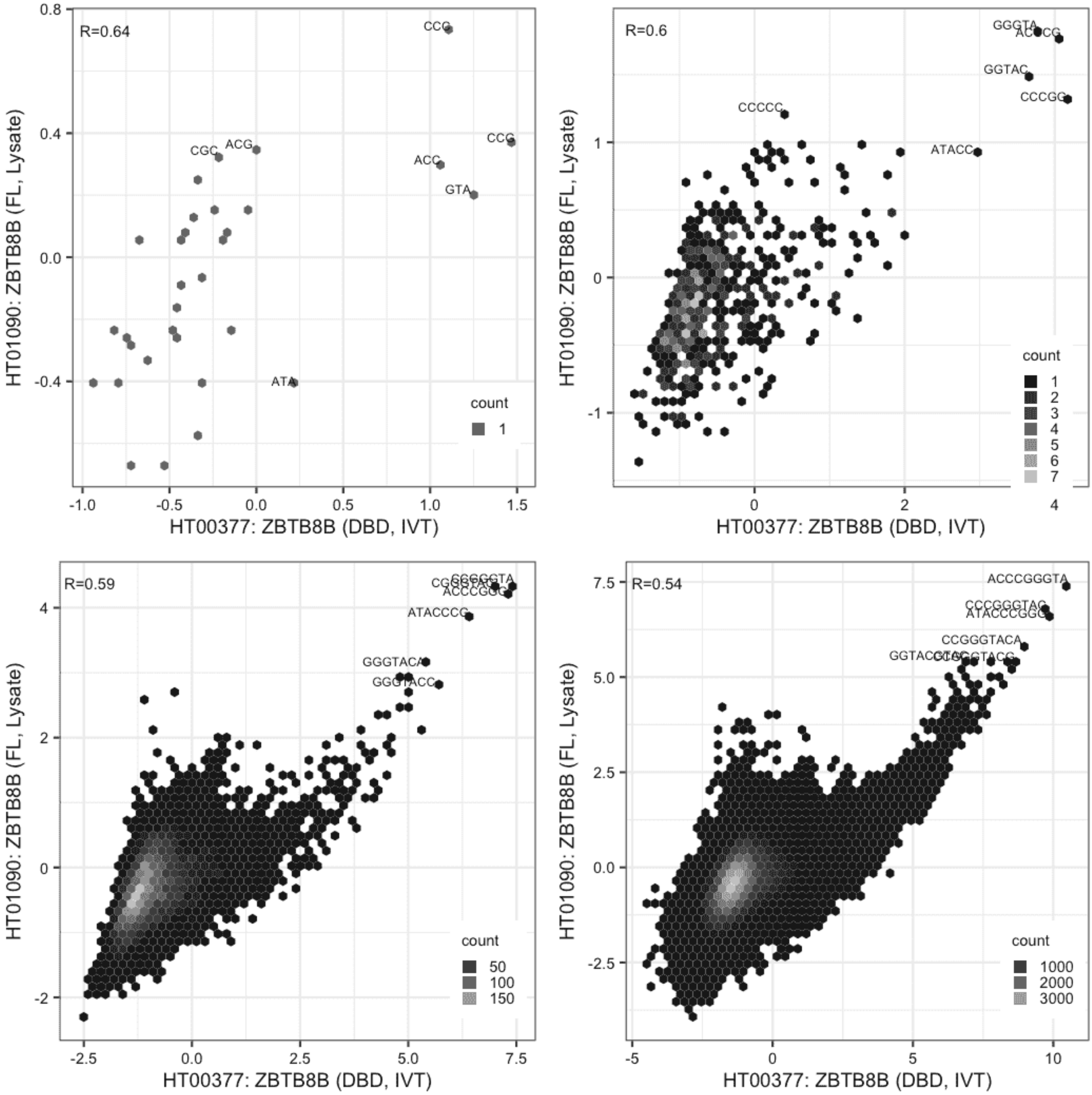

ZBTB8B

Lysate vs IVT, different constructs

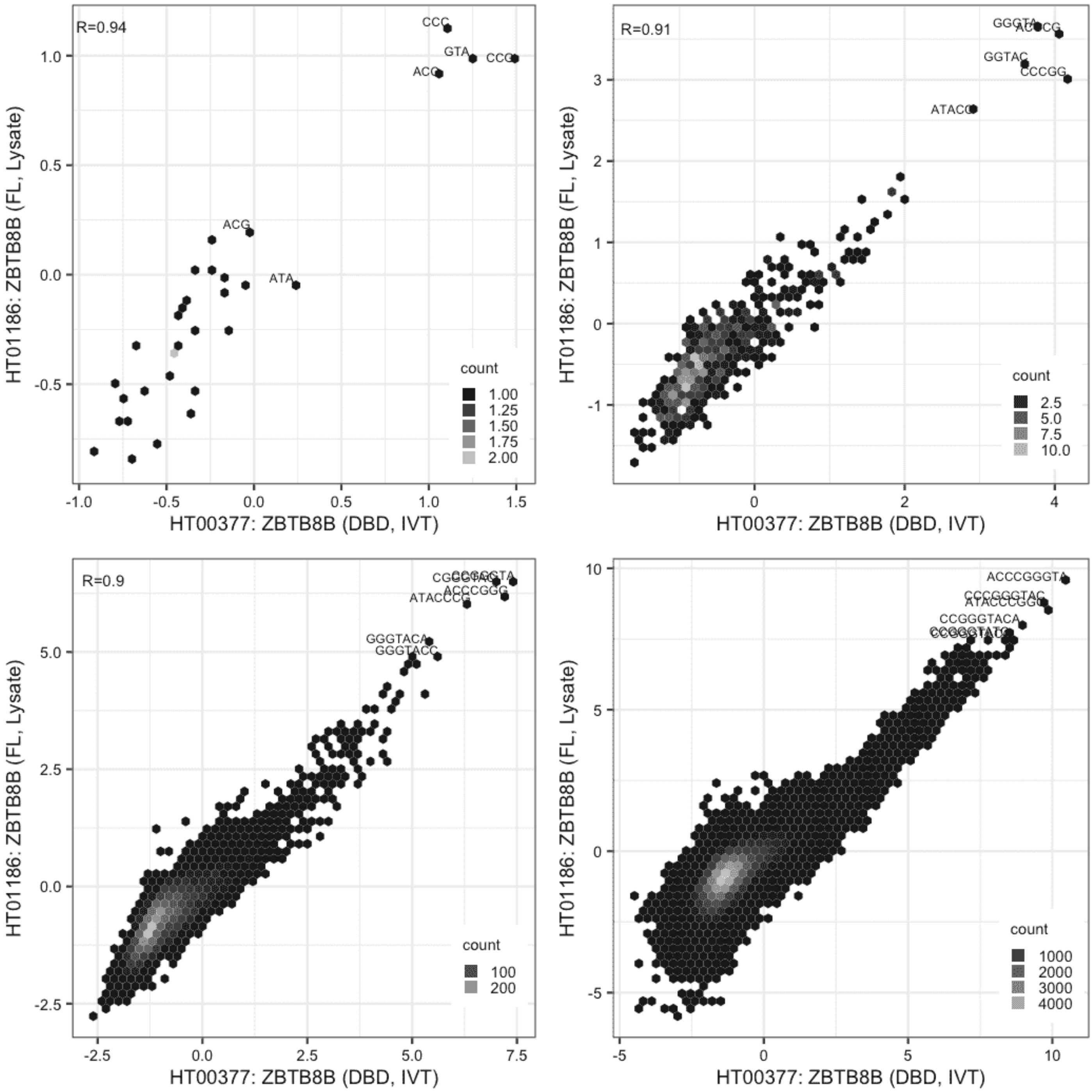

ZBTB8B

Lysate vs Lysate, same construct

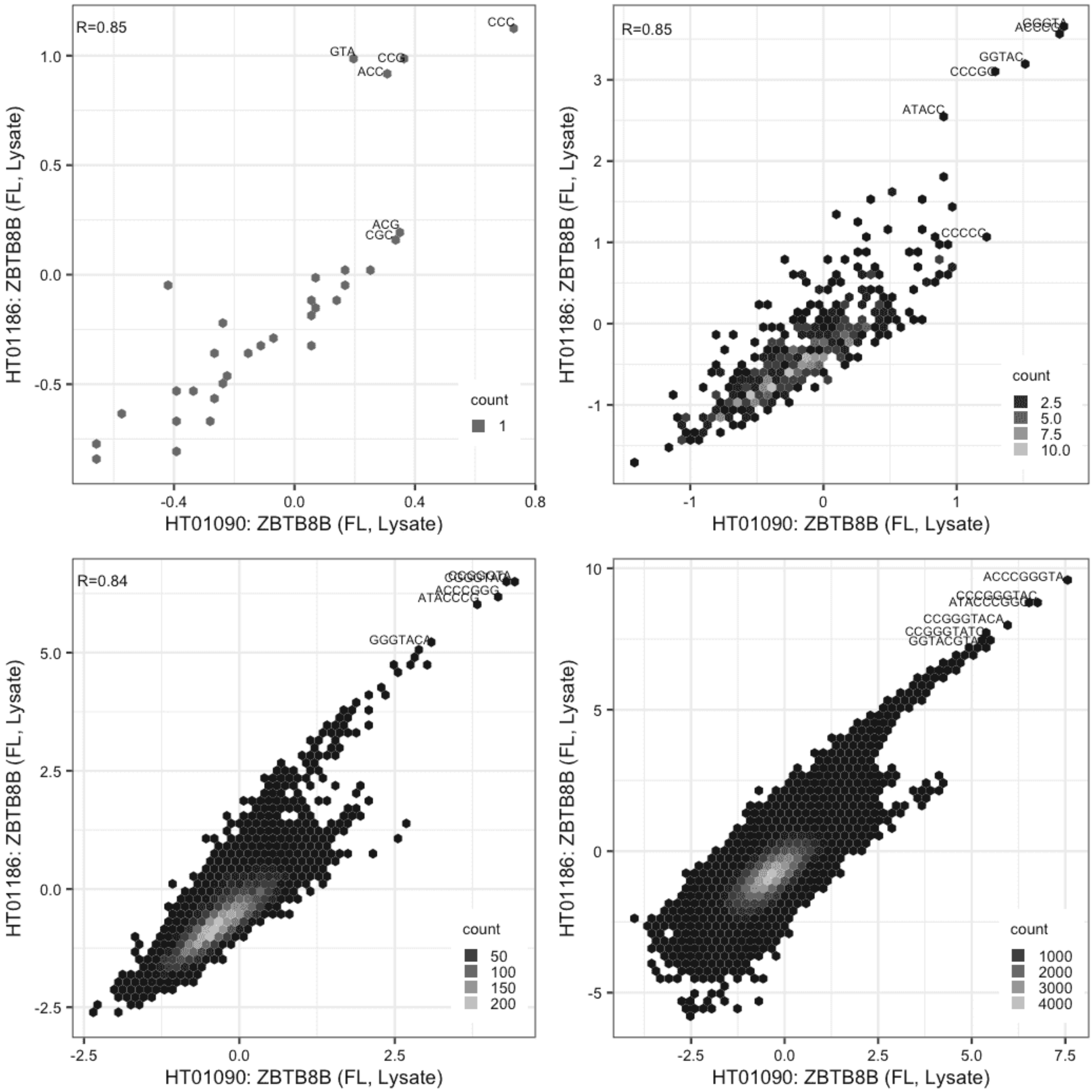

ZFP3

Lysate vs IVT, same construct

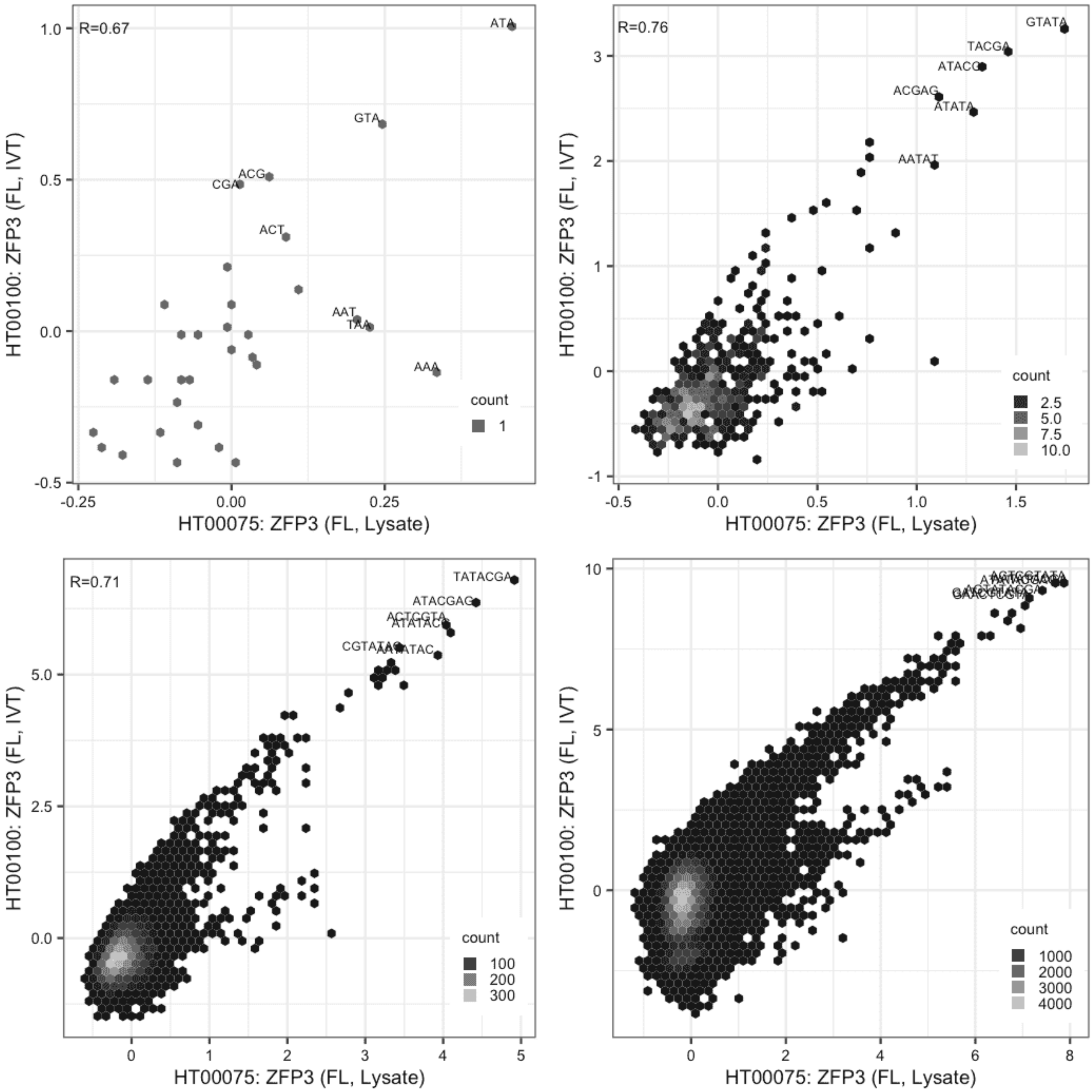

ZGLP1

Lysate vs IVT, different constructs

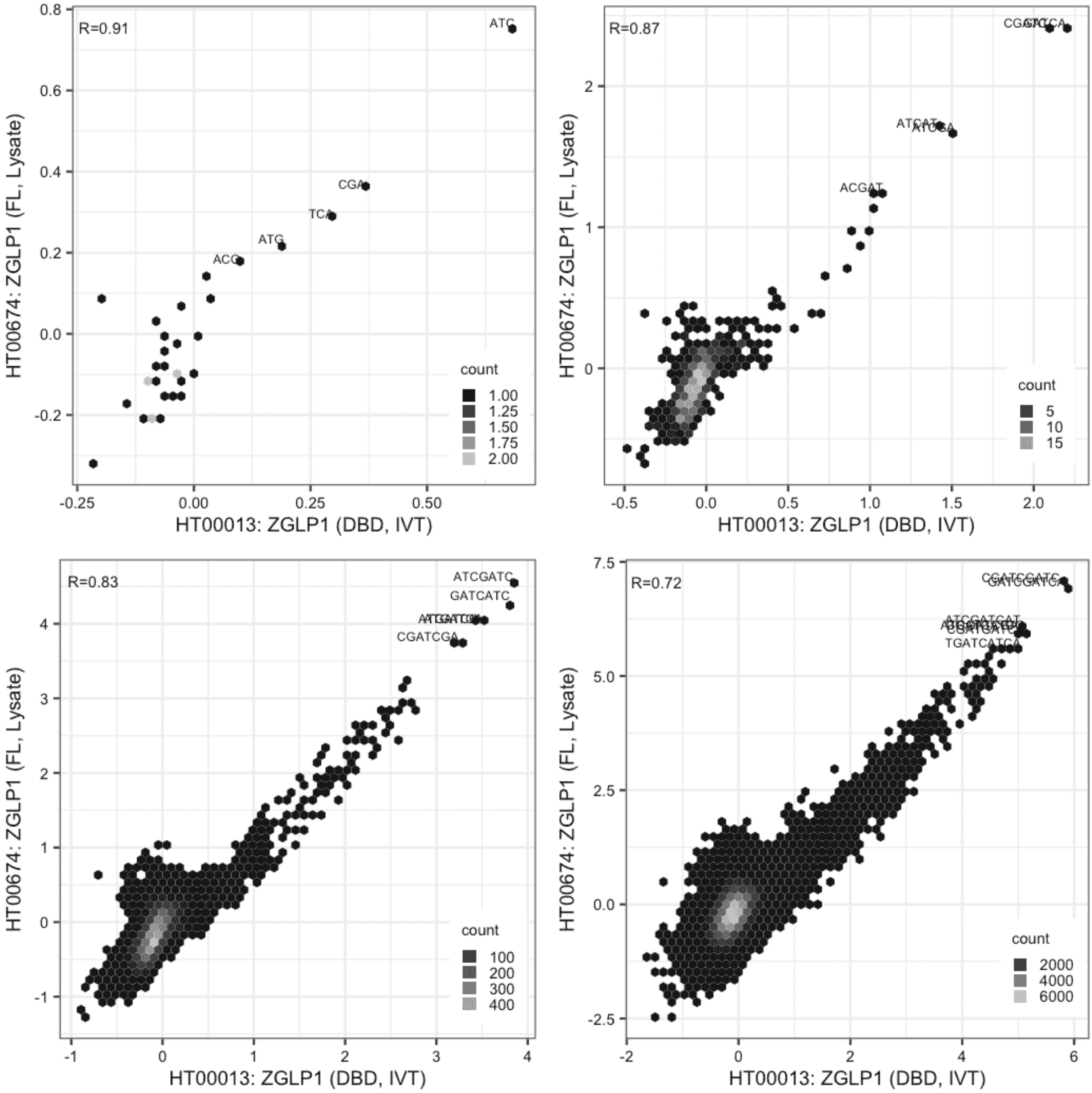

ZGLP1

Lysate vs IVT, different constructs

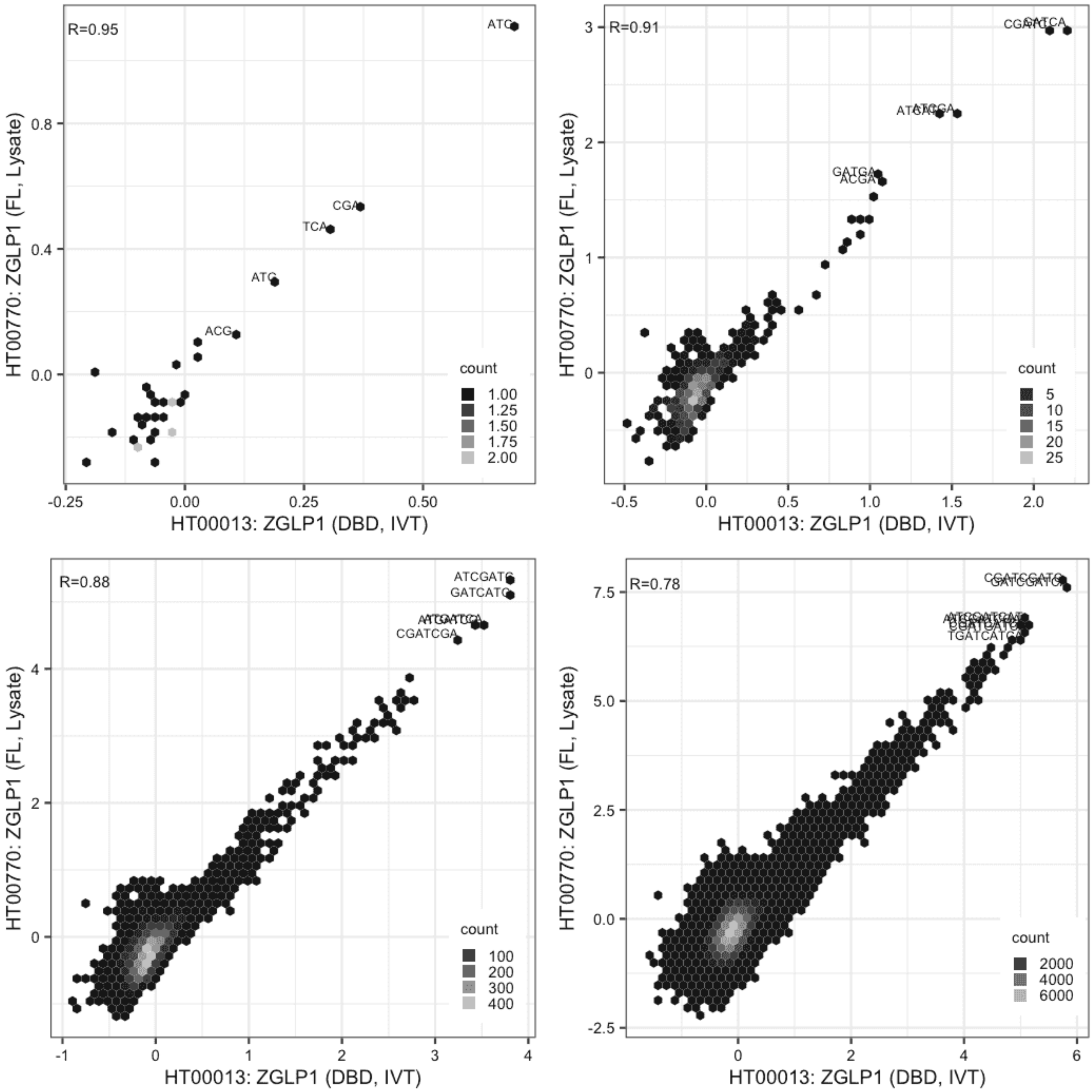

ZGLP1

Lysate vs Lysate, same construct

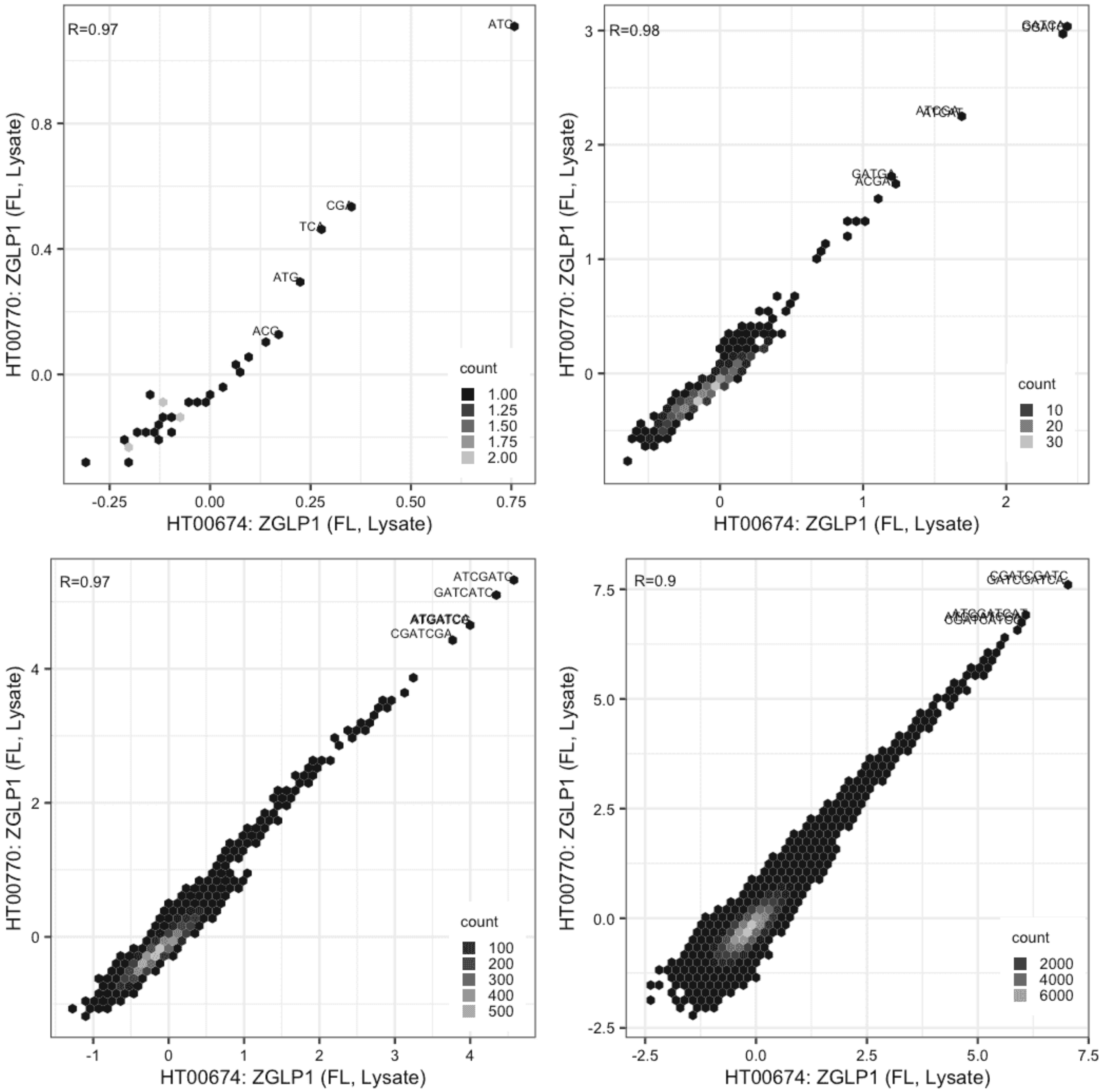

ZNF131

Lysate vs IVT, different constructs

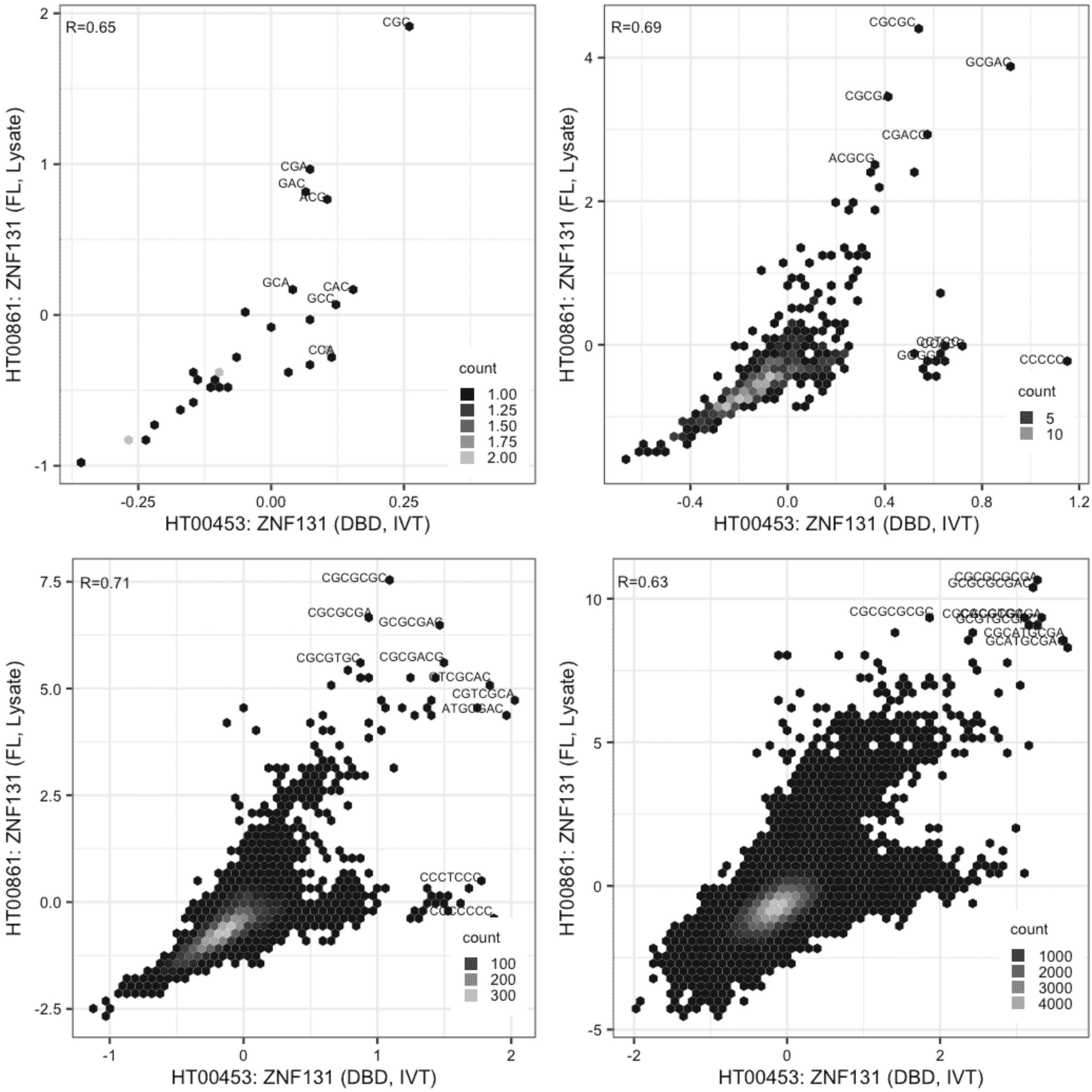

ZNF131

IVT vs GFPIVT, same construct

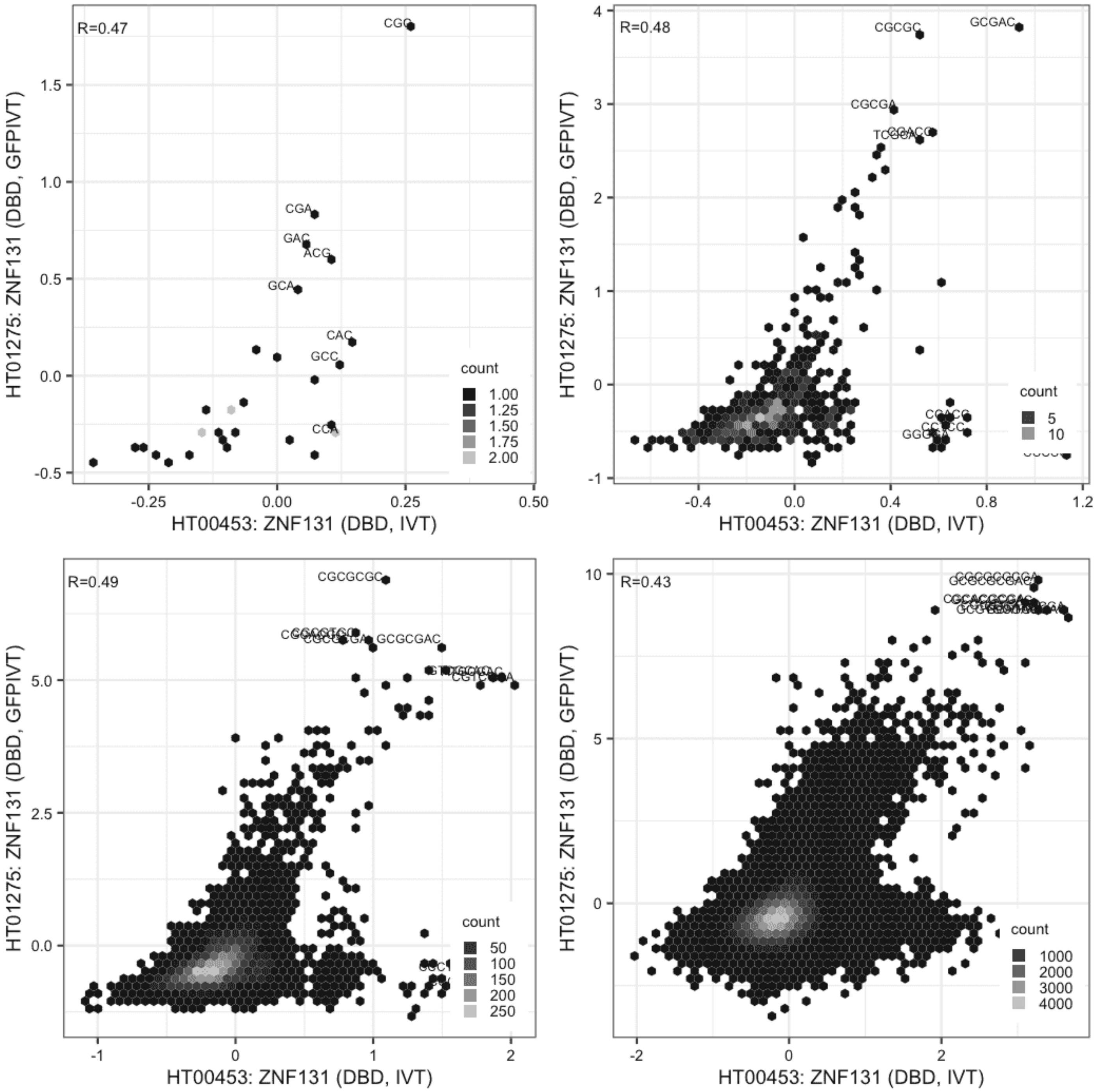

ZNF131

IVT vs GFPIVT, different constructs

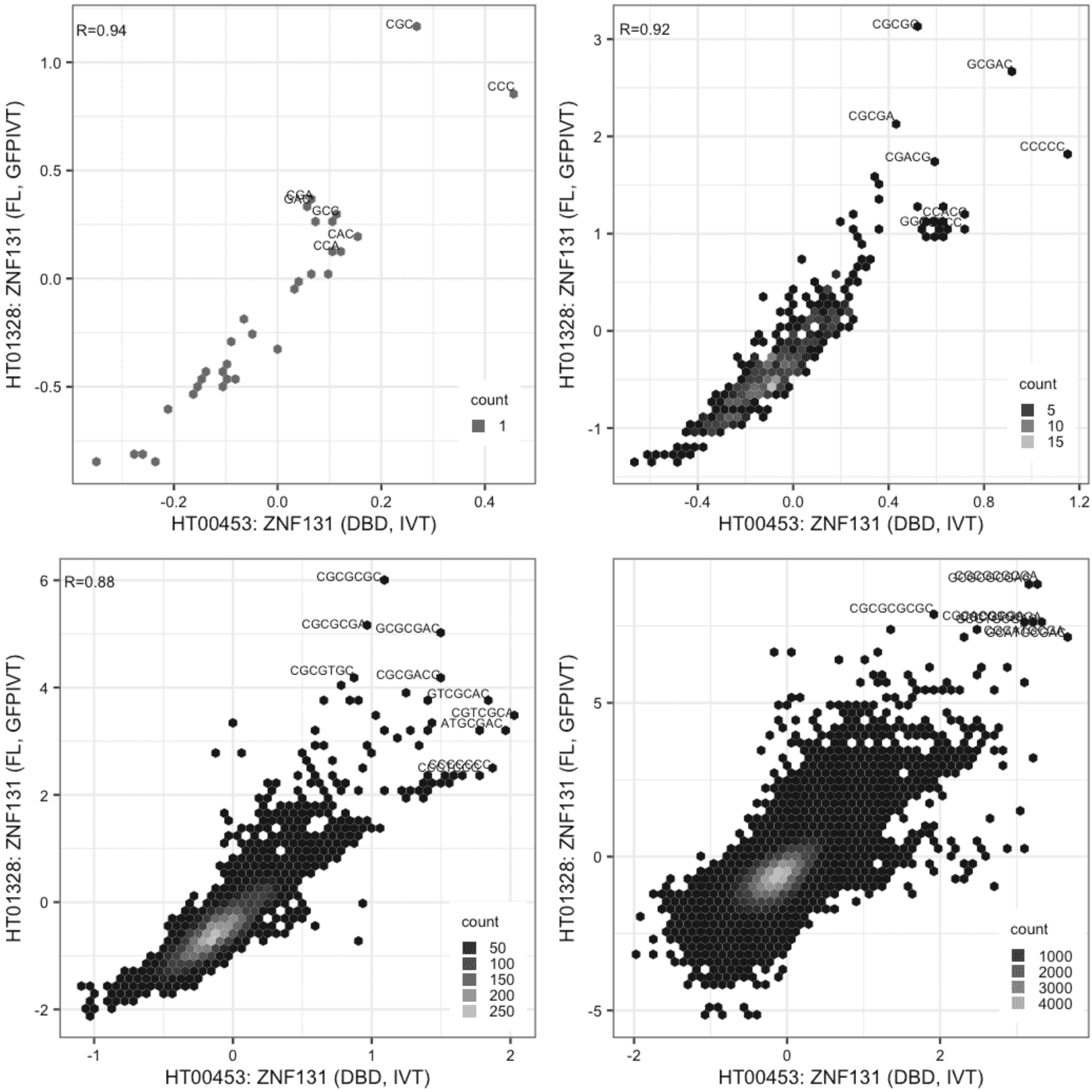

ZNF131

Lysate vs GFPIVT, different constructs

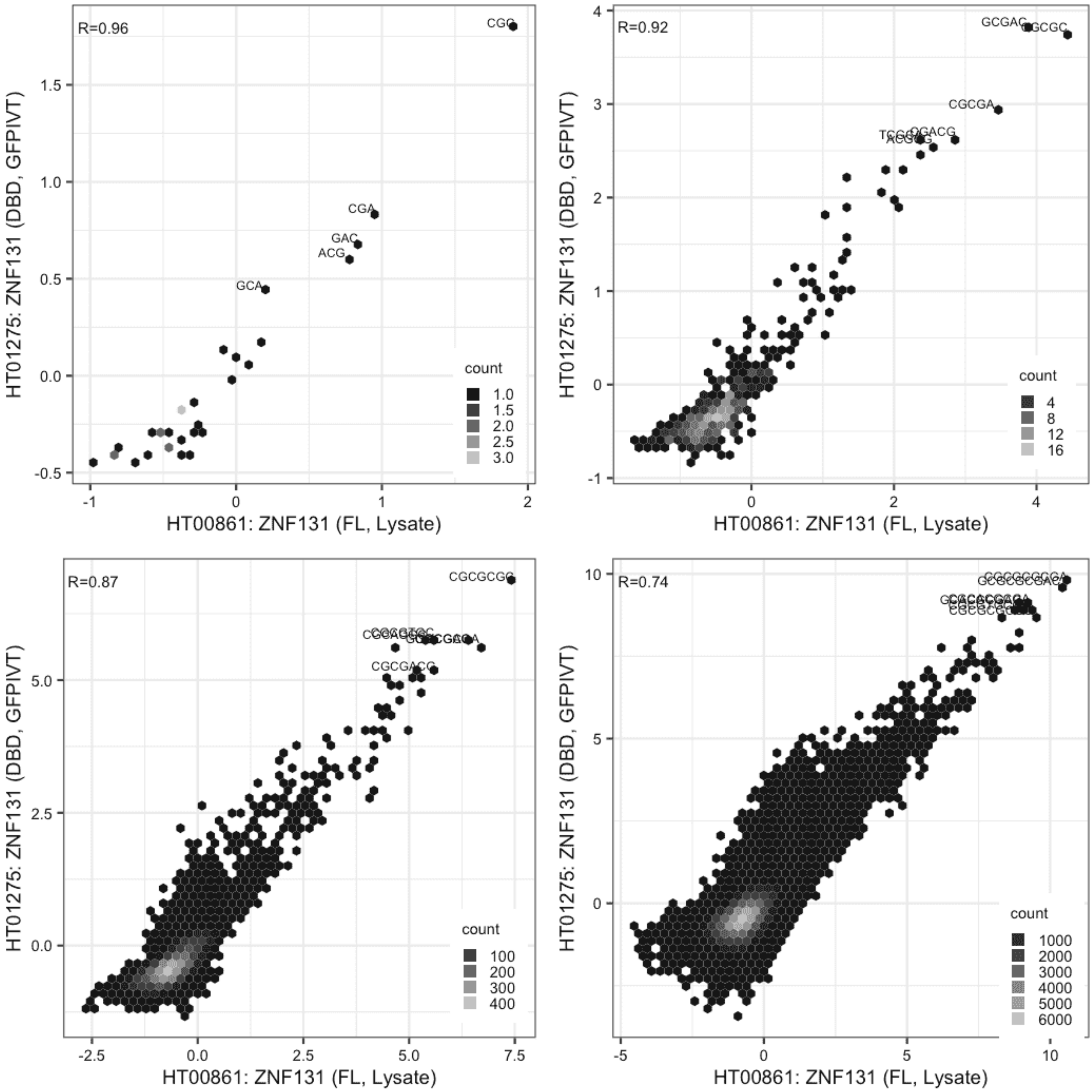

ZNF131

Lysate vs GFPIVT, same construct

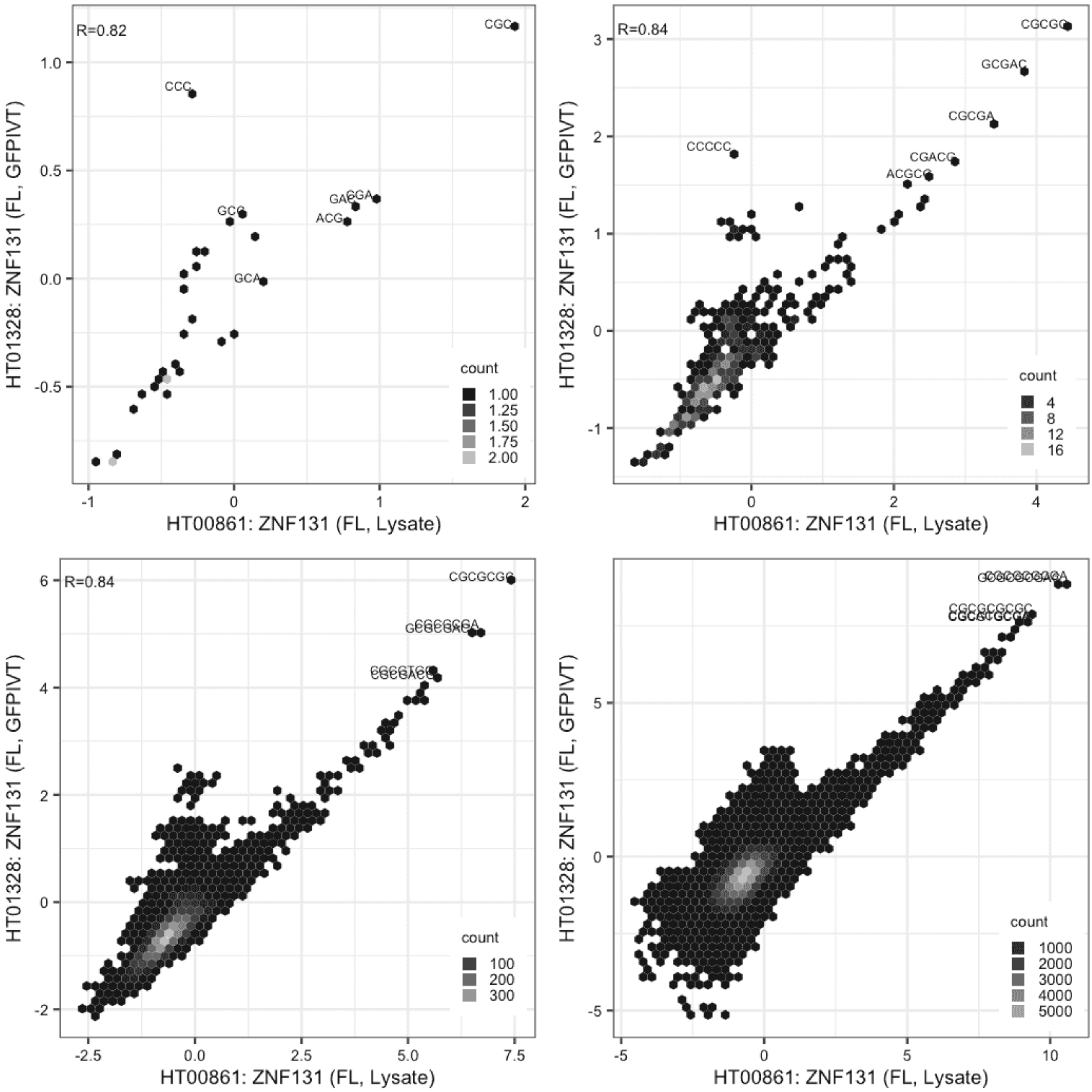

ZNF131

GFPIVT vs GFPIVT, different constructs

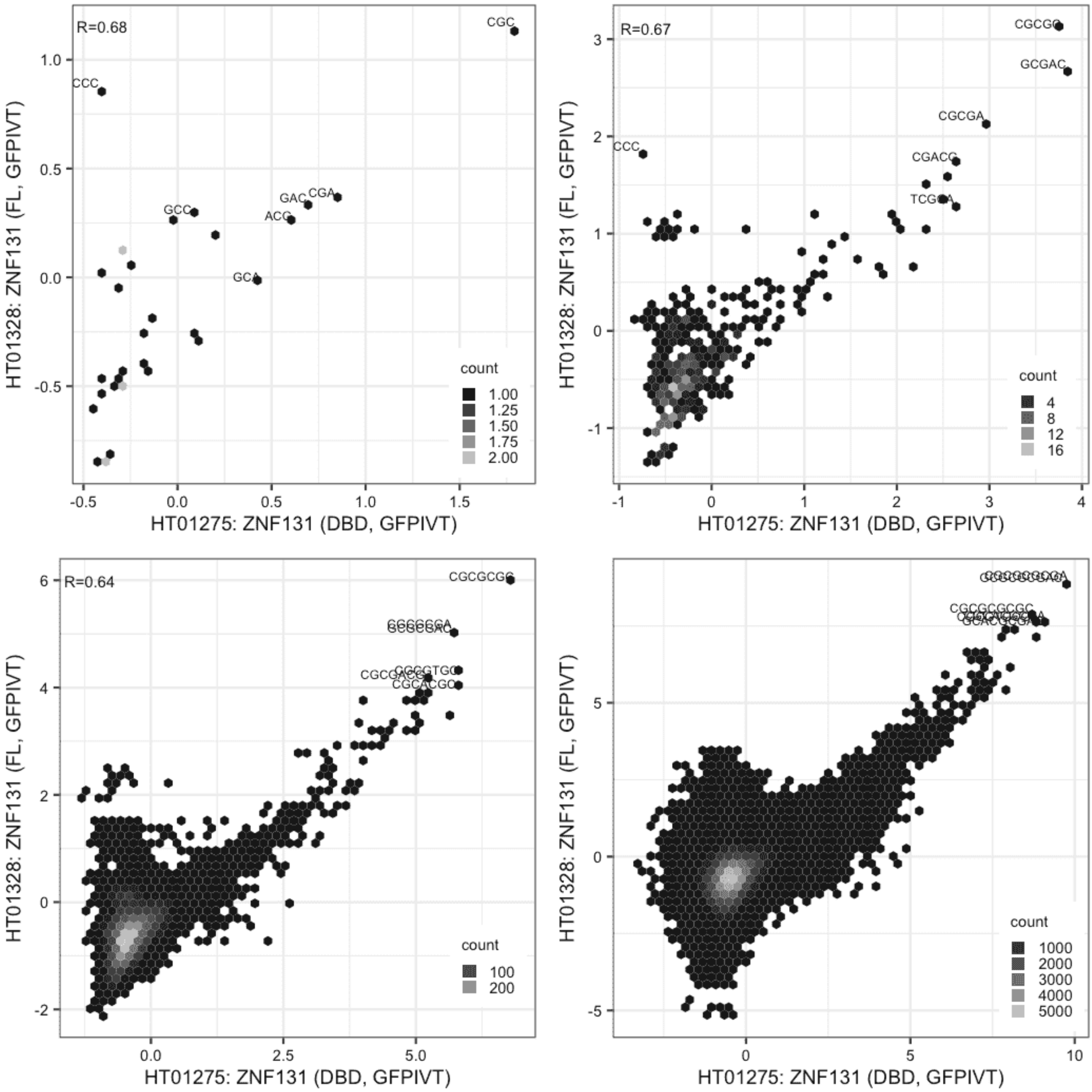

ZNF362

Lysate vs IVT, different constructs

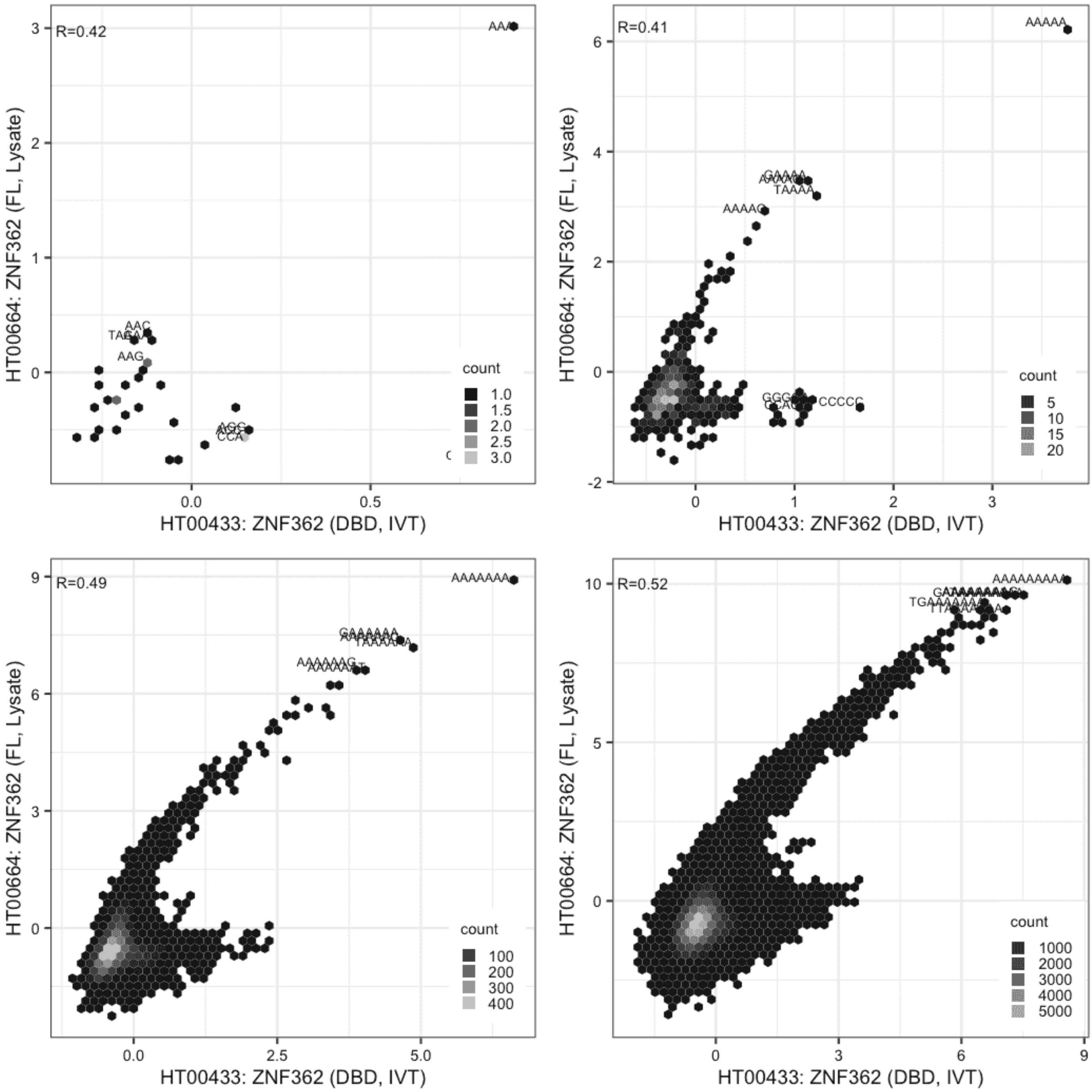

ZNF362

Lysate vs IVT, different constructs

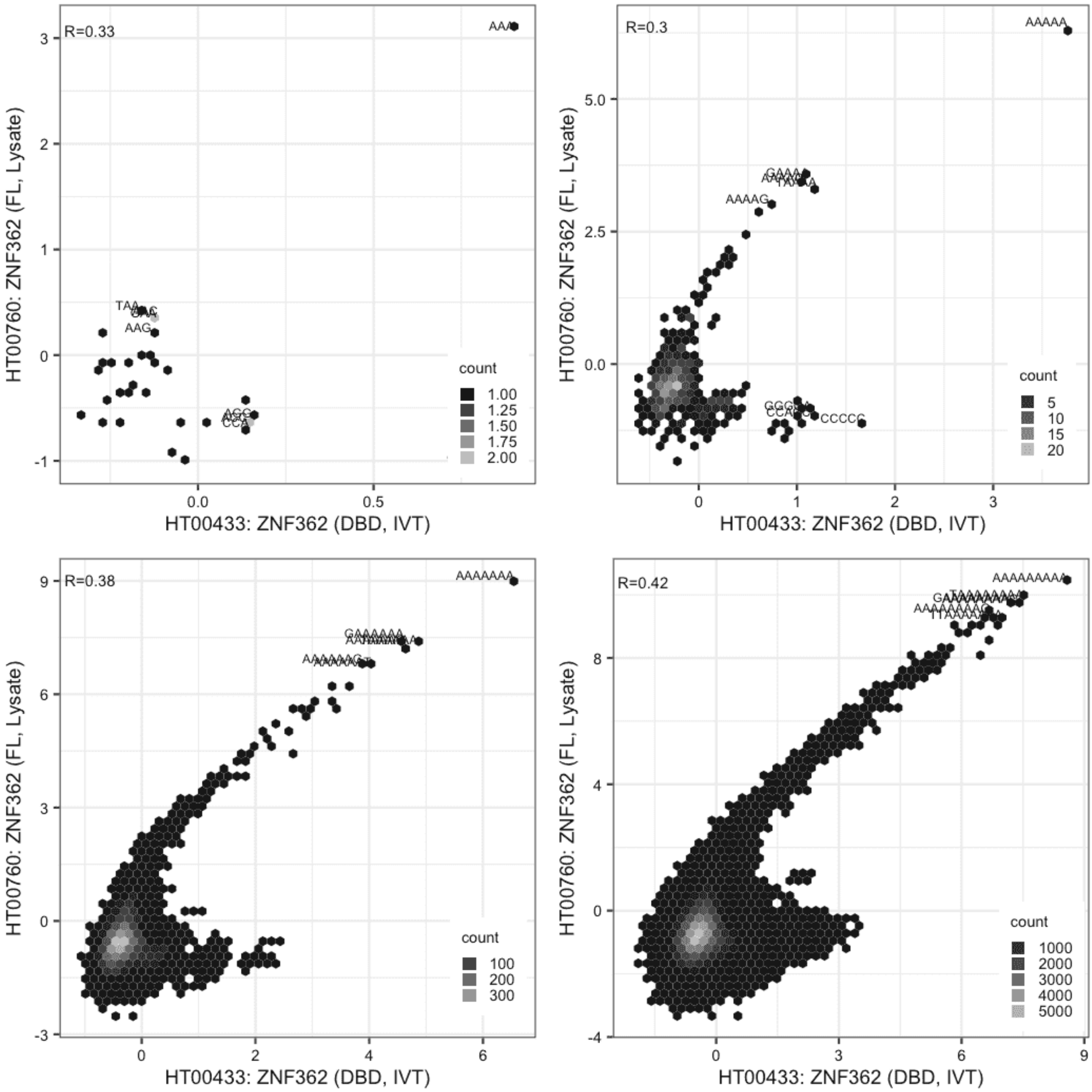

ZNF362

IVT vs GFPIVT, different constructs

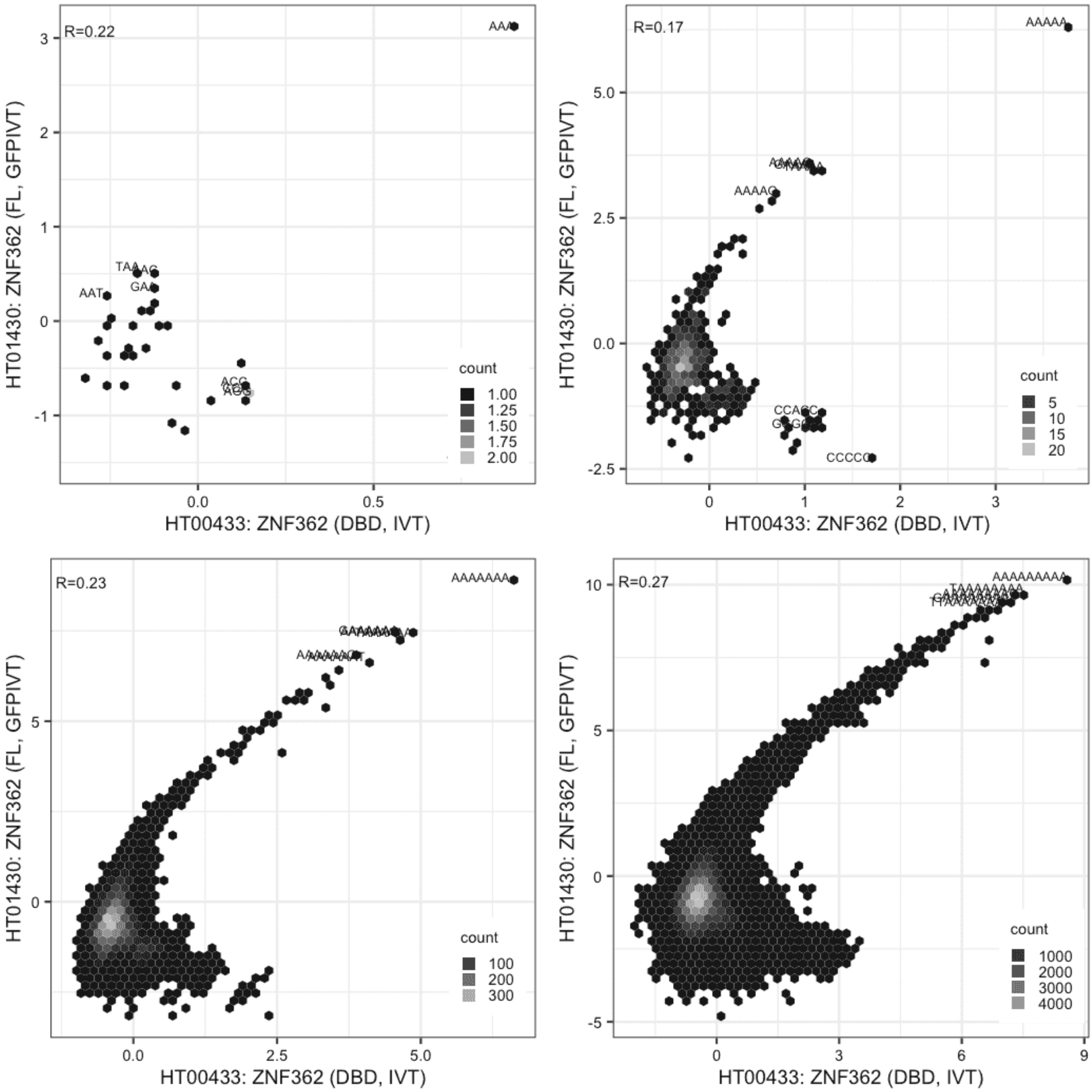

ZNF362

Lysate vs Lysate, same construct

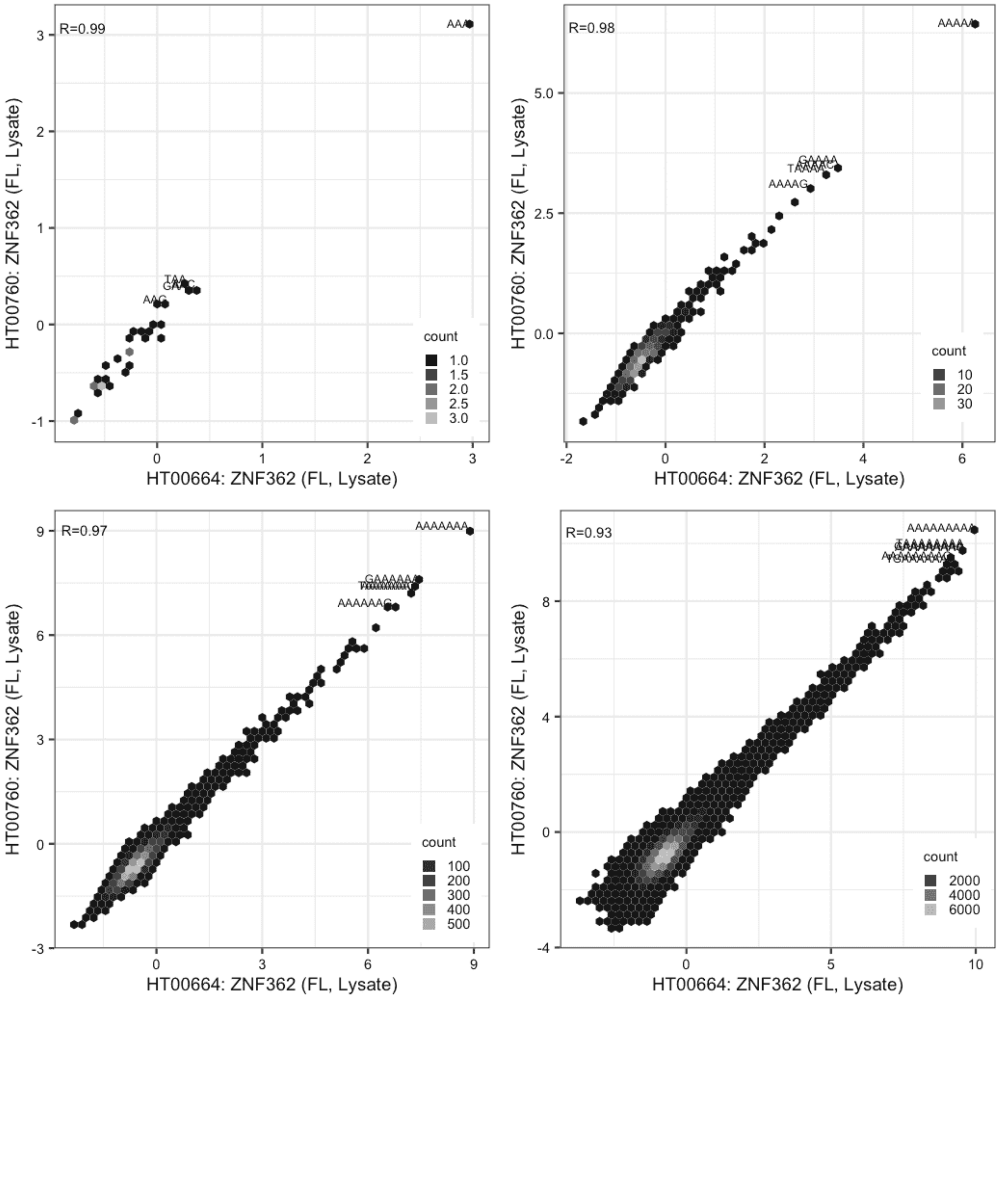

ZNF362

Lysate vs GFPIVT, same construct

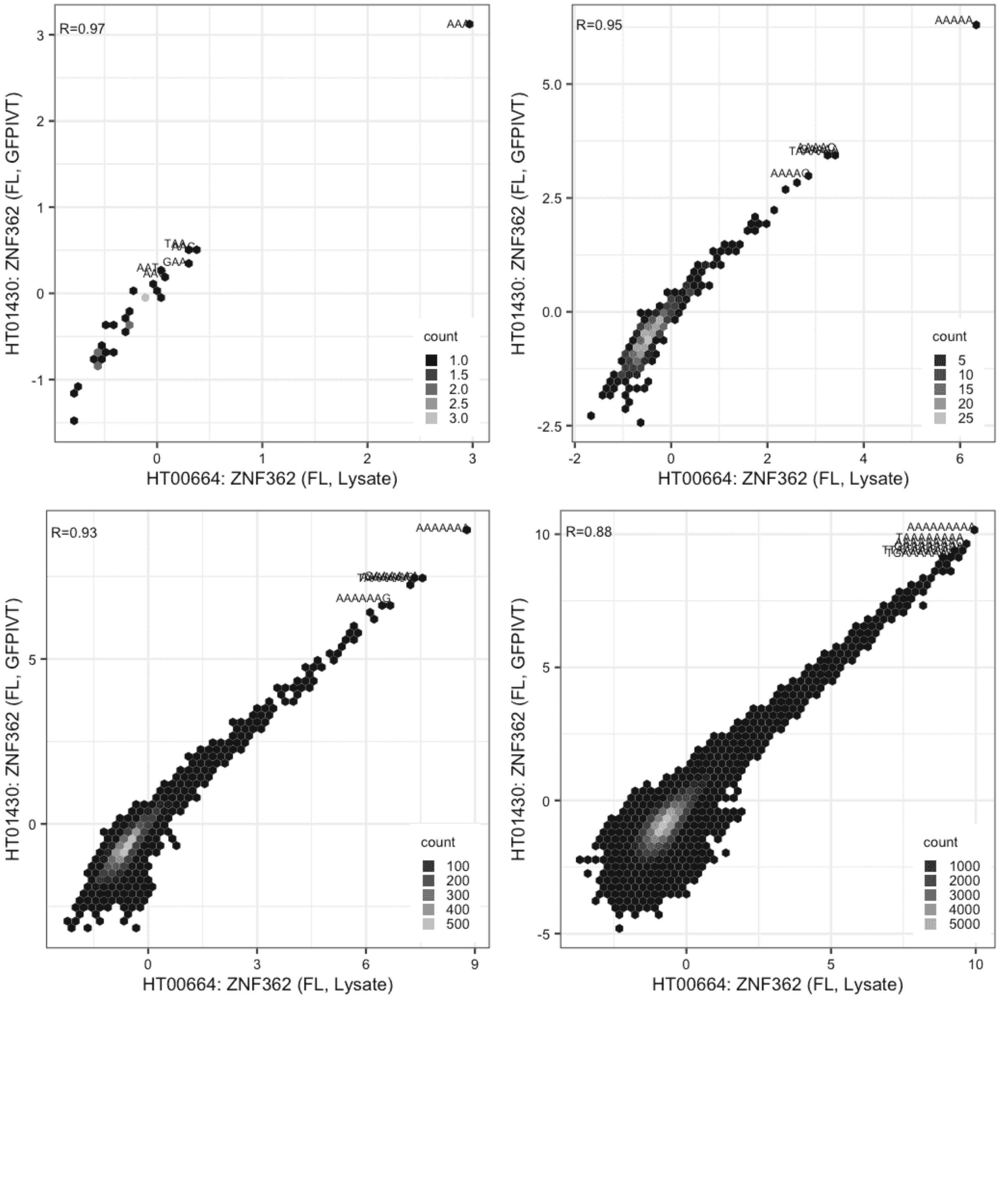

ZNF362

Lysate vs GFPIVT, same construct

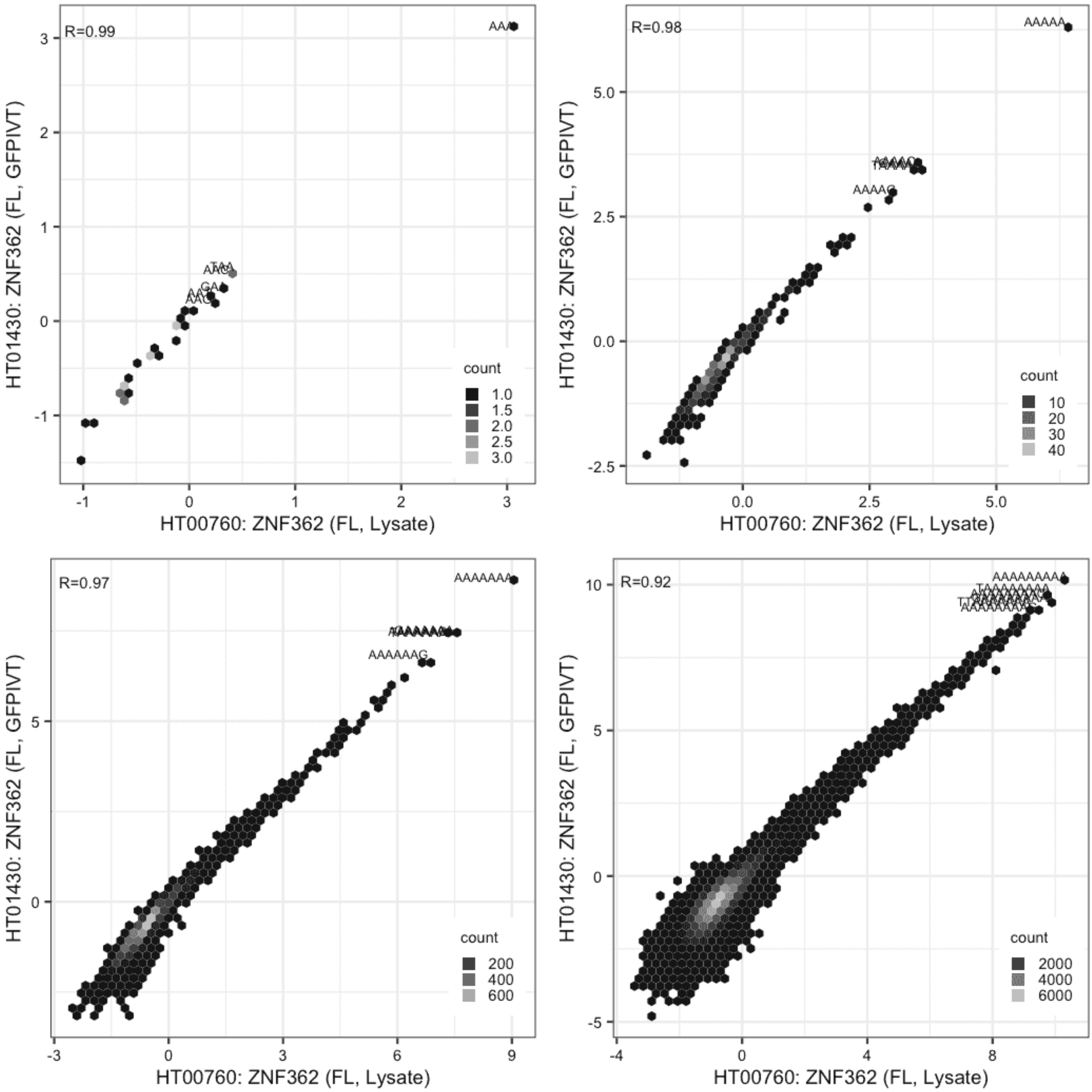

ZNF367

Lysate vs GFPIVT, same construct

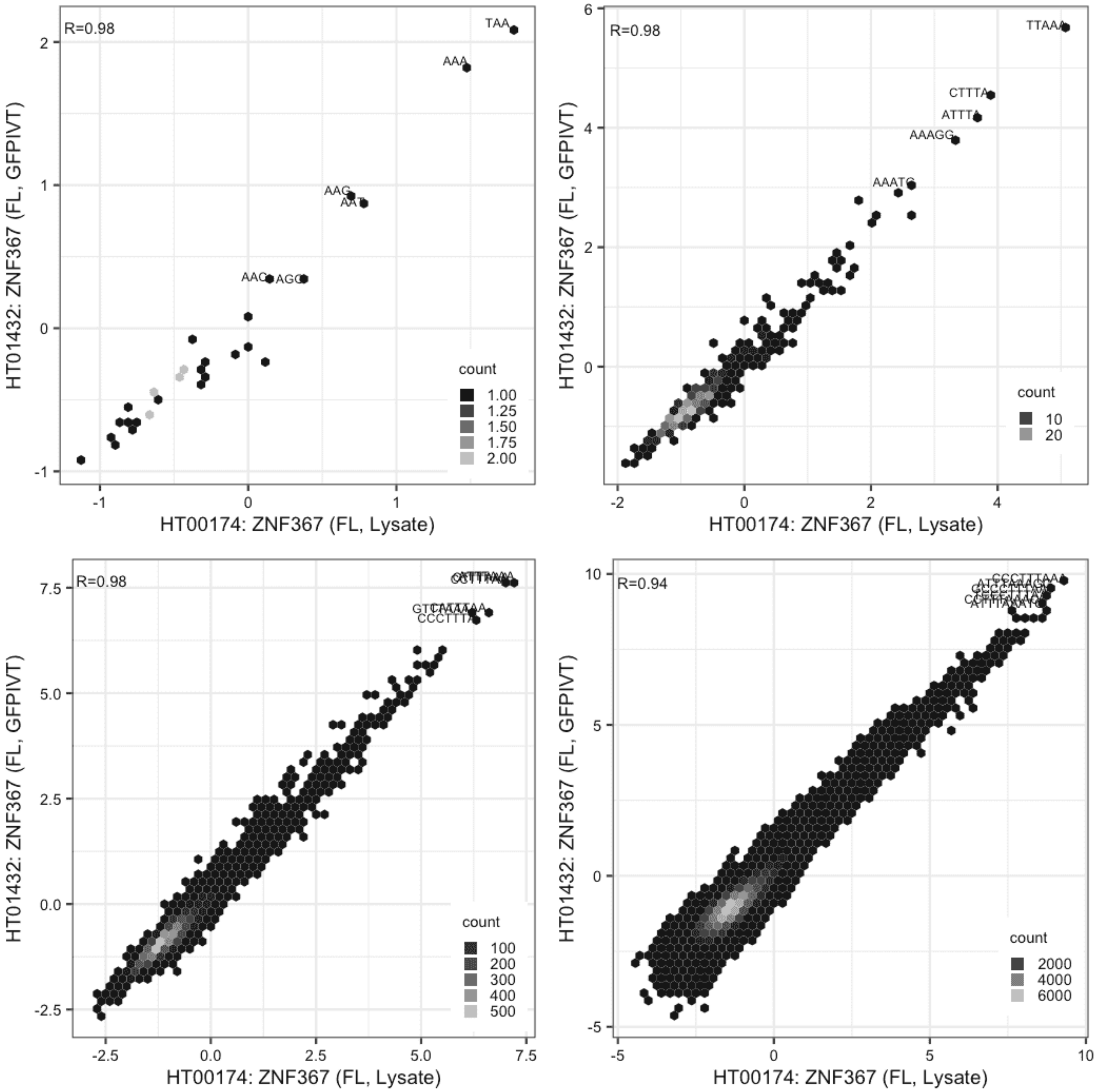

ZNF395

Lysate vs IVT, different constructs

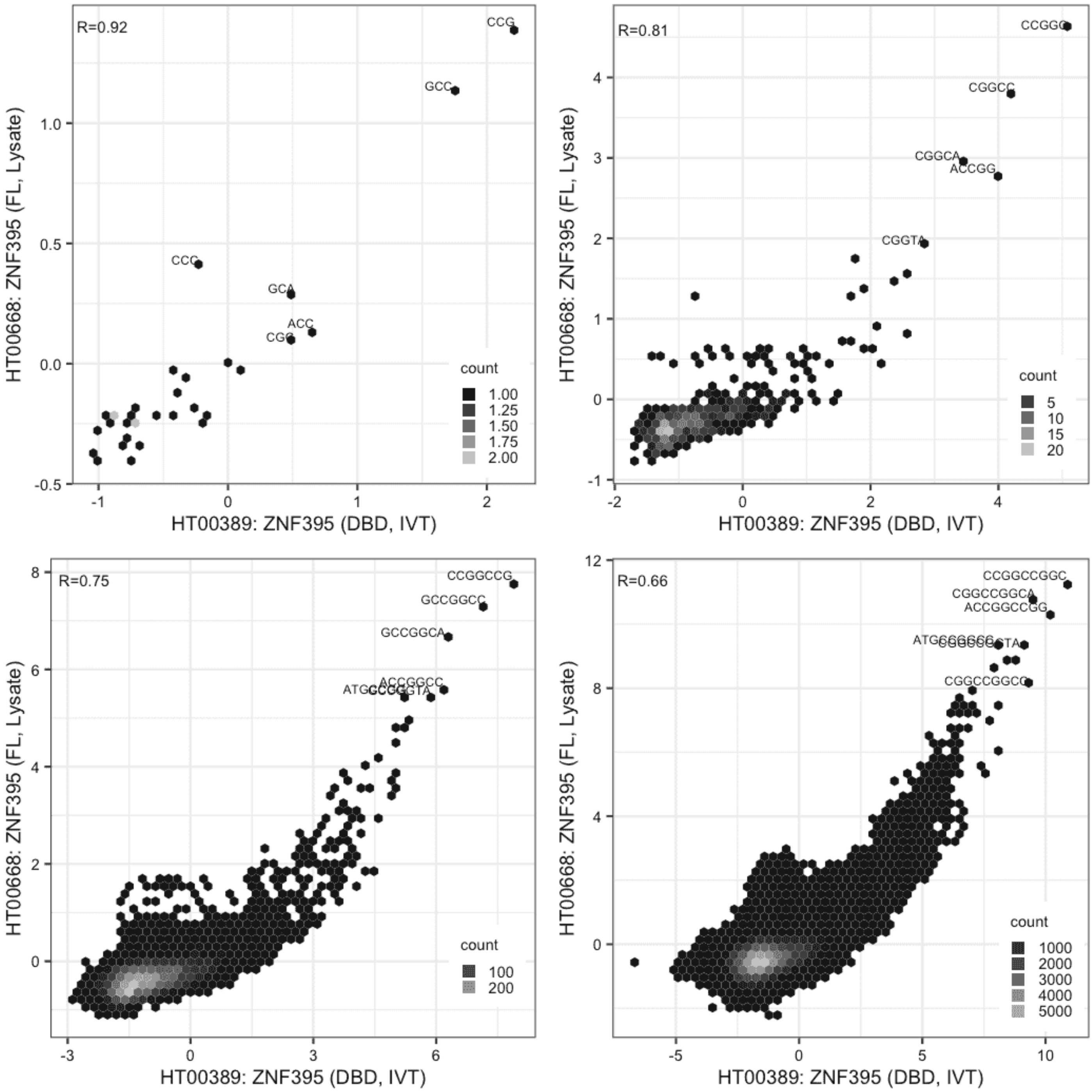

ZNF395

Lysate vs IVT, different constructs

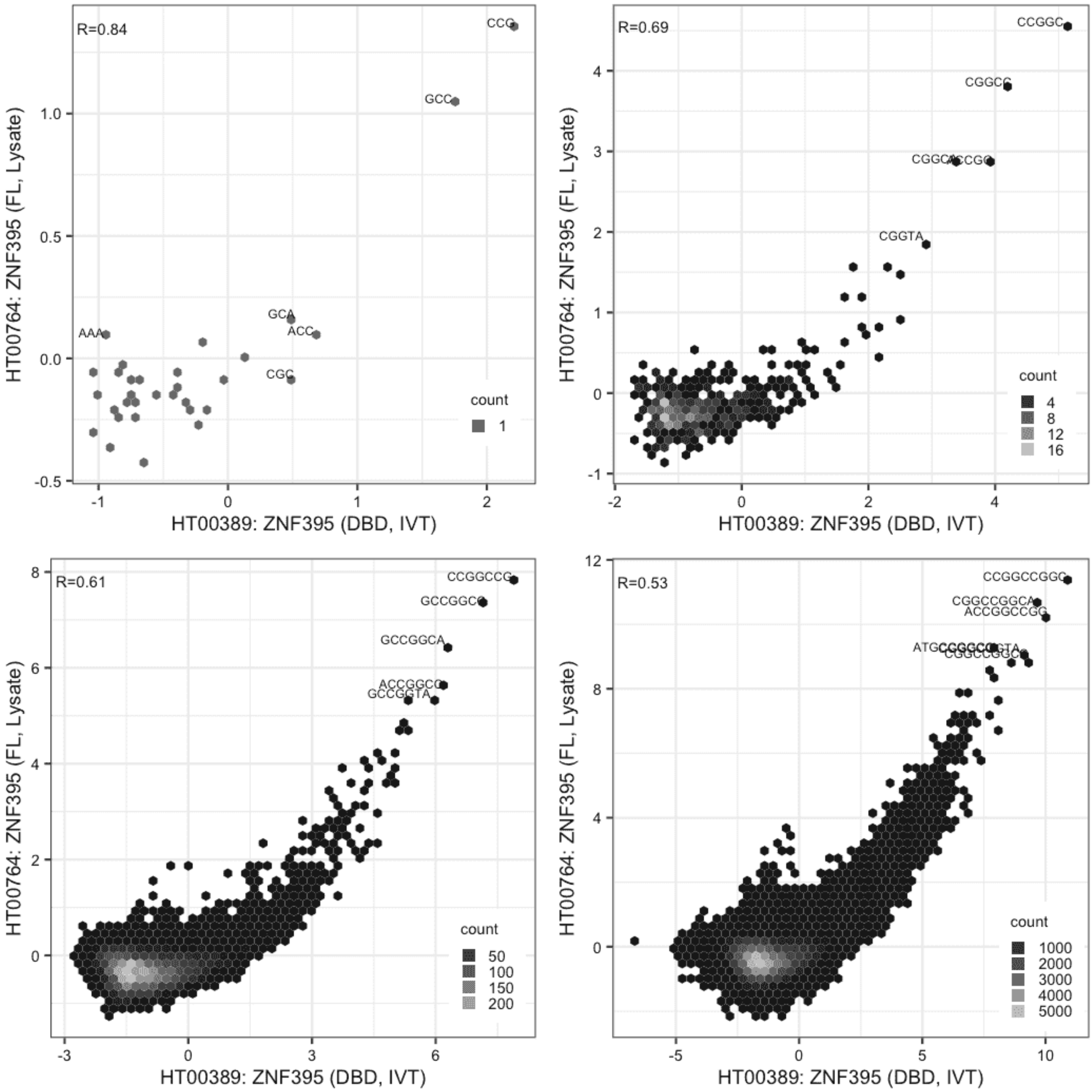

ZNF395

IVT vs GFPIVT, same construct

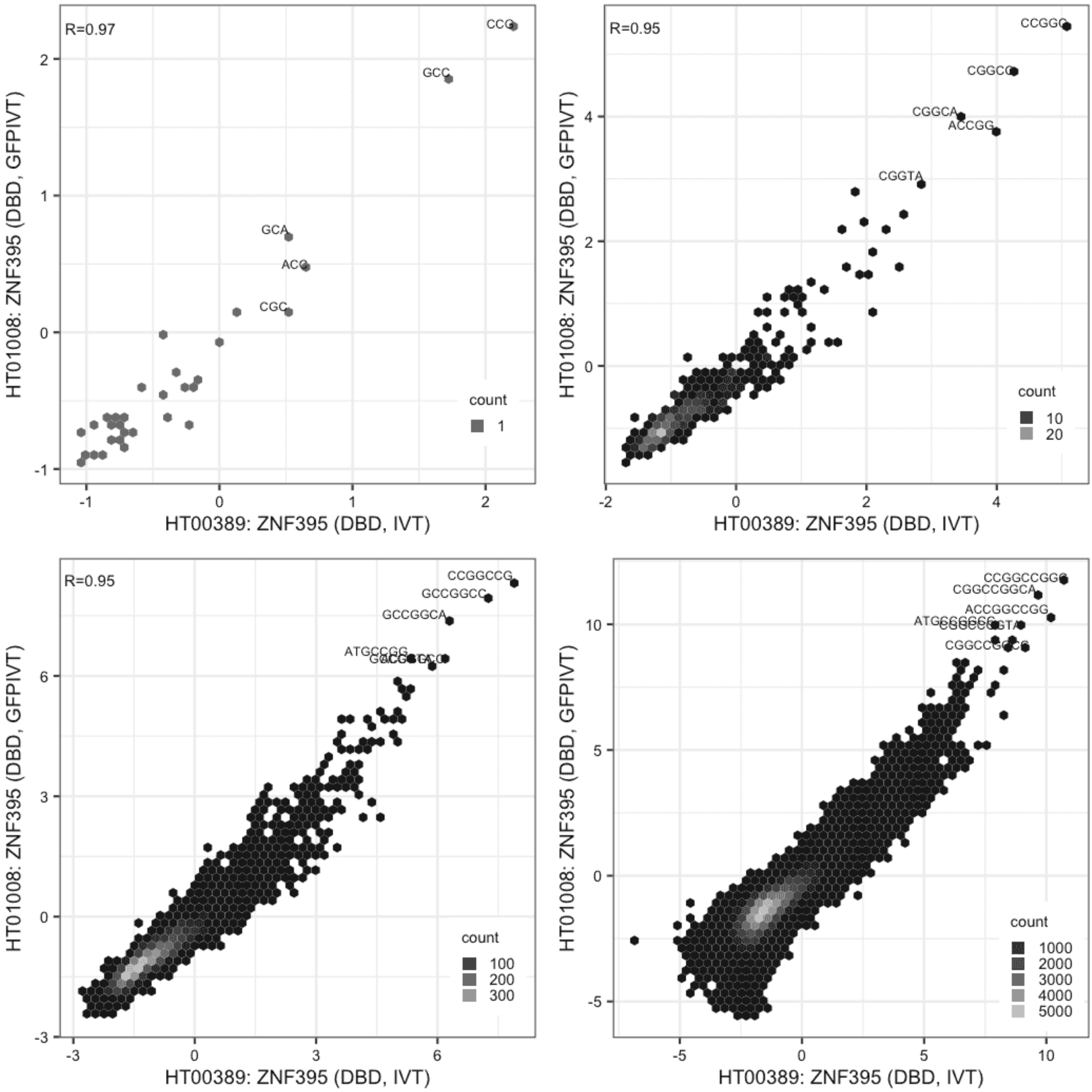

ZNF395

IVT vs GFPIVT, different constructs

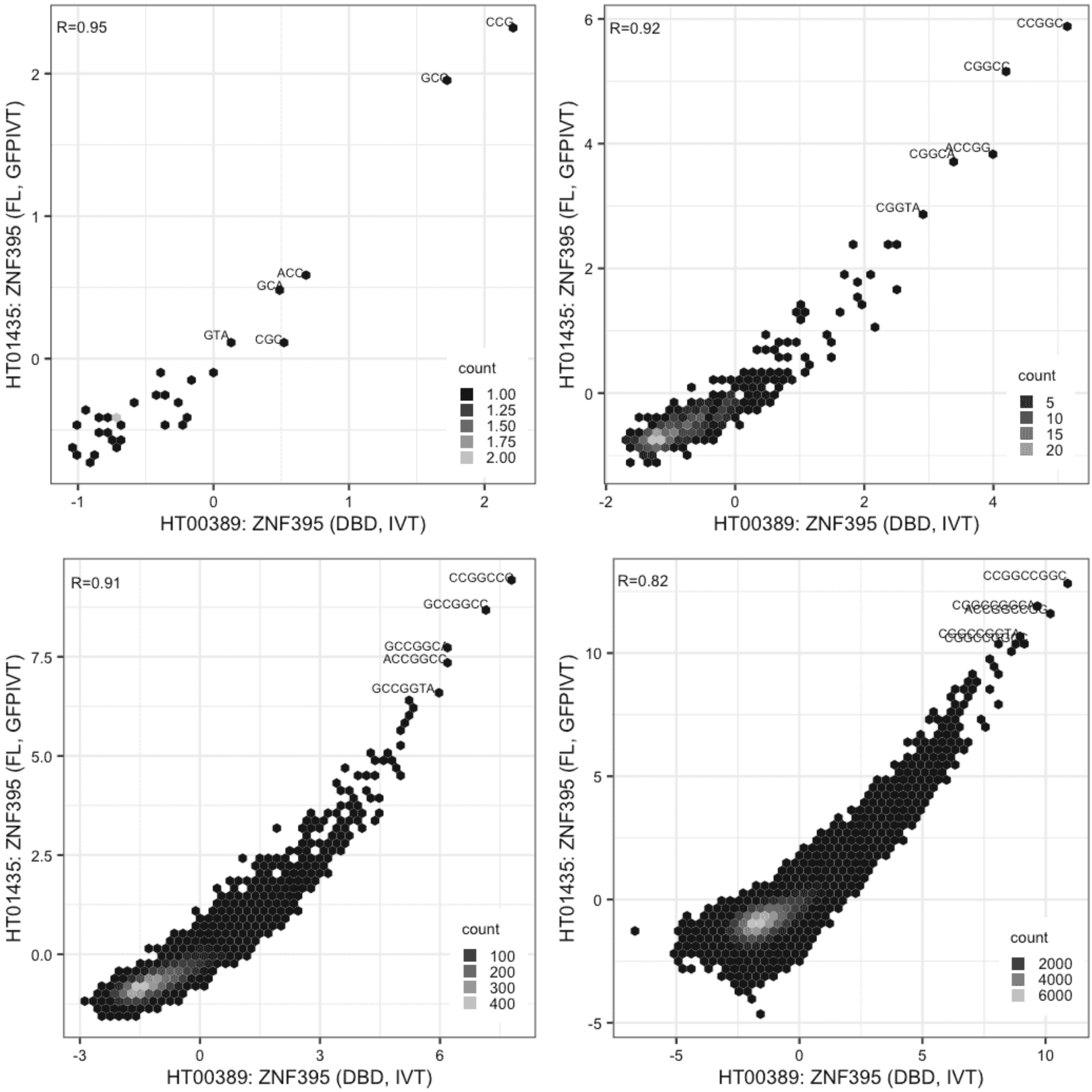

ZNF395

Lysate vs Lysate, same construct

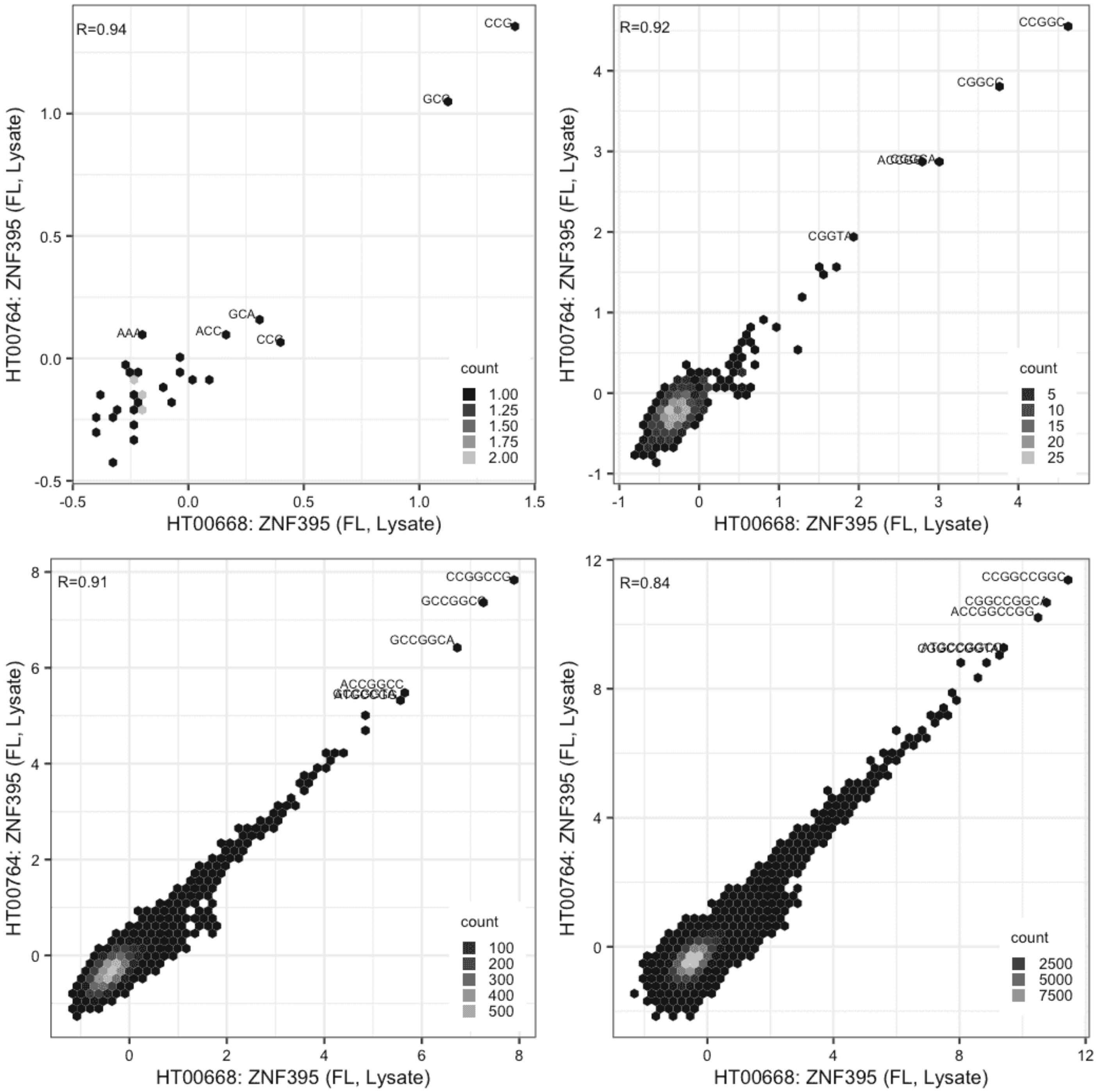

ZNF395

Lysate vs GFPIVT, different constructs

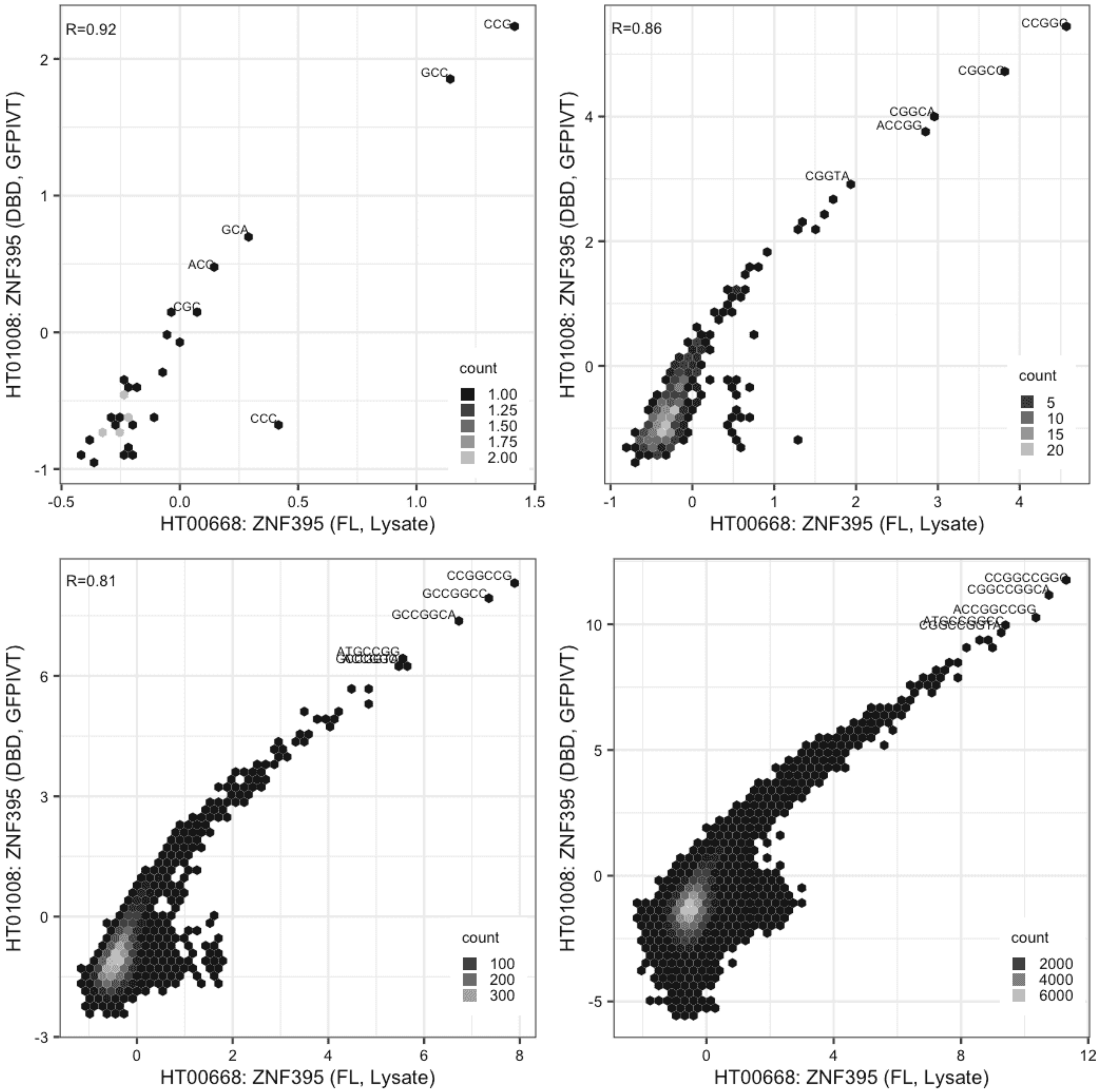

ZNF395

Lysate vs GFPIVT, same construct

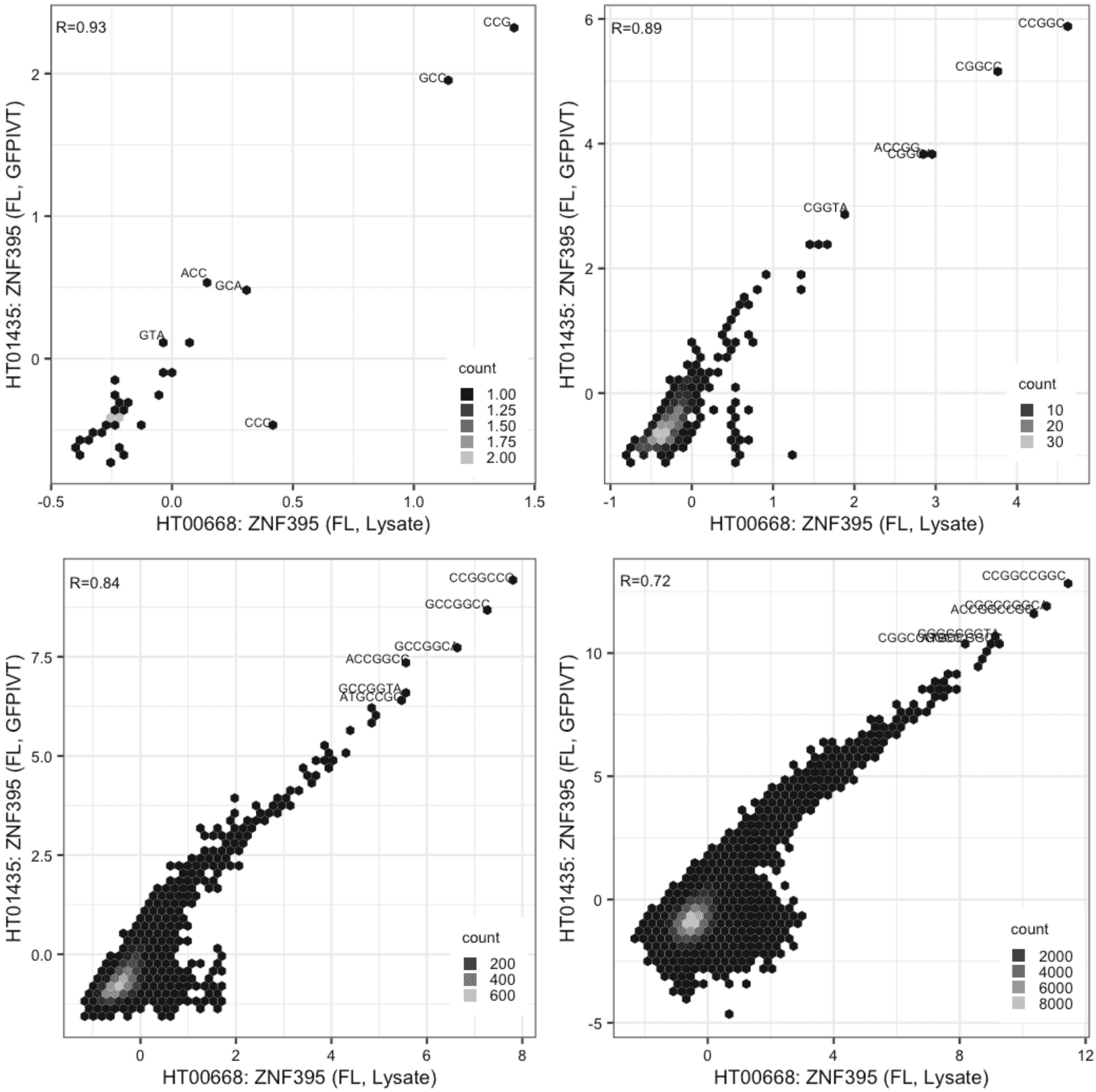

ZNF395

Lysate vs GFPIVT, different constructs

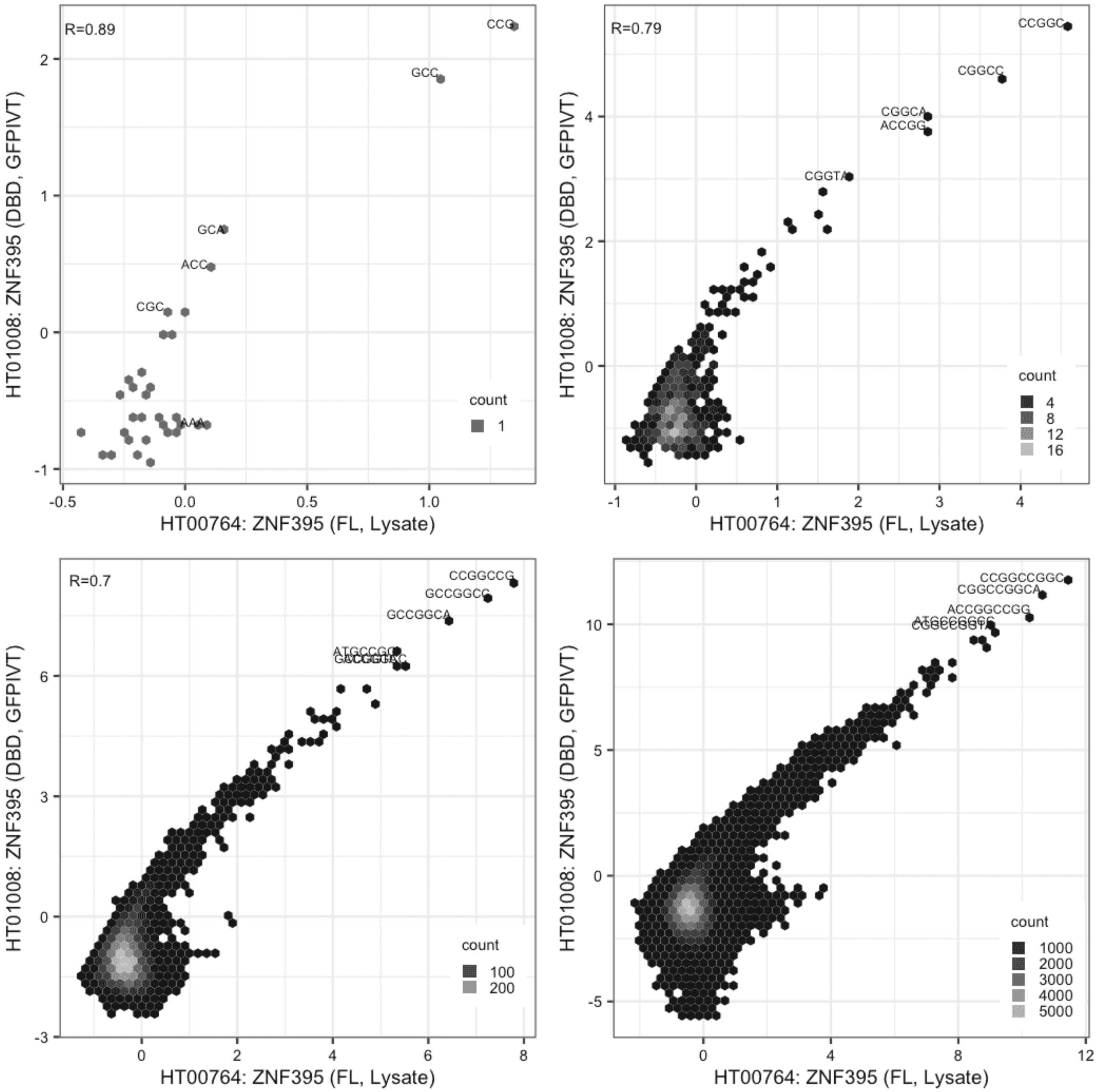

ZNF395

Lysate vs GFPIVT, same construct

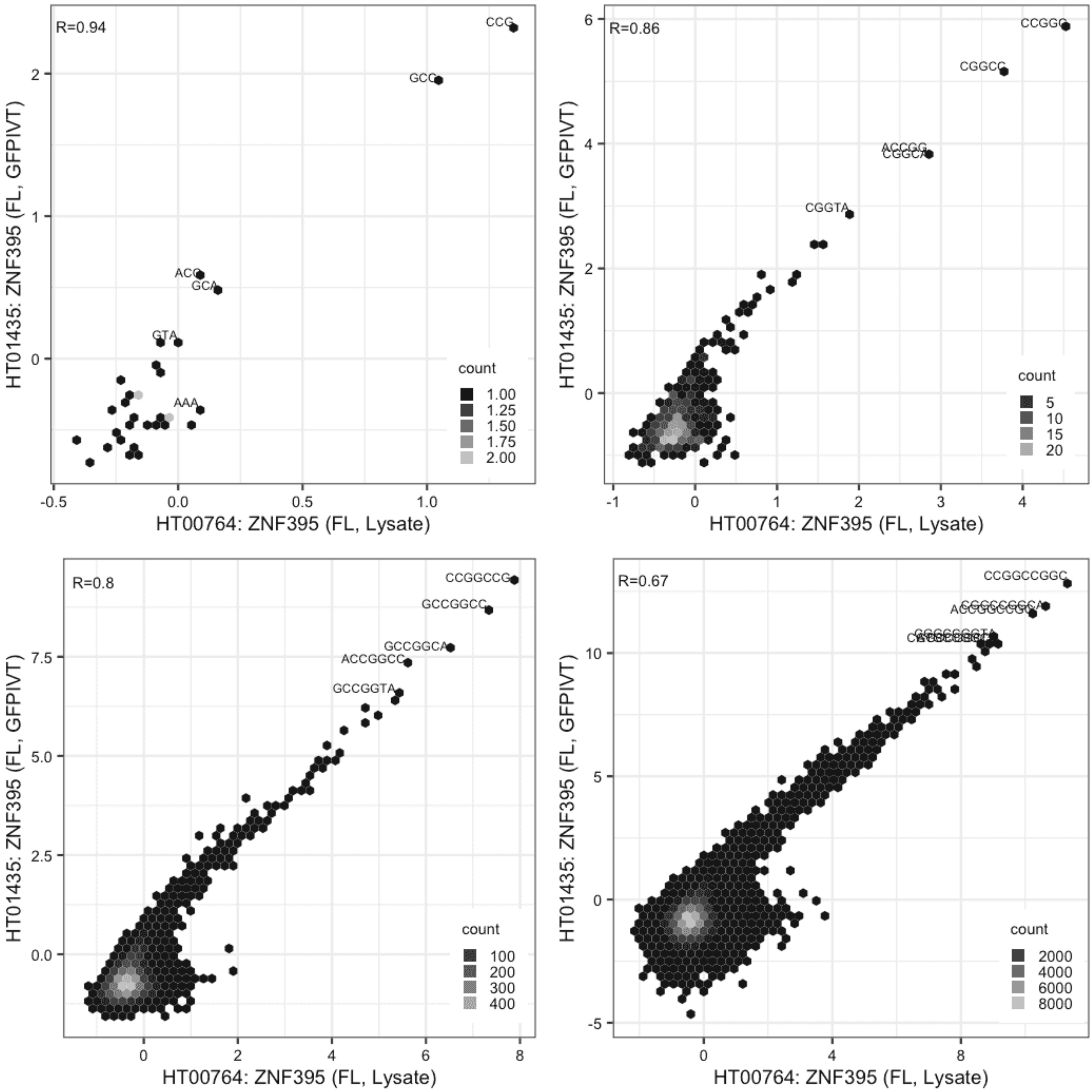

ZNF395

GFPIVT vs GFPIVT, different constructs

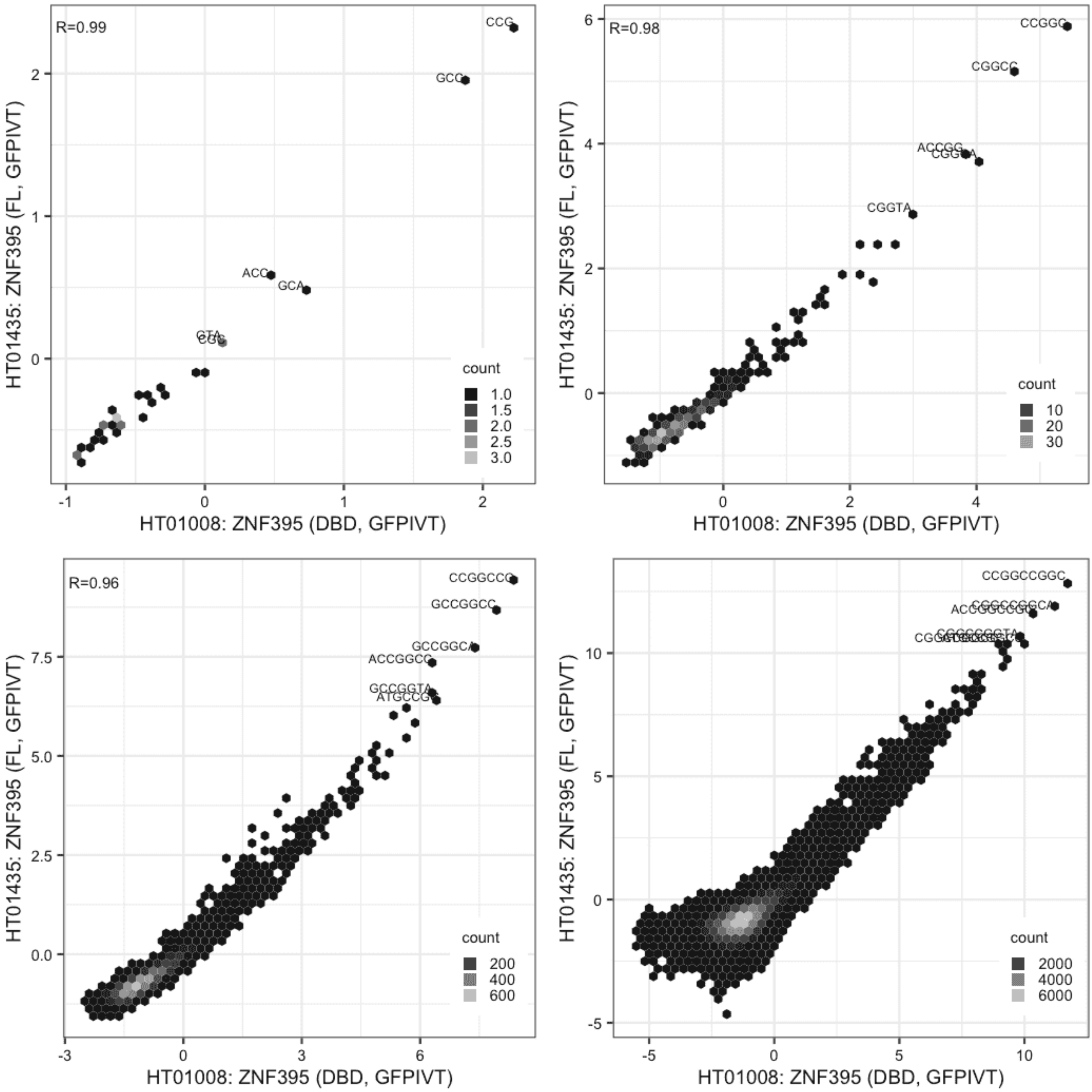

ZNF407

Lysate vs IVT, different constructs

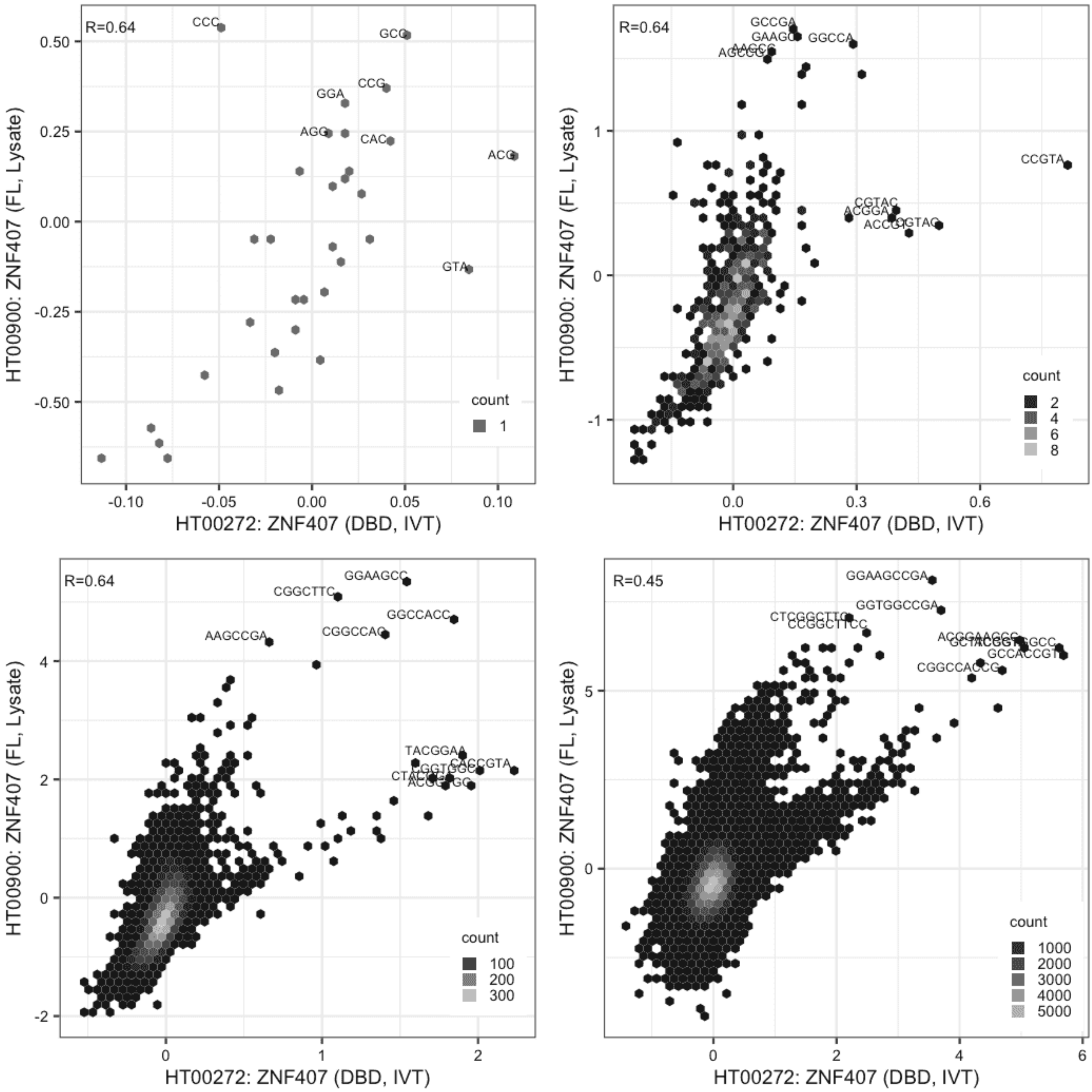

ZNF407

IVT vs GFPIVT, same construct

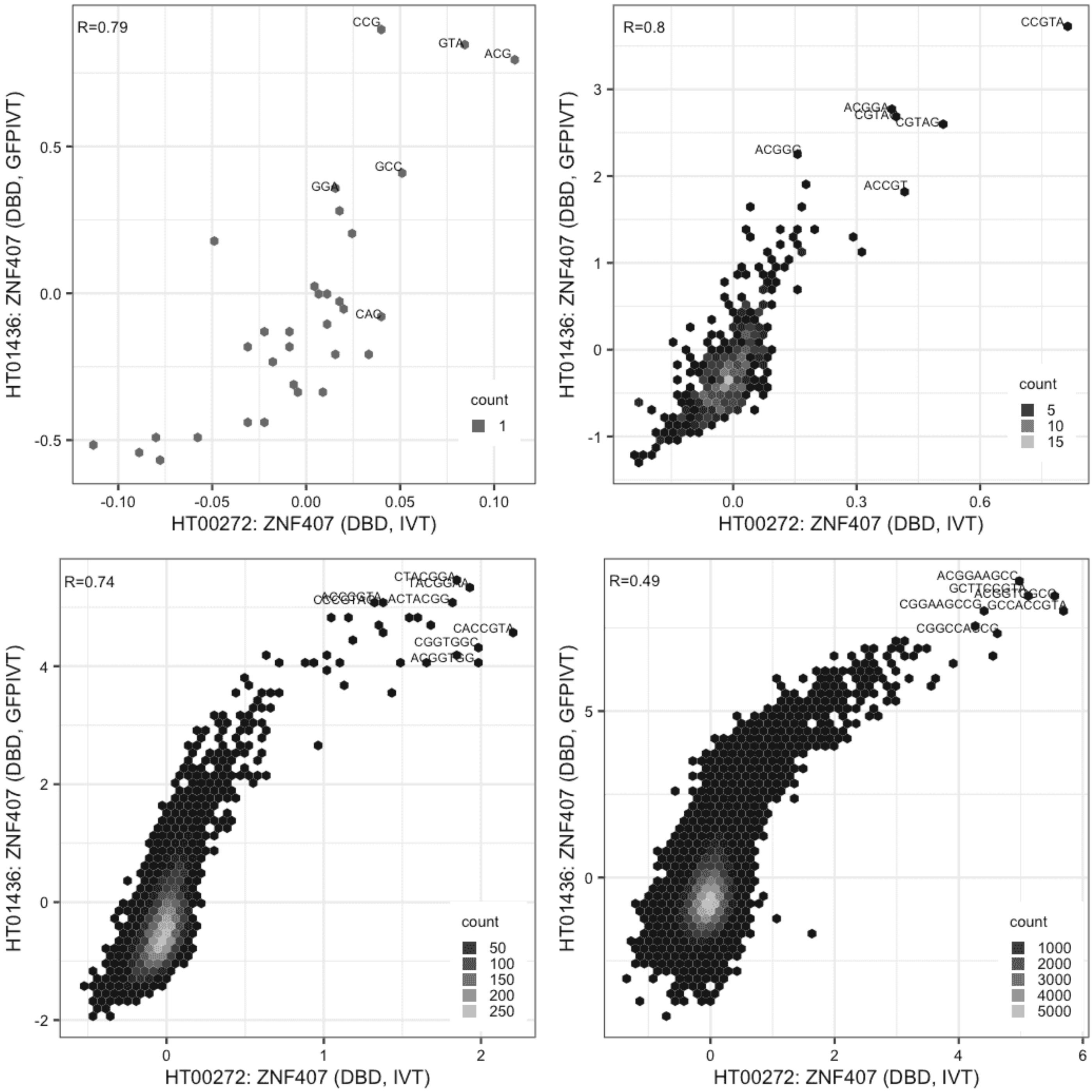

ZNF407

Lysate vs GFPIVT, different constructs

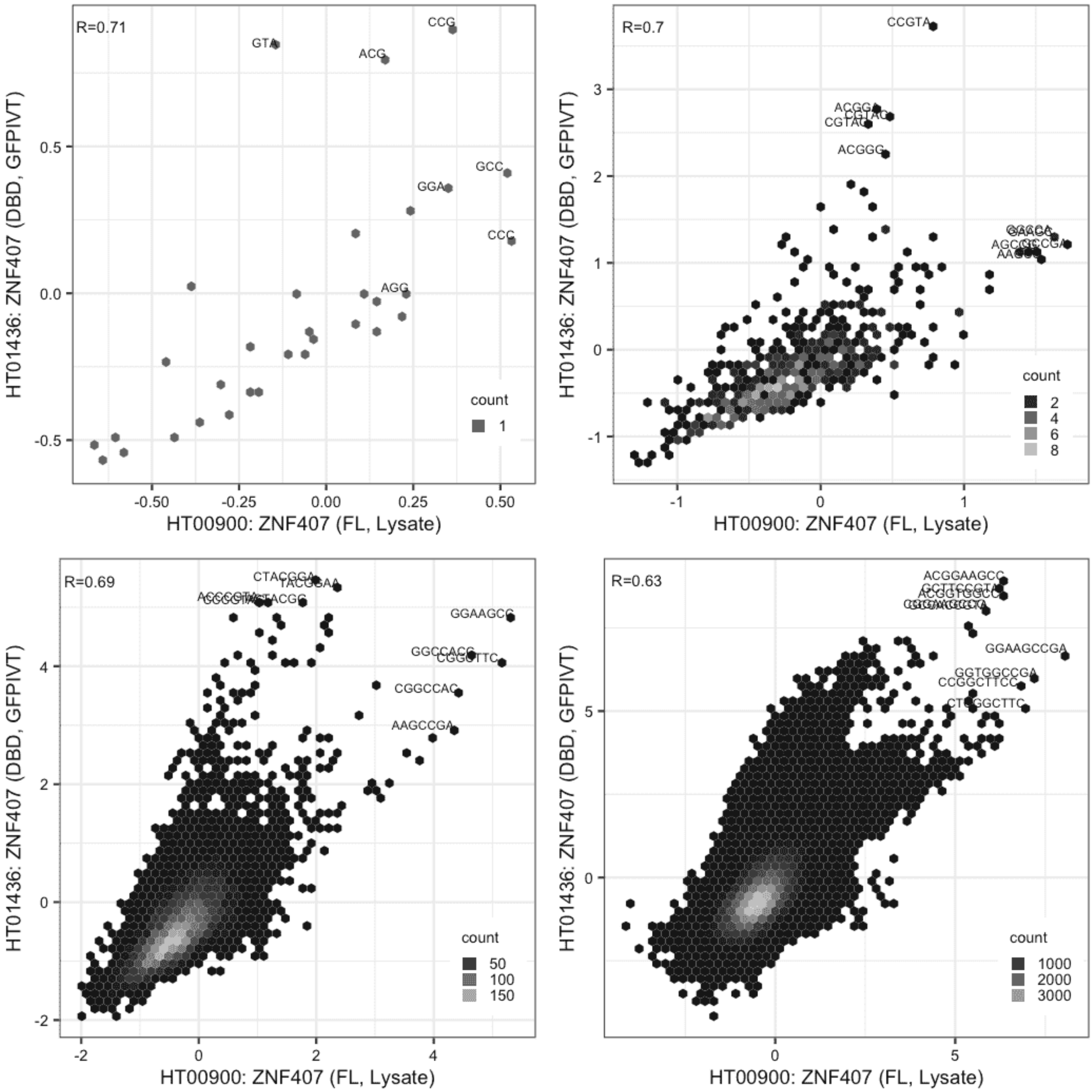

ZNF48

Lysate vs Lysate, same construct

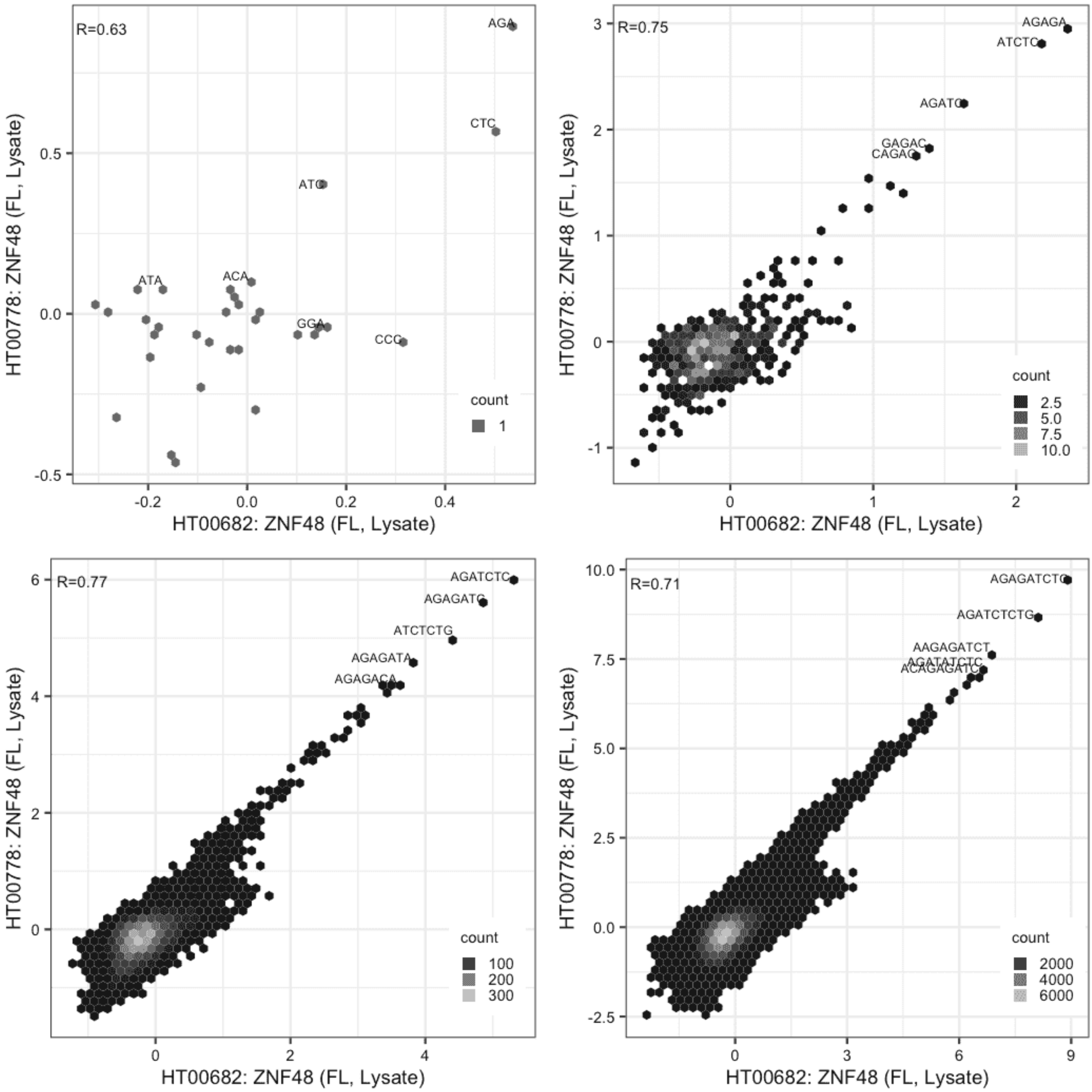

ZNF48

Lysate vs GFPIVT, same construct

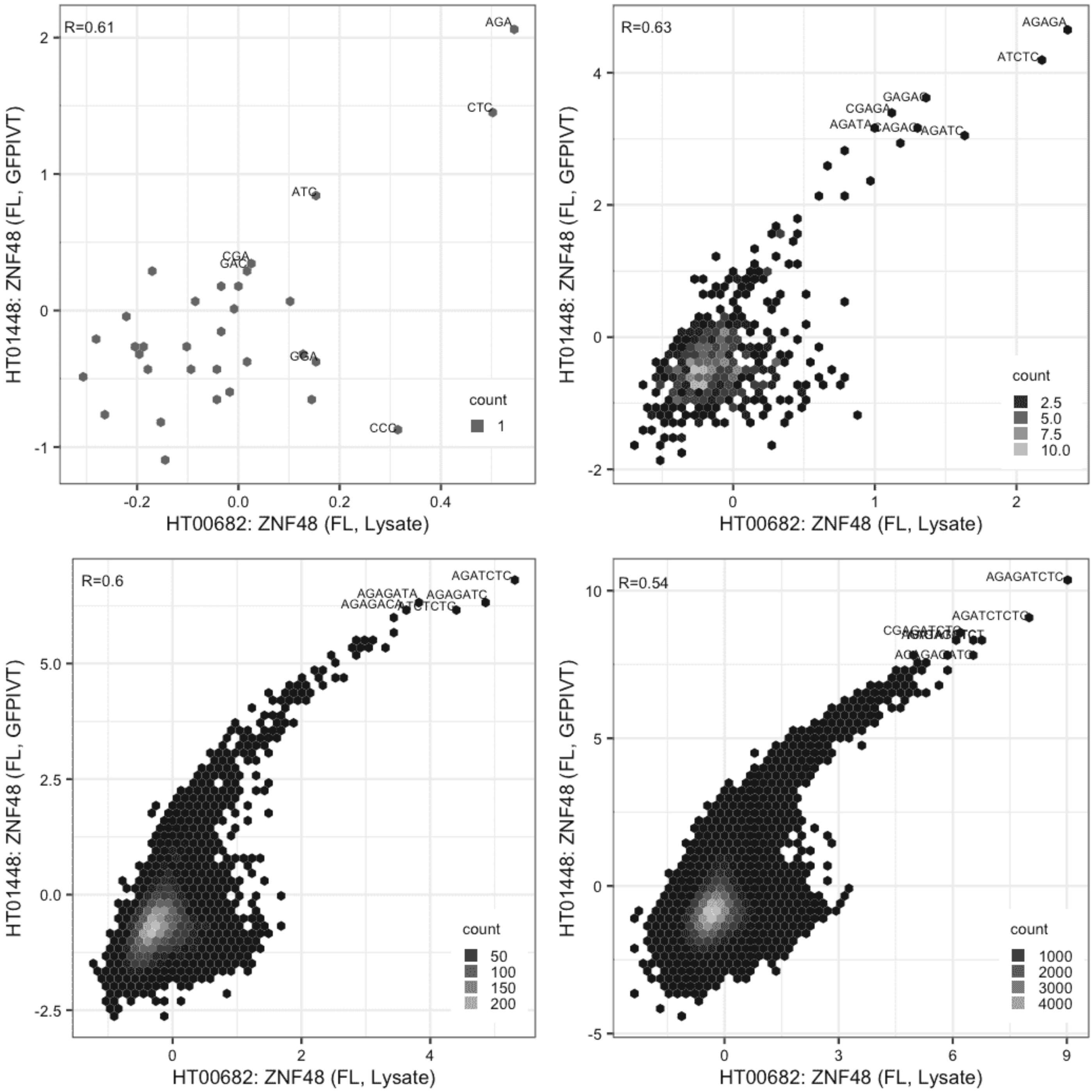

ZNF48

Lysate vs GFPIVT, same construct

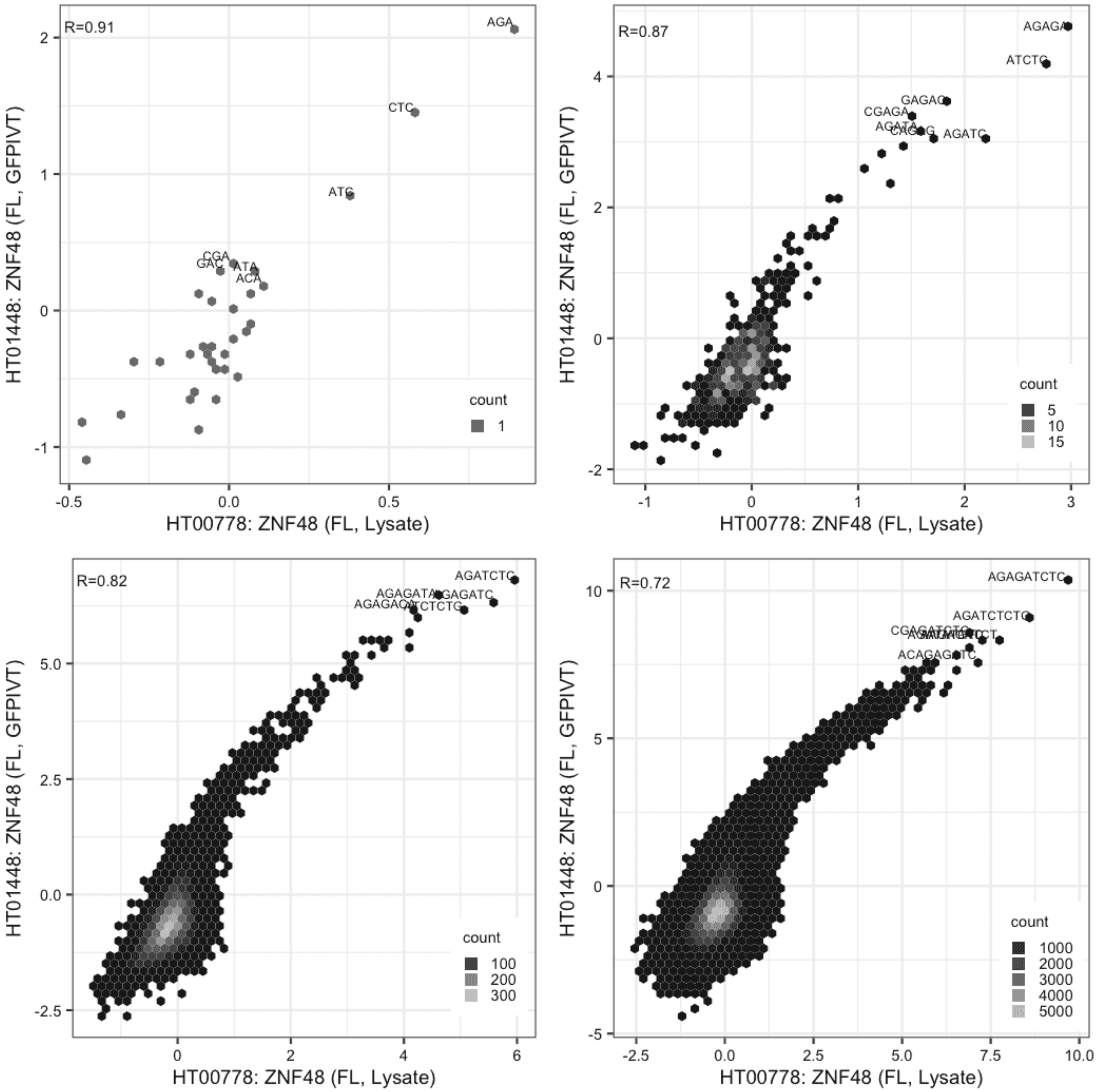

ZNF518B

Lysate vs Lysate, same construct

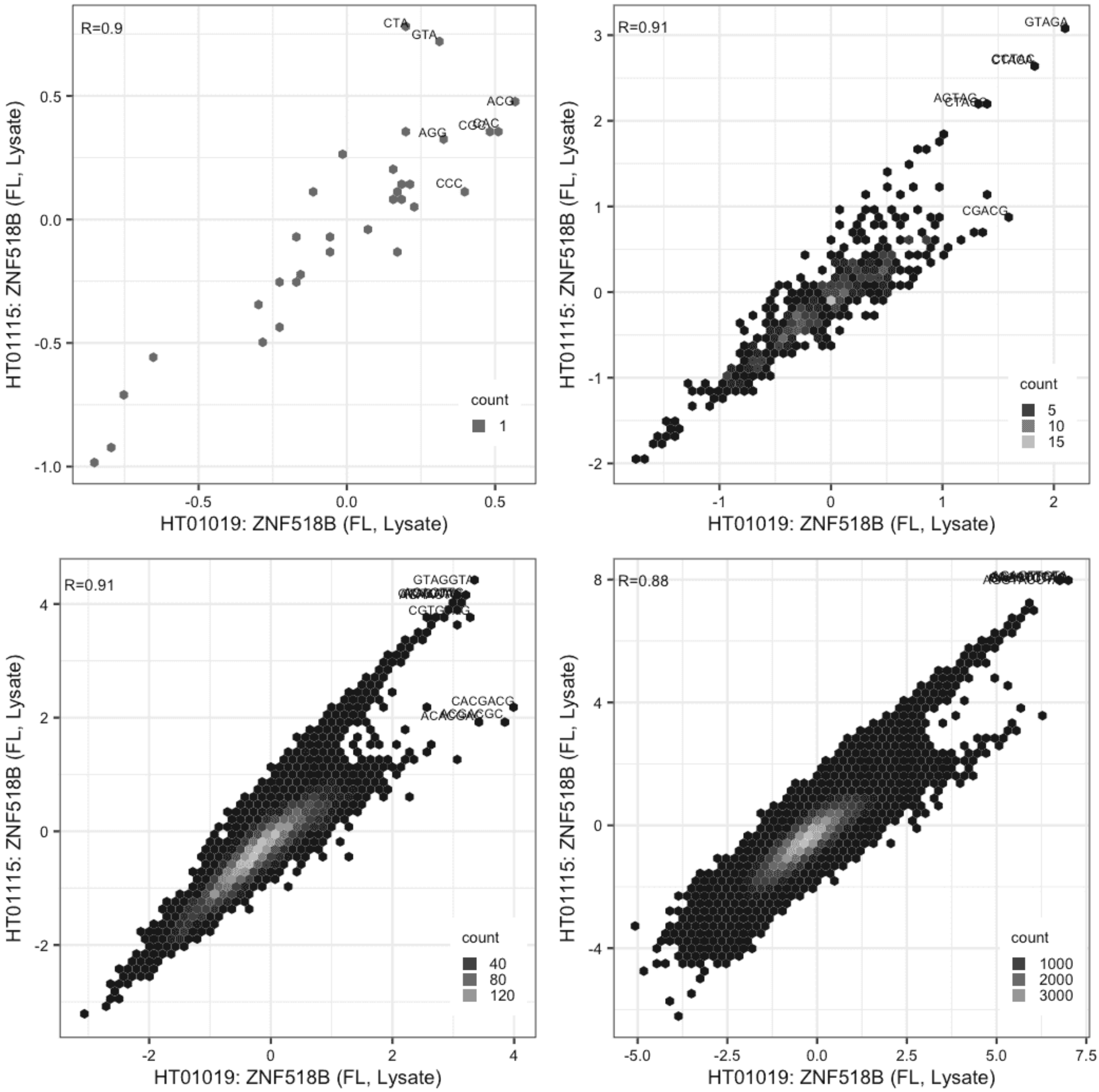

ZNF518B

Lysate vs GFPIVT, different constructs

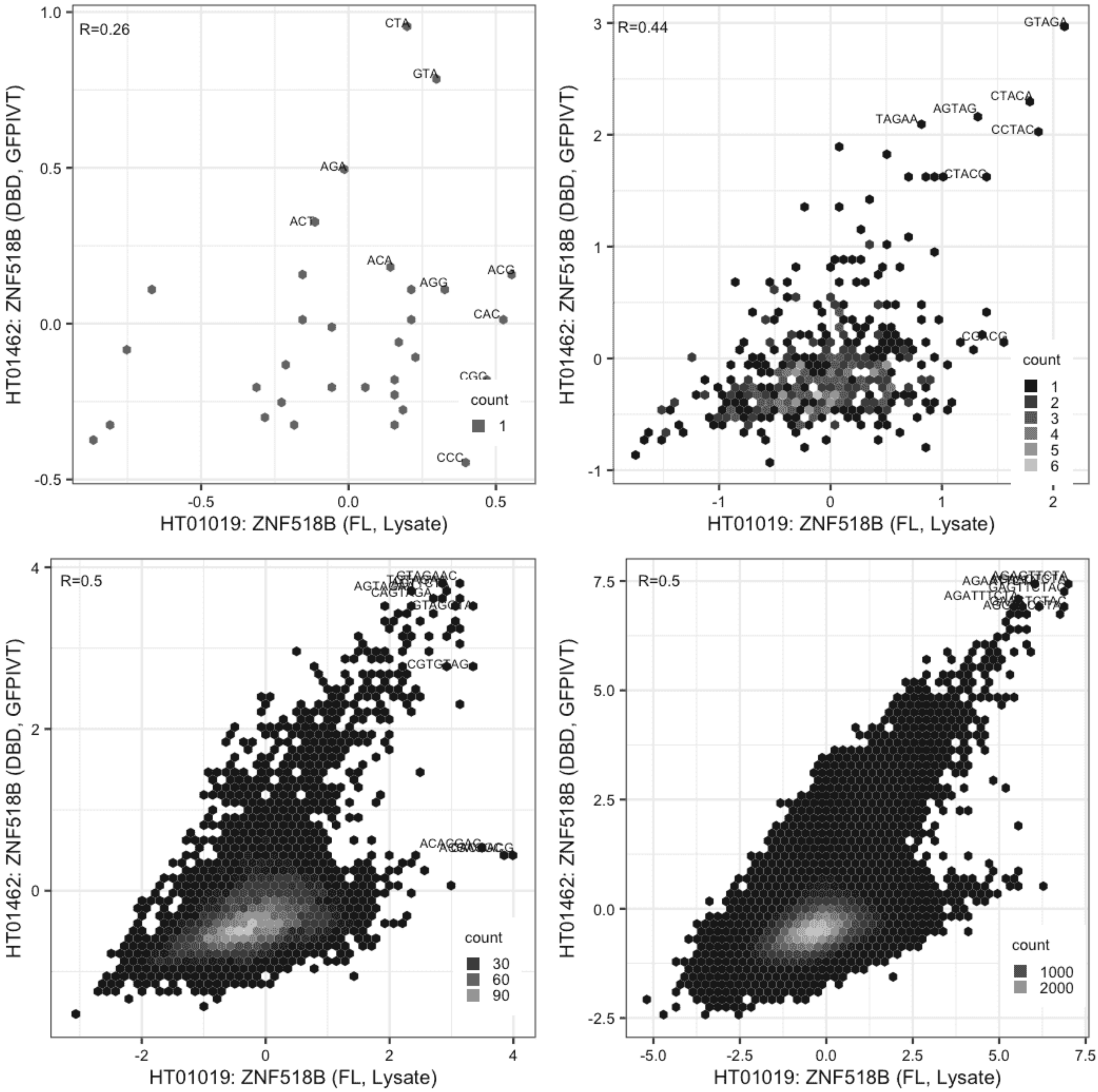

ZNF518B

Lysate vs GFPIVT, different constructs

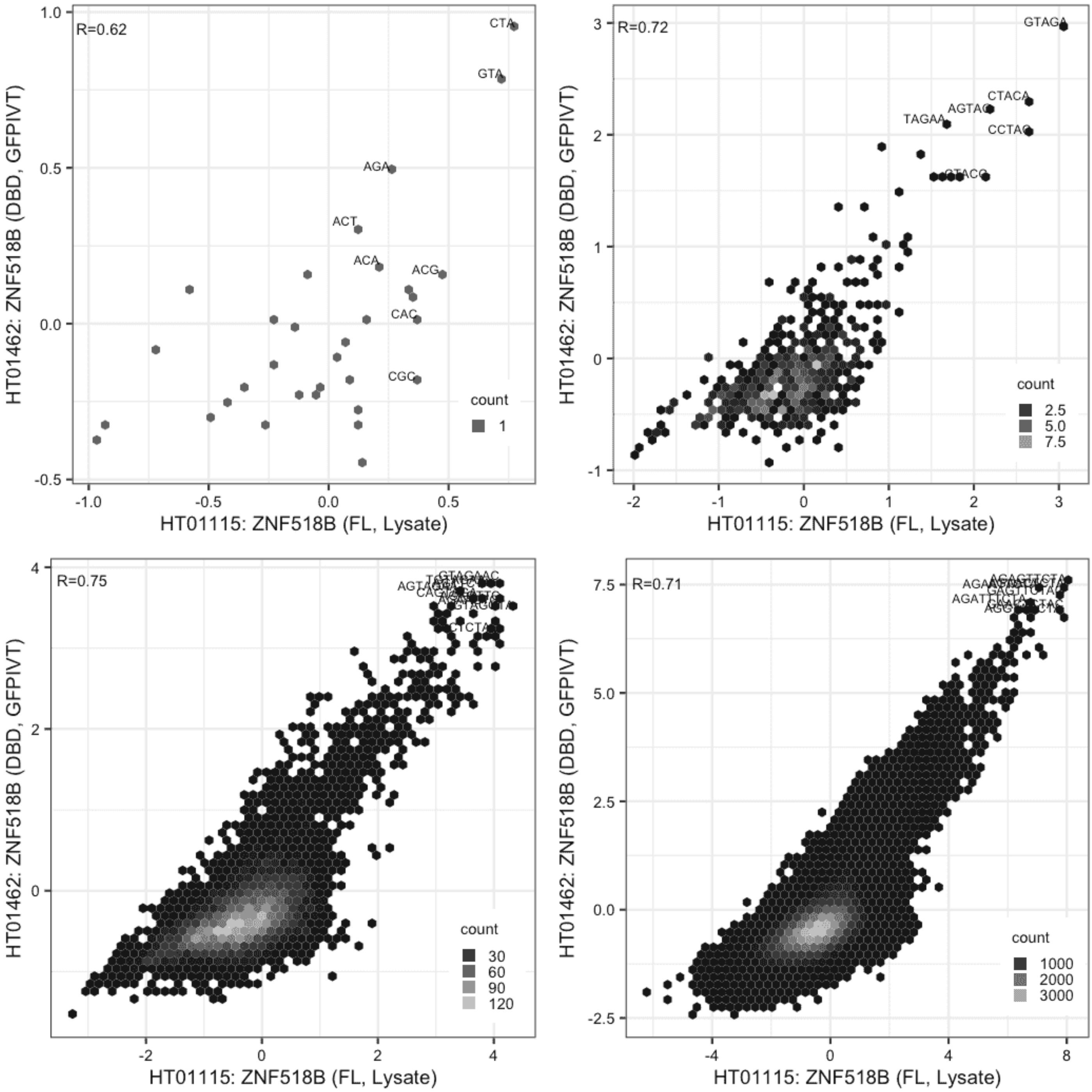

ZNF800

Lysate vs GFPIVT, different constructs

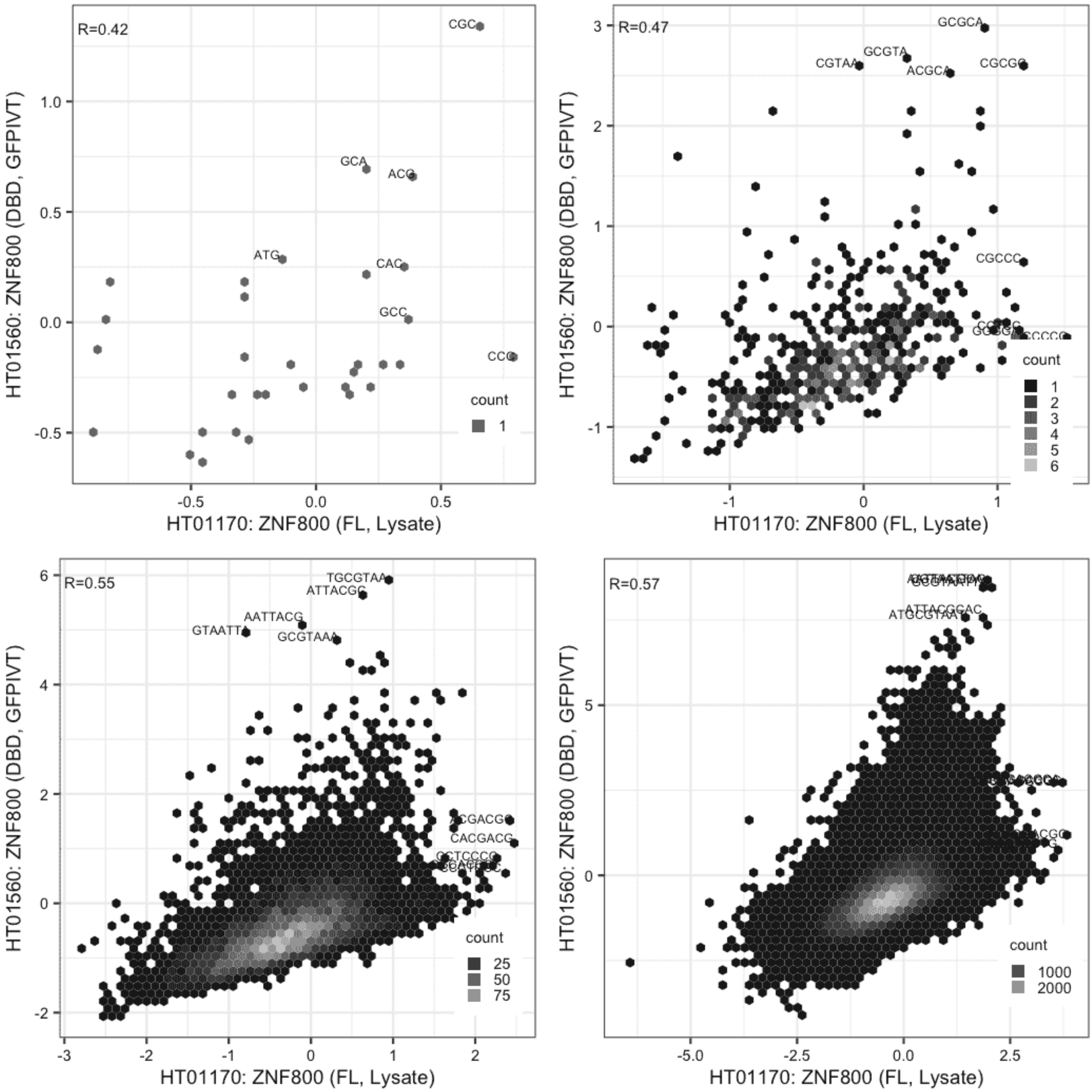

ZNF800

Lysate vs GFPIVT, same construct

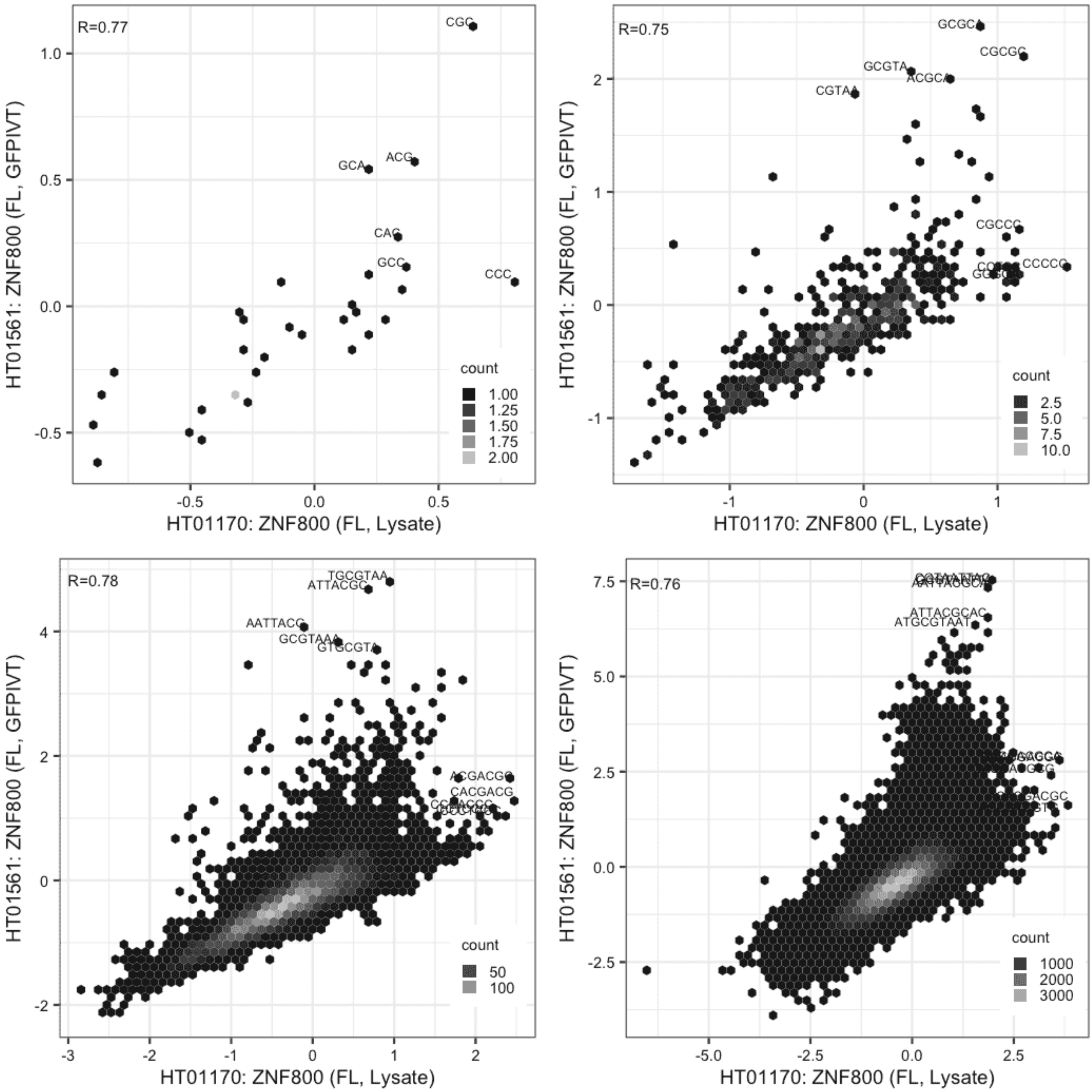

ZNF800

GFPIVT vs GFPIVT, different constructs

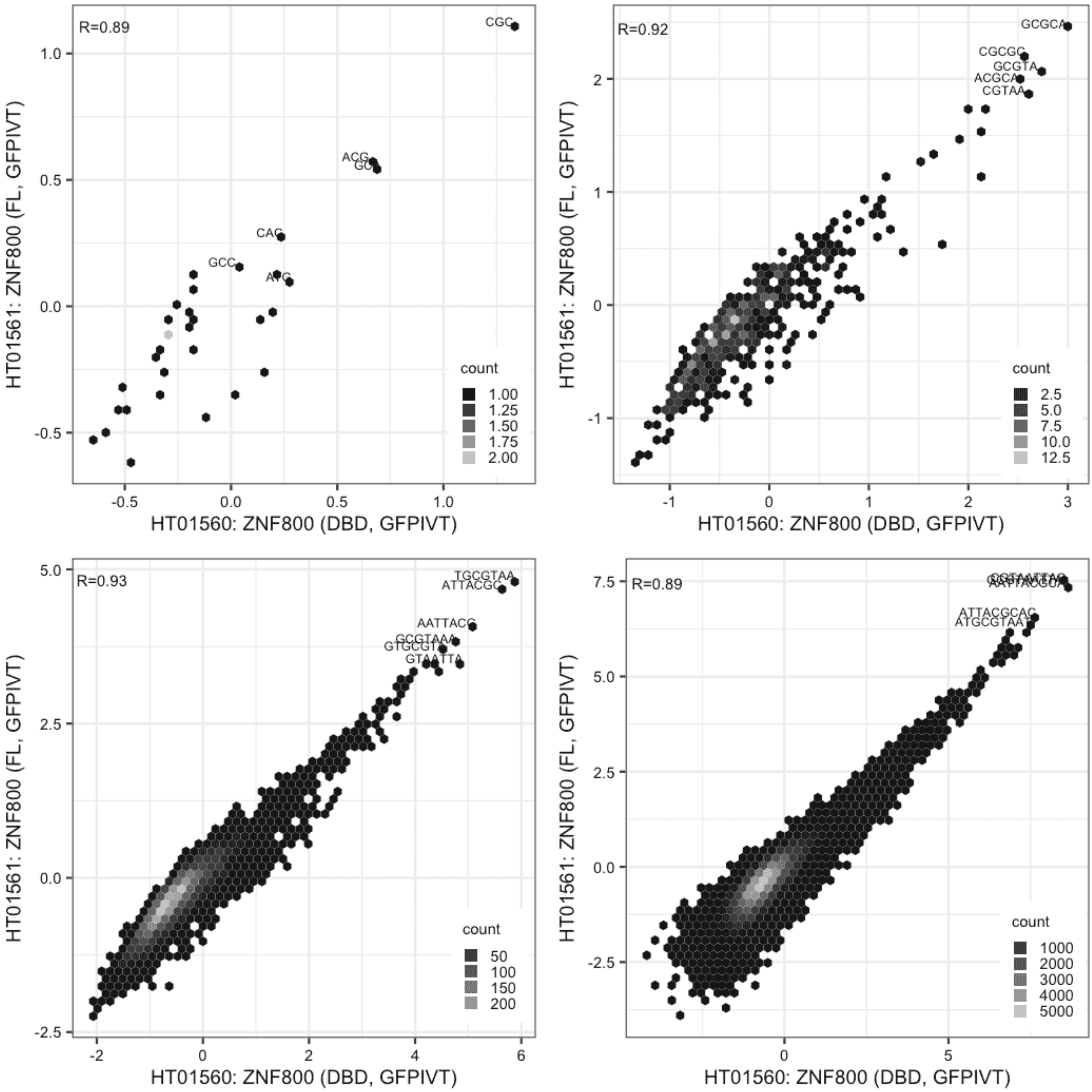

ZSCAN25

Lysate vs IVT, different constructs

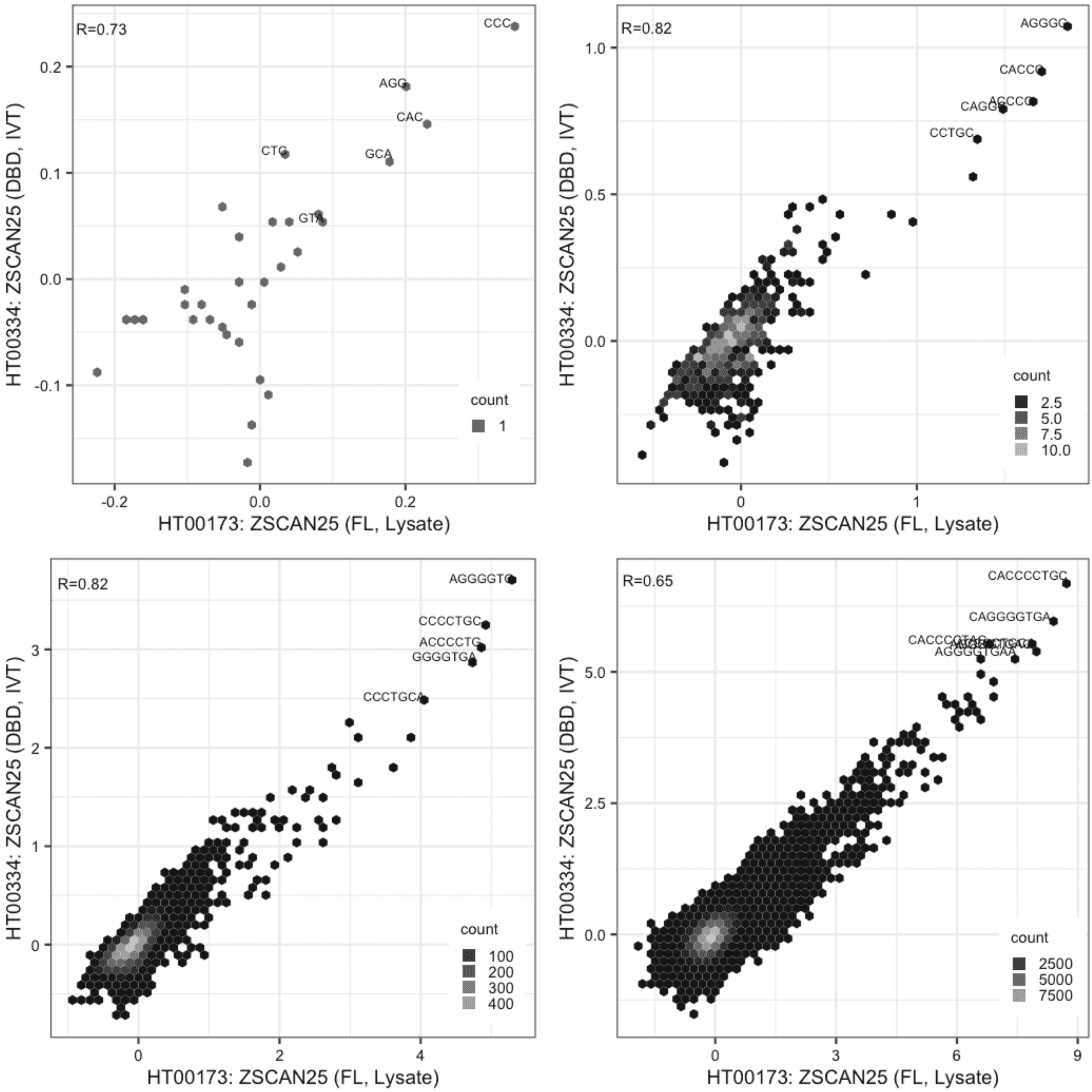

Supplement: Supplementary file 3 [file 41592_2026_3177_MOESM19_ESM.pdf]
