# Supplementary material for: GHT-SELEX demonstrates unexpectedly high intrinsic sequence specificity and complex DNA binding of many human transcription factors
Source: Nat Methods. 2026 Aug 5;23(9):1775–85. doi: 10.1038/s41592-026-03177-9 (PMC13541613; doi:10.1038/s41592-026-03177-9)

nature methods

Supplementary information https://doi.org/10.1038/s41592-026-03177-9

GHT-SELEX demonstrates unexpectedly high intrinsic sequence specificity and complex DNA binding of many human transcription factors

In the format provided by the authors and unedited

General description of the Supplementary Information 2
Supplementary Note 1. Considerations of effects of partial protein products on modular C2H2 binding patterns 2
Supplementary Figure 1. Summary of Western-blot analyses. 3
Supplementary Figure 2. All Western blot analyses 4

General description of the Supplementary Information

Additionally, to Supplementary tables and Figures, this article contains several large Supplementary Data files, containing either PWM target specificity models (Supplementary Data S1 and Supplementary Data S4) or provide large-scale outputs of the analyses, including reproducibility comparisons (Supplementary Data S2), per-transcription factor Jaccard and centrality analyses (Supplementary Data S3), and k-mer enrichment comparisons between HT-SELEX experiments (Supplementary Data S5).

Supplementary Note 1. Considerations of effects of partial protein products on modular C2H2 binding patterns

Protein production methods can generate partial proteins in some cases, and these can potentially impact modular binding results observed in GHT-SELEX. However, this should have very limited impact. For proteins with a single DNA-binding domain, a partial domain would likely be misfolded and/or not functional for DNA-binding, and thus inert in DNA-binding assays. For C2H2-zf proteins, truncations should lead to a bias towards binding sites that engage the C2H2-zf domains towards the N-terminus. Such a bias would lead to greater signal towards the left side in RCADEEM plots.
We assessed RCADEEM outputs for this type of an effect, and only three TFs, ZNF233, ZNF497 and ZNF853 displayed this type of modularity, However, none of these TFs, nor any of the modular binding TFs displayed in **Fig 6** showed relevant amounts of partial fragments in Western blots.

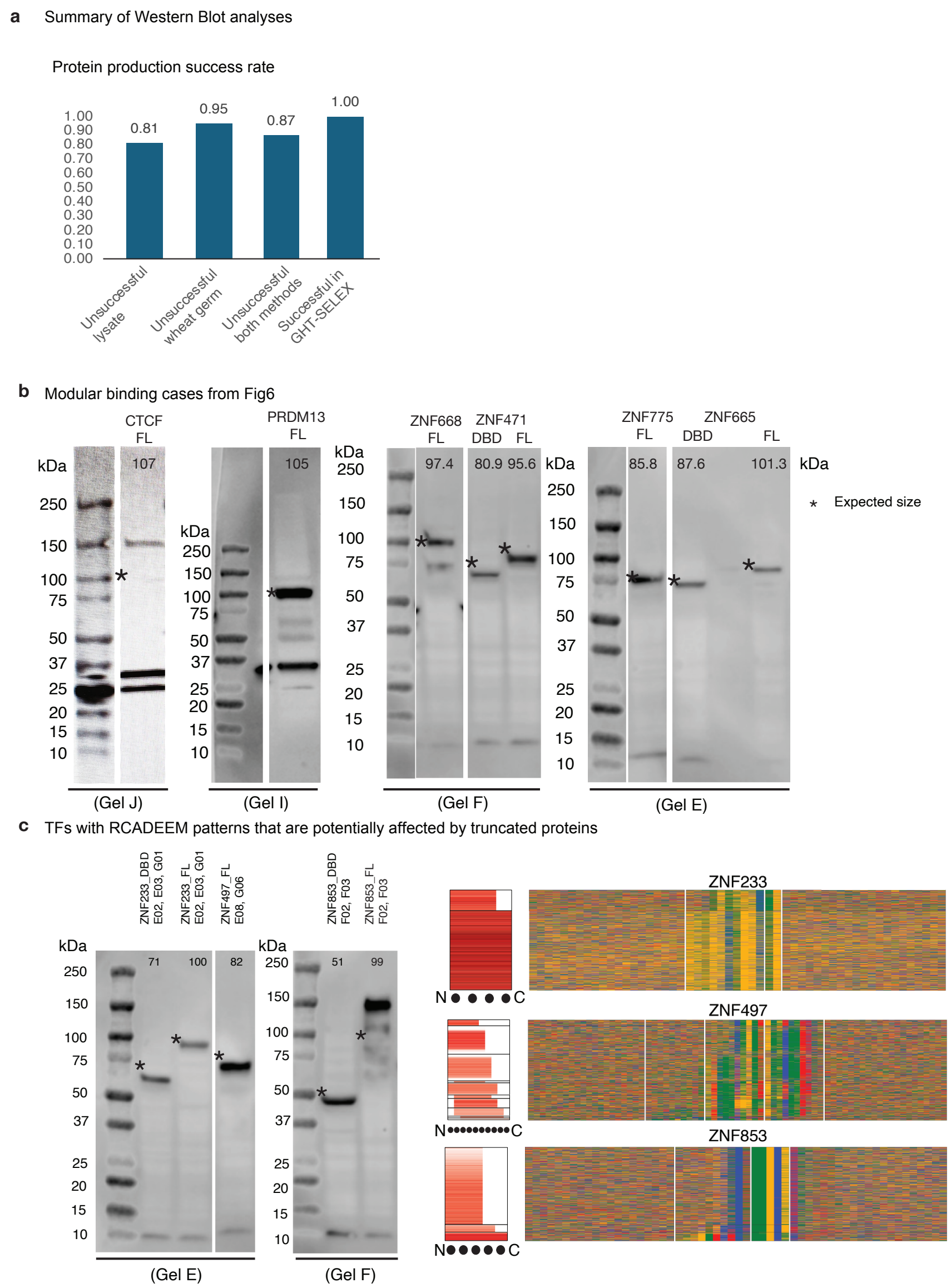

Supplementary Figure 1. Summary of Western-blot analyses.

a. Summary of blot results. **b.** Modular C2H2-zfs shown in Fig6. **c.** C2H2-zfs ZNF233, ZNF497 andZNF853, show sites in RCADEEM analysis that could have been affected by C-terminal truncations. Note, only ZNF853 show smaller than expected size fragments.

Supplementary Figure 2. All Western blot analyses

(**Panels a-l, each on an individual page**) Each Figure panel displays an annotated blot *(top)* and corresponding original gel image *(bottom)*. Wells have been annotated to display following information: HNGC symbol of the TF; its expression construct; gel and well references for all positions with the same TF; Whether the expression was deemed successful (black text) or unsuccessful(red text); If this protein expression yielded a successful experiment in GHT-SELEX (green bar) or not (red bar); If the TF is a C2H2 protein (black bar on top); If the C2H2 TF displayed modular binding specificity (blue bar) and the expected size of the fusion protein (asterisk over the blot). See **Table S7**, for detailed assessment of these blots.

Supplementary Fig. 2 (total of ten panels)

a (gel A)

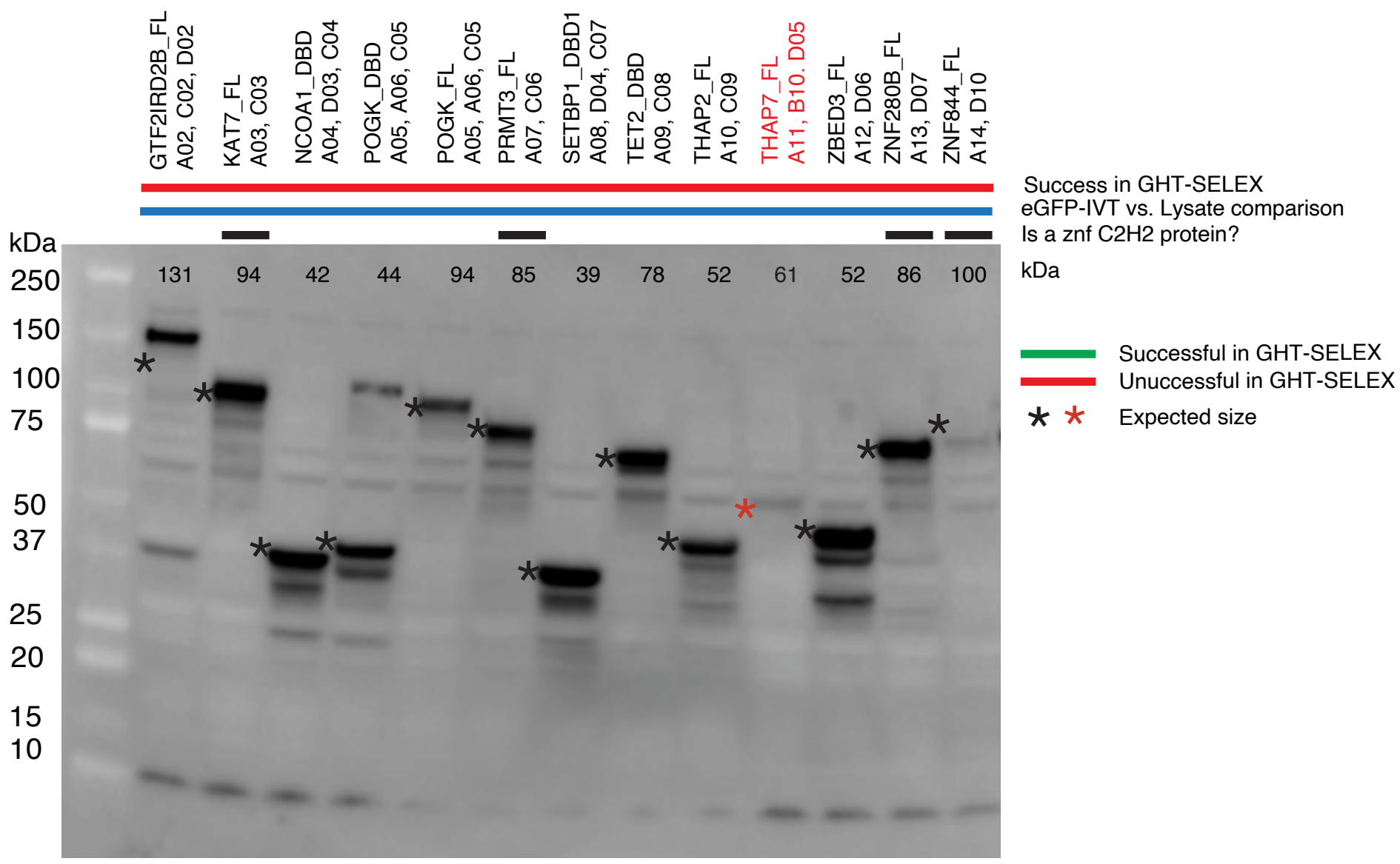

Unsuccessful eGFP-IVT

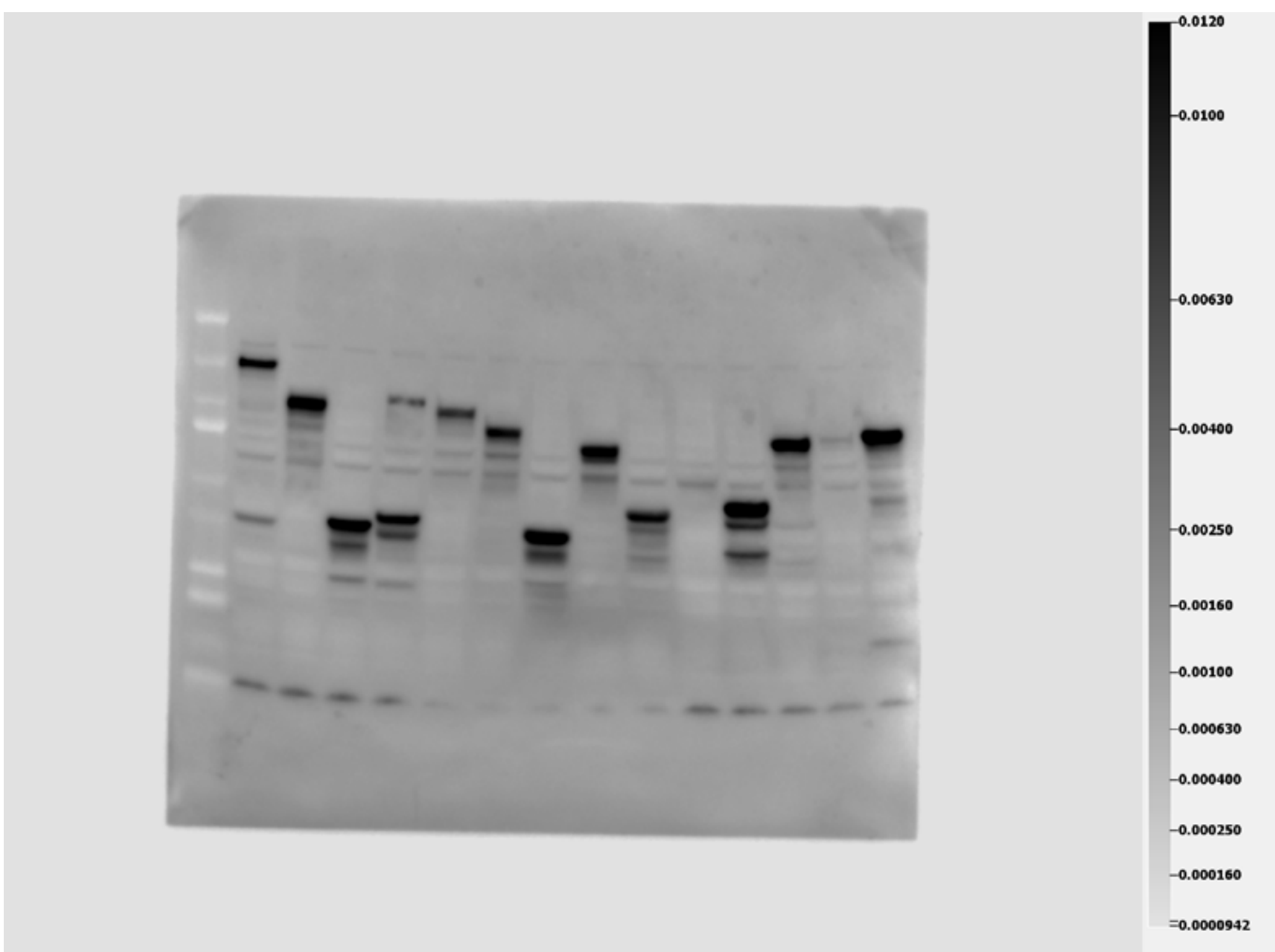

Supplementary Fig. 2 (Continued)

b (gel B)

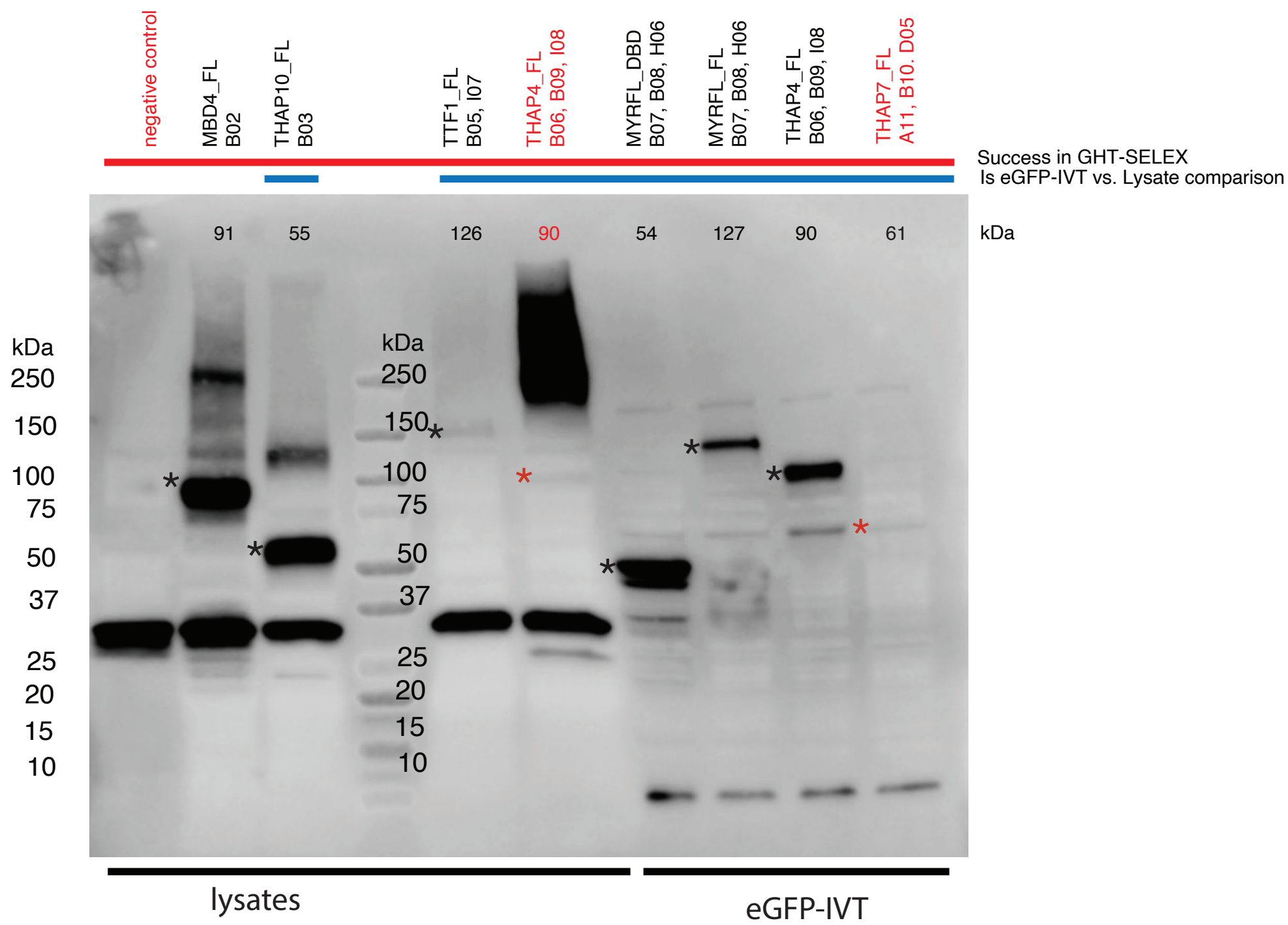

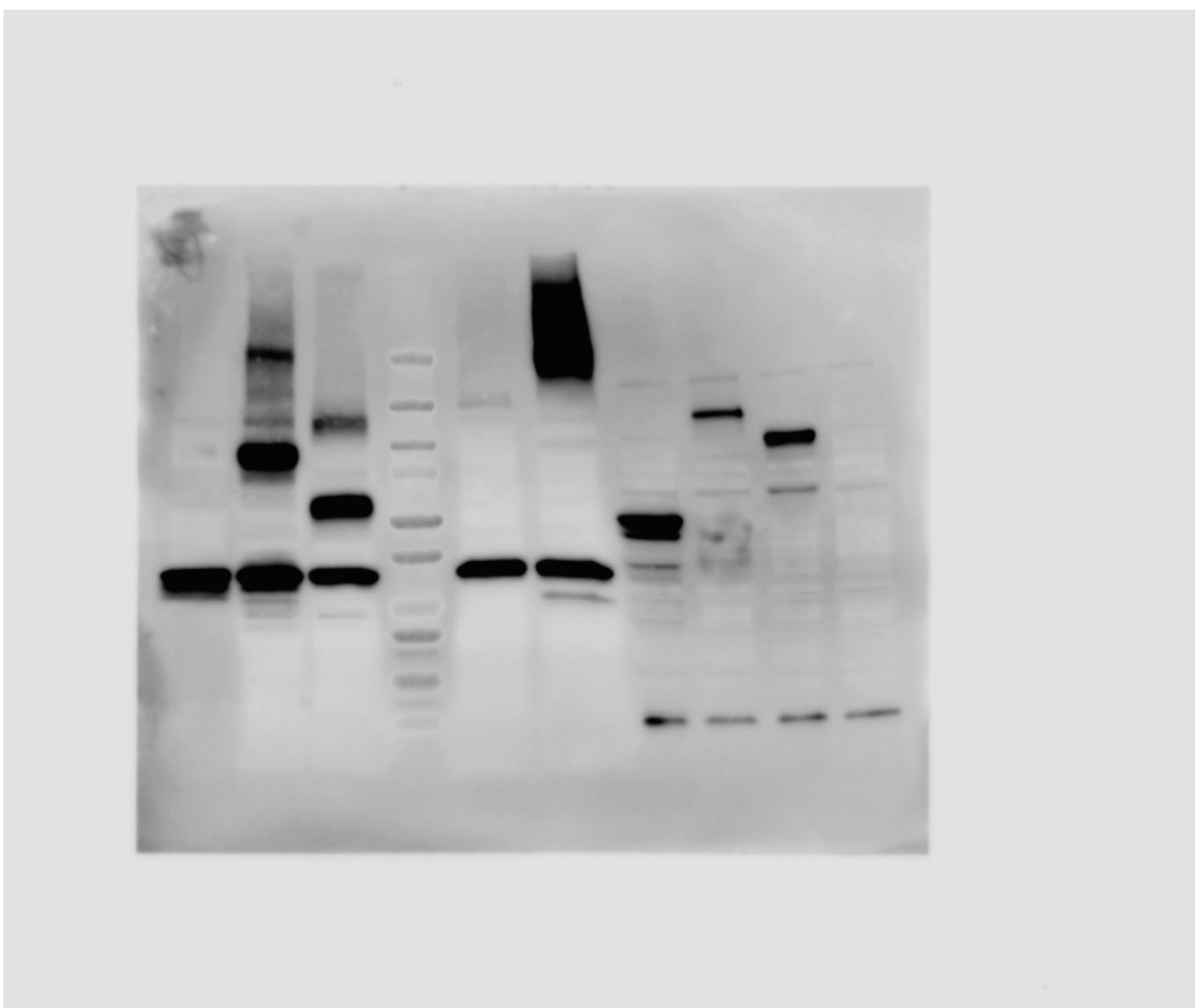

Supplementary Fig. 2 (Continued)

c (Gel C)

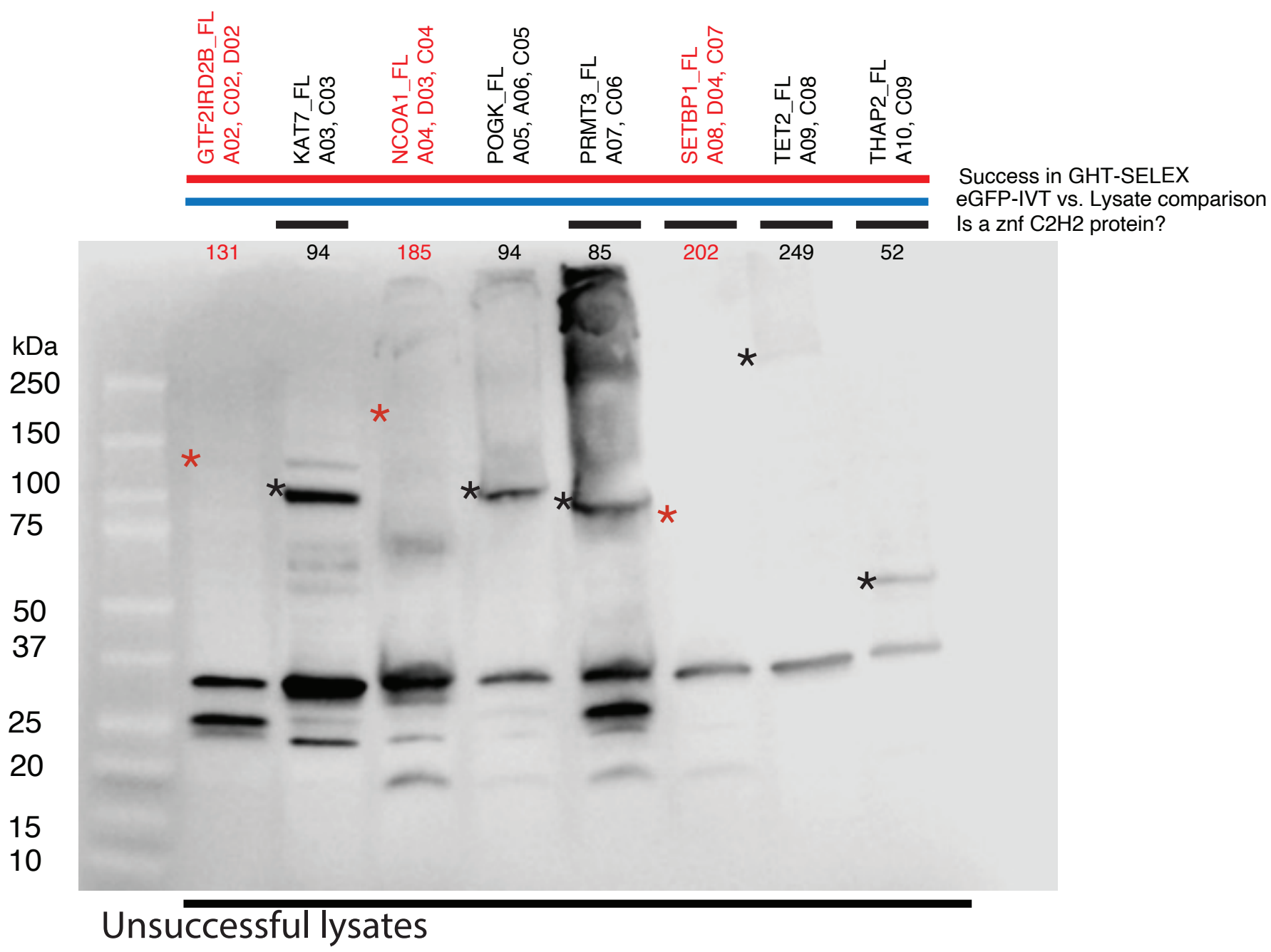

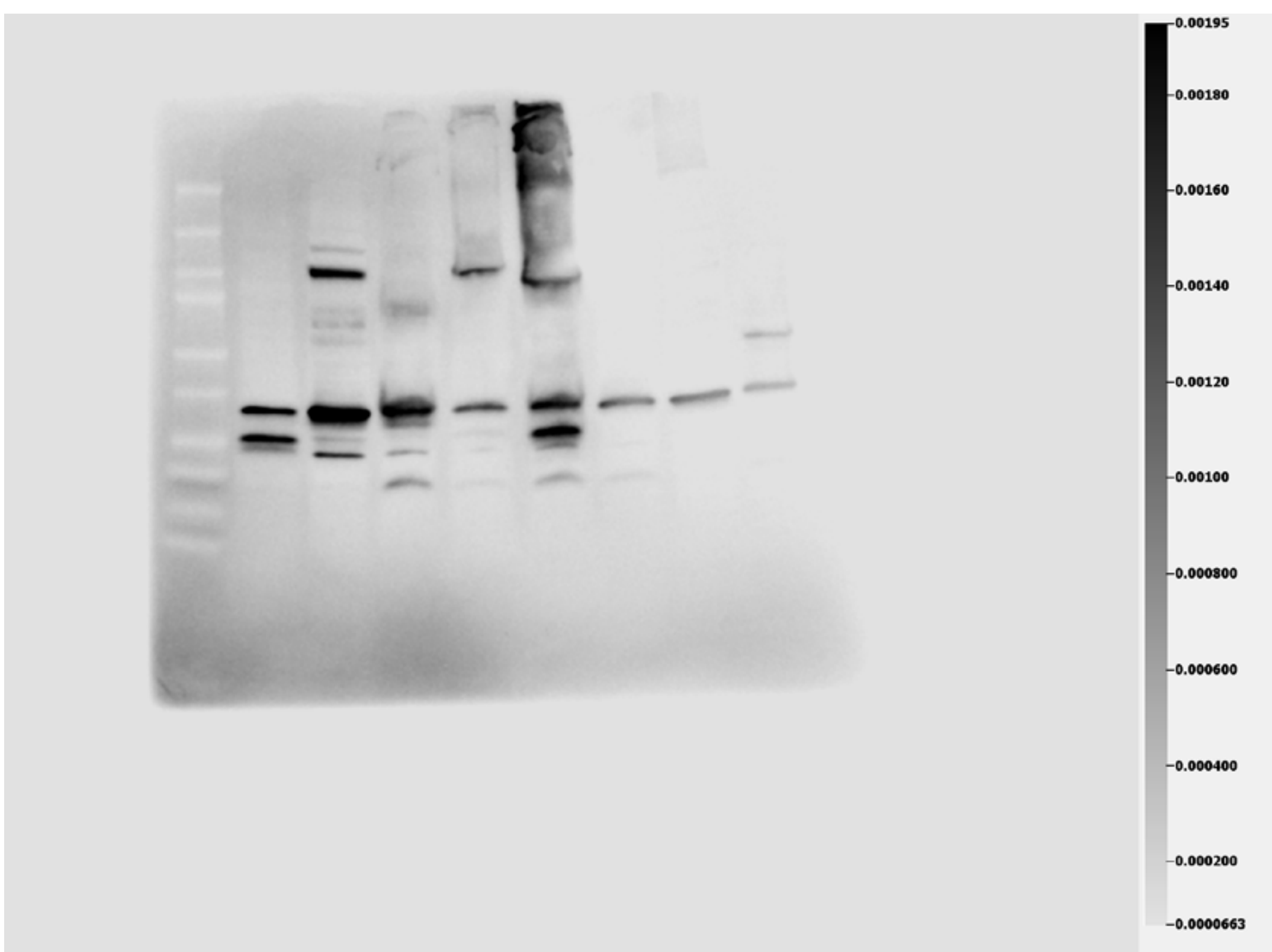

Supplementary Fig. 2 (Continued)

d (Gel D)

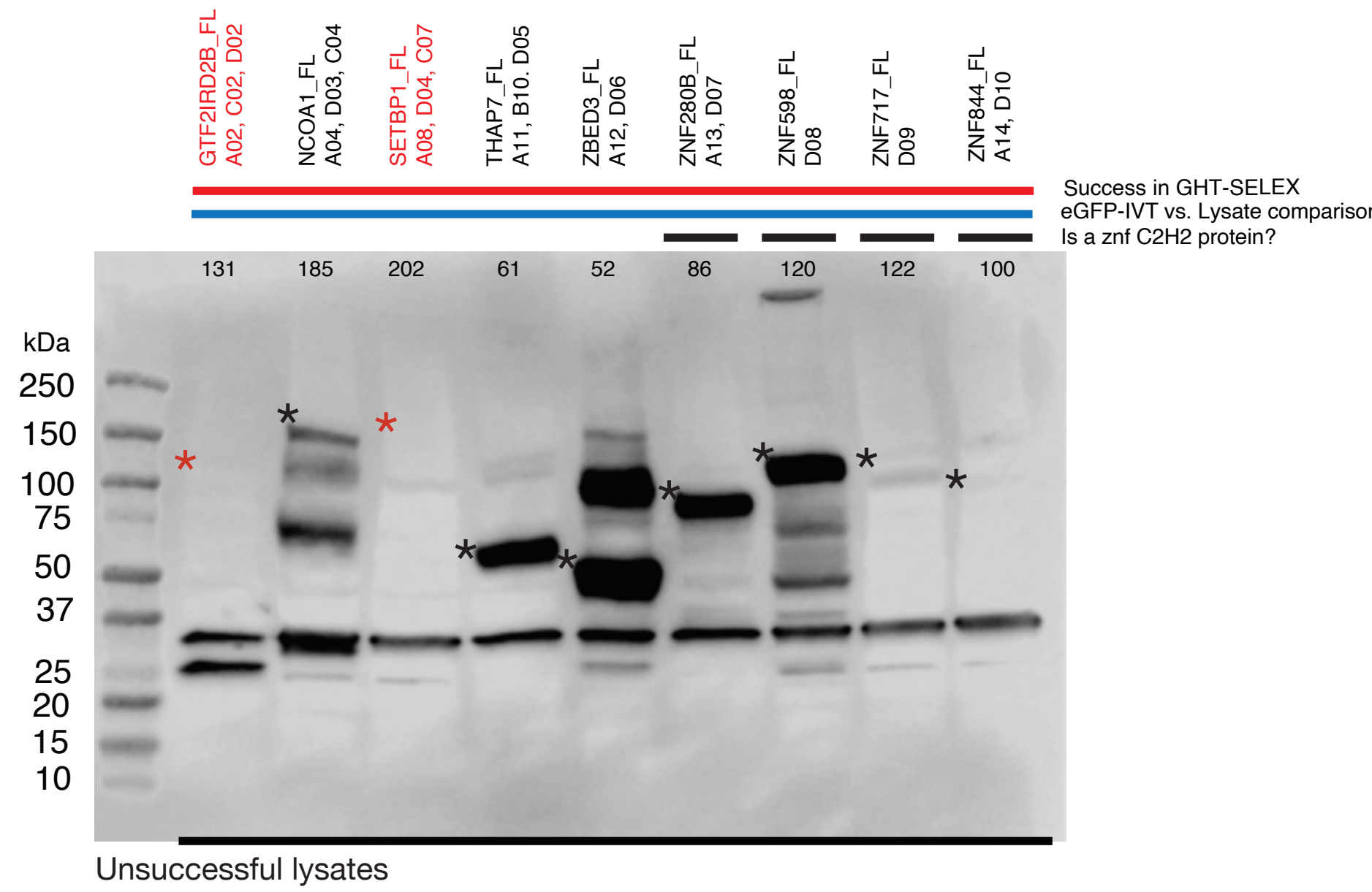

Unucessful in GHT-SELEX
Expected size

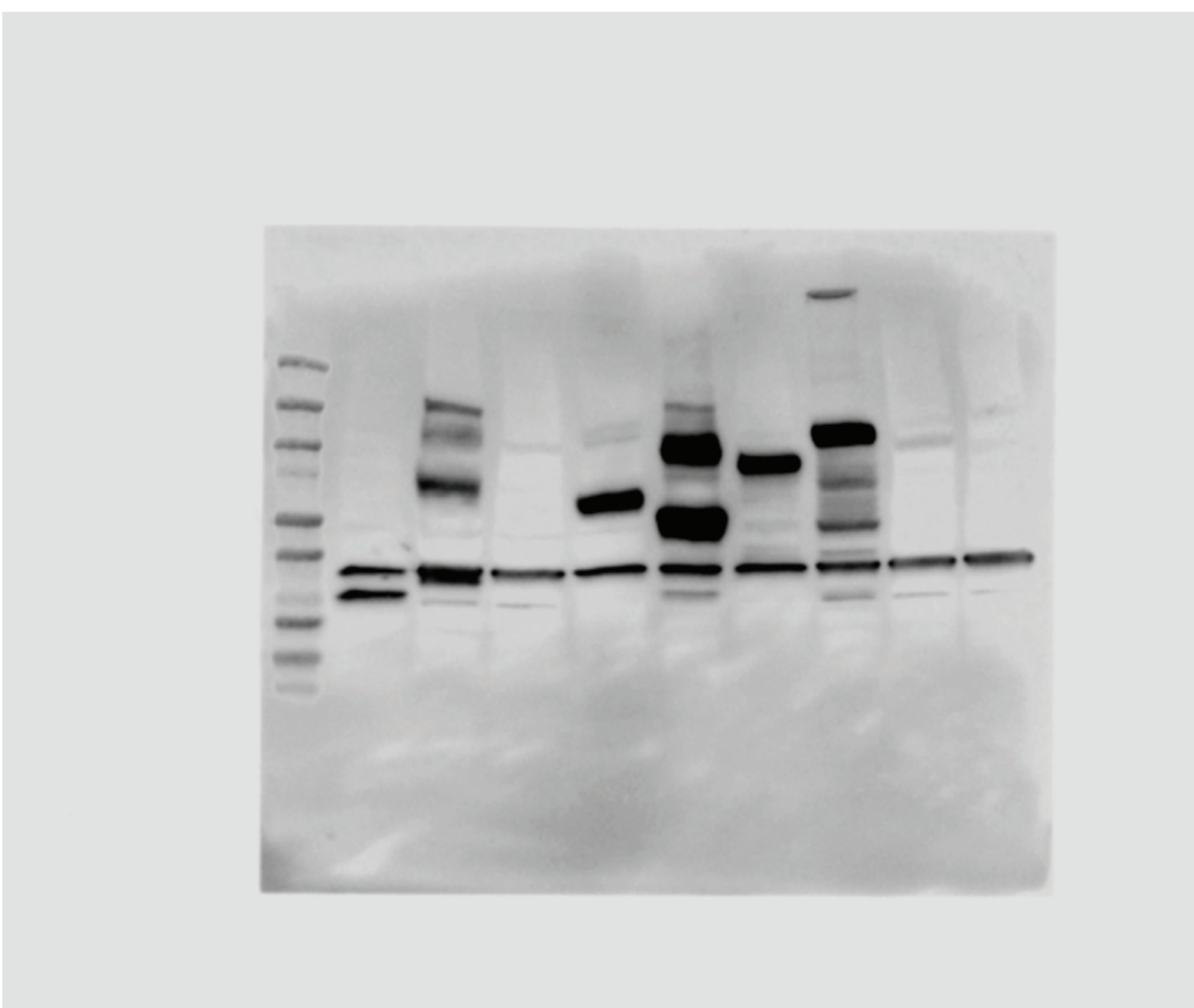

Supplementary Fig. 2 (Continued)

e (Gel E)

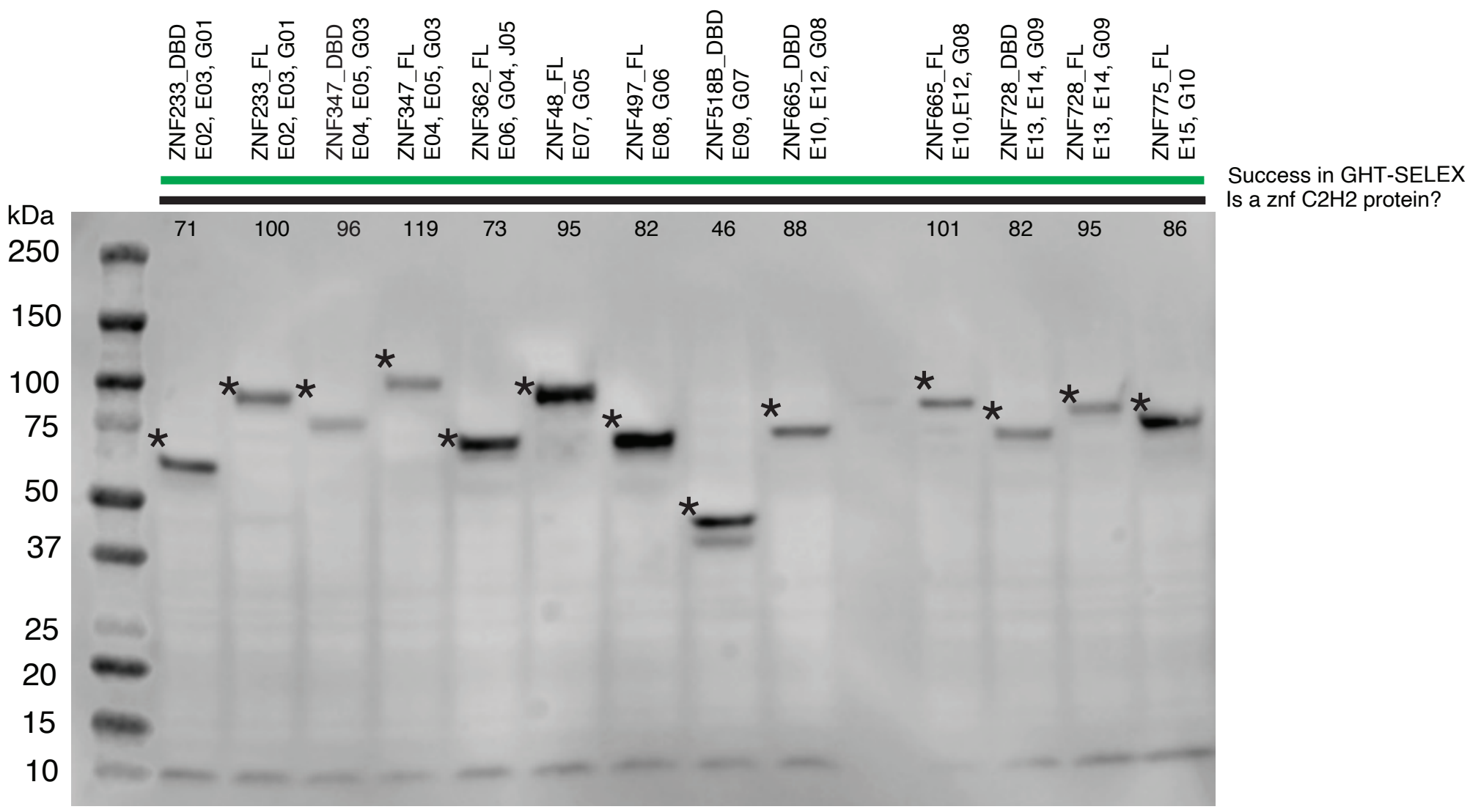

Successful eGFP-IVT

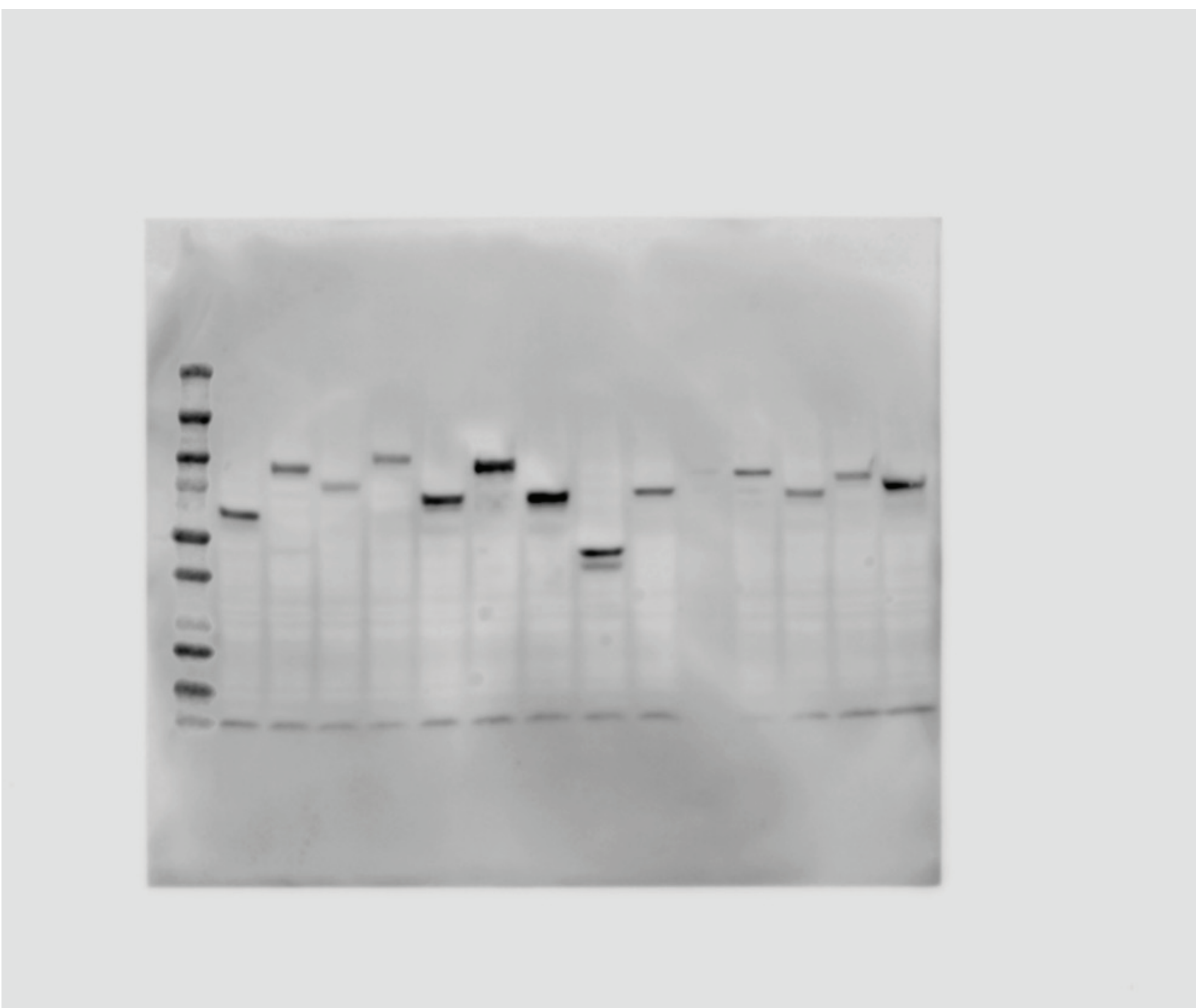

Supplementary Fig. 2 (Continued)

f (Gel F)

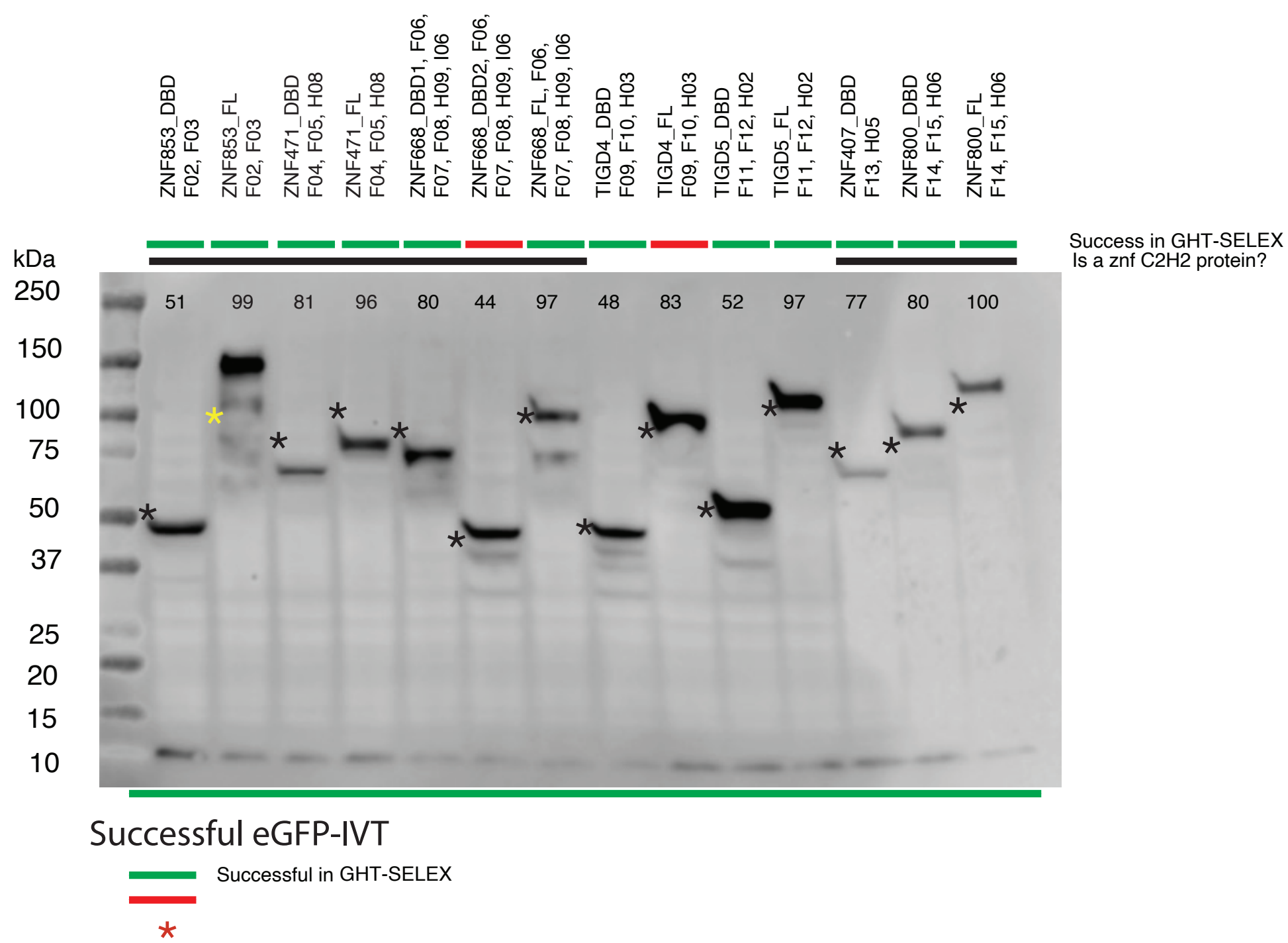

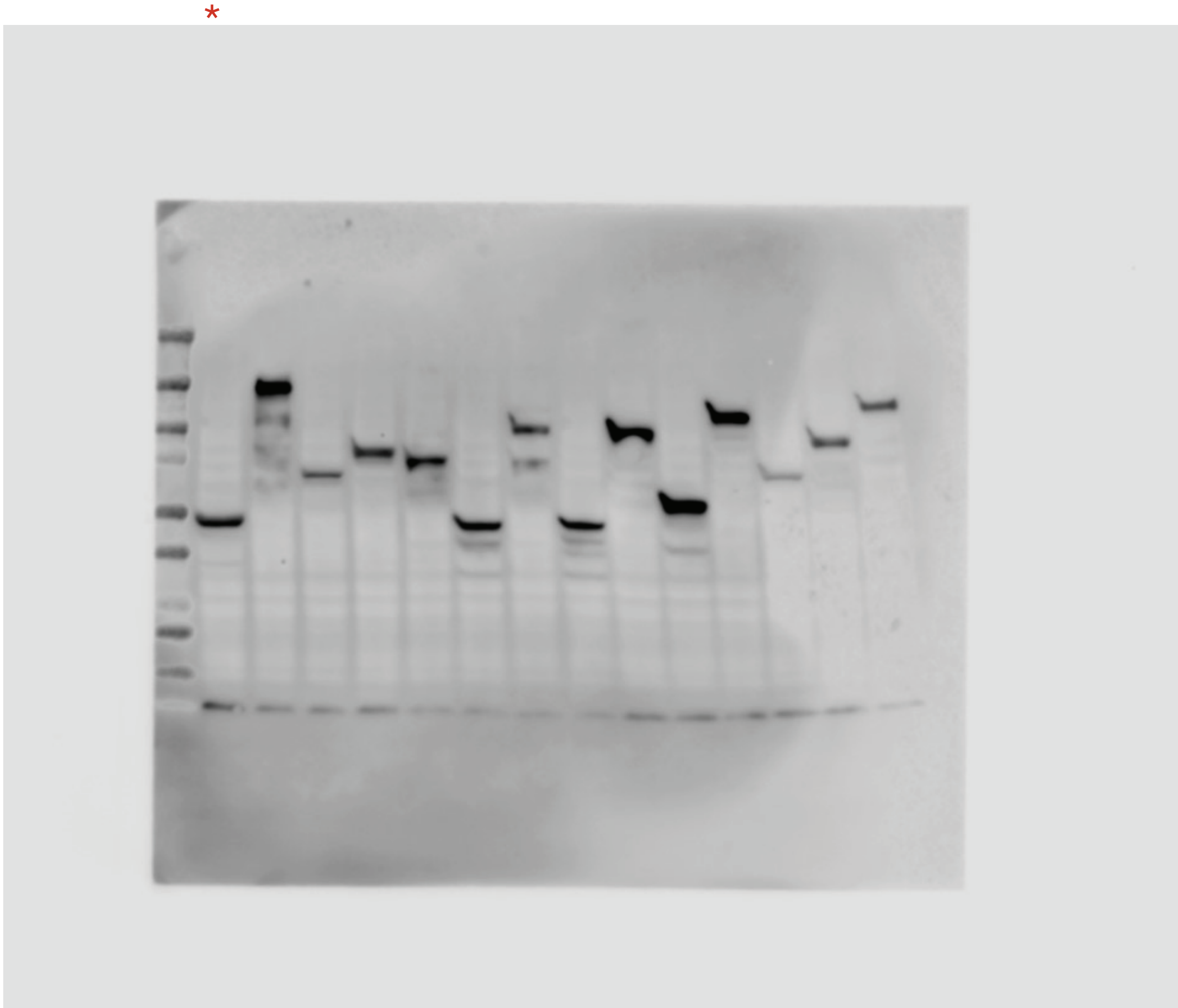

Supplementary Fig. 2 (Continued)

g (Gel G)

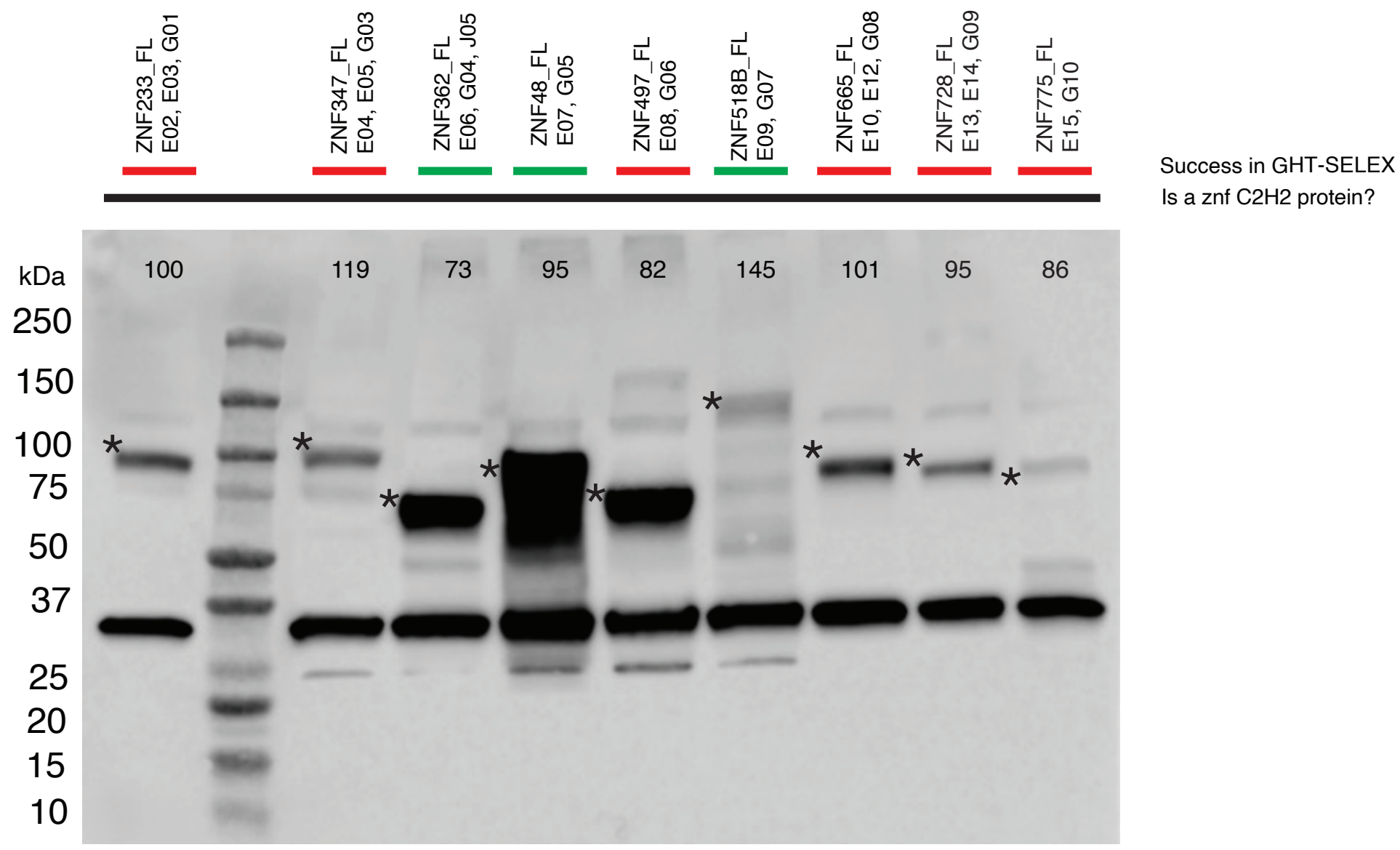

Mixed success Lysate

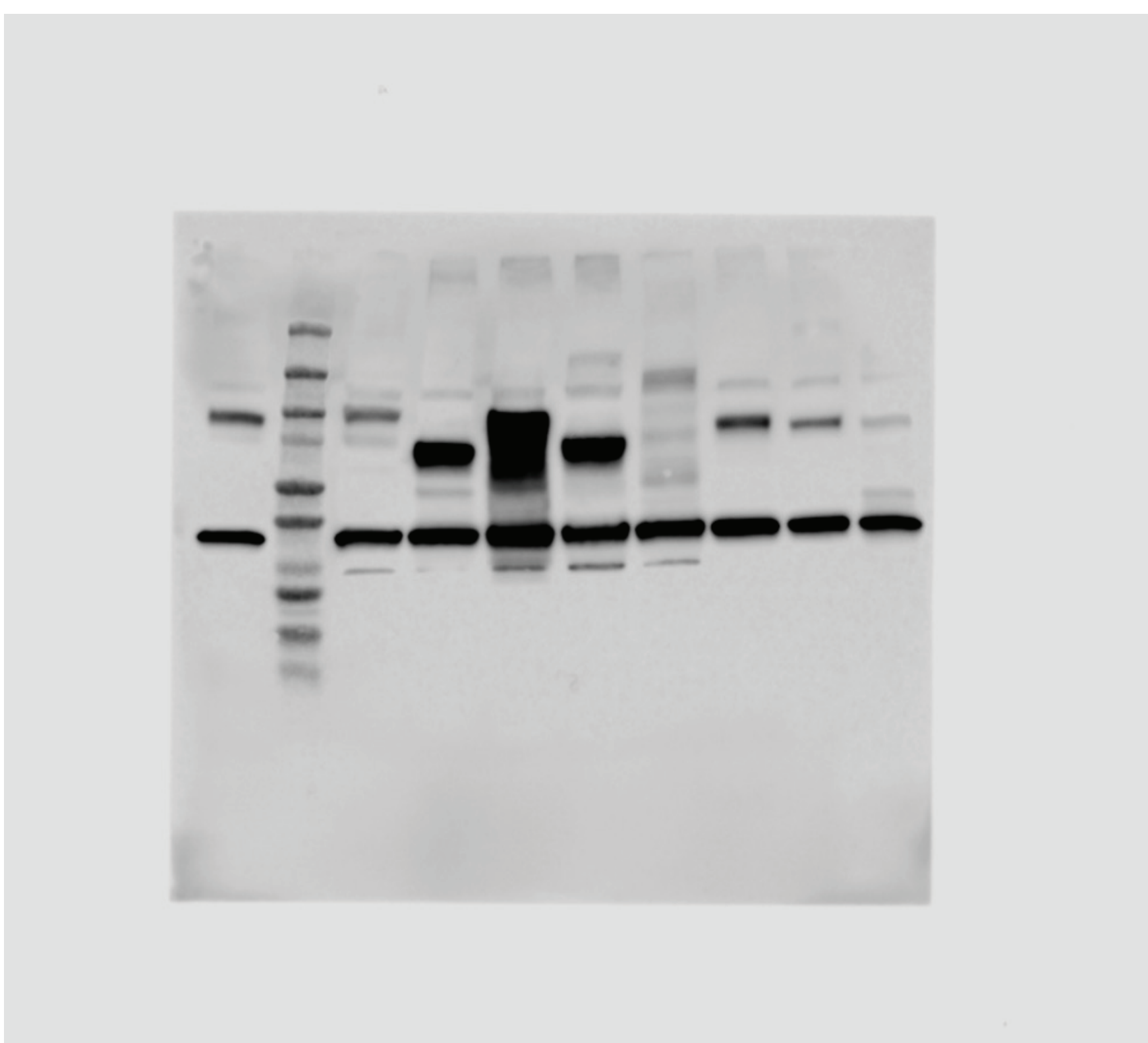

Supplementary Fig. 2 (Continued)

h (Gel H)

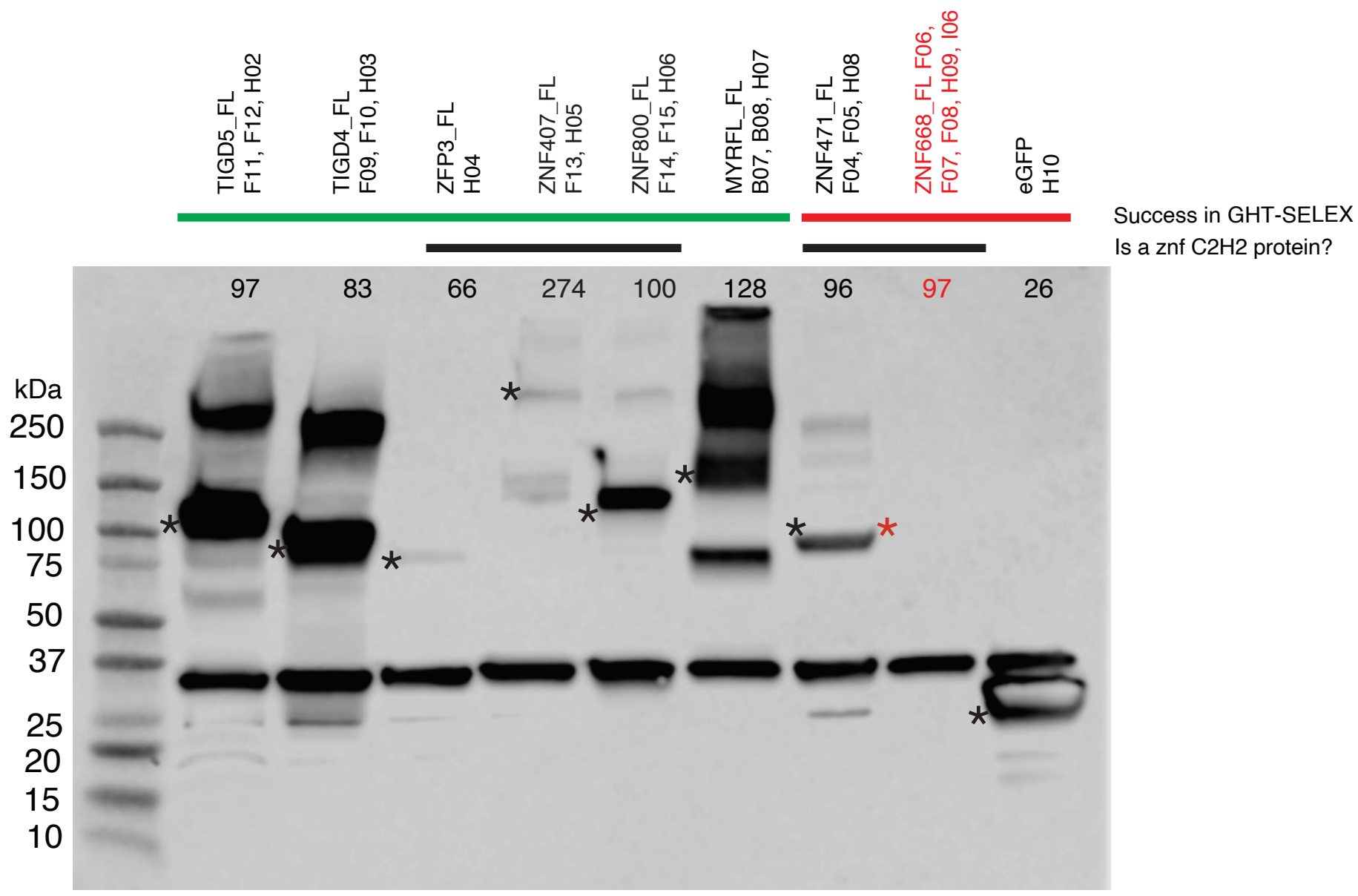

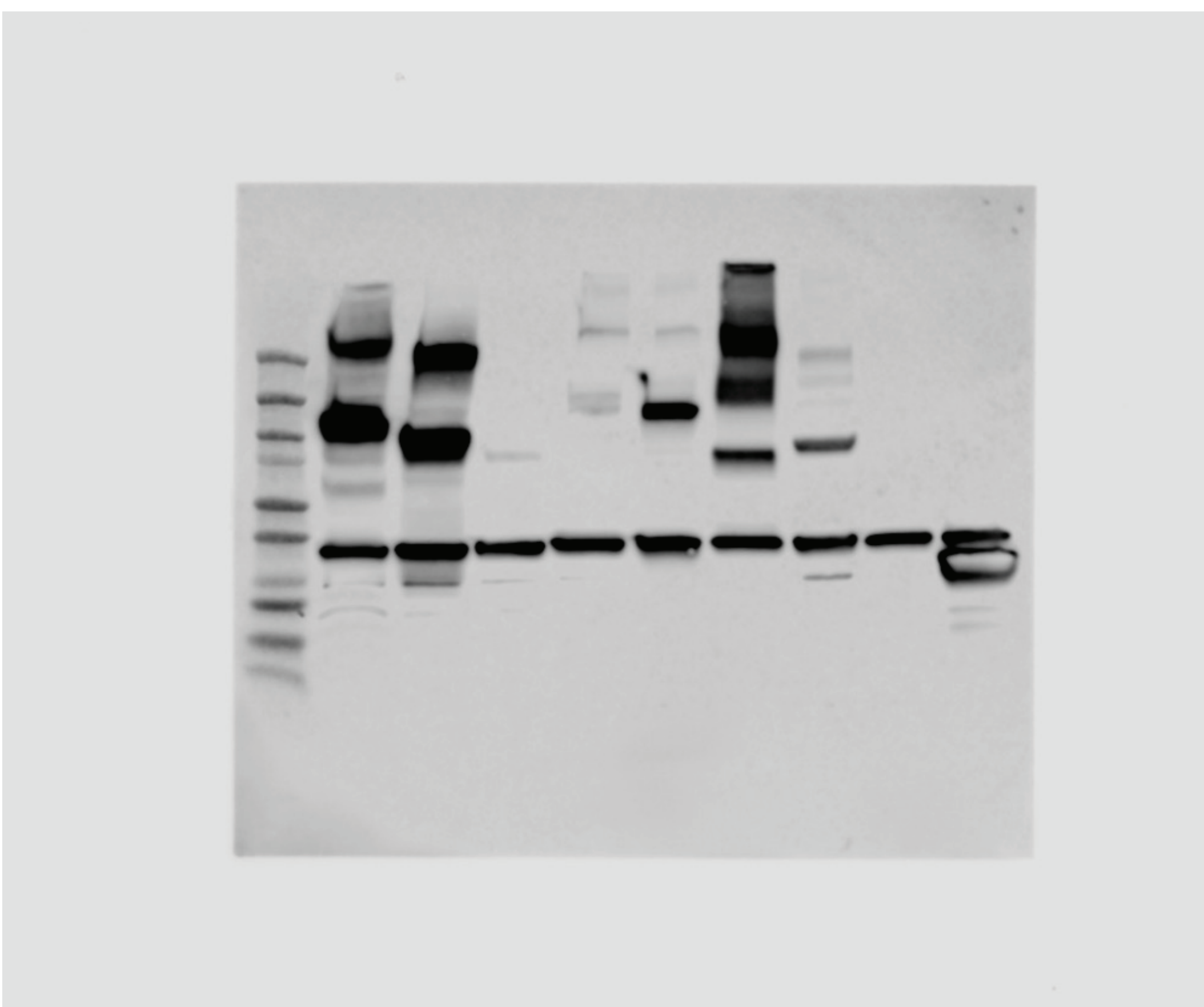

Supplementary Fig. 2 (Continued)

i (Gel I)

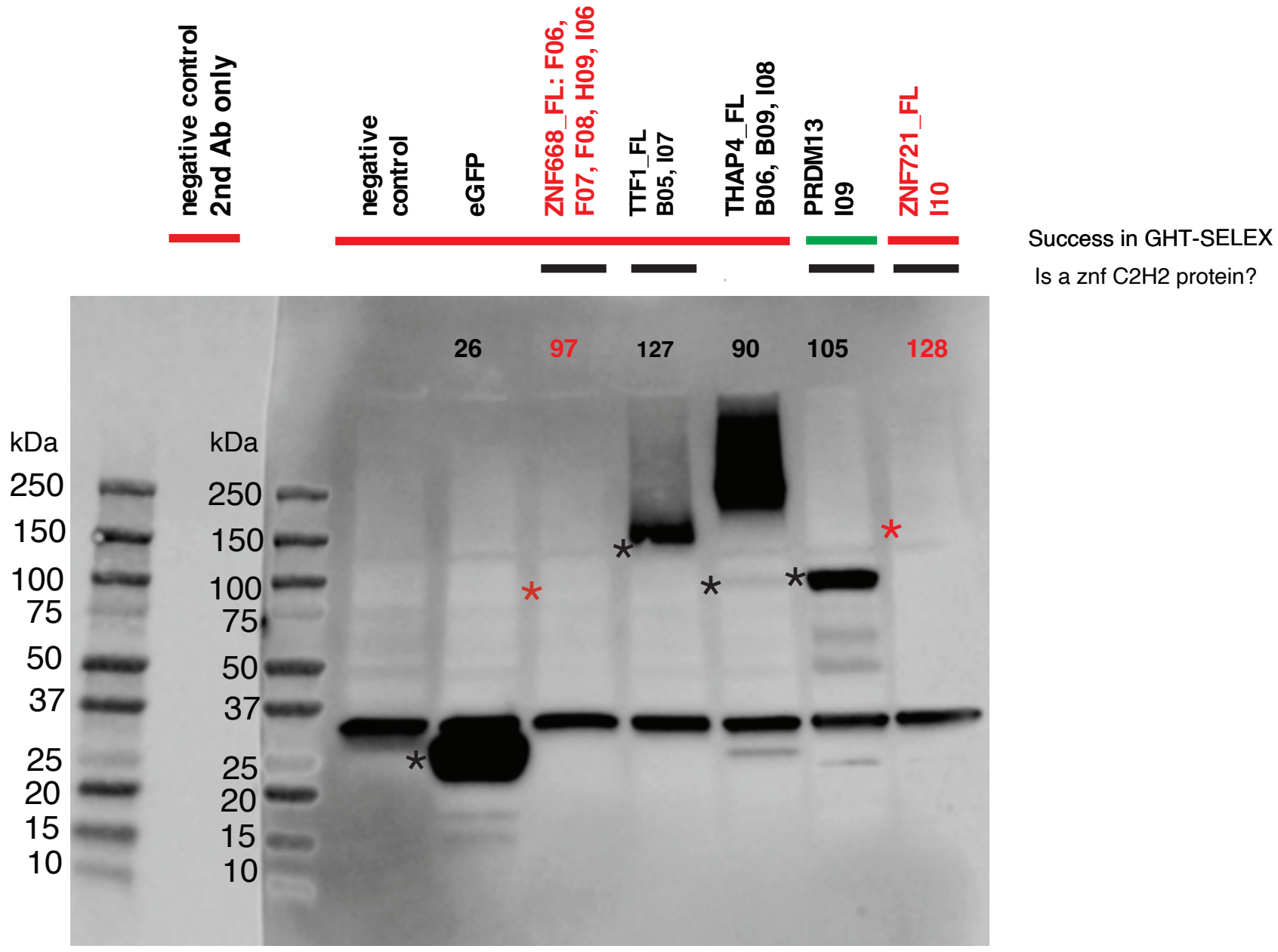

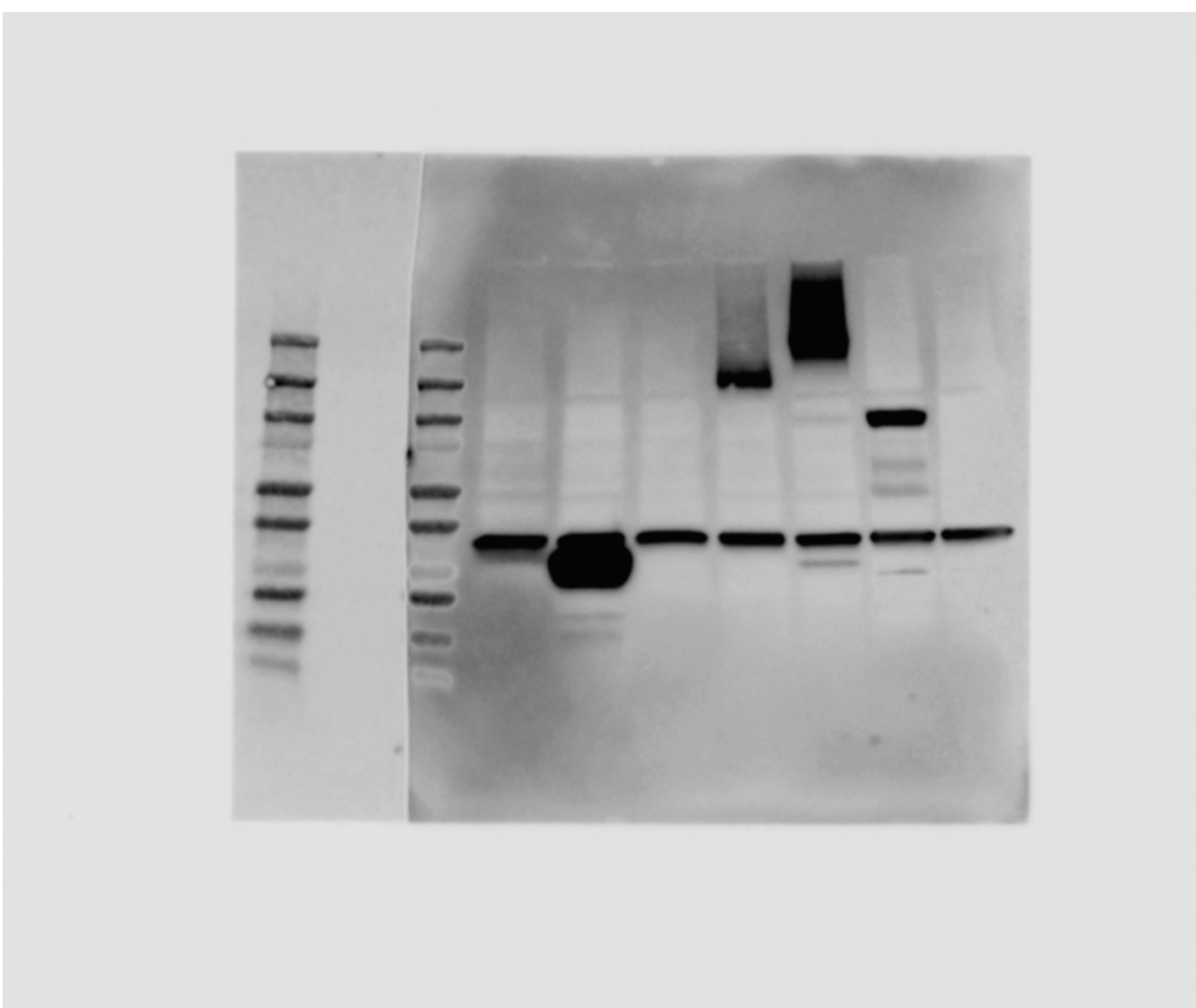

Supplementary Fig. 2 (Continued)

j (Gel J)

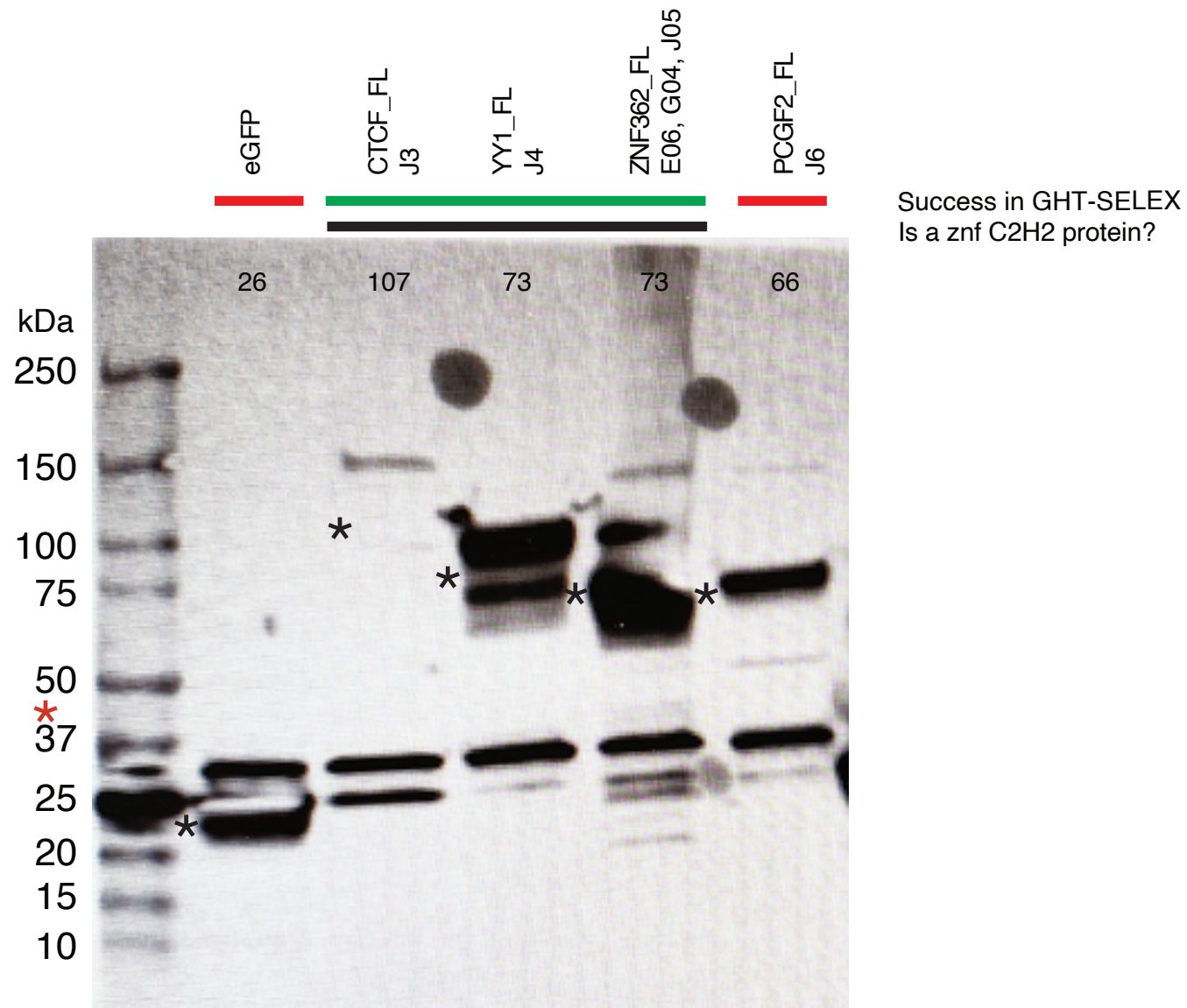

Positive controls and GHT-SELEX eGFP Lysate samples testing

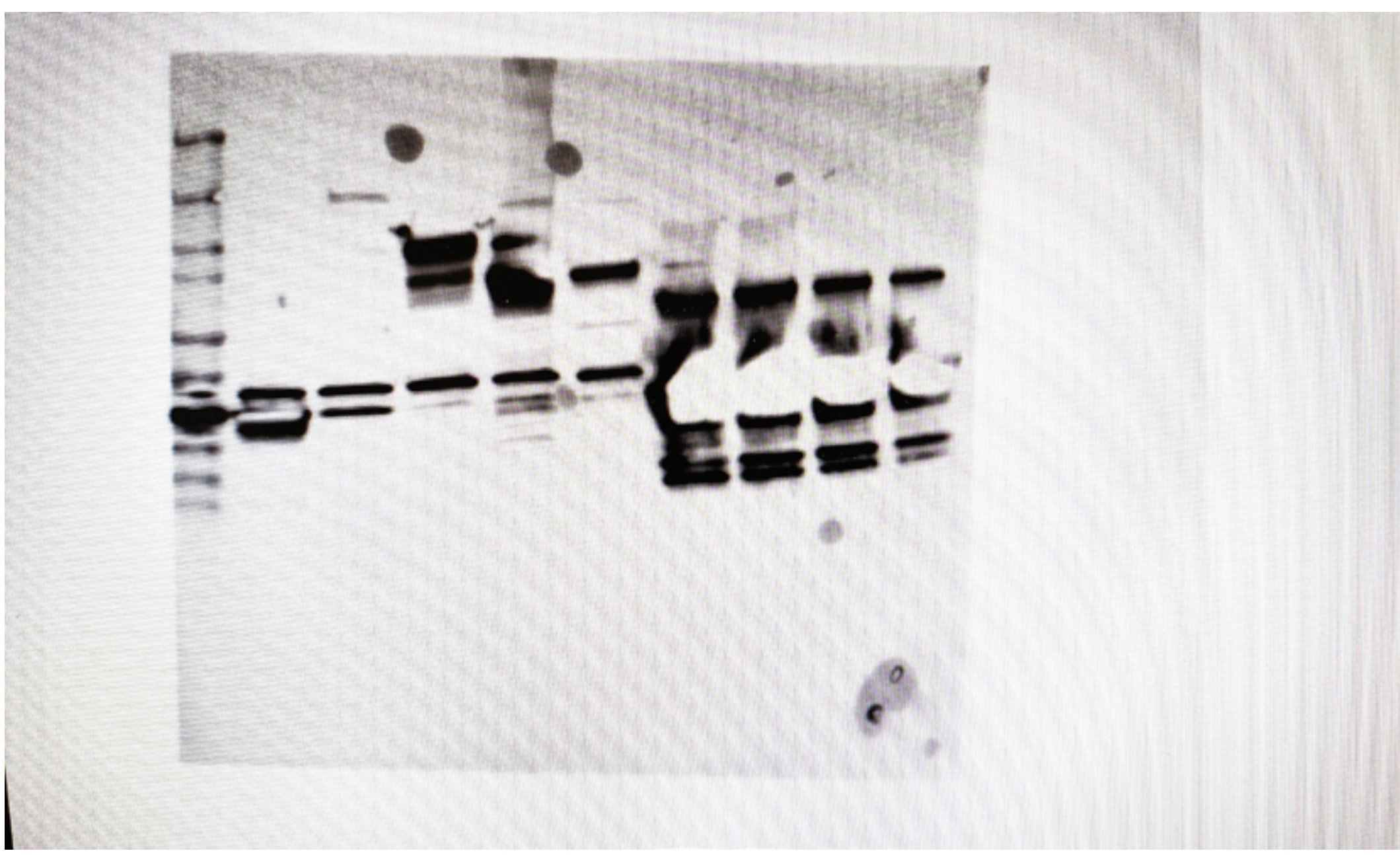

Supplement: Supplementary file 4 [file 41592_2026_3177_MOESM1_ESM.pdf]
