# Supplementary material for: GHT-SELEX demonstrates unexpectedly high intrinsic sequence specificity and complex DNA binding of many human transcription factors
Source: Nat Methods. 2026 Aug 5;23(9):1775–85. doi: 10.1038/s41592-026-03177-9 (PMC13541613; doi:10.1038/s41592-026-03177-9)
Supplement: Supplementary file 5 [file 41592_2026_3177_MOESM2_ESM.pdf]

Corresponding author(s): Arttu Jolma
Timothy R. Hughes
Hamed S. Najafabadi

Last updated by author(s): Apr 6, 2026

Reporting Summary

Nature Portfolio wishes to improve the reproducibility of the work that we publish. This form provides structure for consistency and transparency in reporting. For further information on Nature Portfolio policies, see our Editorial Policies and the Editorial Policy Checklist.

Statistics

For all statistical analyses, confirm that the following items are present in the figure legend, table legend, main text, or Methods section.

n/a	Confirmed	
☐	☒	The exact sample size (n) for each experimental group/condition, given as a discrete number and unit of measurement
☐	☒	A statement on whether measurements were taken from distinct samples or whether the same sample was measured repeatedly
☐	☒	The statistical test(s) used AND whether they are one- or two-sided<br>*Only common tests should be described solely by name; describe more complex techniques in the Methods section.*
☐	☒	A description of all covariates tested
☐	☒	A description of any assumptions or corrections, such as tests of normality and adjustment for multiple comparisons
☐	☒	A full description of the statistical parameters including central tendency (e.g. means) or other basic estimates (e.g. regression coefficient) AND variation (e.g. standard deviation) or associated estimates of uncertainty (e.g. confidence intervals)
☐	☒	For null hypothesis testing, the test statistic (e.g. F, t, r) with confidence intervals, effect sizes, degrees of freedom and P value noted<br>*Give P values as exact values whenever suitable.*
☐	☒	For Bayesian analysis, information on the choice of priors and Markov chain Monte Carlo settings
☐	☒	For hierarchical and complex designs, identification of the appropriate level for tests and full reporting of outcomes
☐	☒	Estimates of effect sizes (e.g. Cohen's d, Pearson's r), indicating how they were calculated

Our web collection on statistics for biologists contains articles on many of the points above.

Software and code

Policy information about availability of computer code

Data collection	Software was not used in data collection.
Data analysis	Trimmomatic (v0.39) was used to remove constant flanks from HT- and GHT-SELEX sequencing reads.<br>Reads were aligned using Bowtie2 (v2.4.1)<br>BEDTools (v2.30.0) was used for various analyses and calculations.<br>Position weight matrices (PWMs) were identified using numerous tools, described in an accompanying manuscript (Voronstov et al 2025. doi: 10.1038/s42003-025-08909-9.).<br>PWM scanning was performed with MOODS (v1.9.4). kMers were counted with Jellyfish (v 2.3.1) ,<br>kmers were analyzed and plotted with R-package Biostrings (v 4.2.1)<br>GHT-SELEX peaks were called using MAGIX: https://github.com/csglab/MAGIX<br>C2H2 DBD modularity analysis was performed with RCADEEM, https://github.com/csglab/RCADEEM).

For manuscripts utilizing custom algorithms or software that are central to the research but not yet described in published literature, software must be made available to editors and reviewers. We strongly encourage code deposition in a community repository (e.g. GitHub). See the Nature Portfolio guidelines for submitting code & software for further information.

Data

Policy information about availability of data

All manuscripts must include a data availability statement. This statement should provide the following information, where applicable:

- Accession codes, unique identifiers, or web links for publicly available datasets
- A description of any restrictions on data availability
- For clinical datasets or third party data, please ensure that the statement adheres to our policy

The sequencing raw data for the experiments have been deposited into the SRA database under identifiers PRJEB78913 (ChIP-seq), PRJEB76622 (GHT-SELEX), and PRJEB61115 (HT-SELEX). Genomic interval information generated for the ChIP-seq and GHT-SELEX have been deposited into GEO under accessions GSE280248 and GSE278858, respectively. Information on constructs, experiments, analyses, processed data, comparison tracks, with many accessory files and browsable results is available at https://codebook.ccbr.utoronto.ca. Larger collection of motifs generated for these experiments in an accompanying study36 can be browsed at https://mex.autosome.org and downloaded at https://doi.org/10.5281/ZENODO.8327372.

Research involving human participants, their data, or biological material

Policy information about studies with human participants or human data. See also policy information about sex, gender (identity/presentation), and sexual orientation and race, ethnicity and racism.

Reporting on sex and gender	*Use the terms sex (biological attribute) and gender (shaped by social and cultural circumstances) carefully in order to avoid confusing both terms. Indicate if findings apply to only one sex or gender; describe whether sex and gender were considered in study design; whether sex and/or gender was determined based on self-reporting or assigned and methods used. Provide in the source data disaggregated sex and gender data, where this information has been collected, and if consent has been obtained for sharing of individual-level data; provide overall numbers in this Reporting Summary. Please state if this information has not been collected. Report sex- and gender-based analyses where performed, justify reasons for lack of sex- and gender-based analysis.*
Reporting on race, ethnicity, or other socially relevant groupings	*Please specify the socially constructed or socially relevant categorization variable(s) used in your manuscript and explain why they were used. Please note that such variables should not be used as proxies for other socially constructed/relevant variables (for example, race or ethnicity should not be used as a proxy for socioeconomic status). Provide clear definitions of the relevant terms used, how they were provided (by the participants/respondents, the researchers, or third parties), and the method(s) used to classify people into the different categories (e.g. self-report, census or administrative data, social media data, etc.) Please provide details about how you controlled for confounding variables in your analyses.*
Population characteristics	*Describe the covariate-relevant population characteristics of the human research participants (e.g. age, genotypic information, past and current diagnosis and treatment categories). If you filled out the behavioural & social sciences study design questions and have nothing to add here, write "See above."*
Recruitment	*Describe how participants were recruited. Outline any potential self-selection bias or other biases that may be present and how these are likely to impact results.*
Ethics oversight	*Identify the organization(s) that approved the study protocol.*

Note that full information on the approval of the study protocol must also be provided in the manuscript.

Field-specific reporting

Please select the one below that is the best fit for your research. If you are not sure, read the appropriate sections before making your selection.

☒ Life sciences ☐ Behavioural & social sciences ☐ Ecological, evolutionary & environmental sciences

For a reference copy of the document with all sections, see nature.com/documents/nr-reporting-summary-flat.pdf

Life sciences study design

All studies must disclose on these points even when the disclosure is negative.

Sample size	332 uncharacterized putative TFs and 61 control proteins were analyzed with GHT- and HT-SELEX approaches. DNA fragments from each experiment were sequenced to approximate depth of 2M reads
Data exclusions	All data is available, including experiments that were not approved in the quality control analyses. Most analyses were performed only with approved experiments. Details are described in supplementary tables.
Replication	Replicates were intended for all proteins, but not all of them were technically successful. The majority of proteins described in the study were analyzed with both HT-, and GHT-SELEX and in most cases as replicates. We note that our QC procedures expected similar motifs to be obtained from different experiments for the same TF.

Randomization	Experiments were performed systematically on different positions of 96-well plates
Blinding	Experimenters were not blind to the identity of the TFs, but each plate contained a mixture of controls and experiments, and all were processed simultaneously by a robot within each batch.

Reporting for specific materials, systems and methods

We require information from authors about some types of materials, experimental systems and methods used in many studies. Here, indicate whether each material, system or method listed is relevant to your study. If you are not sure if a list item applies to your research, read the appropriate section before selecting a response.

Materials & experimental systems

n/a	Involved in the study
☐	☒ Antibodies
☐	☒ Eukaryotic cell lines
☒	☐ Palaeontology and archaeology
☒	☐ Animals and other organisms
☒	☐ Clinical data
☒	☐ Dual use research of concern
☒	☐ Plants

Methods

n/a	Involved in the study
☐	☒ ChIP-seq
☒	☐ Flow cytometry
☒	☐ MRI-based neuroimaging

Antibodies

Antibodies used	Anti-eGFP antibody (Ab290, Abcam), Anti-Rabbit IgG HRP Conjugate, Cat W401B, Promega and GFP-Trap Magnetic Agarose (Chromotek, gtma-100)
Validation	These are common laboratory reagents used for affinity purification, with thousands of citations. The purifications were clearly successful or the study would have failed. Anti-eGFP antibody (Ab290, Abcam) and Anti-Rabbit IgG HRP Conjugate, (Cat W401B, Promega) were used in Western blots that are found in Supplementary Data 5 and showed in ~90% of cases the expected size proteins.

Eukaryotic cell lines

Policy information about cell lines and Sex and Gender in Research

Cell line source(s)	HEK293 Flp-In T-REx cells 608 (Invitrogen). We used one baseline cultured cell line in this study, “Flp-In T-REx HEK293”. That was then recombined to make the individual transgenic cell lines. HEK293 is reported as female origin
Authentication	This is a common cell line with a recombination site. The cell line behaved as expected, including high transfection rate and obtaining recombinants.
Mycoplasma contamination	Negative by PCR
Commonly misidentified lines (See ICLAC register)	*Name any commonly misidentified cell lines used in the study and provide a rationale for their use.*

Plants

Seed stocks	*Report on the source of all seed stocks or other plant material used. If applicable, state the seed stock centre and catalogue number. If plant specimens were collected from the field, describe the collection location, date and sampling procedures.*
Novel plant genotypes	*Describe the methods by which all novel plant genotypes were produced. This includes those generated by transgenic approaches, gene editing, chemical/radiation-based mutagenesis and hybridization. For transgenic lines, describe the transformation method, the number of independent lines analyzed and the generation upon which experiments were performed. For gene-edited lines, describe the editor used, the endogenous sequence targeted for editing, the targeting guide RNA sequence (if applicable) and how the editor was applied.*
Authentication	*Describe any authentication procedures for each seed stock used or novel genotype generated. Describe any experiments used to assess the effect of a mutation and, where applicable, how potential secondary effects (e.g. second site T-DNA insertions, mosiacism, off-target gene editing) were examined.*

ChIP-seq

Data deposition

☒ Confirm that both raw and final processed data have been deposited in a public database such as GEO.

☒ Confirm that you have deposited or provided access to graph files (e.g. BED files) for the called peaks.

Data access links<br>*May remain private before publication.*	The sequencing raw data for the experiments have been deposited into the SRA:<br>ChIP-seq, https://www.ebi.ac.uk/ena/browser/view/PRJEB78913<br>GHT-SELEX, https://www.ebi.ac.uk/ena/browser/view/PRJEB76622 and<br>HT-SELEX, https://www.ebi.ac.uk/ena/browser/view/PRJEB61115. Genomic interval information is in the GEO (ChIP, https://www.ncbi.nlm.nih.gov/geo/query/acc.cgi?acc=GSE280248) and for GHT-SELEX Id. GSE278858
Files in database submission	See web site, there are thousands of files
Genome browser session<br>(e.g. UCSC)	The data are already publicly available.

Methodology

Replicates	Please refer to Supplementary Table S2.
Sequencing depth	20M (targeted depth)
Antibodies	Anti-eGFP antibody (Ab290, Abcam)
Peak calling parameters	MACS2 with sample-specific controls, described in details in the manuscript.
Data quality	Expert curation and quality control for all the experiments are described in an accompanying manuscript (Voronstov et al.).
Software	MACS2
